# Using a periclinal chimera to unravel layer-specific gene expression in plants

**DOI:** 10.1111/tpj.12250

**Published:** 2013-07-19

**Authors:** Ioannis Filippis, Rosa Lopez-Cobollo, James Abbott, Sarah Butcher, Gerard J Bishop

**Affiliations:** 1 Imperial College London, South Kensington CampusLondon, SW7 2AZ, UK; 2 East Malling ResearchEast Malling, Kent, ME19 6BJ, UK

**Keywords:** tomato, L1 layer, periclinal chimera, gene expression, layer-specific, technical advance

## Abstract

Plant organs are made from multiple cell types, and defining the expression level of a gene in any one cell or group of cells from a complex mixture is difficult. Dicotyledonous plants normally have three distinct layers of cells, L1, L2 and L3. Layer L1 is the single layer of cells making up the epidermis, layer L2 the single cell sub-epidermal layer and layer L3 constitutes the rest of the internal cells. Here we show how it is possible to harvest an organ and characterise the level of layer-specific expression by using a periclinal chimera that has its L1 layer from *Solanum pennellii* and its L2 and L3 layers from *Solanum lycopersicum*. This is possible by measuring the level of the frequency of species-specific transcripts. RNA-seq analysis enabled the genome-wide assessment of whether a gene is expressed in the L1 or L2/L3 layers. From 13 277 genes that are expressed in both the chimera and the parental lines and with at least one polymorphism between the parental alleles, we identified 382 genes that are preferentially expressed in L1 in contrast to 1159 genes in L2/L3. Gene ontology analysis shows that many genes preferentially expressed in L1 are involved in cutin and wax biosynthesis, whereas numerous genes that are preferentially expressed in L2/L3 tissue are associated with chloroplastic processes. These data indicate the use of such chimeras and provide detailed information on the level of layer-specific expression of genes.

## Introduction

Ever since Hooke’s seminal observations that organisms are made from cells a fundamental question has existed as to what defines the components that make each cell unique. Plants are made from numerous different cell types that have different shapes, sizes and function. Each unique cell type has its own complement of mRNA and protein to perform its specialised function. Being able to define the components that make each cell unique is of fundamental importance for gaining a better understanding of all processes occurring in plants from vegetative growth to flowering, and from resistance to insect and pathogen attack to plant mineral nutrition. Many approaches have been used to dissect this complexity in which the differences in the RNA complement (transcriptome) and protein levels (proteome) are assessed between different tissue samples. The samples isolated for analysis are obtained from two major sources. The first source is from whole plants, seedlings, calli, etc., or from discrete organs, for example leaf, root, flower, petal, apex, etc., and such samples contain numerous different cell types (Rakwal and Agrawal, 2003; Ko and Han, 2004; Pischke *et al*., 2006; Ruffel *et al*., 2008; Zeller *et al*., 2009). The second source constitutes samples collected from specific cell types or groups of cells. These cells can be identified either by staining or cell-specific reporter gene expression and subsequent dissection and cell sorting, e.g. guard cells, by the isolation of specific cells or groups of cells, for example epidermal peels, or by the mechanical removal of trichomes (Birnbaum *et al*., 2005; Day *et al*., 2005; Bargmann and Birnbaum, 2010; Matas *et al*., 2010, 2011; Hu *et al*., 2011). In both scenarios the transcriptome can then be analysed using either arrays or high-throughput sequencing technologies directly on the cDNA or via the sequence analysis of PCR products that sample the transcriptome, i.e. SAGE (serial analysis of gene expression) (Bao *et al*., 2005; Brady *et al*., 2006).

When mixtures of cell types are used in transcript analysis the level of expression for any one gene is the summation of all the cell types and thus it is not possible to discern whether all cells respond in the same way. For example, it is conceivable that within the same organ certain genes may be up-regulated in one cell type and down-regulated in another. Layer-specific regulation of such genes will, therefore, not be observed. Similarly, when specific cell types are isolated usually only one type is taken for analysis and the remainder discarded, which therefore limits the holistic interpretation of results. In addition, the isolation of specific cell types by mechanical means, for example laser dissection, protoplasting and cell sorting, may lead to the generation of artefacts through the extraction process. The best scenario is to tag each cell type within an individual organ such that it can be discriminated from other cell types but yet carry out the analysis on the whole organ. Here we show that this is possible using a periclinal chimera.

**Figure 1:**
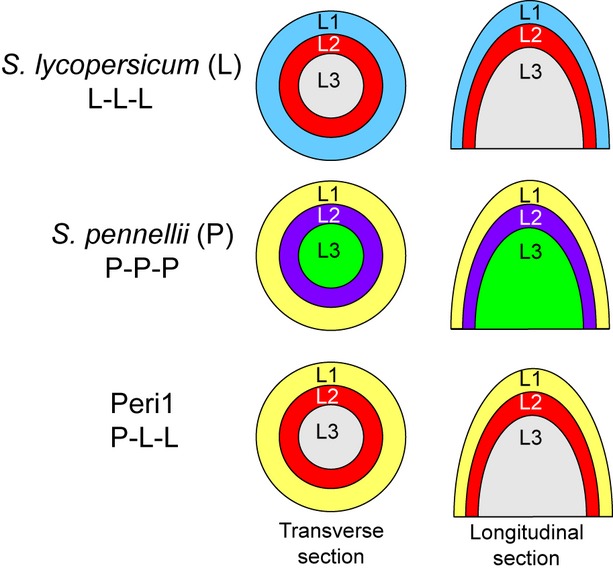
Schematic diagrams indicating the cell layers in an apical meristem. Three different meristems are shown, two parental lines and a periclinal line in which the outermost L1 cell layer is derived from *Solanum pennellii* whereas the internal tissue is from *Solanum lycopersicum*.

Plants that have one layer of cells that is genetically distinct from another layer are called periclinal chimeras. In dicotyledonous plants there are three distinct cell layers, L1, L2 and L3 (Figure 1). The outermost layer, L1, is made up of epidermal cells, stomata and hairs and L2 comprises the sub-epidermal mesophyll cells (in a leaf). Layers L1 and L2 form the tunica in which cell division is normally anticlinal, i.e. divisions that are at right angles to the surface of the growing point and thus do not add new rows of cells. The innermost L3 cells are referred to as the corpus and they can divide both anticlinally and periclinally (periclinal cell divisions are those that are parallel to the surface) (Satina *et al*., 1940; Sussex, 1989; Ingram, 2004). A periclinal chimera may be between plants of the same species or between plants of different species (Marcotrigiano, 1986; Marcotrigiano and Bernetzky, 1995).

Here we show the characterisation of layer-specific gene expression in a mixture of tomato cell types utilising high-throughput sequencing, detection of single nucleotide polymorphisms and quantification of parental-origin allele-specific expression. This was possible by generating a periclinal chimera that has the L1 epidermal layer of a wild species and the L2 and L3 layers from cultivated tomato. RNA-seq analysis and the availability of the tomato genome sequence and annotation enabled the genome-wide assessment of whether a gene is expressed in the L1 or L2/L3 layers.

## Results

### Generation of the epidermal L1 chimera

To generate the periclinal chimera about 120 ‘V’ grafts were made between the tomato lines Heinz 1706 (*Solanum lycopersicum*) and LA716 (*Solanum pennellii*). Graft unions were cut and allowed to callus and generate adventitious shoots. From these shoots one was observed to be a potential periclinal chimera that has the L1 layer of *S. pennellii* and the internal tissue of Heinz 1706. This shoot was maintained as a cutting and called Periclinal 1 (Peri1).

**Figure 2:**
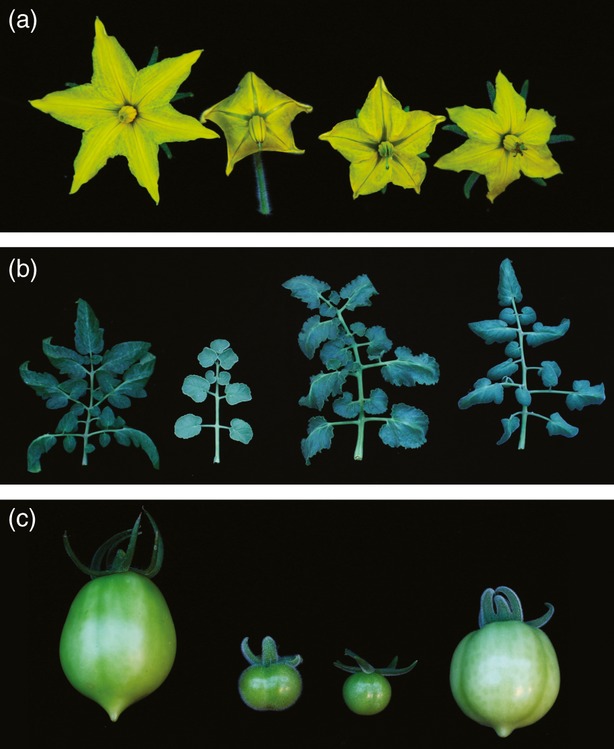
Morphology of L1 periclinal chimera. (a) Flowers at anthesis. (b) Leaves, eighth leaf from the apex. (c) Mature green fruits. From left to right, *Solanum lycopersicum* (Heinz 1706), *Solanum pennellii* (LA716), F_1_ [*S. lycopersicum* (Heinz 1706) × *S. pennellii* (LA716)] and Periclinal 1.

The type of chimera generated was verified by phenotypic observations, crossing experiments and progeny testing. Figure 2 shows the phenotype of tissues from this periclinal line whose leaves have a lighter green colour compared with Heinz 1706 due to the numerous trichomes present on the leaf surface. When touched the leaves have the same oily feel as those of *S. pennellii* (LA716) due to the presence of glandular trichomes, as previously observed by Goffreda *et al*. (1990). The chimera also has altered leaf morphology in comparison with the parental types in that the leaflets are smaller and less serrated than the Heinz variety but more elongated than LA716 (Figure 2). The leaflets of the F_1_ cross between these species, in comparison, are much larger due to heterosis. An additional key observation is that the periclinal chimera does not set fruits by selfing, whereas the Heinz and LA716 lines are self-compatible. In crossing experiments when the chimera was used as the female parent it failed to set seed using pollen from Heinz plants but did set seed when crossed with LA716 pollen (Table 1). Pollen from the chimera was crossed to LA716 plants but no seed was produced; however, seed was set when pollen from the chimera was crossed to Heinz plants. It is known that *S*. *pennellii* LA716 cannot be fertilised by other tomato species, and that this incongruous behaviour is associated with the L1 layer (Liedl *et al*., 1996).

**Table 1:** Compatibility of crosses between parental lines and layer 1 periclinal chimera 1

		Male		
		Heinz 1706	LA716	Periclinal 1
Female	Heinz 1706	Yes	Yes	Yes
	LA716	No	Yes	No
	Periclinal 1	No	Yes	No

These data therefore confirm the fact that the L1 surface of the stigma in the chimera is of *S. pennellii* that will only allow successful fertilisation by *S. pennellii* pollen. However, as pollen is generated from the L2 layer (Huala and Sussex, 1993), the pollen from the chimera is *S. lycopersicum* and it therefore cannot self-fertilise the *S. pennellii*-‘covered’ styles of the chimera. The pollen can, however, be used to fertilise the Heinz plants.

The size and weight of seeds from the parental lines and the crosses generated confirm these observations (Figure S1). The F_1_ seeds from a cross between *S*. *lycopersicum* and *S*. *pennellii* have a size and weight intermediate between those of the parental lines. While the size and weight of seeds from crosses between *S*. *lycopersicum* and the chimera are very similar to Heinz seeds, the seeds from Peri1 and *S*. *pennellii* crosses are similar or the same as those of F_1_ seeds (Figure S1). Progeny testing of these lines also confirmed these results; all seedlings from the Peri1 × LA716 cross had the F_1_ phenotype between these two species and all progeny from Heinz 1706 × Peri1 had a Heinz 1706 phenotype (Table 2).

**Table 2:** Phenotypes of seedlings from progeny test crosses

		Male		
		Heinz 1706	LA716	Periclinal 1
Female	Heinz 1706	100% Heinz	100% F_1_	100% Heinz
		*n* = 12	*n* = 12	*n* = 12
	LA716	No seed	100% LA716	No seed
			*n* = 12	
	Periclinal 1	No seed	100% F_1_	No seed
			*n* = 12	

### Polymorphism detection

To confirm that the periclinal line was a mixture of both species’ genomes, genomic DNA was extracted from the chimera and the parental cultivars. Eleven genes were amplified by PCR (see Table 3) and sequence analysis of the PCR products revealed 1.3% sequence polymorphisms in coding regions [135 single nucleotide polymorphisms (SNPs)/10317 bp] and about 2% in non-coding regions (65 SNPs/3140 bp). Assuming that both alleles amplify equally, the relative frequency of each base at each polymorphic site will give an estimate of the proportion of template DNA from each species. On average the level of the *S. pennellii* allele was about 20% of the total, which provides an approximate level of *S. pennellii* tissue in the sample, i.e. the L1 tissue was about one-fifth of the sample.

**Table 3:** Analysis of layer 1 (L1) specificity of genes involved in processes that may exhibit layer-specific expression

Gene	Locus name	Solyc ID	*Arabidopsis thaliana* protein homologue	% L1 specificity	Reference
*SlML*1	Meristem layer 1	Solyc10g005330	AAB49378.1	100 ± 1	Lu *et al*. (1996)
*SlPDF*1	Protodermal factor 1	Solyc07g055950	AAD33869	98 ± 3	Abe *et al*. (1999)
*SlSYS*	Prosystemin	Solyc05g051750	na	8 ± 2	Jacinto *et al*. (1997)
*SlzFPS*	Z-isoprenyl pyrophosphate synthase	Solyc08g005680	na	98 ± 4	Sallaud *et al*. (2009)
*SlSBS*	Santalene and bergamotene synthase	Solyc08g005640	NM_106594.3	91 ± 8	Sallaud *et al*. (2009)
*SlBRI1*	Brassinosteroid insensitive 1	Solyc04g051510	NP_195650	53 ± 5	Montoya *et al*. (2002)
*SlBIN2*	Brassinosteroid-insensitive 2	Solyc02g072300	NP_193606	56 ± 6	Li and Nam (2002)
*SlBAK1*	Brassinosteroid insensitive1-associated receptor kinase 1	Solyc01g104970	NP_567920	58 ± 4	Li *et al*. (2002)
*SlCYP85A1*	*Dwarf*	Solyc02g089160	NP_974862	31 ± 5	Bishop *et al*. (1996, 1999)
*SlTMM*	Too many mouths	Solyc12g042760	NP_178125	45 ± 4	Yang and Sack (1995)
*SlStomagen*	Stomagen	Solyc08g066610	NM_117366.3	14 ± 4	Sugano *et al*. (2010)

### Layer L1 specificity

**Figure 3:**
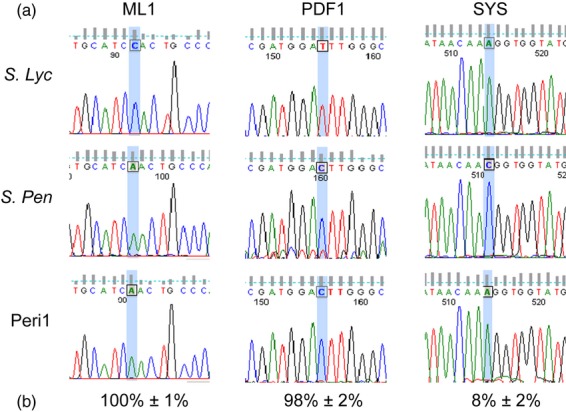
Sanger sequencing detecting polymorphisms and layer-specific expression. (a) Sanger sequencing traces of cDNA sequences of *ML1*, *PDF1* and *SYS* tomato genes using template cDNA from parental (*Sl = Solanum lycopersicum, Sp = Solanum pennellii*) and Peri1 lines. (b) Mean and SE percentage of expression in layer L1 after normalization to the amount of L1 tissue as indicated by the frequency of the *pennellii* allele (*SlML1 n* = 6; *SlPDF1 n* = 3; *SlSYS n* = 6). (Note: L1-specific genes *ML1* and *PDF1* have identical traces to *S. pennellii* whereas the L3-specific gene has an identical trace file to *S. lycopersicum*).

To show that it is possible to discriminate the relative layer-specific expression of a gene from a leaf sample containing a mixture of cell types, analysis of genes known to have layer-specific expression was carried out. First total RNA was extracted from leaf tissue from *S. lycopersicum* (*lyc*), *S. pennellii* (*penn*) and Peri1. These RNAs were used in RT-PCR experiments with PCR primers designed to amplify the tomato homologues of genes known to be L1 specific in Arabidopsis, namely *Meristem Layer 1* (*ML1*) (Lu *et al*., 1996) and *Protodermal Factor 1* (*PDF1*) (Abe *et al*., 1999). As shown in Figure 3, sequence analysis of the PCR products of the *ML1* and *PDF1* homologues shows that the products from the chimera have identical sequence to the *penn* sequences. This is predicted as the L1 layer in the chimera is *S. pennellii* and therefore any genes specifically expressed in L1 will only have the *penn* sequence. This analysis was extended further to the tomato prosystemin gene that is known to be expressed in layer L3 (Jacinto *et al*., 1997). RT-PCR and sequence analysis of the PCR product from the Peri1 chimera showed that the prosystemin sequence was identical to the *S. lycopersicum* allele, confirming L3 specificity. This analysis was also performed on products of various genes involved in different processes, and the percentage expression in layer L1 was calculated after normalising to the amount of L1 tissue (Table 3).

### Genome-wide analysis of layer-specific gene expression

To gain a global understanding of layer-specific gene expression we sequenced transcripts from three major tissue samples from the parental lines and the chimera, namely leaves, leaves undergoing water stress that induced increased ABA levels (Table S1) and fruit. Water stress was used as *S. pennellii* is more drought tolerant than *S. lycopersicum* (Gong *et al*., 2010) (Figure S8) and water stress induces gene expression (Cameron *et al*., 2006). Such water stress samples would therefore enhance our ability to discriminate for layer-specific expression. Sequencing was carried out using the Applied Biosystems SOLiD3 platform, which generated around 162 million reads of 50 base pairs (bp) length for all nine samples. Best alignments of about 61.6 million reads to known coding sequencing regions in the *S. lycopersicum* genome yielded a total transcript coverage of 32×, 16×, 29× for the *lyc*, *penn* and peri1 libraries, respectively (Table S2).

Out of about 35 000 genes annotated in the tomato genome (TGC, 2012), 21 938 were found to be expressed in both parental lines and the chimera independent of tissue sample (Table S4). Mapped reads across tissue samples were pooled for the parental lines to identify polymorphisms between *lyc* and *penn* alleles in the coding sequences of the 22 000 expressed genes. Detection of SNPs by VARiD (Dalca *et al*., 2010) and FreeBayes (Garrison and Marth, 2012) commonly identified 13 277 genes with at least one SNP with a minimum 4 × coverage in both parental lines (Table S3). All methods commonly detected 63 669 SNPs, which correspond to about 0.5% of all examined coding sequence bases with at least 4 × depth in both *lyc* and *penn* pooled samples (Table S3). Results of the comparison of the three variant detection methods are presented in Figure S2.

Expression levels of genes were assessed separately for each tissue sample in all three plant types at identified SNP positions and with respect to the parental origin in the chimera. Our analysis has the structure of a 2 × 2 factorial experiment with one factor being the parental-origin (*lyc* or *penn*) allele-specific expression. The second factor relates to the genome examined, chimeric (peri) or parental wild type (*lyc* or *penn*). The *lyc* allele-specific expression in the parental genome (Lw) and in the chimera (Lc), as well as the pennellii ones (Pw and Pc) were quantified (Table S4). Ideally, Lc must be 0 for a gene that is expressed only in L1. Decreased or increased expression of genes due to the generation of the chimera was accounted for.

**Figure 4:**
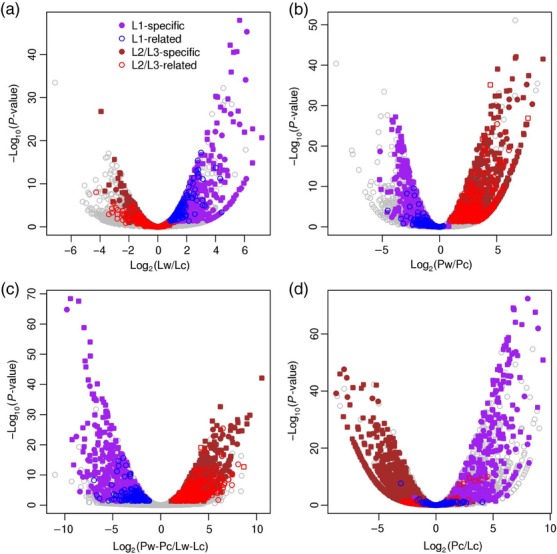
Differential expression analysis on parental-origin allele-specific expression identifies layer-specific genes. The relative abundance of each gene plotted versus the *P*-value, for all tissues and for the four contrasts examined. (a) Difference of *lyc* allele-specific expression between wild type (Lw) and chimera (Lc). (b) Difference of *penn* allele-specific expression between wild type (Pw) and chimera (Pc). (c) Interaction effect, i.e. difference of the differences. (d) Difference of *penn* and *lyc* allele-specific expressions in chimera. Grey circles, all genes examined; purple and blue circles, L1-specific and L1-related genes, respectively; brown and red circles, L2-specific and L2-related genes, respectively. Squares denote genes that are classified based on more than one tissue sample. Expression and the resulting fold changes and *P*-values are calculated based on polymorphisms detected by VARiD.

Differential expression analysis to classify genes as L1 or L2/L3 was conducted using a negative binomial generalized linear model (Robinson *et al*., 2010). Genes that exhibit differential expression in the chimera were identified by treatment-contrast parameterization (Smyth, 2005). L1 genes are those that are down-regulated in the chimera compared with the wild-type genome with respect to their *lyc* origin (Figure 4b) and not with respect to their *penn* origin (Figure 4a), and for which a statistically significant interaction effect is observed (Figure 4c). Those L1 genes which show upregulation in the chimera with respect to their *penn* allele-specific expression and in comparison with their *lyc* one (Figure 4d), are denoted as L1 specific, otherwise as L1 related. In a similar way, genes with a (highly) biased lyc allele-specific expression in the chimera are classified as L2/L3. After correcting for multiple testing (Benjamini and Hochberg, 1995; Benjamini and Yekutieli, 2001) and applying a cut-off value of 0.05 for the false discovery rate (FDR), 382 genes were classified as L1 and 1159 as L2/L3, with 254 of the 382 genes being called L1 specific and 811 of the 1159 being L2/L3 specific. The lists of classified genes are provided in Tables S5 and S6, while comparison of the classification resulting from the three variant detection programmes are shown in Figure S3.

The tomato homologue of Arabidopsis *Meristem Layer 1*, a gene known to be expressed specifically in L1 (Lu *et al*., 1996), was identified in our data (Table S5). *Prosystemin*, a gene known to be expressed in L3 (Jacinto *et al*., 1997), was found in our results to be L2/L3 specific (Table S6). To validate our classifications, we randomly selected 14 predicted L1 genes, three predicted L2/L3 genes, as well as the two predicted known layer-specific genes. The parental-origin allele-specific expression for selected genes is plotted in Figure S5. Layer 1 genes show down-regulation of *lyc* allele expression in the chimera and L2/L3 genes show down-regulation of *penn* allele expression. Analysis of the Sanger trace files confirmed all our classifications (Tables 3 and S7) with the exception of one ambiguous case – a receptor-like kinase involved in brassinosteroid signalling was found to be expressed evenly between L1 and L2/L3 layers, while genome-wide analysis classified it as L2/L3 specific.

**Figure 5:**
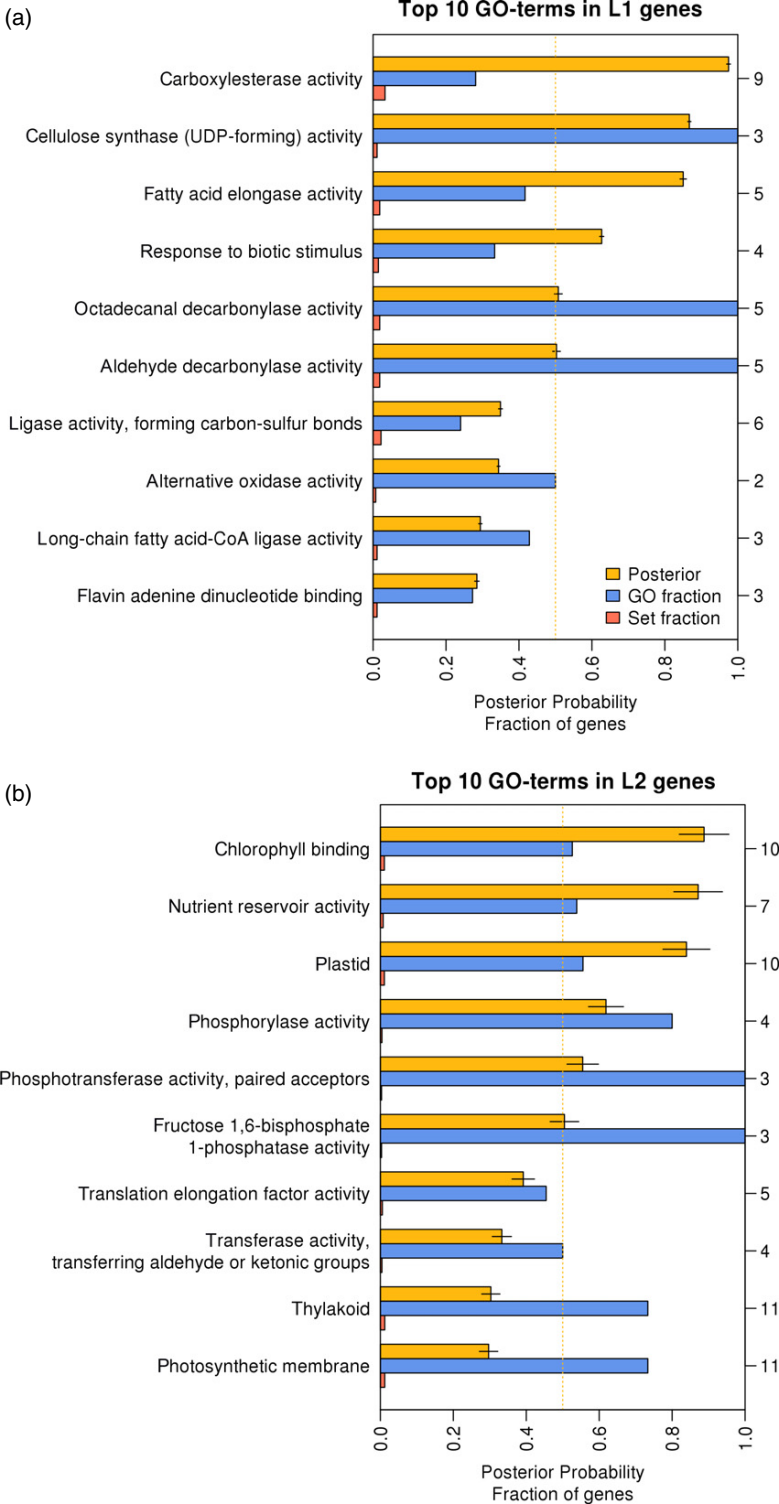
Ranked list of the top 10 over-represented Gene Ontology (GO) terms in layer L1 (a) and layers L2 and L3 (b) identified genes. Terms are ranked based on the marginal posterior probability calculated using the MGSA algorithm as implemented in Ontologizer (Bauer *et al*., 2008, 2010). Posterior probability is shown in yellow. GO terms having a posterior >0.5 are considered to be active, i.e. statistically significantly over-represented. Terms with posterior bars with shadowing lines are active when considering only the genes characterized as L1 specific (or L2/L3 specific) and thus excluding the layer-related ones. Error bars (95% confidence intervals) are obtained with 20 runs. The GO fraction is the fraction of all genes with the specific GO-term that is L1 or L2/L3 (coloured blue). The set fraction is the fraction of all L1 or L2/L3 genes assigned with the specific GO-term (coloured red). The numbers of L1 and L2/L3 genes assigned with a GO-term are denoted in the right *y*-axis.

Gene Ontology (GO) enrichment analysis for genes classified as L1 (Figures 5a and S6) and L2/L3 (Figures 5B and S7) identified the function of genes and biological processes that are layer specific. Genes in L1, as opposed to L2/L3 ones, are involved in lipid and wax synthesis, and in cellulose synthesis in cell wall production. Five of the top 10 genes that are L1 specific are associated with lipid synthesis (Table S5). This fits with the epidermis producing cutin and wax (Javelle *et al*., 2011) that act as a barrier to prevent water loss. Other L1 genes identified are involved in cell wall synthesis and are likely to influence organ expansion (Savaldi-Goldstein *et al*., 2007). Genes preferentially expressed in L2/L3 include those associated with photosynthesis and the chloroplast. This is not unexpected, as the epidermal pavement cells lack chloroplasts and any nuclear gene whose protein is targeted to the chloroplast is expected to be expressed in the L2/L3 tissues rather than the L1.

## Discussion

### Peri1, an L1 chimera

Periclinal chimeras have previously been generated in tomato and used to show how cell layers contribute to different processes (Heichel and Anagostakis, 1978; Goffreda *et al*., 1990; Liedl *et al*., 1996). One key observation from this earlier work was that layer L2 contributes to the gametes and that layer L1 contributes to the compatibility of viable crosses between *S. pennellii* and *S. lycopersicum* (Liedl *et al*., 1996). These preceding observations were used to confirm the structure of the Peri1 chimera; namely crossing experiments and progeny testing showed that Peri1 is an L1 chimera similar to that generated previously by others (Goffreda *et al*., 1990). This is an important observation as Peri1 was made using Heinz 1706, a wild-type variety that has been utilised in the tomato genome sequencing project (TGC, 2012) and does not harbour any visible mutations to confirm the genotype of the different layers.

Sequence analysis of PCR products generated using genomic DNA from Peri1 as the template indicated polymorphic sequences. These polymorphisms were confirmed when comparing the sequence of PCR products from the Heinz 1706 and LA716 parental lines. The level of polymorphism of 1.3% in the 13 457 bp analysed is similar to that detected by others (Yamamoto *et al*., 2005; Kamenetzky *et al*., 2010). The relative proportion of the species-specific alleles provides an estimate of the amount of *pennellii*/*lycopersicum* template DNA and thus a rough estimate of the proportion of *pennellii*/*lycopersicum* cells that contribute to the layers in the chimera. For the leaf sample used in our experiments the amount of *S. pennellii* was approximately 20% of the tissue.

### Layer-specific expression

To show that Peri1 can be used to discriminate layer-specific expression, the tomato homologues of the Arabidopsis *ML1* and *PDF1* genes known to be L1 specific (Lu *et al*., 1996; Abe *et al*., 1999) were identified. When the RT-PCR products of *SLML1* and *SLPDF1* were sequenced from Peri1 template cDNA around 100% of the cDNA sequence had the *S. pennellii* alleles. This highlights that a conserved mechanism exists between Arabidopsis and tomato in which L1-specific transcription takes place. The opposite scenario was also tested. Prosystemin promoter GUS fusion lines exhibit GUS expression in vasculature (Jacinto *et al*., 1997) and the resulting cDNA sequencing of prosystemin from Peri1 only identified the *S. lycopersicum* allele. These data further corroborated the results that Peri1 was an L1 chimera and that sequencing cDNA from this plant can give an indication of the amount of expression of a gene in the L1 or L2/L3 layers.

Genes are classified as L1 or L2/L3 based on multiple tissues. All libraries generated from dehydrated tissues exhibit higher read coverage within each plant type, while across plant types dehydrated libraries have most similar coverage to each other (Table S2). Examining the tissue support (Figure S4), we observe that 70% of gene classifications are verified by analysis of dehydrated leaf samples, while 48% are solely based on these. This indicates that dehydrated samples are highly informative and help better identify layer-specific genes. In addition our conservative method of analysis minimises any false predictions at the expense of coverage of the layer-specific transcriptome. In the future, deeper sequencing using a range of technologies will enable a more detailed analysis of the level of layer-specific expression in various tissues under varying conditions.

The methodology and the data we have generated provide numerous leads for genes that are preferentially expressed in the L1 and L2/L3 layers of plants (Tables S5 and S6) and these data corroborate those generated by laser dissection and sequencing of the fruit epidermis (Matas *et al*., 2011). It is not surprising to see that many of the L1-specific genes being expressed are associated with cutin and wax formation. This includes *Solyc11g006250* that encodes a GDSL1 lipase, so called due it belonging to the family of Gly-Asp-Ser-Leu esterases/acylhydrolases. The *cutin deficient 1* (*cd1*) mutant is defective in this gene and *cd1* tomato fruits show increased sensitivity to water loss as only 5–10% of the amount cutin as in the wild type is present (Yeats *et al*., 2012). When this gene is silenced it leads to a reduction of cuticle thickness and the number of cutin monomers present per unit area (Girard *et al*., 2012). Interestingly, immunogold labelling of this lipase shows that it is embedded in the cuticle (Girard *et al*., 2012; Yeats *et al*., 2012) and thus correlates with the observed L1 expression. Our analysis also revealed L1 expression of a further seven members of the GDSL1 lipases (Table S5). Other genes related to cutin or wax formation that were shown to be preferentially expressed in L1 tissue include the cytochrome P450 enzymes CYP86A69, CYP77A19 and CYP77A20. Mutants of CYP86A69 are defective in cutin biosynthesis (Isaacson *et al*., 2009; Shi *et al*., 2013) and this P450 encodes an end chain fatty acid hydroxylase (Shi *et al*., 2013).

Tomato homologues of the different types of *eceriferum* (*cer*) mutants of Arabidopsis are also found to be expressed in L1. These include homologues of *cer1* and *cer3* mutants that are defective in epicuticular wax (alkane) formation (Aarts *et al*., 1995). Further genes identified as being L1 specific and involved in cutin biosynthesis include those encoding glycerol-3-phosphate acyltransferase activity (Yang *et al*., 2012) and fatty acid elongase 3-ketoacyl-CoA synthases (Joubes *et al*., 2008).

Many of the biosynthesis genes involved in cutin/wax formation were found to be L1 specific; however, the SLShine3 transcription factor that regulates cuticle formation in tomato (Shi *et al*., 2013) was not identified as L1-specific in our analysis. The lack of identification of SLShine3 is a consequence of a lack of sequence coverage preventing SNP detection. If a SNP had been observed, the number of sequence reads corresponding to this gene obtained from the Periclinal libraries was minimal, thus preventing us from assigning the gene as L1-specific. It is also conceivable that there will be genes for which sequence coverage is good but no SNP exists between *lycopersicum* and *pennellii*, making the detection of layer-specific expression impossible. These observations highlight the need for deeper transcriptome sequencing of peri1 tissues. In addition, the future availability of the *S. pennellii* genome sequence will provide detailed and comprehensive SNP data between it and *S. lycopersicum* and thus greatly increase the power of layer-specific gene analysis.

The identification of the L1-expressed sequences provided the opportunity to determine if an L1-box motif was present in the 5′ region of these genes. In Arabidopsis an L1-box motif has been observed (TAAATGYA) that regulates the expression of *Protodermal Factor 1* in L1 cells (Abe *et al*., 2001). Analysis of 1000 bp regions upstream of the translation start sites of the genes expressed and with at least one SNP identified by VARiD, showed an enrichment of the L1 box sequence (*P*-value = 0.005) in L1-related genes, based on Fisher’s exact test. The L1 box was present in 18% of the L1 genes as opposed to 12% of L2/L3 genes and 13% of other genes examined. This enrichment is in agreement with our classification and also indicates the possibility of a conserved mechanism for L1-specific expression between Arabidopsis and tomato.

### Future uses

The periclinal line that has been developed can be utilised to assess the relative level of L1 or L2 and L3 expression in different tissues and under different conditions. Most notably this has the potential to provide information on layer-specific expression of genes involved in epidermal-specific processes such as trichome and guard cell formation. The level of L1 specificity of genes involved in cutin formation and the production of metabolites involved in insect and pathogen resistance can also be discerned. Such chimeras also have the potential to unravel layer-specific gene expression in meristems, especially in the formation of leaf and flower primordia. Further different types of chimeras can be made to increase the power of analysis. It is conceivable to have a chimera in which the three layers are made from three different species. In practice, however, layer invasion between the L2 and L3 layers occurs at a rate that causes problems in maintaining such chimeras. L1 chimeras are, however, very stable and lack significant layer invasion (Schmulling and Schell, 1993; Jenik and Irish, 2000). Chimeras also need not be restricted to those between tomato species and can include other economically important solanaceous species, for example potato and aubergine. Chimeras can be made for shrubs and trees, for example *+Laburnocytisus* ‘*Adamii*’ which is a chimera between a laburnum, *Laburnum anagyroides*, and a broom, *Chamaecytisus purpureus*. Grape and other species in which grafting is easily carried out and explants maintained as cuttings can also be used to generate chimeras. Thus this method of analysis can be extended widely.

## Experimental procedures

### Plant growth

Tomato Heinz (*S. lycopersicum* 1706), *S. pennellii* LA716 and the periclinal line (Peri1) were grown in a glasshouse supplemented with artificial light for a 16-h/8-h photoperiod at 120 μmol m^−2^ sec^−1^ photosynthetically active radiation (PAR) irradiance.

### Generation of chimeras

The grafting was carried out essentially as described previously (Montoya *et al*., 2005). Three rounds of grafting were carried out, with between 30 and 50 grafts being made at each attempt. For the successful generation of a single periclinal chimeric plant about 50 grafts between various plant genotypes were made using plants at the four true leaf stage of development. Graft unions were made and kept in place with appropriate size plastic tubing and either Vaseline or lanolin was used to seal the union. Grafted plants were kept on a misting bench for about 1–2 weeks. Around 90% of grafts were successful. After approximately 1 month the graft junction was cut and the cut tissue was then covered with lanolin paste or Vaseline to prevent desiccation. Callusing tissue and adventitious shoots were then allowed to grow from the graft union. Plants that lacked adventitious shoot formation were discarded. From the 50 lines one striking periclinal chimera was observed and maintained as a cutting.

### DNA and RNA isolation

Genomic DNA and RNA were extracted from tissue of the eighth leaf from the apex of *S. lycopersicum*, *S*. *pennellii* and Peri1 plants. All samples were frozen immediately in liquid nitrogen, ground and kept at −80°C. For DNA extraction the tissue was resuspended in nuclear extraction buffer [120 mm 2-amino-2-(hydroxymethyl)-1,3-propanediol (TRIS)-HCl, 30 mm EDTA, 1.2 m NaCl, 1.2% cetyl trimethylammonium bromide (CTAB), 40 mm sodium bisulphite, pH 7.5) and 1% sarkosyl. After incubation at 65°C the DNA was extracted twice with phenol:chloroform:isoamyl alcohol (25:24:1). The DNA was recovered by isopropanol precipitation. For total RNA isolation the tissue was homogenized in RNA extraction buffer (8 m guanidinium hydrochloride, 20 mm MES, pH 7) and extracted with phenol:chloroform: isoamyl alcohol. After centrifugation at 11 000 ***g*** the aqueous phase was re-extracted with phenol:chloroform:isoamyl alcohol and then subjected to three successive precipitations with ethanol/sodium acetate, 4 m LiCl and then ethanol/sodium acetate again. The final pellet was washed in 70% ethanol and resuspended in diethyl pyrocarbonated treated water. The total RNA concentration was estimated in a NanoDrop 1000 Spectrophotometer (Thermo Scientific, http://www.thermofisher.com) and analysed on a formaldehyde/3-(*N*-morpholino)propanesulphonic acid (MOPS) gel.

### Expression analysis by RT-PCR

The RT-PCR was performed using Moloney Murine Leukaemia Virus (M-MLV) reverse transcriptase (Promega, http://www.promega.com/) according to the manufacturer’s instructions. The single-strand DNA was synthesized in a 25-μl reaction mixture containing 2 μg of total RNA denatured by heating at 65°C for 5 min, 1 μg oligo(dT) anchor primer, 10 nm of deoxynucleotide triphosphate and 200 units M-MLV reverse transcriptase (Promega). The RT reactions were performed at 42°C for 45 min and stopped by incubation at 70°C for 15 min. Single-stranded DNA was amplified by PCR in mixtures containing 1 μl cDNA, 2.5 mm MgCl_2_, 10 mm TRIS–HCl, 50 mm KCl, 0.25 unit of Taq DNA polymerase (New England Biolabs, https://www.neb.com/), 2 mm of each deoxynucleotide triphosphate and 0.5 μm of each gene-specific primer as shown in Table S8.

The PCRs were performed in a GeneAmp PCR system 9700 thermocycler (Applied Biosystems, http://www.lifetechnologies.com) using the following conditions: 94°C for 3 min followed by 35 cycles of 94°C for 20 sec, 55°C for 30 sec, 72°C for 60 sec. The PCR products were analysed on a 1% (w/v) agarose gel containing 0.05 μl ml^−1^ of SYBR Safe (Invitrogen, http://www.invitrogen.com/).

### Sequence analysis and polymorphism detection

The RT-PCR products were purified using QIAquick gel extraction kits (Qiagen, http://www.qiagen.com/) following the manufacturer’s instructions. Sanger sequencing of PCR products was carried out at GATC Biotech, http://www.gatc-biotech.com (London, UK). Sequence analysis to identify the polymorphisms (SNPs and indels) between species was carried out by aligning sequences using the ContigExpress package in the Vector NTI sequence analysis software (Invitrogen).

The proportion of *S*. *pennelli* (L1 expression) of each polymorphic base in the periclinal line (Peri1) was calculated using the chromatogram’s peak height and quality score (Ewing *et al*., 1998) for each base. The peak height was used to calculate the percentage level of the *S*. *pennellii* or *S. lycopersicum* allele at the base. These percentages were used to obtain an arithmetic mean of all the polymorphic sites assessed and this value provides the proportion of L1 expression for an allele in Peri1. We performed a similar analysis using genomic DNA to estimate the proportion of tissue that corresponds to layer L1. Approximately 20% of the tissue in a sample corresponds to layer L1 (*S. pennellii*) and therefore the expression levels were adjusted to account for this level (see supporting data and example calculation in Methods S1).

### RNA-seq

Total RNA was extracted from: (i) eighth leaf from the apex of 8-week-old plants, (ii) eighth leaf with 30% loss of fresh weight (D), (iii) mature green fruits (F) of *S. lycopersicum*, *S. pennelli* and Peri1 plants (see Figure 2b,c, for photographs of samples used). PolyA RNA was isolated as described in the Supporting Information. Whole transcriptome libraries were prepared from each sample, following the procedures of the SOLiD Whole Transcriptome Analysis Kit (Life Technologies, http://www.lifetechnologies.com/). Libraries were barcoded and pooled prior to ePCR amplification. One flowcell was loaded and 50-bp fragments were sequenced with a SOLiD version 3 instrument (Life Technologies).

### Layer-specific transcriptome analysis

Whole transcriptome reads were aligned against the *S. lycopersicum* reference genome (TGC, 2012) using Applied Biosystems SOLiD BioScope Whole Transcriptome Analysis Alignment Pipeline (Life Technologies, 2010). Valid reads mapped to coding sequence regions were extracted and pooled across tissue samples for both parental lines to identify polymorphisms between *lyc* and *penn* alleles. Bases with coverage of less than four reads in either *lyc* or *penn* pooled samples were discarded. Genes were analysed for species-specific SNP expression to determine layer-specific expression. Details of data analysis are provided in Supporting Information.

The transcriptome sequence reads have been deposited with the EBI-SRA under study ERP002648.

A full description of the methods used is provided in Methods S1 in the Supporting Information.

## Supporting Information

Figure S1 Phenotype of seeds.

Figure S2 Comparison of variant detection methods.

Figure S3 Comparison of gene classifications resulting from all three variant detection methods.

Figure S4 Comparison of tissue support for all genes classified as from layer L1 or layers L2/L3.

Figure S5 Parental-origin allele-specific expression values for all Sanger-sequenced genes.

Figure S6 Gene Ontology graph of over-represented terms for layer L1 genes.

Figure S7 Gene Ontology graph of over-represented terms for layers L2/L3 genes.

Figure S8 Water loss.

**Table S1.** Levels of ABA in water-stressed leaves. **Table S2.** Results from mapping of SOLiD reads for all nine libraries. **Table S3.** Results from variant detection. **Table S4.** Parental-origin allele-specific expression values in all samples. **Table S5.** List of genes classified as layer L1, either specific or related. **Table S6.** List of genes classified as layers L2/L3, either specific or related. **Table S7.** Comparison of layer L1 specificity from Sanger sequencing with their Next Generation Sequencing based classification. **Table S8.** Sequences of primers used.
