# Supplementary figures and images for: Using a periclinal chimera to unravel layer-specific gene expression in plants
Source: Plant J. 2013 Jul 19;75(6):1039–49. doi: 10.1111/tpj.12250 (PMC4223383; doi:10.1111/tpj.12250)

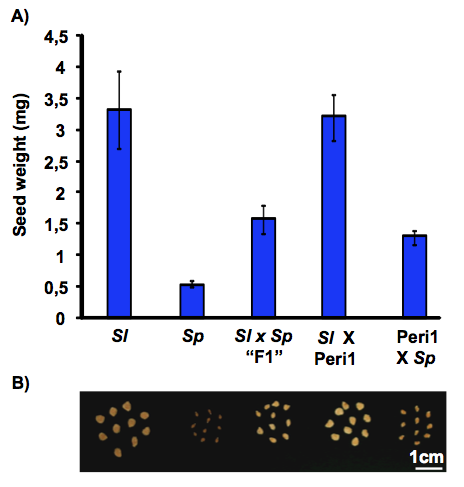

Supplement: Figure S1 — Phenotype of seeds. [file tpj0075-1039-sd1.tiff]

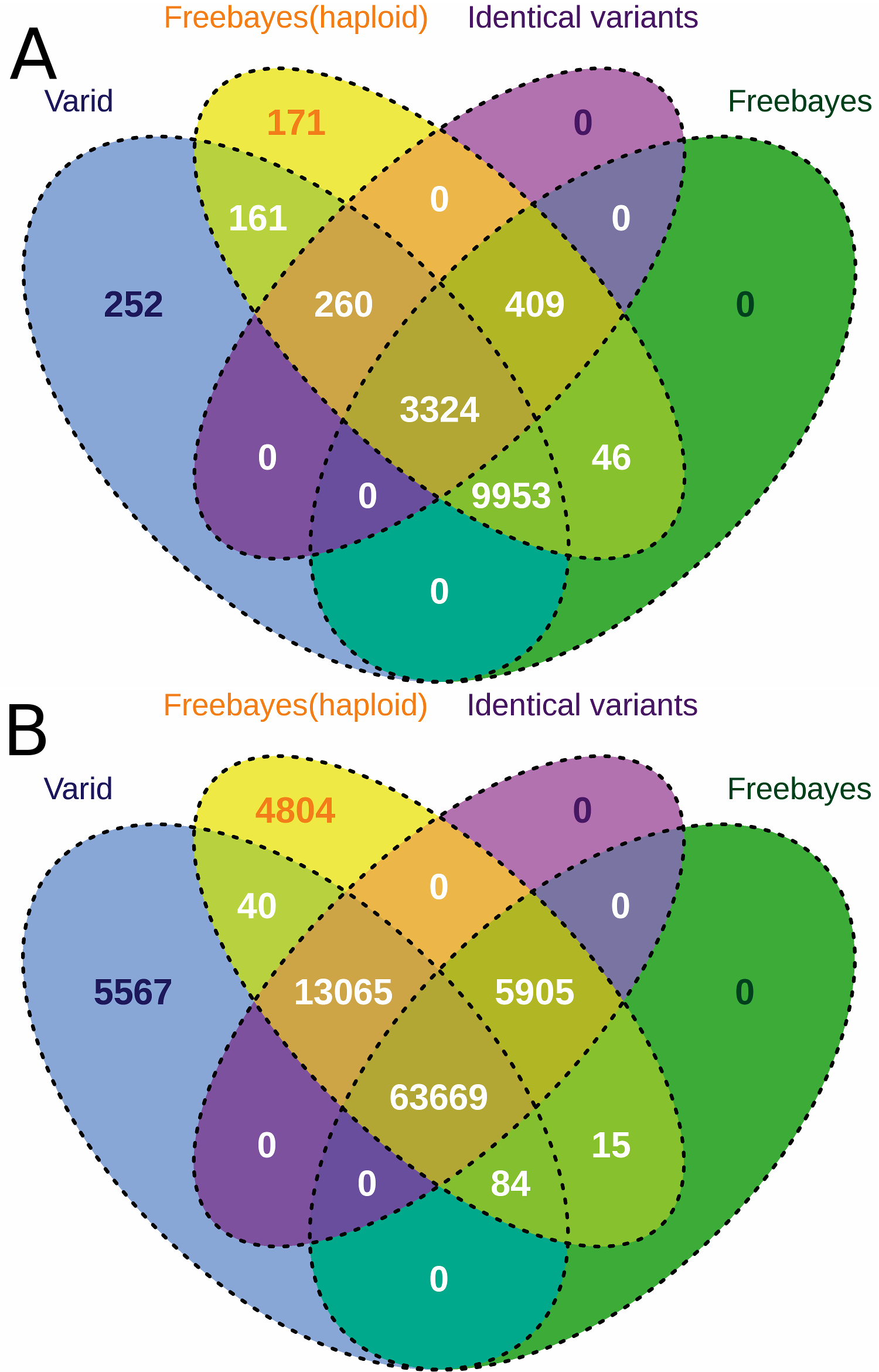

Supplement: Figure S2 — Comparison of variant detection methods. [file tpj0075-1039-sd2.tiff]

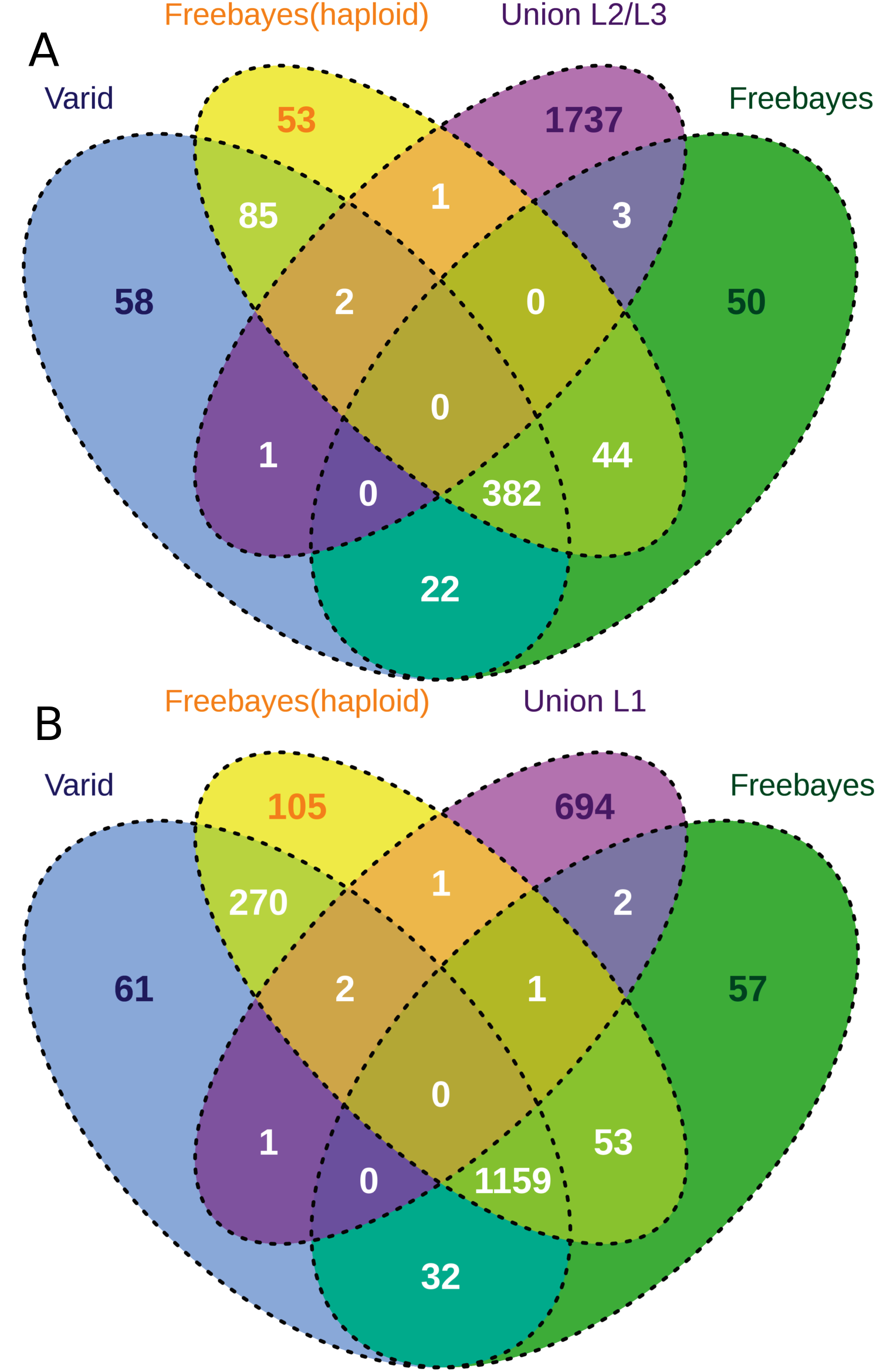

Supplement: Figure S3 — Comparison of gene classifications resulting from all three variant detection methods. [file tpj0075-1039-sd3.tiff]

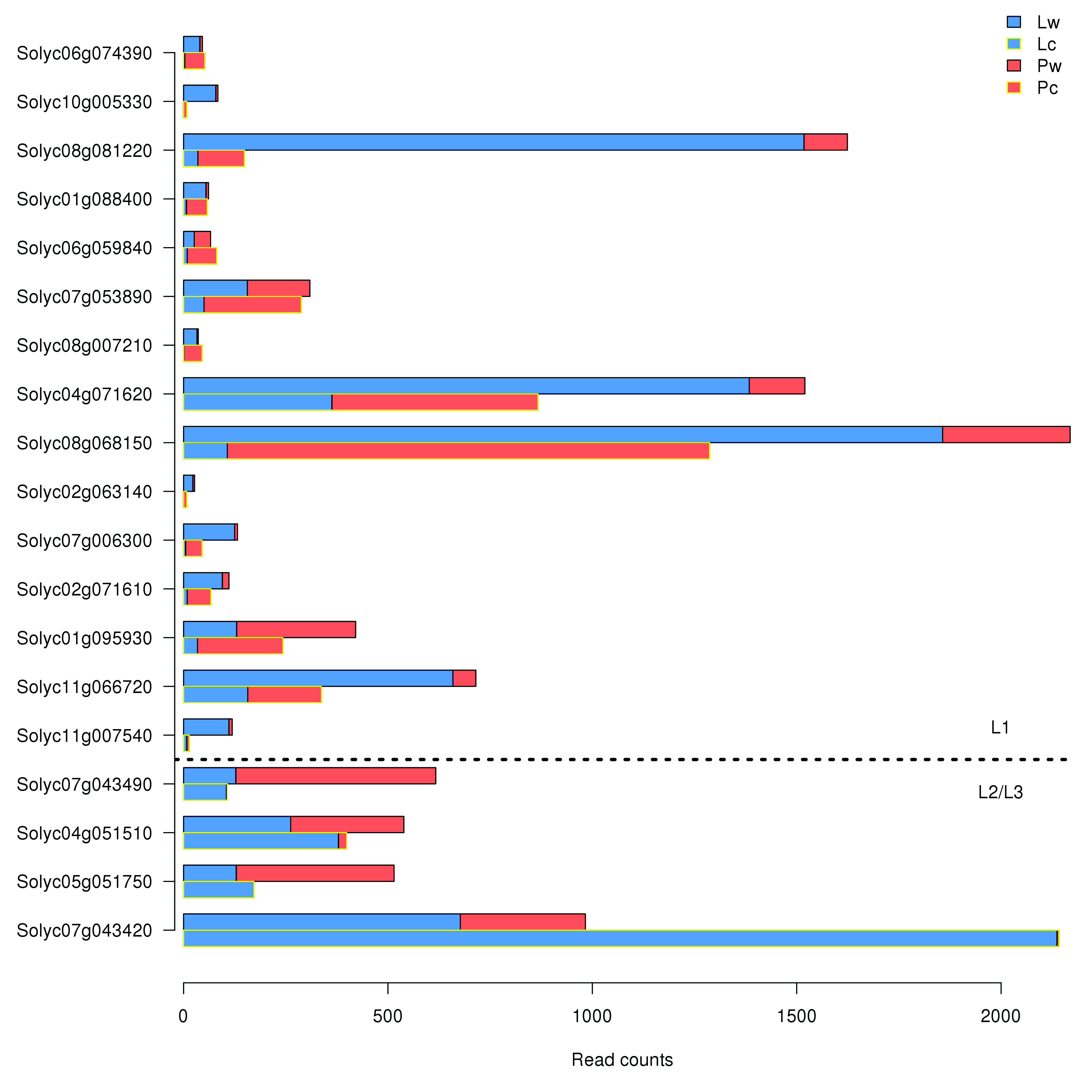

Supplement: Figure S5 — Parental-origin allele-specific expression values for all Sanger-sequenced genes. [file tpj0075-1039-sd5.tiff]

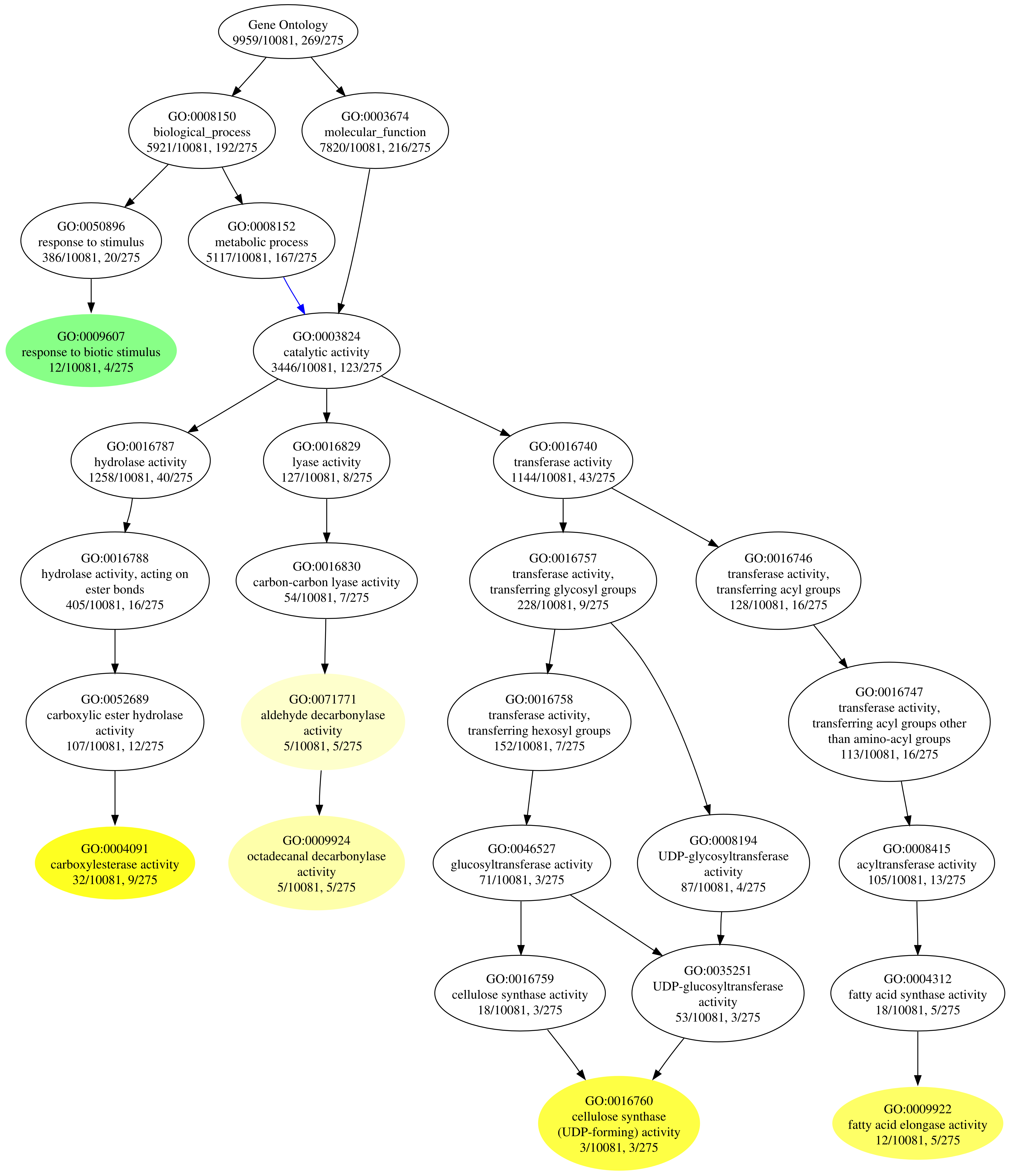

Supplement: Figure S6 — Gene Ontology graph of over-represented terms for layer L1 genes. [file tpj0075-1039-sd6.tiff]

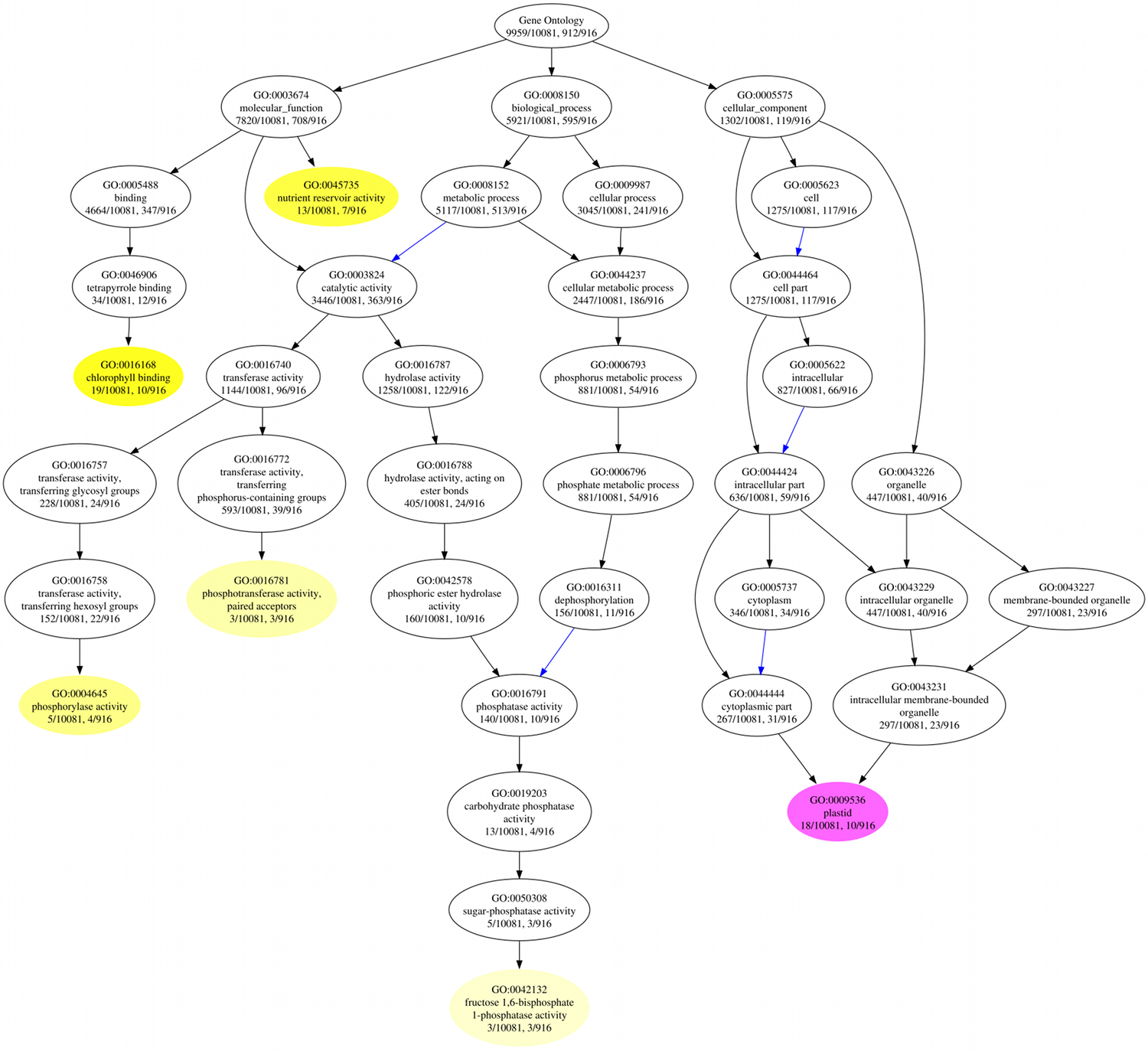

Supplement: Figure S7 — Gene Ontology graph of over-represented terms for layers L2/L3 genes. [file tpj0075-1039-sd7.tiff]

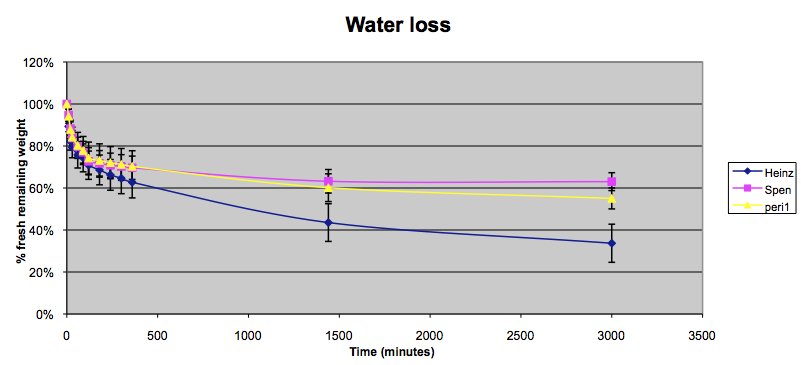

Supplement: Figure S8 — Water loss. [file tpj0075-1039-sd8.tiff]
