# Supplementary material for: Using a periclinal chimera to unravel layer-specific gene expression in plants
Source: Plant J. 2013 Jul 19;75(6):1039–49. doi: 10.1111/tpj.12250 (PMC4223383; doi:10.1111/tpj.12250)
Supplement: Supplementary file 9 [file tpj0075-1039-sd9.docx]

**Supporting information Figure Legends**

**Supporting information Figure S1.**

**Phenotype of seeds**

**A)** Seed weight in mg of the parental lines used to generate the chimera line, *Sl* (*S.* *lycopersicum*), *Sp* (*S.pennellii*), and the crosses *Sl* x *Sp* (F1), *Sl* x chimeric line (Peri1) and Peri1 x S*p*. Data are expressed as means of two independent seed pools with twenty seed of each line measured. Error bars indicate SE. **B)** Morphology of the seeds. Samples are the same as in **(A).**

**Supporting information Figure S2. Comparison of variant detection methods.**

Venn diagrams indicating overlap of genes with variants or overlap of variant positions detected by SNP detection software Varid, FreeBayes and FreeBayes-(haploid setting). The Identical Variants set is the set of genes or polymorphic positions with identical alleles detected by more than one method.

1. Overlap of genes with at least 1 SNP detected.
2. Overlap of variant positions for genes with at least 1 SNP detected by all 3 methods.

**Supporting information Figure S3. Comparison of gene classifications resulting from all three variant detection methods.**

Venn diagrams indicating overlap of genes classified as layer specific based on polymorphisms detected by Varid, FreeBayes and FreeBayes-(haploid setting). The Union sets are the sets of L1 or L2/L3 genes classified based on polymorphisms detected by any method.

A) Overlap of L1 genes among the 3 SNP detection methods and with the union of all L2/L3 genes.

B) Overlap of L2/L3 genes among the 3 SNP detection methods and with the union of all L1 genes.

**Supporting information Figure S4. Comparison of tissue support for all genes classified as L1 or L2/L3.**

Venn diagram indicating the tissue source of RNA-seq data supporting layer specific expression of the genes, with dehydration providing the largest proportion of data.

**Supporting information Figure S5. Parental-origin allele-specific expression values for all Sanger-sequenced genes.**

Raw read counts are reported for all 19 layer-specific genes verified by Sanger sequencing. Read counts are calculated based on polymorphisms detected by Varid and on a single tissue library that support each gene’s classification. Lw and Lc denote the lyc allele-specific expression in the parental genome (lyc) and in chimera, while Pw and Pc denote the penn allele-specific expression in the parental genome (penn) and in the chimera, respectively.

**Supporting information Figure S6. GO-graph of over-represented terms for L1 genes.**

A GO-graph induced by the over-represented terms (coloured nodes). Purple nodes indicate cellular component terms. Green nodes indicate biological process terms. Yellow nodes indicate molecular function terms. Graph is generated using Ontologizer.

**Supporting information Figure S7. GO-graph of over-represented terms for L2/L3 genes.**

A GO-graph induced by the over-represented terms (colored nodes). Purple nodes indicate cellular component terms. Green nodes indicate biological process terms. Yellow nodes indicate molecular function terms. Graph is generated using Ontologizer.

**Supporting information Figure S8. Water loss.**

Water loss was estimated as the percentage of initial FW that remains after excising 8^th^ leaves from *S. lycopersicum* (*S.lyc*), *S. pennellii* (*S.pen*) and periclinal (Peri1) 8 weeks old plants to a dry filter paper and allowing them to dessicate for 24 hours. Data are expressed as arithmetic means of three independent experiments with 6 leaves each. Bars indicate SE.
