# Supplementary material for: Using a periclinal chimera to unravel layer-specific gene expression in plants
Source: Plant J. 2013 Jul 19;75(6):1039–49. doi: 10.1111/tpj.12250 (PMC4223383; doi:10.1111/tpj.12250)
Supplement: Supplementary file 10 — Table S1. Levels of ABA in water-stressed leaves. Table S2. Results from mapping of SOLiD reads for all nine libraries. Table S3. Results from variant detection. Table S4. Parental-origin allele-specific expression values in all samples. Table S5. List of genes classified as layer L1, either specific or related. Table S6. List of genes classified as layers L2/L3, either specific or related. Table S7. Comparison of layer L1 specificity from Sanger sequencing with their Next Generation Sequencing based classification. Table S8. Sequences of primers used. [file tpj0075-1039-sd10.docx]

**Supporting Information Tables**

**Supporting Information Table S1** **ABA levels in water-stressed leaves.**

ABA levels present in the 8^th^ leaf from the apex (control - con) and those after 30% loss of fresh weight (desiccated - des). Plants were ~8 weeks old, *S. lycopersicum* (S.lyc), *S. pennellii* (S.pen) and periclinal (Peri1) plants. Mean values and standard errors of three replicates are provided.

|  | ng ABA/g freeze dried tissue |
| --- | --- |
| Con S.lyc | 26.0±2.1 |
| Des S.lyc | 209.7± 25.4 |
| Con S.pen | 11.6±1.0 |
| Des S.pen | 245.1±27.4 |
| Con Peri1 | 15.0±0.3 |
| Des Peri1 | 93.4±4.9 |

**Supporting Information Table S2. Results from mapping of SOLiD reads for all 9 libraries.**

Information is provided also for pooled samples per plant type.

| **Library** | **Reads** | **% reads**  **mapped to filter reference** | **% reads mapped** | **% valid reads mapped** | **% valid reads mapped to CDS** | **Valid reads mapped to CDS** | **% Genes* covered** | **% CDS bases** covered** | **x Coverage** |
| --- | --- | --- | --- | --- | --- | --- | --- | --- | --- |
|  |  |  |  |  |  |  |  |  |  |
| lyc leaf | 16088186 | 6.9 | 68.6 | 54.2 | 42.6 | 6848676 | 64.22 | 50.09 | 8.52 |
| lyc dehydrated leaf | 20304973 | 4.1 | 72.7 | 59.2 | 45.2 | 9179284 | 62.95 | 45.01 | 11.6 |
| lyc fruit | 19828743 | 1.1 | 73.9 | 59.5 | 45.7 | 9061422 | 61.84 | 47.75 | 11.54 |
| penn leaf | 2940709 | 3.1 | 55.3 | 44.5 | 36.2 | 1064711 | 53.8 | 24.03 | 1.32 |
| penn dehydrated leaf | 22944453 | 4.2 | 55.1 | 45.7 | 36.5 | 8385872 | 64.69 | 46.91 | 10.1 |
| penn fruit | 19267470 | 1 | 53.9 | 29.5 | 18.9 | 3645484 | 62.71 | 38.13 | 4.34 |
| peri leaf | 17578721 | 9 | 56.1 | 37.4 | 26.5 | 4655448 | 61.47 | 39.27 | 5.82 |
| peri dehydrated leaf | 24223072 | 2.8 | 69.5 | 58.1 | 45.5 | 11022859 | 67.44 | 56.4 | 13.53 |
| peri fruit | 18811962 | 1 | 70.6 | 54.6 | 41 | 7707787 | 61.5 | 46.8 | 9.65 |
| lyc all libraries | 56221902 | 3.8 | 72 | 57.9 | 44.6 | 25089382 | 71.01 | 61.9 | 31.66 |
| penn all libraries | 45152632 | 2.8 | 54.6 | 38.7 | 29 | 13096067 | 70.88 | 55.22 | 15.76 |
| peri all libraries | 60613755 | 4 | 65.9 | 51 | 38.6 | 23386094 | 72.1 | 63.01 | 29.01 |

* 34,727 genes

** 35,972,459 CDS bases

**Supporting Information Table S3. Results from variant detection.**

Percentage of polymorphisms is calculated with respect to 12,447,217 bases in CDS with at least 4x coverage in both parental lines.

| **Description** | **Genes** | **% Genes** | **% SNPs** |
| --- | --- | --- | --- |
| All genes | 34727 | 100 |  |
| Expressed in at least one tissue in every plant type | 21938 | 63.17 |  |
| And with at least 1 base with 4x coverage in both parental lines | 17979 | 51.77 |  |
| And with at least 1 SNP detected by all 3 methods | 13277 | 38.23 | 0.51 |
| Or by Varid | 13950 | 40.17 | 0.67 |
| Or by FreeBayes | 13732 | 39.54 | 0.56 |
| Or by FreeBayes (haploid) | 14324 | 41.25 | 0.72 |

**Supporting Information Table S4. Parental-origin allele-specific expression values in all samples.**

Raw read counts are reported for all 21,938 genes found to be expressed in both parental lines and the chimera independent of tissue sample. Number of polymorphisms and allele-specific read counts are calculated based on variant detection by Varid. Lw and Lc denote the lyc allele-specific expression in the parental-genome (lyc) and in the chimera, while Pw and Pc denote the penn allele-specific expression in the parental-genome (penn) and in the chimera, respectively.

*Found in the attached file.*

**Supporting Information Table S5. List of genes classified as L1, either specific or related.**

Within each class, genes are ordered with decreasing fold change with respect to the interaction effect, i.e. the ratio of the difference of penn allele-specific expression between wild type and chimera over the difference for lyc expresssion. Among all tissue samples, the highest fold change is reported. For each gene, we report which tissues support the classification as L1: L(eaf), D(ehydrated leaf), F(ruit). Fold change, p-values, tissue support and classification as related or specific originate from the differential expression analysis conducted on allele-specific expression values based on polymorphims detected by Varid. After correcting for multiple testing using the Benjamini and Hochberg approach (P adjusted value), a cut-off value of 0.05 was applied for the false discovery rate.

*Found in the attached file.*

**Supporting Information Table S6. List of genes classified as L2/L3, either specific or related.**

Within each class, genes are ordered with decreasing fold change with respect to the interaction effect: i.e. the ratio of the difference of penn allele-specific expression between wild type and chimera over the difference for lyc expresssion. Among all tissue samples, the highest fold change is reported. For each gene, we report which tissues support the classification as L2/L3: L(eaf), D(ehydrated leaf), F(ruit). Fold change, p-values, tissue support and classification as related or specific originate from the differential expression analysis conducted on allele-specific expression values based on polymorphims detected by Varid. After correcting for multiple testing using the Benjamini and Hochberg approach (P adjusted value), a cut-off value of 0.05 was applied for the false discovery rate.

*Found in the attached file.*

**Supporting Information Table S7. Comparison of L1 specificity from Sanger sequencing with their Next Generation Sequencing based classification.**

| **Locus name** | **Gene ID** | **NGS**  **Prediction** | **% L1 specificity from Sanger read data** |
| --- | --- | --- | --- |
| Fatty acyl coA reductase | Solyc06g074390.2.1 | L1s | 100±1 |
| Meristem Layer 1 | Solyc10g005330.2.1 | L1s | 100±1 |
| Cytochrome P450 (CYP86A69) | Solyc08g081220.1.1 | L1s | 99±4 |
| CER1 homologue | Solyc01g088400.2.1 | L1s | 99±3 |
| 3-methyl-2-oxobutanoate dehydrogenase | Solyc06g059840.2.1 | L1s | 99±2 |
| O-acyltransferase WSD1 | Solyc07g053890.2.1 | L1s | 99±2 |
| Hydroxycinnamoyl CoA shikimate/quinate hydroxycinnamoyltransferase-like protein | Solyc08g007210.2.1 | L1s | 99±2 |
| ASR4 (SLDS2/SL301 homolog to Arabidopsis RD22) | Solyc04g071620.1.1 | L1s | 99±4 |
| BURP domain-containing protein (SL300) | Solyc08g068150.2.1 | L1s | 99±5 |
| Fatty acid elongase 3-ketoacyl-CoA synthase | Solyc02g063140.2.1 | L1s | 98±5 |
| CER1 homologue | Solyc07g006300.2.1 | L1s | 98±3 |
| GDSL esterase/lipase At5g45670 | Solyc02g071610.2.1 | L1s | 96±22 |
| O-acyltransferase WSD1 | Solyc01g095930.2.1 | L1s | 92±7 |
| Bifunctional polymyxin resistance arnA protein (SL231) | Solyc11g066720.1.1 | L1s | 86±8 |
| Cytochrome P450 CYP77A20 | Solyc11g007540.1.1 | L1s | 83±3 |
| UDP-glucosyltransferase family 1 protein (SGT1) | Solyc07g043490.1.1 | L2/L3r | 7±5 |
| Receptor-like kinase, RLK (BRI1) | Solyc04g051510.1.1 | L2/L3s | 53±5 |
| Prosystemin | Solyc05g051750.2.1 | L2/L3s | 8±2 |
| 2-oxoglutarate-dependent dioxygenase | Solyc07g043420.2.1 | L2/L3s | 3±3 |

**Supporting Information Table S8. Sequences of primers used.**

| **Gene ID** | **Forward Primer** | **Reverse Primer** |
| --- | --- | --- |
| Solyc01g088400.2.1 | 5'-CTACTCTCGTTATCATTCTCATCATCACTC-3' | 5'-CTCTTTGGCACAAGGCAAGGGCAATGGAG-3' |
| Solyc01g095930.2.1 | 5'-TGACGATGATGAAGAAGAAGAAGCAG-3' | 5'-ACCATGTACTGCCATAGCAAGGAC-3' |
| Solyc01g104970.2.1 | 5’-AGCAAGTGGGGTTGTGGTGAAGAGATTAGG-3’ | 5’-TTATAGGCCAGCCAAGTGGTG-3’ |
| Solyc02g063140.2.1 | 5'-TGCTCCTCCCGAATTGTTTGTTCCGAATG-3' | 5'-GGAATATGCACAGGATACTTGTCAATAC-3' |
| Solyc02g071610.2.1 | 5'-ATGGGTGCTGGGGAAGTAAAGAGGAG-3' | 5'-TCAGCTCTATATGATCTTCTCCCAACAAC-3' |
| Solyc02g072300.2.1 | 5’-GTAATGGCTGATGATAAGGAG-3’ | 5’-TCATGCTGGACTCCTCTGCTC-3’ |
| Solyc02g089160.2.1 | 5’-CACAAGTACATGAGGGGTGCATTG-3’ | 5’-TAGTGAAAGAGTGCCTAGCACTAG-3’ |
| Solyc04g051510.1.1 | 5'-GGACCTATTTCTGATATCTCTAGCTTTG-3' | 5'-ATATTCCCTGAGATTGAGTTGTTTCCAAG-3' |
| Solyc04g071620.1.1 | 5'-GAAACATCACTTTGGTGGTCTC-3' | 5'-CTTCCTCTATCTTGTGCTTGTGTG-3' |
| Solyc05g051750.2.1 | 5’-ACTAAGAAAACCATGGGAACTCCTTC-3’ | 5’-GTTTCTACGAGTTTATTGTCTGTTTC-3’ |
| Solyc06g059840.2.1 | 5'-CTCAGGCTGCTGGTGTGGCTTATTC-3' | 5'-ACTTGAAAATCAGTAGGATAGTCTTTAGG-3' |
| Solyc06g074390.2.1 | 5'-TGCATATGTATCAGGGGAAAAAAGAGG-3' | 5'-CAATTCCTGACTCCTTCGCTGCTCTG-3' |
| Solyc07g006300.2.1 | 5'-AACTTGAGGGGTCGATTGCATCAAAC-3' | 5'-AGCTGCATCCCAAACTAAGTCAATTCTG-3' |
| Solyc07g043420.2.1 | 5'-CTTCTCTCGAACTGGTCAAGCAC-3' | 5'-CTTGGATTTGGACATGCTGGATAGTG-3' |
| Solyc07g043490.1.1 | 5'-CCTTCCGTTCTTGTCCGCTGGTC-3' | 5'-GCTCCGTTCCTCCGAAACTCGAAC-3' |
| Solyc07g053890.2.1 | 5'-ACGAGCGATGCAGCGGGAAATATAG-3' | 5'-TGCAATTCTTAGCTTATCCACATAACTCAC-3' |
| Solyc07g055950.2.1 | 5’-CACACCACCCTATCATGCAACTCC-3’ | 5’-CAAGCTCTGGGCTTGAGCCGAC-3’ |
| Solyc08g005640.2.1 | 5’-GATACATTCATTGCTTTGCTTGGTTG-3’ | 5’-AGATCAGATATGGTATAAAACTTCCTC-3’ |
| Solyc08g005680.2.1 | 5’-TAGGAGATGGGCAAAGGATAAGG-3’ | 5’-CTAACTCTTTGATCACCTCCTGTC-3’ |
| Solyc08g007210.2.1 | 5'-AGCTCCATTTTTTTCTCCCAAGTTGCAAG-3' | 5'-TCCACTTTTCTCCTCCTTAATTATCTCCTC-3' |
| Solyc08g066610.2.1 | 5’-ATGCCCAAAATTATACTCTTCCTCTTCC-3’ | 5’-GTACAAATGCAGAGCAGAACAAGTTCCAG-3’ |
| Solyc08g068150.2.1 | 5'-AGTGGCAAGTCATGCAGCTCTTC-3' | 5'-ATACCTGGCTCTTCACATTCTC-3' |
| Solyc08g081220.1.1 | 5'-CATCTGTCGCGTTGAGCTGGTTC-3' | 5'-CAATTCCATTTACTGCAGTGGCTCCG-3' |
| Solyc10g005330.2.1 | 5’-GCAAGTGTTATGGCTAGTAACATC-3’ | 5’-CTTAGGTTTAGGCTAGGCGTTTTCAC-3’ |
| Solyc11g007540.1.1 | 5'-CACAATTTCCGCCATTTTCCTCTC-3' | 5'-GTCACAAGCTCTGCATTCGTCGGTCCTG-3' |
| Solyc11g066720.1.1 | 5'-ATGGCAGGAAGAGTAGATCTGGAC-3' | 5'-GGGAGGTGTCTTCCTTAAGCAC-3' |
| Solyc12g042760.1.1 | 5’-tgctctagtcgatgctccacaaac-3’ | 5’-gtgatccgacccagagagatg-3’ |
| Solyc12g042760.1.1 | 5’-tgctctagtcgatgctccacaaac-3’ | 5’-gtgatccgacccagagagatg-3’ |
