# Supplementary material for: Using a periclinal chimera to unravel layer-specific gene expression in plants
Source: Plant J. 2013 Jul 19;75(6):1039–49. doi: 10.1111/tpj.12250 (PMC4223383; doi:10.1111/tpj.12250)
Supplement: Supplementary file 11 [file tpj0075-1039-sd11.pdf]

Supplemental Table S4. Parental-origin allele-specific expression values in all samples.
Raw read counts are reported for all 21,938 genes found to be expressed in both parental lines and the chimera independent of tissue sample. Number of polymorphisms and allele-specific read counts are calculated based on variant detection by Varid. Lw and Lc denote the lyc allele-specific expression in the parental-genome (lyc) and in the chimera, while Pw and Pc denote the penn allele-specific expression in the parental-genome (penn) and in the chimera, respectively.

Gene ID	# SNPs	Lw Leaf	Pw Leaf	Lc Leaf	Pc Leaf	Lw Dehydrated	Pw Dehydrated	Lc Dehydrated	Pc Dehydrated	Lw Fruit	Pw Fruit	Lc Fruit	Pc Fruit
Solyc00g005000.2.1	0	0	0	0	0	0	0	0	0	0	0	0	0
Solyc00g005040.2.1	0	0	0	0	0	0	0	0	0	0	0	0	0
Solyc00g005050.2.1	1	12	3	5	0	12	30	10	0	8	11	11	0
Solyc00g005440.1.1	0	0	0	0	0	0	0	0	0	0	0	0	0
Solyc00g005840.2.1	6	195	14	99	0	14	31	38	2	36	12	39	1
Solyc00g006470.1.1	0	0	0	0	0	0	0	0	0	0	0	0	0
Solyc00g006490.2.1	22	135	25	112	15	482	252	321	34	233	73	175	4
Solyc00g006670.2.1	0	0	0	0	0	0	0	0	0	0	0	0	0
Solyc00g006680.1.1	0	0	0	0	0	0	0	0	0	0	0	0	0
Solyc00g006690.2.1	0	0	0	0	0	0	0	0	0	0	0	0	0
Solyc00g006800.2.1	15	149	11	119	22	525	181	828	92	998	181	1086	19
Solyc00g006810.2.1	1	6	0	8	0	5	3	3	0	2	0	12	0
Solyc00g006820.2.1	9	39	2	4	21	77	120	26	192	35	15	6	11
Solyc00g006830.2.1	4	31	0	19	4	30	23	112	3	67	12	124	0
Solyc00g007010.2.1	2	10	0	9	0	16	10	19	0	13	0	6	0
Solyc00g007060.2.1	3	28	7	12	0	40	23	18	4	35	19	24	0
Solyc00g007070.2.1	5	122	8	105	4	270	103	236	18	429	54	415	4
Solyc00g007080.2.1	0	0	0	0	0	0	0	0	0	0	0	0	0
Solyc00g007090.2.1	1	3	0	0	0	1	34	3	2	7	2	3	0
Solyc00g007100.2.1	2	13	0	13	0	13	11	29	4	11	3	12	0
Solyc00g007110.2.1	0	0	0	0	0	0	0	0	0	0	0	0	0
Solyc00g007120.2.1	12	21	5	23	3	57	29	51	13	58	11	34	2
Solyc00g007130.2.1	1	1	0	0	0	3	3	7	0	0	0	0	0
Solyc00g007190.2.1	0	0	0	0	0	0	0	0	0	0	0	0	0
Solyc00g007200.2.1	1	6	2	2	2	0	9	1	1	0	0	0	0
Solyc00g007220.2.1	1	4	0	0	0	6	0	4	0	14	6	14	0
Solyc00g007260.2.1	3	10	1	31	0	9	12	32	0	10	5	7	0
Solyc00g007270.2.1	2	86	1	37	0	160	84	110	11	186	24	159	1
Solyc00g007300.2.1	3	21	8	3	1	8	10	4	5	1	4	14	0
Solyc00g008570.2.1	0	0	0	0	0	0	0	0	0	0	0	0	0
Solyc00g008580.1.1	0	0	0	0	0	0	0	0	0	0	0	0	0
Solyc00g008680.2.1	11	231	13	117	10	571	335	500	42	408	72	319	3
Solyc00g009020.2.1	8	229	18	89	7	294	147	467	22	291	50	333	5
Solyc00g009030.1.1	0	0	0	0	0	0	0	0	0	0	0	0	0

Solyc00g009040.2.1	0	0	0	0	0	0	0	0	0	0	0	0	0
Solyc00g009050.1.1	0	0	0	0	0	0	0	0	0	0	0	0	0
Solyc00g009060.2.1	4	50	9	42	0	66	35	106	9	145	6	58	3
Solyc00g009080.2.1	4	17	1	6	0	36	16	22	1	42	12	19	0
Solyc00g009090.2.1	0	0	0	0	0	0	0	0	0	0	0	0	0
Solyc00g009100.2.1	9	50	6	18	0	26	81	56	2	11	5	14	3
Solyc00g009110.2.1	6	12	3	6	0	23	46	27	12	21	0	10	1
Solyc00g009120.2.1	1	1	0	1	0	3	3	4	0	2	0	0	0
Solyc00g009130.2.1	0	0	0	0	0	0	0	0	0	0	0	0	0
Solyc00g009140.2.1	0	0	0	0	0	0	0	0	0	0	0	0	0
Solyc00g009760.2.1	0	0	0	0	0	0	0	0	0	0	0	0	0
Solyc00g009780.1.1	0	0	0	0	0	0	0	0	0	0	0	0	0
Solyc00g009800.1.1	0	0	0	0	0	0	0	0	0	0	0	0	0
Solyc00g010530.1.1	0	0	0	0	0	0	0	0	0	0	0	0	0
Solyc00g010550.1.1	0	0	0	0	0	0	0	0	0	0	0	0	0
Solyc00g011150.1.1	0	0	0	0	0	0	0	0	0	0	0	0	0
Solyc00g011170.1.1	0	0	0	0	0	0	0	0	0	0	0	0	0
Solyc00g011880.2.1	1	2	1	1	0	7	6	2	0	10	3	10	1
Solyc00g011890.2.1	10	40	1	34	6	388	292	313	58	190	45	80	4
Solyc00g012430.1.1	0	0	0	0	0	0	0	0	0	0	0	0	0
Solyc00g012440.1.1	0	0	0	0	0	0	0	0	0	0	0	0	0
Solyc00g013120.2.1	0	0	0	0	0	0	0	0	0	0	0	0	0
Solyc00g013140.1.1	1	1	0	7	4	11	5	8	4	13	5	3	2
Solyc00g013150.1.1	2	1	0	10	0	22	0	15	0	12	3	6	0
Solyc00g013160.1.1	0	0	0	0	0	0	0	0	0	0	0	0	0
Solyc00g013170.1.1	0	0	0	0	0	0	0	0	0	0	0	0	0
Solyc00g013180.1.1	0	0	0	0	0	0	0	0	0	0	0	0	0
Solyc00g013190.1.1	0	0	0	0	0	0	0	0	0	0	0	0	0
Solyc00g014830.2.1	0	0	0	0	0	0	0	0	0	0	0	0	0
Solyc00g014840.1.1	0	0	0	0	0	0	0	0	0	0	0	0	0
Solyc00g014850.1.1	0	0	0	0	0	0	0	0	0	0	0	0	0
Solyc00g017210.1.1	0	0	0	0	0	0	0	0	0	0	0	0	0
Solyc00g018970.1.1	0	0	0	0	0	0	0	0	0	0	0	0	0
Solyc00g019730.1.1	0	0	0	0	0	0	0	0	0	0	0	0	0
Solyc00g019740.1.1	0	0	0	0	0	0	0	0	0	0	0	0	0
Solyc00g019750.1.1	0	0	0	0	0	0	0	0	0	0	0	0	0
Solyc00g019950.1.1	0	0	0	0	0	0	0	0	0	0	0	0	0
Solyc00g019960.1.1	0	0	0	0	0	0	0	0	0	0	0	0	0
Solyc00g019970.2.1	0	0	0	0	0	0	0	0	0	0	0	0	0
Solyc00g019980.2.1	0	0	0	0	0	0	0	0	0	0	0	0	0
Solyc00g020010.1.1	0	0	0	0	0	0	0	0	0	0	0	0	0

Solyc00g020020.2.1	0	0	0	0	0	0	0	0	0	0	0	0	0
Solyc00g020030.1.1	0	0	0	0	0	0	0	0	0	0	0	0	0
Solyc00g020040.1.1	0	0	0	0	0	0	0	0	0	0	0	0	0
Solyc00g021630.1.1	0	0	0	0	0	0	0	0	0	0	0	0	0
Solyc00g021640.2.1	0	0	0	0	0	0	0	0	0	0	0	0	0
Solyc00g022090.1.1	0	0	0	0	0	0	0	0	0	0	0	0	0
Solyc00g022100.1.1	1	1	0	7	0	9	4	6	3	6	7	4	3
Solyc00g022110.2.1	0	0	0	0	0	0	0	0	0	0	0	0	0
Solyc00g024150.2.1	7	736	27	477	4	160	171	226	3	272	26	311	3
Solyc00g025290.1.1	0	0	0	0	0	0	0	0	0	0	0	0	0
Solyc00g026160.2.1	0	0	0	0	0	0	0	0	0	0	0	0	0
Solyc00g027770.2.1	3	26	11	27	5	38	85	98	4	112	29	97	0
Solyc00g028960.1.1	2	17	3	12	0	0	1	0	0	0	0	0	0
Solyc00g029190.2.1	0	0	0	0	0	0	0	0	0	0	0	0	0
Solyc00g030100.1.1	0	0	0	0	0	0	0	0	0	0	0	0	0
Solyc00g030510.2.1	0	0	0	0	0	0	0	0	0	0	0	0	0
Solyc00g030520.2.1	2	2	0	0	2	9	12	4	4	14	3	16	0
Solyc00g036740.1.1	0	0	0	0	0	0	0	0	0	0	0	0	0
Solyc00g039990.2.1	0	0	0	0	0	0	0	0	0	0	0	0	0
Solyc00g042840.1.1	0	0	0	0	0	0	0	0	0	0	0	0	0
Solyc00g044350.2.1	1	38	5	33	0	3	3	4	0	0	11	0	0
Solyc00g047700.2.1	0	0	0	0	0	0	0	0	0	0	0	0	0
Solyc00g048510.2.1	0	0	0	0	0	0	0	0	0	0	0	0	0
Solyc00g049210.1.1	0	0	0	0	0	0	0	0	0	0	0	0	0
Solyc00g049220.1.1	0	0	0	0	0	0	0	0	0	0	0	0	0
Solyc00g049520.2.1	3	18	1	6	0	4	6	10	0	56	0	68	0
Solyc00g050130.1.1	7	49	12	41	11	45	165	69	18	52	23	53	3
Solyc00g050430.2.1	0	0	0	0	0	0	0	0	0	0	0	0	0
Solyc00g052430.2.1	0	0	0	0	0	0	0	0	0	0	0	0	0
Solyc00g052940.2.1	0	0	0	0	0	0	0	0	0	0	0	0	0
Solyc00g053440.2.1	2	29	2	9	0	38	11	21	6	8	2	18	0
Solyc00g056680.1.1	0	0	0	0	0	0	0	0	0	0	0	0	0
Solyc00g059100.2.1	0	0	0	0	0	0	0	0	0	0	0	0	0
Solyc00g059500.1.1	0	0	0	0	0	0	0	0	0	0	0	0	0
Solyc00g060700.1.1	0	0	0	0	0	0	0	0	0	0	0	0	0
Solyc00g068970.2.1	1	0	0	0	11	0	1	0	0	1	53	0	27
Solyc00g068980.1.1	0	0	0	0	0	0	0	0	0	0	0	0	0
Solyc00g069880.2.1	0	0	0	0	0	0	0	0	0	0	0	0	0
Solyc00g071180.2.1	0	0	0	0	0	0	0	0	0	0	0	0	0
Solyc00g072100.2.1	0	0	0	0	0	0	0	0	0	0	0	0	0
Solyc00g075440.1.1	0	0	0	0	0	0	0	0	0	0	0	0	0

Solyc00g075450.2.1	0	0	0	0	0	0	0	0	0	0	0	0	0
Solyc00g080750.2.1	1	3	0	0	0	9	5	4	2	0	0	0	0
Solyc00g082150.2.1	2	33	9	20	0	16	24	27	5	95	17	93	0
Solyc00g082550.1.1	0	0	0	0	0	0	0	0	0	0	0	0	0
Solyc00g085070.2.1	7	301	5	125	1	119	38	139	5	120	14	77	2
Solyc00g090430.2.1	0	0	0	0	0	0	0	0	0	0	0	0	0
Solyc00g090870.2.1	4	19	0	15	0	29	12	43	4	12	8	4	0
Solyc00g091170.2.1	1	5	0	0	0	1	9	4	0	1	0	0	0
Solyc00g094520.1.1	0	0	0	0	0	0	0	0	0	0	0	0	0
Solyc00g094530.1.1	0	0	0	0	0	0	0	0	0	0	0	0	0
Solyc00g094540.1.1	0	0	0	0	0	0	0	0	0	0	0	0	0
Solyc00g094850.1.1	0	0	0	0	0	0	0	0	0	0	0	0	0
Solyc00g095460.1.1	0	0	0	0	0	0	0	0	0	0	0	0	0
Solyc00g096460.1.1	1	1	0	1	0	1	2	10	1	4	2	4	0
Solyc00g097960.1.1	0	0	0	0	0	0	0	0	0	0	0	0	0
Solyc00g098560.2.1	0	0	0	0	0	0	0	0	0	0	0	0	0
Solyc00g099580.1.1	0	0	0	0	0	0	0	0	0	0	0	0	0
Solyc00g102000.1.1	0	0	0	0	0	0	0	0	0	0	0	0	0
Solyc00g106650.1.1	0	0	0	0	0	0	0	0	0	0	0	0	0
Solyc00g108550.1.1	0	0	0	0	0	0	0	0	0	0	0	0	0
Solyc00g110870.2.1	0	0	0	0	0	0	0	0	0	0	0	0	0
Solyc00g118690.1.1	0	0	0	0	0	0	0	0	0	0	0	0	0
Solyc00g125980.1.1	0	0	0	0	0	0	0	0	0	0	0	0	0
Solyc00g125990.1.1	0	0	0	0	0	0	0	0	0	0	0	0	0
Solyc00g126000.1.1	0	0	0	0	0	0	0	0	0	0	0	0	0
Solyc00g126010.1.1	0	0	0	0	0	0	0	0	0	0	0	0	0
Solyc00g127660.2.1	1	4	0	0	0	22	13	6	6	1	7	8	0
Solyc00g127670.1.1	0	0	0	0	0	0	0	0	0	0	0	0	0
Solyc00g127870.2.1	1	8	0	0	0	9	5	0	2	2	1	4	0
Solyc00g128870.2.1	1	13	0	3	0	17	3	27	0	14	0	14	0
Solyc00g136260.1.1	2	15	6	10	0	46	185	127	2	93	12	52	2
Solyc00g136560.2.1	7	38	40	19	1	1029	408	1289	18	8	3	2	0
Solyc00g140060.2.1	2	10	1	3	0	18	26	66	0	27	6	25	0
Solyc00g142160.1.1	0	0	0	0	0	0	0	0	0	0	0	0	0
Solyc00g142170.2.1	0	0	0	0	0	0	0	0	0	0	0	0	0
Solyc00g143770.1.1	0	0	0	0	0	0	0	0	0	0	0	0	0
Solyc00g154980.1.1	0	0	0	0	0	0	0	0	0	0	0	0	0
Solyc00g156980.2.1	0	0	0	0	0	0	0	0	0	0	0	0	0
Solyc00g157080.1.1	0	0	0	0	0	0	0	0	0	0	0	0	0
Solyc00g157180.2.1	0	0	0	0	0	0	0	0	0	0	0	0	0
Solyc00g160780.2.1	0	0	0	0	0	0	0	0	0	0	0	0	0

Solyc00g164680.1.1	0	0	0	0	0	0	0	0	0	0	0	0	0
Solyc00g170200.1.1	1	10	0	0	2	7	10	2	12	0	0	0	0
Solyc00g171810.2.1	0	0	0	0	0	0	0	0	0	0	0	0	0
Solyc00g174330.2.1	0	0	0	0	0	0	0	0	0	0	0	0	0
Solyc00g174340.1.1	0	0	0	0	0	0	0	0	0	0	0	0	0
Solyc00g178340.1.1	0	0	0	0	0	0	0	0	0	0	0	0	0
Solyc00g179240.1.1	2	11	1	7	0	6	5	5	3	0	0	0	0
Solyc00g183050.1.1	0	0	0	0	0	0	0	0	0	0	0	0	0
Solyc00g184350.2.1	0	0	0	0	0	0	0	0	0	0	0	0	0
Solyc00g186050.1.1	0	0	0	0	0	0	0	0	0	0	0	0	0
Solyc00g196060.2.1	4	114	1	77	11	412	103	516	13	439	20	131	0
Solyc00g201160.2.1	0	0	0	0	0	0	0	0	0	0	0	0	0
Solyc00g203660.1.1	0	0	0	0	0	0	0	0	0	0	0	0	0
Solyc00g206460.1.1	0	0	0	0	0	0	0	0	0	0	0	0	0
Solyc00g217960.1.1	0	0	0	0	0	0	0	0	0	0	0	0	0
Solyc00g227860.1.1	2	25	0	13	2	7	4	20	1	65	3	89	0
Solyc00g230060.1.1	0	0	0	0	0	0	0	0	0	0	0	0	0
Solyc00g233480.1.1	0	0	0	0	0	0	0	0	0	0	0	0	0
Solyc00g247300.2.1	7	0	0	5	0	12	48	15	0	61	1	68	0
Solyc00g249900.2.1	0	0	0	0	0	0	0	0	0	0	0	0	0
Solyc00g256710.1.1	0	0	0	0	0	0	0	0	0	0	0	0	0
Solyc00g257110.2.1	0	0	0	0	0	0	0	0	0	0	0	0	0
Solyc00g265510.1.1	2	0	1	0	5	0	1	0	4	167	2251	1	11
Solyc00g272810.1.1	5	114	2	23	0	336	65	518	2	4	2	2	0
Solyc00g278110.2.1	0	0	0	0	0	0	0	0	0	0	0	0	0
Solyc00g282510.1.1	1	5	0	5	0	1	13	17	0	0	0	2	0
Solyc00g289230.1.1	1	2	4	6	0	3	1	8	0	0	0	0	0
Solyc00g311230.2.1	0	0	0	0	0	0	0	0	0	0	0	0	0
Solyc00g313930.1.1	2	3	0	2	0	0	0	0	0	0	19	1	1
Solyc00g316530.1.1	0	0	0	0	0	0	0	0	0	0	0	0	0
Solyc00g320430.1.1	0	0	0	0	0	0	0	0	0	0	0	0	0
Solyc00g323130.2.1	0	0	0	0	0	0	0	0	0	0	0	0	0
Solyc01g005000.2.1	11	143	5	29	0	22	228	47	0	215	145	123	19
Solyc01g005010.2.1	2	28	0	13	0	20	16	32	1	12	2	9	0
Solyc01g005020.2.1	14	48	9	47	3	216	68	294	12	189	45	174	6
Solyc01g005030.2.1	21	85	6	92	1	174	135	346	9	299	76	274	2
Solyc01g005040.2.1	1	3	0	3	0	5	17	1	1	0	0	0	0
Solyc01g005060.2.1	2	11	0	8	0	3	3	7	0	4	0	3	0
Solyc01g005080.2.1	11	142	5	136	5	60	69	106	15	94	101	98	0
Solyc01g005100.2.1	3	18	1	4	1	16	11	27	0	17	7	26	0
Solyc01g005110.2.1	0	0	0	0	0	0	0	0	0	0	0	0	0

Solyc01g005120.2.1	8	108	15	33	5	92	34	104	17	75	26	80	0
Solyc01g005130.2.1	3	39	1	24	0	48	17	68	5	25	1	5	0
Solyc01g005140.2.1	3	3	0	5	2	23	114	20	6	9	28	13	0
Solyc01g005150.2.1	0	0	0	0	0	0	0	0	0	0	0	0	0
Solyc01g005160.2.1	0	0	0	0	0	0	0	0	0	0	0	0	0
Solyc01g005180.1.1	0	0	0	0	0	0	0	0	0	0	0	0	0
Solyc01g005190.1.1	0	0	0	0	0	0	0	0	0	0	0	0	0
Solyc01g005200.1.1	0	0	0	0	0	0	0	0	0	0	0	0	0
Solyc01g005210.2.1	15	161	34	143	18	360	727	833	42	403	64	179	3
Solyc01g005220.2.1	2	6	0	5	0	30	12	32	2	9	1	25	0
Solyc01g005230.2.1	0	0	0	0	0	0	0	0	0	0	0	0	0
Solyc01g005240.2.1	9	41	11	43	1	83	94	181	9	131	62	191	0
Solyc01g005250.2.1	6	89	5	80	4	90	72	191	8	246	25	164	0
Solyc01g005260.2.1	0	0	0	0	0	0	0	0	0	0	0	0	0
Solyc01g005270.2.1	1	15	1	3	0	16	3	12	0	3	0	2	0
Solyc01g005280.2.1	0	0	0	0	0	0	0	0	0	0	0	0	0
Solyc01g005300.2.1	3	39	0	45	0	61	30	103	2	238	6	152	7
Solyc01g005330.2.1	7	162	20	74	22	76	151	176	13	149	39	94	7
Solyc01g005340.2.1	9	24	5	30	5	49	91	102	16	85	24	69	0
Solyc01g005350.2.1	0	0	0	0	0	0	0	0	0	0	0	0	0
Solyc01g005370.2.1	1	3	0	0	0	2	0	2	0	4	8	2	0
Solyc01g005380.2.1	7	23	0	22	0	22	47	45	2	52	20	19	0
Solyc01g005390.2.1	0	0	0	0	0	0	0	0	0	0	0	0	0
Solyc01g005400.2.1	2	14	0	5	0	9	23	7	0	0	0	1	0
Solyc01g005410.2.1	1	22	0	8	0	80	5	26	3	6	0	12	0
Solyc01g005420.2.1	2	42	8	25	2	19	2	1	0	0	0	2	0
Solyc01g005430.2.1	3	8	6	13	0	30	34	21	4	11	8	6	0
Solyc01g005440.2.1	7	1703	43	507	6	644	276	704	29	1058	89	467	23
Solyc01g005450.2.1	2	3	2	10	0	4	25	10	0	2	1	2	0
Solyc01g005460.2.1	1	3	2	6	2	0	0	2	0	6	3	0	0
Solyc01g005470.2.1	4	115	14	34	3	22	90	8	3	10	0	0	0
Solyc01g005480.2.1	3	14	2	11	0	9	7	5	0	19	8	11	0
Solyc01g005490.2.1	2	10	2	6	1	3	19	5	2	23	13	14	1
Solyc01g005500.2.1	0	0	0	0	0	0	0	0	0	0	0	0	0
Solyc01g005510.2.1	1	0	5	1	11	5	0	2	0	2	12	3	0
Solyc01g005520.2.1	9	434	69	208	4	73	163	133	5	55	35	112	6
Solyc01g005530.2.1	4	17	3	13	0	13	12	20	0	22	9	13	0
Solyc01g005540.2.1	10	29	9	15	2	36	52	38	4	17	3	23	0
Solyc01g005550.2.1	5	101	7	55	0	10	34	60	4	96	24	80	3
Solyc01g005560.2.1	9	725	77	421	75	1298	1304	1914	161	446	160	410	1
Solyc01g005570.2.1	1	7	1	14	0	11	6	7	1	7	2	2	0

Solyc01g005580.2.1	3	40	2	14	0	23	16	54	4	61	21	55	0
Solyc01g005590.2.1	0	0	0	0	0	0	0	0	0	0	0	0	0
Solyc01g005600.2.1	9	141	24	111	10	322	252	315	42	236	65	253	9
Solyc01g005620.2.1	4	704	46	242	19	623	153	399	16	1099	63	575	11
Solyc01g005640.2.1	5	29	1	15	1	39	30	42	1	50	33	79	0
Solyc01g005670.2.1	2	6	0	8	0	39	14	35	0	3	3	17	0
Solyc01g005680.2.1	0	0	0	0	0	0	0	0	0	0	0	0	0
Solyc01g005690.2.1	6	38	4	22	1	42	20	62	2	118	34	108	0
Solyc01g005700.2.1	5	14	2	6	0	15	14	18	2	30	7	40	4
Solyc01g005720.2.1	0	0	0	0	0	0	0	0	0	0	0	0	0
Solyc01g005730.2.1	0	0	0	0	0	0	0	0	0	0	0	0	0
Solyc01g005760.2.1	1	15	0	10	0	8	4	3	0	0	0	1	0
Solyc01g005780.1.1	0	0	0	0	0	0	0	0	0	0	0	0	0
Solyc01g005790.1.1	0	0	0	0	0	0	0	0	0	0	0	0	0
Solyc01g005800.2.1	0	0	0	0	0	0	0	0	0	0	0	0	0
Solyc01g005810.2.1	0	0	0	0	0	0	0	0	0	0	0	0	0
Solyc01g005820.2.1	5	362	32	153	9	402	176	704	48	602	80	491	8
Solyc01g005830.2.1	4	48	0	13	0	1	16	16	1	27	1	14	0
Solyc01g005840.2.1	3	20	1	8	12	8	43	22	3	27	7	21	0
Solyc01g005850.1.1	0	0	0	0	0	0	0	0	0	0	0	0	0
Solyc01g005860.2.1	0	0	0	0	0	0	0	0	0	0	0	0	0
Solyc01g005870.1.1	1	9	6	4	0	5	0	3	0	1	1	3	0
Solyc01g005900.2.1	0	0	0	0	0	0	0	0	0	0	0	0	0
Solyc01g005910.2.1	4	26	1	28	0	50	19	52	1	31	1	15	0
Solyc01g005920.2.1	2	5	1	4	0	7	3	11	5	14	0	12	0
Solyc01g005930.2.1	6	47	6	47	0	149	58	93	6	65	25	71	0
Solyc01g005950.2.1	2	12	2	2	0	9	9	12	6	13	7	11	0
Solyc01g005960.2.1	10	92	10	98	14	446	270	246	27	195	106	170	16
Solyc01g005970.2.1	0	0	0	0	0	0	0	0	0	0	0	0	0
Solyc01g005980.2.1	1	0	2	0	0	9	6	9	1	1	0	0	0
Solyc01g005990.2.1	9	262	15	28	55	11	181	22	125	63	14	20	13
Solyc01g006000.2.1	8	20	2	15	8	66	87	91	10	72	9	69	3
Solyc01g006010.2.1	20	40	5	38	2	152	92	149	8	101	30	73	4
Solyc01g006020.2.1	8	305	14	162	2	224	633	487	7	192	10	160	3
Solyc01g006030.2.1	5	27	1	21	2	87	17	66	8	41	14	21	3
Solyc01g006040.2.1	5	6	5	8	0	33	9	30	1	16	6	24	0
Solyc01g006050.2.1	0	0	0	0	0	0	0	0	0	0	0	0	0
Solyc01g006070.2.1	0	0	0	0	0	0	0	0	0	0	0	0	0
Solyc01g006080.1.1	0	0	0	0	0	0	0	0	0	0	0	0	0
Solyc01g006090.2.1	0	0	0	0	0	0	0	0	0	0	0	0	0
Solyc01g006100.2.1	0	0	0	0	0	0	0	0	0	0	0	0	0

Solyc01g006110.2.1	7	68	6	33	2	148	71	138	13	73	20	90	0
Solyc01g006120.2.1	5	73	3	48	6	137	43	141	9	79	12	74	1
Solyc01g006130.2.1	3	60	1	46	0	14	23	29	0	97	3	96	0
Solyc01g006140.2.1	0	0	0	0	0	0	0	0	0	0	0	0	0
Solyc01g006150.2.1	9	79	5	16	0	331	73	173	6	109	23	76	4
Solyc01g006160.2.1	0	0	0	0	0	0	0	0	0	0	0	0	0
Solyc01g006170.2.1	4	13	1	4	0	55	1	41	7	24	10	22	0
Solyc01g006180.2.1	1	1	0	1	0	21	3	25	5	14	2	3	0
Solyc01g006190.2.1	9	139	9	37	0	298	110	214	21	264	22	177	1
Solyc01g006210.2.1	0	0	0	0	0	0	0	0	0	0	0	0	0
Solyc01g006220.2.1	0	0	0	0	0	0	0	0	0	0	0	0	0
Solyc01g006230.2.1	8	132	14	66	6	281	223	289	27	91	37	86	0
Solyc01g006240.2.1	0	0	0	0	0	0	0	0	0	0	0	0	0
Solyc01g006250.1.1	0	0	0	0	0	0	0	0	0	0	0	0	0
Solyc01g006260.2.1	0	0	0	0	0	0	0	0	0	0	0	0	0
Solyc01g006280.2.1	10	192	27	134	29	369	810	713	92	256	113	254	5
Solyc01g006290.2.1	2	93	6	9	3	83	2	4	2	0	24	0	0
Solyc01g006300.2.1	9	546	17	53	65	577	59	39	94	1	0	0	0
Solyc01g006320.2.1	2	19	6	4	0	10	1	6	1	2	0	0	0
Solyc01g006330.2.1	3	52	10	45	0	2	4	4	0	1	2	2	0
Solyc01g006340.2.1	2	4	0	1	0	6	5	7	0	6	2	10	0
Solyc01g006350.2.1	16	58	13	66	15	281	169	334	15	243	43	182	0
Solyc01g006360.2.1	7	15	8	14	4	92	149	174	20	75	34	34	0
Solyc01g006370.2.1	19	78	12	32	0	51	84	126	10	474	64	494	4
Solyc01g006380.2.1	0	0	0	0	0	0	0	0	0	0	0	0	0
Solyc01g006400.2.1	5	344	90	546	105	202	256	262	31	18	6	10	1
Solyc01g006430.2.1	10	564	43	269	6	417	270	554	105	2517	463	1002	0
Solyc01g006440.1.1	0	0	0	0	0	0	0	0	0	0	0	0	0
Solyc01g006450.2.1	3	29	12	23	5	19	45	43	9	90	10	89	0
Solyc01g006480.2.1	1	2	0	1	0	4	6	3	0	1	1	1	1
Solyc01g006490.2.1	9	37	2	36	3	87	42	148	6	89	27	78	0
Solyc01g006500.2.1	1	9	3	7	0	12	25	16	0	12	1	7	0
Solyc01g006510.2.1	7	319	18	177	2	156	156	271	9	273	2	188	0
Solyc01g006520.2.1	4	20	8	18	0	9	4	5	2	20	6	23	0
Solyc01g006530.1.1	0	0	0	0	0	0	0	0	0	0	0	0	0
Solyc01g006540.2.1	6	96	1	0	0	12	0	1	0	95	290	48	1
Solyc01g006550.2.1	0	0	0	0	0	0	0	0	0	0	0	0	0
Solyc01g006560.2.1	7	111	17	181	0	126	17	108	0	1	0	1	0
Solyc01g006580.2.1	0	0	0	0	0	0	0	0	0	0	0	0	0
Solyc01g006630.2.1	4	16	4	20	0	38	56	50	1	72	8	60	0
Solyc01g006640.1.1	0	0	0	0	0	0	0	0	0	0	0	0	0

Solyc01g006650.1.1	0	0	0	0	0	0	0	0	0	0	0	0	0
Solyc01g006660.1.1	0	0	0	0	0	0	0	0	0	0	0	0	0
Solyc01g006670.2.1	6	5	0	8	0	15	28	18	8	25	14	7	0
Solyc01g006680.2.1	7	9	0	1	0	47	89	56	2	138	7	42	1
Solyc01g006690.2.1	3	41	4	25	2	3	15	9	2	12	3	26	3
Solyc01g006700.2.1	7	97	1	40	0	106	51	154	14	160	12	122	7
Solyc01g006710.2.1	10	40	9	29	5	94	92	90	12	99	28	142	3
Solyc01g006720.2.1	14	216	16	103	12	620	233	513	51	28	7	15	0
Solyc01g006730.2.1	0	0	0	0	0	0	0	0	0	0	0	0	0
Solyc01g006740.2.1	5	15	5	4	0	13	36	19	1	42	5	20	0
Solyc01g006780.2.1	0	0	0	0	0	0	0	0	0	0	0	0	0
Solyc01g006790.2.1	1	17	0	6	0	13	10	13	0	9	0	1	0
Solyc01g006800.2.1	7	52	5	43	0	85	90	149	15	150	21	95	0
Solyc01g006810.2.1	3	16	1	15	2	20	15	24	6	43	9	27	2
Solyc01g006820.2.1	0	0	0	0	0	0	0	0	0	0	0	0	0
Solyc01g006830.2.1	14	249	14	178	13	23	64	68	8	432	51	293	2
Solyc01g006840.2.1	0	0	0	0	0	0	0	0	0	0	0	0	0
Solyc01g006850.1.1	1	5	0	3	0	1	6	6	0	1	0	1	0
Solyc01g006860.2.1	2	2	2	2	0	9	12	7	1	14	4	19	0
Solyc01g006870.2.1	6	29	4	13	12	39	35	38	7	36	1	14	0
Solyc01g006880.2.1	14	25	5	17	1	107	71	50	10	43	13	54	1
Solyc01g006890.2.1	2	22	0	5	0	2	4	9	1	8	7	3	0
Solyc01g006900.2.1	6	671	54	296	12	859	491	1362	61	943	261	538	30
Solyc01g006920.2.1	0	0	0	0	0	0	0	0	0	0	0	0	0
Solyc01g006930.2.1	3	77	3	82	0	123	18	75	2	53	5	25	0
Solyc01g006940.2.1	7	124	13	107	0	64	81	127	2	166	9	125	2
Solyc01g006950.2.1	1	5	3	4	0	8	1	4	0	1	1	1	0
Solyc01g006960.2.1	17	70	10	67	10	153	103	141	18	129	47	78	3
Solyc01g006970.2.1	8	30	6	36	10	58	66	105	9	110	9	97	0
Solyc01g006980.2.1	8	65	4	36	2	110	42	122	19	125	15	87	2
Solyc01g006990.2.1	6	39	1	15	1	100	48	68	6	55	18	51	0
Solyc01g007000.2.1	0	0	0	0	0	0	0	0	0	0	0	0	0
Solyc01g007010.2.1	0	0	0	0	0	0	0	0	0	0	0	0	0
Solyc01g007020.2.1	0	0	0	0	0	0	0	0	0	0	0	0	0
Solyc01g007030.2.1	0	0	0	0	0	0	0	0	0	0	0	0	0
Solyc01g007040.2.1	2	28	12	8	0	0	0	1	0	0	0	4	0
Solyc01g007050.1.1	0	0	0	0	0	0	0	0	0	0	0	0	0
Solyc01g007060.2.1	3	9	1	9	1	41	9	33	3	27	10	30	1
Solyc01g007070.2.1	20	365	66	435	42	2115	682	3093	195	1362	470	1088	20
Solyc01g007100.2.1	18	219	17	173	4	71	85	150	28	72	38	67	1
Solyc01g007110.2.1	6	98	9	38	3	420	86	353	20	134	17	113	6

Solyc01g007120.2.1	8	24	2	25	1	65	44	98	9	57	22	79	0
Solyc01g007130.2.1	9	61	5	76	1	45	21	100	0	113	37	103	5
Solyc01g007140.2.1	6	9	2	1	8	34	66	45	17	34	30	17	5
Solyc01g007150.2.1	10	58	2	48	15	81	60	183	14	274	20	229	3
Solyc01g007160.2.1	0	0	0	0	0	0	0	0	0	0	0	0	0
Solyc01g007170.2.1	0	0	0	0	0	0	0	0	0	0	0	0	0
Solyc01g007180.2.1	0	0	0	0	0	0	0	0	0	0	0	0	0
Solyc01g007190.2.1	5	9	5	9	0	27	12	13	5	13	5	8	0
Solyc01g007200.2.1	0	0	0	0	0	0	0	0	0	0	0	0	0
Solyc01g007220.2.1	17	342	10	120	7	245	42	305	4	1214	172	1144	6
Solyc01g007230.2.1	4	24	3	6	0	46	9	35	4	38	2	24	0
Solyc01g007240.2.1	0	0	0	0	0	0	0	0	0	0	0	0	0
Solyc01g007250.2.1	0	0	0	0	0	0	0	0	0	0	0	0	0
Solyc01g007260.2.1	1	1	0	0	0	8	4	6	0	1	0	0	0
Solyc01g007740.2.1	7	127	13	84	3	95	61	214	0	349	38	453	5
Solyc01g007750.2.1	4	51	6	19	1	16	33	23	0	44	16	34	0
Solyc01g007760.2.1	2	20	0	1	0	10	1	13	0	43	15	14	0
Solyc01g007770.2.1	1	38	0	10	0	42	11	108	0	6	1	2	0
Solyc01g007780.2.1	0	0	0	0	0	0	0	0	0	0	0	0	0
Solyc01g007790.2.1	3	18	4	10	3	29	48	42	5	127	6	50	1
Solyc01g007800.2.1	0	0	0	0	0	0	0	0	0	0	0	0	0
Solyc01g007810.1.1	0	0	0	0	0	0	0	0	0	0	0	0	0
Solyc01g007820.1.1	0	0	0	0	0	0	0	0	0	0	0	0	0
Solyc01g007830.1.1	0	0	0	0	0	0	0	0	0	0	0	0	0
Solyc01g007840.2.1	0	0	0	0	0	0	0	0	0	0	0	0	0
Solyc01g007850.2.1	1	0	0	2	0	1	5	2	0	3	0	1	0
Solyc01g007860.2.1	3	220	8	65	3	189	74	161	13	481	77	257	4
Solyc01g007870.2.1	0	0	0	0	0	0	0	0	0	0	0	0	0
Solyc01g007880.2.1	5	46	1	13	0	11	21	25	1	26	9	28	3
Solyc01g007890.1.1	5	22	3	24	0	32	12	33	0	1	6	0	0
Solyc01g007900.2.1	1	7	0	2	0	1	4	13	1	33	2	11	0
Solyc01g007910.2.1	4	48	3	4	0	139	37	141	14	93	34	54	7
Solyc01g007920.2.1	1	5	0	2	0	4	2	3	0	32	5	20	0
Solyc01g007930.2.1	2	4	0	1	0	1	1	11	0	17	0	17	0
Solyc01g007940.2.1	0	0	0	0	0	0	0	0	0	0	0	0	0
Solyc01g007950.2.1	0	0	0	0	0	0	0	0	0	0	0	0	0
Solyc01g007960.2.1	0	0	0	0	0	0	0	0	0	0	0	0	0
Solyc01g007980.2.1	2	9	1	4	2	5	0	2	0	11	6	18	0
Solyc01g007990.2.1	4	21	3	6	0	56	12	29	1	34	18	32	6
Solyc01g008000.2.1	2	188	9	56	3	120	96	175	20	1080	33	676	9
Solyc01g008010.2.1	2	29	6	12	0	69	21	32	1	19	4	3	0

Solyc01g008020.2.1	6	46	3	27	1	23	21	52	2	32	2	10	1
Solyc01g008040.1.1	0	0	0	0	0	0	0	0	0	0	0	0	0
Solyc01g008050.2.1	0	0	0	0	0	0	0	0	0	0	0	0	0
Solyc01g008060.2.1	2	20	0	3	1	19	14	25	0	22	8	8	5
Solyc01g008070.2.1	13	223	33	144	7	1284	1015	1498	48	363	69	210	2
Solyc01g008080.2.1	0	0	0	0	0	0	0	0	0	0	0	0	0
Solyc01g008090.2.1	1	27	11	12	8	16	38	35	13	74	61	44	2
Solyc01g008100.2.1	0	0	0	0	0	0	0	0	0	0	0	0	0
Solyc01g008110.2.1	8	1328	170	630	20	141	188	273	27	435	681	347	9
Solyc01g008120.2.1	8	52	5	37	1	105	54	97	2	81	11	88	0
Solyc01g008130.2.1	3	7	3	18	5	42	21	63	4	71	5	52	1
Solyc01g008140.2.1	4	16	1	4	0	11	4	41	3	20	16	30	0
Solyc01g008160.2.1	3	19	0	20	0	42	3	60	1	48	8	78	0
Solyc01g008170.2.1	14	65	1	78	1	177	32	277	3	196	16	161	1
Solyc01g008180.2.1	11	85	10	22	4	20	50	48	1	60	17	37	1
Solyc01g008190.2.1	2	9	0	10	0	7	1	26	2	9	4	6	0
Solyc01g008200.2.1	0	0	0	0	0	0	0	0	0	0	0	0	0
Solyc01g008210.2.1	1	1	0	0	0	2	9	4	0	1	1	1	0
Solyc01g008220.2.1	0	0	0	0	0	0	0	0	0	0	0	0	0
Solyc01g008230.2.1	6	64	8	45	4	43	25	30	7	295	23	330	1
Solyc01g008240.2.1	0	0	0	0	0	0	0	0	0	0	0	0	0
Solyc01g008250.2.1	7	255	21	101	4	47	73	62	6	60	7	78	3
Solyc01g008260.2.1	3	5	3	3	0	3	6	18	0	9	6	21	0
Solyc01g008270.2.1	5	42	1	33	0	67	20	73	5	60	12	29	0
Solyc01g008280.2.1	10	76	8	57	4	334	147	392	27	253	43	188	3
Solyc01g008290.2.1	3	49	6	17	0	417	47	229	5	34	28	19	0
Solyc01g008300.1.1	0	0	0	0	0	0	0	0	0	0	0	0	0
Solyc01g008310.2.1	8	56	6	26	1	168	60	130	25	166	17	124	0
Solyc01g008320.2.1	8	47	0	55	2	249	62	157	6	207	25	176	7
Solyc01g008330.2.1	8	105	8	51	6	131	94	210	18	220	102	102	7
Solyc01g008340.2.1	2	8	1	2	0	24	7	24	3	21	4	17	0
Solyc01g008360.2.1	3	36	12	27	3	36	61	66	9	98	48	92	2
Solyc01g008370.2.1	7	126	6	49	0	204	104	274	14	251	21	211	2
Solyc01g008390.1.1	0	0	0	0	0	0	0	0	0	0	0	0	0
Solyc01g008410.1.1	0	0	0	0	0	0	0	0	0	0	0	0	0
Solyc01g008420.2.1	2	0	0	0	0	2	16	0	0	15	0	61	0
Solyc01g008440.2.1	13	58	8	23	6	110	123	97	21	84	25	36	0
Solyc01g008460.2.1	2	7	1	4	4	4	18	17	0	21	5	20	3
Solyc01g008470.2.1	1	1	0	2	0	0	1	0	2	3	3	5	3
Solyc01g008480.2.1	0	0	0	0	0	0	0	0	0	0	0	0	0
Solyc01g008490.2.1	5	22	1	12	0	52	20	55	2	25	12	28	0

Solyc01g008500.2.1	0	0	0	0	0	0	0	0	0	0	0	0	0
Solyc01g008510.2.1	0	0	0	0	0	0	0	0	0	0	0	0	0
Solyc01g008520.2.1	0	0	0	0	0	0	0	0	0	0	0	0	0
Solyc01g008530.2.1	2	14	0	9	4	32	8	21	0	7	1	11	0
Solyc01g008540.2.1	0	0	0	0	0	0	0	0	0	0	0	0	0
Solyc01g008550.2.1	11	536	36	290	17	709	506	1171	66	454	31	155	7
Solyc01g008560.2.1	2	37	5	14	1	36	9	27	2	21	2	12	0
Solyc01g008600.2.1	0	0	0	0	0	0	0	0	0	0	0	0	0
Solyc01g008620.2.1	10	333	37	208	5	356	23	626	22	2	0	0	0
Solyc01g008650.2.1	0	0	0	0	0	0	0	0	0	0	0	0	0
Solyc01g008660.2.1	0	0	0	0	0	0	0	0	0	0	0	0	0
Solyc01g008670.2.1	0	0	0	0	0	0	0	0	0	0	0	0	0
Solyc01g008690.2.1	0	0	0	0	0	0	0	0	0	0	0	0	0
Solyc01g008700.2.1	1	2	1	1	0	6	12	3	0	5	1	1	0
Solyc01g008710.2.1	0	0	0	0	0	0	0	0	0	0	0	0	0
Solyc01g008730.2.1	5	21	0	13	6	30	18	38	2	34	14	23	2
Solyc01g008760.2.1	1	0	0	2	0	7	1	9	0	13	0	3	0
Solyc01g008770.2.1	7	87	11	47	3	79	56	99	5	116	42	80	1
Solyc01g008780.2.1	2	74	9	60	0	55	29	77	2	57	13	56	0
Solyc01g008790.2.1	12	89	17	48	4	4	66	34	10	442	18	277	3
Solyc01g008800.1.1	3	12	1	1	0	7	22	18	1	1	0	1	2
Solyc01g008810.2.1	2	10	0	5	2	15	27	8	1	50	2	24	0
Solyc01g008820.2.1	4	70	2	38	6	277	42	179	19	64	45	46	1
Solyc01g008830.1.1	2	7	4	18	0	13	7	19	0	0	0	1	0
Solyc01g008840.2.1	11	79	15	72	0	255	78	262	12	104	40	137	2
Solyc01g008850.2.1	11	402	22	112	4	1450	270	868	21	201	57	163	0
Solyc01g008890.2.1	0	0	0	0	0	0	0	0	0	0	0	0	0
Solyc01g008900.2.1	0	0	0	0	0	0	0	0	0	0	0	0	0
Solyc01g008910.2.1	2	5	0	4	0	26	4	17	0	13	1	2	0
Solyc01g008920.2.1	9	88	20	59	2	1	26	17	3	15	9	38	0
Solyc01g008930.2.1	2	14	1	2	0	35	12	14	1	9	5	19	0
Solyc01g008940.2.1	0	0	0	0	0	0	0	0	0	0	0	0	0
Solyc01g008950.2.1	3	146	4	127	7	251	99	231	10	328	59	468	3
Solyc01g008960.2.1	20	592	50	547	71	473	485	886	46	1844	367	1649	29
Solyc01g008970.2.1	8	423	26	353	21	663	215	698	43	629	129	548	10
Solyc01g008980.2.1	2	9	0	7	0	15	8	17	1	15	6	5	2
Solyc01g008990.2.1	2	3	0	4	0	8	7	12	3	3	3	4	0
Solyc01g009000.2.1	4	62	4	33	11	50	66	82	8	114	30	78	1
Solyc01g009010.2.1	9	87	2	49	0	116	149	122	8	48	15	67	3
Solyc01g009020.2.1	2	59	1	26	1	62	47	49	8	176	60	92	0
Solyc01g009030.2.1	7	27	2	14	0	36	45	103	7	46	22	29	3

Solyc01g009040.2.1	0	0	0	0	0	0	0	0	0	0	0	0	0
Solyc01g009050.2.1	14	101	11	60	18	359	475	201	16	264	68	177	3
Solyc01g009060.2.1	9	52	3	35	7	122	63	98	25	57	29	93	1
Solyc01g009070.2.1	3	3	0	1	0	9	25	24	2	11	0	3	0
Solyc01g009080.2.1	1	66	27	13	0	6	9	6	0	12	10	27	0
Solyc01g009090.2.1	4	62	8	43	1	137	64	196	15	69	32	51	3
Solyc01g009100.2.1	2	295	1	166	10	1646	15	561	7	941	6	720	2
Solyc01g009110.2.1	8	35	19	12	8	64	89	65	6	25	12	30	0
Solyc01g009120.2.1	2	4	0	1	0	1	10	7	1	5	0	3	0
Solyc01g009150.2.1	0	0	0	0	0	0	0	0	0	0	0	0	0
Solyc01g009160.2.1	4	241	8	125	7	39	12	10	4	20	10	61	2
Solyc01g009170.2.1	14	616	50	619	41	544	293	561	15	628	182	719	19
Solyc01g009180.2.1	15	111	11	73	3	254	165	330	12	200	41	114	0
Solyc01g009200.2.1	0	0	0	0	0	0	0	0	0	0	0	0	0
Solyc01g009220.2.1	0	0	0	0	0	0	0	0	0	0	0	0	0
Solyc01g009230.2.1	11	61	13	63	4	98	228	115	6	77	20	58	0
Solyc01g009240.2.1	0	0	0	0	0	0	0	0	0	0	0	0	0
Solyc01g009250.2.1	0	0	0	0	0	0	0	0	0	0	0	0	0
Solyc01g009260.1.1	0	0	0	0	0	0	0	0	0	0	0	0	0
Solyc01g009270.1.1	0	0	0	0	0	0	0	0	0	0	0	0	0
Solyc01g009280.2.1	10	49	11	33	2	88	52	152	17	92	23	34	5
Solyc01g009290.2.1	7	19	5	24	0	62	28	97	2	28	16	28	4
Solyc01g009300.2.1	8	75	7	65	4	26	56	49	7	131	10	70	2
Solyc01g009310.2.1	11	234	9	182	7	204	55	409	11	910	33	674	4
Solyc01g009340.2.1	0	0	0	0	0	0	0	0	0	0	0	0	0
Solyc01g009370.1.1	0	0	0	0	0	0	0	0	0	0	0	0	0
Solyc01g009380.2.1	2	11	0	6	0	34	12	28	3	12	0	5	0
Solyc01g009390.2.1	3	22	0	8	1	51	12	47	4	20	1	14	0
Solyc01g009420.2.1	13	249	50	165	27	294	360	448	25	845	400	579	19
Solyc01g009430.2.1	7	105	10	140	0	292	179	480	1	786	41	423	5
Solyc01g009450.2.1	2	47	1	46	0	108	8	89	1	92	10	79	4
Solyc01g009470.1.1	0	0	0	0	0	0	0	0	0	0	0	0	0
Solyc01g009480.2.1	0	0	0	0	0	0	0	0	0	0	0	0	0
Solyc01g009500.2.1	4	23	2	24	2	23	20	12	6	42	6	43	1
Solyc01g009520.2.1	4	249	7	93	13	174	106	374	27	720	66	711	9
Solyc01g009570.2.1	0	0	0	0	0	0	0	0	0	0	0	0	0
Solyc01g009610.2.1	1	11	0	5	0	3	4	3	2	24	4	3	0
Solyc01g009620.2.1	2	8	1	2	0	18	10	11	1	24	6	9	0
Solyc01g009630.2.1	0	0	0	0	0	0	0	0	0	0	0	0	0
Solyc01g009660.1.1	0	0	0	0	0	0	0	0	0	0	0	0	0
Solyc01g009670.2.1	0	0	0	0	0	0	0	0	0	0	0	0	0

Solyc01g009680.2.1	0	0	0	0	0	0	0	0	0	0	0	0	0
Solyc01g009690.1.1	0	0	0	0	0	0	0	0	0	0	0	0	0
Solyc01g009700.1.1	0	0	0	0	0	0	0	0	0	0	0	0	0
Solyc01g009720.1.1	0	0	0	0	0	0	0	0	0	0	0	0	0
Solyc01g009750.2.1	3	125	5	86	10	422	173	418	136	373	75	335	14
Solyc01g009760.2.1	1	1	2	7	0	4	1	3	0	0	1	1	0
Solyc01g009770.2.1	2	15	0	14	0	61	9	36	0	9	2	15	0
Solyc01g009780.2.1	4	71	4	31	4	84	34	51	11	46	9	29	3
Solyc01g009820.2.1	17	100	15	92	4	202	140	203	28	101	34	86	0
Solyc01g009840.1.1	0	0	0	0	0	0	0	0	0	0	0	0	0
Solyc01g009850.2.1	2	20	0	0	0	26	8	22	0	20	6	2	0
Solyc01g009860.2.1	14	475	16	266	2	1741	232	1085	27	60	13	113	1
Solyc01g009870.2.1	1	0	0	0	0	0	5	2	1	4	0	29	0
Solyc01g009890.1.1	0	0	0	0	0	0	0	0	0	0	0	0	0
Solyc01g009930.1.1	0	0	0	0	0	0	0	0	0	0	0	0	0
Solyc01g009970.2.1	6	88	4	17	0	96	48	194	2	55	9	78	0
Solyc01g009990.2.1	10	206	22	124	4	231	155	291	8	183	47	191	2
Solyc01g010000.2.1	1	5	0	7	0	2	5	2	2	2	0	0	0
Solyc01g010030.2.1	3	12	1	7	0	7	9	14	0	13	1	3	0
Solyc01g010040.2.1	6	37	1	46	2	90	62	110	3	41	8	26	0
Solyc01g010050.2.1	8	87	9	31	4	79	283	139	21	162	16	80	5
Solyc01g010060.2.1	2	17	6	8	0	0	1	3	1	3	1	6	1
Solyc01g010080.2.1	0	0	0	0	0	0	0	0	0	0	0	0	0
Solyc01g010130.2.1	3	4	0	0	0	6	14	8	0	19	1	5	0
Solyc01g010150.2.1	7	30	5	32	0	34	56	86	10	10	3	17	0
Solyc01g010180.2.1	0	0	0	0	0	0	0	0	0	0	0	0	0
Solyc01g010190.2.1	9	113	3	67	6	44	94	114	9	118	26	126	2
Solyc01g010200.2.1	8	162	0	182	0	141	68	208	5	612	7	615	0
Solyc01g010220.2.1	0	0	0	0	0	0	0	0	0	0	0	0	0
Solyc01g010230.2.1	0	0	0	0	0	0	0	0	0	0	0	0	0
Solyc01g010250.2.1	4	15	5	2	0	179	24	112	6	21	9	17	0
Solyc01g010270.2.1	1	15	0	3	0	0	9	4	1	37	0	21	0
Solyc01g010280.2.1	0	0	0	0	0	0	0	0	0	0	0	0	0
Solyc01g010290.2.1	2	25	0	13	2	18	12	36	0	44	2	30	0
Solyc01g010310.2.1	3	35	7	12	2	14	8	28	5	132	20	116	1
Solyc01g010350.2.1	0	0	0	0	0	0	0	0	0	0	0	0	0
Solyc01g010360.2.1	0	0	0	0	0	0	0	0	0	0	0	0	0
Solyc01g010370.2.1	2	6	0	1	0	12	6	8	0	4	0	4	0
Solyc01g010390.2.1	2	7	0	2	0	4	5	25	2	9	0	10	0
Solyc01g010410.2.1	0	0	0	0	0	0	0	0	0	0	0	0	0
Solyc01g010430.2.1	4	26	8	15	1	26	14	16	6	5	9	18	1

Solyc01g010440.2.1	3	16	2	9	0	42	15	92	4	21	3	19	0
Solyc01g010460.2.1	1	4	0	10	0	1	4	2	0	2	0	3	0
Solyc01g010470.2.1	0	0	0	0	0	0	0	0	0	0	0	0	0
Solyc01g010480.2.1	14	549	124	369	20	280	360	418	14	173	6	395	0
Solyc01g010490.2.1	0	0	0	0	0	0	0	0	0	0	0	0	0
Solyc01g010510.2.1	1	0	0	0	0	2	17	1	0	2	0	3	0
Solyc01g010520.2.1	6	48	6	7	0	156	112	121	0	39	3	15	0
Solyc01g010540.2.1	2	60	5	15	0	83	29	106	13	72	28	65	3
Solyc01g010570.2.1	3	13	2	8	0	4	35	40	5	55	6	42	0
Solyc01g010580.2.1	0	0	0	0	0	0	0	0	0	0	0	0	0
Solyc01g010590.2.1	8	107	4	71	0	241	45	315	13	342	40	180	0
Solyc01g010640.2.1	9	168	29	92	0	29	27	16	5	59	21	23	2
Solyc01g010650.2.1	3	14	0	7	0	9	10	32	0	34	8	43	0
Solyc01g010660.2.1	6	34	6	23	2	63	38	43	5	53	23	25	5
Solyc01g010700.2.1	10	80	6	73	5	554	161	462	30	75	28	51	1
Solyc01g010710.2.1	0	0	0	0	0	0	0	0	0	0	0	0	0
Solyc01g010750.2.1	12	645	115	590	45	3669	1943	2024	317	3331	444	2432	32
Solyc01g010760.2.1	7	114	26	35	6	173	299	131	96	165	99	155	5
Solyc01g010770.2.1	9	50	15	66	2	139	401	419	20	147	26	6	0
Solyc01g010780.2.1	7	76	12	73	11	87	101	95	12	167	46	164	7
Solyc01g010810.2.1	3	65	5	61	7	91	118	91	3	95	22	199	4
Solyc01g010820.2.1	11	55	7	54	0	155	83	236	20	76	37	66	1
Solyc01g010830.2.1	6	24	1	15	0	27	25	23	1	58	11	42	1
Solyc01g010860.2.1	6	17	2	17	1	57	27	52	13	39	10	30	0
Solyc01g010870.2.1	4	18	2	16	0	53	26	101	11	70	7	49	0
Solyc01g010880.2.1	0	0	0	0	0	0	0	0	0	0	0	0	0
Solyc01g010920.1.1	0	0	0	0	0	0	0	0	0	0	0	0	0
Solyc01g010930.2.1	0	0	0	0	0	0	0	0	0	0	0	0	0
Solyc01g010940.2.1	2	14	1	3	0	37	13	37	7	12	12	24	0
Solyc01g010950.2.1	0	0	0	0	0	0	0	0	0	0	0	0	0
Solyc01g010960.1.1	0	0	0	0	0	0	0	0	0	0	0	0	0
Solyc01g010970.2.1	0	0	0	0	0	0	0	0	0	0	0	0	0
Solyc01g010990.2.1	1	13	0	3	0	5	6	8	2	3	1	2	0
Solyc01g011000.2.1	1	81	2	22	0	95	52	115	13	89	10	63	0
Solyc01g011010.2.1	1	3	0	0	0	12	11	4	0	18	0	5	0
Solyc01g011030.2.1	5	16	2	21	4	62	28	39	4	192	13	65	4
Solyc01g011040.2.1	1	5	0	6	0	7	6	16	1	6	3	13	1
Solyc01g011050.2.1	0	0	0	0	0	0	0	0	0	0	0	0	0
Solyc01g011080.2.1	0	0	0	0	0	0	0	0	0	0	0	0	0
Solyc01g011090.2.1	2	4	0	2	0	5	7	4	0	10	1	14	0
Solyc01g011100.2.1	5	28	7	14	4	72	44	90	7	76	8	48	0

Solyc01g011200.2.1	0	0	0	0	0	0	0	0	0	0	0	0	0
Solyc01g011220.1.1	0	0	0	0	0	0	0	0	0	0	0	0	0
Solyc01g011340.2.1	1	0	2	1	13	4	20	0	21	0	4	0	15
Solyc01g011350.2.1	4	20	1	22	0	7	27	23	6	259	18	104	2
Solyc01g011430.2.1	0	0	0	0	0	0	0	0	0	0	0	0	0
Solyc01g011510.2.1	1	13	0	5	2	11	2	28	0	32	0	9	0
Solyc01g012700.2.1	0	0	0	0	0	0	0	0	0	0	0	0	0
Solyc01g013890.1.1	0	0	0	0	0	0	0	0	0	0	0	0	0
Solyc01g014100.2.1	0	0	0	0	0	0	0	0	0	0	0	0	0
Solyc01g014110.2.1	0	0	0	0	0	0	0	0	0	0	0	0	0
Solyc01g014120.2.1	0	0	0	0	0	0	0	0	0	0	0	0	0
Solyc01g014140.2.1	0	0	0	0	0	0	0	0	0	0	0	0	0
Solyc01g014180.2.1	2	118	4	39	1	185	187	278	19	336	66	291	2
Solyc01g014210.1.1	0	0	0	0	0	0	0	0	0	0	0	0	0
Solyc01g014230.2.1	4	19	8	20	2	22	45	32	4	37	7	27	0
Solyc01g014290.1.1	0	0	0	0	0	0	0	0	0	0	0	0	0
Solyc01g014330.2.1	0	0	0	0	0	0	0	0	0	0	0	0	0
Solyc01g014370.1.1	0	0	0	0	0	0	0	0	0	0	0	0	0
Solyc01g014380.2.1	0	0	0	0	0	0	0	0	0	0	0	0	0
Solyc01g014470.1.1	0	0	0	0	0	0	0	0	0	0	0	0	0
Solyc01g014480.2.1	2	8	0	8	0	24	7	17	4	3	7	25	1
Solyc01g014490.2.1	2	6	1	2	2	12	10	24	4	11	0	20	0
Solyc01g014620.1.1	2	1	1	8	0	7	37	0	0	0	7	0	0
Solyc01g014840.2.1	0	0	0	0	0	0	0	0	0	0	0	0	0
Solyc01g014850.2.1	10	114	10	74	6	190	44	274	11	233	39	169	6
Solyc01g014860.2.1	0	0	0	0	0	0	0	0	0	0	0	0	0
Solyc01g014950.1.1	0	0	0	0	0	0	0	0	0	0	0	0	0
Solyc01g015020.2.1	17	258	15	180	3	650	272	752	60	309	78	155	6
Solyc01g015070.2.1	0	0	0	0	0	0	0	0	0	0	0	0	0
Solyc01g015140.1.1	0	0	0	0	0	0	0	0	0	0	0	0	0
Solyc01g016360.1.1	0	0	0	0	0	0	0	0	0	0	0	0	0
Solyc01g016370.1.1	0	0	0	0	0	0	0	0	0	0	0	0	0
Solyc01g016410.2.1	1	3	0	0	0	13	1	18	0	23	5	15	0
Solyc01g016470.2.1	0	0	0	0	0	0	0	0	0	0	0	0	0
Solyc01g017050.1.1	0	0	0	0	0	0	0	0	0	0	0	0	0
Solyc01g017430.1.1	0	0	0	0	0	0	0	0	0	0	0	0	0
Solyc01g017490.1.1	0	0	0	0	0	0	0	0	0	0	0	0	0
Solyc01g017600.2.1	3	73	9	43	0	8	3	30	0	0	0	0	0
Solyc01g017710.1.1	0	0	0	0	0	0	0	0	0	0	0	0	0
Solyc01g017860.2.1	0	0	0	0	0	0	0	0	0	0	0	0	0
Solyc01g018020.1.1	0	0	0	0	0	0	0	0	0	0	0	0	0

Solyc01g020130.2.1	0	0	0	0	0	0	0	0	0	0	0	0	0
Solyc01g020190.2.1	0	0	0	0	0	0	0	0	0	0	0	0	0
Solyc01g020220.1.1	0	0	0	0	0	0	0	0	0	0	0	0	0
Solyc01g020240.2.1	2	28	4	24	0	21	19	26	3	66	3	44	0
Solyc01g020320.2.1	1	4	0	1	0	7	22	11	3	5	1	6	0
Solyc01g020340.1.1	0	0	0	0	0	0	0	0	0	0	0	0	0
Solyc01g020440.2.1	0	0	0	0	0	0	0	0	0	0	0	0	0
Solyc01g020460.1.1	0	0	0	0	0	0	0	0	0	0	0	0	0
Solyc01g020520.2.1	0	0	0	0	0	0	0	0	0	0	0	0	0
Solyc01g021600.2.1	0	0	0	0	0	0	0	0	0	0	0	0	0
Solyc01g021640.2.1	3	20	0	16	0	27	22	47	4	21	2	15	0
Solyc01g021670.2.1	1	4	0	4	0	9	8	18	3	11	6	22	6
Solyc01g021740.1.1	0	0	0	0	0	0	0	0	0	0	0	0	0
Solyc01g022740.2.1	2	7	0	4	1	6	9	4	3	1	1	6	0
Solyc01g022750.2.1	2	67	11	44	4	55	72	150	27	63	52	47	2
Solyc01g022780.1.1	1	19	0	56	311	4	80	8	38	9	6	5	19
Solyc01g028800.1.1	0	0	0	0	0	0	0	0	0	0	0	0	0
Solyc01g028810.2.1	4	130	28	81	2	221	186	306	24	314	97	277	5
Solyc01g028830.2.1	8	82	6	51	0	78	12	90	0	93	27	88	3
Solyc01g028860.2.1	20	614	72	519	53	812	566	1522	236	2009	285	1388	12
Solyc01g028870.2.1	5	24	1	18	0	35	27	70	0	66	12	116	0
Solyc01g028900.2.1	6	183	14	120	27	1052	588	644	58	419	65	392	7
Solyc01g028930.2.1	4	51	2	18	0	72	41	98	8	44	9	31	0
Solyc01g034020.2.1	5	64	6	14	4	30	17	59	3	66	25	57	2
Solyc01g034080.1.1	0	0	0	0	0	0	0	0	0	0	0	0	0
Solyc01g034150.2.1	0	0	0	0	0	0	0	0	0	0	0	0	0
Solyc01g034180.1.1	0	0	0	0	0	0	0	0	0	0	0	0	0
Solyc01g034220.1.1	0	0	0	0	0	0	0	0	0	0	0	0	0
Solyc01g044270.2.1	2	4	0	10	0	13	8	13	1	9	12	5	0
Solyc01g044340.2.1	1	4	2	2	0	6	9	26	3	7	4	2	0
Solyc01g044350.2.1	5	23	4	14	6	48	49	108	19	47	8	38	1
Solyc01g044360.2.1	11	86	9	76	4	180	85	246	30	434	65	281	2
Solyc01g044370.1.1	0	0	0	0	0	0	0	0	0	0	0	0	0
Solyc01g044420.2.1	0	0	0	0	0	0	0	0	0	0	0	0	0
Solyc01g044480.2.1	1	5	0	4	0	7	15	24	1	7	2	4	0
Solyc01g044490.2.1	0	0	0	0	0	0	0	0	0	0	0	0	0
Solyc01g047590.1.1	0	0	0	0	0	0	0	0	0	0	0	0	0
Solyc01g048590.1.1	0	0	0	0	0	0	0	0	0	0	0	0	0
Solyc01g049650.2.1	9	162	22	127	3	272	143	281	11	1075	66	720	2
Solyc01g049680.2.1	9	120	7	44	1	141	58	200	16	148	51	79	5
Solyc01g049690.2.1	3	48	4	16	2	36	36	76	12	87	22	93	0

Solyc01g049770.2.1	0	0	0	0	0	0	0	0	0	0	0	0	0
Solyc01g049810.2.1	0	0	0	0	0	0	0	0	0	0	0	0	0
Solyc01g049890.2.1	5	136	4	126	4	459	112	403	13	411	75	452	3
Solyc01g049900.2.1	0	0	0	0	0	0	0	0	0	0	0	0	0
Solyc01g049910.2.1	0	0	0	0	0	0	0	0	0	0	0	0	0
Solyc01g049960.2.1	0	0	0	0	0	0	0	0	0	0	0	0	0
Solyc01g050000.2.1	0	0	0	0	0	0	0	0	0	0	0	0	0
Solyc01g050010.2.1	0	0	0	0	0	0	0	0	0	0	0	0	0
Solyc01g050040.2.1	5	18	6	16	4	11	38	25	3	53	46	55	2
Solyc01g050060.1.1	0	0	0	0	0	0	0	0	0	0	0	0	0
Solyc01g050070.2.1	1	5	0	2	0	8	4	1	1	3	0	7	0
Solyc01g056310.2.1	0	0	0	0	0	0	0	0	0	0	0	0	0
Solyc01g056340.2.1	6	29	0	6	2	42	26	41	2	29	5	22	0
Solyc01g056370.2.1	0	0	0	0	0	0	0	0	0	0	0	0	0
Solyc01g056520.2.1	7	19	4	16	3	95	52	69	10	21	6	7	0
Solyc01g056570.2.1	23	73	19	82	25	128	219	241	26	167	69	147	18
Solyc01g056580.2.1	0	0	0	0	0	0	0	0	0	0	0	0	0
Solyc01g056600.2.1	0	0	0	0	0	0	0	0	0	0	0	0	0
Solyc01g056610.1.1	0	0	0	0	0	0	0	0	0	0	0	0	0
Solyc01g056620.2.1	1	3	0	0	0	0	4	6	0	9	1	4	0
Solyc01g056670.1.1	0	0	0	0	0	0	0	0	0	0	0	0	0
Solyc01g056690.2.1	6	73	6	59	10	14	41	45	7	66	17	38	0
Solyc01g056720.2.1	2	27	0	24	0	116	14	96	2	38	9	18	0
Solyc01g056780.2.1	5	225	18	101	0	51	259	40	1	71	43	83	3
Solyc01g056790.2.1	0	0	0	0	0	0	0	0	0	0	0	0	0
Solyc01g056800.1.1	0	0	0	0	0	0	0	0	0	0	0	0	0
Solyc01g056810.2.1	0	0	0	0	0	0	0	0	0	0	0	0	0
Solyc01g056830.2.1	0	0	0	0	0	0	0	0	0	0	0	0	0
Solyc01g056850.2.1	2	7	1	8	0	8	3	2	1	14	4	7	35
Solyc01g056940.2.1	2	187	15	78	8	184	91	307	63	489	80	302	11
Solyc01g056980.2.1	0	0	0	0	0	0	0	0	0	0	0	0	0
Solyc01g056990.2.1	0	0	0	0	0	0	0	0	0	0	0	0	0
Solyc01g057000.2.1	2	4	0	2	0	3008	80	927	2	2708	113	1387	2
Solyc01g057020.2.1	2	5	0	7	0	24	10	15	1	8	2	6	1
Solyc01g057040.2.1	0	0	0	0	0	0	0	0	0	0	0	0	0
Solyc01g057090.2.1	5	13	6	19	2	32	24	25	4	34	3	37	0
Solyc01g057200.2.1	3	27	0	15	0	7	13	10	1	2	4	4	1
Solyc01g057210.2.1	2	53	5	18	0	69	20	48	10	148	10	53	0
Solyc01g057260.2.1	0	0	0	0	0	0	0	0	0	0	0	0	0
Solyc01g057270.2.1	1	2	0	5	0	2	5	1	0	0	3	0	0
Solyc01g057310.2.1	1	1	0	2	2	8	3	1	0	13	2	4	0

Solyc01g057320.1.1	0	0	0	0	0	0	0	0	0	0	0	0	0
Solyc01g057340.2.1	0	0	0	0	0	0	0	0	0	0	0	0	0
Solyc01g057460.2.1	0	0	0	0	0	0	0	0	0	0	0	0	0
Solyc01g057520.2.1	9	22	2	20	1	9	28	31	4	94	23	47	1
Solyc01g057570.2.1	1	8	2	3	0	9	5	12	3	16	3	26	0
Solyc01g057580.1.1	0	0	0	0	0	0	0	0	0	0	0	0	0
Solyc01g057680.2.1	0	0	0	0	0	0	0	0	0	0	0	0	0
Solyc01g057760.2.1	18	260	13	137	7	706	165	639	36	325	42	240	5
Solyc01g057770.2.1	8	288	35	148	16	20	4	18	1	32	2	138	2
Solyc01g057780.2.1	12	57	12	69	2	103	65	113	4	106	18	109	0
Solyc01g057830.2.1	8	433	32	157	0	145	272	197	3	158	29	164	1
Solyc01g057840.2.1	2	2	0	0	0	4	10	21	9	6	6	6	0
Solyc01g057850.2.1	0	0	0	0	0	0	0	0	0	0	0	0	0
Solyc01g057900.2.1	11	46	2	31	4	179	91	196	11	107	15	90	14
Solyc01g057910.2.1	0	0	0	0	0	0	0	0	0	0	0	0	0
Solyc01g058000.2.1	0	0	0	0	0	0	0	0	0	0	0	0	0
Solyc01g058010.2.1	1	3	2	1	1	4	2	2	0	1	1	1	0
Solyc01g058020.2.1	0	0	0	0	0	0	0	0	0	0	0	0	0
Solyc01g058030.1.1	0	0	0	0	0	0	0	0	0	0	0	0	0
Solyc01g058060.1.1	0	0	0	0	0	0	0	0	0	0	0	0	0
Solyc01g058140.2.1	11	111	7	109	0	328	193	348	11	147	36	115	0
Solyc01g058160.2.1	2	1	0	5	0	0	0	1	0	17	4	8	0
Solyc01g058170.2.1	0	0	0	0	0	0	0	0	0	0	0	0	0
Solyc01g058190.2.1	11	141	6	93	5	328	185	322	6	115	28	92	0
Solyc01g058250.1.1	0	0	0	0	0	0	0	0	0	0	0	0	0
Solyc01g058260.2.1	9	199	15	84	9	401	81	240	27	227	43	192	6
Solyc01g058270.2.1	2	3	0	0	4	2	13	6	4	21	3	19	3
Solyc01g058280.2.1	0	0	0	0	0	0	0	0	0	0	0	0	0
Solyc01g058320.2.1	5	78	6	21	6	29	13	25	2	174	12	96	1
Solyc01g058390.2.1	4	31	2	14	10	50	34	63	9	81	17	71	4
Solyc01g058450.2.1	12	50	4	26	10	118	86	152	10	169	27	120	2
Solyc01g058470.1.1	0	0	0	0	0	0	0	0	0	0	0	0	0
Solyc01g058490.1.1	3	12	24	26	73	19	249	16	40	20	3859	26	20
Solyc01g058500.2.1	0	0	0	0	0	0	0	0	0	0	0	0	0
Solyc01g058520.2.1	0	0	0	0	0	0	0	0	0	0	0	0	0
Solyc01g058530.2.1	0	0	0	0	0	0	0	0	0	0	0	0	0
Solyc01g058540.2.1	0	0	0	0	0	0	0	0	0	0	0	0	0
Solyc01g058670.2.1	6	106	8	81	2	68	38	81	1	407	42	409	4
Solyc01g058720.2.1	1	161	2	56	1	49	0	9	2	0	1	0	0
Solyc01g058730.2.1	4	25	2	29	4	125	29	126	1	124	18	163	1
Solyc01g059830.2.1	5	71	1	37	0	98	129	163	14	108	5	80	0

Solyc01g059860.2.1	8	71	5	38	2	38	30	71	9	53	9	57	0
Solyc01g059870.2.1	10	135	15	99	1	84	49	124	6	77	12	76	0
Solyc01g059880.2.1	11	135	6	78	31	223	64	184	54	279	59	179	2
Solyc01g059900.2.1	0	0	0	0	0	0	0	0	0	0	0	0	0
Solyc01g059920.2.1	0	0	0	0	0	0	0	0	0	0	0	0	0
Solyc01g059930.2.1	6	255	11	147	7	664	74	509	17	260	13	157	3
Solyc01g059940.2.1	0	0	0	0	0	0	0	0	0	0	0	0	0
Solyc01g060030.2.1	5	60	0	32	0	99	40	83	8	181	36	158	3
Solyc01g060040.2.1	6	36	2	25	2	63	38	55	6	50	8	46	0
Solyc01g060050.1.1	0	0	0	0	0	0	0	0	0	0	0	0	0
Solyc01g060070.2.1	1	7	0	0	0	10	0	2	0	6	2	0	2
Solyc01g060080.2.1	6	16	11	34	0	39	31	61	1	41	7	21	3
Solyc01g060090.1.1	0	0	0	0	0	0	0	0	0	0	0	0	0
Solyc01g060130.2.1	2	44	1	10	0	53	25	70	9	23	19	41	2
Solyc01g060140.2.1	8	187	45	154	16	313	239	350	53	185	58	231	6
Solyc01g060150.2.1	3	15	3	19	1	50	42	100	16	34	10	17	0
Solyc01g060160.1.1	0	0	0	0	0	0	0	0	0	0	0	0	0
Solyc01g060170.2.1	1	3	4	7	1	33	15	22	12	30	2	58	0
Solyc01g060180.2.1	10	752	79	454	12	128	111	176	19	198	7	155	0
Solyc01g060240.2.1	0	0	0	0	0	0	0	0	0	0	0	0	0
Solyc01g060260.2.1	0	0	0	0	0	0	0	0	0	0	0	0	0
Solyc01g060270.1.1	0	0	0	0	0	0	0	0	0	0	0	0	0
Solyc01g060280.2.1	3	12	0	9	0	25	20	66	12	10	7	8	0
Solyc01g060320.1.1	0	0	0	0	0	0	0	0	0	0	0	0	0
Solyc01g060410.2.1	0	0	0	0	0	0	0	0	0	0	0	0	0
Solyc01g060450.2.1	0	0	0	0	0	0	0	0	0	0	0	0	0
Solyc01g060470.2.1	6	202	31	142	20	547	270	591	45	951	147	614	17
Solyc01g060490.2.1	0	0	0	0	0	0	0	0	0	0	0	0	0
Solyc01g065490.2.1	12	21	18	20	10	92	520	114	47	74	147	59	12
Solyc01g065500.2.1	0	0	0	0	0	0	0	0	0	0	0	0	0
Solyc01g065510.2.1	2	20	3	15	0	0	4	1	0	18	7	2	0
Solyc01g065530.2.1	12	593	130	286	53	109	20	39	7	135	76	280	17
Solyc01g065540.2.1	8	29	1	19	0	55	30	110	2	123	17	68	0
Solyc01g065550.2.1	4	47	5	14	2	67	18	74	2	51	9	42	0
Solyc01g065560.2.1	5	13	1	9	2	8	12	23	7	23	14	21	2
Solyc01g065580.2.1	9	27	2	26	0	106	67	131	15	76	31	67	0
Solyc01g065590.1.1	0	0	0	0	0	0	0	0	0	0	0	0	0
Solyc01g065600.1.1	0	0	0	0	0	0	0	0	0	0	0	0	0
Solyc01g065690.2.1	6	24	1	18	0	82	48	147	0	17	9	28	0
Solyc01g065700.2.1	0	0	0	0	0	0	0	0	0	0	0	0	0
Solyc01g065710.2.1	0	0	0	0	0	0	0	0	0	0	0	0	0

Solyc01g065720.2.1	8	42	5	47	1	194	91	186	9	117	19	60	1
Solyc01g065740.2.1	0	0	0	0	0	0	0	0	0	0	0	0	0
Solyc01g065780.1.1	0	0	0	0	0	0	0	0	0	0	0	0	0
Solyc01g065840.2.1	4	2	5	13	0	24	23	19	7	48	9	21	1
Solyc01g065980.2.1	10	333	1	313	4	2622	289	3255	22	4447	914	3155	35
Solyc01g066050.2.1	16	294	16	152	2	289	84	498	15	690	46	436	0
Solyc01g066060.2.1	2	17	0	2	0	11	9	23	1	18	5	16	1
Solyc01g066260.2.1	0	0	0	0	0	0	0	0	0	0	0	0	0
Solyc01g066290.2.1	0	0	0	0	0	0	0	0	0	0	0	0	0
Solyc01g066310.2.1	13	80	22	47	10	148	166	201	26	172	41	158	4
Solyc01g066340.2.1	0	0	0	0	0	0	0	0	0	0	0	0	0
Solyc01g066360.2.1	0	0	0	0	0	0	0	0	0	0	0	0	0
Solyc01g066420.2.1	0	0	0	0	0	0	0	0	0	0	0	0	0
Solyc01g066430.2.1	15	632	163	530	95	216	19	183	13	168	24	411	28
Solyc01g066440.2.1	0	0	0	0	0	0	0	0	0	0	0	0	0
Solyc01g066450.2.1	1	0	1	0	2	23	72	1	3	2	2	0	0
Solyc01g066460.2.1	0	0	0	0	0	0	0	0	0	0	0	0	0
Solyc01g066470.2.1	0	0	0	0	0	0	0	0	0	0	0	0	0
Solyc01g066480.2.1	2	7	0	1	2	29	11	20	0	22	3	24	0
Solyc01g066510.1.1	0	0	0	0	0	0	0	0	0	0	0	0	0
Solyc01g066520.2.1	9	40	19	30	104	181	119	143	77	121	99	96	4
Solyc01g066560.2.1	0	0	0	0	0	0	0	0	0	0	0	0	0
Solyc01g066570.2.1	2	5	0	0	0	9	3	18	0	42	3	54	0
Solyc01g066590.2.1	3	37	4	11	5	36	16	39	4	65	6	37	0
Solyc01g066610.2.1	0	0	0	0	0	0	0	0	0	0	0	0	0
Solyc01g066620.2.1	0	0	0	0	0	0	0	0	0	0	0	0	0
Solyc01g066640.2.1	0	0	0	0	0	0	0	0	0	0	0	0	0
Solyc01g066680.2.1	5	63	14	9	0	5	14	16	0	0	0	0	0
Solyc01g066690.2.1	4	16	1	6	5	55	53	55	12	55	20	57	0
Solyc01g066700.2.1	0	0	0	0	0	0	0	0	0	0	0	0	0
Solyc01g066710.2.1	0	0	0	0	0	0	0	0	0	0	0	0	0
Solyc01g066720.2.1	4	74	11	31	3	210	38	109	9	194	33	222	8
Solyc01g066730.2.1	0	0	0	0	0	0	0	0	0	0	0	0	0
Solyc01g066740.2.1	14	42	9	42	0	46	58	43	0	423	53	289	5
Solyc01g066760.2.1	0	0	0	0	0	0	0	0	0	0	0	0	0
Solyc01g066770.2.1	1	8	3	4	0	22	2	15	2	35	0	22	0
Solyc01g066810.2.1	0	0	0	0	0	0	0	0	0	0	0	0	0
Solyc01g066820.2.1	0	0	0	0	0	0	0	0	0	0	0	0	0
Solyc01g066830.2.1	2	8	5	13	0	9	22	25	4	22	3	7	2
Solyc01g066840.2.1	2	226	9	56	10	153	273	263	57	280	172	220	11
Solyc01g066850.2.1	0	0	0	0	0	0	0	0	0	0	0	0	0

Solyc01g066860.2.1	1	10	0	3	0	2	4	2	0	1	0	0	0
Solyc01g066870.2.1	3	30	2	17	0	5	15	11	1	17	7	9	0
Solyc01g066880.2.1	2	5	4	1	0	7	9	12	0	5	0	2	0
Solyc01g066900.2.1	0	0	0	0	0	0	0	0	0	0	0	0	0
Solyc01g066910.2.1	0	0	0	0	0	0	0	0	0	0	0	0	0
Solyc01g066940.2.1	0	0	0	0	0	0	0	0	0	0	0	0	0
Solyc01g066970.2.1	0	0	0	0	0	0	0	0	0	0	0	0	0
Solyc01g066980.2.1	0	0	0	0	0	0	0	0	0	0	0	0	0
Solyc01g067000.2.1	8	109	6	73	6	80	26	84	5	105	9	109	3
Solyc01g067010.2.1	13	94	2	93	0	238	53	131	6	34	18	94	0
Solyc01g067020.2.1	3	9	1	18	0	51	62	60	3	1	7	1	4
Solyc01g067030.2.1	0	0	0	0	0	0	0	0	0	0	0	0	0
Solyc01g067040.1.1	0	0	0	0	0	0	0	0	0	0	0	0	0
Solyc01g067070.2.1	2	5	0	3	0	3	2	4	1	14	4	9	0
Solyc01g067080.2.1	0	0	0	0	0	0	0	0	0	0	0	0	0
Solyc01g067090.2.1	1	1	0	0	0	0	3	3	0	5	1	2	0
Solyc01g067100.2.1	6	56	9	35	4	168	151	262	24	138	27	110	0
Solyc01g067120.2.1	0	0	0	0	0	0	0	0	0	0	0	0	0
Solyc01g067130.2.1	0	0	0	0	0	0	0	0	0	0	0	0	0
Solyc01g067140.1.1	0	0	0	0	0	0	0	0	0	0	0	0	0
Solyc01g067180.2.1	0	0	0	0	0	0	0	0	0	0	0	0	0
Solyc01g067200.1.1	0	0	0	0	0	0	0	0	0	0	0	0	0
Solyc01g067210.2.1	0	0	0	0	0	0	0	0	0	0	0	0	0
Solyc01g067220.1.1	0	0	0	0	0	0	0	0	0	0	0	0	0
Solyc01g067230.2.1	0	0	0	0	0	0	0	0	0	0	0	0	0
Solyc01g067240.2.1	0	0	0	0	0	0	0	0	0	0	0	0	0
Solyc01g067260.2.1	0	0	0	0	0	0	0	0	0	0	0	0	0
Solyc01g067270.2.1	0	0	0	0	0	0	0	0	0	0	0	0	0
Solyc01g067280.2.1	7	26	2	12	0	66	31	73	6	66	2	41	1
Solyc01g067290.2.1	6	114	14	51	0	22	21	31	0	29	14	35	1
Solyc01g067300.2.1	2	1	0	0	0	17	7	4	1	0	0	0	0
Solyc01g067330.2.1	1	9	0	0	1	1	6	0	6	0	1	0	1
Solyc01g067350.2.1	0	0	0	0	0	0	0	0	0	0	0	0	0
Solyc01g067360.2.1	0	0	0	0	0	0	0	0	0	0	0	0	0
Solyc01g067380.2.1	4	8	2	2	0	18	21	28	1	74	12	80	2
Solyc01g067390.2.1	25	295	49	304	31	696	421	1021	73	697	158	478	4
Solyc01g067400.2.1	8	89	11	61	4	120	74	157	22	259	30	186	1
Solyc01g067440.2.1	0	0	0	0	0	0	0	0	0	0	0	0	0
Solyc01g067460.1.1	0	0	0	0	0	0	0	0	0	0	0	0	0
Solyc01g067480.2.1	5	9	5	4	1	5	23	15	0	20	5	32	0
Solyc01g067490.2.1	2	2	4	2	0	11	15	8	1	2	5	4	1

Solyc01g067500.2.1	8	57	9	34	1	126	91	132	22	155	33	91	3
Solyc01g067510.2.1	0	0	0	0	0	0	0	0	0	0	0	0	0
Solyc01g067530.2.1	5	35	3	18	0	30	35	42	5	16	13	19	1
Solyc01g067550.2.1	8	68	6	48	4	71	27	146	24	190	28	120	9
Solyc01g067560.2.1	4	13	2	14	0	64	27	42	8	45	8	24	5
Solyc01g067570.2.1	2	3	1	1	0	8	25	21	3	6	7	17	0
Solyc01g067580.2.1	1	2	0	0	0	4	2	0	0	1	2	4	0
Solyc01g067590.2.1	6	29	4	10	4	40	53	42	22	35	11	32	1
Solyc01g067600.2.1	10	43	9	39	17	141	82	188	18	70	29	99	3
Solyc01g067610.2.1	1	1	0	0	0	4	1	1	0	4	2	3	0
Solyc01g067620.2.1	1	1	0	0	0	23	1	6	0	0	3	0	0
Solyc01g067630.2.1	3	8	0	0	0	3	12	4	1	10	1	19	0
Solyc01g067640.2.1	5	12	2	0	0	28	20	39	6	34	16	12	0
Solyc01g067650.1.1	2	0	0	1	0	19	17	15	0	0	1	0	0
Solyc01g067660.2.1	30	913	41	591	29	4331	1522	4119	102	2315	377	1654	26
Solyc01g067670.2.1	3	24	1	8	1	35	26	46	0	39	6	20	0
Solyc01g067680.2.1	8	99	6	88	10	117	90	190	10	161	30	144	1
Solyc01g067690.2.1	0	0	0	0	0	0	0	0	0	0	0	0	0
Solyc01g067700.2.1	1	1	1	0	0	2	4	1	0	12	0	4	0
Solyc01g067710.2.1	15	64	6	63	5	333	180	244	27	31	25	18	5
Solyc01g067720.2.1	2	18	0	2	0	38	12	18	2	19	3	34	1
Solyc01g067730.2.1	1	139	6	76	20	152	80	219	46	499	259	360	11
Solyc01g067740.2.1	4	151	33	62	19	1029	489	487	68	386	161	312	3
Solyc01g067750.2.1	5	30	4	10	0	60	20	78	9	36	9	44	0
Solyc01g067780.1.1	0	0	0	0	0	0	0	0	0	0	0	0	0
Solyc01g067790.2.1	0	0	0	0	0	0	0	0	0	0	0	0	0
Solyc01g067800.2.1	8	32	2	8	0	72	56	86	6	113	17	138	0
Solyc01g067820.2.1	1	5	0	5	0	9	11	19	1	11	0	10	0
Solyc01g067830.2.1	0	0	0	0	0	0	0	0	0	0	0	0	0
Solyc01g067890.2.1	17	760	70	404	5	174	168	287	4	659	118	439	5
Solyc01g067900.2.1	2	452	9	224	12	141	56	109	9	331	39	282	1
Solyc01g067930.2.1	7	167	17	116	14	107	27	46	12	190	43	120	9
Solyc01g067940.2.1	0	0	0	0	0	0	0	0	0	0	0	0	0
Solyc01g067980.1.1	0	0	0	0	0	0	0	0	0	0	0	0	0
Solyc01g068000.2.1	6	64	14	16	1	70	32	26	6	74	13	61	2
Solyc01g068020.2.1	1	4	1	8	0	5	9	1	0	0	0	0	0
Solyc01g068030.2.1	8	153	13	75	2	87	125	122	4	100	5	91	0
Solyc01g068050.2.1	0	0	0	0	0	0	0	0	0	0	0	0	0
Solyc01g068060.2.1	6	107	7	57	0	125	65	112	13	177	8	107	2
Solyc01g068070.2.1	9	12	3	8	7	37	71	64	3	95	19	75	1
Solyc01g068080.2.1	0	0	0	0	0	0	0	0	0	0	0	0	0

Solyc01g068100.2.1	22	699	67	762	70	1321	487	1542	118	1277	213	1109	21
Solyc01g068120.2.1	0	0	0	0	0	0	0	0	0	0	0	0	0
Solyc01g068130.2.1	4	48	3	18	0	41	16	37	4	82	4	88	0
Solyc01g068140.2.1	0	0	0	0	0	0	0	0	0	0	0	0	0
Solyc01g068150.2.1	1	0	0	0	0	1	2	4	0	3	4	4	0
Solyc01g068160.2.1	4	11	0	11	0	9	7	25	0	7	0	14	0
Solyc01g068170.2.1	6	23	5	41	9	54	53	122	17	34	13	37	0
Solyc01g068180.2.1	3	4	3	0	0	4	9	9	0	13	3	4	0
Solyc01g068190.2.1	0	0	0	0	0	0	0	0	0	0	0	0	0
Solyc01g068200.2.1	5	102	5	77	0	135	40	193	10	136	5	153	1
Solyc01g068210.2.1	3	52	17	31	3	112	57	96	10	97	26	40	1
Solyc01g068240.2.1	10	106	9	47	2	360	159	359	32	186	77	215	11
Solyc01g068250.2.1	0	0	0	0	0	0	0	0	0	0	0	0	0
Solyc01g068260.2.1	1	2	0	0	0	21	0	12	0	6	6	7	0
Solyc01g068270.2.1	5	28	3	0	0	34	17	60	2	139	17	100	0
Solyc01g068280.2.1	0	0	0	0	0	0	0	0	0	0	0	0	0
Solyc01g068290.2.1	5	23	2	13	0	50	56	42	0	27	7	15	0
Solyc01g068330.2.1	19	53	5	33	3	157	79	201	19	48	18	64	14
Solyc01g068340.2.1	0	0	0	0	0	0	0	0	0	0	0	0	0
Solyc01g068350.2.1	0	0	0	0	0	0	0	0	0	0	0	0	0
Solyc01g068360.2.1	0	0	0	0	0	0	0	0	0	0	0	0	0
Solyc01g068370.2.1	1	4	0	2	0	6	2	11	1	1	2	1	0
Solyc01g068380.2.1	14	15	2	7	0	88	264	122	35	0	6	2	0
Solyc01g068390.2.1	19	119	31	93	16	271	270	332	59	187	85	195	3
Solyc01g068400.2.1	0	0	0	0	0	0	0	0	0	0	0	0	0
Solyc01g068410.2.1	1	1	0	0	0	2	0	4	3	6	24	0	0
Solyc01g068430.1.1	0	0	0	0	0	0	0	0	0	0	0	0	0
Solyc01g068440.1.1	0	0	0	0	0	0	0	0	0	0	0	0	0
Solyc01g068450.2.1	0	0	0	0	0	0	0	0	0	0	0	0	0
Solyc01g068460.2.1	20	242	30	312	17	275	274	844	48	1161	110	1220	3
Solyc01g068470.2.1	1	4	0	3	0	0	0	0	0	4	0	7	0
Solyc01g068480.2.1	29	113	15	89	24	266	161	372	17	475	52	438	6
Solyc01g068490.2.1	0	0	0	0	0	0	0	0	0	0	0	0	0
Solyc01g068500.2.1	2	4	0	7	0	19	10	18	0	0	6	0	0
Solyc01g068520.1.1	0	0	0	0	0	0	0	0	0	0	0	0	0
Solyc01g068530.2.1	3	828	32	422	27	2887	413	1865	91	1957	216	1414	18
Solyc01g068540.2.1	0	0	0	0	0	0	0	0	0	0	0	0	0
Solyc01g068550.2.1	0	0	0	0	0	0	0	0	0	0	0	0	0
Solyc01g068560.2.1	1	10	1	1	0	1	0	0	0	9	3	1	0
Solyc01g068580.2.1	5	27	2	8	2	32	10	63	6	42	11	17	0
Solyc01g068590.2.1	1	5	0	10	2	19	11	33	0	4	0	2	0

Solyc01g068600.2.1	1	2	1	4	0	5	10	14	1	2	1	10	0
Solyc01g068610.2.1	14	148	13	105	1	159	152	289	17	163	34	71	2
Solyc01g068620.2.1	6	87	3	38	2	46	39	53	5	54	7	46	1
Solyc01g068630.2.1	1	0	0	0	0	3	1	5	0	4	3	0	0
Solyc01g068640.2.1	2	15	0	4	0	11	15	6	3	27	5	29	0
Solyc01g073640.2.1	10	1638	11	576	3	14	0	63	0	470	150	44	0
Solyc01g073650.2.1	5	129	1	50	1	130	29	134	5	182	8	82	0
Solyc01g073660.2.1	2	5	1	3	0	12	11	7	2	4	0	1	0
Solyc01g073670.2.1	0	0	0	0	0	0	0	0	0	0	0	0	0
Solyc01g073680.2.1	1	11	1	4	0	10	1	14	0	5	14	0	0
Solyc01g073690.2.1	8	151	8	103	2	776	93	475	13	436	62	282	7
Solyc01g073700.2.1	1	8	0	0	0	0	6	0	0	8	0	5	0
Solyc01g073710.2.1	2	11	0	10	0	13	16	8	0	15	7	9	0
Solyc01g073720.2.1	0	0	0	0	0	0	0	0	0	0	0	0	0
Solyc01g073730.2.1	5	12	6	5	0	7	8	15	2	19	8	18	1
Solyc01g073740.2.1	9	128	9	89	15	353	117	239	20	329	16	347	8
Solyc01g073750.2.1	26	212	58	262	15	200	236	474	37	529	94	356	12
Solyc01g073760.2.1	2	34	4	39	5	24	15	68	1	34	5	98	1
Solyc01g073770.2.1	8	49	11	14	3	75	105	118	9	113	30	26	4
Solyc01g073820.2.1	0	0	0	0	0	0	0	0	0	0	0	0	0
Solyc01g073840.1.1	0	0	0	0	0	0	0	0	0	0	0	0	0
Solyc01g073870.2.1	8	31	4	17	1	72	26	86	6	67	21	59	1
Solyc01g073890.2.1	0	0	0	0	0	0	0	0	0	0	0	0	0
Solyc01g073900.2.1	2	18	0	11	2	24	29	28	2	63	20	50	0
Solyc01g073910.2.1	2	8	1	10	0	3	8	13	2	0	0	0	0
Solyc01g073920.2.1	2	11	0	0	1	6	9	20	0	16	11	10	0
Solyc01g073930.2.1	1	1	0	0	0	0	3	6	0	11	0	0	0
Solyc01g073940.2.1	3	9	0	8	0	34	9	25	0	34	9	46	0
Solyc01g073950.2.1	13	78	6	45	4	110	51	167	21	69	31	32	1
Solyc01g073960.2.1	10	114	6	104	6	156	68	173	19	149	50	124	2
Solyc01g073970.2.1	0	0	0	0	0	0	0	0	0	0	0	0	0
Solyc01g074000.2.1	3	25	1	12	0	0	0	7	0	30	11	40	0
Solyc01g074010.2.1	12	62	18	62	9	66	78	110	35	149	47	90	4
Solyc01g074020.2.1	0	0	0	0	0	0	0	0	0	0	0	0	0
Solyc01g074030.2.1	10	444	13	168	10	81	562	242	13	52	7	221	5
Solyc01g079060.2.1	0	0	0	0	0	0	0	0	0	0	0	0	0
Solyc01g079070.2.1	1	0	0	0	0	0	1	3	0	4	4	16	0
Solyc01g079080.2.1	0	0	0	0	0	0	0	0	0	0	0	0	0
Solyc01g079090.2.1	4	33	6	19	1	29	43	43	3	25	14	34	0
Solyc01g079100.2.1	0	0	0	0	0	0	0	0	0	0	0	0	0
Solyc01g079110.2.1	5	40	0	10	0	10	4	11	0	45	12	21	0

Solyc01g079150.2.1	4	70	7	93	0	104	13	41	2	8	5	8	0
Solyc01g079170.2.1	2	7	2	12	0	0	8	3	0	3	3	1	0
Solyc01g079180.2.1	0	0	0	0	0	0	0	0	0	0	0	0	0
Solyc01g079200.2.1	1	4	3	4	0	1	2	2	2	0	0	0	0
Solyc01g079210.2.1	4	6	0	1	0	11	30	19	0	28	1	35	0
Solyc01g079220.2.1	6	45	6	36	0	43	38	40	0	62	5	78	5
Solyc01g079230.2.1	28	160	27	143	11	419	272	477	40	367	92	298	9
Solyc01g079240.2.1	10	80	26	10	65	51	108	22	102	119	22	122	9
Solyc01g079250.2.1	18	321	33	220	18	488	287	485	31	805	121	655	24
Solyc01g079260.2.1	12	166	15	67	14	300	55	170	31	36	20	44	3
Solyc01g079290.1.1	0	0	0	0	0	0	0	0	0	0	0	0	0
Solyc01g079300.2.1	15	3	1	0	0	129	172	103	77	1	0	1	0
Solyc01g079310.2.1	5	24	2	22	0	138	37	56	9	37	10	55	0
Solyc01g079320.2.1	0	0	0	0	0	0	0	0	0	0	0	0	0
Solyc01g079330.2.1	6	21	21	2	4	41	125	50	39	39	41	27	6
Solyc01g079340.2.1	13	43	12	60	4	56	70	180	21	172	25	143	7
Solyc01g079350.2.1	10	86	9	65	0	107	58	208	3	217	10	169	1
Solyc01g079360.2.1	8	71	3	26	1	46	66	95	5	36	7	29	0
Solyc01g079370.2.1	0	0	0	0	0	0	0	0	0	0	0	0	0
Solyc01g079390.2.1	6	24	2	13	0	39	72	33	7	58	10	23	0
Solyc01g079400.2.1	3	3	0	14	4	5	3	5	0	48	31	60	2
Solyc01g079410.2.1	11	213	20	56	2	77	20	43	17	97	46	26	2
Solyc01g079420.2.1	2	79	4	31	0	122	94	123	13	154	11	140	2
Solyc01g079430.2.1	0	0	0	0	0	0	0	0	0	0	0	0	0
Solyc01g079440.1.1	0	0	0	0	0	0	0	0	0	0	0	0	0
Solyc01g079450.2.1	16	357	27	232	9	589	579	1020	54	405	71	255	14
Solyc01g079460.2.1	0	0	0	0	0	0	0	0	0	0	0	0	0
Solyc01g079470.2.1	5	656	32	151	1	17	559	158	5	12	26	3	0
Solyc01g079480.2.1	3	86	6	33	2	70	21	78	2	97	9	170	8
Solyc01g079490.2.1	0	0	0	0	0	0	0	0	0	0	0	0	0
Solyc01g079500.2.1	2	14	1	6	0	4	8	21	0	7	18	6	1
Solyc01g079510.2.1	22	92	11	119	1	184	121	238	24	244	30	197	2
Solyc01g079520.2.1	0	0	0	0	0	0	0	0	0	0	0	0	0
Solyc01g079530.2.1	5	114	5	55	9	49	19	37	1	0	0	16	0
Solyc01g079540.2.1	0	0	0	0	0	0	0	0	0	0	0	0	0
Solyc01g079550.2.1	0	0	0	0	0	0	0	0	0	0	0	0	0
Solyc01g079570.2.1	1	4	0	3	0	0	6	3	0	0	0	0	0
Solyc01g079580.2.1	5	108	3	7	2	6	15	3	1	73	9	38	0
Solyc01g079590.2.1	3	52	5	55	5	79	70	79	5	131	42	150	1
Solyc01g079600.2.1	15	192	53	140	11	618	127	268	23	136	20	129	22
Solyc01g079610.2.1	5	17	6	21	1	113	20	114	20	4	39	24	0

Solyc01g079620.2.1	1	1	0	0	0	0	5	7	0	10	0	4	0
Solyc01g079630.2.1	2	2	0	1	1	13	13	13	0	12	0	2	0
Solyc01g079640.2.1	0	0	0	0	0	0	0	0	0	0	0	0	0
Solyc01g079650.2.1	5	20	2	9	1	33	18	33	10	23	2	32	1
Solyc01g079660.2.1	6	164	28	55	15	14	0	3	1	1	0	2	2
Solyc01g079680.2.1	12	61	7	50	3	63	93	79	11	92	50	114	0
Solyc01g079690.2.1	8	31	8	28	0	88	46	152	8	55	12	26	0
Solyc01g079700.2.1	1	0	0	0	2	1	2	0	4	0	3	0	1
Solyc01g079710.2.1	4	135	4	115	0	1195	58	266	5	571	68	351	0
Solyc01g079720.2.1	10	32	6	28	1	44	52	73	23	75	51	68	4
Solyc01g079740.2.1	0	0	0	0	0	0	0	0	0	0	0	0	0
Solyc01g079750.2.1	0	0	0	0	0	0	0	0	0	0	0	0	0
Solyc01g079760.2.1	2	12	4	1	0	59	25	69	1	35	4	23	0
Solyc01g079770.2.1	0	0	0	0	0	0	0	0	0	0	0	0	0
Solyc01g079780.2.1	10	224	8	93	2	230	70	375	9	500	43	436	2
Solyc01g079790.2.1	9	342	82	132	4	1	81	14	4	19	12	17	0
Solyc01g079800.2.1	0	0	0	0	0	0	0	0	0	0	0	0	0
Solyc01g079810.2.1	4	11	1	13	0	72	4	65	6	14	3	13	0
Solyc01g079820.2.1	4	123	32	111	14	113	246	143	31	138	41	136	7
Solyc01g079840.2.1	7	58	5	49	6	123	41	127	10	99	33	104	2
Solyc01g079850.2.1	0	0	0	0	0	0	0	0	0	0	0	0	0
Solyc01g079860.2.1	6	24	4	21	0	33	40	41	3	48	21	57	6
Solyc01g079870.2.1	6	302	14	195	9	1462	393	1453	89	667	94	485	11
Solyc01g079880.2.1	2	465	43	437	4	357	136	536	1	873	334	843	37
Solyc01g079930.1.1	0	0	0	0	0	0	0	0	0	0	0	0	0
Solyc01g079940.2.1	1	10	3	2	2	67	2	29	0	0	0	0	0
Solyc01g079950.2.1	0	0	0	0	0	0	0	0	0	0	0	0	0
Solyc01g079960.2.1	0	0	0	0	0	0	0	0	0	0	0	0	0
Solyc01g079980.2.1	0	0	0	0	0	0	0	0	0	0	0	0	0
Solyc01g080000.1.1	0	0	0	0	0	0	0	0	0	0	0	0	0
Solyc01g080010.2.1	10	78	7	91	21	2516	2175	440	544	153	9	284	1
Solyc01g080020.2.1	17	174	62	139	28	80	733	306	45	535	68	319	22
Solyc01g080040.2.1	2	13	1	7	0	33	23	21	3	50	3	31	0
Solyc01g080070.2.1	12	115	12	48	6	11	12	32	5	274	110	202	17
Solyc01g080080.2.1	2	19	1	29	3	13	11	19	0	29	2	46	0
Solyc01g080090.2.1	1	8	1	6	0	14	3	9	0	17	1	11	0
Solyc01g080100.2.1	0	0	0	0	0	0	0	0	0	0	0	0	0
Solyc01g080110.2.1	6	33	7	16	3	35	38	51	10	39	22	36	1
Solyc01g080130.2.1	10	182	10	88	3	51	56	67	8	273	57	118	2
Solyc01g080140.2.1	2	22	3	14	2	75	62	49	6	31	16	31	0
Solyc01g080150.2.1	2	17	3	4	0	15	6	44	0	8	1	13	0

Solyc01g080160.2.1	10	56	7	26	3	172	63	93	4	23	34	26	8
Solyc01g080170.2.1	9	48	4	28	2	65	71	119	3	63	9	51	1
Solyc01g080180.2.1	0	0	0	0	0	0	0	0	0	0	0	0	0
Solyc01g080190.2.1	1	2	0	5	0	0	4	1	0	3	0	8	0
Solyc01g080200.2.1	18	91	10	80	0	159	66	294	16	126	26	92	0
Solyc01g080210.2.1	1	3	0	0	1	7	3	3	0	0	2	1	0
Solyc01g080220.2.1	2	35	0	29	0	121	13	110	0	6	0	0	0
Solyc01g080240.2.1	16	456	54	199	29	471	264	546	88	232	72	204	10
Solyc01g080250.2.1	16	224	20	121	9	689	161	624	60	473	65	281	1
Solyc01g080260.2.1	2	5	1	0	0	11	36	9	0	0	0	0	0
Solyc01g080270.2.1	5	27	5	25	0	121	68	44	11	89	23	57	2
Solyc01g080280.2.1	8	952	175	683	2	18	145	40	1	24	26	59	0
Solyc01g080290.2.1	0	0	0	0	0	0	0	0	0	0	0	0	0
Solyc01g080300.2.1	0	0	0	0	0	0	0	0	0	0	0	0	0
Solyc01g080310.2.1	4	3	5	8	0	25	15	20	1	12	10	9	0
Solyc01g080320.1.1	0	0	0	0	0	0	0	0	0	0	0	0	0
Solyc01g080330.2.1	6	612	64	422	9	785	379	904	84	1369	147	792	5
Solyc01g080340.2.1	8	37	3	14	2	72	101	81	12	110	38	55	0
Solyc01g080350.2.1	1	4	0	0	0	6	7	2	3	1	3	1	0
Solyc01g080360.2.1	12	39	5	51	0	172	74	143	15	174	13	140	5
Solyc01g080370.2.1	0	0	0	0	0	0	0	0	0	0	0	0	0
Solyc01g080380.2.1	0	0	0	0	0	0	0	0	0	0	0	0	0
Solyc01g080400.2.1	5	17	2	9	0	32	36	50	0	37	7	25	1
Solyc01g080410.2.1	0	0	0	0	0	0	0	0	0	0	0	0	0
Solyc01g080430.2.1	15	93	9	48	13	184	121	265	19	185	39	161	5
Solyc01g080450.2.1	0	0	0	0	0	0	0	0	0	0	0	0	0
Solyc01g080460.2.1	26	1779	186	1467	35	1109	1139	1618	91	4268	1200	2953	47
Solyc01g080470.2.1	4	12	0	19	0	8	15	21	0	14	10	9	0
Solyc01g080480.2.1	0	0	0	0	0	0	0	0	0	0	0	0	0
Solyc01g080490.2.1	0	0	0	0	0	0	0	0	0	0	0	0	0
Solyc01g080500.2.1	6	850	55	552	10	752	117	501	5	334	32	280	11
Solyc01g080510.2.1	6	173	64	123	19	47	195	148	21	102	14	62	4
Solyc01g080520.2.1	0	0	0	0	0	0	0	0	0	0	0	0	0
Solyc01g080530.2.1	3	14	5	6	0	9	20	26	4	39	7	23	0
Solyc01g080540.2.1	7	216	12	142	8	158	79	273	11	35	7	44	2
Solyc01g080570.2.1	0	0	0	0	0	0	0	0	0	0	0	0	0
Solyc01g080580.2.1	0	0	0	0	0	0	0	0	0	0	0	0	0
Solyc01g080590.2.1	0	0	0	0	0	0	0	0	0	0	0	0	0
Solyc01g080600.2.1	3	14	3	14	3	4	2	8	0	13	7	17	0
Solyc01g080620.2.1	13	50	10	37	5	80	77	97	18	131	17	97	3
Solyc01g080640.2.1	52	1113	82	472	30	3715	2284	3528	658	784	490	426	20

Solyc01g080650.2.1	0	0	0	0	0	0	0	0	0	0	0	0	0
Solyc01g080660.2.1	2	2	0	0	0	4	6	3	1	5	6	5	0
Solyc01g080670.2.1	12	127	7	93	11	210	146	279	14	249	45	119	0
Solyc01g080680.2.1	4	0	1	0	0	9	136	8	0	81	14	46	7
Solyc01g080690.2.1	6	25	6	12	12	34	55	53	9	109	4	41	2
Solyc01g080700.2.1	8	12	8	10	0	10	35	13	2	33	5	29	2
Solyc01g080720.2.1	1	0	0	0	0	5	4	0	7	0	0	0	0
Solyc01g080750.2.1	0	0	0	0	0	0	0	0	0	0	0	0	0
Solyc01g080760.2.1	11	6	0	7	0	32	10	39	1	18	14	16	0
Solyc01g080770.2.1	1	6	0	1	0	4	8	25	0	0	3	1	0
Solyc01g080780.2.1	17	55	11	50	7	67	236	135	14	51	19	52	3
Solyc01g080790.2.1	0	0	0	0	0	0	0	0	0	0	0	0	0
Solyc01g080810.2.1	17	182	37	74	3	73	250	131	7	67	21	78	1
Solyc01g080830.2.1	0	0	0	0	0	0	0	0	0	0	0	0	0
Solyc01g080840.2.1	22	399	58	275	12	259	356	389	22	210	49	169	2
Solyc01g080850.2.1	13	31	9	8	1	106	94	108	20	126	42	148	2
Solyc01g080860.2.1	7	70	7	58	2	81	98	127	15	174	48	199	4
Solyc01g080870.2.1	16	178	15	102	0	930	177	536	2	32	28	39	0
Solyc01g080880.2.1	5	90	3	76	1	247	39	248	13	235	45	194	5
Solyc01g080890.2.1	5	29	1	7	3	14	22	13	3	116	20	127	11
Solyc01g080900.2.1	5	12	1	5	0	7	1	12	0	55	15	66	0
Solyc01g080910.2.1	18	104	8	52	4	72	113	144	6	1139	31	894	6
Solyc01g080930.2.1	7	30	4	20	0	121	74	117	5	97	29	122	0
Solyc01g080940.2.1	8	33	5	47	2	88	87	70	14	82	41	57	1
Solyc01g080970.2.1	0	0	0	0	0	0	0	0	0	0	0	0	0
Solyc01g080990.2.1	0	0	0	0	0	0	0	0	0	0	0	0	0
Solyc01g081000.2.1	0	0	0	0	0	0	0	0	0	0	0	0	0
Solyc01g081010.2.1	17	175	10	149	10	428	177	496	32	2637	181	1893	25
Solyc01g081020.1.1	0	0	0	0	0	0	0	0	0	0	0	0	0
Solyc01g081030.2.1	9	53	2	42	0	70	83	149	8	102	28	88	4
Solyc01g081040.2.1	0	0	0	0	0	0	0	0	0	0	0	0	0
Solyc01g081050.2.1	10	9	5	5	5	52	44	47	2	43	20	25	8
Solyc01g081060.2.1	9	486	23	223	36	597	818	613	154	288	307	27	4
Solyc01g081070.2.1	5	46	2	28	2	88	38	89	11	90	12	100	0
Solyc01g081110.2.1	0	0	0	0	0	0	0	0	0	0	0	0	0
Solyc01g081120.2.1	0	0	0	0	0	0	0	0	0	0	0	0	0
Solyc01g081140.1.1	0	0	0	0	0	0	0	0	0	0	0	0	0
Solyc01g081150.2.1	7	67	9	37	5	85	81	147	11	170	43	169	0
Solyc01g081190.2.1	3	148	30	68	1	76	71	131	17	154	9	93	0
Solyc01g081200.2.1	2	22	0	27	0	3	23	28	1	14	6	11	0
Solyc01g081210.2.1	0	0	0	0	0	0	0	0	0	0	0	0	0

Solyc01g081240.2.1	0	0	0	0	0	0	0	0	0	0	0	0	0
Solyc01g081250.2.1	0	0	0	0	0	0	0	0	0	0	0	0	0
Solyc01g081270.2.1	1	8	0	16	0	6	33	53	2	16	0	9	0
Solyc01g081290.2.1	5	21	2	22	1	70	24	41	5	55	16	25	0
Solyc01g081300.2.1	5	6	2	0	3	27	43	96	5	39	13	41	0
Solyc01g081310.2.1	9	124	10	64	12	995	174	560	81	235	7	229	1
Solyc01g081320.2.1	1	4	0	2	0	0	4	4	1	0	0	7	0
Solyc01g081330.2.1	0	0	0	0	0	0	0	0	0	0	0	0	0
Solyc01g081370.2.1	0	0	0	0	0	0	0	0	0	0	0	0	0
Solyc01g081380.2.1	0	0	0	0	0	0	0	0	0	0	0	0	0
Solyc01g081390.2.1	4	91	11	54	1	40	30	157	0	171	10	95	2
Solyc01g081400.2.1	0	0	0	0	0	0	0	0	0	0	0	0	0
Solyc01g081410.2.1	0	0	0	0	0	0	0	0	0	0	0	0	0
Solyc01g081420.1.1	0	0	0	0	0	0	0	0	0	0	0	0	0
Solyc01g081430.2.1	11	33	2	28	2	103	55	104	4	72	28	93	1
Solyc01g081440.2.1	2	51	4	33	8	16	33	29	0	50	18	37	0
Solyc01g081450.2.1	4	564	40	379	20	1951	301	489	28	199	64	217	10
Solyc01g081460.2.1	0	0	0	0	0	0	0	0	0	0	0	0	0
Solyc01g081470.2.1	2	12	3	8	0	42	8	21	2	16	2	4	0
Solyc01g081480.2.1	2	22	11	5	0	0	3	0	0	3	1	4	1
Solyc01g081490.2.1	8	97	26	53	0	53	200	151	7	69	34	66	5
Solyc01g081500.2.1	7	100	11	73	11	219	72	226	34	146	87	133	9
Solyc01g081510.2.1	5	9	1	5	0	2	20	16	1	17	4	21	0
Solyc01g081520.2.1	12	83	15	158	6	580	207	522	52	238	84	476	7
Solyc01g081540.2.1	9	109	13	55	7	40	52	59	35	290	30	141	5
Solyc01g081560.2.1	0	0	0	0	0	0	0	0	0	0	0	0	0
Solyc01g081570.2.1	8	362	31	166	12	33	101	65	5	267	28	88	2
Solyc01g081600.2.1	0	0	0	0	0	0	0	0	0	0	0	0	0
Solyc01g081610.2.1	14	107	9	80	4	134	133	121	22	147	50	81	3
Solyc01g081620.2.1	0	0	0	0	0	0	0	0	0	0	0	0	0
Solyc01g081630.2.1	0	0	0	0	0	0	0	0	0	0	0	0	0
Solyc01g081640.2.1	0	0	0	0	0	0	0	0	0	0	0	0	0
Solyc01g086640.2.1	7	40	1	49	2	18	112	49	10	7	6	27	0
Solyc01g086650.2.1	1	2	18	2	0	13	39	6	3	53	24	16	7
Solyc01g086670.2.1	0	0	0	0	0	0	0	0	0	0	0	0	0
Solyc01g086680.2.1	0	0	0	0	0	0	0	0	0	0	0	0	0
Solyc01g086690.2.1	0	0	0	0	0	0	0	0	0	0	0	0	0
Solyc01g086710.2.1	10	20	2	21	2	147	73	186	15	126	14	90	1
Solyc01g086720.2.1	0	0	0	0	0	0	0	0	0	0	0	0	0
Solyc01g086730.2.1	0	0	0	0	0	0	0	0	0	0	0	0	0
Solyc01g086740.2.1	11	80	14	87	4	296	117	228	26	223	80	197	2

Solyc01g086750.2.1	29	121	26	87	11	336	294	536	75	412	170	276	5
Solyc01g086810.2.1	9	45	2	91	1	43	41	154	4	128	6	111	2
Solyc01g086820.2.1	0	0	0	0	0	0	0	0	0	0	0	0	0
Solyc01g086850.2.1	4	31	2	12	0	79	61	78	8	48	12	43	4
Solyc01g086860.2.1	2	20	0	18	0	22	8	40	3	42	3	35	0
Solyc01g086870.2.1	9	107	9	67	0	500	59	268	14	134	6	79	0
Solyc01g086890.2.1	15	239	15	161	3	832	125	545	23	453	70	316	4
Solyc01g086900.2.1	2	7	5	1	0	0	16	6	0	4	0	5	0
Solyc01g086910.2.1	3	121	1	72	35	3	5	11	2	707	43	826	18
Solyc01g086920.2.1	4	485	19	255	35	10	11	27	1	1779	63	1886	25
Solyc01g086930.2.1	0	0	0	0	0	0	0	0	0	0	0	0	0
Solyc01g086940.2.1	2	3	0	10	0	1	5	9	0	9	4	7	0
Solyc01g086950.2.1	7	62	3	22	2	95	47	66	3	39	41	41	1
Solyc01g086960.2.1	5	257	38	91	19	212	53	150	9	188	26	143	3
Solyc01g086970.2.1	2	60	6	18	1	264	119	204	22	143	38	76	2
Solyc01g086980.2.1	11	38	6	46	0	130	36	123	8	33	8	48	3
Solyc01g086990.2.1	12	109	7	77	6	226	93	193	17	300	28	260	5
Solyc01g087020.2.1	3	43	1	23	2	28	39	15	7	15	18	28	1
Solyc01g087030.2.1	12	44	12	29	6	70	87	103	14	112	19	69	2
Solyc01g087040.2.1	7	53	10	22	0	5	68	45	4	32	12	20	2
Solyc01g087050.2.1	0	0	0	0	0	0	0	0	0	0	0	0	0
Solyc01g087060.2.1	0	0	0	0	0	0	0	0	0	0	0	0	0
Solyc01g087070.2.1	0	0	0	0	0	0	0	0	0	0	0	0	0
Solyc01g087080.2.1	0	0	0	0	0	0	0	0	0	0	0	0	0
Solyc01g087090.2.1	2	72	19	36	11	89	341	97	12	129	23	47	4
Solyc01g087120.2.1	4	124	2	70	2	220	60	271	22	201	40	173	1
Solyc01g087130.2.1	0	0	0	0	0	0	0	0	0	0	0	0	0
Solyc01g087140.2.1	0	0	0	0	0	0	0	0	0	0	0	0	0
Solyc01g087150.2.1	0	0	0	0	0	0	0	0	0	0	0	0	0
Solyc01g087160.2.1	3	60	6	42	5	166	66	94	21	291	73	203	1
Solyc01g087170.2.1	8	94	8	81	0	135	51	164	20	217	30	169	5
Solyc01g087180.2.1	4	1	0	0	0	73	4	31	1	0	4	0	0
Solyc01g087190.2.1	4	89	9	35	0	9	24	37	3	44	8	22	0
Solyc01g087200.2.1	2	12	1	5	3	43	16	41	2	14	1	2	0
Solyc01g087210.2.1	26	1063	85	682	117	339	319	747	98	3932	496	3319	69
Solyc01g087230.2.1	6	73	1	58	2	605	37	405	8	48	4	30	0
Solyc01g087240.2.1	5	65	5	107	3	212	34	228	15	60	10	66	0
Solyc01g087250.2.1	16	104	27	65	8	942	783	556	53	97	147	80	3
Solyc01g087260.2.1	11	770	92	499	2	477	71	477	0	920	33	892	5
Solyc01g087270.2.1	1	51	4	22	1	1	5	2	0	0	2	0	0
Solyc01g087280.1.1	0	0	0	0	0	0	0	0	0	0	0	0	0

Solyc01g087290.2.1	9	46	9	34	4	35	93	39	9	88	12	64	8
Solyc01g087300.2.1	8	38	2	18	1	75	35	82	8	31	23	39	1
Solyc01g087310.2.1	1	1	0	0	0	1	10	3	0	11	1	3	0
Solyc01g087320.2.1	1	0	0	0	0	5	0	3	0	0	0	8	0
Solyc01g087330.2.1	0	0	0	0	0	0	0	0	0	0	0	0	0
Solyc01g087340.2.1	0	0	0	0	0	0	0	0	0	0	0	0	0
Solyc01g087350.2.1	0	0	0	0	0	0	0	0	0	0	0	0	0
Solyc01g087370.2.1	2	6	0	4	0	1	6	7	0	11	6	1	0
Solyc01g087380.2.1	0	0	0	0	0	0	0	0	0	0	0	0	0
Solyc01g087400.2.1	2	5	4	0	0	9	6	12	0	21	1	9	0
Solyc01g087410.2.1	1	2	0	4	0	3	4	1	0	3	0	0	0
Solyc01g087420.2.1	1	11	0	14	0	10	5	66	0	3	0	12	0
Solyc01g087430.2.1	5	10	0	11	0	38	20	34	3	11	3	15	0
Solyc01g087440.2.1	0	0	0	0	0	0	0	0	0	0	0	0	0
Solyc01g087450.2.1	2	2	3	1	8	9	10	4	0	1	2	1	0
Solyc01g087470.2.1	3	6	1	6	0	65	7	47	0	17	5	15	0
Solyc01g087490.2.1	11	111	5	76	16	319	99	209	23	87	10	71	0
Solyc01g087500.2.1	0	0	0	0	0	0	0	0	0	0	0	0	0
Solyc01g087520.2.1	8	335	28	133	1	49	160	91	3	118	26	106	0
Solyc01g087530.2.1	9	34	5	19	0	59	70	83	30	46	16	27	2
Solyc01g087540.2.1	6	67	6	24	4	23	49	39	37	81	63	82	2
Solyc01g087550.2.1	4	27	0	8	0	38	16	60	4	43	5	54	0
Solyc01g087560.2.1	9	103	10	24	0	57	39	109	8	438	95	352	4
Solyc01g087570.2.1	1	8	0	1	0	5	9	5	0	7	1	5	1
Solyc01g087580.2.1	0	0	0	0	0	0	0	0	0	0	0	0	0
Solyc01g087590.2.1	1	9	6	1	2	0	0	2	0	1	0	4	0
Solyc01g087600.2.1	9	50	3	43	10	25	36	58	9	112	25	75	2
Solyc01g087610.2.1	8	30	5	17	1	67	88	107	6	65	25	59	0
Solyc01g087620.2.1	3	59	7	47	6	287	48	194	2	245	24	123	10
Solyc01g087630.1.1	0	0	0	0	0	0	0	0	0	0	0	0	0
Solyc01g087640.2.1	5	39	5	9	2	231	49	240	3	88	9	106	0
Solyc01g087650.2.1	7	55	6	27	1	84	50	97	8	40	11	47	0
Solyc01g087660.2.1	0	0	0	0	0	0	0	0	0	0	0	0	0
Solyc01g087670.2.1	8	15	1	14	2	41	57	90	21	66	29	38	10
Solyc01g087680.2.1	12	272	41	138	1	231	221	286	5	136	13	118	6
Solyc01g087690.1.1	0	0	0	0	0	0	0	0	0	0	0	0	0
Solyc01g087710.2.1	0	0	0	0	0	0	0	0	0	0	0	0	0
Solyc01g087720.2.1	5	29	3	37	2	83	33	67	6	191	35	171	0
Solyc01g087730.2.1	11	306	102	195	3	141	472	373	9	160	61	137	1
Solyc01g087740.1.1	0	0	0	0	0	0	0	0	0	0	0	0	0
Solyc01g087750.2.1	3	283	27	140	0	16	42	41	1	0	5	1	0

Solyc01g087780.2.1	0	0	0	0	0	0	0	0	0	0	0	0	0
Solyc01g087790.2.1	0	0	0	0	0	0	0	0	0	0	0	0	0
Solyc01g087800.2.1	0	0	0	0	0	0	0	0	0	0	0	0	0
Solyc01g087810.2.1	0	0	0	0	0	0	0	0	0	0	0	0	0
Solyc01g087820.2.1	9	82	21	58	4	166	27	697	0	31	4	63	0
Solyc01g087830.1.1	0	0	0	0	0	0	0	0	0	0	0	0	0
Solyc01g087840.2.1	0	0	0	0	0	0	0	0	0	0	0	0	0
Solyc01g087850.2.1	19	70	6	12	19	111	446	480	56	485	7	418	3
Solyc01g087860.2.1	1	13	0	0	0	7	1	4	1	3	3	0	0
Solyc01g087870.2.1	2	7	0	4	0	15	17	28	1	6	0	2	0
Solyc01g087880.2.1	0	0	0	0	0	0	0	0	0	0	0	0	0
Solyc01g087890.2.1	16	52	8	31	3	61	155	149	10	123	12	89	3
Solyc01g087900.2.1	1	40	2	6	0	37	17	49	3	60	3	32	0
Solyc01g087910.2.1	8	17	4	22	4	20	50	75	13	86	7	51	1
Solyc01g087940.1.1	0	0	0	0	0	0	0	0	0	0	0	0	0
Solyc01g087970.2.1	7	415	17	209	9	35	77	51	17	0	7	0	0
Solyc01g087980.2.1	10	177	30	99	2	359	167	98	10	90	9	252	1
Solyc01g087990.2.1	1	0	2	0	0	3	3	0	0	8	2	14	0
Solyc01g088000.2.1	7	68	4	38	0	87	19	102	0	97	13	80	1
Solyc01g088020.2.1	12	120	16	135	14	366	186	514	69	287	93	280	6
Solyc01g088030.2.1	4	13	1	14	0	21	39	32	1	4	4	7	0
Solyc01g088040.2.1	2	315	22	87	5	631	206	577	36	310	35	226	1
Solyc01g088050.2.1	7	78	9	30	5	54	33	69	9	1	0	7	0
Solyc01g088060.2.1	1	2	0	1	4	0	5	2	0	4	1	3	0
Solyc01g088070.2.1	0	0	0	0	0	0	0	0	0	0	0	0	0
Solyc01g088080.2.1	6	53	3	28	5	136	85	259	14	235	50	164	8
Solyc01g088090.2.1	15	249	29	130	3	388	108	793	7	197	48	144	2
Solyc01g088100.2.1	5	151	13	137	10	145	88	272	20	137	38	120	2
Solyc01g088130.2.1	0	0	0	0	0	0	0	0	0	0	0	0	0
Solyc01g088140.2.1	2	3	1	12	0	4	2	11	0	20	2	3	0
Solyc01g088150.2.1	9	30	4	8	3	61	65	49	16	25	18	25	0
Solyc01g088160.2.1	0	0	0	0	0	0	0	0	0	0	0	0	0
Solyc01g088170.2.1	14	60	6	51	0	190	101	221	4	75	12	65	0
Solyc01g088200.2.1	0	0	0	0	0	0	0	0	0	0	0	0	0
Solyc01g088210.2.1	0	0	0	0	0	0	0	0	0	0	0	0	0
Solyc01g088220.2.1	0	0	0	0	0	0	0	0	0	0	0	0	0
Solyc01g088240.2.1	6	17	0	13	4	64	8	50	1	100	21	71	0
Solyc01g088250.2.1	3	21	0	2	0	5	4	9	0	33	6	44	1
Solyc01g088260.2.1	0	0	0	0	0	0	0	0	0	0	0	0	0
Solyc01g088300.2.1	0	0	0	0	0	0	0	0	0	0	0	0	0
Solyc01g088310.2.1	1	32	0	44	0	23	2	28	1	56	6	27	0

Solyc01g088330.1.1	0	0	0	0	0	0	0	0	0	0	0	0	0
Solyc01g088350.2.1	0	0	0	0	0	0	0	0	0	0	0	0	0
Solyc01g088360.2.1	0	0	0	0	0	0	0	0	0	0	0	0	0
Solyc01g088370.2.1	14	204	28	203	32	746	607	1005	154	552	229	460	7
Solyc01g088380.1.1	0	0	0	0	0	0	0	0	0	0	0	0	0
Solyc01g088390.2.1	1	9	1	2	0	0	6	4	0	6	3	0	0
Solyc01g088400.2.1	23	55	6	7	51	524	567	129	1157	261	2961	17	42
Solyc01g088410.2.1	0	0	0	0	0	0	0	0	0	0	0	0	0
Solyc01g088420.2.1	0	0	0	0	0	0	0	0	0	0	0	0	0
Solyc01g088430.2.1	0	0	0	0	0	0	0	0	0	0	0	0	0
Solyc01g088440.2.1	1	6	5	0	0	0	6	4	1	2	0	9	1
Solyc01g088450.2.1	0	0	0	0	0	0	0	0	0	0	0	0	0
Solyc01g088460.2.1	2	31	0	1	1	10	13	7	4	7	0	5	0
Solyc01g088470.2.1	5	53	3	27	5	18	28	30	3	32	3	31	4
Solyc01g088480.2.1	5	128	14	41	8	122	56	97	35	171	50	139	3
Solyc01g088490.2.1	0	0	0	0	0	0	0	0	0	0	0	0	0
Solyc01g088510.2.1	1	14	2	9	0	8	11	14	1	17	3	17	0
Solyc01g088520.2.1	0	0	0	0	0	0	0	0	0	0	0	0	0
Solyc01g088530.2.1	2	6	0	8	0	6	14	0	5	0	1	0	0
Solyc01g088540.1.1	0	0	0	0	0	0	0	0	0	0	0	0	0
Solyc01g088550.2.1	7	82	9	57	9	166	90	100	1	201	25	149	2
Solyc01g088560.2.1	3	181	7	95	0	375	75	279	12	126	13	89	2
Solyc01g088570.2.1	3	89	10	33	7	221	73	196	15	102	24	83	4
Solyc01g088590.2.1	4	6	0	1	2	3	4	7	0	49	23	11	0
Solyc01g088600.2.1	3	13	4	11	0	13	15	25	3	23	11	15	1
Solyc01g088610.2.1	2	36	2	9	0	11	5	18	0	28	5	30	0
Solyc01g088620.2.1	0	0	0	0	0	0	0	0	0	0	0	0	0
Solyc01g088630.1.1	0	0	0	0	0	0	0	0	0	0	0	0	0
Solyc01g088640.2.1	2	7	1	4	0	4	11	8	1	2	1	2	0
Solyc01g088650.2.1	5	16	0	3	0	15	105	61	6	29	1	16	0
Solyc01g088660.2.1	3	22	0	9	2	78	105	182	2	248	1	167	3
Solyc01g088670.1.1	0	0	0	0	0	0	0	0	0	0	0	0	0
Solyc01g088680.2.1	1	3	1	10	0	1	6	4	0	46	0	41	2
Solyc01g088690.2.1	3	6	3	7	1	20	53	35	5	33	12	43	0
Solyc01g088700.2.1	51	681	68	534	20	1705	838	2594	157	1144	281	1216	18
Solyc01g088710.2.1	2	8	0	3	2	12	3	13	1	14	4	5	0
Solyc01g088720.2.1	3	19	2	4	0	31	25	39	3	19	11	14	1
Solyc01g088730.2.1	6	24	3	16	1	42	73	55	12	79	18	82	2
Solyc01g088740.2.1	2	8	0	8	0	4	11	7	2	0	2	6	0
Solyc01g088750.2.1	1	2	0	0	0	2	4	3	5	4	0	2	0
Solyc01g088760.2.1	3	16	0	24	1	83	17	44	0	28	3	42	0

Solyc01g088770.2.1	0	0	0	0	0	0	0	0	0	0	0	0	0
Solyc01g088780.2.1	0	0	0	0	0	0	0	0	0	0	0	0	0
Solyc01g088800.2.1	12	86	9	51	4	366	88	283	24	206	22	102	6
Solyc01g088810.2.1	0	0	0	0	0	0	0	0	0	0	0	0	0
Solyc01g088820.2.1	3	4	0	3	0	5	4	9	4	18	11	15	0
Solyc01g088830.2.1	0	0	0	0	0	0	0	0	0	0	0	0	0
Solyc01g089850.2.1	0	0	0	0	0	0	0	0	0	0	0	0	0
Solyc01g089860.2.1	9	75	21	35	8	61	117	115	12	187	43	107	6
Solyc01g089870.2.1	17	92	13	57	5	175	149	256	7	104	48	95	2
Solyc01g089880.2.1	0	0	0	0	0	0	0	0	0	0	0	0	0
Solyc01g089890.2.1	0	0	0	0	0	0	0	0	0	0	0	0	0
Solyc01g089900.2.1	2	1	0	0	0	4	6	3	1	8	2	6	0
Solyc01g089910.2.1	0	0	0	0	0	0	0	0	0	0	0	0	0
Solyc01g089950.2.1	0	0	0	0	0	0	0	0	0	0	0	0	0
Solyc01g089960.2.1	0	0	0	0	0	0	0	0	0	0	0	0	0
Solyc01g089970.2.1	6	486	22	158	11	844	295	760	64	1181	221	1185	10
Solyc01g089980.2.1	3	23	5	6	0	7	19	22	1	28	12	16	6
Solyc01g090040.2.1	0	0	0	0	0	0	0	0	0	0	0	0	0
Solyc01g090120.2.1	1	8	0	6	0	31	4	17	0	0	0	0	0
Solyc01g090140.1.1	0	0	0	0	0	0	0	0	0	0	0	0	0
Solyc01g090160.2.1	0	0	0	0	0	0	0	0	0	0	0	0	0
Solyc01g090170.2.1	3	34	3	15	0	68	17	50	0	75	5	48	0
Solyc01g090180.2.1	3	22	2	18	0	0	14	18	3	73	6	30	2
Solyc01g090190.2.1	15	369	35	225	17	198	318	411	32	575	183	316	6
Solyc01g090200.2.1	7	35	2	15	7	53	47	62	7	46	4	35	0
Solyc01g090210.2.1	0	0	0	0	0	0	0	0	0	0	0	0	0
Solyc01g090220.2.1	6	33	11	4	0	45	27	29	4	85	10	72	0
Solyc01g090230.2.1	10	86	4	70	10	374	102	282	26	171	61	113	8
Solyc01g090240.2.1	2	2	0	5	0	2	0	4	1	5	4	2	2
Solyc01g090250.2.1	1	1	0	2	0	3	12	10	4	0	2	2	0
Solyc01g090260.2.1	6	10	3	7	0	55	46	54	4	13	12	1	0
Solyc01g090270.2.1	13	235	26	179	7	131	95	264	13	523	60	258	4
Solyc01g090280.2.1	6	39	6	18	0	154	77	115	36	149	49	102	10
Solyc01g090290.2.1	8	50	2	17	0	123	87	96	14	63	41	96	0
Solyc01g090310.2.1	0	0	0	0	0	0	0	0	0	0	0	0	0
Solyc01g090330.1.1	0	0	0	0	0	0	0	0	0	0	0	0	0
Solyc01g090340.2.1	2	337	4	140	2	101	1	45	0	30	9	19	0
Solyc01g090350.2.1	0	0	0	0	0	0	0	0	0	0	0	0	0
Solyc01g090360.2.1	0	0	0	0	0	0	0	0	0	0	0	0	0
Solyc01g090380.2.1	2	14	2	9	0	8	13	8	4	39	5	70	0
Solyc01g090390.1.1	0	0	0	0	0	0	0	0	0	0	0	0	0

Solyc01g090400.2.1	0	0	0	0	0	0	0	0	0	0	0	0	0
Solyc01g090410.2.1	1	4	1	3	0	14	11	15	0	4	0	4	0
Solyc01g090420.2.1	10	47	4	22	0	78	58	87	11	66	10	54	0
Solyc01g090430.2.1	6	55	5	31	0	66	27	75	5	51	3	28	0
Solyc01g090440.2.1	0	0	0	0	0	0	0	0	0	0	0	0	0
Solyc01g090450.2.1	1	16	1	4	0	1	6	9	1	8	0	3	0
Solyc01g090460.2.1	1	3	2	1	0	4	16	1	3	21	6	5	0
Solyc01g090470.2.1	4	10	1	4	0	50	17	42	1	37	7	30	0
Solyc01g090480.2.1	7	50	5	28	3	70	36	94	11	61	21	47	0
Solyc01g090490.1.1	0	0	0	0	0	0	0	0	0	0	0	0	0
Solyc01g090500.2.1	1	6	4	9	1	7	14	13	0	10	12	18	5
Solyc01g090510.2.1	0	0	0	0	0	0	0	0	0	0	0	0	0
Solyc01g090530.1.1	0	0	0	0	0	0	0	0	0	0	0	0	0
Solyc01g090550.2.1	9	35	11	29	4	157	181	212	36	212	64	105	3
Solyc01g090560.2.1	0	0	0	0	0	0	0	0	0	0	0	0	0
Solyc01g090590.2.1	9	32	6	19	5	110	64	153	15	41	17	27	1
Solyc01g090630.2.1	0	0	0	0	0	0	0	0	0	0	0	0	0
Solyc01g090640.2.1	0	0	0	0	0	0	0	0	0	0	0	0	0
Solyc01g090650.1.1	0	0	0	0	0	0	0	0	0	0	0	0	0
Solyc01g090660.2.1	0	0	0	0	0	0	0	0	0	0	0	0	0
Solyc01g090670.2.1	5	11	0	16	0	14	17	23	1	23	5	43	0
Solyc01g090680.2.1	6	128	8	125	2	50	88	148	3	100	19	46	5
Solyc01g090690.2.1	12	81	8	51	4	288	158	346	29	436	65	326	5
Solyc01g090700.2.1	4	4	0	7	1	25	33	46	1	41	1	48	0
Solyc01g090710.2.1	0	0	0	0	0	0	0	0	0	0	0	0	0
Solyc01g090720.2.1	4	20	0	6	6	16	21	16	1	32	0	72	0
Solyc01g090730.2.1	0	0	0	0	0	0	0	0	0	0	0	0	0
Solyc01g090740.2.1	3	38	1	13	0	52	16	25	10	24	0	5	0
Solyc01g090750.2.1	7	85	2	124	0	189	40	438	8	970	29	815	1
Solyc01g090760.2.1	0	0	0	0	0	0	0	0	0	0	0	0	0
Solyc01g090770.2.1	6	21	2	4	0	62	68	62	10	107	20	43	2
Solyc01g090780.2.1	19	68	19	83	6	232	260	390	57	204	67	219	8
Solyc01g090790.2.1	8	349	17	342	6	211	14	116	1	226	11	162	0
Solyc01g090800.2.1	0	0	0	0	0	0	0	0	0	0	0	0	0
Solyc01g090810.2.1	0	0	0	0	0	0	0	0	0	0	0	0	0
Solyc01g090840.2.1	0	0	0	0	0	0	0	0	0	0	0	0	0
Solyc01g090850.2.1	11	18	3	24	0	96	76	123	24	61	19	23	0
Solyc01g090860.2.1	5	19	1	4	1	80	44	49	3	58	7	67	0
Solyc01g090870.1.1	0	0	0	0	0	0	0	0	0	0	0	0	0
Solyc01g090880.2.1	2	12	0	2	0	27	19	18	0	16	0	40	0
Solyc01g090890.2.1	0	0	0	0	0	0	0	0	0	0	0	0	0

Solyc01g090900.2.1	7	35	2	25	0	110	92	118	3	45	14	59	0
Solyc01g090910.2.1	5	46	4	23	2	69	14	83	4	37	8	8	0
Solyc01g090920.2.1	0	0	0	0	0	0	0	0	0	0	0	0	0
Solyc01g090940.2.1	4	19	2	17	4	29	26	22	0	21	14	23	0
Solyc01g090950.2.1	6	44	8	40	0	82	44	85	0	53	8	36	0
Solyc01g090970.2.1	1	230	7	68	0	0	0	0	0	0	0	0	0
Solyc01g090990.2.1	1	7	0	0	0	23	4	13	0	0	6	1	0
Solyc01g091000.2.1	0	0	0	0	0	0	0	0	0	0	0	0	0
Solyc01g091020.2.1	3	21	3	24	1	69	12	53	2	56	5	62	15
Solyc01g091030.2.1	0	0	0	0	0	0	0	0	0	0	0	0	0
Solyc01g091040.2.1	2	4	0	2	0	2	4	4	0	0	0	0	0
Solyc01g091050.2.1	7	258	40	151	45	1	40	1	5	0	0	2	0
Solyc01g091070.2.1	4	17	1	20	0	49	22	61	3	71	15	42	0
Solyc01g091080.2.1	7	16	1	26	3	24	18	66	7	70	30	59	1
Solyc01g091090.2.1	5	7	0	3	0	8	33	20	2	22	14	12	0
Solyc01g091100.2.1	0	0	0	0	0	0	0	0	0	0	0	0	0
Solyc01g091120.2.1	9	130	13	112	6	327	183	375	41	347	43	178	0
Solyc01g091130.2.1	8	40	3	30	0	96	57	89	12	19	23	10	0
Solyc01g091140.2.1	0	0	0	0	0	0	0	0	0	0	0	0	0
Solyc01g091160.2.1	4	5	1	0	0	62	49	93	3	88	22	84	1
Solyc01g091170.2.1	1	7	0	0	0	0	3	2	0	21	0	42	0
Solyc01g091180.2.1	0	0	0	0	0	0	0	0	0	0	0	0	0
Solyc01g091190.2.1	11	140	24	100	14	404	178	527	94	182	35	192	5
Solyc01g091200.2.1	2	13	3	6	0	5	4	11	0	1	3	2	0
Solyc01g091210.2.1	3	20	0	28	0	12	7	25	0	7	4	20	0
Solyc01g091220.2.1	0	0	0	0	0	0	0	0	0	0	0	0	0
Solyc01g091230.2.1	0	0	0	0	0	0	0	0	0	0	0	0	0
Solyc01g091240.2.1	0	0	0	0	0	0	0	0	0	0	0	0	0
Solyc01g091260.2.1	3	5	3	1	0	14	7	8	0	25	22	42	0
Solyc01g091270.2.1	12	22	7	11	3	232	73	95	45	85	246	97	21
Solyc01g091280.2.1	6	18	1	20	1	21	16	39	2	66	8	41	0
Solyc01g091300.2.1	0	0	0	0	0	0	0	0	0	0	0	0	0
Solyc01g091310.2.1	8	23	1	16	7	72	33	63	5	78	16	67	1
Solyc01g091320.2.1	6	930	35	380	1	7	7	14	0	108	112	7	0
Solyc01g091330.2.1	0	0	0	0	0	0	0	0	0	0	0	0	0
Solyc01g091340.2.1	1	2	2	5	0	6	7	6	0	42	6	19	0
Solyc01g091350.2.1	3	2	0	0	0	8	8	8	0	9	3	22	2
Solyc01g091360.2.1	0	0	0	0	0	0	0	0	0	0	0	0	0
Solyc01g091370.2.1	2	20	2	9	0	2	3	21	4	40	9	24	3
Solyc01g091380.2.1	1	2	0	0	0	6	0	2	1	0	0	1	0
Solyc01g091390.2.1	3	10	1	15	0	14	3	13	0	25	6	12	0

Solyc01g091420.1.1	1	5	0	3	0	73	27	23	0	0	13	0	0
Solyc01g091430.1.1	0	0	0	0	0	0	0	0	0	0	0	0	0
Solyc01g091450.2.1	4	14	6	12	0	1	12	24	0	6	2	3	0
Solyc01g091460.2.1	14	109	18	85	1	256	143	348	23	174	39	170	4
Solyc01g091470.2.1	0	0	0	0	0	0	0	0	0	0	0	0	0
Solyc01g091480.2.1	1	3	1	0	0	0	2	3	0	1	0	3	0
Solyc01g091490.2.1	1	4	2	0	0	2	2	5	0	0	0	0	0
Solyc01g091500.2.1	4	7	2	14	1	22	18	11	2	14	7	12	3
Solyc01g091510.2.1	5	27	3	23	1	38	47	90	8	148	18	68	0
Solyc01g091520.2.1	12	306	22	194	8	124	165	292	22	667	76	461	9
Solyc01g091530.2.1	13	565	135	354	106	16	176	72	53	1872	242	1466	63
Solyc01g091540.1.1	0	0	0	0	0	0	0	0	0	0	0	0	0
Solyc01g091550.2.1	2	21	1	12	0	6	6	19	0	19	0	13	0
Solyc01g091570.2.1	1	9	0	5	0	1	3	6	0	12	1	9	0
Solyc01g091580.2.1	5	56	7	17	0	33	63	33	2	24	4	16	0
Solyc01g091590.2.1	0	0	0	0	0	0	0	0	0	0	0	0	0
Solyc01g091600.2.1	4	28	5	7	0	32	10	43	8	11	7	12	0
Solyc01g091610.1.1	0	0	0	0	0	0	0	0	0	0	0	0	0
Solyc01g091620.2.1	4	35	9	28	0	60	28	80	12	68	8	60	7
Solyc01g091630.2.1	9	202	11	90	9	1163	55	261	27	129	27	70	9
Solyc01g091640.2.1	8	157	12	114	8	123	34	164	13	312	76	141	3
Solyc01g091650.2.1	7	55	2	23	0	101	34	99	7	74	13	69	0
Solyc01g091670.2.1	0	0	0	0	0	0	0	0	0	0	0	0	0
Solyc01g091690.2.1	3	14	0	8	0	61	2	43	3	289	16	271	0
Solyc01g091700.2.1	0	0	0	0	0	0	0	0	0	0	0	0	0
Solyc01g091710.2.1	4	3	3	5	0	11	18	33	6	48	9	23	4
Solyc01g091720.2.1	0	0	0	0	0	0	0	0	0	0	0	0	0
Solyc01g091730.2.1	4	327	44	176	4	393	269	364	17	298	58	133	4
Solyc01g091740.2.1	10	87	10	43	12	36	13	46	3	110	53	125	1
Solyc01g091750.2.1	5	30	4	22	3	54	34	71	12	27	11	28	0
Solyc01g091770.2.1	5	42	4	21	0	28	13	41	0	9	0	10	0
Solyc01g091780.2.1	3	36	2	2	1	20	14	19	1	39	5	14	0
Solyc01g091790.2.1	0	0	0	0	0	0	0	0	0	0	0	0	0
Solyc01g091800.2.1	1	5	0	1	0	10	10	8	0	5	0	6	0
Solyc01g091820.2.1	6	9	0	0	2	15	21	17	3	30	7	24	0
Solyc01g091830.2.1	0	0	0	0	0	0	0	0	0	0	0	0	0
Solyc01g091840.2.1	1	11	0	3	0	3	3	1	0	5	6	4	0
Solyc01g091850.2.1	0	0	0	0	0	0	0	0	0	0	0	0	0
Solyc01g091860.2.1	5	30	8	6	3	28	13	38	3	46	9	33	5
Solyc01g091870.2.1	0	0	0	0	0	0	0	0	0	0	0	0	0
Solyc01g091880.2.1	3	29	2	9	5	28	25	56	10	22	15	15	2

Solyc01g091890.2.1	1	7	1	6	0	1	3	4	0	14	7	17	2
Solyc01g091900.2.1	2	4	1	4	0	16	5	14	1	5	1	9	0
Solyc01g091910.2.1	5	6	1	8	3	121	10	50	9	8	18	16	1
Solyc01g091920.2.1	0	0	0	0	0	0	0	0	0	0	0	0	0
Solyc01g091930.2.1	0	0	0	0	0	0	0	0	0	0	0	0	0
Solyc01g091940.2.1	0	0	0	0	0	0	0	0	0	0	0	0	0
Solyc01g091950.2.1	16	161	57	124	28	305	370	319	46	359	93	247	3
Solyc01g092950.2.1	1	50	1	19	1	63	14	57	0	0	0	0	0
Solyc01g093960.2.1	0	0	0	0	0	0	0	0	0	0	0	0	0
Solyc01g093970.2.1	4	45	3	17	0	37	41	33	4	56	25	46	1
Solyc01g093980.2.1	0	0	0	0	0	0	0	0	0	0	0	0	0
Solyc01g093990.2.1	2	11	1	5	0	7	8	3	0	24	3	31	0
Solyc01g094000.2.1	13	116	14	123	7	179	102	251	19	300	57	276	8
Solyc01g094010.2.1	10	273	8	41	33	823	189	337	85	217	6	39	4
Solyc01g094020.2.1	5	21	8	21	2	11	16	11	0	22	5	13	0
Solyc01g094030.2.1	5	154	3	59	16	145	117	138	20	283	63	82	11
Solyc01g094040.2.1	5	93	11	14	0	434	58	172	23	101	26	82	0
Solyc01g094050.1.1	6	10	0	13	0	50	45	46	6	44	15	39	6
Solyc01g094060.2.1	16	29	7	30	3	100	104	125	22	105	45	58	11
Solyc01g094080.2.1	1	10	0	2	0	14	6	26	0	30	0	18	0
Solyc01g094090.2.1	0	0	0	0	0	0	0	0	0	0	0	0	0
Solyc01g094110.2.1	0	0	0	0	0	0	0	0	0	0	0	0	0
Solyc01g094130.2.1	0	0	0	0	0	0	0	0	0	0	0	0	0
Solyc01g094140.2.1	0	0	0	0	0	0	0	0	0	0	0	0	0
Solyc01g094150.2.1	10	20	4	24	4	79	40	57	10	25	26	28	4
Solyc01g094170.2.1	2	4	2	6	1	0	40	11	9	37	60	41	3
Solyc01g094180.2.1	0	0	0	0	0	0	0	0	0	0	0	0	0
Solyc01g094190.2.1	1	3	1	2	0	0	5	1	0	6	0	2	0
Solyc01g094200.2.1	10	75	6	26	4	187	54	202	16	195	26	167	2
Solyc01g094210.2.1	2	29	1	22	0	0	0	18	1	2	5	12	0
Solyc01g094220.2.1	4	17	4	10	2	43	50	65	6	52	7	38	0
Solyc01g094230.2.1	0	0	0	0	0	0	0	0	0	0	0	0	0
Solyc01g094240.2.1	0	0	0	0	0	0	0	0	0	0	0	0	0
Solyc01g094250.2.1	0	0	0	0	0	0	0	0	0	0	0	0	0
Solyc01g094280.2.1	7	44	4	16	2	55	23	97	0	78	12	38	1
Solyc01g094290.2.1	10	78	18	39	0	61	71	157	10	86	12	101	0
Solyc01g094330.2.1	9	15	4	9	2	61	200	62	8	26	19	15	0
Solyc01g094340.2.1	20	109	32	109	3	87	471	159	7	148	59	145	2
Solyc01g094350.2.1	6	33	5	41	1	101	61	142	13	45	18	57	0
Solyc01g094360.2.1	0	0	0	0	0	0	0	0	0	0	0	0	0
Solyc01g094370.2.1	12	356	26	189	59	1203	760	1484	214	599	73	360	18

Solyc01g094410.2.1	25	571	66	807	7	338	309	659	36	618	99	1103	8
Solyc01g094420.2.1	11	83	7	96	8	184	87	250	22	146	16	97	0
Solyc01g094460.2.1	2	33	1	30	0	3	11	20	0	36	0	15	0
Solyc01g094470.2.1	1	11	1	16	6	13	3	16	2	29	8	10	0
Solyc01g094480.2.1	8	167	10	121	8	190	62	308	8	308	44	308	5
Solyc01g094490.2.1	0	0	0	0	0	0	0	0	0	0	0	0	0
Solyc01g094500.2.1	2	3	0	13	0	13	5	29	0	9	2	22	0
Solyc01g094510.1.1	0	0	0	0	0	0	0	0	0	0	0	0	0
Solyc01g094520.2.1	2	9	0	6	0	7	10	12	0	25	7	5	0
Solyc01g094530.2.1	2	4	0	2	2	6	14	9	3	4	0	15	0
Solyc01g094540.2.1	2	5	0	0	0	10	2	21	0	20	6	22	0
Solyc01g094550.2.1	3	93	6	73	5	514	54	168	7	124	8	120	0
Solyc01g094560.2.1	0	0	0	0	0	0	0	0	0	0	0	0	0
Solyc01g094570.2.1	4	20	2	38	6	30	42	60	1	47	6	45	0
Solyc01g094580.2.1	14	54	9	60	2	110	54	145	9	108	25	49	0
Solyc01g094590.2.1	0	0	0	0	0	0	0	0	0	0	0	0	0
Solyc01g094600.2.1	0	0	0	0	0	0	0	0	0	0	0	0	0
Solyc01g094610.2.1	0	0	0	0	0	0	0	0	0	0	0	0	0
Solyc01g094620.2.1	13	49	10	36	4	206	111	265	14	63	47	73	5
Solyc01g094630.2.1	7	20	8	15	0	55	21	43	6	40	12	5	1
Solyc01g094640.2.1	14	91	17	89	19	256	227	256	66	393	83	280	0
Solyc01g094650.2.1	0	0	0	0	0	0	0	0	0	0	0	0	0
Solyc01g094660.2.1	14	47	2	22	2	116	47	125	21	83	25	61	1
Solyc01g094670.2.1	10	18	2	10	0	25	28	52	6	56	20	37	0
Solyc01g094680.2.1	5	23	3	17	4	86	66	87	10	42	30	36	0
Solyc01g094690.2.1	6	192	1	133	0	21	12	45	0	136	39	176	4
Solyc01g094700.2.1	18	223	11	6	39	264	199	74	369	366	98	22	55
Solyc01g094720.2.1	14	169	14	123	1	82	64	125	20	154	78	183	2
Solyc01g094750.2.1	1	8	3	0	1	0	18	0	6	5	5	0	3
Solyc01g094760.2.1	0	0	0	0	0	0	0	0	0	0	0	0	0
Solyc01g094770.2.1	7	108	23	100	10	446	284	498	34	201	136	163	9
Solyc01g094780.2.1	0	0	0	0	0	0	0	0	0	0	0	0	0
Solyc01g094790.2.1	4	254	1287	111	246	1881	35125	1895	315	226	4057	175	49
Solyc01g094800.2.1	31	161	17	176	15	319	226	724	75	197	84	181	4
Solyc01g094810.2.1	5	31	10	29	0	134	32	130	16	140	28	86	2
Solyc01g094820.2.1	0	0	0	0	0	0	0	0	0	0	0	0	0
Solyc01g094830.2.1	2	13	2	12	0	4	3	8	0	22	2	11	0
Solyc01g094840.2.1	6	30	4	61	0	192	46	160	3	24	1	16	0
Solyc01g094850.1.1	0	0	0	0	0	0	0	0	0	0	0	0	0
Solyc01g094860.2.1	7	13	1	7	2	101	28	35	14	20	15	8	2
Solyc01g094870.1.1	0	0	0	0	0	0	0	0	0	0	0	0	0

Solyc01g094880.2.1	7	41	10	37	6	38	58	64	17	186	23	136	2
Solyc01g094890.2.1	22	449	74	358	0	344	251	624	0	38	22	58	0
Solyc01g094910.2.1	12	73	20	87	0	468	62	486	0	4	0	2	0
Solyc01g094920.2.1	10	291	12	150	14	132	61	223	34	147	43	217	6
Solyc01g094930.2.1	11	133	16	54	6	293	203	382	37	209	40	151	5
Solyc01g094940.2.1	6	111	5	32	3	68	36	69	6	121	7	49	0
Solyc01g094950.2.1	17	1943	599	1322	306	1985	3368	2095	570	3810	729	3222	150
Solyc01g094960.2.1	3	58	3	7	3	49	17	34	3	98	1	41	0
Solyc01g094970.2.1	0	0	0	0	0	0	0	0	0	0	0	0	0
Solyc01g094990.2.1	1	0	0	0	0	10	5	1	4	0	0	0	0
Solyc01g095000.2.1	3	114	9	49	0	50	25	67	5	30	1	19	0
Solyc01g095010.2.1	1	2	0	0	5	2	10	2	1	7	13	9	0
Solyc01g095020.2.1	16	81	27	117	15	228	266	301	32	160	77	127	5
Solyc01g095030.2.1	6	97	5	62	0	112	9	85	5	108	15	91	1
Solyc01g095040.2.1	8	44	2	16	5	60	42	82	11	155	23	94	0
Solyc01g095050.2.1	3	122	14	49	7	203	119	173	11	282	57	274	11
Solyc01g095070.2.1	4	166	13	85	4	49	80	54	4	275	39	194	13
Solyc01g095080.2.1	2	1	0	0	5	30	4	11	0	2	147	16	0
Solyc01g095090.2.1	0	0	0	0	0	0	0	0	0	0	0	0	0
Solyc01g095100.2.1	0	0	0	0	0	0	0	0	0	0	0	0	0
Solyc01g095110.2.1	3	7	1	0	0	0	10	12	0	33	7	15	9
Solyc01g095120.2.1	5	27	6	6	4	37	37	60	2	55	4	78	5
Solyc01g095130.2.1	0	0	0	0	0	0	0	0	0	0	0	0	0
Solyc01g095140.2.1	0	0	0	0	0	0	0	0	0	0	0	0	0
Solyc01g095150.2.1	4	1293	53	804	41	6328	408	1102	173	603	53	769	55
Solyc01g095160.2.1	0	0	0	0	0	0	0	0	0	0	0	0	0
Solyc01g095170.2.1	3	5	1	0	2	20	32	10	62	16	8	10	4
Solyc01g095180.2.1	2	6	0	0	1	10	11	2	10	14	2	0	0
Solyc01g095190.1.1	0	0	0	0	0	0	0	0	0	0	0	0	0
Solyc01g095200.2.1	17	573	62	394	33	2504	965	1853	330	1196	170	1033	16
Solyc01g095210.2.1	10	39	2	30	2	65	30	66	7	81	11	66	0
Solyc01g095230.2.1	0	0	0	0	0	0	0	0	0	0	0	0	0
Solyc01g095240.2.1	0	0	0	0	0	0	0	0	0	0	0	0	0
Solyc01g095270.2.1	7	88	9	88	7	81	75	161	4	155	9	97	1
Solyc01g095280.2.1	2	11	0	7	4	4	19	7	14	6	22	3	1
Solyc01g095290.2.1	0	0	0	0	0	0	0	0	0	0	0	0	0
Solyc01g095300.2.1	9	96	8	46	12	70	81	90	7	82	4	50	3
Solyc01g095310.1.1	1	0	0	0	0	5	6	0	1	0	2	6	0
Solyc01g095320.2.1	74	348	79	307	9	470	394	422	19	8613	2448	8368	9
Solyc01g095330.2.1	9	38	2	35	0	64	13	28	3	724	152	651	0
Solyc01g095340.2.1	2	1	0	0	0	0	2	1	0	14	6	18	0

Solyc01g095350.1.1	0	0	0	0	0	0	0	0	0	0	0	0	0
Solyc01g095360.2.1	0	0	0	0	0	0	0	0	0	0	0	0	0
Solyc01g095370.2.1	10	48	11	39	11	123	113	117	11	176	33	131	7
Solyc01g095390.2.1	3	3	3	10	2	6	12	5	0	11	3	6	0
Solyc01g095400.2.1	18	110	11	122	4	580	245	603	28	551	56	519	5
Solyc01g095410.2.1	6	260	58	154	31	905	710	770	71	322	315	264	33
Solyc01g095420.2.1	4	12	0	10	0	31	14	43	2	27	8	27	0
Solyc01g095430.2.1	6	1363	102	896	18	214	178	183	4	191	47	227	5
Solyc01g095450.2.1	0	0	0	0	0	0	0	0	0	0	0	0	0
Solyc01g095460.2.1	11	59	13	38	2	342	243	371	35	45	25	52	0
Solyc01g095470.2.1	6	55	4	19	1	1264	534	890	3	42	68	60	0
Solyc01g095480.2.1	5	13	1	8	4	42	8	41	1	107	14	128	1
Solyc01g095490.2.1	4	68	9	13	7	113	103	121	18	55	23	26	0
Solyc01g095500.2.1	0	0	0	0	0	0	0	0	0	0	0	0	0
Solyc01g095510.2.1	4	20	4	6	0	43	30	24	4	25	7	33	2
Solyc01g095520.2.1	0	0	0	0	0	0	0	0	0	0	0	0	0
Solyc01g095530.2.1	7	2587	33	1419	25	42	5	38	0	925	106	2455	0
Solyc01g095540.2.1	4	20	0	1	0	14	11	24	1	19	5	20	1
Solyc01g095550.2.1	0	0	0	0	0	0	0	0	0	0	0	0	0
Solyc01g095560.2.1	0	0	0	0	0	0	0	0	0	0	0	0	0
Solyc01g095570.2.1	4	55	4	23	2	17	11	12	0	25	25	52	1
Solyc01g095580.2.1	14	294	44	243	22	74	177	188	13	218	33	188	8
Solyc01g095590.2.1	3	22	2	17	1	12	0	10	1	23	14	28	0
Solyc01g095600.2.1	0	0	0	0	0	0	0	0	0	0	0	0	0
Solyc01g095610.2.1	7	48	7	20	6	18	61	36	1	42	0	11	1
Solyc01g095620.2.1	24	1386	234	1120	39	1079	1551	448	16	218	46	161	4
Solyc01g095630.2.1	11	981	104	1152	39	279	124	135	2	48	4	421	11
Solyc01g095650.2.1	0	0	0	0	0	0	0	0	0	0	0	0	0
Solyc01g095670.2.1	7	173	14	94	1	48	58	102	3	100	44	79	0
Solyc01g095680.2.1	9	18	2	4	0	107	80	113	5	11	4	30	0
Solyc01g095690.2.1	1	1	0	5	0	12	5	1	0	1	0	0	0
Solyc01g095700.2.1	5	103	18	57	7	59	194	63	31	115	8	82	3
Solyc01g095710.2.1	4	21	4	18	0	47	43	74	11	15	7	9	0
Solyc01g095720.2.1	6	7	3	0	11	115	18	62	26	19	25	3	3
Solyc01g095730.2.1	7	20	1	25	1	71	19	58	2	52	12	25	2
Solyc01g095740.2.1	20	86	19	89	19	219	188	451	33	166	52	104	7
Solyc01g095750.2.1	1	1	0	0	0	14	5	0	3	6	4	0	2
Solyc01g095760.2.1	0	0	0	0	0	0	0	0	0	0	0	0	0
Solyc01g095770.2.1	0	0	0	0	0	0	0	0	0	0	0	0	0
Solyc01g095790.2.1	23	186	21	164	13	336	296	383	32	453	79	235	6
Solyc01g095800.2.1	4	27	1	5	0	31	22	37	0	17	2	3	0

Solyc01g095810.2.1	0	0	0	0	0	0	0	0	0	0	0	0	0
Solyc01g095890.2.1	8	30	3	17	1	64	34	54	8	24	2	29	0
Solyc01g095900.2.1	34	236	67	225	0	435	504	1277	2	106	55	107	2
Solyc01g095920.2.1	0	0	0	0	0	0	0	0	0	0	0	0	0
Solyc01g095930.2.1	10	1	0	0	1	130	291	34	209	51	21	11	0
Solyc01g095940.2.1	5	0	1	0	3	92	356	3	235	26	11	5	0
Solyc01g095960.2.1	0	0	0	0	0	0	0	0	0	0	0	0	0
Solyc01g095970.2.1	2	12	0	6	0	54	11	16	2	44	0	19	0
Solyc01g095980.2.1	3	28	8	22	0	25	26	7	0	32	5	22	2
Solyc01g095990.2.1	9	288	26	131	9	305	87	290	22	115	22	101	4
Solyc01g096000.2.1	2	9	0	8	1	4	12	7	0	149	47	52	1
Solyc01g096010.2.1	11	35	3	44	4	115	53	169	1	34	13	79	1
Solyc01g096020.2.1	3	40	5	51	4	43	56	87	15	123	3	56	0
Solyc01g096030.2.1	3	8	1	8	0	3	20	9	8	7	1	7	0
Solyc01g096040.2.1	24	298	13	163	42	477	234	285	64	3188	286	2004	23
Solyc01g096050.2.1	13	49	6	71	6	209	74	76	5	121	36	78	0
Solyc01g096060.2.1	10	31	4	45	22	93	55	122	10	153	38	129	2
Solyc01g096070.2.1	3	40	0	36	5	61	7	72	2	1	10	0	0
Solyc01g096080.2.1	4	18	5	33	0	16	45	35	4	50	2	30	0
Solyc01g096090.2.1	0	0	0	0	0	0	0	0	0	0	0	0	0
Solyc01g096100.2.1	16	198	14	136	28	186	141	351	38	1059	68	876	6
Solyc01g096110.2.1	22	66	2	64	5	250	154	191	21	209	30	122	4
Solyc01g096120.2.1	0	0	0	0	0	0	0	0	0	0	0	0	0
Solyc01g096130.2.1	3	25	0	16	2	131	31	106	10	28	13	26	3
Solyc01g096140.2.1	0	0	0	0	0	0	0	0	0	0	0	0	0
Solyc01g096150.2.1	4	21	2	12	0	71	21	41	0	92	7	31	0
Solyc01g096160.2.1	12	37	5	58	3	151	52	130	5	108	17	74	0
Solyc01g096170.2.1	0	0	0	0	0	0	0	0	0	0	0	0	0
Solyc01g096180.2.1	6	66	10	27	4	65	123	57	19	42	22	45	0
Solyc01g096190.2.1	3	58	4	17	1	13	7	26	0	5	0	3	4
Solyc01g096200.2.1	6	44	5	26	0	96	25	45	3	22	1	30	8
Solyc01g096210.2.1	4	8	4	8	0	2	9	10	0	17	8	18	1
Solyc01g096220.2.1	8	106	3	23	6	3	0	8	0	58	65	73	2
Solyc01g096230.2.1	10	89	2	59	8	126	75	53	23	70	88	98	10
Solyc01g096240.2.1	7	477	31	167	3	66	121	100	0	397	33	453	4
Solyc01g096250.2.1	1	4	2	7	0	5	5	0	0	16	0	9	0
Solyc01g096260.2.1	0	0	0	0	0	0	0	0	0	0	0	0	0
Solyc01g096270.2.1	0	0	0	0	0	0	0	0	0	0	0	0	0
Solyc01g096280.1.1	0	0	0	0	0	0	0	0	0	0	0	0	0
Solyc01g096290.2.1	3	183	37	83	23	500	117	384	58	405	102	429	6
Solyc01g096300.2.1	0	0	0	0	0	0	0	0	0	0	0	0	0

Solyc01g096320.2.1	8	13	4	18	1	1348	1521	1067	169	46	30	16	0
Solyc01g096330.2.1	0	0	0	0	0	0	0	0	0	0	0	0	0
Solyc01g096340.2.1	5	170	20	101	4	322	232	365	30	48	12	36	1
Solyc01g096350.2.1	6	63	5	47	6	78	30	82	9	105	14	68	0
Solyc01g096360.2.1	6	156	13	81	6	6	11	10	0	38	4	26	7
Solyc01g096370.2.1	4	48	6	25	0	2	15	7	2	0	0	1	0
Solyc01g096380.1.1	0	0	0	0	0	0	0	0	0	0	0	0	0
Solyc01g096390.2.1	6	15	0	15	1	11	32	24	1	69	17	55	1
Solyc01g096400.2.1	5	16	10	17	1	10	134	7	6	7	19	5	1
Solyc01g096410.2.1	0	0	0	0	0	0	0	0	0	0	0	0	0
Solyc01g096420.2.1	1	11	0	2	0	0	1	14	0	8	0	4	0
Solyc01g096430.2.1	3	8	1	0	2	4	0	9	0	44	8	8	0
Solyc01g096440.2.1	0	0	0	0	0	0	0	0	0	0	0	0	0
Solyc01g096450.2.1	8	212	5	116	0	27	47	85	14	182	6	79	0
Solyc01g096460.2.1	5	17	2	8	0	45	26	58	15	35	10	26	0
Solyc01g096470.2.1	7	110	8	70	6	55	63	113	5	115	17	85	0
Solyc01g096480.2.1	3	6	0	1	0	10	30	10	0	9	4	21	0
Solyc01g096490.2.1	23	137	14	59	9	240	140	289	28	238	68	165	2
Solyc01g096500.2.1	13	122	16	80	6	163	166	217	16	164	28	167	4
Solyc01g096510.2.1	8	59	91	127	30	33	19	17	1	9	0	2	4
Solyc01g096520.2.1	1	56	3	28	0	396	25	166	6	72	22	90	3
Solyc01g096530.2.1	1	7	0	3	0	11	2	5	0	27	2	25	0
Solyc01g096540.2.1	14	58	6	38	1	152	82	152	20	188	30	116	5
Solyc01g096550.2.1	1	3	0	2	0	2	1	10	0	2	3	19	0
Solyc01g096560.1.1	0	0	0	0	0	0	0	0	0	0	0	0	0
Solyc01g096570.2.1	11	154	18	96	14	254	210	302	27	753	103	523	5
Solyc01g096580.2.1	0	0	0	0	0	0	0	0	0	0	0	0	0
Solyc01g096590.2.1	0	0	0	0	0	0	0	0	0	0	0	0	0
Solyc01g096600.2.1	8	59	5	53	0	96	72	91	4	179	35	96	0
Solyc01g096610.2.1	4	32	1	6	0	29	27	38	4	17	1	9	1
Solyc01g096620.2.1	2	2	0	10	2	6	0	2	1	13	5	3	0
Solyc01g096630.2.1	1	4	3	1	0	4	3	3	0	1	4	0	0
Solyc01g096640.2.1	0	0	0	0	0	0	0	0	0	0	0	0	0
Solyc01g096650.2.1	0	0	0	0	0	0	0	0	0	0	0	0	0
Solyc01g096660.2.1	8	862	64	388	2	299	123	209	2	197	47	159	0
Solyc01g096670.2.1	10	71	8	76	5	80	39	154	44	31	27	32	1
Solyc01g096680.2.1	0	0	0	0	0	0	0	0	0	0	0	0	0
Solyc01g096700.2.1	16	208	41	283	25	486	264	569	44	1050	221	660	27
Solyc01g096710.2.1	5	84	9	34	4	170	43	193	7	141	18	94	0
Solyc01g096730.2.1	5	3	2	0	3	105	16	39	4	10	4	1	2
Solyc01g096750.1.1	0	0	0	0	0	0	0	0	0	0	0	0	0

Solyc01g096760.2.1	1	4	0	0	0	5	2	22	4	8	4	3	0
Solyc01g096770.2.1	1	18	0	2	0	31	10	24	4	19	3	16	0
Solyc01g096780.2.1	9	89	4	64	8	257	67	211	20	133	43	132	1
Solyc01g096790.2.1	0	0	0	0	0	0	0	0	0	0	0	0	0
Solyc01g096800.2.1	2	6	0	3	0	32	7	30	4	23	4	13	0
Solyc01g096810.2.1	20	459	35	530	13	730	193	966	31	605	126	428	3
Solyc01g096820.2.1	0	0	0	0	0	0	0	0	0	0	0	0	0
Solyc01g096830.2.1	12	120	1	75	2	254	112	257	10	235	57	153	1
Solyc01g096840.2.1	0	0	0	0	0	0	0	0	0	0	0	0	0
Solyc01g096850.2.1	2	3	3	2	0	5	13	23	0	40	5	23	3
Solyc01g096860.1.1	0	0	0	0	0	0	0	0	0	0	0	0	0
Solyc01g096870.2.1	11	168	17	154	1	215	168	332	12	175	31	229	3
Solyc01g096880.2.1	8	186	20	134	0	107	70	42	0	27	12	68	0
Solyc01g096890.2.1	5	17	2	4	0	35	23	10	1	12	7	5	0
Solyc01g096900.2.1	16	44	6	6	0	50	70	87	7	41	29	75	2
Solyc01g096910.2.1	2	3	1	9	0	21	18	14	1	8	10	4	0
Solyc01g096920.2.1	6	17	2	9	5	45	29	59	6	56	14	56	3
Solyc01g096930.2.1	1	0	1	0	0	5	3	7	0	0	1	3	0
Solyc01g096940.2.1	0	0	0	0	0	0	0	0	0	0	0	0	0
Solyc01g096950.2.1	1	0	0	2	0	6	0	1	0	30	2	18	3
Solyc01g096960.2.1	0	0	0	0	0	0	0	0	0	0	0	0	0
Solyc01g096970.2.1	0	0	0	0	0	0	0	0	0	0	0	0	0
Solyc01g096980.1.1	0	0	0	0	0	0	0	0	0	0	0	0	0
Solyc01g096990.2.1	14	46	5	45	0	153	89	206	14	99	33	97	0
Solyc01g097000.2.1	2	12	2	8	2	1	9	1	4	89	0	101	1
Solyc01g097010.2.1	5	73	3	53	1	126	51	251	21	62	19	58	2
Solyc01g097020.2.1	2	38	1	26	0	114	28	85	8	94	10	84	3
Solyc01g097030.2.1	7	21	6	39	11	77	120	117	23	53	35	33	4
Solyc01g097040.2.1	11	44	4	70	1	38	46	93	26	69	6	68	0
Solyc01g097070.2.1	0	0	0	0	0	0	0	0	0	0	0	0	0
Solyc01g097080.1.1	0	0	0	0	0	0	0	0	0	0	0	0	0
Solyc01g097110.1.1	0	0	0	0	0	0	0	0	0	0	0	0	0
Solyc01g097120.2.1	6	52	3	26	2	57	23	69	17	108	15	62	2
Solyc01g097130.2.1	2	4	0	7	0	10	14	12	1	26	9	4	0
Solyc01g097140.2.1	6	216	9	103	9	279	116	205	15	210	33	127	3
Solyc01g097160.2.1	10	41	12	60	1	25	107	72	5	45	10	65	0
Solyc01g097170.2.1	2	95	15	32	1	60	14	48	5	277	14	161	1
Solyc01g097190.2.1	7	40	9	19	3	87	33	109	6	104	18	113	1
Solyc01g097220.1.1	0	0	0	0	0	0	0	0	0	0	0	0	0
Solyc01g097230.2.1	0	0	0	0	0	0	0	0	0	0	0	0	0
Solyc01g097240.2.1	10	417	9	104	2	1654	129	996	114	128	45	36	1

Solyc01g097250.2.1	0	0	0	0	0	0	0	0	0	0	0	0	0
Solyc01g097260.2.1	0	0	0	0	0	0	0	0	0	0	0	0	0
Solyc01g097270.2.1	8	890	8	213	0	224	119	521	21	17	6	14	1
Solyc01g097280.2.1	0	0	0	0	0	0	0	0	0	0	0	0	0
Solyc01g097290.2.1	5	192	15	64	1	63	37	38	1	131	27	115	0
Solyc01g097300.2.1	2	21	2	7	0	60	4	36	1	56	8	33	0
Solyc01g097310.2.1	6	239	8	102	7	271	56	179	4	117	21	88	1
Solyc01g097320.2.1	11	14	2	18	7	82	74	104	6	24	4	26	0
Solyc01g097330.2.1	8	12	11	11	7	10	97	27	14	48	10	27	1
Solyc01g097340.2.1	13	629	84	249	10	35	174	118	8	720	210	790	7
Solyc01g097350.2.1	6	90	8	53	9	103	40	94	12	52	17	115	20
Solyc01g097360.2.1	3	91	10	41	0	176	47	110	11	77	11	32	0
Solyc01g097380.1.1	0	0	0	0	0	0	0	0	0	0	0	0	0
Solyc01g097390.2.1	0	0	0	0	0	0	0	0	0	0	0	0	0
Solyc01g097400.1.1	0	0	0	0	0	0	0	0	0	0	0	0	0
Solyc01g097420.1.1	0	0	0	0	0	0	0	0	0	0	0	0	0
Solyc01g097430.2.1	0	0	0	0	0	0	0	0	0	0	0	0	0
Solyc01g097440.2.1	15	94	10	95	3	230	368	188	33	192	48	149	2
Solyc01g097450.2.1	1	36	1	10	0	28	10	50	1	21	4	32	0
Solyc01g097460.2.1	5	308	17	192	0	123	52	107	1	82	22	48	1
Solyc01g097470.2.1	0	0	0	0	0	0	0	0	0	0	0	0	0
Solyc01g097480.1.1	0	0	0	0	0	0	0	0	0	0	0	0	0
Solyc01g097500.2.1	4	27	3	3	1	18	7	47	0	22	1	37	0
Solyc01g097510.2.1	6	68	5	59	9	67	151	71	13	154	16	89	6
Solyc01g097520.2.1	7	122	62	89	66	134	362	127	58	108	57	151	8
Solyc01g097530.1.1	0	0	0	0	0	0	0	0	0	0	0	0	0
Solyc01g097540.2.1	0	0	0	0	0	0	0	0	0	0	0	0	0
Solyc01g097570.2.1	0	0	0	0	0	0	0	0	0	0	0	0	0
Solyc01g097590.2.1	1	5	3	5	1	5	2	16	2	14	0	2	0
Solyc01g097600.1.1	2	3	0	0	3	14	6	5	0	3	2	8	0
Solyc01g097610.2.1	5	19	1	5	0	61	41	34	5	29	4	23	0
Solyc01g097630.1.1	0	0	0	0	0	0	0	0	0	0	0	0	0
Solyc01g097640.2.1	2	3	1	6	0	16	9	23	4	16	4	10	0
Solyc01g097650.2.1	1	4	0	2	0	4	2	16	0	1	2	1	0
Solyc01g097670.2.1	0	0	0	0	0	0	0	0	0	0	0	0	0
Solyc01g097700.1.1	0	0	0	0	0	0	0	0	0	0	0	0	0
Solyc01g097710.1.1	0	0	0	0	0	0	0	0	0	0	0	0	0
Solyc01g097730.2.1	1	0	2	2	0	3	2	9	2	3	0	0	0
Solyc01g097740.2.1	8	121	8	54	3	142	60	223	25	144	41	78	2
Solyc01g097760.2.1	0	0	0	0	0	0	0	0	0	0	0	0	0
Solyc01g097770.2.1	17	739	88	515	18	207	107	176	8	383	87	499	11

Solyc01g097790.2.1	3	23	1	5	2	4	6	16	0	12	19	10	0
Solyc01g097800.2.1	14	55	0	22	1	54	90	82	3	120	21	72	1
Solyc01g097810.2.1	14	378	49	229	7	344	480	514	34	191	74	141	2
Solyc01g097820.2.1	0	0	0	0	0	0	0	0	0	0	0	0	0
Solyc01g097830.2.1	1	7	0	8	0	0	0	0	0	1	0	0	0
Solyc01g097840.2.1	3	11	1	4	0	7	13	32	9	14	15	6	4
Solyc01g097860.2.1	5	330	56	207	0	55	94	55	7	48	33	62	0
Solyc01g097870.2.1	4	111	6	71	7	161	88	176	5	431	109	263	15
Solyc01g097880.2.1	3	53	0	26	0	313	94	181	10	45	34	69	0
Solyc01g097890.2.1	5	45	5	19	9	132	42	96	14	177	18	75	0
Solyc01g097910.2.1	6	187	49	157	2	63	250	133	2	108	6	58	7
Solyc01g097920.2.1	2	12	4	14	0	21	14	12	4	0	1	0	0
Solyc01g097930.2.1	0	0	0	0	0	0	0	0	0	0	0	0	0
Solyc01g097950.2.1	0	0	0	0	0	0	0	0	0	0	0	0	0
Solyc01g097970.2.1	1	6	0	2	0	1	5	7	0	0	1	6	0
Solyc01g097980.2.1	12	63	14	46	0	320	99	338	24	120	23	69	1
Solyc01g097990.2.1	0	0	0	0	0	0	0	0	0	0	0	0	0
Solyc01g098000.2.1	6	74	6	32	0	183	28	143	7	182	53	209	20
Solyc01g098010.2.1	0	0	0	0	0	0	0	0	0	0	0	0	0
Solyc01g098020.2.1	4	54	3	24	0	64	10	134	1	17	3	8	0
Solyc01g098030.2.1	11	96	6	58	5	147	103	250	20	170	42	108	0
Solyc01g098040.2.1	1	4	1	0	0	0	2	6	2	4	2	4	0
Solyc01g098080.2.1	3	5	2	8	0	12	49	23	7	6	13	11	0
Solyc01g098090.2.1	14	168	22	107	7	271	171	377	27	246	38	163	2
Solyc01g098100.2.1	6	121	1	32	6	191	60	173	15	128	35	186	0
Solyc01g098110.2.1	5	16	1	13	3	26	99	34	4	100	26	78	0
Solyc01g098120.2.1	10	50	9	74	0	63	85	351	0	9	2	6	0
Solyc01g098130.2.1	1	8	1	8	0	15	16	57	0	1	1	2	0
Solyc01g098140.2.1	0	0	0	0	0	0	0	0	0	0	0	0	0
Solyc01g098150.2.1	5	12	4	9	0	34	42	73	2	13	5	22	0
Solyc01g098160.2.1	8	148	12	101	5	256	114	286	49	139	41	111	2
Solyc01g098170.2.1	10	15	2	5	0	36	34	62	1	60	24	39	1
Solyc01g098180.2.1	9	24	5	23	2	90	25	147	4	84	18	68	1
Solyc01g098190.2.1	7	82	7	42	27	209	90	323	26	500	42	298	0
Solyc01g098200.2.1	5	13	5	3	6	18	29	14	5	66	13	43	1
Solyc01g098210.2.1	3	13	3	12	0	18	15	9	3	16	8	31	0
Solyc01g098230.2.1	2	7	4	4	1	21	24	28	7	33	5	22	0
Solyc01g098240.1.1	0	0	0	0	0	0	0	0	0	0	0	0	0
Solyc01g098250.2.1	9	34	12	34	5	71	84	98	18	86	40	69	1
Solyc01g098320.2.1	0	0	0	0	0	0	0	0	0	0	0	0	0
Solyc01g098340.2.1	11	25	4	44	5	137	77	235	6	72	15	90	3

Solyc01g098350.2.1	8	104	3	50	0	161	38	152	7	114	12	76	0
Solyc01g098370.1.1	0	0	0	0	0	0	0	0	0	0	0	0	0
Solyc01g098380.2.1	1	1	0	2	0	1	2	16	0	12	1	28	0
Solyc01g098390.2.1	9	78	3	76	1	106	47	140	4	67	0	33	0
Solyc01g098400.2.1	1	0	0	2	0	15	5	11	2	10	3	4	0
Solyc01g098410.2.1	0	0	0	0	0	0	0	0	0	0	0	0	0
Solyc01g098430.2.1	5	35	4	20	7	98	51	97	7	69	13	32	0
Solyc01g098440.1.1	0	0	0	0	0	0	0	0	0	0	0	0	0
Solyc01g098450.2.1	6	7	2	17	2	13	17	18	1	7	9	17	0
Solyc01g098460.2.1	2	7	2	5	0	25	7	44	2	11	2	16	0
Solyc01g098470.2.1	3	4	0	0	0	12	24	6	2	9	6	13	0
Solyc01g098480.1.1	0	0	0	0	0	0	0	0	0	0	0	0	0
Solyc01g098490.2.1	2	0	2	0	0	58	5	7	13	24	1	22	0
Solyc01g098500.2.1	13	479	10	224	16	26	59	100	23	567	15	1085	12
Solyc01g098510.2.1	0	0	0	0	0	0	0	0	0	0	0	0	0
Solyc01g098520.2.1	0	0	0	0	0	0	0	0	0	0	0	0	0
Solyc01g098530.2.1	0	0	0	0	0	0	0	0	0	0	0	0	0
Solyc01g098540.2.1	0	0	0	0	0	0	0	0	0	0	0	0	0
Solyc01g098550.2.1	5	35	1	24	0	41	31	73	5	62	24	53	1
Solyc01g098560.2.1	12	74	7	52	6	189	117	178	3	73	48	82	0
Solyc01g098570.2.1	1	7	1	3	0	0	0	1	0	0	4	13	0
Solyc01g098580.2.1	2	21	5	16	0	5	4	2	4	1	4	13	0
Solyc01g098590.2.1	14	359	49	270	9	405	65	176	3	277	84	309	8
Solyc01g098600.2.1	0	0	0	0	0	0	0	0	0	0	0	0	0
Solyc01g098610.2.1	11	68	17	51	1	98	65	114	1	141	42	95	0
Solyc01g098620.2.1	0	0	0	0	0	0	0	0	0	0	0	0	0
Solyc01g098630.2.1	1	1	0	0	0	3	1	4	1	3	2	6	0
Solyc01g098640.2.1	11	690	146	565	4	61	354	223	10	147	50	172	2
Solyc01g098650.2.1	5	9	0	8	0	2	25	16	7	69	6	63	0
Solyc01g098670.1.1	0	0	0	0	0	0	0	0	0	0	0	0	0
Solyc01g098690.2.1	0	0	0	0	0	0	0	0	0	0	0	0	0
Solyc01g098700.2.1	6	12	0	10	0	7	43	48	0	46	17	29	0
Solyc01g098710.2.1	2	7	1	7	0	8	1	13	0	9	1	0	0
Solyc01g098720.2.1	0	0	0	0	0	0	0	0	0	0	0	0	0
Solyc01g098730.2.1	0	0	0	0	0	0	0	0	0	0	0	0	0
Solyc01g098740.2.1	1	31	0	11	0	2	21	3	0	2	0	2	0
Solyc01g098750.2.1	3	11	0	5	0	28	31	23	11	21	6	8	0
Solyc01g098760.2.1	11	675	32	302	30	513	194	501	25	1081	65	365	9
Solyc01g098770.1.1	0	0	0	0	0	0	0	0	0	0	0	0	0
Solyc01g098780.2.1	1	9	0	2	0	37	12	31	4	15	6	34	0
Solyc01g098790.1.1	0	0	0	0	0	0	0	0	0	0	0	0	0

Solyc01g098800.2.1	5	43	8	45	5	35	10	16	0	32	5	73	0
Solyc01g098810.2.1	3	30	4	15	1	67	21	38	3	21	6	17	0
Solyc01g098830.2.1	2	11	1	2	0	15	10	15	0	24	2	7	0
Solyc01g098840.2.1	6	122	1	40	1	55	30	84	1	37	3	29	0
Solyc01g098850.2.1	0	0	0	0	0	0	0	0	0	0	0	0	0
Solyc01g098860.2.1	0	0	0	0	0	0	0	0	0	0	0	0	0
Solyc01g098870.2.1	30	128	11	88	2	253	240	279	53	289	148	263	1
Solyc01g098880.2.1	9	83	12	44	3	273	138	438	39	174	66	150	3
Solyc01g098890.1.1	0	0	0	0	0	0	0	0	0	0	0	0	0
Solyc01g098900.2.1	2	8	1	17	0	14	11	15	1	13	7	8	0
Solyc01g098910.2.1	9	93	14	67	8	585	96	171	18	123	72	125	21
Solyc01g098920.2.1	13	89	18	89	13	272	153	337	35	288	67	356	4
Solyc01g098930.2.1	1	9	2	5	0	1	2	2	0	0	0	0	0
Solyc01g098940.2.1	0	0	0	0	0	0	0	0	0	0	0	0	0
Solyc01g098950.1.1	0	0	0	0	0	0	0	0	0	0	0	0	0
Solyc01g098980.2.1	0	0	0	0	0	0	0	0	0	0	0	0	0
Solyc01g098990.2.1	5	173	14	168	1	54	56	99	5	103	12	121	1
Solyc01g099000.1.1	1	2	0	0	0	1	5	2	2	2	1	3	0
Solyc01g099010.2.1	0	0	0	0	0	0	0	0	0	0	0	0	0
Solyc01g099020.2.1	1	42	0	23	0	3	0	16	0	0	6	0	0
Solyc01g099030.2.1	0	0	0	0	0	0	0	0	0	0	0	0	0
Solyc01g099040.2.1	0	0	0	0	0	0	0	0	0	0	0	0	0
Solyc01g099050.2.1	0	0	0	0	0	0	0	0	0	0	0	0	0
Solyc01g099060.1.1	0	0	0	0	0	0	0	0	0	0	0	0	0
Solyc01g099080.2.1	0	0	0	0	0	0	0	0	0	0	0	0	0
Solyc01g099090.2.1	19	139	6	131	7	108	113	303	16	557	21	468	17
Solyc01g099100.2.1	8	70	13	58	1	296	218	411	17	175	23	125	4
Solyc01g099110.2.1	18	86	14	76	2	121	108	131	3	112	24	86	0
Solyc01g099120.2.1	1	1	1	0	0	0	3	8	0	13	0	2	0
Solyc01g099130.2.1	6	78	3	63	10	36	23	86	7	72	13	66	0
Solyc01g099140.2.1	4	14	2	3	0	38	25	40	3	0	8	3	0
Solyc01g099150.2.1	0	0	0	0	0	0	0	0	0	0	0	0	0
Solyc01g099160.2.1	14	9	20	12	46	477	97	290	112	56	134	70	2
Solyc01g099170.2.1	0	0	0	0	0	0	0	0	0	0	0	0	0
Solyc01g099180.2.1	1	12	1	8	0	1	6	3	0	0	0	0	0
Solyc01g099190.2.1	26	1	0	2	0	13	1	22	0	773	1184	1565	12
Solyc01g099200.2.1	1	0	0	0	0	0	0	0	0	4	7	4	0
Solyc01g099210.2.1	0	0	0	0	0	0	0	0	0	0	0	0	0
Solyc01g099220.2.1	8	16	1	17	1	49	34	84	9	52	19	62	3
Solyc01g099230.2.1	4	5	0	3	0	29	4	33	2	17	11	33	0
Solyc01g099240.2.1	7	105	5	74	5	44	58	83	3	93	28	76	0

Solyc01g099250.1.1	0	0	0	0	0	0	0	0	0	0	0	0	0
Solyc01g099260.2.1	5	95	15	60	0	67	33	147	0	85	14	130	1
Solyc01g099270.2.1	0	0	0	0	0	0	0	0	0	0	0	0	0
Solyc01g099280.2.1	3	30	1	16	4	86	16	69	5	69	11	23	0
Solyc01g099290.2.1	2	6	0	4	0	12	6	9	0	9	5	12	0
Solyc01g099300.2.1	7	30	5	8	0	95	24	54	5	69	8	46	9
Solyc01g099310.2.1	4	0	3	1	0	8	26	18	0	28	9	10	0
Solyc01g099330.2.1	0	0	0	0	0	0	0	0	0	0	0	0	0
Solyc01g099340.2.1	5	12	1	1	1	7	27	23	4	64	5	35	0
Solyc01g099350.2.1	10	52	6	28	3	131	61	152	18	91	41	95	0
Solyc01g099360.2.1	15	76	17	53	16	154	168	299	44	109	45	82	2
Solyc01g099370.2.1	11	380	72	576	32	251	94	256	19	144	39	336	8
Solyc01g099380.2.1	2	6	3	0	2	9	12	2	2	3	2	14	5
Solyc01g099390.2.1	0	0	0	0	0	0	0	0	0	0	0	0	0
Solyc01g099400.2.1	9	153	20	105	11	230	196	305	26	259	41	161	9
Solyc01g099410.2.1	3	56	5	8	0	7	25	19	2	93	14	72	0
Solyc01g099420.1.1	0	0	0	0	0	0	0	0	0	0	0	0	0
Solyc01g099450.2.1	0	0	0	0	0	0	0	0	0	0	0	0	0
Solyc01g099460.1.1	0	0	0	0	0	0	0	0	0	0	0	0	0
Solyc01g099470.2.1	0	0	0	0	0	0	0	0	0	0	0	0	0
Solyc01g099490.1.1	0	0	0	0	0	0	0	0	0	0	0	0	0
Solyc01g099500.1.1	0	0	0	0	0	0	0	0	0	0	0	0	0
Solyc01g099520.2.1	0	0	0	0	0	0	0	0	0	0	0	0	0
Solyc01g099530.2.1	0	0	0	0	0	0	0	0	0	0	0	0	0
Solyc01g099560.2.1	0	0	0	0	0	0	0	0	0	0	0	0	0
Solyc01g099580.1.1	0	0	0	0	0	0	0	0	0	0	0	0	0
Solyc01g099590.2.1	7	25	10	20	1	196	111	241	6	179	43	286	6
Solyc01g099600.2.1	11	92	4	55	0	65	31	88	0	247	28	168	2
Solyc01g099620.2.1	0	0	0	0	0	0	0	0	0	0	0	0	0
Solyc01g099630.2.1	14	817	32	364	4	24	4067	46	7	906	3197	717	0
Solyc01g099650.2.1	0	0	0	0	0	0	0	0	0	0	0	0	0
Solyc01g099660.2.1	0	0	0	0	0	0	0	0	0	0	0	0	0
Solyc01g099670.2.1	5	291	17	89	18	425	284	416	51	531	124	634	15
Solyc01g099680.2.1	9	680	71	258	23	989	264	1245	204	902	121	534	15
Solyc01g099690.2.1	10	446	69	323	22	477	444	803	23	529	127	474	6
Solyc01g099700.2.1	8	52	4	41	4	139	92	149	6	116	18	97	3
Solyc01g099710.2.1	15	133	4	87	7	189	134	313	22	530	48	312	10
Solyc01g099720.2.1	0	0	0	0	0	0	0	0	0	0	0	0	0
Solyc01g099730.2.1	1	2	0	6	0	1	5	3	0	26	1	12	0
Solyc01g099740.2.1	2	97	1	50	0	115	21	147	2	126	13	87	0
Solyc01g099750.2.1	9	51	24	56	1	107	684	143	9	200	146	164	1

Solyc01g099760.2.1	6	163	17	126	4	442	159	462	43	355	101	381	11
Solyc01g099770.2.1	5	1770	92	1644	60	1769	737	1952	78	9420	1724	10446	84
Solyc01g099780.2.1	4	42	62	17	6	67	341	86	20	35	215	54	26
Solyc01g099790.2.1	10	155	4	87	10	236	66	261	16	281	28	215	0
Solyc01g099800.2.1	5	22	2	11	0	313	137	94	10	118	18	79	0
Solyc01g099810.2.1	10	94	11	36	1	655	383	514	13	149	46	100	0
Solyc01g099820.2.1	0	0	0	0	0	0	0	0	0	0	0	0	0
Solyc01g099830.2.1	0	0	0	0	0	0	0	0	0	0	0	0	0
Solyc01g099840.2.1	3	89	46	207	50	134	897	363	48	354	49	342	16
Solyc01g099850.2.1	4	28	0	14	3	35	15	44	5	67	6	52	6
Solyc01g099860.2.1	4	16	0	15	0	39	33	43	3	27	11	33	1
Solyc01g099870.1.1	0	0	0	0	0	0	0	0	0	0	0	0	0
Solyc01g099880.2.1	6	7	0	8	7	434	72	292	2	7	11	18	0
Solyc01g099890.2.1	0	0	0	0	0	0	0	0	0	0	0	0	0
Solyc01g099900.2.1	8	847	112	387	52	2391	859	2691	273	4007	558	3049	99
Solyc01g099910.2.1	3	97	5	39	0	97	14	8	9	2	17	0	0
Solyc01g099920.2.1	4	1	0	0	0	2	0	1	0	162	23	136	2
Solyc01g099930.2.1	6	59	4	16	4	73	33	52	3	93	9	36	2
Solyc01g099940.2.1	0	0	0	0	0	0	0	0	0	0	0	0	0
Solyc01g099960.2.1	1	3	0	3	0	3	3	3	1	16	0	7	0
Solyc01g099970.2.1	1	4	0	4	0	6	9	5	0	2	0	1	0
Solyc01g099980.2.1	8	14	4	12	0	207	273	254	19	121	9	83	1
Solyc01g099990.2.1	6	2	0	1	0	40	9	38	15	18	44	0	0
Solyc01g100000.2.1	3	46	0	1	0	11	10	16	2	110	0	76	0
Solyc01g100010.2.1	2	20	8	24	4	2	0	3	0	4	4	10	7
Solyc01g100020.2.1	0	0	0	0	0	0	0	0	0	0	0	0	0
Solyc01g100030.2.1	2	41	2	4	0	5	0	10	0	25	23	36	3
Solyc01g100040.2.1	8	186	19	70	9	628	168	185	2	17	15	65	0
Solyc01g100050.2.1	10	145	16	145	27	346	213	461	50	268	124	254	6
Solyc01g100060.2.1	4	55	6	42	1	81	49	136	19	52	22	39	1
Solyc01g100070.2.1	0	0	0	0	0	0	0	0	0	0	0	0	0
Solyc01g100080.2.1	3	5	0	0	0	14	14	46	1	40	4	34	1
Solyc01g100100.2.1	0	0	0	0	0	0	0	0	0	0	0	0	0
Solyc01g100110.2.1	7	172	17	75	11	92	30	89	13	156	61	190	4
Solyc01g100120.2.1	0	0	0	0	0	0	0	0	0	0	0	0	0
Solyc01g100130.2.1	7	31	6	15	6	53	49	49	30	16	17	14	0
Solyc01g100140.2.1	4	50	4	12	0	33	25	35	4	78	3	45	0
Solyc01g100160.2.1	3	22	6	31	0	19	27	34	2	39	6	25	0
Solyc01g100170.2.1	5	20	2	35	0	86	40	81	22	54	14	46	2
Solyc01g100180.2.1	0	0	0	0	0	0	0	0	0	0	0	0	0
Solyc01g100190.2.1	8	13	6	12	1	36	55	34	4	46	15	40	0

Solyc01g100200.2.1	13	271	43	287	16	90	9	31	3	126	13	160	0
Solyc01g100210.2.1	0	0	0	0	0	0	0	0	0	0	0	0	0
Solyc01g100220.2.1	0	0	0	0	0	0	0	0	0	0	0	0	0
Solyc01g100230.2.1	6	336	5	148	7	312	54	262	17	140	38	78	3
Solyc01g100240.2.1	3	40	5	31	3	84	34	60	12	8	4	11	1
Solyc01g100250.2.1	4	65	5	26	0	43	24	97	2	21	0	18	0
Solyc01g100260.2.1	10	59	8	21	0	47	102	68	16	63	23	73	3
Solyc01g100270.2.1	4	12	0	1	0	22	38	34	2	8	3	6	1
Solyc01g100280.2.1	0	0	0	0	0	0	0	0	0	0	0	0	0
Solyc01g100290.2.1	3	11	1	5	4	9	10	31	0	21	5	6	0
Solyc01g100300.2.1	0	0	0	0	0	0	0	0	0	0	0	0	0
Solyc01g100310.2.1	2	0	3	1	0	7	1	1	0	13	4	22	0
Solyc01g100320.2.1	8	222	18	86	8	749	143	761	63	608	240	608	6
Solyc01g100330.2.1	3	23	4	2	1	21	20	37	2	22	14	12	0
Solyc01g100340.2.1	0	0	0	0	0	0	0	0	0	0	0	0	0
Solyc01g100350.2.1	4	82	24	51	5	214	145	156	33	323	66	299	8
Solyc01g100360.2.1	8	69	1	32	14	105	34	266	5	476	45	248	1
Solyc01g100370.2.1	8	100	2	60	7	503	70	244	11	1205	85	1045	13
Solyc01g100380.2.1	8	557	48	515	32	1942	385	2466	111	1831	846	1698	35
Solyc01g100390.2.1	0	0	0	0	0	0	0	0	0	0	0	0	0
Solyc01g100400.2.1	1	0	3	2	0	10	1	3	0	0	0	0	0
Solyc01g100410.2.1	3	73	4	13	0	388	18	112	7	28	8	30	0
Solyc01g100420.2.1	3	3	0	4	0	9	8	8	2	9	0	7	0
Solyc01g100430.2.1	0	0	0	0	0	0	0	0	0	0	0	0	0
Solyc01g100450.1.1	0	0	0	0	0	0	0	0	0	0	0	0	0
Solyc01g100460.2.1	5	4	0	2	0	544	37	149	3	14	8	5	0
Solyc01g100480.2.1	7	60	7	45	4	86	32	103	10	124	20	141	0
Solyc01g100490.2.1	11	440	21	274	0	163	358	245	1	3	22	1	3
Solyc01g100500.2.1	5	86	3	51	2	316	55	198	6	184	28	122	0
Solyc01g100510.2.1	0	0	0	0	0	0	0	0	0	0	0	0	0
Solyc01g100520.2.1	6	326	38	146	11	357	354	568	21	352	139	280	9
Solyc01g100530.2.1	4	27	1	23	2	6	10	2	8	6	22	4	0
Solyc01g100540.2.1	0	0	0	0	0	0	0	0	0	0	0	0	0
Solyc01g100570.2.1	11	91	4	50	8	250	92	385	25	381	56	291	3
Solyc01g100580.2.1	5	11	2	5	0	9	17	26	6	20	22	13	0
Solyc01g100590.2.1	2	22	1	11	0	25	17	27	3	38	3	33	0
Solyc01g100600.2.1	0	0	0	0	0	0	0	0	0	0	0	0	0
Solyc01g100610.2.1	11	90	20	59	9	83	102	165	13	145	32	128	8
Solyc01g100630.1.1	0	0	0	0	0	0	0	0	0	0	0	0	0
Solyc01g100640.2.1	0	0	0	0	0	0	0	0	0	0	0	0	0
Solyc01g100650.2.1	16	161	51	104	9	118	128	151	2	100	22	98	3

Solyc01g100660.2.1	7	97	10	87	1	96	72	73	2	49	13	39	1
Solyc01g100690.2.1	1	5	1	4	2	13	14	2	0	17	1	17	0
Solyc01g100700.2.1	3	22	1	19	0	48	32	56	11	47	5	50	0
Solyc01g100710.2.1	0	0	0	0	0	0	0	0	0	0	0	0	0
Solyc01g100720.2.1	8	276	7	132	8	424	214	532	46	455	35	317	1
Solyc01g100730.2.1	1	2	0	0	0	0	0	6	0	3	4	9	0
Solyc01g100740.2.1	0	0	0	0	0	0	0	0	0	0	0	0	0
Solyc01g100750.2.1	0	0	0	0	0	0	0	0	0	0	0	0	0
Solyc01g100760.2.1	5	105	0	3	5	468	57	34	118	245	2	12	0
Solyc01g100770.2.1	1	21	0	0	3	20	7	9	14	4	0	0	0
Solyc01g100780.2.1	1	2	0	0	0	5	4	3	0	21	4	18	0
Solyc01g100790.1.1	0	0	0	0	0	0	0	0	0	0	0	0	0
Solyc01g100800.1.1	0	0	0	0	0	0	0	0	0	0	0	0	0
Solyc01g100810.2.1	13	49	4	16	2	299	156	226	9	55	12	54	3
Solyc01g100820.2.1	7	33	3	15	3	79	51	87	11	64	6	60	0
Solyc01g100830.1.1	0	0	0	0	0	0	0	0	0	0	0	0	0
Solyc01g100840.2.1	4	17	5	2	0	31	28	24	2	64	5	24	0
Solyc01g100850.2.1	3	37	1	15	1	24	12	23	14	48	13	72	0
Solyc01g100860.2.1	1	61	2	7	9	102	46	81	4	265	52	115	1
Solyc01g100870.2.1	4	359	64	166	54	430	366	448	77	1787	480	1468	81
Solyc01g100900.2.1	0	0	0	0	0	0	0	0	0	0	0	0	0
Solyc01g100910.2.1	0	0	0	0	0	0	0	0	0	0	0	0	0
Solyc01g100920.2.1	1	4	3	0	0	24	0	21	0	4	1	9	0
Solyc01g100930.2.1	2	11	0	0	0	1	0	4	0	17	7	11	0
Solyc01g100940.2.1	0	0	0	0	0	0	0	0	0	0	0	0	0
Solyc01g100950.2.1	1	5	0	0	0	2	7	7	0	3	0	8	0
Solyc01g100960.2.1	3	8	1	4	0	33	11	17	2	22	0	9	0
Solyc01g100970.2.1	9	153	18	79	8	126	246	256	22	369	58	391	5
Solyc01g100980.2.1	0	0	0	0	0	0	0	0	0	0	0	0	0
Solyc01g100990.2.1	1	9	0	3	0	15	6	30	0	18	0	12	0
Solyc01g101000.2.1	2	54	7	64	1	74	112	87	25	82	38	96	7
Solyc01g101010.2.1	8	28	8	11	3	63	45	70	7	116	32	42	0
Solyc01g101020.2.1	19	209	19	158	22	575	416	508	69	336	158	258	27
Solyc01g101030.2.1	2	1	0	8	0	2	0	5	3	8	10	1	0
Solyc01g101040.2.1	5	142	14	74	5	38	152	155	20	185	52	250	6
Solyc01g101060.2.1	11	2480	332	1269	201	2341	2248	3716	377	19096	2169	8415	117
Solyc01g101070.2.1	1	10	5	5	0	14	0	32	0	32	0	26	0
Solyc01g101090.2.1	2	11	1	2	0	31	11	35	1	19	3	20	0
Solyc01g101100.2.1	2	51	10	28	3	6	42	22	0	46	40	22	3
Solyc01g101110.2.1	0	0	0	0	0	0	0	0	0	0	0	0	0
Solyc01g101120.2.1	7	40	2	17	0	110	51	119	0	39	15	65	2

Solyc01g101130.2.1	5	57	36	67	8	61	119	114	10	3	6	5	4
Solyc01g101140.2.1	11	105	8	65	1	126	58	212	12	94	4	76	0
Solyc01g101150.1.1	0	0	0	0	0	0	0	0	0	0	0	0	0
Solyc01g101160.2.1	6	34	2	5	7	45	12	39	2	66	18	41	0
Solyc01g101240.2.1	14	1031	120	720	34	1035	1382	1683	142	1421	216	1049	41
Solyc01g101250.2.1	2	17	2	11	0	20	19	12	3	36	1	36	0
Solyc01g101260.2.1	0	0	0	0	0	0	0	0	0	0	0	0	0
Solyc01g102260.2.1	0	0	0	0	0	0	0	0	0	0	0	0	0
Solyc01g102270.2.1	1	15	1	6	0	35	3	20	4	29	5	38	0
Solyc01g102280.2.1	0	0	0	0	0	0	0	0	0	0	0	0	0
Solyc01g102290.2.1	4	230	26	68	22	864	1333	456	296	149	305	104	7
Solyc01g102300.2.1	14	34	6	90	0	828	122	684	9	418	22	222	0
Solyc01g102310.2.1	30	569	57	325	19	85	185	218	11	975	159	732	12
Solyc01g102330.2.1	11	105	21	38	12	82	69	171	0	763	91	557	7
Solyc01g102340.2.1	5	27	6	13	2	1	7	0	0	68	15	81	0
Solyc01g102350.2.1	7	144	9	120	7	559	424	290	108	40	17	18	0
Solyc01g102370.2.1	1	11	0	0	0	4	7	10	0	8	0	10	0
Solyc01g102390.2.1	0	0	0	0	0	0	0	0	0	0	0	0	0
Solyc01g102400.2.1	2	14	0	7	0	22	22	18	5	10	6	3	0
Solyc01g102410.2.1	13	119	5	85	0	299	109	373	18	510	44	558	5
Solyc01g102440.2.1	0	0	0	0	0	0	0	0	0	0	0	0	0
Solyc01g102460.2.1	1	5	0	0	0	3	2	0	0	5	9	14	0
Solyc01g102470.2.1	0	0	0	0	0	0	0	0	0	0	0	0	0
Solyc01g102480.2.1	2	15	2	5	0	28	18	9	1	17	13	10	0
Solyc01g102490.2.1	3	13	4	9	8	46	34	23	5	16	27	37	1
Solyc01g102500.2.1	8	34	10	18	5	102	31	62	15	41	10	25	0
Solyc01g102510.2.1	14	90	14	59	19	114	72	181	8	206	54	158	4
Solyc01g102520.2.1	1	9	1	10	0	0	6	7	0	13	2	4	0
Solyc01g102530.2.1	6	41	5	20	12	72	26	73	10	79	10	52	2
Solyc01g102540.2.1	10	23	5	20	1	75	66	129	22	130	25	192	2
Solyc01g102550.2.1	0	0	0	0	0	0	0	0	0	0	0	0	0
Solyc01g102560.2.1	1	23	0	16	0	19	4	37	1	22	0	23	0
Solyc01g102570.2.1	1	3	4	2	0	0	15	16	1	5	10	12	2
Solyc01g102580.2.1	11	416	33	187	15	99	155	296	55	158	59	136	13
Solyc01g102590.2.1	3	64	10	36	0	196	35	162	10	73	10	62	2
Solyc01g102600.2.1	0	0	0	0	0	0	0	0	0	0	0	0	0
Solyc01g102610.2.1	10	740	142	660	39	18	26	53	2	72	38	238	1
Solyc01g102630.2.1	0	0	0	0	0	0	0	0	0	0	0	0	0
Solyc01g102640.2.1	0	0	0	0	0	0	0	0	0	0	0	0	0
Solyc01g102650.2.1	0	0	0	0	0	0	0	0	0	0	0	0	0
Solyc01g102660.2.1	6	184	19	103	12	1193	296	746	33	924	136	1223	9

Solyc01g102670.2.1	1	3	0	0	3	27	12	8	3	5	3	1	0
Solyc01g102680.2.1	0	0	0	0	0	0	0	0	0	0	0	0	0
Solyc01g102690.2.1	1	1	0	0	0	0	10	7	0	10	0	5	0
Solyc01g102700.2.1	1	3	2	1	3	6	7	6	6	2	2	6	0
Solyc01g102710.2.1	1	10	1	15	0	11	4	3	0	1	0	0	0
Solyc01g102720.2.1	0	0	0	0	0	0	0	0	0	0	0	0	0
Solyc01g102730.2.1	2	3	0	0	0	2	3	1	0	18	6	7	3
Solyc01g102740.2.1	0	0	0	0	0	0	0	0	0	0	0	0	0
Solyc01g102750.2.1	4	124	7	72	10	185	84	282	12	260	30	226	16
Solyc01g102760.2.1	3	98	8	48	9	78	122	176	42	161	22	96	3
Solyc01g102770.1.1	0	0	0	0	0	0	0	0	0	0	0	0	0
Solyc01g102790.2.1	0	0	0	0	0	0	0	0	0	0	0	0	0
Solyc01g102800.2.1	10	44	10	54	4	273	57	222	19	89	26	83	0
Solyc01g102810.2.1	8	24	3	18	6	2	40	29	3	48	9	39	3
Solyc01g102820.2.1	3	55	2	33	1	43	34	58	0	31	8	50	3
Solyc01g102830.2.1	2	55	4	11	3	61	15	41	14	119	11	94	1
Solyc01g102840.2.1	7	116	22	74	6	24	20	34	7	16	0	43	1
Solyc01g102850.1.1	3	8	1	22	0	1	7	5	0	0	0	0	0
Solyc01g102860.2.1	4	30	0	15	0	2	38	31	0	40	2	21	0
Solyc01g102870.1.1	0	0	0	0	0	0	0	0	0	0	0	0	0
Solyc01g102880.1.1	3	4	0	5	0	2	5	4	0	13	2	9	0
Solyc01g102910.2.1	0	0	0	0	0	0	0	0	0	0	0	0	0
Solyc01g102940.2.1	13	85	4	22	0	92	78	233	11	27	20	25	0
Solyc01g102950.2.1	2	10	0	10	0	0	11	2	0	3	3	8	0
Solyc01g102960.2.1	1	2	0	3	0	0	1	0	0	8	17	10	0
Solyc01g102970.2.1	19	97	8	85	5	244	180	192	44	178	32	136	1
Solyc01g102980.2.1	0	0	0	0	0	0	0	0	0	0	0	0	0
Solyc01g102990.2.1	5	16	5	12	1	68	35	46	3	55	8	32	0
Solyc01g103010.2.1	28	153	22	139	18	605	363	743	63	452	153	416	6
Solyc01g103020.2.1	7	34	4	23	9	140	87	85	7	67	16	75	0
Solyc01g103030.2.1	11	43	12	8	0	380	119	333	0	1	6	1	1
Solyc01g103040.2.1	2	8	2	5	1	1	10	23	1	15	0	20	0
Solyc01g103050.2.1	22	1305	84	1260	82	4961	702	2454	147	680	133	804	12
Solyc01g103060.2.1	2	0	0	0	0	5	4	5	0	0	0	0	0
Solyc01g103080.2.1	15	105	33	149	18	196	457	419	66	392	136	343	22
Solyc01g103090.2.1	11	21	9	17	6	28	37	57	5	43	6	35	0
Solyc01g103100.2.1	3	71	2	31	0	31	2	24	0	10	5	13	0
Solyc01g103110.2.1	4	70	3	25	0	30	22	24	1	26	2	36	7
Solyc01g103120.2.1	0	0	0	0	0	0	0	0	0	0	0	0	0
Solyc01g103130.1.1	0	0	0	0	0	0	0	0	0	0	0	0	0
Solyc01g103140.2.1	13	30	2	14	1	34	29	16	1	233	115	305	26

Solyc01g103150.2.1	4	13	2	4	0	38	12	26	2	25	4	17	0
Solyc01g103160.2.1	10	30	3	24	1	177	104	98	29	73	64	53	4
Solyc01g103170.2.1	1	3	0	1	0	7	4	6	0	2	0	0	2
Solyc01g103180.2.1	7	12	1	10	1	33	27	35	6	59	21	33	2
Solyc01g103190.2.1	1	14	6	9	1	7	7	18	7	19	10	18	0
Solyc01g103200.2.1	1	5	0	3	0	3	0	5	0	3	0	4	0
Solyc01g103210.2.1	0	0	0	0	0	0	0	0	0	0	0	0	0
Solyc01g103220.2.1	6	216	10	94	18	587	73	393	81	326	78	250	11
Solyc01g103230.2.1	7	38	7	17	23	4	36	22	7	51	8	46	13
Solyc01g103240.2.1	0	0	0	0	0	0	0	0	0	0	0	0	0
Solyc01g103250.2.1	3	9	1	6	0	4	10	6	1	170	58	208	5
Solyc01g103260.2.1	0	0	0	0	0	0	0	0	0	0	0	0	0
Solyc01g103270.2.1	0	0	0	0	0	0	0	0	0	0	0	0	0
Solyc01g103320.2.1	0	0	0	0	0	0	0	0	0	0	0	0	0
Solyc01g103340.2.1	2	9	0	0	0	17	31	4	2	20	1	2	0
Solyc01g103350.2.1	5	26	5	6	2	20	8	23	11	32	15	7	1
Solyc01g103360.2.1	0	0	0	0	0	0	0	0	0	0	0	0	0
Solyc01g103370.2.1	4	135	14	71	2	153	142	245	19	338	35	227	4
Solyc01g103380.2.1	1	13	3	19	0	26	9	24	1	22	1	21	1
Solyc01g103390.2.1	0	0	0	0	0	0	0	0	0	0	0	0	0
Solyc01g103400.2.1	5	58	1	27	1	54	26	65	1	117	13	103	2
Solyc01g103410.2.1	6	473	51	190	11	325	281	455	21	492	104	264	8
Solyc01g103420.2.1	10	37	4	22	5	122	44	157	31	96	32	85	1
Solyc01g103430.2.1	6	114	11	65	0	80	21	175	3	205	29	166	0
Solyc01g103440.2.1	6	19	3	16	0	61	29	71	4	25	7	50	0
Solyc01g103450.2.1	16	1185	229	1121	57	1508	1467	2226	92	1887	700	1571	35
Solyc01g103460.2.1	3	26	0	16	0	10	5	33	0	27	6	12	0
Solyc01g103470.2.1	0	0	0	0	0	0	0	0	0	0	0	0	0
Solyc01g103480.2.1	16	332	12	208	21	656	343	909	63	728	84	542	7
Solyc01g103490.2.1	3	29	0	10	2	9	19	10	1	42	6	27	0
Solyc01g103500.2.1	0	0	0	0	0	0	0	0	0	0	0	0	0
Solyc01g103510.2.1	13	42	7	19	5	295	114	275	58	36	70	53	0
Solyc01g103530.2.1	5	44	2	35	0	21	32	115	0	36	6	80	0
Solyc01g103540.2.1	22	1907	277	1898	91	3323	3424	3995	227	4045	821	3337	64
Solyc01g103560.2.1	2	4	1	9	0	6	3	5	2	16	5	15	0
Solyc01g103570.2.1	5	7	1	13	3	42	25	39	2	29	9	16	0
Solyc01g103580.2.1	1	10	0	0	0	1	6	4	0	5	2	1	0
Solyc01g103590.2.1	4	70	6	15	14	15	24	17	12	65	2	26	2
Solyc01g103600.2.1	0	0	0	0	0	0	0	0	0	0	0	0	0
Solyc01g103610.2.1	0	0	0	0	0	0	0	0	0	0	0	0	0
Solyc01g103620.2.1	0	0	0	0	0	0	0	0	0	0	0	0	0

Solyc01g103640.2.1	0	0	0	0	0	0	0	0	0	0	0	0	0
Solyc01g103650.2.1	0	0	0	0	0	0	0	0	0	0	0	0	0
Solyc01g103660.2.1	4	4	3	0	0	66	13	28	3	3	8	0	0
Solyc01g103670.2.1	7	26	4	17	7	18	53	59	8	44	40	46	8
Solyc01g103680.2.1	13	171	21	133	12	639	288	971	80	410	100	350	2
Solyc01g103690.2.1	10	34	5	20	6	79	110	104	12	44	15	31	2
Solyc01g103700.2.1	0	0	0	0	0	0	0	0	0	0	0	0	0
Solyc01g103710.2.1	8	224	51	114	4	659	409	474	14	138	28	80	0
Solyc01g103720.2.1	0	0	0	0	0	0	0	0	0	0	0	0	0
Solyc01g103730.2.1	1	5	0	3	0	17	1	23	0	11	5	6	0
Solyc01g103740.2.1	8	24	1	24	5	168	54	257	7	170	20	159	0
Solyc01g103750.2.1	6	92	10	43	1	23	55	76	5	96	13	100	3
Solyc01g103760.2.1	2	78	4	20	2	18	7	12	1	30	2	10	0
Solyc01g103770.2.1	0	0	0	0	0	0	0	0	0	0	0	0	0
Solyc01g103780.2.1	15	177	28	136	17	66	62	198	8	318	55	199	3
Solyc01g103790.2.1	0	0	0	0	0	0	0	0	0	0	0	0	0
Solyc01g103800.2.1	4	382	13	182	1	735	64	550	11	3405	76	3001	33
Solyc01g103830.1.1	0	0	0	0	0	0	0	0	0	0	0	0	0
Solyc01g103850.2.1	6	53	7	44	9	105	93	138	14	64	31	52	0
Solyc01g103860.2.1	1	0	0	0	0	7	2	14	0	0	2	0	0
Solyc01g103870.1.1	0	0	0	0	0	0	0	0	0	0	0	0	0
Solyc01g103890.2.1	9	119	17	62	8	176	231	229	72	118	49	138	7
Solyc01g103920.2.1	2	8	3	0	0	13	7	5	0	11	7	22	0
Solyc01g103930.2.1	14	205	17	137	11	459	222	589	70	361	59	333	11
Solyc01g103940.2.1	2	85	7	34	0	112	92	157	9	197	48	282	7
Solyc01g103950.2.1	5	37	0	15	0	28	17	37	2	60	10	100	0
Solyc01g103960.2.1	0	0	0	0	0	0	0	0	0	0	0	0	0
Solyc01g103970.2.1	7	28	5	5	6	62	101	117	16	81	15	47	2
Solyc01g103980.2.1	15	77	12	86	9	154	286	303	28	215	34	160	4
Solyc01g103990.2.1	50	1080	75	787	42	7949	2085	7323	230	968	360	788	15
Solyc01g104000.2.1	12	124	7	93	9	192	69	157	12	142	55	129	0
Solyc01g104010.2.1	0	0	0	0	0	0	0	0	0	0	0	0	0
Solyc01g104020.2.1	5	123	18	40	13	51	300	96	86	91	103	55	17
Solyc01g104030.2.1	16	162	40	154	17	192	149	454	41	239	67	201	11
Solyc01g104040.2.1	17	97	13	92	5	335	246	512	53	127	43	113	9
Solyc01g104050.2.1	4	8	5	10	0	68	20	36	3	22	5	12	0
Solyc01g104060.2.1	0	0	0	0	0	0	0	0	0	0	0	0	0
Solyc01g104070.2.1	7	28	5	17	2	83	62	62	14	256	49	305	12
Solyc01g104080.2.1	5	47	6	25	2	268	97	179	27	135	23	121	1
Solyc01g104090.2.1	0	0	0	0	0	0	0	0	0	0	0	0	0
Solyc01g104100.2.1	5	12	22	3	22	41	103	3	7	0	103	0	0

Solyc01g104110.2.1	10	16	1	6	6	3537	15	309	4	5	2313	16	3
Solyc01g104130.2.1	8	50	1	41	2	155	44	129	2	219	21	116	0
Solyc01g104140.2.1	8	58	3	22	3	158	44	165	14	93	30	62	0
Solyc01g104160.2.1	10	76	16	89	8	187	180	185	46	123	33	65	10
Solyc01g104170.2.1	11	878	75	844	32	1667	960	1658	100	1505	400	1188	19
Solyc01g104190.2.1	3	14	7	0	3	9	25	11	3	13	4	6	0
Solyc01g104200.2.1	0	0	0	0	0	0	0	0	0	0	0	0	0
Solyc01g104220.2.1	6	26	1	25	2	85	22	90	9	73	3	90	0
Solyc01g104230.2.1	0	0	0	0	0	0	0	0	0	0	0	0	0
Solyc01g104240.2.1	6	28	4	20	1	37	42	38	7	25	13	25	2
Solyc01g104250.2.1	0	0	0	0	0	0	0	0	0	0	0	0	0
Solyc01g104260.2.1	0	0	0	0	0	0	0	0	0	0	0	0	0
Solyc01g104270.2.1	10	163	14	108	4	255	169	320	13	181	26	166	0
Solyc01g104280.2.1	0	0	0	0	0	0	0	0	0	0	0	0	0
Solyc01g104290.1.1	0	0	0	0	0	0	0	0	0	0	0	0	0
Solyc01g104300.2.1	18	43	7	22	1	424	459	295	23	91	27	95	1
Solyc01g104310.2.1	17	74	4	53	10	76	150	144	6	193	32	121	1
Solyc01g104320.2.1	13	32	9	18	1	47	357	27	6	155	25	73	1
Solyc01g104340.1.1	0	0	0	0	0	0	0	0	0	0	0	0	0
Solyc01g104350.2.1	4	11	0	22	0	47	14	28	3	21	4	31	0
Solyc01g104370.2.1	4	122	4	82	0	116	20	169	11	203	17	166	0
Solyc01g104380.2.1	0	0	0	0	0	0	0	0	0	0	0	0	0
Solyc01g104390.1.1	0	0	0	0	0	0	0	0	0	0	0	0	0
Solyc01g104400.2.1	3	34	0	8	9	9	8	11	0	37	0	4	0
Solyc01g104410.2.1	1	1	0	0	0	14	3	3	2	9	2	5	0
Solyc01g104430.1.1	0	0	0	0	0	0	0	0	0	0	0	0	0
Solyc01g104460.2.1	1	6	1	2	0	3	0	4	2	7	10	7	0
Solyc01g104470.2.1	5	128	25	86	15	102	145	145	18	741	135	734	21
Solyc01g104480.2.1	1	12	2	6	2	28	4	33	1	4	2	10	0
Solyc01g104490.2.1	0	0	0	0	0	0	0	0	0	0	0	0	0
Solyc01g104500.2.1	4	52	8	27	1	37	59	99	20	90	89	39	1
Solyc01g104510.2.1	4	32	4	18	0	45	29	49	10	62	15	16	0
Solyc01g104520.1.1	0	0	0	0	0	0	0	0	0	0	0	0	0
Solyc01g104530.2.1	15	128	9	58	2	170	113	163	29	147	38	110	3
Solyc01g104540.2.1	2	7	2	6	0	23	5	23	4	24	0	18	0
Solyc01g104550.2.1	0	0	0	0	0	0	0	0	0	0	0	0	0
Solyc01g104560.2.1	11	114	8	37	7	261	68	201	18	257	62	216	8
Solyc01g104570.2.1	1	3	0	1	0	5	4	2	1	0	0	2	0
Solyc01g104580.2.1	1	10	0	0	0	5	1	3	2	19	3	3	0
Solyc01g104590.2.1	6	537	23	501	44	1051	147	1836	35	1541	250	1940	37
Solyc01g104600.2.1	1	14	0	2	0	29	16	19	2	18	2	14	1

Solyc01g104630.2.1	0	0	0	0	0	0	0	0	0	0	0	0	0
Solyc01g104640.2.1	7	13	3	7	0	25	59	24	6	56	23	51	4
Solyc01g104650.2.1	7	7	0	15	3	189	31	255	4	48	21	34	0
Solyc01g104660.2.1	6	95	6	37	4	271	64	223	15	157	24	107	1
Solyc01g104670.2.1	9	108	6	57	5	80	59	94	16	129	24	164	1
Solyc01g104680.2.1	3	110	11	54	6	188	126	191	14	505	137	435	11
Solyc01g104690.2.1	4	51	26	9	18	6	405	40	17	0	117	1	9
Solyc01g104700.2.1	1	1	0	2	0	28	5	15	0	9	0	6	0
Solyc01g104710.2.1	2	81	0	15	0	110	52	86	0	73	22	41	0
Solyc01g104720.2.1	4	58	195	44	45	72	2699	180	92	602	447	361	24
Solyc01g104740.2.1	4	16	0	16	1	41	6	27	8	59	41	36	0
Solyc01g104750.2.1	0	0	0	0	0	0	0	0	0	0	0	0	0
Solyc01g104760.2.1	0	0	0	0	0	0	0	0	0	0	0	0	0
Solyc01g104770.2.1	7	119	8	59	16	143	50	164	29	629	94	363	32
Solyc01g104780.2.1	6	28	1	31	0	17	18	16	0	5	2	52	0
Solyc01g104790.2.1	6	27	2	30	1	51	26	76	1	123	18	89	1
Solyc01g104800.2.1	0	0	0	0	0	0	0	0	0	0	0	0	0
Solyc01g104840.2.1	8	398	33	249	4	438	260	644	71	1067	140	811	11
Solyc01g104850.2.1	0	0	0	0	0	0	0	0	0	0	0	0	0
Solyc01g104860.2.1	1	0	0	1	2	3	0	0	0	4	4	3	0
Solyc01g104880.2.1	6	19	2	6	1	43	42	27	6	51	11	46	5
Solyc01g104890.2.1	0	0	0	0	0	0	0	0	0	0	0	0	0
Solyc01g104900.2.1	2	5	1	1	0	0	13	2	0	10	2	15	0
Solyc01g104910.2.1	10	237	90	194	12	163	251	524	20	1320	166	210	6
Solyc01g104920.2.1	9	184	16	50	0	487	201	487	82	414	73	331	5
Solyc01g104930.1.1	0	0	0	0	0	0	0	0	0	0	0	0	0
Solyc01g104950.2.1	13	374	40	239	14	103	44	333	2	629	51	74	9
Solyc01g104970.2.1	5	63	5	44	1	37	34	73	1	8	1	6	0
Solyc01g104980.2.1	10	54	4	63	2	127	94	177	22	255	31	200	1
Solyc01g104990.2.1	0	0	0	0	0	0	0	0	0	0	0	0	0
Solyc01g105000.2.1	0	0	0	0	0	0	0	0	0	0	0	0	0
Solyc01g105010.2.1	1	1	0	1	0	9	5	5	0	3	0	0	0
Solyc01g105020.2.1	9	213	48	149	4	41	34	55	5	44	23	37	2
Solyc01g105030.2.1	7	4928	137	3945	1	59	23	82	2	1146	107	879	7
Solyc01g105040.2.1	3	13	1	13	0	6	10	11	6	15	12	3	0
Solyc01g105050.2.1	6	1418	163	730	0	7	17	21	2	116	81	101	2
Solyc01g105060.2.1	6	48	0	19	2	120	33	178	8	94	19	73	1
Solyc01g105070.2.1	1	2	0	1	0	6	7	13	0	3	0	6	0
Solyc01g105090.2.1	1	1	0	0	0	2	2	8	5	8	0	3	0
Solyc01g105100.2.1	4	43	4	37	2	169	84	237	20	200	32	164	4
Solyc01g105120.2.1	21	705	112	625	35	694	140	196	21	290	282	299	9

Solyc01g105130.2.1	4	10	7	4	0	49	28	39	8	50	10	49	0
Solyc01g105140.2.1	4	35	7	19	8	137	56	101	11	54	27	71	0
Solyc01g105150.2.1	14	63	8	46	5	143	83	164	34	111	41	104	1
Solyc01g105160.2.1	5	11	0	6	2	51	15	58	2	49	10	33	2
Solyc01g105170.2.1	3	77	10	74	9	139	76	111	8	197	45	155	4
Solyc01g105180.2.1	0	0	0	0	0	0	0	0	0	0	0	0	0
Solyc01g105200.2.1	0	0	0	0	0	0	0	0	0	0	0	0	0
Solyc01g105210.2.1	0	0	0	0	0	0	0	0	0	0	0	0	0
Solyc01g105220.2.1	1	1	0	1	0	2	4	0	1	1	0	4	0
Solyc01g105230.2.1	18	56	12	45	5	283	225	401	88	214	59	185	0
Solyc01g105240.2.1	7	39	17	23	17	111	198	164	180	70	71	65	34
Solyc01g105270.2.1	0	0	0	0	0	0	0	0	0	0	0	0	0
Solyc01g105280.2.1	4	59	0	28	1	63	41	50	18	38	27	24	1
Solyc01g105290.2.1	11	594	38	395	20	242	77	416	11	92	70	140	5
Solyc01g105300.2.1	0	0	0	0	0	0	0	0	0	0	0	0	0
Solyc01g105310.2.1	3	19	0	2	0	19	14	12	0	15	0	6	0
Solyc01g105320.2.1	1	1	0	1	0	0	1	3	0	3	0	4	0
Solyc01g105330.2.1	2	3	0	5	0	4	11	21	2	5	4	0	0
Solyc01g105340.2.1	4	340	18	263	2	418	119	679	19	801	63	603	6
Solyc01g105350.1.1	0	0	0	0	0	0	0	0	0	0	0	0	0
Solyc01g105360.2.1	0	0	0	0	0	0	0	0	0	0	0	0	0
Solyc01g105370.2.1	6	7	1	11	0	234	112	176	5	14	101	3	0
Solyc01g105390.2.1	0	0	0	0	0	0	0	0	0	0	0	0	0
Solyc01g105410.2.1	16	339	35	255	28	31	241	125	177	241	12	285	0
Solyc01g105420.2.1	0	0	0	0	0	0	0	0	0	0	0	0	0
Solyc01g105430.2.1	0	0	0	0	0	0	0	0	0	0	0	0	0
Solyc01g105440.1.1	0	0	0	0	0	0	0	0	0	0	0	0	0
Solyc01g105450.2.1	2	3	4	0	0	456	6	7	6	9	0	0	0
Solyc01g105460.2.1	13	84	13	69	2	125	150	140	1	76	19	68	0
Solyc01g105470.2.1	0	0	0	0	0	0	0	0	0	0	0	0	0
Solyc01g105490.2.1	0	0	0	0	0	0	0	0	0	0	0	0	0
Solyc01g105510.2.1	0	0	0	0	0	0	0	0	0	0	0	0	0
Solyc01g105520.2.1	0	0	0	0	0	0	0	0	0	0	0	0	0
Solyc01g105530.2.1	0	0	0	0	0	0	0	0	0	0	0	0	0
Solyc01g105540.2.1	4	72	16	60	0	40	42	86	9	72	58	65	0
Solyc01g105560.2.1	15	272	30	223	6	346	119	387	15	137	44	169	0
Solyc01g105570.2.1	0	0	0	0	0	0	0	0	0	0	0	0	0
Solyc01g105600.2.1	0	0	0	0	0	0	0	0	0	0	0	0	0
Solyc01g105620.2.1	0	0	0	0	0	0	0	0	0	0	0	0	0
Solyc01g105630.2.1	0	0	0	0	0	0	0	0	0	0	0	0	0
Solyc01g105660.2.1	23	262	7	224	5	2844	2018	3600	1	2916	241	1645	14

Solyc01g105670.2.1	3	23	6	21	0	25	55	15	6	50	5	31	0
Solyc01g105680.2.1	6	148	13	128	9	18	151	205	20	5	1	1	1
Solyc01g105690.2.1	3	18	0	7	0	11	14	44	0	26	3	17	0
Solyc01g105700.2.1	2	9	2	12	0	23	10	22	0	5	0	3	0
Solyc01g105710.2.1	0	0	0	0	0	0	0	0	0	0	0	0	0
Solyc01g105720.2.1	5	55	4	64	0	48	37	94	4	608	10	517	0
Solyc01g105730.1.1	0	0	0	0	0	0	0	0	0	0	0	0	0
Solyc01g105740.1.1	0	0	0	0	0	0	0	0	0	0	0	0	0
Solyc01g105750.1.1	0	0	0	0	0	0	0	0	0	0	0	0	0
Solyc01g105760.1.1	0	0	0	0	0	0	0	0	0	0	0	0	0
Solyc01g105770.2.1	15	11	0	9	0	276	253	166	190	3	10	2	0
Solyc01g105780.2.1	6	23	2	18	1	36	23	71	7	28	7	50	0
Solyc01g105800.2.1	8	94	4	36	0	66	56	72	8	0	1	2	0
Solyc01g105810.2.1	0	0	0	0	0	0	0	0	0	0	0	0	0
Solyc01g105820.2.1	0	0	0	0	0	0	0	0	0	0	0	0	0
Solyc01g105830.2.1	10	43	17	32	3	91	73	101	16	77	31	72	1
Solyc01g105850.2.1	0	0	0	0	0	0	0	0	0	0	0	0	0
Solyc01g105860.2.1	0	0	0	0	0	0	0	0	0	0	0	0	0
Solyc01g105870.2.1	0	0	0	0	0	0	0	0	0	0	0	0	0
Solyc01g105880.2.1	0	0	0	0	0	0	0	0	0	0	0	0	0
Solyc01g105890.2.1	0	0	0	0	0	0	0	0	0	0	0	0	0
Solyc01g105920.2.1	0	0	0	0	0	0	0	0	0	0	0	0	0
Solyc01g105970.2.1	2	12	0	9	0	33	14	51	2	33	1	20	0
Solyc01g105980.2.1	1	1	0	0	0	7	3	1	0	2	2	12	1
Solyc01g105990.2.1	15	154	24	164	11	183	191	310	28	242	24	126	2
Solyc01g106000.2.1	7	382	18	263	14	752	573	1340	175	650	85	342	2
Solyc01g106010.2.1	10	79	20	54	9	59	140	79	7	93	12	64	1
Solyc01g106020.2.1	3	3	2	3	1	1	25	8	3	12	3	8	0
Solyc01g106030.2.1	5	61	4	23	3	104	44	102	11	141	16	84	7
Solyc01g106040.2.1	0	0	0	0	0	0	0	0	0	0	0	0	0
Solyc01g106050.2.1	9	47	6	37	2	181	62	108	31	69	33	61	0
Solyc01g106060.2.1	5	81	7	43	5	131	128	212	15	270	27	109	3
Solyc01g106080.2.1	14	116	20	67	4	355	613	532	76	301	61	181	12
Solyc01g106090.2.1	4	22	1	3	6	1	13	16	2	3	8	15	2
Solyc01g106100.2.1	1	3	0	2	0	7	0	6	0	2	1	1	0
Solyc01g106130.1.1	0	0	0	0	0	0	0	0	0	0	0	0	0
Solyc01g106150.1.1	0	0	0	0	0	0	0	0	0	0	0	0	0
Solyc01g106170.2.1	0	0	0	0	0	0	0	0	0	0	0	0	0
Solyc01g106190.2.1	4	5	18	6	9	11	112	17	11	16	7	34	9
Solyc01g106210.2.1	12	334	25	186	26	976	281	1206	186	463	372	458	2
Solyc01g106260.2.1	6	57	9	26	9	159	61	115	18	66	52	49	0

Solyc01g106270.1.1	0	0	0	0	0	0	0	0	0	0	0	0	0
Solyc01g106280.2.1	17	1399	112	852	45	1379	564	1129	95	947	218	948	17
Solyc01g106310.2.1	1	25	2	0	0	12	19	10	0	11	11	12	0
Solyc01g106320.2.1	16	238	168	254	88	739	272	765	151	1649	266	1046	29
Solyc01g106330.2.1	7	43	5	38	5	21	37	23	14	235	23	210	1
Solyc01g106340.2.1	0	0	0	0	0	0	0	0	0	0	0	0	0
Solyc01g106350.2.1	1	0	1	2	0	8	11	3	0	0	1	1	0
Solyc01g106360.2.1	6	63	7	22	7	36	63	63	11	376	23	201	8
Solyc01g106370.2.1	1	4	0	2	0	15	7	19	0	27	2	5	0
Solyc01g106380.2.1	1	14	0	0	0	1	3	5	2	6	1	6	0
Solyc01g106390.2.1	10	133	53	92	29	27	1	34	4	14	4	44	1
Solyc01g106400.2.1	4	65	7	30	0	2	28	4	0	2	0	2	0
Solyc01g106410.1.1	0	0	0	0	0	0	0	0	0	0	0	0	0
Solyc01g106420.2.1	2	5	4	10	1	4	8	16	1	9	0	7	1
Solyc01g106430.2.1	6	253	29	111	1	37	50	107	8	114	11	119	1
Solyc01g106440.2.1	7	18	4	9	5	46	95	52	19	68	30	83	1
Solyc01g106450.2.1	6	55	5	27	4	22	49	44	6	99	33	63	6
Solyc01g106460.2.1	0	0	0	0	0	0	0	0	0	0	0	0	0
Solyc01g106470.2.1	14	133	16	64	7	113	47	148	27	202	286	177	19
Solyc01g106480.2.1	5	824	83	432	1	45	88	87	4	110	26	102	1
Solyc01g106500.2.1	5	13	4	6	1	55	6	56	0	15	7	13	0
Solyc01g106540.2.1	0	0	0	0	0	0	0	0	0	0	0	0	0
Solyc01g106560.2.1	3	5	0	5	0	18	10	33	0	22	4	17	0
Solyc01g106570.2.1	5	52	1	29	6	58	33	68	14	61	4	41	1
Solyc01g106580.2.1	11	142	12	47	11	32	42	132	7	389	48	338	5
Solyc01g106590.2.1	6	20	6	4	7	4	5	8	0	52	11	15	0
Solyc01g106620.2.1	3	67	0	15	0	51	8	108	2	6	3	1	0
Solyc01g106650.2.1	0	0	0	0	0	0	0	0	0	0	0	0	0
Solyc01g106680.2.1	16	112	13	47	14	179	157	293	15	282	57	162	4
Solyc01g106690.2.1	9	741	14	179	6	22	32	54	4	215	16	101	0
Solyc01g106700.2.1	0	0	0	0	0	0	0	0	0	0	0	0	0
Solyc01g106740.2.1	1	5	0	2	0	18	3	12	0	4	5	5	0
Solyc01g106750.1.1	0	0	0	0	0	0	0	0	0	0	0	0	0
Solyc01g106770.2.1	11	40	6	25	0	58	70	86	4	117	29	63	4
Solyc01g106780.2.1	7	21	3	11	6	96	41	85	18	161	18	61	6
Solyc01g106790.2.1	5	88	8	26	9	16	73	33	5	9	32	35	0
Solyc01g106800.2.1	1	4	0	2	0	4	2	25	4	4	2	4	0
Solyc01g106810.2.1	1	50	0	27	0	174	10	58	1	89	4	23	0
Solyc01g106820.2.1	17	99	11	137	7	383	101	671	0	34	54	36	1
Solyc01g106830.2.1	0	0	0	0	0	0	0	0	0	0	0	0	0
Solyc01g106870.2.1	0	0	0	0	0	0	0	0	0	0	0	0	0

Solyc01g106880.2.1	0	0	0	0	0	0	0	0	0	0	0	0	0
Solyc01g106890.2.1	1	8	0	0	0	1	6	8	0	2	0	0	0
Solyc01g106900.2.1	5	91	13	60	4	118	51	119	5	168	27	92	0
Solyc01g106910.2.1	3	38	2	8	0	50	16	9	0	1	0	5	0
Solyc01g106920.2.1	0	0	0	0	0	0	0	0	0	0	0	0	0
Solyc01g106940.2.1	0	0	0	0	0	0	0	0	0	0	0	0	0
Solyc01g106950.2.1	3	12	2	6	0	10	17	20	2	22	3	12	0
Solyc01g106970.1.1	0	0	0	0	0	0	0	0	0	0	0	0	0
Solyc01g107010.2.1	0	0	0	0	0	0	0	0	0	0	0	0	0
Solyc01g107020.2.1	0	0	0	0	0	0	0	0	0	0	0	0	0
Solyc01g107030.2.1	8	63	12	32	6	106	106	131	16	159	55	116	5
Solyc01g107040.2.1	9	59	13	60	5	195	90	181	11	353	25	305	8
Solyc01g107050.2.1	0	0	0	0	0	0	0	0	0	0	0	0	0
Solyc01g107070.2.1	0	0	0	0	0	0	0	0	0	0	0	0	0
Solyc01g107080.2.1	0	0	0	0	0	0	0	0	0	0	0	0	0
Solyc01g107090.2.1	0	0	0	0	0	0	0	0	0	0	0	0	0
Solyc01g107100.2.1	1	6	0	5	0	7	6	1	0	4	0	0	0
Solyc01g107130.2.1	9	168	20	73	3	380	188	476	24	183	59	141	13
Solyc01g107150.2.1	4	17	4	2	1	35	10	33	13	37	11	27	7
Solyc01g107160.2.1	4	36	3	15	1	25	20	49	0	31	11	39	0
Solyc01g107170.2.1	16	154	19	126	4	2347	300	710	81	98	41	75	4
Solyc01g107180.2.1	7	73	17	19	8	50	24	30	23	18	36	23	1
Solyc01g107190.2.1	3	41	4	34	0	47	20	29	0	33	0	52	0
Solyc01g107200.2.1	1	8	0	7	0	10	6	12	0	8	2	4	1
Solyc01g107210.2.1	0	0	0	0	0	0	0	0	0	0	0	0	0
Solyc01g107220.2.1	0	0	0	0	0	0	0	0	0	0	0	0	0
Solyc01g107230.2.1	0	0	0	0	0	0	0	0	0	0	0	0	0
Solyc01g107240.2.1	0	0	0	0	0	0	0	0	0	0	0	0	0
Solyc01g107250.2.1	5	39	4	10	6	57	72	64	10	29	18	46	0
Solyc01g107260.2.1	0	0	0	0	0	0	0	0	0	0	0	0	0
Solyc01g107270.2.1	0	0	0	0	0	0	0	0	0	0	0	0	0
Solyc01g107280.2.1	0	0	0	0	0	0	0	0	0	0	0	0	0
Solyc01g107290.2.1	8	258	121	124	23	90	80	76	33	73	17	104	6
Solyc01g107300.2.1	4	19	3	8	0	5	15	12	0	14	3	7	0
Solyc01g107310.2.1	4	31	11	24	5	30	33	11	4	19	8	30	0
Solyc01g107320.2.1	6	34	3	14	1	45	39	69	2	26	2	15	0
Solyc01g107330.2.1	3	159	99	88	12	54	374	130	8	111	51	152	4
Solyc01g107340.2.1	7	114	16	23	14	185	82	213	8	29	3	3	0
Solyc01g107350.2.1	3	22	4	14	2	3	7	12	0	3	2	5	2
Solyc01g107360.2.1	1	3	0	7	0	1	5	23	1	5	0	0	0
Solyc01g107380.2.1	8	282	18	167	16	211	230	366	10	293	37	306	1

Solyc01g107390.2.1	2	1	1	0	3	1	25	0	7	0	13	0	0
Solyc01g107400.2.1	0	0	0	0	0	0	0	0	0	0	0	0	0
Solyc01g107410.2.1	4	8	0	14	0	9	8	9	2	27	3	12	0
Solyc01g107420.2.1	0	0	0	0	0	0	0	0	0	0	0	0	0
Solyc01g107430.2.1	0	0	0	0	0	0	0	0	0	0	0	0	0
Solyc01g107450.2.1	4	33	2	18	2	28	26	54	4	60	11	39	1
Solyc01g107460.2.1	1	52	2	56	4	2	4	1	0	0	0	1	0
Solyc01g107470.2.1	0	0	0	0	0	0	0	0	0	0	0	0	0
Solyc01g107500.2.1	0	0	0	0	0	0	0	0	0	0	0	0	0
Solyc01g107510.2.1	16	46	10	34	3	157	95	68	11	69	29	62	2
Solyc01g107520.2.1	0	0	0	0	0	0	0	0	0	0	0	0	0
Solyc01g107550.2.1	8	97	4	45	2	284	122	368	30	196	38	128	4
Solyc01g107560.2.1	8	35	14	19	4	27	85	56	3	13	8	3	0
Solyc01g107580.2.1	1	0	1	0	0	4	3	1	0	0	1	7	0
Solyc01g107590.2.1	6	233	31	188	5	112	179	362	15	26	8	34	0
Solyc01g107600.2.1	4	26	1	10	1	54	31	39	6	9	7	27	7
Solyc01g107610.2.1	3	5	3	3	0	15	20	20	2	26	8	10	0
Solyc01g107620.2.1	0	0	0	0	0	0	0	0	0	0	0	0	0
Solyc01g107630.2.1	1	1	3	6	0	5	14	8	1	1	3	0	0
Solyc01g107640.2.1	13	267	19	118	4	446	157	548	19	353	41	365	2
Solyc01g107650.2.1	0	0	0	0	0	0	0	0	0	0	0	0	0
Solyc01g107660.2.1	4	187	36	82	0	30	123	55	7	48	33	66	4
Solyc01g107670.2.1	1	9	1	8	0	2	3	7	1	4	0	12	1
Solyc01g107680.2.1	0	0	0	0	0	0	0	0	0	0	0	0	0
Solyc01g107690.2.1	0	0	0	0	0	0	0	0	0	0	0	0	0
Solyc01g107700.2.1	10	86	17	63	7	486	427	427	58	123	101	81	2
Solyc01g107730.2.1	2	2	0	2	0	1	8	0	0	45	6	59	0
Solyc01g107740.2.1	4	12	2	12	0	50	29	30	18	19	12	57	0
Solyc01g107750.2.1	21	232	19	241	21	326	466	503	51	404	143	350	5
Solyc01g107760.2.1	0	0	0	0	0	0	0	0	0	0	0	0	0
Solyc01g107780.2.1	0	0	0	0	0	0	0	0	0	0	0	0	0
Solyc01g107790.2.1	0	0	0	0	0	0	0	0	0	0	0	0	0
Solyc01g107800.2.1	3	58	10	18	4	15	3	6	7	75	53	47	4
Solyc01g107810.2.1	0	0	0	0	0	0	0	0	0	0	0	0	0
Solyc01g107820.2.1	16	673	22	348	11	1761	92	566	22	63	71	22	3
Solyc01g107830.2.1	3	0	0	0	0	7	0	15	0	13	72	10	0
Solyc01g107850.2.1	0	0	0	0	0	0	0	0	0	0	0	0	0
Solyc01g107860.2.1	6	77	10	38	7	334	114	424	14	38	12	80	3
Solyc01g107870.2.1	14	1327	142	1152	84	2709	2093	3302	189	2294	1126	2581	47
Solyc01g107880.2.1	28	413	42	402	37	1089	467	1268	120	805	180	659	5
Solyc01g107890.2.1	11	49	8	37	17	13	3	21	3	461	47	353	0

Solyc01g107900.2.1	12	288	18	159	40	576	157	455	137	200	36	165	2
Solyc01g107910.2.1	6	92	22	73	0	42	82	71	5	20	32	13	0
Solyc01g107920.2.1	2	0	4	0	0	2	3	16	1	6	3	2	0
Solyc01g107930.2.1	11	20	1	34	0	25	48	54	5	69	8	31	0
Solyc01g107940.2.1	6	9	5	4	2	18	47	32	3	29	6	18	4
Solyc01g107980.2.1	14	564	71	249	17	81	37	60	0	85	18	54	1
Solyc01g107990.2.1	9	29	3	6	0	27	68	21	0	68	7	88	5
Solyc01g108000.2.1	0	0	0	0	0	0	0	0	0	0	0	0	0
Solyc01g108010.2.1	5	24	8	17	1	52	47	74	8	51	7	23	1
Solyc01g108020.2.1	3	88	5	31	0	28	2	23	0	108	6	44	0
Solyc01g108030.2.1	15	30	8	28	14	658	881	814	25	193	58	173	4
Solyc01g108040.1.1	1	1	0	2	0	2	6	3	1	8	0	4	0
Solyc01g108070.2.1	1	13	1	7	0	13	32	5	3	22	0	17	1
Solyc01g108080.2.1	11	30	7	26	2	232	100	126	4	124	54	93	1
Solyc01g108100.2.1	3	7	0	0	0	1	22	11	0	50	3	36	5
Solyc01g108110.2.1	3	3	0	0	0	51	9	14	3	6	8	6	0
Solyc01g108120.2.1	0	0	0	0	0	0	0	0	0	0	0	0	0
Solyc01g108130.2.1	2	27	4	12	0	18	7	10	0	15	2	20	0
Solyc01g108140.2.1	1	5	0	1	0	8	11	4	2	1	8	1	0
Solyc01g108150.2.1	13	100	14	62	3	473	277	402	51	444	57	244	2
Solyc01g108160.2.1	23	44	5	41	1	243	154	185	13	84	23	92	1
Solyc01g108170.2.1	0	0	0	0	0	0	0	0	0	0	0	0	0
Solyc01g108180.2.1	9	27	8	8	0	43	45	97	6	32	21	24	1
Solyc01g108190.2.1	3	27	16	23	0	56	161	37	0	8	21	3	0
Solyc01g108200.2.1	2	15	0	0	0	17	5	12	0	3	0	0	0
Solyc01g108210.2.1	0	0	0	0	0	0	0	0	0	0	0	0	0
Solyc01g108230.2.1	0	0	0	0	0	0	0	0	0	0	0	0	0
Solyc01g108240.2.1	4	109	10	42	6	11	0	1	0	0	0	10	0
Solyc01g108250.2.1	17	242	20	219	8	1621	156	503	115	209	62	164	1
Solyc01g108260.2.1	7	91	14	41	4	148	113	95	21	222	90	141	1
Solyc01g108280.2.1	4	46	5	29	6	49	81	86	16	94	11	71	0
Solyc01g108290.2.1	5	23	2	9	1	36	22	39	9	28	14	19	0
Solyc01g108300.2.1	12	61	10	59	0	210	148	258	4	136	17	246	1
Solyc01g108330.2.1	0	0	0	0	0	0	0	0	0	0	0	0	0
Solyc01g108340.2.1	8	56	4	47	4	73	72	115	9	365	91	329	0
Solyc01g108390.2.1	2	17	0	2	0	5	3	12	0	2	1	1	0
Solyc01g108400.2.1	4	33	0	19	0	39	4	46	0	1	13	3	0
Solyc01g108410.1.1	1	1	0	0	0	2	3	4	0	6	1	7	0
Solyc01g108420.2.1	0	0	0	0	0	0	0	0	0	0	0	0	0
Solyc01g108430.2.1	2	9	0	5	0	7	7	10	2	3	4	4	0
Solyc01g108440.2.1	0	0	0	0	0	0	0	0	0	0	0	0	0

Solyc01g108450.2.1	0	0	0	0	0	0	0	0	0	0	0	0	0
Solyc01g108480.1.1	0	0	0	0	0	0	0	0	0	0	0	0	0
Solyc01g108490.2.1	11	129	29	64	3	196	160	406	6	357	15	247	0
Solyc01g108500.2.1	8	937	33	435	39	741	359	1048	52	2797	310	1622	16
Solyc01g108510.2.1	1	1	1	3	0	4	2	2	0	6	2	4	0
Solyc01g108520.1.1	0	0	0	0	0	0	0	0	0	0	0	0	0
Solyc01g108530.2.1	7	65	0	58	13	128	32	187	5	145	6	41	0
Solyc01g108540.2.1	9	57	13	41	0	371	272	347	3	44	11	25	1
Solyc01g108550.1.1	0	0	0	0	0	0	0	0	0	0	0	0	0
Solyc01g108560.2.1	1	0	0	0	0	9	16	7	1	0	0	1	0
Solyc01g108570.2.1	2	4	1	1	0	10	19	33	0	6	0	7	0
Solyc01g108580.2.1	6	87	2	38	0	100	60	145	7	74	9	50	0
Solyc01g108600.2.1	23	694	172	501	20	1371	464	1208	59	194	106	211	6
Solyc01g108610.2.1	0	0	0	0	0	0	0	0	0	0	0	0	0
Solyc01g108620.2.1	2	37	0	18	5	31	6	38	0	33	10	17	0
Solyc01g108630.2.1	10	319	83	153	0	31	11	39	0	9	8	42	1
Solyc01g108640.1.1	0	0	0	0	0	0	0	0	0	0	0	0	0
Solyc01g108650.2.1	3	12	1	0	0	23	16	20	0	17	0	24	0
Solyc01g108660.2.1	7	62	8	55	1	91	68	162	8	94	31	95	7
Solyc01g108670.2.1	0	0	0	0	0	0	0	0	0	0	0	0	0
Solyc01g108710.2.1	0	0	0	0	0	0	0	0	0	0	0	0	0
Solyc01g108750.2.1	0	0	0	0	0	0	0	0	0	0	0	0	0
Solyc01g108780.2.1	1	3	3	0	0	0	1	0	0	4	41	6	0
Solyc01g108790.1.1	0	0	0	0	0	0	0	0	0	0	0	0	0
Solyc01g108800.2.1	1	2	0	3	0	19	56	49	0	0	0	0	0
Solyc01g108840.2.1	15	152	13	68	12	99	49	166	8	116	42	79	0
Solyc01g108860.2.1	0	0	0	0	0	0	0	0	0	0	0	0	0
Solyc01g108880.2.1	10	8	1	5	0	365	13	128	2	2	59	1	0
Solyc01g108890.2.1	12	61	3	79	14	93	106	146	13	153	29	159	1
Solyc01g108900.2.1	0	0	0	0	0	0	0	0	0	0	0	0	0
Solyc01g108910.2.1	6	406	6	111	3	169	733	553	18	461	9	261	9
Solyc01g108920.2.1	7	15	4	20	2	40	38	58	1	27	6	30	0
Solyc01g108930.2.1	0	0	0	0	0	0	0	0	0	0	0	0	0
Solyc01g108960.1.1	0	0	0	0	0	0	0	0	0	0	0	0	0
Solyc01g108970.2.1	0	0	0	0	0	0	0	0	0	0	0	0	0
Solyc01g109040.2.1	4	877	54	507	1	2	44	32	1	71	19	106	3
Solyc01g109050.2.1	0	0	0	0	0	0	0	0	0	0	0	0	0
Solyc01g109060.2.1	0	0	0	0	0	0	0	0	0	0	0	0	0
Solyc01g109070.2.1	4	29	2	24	0	40	50	37	11	34	24	38	0
Solyc01g109080.2.1	10	17	2	21	0	62	76	95	4	59	8	33	0
Solyc01g109090.2.1	5	918	14	573	6	1014	417	1354	23	1091	42	512	0

Solyc01g109100.2.1	2	22	2	21	1	12	24	6	0	24	0	28	0
Solyc01g109110.2.1	13	121	49	139	2	42	73	76	19	29	23	78	0
Solyc01g109120.2.1	9	18	8	5	4	554	85	93	19	0	0	0	0
Solyc01g109130.2.1	3	15	1	0	0	31	47	31	6	21	17	11	0
Solyc01g109170.2.1	10	96	5	61	11	801	369	580	31	175	63	188	1
Solyc01g109180.2.1	10	39	7	2	11	5	15	2	17	173	39	35	48
Solyc01g109200.2.1	3	55	1	47	4	3	17	13	3	30	4	39	2
Solyc01g109210.2.1	0	0	0	0	0	0	0	0	0	0	0	0	0
Solyc01g109220.2.1	1	8	0	0	0	4	8	8	0	12	2	17	0
Solyc01g109230.2.1	0	0	0	0	0	0	0	0	0	0	0	0	0
Solyc01g109250.2.1	5	107	34	85	1	38	60	56	0	9	0	26	0
Solyc01g109260.2.1	9	89	7	63	2	92	21	119	3	85	41	57	0
Solyc01g109270.2.1	0	0	0	0	0	0	0	0	0	0	0	0	0
Solyc01g109280.2.1	5	21	3	8	2	50	28	47	3	21	5	29	0
Solyc01g109290.2.1	1	5	2	4	0	9	2	11	0	5	0	3	0
Solyc01g109300.2.1	8	1576	137	768	3	1971	901	1587	62	792	158	439	13
Solyc01g109310.2.1	0	0	0	0	0	0	0	0	0	0	0	0	0
Solyc01g109320.2.1	0	0	0	0	0	0	0	0	0	0	0	0	0
Solyc01g109330.2.1	2	15	0	5	0	28	5	40	0	5	1	7	0
Solyc01g109340.2.1	3	50	6	45	10	33	86	49	14	368	45	241	11
Solyc01g109350.2.1	25	174	30	113	13	605	308	798	81	526	107	401	3
Solyc01g109360.2.1	9	107	13	54	9	184	249	239	40	480	113	334	23
Solyc01g109370.2.1	1	0	0	0	0	4	3	2	0	14	3	10	1
Solyc01g109380.2.1	1	2	0	10	0	0	0	2	0	3	0	6	0
Solyc01g109390.2.1	0	0	0	0	0	0	0	0	0	0	0	0	0
Solyc01g109400.2.1	3	38	1	14	1	52	19	68	13	15	5	51	0
Solyc01g109410.2.1	10	137	7	28	6	301	106	332	21	297	53	228	0
Solyc01g109420.2.1	1	6	1	0	0	5	1	7	1	0	6	1	1
Solyc01g109430.2.1	0	0	0	0	0	0	0	0	0	0	0	0	0
Solyc01g109440.2.1	0	0	0	0	0	0	0	0	0	0	0	0	0
Solyc01g109450.2.1	0	0	0	0	0	0	0	0	0	0	0	0	0
Solyc01g109460.2.1	9	288	18	186	15	343	37	425	35	62	26	33	3
Solyc01g109470.2.1	0	0	0	0	0	0	0	0	0	0	0	0	0
Solyc01g109480.2.1	0	0	0	0	0	0	0	0	0	0	0	0	0
Solyc01g109500.2.1	0	0	0	0	0	0	0	0	0	0	0	0	0
Solyc01g109510.2.1	13	110	19	104	16	220	171	376	35	216	42	119	3
Solyc01g109520.2.1	5	165	10	104	11	317	250	265	30	269	77	195	5
Solyc01g109530.2.1	0	0	0	0	0	0	0	0	0	0	0	0	0
Solyc01g109540.2.1	5	76	3	47	12	263	86	232	20	201	52	205	0
Solyc01g109550.2.1	0	0	0	0	0	0	0	0	0	0	0	0	0
Solyc01g109560.2.1	5	64	11	19	3	93	61	140	15	74	27	65	3

Solyc01g109570.2.1	10	262	13	133	20	175	95	558	50	448	48	256	15
Solyc01g109580.2.1	0	0	0	0	0	0	0	0	0	0	0	0	0
Solyc01g109590.2.1	9	37	3	9	2	44	60	53	11	210	28	150	0
Solyc01g109600.2.1	6	100	13	97	1	182	416	365	46	785	60	388	8
Solyc01g109610.2.1	0	0	0	0	0	0	0	0	0	0	0	0	0
Solyc01g109620.2.1	3	51	12	33	21	103	82	102	20	105	19	132	3
Solyc01g109630.2.1	10	21	1	17	0	13	77	32	0	27	6	34	0
Solyc01g109640.2.1	1	2	1	0	3	5	8	0	0	0	0	3	0
Solyc01g109650.2.1	8	44	2	23	3	16	30	21	12	35	23	23	0
Solyc01g109660.2.1	7	2005	112	884	42	1492	772	2419	231	10885	892	5850	60
Solyc01g109680.2.1	1	5	0	6	7	0	11	4	0	3	6	9	0
Solyc01g109690.1.1	0	0	0	0	0	0	0	0	0	0	0	0	0
Solyc01g109700.2.1	1	15	5	2	0	2	0	4	0	2	1	3	0
Solyc01g109710.2.1	2	32	0	25	0	69	35	62	6	41	52	22	1
Solyc01g109720.2.1	1	0	0	0	0	3	0	0	3	0	4	0	2
Solyc01g109750.2.1	0	0	0	0	0	0	0	0	0	0	0	0	0
Solyc01g109760.2.1	1	0	0	0	0	5	2	4	2	2	2	8	0
Solyc01g109770.2.1	4	132	20	82	9	272	119	227	16	248	128	194	12
Solyc01g109780.2.1	12	101	6	35	0	104	55	125	10	86	24	100	4
Solyc01g109790.2.1	5	49	11	50	2	198	20	141	11	71	46	70	3
Solyc01g109800.2.1	9	6	0	7	0	73	77	235	4	48	1	20	0
Solyc01g109810.2.1	4	16	0	15	0	102	31	265	0	8	0	12	0
Solyc01g109830.2.1	2	6	0	1	0	8	9	18	2	4	6	2	3
Solyc01g109850.2.1	12	190	21	179	16	130	233	249	19	599	165	525	18
Solyc01g109860.2.1	1	2	4	3	0	4	14	1	0	0	3	0	0
Solyc01g109870.2.1	9	340	29	228	19	1676	660	1675	174	607	94	325	3
Solyc01g109880.2.1	4	49	10	6	1	376	175	325	22	254	83	256	4
Solyc01g109890.2.1	1	32	6	21	1	71	34	31	4	55	5	27	4
Solyc01g109900.2.1	0	0	0	0	0	0	0	0	0	0	0	0	0
Solyc01g109910.2.1	19	117	15	105	11	658	96	474	25	102	39	93	8
Solyc01g109920.2.1	1	0	0	0	0	16	0	10	0	0	13	1	0
Solyc01g109930.2.1	2	3	2	1	0	13	4	13	0	19	2	6	0
Solyc01g109940.2.1	12	366	24	161	23	847	292	774	110	703	170	497	15
Solyc01g109950.2.1	0	0	0	0	0	0	0	0	0	0	0	0	0
Solyc01g109960.2.1	2	8	1	7	0	22	8	20	1	12	3	17	0
Solyc01g109970.2.1	3	16	0	1	0	18	28	18	0	24	1	11	1
Solyc01g109980.2.1	17	43	7	42	12	106	181	178	60	280	44	178	7
Solyc01g109990.2.1	10	28	2	11	0	100	59	93	21	37	13	53	0
Solyc01g110000.2.1	13	59	25	25	16	108	1916	416	50	337	37	202	2
Solyc01g110010.2.1	1	1	0	1	0	2	4	2	0	2	0	1	0
Solyc01g110020.2.1	3	40	9	9	3	55	61	104	15	81	9	22	0

Solyc01g110030.2.1	2	7	1	2	0	10	8	8	0	17	3	3	0
Solyc01g110040.2.1	2	9	0	47	1	163	9	29	7	24	0	44	0
Solyc01g110060.2.1	4	20	9	6	6	27	26	48	2	11	10	8	3
Solyc01g110110.2.1	6	92	2	62	0	13	157	59	0	13	37	9	0
Solyc01g110120.2.1	13	288	28	108	12	582	434	618	44	590	170	458	19
Solyc01g110130.2.1	4	8	4	6	0	8	2	7	0	27	44	44	0
Solyc01g110150.2.1	2	53	0	38	0	71	9	59	4	23	14	4	0
Solyc01g110160.2.1	0	0	0	0	0	0	0	0	0	0	0	0	0
Solyc01g110180.2.1	0	0	0	0	0	0	0	0	0	0	0	0	0
Solyc01g110190.2.1	0	0	0	0	0	0	0	0	0	0	0	0	0
Solyc01g110270.2.1	5	100	24	69	6	20	199	72	7	25	9	60	3
Solyc01g110280.2.1	3	36	8	14	0	13	6	28	0	5	4	5	0
Solyc01g110290.2.1	11	790	42	276	6	317	66	367	12	289	55	182	5
Solyc01g110300.2.1	4	59	2	22	1	36	37	53	10	58	9	51	0
Solyc01g110310.2.1	6	93	29	48	5	44	96	66	3	34	13	35	0
Solyc01g110340.2.1	1	14	0	15	32	0	5	4	0	4	1	0	0
Solyc01g110350.2.1	2	1	2	6	0	5	9	13	3	18	10	14	2
Solyc01g110360.2.1	11	3385	831	1515	6	61	568	102	2	787	583	1698	6
Solyc01g110370.2.1	1	6	2	0	0	6	6	2	2	8	2	10	0
Solyc01g110380.2.1	13	33	4	17	2	129	96	169	22	79	21	47	2
Solyc01g110390.2.1	2	66	2	18	0	66	60	79	10	157	29	109	1
Solyc01g110400.2.1	0	0	0	0	0	0	0	0	0	0	0	0	0
Solyc01g110410.2.1	3	55	3	23	0	183	17	114	6	68	9	54	0
Solyc01g110430.2.1	1	47	5	31	0	53	8	98	2	20	7	11	0
Solyc01g110440.2.1	14	6023	206	2023	37	12859	205	6446	62	6425	344	1935	8
Solyc01g110450.2.1	7	58	9	75	3	95	111	120	21	344	114	612	9
Solyc01g110460.2.1	7	17	3	12	0	48	54	69	1	39	7	35	1
Solyc01g110470.2.1	2	9	0	0	0	12	8	13	1	13	8	1	0
Solyc01g110480.2.1	8	438	64	272	37	299	136	274	58	240	73	179	7
Solyc01g110490.2.1	9	21	3	21	8	58	45	49	7	39	17	26	2
Solyc01g110500.2.1	0	0	0	0	0	0	0	0	0	0	0	0	0
Solyc01g110510.2.1	0	0	0	0	0	0	0	0	0	0	0	0	0
Solyc01g110520.2.1	12	283	12	157	7	121	37	144	17	220	42	240	6
Solyc01g110530.2.1	1	40	8	23	5	0	23	18	0	37	22	44	2
Solyc01g110540.2.1	0	0	0	0	0	0	0	0	0	0	0	0	0
Solyc01g110550.2.1	3	9	3	3	0	0	22	7	2	6	4	6	1
Solyc01g110560.2.1	0	0	0	0	0	0	0	0	0	0	0	0	0
Solyc01g110570.2.1	0	0	0	0	0	0	0	0	0	0	0	0	0
Solyc01g110660.2.1	0	0	0	0	0	0	0	0	0	0	0	0	0
Solyc01g110680.2.1	0	0	0	0	0	0	0	0	0	0	0	0	0
Solyc01g110690.2.1	0	0	0	0	0	0	0	0	0	0	0	0	0

Solyc01g110700.2.1	0	0	0	0	0	0	0	0	0	0	0	0	0
Solyc01g110760.1.1	0	0	0	0	0	0	0	0	0	0	0	0	0
Solyc01g110800.2.1	0	0	0	0	0	0	0	0	0	0	0	0	0
Solyc01g110810.2.1	3	4	9	2	1	8	52	7	2	10	14	14	3
Solyc01g110820.2.1	1	3	0	0	0	5	1	4	0	2	3	0	0
Solyc01g110860.1.1	0	0	0	0	0	0	0	0	0	0	0	0	0
Solyc01g110880.1.1	0	0	0	0	0	0	0	0	0	0	0	0	0
Solyc01g110920.2.1	0	0	0	0	0	0	0	0	0	0	0	0	0
Solyc01g110940.2.1	0	0	0	0	0	0	0	0	0	0	0	0	0
Solyc01g110950.2.1	0	0	0	0	0	0	0	0	0	0	0	0	0
Solyc01g110960.2.1	1	0	0	0	0	1	2	3	0	3	3	2	0
Solyc01g110990.2.1	7	74	14	28	2	271	155	216	26	136	42	87	4
Solyc01g111000.2.1	0	0	0	0	0	0	0	0	0	0	0	0	0
Solyc01g111010.2.1	0	0	0	0	0	0	0	0	0	0	0	0	0
Solyc01g111020.2.1	22	321	23	186	0	247	277	340	19	151	52	122	4
Solyc01g111030.1.1	0	0	0	0	0	0	0	0	0	0	0	0	0
Solyc01g111040.2.1	8	59	4	31	1	233	45	148	13	87	84	66	0
Solyc01g111060.2.1	8	40	4	25	4	215	170	153	39	144	36	90	8
Solyc01g111070.2.1	0	0	0	0	0	0	0	0	0	0	0	0	0
Solyc01g111080.2.1	3	1137	35	364	2	616	52	191	4	75	3	102	0
Solyc01g111090.2.1	2	35	0	21	0	70	9	45	2	49	1	14	0
Solyc01g111100.2.1	0	0	0	0	0	0	0	0	0	0	0	0	0
Solyc01g111110.2.1	2	35	1	12	0	7	1	17	0	32	6	30	0
Solyc01g111120.2.1	8	327	52	155	9	301	162	305	22	362	60	300	2
Solyc01g111130.2.1	3	11	1	6	0	22	22	24	3	23	3	16	0
Solyc01g111140.2.1	2	10	1	4	1	2	15	4	3	11	5	6	1
Solyc01g111150.2.1	7	216	20	121	4	660	191	788	63	559	67	489	7
Solyc01g111160.2.1	5	527	33	53	104	52	78	25	78	1994	22	688	9
Solyc01g111170.2.1	5	7619	637	1990	299	7611	5340	5689	887	8967	1435	3299	122
Solyc01g111180.2.1	3	606	59	237	20	597	293	604	59	575	111	514	7
Solyc01g111190.2.1	0	0	0	0	0	0	0	0	0	0	0	0	0
Solyc01g111200.2.1	6	109	6	44	0	292	80	239	39	170	45	215	2
Solyc01g111210.2.1	14	30	2	4	8	179	584	106	1031	25	5	16	2
Solyc01g111220.2.1	0	0	0	0	0	0	0	0	0	0	0	0	0
Solyc01g111230.2.1	4	0	2	4	0	0	41	4	9	29	3	138	1
Solyc01g111240.2.1	31	252	23	246	4	358	295	715	34	475	102	342	16
Solyc01g111250.2.1	4	0	6	0	2	27	9	18	13	5	24	1	1
Solyc01g111260.2.1	9	381	35	156	28	201	157	212	51	424	57	328	12
Solyc01g111270.2.1	2	0	0	0	0	0	0	1	0	33	13	42	0
Solyc01g111280.2.1	2	15	0	21	0	3	9	0	0	121	9	131	1
Solyc01g111300.2.1	5	496	32	225	4	751	178	861	23	1700	172	974	10

Solyc01g111310.2.1	0	0	0	0	0	0	0	0	0	0	0	0	0
Solyc01g111320.2.1	3	68	0	41	3	59	21	227	2	136	16	104	0
Solyc01g111330.2.1	2	7	2	4	0	0	7	1	2	6	3	1	0
Solyc01g111340.2.1	1	3	0	0	0	0	5	0	0	6	0	4	0
Solyc01g111350.2.1	12	85	10	52	2	64	163	76	21	963	242	606	4
Solyc01g111360.2.1	4	89	7	43	1	75	23	100	4	142	34	84	0
Solyc01g111370.2.1	10	66	13	81	3	111	77	131	11	219	18	137	0
Solyc01g111380.2.1	2	15	2	22	0	31	56	53	2	44	4	31	0
Solyc01g111400.2.1	21	143	22	128	19	530	321	1020	109	577	125	396	7
Solyc01g111410.2.1	5	13	1	5	2	7	39	21	10	27	17	13	1
Solyc01g111420.2.1	4	11	0	6	1	29	11	30	5	31	30	42	0
Solyc01g111430.2.1	0	0	0	0	0	0	0	0	0	0	0	0	0
Solyc01g111440.2.1	10	260	37	136	30	911	138	340	154	205	34	162	5
Solyc01g111450.2.1	1	25	1	3	0	50	13	45	1	44	8	45	0
Solyc01g111460.2.1	0	0	0	0	0	0	0	0	0	0	0	0	0
Solyc01g111470.2.1	3	8	0	0	0	6	13	9	2	28	15	8	0
Solyc01g111500.2.1	0	0	0	0	0	0	0	0	0	0	0	0	0
Solyc01g111510.2.1	6	427	66	210	8	656	492	446	28	420	215	376	5
Solyc01g111520.2.1	12	338	31	192	14	527	460	836	60	687	115	508	14
Solyc01g111530.2.1	30	186	14	180	27	705	480	802	97	387	95	327	5
Solyc01g111550.2.1	1	7	0	4	2	0	0	0	0	14	5	12	0
Solyc01g111560.2.1	2	40	2	11	3	24	39	14	2	74	20	70	0
Solyc01g111570.2.1	11	27	2	10	0	72	14	51	0	482	166	345	3
Solyc01g111580.2.1	0	0	0	0	0	0	0	0	0	0	0	0	0
Solyc01g111590.2.1	2	3	2	1	0	5	16	6	4	3	7	0	4
Solyc01g111600.2.1	8	301	21	205	7	175	183	223	16	1064	159	745	28
Solyc01g111610.2.1	0	0	0	0	0	0	0	0	0	0	0	0	0
Solyc01g111620.2.1	5	24	1	21	1	39	12	58	5	47	15	25	0
Solyc01g111630.2.1	10	1071	158	1012	20	151	378	208	7	370	119	543	8
Solyc01g111640.2.1	2	17	3	4	4	8	5	97	6	6	16	7	1
Solyc01g111650.2.1	2	148	4	45	3	206	111	176	17	153	12	122	0
Solyc01g111660.2.1	2	103	14	49	3	20	16	33	0	20	5	16	2
Solyc01g111670.2.1	0	0	0	0	0	0	0	0	0	0	0	0	0
Solyc01g111680.2.1	7	23	0	7	0	56	33	30	3	57	16	30	2
Solyc01g111690.2.1	3	24	4	4	3	12	29	23	2	20	5	26	0
Solyc01g111700.1.1	0	0	0	0	0	0	0	0	0	0	0	0	0
Solyc01g111710.2.1	9	164	19	221	26	346	220	436	62	293	104	332	9
Solyc01g111720.2.1	10	78	5	43	2	202	94	210	18	103	24	55	3
Solyc01g111730.2.1	7	134	9	70	3	65	148	142	11	140	63	129	5
Solyc01g111740.2.1	0	0	0	0	0	0	0	0	0	0	0	0	0
Solyc01g111750.2.1	7	287	37	195	9	293	536	373	6	44	66	63	1

Solyc01g111760.2.1	6	200	33	139	15	635	657	822	47	427	179	276	17
Solyc01g111770.2.1	8	27	2	17	1	64	22	56	6	66	7	61	0
Solyc01g111780.2.1	12	124	8	95	10	276	129	347	24	509	48	490	21
Solyc01g111800.2.1	2	3	0	1	4	2	10	4	1	4	2	1	1
Solyc01g111810.2.1	0	0	0	0	0	0	0	0	0	0	0	0	0
Solyc01g111820.2.1	0	0	0	0	0	0	0	0	0	0	0	0	0
Solyc01g111830.2.1	8	503	25	186	14	24	81	37	3	755	100	454	3
Solyc01g111840.2.1	0	0	0	0	0	0	0	0	0	0	0	0	0
Solyc01g111860.2.1	2	15	1	3	1	9	9	22	3	20	0	17	0
Solyc01g111870.1.1	0	0	0	0	0	0	0	0	0	0	0	0	0
Solyc01g111880.2.1	2	4	4	1	0	0	93	4	4	5	0	7	2
Solyc01g111890.2.1	9	51	23	34	7	48	89	98	18	81	19	86	2
Solyc01g111910.2.1	18	65	3	34	15	126	120	230	13	176	27	82	0
Solyc01g111920.2.1	5	37	6	54	8	42	41	42	0	84	9	109	1
Solyc01g111930.2.1	9	59	4	35	6	6	17	36	4	94	19	56	0
Solyc01g111940.2.1	3	79	0	34	3	137	65	154	23	187	30	111	2
Solyc01g111950.2.1	0	0	0	0	0	0	0	0	0	0	0	0	0
Solyc01g111960.2.1	0	0	0	0	0	0	0	0	0	0	0	0	0
Solyc01g111970.2.1	0	0	0	0	0	0	0	0	0	0	0	0	0
Solyc01g111980.2.1	19	2353	153	2200	69	1182	59	530	51	140	6	437	18
Solyc01g111990.2.1	10	93	38	82	10	35	152	143	15	45	29	43	6
Solyc01g112000.2.1	3	168	8	48	3	79	22	51	0	29	7	36	0
Solyc01g112010.2.1	0	0	0	0	0	0	0	0	0	0	0	0	0
Solyc01g112020.2.1	7	66	5	92	2	723	42	429	13	159	24	176	1
Solyc01g112040.2.1	2	15	1	8	0	32	13	21	1	14	1	13	0
Solyc01g112050.2.1	12	62	4	42	0	179	57	143	15	113	27	64	0
Solyc01g112060.2.1	4	150	28	92	0	19	61	14	0	41	29	92	0
Solyc01g112070.2.1	0	0	0	0	0	0	0	0	0	0	0	0	0
Solyc01g112080.2.1	9	59	19	45	28	164	71	84	40	76	135	44	11
Solyc01g112090.2.1	1	1	0	0	0	10	11	2	0	2	0	3	0
Solyc01g112100.2.1	16	77	10	89	0	510	166	616	14	587	114	648	24
Solyc01g112120.2.1	2	0	0	0	0	11	0	1	1	47	6	42	0
Solyc01g112130.2.1	0	0	0	0	0	0	0	0	0	0	0	0	0
Solyc01g112140.2.1	3	1	0	2	0	3	0	7	0	9	8	2	0
Solyc01g112150.2.1	12	132	9	112	1	252	113	333	28	271	87	181	9
Solyc01g112160.2.1	2	21	2	18	7	20	5	23	0	26	1	9	0
Solyc01g112170.2.1	2	14	2	15	1	35	111	28	13	43	6	15	1
Solyc01g112180.2.1	3	27	1	37	0	54	16	82	4	55	16	65	0
Solyc01g112190.2.1	0	0	0	0	0	0	0	0	0	0	0	0	0
Solyc01g112200.2.1	0	0	0	0	0	0	0	0	0	0	0	0	0
Solyc01g112210.2.1	4	16	2	18	3	36	24	73	8	78	15	41	0

Solyc01g112220.2.1	3	14	5	2	4	13	17	16	6	10	0	0	1
Solyc01g112230.2.1	1	0	0	0	0	32	9	5	0	0	4	0	0
Solyc01g112240.2.1	7	19	3	35	0	52	26	100	1	58	18	61	3
Solyc01g112250.2.1	6	262	34	179	18	450	558	895	123	1012	78	659	12
Solyc01g112260.2.1	4	334	19	120	22	11	8	15	0	333	19	293	1
Solyc01g112270.1.1	0	0	0	0	0	0	0	0	0	0	0	0	0
Solyc01g112280.2.1	6	81	14	59	0	130	49	247	15	230	40	254	2
Solyc01g112290.2.1	10	168	18	84	9	220	190	319	19	265	58	272	1
Solyc01g112300.2.1	1	3	3	6	0	3	27	11	0	7	0	16	0
Solyc01g112310.2.1	2	6	0	9	0	14	22	9	3	7	5	5	0
Solyc01g112320.2.1	5	21	1	27	0	84	31	99	3	40	15	39	0
Solyc01g112340.2.1	0	0	0	0	0	0	0	0	0	0	0	0	0
Solyc01g112350.2.1	9	46	3	33	0	107	60	147	18	82	29	63	1
Solyc01g112370.2.1	0	0	0	0	0	0	0	0	0	0	0	0	0
Solyc01g113620.1.1	0	0	0	0	0	0	0	0	0	0	0	0	0
Solyc02g005110.2.1	0	0	0	0	0	0	0	0	0	0	0	0	0
Solyc02g005180.2.1	0	0	0	0	0	0	0	0	0	0	0	0	0
Solyc02g005190.1.1	0	0	0	0	0	0	0	0	0	0	0	0	0
Solyc02g005200.2.1	9	113	19	100	0	390	251	258	3	202	79	288	2
Solyc02g005210.2.1	7	138	7	91	1	121	113	112	5	70	10	64	5
Solyc02g005280.1.1	0	0	0	0	0	0	0	0	0	0	0	0	0
Solyc02g005290.2.1	2	17	2	7	1	2	13	9	0	12	3	9	0
Solyc02g005350.2.1	5	53	6	35	0	86	87	82	23	125	20	84	2
Solyc02g005480.2.1	5	89	5	72	2	121	35	192	7	241	11	151	0
Solyc02g005510.2.1	2	3	0	3	0	9	6	37	0	4	0	3	0
Solyc02g005520.2.1	0	0	0	0	0	0	0	0	0	0	0	0	0
Solyc02g011680.2.1	0	0	0	0	0	0	0	0	0	0	0	0	0
Solyc02g011880.1.1	0	0	0	0	0	0	0	0	0	0	0	0	0
Solyc02g011990.1.1	0	0	0	0	0	0	0	0	0	0	0	0	0
Solyc02g014030.1.1	0	0	0	0	0	0	0	0	0	0	0	0	0
Solyc02g014040.1.1	0	0	0	0	0	0	0	0	0	0	0	0	0
Solyc02g014070.1.1	0	0	0	0	0	0	0	0	0	0	0	0	0
Solyc02g014090.2.1	7	49	4	41	19	102	62	67	13	55	18	64	0
Solyc02g014120.1.1	0	0	0	0	0	0	0	0	0	0	0	0	0
Solyc02g014130.1.1	16	63	7	56	7	153	117	199	23	91	49	78	3
Solyc02g014150.2.1	9	388	39	209	0	65	263	166	1	133	70	165	2
Solyc02g014170.2.1	7	43	4	18	13	162	73	125	5	88	30	42	1
Solyc02g014180.1.1	15	27	2	24	5	86	66	142	6	42	24	45	0
Solyc02g014190.2.1	8	7	2	5	3	62	68	65	14	21	16	38	0
Solyc02g014220.2.1	0	0	0	0	0	0	0	0	0	0	0	0	0
Solyc02g014310.2.1	11	266	20	157	15	683	221	1015	78	796	171	579	3

Solyc02g014350.2.1	7	5	4	13	1	39	51	44	18	17	20	12	2
Solyc02g014360.2.1	1	0	0	0	0	3	7	4	0	5	2	11	0
Solyc02g014380.2.1	6	52	4	26	0	121	52	121	8	100	15	53	0
Solyc02g014430.2.1	0	0	0	0	0	0	0	0	0	0	0	0	0
Solyc02g014450.1.1	2	3	0	4	0	5	9	12	2	18	6	8	0
Solyc02g014460.2.1	2	21	0	7	0	2	3	17	0	33	6	11	0
Solyc02g014470.2.1	0	0	0	0	0	0	0	0	0	0	0	0	0
Solyc02g014490.2.1	0	0	0	0	0	0	0	0	0	0	0	0	0
Solyc02g014500.1.1	1	0	3	4	0	1	10	2	1	7	1	6	0
Solyc02g014520.2.1	3	14	1	5	2	11	24	18	0	30	5	28	0
Solyc02g014560.2.1	0	0	0	0	0	0	0	0	0	0	0	0	0
Solyc02g014670.1.1	0	0	0	0	0	0	0	0	0	0	0	0	0
Solyc02g014720.2.1	4	64	0	50	0	52	16	74	1	354	5	374	2
Solyc02g014730.2.1	0	0	0	0	0	0	0	0	0	0	0	0	0
Solyc02g014770.2.1	3	2	0	3	0	6	13	13	5	17	0	6	0
Solyc02g014830.2.1	0	0	0	0	0	0	0	0	0	0	0	0	0
Solyc02g014860.2.1	11	249	8	100	5	428	1026	1089	29	401	60	330	3
Solyc02g014870.2.1	2	22	0	8	0	40	23	33	6	68	7	59	3
Solyc02g020890.2.1	3	10	3	5	3	17	8	5	1	4	3	3	0
Solyc02g020900.1.1	3	29	4	16	3	36	17	33	4	37	7	22	0
Solyc02g020910.2.1	8	55	17	23	0	56	26	55	5	61	14	46	1
Solyc02g020940.2.1	11	1666	336	853	0	10	173	135	4	201	71	360	9
Solyc02g020980.2.1	13	92	28	98	2	158	381	204	47	143	105	104	2
Solyc02g020990.2.1	0	0	0	0	0	0	0	0	0	0	0	0	0
Solyc02g021000.2.1	2	22	5	3	0	0	29	4	0	4	3	23	0
Solyc02g021020.2.1	5	9	1	11	1	11	19	31	2	10	4	29	0
Solyc02g021050.2.1	2	20	4	9	0	19	15	30	1	52	0	23	0
Solyc02g021060.2.1	0	0	0	0	0	0	0	0	0	0	0	0	0
Solyc02g021100.2.1	3	11	4	8	0	6	11	21	0	16	3	20	0
Solyc02g021140.2.1	2	26	0	18	0	22	11	13	0	17	1	22	0
Solyc02g021220.1.1	3	0	0	0	0	1	0	0	0	33	17	14	0
Solyc02g021230.2.1	0	0	0	0	0	0	0	0	0	0	0	0	0
Solyc02g021240.2.1	6	55	12	63	7	109	166	219	27	110	31	73	1
Solyc02g021260.1.1	0	0	0	0	0	0	0	0	0	0	0	0	0
Solyc02g021270.2.1	0	0	0	0	0	0	0	0	0	0	0	0	0
Solyc02g021280.1.1	0	0	0	0	0	0	0	0	0	0	0	0	0
Solyc02g021340.2.1	2	16	0	1	0	55	10	17	0	18	2	29	1
Solyc02g021350.2.1	0	0	0	0	0	0	0	0	0	0	0	0	0
Solyc02g021360.2.1	2	1	0	0	0	8	7	4	1	12	2	8	2
Solyc02g021400.1.1	1	118	10	37	0	275	66	77	12	303	34	120	7
Solyc02g021410.2.1	0	0	0	0	0	0	0	0	0	0	0	0	0

Solyc02g021420.2.1	11	109	16	82	7	249	103	204	23	269	47	151	5
Solyc02g021430.1.1	3	23	6	14	2	4	16	0	3	8	13	0	0
Solyc02g021440.2.1	8	84	16	51	1	302	110	321	21	323	77	386	1
Solyc02g021470.2.1	13	144	20	136	10	247	173	317	39	528	40	369	2
Solyc02g021480.2.1	4	19	5	11	0	30	42	13	8	32	12	47	2
Solyc02g021490.1.1	5	3	2	12	0	19	21	21	2	15	17	4	0
Solyc02g021550.2.1	3	41	0	31	5	89	30	97	15	70	20	31	0
Solyc02g021560.2.1	9	306	20	276	23	2157	274	1002	65	394	74	463	11
Solyc02g021590.2.1	8	64	3	43	5	45	45	38	3	82	4	136	7
Solyc02g021600.1.1	4	23	2	7	0	34	36	23	3	15	4	10	0
Solyc02g021620.2.1	6	8	1	4	2	23	19	15	0	53	11	49	0
Solyc02g021640.2.1	10	149	4	65	2	50	28	103	6	99	20	79	0
Solyc02g021650.2.1	10	103	8	60	10	271	86	225	11	207	45	153	6
Solyc02g021680.2.1	0	0	0	0	0	0	0	0	0	0	0	0	0
Solyc02g021690.2.1	2	54	1	8	0	20	44	41	2	462	4	158	0
Solyc02g021700.2.1	1	5	1	0	0	11	5	4	0	2	1	2	1
Solyc02g021760.2.1	3	8	1	1	0	25	10	15	0	6	7	12	0
Solyc02g021770.1.1	0	0	0	0	0	0	0	0	0	0	0	0	0
Solyc02g022830.2.1	1	4	0	0	0	2	3	6	0	0	1	7	0
Solyc02g022850.1.1	10	72	14	26	6	23	112	85	28	50	24	45	2
Solyc02g022870.2.1	6	12	1	6	1	17	19	24	1	11	14	22	0
Solyc02g022920.1.1	5	11	3	7	0	96	45	52	15	36	6	8	0
Solyc02g022930.2.1	3	10	3	9	0	53	21	48	2	25	9	33	1
Solyc02g023950.2.1	1	4	0	0	1	0	8	7	0	10	0	1	0
Solyc02g023970.2.1	2	33	4	21	0	143	53	120	9	119	33	69	0
Solyc02g023980.2.1	5	16	1	22	0	28	23	32	2	51	6	25	1
Solyc02g023990.2.1	1	4	0	0	0	3	20	4	0	2	0	4	0
Solyc02g024000.2.1	5	26	1	18	0	47	12	61	2	26	6	25	0
Solyc02g024010.1.1	1	3	0	0	0	1	0	6	0	0	0	2	0
Solyc02g024020.2.1	6	12	3	6	2	79	57	71	4	104	23	53	0
Solyc02g024050.2.1	7	125	3	62	8	185	44	258	14	79	32	43	0
Solyc02g024070.2.1	3	13	5	8	3	15	22	9	7	2	1	3	1
Solyc02g027080.1.1	2	7	0	5	0	2	8	11	0	11	0	2	0
Solyc02g030080.2.1	7	9	4	5	5	51	34	50	8	85	34	89	0
Solyc02g030100.2.1	1	2	0	1	0	7	4	12	0	8	1	6	0
Solyc02g030110.2.1	4	24	0	6	11	13	31	26	1	20	8	28	0
Solyc02g030120.1.1	0	0	0	0	0	0	0	0	0	0	0	0	0
Solyc02g030130.2.1	0	0	0	0	0	0	0	0	0	0	0	0	0
Solyc02g030170.2.1	9	1445	82	900	44	277	414	694	28	3572	509	1801	34
Solyc02g030200.1.1	0	0	0	0	0	0	0	0	0	0	0	0	0
Solyc02g030210.2.1	1	6	1	5	3	9	7	12	0	2	9	5	0

Solyc02g030230.2.1	2	2	0	0	0	0	12	7	2	14	1	8	0
Solyc02g030250.2.1	4	45	14	31	3	92	141	123	44	139	27	122	26
Solyc02g030300.2.1	6	26	5	53	0	35	50	61	0	43	2	97	0
Solyc02g030450.2.1	3	5	3	0	0	36	19	16	5	44	7	26	0
Solyc02g030460.2.1	0	0	0	0	0	0	0	0	0	0	0	0	0
Solyc02g030480.2.1	0	0	0	0	0	0	0	0	0	0	0	0	0
Solyc02g031710.2.1	5	24	3	12	0	51	52	53	13	18	18	15	0
Solyc02g031740.2.1	6	175	8	120	0	152	145	105	0	71	6	76	0
Solyc02g031750.2.1	0	0	0	0	0	0	0	0	0	0	0	0	0
Solyc02g031770.2.1	3	17	1	15	0	23	23	22	3	34	2	8	0
Solyc02g031780.1.1	0	0	0	0	0	0	0	0	0	0	0	0	0
Solyc02g031790.1.1	0	0	0	0	0	0	0	0	0	0	0	0	0
Solyc02g031830.1.1	3	39	3	22	1	27	20	20	6	59	20	48	2
Solyc02g031840.2.1	9	83	13	55	13	198	267	286	18	189	192	171	6
Solyc02g031850.1.1	0	0	0	0	0	0	0	0	0	0	0	0	0
Solyc02g031860.2.1	11	120	39	102	28	288	316	541	55	356	100	350	12
Solyc02g031900.1.1	2	3	0	4	0	11	5	13	0	9	0	4	0
Solyc02g031910.2.1	9	74	20	69	7	178	305	255	31	200	73	205	2
Solyc02g031920.2.1	0	0	0	0	0	0	0	0	0	0	0	0	0
Solyc02g031940.1.1	0	0	0	0	0	0	0	0	0	0	0	0	0
Solyc02g031950.2.1	2	0	0	0	0	5	0	0	0	1	7	7	0
Solyc02g031960.2.1	0	0	0	0	0	0	0	0	0	0	0	0	0
Solyc02g031970.1.1	6	93	1	23	0	230	63	249	12	121	50	130	1
Solyc02g031980.2.1	3	2	1	2	0	2	3	0	1	0	0	2	0
Solyc02g031990.1.1	0	0	0	0	0	0	0	0	0	0	0	0	0
Solyc02g032100.2.1	6	8	1	4	0	143	32	131	2	3	4	1	0
Solyc02g032120.2.1	1	16	1	5	0	8	9	31	0	6	2	9	0
Solyc02g032200.2.1	13	72	12	38	13	178	136	191	17	93	28	69	9
Solyc02g032300.2.1	5	20	3	5	2	30	29	37	6	49	15	33	4
Solyc02g032330.2.1	2	9	2	1	0	0	41	18	3	23	6	1	0
Solyc02g032400.1.1	0	0	0	0	0	0	0	0	0	0	0	0	0
Solyc02g032450.2.1	11	30	2	32	0	283	60	172	11	174	23	143	1
Solyc02g032460.1.1	0	0	0	0	0	0	0	0	0	0	0	0	0
Solyc02g032470.2.1	0	0	0	0	0	0	0	0	0	0	0	0	0
Solyc02g032480.1.1	0	0	0	0	0	0	0	0	0	0	0	0	0
Solyc02g032490.2.1	0	0	0	0	0	0	0	0	0	0	0	0	0
Solyc02g032510.2.1	9	34	5	37	0	74	90	132	16	71	27	67	5
Solyc02g032640.1.1	0	0	0	0	0	0	0	0	0	0	0	0	0
Solyc02g032650.2.1	0	0	0	0	0	0	0	0	0	0	0	0	0
Solyc02g032660.2.1	3	24	11	23	0	35	24	64	0	6	0	1	0
Solyc02g032800.1.1	2	20	2	57	3	9	0	37	0	366	15	128	4

Solyc02g032820.2.1	0	0	0	0	0	0	0	0	0	0	0	0	0
Solyc02g032830.1.1	0	0	0	0	0	0	0	0	0	0	0	0	0
Solyc02g032840.1.1	0	0	0	0	0	0	0	0	0	0	0	0	0
Solyc02g032850.2.1	10	74	15	85	7	260	85	235	14	59	7	26	3
Solyc02g032860.2.1	15	216	22	87	2	158	53	147	8	67	101	30	0
Solyc02g032870.2.1	0	0	0	0	0	0	0	0	0	0	0	0	0
Solyc02g032910.1.1	0	0	0	0	0	0	0	0	0	0	0	0	0
Solyc02g032920.2.1	1	5	0	6	0	19	11	2	0	5	10	6	0
Solyc02g032930.2.1	4	58	6	28	2	182	65	193	14	206	98	141	7
Solyc02g032940.2.1	3	10	2	0	0	29	15	31	1	13	26	10	0
Solyc02g032950.2.1	0	0	0	0	0	0	0	0	0	0	0	0	0
Solyc02g032960.2.1	10	53	22	33	4	104	155	241	5	88	26	93	1
Solyc02g032970.2.1	1	2	1	0	0	3	1	3	1	5	4	1	0
Solyc02g033040.2.1	0	0	0	0	0	0	0	0	0	0	0	0	0
Solyc02g033060.2.1	0	0	0	0	0	0	0	0	0	0	0	0	0
Solyc02g036260.2.1	0	0	0	0	0	0	0	0	0	0	0	0	0
Solyc02g036270.2.1	1	0	0	6	1	0	1	6	0	5	4	1	0
Solyc02g036280.2.1	0	0	0	0	0	0	0	0	0	0	0	0	0
Solyc02g036290.2.1	0	0	0	0	0	0	0	0	0	0	0	0	0
Solyc02g036300.2.1	0	0	0	0	0	0	0	0	0	0	0	0	0
Solyc02g036310.2.1	0	0	0	0	0	0	0	0	0	0	0	0	0
Solyc02g036330.2.1	3	12	3	5	0	18	15	18	4	52	7	29	1
Solyc02g036340.2.1	6	37	5	22	3	62	31	89	13	63	15	57	0
Solyc02g036350.2.1	8	781	123	418	101	442	1355	1268	11	418	987	227	5
Solyc02g036360.1.1	11	77	12	66	3	213	104	211	6	51	15	68	3
Solyc02g036370.2.1	10	39	0	6	0	214	57	216	6	94	2	121	0
Solyc02g036380.2.1	1	1	1	4	0	3	3	5	0	5	1	6	0
Solyc02g036430.1.1	0	0	0	0	0	0	0	0	0	0	0	0	0
Solyc02g036450.2.1	7	198	3	86	8	515	67	202	15	432	36	134	3
Solyc02g036460.2.1	6	11	4	16	1	65	24	61	0	16	11	19	0
Solyc02g036470.2.1	14	108	12	63	8	122	151	178	5	170	12	87	1
Solyc02g036480.1.1	4	119	36	183	3	161	22	191	32	7	2	30	0
Solyc02g037500.1.1	1	0	0	0	0	27	15	25	0	8	1	1	0
Solyc02g037510.2.1	7	26	3	14	0	29	43	68	9	57	13	59	0
Solyc02g037530.2.1	13	93	22	168	6	97	32	90	6	363	78	331	12
Solyc02g037550.2.1	0	0	0	0	0	0	0	0	0	0	0	0	0
Solyc02g038690.1.1	0	0	0	0	0	0	0	0	0	0	0	0	0
Solyc02g038740.2.1	4	0	0	0	0	14	17	20	4	6	10	9	0
Solyc02g038750.2.1	1	4	0	1	0	25	3	13	2	8	1	8	0
Solyc02g038760.2.1	5	25	4	8	5	33	19	70	11	91	6	43	0
Solyc02g043830.2.1	4	9	1	1	2	33	15	29	0	28	11	12	1

Solyc02g043860.2.1	10	44	13	61	0	321	181	255	12	237	46	155	12
Solyc02g044030.1.1	0	0	0	0	0	0	0	0	0	0	0	0	0
Solyc02g044050.1.1	0	0	0	0	0	0	0	0	0	0	0	0	0
Solyc02g049070.2.1	8	42	5	46	3	83	65	98	7	47	17	52	2
Solyc02g050140.2.1	2	5	0	2	0	5	9	7	1	7	0	16	0
Solyc02g050180.2.1	0	0	0	0	0	0	0	0	0	0	0	0	0
Solyc02g050200.1.1	0	0	0	0	0	0	0	0	0	0	0	0	0
Solyc02g050210.1.1	0	0	0	0	0	0	0	0	0	0	0	0	0
Solyc02g050240.2.1	5	15	4	34	0	37	31	58	0	53	10	45	0
Solyc02g050260.2.1	10	70	10	41	0	104	275	150	12	115	35	86	1
Solyc02g050270.1.1	0	0	0	0	0	0	0	0	0	0	0	0	0
Solyc02g050280.2.1	6	31	1	34	0	79	36	68	4	36	14	41	3
Solyc02g050290.1.1	0	0	0	0	0	0	0	0	0	0	0	0	0
Solyc02g055370.2.1	0	0	0	0	0	0	0	0	0	0	0	0	0
Solyc02g055440.2.1	4	225	19	125	4	19	127	34	0	90	17	136	4
Solyc02g055450.1.1	1	1	0	3	0	7	5	6	2	21	1	8	0
Solyc02g055460.1.1	3	6	6	7	4	8	45	20	3	27	5	5	0
Solyc02g055470.2.1	0	0	0	0	0	0	0	0	0	0	0	0	0
Solyc02g055550.2.1	0	0	0	0	0	0	0	0	0	0	0	0	0
Solyc02g061760.2.1	1	21	6	6	0	1	1	2	0	7	6	10	0
Solyc02g061770.2.1	0	0	0	0	0	0	0	0	0	0	0	0	0
Solyc02g061780.2.1	2	19	3	57	0	11	0	6	0	4	21	14	0
Solyc02g061790.2.1	2	29	0	14	0	15	3	27	2	47	2	45	0
Solyc02g061800.2.1	0	0	0	0	0	0	0	0	0	0	0	0	0
Solyc02g061810.2.1	4	7	1	2	0	24	19	15	3	41	2	9	1
Solyc02g061820.2.1	6	35	4	22	1	93	132	77	4	32	15	34	3
Solyc02g061840.2.1	1	36	0	10	0	55	12	25	7	15	0	5	0
Solyc02g061850.2.1	0	0	0	0	0	0	0	0	0	0	0	0	0
Solyc02g061940.2.1	0	0	0	0	0	0	0	0	0	0	0	0	0
Solyc02g061950.2.1	1	2	0	0	0	0	8	1	0	3	0	8	0
Solyc02g061960.2.1	12	88	2	60	1	272	92	252	32	218	74	203	5
Solyc02g061980.2.1	11	155	22	170	18	398	202	411	74	185	48	236	6
Solyc02g061990.2.1	0	0	0	0	0	0	0	0	0	0	0	0	0
Solyc02g062000.2.1	11	172	12	147	9	518	127	383	32	263	68	239	0
Solyc02g062010.1.1	0	0	0	0	0	0	0	0	0	0	0	0	0
Solyc02g062020.2.1	0	0	0	0	0	0	0	0	0	0	0	0	0
Solyc02g062030.1.1	0	0	0	0	0	0	0	0	0	0	0	0	0
Solyc02g062040.2.1	2	11	0	7	0	26	5	17	0	32	3	28	0
Solyc02g062050.1.1	0	0	0	0	0	0	0	0	0	0	0	0	0
Solyc02g062100.2.1	3	12	0	2	0	21	12	13	6	16	3	1	0
Solyc02g062110.2.1	12	84	13	90	13	137	146	146	16	136	31	149	4

Solyc02g062130.2.1	8	16	4	15	0	2	28	17	3	44	15	37	0
Solyc02g062140.2.1	11	82	9	54	4	53	68	102	26	272	9	192	3
Solyc02g062160.1.1	0	0	0	0	0	0	0	0	0	0	0	0	0
Solyc02g062170.2.1	0	0	0	0	0	0	0	0	0	0	0	0	0
Solyc02g062180.2.1	2	2	2	8	1	6	7	13	0	34	6	53	0
Solyc02g062230.1.1	0	0	0	0	0	0	0	0	0	0	0	0	0
Solyc02g062240.2.1	4	21	1	12	0	20	6	26	2	43	9	67	0
Solyc02g062250.2.1	3	16	0	4	4	37	11	25	8	16	7	11	3
Solyc02g062270.2.1	7	35	4	22	2	65	61	159	13	37	5	26	0
Solyc02g062280.2.1	0	0	0	0	0	0	0	0	0	0	0	0	0
Solyc02g062290.2.1	16	146	5	100	10	539	153	537	28	135	57	175	0
Solyc02g062330.1.1	0	0	0	0	0	0	0	0	0	0	0	0	0
Solyc02g062340.2.1	6	1316	327	644	11	18	207	33	0	50	119	154	5
Solyc02g062350.2.1	9	56	7	33	6	65	146	149	5	57	19	53	6
Solyc02g062360.2.1	1	1	3	1	6	9	26	15	11	7	2	1	0
Solyc02g062370.2.1	24	116	14	95	7	493	210	279	71	128	34	146	3
Solyc02g062380.1.1	0	0	0	0	0	0	0	0	0	0	0	0	0
Solyc02g062390.2.1	8	1	0	2	0	1537	219	520	12	10	31	1	0
Solyc02g062400.2.1	0	0	0	0	0	0	0	0	0	0	0	0	0
Solyc02g062420.2.1	0	0	0	0	0	0	0	0	0	0	0	0	0
Solyc02g062430.2.1	10	26	1	18	0	62	137	59	3	47	10	60	1
Solyc02g062460.2.1	4	1	0	1	0	53	22	52	18	70	36	70	11
Solyc02g062470.1.1	3	14	0	24	0	1	7	9	18	10	6	3	1
Solyc02g062480.1.1	0	0	0	0	0	0	0	0	0	0	0	0	0
Solyc02g062490.2.1	0	0	0	0	0	0	0	0	0	0	0	0	0
Solyc02g062500.2.1	4	5	2	13	0	133	65	185	68	423	90	322	9
Solyc02g062520.2.1	5	25	1	25	1	32	46	33	3	54	19	37	0
Solyc02g062530.2.1	2	48	0	30	0	144	23	155	4	75	6	66	0
Solyc02g062540.2.1	4	23	1	4	0	45	48	51	3	42	9	27	2
Solyc02g062550.2.1	4	14	20	4	10	27	7	28	0	0	0	1	2
Solyc02g062560.2.1	10	46	6	38	4	58	74	63	10	18	4	5	4
Solyc02g062570.2.1	2	37	2	11	0	63	23	37	0	28	7	20	0
Solyc02g062580.2.1	5	27	19	28	1	57	32	44	1	5	5	9	0
Solyc02g062590.2.1	0	0	0	0	0	0	0	0	0	0	0	0	0
Solyc02g062600.2.1	5	45	4	18	3	110	25	129	11	88	20	56	0
Solyc02g062610.2.1	1	19	0	3	0	2	0	0	0	3	0	2	0
Solyc02g062620.2.1	3	43	4	24	0	195	25	122	1	117	9	102	0
Solyc02g062630.1.1	0	0	0	0	0	0	0	0	0	0	0	0	0
Solyc02g062640.2.1	4	14	5	3	0	8	17	14	5	60	14	51	0
Solyc02g062650.2.1	0	0	0	0	0	0	0	0	0	0	0	0	0
Solyc02g062670.2.1	1	5	0	1	6	1	6	1	0	2	1	3	0

Solyc02g062680.2.1	0	0	0	0	0	0	0	0	0	0	0	0	0
Solyc02g062690.2.1	0	0	0	0	0	0	0	0	0	0	0	0	0
Solyc02g062700.2.1	2	9	0	2	0	18	9	8	1	9	0	8	0
Solyc02g062710.1.1	4	1	0	13	0	21	10	10	5	0	1	2	1
Solyc02g062720.2.1	1	17	1	12	0	11	3	21	1	33	7	21	0
Solyc02g062740.2.1	9	40	17	35	7	105	139	149	25	82	44	36	3
Solyc02g062780.2.1	0	0	0	0	0	0	0	0	0	0	0	0	0
Solyc02g062790.2.1	0	0	0	0	0	0	0	0	0	0	0	0	0
Solyc02g062800.2.1	12	45	2	22	1	102	56	72	15	59	13	64	4
Solyc02g062810.2.1	2	6	2	9	3	18	6	20	0	24	2	20	3
Solyc02g062890.1.1	0	0	0	0	0	0	0	0	0	0	0	0	0
Solyc02g062910.1.1	4	16	0	8	1	46	8	30	4	83	12	28	0
Solyc02g062920.2.1	7	54	10	57	5	115	70	126	8	161	37	96	8
Solyc02g062930.2.1	0	0	0	0	0	0	0	0	0	0	0	0	0
Solyc02g062940.2.1	6	36	4	12	5	25	21	44	0	21	9	39	0
Solyc02g062950.2.1	6	48	1	19	4	25	61	74	7	43	25	47	3
Solyc02g062960.2.1	0	0	0	0	0	0	0	0	0	0	0	0	0
Solyc02g062970.2.1	20	363	80	319	49	483	1152	668	93	1410	410	868	55
Solyc02g062990.1.1	0	0	0	0	0	0	0	0	0	0	0	0	0
Solyc02g063000.2.1	9	521	20	78	0	7	0	1	0	4	63	17	0
Solyc02g063010.2.1	7	200	28	83	9	94	89	108	9	343	40	277	1
Solyc02g063030.2.1	7	302	14	173	0	7	46	12	0	477	18	255	1
Solyc02g063040.2.1	0	0	0	0	0	0	0	0	0	0	0	0	0
Solyc02g063050.2.1	13	72	3	71	0	244	100	177	20	145	24	95	0
Solyc02g063060.2.1	0	0	0	0	0	0	0	0	0	0	0	0	0
Solyc02g063070.2.1	4	98	4	81	5	224	53	298	14	338	36	260	2
Solyc02g063090.2.1	9	42	6	39	6	55	112	199	25	163	71	150	2
Solyc02g063130.2.1	10	227	12	140	9	278	154	412	30	431	67	288	11
Solyc02g063140.2.1	5	23	4	0	8	21	97	11	177	12	0	0	0
Solyc02g063150.2.1	7	5659	333	3031	11	296	478	589	0	667	94	659	2
Solyc02g063160.2.1	3	29	2	8	0	175	68	76	8	13	3	6	0
Solyc02g063170.2.1	1	4	3	5	0	16	1	7	0	7	1	13	0
Solyc02g063180.2.1	0	0	0	0	0	0	0	0	0	0	0	0	0
Solyc02g063190.2.1	0	0	0	0	0	0	0	0	0	0	0	0	0
Solyc02g063200.1.1	0	0	0	0	0	0	0	0	0	0	0	0	0
Solyc02g063220.2.1	6	84	15	65	21	19	136	63	16	248	67	176	1
Solyc02g063230.1.1	0	0	0	0	0	0	0	0	0	0	0	0	0
Solyc02g063240.2.1	1	4	0	4	1	0	3	4	0	13	2	14	0
Solyc02g063250.2.1	0	0	0	0	0	0	0	0	0	0	0	0	0
Solyc02g063260.2.1	0	0	0	0	0	0	0	0	0	0	0	0	0
Solyc02g063270.2.1	3	25	0	8	2	30	9	57	1	270	3	120	1

Solyc02g063280.2.1	3	16	1	6	0	68	5	67	4	34	7	37	0
Solyc02g063290.2.1	0	0	0	0	0	0	0	0	0	0	0	0	0
Solyc02g063300.2.1	0	0	0	0	0	0	0	0	0	0	0	0	0
Solyc02g063320.2.1	0	0	0	0	0	0	0	0	0	0	0	0	0
Solyc02g063330.2.1	0	0	0	0	0	0	0	0	0	0	0	0	0
Solyc02g063350.1.1	2	29	4	11	2	10	15	5	0	0	0	1	5
Solyc02g063360.2.1	1	0	1	1	4	0	0	0	0	19	18	9	0
Solyc02g063370.2.1	0	0	0	0	0	0	0	0	0	0	0	0	0
Solyc02g063390.2.1	4	47	3	8	0	9	14	26	2	30	8	9	0
Solyc02g063400.1.1	0	0	0	0	0	0	0	0	0	0	0	0	0
Solyc02g063410.2.1	2	9	1	2	1	0	7	0	3	1	0	0	3
Solyc02g063420.2.1	1	5	1	0	0	0	4	0	0	26	3	12	1
Solyc02g063430.2.1	0	0	0	0	0	0	0	0	0	0	0	0	0
Solyc02g063440.2.1	2	14	1	11	1	25	9	12	5	34	0	43	1
Solyc02g063450.2.1	23	1409	69	2332	64	2968	388	2623	86	5424	245	2641	32
Solyc02g063460.1.1	0	0	0	0	0	0	0	0	0	0	0	0	0
Solyc02g063490.2.1	9	126	31	144	8	336	404	415	63	413	124	305	9
Solyc02g063510.1.1	0	0	0	0	0	0	0	0	0	0	0	0	0
Solyc02g063520.2.1	2	9	0	8	0	8	15	20	2	6	2	9	0
Solyc02g063530.2.1	2	115	5	128	3	26	16	127	9	28	8	23	0
Solyc02g063540.1.1	0	0	0	0	0	0	0	0	0	0	0	0	0
Solyc02g064550.2.1	4	146	5	62	2	52	28	106	1	87	4	66	0
Solyc02g064580.2.1	7	14	3	7	0	19	26	35	4	24	4	13	0
Solyc02g064630.2.1	6	29	1	11	0	41	32	56	11	105	27	67	1
Solyc02g064640.2.1	2	45	1	23	0	30	10	29	2	30	4	64	0
Solyc02g064650.2.1	3	24	2	6	0	36	7	21	5	18	10	26	1
Solyc02g064670.1.1	4	137	10	50	3	132	78	125	15	169	54	110	0
Solyc02g064680.2.1	15	1417	78	571	23	1655	143	724	32	439	26	560	8
Solyc02g064690.2.1	0	0	0	0	0	0	0	0	0	0	0	0	0
Solyc02g064700.2.1	2	52	0	8	0	64	26	72	6	81	11	49	1
Solyc02g064710.2.1	0	0	0	0	0	0	0	0	0	0	0	0	0
Solyc02g064720.2.1	15	76	10	41	7	156	365	307	15	294	58	165	1
Solyc02g064730.2.1	2	54	0	25	4	73	14	82	10	319	51	191	1
Solyc02g064740.2.1	3	8	1	6	7	18	10	12	1	16	3	10	0
Solyc02g064750.2.1	7	59	9	30	5	95	64	102	17	338	30	235	2
Solyc02g064770.2.1	9	243	43	189	18	258	303	212	86	16	8	4	3
Solyc02g064800.2.1	9	42	5	30	1	10	81	52	7	67	11	58	3
Solyc02g064810.1.1	0	0	0	0	0	0	0	0	0	0	0	0	0
Solyc02g064830.2.1	0	0	0	0	0	0	0	0	0	0	0	0	0
Solyc02g064940.1.1	15	688	48	658	23	218	65	373	4	160	4	92	0
Solyc02g064950.2.1	6	223	11	120	0	105	43	154	0	106	14	69	4

Solyc02g064960.2.1	0	0	0	0	0	0	0	0	0	0	0	0	0
Solyc02g064970.2.1	0	0	0	0	0	0	0	0	0	0	0	0	0
Solyc02g064980.1.1	0	0	0	0	0	0	0	0	0	0	0	0	0
Solyc02g064990.2.1	0	0	0	0	0	0	0	0	0	0	0	0	0
Solyc02g065000.1.1	2	2	4	3	0	6	0	5	0	5	0	4	0
Solyc02g065010.1.1	0	0	0	0	0	0	0	0	0	0	0	0	0
Solyc02g065030.1.1	0	0	0	0	0	0	0	0	0	0	0	0	0
Solyc02g065050.1.1	2	8	4	5	0	3	4	3	1	2	1	0	0
Solyc02g065060.2.1	4	4	1	1	2	3	0	4	0	78	17	49	1
Solyc02g065070.2.1	3	40	1	17	0	35	8	38	1	11	0	17	0
Solyc02g065080.2.1	3	23	1	11	4	37	29	37	5	28	35	35	9
Solyc02g065090.2.1	0	0	0	0	0	0	0	0	0	0	0	0	0
Solyc02g065110.2.1	1	8	4	6	0	0	4	5	2	1	1	3	0
Solyc02g065160.1.1	0	0	0	0	0	0	0	0	0	0	0	0	0
Solyc02g065170.2.1	4	28	1	6	1	0	12	16	1	19	5	57	1
Solyc02g065180.2.1	2	7	5	0	0	3	17	25	0	1	1	6	1
Solyc02g065190.2.1	0	0	0	0	0	0	0	0	0	0	0	0	0
Solyc02g065210.2.1	0	0	0	0	0	0	0	0	0	0	0	0	0
Solyc02g065220.2.1	7	233	19	188	3	48	22	73	6	0	3	2	0
Solyc02g065230.2.1	0	0	0	0	0	0	0	0	0	0	0	0	0
Solyc02g065240.2.1	5	66	0	22	0	381	1	184	0	152	136	19	0
Solyc02g065250.1.1	0	0	0	0	0	0	0	0	0	0	0	0	0
Solyc02g065260.2.1	0	0	0	0	0	0	0	0	0	0	0	0	0
Solyc02g065280.2.1	2	39	1	35	0	22	7	48	0	3	1	2	0
Solyc02g065290.1.1	0	0	0	0	0	0	0	0	0	0	0	0	0
Solyc02g065300.1.1	19	214	19	176	0	473	136	534	15	501	63	422	3
Solyc02g065350.2.1	1	0	0	0	0	0	0	0	0	4	1	8	0
Solyc02g065360.2.1	8	16	3	12	3	56	48	45	4	34	19	26	1
Solyc02g065370.2.1	3	12	2	21	0	65	20	51	6	47	11	41	0
Solyc02g065380.2.1	7	173	29	70	11	321	447	292	31	420	64	226	1
Solyc02g065390.1.1	0	0	0	0	0	0	0	0	0	0	0	0	0
Solyc02g065400.2.1	3	2389	313	1686	3	92	474	165	11	410	270	670	15
Solyc02g065410.1.1	0	0	0	0	0	0	0	0	0	0	0	0	0
Solyc02g065420.2.1	1	0	0	0	0	0	0	0	0	6	4	4	0
Solyc02g065430.1.1	0	0	0	0	0	0	0	0	0	0	0	0	0
Solyc02g065470.1.1	5	18	2	7	0	12	9	50	4	33	7	48	0
Solyc02g065480.2.1	0	0	0	0	0	0	0	0	0	0	0	0	0
Solyc02g065490.2.1	0	0	0	0	0	0	0	0	0	0	0	0	0
Solyc02g065500.2.1	7	9	1	6	4	40	26	46	3	47	4	35	2
Solyc02g065510.1.1	1	2	0	4	0	4	5	0	1	1	0	3	0
Solyc02g065520.1.1	1	10	0	2	0	4	3	6	0	19	0	19	1

Solyc02g065530.2.1	2	10	5	6	0	1	7	3	2	31	0	22	0
Solyc02g065540.1.1	3	34	7	20	2	20	23	14	6	32	9	28	0
Solyc02g065550.2.1	16	88	13	45	8	309	155	165	21	353	120	369	13
Solyc02g065560.1.1	1	0	0	0	0	0	3	3	0	5	1	6	0
Solyc02g065570.1.1	0	0	0	0	0	0	0	0	0	0	0	0	0
Solyc02g065580.2.1	4	24	1	9	0	51	21	64	3	79	14	89	7
Solyc02g065590.2.1	2	20	1	8	2	13	16	1	0	0	0	0	0
Solyc02g065600.2.1	1	1	0	1	0	4	5	2	1	0	0	0	0
Solyc02g065610.2.1	1	1	1	0	0	4	1	4	0	5	2	2	0
Solyc02g065620.2.1	1	12	1	10	0	12	6	21	0	18	0	15	0
Solyc02g065630.2.1	8	26	6	26	8	34	75	51	21	70	17	40	4
Solyc02g065650.1.1	4	5	1	8	0	35	6	18	2	12	10	20	0
Solyc02g065660.2.1	9	20	4	18	0	117	55	162	5	53	8	54	0
Solyc02g065680.2.1	16	440	73	248	6	678	630	843	48	747	259	956	11
Solyc02g065690.2.1	0	0	0	0	0	0	0	0	0	0	0	0	0
Solyc02g065700.2.1	1	15	1	10	0	23	2	11	5	44	5	11	0
Solyc02g065720.2.1	1	5	1	2	0	12	8	11	0	5	1	5	0
Solyc02g065730.1.1	0	0	0	0	0	0	0	0	0	0	0	0	0
Solyc02g065770.2.1	10	578	21	407	54	78	187	413	237	1430	121	851	13
Solyc02g065780.1.1	8	388	14	127	10	17	115	79	22	1530	28	1843	9
Solyc02g065790.2.1	5	15	4	8	5	20	65	24	8	15	24	12	0
Solyc02g066830.2.1	0	0	0	0	0	0	0	0	0	0	0	0	0
Solyc02g066910.2.1	2	9	1	5	0	6	8	6	0	16	1	9	0
Solyc02g066920.2.1	3	12	1	12	0	3	15	3	0	5	0	10	0
Solyc02g066930.2.1	7	8	3	13	0	5	44	13	7	62	55	96	1
Solyc02g066940.2.1	16	112	16	77	18	281	142	225	22	168	55	108	4
Solyc02g066950.2.1	13	132	19	104	8	135	172	298	10	100	31	42	5
Solyc02g066990.2.1	0	0	0	0	0	0	0	0	0	0	0	0	0
Solyc02g067010.2.1	2	4	1	0	1	10	9	1	0	8	1	13	0
Solyc02g067020.1.1	0	0	0	0	0	0	0	0	0	0	0	0	0
Solyc02g067030.2.1	8	88	10	69	6	196	94	325	16	121	16	103	4
Solyc02g067040.2.1	0	0	0	0	0	0	0	0	0	0	0	0	0
Solyc02g067050.2.1	1	7	0	6	0	7	1	8	0	41	6	79	2
Solyc02g067060.2.1	2	9	1	1	0	18	11	7	0	8	0	5	0
Solyc02g067070.2.1	9	28	5	25	0	46	49	78	6	46	14	55	0
Solyc02g067080.2.1	10	277	14	198	14	223	142	301	27	947	98	668	22
Solyc02g067090.2.1	9	35	2	18	0	37	39	52	5	104	17	55	2
Solyc02g067100.2.1	0	0	0	0	0	0	0	0	0	0	0	0	0
Solyc02g067120.2.1	1	11	2	5	0	76	6	26	1	6	0	5	3
Solyc02g067150.2.1	0	0	0	0	0	0	0	0	0	0	0	0	0
Solyc02g067160.2.1	2	17	4	6	0	61	15	43	3	6	2	6	2

Solyc02g067170.1.1	0	0	0	0	0	0	0	0	0	0	0	0	0
Solyc02g067180.2.1	20	772	206	466	72	1182	953	861	83	1308	2760	808	23
Solyc02g067200.2.1	3	11	0	2	0	14	20	23	0	19	8	18	0
Solyc02g067210.2.1	4	34	0	38	1	77	51	85	5	37	20	62	1
Solyc02g067230.2.1	0	0	0	0	0	0	0	0	0	0	0	0	0
Solyc02g067250.1.1	0	0	0	0	0	0	0	0	0	0	0	0	0
Solyc02g067260.1.1	0	0	0	0	0	0	0	0	0	0	0	0	0
Solyc02g067280.1.1	0	0	0	0	0	0	0	0	0	0	0	0	0
Solyc02g067290.2.1	0	0	0	0	0	0	0	0	0	0	0	0	0
Solyc02g067300.2.1	1	2	1	0	0	26	2	7	0	2	9	3	0
Solyc02g067310.2.1	4	30	5	22	0	47	51	87	3	0	4	0	0
Solyc02g067320.1.1	9	65	7	43	2	239	77	151	11	15	3	6	0
Solyc02g067340.2.1	0	0	0	0	0	0	0	0	0	0	0	0	0
Solyc02g067350.2.1	1	4	0	2	0	13	4	5	1	1	1	8	0
Solyc02g067360.2.1	4	26	2	26	0	21	12	13	0	119	1	79	0
Solyc02g067370.2.1	1	10	2	7	0	66	4	29	1	31	1	41	0
Solyc02g067380.2.1	0	0	0	0	0	0	0	0	0	0	0	0	0
Solyc02g067390.2.1	4	37	4	44	5	43	36	47	15	55	19	65	3
Solyc02g067420.2.1	0	0	0	0	0	0	0	0	0	0	0	0	0
Solyc02g067440.2.1	0	0	0	0	0	0	0	0	0	0	0	0	0
Solyc02g067450.2.1	0	0	0	0	0	0	0	0	0	0	0	0	0
Solyc02g067460.2.1	6	485	34	98	18	613	128	598	41	496	165	373	9
Solyc02g067470.2.1	3	10	2	3	0	61	21	40	4	41	2	26	0
Solyc02g067480.2.1	2	4	0	0	0	4	10	2	0	6	0	12	0
Solyc02g067490.2.1	1	0	0	0	0	6	4	5	2	0	0	0	0
Solyc02g067510.2.1	0	0	0	0	0	0	0	0	0	0	0	0	0
Solyc02g067530.2.1	1	8	2	16	0	2	1	7	0	19	2	9	0
Solyc02g067540.2.1	3	12	1	10	0	22	12	19	0	18	2	26	0
Solyc02g067550.1.1	0	0	0	0	0	0	0	0	0	0	0	0	0
Solyc02g067560.1.1	2	21	1	7	2	8	16	29	2	15	12	19	0
Solyc02g067570.2.1	30	114	20	84	14	165	184	329	36	180	58	192	4
Solyc02g067580.2.1	1	3	0	2	0	3	0	1	0	63	9	34	0
Solyc02g067620.1.1	11	34	3	12	4	175	80	179	15	112	32	40	0
Solyc02g067660.2.1	8	61	11	30	0	2	17	13	1	13	7	17	6
Solyc02g067670.2.1	32	94	15	75	4	159	156	237	28	276	52	204	3
Solyc02g067680.1.1	1	6	0	1	0	4	0	5	0	0	0	2	0
Solyc02g067690.2.1	17	108	11	66	6	259	172	256	20	512	66	405	4
Solyc02g067700.1.1	0	0	0	0	0	0	0	0	0	0	0	0	0
Solyc02g067710.1.1	0	0	0	0	0	0	0	0	0	0	0	0	0
Solyc02g067740.2.1	7	18	2	14	0	38	29	56	8	27	4	22	1
Solyc02g067750.2.1	15	1	0	1	0	662	145	421	0	2	105	2	0

Solyc02g067760.2.1	0	0	0	0	0	0	0	0	0	0	0	0	0
Solyc02g067780.2.1	0	0	0	0	0	0	0	0	0	0	0	0	0
Solyc02g067790.2.1	0	0	0	0	0	0	0	0	0	0	0	0	0
Solyc02g067800.2.1	1	2	0	0	0	6	2	7	2	6	2	3	0
Solyc02g067810.1.1	0	0	0	0	0	0	0	0	0	0	0	0	0
Solyc02g067820.2.1	15	57	11	30	0	151	55	153	6	141	28	96	5
Solyc02g067830.2.1	3	13	5	2	6	8	3	16	1	19	7	9	0
Solyc02g067840.2.1	10	67	1	13	3	197	70	118	11	80	21	29	0
Solyc02g067850.2.1	0	0	0	0	0	0	0	0	0	0	0	0	0
Solyc02g067860.2.1	3	31	2	30	0	138	42	103	4	31	3	58	0
Solyc02g067870.2.1	0	0	0	0	0	0	0	0	0	0	0	0	0
Solyc02g067890.2.1	6	36	1	27	0	13	24	32	2	94	19	69	1
Solyc02g067900.2.1	3	4	1	8	0	35	17	26	0	13	6	9	0
Solyc02g067910.1.1	1	3	0	3	0	14	1	8	2	20	5	5	0
Solyc02g067920.2.1	5	29	1	21	0	5	13	6	0	53	15	44	4
Solyc02g067930.2.1	1	3	0	0	0	0	4	0	2	2	0	11	0
Solyc02g067960.1.1	4	6	1	12	0	55	6	33	0	28	0	9	0
Solyc02g067970.2.1	0	0	0	0	0	0	0	0	0	0	0	0	0
Solyc02g067980.2.1	8	75	3	17	0	110	58	86	6	100	14	75	2
Solyc02g067990.2.1	3	18	1	2	4	33	13	17	3	15	2	0	0
Solyc02g068000.2.1	3	5	0	0	0	9	17	17	4	24	11	12	0
Solyc02g068010.2.1	4	41	4	23	0	329	46	111	13	83	41	49	7
Solyc02g068030.1.1	9	32	0	18	6	274	79	217	8	81	28	47	1
Solyc02g068040.2.1	0	0	0	0	0	0	0	0	0	0	0	0	0
Solyc02g068050.2.1	4	31	1	13	3	25	12	47	0	13	1	16	0
Solyc02g068060.2.1	2	8	3	11	0	8	9	4	0	0	0	0	0
Solyc02g068070.2.1	0	0	0	0	0	0	0	0	0	0	0	0	0
Solyc02g068080.2.1	12	67	12	64	1	334	95	319	0	102	3	117	1
Solyc02g068090.2.1	7	497	30	209	5	110	287	251	6	136	30	138	0
Solyc02g068100.2.1	12	126	19	113	17	513	283	741	53	195	73	280	0
Solyc02g068110.2.1	0	0	0	0	0	0	0	0	0	0	0	0	0
Solyc02g068120.2.1	1	1	0	2	2	0	3	8	5	9	0	9	0
Solyc02g068130.2.1	0	0	0	0	0	0	0	0	0	0	0	0	0
Solyc02g068140.2.1	1	3	0	1	0	1	2	0	0	0	2	4	0
Solyc02g068150.2.1	0	0	0	0	0	0	0	0	0	0	0	0	0
Solyc02g068160.1.1	0	0	0	0	0	0	0	0	0	0	0	0	0
Solyc02g068170.1.1	0	0	0	0	0	0	0	0	0	0	0	0	0
Solyc02g068180.2.1	1	1	0	0	0	5	11	11	0	3	1	3	0
Solyc02g068190.1.1	0	0	0	0	0	0	0	0	0	0	0	0	0
Solyc02g068200.1.1	7	138	46	80	23	312	89	362	13	500	25	257	9
Solyc02g068230.2.1	2	26	0	16	0	27	19	44	5	78	7	21	3

Solyc02g068240.2.1	11	75	4	44	7	102	107	157	20	108	28	75	10
Solyc02g068260.1.1	1	1	0	1	0	3	2	5	0	14	1	8	0
Solyc02g068270.2.1	4	56	2	25	0	25	39	44	2	41	8	27	0
Solyc02g068280.2.1	7	13	2	12	5	61	34	81	0	52	5	44	0
Solyc02g068290.1.1	1	4	0	5	0	9	5	10	1	13	0	4	0
Solyc02g068300.2.1	0	0	0	0	0	0	0	0	0	0	0	0	0
Solyc02g068310.2.1	3	4	1	2	0	1	40	24	0	19	33	15	1
Solyc02g068320.2.1	0	0	0	0	0	0	0	0	0	0	0	0	0
Solyc02g068330.1.1	0	0	0	0	0	0	0	0	0	0	0	0	0
Solyc02g068340.2.1	8	26	3	21	8	7	48	39	7	29	16	28	6
Solyc02g068350.1.1	3	19	8	33	0	14	30	31	1	29	5	32	1
Solyc02g068360.2.1	2	9	0	4	1	37	15	12	2	35	7	27	0
Solyc02g068370.2.1	3	8	2	15	4	31	20	23	2	16	1	3	2
Solyc02g068380.2.1	11	105	18	35	1	194	76	162	9	689	97	402	12
Solyc02g068390.2.1	0	0	0	0	0	0	0	0	0	0	0	0	0
Solyc02g068400.2.1	2	1	0	3	0	30	4	8	1	0	1	1	0
Solyc02g068410.1.1	0	0	0	0	0	0	0	0	0	0	0	0	0
Solyc02g068420.2.1	6	68	9	23	0	90	32	89	7	704	26	260	0
Solyc02g068430.2.1	3	23	9	17	0	78	49	55	8	46	3	34	0
Solyc02g068440.2.1	1	38	1	20	0	54	21	53	3	33	6	48	0
Solyc02g068450.2.1	2	78	10	19	0	168	32	119	4	85	21	58	1
Solyc02g068480.2.1	5	30	2	17	8	30	28	57	2	39	8	57	0
Solyc02g068490.2.1	13	95	20	77	7	157	145	234	15	111	29	118	0
Solyc02g068500.2.1	3	43	4	16	0	40	38	59	5	66	14	46	0
Solyc02g068530.2.1	5	121	5	39	4	51	32	69	9	337	12	199	1
Solyc02g068540.1.1	0	0	0	0	0	0	0	0	0	0	0	0	0
Solyc02g068550.1.1	1	3	0	0	0	0	7	3	0	1	0	2	0
Solyc02g068560.2.1	23	91	15	83	5	203	126	277	34	131	56	109	15
Solyc02g068580.1.1	8	28	12	25	6	37	50	54	23	41	30	33	0
Solyc02g068590.2.1	11	16	3	14	3	100	71	120	4	66	108	32	0
Solyc02g068600.2.1	0	0	0	0	0	0	0	0	0	0	0	0	0
Solyc02g068610.1.1	0	0	0	0	0	0	0	0	0	0	0	0	0
Solyc02g068620.1.1	1	0	0	0	0	0	0	0	4	4	106	0	7
Solyc02g068630.1.1	0	0	0	0	0	0	0	0	0	0	0	0	0
Solyc02g068640.2.1	5	21	3	30	0	189	135	165	15	84	12	139	0
Solyc02g068650.1.1	3	9	6	3	0	57	16	22	1	14	10	22	0
Solyc02g068660.1.1	0	0	0	0	0	0	0	0	0	0	0	0	0
Solyc02g068670.1.1	0	0	0	0	0	0	0	0	0	0	0	0	0
Solyc02g068680.1.1	1	3	3	2	1	11	0	1	1	0	0	6	2
Solyc02g068690.2.1	0	0	0	0	0	0	0	0	0	0	0	0	0
Solyc02g068700.2.1	7	60	7	39	1	48	39	63	2	73	29	50	5

Solyc02g068710.2.1	1	10	0	2	0	0	6	4	0	12	0	4	0
Solyc02g068720.2.1	8	78	1	46	2	74	60	105	6	186	9	126	8
Solyc02g068730.2.1	4	3	2	0	0	7	30	31	8	13	0	9	0
Solyc02g068740.2.1	4	34	1	15	0	62	14	85	4	132	40	146	0
Solyc02g068750.1.1	6	16	1	15	2	46	13	50	6	25	19	16	0
Solyc02g068760.2.1	4	68	17	70	0	12	86	16	0	5	0	0	0
Solyc02g068770.2.1	2	14	0	18	0	53	11	36	5	116	5	69	0
Solyc02g068780.2.1	8	36	4	23	2	47	69	80	6	62	7	51	2
Solyc02g068790.2.1	1	4	0	1	6	6	2	9	4	5	4	10	0
Solyc02g068800.1.1	0	0	0	0	0	0	0	0	0	0	0	0	0
Solyc02g068820.1.1	0	0	0	0	0	0	0	0	0	0	0	0	0
Solyc02g068830.1.1	0	0	0	0	0	0	0	0	0	0	0	0	0
Solyc02g068890.2.1	0	0	0	0	0	0	0	0	0	0	0	0	0
Solyc02g068900.2.1	8	89	9	60	8	458	173	440	42	127	84	121	1
Solyc02g068910.2.1	5	73	11	37	4	20	74	73	5	273	30	204	2
Solyc02g068920.2.1	16	211	55	203	23	339	497	366	33	790	215	371	14
Solyc02g068930.2.1	2	48	1	11	2	25	8	36	1	17	1	8	0
Solyc02g068990.2.1	4	56	2	42	0	57	37	55	1	111	21	48	2
Solyc02g069010.2.1	3	33	5	13	1	41	12	53	1	16	8	21	0
Solyc02g069020.2.1	1	4	4	3	0	13	34	19	0	5	1	5	0
Solyc02g069030.2.1	0	0	0	0	0	0	0	0	0	0	0	0	0
Solyc02g069040.2.1	0	0	0	0	0	0	0	0	0	0	0	0	0
Solyc02g069050.1.1	0	0	0	0	0	0	0	0	0	0	0	0	0
Solyc02g069060.2.1	0	0	0	0	0	0	0	0	0	0	0	0	0
Solyc02g069070.2.1	0	0	0	0	0	0	0	0	0	0	0	0	0
Solyc02g069080.2.1	7	75	6	24	4	129	27	98	4	130	45	98	4
Solyc02g069090.2.1	14	377	35	188	11	698	604	1118	62	521	104	395	5
Solyc02g069100.2.1	3	173	23	85	6	506	1273	762	81	333	199	141	0
Solyc02g069110.2.1	0	0	0	0	0	0	0	0	0	0	0	0	0
Solyc02g069120.2.1	4	11	1	2	0	6	20	6	0	28	11	25	1
Solyc02g069130.2.1	0	0	0	0	0	0	0	0	0	0	0	0	0
Solyc02g069140.1.1	0	0	0	0	0	0	0	0	0	0	0	0	0
Solyc02g069150.2.1	5	187	14	101	9	258	105	339	31	499	52	296	3
Solyc02g069160.2.1	11	113	14	107	5	283	189	329	23	202	23	150	7
Solyc02g069170.1.1	0	0	0	0	0	0	0	0	0	0	0	0	0
Solyc02g069180.2.1	7	51	3	85	4	122	20	101	5	497	51	233	1
Solyc02g069220.1.1	0	0	0	0	0	0	0	0	0	0	0	0	0
Solyc02g069230.2.1	13	148	6	82	6	236	109	375	18	191	28	120	4
Solyc02g069240.1.1	1	1	2	0	0	2	0	1	0	3	5	4	0
Solyc02g069250.2.1	0	0	0	0	0	0	0	0	0	0	0	0	0
Solyc02g069260.2.1	14	239	34	189	4	209	27	212	20	118	39	142	8

Solyc02g069270.2.1	0	0	0	0	0	0	0	0	0	0	0	0	0
Solyc02g069280.2.1	0	0	0	0	0	0	0	0	0	0	0	0	0
Solyc02g069290.2.1	17	144	19	161	0	121	139	251	10	110	11	138	1
Solyc02g069300.1.1	0	0	0	0	0	0	0	0	0	0	0	0	0
Solyc02g069310.2.1	8	52	9	22	7	99	44	83	18	111	18	57	1
Solyc02g069320.1.1	0	0	0	0	0	0	0	0	0	0	0	0	0
Solyc02g069340.2.1	2	13	4	0	0	3	9	19	2	3	6	23	1
Solyc02g069350.2.1	2	13	0	0	0	14	16	19	0	4	0	1	0
Solyc02g069370.2.1	2	80	1	35	0	215	16	169	7	48	5	36	0
Solyc02g069380.2.1	1	2	3	5	1	5	14	2	3	5	1	11	0
Solyc02g069390.1.1	0	0	0	0	0	0	0	0	0	0	0	0	0
Solyc02g069400.2.1	6	24	1	17	0	26	47	58	8	61	1	24	1
Solyc02g069410.2.1	5	51	3	23	1	21	20	47	0	42	25	15	3
Solyc02g069420.2.1	1	0	0	0	0	3	0	1	0	10	11	7	0
Solyc02g069430.2.1	1	0	0	0	0	0	0	0	0	4	7	3	5
Solyc02g069450.2.1	3	432	207	209	7	1	100	2	2	15	84	20	4
Solyc02g069460.2.1	5	4391	255	1727	3	68	321	184	11	491	136	377	10
Solyc02g069470.2.1	11	45	5	35	2	64	93	69	17	83	40	52	2
Solyc02g069490.2.1	14	3148	167	850	10	106	30	338	13	46	364	12	0
Solyc02g069500.1.1	2	9	1	0	0	6	5	4	0	4	5	5	0
Solyc02g069510.1.1	0	0	0	0	0	0	0	0	0	0	0	0	0
Solyc02g069520.2.1	2	16	4	5	0	5	24	18	2	2	2	22	0
Solyc02g069560.2.1	0	0	0	0	0	0	0	0	0	0	0	0	0
Solyc02g069570.2.1	9	43	14	27	2	109	92	122	3	37	12	15	0
Solyc02g069580.2.1	10	106	8	61	4	459	81	177	68	125	40	83	5
Solyc02g069590.2.1	7	211	12	44	5	198	137	292	26	377	75	383	6
Solyc02g069600.2.1	0	0	0	0	0	0	0	0	0	0	0	0	0
Solyc02g069610.2.1	3	28	2	8	5	49	40	57	4	78	12	49	0
Solyc02g069620.2.1	2	11	1	22	0	3	13	2	1	6	1	22	0
Solyc02g069630.2.1	0	0	0	0	0	0	0	0	0	0	0	0	0
Solyc02g069640.2.1	4	5	1	8	2	16	51	16	13	18	7	15	3
Solyc02g069650.1.1	8	35	6	28	0	71	44	47	14	78	16	75	0
Solyc02g069660.2.1	2	6	0	1	0	14	7	11	2	9	1	6	0
Solyc02g069670.2.1	1	1	0	3	1	0	6	0	1	13	0	6	0
Solyc02g069680.2.1	2	13	0	14	0	11	12	16	2	29	9	21	0
Solyc02g069690.1.1	0	0	0	0	0	0	0	0	0	0	0	0	0
Solyc02g069710.2.1	5	18	1	8	0	48	27	23	2	11	5	15	0
Solyc02g069720.2.1	1	39	3	11	1	42	3	48	2	64	2	18	0
Solyc02g069730.2.1	0	0	0	0	0	0	0	0	0	0	0	0	0
Solyc02g069740.2.1	0	0	0	0	0	0	0	0	0	0	0	0	0
Solyc02g069750.1.1	2	1	2	5	0	4	14	11	1	21	1	13	0

Solyc02g069770.2.1	1	6	0	7	0	22	7	7	1	10	8	17	1
Solyc02g069780.2.1	11	50	13	33	5	144	88	133	10	72	27	73	2
Solyc02g069790.2.1	4	36	3	9	3	71	19	60	6	55	18	53	0
Solyc02g069800.1.1	13	312	56	231	13	476	130	636	15	131	74	197	13
Solyc02g069810.1.1	0	0	0	0	0	0	0	0	0	0	0	0	0
Solyc02g069820.2.1	4	21	1	4	0	26	22	26	0	68	8	70	6
Solyc02g069830.2.1	3	10	2	4	0	7	7	25	0	18	5	9	1
Solyc02g069840.2.1	2	17	0	12	0	23	2	61	2	40	6	32	0
Solyc02g069850.2.1	2	191	19	173	20	413	325	646	119	589	355	512	21
Solyc02g069860.2.1	4	42	6	18	1	30	10	39	1	83	16	40	0
Solyc02g069880.2.1	0	0	0	0	0	0	0	0	0	0	0	0	0
Solyc02g069910.1.1	1	47	3	35	1	7	1	1	0	1	0	5	2
Solyc02g069920.2.1	2	18	0	8	0	11	11	27	2	1	1	1	0
Solyc02g069930.1.1	1	4	0	0	0	0	27	2	26	0	0	0	0
Solyc02g069940.2.1	6	23	0	11	2	31	35	52	9	24	3	17	0
Solyc02g069950.2.1	3	13	2	16	0	34	2	47	2	121	14	112	1
Solyc02g069970.2.1	1	4	0	0	0	4	2	4	0	1	1	3	0
Solyc02g069980.2.1	2	5	0	0	0	3	4	6	1	2	4	14	2
Solyc02g070000.2.1	5	19	1	18	0	13	1	22	0	44	22	24	0
Solyc02g070010.1.1	2	6	0	14	0	29	18	40	0	4	1	7	0
Solyc02g070020.1.1	8	5	0	9	0	339	26	488	0	18	37	14	1
Solyc02g070030.2.1	7	21	4	3	4	63	77	71	17	52	25	58	1
Solyc02g070040.1.1	2	83	9	83	2	5	7	1	0	11	0	35	0
Solyc02g070050.2.1	5	125	5	42	6	34	32	47	5	67	30	77	0
Solyc02g070070.1.1	1	0	0	4	0	13	8	12	0	0	0	1	0
Solyc02g070090.1.1	0	0	0	0	0	0	0	0	0	0	0	0	0
Solyc02g070100.2.1	5	6	0	2	3	18	17	23	3	8	8	22	20
Solyc02g070110.1.1	0	0	0	0	0	0	0	0	0	0	0	0	0
Solyc02g070180.1.1	0	0	0	0	0	0	0	0	0	0	0	0	0
Solyc02g070210.2.1	2	5	0	4	0	12	4	2	0	4	3	0	0
Solyc02g070220.2.1	2	3	0	1	0	8	5	1	4	2	2	5	0
Solyc02g070230.1.1	2	8	1	16	0	6	8	10	1	10	4	9	0
Solyc02g070240.2.1	7	18	0	16	0	46	12	75	0	27	15	29	0
Solyc02g070250.2.1	5	34	6	28	2	61	49	67	7	43	9	77	0
Solyc02g070260.2.1	10	62	8	65	7	75	52	100	3	372	35	334	0
Solyc02g070280.2.1	4	161	10	129	35	165	45	175	24	10	43	6	2
Solyc02g070290.2.1	2	7	0	0	0	6	7	10	0	10	1	7	0
Solyc02g070300.2.1	1	0	3	0	0	5	4	1	2	2	2	2	0
Solyc02g070310.2.1	1	427	13	526	13	2196	634	1172	59	3319	157	2187	20
Solyc02g070320.2.1	2	27	1	23	0	57	12	94	2	46	9	56	0
Solyc02g070330.2.1	0	0	0	0	0	0	0	0	0	0	0	0	0

Solyc02g070340.2.1	0	0	0	0	0	0	0	0	0	0	0	0	0
Solyc02g070350.2.1	0	0	0	0	0	0	0	0	0	0	0	0	0
Solyc02g070360.2.1	2	38	0	6	0	31	13	62	5	34	8	27	0
Solyc02g070370.2.1	0	0	0	0	0	0	0	0	0	0	0	0	0
Solyc02g070390.2.1	12	28	2	9	0	74	129	119	7	50	10	56	5
Solyc02g070400.2.1	6	47	3	45	3	122	37	228	11	62	10	58	1
Solyc02g070420.2.1	0	0	0	0	0	0	0	0	0	0	0	0	0
Solyc02g070430.2.1	0	0	0	0	0	0	0	0	0	0	0	0	0
Solyc02g070440.2.1	6	205	28	95	4	25	39	92	7	83	80	26	5
Solyc02g070450.1.1	0	0	0	0	0	0	0	0	0	0	0	0	0
Solyc02g070460.2.1	8	41	2	22	12	41	34	87	11	59	23	56	3
Solyc02g070470.1.1	5	55	8	17	4	54	56	51	4	163	8	80	1
Solyc02g070480.2.1	2	34	7	17	2	27	7	33	1	36	6	29	0
Solyc02g070490.2.1	11	172	19	72	3	22	98	70	4	75	12	102	9
Solyc02g070500.1.1	6	199	6	66	15	335	207	98	172	105	43	72	5
Solyc02g070510.2.1	5	58	6	24	3	175	39	118	11	140	9	164	1
Solyc02g070520.2.1	5	16	3	14	0	29	41	85	11	59	8	45	1
Solyc02g070530.2.1	1	4	2	4	3	45	2	16	2	2	0	4	0
Solyc02g070540.2.1	5	84	0	30	3	51	13	87	2	94	14	88	3
Solyc02g070550.2.1	5	261	42	186	10	389	463	532	22	1705	79	509	7
Solyc02g070560.2.1	2	11	2	1	3	9	6	13	2	13	8	10	0
Solyc02g070570.2.1	1	6	0	2	0	2	6	7	0	24	7	20	0
Solyc02g070580.1.1	6	45	0	11	0	78	19	63	2	25	11	14	0
Solyc02g070610.2.1	0	0	0	0	0	0	0	0	0	0	0	0	0
Solyc02g070630.2.1	1	4	0	1	0	1	5	14	0	1	0	7	0
Solyc02g070640.2.1	1	28	0	6	0	66	23	55	2	30	12	42	2
Solyc02g070650.2.1	0	0	0	0	0	0	0	0	0	0	0	0	0
Solyc02g070660.2.1	1	0	0	0	0	14	4	10	0	0	0	0	0
Solyc02g070670.2.1	6	175	3	148	2	186	49	256	1	339	13	86	0
Solyc02g070680.2.1	4	22	0	13	8	41	48	70	3	34	13	20	1
Solyc02g070690.1.1	0	0	0	0	0	0	0	0	0	0	0	0	0
Solyc02g070700.1.1	0	0	0	0	0	0	0	0	0	0	0	0	0
Solyc02g070710.1.1	0	0	0	0	0	0	0	0	0	0	0	0	0
Solyc02g070730.1.1	0	0	0	0	0	0	0	0	0	0	0	0	0
Solyc02g070760.2.1	4	40	3	7	0	54	64	16	1	6	6	1	1
Solyc02g070770.2.1	3	39	14	7	0	1	47	18	1	85	8	94	5
Solyc02g070780.2.1	2	18	0	2	1	12	0	8	0	22	9	16	0
Solyc02g070790.2.1	12	86	8	30	17	95	115	104	46	113	64	29	10
Solyc02g070800.2.1	12	970	109	509	13	341	4508	1041	29	1437	83	765	17
Solyc02g070810.2.1	11	12	4	19	0	15	47	67	16	55	26	47	1
Solyc02g070820.1.1	0	0	0	0	0	0	0	0	0	0	0	0	0

Solyc02g070860.2.1	1	13	2	0	0	18	8	17	4	15	0	4	0
Solyc02g070870.2.1	2	3	1	1	0	1	8	10	2	12	0	4	0
Solyc02g070880.1.1	7	32	2	30	2	20	6	35	5	113	13	54	0
Solyc02g070890.2.1	1	2	0	0	0	10	5	18	0	0	0	0	0
Solyc02g070900.1.1	0	0	0	0	0	0	0	0	0	0	0	0	0
Solyc02g070910.1.1	1	6	1	9	0	0	1	0	0	0	3	2	0
Solyc02g070930.1.1	0	0	0	0	0	0	0	0	0	0	0	0	0
Solyc02g070940.1.1	1	253	9	158	0	0	0	0	0	25	2	12	0
Solyc02g070950.1.1	2	831	274	241	3	4	94	3	0	19	120	6	3
Solyc02g070970.1.1	4	2373	890	1237	8	7	16	6	1	9	339	9	3
Solyc02g070980.1.1	1	192	126	84	7	3	2	0	0	3	66	0	2
Solyc02g070990.1.1	2	216	9	107	0	1	0	1	0	3	0	0	0
Solyc02g071000.1.1	4	870	463	500	0	3	1	3	0	9	56	0	0
Solyc02g071010.1.1	1	524	81	122	6	2	1	0	0	1	36	1	0
Solyc02g071030.1.1	2	2106	155	851	2	143	29	93	5	53	126	47	1
Solyc02g071040.2.1	16	43	5	35	0	160	194	166	18	120	50	65	7
Solyc02g071050.2.1	0	0	0	0	0	0	0	0	0	0	0	0	0
Solyc02g071060.2.1	0	0	0	0	0	0	0	0	0	0	0	0	0
Solyc02g071070.2.1	0	0	0	0	0	0	0	0	0	0	0	0	0
Solyc02g071080.1.1	3	16	0	4	2	7	2	22	12	56	35	7	0
Solyc02g071090.2.1	5	32	3	40	0	23	7	31	18	68	17	7	0
Solyc02g071100.2.1	5	55	2	52	1	35	38	142	25	110	33	81	6
Solyc02g071110.2.1	2	9	2	5	0	0	0	1	2	11	6	11	0
Solyc02g071120.2.1	10	185	14	49	5	236	80	200	17	139	76	81	4
Solyc02g071130.2.1	2	0	0	0	1	28	23	8	13	0	6	0	0
Solyc02g071140.2.1	4	6	1	14	2	15	35	21	1	13	3	15	0
Solyc02g071150.2.1	1	1	0	1	0	3	3	3	0	12	4	4	1
Solyc02g071160.2.1	5	66	2	24	7	41	27	73	5	91	7	93	0
Solyc02g071170.2.1	2	23	2	23	0	35	14	61	2	41	16	46	5
Solyc02g071180.2.1	20	157	11	128	10	351	184	420	24	241	68	180	3
Solyc02g071190.2.1	2	7	3	8	0	6	19	15	0	4	0	2	0
Solyc02g071200.2.1	0	0	0	0	0	0	0	0	0	0	0	0	0
Solyc02g071210.2.1	3	57	1	38	0	528	290	263	6	25	17	32	0
Solyc02g071220.2.1	4	40	4	21	0	31	3	8	7	6	1	15	0
Solyc02g071230.1.1	0	0	0	0	0	0	0	0	0	0	0	0	0
Solyc02g071240.2.1	2	5	0	2	0	30	17	3	1	2	1	2	0
Solyc02g071250.2.1	13	174	19	158	8	433	127	438	37	199	16	142	0
Solyc02g071260.2.1	4	12	2	15	1	10	22	12	0	12	4	7	0
Solyc02g071270.1.1	12	32	10	59	4	157	158	163	21	93	29	102	0
Solyc02g071280.2.1	17	70	7	70	0	207	160	279	5	127	10	76	1
Solyc02g071300.1.1	0	0	0	0	0	0	0	0	0	0	0	0	0

Solyc02g071310.2.1	0	0	0	0	0	0	0	0	0	0	0	0	0
Solyc02g071320.2.1	7	325	23	185	23	252	71	221	48	235	61	199	4
Solyc02g071330.2.1	3	16	5	7	3	27	16	23	3	46	13	19	0
Solyc02g071350.2.1	0	0	0	0	0	0	0	0	0	0	0	0	0
Solyc02g071360.2.1	1	2	6	0	0	4	1	9	0	2	1	0	0
Solyc02g071380.2.1	7	117	26	103	3	3	13	9	0	51	7	65	9
Solyc02g071400.2.1	0	0	0	0	0	0	0	0	0	0	0	0	0
Solyc02g071410.2.1	0	0	0	0	0	0	0	0	0	0	0	0	0
Solyc02g071420.2.1	0	0	0	0	0	0	0	0	0	0	0	0	0
Solyc02g071430.2.1	5	1	3	1	2	123	231	76	19	3	10	7	0
Solyc02g071450.2.1	2	12	0	2	0	34	15	35	0	44	12	45	2
Solyc02g071470.2.1	0	0	0	0	0	0	0	0	0	0	0	0	0
Solyc02g071490.2.1	0	0	0	0	0	0	0	0	0	0	0	0	0
Solyc02g071500.2.1	0	0	0	0	0	0	0	0	0	0	0	0	0
Solyc02g071510.2.1	18	87	19	106	7	183	202	315	29	149	30	119	4
Solyc02g071520.2.1	2	20	0	1	0	19	16	16	1	2	0	0	0
Solyc02g071530.1.1	0	0	0	0	0	0	0	0	0	0	0	0	0
Solyc02g071560.2.1	0	0	0	0	0	0	0	0	0	0	0	0	0
Solyc02g071570.2.1	3	9	0	5	2	17	6	4	4	6	6	3	3
Solyc02g071580.2.1	0	0	0	0	0	0	0	0	0	0	0	0	0
Solyc02g071590.1.1	0	0	0	0	0	0	0	0	0	0	0	0	0
Solyc02g071600.2.1	0	0	0	0	0	0	0	0	0	0	0	0	0
Solyc02g071610.2.1	6	95	16	9	58	8	17	1	43	12	36	16	67
Solyc02g071620.2.1	1	9	2	0	4	16	2	8	13	0	2	0	3
Solyc02g071700.2.1	0	0	0	0	0	0	0	0	0	0	0	0	0
Solyc02g071710.2.1	2	80	11	2	8	1	17	0	9	0	0	0	0
Solyc02g071720.2.1	0	0	0	0	0	0	0	0	0	0	0	0	0
Solyc02g071730.2.1	3	0	0	0	0	0	0	3	0	37	46	54	0
Solyc02g071740.2.1	3	30	0	9	0	21	19	19	0	6	0	8	0
Solyc02g071750.2.1	8	24	1	19	9	94	55	93	8	63	18	48	0
Solyc02g071760.2.1	0	0	0	0	0	0	0	0	0	0	0	0	0
Solyc02g071770.2.1	0	0	0	0	0	0	0	0	0	0	0	0	0
Solyc02g071780.2.1	3	11	3	8	0	30	3	5	1	0	9	16	0
Solyc02g071790.1.1	0	0	0	0	0	0	0	0	0	0	0	0	0
Solyc02g071800.2.1	1	8	0	5	0	1	8	2	0	3	0	0	0
Solyc02g071810.2.1	0	0	0	0	0	0	0	0	0	0	0	0	0
Solyc02g071820.2.1	1	2	0	3	0	6	8	14	2	6	3	0	0
Solyc02g071830.1.1	0	0	0	0	0	0	0	0	0	0	0	0	0
Solyc02g071860.2.1	28	178	11	107	4	364	568	212	49	246	66	177	9
Solyc02g071870.2.1	1	0	2	0	0	0	0	2	0	6	2	7	0
Solyc02g071880.2.1	0	0	0	0	0	0	0	0	0	0	0	0	0

Solyc02g071890.2.1	5	77	10	59	0	126	249	105	13	58	46	77	0
Solyc02g071900.2.1	3	1	0	1	0	8	8	5	0	30	1	8	0
Solyc02g071920.2.1	0	0	0	0	0	0	0	0	0	0	0	0	0
Solyc02g071940.1.1	2	5	2	4	0	6	17	1	3	19	43	15	0
Solyc02g071970.1.1	0	0	0	0	0	0	0	0	0	0	0	0	0
Solyc02g071980.2.1	15	75	4	47	0	118	177	170	47	63	9	74	2
Solyc02g071990.2.1	3	26	4	23	0	60	16	50	9	16	8	6	0
Solyc02g072000.2.1	2	4	2	15	0	7	14	3	0	12	3	6	0
Solyc02g072010.1.1	0	0	0	0	0	0	0	0	0	0	0	0	0
Solyc02g072020.1.1	0	0	0	0	0	0	0	0	0	0	0	0	0
Solyc02g072030.1.1	0	0	0	0	0	0	0	0	0	0	0	0	0
Solyc02g072040.2.1	0	0	0	0	0	0	0	0	0	0	0	0	0
Solyc02g072050.1.1	0	0	0	0	0	0	0	0	0	0	0	0	0
Solyc02g072070.2.1	0	0	0	0	0	0	0	0	0	0	0	0	0
Solyc02g072080.1.1	16	442	115	308	51	206	106	156	53	129	28	191	7
Solyc02g072090.1.1	7	21	4	6	10	55	28	43	1	11	17	23	1
Solyc02g072100.2.1	0	0	0	0	0	0	0	0	0	0	0	0	0
Solyc02g072120.2.1	2	0	0	0	0	7	5	10	0	9	1	2	0
Solyc02g072130.2.1	10	1001	85	555	99	2511	1020	2684	362	1144	496	883	16
Solyc02g072140.1.1	0	0	0	0	0	0	0	0	0	0	0	0	0
Solyc02g072150.2.1	9	105	20	75	6	38	49	56	6	52	6	23	0
Solyc02g072160.2.1	11	204	93	178	17	115	189	132	21	103	85	67	4
Solyc02g072170.2.1	0	0	0	0	0	0	0	0	0	0	0	0	0
Solyc02g072180.2.1	1	3	0	0	0	2	7	5	0	8	3	1	0
Solyc02g072190.2.1	0	0	0	0	0	0	0	0	0	0	0	0	0
Solyc02g072200.1.1	0	0	0	0	0	0	0	0	0	0	0	0	0
Solyc02g072210.1.1	1	2	1	12	4	39	9	4	2	3	1	2	0
Solyc02g072220.1.1	0	0	0	0	0	0	0	0	0	0	0	0	0
Solyc02g072230.2.1	4	17	2	10	0	12	19	51	5	16	11	35	0
Solyc02g072240.2.1	9	63	8	61	0	35	37	63	0	3	12	0	0
Solyc02g072260.2.1	0	0	0	0	0	0	0	0	0	0	0	0	0
Solyc02g072270.2.1	1	5	0	4	0	2	4	2	0	7	0	1	0
Solyc02g072300.2.1	2	71	3	27	1	67	26	95	17	74	12	64	1
Solyc02g072310.2.1	0	0	0	0	0	0	0	0	0	0	0	0	0
Solyc02g072320.1.1	0	0	0	0	0	0	0	0	0	0	0	0	0
Solyc02g072370.1.1	0	0	0	0	0	0	0	0	0	0	0	0	0
Solyc02g072400.1.1	0	0	0	0	0	0	0	0	0	0	0	0	0
Solyc02g072420.1.1	0	0	0	0	0	0	0	0	0	0	0	0	0
Solyc02g072440.2.1	3	0	0	3	0	4	11	6	0	16	7	28	1
Solyc02g072460.1.1	2	27	10	16	0	47	34	29	2	67	1	50	0
Solyc02g072470.2.1	1	3	0	2	0	3	6	7	0	0	0	2	0

Solyc02g072480.2.1	0	0	0	0	0	0	0	0	0	0	0	0	0
Solyc02g072490.2.1	0	0	0	0	0	0	0	0	0	0	0	0	0
Solyc02g072500.2.1	0	0	0	0	0	0	0	0	0	0	0	0	0
Solyc02g072510.2.1	6	34	3	17	3	58	43	82	7	51	0	26	0
Solyc02g072520.2.1	11	80	31	46	9	21	125	22	13	28	18	18	0
Solyc02g072530.1.1	7	134	12	69	7	256	154	279	36	32	24	35	0
Solyc02g072540.2.1	1	15	1	4	0	34	2	11	0	0	1	0	0
Solyc02g072550.1.1	0	0	0	0	0	0	0	0	0	0	0	0	0
Solyc02g072560.1.1	0	0	0	0	0	0	0	0	0	0	0	0	0
Solyc02g072570.1.1	0	0	0	0	0	0	0	0	0	0	0	0	0
Solyc02g073570.1.1	0	0	0	0	0	0	0	0	0	0	0	0	0
Solyc02g073580.1.1	0	0	0	0	0	0	0	0	0	0	0	0	0
Solyc02g075610.2.1	1	35	0	21	0	22	10	70	0	120	0	18	0
Solyc02g075630.1.1	2	46	2	26	1	70	36	67	4	51	12	31	0
Solyc02g076660.2.1	6	20	0	13	2	59	24	27	5	6	0	3	0
Solyc02g076670.2.1	0	0	0	0	0	0	0	0	0	0	0	0	0
Solyc02g076680.2.1	7	6	1	8	1	38	29	27	10	35	11	20	4
Solyc02g076690.2.1	16	182	44	71	3	5	129	37	0	285	40	249	3
Solyc02g076700.1.1	0	0	0	0	0	0	0	0	0	0	0	0	0
Solyc02g076710.2.1	13	0	0	0	0	25	0	70	0	1292	454	754	7
Solyc02g076720.2.1	22	142	47	127	5	219	235	451	25	327	83	279	3
Solyc02g076730.2.1	1	12	0	1	1	0	2	3	4	4	5	0	0
Solyc02g076740.1.1	0	0	0	0	0	0	0	0	0	0	0	0	0
Solyc02g076750.1.1	7	45	4	17	27	17	87	16	11	31	50	28	14
Solyc02g076760.2.1	5	20	4	15	1	79	45	55	6	52	5	39	0
Solyc02g076780.2.1	1	1	0	4	2	2	5	4	0	1	0	1	0
Solyc02g076790.2.1	3	21	3	8	0	25	17	39	5	45	2	17	0
Solyc02g076800.1.1	1	23	0	39	0	47	10	17	0	99	0	86	0
Solyc02g076810.2.1	3	46	3	24	0	163	31	135	3	31	0	29	0
Solyc02g076820.2.1	1	9	0	5	0	4	3	10	0	140	32	171	2
Solyc02g076840.2.1	12	61	14	42	6	50	74	152	19	94	29	88	2
Solyc02g076850.1.1	0	0	0	0	0	0	0	0	0	0	0	0	0
Solyc02g076860.2.1	0	0	0	0	0	0	0	0	0	0	0	0	0
Solyc02g076870.2.1	9	80	7	44	3	297	40	101	15	90	27	65	10
Solyc02g076900.2.1	0	0	0	0	0	0	0	0	0	0	0	0	0
Solyc02g076910.2.1	5	2	3	262	0	554	66	382	0	0	1	0	0
Solyc02g076920.2.1	0	0	0	0	0	0	0	0	0	0	0	0	0
Solyc02g076930.1.1	0	0	0	0	0	0	0	0	0	0	0	0	0
Solyc02g076950.2.1	0	0	0	0	0	0	0	0	0	0	0	0	0
Solyc02g076960.2.1	0	0	0	0	0	0	0	0	0	0	0	0	0
Solyc02g076970.1.1	0	0	0	0	0	0	0	0	0	0	0	0	0

Solyc02g076980.2.1	1	19	1	24	0	3	3	10	1	0	0	0	0
Solyc02g077000.2.1	0	0	0	0	0	0	0	0	0	0	0	0	0
Solyc02g077020.2.1	0	0	0	0	0	0	0	0	0	0	0	0	0
Solyc02g077030.2.1	1	6	0	0	0	4	4	4	0	3	0	0	0
Solyc02g077040.2.1	3	61	7	31	0	102	0	210	3	1	0	4	0
Solyc02g077050.2.1	6	142	8	87	1	5	8	11	1	1	0	10	0
Solyc02g077060.1.1	0	0	0	0	0	0	0	0	0	0	0	0	0
Solyc02g077080.2.1	11	570	6	443	0	715	462	2537	18	1973	17	1241	0
Solyc02g077090.2.1	4	47	8	16	6	3	6	1	2	10	7	15	0
Solyc02g077100.2.1	9	89	1	50	0	130	64	156	2	47	0	74	0
Solyc02g077110.2.1	11	30	5	5	3	75	110	135	91	2	2	8	0
Solyc02g077140.2.1	0	0	0	0	0	0	0	0	0	0	0	0	0
Solyc02g077150.1.1	0	0	0	0	0	0	0	0	0	0	0	0	0
Solyc02g077160.2.1	11	36	5	11	4	15	89	47	2	83	12	63	1
Solyc02g077190.1.1	0	0	0	0	0	0	0	0	0	0	0	0	0
Solyc02g077220.2.1	0	0	0	0	0	0	0	0	0	0	0	0	0
Solyc02g077230.2.1	0	0	0	0	0	0	0	0	0	0	0	0	0
Solyc02g077240.2.1	6	0	0	4	0	11	9	17	2	210	22	215	6
Solyc02g077250.2.1	0	0	0	0	0	0	0	0	0	0	0	0	0
Solyc02g077260.2.1	0	0	0	0	0	0	0	0	0	0	0	0	0
Solyc02g077270.2.1	0	0	0	0	0	0	0	0	0	0	0	0	0
Solyc02g077280.2.1	2	6	0	6	0	1	8	14	0	9	2	11	0
Solyc02g077290.1.1	0	0	0	0	0	0	0	0	0	0	0	0	0
Solyc02g077300.1.1	0	0	0	0	0	0	0	0	0	0	0	0	0
Solyc02g077310.2.1	0	0	0	0	0	0	0	0	0	0	0	0	0
Solyc02g077320.2.1	1	0	0	2	0	0	7	3	0	10	3	4	0
Solyc02g077330.2.1	21	236	6	0	14	569	759	171	1304	280	24	58	24
Solyc02g077370.1.1	1	27	2	4	3	13	3	7	2	12	0	44	0
Solyc02g077400.2.1	2	22	0	24	0	22	12	51	0	3	0	4	0
Solyc02g077410.2.1	2	51	5	40	0	89	13	113	1	116	12	93	0
Solyc02g077420.2.1	4	67	3	45	2	55	8	247	3	9	0	3	0
Solyc02g077430.2.1	4	26	14	11	16	28	35	85	0	1	0	2	0
Solyc02g077440.2.1	0	0	0	0	0	0	0	0	0	0	0	0	0
Solyc02g077450.2.1	11	103	13	77	11	178	232	269	33	313	53	210	0
Solyc02g077460.1.1	0	0	0	0	0	0	0	0	0	0	0	0	0
Solyc02g077470.2.1	2	17	4	4	0	6	3	5	0	0	0	3	0
Solyc02g077480.1.1	0	0	0	0	0	0	0	0	0	0	0	0	0
Solyc02g077510.2.1	0	0	0	0	0	0	0	0	0	0	0	0	0
Solyc02g077550.2.1	0	0	0	0	0	0	0	0	0	0	0	0	0
Solyc02g077560.2.1	17	121	12	99	23	259	126	390	32	614	41	384	4
Solyc02g077590.1.1	5	19	5	23	0	29	15	25	0	30	9	6	0

Solyc02g077600.2.1	8	20	6	15	6	20	40	61	8	19	15	17	0
Solyc02g077610.2.1	0	0	0	0	0	0	0	0	0	0	0	0	0
Solyc02g077620.1.1	1	2	0	2	0	7	9	6	1	3	0	1	0
Solyc02g077630.2.1	5	31	1	12	2	71	16	34	8	108	49	69	0
Solyc02g077640.1.1	0	0	0	0	0	0	0	0	0	0	0	0	0
Solyc02g077650.1.1	0	0	0	0	0	0	0	0	0	0	0	0	0
Solyc02g077660.2.1	38	223	21	185	11	439	217	507	53	367	88	292	8
Solyc02g077670.2.1	11	44	8	53	13	70	70	106	5	104	72	112	2
Solyc02g077680.2.1	9	58	6	49	3	119	55	143	7	137	23	115	1
Solyc02g077690.2.1	3	6	2	4	3	1	24	20	2	26	9	17	6
Solyc02g077700.1.1	2	11	1	0	0	2	3	7	1	7	1	20	1
Solyc02g077710.1.1	18	832	108	635	254	275	322	520	182	2210	421	224	145
Solyc02g077720.2.1	2	15	3	8	0	34	0	19	5	31	5	2	0
Solyc02g077730.2.1	1	4	0	0	0	0	1	7	0	4	3	7	0
Solyc02g077740.2.1	1	5	2	9	0	15	3	17	1	13	0	9	0
Solyc02g077750.2.1	1	8	0	17	0	3	2	15	0	22	1	31	0
Solyc02g077760.1.1	0	0	0	0	0	0	0	0	0	0	0	0	0
Solyc02g077770.2.1	0	0	0	0	0	0	0	0	0	0	0	0	0
Solyc02g077780.2.1	7	29	5	24	2	73	54	69	7	69	6	53	1
Solyc02g077840.1.1	0	0	0	0	0	0	0	0	0	0	0	0	0
Solyc02g077850.2.1	6	30	0	23	0	10	26	34	1	36	7	41	0
Solyc02g077880.2.1	1	135	7	143	6	52	102	231	7	462	10	257	0
Solyc02g077890.2.1	1	3	1	0	0	5	17	4	1	5	1	3	2
Solyc02g077900.2.1	8	38	2	22	0	73	37	92	6	76	20	37	1
Solyc02g077910.2.1	0	0	0	0	0	0	0	0	0	0	0	0	0
Solyc02g077920.2.1	0	0	0	0	0	0	0	0	0	0	0	0	0
Solyc02g077930.1.1	0	0	0	0	0	0	0	0	0	0	0	0	0
Solyc02g077940.1.1	0	0	0	0	0	0	0	0	0	0	0	0	0
Solyc02g077950.1.1	3	10	0	13	0	10	15	46	0	2	0	0	0
Solyc02g077960.1.1	0	0	0	0	0	0	0	0	0	0	0	0	0
Solyc02g077970.2.1	10	2	0	0	0	675	178	165	15	1	23	1	2
Solyc02g077980.1.1	5	2	0	0	0	79	65	38	3	0	2	0	0
Solyc02g077990.2.1	7	861	52	468	5	257	317	421	9	283	48	181	4
Solyc02g078000.2.1	8	36	4	20	2	61	45	58	6	45	14	21	1
Solyc02g078010.2.1	0	0	0	0	0	0	0	0	0	0	0	0	0
Solyc02g078020.2.1	13	52	1	56	1	205	132	230	41	69	34	85	3
Solyc02g078030.1.1	6	164	15	74	9	154	33	216	20	110	39	76	4
Solyc02g078040.2.1	14	286	6	198	1	115	66	213	0	291	171	438	238
Solyc02g078070.1.1	0	0	0	0	0	0	0	0	0	0	0	0	0
Solyc02g078080.1.1	0	0	0	0	0	0	0	0	0	0	0	0	0
Solyc02g078100.2.1	0	0	0	0	0	0	0	0	0	0	0	0	0

Solyc02g078110.1.1	0	0	0	0	0	0	0	0	0	0	0	0	0
Solyc02g078120.1.1	20	390	28	175	20	790	344	912	78	672	200	624	9
Solyc02g078130.2.1	0	0	0	0	0	0	0	0	0	0	0	0	0
Solyc02g078140.2.1	14	490	65	259	6	432	254	538	47	234	59	233	6
Solyc02g078150.2.1	12	2	0	1	2	14	410	47	2	211	9	428	26
Solyc02g078160.2.1	6	9	2	2	2	42	53	51	0	68	3	173	2
Solyc02g078170.1.1	0	0	0	0	0	0	0	0	0	0	0	0	0
Solyc02g078180.2.1	0	0	0	0	0	0	0	0	0	0	0	0	0
Solyc02g078200.2.1	5	15	0	8	0	48	14	24	1	19	10	35	4
Solyc02g078210.2.1	31	543	153	537	14	739	598	1341	33	428	172	937	14
Solyc02g078220.1.1	19	50	5	31	15	419	64	225	11	150	48	112	9
Solyc02g078230.1.1	13	48	12	88	1	101	81	221	8	82	28	54	3
Solyc02g078240.2.1	0	0	0	0	0	0	0	0	0	0	0	0	0
Solyc02g078250.2.1	0	0	0	0	0	0	0	0	0	0	0	0	0
Solyc02g078260.2.1	23	252	23	215	10	536	238	730	52	654	72	439	4
Solyc02g078270.2.1	8	17	2	14	0	57	28	74	8	63	10	42	3
Solyc02g078300.2.1	0	0	0	0	0	0	0	0	0	0	0	0	0
Solyc02g078320.1.1	7	28	6	11	6	63	72	94	33	11	11	15	4
Solyc02g078330.2.1	1	0	1	0	0	0	7	6	4	5	1	0	0
Solyc02g078340.2.1	0	0	0	0	0	0	0	0	0	0	0	0	0
Solyc02g078350.2.1	0	0	0	0	0	0	0	0	0	0	0	0	0
Solyc02g078360.2.1	9	26	1	16	4	66	27	98	5	63	23	54	0
Solyc02g078380.2.1	5	23	34	19	1	44	316	97	5	260	137	188	18
Solyc02g078390.2.1	8	36	6	28	0	59	59	52	0	22	3	23	0
Solyc02g078400.2.1	14	598	28	361	0	626	17	596	8	134	97	11	0
Solyc02g078410.1.1	0	0	0	0	0	0	0	0	0	0	0	0	0
Solyc02g078440.2.1	3	27	2	12	3	82	29	56	2	23	14	34	0
Solyc02g078450.2.1	3	49	4	13	0	11	8	26	0	1	0	2	0
Solyc02g078460.2.1	6	24	1	38	7	59	56	132	11	152	29	106	3
Solyc02g078470.2.1	1	3	0	2	0	2	3	10	0	5	0	2	0
Solyc02g078480.2.1	2	25	3	0	0	38	0	14	0	21	0	10	0
Solyc02g078490.2.1	0	0	0	0	0	0	0	0	0	0	0	0	0
Solyc02g078500.2.1	0	0	0	0	0	0	0	0	0	0	0	0	0
Solyc02g078510.2.1	0	0	0	0	0	0	0	0	0	0	0	0	0
Solyc02g078520.2.1	12	67	21	65	3	173	98	262	27	140	38	135	1
Solyc02g078530.2.1	1	3	2	0	0	1	5	3	0	3	3	6	0
Solyc02g078540.2.1	6	625	66	400	55	1606	667	998	155	2769	222	1335	29
Solyc02g078550.2.1	7	21	1	20	4	54	36	77	9	40	8	21	1
Solyc02g078560.2.1	12	74	8	60	4	114	74	171	15	136	46	73	1
Solyc02g078570.2.1	5	10	1	3	1	2	7	7	4	611	68	244	105
Solyc02g078580.1.1	9	41	11	27	4	59	115	141	18	116	38	89	0

Solyc02g078590.1.1	8	26	0	5	8	28	10	30	1	190	66	133	4
Solyc02g078600.2.1	6	26	1	19	1	17	24	23	3	30	6	12	5
Solyc02g078610.2.1	6	58	5	24	3	42	51	81	12	94	20	85	2
Solyc02g078620.1.1	0	0	0	0	0	0	0	0	0	0	0	0	0
Solyc02g078630.2.1	1	21	0	7	0	16	2	23	2	11	1	30	0
Solyc02g078640.2.1	21	103	14	60	4	108	99	133	15	174	58	177	4
Solyc02g078650.2.1	4	7	0	0	0	167	12	103	5	1	1	0	0
Solyc02g078660.2.1	1	2	0	4	0	4	0	12	0	0	0	2	0
Solyc02g078670.2.1	18	371	16	173	27	109	81	150	26	538	83	474	6
Solyc02g078700.2.1	0	0	0	0	0	0	0	0	0	0	0	0	0
Solyc02g078720.2.1	0	0	0	0	0	0	0	0	0	0	0	0	0
Solyc02g078730.2.1	0	0	0	0	0	0	0	0	0	0	0	0	0
Solyc02g078740.2.1	0	0	0	0	0	0	0	0	0	0	0	0	0
Solyc02g078750.2.1	4	55	8	43	2	41	19	46	0	27	36	20	3
Solyc02g078760.1.1	2	27	7	4	2	0	27	4	3	14	8	6	0
Solyc02g078780.2.1	3	35	5	23	3	37	191	66	14	61	27	35	2
Solyc02g078790.2.1	10	48	2	21	3	76	68	69	11	42	19	30	0
Solyc02g078800.2.1	15	97	13	116	4	210	306	129	27	190	26	105	1
Solyc02g078810.2.1	1	1	1	0	0	3	3	3	0	1	1	1	0
Solyc02g078820.2.1	3	7	0	7	0	43	19	42	6	22	0	15	0
Solyc02g078830.2.1	4	7	1	2	1	9	14	15	3	27	7	41	0
Solyc02g078840.2.1	5	16	0	7	1	50	24	68	7	35	17	39	1
Solyc02g078850.1.1	4	38	2	7	15	0	35	10	46	0	24	1	13
Solyc02g078860.2.1	2	12	0	15	0	17	13	48	0	9	4	27	0
Solyc02g078880.2.1	10	45	2	27	6	55	33	101	2	67	23	67	4
Solyc02g078890.1.1	0	0	0	0	0	0	0	0	0	0	0	0	0
Solyc02g078900.2.1	5	42	1	12	5	121	14	107	4	109	15	55	0
Solyc02g078910.2.1	0	0	0	0	0	0	0	0	0	0	0	0	0
Solyc02g078920.2.1	8	11	7	1	4	8	37	15	10	54	11	39	0
Solyc02g078930.1.1	0	0	0	0	0	0	0	0	0	0	0	0	0
Solyc02g078940.2.1	14	295	5	227	6	2112	383	1656	40	1433	202	809	8
Solyc02g078950.2.1	15	37	11	26	0	37	93	56	1	179	10	192	0
Solyc02g078960.2.1	7	55	17	26	5	57	90	64	10	104	47	75	1
Solyc02g078970.2.1	11	71	2	44	5	80	80	185	21	96	13	109	6
Solyc02g078980.2.1	0	0	0	0	0	0	0	0	0	0	0	0	0
Solyc02g078990.2.1	6	169	15	307	17	685	102	413	28	412	25	309	4
Solyc02g079000.2.1	5	6	2	5	2	9	19	15	8	15	3	12	6
Solyc02g079010.2.1	2	22	5	3	0	2	3	3	1	3	11	10	0
Solyc02g079020.2.1	10	11	13	26	2	34	79	57	16	32	21	52	6
Solyc02g079030.2.1	8	17	5	5	0	119	64	60	9	22	17	4	0
Solyc02g079040.2.1	4	74	9	54	4	110	39	81	7	20	3	55	4

Solyc02g079050.2.1	2	6	0	6	0	71	23	26	1	50	6	19	0
Solyc02g079060.2.1	6	79	14	54	1	206	107	202	23	273	49	131	6
Solyc02g079070.2.1	0	0	0	0	0	0	0	0	0	0	0	0	0
Solyc02g079080.1.1	0	0	0	0	0	0	0	0	0	0	0	0	0
Solyc02g079090.2.1	2	2	2	1	2	2	9	5	1	11	4	2	0
Solyc02g079100.2.1	9	50	0	45	3	138	55	124	10	85	21	81	0
Solyc02g079110.2.1	2	8	6	15	0	153	175	137	17	16	12	13	0
Solyc02g079130.2.1	3	10	0	15	0	11	22	23	6	17	21	7	1
Solyc02g079140.1.1	0	0	0	0	0	0	0	0	0	0	0	0	0
Solyc02g079160.2.1	0	0	0	0	0	0	0	0	0	0	0	0	0
Solyc02g079170.2.1	15	69	11	41	5	327	162	313	53	12	109	27	3
Solyc02g079180.1.1	0	0	0	0	0	0	0	0	0	0	0	0	0
Solyc02g079190.2.1	10	110	24	107	2	88	71	133	7	159	15	152	2
Solyc02g079200.1.1	3	42	1	40	0	25	18	24	4	12	2	7	0
Solyc02g079210.2.1	9	52	1	35	6	69	150	143	15	155	56	107	2
Solyc02g079220.2.1	9	158	10	113	26	71	51	40	22	58	14	39	1
Solyc02g079230.2.1	5	47	0	37	0	71	21	67	2	36	2	47	0
Solyc02g079240.1.1	0	0	0	0	0	0	0	0	0	0	0	0	0
Solyc02g079250.2.1	0	0	0	0	0	0	0	0	0	0	0	0	0
Solyc02g079260.1.1	1	0	0	0	0	4	7	4	0	0	0	0	0
Solyc02g079270.2.1	0	0	0	0	0	0	0	0	0	0	0	0	0
Solyc02g079280.2.1	9	0	0	0	5	141	107	23	84	0	1	0	1
Solyc02g079290.2.1	6	4	0	4	2	54	38	30	3	14	3	17	0
Solyc02g079300.2.1	0	0	0	0	0	0	0	0	0	0	0	0	0
Solyc02g079320.2.1	1	2	8	1	0	4	19	9	0	16	0	1	0
Solyc02g079330.2.1	0	0	0	0	0	0	0	0	0	0	0	0	0
Solyc02g079340.2.1	0	0	0	0	0	0	0	0	0	0	0	0	0
Solyc02g079350.2.1	0	0	0	0	0	0	0	0	0	0	0	0	0
Solyc02g079360.1.1	8	84	3	31	0	16	29	25	1	1	3	7	0
Solyc02g079370.2.1	0	0	0	0	0	0	0	0	0	0	0	0	0
Solyc02g079380.1.1	0	0	0	0	0	0	0	0	0	0	0	0	0
Solyc02g079390.2.1	2	12	1	4	0	6	13	28	10	22	0	5	0
Solyc02g079400.2.1	4	269	124	295	106	2868	2605	961	328	589	453	396	22
Solyc02g079410.2.1	3	9	0	16	0	11	8	21	4	12	7	13	0
Solyc02g079430.2.1	3	20	2	2	1	51	35	23	0	7	1	11	0
Solyc02g079440.1.1	1	1	0	0	0	57	8	7	4	2	8	0	0
Solyc02g079460.1.1	0	0	0	0	0	0	0	0	0	0	0	0	0
Solyc02g079470.1.1	0	0	0	0	0	0	0	0	0	0	0	0	0
Solyc02g079480.1.1	0	0	0	0	0	0	0	0	0	0	0	0	0
Solyc02g079490.2.1	1	9	0	5	0	0	0	0	0	0	7	0	0
Solyc02g079500.2.1	7	65	4	28	2	57	59	61	4	791	20	1128	3

Solyc02g079510.2.1	1	2	0	7	0	2	1	2	0	3	3	5	0
Solyc02g079520.1.1	1	37	8	21	0	0	3	8	0	0	0	0	0
Solyc02g079530.2.1	0	0	0	0	0	0	0	0	0	0	0	0	0
Solyc02g079540.1.1	0	0	0	0	0	0	0	0	0	0	0	0	0
Solyc02g079550.1.1	0	0	0	0	0	0	0	0	0	0	0	0	0
Solyc02g079560.1.1	0	0	0	0	0	0	0	0	0	0	0	0	0
Solyc02g079570.2.1	5	53	19	54	6	56	11	24	6	2	2	9	0
Solyc02g079580.2.1	0	0	0	0	0	0	0	0	0	0	0	0	0
Solyc02g079590.2.1	12	39	8	20	7	139	109	68	146	47	8	46	5
Solyc02g079610.2.1	0	0	0	0	0	0	0	0	0	0	0	0	0
Solyc02g079640.2.1	4	8	0	5	4	21	16	3	17	15	7	23	0
Solyc02g079650.2.1	2	5	0	0	0	10	14	2	18	10	2	5	1
Solyc02g079710.2.1	0	0	0	0	0	0	0	0	0	0	0	0	0
Solyc02g079730.2.1	3	12	0	6	0	13	19	25	0	12	0	28	0
Solyc02g079740.1.1	7	37	4	31	1	49	58	58	26	36	17	51	0
Solyc02g079750.2.1	1	22	4	12	4	50	7	63	26	15	82	23	6
Solyc02g079760.2.1	3	21	2	13	0	97	15	41	0	11	3	5	0
Solyc02g079770.2.1	5	128	0	83	4	101	64	253	4	48	5	31	1
Solyc02g079780.1.1	15	250	10	129	1	40	83	161	0	356	39	316	5
Solyc02g079790.2.1	1	20	0	10	1	2	4	4	0	58	3	41	0
Solyc02g079800.1.1	0	0	0	0	0	0	0	0	0	0	0	0	0
Solyc02g079820.2.1	0	0	0	0	0	0	0	0	0	0	0	0	0
Solyc02g079830.1.1	1	0	0	7	0	16	4	8	2	21	0	62	0
Solyc02g079850.2.1	20	71	12	67	10	214	133	218	22	358	60	164	3
Solyc02g079860.1.1	0	0	0	0	0	0	0	0	0	0	0	0	0
Solyc02g079870.2.1	0	0	0	0	0	0	0	0	0	0	0	0	0
Solyc02g079880.2.1	23	179	23	123	8	306	162	401	50	378	126	238	10
Solyc02g079890.1.1	0	0	0	0	0	0	0	0	0	0	0	0	0
Solyc02g079920.2.1	0	0	0	0	0	0	0	0	0	0	0	0	0
Solyc02g079930.2.1	2	8	0	9	1	80	0	19	12	240	9	161	66
Solyc02g079940.2.1	0	0	0	0	0	0	0	0	0	0	0	0	0
Solyc02g079950.2.1	3	1400	92	600	2	44	161	61	1	444	64	322	1
Solyc02g079960.2.1	3	59	4	38	0	77	74	85	3	160	4	139	0
Solyc02g079970.2.1	4	152	13	118	1	46	36	82	5	133	67	241	4
Solyc02g079980.2.1	0	0	0	0	0	0	0	0	0	0	0	0	0
Solyc02g079990.2.1	0	0	0	0	0	0	0	0	0	0	0	0	0
Solyc02g080010.2.1	0	0	0	0	0	0	0	0	0	0	0	0	0
Solyc02g080020.1.1	0	0	0	0	0	0	0	0	0	0	0	0	0
Solyc02g080030.1.1	0	0	0	0	0	0	0	0	0	0	0	0	0
Solyc02g080040.2.1	0	0	0	0	0	0	0	0	0	0	0	0	0
Solyc02g080050.1.1	0	0	0	0	0	0	0	0	0	0	0	0	0

Solyc02g080070.2.1	4	81	4	48	10	81	8	71	5	19	6	9	1
Solyc02g080080.2.1	0	0	0	0	0	0	0	0	0	0	0	0	0
Solyc02g080100.1.1	0	0	0	0	0	0	0	0	0	0	0	0	0
Solyc02g080110.2.1	2	6	1	5	4	28	9	20	8	8	7	1	0
Solyc02g080120.1.1	5	14	0	1	1	211	31	324	32	2	0	0	0
Solyc02g080130.2.1	2	8	0	4	1	62	18	38	3	26	9	16	2
Solyc02g080140.2.1	0	0	0	0	0	0	0	0	0	0	0	0	0
Solyc02g080150.1.1	0	0	0	0	0	0	0	0	0	0	0	0	0
Solyc02g080160.2.1	2	3	1	11	0	0	0	0	0	53	10	40	0
Solyc02g080170.1.1	0	0	0	0	0	0	0	0	0	0	0	0	0
Solyc02g080180.1.1	0	0	0	0	0	0	0	0	0	0	0	0	0
Solyc02g080190.2.1	0	0	0	0	0	0	0	0	0	0	0	0	0
Solyc02g080210.2.1	1	183	25	247	0	0	88	0	0	0	1	0	3
Solyc02g080220.2.1	0	0	0	0	0	0	0	0	0	0	0	0	0
Solyc02g080230.2.1	0	0	0	0	0	0	0	0	0	0	0	0	0
Solyc02g080260.2.1	0	0	0	0	0	0	0	0	0	0	0	0	0
Solyc02g080270.2.1	2	19	0	6	0	32	11	9	0	20	15	39	0
Solyc02g080290.2.1	2	1	2	0	0	0	13	6	1	7	1	9	0
Solyc02g080300.2.1	3	3	1	9	0	34	11	36	0	9	6	8	0
Solyc02g080310.2.1	1	5	1	8	0	2	5	3	2	0	4	1	0
Solyc02g080320.2.1	2	18	1	7	0	37	24	38	0	3	1	32	0
Solyc02g080330.2.1	2	6	0	0	5	48	0	0	6	0	9	0	0
Solyc02g080340.2.1	1	4	1	1	0	44	3	13	0	15	1	19	0
Solyc02g080350.1.1	0	0	0	0	0	0	0	0	0	0	0	0	0
Solyc02g080360.1.1	0	0	0	0	0	0	0	0	0	0	0	0	0
Solyc02g080370.2.1	8	79	8	56	5	193	107	247	7	174	42	229	3
Solyc02g080380.2.1	5	32	7	2	7	64	22	60	5	69	12	76	1
Solyc02g080390.2.1	0	0	0	0	0	0	0	0	0	0	0	0	0
Solyc02g080400.1.1	0	0	0	0	0	0	0	0	0	0	0	0	0
Solyc02g080410.2.1	1	35	1	24	2	6	6	18	0	2	0	0	0
Solyc02g080420.2.1	11	537	44	515	22	1901	216	1027	66	2462	156	2333	19
Solyc02g080430.2.1	4	39	9	5	8	54	85	36	24	31	43	19	1
Solyc02g080440.2.1	13	272	24	201	8	303	258	242	16	308	64	227	22
Solyc02g080450.1.1	0	0	0	0	0	0	0	0	0	0	0	0	0
Solyc02g080470.2.1	9	86	4	35	0	107	51	135	11	111	10	120	0
Solyc02g080480.2.1	4	12	3	9	0	0	10	6	1	11	10	13	5
Solyc02g080490.2.1	0	0	0	0	0	0	0	0	0	0	0	0	0
Solyc02g080500.2.1	6	41	8	13	3	22	5	24	6	31	26	37	2
Solyc02g080510.1.1	7	259	2	130	6	895	49	658	8	708	27	177	0
Solyc02g080520.2.1	4	4	0	6	2	50	14	54	2	5	2	3	0
Solyc02g080530.2.1	6	1638	37	1159	31	1975	313	1347	54	1127	136	886	21

Solyc02g080540.1.1	9	1557	232	996	0	101	263	357	2	284	120	366	4
Solyc02g080550.1.1	0	0	0	0	0	0	0	0	0	0	0	0	0
Solyc02g080560.1.1	0	0	0	0	0	0	0	0	0	0	0	0	0
Solyc02g080570.2.1	32	380	91	326	8	204	242	266	15	164	101	263	13
Solyc02g080580.1.1	0	0	0	0	0	0	0	0	0	0	0	0	0
Solyc02g080590.2.1	1	12	1	26	5	46	21	41	7	29	3	33	0
Solyc02g080600.1.1	1	26	2	27	0	8	8	3	0	4	0	3	0
Solyc02g080610.2.1	2	81	10	61	0	165	46	9	6	72	27	41	1
Solyc02g080620.2.1	0	0	0	0	0	0	0	0	0	0	0	0	0
Solyc02g080630.2.1	9	296	29	159	17	709	434	785	68	930	268	893	17
Solyc02g080640.2.1	8	636	148	291	10	145	18	191	4	112	115	53	0
Solyc02g080650.2.1	6	16	1	4	1	20	25	25	7	23	23	30	0
Solyc02g080660.2.1	2	15	3	8	7	6	11	10	2	9	0	6	0
Solyc02g080670.2.1	0	0	0	0	0	0	0	0	0	0	0	0	0
Solyc02g080770.2.1	0	0	0	0	0	0	0	0	0	0	0	0	0
Solyc02g080780.2.1	5	195	21	97	8	135	79	151	7	41	12	43	0
Solyc02g080800.2.1	3	19	1	2	2	65	30	65	4	38	3	48	0
Solyc02g080810.2.1	11	2827	282	1645	15	474	619	509	7	728	224	795	13
Solyc02g080820.2.1	5	15	4	19	7	61	48	64	6	49	30	44	4
Solyc02g080880.2.1	20	313	67	144	52	1505	1534	1883	436	293	195	187	12
Solyc02g080890.2.1	4	17	0	2	5	71	25	23	31	1	5	8	0
Solyc02g080900.2.1	0	0	0	0	0	0	0	0	0	0	0	0	0
Solyc02g080940.2.1	4	29	2	29	1	78	22	63	2	73	21	101	2
Solyc02g080950.1.1	0	0	0	0	0	0	0	0	0	0	0	0	0
Solyc02g080960.1.1	0	0	0	0	0	0	0	0	0	0	0	0	0
Solyc02g080970.2.1	5	22	1	3	3	94	33	81	12	24	16	39	0
Solyc02g080980.2.1	3	14	1	5	0	8	8	4	1	14	4	1	0
Solyc02g080990.1.1	3	10	0	2	0	1	12	12	1	5	9	3	2
Solyc02g081000.1.1	0	0	0	0	0	0	0	0	0	0	0	0	0
Solyc02g081010.1.1	7	35	3	20	3	114	50	48	5	58	11	42	0
Solyc02g081030.2.1	17	269	9	150	54	2865	336	1779	409	415	24	182	22
Solyc02g081040.2.1	0	0	0	0	0	0	0	0	0	0	0	0	0
Solyc02g081050.2.1	0	0	0	0	0	0	0	0	0	0	0	0	0
Solyc02g081060.2.1	0	0	0	0	0	0	0	0	0	0	0	0	0
Solyc02g081070.2.1	6	43	1	26	2	12	20	42	12	83	12	109	1
Solyc02g081080.2.1	5	25	0	16	0	38	27	33	4	18	10	18	0
Solyc02g081120.2.1	1	5	0	0	0	0	0	0	0	3	9	5	0
Solyc02g081130.1.1	0	0	0	0	0	0	0	0	0	0	0	0	0
Solyc02g081140.2.1	2	22	3	9	0	20	8	36	9	18	1	21	0
Solyc02g081150.1.1	0	0	0	0	0	0	0	0	0	0	0	0	0
Solyc02g081160.2.1	14	294	24	203	29	275	222	773	59	1274	144	966	25

Solyc02g081170.2.1	13	728	213	414	23	2705	1271	2231	167	160	116	174	5
Solyc02g081180.1.1	0	0	0	0	0	0	0	0	0	0	0	0	0
Solyc02g081190.2.1	5	433	68	265	14	613	469	660	16	96	91	118	6
Solyc02g081200.1.1	7	33	5	20	3	58	55	115	5	61	11	50	0
Solyc02g081210.2.1	0	0	0	0	0	0	0	0	0	0	0	0	0
Solyc02g081220.1.1	0	0	0	0	0	0	0	0	0	0	0	0	0
Solyc02g081230.2.1	0	0	0	0	0	0	0	0	0	0	0	0	0
Solyc02g081250.1.1	0	0	0	0	0	0	0	0	0	0	0	0	0
Solyc02g081260.1.1	0	0	0	0	0	0	0	0	0	0	0	0	0
Solyc02g081270.2.1	0	0	0	0	0	0	0	0	0	0	0	0	0
Solyc02g081290.2.1	7	83	5	43	1	136	81	180	23	98	22	58	2
Solyc02g081300.2.1	0	0	0	0	0	0	0	0	0	0	0	0	0
Solyc02g081310.2.1	6	37	2	21	0	65	26	80	0	37	8	22	0
Solyc02g081320.2.1	5	19	6	12	5	51	80	26	3	69	22	49	4
Solyc02g081330.2.1	16	628	160	281	8	1358	1344	586	42	122	60	133	7
Solyc02g081340.2.1	0	0	0	0	0	0	0	0	0	0	0	0	0
Solyc02g081350.2.1	0	0	0	0	0	0	0	0	0	0	0	0	0
Solyc02g081360.2.1	0	0	0	0	0	0	0	0	0	0	0	0	0
Solyc02g081370.2.1	0	0	0	0	0	0	0	0	0	0	0	0	0
Solyc02g081380.2.1	1	16	1	1	0	9	1	10	3	16	5	15	1
Solyc02g081390.2.1	9	153	27	47	15	89	172	132	32	506	224	464	22
Solyc02g081400.2.1	8	221	27	129	24	465	573	461	81	867	212	688	14
Solyc02g081410.2.1	6	35	4	21	4	63	46	48	10	74	5	58	1
Solyc02g081420.2.1	0	0	0	0	0	0	0	0	0	0	0	0	0
Solyc02g081430.2.1	4	225	14	34	1	320	171	227	109	175	48	90	6
Solyc02g081440.2.1	0	0	0	0	0	0	0	0	0	0	0	0	0
Solyc02g081450.2.1	5	28	2	5	2	21	18	11	52	9	3	33	1
Solyc02g081460.2.1	8	27	3	6	0	16	39	46	4	77	5	108	4
Solyc02g081480.2.1	2	3	2	0	3	6	5	0	16	3	3	1	2
Solyc02g081490.2.1	0	0	0	0	0	0	0	0	0	0	0	0	0
Solyc02g081500.2.1	0	0	0	0	0	0	0	0	0	0	0	0	0
Solyc02g081510.1.1	0	0	0	0	0	0	0	0	0	0	0	0	0
Solyc02g081520.2.1	3	6	2	5	0	27	59	21	1	17	4	25	2
Solyc02g081530.1.1	0	0	0	0	0	0	0	0	0	0	0	0	0
Solyc02g081550.2.1	1	0	0	5	0	4	4	1	0	0	0	0	0
Solyc02g081560.1.1	2	62	5	63	0	26	3	20	0	22	5	20	0
Solyc02g081570.2.1	2	0	0	1	3	12	3	0	6	3	1	1	0
Solyc02g081580.2.1	4	69	7	45	2	95	50	107	9	329	14	266	8
Solyc02g081590.2.1	26	208	27	242	11	662	389	649	73	335	116	256	9
Solyc02g081600.2.1	3	23	1	23	0	26	9	28	0	91	2	56	0
Solyc02g081610.1.1	1	0	1	0	0	1	5	3	0	9	0	2	0

Solyc02g081620.2.1	1	5	1	6	0	5	7	9	4	4	8	7	0
Solyc02g081640.2.1	8	57	6	26	0	79	25	112	7	139	11	74	0
Solyc02g081650.2.1	6	17	2	10	0	17	31	44	5	115	16	79	0
Solyc02g081660.2.1	0	0	0	0	0	0	0	0	0	0	0	0	0
Solyc02g081670.1.1	0	0	0	0	0	0	0	0	0	0	0	0	0
Solyc02g081680.2.1	4	19	0	5	0	98	22	86	10	34	4	30	0
Solyc02g081690.1.1	0	0	0	0	0	0	0	0	0	0	0	0	0
Solyc02g081700.1.1	6	119	19	92	16	309	231	299	63	228	96	228	9
Solyc02g081730.2.1	9	188	16	69	7	20	36	26	0	79	55	66	1
Solyc02g081770.1.1	0	0	0	0	0	0	0	0	0	0	0	0	0
Solyc02g081810.2.1	12	93	5	26	2	263	98	353	32	228	92	204	0
Solyc02g081830.2.1	0	0	0	0	0	0	0	0	0	0	0	0	0
Solyc02g081840.2.1	2	4	0	0	0	10	3	8	2	11	5	6	1
Solyc02g081850.2.1	8	72	9	28	2	24	88	82	4	28	12	1	0
Solyc02g081860.1.1	0	0	0	0	0	0	0	0	0	0	0	0	0
Solyc02g081880.2.1	8	185	34	175	18	169	569	333	59	704	127	448	24
Solyc02g081890.2.1	3	11	0	3	0	4	21	14	18	2	2	1	0
Solyc02g081900.2.1	1	1	0	0	0	10	4	4	0	2	0	0	0
Solyc02g081910.1.1	3	2	1	1	0	15	32	28	0	8	2	10	0
Solyc02g081920.2.1	4	13	0	9	0	3	27	18	0	23	13	29	1
Solyc02g081930.2.1	0	0	0	0	0	0	0	0	0	0	0	0	0
Solyc02g081940.2.1	5	22	0	9	0	46	25	50	7	40	9	43	2
Solyc02g081950.2.1	4	21	2	8	0	59	17	27	1	36	8	31	0
Solyc02g081960.2.1	8	27	2	21	4	54	56	93	3	46	9	42	0
Solyc02g081970.2.1	12	100	18	115	4	136	38	283	20	11	19	8	0
Solyc02g081980.2.1	3	74	2	14	0	3	15	6	0	75	5	102	0
Solyc02g082000.2.1	2	257	12	198	10	605	121	656	54	471	84	583	13
Solyc02g082010.1.1	6	19	3	15	0	14	15	41	0	7	2	20	0
Solyc02g082020.2.1	2	8	6	9	0	24	29	24	12	19	16	12	3
Solyc02g082030.2.1	6	34	3	12	1	52	24	53	1	31	8	26	1
Solyc02g082050.2.1	2	4	0	6	3	3	7	3	0	1	2	0	0
Solyc02g082060.1.1	4	124	7	87	0	80	50	226	0	31	1	17	0
Solyc02g082070.2.1	5	1	9	23	0	23	120	46	0	4	8	0	0
Solyc02g082080.1.1	6	189	2	80	0	98	53	103	3	101	9	137	1
Solyc02g082100.2.1	6	51	8	24	0	21	22	16	0	14	9	25	0
Solyc02g082110.2.1	2	6	0	0	0	16	11	12	0	9	0	9	0
Solyc02g082120.2.1	4	52	6	49	5	65	49	58	4	15	2	2	0
Solyc02g082130.1.1	5	83	18	35	1	160	70	195	28	179	40	64	0
Solyc02g082140.2.1	21	718	49	491	23	547	178	725	94	1420	165	502	1
Solyc02g082150.1.1	0	0	0	0	0	0	0	0	0	0	0	0	0
Solyc02g082160.1.1	1	0	0	3	0	0	4	0	0	14	1	22	0

Solyc02g082170.1.1	0	0	0	0	0	0	0	0	0	0	0	0	0
Solyc02g082180.2.1	2	13	0	5	0	4	0	7	0	10	3	11	0
Solyc02g082190.2.1	2	3	0	1	0	7	10	22	2	11	3	2	2
Solyc02g082200.2.1	6	383	2	215	0	388	74	514	3	437	17	316	0
Solyc02g082210.2.1	1	2	0	0	0	8	7	20	0	4	0	14	0
Solyc02g082220.2.1	16	116	9	76	17	256	141	363	36	194	74	247	6
Solyc02g082230.2.1	5	37	9	42	2	82	92	108	18	135	27	140	5
Solyc02g082240.1.1	1	0	0	0	0	5	6	3	1	0	0	0	0
Solyc02g082250.2.1	5	45	3	34	5	191	130	170	32	55	24	53	0
Solyc02g082260.2.1	19	810	35	213	7	54	25	49	5	105	737	135	19
Solyc02g082270.2.1	4	52	3	4	3	82	51	92	6	56	18	49	3
Solyc02g082280.2.1	8	28	9	27	2	71	44	104	10	127	24	71	0
Solyc02g082290.2.1	1	0	0	0	0	0	5	0	0	10	0	4	0
Solyc02g082300.2.1	2	2	1	0	0	3	3	5	2	7	0	1	0
Solyc02g082310.1.1	0	0	0	0	0	0	0	0	0	0	0	0	0
Solyc02g082340.2.1	18	163	13	116	2	237	157	303	13	1076	73	945	11
Solyc02g082350.2.1	10	55	10	33	2	40	84	33	10	73	23	81	2
Solyc02g082360.2.1	0	0	0	0	0	0	0	0	0	0	0	0	0
Solyc02g082400.2.1	0	0	0	0	0	0	0	0	0	0	0	0	0
Solyc02g082410.2.1	0	0	0	0	0	0	0	0	0	0	0	0	0
Solyc02g082420.2.1	1	2	0	2	1	2	4	2	1	7	0	3	0
Solyc02g082430.2.1	7	17	0	8	0	51	53	54	2	72	28	76	0
Solyc02g082440.1.1	1	1	0	0	0	8	4	2	2	0	0	6	4
Solyc02g082450.2.1	3	3	0	0	0	22	6	13	0	6	17	4	0
Solyc02g082460.2.1	12	60	9	34	2	185	68	208	2	136	24	95	2
Solyc02g082470.2.1	23	158	15	125	5	434	359	658	55	402	76	284	4
Solyc02g082480.2.1	4	19	0	9	0	3	26	38	0	32	3	41	0
Solyc02g082490.2.1	0	0	0	0	0	0	0	0	0	0	0	0	0
Solyc02g082500.1.1	0	0	0	0	0	0	0	0	0	0	0	0	0
Solyc02g082510.1.1	2	27	2	43	0	11	4	22	1	25	3	59	0
Solyc02g082520.1.1	0	0	0	0	0	0	0	0	0	0	0	0	0
Solyc02g082530.1.1	0	0	0	0	0	0	0	0	0	0	0	0	0
Solyc02g082540.2.1	8	88	6	68	13	187	103	240	21	161	36	111	1
Solyc02g082550.2.1	2	8	3	11	0	2	14	6	0	7	0	5	1
Solyc02g082570.1.1	11	68	12	32	8	181	83	157	17	151	37	104	1
Solyc02g082590.2.1	0	0	0	0	0	0	0	0	0	0	0	0	0
Solyc02g082600.2.1	0	0	0	0	0	0	0	0	0	0	0	0	0
Solyc02g082610.1.1	0	0	0	0	0	0	0	0	0	0	0	0	0
Solyc02g082630.2.1	0	0	0	0	0	0	0	0	0	0	0	0	0
Solyc02g082640.1.1	0	0	0	0	0	0	0	0	0	0	0	0	0
Solyc02g082660.2.1	3	10	5	7	0	19	36	8	3	10	4	5	0

Solyc02g082670.2.1	5	62	0	31	0	115	9	136	9	102	15	20	3
Solyc02g082680.2.1	13	96	9	56	5	79	59	71	7	179	22	159	0
Solyc02g082690.2.1	22	39	4	22	0	629	521	376	13	58	251	91	6
Solyc02g082700.2.1	7	858	55	374	26	738	345	845	95	620	124	481	8
Solyc02g082710.2.1	2	6	1	7	1	3	8	15	2	15	0	6	5
Solyc02g082720.2.1	0	0	0	0	0	0	0	0	0	0	0	0	0
Solyc02g082730.2.1	4	26	4	17	4	49	26	62	8	72	16	26	1
Solyc02g082740.1.1	1	11	0	6	0	1	8	20	0	2	0	11	0
Solyc02g082750.2.1	0	0	0	0	0	0	0	0	0	0	0	0	0
Solyc02g082760.2.1	7	1508	206	878	22	73	172	170	12	119	145	157	12
Solyc02g082770.2.1	1	1	0	0	0	4	5	3	0	5	2	1	0
Solyc02g082800.2.1	7	143	13	114	17	417	165	336	21	179	32	117	1
Solyc02g082810.2.1	6	31	0	14	0	43	27	26	8	29	19	30	1
Solyc02g082830.1.1	10	53	7	37	4	124	59	153	14	157	132	154	9
Solyc02g082840.2.1	7	18	6	11	2	53	97	62	30	74	46	58	0
Solyc02g082850.2.1	4	8	3	1	0	26	51	56	1	0	1	0	0
Solyc02g082860.2.1	6	25	3	15	1	25	38	55	6	53	4	15	1
Solyc02g082870.2.1	0	0	0	0	0	0	0	0	0	0	0	0	0
Solyc02g082880.2.1	0	0	0	0	0	0	0	0	0	0	0	0	0
Solyc02g082900.2.1	12	62	7	39	5	307	97	373	2	77	21	53	1
Solyc02g082910.2.1	2	2	0	0	0	12	0	3	0	0	3	3	0
Solyc02g082920.2.1	17	3079	401	1718	60	74937	32059	35869	1663	395	301	66	2
Solyc02g082930.2.1	0	0	0	0	0	0	0	0	0	0	0	0	0
Solyc02g082950.2.1	0	0	0	0	0	0	0	0	0	0	0	0	0
Solyc02g082960.2.1	0	0	0	0	0	0	0	0	0	0	0	0	0
Solyc02g082970.1.1	2	7	0	6	0	19	9	8	1	18	7	21	0
Solyc02g083000.1.1	0	0	0	0	0	0	0	0	0	0	0	0	0
Solyc02g083020.1.1	0	0	0	0	0	0	0	0	0	0	0	0	0
Solyc02g083030.1.1	0	0	0	0	0	0	0	0	0	0	0	0	0
Solyc02g083040.1.1	0	0	0	0	0	0	0	0	0	0	0	0	0
Solyc02g083050.1.1	0	0	0	0	0	0	0	0	0	0	0	0	0
Solyc02g083060.1.1	0	0	0	0	0	0	0	0	0	0	0	0	0
Solyc02g083090.1.1	0	0	0	0	0	0	0	0	0	0	0	0	0
Solyc02g083110.1.1	3	41	2	16	1	28	37	53	1	143	22	85	0
Solyc02g083120.1.1	3	15	0	15	4	7	7	6	2	33	4	18	3
Solyc02g083130.2.1	4	11	0	4	0	78	28	113	14	18	4	15	1
Solyc02g083140.2.1	8	30	1	4	3	63	83	57	3	44	10	18	1
Solyc02g083150.2.1	5	48	3	15	1	95	69	58	7	67	22	40	1
Solyc02g083170.2.1	2	24	14	17	4	50	22	27	6	22	7	13	0
Solyc02g083180.2.1	6	19	11	46	0	32	94	64	8	57	18	103	3
Solyc02g083190.1.1	1	6	1	2	3	0	9	0	1	4	2	0	0

Solyc02g083200.2.1	0	0	0	0	0	0	0	0	0	0	0	0	0
Solyc02g083220.1.1	0	0	0	0	0	0	0	0	0	0	0	0	0
Solyc02g083230.2.1	0	0	0	0	0	0	0	0	0	0	0	0	0
Solyc02g083240.2.1	0	0	0	0	0	0	0	0	0	0	0	0	0
Solyc02g083250.2.1	3	38	3	37	0	74	17	138	2	155	8	101	4
Solyc02g083260.2.1	3	9	1	1	0	16	5	8	0	11	9	12	3
Solyc02g083270.2.1	10	190	26	91	7	315	141	431	7	87	8	92	4
Solyc02g083280.2.1	9	528	137	598	8	2093	10618	3096	60	344	106	438	4
Solyc02g083290.2.1	6	37	12	31	0	54	95	93	13	73	27	29	2
Solyc02g083310.2.1	9	66	10	40	1	70	109	125	4	133	14	90	1
Solyc02g083320.2.1	7	201	7	166	5	909	61	651	8	860	21	577	8
Solyc02g083340.2.1	17	74	6	51	14	68	181	140	19	289	18	228	2
Solyc02g083350.2.1	5	45	3	49	0	90	21	164	15	64	10	57	4
Solyc02g083360.1.1	0	0	0	0	0	0	0	0	0	0	0	0	0
Solyc02g083370.1.1	0	0	0	0	0	0	0	0	0	0	0	0	0
Solyc02g083380.2.1	0	0	0	0	0	0	0	0	0	0	0	0	0
Solyc02g083390.2.1	3	31	0	5	0	96	30	52	8	119	14	44	3
Solyc02g083400.2.1	3	28	3	15	0	42	9	17	3	12	2	14	1
Solyc02g083410.2.1	6	102	21	75	3	27	38	40	2	36	11	28	0
Solyc02g083420.2.1	3	30	9	30	0	31	18	49	11	28	22	9	2
Solyc02g083430.1.1	8	10	2	5	1	60	20	46	10	59	11	28	0
Solyc02g083450.2.1	0	0	0	0	0	0	0	0	0	0	0	0	0
Solyc02g083460.2.1	10	78	10	79	13	278	179	163	82	40	7	48	1
Solyc02g083470.2.1	3	8	0	0	2	44	14	42	3	25	14	33	0
Solyc02g083480.2.1	0	0	0	0	0	0	0	0	0	0	0	0	0
Solyc02g083490.2.1	0	0	0	0	0	0	0	0	0	0	0	0	0
Solyc02g083500.2.1	5	38	2	21	0	39	60	51	2	24	9	24	0
Solyc02g083510.2.1	0	0	0	0	0	0	0	0	0	0	0	0	0
Solyc02g083520.2.1	0	0	0	0	0	0	0	0	0	0	0	0	0
Solyc02g083540.2.1	3	24	3	20	0	36	33	22	0	17	3	30	0
Solyc02g083560.2.1	2	6	1	6	0	27	6	17	3	8	7	26	0
Solyc02g083570.2.1	0	0	0	0	0	0	0	0	0	0	0	0	0
Solyc02g083580.2.1	0	0	0	0	0	0	0	0	0	0	0	0	0
Solyc02g083590.2.1	11	153	41	94	6	149	291	295	54	216	146	208	10
Solyc02g083600.2.1	1	0	0	0	0	10	8	1	5	3	0	0	0
Solyc02g083610.2.1	0	0	0	0	0	0	0	0	0	0	0	0	0
Solyc02g083620.2.1	0	0	0	0	0	0	0	0	0	0	0	0	0
Solyc02g083630.2.1	1	3	2	5	0	11	7	6	0	0	0	1	0
Solyc02g083640.2.1	0	0	0	0	0	0	0	0	0	0	0	0	0
Solyc02g083650.2.1	1	22	2	13	0	25	19	72	1	98	6	36	0
Solyc02g083660.1.1	4	14	0	5	0	16	37	29	4	41	4	34	6

Solyc02g083670.2.1	0	0	0	0	0	0	0	0	0	0	0	0	0
Solyc02g083680.1.1	2	7	3	27	0	7	4	28	0	18	9	21	0
Solyc02g083690.2.1	0	0	0	0	0	0	0	0	0	0	0	0	0
Solyc02g083700.2.1	14	7	0	0	0	31	48	13	1	224	70	132	13
Solyc02g083710.2.1	10	243	23	159	9	500	235	503	60	489	123	342	9
Solyc02g083720.2.1	0	0	0	0	0	0	0	0	0	0	0	0	0
Solyc02g083730.2.1	0	0	0	0	0	0	0	0	0	0	0	0	0
Solyc02g083740.1.1	0	0	0	0	0	0	0	0	0	0	0	0	0
Solyc02g083750.1.1	11	111	6	74	5	575	99	287	35	233	42	215	2
Solyc02g083760.2.1	4	59	3	39	0	80	7	53	0	112	2	75	0
Solyc02g083780.1.1	1	2	0	6	0	1	1	1	0	8	4	6	0
Solyc02g083790.2.1	2	3	1	6	0	4	4	0	0	1	0	2	0
Solyc02g083800.2.1	2	5	0	1	0	14	2	17	0	33	4	12	0
Solyc02g083810.2.1	9	5046	520	2495	15	346	922	976	17	607	214	803	7
Solyc02g083820.1.1	11	233	21	94	5	87	47	83	6	120	31	116	4
Solyc02g083850.2.1	1	1	1	0	0	18	14	11	5	0	2	5	0
Solyc02g083860.2.1	13	212	2	48	20	192	14	80	14	775	58	628	2
Solyc02g083880.2.1	4	38	4	4	2	2	12	2	1	201	25	232	0
Solyc02g083890.2.1	0	0	0	0	0	0	0	0	0	0	0	0	0
Solyc02g083900.2.1	18	66	9	63	14	156	138	269	13	197	57	147	2
Solyc02g083930.1.1	0	0	0	0	0	0	0	0	0	0	0	0	0
Solyc02g083940.2.1	1	1	0	0	0	4	4	4	1	2	0	1	0
Solyc02g083960.2.1	0	0	0	0	0	0	0	0	0	0	0	0	0
Solyc02g083970.1.1	4	110	4	53	0	24	66	57	1	55	5	45	0
Solyc02g083980.2.1	1	3	1	6	0	0	16	3	1	3	0	4	0
Solyc02g083990.2.1	5	29	1	33	0	46	28	50	5	30	6	49	0
Solyc02g084000.2.1	4	20	2	5	0	42	15	56	10	31	6	15	0
Solyc02g084010.1.1	1	6	0	5	0	1	3	2	0	16	4	86	0
Solyc02g084020.2.1	1	5	1	11	0	20	4	22	0	17	2	10	0
Solyc02g084030.2.1	5	91	4	26	0	61	39	85	14	187	16	59	0
Solyc02g084090.1.1	0	0	0	0	0	0	0	0	0	0	0	0	0
Solyc02g084120.1.1	0	0	0	0	0	0	0	0	0	0	0	0	0
Solyc02g084170.1.1	0	0	0	0	0	0	0	0	0	0	0	0	0
Solyc02g084180.1.1	0	0	0	0	0	0	0	0	0	0	0	0	0
Solyc02g084200.1.1	0	0	0	0	0	0	0	0	0	0	0	0	0
Solyc02g084210.1.1	0	0	0	0	0	0	0	0	0	0	0	0	0
Solyc02g084220.2.1	1	1	2	2	0	8	4	1	1	1	0	0	0
Solyc02g084230.1.1	4	33	3	29	0	49	44	77	7	114	19	55	0
Solyc02g084240.2.1	6	87	16	113	24	362	1126	609	123	378	222	202	12
Solyc02g084260.1.1	0	0	0	0	0	0	0	0	0	0	0	0	0
Solyc02g084280.2.1	0	0	0	0	0	0	0	0	0	0	0	0	0

Solyc02g084290.1.1	0	0	0	0	0	0	0	0	0	0	0	0	0
Solyc02g084300.1.1	0	0	0	0	0	0	0	0	0	0	0	0	0
Solyc02g084330.2.1	0	0	0	0	0	0	0	0	0	0	0	0	0
Solyc02g084340.1.1	10	52	4	36	7	55	89	82	4	96	31	61	0
Solyc02g084350.2.1	1	31	1	18	0	99	27	37	0	1	0	6	0
Solyc02g084360.2.1	1	345	2	74	4	806	26	429	2	811	20	316	2
Solyc02g084370.1.1	0	0	0	0	0	0	0	0	0	0	0	0	0
Solyc02g084380.2.1	4	3	0	0	0	22	15	23	4	8	6	16	0
Solyc02g084390.2.1	0	0	0	0	0	0	0	0	0	0	0	0	0
Solyc02g084400.2.1	1	1	1	0	0	0	1	0	0	4	3	11	0
Solyc02g084410.2.1	6	546	34	131	0	67	23	97	0	9	1	11	0
Solyc02g084420.2.1	0	0	0	0	0	0	0	0	0	0	0	0	0
Solyc02g084430.2.1	0	0	0	0	0	0	0	0	0	0	0	0	0
Solyc02g084440.2.1	6	5227	947	3634	35	547	2281	718	44	2355	414	2900	17
Solyc02g084480.1.1	0	0	0	0	0	0	0	0	0	0	0	0	0
Solyc02g084490.1.1	0	0	0	0	0	0	0	0	0	0	0	0	0
Solyc02g084500.2.1	2	22	3	15	0	27	32	38	9	28	1	8	0
Solyc02g084510.2.1	1	4	0	5	0	15	6	8	5	6	2	10	0
Solyc02g084520.2.1	14	47	9	33	6	68	65	87	16	80	36	70	5
Solyc02g084530.1.1	0	0	0	0	0	0	0	0	0	0	0	0	0
Solyc02g084540.2.1	0	0	0	0	0	0	0	0	0	0	0	0	0
Solyc02g084550.2.1	8	114	19	45	5	126	159	188	43	160	26	59	1
Solyc02g084560.2.1	0	0	0	0	0	0	0	0	0	0	0	0	0
Solyc02g084570.2.1	8	98	2	78	0	95	51	76	47	4	5	1	0
Solyc02g084580.1.1	0	0	0	0	0	0	0	0	0	0	0	0	0
Solyc02g084590.2.1	0	0	0	0	0	0	0	0	0	0	0	0	0
Solyc02g084600.2.1	1	0	0	0	0	0	0	1	0	9	10	15	4
Solyc02g084610.1.1	5	46	3	33	0	26	40	63	1	62	16	45	0
Solyc02g084620.2.1	8	25	0	22	0	60	27	56	2	41	2	50	1
Solyc02g084630.2.1	7	55	4	15	0	74	17	35	4	232	51	287	11
Solyc02g084640.2.1	17	137	17	15	19	81	190	26	169	200	73	62	42
Solyc02g084650.2.1	2	45	6	14	0	21	26	13	3	41	9	18	0
Solyc02g084660.2.1	1	0	0	1	0	4	4	1	1	0	0	2	0
Solyc02g084670.2.1	0	0	0	0	0	0	0	0	0	0	0	0	0
Solyc02g084680.2.1	0	0	0	0	0	0	0	0	0	0	0	0	0
Solyc02g084690.2.1	10	51	9	70	1	96	68	242	28	301	43	191	5
Solyc02g084700.2.1	8	29	4	12	0	76	59	93	11	100	19	82	4
Solyc02g084710.2.1	2	7	0	1	0	20	12	17	3	11	11	18	1
Solyc02g084720.2.1	18	364	21	189	57	129	142	147	54	933	175	608	8
Solyc02g084730.2.1	0	0	0	0	0	0	0	0	0	0	0	0	0
Solyc02g084740.2.1	1	25	0	9	0	23	5	39	0	14	0	14	0

Solyc02g084750.2.1	7	75	18	35	0	91	64	95	1	48	20	45	2
Solyc02g084760.2.1	4	258	13	136	8	463	122	490	15	242	15	172	3
Solyc02g084770.2.1	0	0	0	0	0	0	0	0	0	0	0	0	0
Solyc02g084790.2.1	0	0	0	0	0	0	0	0	0	0	0	0	0
Solyc02g084800.2.1	0	0	0	0	0	0	0	0	0	0	0	0	0
Solyc02g084810.2.1	2	10	0	9	0	16	8	44	2	19	0	26	2
Solyc02g084820.2.1	6	22	5	32	3	56	65	68	6	64	37	57	2
Solyc02g084830.2.1	0	0	0	0	0	0	0	0	0	0	0	0	0
Solyc02g084840.2.1	5	21	2	0	0	807	107	295	235	1	96	2	0
Solyc02g084850.2.1	1	235	0	146	6	19306	225	10776	320	90	6	31	0
Solyc02g084860.1.1	0	0	0	0	0	0	0	0	0	0	0	0	0
Solyc02g084870.2.1	6	4	2	8	0	56	50	46	14	31	13	14	0
Solyc02g084880.2.1	0	0	0	0	0	0	0	0	0	0	0	0	0
Solyc02g084890.1.1	7	126	10	124	4	6	51	15	6	22	8	122	2
Solyc02g084900.2.1	2	4	1	6	3	3	4	6	0	16	2	23	0
Solyc02g084910.2.1	0	0	0	0	0	0	0	0	0	0	0	0	0
Solyc02g084920.2.1	4	218	9	79	1	226	46	243	16	500	40	266	1
Solyc02g084930.2.1	0	0	0	0	0	0	0	0	0	0	0	0	0
Solyc02g084950.2.1	3	7	0	4	0	53	17	36	0	2	0	0	0
Solyc02g084960.1.1	0	0	0	0	0	0	0	0	0	0	0	0	0
Solyc02g084970.2.1	0	0	0	0	0	0	0	0	0	0	0	0	0
Solyc02g084980.2.1	12	23	9	6	15	274	61	103	152	4	1	2	2
Solyc02g084990.2.1	9	162	17	156	6	62	79	99	28	297	183	322	23
Solyc02g085000.2.1	9	25	7	30	2	199	91	152	14	104	55	89	3
Solyc02g085010.1.1	6	42	34	50	19	30	14	31	23	18	1	75	2
Solyc02g085020.2.1	0	0	0	0	0	0	0	0	0	0	0	0	0
Solyc02g085030.2.1	5	10	1	4	0	4	21	18	12	20	16	21	2
Solyc02g085040.2.1	8	93	24	74	32	473	461	326	71	270	97	256	4
Solyc02g085050.2.1	6	52	17	23	6	136	179	205	15	58	56	59	4
Solyc02g085060.1.1	0	0	0	0	0	0	0	0	0	0	0	0	0
Solyc02g085080.2.1	7	30	3	11	0	34	39	37	4	26	11	48	0
Solyc02g085090.1.1	0	0	0	0	0	0	0	0	0	0	0	0	0
Solyc02g085100.2.1	6	208	38	100	1	34	207	102	9	198	13	180	3
Solyc02g085110.2.1	0	0	0	0	0	0	0	0	0	0	0	0	0
Solyc02g085120.2.1	0	0	0	0	0	0	0	0	0	0	0	0	0
Solyc02g085130.2.1	1	2	2	6	0	5	0	0	0	0	9	7	2
Solyc02g085140.2.1	8	51	9	34	4	17	43	40	1	17	11	28	0
Solyc02g085150.2.1	0	0	0	0	0	0	0	0	0	0	0	0	0
Solyc02g085160.1.1	0	0	0	0	0	0	0	0	0	0	0	0	0
Solyc02g085170.2.1	0	0	0	0	0	0	0	0	0	0	0	0	0
Solyc02g085180.2.1	1	2	0	0	0	0	3	7	0	33	5	13	0

Solyc02g085210.1.1	0	0	0	0	0	0	0	0	0	0	0	0	0
Solyc02g085220.2.1	0	0	0	0	0	0	0	0	0	0	0	0	0
Solyc02g085230.2.1	0	0	0	0	0	0	0	0	0	0	0	0	0
Solyc02g085240.2.1	0	0	0	0	0	0	0	0	0	0	0	0	0
Solyc02g085250.2.1	5	17	1	16	0	6	27	10	0	42	1	38	0
Solyc02g085280.1.1	1	19	2	4	3	44	0	25	2	33	4	31	0
Solyc02g085290.2.1	4	18	1	16	0	3	16	10	3	11	5	31	0
Solyc02g085300.2.1	0	0	0	0	0	0	0	0	0	0	0	0	0
Solyc02g085310.2.1	0	0	0	0	0	0	0	0	0	0	0	0	0
Solyc02g085320.1.1	1	5	0	4	0	7	7	4	0	1	6	0	0
Solyc02g085340.1.1	7	55	1	21	2	62	23	31	0	56	14	69	4
Solyc02g085350.2.1	14	286	51	253	35	1565	615	1629	164	1408	466	1035	14
Solyc02g085360.2.1	0	0	0	0	0	0	0	0	0	0	0	0	0
Solyc02g085370.2.1	0	0	0	0	0	0	0	0	0	0	0	0	0
Solyc02g085390.2.1	0	0	0	0	0	0	0	0	0	0	0	0	0
Solyc02g085400.2.1	1	5	0	7	0	5	5	3	0	0	1	1	0
Solyc02g085420.2.1	12	298	35	169	25	765	275	658	41	597	119	595	24
Solyc02g085430.2.1	0	0	0	0	0	0	0	0	0	0	0	0	0
Solyc02g085440.2.1	0	0	0	0	0	0	0	0	0	0	0	0	0
Solyc02g085450.1.1	0	0	0	0	0	0	0	0	0	0	0	0	0
Solyc02g085460.1.1	0	0	0	0	0	0	0	0	0	0	0	0	0
Solyc02g085470.2.1	3	28	6	30	0	115	97	88	4	35	5	28	1
Solyc02g085480.2.1	0	0	0	0	0	0	0	0	0	0	0	0	0
Solyc02g085500.2.1	0	0	0	0	0	0	0	0	0	0	0	0	0
Solyc02g085510.1.1	0	0	0	0	0	0	0	0	0	0	0	0	0
Solyc02g085520.2.1	4	23	5	5	0	18	40	38	0	59	9	45	0
Solyc02g085540.1.1	2	27	1	12	1	47	17	65	5	76	13	49	1
Solyc02g085560.2.1	1	1	0	0	0	0	0	10	0	5	5	3	0
Solyc02g085570.2.1	9	80	16	79	1	371	126	249	30	249	47	171	6
Solyc02g085580.2.1	6	66	14	58	7	144	78	119	30	7	10	5	1
Solyc02g085590.2.1	22	5	0	0	1	755	319	215	1	38	52	24	0
Solyc02g085600.1.1	10	177	11	105	3	549	104	470	27	413	9	105	0
Solyc02g085610.2.1	2	6	4	10	0	16	14	9	2	8	6	6	0
Solyc02g085630.2.1	0	0	0	0	0	0	0	0	0	0	0	0	0
Solyc02g085640.2.1	10	31	4	68	11	190	181	330	18	82	28	76	0
Solyc02g085650.2.1	1	3	0	0	0	1	1	3	1	3	3	2	0
Solyc02g085660.1.1	0	0	0	0	0	0	0	0	0	0	0	0	0
Solyc02g085670.2.1	3	12	1	13	2	64	31	32	2	32	2	38	0
Solyc02g085690.2.1	0	0	0	0	0	0	0	0	0	0	0	0	0
Solyc02g085700.1.1	8	151	19	129	3	64	145	143	7	63	25	81	1
Solyc02g085710.2.1	0	0	0	0	0	0	0	0	0	0	0	0	0

Solyc02g085720.1.1	0	0	0	0	0	0	0	0	0	0	0	0	0
Solyc02g085730.2.1	8	473	46	231	4	704	114	503	5	392	58	344	4
Solyc02g085740.2.1	1	1	0	0	0	6	2	4	0	2	0	2	0
Solyc02g085760.2.1	13	662	81	390	0	43	63	180	0	126	23	67	0
Solyc02g085770.2.1	0	0	0	0	0	0	0	0	0	0	0	0	0
Solyc02g085780.2.1	4	44	0	13	3	22	33	60	2	119	15	41	0
Solyc02g085790.2.1	9	47	4	35	2	56	67	119	9	95	27	75	6
Solyc02g085810.2.1	31	120	29	73	9	317	177	343	21	224	67	151	3
Solyc02g085820.2.1	5	5	1	2	0	30	23	29	6	23	7	17	0
Solyc02g085830.2.1	0	0	0	0	0	0	0	0	0	0	0	0	0
Solyc02g085840.2.1	9	209	8	136	7	260	95	348	8	365	53	243	4
Solyc02g085850.1.1	2	10	0	1	0	7	11	9	0	8	2	15	0
Solyc02g085860.2.1	4	7	1	1	0	16	21	11	4	24	9	20	0
Solyc02g085870.2.1	15	464	134	61	298	907	1565	337	2147	361	101	24	49
Solyc02g085910.2.1	0	0	0	0	0	0	0	0	0	0	0	0	0
Solyc02g085920.2.1	3	28	4	12	6	28	25	25	8	96	17	52	0
Solyc02g085930.2.1	0	0	0	0	0	0	0	0	0	0	0	0	0
Solyc02g085940.2.1	0	0	0	0	0	0	0	0	0	0	0	0	0
Solyc02g085950.2.1	5	28331	926	13776	931	319	486	119	7	70	44	101	9
Solyc02g086050.2.1	4	9	2	16	3	4	2	13	0	17	5	9	0
Solyc02g086060.2.1	0	0	0	0	0	0	0	0	0	0	0	0	0
Solyc02g086070.2.1	0	0	0	0	0	0	0	0	0	0	0	0	0
Solyc02g086080.2.1	4	25	4	14	2	21	31	33	4	20	7	19	0
Solyc02g086090.2.1	8	39	1	22	0	18	12	33	0	71	10	26	4
Solyc02g086100.2.1	2	5	0	4	0	11	22	13	0	7	5	19	0
Solyc02g086110.2.1	0	0	0	0	0	0	0	0	0	0	0	0	0
Solyc02g086120.2.1	0	0	0	0	0	0	0	0	0	0	0	0	0
Solyc02g086130.2.1	11	91	14	67	8	40	28	154	8	159	49	93	0
Solyc02g086140.1.1	0	0	0	0	0	0	0	0	0	0	0	0	0
Solyc02g086150.2.1	3	41	2	10	1	78	85	120	1	47	7	29	0
Solyc02g086160.2.1	10	94	10	70	9	203	369	241	18	241	72	142	5
Solyc02g086170.2.1	1	3	1	0	0	0	4	1	0	3	4	3	0
Solyc02g086180.2.1	4	505	36	115	1	17	6	21	1	29	176	5	0
Solyc02g086200.1.1	1	2	2	5	0	3	0	6	0	6	3	1	0
Solyc02g086210.2.1	1	16	0	7	5	6	4	6	8	7	2	11	0
Solyc02g086220.2.1	5	25	6	23	0	55	40	83	8	64	4	21	1
Solyc02g086230.1.1	0	0	0	0	0	0	0	0	0	0	0	0	0
Solyc02g086240.2.1	3	95	7	38	8	261	63	279	26	177	52	206	7
Solyc02g086250.2.1	0	0	0	0	0	0	0	0	0	0	0	0	0
Solyc02g086260.1.1	3	16	16	3	6	62	69	45	6	38	24	56	8
Solyc02g086270.2.1	1	0	0	1	0	3	3	7	3	4	2	14	0

Solyc02g086280.2.1	0	0	0	0	0	0	0	0	0	0	0	0	0
Solyc02g086290.1.1	0	0	0	0	0	0	0	0	0	0	0	0	0
Solyc02g086300.2.1	4	47	3	9	0	1	3	5	1	153	59	457	67
Solyc02g086310.1.1	1	0	0	0	0	6	0	3	0	0	14	3	0
Solyc02g086320.1.1	0	0	0	0	0	0	0	0	0	0	0	0	0
Solyc02g086330.2.1	0	0	0	0	0	0	0	0	0	0	0	0	0
Solyc02g086340.2.1	12	60	5	30	1	61	69	67	15	123	16	100	0
Solyc02g086350.2.1	4	30	1	20	0	7	7	18	0	6	14	8	0
Solyc02g086360.2.1	5	7	2	8	0	30	8	29	11	39	8	25	0
Solyc02g086370.2.1	0	0	0	0	0	0	0	0	0	0	0	0	0
Solyc02g086380.1.1	0	0	0	0	0	0	0	0	0	0	0	0	0
Solyc02g086410.1.1	0	0	0	0	0	0	0	0	0	0	0	0	0
Solyc02g086420.1.1	0	0	0	0	0	0	0	0	0	0	0	0	0
Solyc02g086430.2.1	0	0	0	0	0	0	0	0	0	0	0	0	0
Solyc02g086440.2.1	2	8	1	4	0	2	7	2	2	16	4	6	0
Solyc02g086450.1.1	0	0	0	0	0	0	0	0	0	0	0	0	0
Solyc02g086460.2.1	90	1166	50	792	49	894	331	2428	7	1511	507	1769	44
Solyc02g086470.2.1	0	0	0	0	0	0	0	0	0	0	0	0	0
Solyc02g086480.1.1	0	0	0	0	0	0	0	0	0	0	0	0	0
Solyc02g086490.2.1	1	1	0	0	0	0	0	1	0	4	39	2	1
Solyc02g086500.2.1	14	12	4	15	4	30	120	52	14	128	27	131	4
Solyc02g086510.2.1	14	36	21	38	2	119	239	153	33	53	61	57	3
Solyc02g086520.2.1	8	138	12	87	3	171	81	197	25	247	51	154	0
Solyc02g086530.2.1	13	15	0	2	0	389	84	165	106	1	0	0	0
Solyc02g086550.2.1	4	11	5	7	4	16	26	20	9	23	1	14	1
Solyc02g086560.2.1	4	63	1	32	0	47	56	73	6	83	9	27	0
Solyc02g086570.1.1	0	0	0	0	0	0	0	0	0	0	0	0	0
Solyc02g086580.2.1	0	0	0	0	0	0	0	0	0	0	0	0	0
Solyc02g086590.2.1	8	26	15	12	1	47	78	37	55	14	3	11	0
Solyc02g086600.2.1	3	7	0	0	0	20	5	40	3	23	5	31	0
Solyc02g086610.2.1	4	8	1	9	0	35	25	50	2	12	3	4	0
Solyc02g086620.2.1	0	0	0	0	0	0	0	0	0	0	0	0	0
Solyc02g086630.1.1	0	0	0	0	0	0	0	0	0	0	0	0	0
Solyc02g086640.2.1	2	19	5	5	0	12	11	33	0	22	5	37	2
Solyc02g086650.2.1	7	1155	40	227	1	11	0	13	0	6	10	3	0
Solyc02g086660.2.1	6	32	11	30	0	46	42	63	5	46	30	54	0
Solyc02g086670.2.1	9	104	9	110	6	111	98	146	5	124	36	121	3
Solyc02g086680.2.1	9	94	9	51	0	112	27	201	3	53	10	57	0
Solyc02g086700.2.1	0	0	0	0	0	0	0	0	0	0	0	0	0
Solyc02g086710.2.1	7	81	20	63	0	385	224	265	17	204	57	99	5
Solyc02g086730.1.1	5	258	37	211	3	102	196	138	0	175	29	165	6

Solyc02g086740.1.1	9	141	20	112	3	15	64	62	0	82	20	143	2
Solyc02g086750.1.1	27	147	16	61	4	359	186	315	23	155	73	118	4
Solyc02g086760.1.1	0	0	0	0	0	0	0	0	0	0	0	0	0
Solyc02g086770.2.1	2	13	4	16	0	0	3	2	1	12	2	1	0
Solyc02g086780.2.1	1	0	0	0	0	3	2	3	1	7	5	4	0
Solyc02g086790.2.1	10	87	13	44	8	143	82	128	16	223	20	178	15
Solyc02g086800.2.1	6	11	4	9	1	19	13	28	1	17	6	9	1
Solyc02g086810.1.1	1	1	0	1	2	1	4	1	1	11	0	10	0
Solyc02g086820.2.1	8	3305	346	2437	8	20	10	58	2	15	25	13	0
Solyc02g086830.2.1	11	359	74	208	14	82	395	190	19	148	92	137	9
Solyc02g086840.2.1	1	4	1	2	0	1	14	9	2	0	1	0	0
Solyc02g086860.2.1	5	35	0	28	0	89	42	175	4	46	11	52	2
Solyc02g086870.2.1	10	24	5	12	10	43	56	85	14	83	11	42	4
Solyc02g086880.2.1	6	214	46	149	22	5497	1862	5742	110	1488	296	655	11
Solyc02g086890.2.1	9	170	19	127	0	26	90	93	9	183	17	187	3
Solyc02g086900.2.1	2	4	1	0	0	6	4	8	0	4	1	5	0
Solyc02g086910.2.1	11	236	23	122	8	15	117	73	0	49	12	36	0
Solyc02g086930.2.1	8	642	54	470	27	1338	328	1192	58	1050	81	1088	12
Solyc02g086940.2.1	5	35	1	26	1	32	15	52	5	95	5	46	0
Solyc02g086950.1.1	0	0	0	0	0	0	0	0	0	0	0	0	0
Solyc02g086960.2.1	0	0	0	0	0	0	0	0	0	0	0	0	0
Solyc02g086970.2.1	3	69	5	10	5	657	71	30	3	293	10	195	0
Solyc02g086980.2.1	1	5	0	1	0	2	6	5	0	6	2	27	0
Solyc02g086990.2.1	1	0	2	0	0	13	2	7	0	4	0	1	0
Solyc02g087000.2.1	14	111	51	131	4	410	98	590	2	124	28	81	0
Solyc02g087010.2.1	5	10	2	5	0	71	23	65	0	4	3	4	0
Solyc02g087030.1.1	0	0	0	0	0	0	0	0	0	0	0	0	0
Solyc02g087040.1.1	0	0	0	0	0	0	0	0	0	0	0	0	0
Solyc02g087050.2.1	4	26	5	13	0	35	28	38	0	36	15	90	1
Solyc02g087060.2.1	4	172	10	190	0	124	46	329	2	91	7	121	0
Solyc02g087070.2.1	0	0	0	0	0	0	0	0	0	0	0	0	0
Solyc02g087090.2.1	0	0	0	0	0	0	0	0	0	0	0	0	0
Solyc02g087100.1.1	0	0	0	0	0	0	0	0	0	0	0	0	0
Solyc02g087110.2.1	18	103	55	76	4	468	186	255	22	49	4	13	0
Solyc02g087120.2.1	0	0	0	0	0	0	0	0	0	0	0	0	0
Solyc02g087130.2.1	6	48	9	33	12	33	70	77	13	53	30	36	2
Solyc02g087140.2.1	0	0	0	0	0	0	0	0	0	0	0	0	0
Solyc02g087150.2.1	9	310	16	134	1	88	93	140	6	136	13	134	1
Solyc02g087170.2.1	3	7	3	2	5	11	14	26	2	19	3	22	0
Solyc02g087180.2.1	5	43	1	17	0	53	40	43	2	88	5	60	2
Solyc02g087190.1.1	8	398	21	113	17	9	21	26	3	149	80	136	3

Solyc02g087200.1.1	3	31	0	17	1	15	13	29	1	8	9	32	0
Solyc02g087210.2.1	3	83	13	18	5	122	1	26	8	3	4	3	0
Solyc02g087220.2.1	6	18	3	22	0	16	58	39	1	40	13	54	0
Solyc02g087230.2.1	1	141	4	41	0	46	13	41	1	31	0	17	0
Solyc02g087240.2.1	9	491	40	274	49	951	832	1334	194	1059	274	674	8
Solyc02g087250.2.1	23	169	18	75	5	186	264	319	20	335	54	143	1
Solyc02g087260.1.1	2	13	0	6	0	9	9	6	4	8	0	11	0
Solyc02g087270.2.1	0	0	0	0	0	0	0	0	0	0	0	0	0
Solyc02g087280.2.1	10	26	2	23	0	137	120	204	0	22	23	14	0
Solyc02g087290.2.1	7	46	3	32	3	67	54	160	6	42	5	41	0
Solyc02g087300.1.1	2	56	5	35	6	116	17	87	17	59	8	62	1
Solyc02g087310.2.1	1	2	1	2	0	10	2	3	1	1	1	9	0
Solyc02g087330.2.1	1	22	3	13	5	46	26	13	20	7	7	8	3
Solyc02g087340.2.1	16	350	117	254	4	139	201	182	20	311	131	709	17
Solyc02g087350.1.1	3	37	23	14	6	5	1	4	0	4	1	25	3
Solyc02g087360.2.1	20	81	14	77	8	249	123	267	23	105	44	130	4
Solyc02g087400.1.1	12	132	15	89	4	21	135	47	9	95	28	61	0
Solyc02g087410.2.1	0	0	0	0	0	0	0	0	0	0	0	0	0
Solyc02g087430.2.1	6	39	2	22	3	173	9	76	2	154	17	79	0
Solyc02g087440.2.1	0	0	0	0	0	0	0	0	0	0	0	0	0
Solyc02g087450.2.1	0	0	0	0	0	0	0	0	0	0	0	0	0
Solyc02g087460.1.1	12	96	13	66	14	106	77	147	33	122	39	86	0
Solyc02g087480.2.1	2	22	5	2	0	30	6	14	0	4	1	5	0
Solyc02g087510.2.1	0	0	0	0	0	0	0	0	0	0	0	0	0
Solyc02g087520.2.1	2	6	0	1	0	7	11	28	0	0	0	0	0
Solyc02g087550.2.1	14	99	5	48	7	431	313	432	55	215	124	176	4
Solyc02g087560.1.1	5	17	4	42	0	27	27	24	2	51	7	18	0
Solyc02g087580.2.1	0	0	0	0	0	0	0	0	0	0	0	0	0
Solyc02g087600.2.1	2	11	0	8	0	54	18	26	0	32	3	25	0
Solyc02g087610.1.1	0	0	0	0	0	0	0	0	0	0	0	0	0
Solyc02g087620.2.1	7	30	4	10	5	19	39	66	3	44	9	27	0
Solyc02g087630.2.1	4	35	3	18	4	47	31	52	6	23	32	36	5
Solyc02g087640.2.1	2	3	1	22	0	27	25	16	4	51	1	33	10
Solyc02g087650.2.1	0	0	0	0	0	0	0	0	0	0	0	0	0
Solyc02g087670.2.1	0	0	0	0	0	0	0	0	0	0	0	0	0
Solyc02g087710.2.1	4	49	3	33	2	73	25	138	4	65	10	67	0
Solyc02g087720.1.1	11	55	6	33	0	100	67	105	17	96	32	107	1
Solyc02g087730.2.1	1	0	0	0	0	10	3	3	0	3	2	6	0
Solyc02g087740.2.1	6	11	1	6	0	332	41	100	4	222	8	155	5
Solyc02g087750.2.1	2	90	1	35	3	48	12	54	4	129	7	107	2
Solyc02g087760.2.1	20	519	28	219	43	341	124	517	7	600	106	460	8

Solyc02g087770.2.1	5	20	4	10	1	4	15	7	0	87	19	58	3
Solyc02g087780.2.1	3	22	2	3	0	39	11	13	5	20	2	6	0
Solyc02g087800.2.1	2	1	0	0	0	3	4	0	1	1	0	7	0
Solyc02g087810.2.1	0	0	0	0	0	0	0	0	0	0	0	0	0
Solyc02g087830.2.1	1	0	3	0	0	0	6	0	0	39	13	10	3
Solyc02g087840.2.1	6	11	9	21	0	15	59	29	0	257	34	255	2
Solyc02g087850.1.1	2	32	2	23	2	8	5	15	2	25	1	17	0
Solyc02g087860.2.1	0	0	0	0	0	0	0	0	0	0	0	0	0
Solyc02g087870.2.1	14	54	7	49	4	33	91	66	2	137	26	80	1
Solyc02g087880.2.1	2	9	0	8	0	5	1	6	0	4	4	0	0
Solyc02g087890.2.1	3	1	0	1	0	664	127	454	13	0	8	0	0
Solyc02g087900.2.1	17	144	9	65	10	470	160	401	27	349	74	235	4
Solyc02g087910.1.1	0	0	0	0	0	0	0	0	0	0	0	0	0
Solyc02g087930.2.1	2	53	19	25	7	72	89	101	52	264	72	168	9
Solyc02g087940.2.1	3	7	0	4	0	12	20	10	2	50	2	21	0
Solyc02g087950.2.1	1	1	0	3	0	5	4	2	0	3	1	2	0
Solyc02g087960.2.1	0	0	0	0	0	0	0	0	0	0	0	0	0
Solyc02g087970.1.1	1	4	0	3	0	1	0	12	0	1898	11	1562	5
Solyc02g087980.2.1	0	0	0	0	0	0	0	0	0	0	0	0	0
Solyc02g087990.2.1	0	0	0	0	0	0	0	0	0	0	0	0	0
Solyc02g088000.2.1	13	468	47	200	1	385	255	453	10	252	30	131	1
Solyc02g088010.2.1	3	14	0	14	0	152	7	61	2	87	6	41	1
Solyc02g088040.1.1	0	0	0	0	0	0	0	0	0	0	0	0	0
Solyc02g088070.2.1	0	0	0	0	0	0	0	0	0	0	0	0	0
Solyc02g088090.1.1	3	172	46	122	9	41	5	4	8	41	1	23	12
Solyc02g088100.2.1	5	190	9	104	7	31	53	23	16	264	61	357	24
Solyc02g088110.2.1	6	36	8	32	0	32	43	67	1	76	24	68	2
Solyc02g088120.2.1	0	0	0	0	0	0	0	0	0	0	0	0	0
Solyc02g088130.1.1	0	0	0	0	0	0	0	0	0	0	0	0	0
Solyc02g088140.1.1	0	0	0	0	0	0	0	0	0	0	0	0	0
Solyc02g088160.2.1	0	0	0	0	0	0	0	0	0	0	0	0	0
Solyc02g088170.1.1	0	0	0	0	0	0	0	0	0	0	0	0	0
Solyc02g088180.2.1	5	13	1	0	1	57	27	30	7	1	0	3	0
Solyc02g088190.2.1	0	0	0	0	0	0	0	0	0	0	0	0	0
Solyc02g088200.2.1	0	0	0	0	0	0	0	0	0	0	0	0	0
Solyc02g088210.2.1	1	10	2	1	0	6	12	5	1	14	0	1	0
Solyc02g088220.2.1	4	16	15	4	2	5	21	4	5	26	21	18	1
Solyc02g088230.2.1	5	72	3	32	5	9	15	11	0	8	3	13	0
Solyc02g088240.2.1	3	9	11	10	5	4	2	0	2	11	10	18	0
Solyc02g088250.2.1	1	5	0	0	0	0	4	0	0	0	2	6	0
Solyc02g088260.2.1	13	1013	42	541	37	2369	436	2677	86	1596	209	908	30

Solyc02g088270.2.1	12	17	0	3	0	216	101	162	2	33	46	0	0
Solyc02g088280.2.1	0	0	0	0	0	0	0	0	0	0	0	0	0
Solyc02g088290.2.1	23	57	8	44	6	181	158	238	32	116	49	100	15
Solyc02g088300.2.1	9	72	10	76	5	119	147	173	15	118	45	150	4
Solyc02g088310.1.1	0	0	0	0	0	0	0	0	0	0	0	0	0
Solyc02g088320.2.1	0	0	0	0	0	0	0	0	0	0	0	0	0
Solyc02g088330.2.1	0	0	0	0	0	0	0	0	0	0	0	0	0
Solyc02g088340.2.1	2	52	2	28	1	22	12	34	0	70	14	36	0
Solyc02g088350.2.1	6	258	15	107	0	83	106	124	0	113	4	54	0
Solyc02g088380.2.1	5	3	0	0	0	0	0	0	0	65	22	147	1
Solyc02g088390.2.1	4	111	0	41	0	4	11	11	11	182	28	62	0
Solyc02g088400.2.1	0	0	0	0	0	0	0	0	0	0	0	0	0
Solyc02g088430.1.1	0	0	0	0	0	0	0	0	0	0	0	0	0
Solyc02g088450.2.1	0	0	0	0	0	0	0	0	0	0	0	0	0
Solyc02g088460.2.1	8	52	10	24	0	272	118	230	23	43	13	37	0
Solyc02g088470.1.1	5	21	10	9	1	8	67	11	2	60	2	13	0
Solyc02g088480.2.1	11	71	11	66	13	163	63	218	19	132	27	102	2
Solyc02g088490.2.1	12	101	22	72	2	90	84	134	5	297	44	146	2
Solyc02g088500.1.1	0	0	0	0	0	0	0	0	0	0	0	0	0
Solyc02g088510.2.1	0	0	0	0	0	0	0	0	0	0	0	0	0
Solyc02g088530.1.1	0	0	0	0	0	0	0	0	0	0	0	0	0
Solyc02g088540.2.1	0	0	0	0	0	0	0	0	0	0	0	0	0
Solyc02g088550.2.1	3	75	4	40	0	133	67	165	13	135	19	124	0
Solyc02g088560.2.1	14	423	18	247	6	2115	911	2119	23	258	17	128	0
Solyc02g088570.1.1	10	62	0	33	0	131	86	142	2	142	13	79	3
Solyc02g088580.1.1	5	16	4	32	0	29	37	30	8	17	8	23	0
Solyc02g088590.2.1	1	2	1	3	0	0	6	2	0	2	0	1	0
Solyc02g088600.2.1	4	20	1	3	0	29	9	7	0	17	0	20	0
Solyc02g088610.2.1	29	96	46	114	11	370	646	461	29	359	291	324	8
Solyc02g088620.2.1	5	58	7	29	2	123	30	71	6	66	13	51	0
Solyc02g088630.2.1	6	62	7	20	16	409	31	77	24	44	10	44	1
Solyc02g088650.1.1	0	0	0	0	0	0	0	0	0	0	0	0	0
Solyc02g088660.2.1	6	10	2	12	0	31	12	28	3	42	18	22	1
Solyc02g088670.1.1	0	0	0	0	0	0	0	0	0	0	0	0	0
Solyc02g088680.1.1	0	0	0	0	0	0	0	0	0	0	0	0	0
Solyc02g088690.2.1	4	590	34	238	19	274	51	224	20	468	70	409	7
Solyc02g088700.2.1	12	264	40	198	21	529	377	841	174	437	209	357	18
Solyc02g088710.2.1	0	0	0	0	0	0	0	0	0	0	0	0	0
Solyc02g088720.2.1	16	105	18	99	11	191	189	253	32	236	40	215	5
Solyc02g088730.1.1	4	14	3	8	0	33	39	66	1	38	7	44	0
Solyc02g088740.1.1	0	0	0	0	0	0	0	0	0	0	0	0	0

Solyc02g088760.2.1	3	12	0	13	2	38	14	48	3	135	7	79	0
Solyc02g088770.2.1	0	0	0	0	0	0	0	0	0	0	0	0	0
Solyc02g088780.2.1	0	0	0	0	0	0	0	0	0	0	0	0	0
Solyc02g088790.2.1	8	39	2	12	1	162	55	157	10	108	19	87	3
Solyc02g088800.1.1	6	71	8	29	0	121	44	96	5	28	11	51	1
Solyc02g088820.2.1	1	6	3	0	11	0	3	0	0	0	0	0	0
Solyc02g088830.2.1	0	0	0	0	0	0	0	0	0	0	0	0	0
Solyc02g088840.2.1	0	0	0	0	0	0	0	0	0	0	0	0	0
Solyc02g088850.2.1	0	0	0	0	0	0	0	0	0	0	0	0	0
Solyc02g088900.2.1	1	4	1	0	0	11	5	5	0	0	0	0	0
Solyc02g088910.2.1	12	27	4	20	0	222	213	166	44	18	11	20	1
Solyc02g088920.2.1	6	11	1	11	2	40	21	75	6	36	1	13	1
Solyc02g088930.2.1	1	7	1	2	1	29	13	10	3	22	12	22	2
Solyc02g088940.2.1	4	10	3	3	0	106	91	21	10	13	18	14	2
Solyc02g088950.1.1	0	0	0	0	0	0	0	0	0	0	0	0	0
Solyc02g088960.2.1	0	0	0	0	0	0	0	0	0	0	0	0	0
Solyc02g088980.1.1	0	0	0	0	0	0	0	0	0	0	0	0	0
Solyc02g088990.1.1	0	0	0	0	0	0	0	0	0	0	0	0	0
Solyc02g089010.1.1	2	4	3	0	0	8	2	4	0	2	9	3	0
Solyc02g089020.1.1	7	32	0	36	2	73	37	36	9	4	2	15	0
Solyc02g089030.1.1	0	0	0	0	0	0	0	0	0	0	0	0	0
Solyc02g089040.2.1	0	0	0	0	0	0	0	0	0	0	0	0	0
Solyc02g089050.2.1	0	0	0	0	0	0	0	0	0	0	0	0	0
Solyc02g089060.2.1	4	6	1	2	1	18	10	34	7	24	16	6	3
Solyc02g089070.2.1	13	165	21	103	13	525	203	446	56	412	115	344	9
Solyc02g089080.2.1	2	441	6	109	4	4	10	15	7	162	2	202	7
Solyc02g089090.2.1	21	525	49	417	19	445	137	723	19	439	24	360	3
Solyc02g089100.2.1	5	35	0	19	4	104	30	47	1	55	11	77	1
Solyc02g089110.2.1	4	26	8	29	8	100	57	79	5	39	21	22	0
Solyc02g089120.2.1	6	68	2	20	0	38	9	48	0	44	15	79	0
Solyc02g089130.2.1	0	0	0	0	0	0	0	0	0	0	0	0	0
Solyc02g089150.2.1	1	0	0	0	0	0	1	1	1	4	13	0	2
Solyc02g089160.2.1	7	14	2	8	0	255	14	168	0	4	21	10	0
Solyc02g089170.2.1	0	0	0	0	0	0	0	0	0	0	0	0	0
Solyc02g089190.1.1	0	0	0	0	0	0	0	0	0	0	0	0	0
Solyc02g089200.2.1	2	0	0	0	0	0	0	0	0	263	58	263	4
Solyc02g089210.2.1	0	0	0	0	0	0	0	0	0	0	0	0	0
Solyc02g089220.2.1	8	95	3	51	3	86	90	122	13	26	8	19	0
Solyc02g089230.2.1	4	173	4	71	0	473	117	328	21	103	20	56	1
Solyc02g089240.2.1	6	38	4	20	0	32	44	70	8	101	12	47	0
Solyc02g089250.2.1	4	65	1	22	2	82	12	202	0	20	9	5	0

Solyc02g089260.2.1	53	298	33	294	33	1138	611	1691	75	837	219	669	17
Solyc02g089270.1.1	0	0	0	0	0	0	0	0	0	0	0	0	0
Solyc02g089280.2.1	2	20	1	16	0	46	9	50	3	16	2	26	0
Solyc02g089290.2.1	1	0	0	0	0	2	0	1	0	4	4	3	0
Solyc02g089310.1.1	2	11	0	5	0	4	7	22	0	17	2	4	0
Solyc02g089320.2.1	7	17	5	6	3	65	37	87	1	24	11	13	0
Solyc02g089330.2.1	0	0	0	0	0	0	0	0	0	0	0	0	0
Solyc02g089340.2.1	3	8	1	9	7	15	29	31	7	15	4	10	0
Solyc02g089350.2.1	2	90	1	63	5	3	2	3	0	55	14	120	5
Solyc02g089360.2.1	12	66	5	41	4	141	49	144	9	153	20	70	3
Solyc02g089370.2.1	10	29	4	22	3	77	65	150	8	53	19	43	0
Solyc02g089380.1.1	1	3	0	5	0	8	5	8	0	1	0	4	0
Solyc02g089390.1.1	3	31	4	34	2	62	52	69	2	33	9	16	0
Solyc02g089400.2.1	5	59	10	49	11	289	106	338	33	31	6	14	0
Solyc02g089410.2.1	0	0	0	0	0	0	0	0	0	0	0	0	0
Solyc02g089420.1.1	0	0	0	0	0	0	0	0	0	0	0	0	0
Solyc02g089440.2.1	7	66	17	58	10	38	47	79	9	120	45	147	6
Solyc02g089460.1.1	0	0	0	0	0	0	0	0	0	0	0	0	0
Solyc02g089470.1.1	0	0	0	0	0	0	0	0	0	0	0	0	0
Solyc02g089490.2.1	0	0	0	0	0	0	0	0	0	0	0	0	0
Solyc02g089500.2.1	0	0	0	0	0	0	0	0	0	0	0	0	0
Solyc02g089520.1.1	0	0	0	0	0	0	0	0	0	0	0	0	0
Solyc02g089540.2.1	0	0	0	0	0	0	0	0	0	0	0	0	0
Solyc02g089550.2.1	1	5	3	1	0	0	0	2	0	9	3	20	0
Solyc02g089560.2.1	1	2	1	2	0	4	0	7	0	0	0	0	0
Solyc02g089600.2.1	10	31	3	26	0	56	29	57	13	207	28	153	5
Solyc02g089610.1.1	12	2212	353	745	107	8905	1068	2106	536	345	245	416	11
Solyc02g089620.2.1	25	1142	13	501	83	168	456	266	73	3808	481	3894	40
Solyc02g089630.2.1	19	187	15	123	31	9	65	26	40	791	282	1089	36
Solyc02g089640.2.1	11	595	56	383	45	187	50	133	11	101	26	144	19
Solyc02g089650.2.1	8	7	8	15	7	50	70	84	7	1	2	8	0
Solyc02g089660.1.1	0	0	0	0	0	0	0	0	0	0	0	0	0
Solyc02g089680.2.1	2	36	2	25	1	4	9	19	1	12	8	26	0
Solyc02g089690.2.1	5	25	1	6	0	6	6	27	10	49	18	28	1
Solyc02g089700.2.1	0	0	0	0	0	0	0	0	0	0	0	0	0
Solyc02g089720.1.1	18	48	4	61	2	762	234	725	57	147	7	130	7
Solyc02g089730.1.1	0	0	0	0	0	0	0	0	0	0	0	0	0
Solyc02g089750.2.1	0	0	0	0	0	0	0	0	0	0	0	0	0
Solyc02g089760.2.1	0	0	0	0	0	0	0	0	0	0	0	0	0
Solyc02g089770.2.1	1	0	0	0	0	0	0	7	0	5	6	5	0
Solyc02g089790.2.1	0	0	0	0	0	0	0	0	0	0	0	0	0

Solyc02g089800.2.1	14	76	14	76	8	148	97	197	21	114	37	87	0
Solyc02g089810.2.1	7	14	2	10	0	26	36	50	3	20	17	23	1
Solyc02g089820.2.1	4	31	2	13	1	53	28	63	3	79	7	71	1
Solyc02g089840.2.1	4	24	0	13	0	31	13	45	3	117	9	60	0
Solyc02g089850.2.1	5	34	7	18	0	19	15	34	2	25	13	13	0
Solyc02g089860.1.1	0	0	0	0	0	0	0	0	0	0	0	0	0
Solyc02g089870.1.1	6	51	3	35	0	219	45	134	5	177	4	88	0
Solyc02g089880.2.1	6	10	2	9	2	16	35	46	11	68	17	42	0
Solyc02g089890.2.1	0	0	0	0	0	0	0	0	0	0	0	0	0
Solyc02g089900.1.1	8	134	39	148	3	99	17	27	2	9	3	52	4
Solyc02g089930.2.1	7	76	5	49	10	62	40	120	41	68	23	63	2
Solyc02g089940.2.1	11	134	9	88	6	190	108	171	5	14	14	12	0
Solyc02g089950.1.1	3	8	9	5	4	14	146	16	9	7	14	10	1
Solyc02g089960.2.1	1	4	3	4	0	0	6	2	0	0	0	0	0
Solyc02g089970.2.1	17	72	9	97	2	244	95	245	17	98	22	77	4
Solyc02g089980.2.1	0	0	0	0	0	0	0	0	0	0	0	0	0
Solyc02g089990.1.1	4	182	3	83	2	23	87	29	16	13	5	11	0
Solyc02g090000.2.1	1	4	2	0	0	0	6	3	1	2	0	9	0
Solyc02g090010.1.1	1	10	0	22	1	42	11	22	0	38	5	20	0
Solyc02g090020.2.1	0	0	0	0	0	0	0	0	0	0	0	0	0
Solyc02g090030.2.1	12	4643	585	2759	32	192	1062	366	19	902	399	1095	61
Solyc02g090040.1.1	0	0	0	0	0	0	0	0	0	0	0	0	0
Solyc02g090050.2.1	0	0	0	0	0	0	0	0	0	0	0	0	0
Solyc02g090070.2.1	4	38	1	5	3	26	5	14	2	44	9	36	0
Solyc02g090090.2.1	8	79	5	30	1	179	82	178	10	146	30	86	0
Solyc02g090100.1.1	6	101	37	44	5	11	20	17	1	38	32	47	3
Solyc02g090110.2.1	10	50	16	28	13	171	17	133	15	48	22	57	2
Solyc02g090120.1.1	0	0	0	0	0	0	0	0	0	0	0	0	0
Solyc02g090130.2.1	10	23	7	35	1	66	63	35	15	53	10	24	3
Solyc02g090140.1.1	2	0	2	0	0	2	8	20	1	8	0	3	0
Solyc02g090150.1.1	0	0	0	0	0	0	0	0	0	0	0	0	0
Solyc02g090160.2.1	14	111	16	87	6	138	88	220	22	94	47	83	3
Solyc02g090170.2.1	0	0	0	0	0	0	0	0	0	0	0	0	0
Solyc02g090180.2.1	0	0	0	0	0	0	0	0	0	0	0	0	0
Solyc02g090190.1.1	15	135	18	109	14	214	173	241	31	154	56	176	6
Solyc02g090200.2.1	0	0	0	0	0	0	0	0	0	0	0	0	0
Solyc02g090210.2.1	2	0	0	0	0	13	7	3	6	0	1	0	0
Solyc02g090220.2.1	0	0	0	0	0	0	0	0	0	0	0	0	0
Solyc02g090230.2.1	2	10	1	9	0	4	7	13	2	6	2	3	0
Solyc02g090240.2.1	0	0	0	0	0	0	0	0	0	0	0	0	0
Solyc02g090250.2.1	0	0	0	0	0	0	0	0	0	0	0	0	0

Solyc02g090260.1.1	0	0	0	0	0	0	0	0	0	0	0	0	0
Solyc02g090290.2.1	2	2	5	1	18	20	22	6	15	0	0	0	0
Solyc02g090300.2.1	0	0	0	0	0	0	0	0	0	0	0	0	0
Solyc02g090310.1.1	0	0	0	0	0	0	0	0	0	0	0	0	0
Solyc02g090330.2.1	0	0	0	0	0	0	0	0	0	0	0	0	0
Solyc02g090340.2.1	2	160	8	56	3	20	23	22	38	163	0	137	0
Solyc02g090350.2.1	5	35	1	8	3	8	0	1	2	61	24	52	1
Solyc02g090360.2.1	2	75	7	23	2	12	0	9	0	7	2	35	0
Solyc02g090380.2.1	13	132	25	147	17	11	67	45	5	102	29	245	3
Solyc02g090390.2.1	5	85	3	32	6	94	149	174	24	238	8	142	2
Solyc02g090400.2.1	0	0	0	0	0	0	0	0	0	0	0	0	0
Solyc02g090410.2.1	0	0	0	0	0	0	0	0	0	0	0	0	0
Solyc02g090420.2.1	1	16	2	11	0	148	9	50	2	45	29	32	1
Solyc02g090430.2.1	1	2	0	0	0	6	0	0	1	0	4	8	0
Solyc02g090440.2.1	0	0	0	0	0	0	0	0	0	0	0	0	0
Solyc02g090450.2.1	0	0	0	0	0	0	0	0	0	0	0	0	0
Solyc02g090460.2.1	0	0	0	0	0	0	0	0	0	0	0	0	0
Solyc02g090470.2.1	0	0	0	0	0	0	0	0	0	0	0	0	0
Solyc02g090480.2.1	1	3	0	0	0	0	0	4	0	3	4	0	0
Solyc02g090490.2.1	6	51	27	50	0	39	47	32	6	28	3	49	0
Solyc02g090500.2.1	0	0	0	0	0	0	0	0	0	0	0	0	0
Solyc02g090510.2.1	0	0	0	0	0	0	0	0	0	0	0	0	0
Solyc02g090530.2.1	0	0	0	0	0	0	0	0	0	0	0	0	0
Solyc02g090540.2.1	0	0	0	0	0	0	0	0	0	0	0	0	0
Solyc02g090560.2.1	0	0	0	0	0	0	0	0	0	0	0	0	0
Solyc02g090570.2.1	20	158	16	112	18	243	150	313	37	343	67	206	1
Solyc02g090580.2.1	11	38	9	32	13	16	109	40	47	18	3	30	1
Solyc02g090620.2.1	0	0	0	0	0	0	0	0	0	0	0	0	0
Solyc02g090650.2.1	11	34	5	31	7	71	91	100	13	58	16	41	8
Solyc02g090670.2.1	7	113	14	63	0	172	102	141	12	56	13	60	5
Solyc02g090680.2.1	4	10	2	25	0	3	68	42	1	121	3	181	4
Solyc02g090690.1.1	0	0	0	0	0	0	0	0	0	0	0	0	0
Solyc02g090700.2.1	0	0	0	0	0	0	0	0	0	0	0	0	0
Solyc02g090710.2.1	10	85	9	49	3	126	66	77	9	198	19	138	1
Solyc02g090740.2.1	0	0	0	0	0	0	0	0	0	0	0	0	0
Solyc02g090750.2.1	2	2	2	0	1	0	4	8	2	2	6	3	0
Solyc02g090760.2.1	43	301	29	238	14	677	445	983	95	413	165	361	7
Solyc02g090770.1.1	0	0	0	0	0	0	0	0	0	0	0	0	0
Solyc02g090810.2.1	5	7	0	1	0	16	28	14	5	116	3	42	0
Solyc02g090820.2.1	10	143	6	44	4	314	40	383	54	44	24	75	1
Solyc02g090840.2.1	10	331	49	163	32	140	332	288	83	1542	81	609	16

Solyc02g090850.2.1	11	106	19	90	19	276	161	172	23	351	82	309	3
Solyc02g090860.2.1	0	0	0	0	0	0	0	0	0	0	0	0	0
Solyc02g090870.1.1	1	7	0	2	2	1	2	1	1	1	5	4	0
Solyc02g090890.2.1	30	1807	436	1067	35	649	412	694	12	335	204	322	10
Solyc02g090900.2.1	4	19	2	11	1	6	20	7	3	9	2	9	0
Solyc02g090910.2.1	6	97	11	38	14	392	181	244	23	159	51	120	0
Solyc02g090920.2.1	0	0	0	0	0	0	0	0	0	0	0	0	0
Solyc02g090930.2.1	2	12	0	0	0	0	2	0	0	13	0	11	0
Solyc02g090960.1.1	0	0	0	0	0	0	0	0	0	0	0	0	0
Solyc02g090970.1.1	2	18	2	2	0	9	0	2	4	0	2	0	0
Solyc02g090980.1.1	1	1	0	8	0	3	24	1	0	0	1	0	0
Solyc02g090990.1.1	0	0	0	0	0	0	0	0	0	0	0	0	0
Solyc02g091000.2.1	3	6	0	3	0	5	9	10	0	13	9	13	0
Solyc02g091010.1.1	0	0	0	0	0	0	0	0	0	0	0	0	0
Solyc02g091020.1.1	0	0	0	0	0	0	0	0	0	0	0	0	0
Solyc02g091030.2.1	4	9	2	12	0	67	23	19	3	31	3	31	0
Solyc02g091040.2.1	1	0	0	0	1	5	18	26	13	2	4	0	0
Solyc02g091070.2.1	0	0	0	0	0	0	0	0	0	0	0	0	0
Solyc02g091080.1.1	0	0	0	0	0	0	0	0	0	0	0	0	0
Solyc02g091090.2.1	1	4	1	0	0	1	3	3	0	0	1	2	0
Solyc02g091100.2.1	13	51	9	38	5	183	83	231	15	284	46	305	2
Solyc02g091110.2.1	1	0	0	2	0	0	0	0	0	5	3	2	0
Solyc02g091120.2.1	7	19	5	22	0	28	47	29	0	53	5	35	1
Solyc02g091130.2.1	0	0	0	0	0	0	0	0	0	0	0	0	0
Solyc02g091140.2.1	8	38	12	52	0	8	99	57	2	6	7	16	0
Solyc02g091160.2.1	0	0	0	0	0	0	0	0	0	0	0	0	0
Solyc02g091170.2.1	0	0	0	0	0	0	0	0	0	0	0	0	0
Solyc02g091180.1.1	3	172	21	82	20	3	17	5	0	8	0	31	0
Solyc02g091190.2.1	3	29	2	19	0	24	11	33	8	45	20	39	1
Solyc02g091210.1.1	4	12	1	6	0	52	22	34	1	53	23	40	0
Solyc02g091220.1.1	11	78	3	36	0	460	67	254	32	248	38	178	3
Solyc02g091240.1.1	12	290	175	292	45	1087	1522	881	63	1924	145	836	16
Solyc02g091250.1.1	1	14	1	1	0	15	4	6	0	0	0	5	0
Solyc02g091270.2.1	9	80	4	56	7	179	117	192	12	73	22	68	2
Solyc02g091280.2.1	7	142	19	73	1	239	136	366	9	188	31	117	1
Solyc02g091290.2.1	2	3	0	1	0	7	22	8	0	9	1	10	1
Solyc02g091300.2.1	0	0	0	0	0	0	0	0	0	0	0	0	0
Solyc02g091320.2.1	2	6	0	7	0	4	7	17	0	7	1	5	0
Solyc02g091330.2.1	5	10	1	4	0	16	23	31	1	32	8	26	0
Solyc02g091340.2.1	5	34	7	28	2	81	27	133	10	53	10	20	1
Solyc02g091350.2.1	0	0	0	0	0	0	0	0	0	0	0	0	0

Solyc02g091360.1.1	1	4	0	13	0	5	5	1	1	0	0	0	0
Solyc02g091370.2.1	0	0	0	0	0	0	0	0	0	0	0	0	0
Solyc02g091380.1.1	2	50	10	88	0	10	0	15	0	10	0	19	0
Solyc02g091410.2.1	8	27	3	33	0	109	55	73	9	78	18	76	3
Solyc02g091420.2.1	0	0	0	0	0	0	0	0	0	0	0	0	0
Solyc02g091430.2.1	6	46	1	43	3	636	68	253	31	233	7	113	1
Solyc02g091450.2.1	3	8	0	11	0	1	13	24	4	11	7	24	0
Solyc02g091460.2.1	1	4	1	0	0	2	9	8	1	12	0	1	0
Solyc02g091470.2.1	2	1	1	3	0	20	8	9	5	1	0	0	0
Solyc02g091490.2.1	7	237	19	91	6	178	90	262	4	330	66	257	2
Solyc02g091500.1.1	0	0	0	0	0	0	0	0	0	0	0	0	0
Solyc02g091510.2.1	14	395	12	152	8	585	93	419	34	609	46	215	3
Solyc02g091520.2.1	0	0	0	0	0	0	0	0	0	0	0	0	0
Solyc02g091530.2.1	2	11	0	4	0	12	9	21	0	10	1	5	0
Solyc02g091540.2.1	0	0	0	0	0	0	0	0	0	0	0	0	0
Solyc02g091560.2.1	6	4085	1126	2396	17	168	1651	216	1	747	378	1000	7
Solyc02g091580.2.1	11	190	35	112	19	374	380	354	63	276	116	338	5
Solyc02g091590.2.1	0	0	0	0	0	0	0	0	0	0	0	0	0
Solyc02g091600.1.1	0	0	0	0	0	0	0	0	0	0	0	0	0
Solyc02g091620.1.1	0	0	0	0	0	0	0	0	0	0	0	0	0
Solyc02g091630.1.1	0	0	0	0	0	0	0	0	0	0	0	0	0
Solyc02g091640.2.1	7	32	2	15	1	67	84	124	5	118	17	78	0
Solyc02g091650.2.1	1	2	0	1	0	15	5	7	3	0	0	0	0
Solyc02g091660.2.1	5	19	1	7	0	35	15	33	4	22	6	11	1
Solyc02g091670.1.1	5	40	8	53	1	36	45	23	0	162	87	116	14
Solyc02g091680.2.1	5	17	0	8	5	73	19	59	0	60	12	72	0
Solyc02g091690.2.1	3	34	0	4	0	3	0	3	0	311	31	349	3
Solyc02g091700.2.1	3	19	0	11	0	20	20	28	4	51	15	37	0
Solyc02g091710.2.1	3	6	1	0	0	29	77	42	3	0	0	1	0
Solyc02g091720.1.1	5	71	4	23	5	87	66	109	15	105	19	57	0
Solyc02g091730.1.1	0	0	0	0	0	0	0	0	0	0	0	0	0
Solyc02g091740.2.1	0	0	0	0	0	0	0	0	0	0	0	0	0
Solyc02g091750.2.1	0	0	0	0	0	0	0	0	0	0	0	0	0
Solyc02g091760.1.1	0	0	0	0	0	0	0	0	0	0	0	0	0
Solyc02g091770.1.1	0	0	0	0	0	0	0	0	0	0	0	0	0
Solyc02g091780.1.1	2	10	1	11	0	9	10	9	3	19	1	3	0
Solyc02g091790.2.1	5	29	0	24	0	25	20	44	3	16	12	12	0
Solyc02g091800.2.1	0	0	0	0	0	0	0	0	0	0	0	0	0
Solyc02g091830.2.1	0	0	0	0	0	0	0	0	0	0	0	0	0
Solyc02g091840.2.1	25	448	30	439	22	121	123	329	20	3622	366	2474	39
Solyc02g091860.2.1	11	54	3	74	1	60	66	135	17	77	21	53	0

Solyc02g091870.2.1	0	0	0	0	0	0	0	0	0	0	0	0	0
Solyc02g091880.2.1	4	119	5	50	7	296	94	224	24	196	44	86	2
Solyc02g091890.1.1	11	65	5	21	3	34	23	70	12	125	40	73	5
Solyc02g091900.2.1	4	36	8	15	6	67	35	92	6	86	22	98	0
Solyc02g091920.2.1	12	1783	196	428	266	9	235	15	52	994	17	678	2
Solyc02g091930.2.1	7	40	4	40	1	963	653	1145	54	119	23	64	0
Solyc02g091940.2.1	5	22	2	19	3	71	41	64	5	25	5	38	1
Solyc02g091950.2.1	8	17	6	6	6	115	41	73	3	94	9	48	0
Solyc02g091960.2.1	14	50	1	33	3	92	94	198	17	103	42	62	0
Solyc02g091970.2.1	10	127	14	64	0	252	163	233	7	447	51	163	6
Solyc02g091980.1.1	0	0	0	0	0	0	0	0	0	0	0	0	0
Solyc02g091990.2.1	0	0	0	0	0	0	0	0	0	0	0	0	0
Solyc02g092000.2.1	1	28	3	7	0	7	11	13	14	24	3	31	0
Solyc02g092010.2.1	3	9	2	9	0	40	24	56	7	12	7	16	0
Solyc02g092020.1.1	0	0	0	0	0	0	0	0	0	0	0	0	0
Solyc02g092040.1.1	0	0	0	0	0	0	0	0	0	0	0	0	0
Solyc02g092050.2.1	0	0	0	0	0	0	0	0	0	0	0	0	0
Solyc02g092070.2.1	0	0	0	0	0	0	0	0	0	0	0	0	0
Solyc02g092080.1.1	1	1	0	0	0	0	4	6	1	9	1	8	0
Solyc02g092090.1.1	1	0	1	6	0	10	4	12	0	5	3	1	0
Solyc02g092110.2.1	1	23	1	3	0	41	5	7	1	8	0	0	0
Solyc02g092120.2.1	1	3	2	1	0	27	0	26	6	0	2	0	3
Solyc02g092130.2.1	2	40	2	16	4	58	32	65	6	41	11	24	3
Solyc02g092140.1.1	7	19	2	12	0	157	62	67	3	8	8	5	0
Solyc02g092150.2.1	7	21	2	14	0	769	112	326	4	29	44	10	1
Solyc02g092160.2.1	12	206	16	139	14	46	72	111	12	341	52	179	5
Solyc02g092200.2.1	3	6	0	1	0	18	6	12	0	2	1	3	0
Solyc02g092210.1.1	1	11	0	3	0	0	4	1	3	4	0	0	0
Solyc02g092220.1.1	2	12	1	8	0	2	1	20	1	56	9	9	0
Solyc02g092230.2.1	2	10	0	4	0	39	4	45	3	8	0	3	0
Solyc02g092240.2.1	0	0	0	0	0	0	0	0	0	0	0	0	0
Solyc02g092250.2.1	0	0	0	0	0	0	0	0	0	0	0	0	0
Solyc02g092260.2.1	8	21	4	15	0	121	55	160	18	59	9	28	3
Solyc02g092270.2.1	3	376	17	137	6	902	48	751	21	539	62	355	4
Solyc02g092290.2.1	2	5	2	3	0	15	7	10	1	23	1	5	0
Solyc02g092300.1.1	0	0	0	0	0	0	0	0	0	0	0	0	0
Solyc02g092310.2.1	13	46	9	36	0	51	132	98	4	51	6	31	0
Solyc02g092320.2.1	2	8	0	7	0	8	26	12	1	2	2	5	0
Solyc02g092330.2.1	3	68	8	41	1	107	91	161	6	140	25	94	1
Solyc02g092340.2.1	0	0	0	0	0	0	0	0	0	0	0	0	0
Solyc02g092350.2.1	0	0	0	0	0	0	0	0	0	0	0	0	0

Solyc02g092360.2.1	9	36	8	29	0	70	31	49	10	62	12	84	0
Solyc02g092370.1.1	0	0	0	0	0	0	0	0	0	0	0	0	0
Solyc02g092380.2.1	10	50	2	29	0	115	49	123	9	72	25	77	1
Solyc02g092390.2.1	4	9	2	4	0	26	11	18	4	16	6	11	0
Solyc02g092400.1.1	0	0	0	0	0	0	0	0	0	0	0	0	0
Solyc02g092410.2.1	1	4	0	1	0	0	6	0	0	8	0	8	0
Solyc02g092420.2.1	0	0	0	0	0	0	0	0	0	0	0	0	0
Solyc02g092430.1.1	0	0	0	0	0	0	0	0	0	0	0	0	0
Solyc02g092440.2.1	7	200	14	102	9	213	51	181	23	460	23	200	2
Solyc02g092450.2.1	0	0	0	0	0	0	0	0	0	0	0	0	0
Solyc02g092460.2.1	1	1	0	4	0	0	3	2	1	5	2	3	0
Solyc02g092470.2.1	10	50	11	41	2	43	51	115	2	45	8	44	3
Solyc02g092480.2.1	9	136	6	112	2	100	82	289	26	200	19	185	0
Solyc02g092490.2.1	1	3	2	0	0	0	1	2	0	6	1	15	0
Solyc02g092500.1.1	0	0	0	0	0	0	0	0	0	0	0	0	0
Solyc02g092510.2.1	0	0	0	0	0	0	0	0	0	0	0	0	0
Solyc02g092520.1.1	0	0	0	0	0	0	0	0	0	0	0	0	0
Solyc02g092530.2.1	2	40	1	23	0	0	1	11	0	20	2	23	0
Solyc02g092550.2.1	0	0	0	0	0	0	0	0	0	0	0	0	0
Solyc02g092560.2.1	15	407	33	291	8	280	269	556	12	175	36	122	0
Solyc02g092570.1.1	0	0	0	0	0	0	0	0	0	0	0	0	0
Solyc02g092580.2.1	2	30	0	11	0	16	16	19	0	1	0	0	0
Solyc02g092590.2.1	0	0	0	0	0	0	0	0	0	0	0	0	0
Solyc02g092600.2.1	6	11	1	15	4	16	31	20	1	29	5	6	4
Solyc02g092610.2.1	0	0	0	0	0	0	0	0	0	0	0	0	0
Solyc02g092620.2.1	9	24	8	14	3	40	67	37	5	38	31	31	0
Solyc02g092640.2.1	5	67	7	26	5	77	41	92	4	203	27	123	2
Solyc02g092650.2.1	0	0	0	0	0	0	0	0	0	0	0	0	0
Solyc02g092660.2.1	0	0	0	0	0	0	0	0	0	0	0	0	0
Solyc02g092670.1.1	24	1128	61	741	5	42	314	306	1	982	553	517	2
Solyc02g092680.1.1	0	0	0	0	0	0	0	0	0	0	0	0	0
Solyc02g092700.2.1	9	976	73	433	2	296	112	192	0	136	69	103	4
Solyc02g092710.2.1	4	24	10	10	1	13	45	17	4	32	11	18	1
Solyc02g092720.1.1	0	0	0	0	0	0	0	0	0	0	0	0	0
Solyc02g092730.2.1	2	3	2	0	0	0	15	0	7	7	5	0	1
Solyc02g092740.1.1	0	0	0	0	0	0	0	0	0	0	0	0	0
Solyc02g092750.2.1	7	103	15	107	11	97	27	73	8	18	4	46	6
Solyc02g092760.2.1	1	4	0	3	0	1	4	4	0	1	0	13	0
Solyc02g092770.2.1	4	9	1	8	3	25	36	26	11	41	3	8	0
Solyc02g092780.1.1	14	67	14	61	0	163	98	133	27	102	21	145	4
Solyc02g092790.2.1	5	1121	20	303	38	199	63	103	3	926	316	507	13

Solyc02g092800.2.1	8	33	0	13	0	53	36	108	1	74	22	89	0
Solyc02g092810.2.1	2	8	0	11	1	2	14	7	1	10	1	6	0
Solyc02g092820.2.1	0	0	0	0	0	0	0	0	0	0	0	0	0
Solyc02g092830.2.1	2	16	0	15	0	10	11	16	1	22	4	9	0
Solyc02g092840.1.1	12	186	13	45	9	111	48	149	16	635	76	325	9
Solyc02g092860.2.1	6	12	4	3	4	89	29	66	10	59	37	33	1
Solyc02g092870.1.1	0	0	0	0	0	0	0	0	0	0	0	0	0
Solyc02g092880.2.1	3	0	1	2	0	6	1	16	0	82	23	51	1
Solyc02g092890.1.1	0	0	0	0	0	0	0	0	0	0	0	0	0
Solyc02g092910.2.1	2	11	0	19	0	44	7	32	4	11	21	10	0
Solyc02g092920.2.1	16	148	27	153	19	137	203	380	28	181	64	177	10
Solyc02g092930.1.1	15	1023	47	541	4	337	93	176	19	294	37	374	4
Solyc02g092940.2.1	15	107	13	60	12	135	91	84	5	112	18	89	1
Solyc02g092950.2.1	0	0	0	0	0	0	0	0	0	0	0	0	0
Solyc02g092970.2.1	1	1	0	0	0	0	12	4	0	3	0	0	0
Solyc02g092980.2.1	3	24	11	17	1	9	5	15	1	38	4	68	1
Solyc02g092990.1.1	10	111	44	59	3	136	56	54	43	87	12	77	0
Solyc02g093000.2.1	5	11	2	30	2	34	41	43	6	76	23	38	3
Solyc02g093010.2.1	3	6	3	11	7	16	34	31	1	63	7	59	6
Solyc02g093020.2.1	4	25	1	14	0	29	14	17	0	23	2	40	0
Solyc02g093030.2.1	1	3	2	5	0	7	2	5	0	12	1	3	1
Solyc02g093040.2.1	0	0	0	0	0	0	0	0	0	0	0	0	0
Solyc02g093050.2.1	3	16	1	9	2	95	15	68	17	1	2	5	0
Solyc02g093060.2.1	8	49	2	85	5	47	56	99	4	68	15	69	6
Solyc02g093080.2.1	8	66	5	49	4	33	55	52	3	242	31	111	1
Solyc02g093090.1.1	1	10	0	3	0	2	4	9	0	2	2	0	0
Solyc02g093100.2.1	1	1	0	2	0	1	0	0	0	3	4	6	0
Solyc02g093110.2.1	3	63	2	55	0	87	19	56	2	81	27	194	0
Solyc02g093120.2.1	3	22	0	16	0	9	12	21	2	22	3	13	0
Solyc02g093130.1.1	2	19	1	10	0	52	19	41	4	24	3	10	1
Solyc02g093140.2.1	3	57	19	23	4	28	14	34	6	23	4	9	0
Solyc02g093150.2.1	4	111	26	112	15	191	43	88	32	729	17	419	3
Solyc02g093160.2.1	5	44	5	26	1	58	23	62	9	23	4	22	0
Solyc02g093170.2.1	4	60	4	19	0	214	11	253	6	52	8	51	0
Solyc02g093180.2.1	19	9	0	23	0	5871	903	5631	2	62	16	20	0
Solyc02g093190.2.1	2	0	0	0	0	5	7	3	0	0	0	2	0
Solyc02g093200.2.1	0	0	0	0	0	0	0	0	0	0	0	0	0
Solyc02g093210.2.1	4	28	7	9	3	4	9	9	4	15	0	19	0
Solyc02g093220.2.1	9	122	2	51	11	204	47	149	6	42	35	31	1
Solyc02g093230.2.1	0	0	0	0	0	0	0	0	0	0	0	0	0
Solyc02g093240.1.1	0	0	0	0	0	0	0	0	0	0	0	0	0

Solyc02g093250.2.1	2	1	1	0	0	5	9	3	12	1	1	5	0
Solyc02g093270.2.1	5	113	4	100	3	2505	254	1608	236	49	12	29	0
Solyc02g093280.2.1	0	0	0	0	0	0	0	0	0	0	0	0	0
Solyc02g093290.2.1	3	23	5	7	2	13	93	26	0	23	32	35	1
Solyc02g093300.2.1	1	0	0	0	3	2	0	1	0	0	4	1	0
Solyc02g093310.2.1	3	13	2	4	5	16	16	10	6	32	11	12	0
Solyc02g093320.2.1	1	0	0	0	0	11	11	2	5	7	0	0	0
Solyc02g093330.2.1	11	16	1	16	0	44	55	61	8	20	2	12	0
Solyc02g093340.2.1	5	107	11	58	9	101	36	134	12	126	27	111	2
Solyc02g093350.2.1	0	0	0	0	0	0	0	0	0	0	0	0	0
Solyc02g093360.2.1	11	63	12	66	3	153	112	220	12	26	5	52	3
Solyc02g093370.2.1	5	93	10	80	2	254	103	240	28	227	56	158	4
Solyc02g093380.1.1	0	0	0	0	0	0	0	0	0	0	0	0	0
Solyc02g093390.2.1	5	20	10	5	7	52	119	48	35	17	52	17	2
Solyc02g093400.1.1	0	0	0	0	0	0	0	0	0	0	0	0	0
Solyc02g093410.2.1	8	136	7	92	1	147	52	153	3	114	17	76	0
Solyc02g093420.2.1	0	0	0	0	0	0	0	0	0	0	0	0	0
Solyc02g093430.2.1	1	13	3	3	0	1	15	6	1	1	6	4	0
Solyc02g093440.2.1	7	80	2	85	0	139	103	240	29	117	20	75	1
Solyc02g093450.2.1	1	2	1	2	1	3	11	17	0	15	0	6	0
Solyc02g093460.2.1	9	26	2	15	0	25	60	54	1	28	19	53	3
Solyc02g093470.2.1	6	87	6	42	9	80	77	96	25	122	16	73	4
Solyc02g093480.2.1	0	0	0	0	0	0	0	0	0	0	0	0	0
Solyc02g093490.2.1	3	37	4	13	2	50	57	53	11	161	8	74	1
Solyc02g093500.2.1	1	1	0	0	0	3	3	0	0	1	4	8	0
Solyc02g093510.2.1	4	9	2	7	0	39	30	30	3	34	11	18	0
Solyc02g093520.2.1	10	101	5	76	3	97	83	107	8	103	19	98	1
Solyc02g093530.2.1	1	7	0	0	0	11	11	13	0	7	0	1	0
Solyc02g093540.2.1	0	0	0	0	0	0	0	0	0	0	0	0	0
Solyc02g093550.2.1	4	33	1	19	0	6	23	22	0	25	1	12	0
Solyc02g093560.1.1	1	2	3	4	0	2	0	4	0	4	0	1	0
Solyc02g093570.2.1	0	0	0	0	0	0	0	0	0	0	0	0	0
Solyc02g093580.2.1	1	2	0	2	0	12	5	12	0	0	0	0	0
Solyc02g093590.2.1	8	21	2	15	0	47	58	86	4	74	34	33	9
Solyc02g093600.2.1	0	0	0	0	0	0	0	0	0	0	0	0	0
Solyc02g093610.2.1	1	2	0	1	0	5	4	0	0	6	0	9	0
Solyc02g093620.1.1	18	219	42	136	5	118	171	168	10	126	55	138	2
Solyc02g093630.2.1	9	408	69	233	25	477	407	659	47	807	144	510	7
Solyc02g093640.2.1	6	72	3	19	15	54	51	64	111	39	19	25	0
Solyc02g093670.2.1	0	0	0	0	0	0	0	0	0	0	0	0	0
Solyc02g093680.2.1	5	88	7	60	4	326	125	376	38	259	14	166	3

Solyc02g093690.2.1	1	6	1	2	0	11	7	9	0	13	2	19	0
Solyc02g093700.2.1	0	0	0	0	0	0	0	0	0	0	0	0	0
Solyc02g093710.1.1	0	0	0	0	0	0	0	0	0	0	0	0	0
Solyc02g093720.2.1	3	69	4	21	0	0	5	3	0	1	0	0	0
Solyc02g093730.2.1	2	3	0	3	0	0	0	4	0	26	5	5	0
Solyc02g093740.2.1	0	0	0	0	0	0	0	0	0	0	0	0	0
Solyc02g093750.2.1	2	2	0	7	0	2	7	22	1	7	0	2	0
Solyc02g093760.2.1	0	0	0	0	0	0	0	0	0	0	0	0	0
Solyc02g093770.2.1	15	74	3	41	4	82	98	218	14	266	16	305	3
Solyc02g093790.2.1	2	10	1	1	1	3	7	5	2	6	1	3	4
Solyc02g093800.2.1	9	72	3	31	3	39	41	70	5	77	13	102	4
Solyc02g093810.2.1	2	2	1	0	0	7	6	7	1	19	1	17	0
Solyc02g093820.2.1	7	42	3	62	0	191	52	163	5	151	40	125	2
Solyc02g093830.2.1	7	198	10	89	7	612	370	783	105	729	105	391	11
Solyc02g093840.2.1	8	29	2	18	0	71	108	85	21	30	19	32	1
Solyc02g093850.2.1	1	0	2	0	0	6	6	20	0	3	1	6	0
Solyc02g093860.2.1	0	0	0	0	0	0	0	0	0	0	0	0	0
Solyc02g093880.2.1	9	114	8	116	1	182	57	263	13	134	11	78	2
Solyc02g093890.1.1	13	3	0	0	0	323	87	286	8	0	0	0	0
Solyc02g093900.2.1	4	76	0	35	4	219	32	192	9	313	22	196	0
Solyc02g093910.2.1	1	1	0	0	0	6	2	5	0	8	2	3	0
Solyc02g093920.2.1	10	72	11	53	1	237	143	278	36	162	27	135	5
Solyc02g093930.2.1	2	7	1	6	0	3	2	11	0	9	4	8	0
Solyc02g093940.2.1	4	22	3	5	0	12	12	38	0	14	7	8	0
Solyc02g093950.2.1	0	0	0	0	0	0	0	0	0	0	0	0	0
Solyc02g093960.2.1	9	1	8	0	0	145	453	208	194	4	1	0	0
Solyc02g093970.2.1	2	23	2	5	0	59	15	41	6	9	0	11	0
Solyc02g093980.2.1	11	176	11	75	3	37	45	144	16	146	39	111	3
Solyc02g093990.2.1	0	0	0	0	0	0	0	0	0	0	0	0	0
Solyc02g094000.1.1	0	0	0	0	0	0	0	0	0	0	0	0	0
Solyc02g094010.1.1	0	0	0	0	0	0	0	0	0	0	0	0	0
Solyc02g094030.2.1	10	261	42	261	10	1254	159	1004	113	249	39	271	3
Solyc02g094050.2.1	2	4	0	2	0	59	5	31	0	0	0	0	0
Solyc02g094060.2.1	1	1	0	0	0	0	3	15	0	3	0	0	0
Solyc02g094090.2.1	9	64	6	52	15	139	137	154	18	250	41	183	0
Solyc02g094100.2.1	0	0	0	0	0	0	0	0	0	0	0	0	0
Solyc02g094120.2.1	8	433	37	228	3	90	86	112	15	809	106	508	9
Solyc02g094130.2.1	5	321	22	181	5	207	129	260	16	509	51	384	9
Solyc02g094140.2.1	2	38	0	29	0	58	23	79	5	37	14	44	0
Solyc02g094150.2.1	4	5	2	13	1	12	38	13	0	12	6	14	0
Solyc02g094160.1.1	0	0	0	0	0	0	0	0	0	0	0	0	0

Solyc02g094170.2.1	7	35	3	6	3	87	71	52	7	50	21	35	1
Solyc02g094180.2.1	0	0	0	0	0	0	0	0	0	0	0	0	0
Solyc02g094190.2.1	2	3	0	0	0	52	17	32	0	9	1	0	0
Solyc02g094200.2.1	3	192	10	69	2	172	125	232	18	356	25	145	2
Solyc02g094210.1.1	0	0	0	0	0	0	0	0	0	0	0	0	0
Solyc02g094220.1.1	2	32	7	37	3	31	77	28	5	71	12	45	9
Solyc02g094230.1.1	0	0	0	0	0	0	0	0	0	0	0	0	0
Solyc02g094240.1.1	0	0	0	0	0	0	0	0	0	0	0	0	0
Solyc02g094250.2.1	1	8	0	0	0	6	10	16	1	11	4	11	0
Solyc02g094260.1.1	10	14	2	14	1	41	60	64	6	37	11	40	14
Solyc02g094280.2.1	9	51	10	37	6	66	80	100	11	91	21	61	1
Solyc02g094290.1.1	0	0	0	0	0	0	0	0	0	0	0	0	0
Solyc02g094300.2.1	1	12	0	12	2	8	9	4	0	7	0	3	0
Solyc02g094310.1.1	1	0	0	0	0	5	4	3	1	5	1	1	0
Solyc02g094320.2.1	5	54	2	22	4	87	36	82	3	178	11	102	0
Solyc02g094330.2.1	1	6	3	3	1	0	1	0	0	3	2	4	1
Solyc02g094350.2.1	2	5	1	2	0	9	12	16	4	45	13	19	0
Solyc02g094360.2.1	10	38	1	29	5	112	43	93	10	86	24	103	2
Solyc02g094370.1.1	0	0	0	0	0	0	0	0	0	0	0	0	0
Solyc02g094390.2.1	3	25	3	4	0	0	20	27	4	60	19	72	0
Solyc02g094400.2.1	8	54	19	36	5	27	19	44	0	185	5	134	6
Solyc02g094410.2.1	9	41	10	48	15	69	112	106	15	99	43	82	0
Solyc02g094420.2.1	3	91	6	38	0	9	88	32	6	6	11	23	0
Solyc02g094430.2.1	5	11	0	15	3	38	46	70	3	34	10	23	0
Solyc02g094440.2.1	3	108	0	31	5	238	45	220	20	93	22	159	3
Solyc02g094450.2.1	1	17	0	2	0	5	6	8	1	15	1	10	0
Solyc02g094470.2.1	5	85	7	50	3	266	65	332	18	268	31	131	0
Solyc02g094480.2.1	3	34	1	21	1	37	3	27	2	46	9	15	2
Solyc02g094490.2.1	2	28	2	24	3	49	4	62	3	46	5	22	0
Solyc02g094500.2.1	8	40	8	25	5	346	99	125	17	175	45	95	1
Solyc02g094510.2.1	11	74	6	57	0	173	59	202	12	173	32	103	1
Solyc02g094520.2.1	0	0	0	0	0	0	0	0	0	0	0	0	0
Solyc02g094530.1.1	5	16	6	24	0	50	49	78	5	46	6	37	0
Solyc02g094540.2.1	17	212	36	150	16	510	347	541	46	258	99	236	15
Solyc02g094550.2.1	2	22	0	16	2	18	14	38	4	20	3	37	0
Solyc02g094560.2.1	1	2	0	10	0	4	14	22	0	7	0	2	2
Solyc02g094570.2.1	0	0	0	0	0	0	0	0	0	0	0	0	0
Solyc02g094580.2.1	0	0	0	0	0	0	0	0	0	0	0	0	0
Solyc02g094590.2.1	2	4	3	1	0	4	25	9	0	15	2	6	1
Solyc02g094600.2.1	1	7	0	0	0	4	6	7	1	6	6	6	1
Solyc02g094610.1.1	1	1	1	0	0	7	3	5	1	3	2	8	0

Solyc02g094620.1.1	0	0	0	0	0	0	0	0	0	0	0	0	0
Solyc02g094630.2.1	0	0	0	0	0	0	0	0	0	0	0	0	0
Solyc02g094640.2.1	10	219	24	141	10	109	370	129	17	122	57	99	1
Solyc02g094830.2.1	0	0	0	0	0	0	0	0	0	0	0	0	0
Solyc03g005000.2.1	5	152	33	81	9	55	386	103	2	126	58	160	5
Solyc03g005010.2.1	1	2	0	1	0	0	5	1	0	2	0	8	0
Solyc03g005020.2.1	11	39	0	20	0	18	2	29	0	80	670	146	2
Solyc03g005030.2.1	6	49	3	35	10	5	18	22	9	41	14	51	0
Solyc03g005040.1.1	0	0	0	0	0	0	0	0	0	0	0	0	0
Solyc03g005050.2.1	2	20	2	13	0	30	5	23	0	119	36	131	2
Solyc03g005060.2.1	0	0	0	0	0	0	0	0	0	0	0	0	0
Solyc03g005070.1.1	3	9	7	17	0	28	37	60	2	18	7	9	0
Solyc03g005080.2.1	3	18	2	4	2	45	60	53	8	48	1	54	0
Solyc03g005090.2.1	6	78	6	71	0	50	28	52	3	80	7	60	0
Solyc03g005100.2.1	4	51	4	22	2	25	37	39	5	94	18	42	0
Solyc03g005110.2.1	6	58	1	42	1	267	39	139	7	177	6	132	0
Solyc03g005120.2.1	2	32	9	25	1	22	23	30	3	20	2	11	0
Solyc03g005130.1.1	10	75	14	70	1	106	86	144	25	66	39	49	3
Solyc03g005140.1.1	0	0	0	0	0	0	0	0	0	0	0	0	0
Solyc03g005150.2.1	0	0	0	0	0	0	0	0	0	0	0	0	0
Solyc03g005170.2.1	6	20	0	24	2	42	33	50	10	22	4	36	0
Solyc03g005180.1.1	0	0	0	0	0	0	0	0	0	0	0	0	0
Solyc03g005190.2.1	5	93	29	42	2	22	95	41	3	94	8	64	9
Solyc03g005200.2.1	0	0	0	0	0	0	0	0	0	0	0	0	0
Solyc03g005210.2.1	3	2	0	0	0	25	90	21	42	1	5	0	0
Solyc03g005220.2.1	4	13	7	11	1	4	13	11	1	29	25	58	3
Solyc03g005230.2.1	13	1657	216	556	8	254	506	428	15	382	97	261	3
Solyc03g005250.2.1	2	6	3	0	1	14	16	7	0	9	5	8	2
Solyc03g005260.2.1	6	50	11	22	1	11	8	30	4	12	14	10	0
Solyc03g005270.2.1	6	83	2	15	5	184	64	192	20	160	31	61	4
Solyc03g005280.2.1	0	0	0	0	0	0	0	0	0	0	0	0	0
Solyc03g005290.1.1	0	0	0	0	0	0	0	0	0	0	0	0	0
Solyc03g005300.2.1	6	112	9	60	3	116	106	150	19	123	35	122	3
Solyc03g005310.2.1	1	2	0	1	0	4	4	5	0	2	1	12	0
Solyc03g005320.2.1	3	13	0	3	0	23	6	7	15	1	9	0	0
Solyc03g005330.1.1	1	2	0	10	0	1	1	7	1	13	3	3	0
Solyc03g005340.2.1	11	44	7	60	5	110	64	144	7	138	31	90	1
Solyc03g005350.2.1	6	14	4	14	2	29	62	41	6	22	18	38	0
Solyc03g005380.2.1	3	8	11	3	0	56	113	25	6	47	34	30	1
Solyc03g005390.2.1	1	3	2	1	0	1	0	9	0	22	1	10	0
Solyc03g005410.2.1	5	62	8	29	2	52	52	84	9	106	28	125	3

Solyc03g005420.1.1	1	8	0	7	0	1	6	2	0	4	0	0	0
Solyc03g005430.2.1	10	114	11	81	10	178	105	199	11	205	48	149	7
Solyc03g005440.2.1	1	1	0	5	0	12	3	5	1	12	2	0	0
Solyc03g005450.2.1	4	21	1	40	0	84	17	80	0	21	4	9	0
Solyc03g005460.2.1	0	0	0	0	0	0	0	0	0	0	0	0	0
Solyc03g005470.2.1	4	100	5	47	5	131	35	124	7	175	22	119	1
Solyc03g005480.2.1	0	0	0	0	0	0	0	0	0	0	0	0	0
Solyc03g005490.2.1	0	0	0	0	0	0	0	0	0	0	0	0	0
Solyc03g005520.1.1	0	0	0	0	0	0	0	0	0	0	0	0	0
Solyc03g005570.2.1	1	6	4	3	1	2	0	1	0	0	0	0	0
Solyc03g005580.2.1	3	1	0	0	0	0	0	0	0	25	693	15	5
Solyc03g005600.2.1	10	104	17	63	8	50	95	106	15	275	91	246	12
Solyc03g005620.2.1	0	0	0	0	0	0	0	0	0	0	0	0	0
Solyc03g005630.2.1	0	0	0	0	0	0	0	0	0	0	0	0	0
Solyc03g005660.2.1	13	15	10	20	1	43	99	91	6	73	19	52	0
Solyc03g005670.2.1	1	0	0	2	0	5	14	4	0	0	0	0	0
Solyc03g005680.2.1	11	166	7	101	7	175	59	302	9	249	33	127	1
Solyc03g005690.2.1	1	1	1	0	0	0	1	4	11	6	2	1	21
Solyc03g005700.1.1	0	0	0	0	0	0	0	0	0	0	0	0	0
Solyc03g005730.2.1	16	442	102	291	46	244	300	372	51	330	200	248	4
Solyc03g005740.1.1	1	28	6	27	0	71	39	91	9	82	46	64	7
Solyc03g005750.2.1	15	130	12	139	11	303	133	538	18	344	28	336	2
Solyc03g005760.1.1	1	690	31	855	2	80	1	53	1	54	7	80	0
Solyc03g005770.1.1	8	3313	997	2276	32	2	51	13	6	76	1427	62	19
Solyc03g005780.1.1	3	2738	689	1304	5	6	1	8	1	9	136	3	3
Solyc03g005790.2.1	0	0	0	0	0	0	0	0	0	0	0	0	0
Solyc03g005800.2.1	9	48	17	38	3	79	313	79	72	101	75	64	4
Solyc03g005810.2.1	0	0	0	0	0	0	0	0	0	0	0	0	0
Solyc03g005820.2.1	1	6	0	5	0	0	4	10	0	0	0	0	0
Solyc03g005830.2.1	3	14	0	10	1	23	13	49	1	25	2	4	0
Solyc03g005840.1.1	0	0	0	0	0	0	0	0	0	0	0	0	0
Solyc03g005860.2.1	0	0	0	0	0	0	0	0	0	0	0	0	0
Solyc03g005870.2.1	2	26	4	10	0	33	22	33	4	80	9	50	1
Solyc03g005880.2.1	0	0	0	0	0	0	0	0	0	0	0	0	0
Solyc03g005890.1.1	0	0	0	0	0	0	0	0	0	0	0	0	0
Solyc03g005900.2.1	0	0	0	0	0	0	0	0	0	0	0	0	0
Solyc03g005910.2.1	0	0	0	0	0	0	0	0	0	0	0	0	0
Solyc03g005920.2.1	1	8	0	6	3	9	3	7	0	16	5	18	0
Solyc03g005930.2.1	0	0	0	0	0	0	0	0	0	0	0	0	0
Solyc03g005940.2.1	0	0	0	0	0	0	0	0	0	0	0	0	0
Solyc03g005960.2.1	0	0	0	0	0	0	0	0	0	0	0	0	0

Solyc03g005970.2.1	0	0	0	0	0	0	0	0	0	0	0	0	0
Solyc03g005980.2.1	0	0	0	0	0	0	0	0	0	0	0	0	0
Solyc03g005990.2.1	3	17	2	8	5	13	14	7	0	7	1	4	0
Solyc03g006000.2.1	2	6	2	0	0	22	8	8	4	17	4	8	0
Solyc03g006010.2.1	0	0	0	0	0	0	0	0	0	0	0	0	0
Solyc03g006020.2.1	3	14	0	2	4	4	6	7	0	19	3	20	0
Solyc03g006030.2.1	0	0	0	0	0	0	0	0	0	0	0	0	0
Solyc03g006050.2.1	0	0	0	0	0	0	0	0	0	0	0	0	0
Solyc03g006060.2.1	3	17	0	9	3	19	13	36	1	20	4	6	0
Solyc03g006070.2.1	3	82	4	56	1	107	60	132	5	149	19	60	6
Solyc03g006080.2.1	0	0	0	0	0	0	0	0	0	0	0	0	0
Solyc03g006100.2.1	0	0	0	0	0	0	0	0	0	0	0	0	0
Solyc03g006130.2.1	9	40	3	10	0	149	40	146	5	94	7	87	1
Solyc03g006140.2.1	17	402	42	174	16	354	353	590	21	528	190	369	12
Solyc03g006150.2.1	0	0	0	0	0	0	0	0	0	0	0	0	0
Solyc03g006160.1.1	0	0	0	0	0	0	0	0	0	0	0	0	0
Solyc03g006180.2.1	1	2	0	0	0	0	3	4	1	2	1	0	0
Solyc03g006190.2.1	0	0	0	0	0	0	0	0	0	0	0	0	0
Solyc03g006210.1.1	0	0	0	0	0	0	0	0	0	0	0	0	0
Solyc03g006220.2.1	2	1	1	4	0	59	12	38	0	11	0	1	0
Solyc03g006240.2.1	0	0	0	0	0	0	0	0	0	0	0	0	0
Solyc03g006250.2.1	3	42	11	49	0	27	16	57	8	11	2	27	3
Solyc03g006260.2.1	1	5	2	8	0	1	27	3	0	3	7	7	0
Solyc03g006270.2.1	0	0	0	0	0	0	0	0	0	0	0	0	0
Solyc03g006280.2.1	0	0	0	0	0	0	0	0	0	0	0	0	0
Solyc03g006290.2.1	0	0	0	0	0	0	0	0	0	0	0	0	0
Solyc03g006300.1.1	0	0	0	0	0	0	0	0	0	0	0	0	0
Solyc03g006310.2.1	1	1	0	0	0	6	3	7	0	30	2	18	0
Solyc03g006320.1.1	4	11	0	3	0	15	34	24	17	3	0	10	1
Solyc03g006330.2.1	0	0	0	0	0	0	0	0	0	0	0	0	0
Solyc03g006340.2.1	0	0	0	0	0	0	0	0	0	0	0	0	0
Solyc03g006350.2.1	10	22	2	22	2	120	72	90	8	54	13	55	2
Solyc03g006360.2.1	3	0	3	6	7	37	1449	66	19	0	7	0	0
Solyc03g006380.2.1	0	0	0	0	0	0	0	0	0	0	0	0	0
Solyc03g006390.1.1	0	0	0	0	0	0	0	0	0	0	0	0	0
Solyc03g006400.2.1	1	0	0	0	0	4	2	0	5	0	2	0	0
Solyc03g006410.2.1	3	17	3	5	1	0	10	0	0	0	0	1	0
Solyc03g006420.2.1	3	6	1	12	1	2	9	11	3	46	5	24	0
Solyc03g006430.2.1	1	11	0	9	0	2	6	2	0	16	0	1	0
Solyc03g006440.1.1	0	0	0	0	0	0	0	0	0	0	0	0	0
Solyc03g006450.1.1	2	12	2	0	6	2	0	1	3	0	115	1	13

Solyc03g006460.2.1	4	8	0	7	0	14	12	9	1	17	0	8	0
Solyc03g006470.2.1	0	0	0	0	0	0	0	0	0	0	0	0	0
Solyc03g006480.1.1	0	0	0	0	0	0	0	0	0	0	0	0	0
Solyc03g006490.2.1	2	15	1	16	4	5	129	30	9	200	1	102	2
Solyc03g006500.2.1	3	50	6	5	0	304	26	127	8	57	11	12	0
Solyc03g006510.2.1	8	86	6	67	12	147	70	135	17	93	30	72	3
Solyc03g006520.2.1	6	19	7	8	1	54	33	58	4	43	10	34	1
Solyc03g006540.2.1	4	46	1	19	0	1	25	4	0	0	0	0	0
Solyc03g006550.2.1	0	0	0	0	0	0	0	0	0	0	0	0	0
Solyc03g006560.2.1	2	5	0	4	0	11	9	12	3	9	3	7	0
Solyc03g006570.2.1	7	35	8	40	2	139	20	118	8	48	11	31	0
Solyc03g006580.2.1	1	6	0	10	0	4	8	10	1	10	1	3	0
Solyc03g006600.1.1	0	0	0	0	0	0	0	0	0	0	0	0	0
Solyc03g006610.2.1	2	14	3	4	0	1	5	7	0	2	2	0	0
Solyc03g006620.2.1	9	50	4	53	0	41	12	5	4	21	25	44	1
Solyc03g006630.2.1	0	0	0	0	0	0	0	0	0	0	0	0	0
Solyc03g006640.2.1	7	14	2	5	1	31	9	44	3	11	11	22	0
Solyc03g006650.1.1	0	0	0	0	0	0	0	0	0	0	0	0	0
Solyc03g006660.2.1	4	15	3	13	0	25	16	22	3	21	8	19	0
Solyc03g006670.2.1	0	0	0	0	0	0	0	0	0	0	0	0	0
Solyc03g006680.2.1	8	119	8	82	17	142	72	119	13	319	66	213	9
Solyc03g006700.2.1	2	4	0	1	0	62	20	54	7	0	0	0	0
Solyc03g006740.1.1	0	0	0	0	0	0	0	0	0	0	0	0	0
Solyc03g006750.1.1	0	0	0	0	0	0	0	0	0	0	0	0	0
Solyc03g006760.2.1	3	1	0	1	0	2	11	0	4	7	6	16	0
Solyc03g006770.2.1	0	0	0	0	0	0	0	0	0	0	0	0	0
Solyc03g006780.2.1	0	0	0	0	0	0	0	0	0	0	0	0	0
Solyc03g006790.1.1	0	0	0	0	0	0	0	0	0	0	0	0	0
Solyc03g006800.1.1	0	0	0	0	0	0	0	0	0	0	0	0	0
Solyc03g006810.2.1	1	1	0	4	0	23	11	33	0	0	0	0	0
Solyc03g006820.2.1	2	3	0	2	0	14	14	8	9	6	1	6	0
Solyc03g006830.2.1	7	92	9	47	10	65	24	48	2	12	7	10	0
Solyc03g006840.2.1	0	0	0	0	0	0	0	0	0	0	0	0	0
Solyc03g006850.2.1	1	5	1	1	0	2	0	4	1	5	3	4	0
Solyc03g006860.2.1	4	14	1	13	6	121	20	86	6	89	19	70	0
Solyc03g006870.2.1	7	105	12	86	0	107	29	108	8	29	14	188	0
Solyc03g006880.2.1	3	66	18	40	0	2	38	15	0	1	2	0	0
Solyc03g006900.1.1	0	0	0	0	0	0	0	0	0	0	0	0	0
Solyc03g006910.2.1	0	0	0	0	0	0	0	0	0	0	0	0	0
Solyc03g006920.2.1	3	16	0	10	2	11	22	21	10	23	4	9	0
Solyc03g006930.1.1	0	0	0	0	0	0	0	0	0	0	0	0	0

Solyc03g006960.2.1	8	9	5	10	0	37	34	37	13	65	26	40	0
Solyc03g006970.1.1	4	81	1	5	3	21	8	48	8	63	12	22	5
Solyc03g006980.2.1	7	94	8	53	0	27	85	59	1	0	4	2	0
Solyc03g006990.1.1	0	0	0	0	0	0	0	0	0	0	0	0	0
Solyc03g007000.2.1	3	10	0	1	2	7	36	22	11	14	13	3	6
Solyc03g007010.2.1	1	4	1	1	0	6	2	6	1	3	1	0	0
Solyc03g007030.2.1	1	32	4	9	1	0	0	0	0	9	0	11	0
Solyc03g007040.2.1	0	0	0	0	0	0	0	0	0	0	0	0	0
Solyc03g007050.2.1	1	2	4	0	12	0	13	0	2	3	0	0	0
Solyc03g007060.2.1	1	0	0	1	0	4	0	1	0	4	0	1	0
Solyc03g007070.2.1	1	5	1	3	0	3	5	11	0	3	0	6	0
Solyc03g007080.2.1	0	0	0	0	0	0	0	0	0	0	0	0	0
Solyc03g007090.1.1	6	10	4	17	0	16	16	20	2	23	20	23	2
Solyc03g007100.2.1	3	3	2	0	0	31	15	15	2	7	6	10	0
Solyc03g007110.2.1	7	59	8	32	0	66	85	83	3	81	22	58	3
Solyc03g007120.2.1	11	55	12	69	3	85	111	133	37	148	89	109	12
Solyc03g007130.2.1	0	0	0	0	0	0	0	0	0	0	0	0	0
Solyc03g007140.2.1	3	19	0	13	0	28	48	14	8	10	6	16	0
Solyc03g007150.2.1	0	0	0	0	0	0	0	0	0	0	0	0	0
Solyc03g007170.2.1	10	23	3	21	3	37	32	72	17	46	29	49	4
Solyc03g007180.1.1	4	16	18	17	1	4	7	2	3	19	25	34	4
Solyc03g007190.2.1	6	139	5	55	0	159	42	166	31	198	39	167	8
Solyc03g007200.1.1	0	0	0	0	0	0	0	0	0	0	0	0	0
Solyc03g007210.2.1	11	43	13	18	4	94	48	53	12	56	16	59	2
Solyc03g007220.2.1	0	0	0	0	0	0	0	0	0	0	0	0	0
Solyc03g007230.2.1	20	121	5	115	9	4223	633	2273	85	570	168	302	6
Solyc03g007240.2.1	11	193	15	83	32	168	128	198	55	139	68	161	1
Solyc03g007250.1.1	3	34	0	36	0	39	8	81	7	26	6	24	0
Solyc03g007260.2.1	2	17	5	15	0	19	16	50	9	10	5	10	0
Solyc03g007270.2.1	8	73	3	29	5	171	123	210	19	210	26	142	10
Solyc03g007280.2.1	1	6	0	4	0	2	2	5	3	5	2	3	0
Solyc03g007290.2.1	0	0	0	0	0	0	0	0	0	0	0	0	0
Solyc03g007300.1.1	0	0	0	0	0	0	0	0	0	0	0	0	0
Solyc03g007310.2.1	0	0	0	0	0	0	0	0	0	0	0	0	0
Solyc03g007320.2.1	8	58	16	44	23	146	109	153	43	151	55	120	49
Solyc03g007330.2.1	11	75	27	71	6	32	74	69	11	52	20	100	6
Solyc03g007340.2.1	2	4	0	0	0	24	0	13	0	10	9	2	0
Solyc03g007350.1.1	0	0	0	0	0	0	0	0	0	0	0	0	0
Solyc03g007360.2.1	0	0	0	0	0	0	0	0	0	0	0	0	0
Solyc03g007370.2.1	1	11	0	6	0	0	2	1	0	2	2	4	0
Solyc03g007380.1.1	0	0	0	0	0	0	0	0	0	0	0	0	0

Solyc03g007390.2.1	1	5	0	0	0	8	0	5	0	3	3	13	0
Solyc03g007410.2.1	0	0	0	0	0	0	0	0	0	0	0	0	0
Solyc03g007420.2.1	5	5	1	20	0	30	21	43	10	29	4	10	0
Solyc03g007430.2.1	8	21	2	18	0	78	27	47	5	32	16	31	0
Solyc03g007440.2.1	0	0	0	0	0	0	0	0	0	0	0	0	0
Solyc03g007460.1.1	0	0	0	0	0	0	0	0	0	0	0	0	0
Solyc03g007470.2.1	2	30	1	5	2	6	1	9	1	198	4	102	3
Solyc03g007480.2.1	2	13	3	20	0	31	33	39	3	63	14	27	3
Solyc03g007490.2.1	3	10	3	15	0	14	8	21	3	11	5	9	0
Solyc03g007500.2.1	0	0	0	0	0	0	0	0	0	0	0	0	0
Solyc03g007510.2.1	2	4	0	0	4	1	9	7	0	3	0	3	0
Solyc03g007520.2.1	3	66	0	34	1	57	15	78	2	82	14	57	1
Solyc03g007530.2.1	14	67	10	60	3	184	66	176	3	141	25	121	0
Solyc03g007540.2.1	2	14	2	9	0	39	22	36	0	6	1	13	0
Solyc03g007550.2.1	0	0	0	0	0	0	0	0	0	0	0	0	0
Solyc03g007560.2.1	0	0	0	0	0	0	0	0	0	0	0	0	0
Solyc03g007590.2.1	3	44	6	18	2	37	16	38	0	46	2	55	2
Solyc03g007600.2.1	12	153	15	81	8	198	257	216	7	159	32	114	0
Solyc03g007610.2.1	15	131	19	81	6	237	253	415	55	259	77	164	4
Solyc03g007620.2.1	5	34	5	42	0	36	62	95	5	52	15	30	0
Solyc03g007650.1.1	0	0	0	0	0	0	0	0	0	0	0	0	0
Solyc03g007660.2.1	4	154	13	137	6	686	192	246	18	676	71	350	8
Solyc03g007670.2.1	11	384	47	158	19	531	251	467	30	409	81	489	13
Solyc03g007680.2.1	1	0	1	0	0	3	6	1	1	3	2	11	0
Solyc03g007690.1.1	0	0	0	0	0	0	0	0	0	0	0	0	0
Solyc03g007700.1.1	0	0	0	0	0	0	0	0	0	0	0	0	0
Solyc03g007710.2.1	0	0	0	0	0	0	0	0	0	0	0	0	0
Solyc03g007720.2.1	0	0	0	0	0	0	0	0	0	0	0	0	0
Solyc03g007740.2.1	3	122	12	102	8	227	58	233	22	444	69	340	7
Solyc03g007750.2.1	3	16	0	3	6	12	18	24	2	14	10	13	0
Solyc03g007760.2.1	3	32	2	22	0	49	10	51	1	36	1	23	0
Solyc03g007770.2.1	12	538	31	160	21	972	68	156	16	218	118	268	5
Solyc03g007790.2.1	4	1	0	0	0	116	53	17	51	0	3	0	0
Solyc03g007800.2.1	0	0	0	0	0	0	0	0	0	0	0	0	0
Solyc03g007810.2.1	11	45	9	57	24	288	438	448	35	479	177	281	5
Solyc03g007820.2.1	0	0	0	0	0	0	0	0	0	0	0	0	0
Solyc03g007830.2.1	0	0	0	0	0	0	0	0	0	0	0	0	0
Solyc03g007840.1.1	0	0	0	0	0	0	0	0	0	0	0	0	0
Solyc03g007850.1.1	0	0	0	0	0	0	0	0	0	0	0	0	0
Solyc03g007860.1.1	4	59	5	13	4	4	21	2	0	21	1	16	3
Solyc03g007870.2.1	4	197	31	218	5	49	4	73	1	56	10	57	1

Solyc03g007880.1.1	0	0	0	0	0	0	0	0	0	0	0	0	0
Solyc03g007890.2.1	9	11	0	5	3	9	6	7	2	43	92	142	0
Solyc03g007900.2.1	3	35	0	23	0	220	13	96	1	73	11	51	6
Solyc03g007920.2.1	5	48	3	21	0	66	33	87	0	45	7	26	1
Solyc03g007930.2.1	0	0	0	0	0	0	0	0	0	0	0	0	0
Solyc03g007940.2.1	0	0	0	0	0	0	0	0	0	0	0	0	0
Solyc03g007950.2.1	0	0	0	0	0	0	0	0	0	0	0	0	0
Solyc03g007960.2.1	4	9	1	1	1	161	54	52	0	22	1	14	1
Solyc03g007970.1.1	0	0	0	0	0	0	0	0	0	0	0	0	0
Solyc03g007980.1.1	3	24	3	15	0	19	19	41	0	39	3	26	3
Solyc03g007990.1.1	0	0	0	0	0	0	0	0	0	0	0	0	0
Solyc03g008000.1.1	4	99	4	56	0	185	84	227	16	347	123	275	4
Solyc03g008010.2.1	2	76	18	43	3	47	37	45	7	192	11	77	5
Solyc03g013030.2.1	4	65	1	84	3	1558	65	468	5	257	35	59	1
Solyc03g013240.2.1	10	183	6	106	1	20	48	39	1	101	14	120	1
Solyc03g013250.2.1	0	0	0	0	0	0	0	0	0	0	0	0	0
Solyc03g013340.2.1	0	0	0	0	0	0	0	0	0	0	0	0	0
Solyc03g013430.1.1	0	0	0	0	0	0	0	0	0	0	0	0	0
Solyc03g013440.2.1	8	5	0	7	0	342	56	331	0	2	0	0	0
Solyc03g013460.1.1	0	0	0	0	0	0	0	0	0	0	0	0	0
Solyc03g013630.2.1	1	3	0	2	0	14	5	14	2	7	0	9	0
Solyc03g019650.2.1	10	104	39	45	0	420	546	427	59	543	135	419	1
Solyc03g019660.2.1	2	25	3	6	0	16	9	11	0	28	1	33	1
Solyc03g019670.2.1	9	92	5	29	13	68	90	65	6	182	50	111	26
Solyc03g019680.2.1	4	36	4	29	3	72	61	68	4	23	18	9	0
Solyc03g019690.1.1	0	0	0	0	0	0	0	0	0	0	0	0	0
Solyc03g019700.1.1	1	3	0	3	0	1	2	6	1	30	2	15	0
Solyc03g019710.2.1	0	0	0	0	0	0	0	0	0	0	0	0	0
Solyc03g019720.2.1	10	121	2	63	7	174	45	163	9	348	40	167	2
Solyc03g019730.2.1	4	38	5	23	9	83	34	72	11	88	13	100	0
Solyc03g019750.2.1	0	0	0	0	0	0	0	0	0	0	0	0	0
Solyc03g019760.2.1	24	176	20	12	56	448	444	100	858	231	20	31	21
Solyc03g019770.2.1	2	2	1	0	0	5	10	6	5	0	1	0	0
Solyc03g019780.2.1	3	87	3	39	4	120	34	163	15	1120	42	768	4
Solyc03g019790.2.1	4	92	4	76	2	5	14	13	0	222	36	230	1
Solyc03g019800.2.1	3	8	1	1	5	28	5	17	2	16	10	18	0
Solyc03g019810.2.1	1	6	1	0	0	2	6	8	2	8	0	1	1
Solyc03g019820.2.1	8	19	0	15	0	459	126	401	0	13	107	5	0
Solyc03g019830.2.1	0	0	0	0	0	0	0	0	0	0	0	0	0
Solyc03g019840.2.1	0	0	0	0	0	0	0	0	0	0	0	0	0
Solyc03g019850.2.1	8	66	5	39	0	102	66	143	14	151	4	82	0

Solyc03g019860.2.1	1	30	0	12	0	34	13	31	2	33	6	23	0
Solyc03g019870.2.1	0	0	0	0	0	0	0	0	0	0	0	0	0
Solyc03g019880.2.1	6	148	35	115	4	207	174	167	4	288	63	205	5
Solyc03g019890.2.1	5	40	1	41	1	34	23	56	1	5	4	5	0
Solyc03g019900.2.1	4	37	5	27	4	60	56	103	15	64	23	47	1
Solyc03g019910.2.1	5	10	0	4	0	30	11	27	2	39	13	27	0
Solyc03g019920.1.1	0	0	0	0	0	0	0	0	0	0	0	0	0
Solyc03g019930.2.1	9	22	1	22	0	37	71	72	6	44	12	29	0
Solyc03g019940.2.1	9	63	8	73	0	73	62	147	11	72	12	74	0
Solyc03g019950.2.1	10	112	17	73	7	1265	292	630	110	145	61	103	2
Solyc03g019960.2.1	0	0	0	0	0	0	0	0	0	0	0	0	0
Solyc03g019990.1.1	0	0	0	0	0	0	0	0	0	0	0	0	0
Solyc03g020000.2.1	0	0	0	0	0	0	0	0	0	0	0	0	0
Solyc03g020010.1.1	0	0	0	0	0	0	0	0	0	0	0	0	0
Solyc03g020020.2.1	19	43	17	60	0	101	144	174	27	127	36	88	4
Solyc03g020040.2.1	0	0	0	0	0	0	0	0	0	0	0	0	0
Solyc03g020050.2.1	0	0	0	0	0	0	0	0	0	0	0	0	0
Solyc03g020060.2.1	2	0	0	0	0	0	18	0	0	42	2	283	6
Solyc03g025110.1.1	0	0	0	0	0	0	0	0	0	0	0	0	0
Solyc03g025130.1.1	2	0	0	7	0	9	6	3	0	4	1	5	0
Solyc03g025150.2.1	24	189	16	111	7	437	119	371	38	253	73	256	4
Solyc03g025160.2.1	6	46	4	32	5	87	46	88	21	67	30	37	4
Solyc03g025170.1.1	14	418	21	358	16	408	122	377	9	587	51	510	5
Solyc03g025190.2.1	0	0	0	0	0	0	0	0	0	0	0	0	0
Solyc03g025200.2.1	1	5	0	1	0	9	13	3	0	12	1	25	0
Solyc03g025220.2.1	0	0	0	0	0	0	0	0	0	0	0	0	0
Solyc03g025230.2.1	3	70	44	31	0	15	103	24	4	241	30	155	0
Solyc03g025260.1.1	0	0	0	0	0	0	0	0	0	0	0	0	0
Solyc03g025270.2.1	9	504	22	304	29	1330	267	1538	96	807	205	900	16
Solyc03g025280.2.1	13	31	8	25	10	123	92	123	13	86	15	92	4
Solyc03g025290.2.1	12	74	19	63	2	132	92	120	20	52	15	66	3
Solyc03g025310.2.1	9	110	22	79	0	250	88	226	9	93	10	116	1
Solyc03g025320.2.1	19	713	19	50	93	388	214	223	513	1635	57	190	88
Solyc03g025330.2.1	1	3	0	1	0	11	14	14	1	2	1	1	0
Solyc03g025340.1.1	13	796	76	307	33	582	788	596	161	1223	185	664	18
Solyc03g025350.2.1	0	0	0	0	0	0	0	0	0	0	0	0	0
Solyc03g025360.2.1	12	42	8	32	0	95	54	140	13	75	21	85	1
Solyc03g025370.2.1	1	1	2	2	0	2	18	2	7	4	0	0	1
Solyc03g025380.2.1	5	1	0	0	3	23	18	45	28	5	0	4	0
Solyc03g025390.2.1	5	417	19	97	0	18	13	38	0	130	28	106	0
Solyc03g025400.2.1	2	16	0	6	0	23	7	4	0	31	4	42	1

Solyc03g025410.2.1	13	385	61	275	0	260	342	449	14	47	4	108	1
Solyc03g025420.2.1	4	22	3	9	3	29	12	27	2	18	5	14	0
Solyc03g025430.1.1	2	2	0	4	2	8	12	20	2	3	1	12	0
Solyc03g025440.2.1	9	29	4	36	5	63	63	80	5	92	24	69	4
Solyc03g025450.2.1	13	84	6	79	1	133	63	140	8	62	28	54	4
Solyc03g025460.2.1	0	0	0	0	0	0	0	0	0	0	0	0	0
Solyc03g025470.2.1	0	0	0	0	0	0	0	0	0	0	0	0	0
Solyc03g025480.2.1	0	0	0	0	0	0	0	0	0	0	0	0	0
Solyc03g025490.2.1	0	0	0	0	0	0	0	0	0	0	0	0	0
Solyc03g025500.2.1	1	15	0	0	0	17	4	17	0	24	1	13	0
Solyc03g025510.2.1	3	32	4	4	4	28	53	60	2	13	8	28	0
Solyc03g025520.2.1	4	303	15	170	13	764	139	552	43	753	81	466	3
Solyc03g025530.2.1	19	85	8	63	10	160	119	235	41	222	76	197	4
Solyc03g025540.2.1	7	27	4	29	2	47	49	75	7	72	24	55	3
Solyc03g025550.2.1	13	252	23	149	19	362	228	578	59	732	103	420	16
Solyc03g025560.2.1	2	10	0	2	0	15	5	10	0	6	6	22	0
Solyc03g025580.1.1	0	0	0	0	0	0	0	0	0	0	0	0	0
Solyc03g025590.2.1	0	0	0	0	0	0	0	0	0	0	0	0	0
Solyc03g025600.2.1	6	34	4	27	11	91	90	227	5	186	14	72	1
Solyc03g025610.1.1	3	16	2	7	0	26	70	52	5	66	18	76	0
Solyc03g025620.2.1	0	0	0	0	0	0	0	0	0	0	0	0	0
Solyc03g025630.2.1	4	40	3	58	7	93	138	108	7	212	49	202	3
Solyc03g025660.1.1	0	0	0	0	0	0	0	0	0	0	0	0	0
Solyc03g025670.2.1	3	117	5	81	1	324	11	327	24	4	4	1	0
Solyc03g025680.2.1	1	8	7	0	0	18	20	1	29	0	0	0	0
Solyc03g025690.2.1	0	0	0	0	0	0	0	0	0	0	0	0	0
Solyc03g025700.1.1	0	0	0	0	0	0	0	0	0	0	0	0	0
Solyc03g025710.2.1	5	141	17	65	0	7	2	0	0	1	2	0	0
Solyc03g025720.2.1	5	115	5	127	0	96	17	79	4	52	14	217	1
Solyc03g025730.2.1	5	73	0	18	0	17	13	26	1	47	6	54	0
Solyc03g025740.2.1	0	0	0	0	0	0	0	0	0	0	0	0	0
Solyc03g025750.2.1	0	0	0	0	0	0	0	0	0	0	0	0	0
Solyc03g025760.2.1	0	0	0	0	0	0	0	0	0	0	0	0	0
Solyc03g025790.2.1	0	0	0	0	0	0	0	0	0	0	0	0	0
Solyc03g025800.2.1	1	38	7	9	0	38	11	32	1	115	9	54	0
Solyc03g025810.2.1	38	5	1	3	7	4532	1267	2638	61	16	23	8	0
Solyc03g025820.1.1	0	0	0	0	0	0	0	0	0	0	0	0	0
Solyc03g025830.2.1	3	15	0	4	1	250	27	121	2	4	13	0	0
Solyc03g025840.2.1	8	151	20	117	3	76	108	116	19	219	78	157	12
Solyc03g025850.2.1	3	137	6	56	10	64	35	155	57	391	36	163	3
Solyc03g025860.2.1	0	0	0	0	0	0	0	0	0	0	0	0	0

Solyc03g025870.2.1	0	0	0	0	0	0	0	0	0	0	0	0	0
Solyc03g025880.2.1	3	22	3	7	0	46	25	48	2	110	17	65	0
Solyc03g025890.1.1	2	4	0	0	1	4	17	12	0	12	4	15	0
Solyc03g025900.2.1	0	0	0	0	0	0	0	0	0	0	0	0	0
Solyc03g025910.2.1	0	0	0	0	0	0	0	0	0	0	0	0	0
Solyc03g025920.2.1	2	3	1	0	0	1	17	5	3	1	13	2	0
Solyc03g025930.2.1	1	7	2	1	1	2	20	9	4	17	9	4	0
Solyc03g025940.1.1	0	0	0	0	0	0	0	0	0	0	0	0	0
Solyc03g025950.2.1	4	153	6	69	20	387	543	327	106	355	272	323	14
Solyc03g025960.1.1	0	0	0	0	0	0	0	0	0	0	0	0	0
Solyc03g025970.2.1	6	17	4	8	4	53	15	39	2	7	6	13	0
Solyc03g025980.2.1	8	39	11	26	0	63	64	77	10	45	21	38	0
Solyc03g025990.1.1	7	57	8	25	0	26	20	20	11	50	14	25	1
Solyc03g026000.2.1	0	0	0	0	0	0	0	0	0	0	0	0	0
Solyc03g026010.1.1	1	5	0	0	0	0	6	3	0	10	0	3	0
Solyc03g026020.2.1	9	84	7	93	13	81	79	161	25	232	35	177	6
Solyc03g026040.2.1	0	0	0	0	0	0	0	0	0	0	0	0	0
Solyc03g026050.2.1	0	0	0	0	0	0	0	0	0	0	0	0	0
Solyc03g026060.2.1	6	27	4	12	7	91	32	54	12	45	7	36	0
Solyc03g026070.2.1	5	4	0	1	5	3	30	0	13	10	10	1	0
Solyc03g026080.2.1	0	0	0	0	0	0	0	0	0	0	0	0	0
Solyc03g026100.1.1	2	4	0	1	0	13	8	10	0	15	0	9	0
Solyc03g026110.2.1	9	111	18	85	27	18	6	41	5	103	47	95	7
Solyc03g026120.2.1	1	4	2	1	2	0	7	0	0	2	6	0	3
Solyc03g026130.2.1	6	8	4	14	1	15	77	24	5	9	4	15	0
Solyc03g026140.2.1	0	0	0	0	0	0	0	0	0	0	0	0	0
Solyc03g026150.2.1	2	22	1	1	0	4	7	4	2	26	0	9	0
Solyc03g026160.2.1	0	0	0	0	0	0	0	0	0	0	0	0	0
Solyc03g026170.2.1	7	18	2	20	0	99	54	102	3	90	3	40	0
Solyc03g026180.1.1	1	5	0	0	3	3	5	9	2	18	5	3	0
Solyc03g026190.2.1	11	50	9	57	1	172	99	153	35	103	37	78	3
Solyc03g026200.2.1	1	5	0	1	0	8	3	3	0	7	1	0	0
Solyc03g026210.2.1	1	14	6	6	2	0	13	7	0	10	0	20	0
Solyc03g026220.2.1	12	679	35	269	30	452	21	137	25	566	21	477	4
Solyc03g026230.1.1	0	0	0	0	0	0	0	0	0	0	0	0	0
Solyc03g026240.2.1	7	52	3	49	2	258	32	85	4	170	24	74	0
Solyc03g026260.2.1	1	10	1	4	0	1	5	7	0	1	0	4	0
Solyc03g026270.1.1	0	0	0	0	0	0	0	0	0	0	0	0	0
Solyc03g026280.2.1	5	183	26	117	9	0	0	0	0	2	0	16	1
Solyc03g026290.2.1	0	0	0	0	0	0	0	0	0	0	0	0	0
Solyc03g026300.2.1	2	10	1	14	1	7	17	8	2	6	1	0	0

Solyc03g026310.2.1	6	58	13	61	8	98	102	75	5	24	17	17	0
Solyc03g026320.2.1	0	0	0	0	0	0	0	0	0	0	0	0	0
Solyc03g026330.2.1	0	0	0	0	0	0	0	0	0	0	0	0	0
Solyc03g026340.2.1	11	143	35	91	39	144	217	212	19	204	82	111	12
Solyc03g026350.2.1	10	61	4	50	8	129	46	209	12	132	40	56	2
Solyc03g026370.1.1	1	10	0	4	0	146	6	48	1	3	5	1	0
Solyc03g031420.1.1	17	73	18	51	14	25	66	41	12	187	29	308	2
Solyc03g031430.1.1	0	0	0	0	0	0	0	0	0	0	0	0	0
Solyc03g031440.2.1	0	0	0	0	0	0	0	0	0	0	0	0	0
Solyc03g031460.1.1	4	16	0	0	0	24	24	20	1	20	7	37	1
Solyc03g031470.2.1	0	0	0	0	0	0	0	0	0	0	0	0	0
Solyc03g031530.2.1	0	0	0	0	0	0	0	0	0	0	0	0	0
Solyc03g031560.2.1	0	0	0	0	0	0	0	0	0	0	0	0	0
Solyc03g031570.2.1	0	0	0	0	0	0	0	0	0	0	0	0	0
Solyc03g031600.2.1	0	0	0	0	0	0	0	0	0	0	0	0	0
Solyc03g031620.2.1	6	59	0	22	0	30	6	5	0	77	38	24	0
Solyc03g031650.2.1	1	9	3	2	0	3	4	13	5	24	13	18	0
Solyc03g031670.2.1	11	60	2	50	1	295	53	284	9	97	17	70	0
Solyc03g031680.2.1	1	10	1	2	0	2	0	4	0	13	4	3	0
Solyc03g031690.2.1	4	48	2	31	0	70	76	82	8	126	24	90	1
Solyc03g031700.2.1	8	202	45	133	1	228	309	268	18	153	47	109	2
Solyc03g031720.2.1	5	247	20	120	18	294	126	342	31	1081	190	929	5
Solyc03g031730.2.1	1	20	4	18	0	0	0	1	0	4	0	0	0
Solyc03g031750.1.1	5	47	5	31	6	134	9	44	8	32	27	46	0
Solyc03g031760.2.1	3	41	3	4	8	5	17	2	4	7	8	0	13
Solyc03g031780.2.1	0	0	0	0	0	0	0	0	0	0	0	0	0
Solyc03g031790.1.1	10	139	11	66	2	167	79	209	49	255	29	132	0
Solyc03g031800.2.1	1	12	0	5	0	0	3	3	0	22	4	16	0
Solyc03g031820.2.1	1	9	0	0	0	3	5	7	0	11	0	7	0
Solyc03g031830.1.1	0	0	0	0	0	0	0	0	0	0	0	0	0
Solyc03g031840.2.1	1	31	1	26	1	2	3	13	0	99	6	107	1
Solyc03g031850.1.1	2	2	2	5	0	8	7	8	3	15	0	2	0
Solyc03g031860.2.1	9	283	27	147	6	1330	54	2423	2	603	43	428	0
Solyc03g031880.2.1	0	0	0	0	0	0	0	0	0	0	0	0	0
Solyc03g031890.2.1	3	6	0	11	1	17	29	35	3	14	3	12	0
Solyc03g031910.2.1	1	0	0	1	3	0	3	1	21	0	1	0	9
Solyc03g031920.2.1	20	459	23	468	24	515	256	887	102	151	17	88	5
Solyc03g031940.2.1	3	17	0	2	39	0	36	3	168	2	6	0	0
Solyc03g031950.1.1	3	61	4	26	0	75	49	129	21	159	24	66	1
Solyc03g031970.2.1	20	273	36	140	7	281	186	293	27	772	39	383	8
Solyc03g031980.2.1	3	6	8	5	0	9	5	10	0	3	1	0	0

Solyc03g031990.2.1	5	185	1	139	0	13	12	28	0	26	0	9	0
Solyc03g032000.2.1	8	78	11	35	5	58	64	63	12	128	50	92	5
Solyc03g032010.2.1	2	13	4	0	3	29	23	17	3	17	2	7	0
Solyc03g032020.2.1	4	19	5	21	0	206	13	227	2	42	31	20	1
Solyc03g032030.2.1	0	0	0	0	0	0	0	0	0	0	0	0	0
Solyc03g032040.2.1	3	7	0	2	3	4	10	17	0	131	2	64	0
Solyc03g032050.2.1	10	56	3	45	4	154	92	157	19	110	11	62	1
Solyc03g032060.1.1	0	0	0	0	0	0	0	0	0	0	0	0	0
Solyc03g032070.2.1	4	34	0	23	0	116	18	112	5	62	13	70	4
Solyc03g032080.2.1	0	0	0	0	0	0	0	0	0	0	0	0	0
Solyc03g032090.1.1	7	59	1	30	3	503	89	303	11	85	60	35	0
Solyc03g032100.1.1	0	0	0	0	0	0	0	0	0	0	0	0	0
Solyc03g032120.2.1	1	2	0	0	0	0	11	2	0	2	0	8	5
Solyc03g032130.2.1	6	260	102	239	38	156	701	278	94	872	406	496	64
Solyc03g032140.2.1	7	16	2	1	1	32	22	40	9	63	10	31	0
Solyc03g032150.2.1	8	59	1	33	0	149	41	131	12	59	10	79	0
Solyc03g032160.2.1	15	276	18	208	14	514	199	740	50	466	63	418	1
Solyc03g032170.1.1	0	0	0	0	0	0	0	0	0	0	0	0	0
Solyc03g032180.2.1	7	13	2	14	0	67	40	59	1	25	10	36	1
Solyc03g032190.2.1	0	0	0	0	0	0	0	0	0	0	0	0	0
Solyc03g032210.2.1	2	6	0	0	0	2	26	12	0	5	4	2	0
Solyc03g032220.2.1	0	0	0	0	0	0	0	0	0	0	0	0	0
Solyc03g032240.2.1	0	0	0	0	0	0	0	0	0	0	0	0	0
Solyc03g033250.1.1	0	0	0	0	0	0	0	0	0	0	0	0	0
Solyc03g033260.2.1	0	0	0	0	0	0	0	0	0	0	0	0	0
Solyc03g033280.1.1	0	0	0	0	0	0	0	0	0	0	0	0	0
Solyc03g033290.1.1	0	0	0	0	0	0	0	0	0	0	0	0	0
Solyc03g033310.2.1	2	12	1	9	0	14	6	6	0	12	4	5	0
Solyc03g033320.2.1	4	16	0	13	0	31	16	44	2	48	16	71	3
Solyc03g033330.2.1	3	20	3	3	4	10	4	3	4	33	21	9	1
Solyc03g033340.2.1	3	12	0	18	4	31	8	22	0	49	11	29	0
Solyc03g033350.2.1	0	0	0	0	0	0	0	0	0	0	0	0	0
Solyc03g033360.1.1	0	0	0	0	0	0	0	0	0	0	0	0	0
Solyc03g033370.2.1	0	0	0	0	0	0	0	0	0	0	0	0	0
Solyc03g033380.2.1	5	37	1	10	2	25	31	38	9	42	8	32	4
Solyc03g033390.2.1	0	0	0	0	0	0	0	0	0	0	0	0	0
Solyc03g033400.2.1	6	21	7	21	0	103	39	110	5	27	9	41	7
Solyc03g033410.2.1	4	59	5	26	7	54	17	67	16	8	2	12	13
Solyc03g033420.2.1	1	27	1	20	3	18	19	28	2	50	18	27	0
Solyc03g033440.1.1	0	0	0	0	0	0	0	0	0	0	0	0	0
Solyc03g033450.2.1	2	1	1	0	0	6	4	9	1	5	0	9	0

Solyc03g033460.2.1	0	0	0	0	0	0	0	0	0	0	0	0	0
Solyc03g033470.2.1	0	0	0	0	0	0	0	0	0	0	0	0	0
Solyc03g033480.2.1	0	0	0	0	0	0	0	0	0	0	0	0	0
Solyc03g033490.1.1	1	22	0	18	0	21	2	21	0	18	2	11	0
Solyc03g033500.2.1	5	95	10	52	7	192	52	235	19	117	40	66	1
Solyc03g033510.2.1	0	0	0	0	0	0	0	0	0	0	0	0	0
Solyc03g033520.2.1	1	1	0	0	0	0	6	4	0	6	1	1	0
Solyc03g033530.2.1	0	0	0	0	0	0	0	0	0	0	0	0	0
Solyc03g033540.2.1	13	218	22	134	19	90	105	110	21	149	28	123	5
Solyc03g033550.2.1	0	0	0	0	0	0	0	0	0	0	0	0	0
Solyc03g033560.2.1	4	37	5	9	2	28	29	65	10	85	11	62	1
Solyc03g033580.2.1	5	30	4	11	0	72	28	49	8	62	15	55	4
Solyc03g033590.1.1	0	0	0	0	0	0	0	0	0	0	0	0	0
Solyc03g033600.2.1	2	15	3	10	0	11	14	16	2	12	5	4	0
Solyc03g033610.1.1	0	0	0	0	0	0	0	0	0	0	0	0	0
Solyc03g033620.2.1	12	80	7	44	3	162	138	266	18	155	51	158	5
Solyc03g033630.2.1	7	26	2	11	3	49	82	82	21	57	9	53	0
Solyc03g033640.2.1	3	12	0	2	0	49	16	42	9	88	1	23	1
Solyc03g033650.2.1	0	0	0	0	0	0	0	0	0	0	0	0	0
Solyc03g033660.2.1	0	0	0	0	0	0	0	0	0	0	0	0	0
Solyc03g033690.1.1	0	0	0	0	0	0	0	0	0	0	0	0	0
Solyc03g033720.2.1	1	6	2	0	0	0	3	4	0	2	0	0	0
Solyc03g033730.1.1	0	0	0	0	0	0	0	0	0	0	0	0	0
Solyc03g033750.1.1	0	0	0	0	0	0	0	0	0	0	0	0	0
Solyc03g033790.2.1	0	0	0	0	0	0	0	0	0	0	0	0	0
Solyc03g033820.1.1	0	0	0	0	0	0	0	0	0	0	0	0	0
Solyc03g033830.2.1	0	0	0	0	0	0	0	0	0	0	0	0	0
Solyc03g033840.2.1	2	11	1	29	0	12	0	6	0	3	1	0	0
Solyc03g033850.2.1	0	0	0	0	0	0	0	0	0	0	0	0	0
Solyc03g033860.1.1	0	0	0	0	0	0	0	0	0	0	0	0	0
Solyc03g033950.2.1	13	101	11	131	5	131	64	235	9	316	28	262	1
Solyc03g033960.1.1	0	0	0	0	0	0	0	0	0	0	0	0	0
Solyc03g033980.2.1	8	97	0	33	5	148	81	128	18	98	6	67	5
Solyc03g033990.1.1	1	0	2	0	0	0	1	0	7	4	3	0	0
Solyc03g034000.2.1	0	0	0	0	0	0	0	0	0	0	0	0	0
Solyc03g034010.2.1	0	0	0	0	0	0	0	0	0	0	0	0	0
Solyc03g034020.2.1	2	10	1	15	2	1	6	1	0	3	15	2	0
Solyc03g034030.2.1	0	0	0	0	0	0	0	0	0	0	0	0	0
Solyc03g034050.2.1	1	3	0	0	0	7	5	14	0	2	1	3	1
Solyc03g034060.2.1	0	0	0	0	0	0	0	0	0	0	0	0	0
Solyc03g034070.1.1	4	179	33	70	0	20	158	23	2	9	19	14	0

Solyc03g034080.2.1	9	215	24	141	5	337	165	374	72	243	58	151	0
Solyc03g034090.2.1	0	0	0	0	0	0	0	0	0	0	0	0	0
Solyc03g034100.2.1	0	0	0	0	0	0	0	0	0	0	0	0	0
Solyc03g034120.2.1	0	0	0	0	0	0	0	0	0	0	0	0	0
Solyc03g034140.2.1	0	0	0	0	0	0	0	0	0	0	0	0	0
Solyc03g034160.2.1	0	0	0	0	0	0	0	0	0	0	0	0	0
Solyc03g034170.2.1	5	195	17	83	0	20	33	37	2	32	3	50	2
Solyc03g034180.2.1	5	38	5	5	0	55	6	64	12	45	7	82	0
Solyc03g034190.2.1	3	25	2	7	0	8	23	9	2	22	1	15	0
Solyc03g034200.2.1	0	0	0	0	0	0	0	0	0	0	0	0	0
Solyc03g034220.2.1	1	3647	80	1828	5	317	76	341	2	235	63	552	2
Solyc03g034230.2.1	0	0	0	0	0	0	0	0	0	0	0	0	0
Solyc03g034240.2.1	1	0	0	4	0	10	4	6	0	0	0	0	0
Solyc03g034250.2.1	4	8	0	13	0	16	5	18	5	13	7	4	0
Solyc03g034270.2.1	0	0	0	0	0	0	0	0	0	0	0	0	0
Solyc03g034300.1.1	0	0	0	0	0	0	0	0	0	0	0	0	0
Solyc03g034320.2.1	0	0	0	0	0	0	0	0	0	0	0	0	0
Solyc03g034330.1.1	0	0	0	0	0	0	0	0	0	0	0	0	0
Solyc03g034370.1.1	3	42	1	2	2	106	25	109	4	37	4	53	3
Solyc03g034400.2.1	0	0	0	0	0	0	0	0	0	0	0	0	0
Solyc03g034430.1.1	6	129	3	107	0	155	76	466	32	154	44	156	0
Solyc03g034440.2.1	9	107	9	95	1	234	82	218	6	336	42	286	1
Solyc03g034450.2.1	9	25	9	22	7	64	83	81	9	66	26	68	2
Solyc03g034460.2.1	0	0	0	0	0	0	0	0	0	0	0	0	0
Solyc03g036470.1.1	0	0	0	0	0	0	0	0	0	0	0	0	0
Solyc03g036480.1.1	0	0	0	0	0	0	0	0	0	0	0	0	0
Solyc03g042560.1.1	0	0	0	0	0	0	0	0	0	0	0	0	0
Solyc03g043600.1.1	7	24	12	29	3	36	162	62	23	156	16	81	6
Solyc03g043640.2.1	0	0	0	0	0	0	0	0	0	0	0	0	0
Solyc03g043660.2.1	13	42	5	45	6	150	56	195	7	102	30	108	7
Solyc03g043700.2.1	0	0	0	0	0	0	0	0	0	0	0	0	0
Solyc03g043720.2.1	1	22	0	8	0	4	9	16	1	56	0	22	0
Solyc03g043740.2.1	1	93	3	40	0	27	0	25	0	36	11	25	0
Solyc03g043750.2.1	13	107	8	41	4	74	52	128	9	314	90	326	2
Solyc03g043760.2.1	13	95	7	50	2	104	80	195	20	152	30	83	0
Solyc03g043770.2.1	2	9	0	19	0	2	8	31	0	25	9	5	0
Solyc03g043850.2.1	1	1	0	0	0	0	6	2	0	17	0	14	0
Solyc03g043860.2.1	0	0	0	0	0	0	0	0	0	0	0	0	0
Solyc03g043870.2.1	1	7	0	0	0	1	3	8	0	3	0	3	0
Solyc03g043880.2.1	2	0	0	0	0	13	53	16	0	0	0	0	0
Solyc03g043900.1.1	1	2	0	0	0	17	1	56	0	63	4	19	0

Solyc03g043910.2.1	0	0	0	0	0	0	0	0	0	0	0	0	0
Solyc03g043920.2.1	0	0	0	0	0	0	0	0	0	0	0	0	0
Solyc03g043930.2.1	3	15	1	5	0	61	31	49	4	28	4	16	1
Solyc03g043950.2.1	5	92	5	63	19	59	7	58	9	23	6	46	13
Solyc03g043960.2.1	2	85	3	46	2	53	54	80	11	150	9	97	2
Solyc03g044000.1.1	1	20	5	12	8	34	22	18	4	94	26	61	0
Solyc03g044010.2.1	3	33	12	26	0	249	271	103	16	71	98	104	0
Solyc03g044020.2.1	0	0	0	0	0	0	0	0	0	0	0	0	0
Solyc03g044030.1.1	0	0	0	0	0	0	0	0	0	0	0	0	0
Solyc03g044060.2.1	12	56	7	18	4	60	70	84	8	104	26	124	0
Solyc03g044080.2.1	0	0	0	0	0	0	0	0	0	0	0	0	0
Solyc03g044090.2.1	1	5	0	5	0	0	7	9	0	0	2	0	0
Solyc03g044100.2.1	0	0	0	0	0	0	0	0	0	0	0	0	0
Solyc03g044120.1.1	0	0	0	0	0	0	0	0	0	0	0	0	0
Solyc03g044140.2.1	5	78	7	47	5	72	43	161	8	157	15	105	0
Solyc03g044150.2.1	16	347	14	169	15	177	329	295	21	909	133	702	6
Solyc03g044160.1.1	5	29	6	9	0	7	32	17	7	27	9	10	2
Solyc03g044190.2.1	4	29	0	10	0	38	15	63	7	191	3	242	1
Solyc03g044200.2.1	21	78	1	66	3	151	3	194	10	276	443	223	0
Solyc03g044220.1.1	5	22	1	11	1	17	16	16	6	35	14	20	0
Solyc03g044260.2.1	2	53	2	32	9	83	36	140	10	78	11	50	0
Solyc03g044270.2.1	5	41	11	27	8	83	286	173	56	73	60	62	0
Solyc03g044300.2.1	1	1	0	0	0	1	0	0	0	5	7	6	0
Solyc03g044330.1.1	13	458	43	228	60	142	390	336	111	250	215	590	2
Solyc03g044350.1.1	0	0	0	0	0	0	0	0	0	0	0	0	0
Solyc03g044370.2.1	1	2	1	1	0	6	2	2	1	7	0	0	0
Solyc03g044380.2.1	2	7	2	3	0	27	11	11	2	3	4	4	0
Solyc03g044420.1.1	0	0	0	0	0	0	0	0	0	0	0	0	0
Solyc03g044430.2.1	1	4	1	0	0	8	3	7	0	22	5	8	0
Solyc03g044460.1.1	0	0	0	0	0	0	0	0	0	0	0	0	0
Solyc03g044470.2.1	6	52	6	50	1	27	39	86	3	58	3	30	0
Solyc03g044480.2.1	0	0	0	0	0	0	0	0	0	0	0	0	0
Solyc03g044510.2.1	1	3	0	0	0	5	2	2	0	1	1	5	0
Solyc03g044560.1.1	0	0	0	0	0	0	0	0	0	0	0	0	0
Solyc03g044600.1.1	0	0	0	0	0	0	0	0	0	0	0	0	0
Solyc03g044620.2.1	22	47	5	64	13	100	111	153	30	113	43	109	1
Solyc03g044630.1.1	1	7	1	3	0	0	15	2	0	0	8	0	0
Solyc03g044660.2.1	2	4	0	0	0	15	14	25	2	17	0	27	0
Solyc03g044670.1.1	0	0	0	0	0	0	0	0	0	0	0	0	0
Solyc03g044710.2.1	0	0	0	0	0	0	0	0	0	0	0	0	0
Solyc03g044720.1.1	4	33	0	11	1	28	9	26	1	14	3	16	0

Solyc03g044740.2.1	0	0	0	0	0	0	0	0	0	0	0	0	0
Solyc03g044790.2.1	0	0	0	0	0	0	0	0	0	0	0	0	0
Solyc03g044800.1.1	0	0	0	0	0	0	0	0	0	0	0	0	0
Solyc03g044820.2.1	0	0	0	0	0	0	0	0	0	0	0	0	0
Solyc03g044840.1.1	0	0	0	0	0	0	0	0	0	0	0	0	0
Solyc03g044850.1.1	0	0	0	0	0	0	0	0	0	0	0	0	0
Solyc03g044880.2.1	1	0	0	0	0	1	3	7	0	37	4	36	0
Solyc03g044890.1.1	0	0	0	0	0	0	0	0	0	0	0	0	0
Solyc03g044900.2.1	2	18	3	10	0	38	14	99	0	2	0	2	0
Solyc03g044910.1.1	10	126	17	87	11	89	103	165	159	121	20	67	4
Solyc03g045000.1.1	0	0	0	0	0	0	0	0	0	0	0	0	0
Solyc03g045020.2.1	0	0	0	0	0	0	0	0	0	0	0	0	0
Solyc03g045050.2.1	28	236	29	159	28	360	221	481	55	259	92	180	3
Solyc03g045060.2.1	8	40	3	24	12	22	57	63	7	27	15	66	0
Solyc03g045070.1.1	6	198	44	130	0	0	9	2	0	14	4	9	0
Solyc03g045090.2.1	7	77	16	39	17	294	188	333	52	128	59	148	3
Solyc03g045110.2.1	1	2	0	4	0	7	0	8	0	6	5	3	0
Solyc03g045120.1.1	0	0	0	0	0	0	0	0	0	0	0	0	0
Solyc03g045140.2.1	1	4	4	14	1	0	0	7	0	0	0	0	0
Solyc03g046200.1.1	0	0	0	0	0	0	0	0	0	0	0	0	0
Solyc03g046250.1.1	0	0	0	0	0	0	0	0	0	0	0	0	0
Solyc03g046260.1.1	0	0	0	0	0	0	0	0	0	0	0	0	0
Solyc03g046270.2.1	0	0	0	0	0	0	0	0	0	0	0	0	0
Solyc03g046340.2.1	13	136	26	130	10	393	286	358	11	105	8	79	1
Solyc03g046350.2.1	1	9	0	1	0	11	0	11	0	17	9	13	0
Solyc03g046380.1.1	2	15	0	19	2	633	9	65	1	2	21	0	0
Solyc03g046410.2.1	0	0	0	0	0	0	0	0	0	0	0	0	0
Solyc03g046450.2.1	8	1	4	4	2	24	44	17	10	33	4	30	3
Solyc03g046470.2.1	5	20	5	4	1	79	16	49	0	64	7	47	7
Solyc03g046570.2.1	3	33	0	32	0	74	25	79	2	61	13	20	4
Solyc03g046580.1.1	0	0	0	0	0	0	0	0	0	0	0	0	0
Solyc03g046590.2.1	5	40	21	12	0	64	127	68	11	132	12	80	3
Solyc03g051770.2.1	1	3	1	2	0	11	12	22	2	8	6	4	0
Solyc03g051780.1.1	0	0	0	0	0	0	0	0	0	0	0	0	0
Solyc03g051790.2.1	5	28	6	16	7	43	44	33	11	89	49	85	4
Solyc03g051810.2.1	5	83	6	46	0	32	36	29	5	26	1	24	0
Solyc03g051900.2.1	10	21	1	8	1	82	33	82	12	68	33	83	3
Solyc03g051920.2.1	2	22	1	3	4	25	20	43	4	28	3	20	4
Solyc03g051930.2.1	0	0	0	0	0	0	0	0	0	0	0	0	0
Solyc03g051940.1.1	0	0	0	0	0	0	0	0	0	0	0	0	0
Solyc03g051950.2.1	2	2	0	0	0	6	4	7	3	8	0	6	0

Solyc03g051970.2.1	2	9	0	5	2	0	14	16	2	27	3	36	0
Solyc03g052980.2.1	10	202	21	92	13	247	179	465	51	203	91	149	5
Solyc03g053000.2.1	3	6	1	1	0	21	18	19	10	27	10	24	0
Solyc03g053010.2.1	11	117	14	74	3	482	113	390	14	150	32	178	0
Solyc03g053020.2.1	1	13	4	4	0	9	22	11	2	8	6	22	4
Solyc03g053040.1.1	0	0	0	0	0	0	0	0	0	0	0	0	0
Solyc03g053080.1.1	4	12	0	15	0	27	20	24	2	18	1	28	2
Solyc03g053100.2.1	4	22	1	13	9	22	23	28	3	35	6	32	0
Solyc03g053110.2.1	7	392	44	183	4	177	205	141	12	56	59	96	4
Solyc03g058160.2.1	0	0	0	0	0	0	0	0	0	0	0	0	0
Solyc03g058190.2.1	9	286	47	248	0	280	453	363	9	193	67	259	10
Solyc03g058310.1.1	0	0	0	0	0	0	0	0	0	0	0	0	0
Solyc03g058340.2.1	1	7	1	3	0	1	7	5	1	10	0	7	0
Solyc03g058350.2.1	12	74	2	36	1	83	57	135	10	98	15	56	3
Solyc03g058380.2.1	2	4	0	0	0	4	2	6	1	7	0	3	0
Solyc03g058390.1.1	3	20	0	12	1	59	11	48	2	45	4	30	1
Solyc03g058400.2.1	0	0	0	0	0	0	0	0	0	0	0	0	0
Solyc03g058430.1.1	2	25	2	5	1	0	3	7	0	0	2	0	0
Solyc03g058450.2.1	0	0	0	0	0	0	0	0	0	0	0	0	0
Solyc03g058470.1.1	1	3	1	2	0	21	6	10	1	5	1	2	1
Solyc03g058480.1.1	0	0	0	0	0	0	0	0	0	0	0	0	0
Solyc03g058490.1.1	0	0	0	0	0	0	0	0	0	0	0	0	0
Solyc03g058500.1.1	0	0	0	0	0	0	0	0	0	0	0	0	0
Solyc03g058860.2.1	8	113	14	63	1	43	63	83	3	62	11	78	0
Solyc03g058880.2.1	8	15	2	5	0	82	57	92	6	50	9	47	0
Solyc03g058890.2.1	0	0	0	0	0	0	0	0	0	0	0	0	0
Solyc03g058910.2.1	0	0	0	0	0	0	0	0	0	0	0	0	0
Solyc03g058920.2.1	10	162	11	52	35	328	219	357	48	153	46	136	6
Solyc03g058930.2.1	2	2	0	1	0	24	9	12	1	16	0	2	0
Solyc03g058940.2.1	0	0	0	0	0	0	0	0	0	0	0	0	0
Solyc03g058950.2.1	1	2	0	3	0	8	9	9	0	7	0	0	0
Solyc03g058970.2.1	17	133	27	89	12	846	795	745	21	347	192	338	6
Solyc03g058990.1.1	0	0	0	0	0	0	0	0	0	0	0	0	0
Solyc03g059010.2.1	4	43	5	21	1	130	25	114	6	114	14	63	0
Solyc03g059030.2.1	16	40	13	35	5	210	107	164	18	152	38	107	1
Solyc03g059070.2.1	7	38	6	16	0	79	53	129	2	79	36	87	4
Solyc03g059100.1.1	3	29	1	6	1	55	21	44	2	25	5	45	0
Solyc03g059150.2.1	0	0	0	0	0	0	0	0	0	0	0	0	0
Solyc03g059160.1.1	2	9	0	9	0	14	7	13	0	105	0	51	0
Solyc03g059170.2.1	0	0	0	0	0	0	0	0	0	0	0	0	0
Solyc03g059180.2.1	20	423	120	283	6	84	222	160	12	183	38	257	9

Solyc03g059190.2.1	0	0	0	0	0	0	0	0	0	0	0	0	0
Solyc03g059250.2.1	12	37	8	29	5	44	74	101	11	62	21	83	8
Solyc03g059260.2.1	14	576	72	382	15	782	219	620	20	288	117	281	1
Solyc03g059270.2.1	0	0	0	0	0	0	0	0	0	0	0	0	0
Solyc03g059310.2.1	3	50	4	63	0	142	97	168	3	72	6	60	0
Solyc03g059390.2.1	1	5	0	0	0	5	7	7	0	0	0	3	0
Solyc03g059400.1.1	1	2	0	0	0	1	4	2	0	2	0	6	0
Solyc03g059420.2.1	13	78	16	112	13	217	143	259	32	100	40	73	3
Solyc03g059490.1.1	8	60	6	31	0	70	33	70	14	68	22	63	0
Solyc03g060510.2.1	0	0	0	0	0	0	0	0	0	0	0	0	0
Solyc03g061540.1.1	0	0	0	0	0	0	0	0	0	0	0	0	0
Solyc03g061590.2.1	3	12	3	2	4	7	25	29	3	26	10	13	1
Solyc03g061600.2.1	4	14	1	19	0	27	27	104	8	19	4	28	0
Solyc03g061620.1.1	0	0	0	0	0	0	0	0	0	0	0	0	0
Solyc03g062650.2.1	19	32	7	37	12	110	115	166	14	106	49	63	0
Solyc03g062660.2.1	2	3	0	0	0	0	0	3	0	1	4	2	1
Solyc03g062680.2.1	3	18	1	8	4	38	11	50	4	40	8	28	2
Solyc03g062700.2.1	0	0	0	0	0	0	0	0	0	0	0	0	0
Solyc03g062710.2.1	1	42	4	36	0	0	2	0	0	0	0	0	0
Solyc03g062720.2.1	5	139	29	72	0	0	22	5	0	14	0	13	1
Solyc03g062730.2.1	0	0	0	0	0	0	0	0	0	0	0	0	0
Solyc03g062740.2.1	0	0	0	0	0	0	0	0	0	0	0	0	0
Solyc03g062750.1.1	0	0	0	0	0	0	0	0	0	0	0	0	0
Solyc03g062790.2.1	6	131	15	47	3	114	108	124	0	61	20	64	0
Solyc03g062820.1.1	0	0	0	0	0	0	0	0	0	0	0	0	0
Solyc03g062830.2.1	6	55	5	31	2	55	45	73	7	38	0	25	0
Solyc03g062890.2.1	2	67	2	16	0	65	19	55	3	107	10	81	0
Solyc03g062900.2.1	0	0	0	0	0	0	0	0	0	0	0	0	0
Solyc03g062910.2.1	1	1	0	1	1	3	7	2	0	2	2	8	0
Solyc03g062920.1.1	0	0	0	0	0	0	0	0	0	0	0	0	0
Solyc03g062930.1.1	0	0	0	0	0	0	0	0	0	0	0	0	0
Solyc03g062940.2.1	4	60	13	57	6	136	68	143	7	290	28	158	8
Solyc03g062950.1.1	0	0	0	0	0	0	0	0	0	0	0	0	0
Solyc03g063030.2.1	0	0	0	0	0	0	0	0	0	0	0	0	0
Solyc03g063040.2.1	2	32	2	19	0	16	7	17	0	39	2	19	0
Solyc03g063060.2.1	3	20	2	3	4	6	19	31	5	15	1	5	0
Solyc03g063100.1.1	0	0	0	0	0	0	0	0	0	0	0	0	0
Solyc03g063110.2.1	7	29	9	18	4	81	27	106	7	159	48	94	6
Solyc03g063140.2.1	0	0	0	0	0	0	0	0	0	0	0	0	0
Solyc03g063150.2.1	0	0	0	0	0	0	0	0	0	0	0	0	0
Solyc03g063160.1.1	0	0	0	0	0	0	0	0	0	0	0	0	0

Solyc03g063190.2.1	1	0	0	1	0	9	3	1	3	4	2	2	0
Solyc03g063200.2.1	1	4	0	12	2	24	8	23	0	23	1	6	0
Solyc03g063210.2.1	7	24	3	8	2	77	64	78	5	24	20	20	0
Solyc03g063220.1.1	2	4	1	6	0	18	14	20	4	14	3	4	0
Solyc03g063240.2.1	8	141	13	64	4	348	52	272	4	120	16	99	2
Solyc03g063260.2.1	0	0	0	0	0	0	0	0	0	0	0	0	0
Solyc03g063270.2.1	0	0	0	0	0	0	0	0	0	0	0	0	0
Solyc03g063280.2.1	10	208	9	166	1	424	101	433	26	312	56	218	9
Solyc03g063290.2.1	5	71	8	16	6	121	58	104	29	131	16	75	0
Solyc03g063320.2.1	5	12	4	7	0	35	26	48	2	15	15	27	1
Solyc03g063340.2.1	6	32	3	26	3	65	28	75	3	93	18	69	1
Solyc03g063350.2.1	5	50	2	33	1	61	26	65	3	42	8	41	0
Solyc03g063360.2.1	0	0	0	0	0	0	0	0	0	0	0	0	0
Solyc03g063390.1.1	0	0	0	0	0	0	0	0	0	0	0	0	0
Solyc03g063420.1.1	0	0	0	0	0	0	0	0	0	0	0	0	0
Solyc03g063470.1.1	0	0	0	0	0	0	0	0	0	0	0	0	0
Solyc03g063480.2.1	0	0	0	0	0	0	0	0	0	0	0	0	0
Solyc03g063550.1.1	0	0	0	0	0	0	0	0	0	0	0	0	0
Solyc03g063560.2.1	29	1198	257	941	15	229	682	590	19	698	99	738	10
Solyc03g063600.2.1	12	448	23	201	10	7211	248	1819	127	375	114	331	6
Solyc03g063650.1.1	2	7	0	8	0	6	4	1	0	7	0	3	0
Solyc03g063680.1.1	0	0	0	0	0	0	0	0	0	0	0	0	0
Solyc03g063690.1.1	1	1	0	2	0	4	5	3	1	0	1	0	0
Solyc03g063720.2.1	0	0	0	0	0	0	0	0	0	0	0	0	0
Solyc03g063730.2.1	7	26	5	21	1	124	76	76	0	4	10	12	2
Solyc03g063750.2.1	6	48	6	25	1	88	35	111	4	106	26	64	1
Solyc03g063760.2.1	3	13	4	17	0	19	8	17	1	21	4	24	0
Solyc03g063780.1.1	0	0	0	0	0	0	0	0	0	0	0	0	0
Solyc03g063880.2.1	7	70	4	43	7	88	45	98	20	251	33	205	11
Solyc03g063970.2.1	0	0	0	0	0	0	0	0	0	0	0	0	0
Solyc03g064010.2.1	0	0	0	0	0	0	0	0	0	0	0	0	0
Solyc03g064030.2.1	5	43	0	16	2	52	78	79	4	57	24	53	0
Solyc03g064040.1.1	0	0	0	0	0	0	0	0	0	0	0	0	0
Solyc03g065200.2.1	0	0	0	0	0	0	0	0	0	0	0	0	0
Solyc03g065250.2.1	10	254	32	38	164	3598	2144	1599	1688	36	78	27	12
Solyc03g065340.2.1	16	98	12	74	11	290	105	386	3	724	85	974	56
Solyc03g070380.2.1	0	0	0	0	0	0	0	0	0	0	0	0	0
Solyc03g070390.2.1	0	0	0	0	0	0	0	0	0	0	0	0	0
Solyc03g070440.2.1	0	0	0	0	0	0	0	0	0	0	0	0	0
Solyc03g070470.1.1	0	0	0	0	0	0	0	0	0	0	0	0	0
Solyc03g071540.1.1	0	0	0	0	0	0	0	0	0	0	0	0	0

Solyc03g071550.1.1	1	0	0	0	0	0	5	1	3	1	2	1	5
Solyc03g071560.2.1	3	23	0	9	0	44	20	42	1	12	8	8	0
Solyc03g071570.2.1	4	27	1	13	6	1	0	2	0	33	24	35	0
Solyc03g071590.2.1	9	92	17	76	0	30	123	81	0	45	13	97	0
Solyc03g071620.1.1	4	158	2	65	5	104	40	120	7	134	25	68	1
Solyc03g071660.1.1	0	0	0	0	0	0	0	0	0	0	0	0	0
Solyc03g071670.1.1	0	0	0	0	0	0	0	0	0	0	0	0	0
Solyc03g071690.2.1	6	123	18	40	0	137	127	113	0	10	2	0	0
Solyc03g071700.1.1	0	0	0	0	0	0	0	0	0	0	0	0	0
Solyc03g071710.1.1	1	32	0	7	0	49	10	27	1	92	3	97	1
Solyc03g071720.2.1	0	0	0	0	0	0	0	0	0	0	0	0	0
Solyc03g071750.2.1	0	0	0	0	0	0	0	0	0	0	0	0	0
Solyc03g071850.1.1	0	0	0	0	0	0	0	0	0	0	0	0	0
Solyc03g071860.2.1	0	0	0	0	0	0	0	0	0	0	0	0	0
Solyc03g071870.1.1	0	0	0	0	0	0	0	0	0	0	0	0	0
Solyc03g077910.2.1	1	0	0	0	0	0	20	9	1	7	0	0	0
Solyc03g077920.1.1	9	23	1	15	1	50	55	111	0	13	11	18	0
Solyc03g077950.1.1	1	3	0	0	0	0	1	2	3	17	5	3	0
Solyc03g077960.1.1	0	0	0	0	0	0	0	0	0	0	0	0	0
Solyc03g077980.2.1	0	0	0	0	0	0	0	0	0	0	0	0	0
Solyc03g078000.2.1	15	435	148	371	7	581	724	505	11	480	65	395	3
Solyc03g078020.2.1	0	0	0	0	0	0	0	0	0	0	0	0	0
Solyc03g078030.1.1	0	0	0	0	0	0	0	0	0	0	0	0	0
Solyc03g078060.2.1	0	0	0	0	0	0	0	0	0	0	0	0	0
Solyc03g078080.2.1	7	54	3	22	2	60	66	84	3	54	7	55	0
Solyc03g078090.2.1	0	0	0	0	0	0	0	0	0	0	0	0	0
Solyc03g078100.2.1	3	15	4	26	0	28	31	70	2	46	6	48	0
Solyc03g078120.2.1	0	0	0	0	0	0	0	0	0	0	0	0	0
Solyc03g078150.2.1	4	2	0	1	0	186	16	98	0	7	0	10	0
Solyc03g078160.2.1	2	30	1	12	0	58	31	41	17	36	25	22	1
Solyc03g078200.1.1	0	0	0	0	0	0	0	0	0	0	0	0	0
Solyc03g078230.2.1	3	18	2	1	5	12	34	18	5	14	8	11	0
Solyc03g078240.1.1	0	0	0	0	0	0	0	0	0	0	0	0	0
Solyc03g078260.2.1	0	0	0	0	0	0	0	0	0	0	0	0	0
Solyc03g078280.1.1	0	0	0	0	0	0	0	0	0	0	0	0	0
Solyc03g078290.2.1	2	35	2	15	0	64	7	81	2	52	24	42	3
Solyc03g078300.1.1	5	16	2	13	0	41	57	44	4	85	1	27	4
Solyc03g078350.2.1	0	0	0	0	0	0	0	0	0	0	0	0	0
Solyc03g078360.1.1	0	0	0	0	0	0	0	0	0	0	0	0	0
Solyc03g078370.1.1	3	9	3	10	12	21	2	12	2	22	12	27	10
Solyc03g078380.1.1	9	261	20	168	8	162	89	222	6	558	59	350	0

Solyc03g078390.2.1	5	11	1	15	0	24	26	31	9	28	8	20	3
Solyc03g078400.2.1	5	1738	122	1196	80	4484	1264	4407	192	8298	969	5297	44
Solyc03g078490.2.1	8	141	0	51	0	1606	46	786	0	3	0	2	0
Solyc03g078520.2.1	5	40	3	27	0	235	66	168	29	47	4	37	5
Solyc03g078570.2.1	2	36	10	34	2	49	37	54	17	75	41	56	10
Solyc03g078610.2.1	1	2	0	13	0	2	7	7	0	0	0	4	0
Solyc03g078620.1.1	0	0	0	0	0	0	0	0	0	0	0	0	0
Solyc03g078630.2.1	0	0	0	0	0	0	0	0	0	0	0	0	0
Solyc03g078640.1.1	14	317	40	146	35	43	26	85	14	86	59	108	7
Solyc03g078650.2.1	0	0	0	0	0	0	0	0	0	0	0	0	0
Solyc03g078690.2.1	0	0	0	0	0	0	0	0	0	0	0	0	0
Solyc03g078700.2.1	0	0	0	0	0	0	0	0	0	0	0	0	0
Solyc03g078710.1.1	0	0	0	0	0	0	0	0	0	0	0	0	0
Solyc03g078720.2.1	0	0	0	0	0	0	0	0	0	0	0	0	0
Solyc03g078730.1.1	0	0	0	0	0	0	0	0	0	0	0	0	0
Solyc03g078740.1.1	0	0	0	0	0	0	0	0	0	0	0	0	0
Solyc03g078770.2.1	0	0	0	0	0	0	0	0	0	0	0	0	0
Solyc03g078780.1.1	0	0	0	0	0	0	0	0	0	0	0	0	0
Solyc03g078790.1.1	0	0	0	0	0	0	0	0	0	0	0	0	0
Solyc03g078800.2.1	0	0	0	0	0	0	0	0	0	0	0	0	0
Solyc03g078830.1.1	1	0	0	1	0	4	6	3	0	0	0	0	0
Solyc03g079850.2.1	1	12	0	2	0	1	1	23	1	4	4	11	1
Solyc03g079870.1.1	0	0	0	0	0	0	0	0	0	0	0	0	0
Solyc03g079910.2.1	0	0	0	0	0	0	0	0	0	0	0	0	0
Solyc03g079920.2.1	2	7	5	13	0	1	14	12	0	28	5	10	0
Solyc03g079930.2.1	7	92	1	49	4	561	39	450	19	301	58	289	5
Solyc03g079940.2.1	5	77	15	36	3	236	127	188	21	130	23	119	3
Solyc03g079950.2.1	3	11	3	10	0	62	13	18	1	11	2	24	0
Solyc03g079960.2.1	0	0	0	0	0	0	0	0	0	0	0	0	0
Solyc03g079970.1.1	0	0	0	0	0	0	0	0	0	0	0	0	0
Solyc03g079980.1.1	0	0	0	0	0	0	0	0	0	0	0	0	0
Solyc03g080010.2.1	4	15	0	3	0	7	33	7	4	10	1	10	3
Solyc03g080020.2.1	6	39	6	19	8	184	24	128	12	14	30	24	2
Solyc03g080030.2.1	0	0	0	0	0	0	0	0	0	0	0	0	0
Solyc03g080040.1.1	0	0	0	0	0	0	0	0	0	0	0	0	0
Solyc03g080050.2.1	6	20	2	25	7	78	41	82	14	46	7	25	1
Solyc03g080060.1.1	11	104	11	72	2	213	173	323	3	160	38	190	1
Solyc03g080070.2.1	0	0	0	0	0	0	0	0	0	0	0	0	0
Solyc03g080080.2.1	6	49	12	43	2	82	85	111	10	40	5	51	9
Solyc03g080090.2.1	15	312	63	227	63	353	162	328	50	310	25	435	30
Solyc03g080100.2.1	10	32	2	15	14	16	77	12	8	736	30	460	14

Solyc03g080110.2.1	0	0	0	0	0	0	0	0	0	0	0	0	0
Solyc03g080120.1.1	0	0	0	0	0	0	0	0	0	0	0	0	0
Solyc03g080140.2.1	40	221	24	150	11	373	210	483	51	558	72	421	6
Solyc03g080160.2.1	3	429	20	106	3	855	397	612	37	1548	120	498	6
Solyc03g080170.2.1	1	4	3	0	0	4	9	1	2	7	8	27	4
Solyc03g080180.2.1	20	532	98	303	0	905	495	703	45	64	114	81	1
Solyc03g080190.2.1	0	0	0	0	0	0	0	0	0	0	0	0	0
Solyc03g081200.1.1	0	0	0	0	0	0	0	0	0	0	0	0	0
Solyc03g081210.1.1	0	0	0	0	0	0	0	0	0	0	0	0	0
Solyc03g081220.1.1	7	42	2	10	2	25	29	41	4	88	20	54	0
Solyc03g081230.1.1	0	0	0	0	0	0	0	0	0	0	0	0	0
Solyc03g081240.2.1	6	15	1	11	2	28	67	40	15	47	24	19	11
Solyc03g081250.1.1	0	0	0	0	0	0	0	0	0	0	0	0	0
Solyc03g081260.2.1	10	41	12	20	0	13	48	46	0	82	20	48	0
Solyc03g081300.2.1	6	47	6	28	2	24	55	51	6	39	9	35	1
Solyc03g081310.2.1	6	48	3	32	0	36	55	46	16	32	7	29	1
Solyc03g081320.2.1	10	116	8	87	2	160	189	148	13	184	33	145	1
Solyc03g081330.1.1	0	0	0	0	0	0	0	0	0	0	0	0	0
Solyc03g082350.2.1	0	0	0	0	0	0	0	0	0	0	0	0	0
Solyc03g082370.1.1	1	59	24	66	4	17	20	4	1	17	1	22	0
Solyc03g082380.2.1	5	121	18	129	5	275	102	249	18	221	40	194	2
Solyc03g082390.2.1	3	37	0	24	0	62	20	63	2	30	9	27	1
Solyc03g082400.2.1	9	56	3	29	1	175	62	220	7	128	22	64	0
Solyc03g082410.2.1	2	7	0	3	0	12	10	8	0	10	1	6	0
Solyc03g082420.2.1	0	0	0	0	0	0	0	0	0	0	0	0	0
Solyc03g082440.1.1	13	119	18	64	15	132	99	152	45	386	82	341	13
Solyc03g082450.2.1	0	0	0	0	0	0	0	0	0	0	0	0	0
Solyc03g082470.2.1	0	0	0	0	0	0	0	0	0	0	0	0	0
Solyc03g082480.2.1	1	38	2	14	0	81	41	59	2	42	15	33	14
Solyc03g082490.2.1	16	218	17	190	6	358	192	490	24	411	56	295	4
Solyc03g082500.2.1	6	38	8	52	5	94	27	72	11	52	19	21	0
Solyc03g082510.1.1	0	0	0	0	0	0	0	0	0	0	0	0	0
Solyc03g082520.1.1	0	0	0	0	0	0	0	0	0	0	0	0	0
Solyc03g082530.1.1	0	0	0	0	0	0	0	0	0	0	0	0	0
Solyc03g082540.2.1	10	62	8	49	5	71	60	77	15	110	43	81	0
Solyc03g082550.2.1	12	11	0	10	0	1345	797	496	90	12	5	8	0
Solyc03g082560.2.1	12	103	25	54	0	93	495	127	6	54	15	37	5
Solyc03g082570.2.1	4	119	10	90	2	364	60	141	20	166	26	133	0
Solyc03g082580.2.1	10	87	6	41	8	98	59	143	22	202	48	152	6
Solyc03g082590.2.1	4	31	2	33	8	96	70	115	7	118	20	54	0
Solyc03g082600.2.1	4	233	31	39	0	132	102	103	41	159	89	131	7

Solyc03g082610.1.1	3	80	2	21	0	7	4	30	2	25	9	13	0
Solyc03g082620.2.1	20	458	88	441	24	1087	564	1122	88	144	89	189	7
Solyc03g082630.2.1	1	0	0	3	0	17	3	12	1	11	1	1	0
Solyc03g082640.1.1	0	0	0	0	0	0	0	0	0	0	0	0	0
Solyc03g082660.2.1	8	619	42	307	32	388	85	176	28	638	305	603	25
Solyc03g082670.2.1	2	21	0	5	3	6	5	8	2	15	1	11	0
Solyc03g082680.2.1	17	55	5	60	9	202	111	155	11	78	24	48	1
Solyc03g082690.2.1	1	5	0	7	0	4	5	9	0	2	0	0	0
Solyc03g082700.2.1	0	0	0	0	0	0	0	0	0	0	0	0	0
Solyc03g082710.2.1	2	11	0	2	0	20	9	15	0	28	6	25	0
Solyc03g082720.2.1	1	56	0	18	0	47	2	56	1	184	4	80	0
Solyc03g082740.2.1	1	4	0	5	0	2	0	9	1	7	3	9	0
Solyc03g082820.2.1	8	51	13	21	4	106	124	78	18	63	19	83	0
Solyc03g082830.1.1	0	0	0	0	0	0	0	0	0	0	0	0	0
Solyc03g082840.2.1	1	5	0	0	0	2	0	5	0	2	4	0	0
Solyc03g082850.2.1	2	17	1	16	2	12	13	41	4	52	12	27	0
Solyc03g082860.2.1	0	0	0	0	0	0	0	0	0	0	0	0	0
Solyc03g082870.2.1	5	14	3	1	2	7	34	12	9	21	10	19	0
Solyc03g082880.1.1	3	12	0	2	0	30	27	17	5	13	5	14	0
Solyc03g082890.2.1	6	95	6	39	1	24	86	39	1	58	21	69	0
Solyc03g082900.2.1	0	0	0	0	0	0	0	0	0	0	0	0	0
Solyc03g082910.2.1	3	12	2	8	0	14	13	21	7	19	9	23	0
Solyc03g082920.2.1	11	476	22	222	16	868	288	1000	92	484	340	491	0
Solyc03g082940.2.1	15	187	23	209	18	259	269	501	47	453	131	416	13
Solyc03g082960.2.1	9	168	37	124	0	94	69	158	3	105	6	104	0
Solyc03g082970.2.1	7	62	2	41	0	45	113	47	4	56	6	73	0
Solyc03g082980.2.1	13	107	16	77	7	377	135	338	19	259	42	192	0
Solyc03g082990.2.1	3	2	0	5	0	4	11	3	0	7	0	1	0
Solyc03g083000.2.1	6	20	3	19	0	29	57	46	9	50	8	45	0
Solyc03g083010.2.1	1	12	13	6	0	7	0	13	0	2	0	1	0
Solyc03g083020.2.1	0	0	0	0	0	0	0	0	0	0	0	0	0
Solyc03g083030.2.1	4	24	0	11	0	37	9	68	2	27	12	21	0
Solyc03g083050.2.1	0	0	0	0	0	0	0	0	0	0	0	0	0
Solyc03g083060.2.1	2	13	2	9	0	0	3	7	0	8	6	6	0
Solyc03g083070.1.1	5	45	2	11	0	10	14	32	3	58	6	32	2
Solyc03g083080.1.1	1	1	14	0	8	23	16	1	4	1	31	0	2
Solyc03g083090.2.1	15	284	16	135	9	595	178	618	11	803	107	724	2
Solyc03g083100.2.1	3	29	1	5	0	0	6	6	6	45	2	50	0
Solyc03g083130.1.1	0	0	0	0	0	0	0	0	0	0	0	0	0
Solyc03g083140.2.1	0	0	0	0	0	0	0	0	0	0	0	0	0
Solyc03g083150.2.1	0	0	0	0	0	0	0	0	0	0	0	0	0

Solyc03g083170.2.1	0	0	0	0	0	0	0	0	0	0	0	0	0
Solyc03g083190.1.1	0	0	0	0	0	0	0	0	0	0	0	0	0
Solyc03g083210.2.1	5	19	1	8	3	27	12	45	8	28	16	34	1
Solyc03g083240.2.1	8	36	1	1	4	26	52	42	8	65	4	31	1
Solyc03g083250.2.1	13	189	11	105	0	249	307	420	26	191	42	158	7
Solyc03g083260.1.1	0	0	0	0	0	0	0	0	0	0	0	0	0
Solyc03g083270.2.1	3	25	6	16	0	75	54	45	8	49	9	16	1
Solyc03g083280.2.1	10	86	9	77	2	381	79	306	16	153	32	104	12
Solyc03g083290.2.1	5	80	3	44	0	94	56	133	12	74	8	36	1
Solyc03g083300.2.1	1	28	3	6	0	0	9	13	1	10	3	15	0
Solyc03g083310.2.1	9	108	4	84	4	250	68	174	16	217	26	103	4
Solyc03g083320.2.1	0	0	0	0	0	0	0	0	0	0	0	0	0
Solyc03g083330.2.1	21	66	15	42	1	513	249	417	60	117	52	159	5
Solyc03g083350.2.1	10	30	3	71	12	45	67	86	17	992	89	949	6
Solyc03g083360.2.1	5	219	3	89	0	4	7	8	3	36	9	38	3
Solyc03g083390.2.1	8	155	20	187	9	432	168	520	38	675	123	518	4
Solyc03g083400.2.1	5	28	0	6	4	324	59	163	3	77	4	64	0
Solyc03g083410.2.1	4	13	2	11	0	74	11	22	1	7	1	8	0
Solyc03g083420.2.1	18	107	20	105	0	639	123	649	11	149	32	125	4
Solyc03g083430.2.1	11	138	25	91	8	310	191	378	31	381	83	278	3
Solyc03g083440.2.1	12	31	5	19	10	139	94	168	37	8	11	5	0
Solyc03g083450.1.1	4	38	4	16	0	128	39	107	7	53	19	41	2
Solyc03g083460.2.1	1	3	0	0	0	24	2	4	0	0	0	2	0
Solyc03g083470.2.1	2	9	3	11	0	1	4	5	0	1	3	15	0
Solyc03g083480.2.1	4	203	34	276	2	20	1	21	0	3	0	55	0
Solyc03g083510.2.1	0	0	0	0	0	0	0	0	0	0	0	0	0
Solyc03g083520.2.1	1	19	0	10	0	17	9	13	1	22	1	1	0
Solyc03g083530.2.1	4	134	2	99	0	125	16	144	2	379	38	671	4
Solyc03g083540.2.1	5	22	1	27	5	20	25	53	7	17	10	35	17
Solyc03g083550.1.1	0	0	0	0	0	0	0	0	0	0	0	0	0
Solyc03g083560.1.1	3	34	4	18	0	0	1	2	4	68	4	42	2
Solyc03g083570.2.1	6	132	16	77	3	63	147	89	1	22	14	14	0
Solyc03g083580.2.1	0	0	0	0	0	0	0	0	0	0	0	0	0
Solyc03g083590.2.1	0	0	0	0	0	0	0	0	0	0	0	0	0
Solyc03g083600.1.1	0	0	0	0	0	0	0	0	0	0	0	0	0
Solyc03g083610.2.1	3	35	0	16	0	28	3	14	3	81	9	59	0
Solyc03g083620.1.1	5	765	61	495	38	2210	400	1328	127	6733	183	1381	11
Solyc03g083650.2.1	1	4	6	0	1	2	0	5	0	3	0	9	0
Solyc03g083680.1.1	0	0	0	0	0	0	0	0	0	0	0	0	0
Solyc03g083710.1.1	0	0	0	0	0	0	0	0	0	0	0	0	0
Solyc03g083720.1.1	0	0	0	0	0	0	0	0	0	0	0	0	0

Solyc03g083730.1.1	7	32	30	31	13	17	100	15	14	0	0	0	0
Solyc03g083770.1.1	2	72	14	13	11	3	91	5	5	5	0	6	0
Solyc03g083810.2.1	0	0	0	0	0	0	0	0	0	0	0	0	0
Solyc03g083880.2.1	0	0	0	0	0	0	0	0	0	0	0	0	0
Solyc03g083900.2.1	5	23	0	3	1	4	6	31	1	2	23	10	0
Solyc03g083910.2.1	12	182	18	193	4	539	360	1331	26	726	8	1079	3
Solyc03g083960.2.1	0	0	0	0	0	0	0	0	0	0	0	0	0
Solyc03g083970.2.1	9	136	6	47	11	181	83	285	24	351	69	314	1
Solyc03g093080.2.1	0	0	0	0	0	0	0	0	0	0	0	0	0
Solyc03g093110.2.1	0	0	0	0	0	0	0	0	0	0	0	0	0
Solyc03g093120.2.1	0	0	0	0	0	0	0	0	0	0	0	0	0
Solyc03g093130.2.1	0	0	0	0	0	0	0	0	0	0	0	0	0
Solyc03g093140.2.1	9	38	8	32	4	67	45	68	17	181	29	206	0
Solyc03g093150.1.1	1	2	0	0	0	16	5	8	0	12	0	2	0
Solyc03g093160.2.1	5	48	7	23	6	89	40	87	19	55	13	51	0
Solyc03g093180.1.1	2	29	2	14	0	2	7	9	0	3	0	6	0
Solyc03g093200.2.1	4	33	2	28	1	34	36	25	7	102	8	96	0
Solyc03g093210.2.1	2	3	0	1	0	3	5	2	0	1	2	3	0
Solyc03g093220.2.1	2	28	6	13	1	16	22	51	9	89	11	70	2
Solyc03g093240.2.1	14	55	8	31	3	25	110	74	3	61	21	42	2
Solyc03g093250.1.1	0	0	0	0	0	0	0	0	0	0	0	0	0
Solyc03g093260.2.1	0	0	0	0	0	0	0	0	0	0	0	0	0
Solyc03g093300.2.1	1	1	0	0	0	13	35	2	1	5	0	6	0
Solyc03g093310.2.1	0	0	0	0	0	0	0	0	0	0	0	0	0
Solyc03g093330.2.1	0	0	0	0	0	0	0	0	0	0	0	0	0
Solyc03g093340.2.1	5	9	0	11	0	12	21	32	1	5	4	5	0
Solyc03g093350.2.1	16	41	10	22	5	43	140	101	34	82	65	75	13
Solyc03g093360.2.1	4	55	2	19	4	4	48	15	0	378	126	555	7
Solyc03g093370.1.1	0	0	0	0	0	0	0	0	0	0	0	0	0
Solyc03g093380.1.1	0	0	0	0	0	0	0	0	0	0	0	0	0
Solyc03g093390.2.1	0	0	0	0	0	0	0	0	0	0	0	0	0
Solyc03g093400.2.1	7	58	26	37	9	49	52	61	19	4	7	0	1
Solyc03g093410.2.1	0	0	0	0	0	0	0	0	0	0	0	0	0
Solyc03g093430.2.1	1	4	2	15	0	106	19	47	3	54	4	28	0
Solyc03g093440.2.1	1	4	0	2	0	0	1	1	1	1	3	0	0
Solyc03g093450.2.1	3	43	26	19	14	57	208	38	40	116	48	21	42
Solyc03g093460.2.1	3	22	6	1	0	10	33	7	21	5	7	0	0
Solyc03g093470.2.1	4	16	0	7	0	15	28	28	1	22	0	4	0
Solyc03g093480.2.1	10	17	3	36	3	86	37	135	7	89	16	95	1
Solyc03g093490.2.1	0	0	0	0	0	0	0	0	0	0	0	0	0
Solyc03g093500.2.1	0	0	0	0	0	0	0	0	0	0	0	0	0

Solyc03g093510.2.1	2	13	3	7	1	38	13	25	2	10	3	10	0
Solyc03g093520.2.1	8	29	5	12	0	148	107	142	16	46	1	49	0
Solyc03g093530.1.1	0	0	0	0	0	0	0	0	0	0	0	0	0
Solyc03g093540.1.1	0	0	0	0	0	0	0	0	0	0	0	0	0
Solyc03g093550.1.1	5	220	46	501	4	8	7	20	0	84	3	159	1
Solyc03g093560.1.1	7	92	21	193	6	7	7	13	0	94	2	167	2
Solyc03g093590.1.1	0	0	0	0	0	0	0	0	0	0	0	0	0
Solyc03g093610.1.1	5	98	7	41	4	22	3	1	0	76	1	65	0
Solyc03g093630.1.1	0	0	0	0	0	0	0	0	0	0	0	0	0
Solyc03g093640.2.1	4	58	1	17	0	93	33	91	14	57	7	22	5
Solyc03g093680.2.1	2	46	11	27	5	72	93	82	13	84	20	88	0
Solyc03g093690.2.1	12	1643	136	1089	21	782	887	1179	48	1518	207	1597	5
Solyc03g093700.1.1	0	0	0	0	0	0	0	0	0	0	0	0	0
Solyc03g093780.1.1	0	0	0	0	0	0	0	0	0	0	0	0	0
Solyc03g093790.2.1	1	5	15	2	18	0	10	3	5	0	0	0	0
Solyc03g093800.1.1	7	475	4	68	0	462	223	696	2	25	1	26	0
Solyc03g093830.2.1	12	390	109	309	12	474	1059	753	67	293	153	253	4
Solyc03g093840.1.1	0	0	0	0	0	0	0	0	0	0	0	0	0
Solyc03g093850.1.1	0	0	0	0	0	0	0	0	0	0	0	0	0
Solyc03g093860.1.1	0	0	0	0	0	0	0	0	0	0	0	0	0
Solyc03g093880.2.1	1	1	2	0	0	6	0	5	0	29	1	5	0
Solyc03g093890.2.1	0	0	0	0	0	0	0	0	0	0	0	0	0
Solyc03g093970.2.1	1	2	1	0	0	2	13	7	1	2	0	4	0
Solyc03g093990.2.1	7	28	5	19	0	36	29	46	9	43	8	24	0
Solyc03g094000.2.1	0	0	0	0	0	0	0	0	0	0	0	0	0
Solyc03g094010.2.1	1	0	0	0	0	2	8	3	1	2	1	1	0
Solyc03g094020.2.1	0	0	0	0	0	0	0	0	0	0	0	0	0
Solyc03g094030.2.1	0	0	0	0	0	0	0	0	0	0	0	0	0
Solyc03g094040.2.1	0	0	0	0	0	0	0	0	0	0	0	0	0
Solyc03g094050.2.1	0	0	0	0	0	0	0	0	0	0	0	0	0
Solyc03g094060.2.1	0	0	0	0	0	0	0	0	0	0	0	0	0
Solyc03g094080.2.1	6	190	18	93	17	629	269	786	42	474	83	410	0
Solyc03g094090.2.1	2	65	4	52	4	44	71	121	13	174	19	95	1
Solyc03g094100.1.1	0	0	0	0	0	0	0	0	0	0	0	0	0
Solyc03g094110.2.1	0	0	0	0	0	0	0	0	0	0	0	0	0
Solyc03g094120.2.1	1	2	0	1	0	7	2	6	4	0	3	0	0
Solyc03g094160.2.1	7	46	12	61	2	35	24	47	11	18	8	83	9
Solyc03g094170.1.1	0	0	0	0	0	0	0	0	0	0	0	0	0
Solyc03g095180.2.1	1	4	0	0	0	1	5	6	1	3	1	2	0
Solyc03g095190.2.1	7	105	11	88	6	413	202	287	10	132	20	93	0
Solyc03g095200.1.1	3	5	1	4	0	7	8	18	0	13	2	18	0

Solyc03g095210.1.1	0	0	0	0	0	0	0	0	0	0	0	0	0
Solyc03g095220.2.1	9	51	9	43	0	138	121	146	10	116	35	180	0
Solyc03g095230.2.1	0	0	0	0	0	0	0	0	0	0	0	0	0
Solyc03g095240.2.1	1	2	0	0	1	5	1	5	0	0	3	8	0
Solyc03g095250.2.1	6	30	4	21	0	18	30	45	3	26	3	34	0
Solyc03g095260.2.1	7	65	3	50	2	96	62	130	8	62	21	65	0
Solyc03g095290.2.1	9	17	6	8	6	30	56	48	5	48	16	37	0
Solyc03g095300.2.1	0	0	0	0	0	0	0	0	0	0	0	0	0
Solyc03g095310.2.1	7	6	1	6	0	319	205	449	3	12	3	18	1
Solyc03g095320.1.1	0	0	0	0	0	0	0	0	0	0	0	0	0
Solyc03g095350.1.1	0	0	0	0	0	0	0	0	0	0	0	0	0
Solyc03g095360.2.1	4	36	6	14	3	60	28	75	1	105	2	119	0
Solyc03g095410.2.1	17	127	9	95	1	197	125	281	19	204	58	155	2
Solyc03g095450.2.1	1	6	1	6	0	11	2	3	0	9	1	1	0
Solyc03g095470.2.1	9	106	5	45	2	279	177	311	29	90	67	80	1
Solyc03g095490.2.1	8	54	3	27	8	40	66	65	21	31	2	44	0
Solyc03g095500.2.1	0	0	0	0	0	0	0	0	0	0	0	0	0
Solyc03g095510.2.1	10	10	0	7	1	104	56	104	23	11	6	10	0
Solyc03g095600.1.1	1	2	1	5	0	0	2	1	0	18	2	23	0
Solyc03g095610.2.1	6	36	3	38	5	54	41	31	19	14	9	18	0
Solyc03g095620.2.1	9	181	44	236	2	416	811	532	21	272	27	207	4
Solyc03g095650.2.1	0	0	0	0	0	0	0	0	0	0	0	0	0
Solyc03g095670.1.1	0	0	0	0	0	0	0	0	0	0	0	0	0
Solyc03g095680.1.1	7	5	2	0	0	25	32	30	6	15	1	24	0
Solyc03g095690.2.1	5	78	1	70	1	97	26	150	2	328	5	313	0
Solyc03g095700.1.1	6	0	0	0	0	121	66	45	16	0	45	0	0
Solyc03g095710.2.1	10	36	0	15	0	201	125	238	10	72	30	49	0
Solyc03g095720.2.1	5	41	6	25	2	117	64	128	24	36	6	21	0
Solyc03g095730.2.1	3	21	2	5	0	47	1	27	2	37	9	12	1
Solyc03g095760.2.1	6	22	5	18	0	65	30	60	2	40	3	22	0
Solyc03g095770.2.1	0	0	0	0	0	0	0	0	0	0	0	0	0
Solyc03g095780.1.1	0	0	0	0	0	0	0	0	0	0	0	0	0
Solyc03g095810.2.1	0	0	0	0	0	0	0	0	0	0	0	0	0
Solyc03g095820.2.1	1	0	0	1	0	1	3	2	1	20	0	8	0
Solyc03g095840.2.1	3	26	0	25	1	41	34	99	10	55	22	30	0
Solyc03g095850.1.1	2	13	0	4	0	25	4	48	0	25	3	22	0
Solyc03g095860.2.1	0	0	0	0	0	0	0	0	0	0	0	0	0
Solyc03g095900.2.1	0	0	0	0	0	0	0	0	0	0	0	0	0
Solyc03g095940.1.1	0	0	0	0	0	0	0	0	0	0	0	0	0
Solyc03g095960.1.1	6	10	6	8	0	39	24	33	14	23	11	26	1
Solyc03g095970.2.1	0	0	0	0	0	0	0	0	0	0	0	0	0

Solyc03g095980.2.1	0	0	0	0	0	0	0	0	0	0	0	0	0
Solyc03g095990.1.1	3	110	15	68	3	99	51	111	0	72	7	71	0
Solyc03g096000.2.1	12	77	18	69	2	184	88	282	13	103	23	81	1
Solyc03g096030.2.1	1	6	0	4	0	10	9	3	0	0	0	0	0
Solyc03g096040.2.1	1	0	0	0	0	2	57	0	2	3	99	2	7
Solyc03g096050.2.1	1	3	1	3	0	1	4	2	0	0	1	0	0
Solyc03g096070.2.1	0	0	0	0	0	0	0	0	0	0	0	0	0
Solyc03g096080.2.1	0	0	0	0	0	0	0	0	0	0	0	0	0
Solyc03g096090.2.1	0	0	0	0	0	0	0	0	0	0	0	0	0
Solyc03g096220.2.1	0	0	0	0	0	0	0	0	0	0	0	0	0
Solyc03g096290.2.1	9	1979	50	378	31	200	533	544	739	25	58	52	2
Solyc03g096350.2.1	0	0	0	0	0	0	0	0	0	0	0	0	0
Solyc03g096360.2.1	2	80	6	29	2	290	28	359	6	126	7	125	0
Solyc03g096380.2.1	9	74	3	28	0	181	64	233	1	13	5	10	0
Solyc03g096390.2.1	7	32	2	27	5	140	43	173	39	23	28	40	0
Solyc03g096450.1.1	2	11	0	0	0	21	1	12	0	12	1	0	0
Solyc03g096460.2.1	0	0	0	0	0	0	0	0	0	0	0	0	0
Solyc03g096550.2.1	0	0	0	0	0	0	0	0	0	0	0	0	0
Solyc03g096610.1.1	0	0	0	0	0	0	0	0	0	0	0	0	0
Solyc03g096640.2.1	3	5	0	0	0	1	8	12	0	8	5	13	0
Solyc03g096660.1.1	0	0	0	0	0	0	0	0	0	0	0	0	0
Solyc03g096670.2.1	15	46	5	21	2	2612	747	1820	189	107	40	116	0
Solyc03g096730.2.1	8	834	96	401	50	227	308	421	24	4048	188	1984	17
Solyc03g096740.2.1	0	0	0	0	0	0	0	0	0	0	0	0	0
Solyc03g096760.1.1	0	0	0	0	0	0	0	0	0	0	0	0	0
Solyc03g096770.1.1	0	0	0	0	0	0	0	0	0	0	0	0	0
Solyc03g096780.1.1	0	0	0	0	0	0	0	0	0	0	0	0	0
Solyc03g096790.2.1	1	2	0	0	0	1	10	8	2	4	1	3	0
Solyc03g096800.2.1	20	360	39	155	38	76	264	76	92	108	28	94	0
Solyc03g096810.2.1	9	65	4	49	10	106	56	129	17	73	32	81	7
Solyc03g096820.2.1	5	63	7	33	2	79	51	102	18	75	5	21	1
Solyc03g096830.2.1	3	2	1	0	0	6	11	7	0	19	7	8	0
Solyc03g096840.2.1	6	237	47	86	24	74	454	290	34	1290	98	664	56
Solyc03g096850.2.1	5	624	48	376	0	49	98	180	1	208	30	284	2
Solyc03g096860.2.1	4	36	4	16	11	8	36	46	3	132	58	106	4
Solyc03g096870.2.1	0	0	0	0	0	0	0	0	0	0	0	0	0
Solyc03g096880.2.1	10	32	5	17	0	35	16	42	6	23	8	28	0
Solyc03g096890.1.1	0	0	0	0	0	0	0	0	0	0	0	0	0
Solyc03g096900.2.1	14	223	44	118	7	102	150	165	17	42	7	42	3
Solyc03g096910.2.1	3	40	2	26	0	84	15	87	7	196	4	168	3
Solyc03g096920.2.1	20	155	15	111	8	327	296	504	69	357	77	278	11

Solyc03g096930.2.1	2	21	1	12	0	38	21	103	6	47	1	44	1
Solyc03g096940.2.1	1	36	3	11	0	80	39	70	5	31	10	29	2
Solyc03g096950.2.1	6	31	6	43	5	62	68	97	9	38	10	33	0
Solyc03g096960.2.1	0	0	0	0	0	0	0	0	0	0	0	0	0
Solyc03g096980.1.1	11	181	19	122	15	24	84	157	16	374	51	302	5
Solyc03g096990.2.1	15	114	62	63	24	262	230	329	34	332	108	267	8
Solyc03g097000.2.1	2	9	2	1	0	10	65	23	1	4	2	2	1
Solyc03g097010.2.1	4	22	1	12	0	38	19	40	10	115	10	89	0
Solyc03g097020.2.1	2	3	0	13	0	30	6	13	2	6	5	12	0
Solyc03g097030.2.1	8	47	2	12	19	9	43	10	65	42	11	43	5
Solyc03g097050.2.1	5	100	23	36	7	3	3	4	0	12	9	52	3
Solyc03g097060.2.1	6	17	0	5	1	20	18	3	6	36	4	40	0
Solyc03g097070.2.1	7	224	21	133	16	614	237	693	94	203	111	151	13
Solyc03g097080.2.1	0	0	0	0	0	0	0	0	0	0	0	0	0
Solyc03g097100.1.1	0	0	0	0	0	0	0	0	0	0	0	0	0
Solyc03g097110.2.1	10	116	8	66	5	694	144	631	34	247	98	229	0
Solyc03g097120.2.1	13	60	6	42	2	310	96	286	30	221	42	212	1
Solyc03g097130.2.1	4	28	5	27	0	43	28	33	1	5	2	4	0
Solyc03g097140.2.1	3	33	6	13	4	24	33	17	7	34	4	33	1
Solyc03g097150.2.1	4	15	2	11	0	40	17	101	3	37	10	26	0
Solyc03g097160.2.1	5	23	1	25	0	53	19	72	6	123	4	82	11
Solyc03g097170.2.1	10	160	11	24	23	103	111	24	250	8	15	13	1
Solyc03g097180.2.1	5	46	6	18	1	98	56	132	28	70	44	51	0
Solyc03g097190.2.1	13	225	16	181	18	128	64	151	18	369	142	319	14
Solyc03g097200.2.1	4	17	2	23	1	47	46	19	3	47	21	33	0
Solyc03g097210.2.1	13	182	18	121	3	260	216	384	14	185	19	138	1
Solyc03g097230.1.1	1	4	1	0	0	2	2	5	0	0	1	0	0
Solyc03g097240.2.1	0	0	0	0	0	0	0	0	0	0	0	0	0
Solyc03g097250.2.1	8	212	13	74	6	182	79	186	8	397	28	188	4
Solyc03g097260.2.1	32	138	22	131	12	335	276	442	61	312	72	182	10
Solyc03g097270.2.1	5	237	16	113	13	1146	269	807	115	371	86	388	10
Solyc03g097280.2.1	7	26	10	20	1	67	102	95	14	49	18	57	4
Solyc03g097290.2.1	3	31	4	22	12	45	60	100	5	48	30	44	0
Solyc03g097300.2.1	1	4	1	0	0	1	5	7	0	2	0	2	0
Solyc03g097310.2.1	7	52	2	25	0	54	17	115	6	140	20	58	1
Solyc03g097320.2.1	0	0	0	0	0	0	0	0	0	0	0	0	0
Solyc03g097340.1.1	6	33	10	53	1	50	12	50	5	39	16	93	1
Solyc03g097350.2.1	17	255	25	171	23	659	313	658	45	491	117	361	5
Solyc03g097360.2.1	2	9	4	10	1	3	16	14	2	13	7	13	0
Solyc03g097370.2.1	14	1256	82	746	8	238	58	171	9	160	147	216	3
Solyc03g097380.2.1	9	155	2	39	0	57	85	43	0	37	20	19	0

Solyc03g097390.2.1	0	0	0	0	0	0	0	0	0	0	0	0	0
Solyc03g097400.1.1	0	0	0	0	0	0	0	0	0	0	0	0	0
Solyc03g097410.1.1	0	0	0	0	0	0	0	0	0	0	0	0	0
Solyc03g097430.1.1	0	0	0	0	0	0	0	0	0	0	0	0	0
Solyc03g097440.2.1	0	0	0	0	0	0	0	0	0	0	0	0	0
Solyc03g097450.2.1	13	48	5	6	2	85	108	130	20	34	29	48	0
Solyc03g097460.2.1	9	46	10	37	12	85	146	90	13	195	35	108	12
Solyc03g097470.2.1	12	74	8	28	9	202	105	221	20	139	393	100	4
Solyc03g097490.2.1	11	48	8	40	2	92	44	136	21	64	29	53	0
Solyc03g097500.2.1	3	3	0	0	0	29	8	7	13	0	14	0	0
Solyc03g097520.2.1	6	29	1	9	3	23	31	31	2	32	23	35	1
Solyc03g097530.2.1	0	0	0	0	0	0	0	0	0	0	0	0	0
Solyc03g097540.1.1	0	0	0	0	0	0	0	0	0	0	0	0	0
Solyc03g097550.2.1	2	8	0	1	0	27	19	14	1	21	19	21	0
Solyc03g097560.2.1	1	0	0	0	0	0	0	0	0	7	5	6	0
Solyc03g097570.2.1	2	7	3	0	0	94	12	19	0	0	4	0	0
Solyc03g097580.2.1	3	49	2	7	0	32	0	10	0	3	29	11	0
Solyc03g097590.2.1	5	214	16	143	0	37	29	63	0	3	7	1	0
Solyc03g097600.2.1	1	4	0	5	1	27	26	25	0	0	2	3	0
Solyc03g097610.2.1	0	0	0	0	0	0	0	0	0	0	0	0	0
Solyc03g097630.2.1	6	127	2	93	0	174	37	176	10	135	25	108	0
Solyc03g097640.2.1	0	0	0	0	0	0	0	0	0	0	0	0	0
Solyc03g097650.2.1	0	0	0	0	0	0	0	0	0	0	0	0	0
Solyc03g097670.2.1	9	84	1	41	2	86	23	151	0	307	5	233	10
Solyc03g097680.2.1	4	108	6	44	2	115	119	138	16	281	41	189	9
Solyc03g097690.2.1	1	2	3	6	0	9	8	9	0	1	1	0	0
Solyc03g097700.2.1	0	0	0	0	0	0	0	0	0	0	0	0	0
Solyc03g097710.2.1	18	79	12	60	4	296	280	457	31	281	44	246	2
Solyc03g097720.2.1	0	0	0	0	0	0	0	0	0	0	0	0	0
Solyc03g097740.1.1	2	10	4	15	4	6	6	10	4	45	4	15	0
Solyc03g097750.2.1	4	5	1	0	1	14	43	14	3	78	10	91	2
Solyc03g097760.2.1	0	0	0	0	0	0	0	0	0	0	0	0	0
Solyc03g097780.2.1	7	30	1	14	7	68	9	39	1	48	21	68	0
Solyc03g097790.2.1	17	355	49	201	33	1017	462	793	76	574	245	646	12
Solyc03g097800.2.1	2	51	8	30	3	134	31	116	9	90	8	60	1
Solyc03g097820.1.1	2	4	0	0	0	17	14	11	0	0	0	0	0
Solyc03g097830.2.1	3	21	6	16	5	80	71	74	4	76	19	86	6
Solyc03g097840.2.1	5	35	4	8	0	183	31	127	15	9	0	9	0
Solyc03g097850.1.1	0	0	0	0	0	0	0	0	0	0	0	0	0
Solyc03g097860.1.1	0	0	0	0	0	0	0	0	0	0	0	0	0
Solyc03g097870.2.1	0	0	0	0	0	0	0	0	0	0	0	0	0

Solyc03g097900.2.1	0	0	0	0	0	0	0	0	0	0	0	0	0
Solyc03g097910.2.1	5	120	11	93	5	410	231	352	19	251	124	106	1
Solyc03g097920.1.1	5	320	48	280	10	296	195	263	11	250	31	112	0
Solyc03g097930.2.1	0	0	0	0	0	0	0	0	0	0	0	0	0
Solyc03g097940.2.1	1	3	0	0	4	4	1	2	0	1	6	2	1
Solyc03g097950.2.1	2	56	2	29	0	8	26	19	0	21	8	32	0
Solyc03g097960.1.1	0	0	0	0	0	0	0	0	0	0	0	0	0
Solyc03g097970.2.1	0	0	0	0	0	0	0	0	0	0	0	0	0
Solyc03g097980.2.1	6	7	1	19	3	26	37	27	3	18	11	23	3
Solyc03g097990.2.1	2	26	0	7	0	19	10	17	0	8	2	9	0
Solyc03g098000.2.1	13	307	52	174	8	489	682	600	40	138	160	119	10
Solyc03g098010.2.1	0	0	0	0	0	0	0	0	0	0	0	0	0
Solyc03g098020.2.1	10	336	11	268	1	352	46	146	12	122	28	228	0
Solyc03g098030.2.1	0	0	0	0	0	0	0	0	0	0	0	0	0
Solyc03g098050.2.1	2	36	5	25	4	20	36	26	5	34	33	65	0
Solyc03g098060.1.1	0	0	0	0	0	0	0	0	0	0	0	0	0
Solyc03g098070.2.1	2	5	0	9	0	6	15	15	1	0	7	5	0
Solyc03g098090.1.1	2	0	0	0	0	89	10	57	0	0	1	0	0
Solyc03g098100.2.1	0	0	0	0	0	0	0	0	0	0	0	0	0
Solyc03g098110.2.1	7	54	32	30	9	28	116	43	5	70	32	57	8
Solyc03g098120.2.1	1	5	1	0	0	3	1	4	0	6	4	24	0
Solyc03g098130.2.1	1	3	0	3	0	6	3	1	2	5	3	4	0
Solyc03g098140.2.1	8	180	4	36	11	122	97	53	17	247	9	197	1
Solyc03g098150.2.1	16	168	15	121	5	279	183	299	64	100	50	92	3
Solyc03g098160.2.1	10	23	6	16	8	63	57	43	4	48	0	35	0
Solyc03g098170.2.1	3	8	1	6	2	9	23	29	1	80	11	61	0
Solyc03g098180.2.1	2	4	0	0	0	16	23	8	1	15	7	18	0
Solyc03g098190.2.1	0	0	0	0	0	0	0	0	0	0	0	0	0
Solyc03g098200.2.1	1	3	0	2	0	2	0	0	0	0	0	0	0
Solyc03g098210.2.1	3	33	5	29	0	68	24	57	6	39	1	17	0
Solyc03g098220.2.1	5	67	1	18	8	52	36	54	4	69	7	58	0
Solyc03g098230.2.1	10	124	8	89	0	244	108	270	17	240	61	120	2
Solyc03g098240.2.1	15	69	6	20	7	646	815	460	77	461	623	211	20
Solyc03g098250.2.1	0	0	0	0	0	0	0	0	0	0	0	0	0
Solyc03g098270.1.1	0	0	0	0	0	0	0	0	0	0	0	0	0
Solyc03g098280.2.1	11	147	8	246	13	281	111	464	11	626	48	681	7
Solyc03g098290.2.1	0	0	0	0	0	0	0	0	0	0	0	0	0
Solyc03g098300.1.1	0	0	0	0	0	0	0	0	0	0	0	0	0
Solyc03g098320.2.1	6	105	10	86	8	17	2	32	0	18	18	35	0
Solyc03g098330.2.1	0	0	0	0	0	0	0	0	0	0	0	0	0
Solyc03g098340.2.1	4	14	0	4	6	18	15	26	6	43	9	45	3

Solyc03g098400.1.1	0	0	0	0	0	0	0	0	0	0	0	0	0
Solyc03g098410.1.1	4	50	1	25	0	85	31	70	3	152	19	122	5
Solyc03g098420.2.1	0	0	0	0	0	0	0	0	0	0	0	0	0
Solyc03g098430.2.1	1	0	1	0	4	4	4	0	1	0	0	0	0
Solyc03g098440.2.1	5	75	55	66	8	12	489	46	12	223	50	240	20
Solyc03g098450.2.1	0	0	0	0	0	0	0	0	0	0	0	0	0
Solyc03g098460.2.1	5	113	10	68	0	49	8	80	4	40	26	29	9
Solyc03g098470.2.1	2	17	1	13	0	32	20	30	0	39	13	40	0
Solyc03g098480.1.1	0	0	0	0	0	0	0	0	0	0	0	0	0
Solyc03g098490.1.1	0	0	0	0	0	0	0	0	0	0	0	0	0
Solyc03g098580.1.1	0	0	0	0	0	0	0	0	0	0	0	0	0
Solyc03g098590.2.1	4	26	4	15	0	15	34	28	4	57	24	47	0
Solyc03g098600.2.1	8	87	2	55	4	128	69	312	21	147	47	262	0
Solyc03g098610.2.1	4	13	0	13	0	35	31	40	2	12	2	18	0
Solyc03g098620.2.1	6	22	0	16	2	57	35	61	4	118	24	105	7
Solyc03g098630.2.1	8	22	5	7	0	56	49	66	7	26	1	17	0
Solyc03g098640.2.1	2	5	0	8	0	18	30	31	0	39	0	62	0
Solyc03g098650.2.1	1	0	0	0	0	7	6	5	0	0	0	0	0
Solyc03g098660.2.1	3	102	17	42	0	4	1	9	0	4	0	28	0
Solyc03g098670.1.1	0	0	0	0	0	0	0	0	0	0	0	0	0
Solyc03g098690.1.1	0	0	0	0	0	0	0	0	0	0	0	0	0
Solyc03g098710.1.1	0	0	0	0	0	0	0	0	0	0	0	0	0
Solyc03g098730.1.1	0	0	0	0	0	0	0	0	0	0	0	0	0
Solyc03g098740.1.1	4	16	0	5	0	460	28	219	27	1	8	0	0
Solyc03g098750.2.1	1	26	0	27	0	40	2	72	0	16	2	12	0
Solyc03g098760.1.1	0	0	0	0	0	0	0	0	0	0	0	0	0
Solyc03g098780.1.1	2	88	0	173	0	48	3	110	0	3	0	0	0
Solyc03g098790.1.1	8	144	60	1385	1	174	459	1244	1	45	1	47	0
Solyc03g104810.2.1	18	149	14	113	2	343	214	596	25	267	125	206	28
Solyc03g110860.2.1	5	80	8	57	7	81	74	81	3	228	25	146	1
Solyc03g110880.2.1	17	57	14	46	2	104	70	175	15	190	61	134	2
Solyc03g110890.1.1	0	0	0	0	0	0	0	0	0	0	0	0	0
Solyc03g110900.2.1	23	165	15	180	8	362	281	578	49	417	62	275	3
Solyc03g110910.2.1	8	36	6	20	0	56	40	75	7	119	39	95	0
Solyc03g110920.2.1	2	8	0	3	0	22	13	21	0	25	1	23	0
Solyc03g110930.2.1	0	0	0	0	0	0	0	0	0	0	0	0	0
Solyc03g110940.2.1	4	34	8	20	0	20	1	21	2	16	16	44	0
Solyc03g110960.2.1	5	26	8	23	0	37	22	25	0	17	11	13	11
Solyc03g110970.2.1	0	0	0	0	0	0	0	0	0	0	0	0	0
Solyc03g110980.2.1	0	0	0	0	0	0	0	0	0	0	0	0	0
Solyc03g110990.1.1	0	0	0	0	0	0	0	0	0	0	0	0	0

Solyc03g111000.2.1	0	0	0	0	0	0	0	0	0	0	0	0	0
Solyc03g111010.2.1	7	347	17	177	12	1560	211	1419	33	296	64	262	12
Solyc03g111030.2.1	2	1	0	0	1	5	7	7	4	16	1	7	0
Solyc03g111050.2.1	8	23	5	33	0	35	100	39	0	41	20	35	2
Solyc03g111060.2.1	0	0	0	0	0	0	0	0	0	0	0	0	0
Solyc03g111070.2.1	0	0	0	0	0	0	0	0	0	0	0	0	0
Solyc03g111080.2.1	0	0	0	0	0	0	0	0	0	0	0	0	0
Solyc03g111090.2.1	20	300	34	336	37	5541	374	1283	49	766	118	587	10
Solyc03g111100.1.1	15	346	49	204	45	3516	1019	2667	289	45	6	22	0
Solyc03g111120.2.1	0	0	0	0	0	0	0	0	0	0	0	0	0
Solyc03g111130.1.1	0	0	0	0	0	0	0	0	0	0	0	0	0
Solyc03g111140.2.1	0	0	0	0	0	0	0	0	0	0	0	0	0
Solyc03g111150.2.1	2	11	0	8	0	1	5	6	1	13	7	8	0
Solyc03g111160.2.1	6	41	1	39	0	143	164	121	1	255	10	144	1
Solyc03g111170.2.1	8	60	5	24	1	326	31	207	44	160	20	44	0
Solyc03g111180.2.1	8	61	4	41	1	198	81	256	21	114	21	69	0
Solyc03g111200.2.1	1	18	0	3	4	37	5	32	6	27	19	24	0
Solyc03g111210.2.1	1	0	1	0	0	3	3	5	0	3	1	3	0
Solyc03g111230.2.1	1	194	22	28	8	342	293	422	66	434	163	257	11
Solyc03g111250.2.1	6	19	0	14	4	107	39	89	5	60	7	32	2
Solyc03g111260.2.1	14	76	6	66	1	81	183	232	8	222	15	176	5
Solyc03g111270.2.1	0	0	0	0	0	0	0	0	0	0	0	0	0
Solyc03g111290.1.1	0	0	0	0	0	0	0	0	0	0	0	0	0
Solyc03g111300.1.1	0	0	0	0	0	0	0	0	0	0	0	0	0
Solyc03g111310.2.1	12	141	16	169	3	877	913	2097	91	1102	71	440	4
Solyc03g111320.1.1	5	157	34	164	1	208	174	300	35	196	45	163	6
Solyc03g111330.2.1	0	0	0	0	0	0	0	0	0	0	0	0	0
Solyc03g111340.2.1	2	19	1	9	0	104	3	116	7	28	8	28	0
Solyc03g111350.2.1	0	0	0	0	0	0	0	0	0	0	0	0	0
Solyc03g111360.2.1	0	0	0	0	0	0	0	0	0	0	0	0	0
Solyc03g111370.2.1	2	22	11	9	3	92	36	57	6	126	10	16	0
Solyc03g111380.2.1	1	1	0	0	0	4	5	6	0	4	2	9	0
Solyc03g111390.2.1	8	40	2	16	0	105	92	124	13	111	29	81	1
Solyc03g111400.1.1	0	0	0	0	0	0	0	0	0	0	0	0	0
Solyc03g111410.2.1	3	8	0	3	0	19	5	11	0	37	8	27	0
Solyc03g111420.1.1	0	0	0	0	0	0	0	0	0	0	0	0	0
Solyc03g111430.1.1	0	0	0	0	0	0	0	0	0	0	0	0	0
Solyc03g111440.1.1	0	0	0	0	0	0	0	0	0	0	0	0	0
Solyc03g111450.1.1	0	0	0	0	0	0	0	0	0	0	0	0	0
Solyc03g111500.2.1	1	1	0	0	0	0	5	5	0	4	0	0	0
Solyc03g111510.2.1	0	0	0	0	0	0	0	0	0	0	0	0	0

Solyc03g111540.1.1	0	0	0	0	0	0	0	0	0	0	0	0	0
Solyc03g111550.2.1	6	21	6	3	13	336	44	55	156	274	19	38	29
Solyc03g111560.2.1	8	14	1	24	0	72	46	126	14	23	14	68	5
Solyc03g111570.2.1	13	61	4	40	3	242	103	289	27	134	27	115	2
Solyc03g111580.2.1	12	131	15	93	4	223	142	354	28	229	51	191	4
Solyc03g111590.2.1	6	7	3	13	6	28	14	41	10	30	11	15	0
Solyc03g111600.2.1	4	6	0	6	0	24	14	20	1	21	16	20	1
Solyc03g111610.2.1	6	545	77	163	2	88	245	66	1	17	16	9	0
Solyc03g111620.1.1	0	0	0	0	0	0	0	0	0	0	0	0	0
Solyc03g111630.2.1	23	21	1	20	1	404	452	580	28	113	39	73	1
Solyc03g111640.2.1	9	111	8	77	5	371	415	626	30	300	33	194	0
Solyc03g111650.2.1	4	4	3	3	0	18	33	33	3	33	5	45	0
Solyc03g111660.2.1	8	8	3	3	1	11	20	29	5	38	6	33	4
Solyc03g111670.2.1	6	79	6	68	2	10	38	35	5	80	34	62	2
Solyc03g111680.2.1	0	0	0	0	0	0	0	0	0	0	0	0	0
Solyc03g111690.2.1	13	108	8	84	27	49	31	71	13	60	225	41	6
Solyc03g111700.2.1	15	187	40	193	2	72	161	152	11	227	34	315	8
Solyc03g111710.2.1	6	95	21	24	16	10	4	1	3	193	1	85	0
Solyc03g111720.2.1	8	50	1	22	0	1346	127	1377	12	169	436	55	0
Solyc03g111730.2.1	9	7	0	12	0	109	263	127	0	112	578	5	1
Solyc03g111740.2.1	3	8	2	9	0	32	8	17	4	45	5	53	0
Solyc03g111760.2.1	0	0	0	0	0	0	0	0	0	0	0	0	0
Solyc03g111770.2.1	5	28	2	16	0	17	16	31	2	47	11	30	2
Solyc03g111790.2.1	0	0	0	0	0	0	0	0	0	0	0	0	0
Solyc03g111800.2.1	13	97	15	74	16	44	73	84	19	15	1	11	0
Solyc03g111810.2.1	5	96	1	53	0	13	31	27	0	81	2	52	0
Solyc03g111820.2.1	0	0	0	0	0	0	0	0	0	0	0	0	0
Solyc03g111830.2.1	9	91	6	35	1	155	80	133	10	84	23	76	0
Solyc03g111840.2.1	7	586	38	308	1	200	217	378	16	194	49	208	1
Solyc03g111850.2.1	8	103	16	40	0	138	163	177	13	194	51	209	2
Solyc03g111860.1.1	0	0	0	0	0	0	0	0	0	0	0	0	0
Solyc03g111870.2.1	3	18	1	12	0	40	48	26	4	26	18	13	0
Solyc03g111880.2.1	0	0	0	0	0	0	0	0	0	0	0	0	0
Solyc03g111930.2.1	0	0	0	0	0	0	0	0	0	0	0	0	0
Solyc03g111940.2.1	19	16	11	34	2	5	110	7	2	131	98	90	6
Solyc03g111950.2.1	0	0	0	0	0	0	0	0	0	0	0	0	0
Solyc03g111970.2.1	3	19	0	9	0	0	14	10	0	17	3	42	0
Solyc03g112010.2.1	5	33	8	22	0	26	4	48	0	10	9	8	0
Solyc03g112030.1.1	0	0	0	0	0	0	0	0	0	0	0	0	0
Solyc03g112040.1.1	0	0	0	0	0	0	0	0	0	0	0	0	0
Solyc03g112050.2.1	6	91	11	77	13	209	121	206	22	104	39	86	3

Solyc03g112060.2.1	8	128	17	66	5	66	36	40	5	10	3	31	0
Solyc03g112070.2.1	7	53	8	12	11	72	91	167	14	183	26	145	2
Solyc03g112080.1.1	2	1	1	3	0	16	64	12	2	2	7	1	0
Solyc03g112090.2.1	0	0	0	0	0	0	0	0	0	0	0	0	0
Solyc03g112100.2.1	0	0	0	0	0	0	0	0	0	0	0	0	0
Solyc03g112130.1.1	0	0	0	0	0	0	0	0	0	0	0	0	0
Solyc03g112140.2.1	5	34	0	29	0	74	32	70	12	51	2	43	1
Solyc03g112150.1.1	5	211	35	135	2	71	98	89	10	110	54	116	2
Solyc03g112160.2.1	4	22	0	7	0	52	22	54	1	31	4	20	0
Solyc03g112170.1.1	0	0	0	0	0	0	0	0	0	0	0	0	0
Solyc03g112190.2.1	0	0	0	0	0	0	0	0	0	0	0	0	0
Solyc03g112200.1.1	6	209	21	95	0	50	105	228	2	61	23	75	0
Solyc03g112210.1.1	3	32	10	12	0	7	25	16	0	5	3	2	0
Solyc03g112220.1.1	3	170	15	54	0	253	97	294	0	53	7	66	0
Solyc03g112230.2.1	28	508	85	491	95	4906	2392	4522	832	2094	498	1775	124
Solyc03g112240.1.1	0	0	0	0	0	0	0	0	0	0	0	0	0
Solyc03g112270.2.1	16	154	26	120	8	313	192	351	67	220	88	120	3
Solyc03g112280.2.1	7	0	2	0	2	72	142	22	12	9	4	3	0
Solyc03g112290.2.1	1	20	1	2	0	28	12	15	2	31	4	7	0
Solyc03g112300.2.1	0	0	0	0	0	0	0	0	0	0	0	0	0
Solyc03g112310.1.1	5	22	3	39	4	64	35	37	9	39	20	31	0
Solyc03g112320.2.1	4	22	3	29	0	34	28	71	0	1	2	0	0
Solyc03g112330.2.1	4	21	0	40	0	15	29	16	1	2	3	13	0
Solyc03g112340.1.1	3	148	9	84	12	35	9	16	1	49	0	53	0
Solyc03g112350.2.1	5	22	6	8	3	63	13	76	13	38	12	19	3
Solyc03g112360.1.1	4	120	8	86	4	457	58	336	20	571	79	591	3
Solyc03g112370.1.1	2	8	0	0	0	19	4	26	0	18	1	27	0
Solyc03g112380.1.1	0	0	0	0	0	0	0	0	0	0	0	0	0
Solyc03g112390.2.1	1	3	3	0	10	0	81	0	17	9	28	1	5
Solyc03g112420.1.1	0	0	0	0	0	0	0	0	0	0	0	0	0
Solyc03g112440.1.1	5	10	0	2	0	73	20	42	0	39	95	8	0
Solyc03g112450.2.1	3	19	2	21	1	37	27	38	1	47	9	23	0
Solyc03g112460.2.1	0	0	0	0	0	0	0	0	0	0	0	0	0
Solyc03g112500.2.1	0	0	0	0	0	0	0	0	0	0	0	0	0
Solyc03g112510.1.1	0	0	0	0	0	0	0	0	0	0	0	0	0
Solyc03g112520.2.1	6	30	5	21	6	82	56	101	17	35	24	39	0
Solyc03g112530.2.1	7	34	4	14	0	168	36	189	7	116	22	142	1
Solyc03g112540.2.1	0	0	0	0	0	0	0	0	0	0	0	0	0
Solyc03g112550.2.1	1	0	0	0	0	16	0	4	0	4	0	1	0
Solyc03g112560.2.1	3	8	0	3	1	24	8	19	0	26	2	36	0
Solyc03g112570.2.1	1	5	0	0	0	3	3	2	0	9	2	8	0

Solyc03g112580.2.1	0	0	0	0	0	0	0	0	0	0	0	0	0
Solyc03g112590.2.1	9	2	7	2	1	94	264	39	21	12	39	30	1
Solyc03g112600.2.1	1	5	0	0	0	26	3	8	0	4	1	3	0
Solyc03g112610.1.1	1	6	2	5	0	9	5	5	0	9	1	15	0
Solyc03g112620.2.1	2	26	1	12	2	11	14	17	0	17	0	20	0
Solyc03g112630.2.1	2	7	0	10	0	28	10	25	1	0	0	13	0
Solyc03g112640.2.1	5	69	7	47	0	47	49	52	0	45	0	23	0
Solyc03g112650.2.1	6	41	5	22	0	65	28	100	8	72	6	54	0
Solyc03g112660.2.1	42	269	19	259	15	690	362	976	81	453	215	327	9
Solyc03g112670.2.1	10	101	12	54	7	66	113	146	11	114	17	100	0
Solyc03g112680.1.1	0	0	0	0	0	0	0	0	0	0	0	0	0
Solyc03g112700.1.1	2	16	7	24	1	2	9	3	2	0	4	17	0
Solyc03g112720.2.1	1	9	0	0	0	5	6	12	1	18	2	0	0
Solyc03g112730.2.1	0	0	0	0	0	0	0	0	0	0	0	0	0
Solyc03g112750.2.1	0	0	0	0	0	0	0	0	0	0	0	0	0
Solyc03g112760.2.1	20	277	90	375	0	5	70	89	0	210	57	299	7
Solyc03g112770.2.1	0	0	0	0	0	0	0	0	0	0	0	0	0
Solyc03g112780.1.1	0	0	0	0	0	0	0	0	0	0	0	0	0
Solyc03g112790.2.1	0	0	0	0	0	0	0	0	0	0	0	0	0
Solyc03g112810.2.1	3	43	2	20	1	59	19	82	2	60	10	35	1
Solyc03g112820.2.1	2	3	0	0	0	5	2	10	0	6	3	6	0
Solyc03g112830.2.1	14	28	1	33	0	95	50	116	9	54	14	38	1
Solyc03g112840.1.1	0	0	0	0	0	0	0	0	0	0	0	0	0
Solyc03g112850.2.1	2	136	38	21	7	316	255	217	110	211	179	111	9
Solyc03g112860.2.1	0	0	0	0	0	0	0	0	0	0	0	0	0
Solyc03g112870.2.1	11	186	17	111	12	233	60	344	33	185	61	139	1
Solyc03g112880.1.1	0	0	0	0	0	0	0	0	0	0	0	0	0
Solyc03g112890.1.1	0	0	0	0	0	0	0	0	0	0	0	0	0
Solyc03g112900.2.1	4	204	38	143	16	542	378	443	33	536	74	290	6
Solyc03g112910.2.1	13	104	8	57	32	367	151	469	26	187	49	140	13
Solyc03g112920.1.1	0	0	0	0	0	0	0	0	0	0	0	0	0
Solyc03g112930.2.1	0	0	0	0	0	0	0	0	0	0	0	0	0
Solyc03g112940.2.1	1	1	0	0	0	5	4	2	2	0	3	5	0
Solyc03g112950.2.1	1	0	0	7	2	0	12	1	1	5	0	1	0
Solyc03g112960.1.1	0	0	0	0	0	0	0	0	0	0	0	0	0
Solyc03g112970.1.1	1	1	1	1	0	1	6	2	0	2	0	1	14
Solyc03g112980.1.1	0	0	0	0	0	0	0	0	0	0	0	0	0
Solyc03g112990.1.1	4	29	0	49	1	35	22	48	1	78	7	28	0
Solyc03g113000.2.1	13	57	6	54	4	159	113	189	16	123	24	82	0
Solyc03g113010.1.1	8	93	6	64	1	47	52	108	3	209	33	147	4
Solyc03g113020.2.1	2	25	1	4	0	26	16	58	2	11	3	20	2

Solyc03g113030.2.1	12	50	2	27	2	88	55	155	6	274	41	237	4
Solyc03g113040.2.1	0	0	0	0	0	0	0	0	0	0	0	0	0
Solyc03g113060.2.1	1	15	3	0	0	2	0	0	0	10	0	19	0
Solyc03g113070.2.1	0	0	0	0	0	0	0	0	0	0	0	0	0
Solyc03g113080.2.1	1	7	1	1	0	5	0	5	0	3	4	3	0
Solyc03g113090.2.1	3	9	0	8	0	2	6	0	0	46	11	25	1
Solyc03g113100.2.1	2	79	7	74	3	91	33	142	6	167	39	159	1
Solyc03g113120.2.1	0	0	0	0	0	0	0	0	0	0	0	0	0
Solyc03g113130.2.1	1	0	0	2	0	11	2	3	0	12	2	0	0
Solyc03g113140.2.1	4	14	2	5	0	8	18	23	4	30	6	17	1
Solyc03g113160.1.1	0	0	0	0	0	0	0	0	0	0	0	0	0
Solyc03g113180.2.1	0	0	0	0	0	0	0	0	0	0	0	0	0
Solyc03g113190.1.1	3	10	5	2	0	22	9	22	1	3	4	3	2
Solyc03g113210.2.1	0	0	0	0	0	0	0	0	0	0	0	0	0
Solyc03g113220.2.1	3	87	13	113	4	235	122	188	18	83	45	76	3
Solyc03g113240.2.1	6	385	28	140	2	37	56	81	3	113	30	76	2
Solyc03g113250.2.1	0	0	0	0	0	0	0	0	0	0	0	0	0
Solyc03g113260.1.1	0	0	0	0	0	0	0	0	0	0	0	0	0
Solyc03g113270.2.1	10	670	40	512	16	1891	465	1803	79	664	124	731	2
Solyc03g113280.2.1	4	458	11	291	0	153	14	53	0	70	0	81	0
Solyc03g113290.2.1	1	3	0	0	0	4	1	14	0	0	3	5	0
Solyc03g113300.2.1	0	0	0	0	0	0	0	0	0	0	0	0	0
Solyc03g113330.2.1	4	10	1	13	0	102	37	74	9	52	31	22	1
Solyc03g113340.2.1	7	14	0	6	2	34	29	80	8	42	5	25	0
Solyc03g113350.2.1	3	20	6	9	0	10	15	24	1	51	2	85	1
Solyc03g113370.2.1	3	2	1	8	0	1	19	9	0	23	2	23	0
Solyc03g113380.2.1	11	79	12	57	18	81	66	81	26	88	50	109	1
Solyc03g113390.2.1	9	66	0	71	8	178	67	160	30	59	23	83	3
Solyc03g113400.2.1	13	205	19	197	21	347	440	720	66	280	140	277	13
Solyc03g113410.1.1	0	0	0	0	0	0	0	0	0	0	0	0	0
Solyc03g113420.2.1	22	115	123	101	35	809	352	794	70	51	46	15	7
Solyc03g113430.2.1	18	151	28	119	0	927	45	1245	8	34	247	15	0
Solyc03g113450.2.1	0	0	0	0	0	0	0	0	0	0	0	0	0
Solyc03g113460.1.1	4	27	3	3	5	0	6	8	0	73	24	38	2
Solyc03g113480.1.1	0	0	0	0	0	0	0	0	0	0	0	0	0
Solyc03g113490.2.1	0	0	0	0	0	0	0	0	0	0	0	0	0
Solyc03g113500.2.1	15	47	1	37	1	94	102	138	21	103	31	71	0
Solyc03g113510.1.1	0	0	0	0	0	0	0	0	0	0	0	0	0
Solyc03g113520.2.1	2	19	0	4	1	4	0	8	0	2	11	14	0
Solyc03g113540.2.1	0	0	0	0	0	0	0	0	0	0	0	0	0
Solyc03g113550.2.1	0	0	0	0	0	0	0	0	0	0	0	0	0

Solyc03g113560.2.1	0	0	0	0	0	0	0	0	0	0	0	0	0
Solyc03g113590.2.1	7	46	9	29	2	59	169	88	3	88	19	103	2
Solyc03g113600.2.1	0	0	0	0	0	0	0	0	0	0	0	0	0
Solyc03g113610.2.1	1	3	0	0	0	0	11	6	1	3	0	3	0
Solyc03g113620.2.1	6	91	26	70	1	198	224	282	5	452	49	224	9
Solyc03g113630.2.1	11	30	8	24	0	88	187	127	8	64	22	61	5
Solyc03g113640.2.1	13	47	4	27	3	154	140	151	17	99	29	60	6
Solyc03g113660.2.1	4	7	0	2	0	20	17	16	0	37	3	8	0
Solyc03g113670.2.1	0	0	0	0	0	0	0	0	0	0	0	0	0
Solyc03g113680.2.1	7	66	7	86	23	12	25	24	3	82	14	34	2
Solyc03g113690.1.1	0	0	0	0	0	0	0	0	0	0	0	0	0
Solyc03g113700.2.1	9	42	7	17	3	61	47	85	4	92	22	53	8
Solyc03g113710.2.1	0	0	0	0	0	0	0	0	0	0	0	0	0
Solyc03g113720.2.1	0	0	0	0	0	0	0	0	0	0	0	0	0
Solyc03g113730.2.1	1	553	31	282	4	213	177	409	7	841	17	640	4
Solyc03g113740.2.1	1	7	0	1	4	14	21	16	1	13	1	8	0
Solyc03g113750.1.1	1	1	0	0	0	0	0	1	0	15	4	14	0
Solyc03g113760.2.1	0	0	0	0	0	0	0	0	0	0	0	0	0
Solyc03g113770.2.1	6	8	3	6	0	21	32	30	1	17	10	39	0
Solyc03g113780.2.1	7	17	2	20	0	30	61	36	6	81	7	77	0
Solyc03g113790.2.1	6	187	9	108	3	256	30	301	16	267	28	224	1
Solyc03g113800.2.1	11	629	46	431	20	472	239	709	82	2524	110	1693	9
Solyc03g113820.1.1	0	0	0	0	0	0	0	0	0	0	0	0	0
Solyc03g113850.1.1	0	0	0	0	0	0	0	0	0	0	0	0	0
Solyc03g113860.1.1	0	0	0	0	0	0	0	0	0	0	0	0	0
Solyc03g113870.1.1	4	35	7	14	3	33	51	38	9	17	3	12	0
Solyc03g113880.2.1	0	0	0	0	0	0	0	0	0	0	0	0	0
Solyc03g113900.2.1	2	5	1	0	0	15	9	11	0	10	1	4	0
Solyc03g113910.2.1	0	0	0	0	0	0	0	0	0	0	0	0	0
Solyc03g113920.2.1	5	63	19	41	0	207	106	137	0	19	13	8	0
Solyc03g113940.2.1	0	0	0	0	0	0	0	0	0	0	0	0	0
Solyc03g113950.2.1	4	91	57	20	95	46	2	20	19	0	4	0	0
Solyc03g113980.2.1	1	1	6	6	0	0	1	0	1	3	0	1	0
Solyc03g113990.2.1	7	82	16	51	0	74	32	79	11	101	35	81	0
Solyc03g114000.2.1	3	25	5	20	3	38	24	35	4	25	9	12	5
Solyc03g114010.1.1	0	0	0	0	0	0	0	0	0	0	0	0	0
Solyc03g114020.2.1	4	64	3	39	1	13	44	34	2	68	26	44	0
Solyc03g114030.2.1	9	254	40	212	63	163	127	173	23	1163	526	915	14
Solyc03g114040.2.1	0	0	0	0	0	0	0	0	0	0	0	0	0
Solyc03g114050.2.1	3	29	0	9	0	104	25	53	5	18	5	22	0
Solyc03g114060.2.1	27	199	18	137	23	634	342	714	60	270	97	258	1

Solyc03g114070.2.1	2	10	2	8	2	4	7	22	1	0	2	0	0
Solyc03g114090.1.1	0	0	0	0	0	0	0	0	0	0	0	0	0
Solyc03g114100.1.1	1	1	0	0	0	9	43	12	4	10	0	0	3
Solyc03g114110.2.1	0	0	0	0	0	0	0	0	0	0	0	0	0
Solyc03g114120.2.1	3	15	0	10	0	19	12	22	0	19	8	11	1
Solyc03g114130.1.1	2	54	6	8	0	9	7	4	2	10	0	4	0
Solyc03g114140.2.1	14	60	5	43	3	114	176	229	16	120	29	59	0
Solyc03g114150.2.1	15	666	31	1091	66	756	903	1253	206	372	180	370	24
Solyc03g114160.1.1	19	31	28	18	14	240	117	116	85	14	0	32	6
Solyc03g114170.2.1	0	0	0	0	0	0	0	0	0	0	0	0	0
Solyc03g114180.2.1	0	0	0	0	0	0	0	0	0	0	0	0	0
Solyc03g114190.1.1	0	0	0	0	0	0	0	0	0	0	0	0	0
Solyc03g114210.2.1	3	21	1	10	1	97	61	55	6	8	10	9	0
Solyc03g114240.2.1	2	14	0	1	0	31	16	14	0	2	0	7	0
Solyc03g114250.2.1	10	152	19	97	0	29	58	58	4	80	18	66	2
Solyc03g114280.2.1	3	8	3	6	4	7	2	4	4	13	3	10	0
Solyc03g114290.2.1	2	11	0	16	0	33	28	34	0	3	3	8	0
Solyc03g114300.2.1	5	20	1	13	0	64	20	30	2	26	5	41	0
Solyc03g114310.2.1	0	0	0	0	0	0	0	0	0	0	0	0	0
Solyc03g114320.2.1	10	189	19	59	8	29	41	61	8	79	28	79	1
Solyc03g114330.2.1	4	24	3	30	0	24	30	26	5	12	16	19	0
Solyc03g114340.2.1	16	743	77	283	20	363	288	320	9	263	128	191	7
Solyc03g114350.1.1	0	0	0	0	0	0	0	0	0	0	0	0	0
Solyc03g114360.2.1	7	27	6	24	7	133	147	97	8	19	13	11	0
Solyc03g114370.2.1	4	32	4	36	1	127	27	142	18	45	31	74	1
Solyc03g114380.2.1	0	0	0	0	0	0	0	0	0	0	0	0	0
Solyc03g114390.1.1	0	0	0	0	0	0	0	0	0	0	0	0	0
Solyc03g114400.2.1	0	0	0	0	0	0	0	0	0	0	0	0	0
Solyc03g114410.2.1	11	44	11	39	1	111	91	133	20	81	17	69	5
Solyc03g114420.1.1	0	0	0	0	0	0	0	0	0	0	0	0	0
Solyc03g114430.1.1	0	0	0	0	0	0	0	0	0	0	0	0	0
Solyc03g114450.2.1	10	1193	76	522	2	120	84	109	1	102	129	98	8
Solyc03g114460.2.1	9	81	17	21	2	115	104	146	16	112	10	70	4
Solyc03g114470.2.1	6	87	36	33	2	93	176	146	14	170	42	170	5
Solyc03g114480.2.1	0	0	0	0	0	0	0	0	0	0	0	0	0
Solyc03g114500.2.1	10	88	7	55	24	54	50	104	28	187	38	178	3
Solyc03g114510.2.1	3	78	4	43	0	19	49	102	2	73	22	53	1
Solyc03g114520.2.1	3	0	5	0	1	0	46	2	6	21	25	17	2
Solyc03g114580.2.1	15	94	19	84	12	182	201	229	37	218	84	146	0
Solyc03g114590.2.1	3	23	3	10	3	62	21	48	1	65	4	21	1
Solyc03g114600.2.1	0	0	0	0	0	0	0	0	0	0	0	0	0

Solyc03g114640.2.1	11	39	21	18	1	49	131	74	4	72	24	72	0
Solyc03g114660.1.1	11	14	4	21	0	36	53	41	2	55	13	70	1
Solyc03g114670.2.1	2	14	2	10	1	16	8	8	1	14	4	29	0
Solyc03g114680.2.1	12	90	18	84	8	136	62	112	12	204	21	158	5
Solyc03g114690.2.1	2	7	1	3	0	5	0	9	0	8	6	12	5
Solyc03g114700.1.1	0	0	0	0	0	0	0	0	0	0	0	0	0
Solyc03g114710.2.1	6	18	6	12	0	110	45	73	11	6	63	8	3
Solyc03g114720.2.1	2	22	1	9	3	14	3	14	1	45	5	22	1
Solyc03g114730.2.1	10	112	7	50	13	17	53	56	38	263	34	154	18
Solyc03g114750.2.1	0	0	0	0	0	0	0	0	0	0	0	0	0
Solyc03g114760.2.1	3	18	0	8	1	22	26	17	4	23	12	17	0
Solyc03g114780.2.1	2	1	0	1	0	24	8	9	3	9	5	0	0
Solyc03g114790.2.1	3	13	0	3	0	14	32	15	2	49	4	50	2
Solyc03g114800.1.1	3	11	0	2	0	0	19	5	1	23	4	3	0
Solyc03g114810.2.1	2	17	0	18	2	81	18	64	6	28	4	34	0
Solyc03g114820.2.1	0	0	0	0	0	0	0	0	0	0	0	0	0
Solyc03g114830.2.1	5	9	3	7	8	45	16	13	11	1751	273	1191	17
Solyc03g114840.2.1	3	2	0	1	0	1	0	3	0	295	42	177	4
Solyc03g114850.2.1	0	0	0	0	0	0	0	0	0	0	0	0	0
Solyc03g114860.2.1	14	102	24	29	21	124	333	64	117	118	34	84	1
Solyc03g114890.2.1	0	0	0	0	0	0	0	0	0	0	0	0	0
Solyc03g114900.2.1	0	0	0	0	0	0	0	0	0	0	0	0	0
Solyc03g114910.2.1	0	0	0	0	0	0	0	0	0	0	0	0	0
Solyc03g114920.1.1	8	43	4	34	0	75	41	112	6	42	16	32	3
Solyc03g114930.2.1	3	168	10	52	1	5	47	10	2	56	11	54	0
Solyc03g114940.2.1	1	7	1	6	0	0	0	0	0	6	3	0	0
Solyc03g114950.2.1	1	20	10	9	0	27	5	18	1	14	7	3	4
Solyc03g114960.2.1	7	40	2	15	1	120	23	172	9	57	26	58	0
Solyc03g114970.2.1	2	138	17	56	5	15	61	45	3	431	92	275	23
Solyc03g114980.2.1	0	0	0	0	0	0	0	0	0	0	0	0	0
Solyc03g114990.2.1	5	22	2	37	0	76	37	39	7	44	11	29	1
Solyc03g115000.2.1	8	111	15	67	7	13	18	22	8	27	25	31	0
Solyc03g115010.1.1	0	0	0	0	0	0	0	0	0	0	0	0	0
Solyc03g115020.2.1	6	178	16	74	0	24	32	40	3	87	9	117	1
Solyc03g115030.2.1	1	7	2	0	0	9	18	11	6	29	4	12	0
Solyc03g115040.2.1	0	0	0	0	0	0	0	0	0	0	0	0	0
Solyc03g115050.2.1	1	7	0	0	0	3	1	3	0	8	2	5	0
Solyc03g115060.2.1	0	0	0	0	0	0	0	0	0	0	0	0	0
Solyc03g115070.1.1	3	47	11	14	5	18	15	10	0	8	0	25	0
Solyc03g115080.2.1	1	0	0	0	1	3	0	3	2	2	0	1	0
Solyc03g115100.2.1	13	54	12	50	7	48	113	75	20	103	32	51	9

Solyc03g115110.2.1	4	171	11	87	8	521	101	509	26	323	44	305	10
Solyc03g115120.1.1	0	0	0	0	0	0	0	0	0	0	0	0	0
Solyc03g115140.2.1	2	1	0	0	2	22	8	15	2	10	2	3	0
Solyc03g115150.2.1	11	48	2	25	5	63	70	77	7	121	33	68	0
Solyc03g115160.2.1	0	0	0	0	0	0	0	0	0	0	0	0	0
Solyc03g115170.1.1	0	0	0	0	0	0	0	0	0	0	0	0	0
Solyc03g115180.1.1	0	0	0	0	0	0	0	0	0	0	0	0	0
Solyc03g115190.2.1	0	0	0	0	0	0	0	0	0	0	0	0	0
Solyc03g115200.2.1	8	195	5	91	11	9	23	34	0	188	23	108	0
Solyc03g115210.2.1	0	0	0	0	0	0	0	0	0	0	0	0	0
Solyc03g115220.2.1	6	56	4	37	26	19	1	13	7	301	18	123	11
Solyc03g115230.2.1	21	33	2	37	2	104	1	101	1	251	411	283	1
Solyc03g115240.2.1	9	126	12	81	6	295	149	383	36	245	82	216	2
Solyc03g115250.2.1	29	139	25	104	16	295	246	458	26	264	86	259	4
Solyc03g115260.1.1	0	0	0	0	0	0	0	0	0	0	0	0	0
Solyc03g115280.1.1	0	0	0	0	0	0	0	0	0	0	0	0	0
Solyc03g115290.2.1	0	0	0	0	0	0	0	0	0	0	0	0	0
Solyc03g115300.1.1	0	0	0	0	0	0	0	0	0	0	0	0	0
Solyc03g115310.1.1	0	0	0	0	0	0	0	0	0	0	0	0	0
Solyc03g115320.1.1	0	0	0	0	0	0	0	0	0	0	0	0	0
Solyc03g115330.1.1	0	0	0	0	0	0	0	0	0	0	0	0	0
Solyc03g115360.2.1	4	168	6	53	6	360	34	263	9	1143	43	821	10
Solyc03g115370.2.1	20	78	27	17	40	1161	1030	787	443	150	92	108	5
Solyc03g115380.1.1	4	17	4	17	0	18	11	25	0	8	5	9	0
Solyc03g115390.2.1	7	17	1	12	3	28	49	31	3	39	10	25	0
Solyc03g115400.1.1	1	2	0	0	0	0	3	3	0	1	0	0	0
Solyc03g115410.2.1	1	5	0	0	0	5	13	4	0	4	0	0	0
Solyc03g115420.1.1	7	20	3	9	2	62	26	43	12	22	10	31	0
Solyc03g115440.1.1	10	74	13	68	5	119	87	151	22	149	25	84	0
Solyc03g115450.1.1	0	0	0	0	0	0	0	0	0	0	0	0	0
Solyc03g115460.1.1	7	56	7	9	0	78	76	74	23	60	28	17	1
Solyc03g115470.2.1	5	15	3	11	0	18	22	17	6	41	20	12	0
Solyc03g115490.1.1	4	57	2	15	1	39	17	73	6	78	5	55	0
Solyc03g115500.2.1	15	80	11	41	7	75	100	111	8	53	10	30	0
Solyc03g115510.1.1	11	24	5	8	0	111	47	92	11	45	12	30	0
Solyc03g115520.2.1	1	0	0	1	0	8	8	8	0	0	0	1	0
Solyc03g115530.2.1	0	0	0	0	0	0	0	0	0	0	0	0	0
Solyc03g115540.1.1	0	0	0	0	0	0	0	0	0	0	0	0	0
Solyc03g115550.2.1	0	0	0	0	0	0	0	0	0	0	0	0	0
Solyc03g115560.2.1	0	0	0	0	0	0	0	0	0	0	0	0	0
Solyc03g115570.1.1	0	0	0	0	0	0	0	0	0	0	0	0	0

Solyc03g115580.2.1	3	7	0	4	1	7	11	4	1	17	0	11	0
Solyc03g115590.2.1	17	131	23	105	13	274	221	344	29	63	34	60	0
Solyc03g115610.2.1	0	0	0	0	0	0	0	0	0	0	0	0	0
Solyc03g115630.2.1	11	177	23	89	5	233	213	658	50	302	65	304	14
Solyc03g115640.2.1	0	0	0	0	0	0	0	0	0	0	0	0	0
Solyc03g115650.2.1	4	263	6	249	7	316	255	374	20	1937	107	1491	12
Solyc03g115660.2.1	2	23	0	15	6	37	17	19	4	8	0	2	0
Solyc03g115670.1.1	2	13	2	1	0	16	2	17	5	8	8	12	0
Solyc03g115680.2.1	7	59	6	32	7	77	82	105	6	85	13	66	0
Solyc03g115690.1.1	3	1	0	10	0	19	12	16	1	1	5	2	0
Solyc03g115700.2.1	14	279	6	577	13	889	337	1178	32	1863	80	2718	11
Solyc03g115710.1.1	2	3	0	6	0	1	0	2	0	0	4	0	0
Solyc03g115720.2.1	1	11	0	3	0	2	5	5	1	7	1	8	0
Solyc03g115730.2.1	2	1	3	5	0	5	4	11	4	2	6	6	0
Solyc03g115740.1.1	12	195	14	120	14	96	67	106	24	195	36	192	18
Solyc03g115760.2.1	2	30	1	11	1	34	56	20	11	74	7	40	2
Solyc03g115770.2.1	17	36	0	8	0	142	166	257	14	320	21	422	1
Solyc03g115780.1.1	0	0	0	0	0	0	0	0	0	0	0	0	0
Solyc03g115790.1.1	0	0	0	0	0	0	0	0	0	0	0	0	0
Solyc03g115800.1.1	0	0	0	0	0	0	0	0	0	0	0	0	0
Solyc03g115810.2.1	4	17	1	6	0	45	65	28	6	42	18	31	1
Solyc03g115820.2.1	4	662	26	370	1	304	70	172	6	99	17	157	2
Solyc03g115830.1.1	0	0	0	0	0	0	0	0	0	0	0	0	0
Solyc03g115840.2.1	8	31	6	24	2	21	38	79	8	46	6	47	2
Solyc03g115850.2.1	4	7	6	13	0	23	34	55	23	0	4	0	0
Solyc03g115860.2.1	7	51	4	8	0	181	196	201	10	30	40	38	7
Solyc03g115870.2.1	1	36	13	10	3	0	49	10	2	23	0	20	1
Solyc03g115880.1.1	0	0	0	0	0	0	0	0	0	0	0	0	0
Solyc03g115890.2.1	0	0	0	0	0	0	0	0	0	0	0	0	0
Solyc03g115900.2.1	5	8876	1309	3877	29	32	46	43	6	210	578	136	5
Solyc03g115920.2.1	2	22	3	27	0	11	5	25	0	5	0	1	0
Solyc03g115930.1.1	0	0	0	0	0	0	0	0	0	0	0	0	0
Solyc03g115940.2.1	7	157	12	83	11	235	33	185	25	40	7	32	5
Solyc03g115950.2.1	0	0	0	0	0	0	0	0	0	0	0	0	0
Solyc03g115960.2.1	0	0	0	0	0	0	0	0	0	0	0	0	0
Solyc03g115980.1.1	14	3224	501	1144	23	46	105	80	19	326	202	188	3
Solyc03g115990.1.1	9	144	5	63	12	969	75	549	27	228	36	154	5
Solyc03g116000.1.1	0	0	0	0	0	0	0	0	0	0	0	0	0
Solyc03g116020.2.1	9	66	13	37	2	120	64	136	12	205	46	144	12
Solyc03g116030.2.1	3	26	1	13	0	15	7	16	2	2	2	5	0
Solyc03g116040.2.1	3	12	0	6	0	10	2	15	1	28	13	34	4

Solyc03g116060.2.1	0	0	0	0	0	0	0	0	0	0	0	0	0
Solyc03g116070.1.1	0	0	0	0	0	0	0	0	0	0	0	0	0
Solyc03g116100.2.1	8	31	3	3	1	286	110	90	103	7	1	0	0
Solyc03g116110.2.1	5	67	12	24	3	220	120	221	29	67	12	105	0
Solyc03g116130.1.1	0	0	0	0	0	0	0	0	0	0	0	0	0
Solyc03g116140.2.1	12	37	11	23	10	111	118	105	15	69	29	37	0
Solyc03g116150.2.1	4	23	0	5	7	3	25	11	10	12	9	9	2
Solyc03g116160.1.1	1	26	4	0	4	0	2	1	0	0	0	2	0
Solyc03g116170.2.1	1	2	1	0	2	21	6	15	1	11	1	12	0
Solyc03g116180.1.1	1	13	1	14	1	26	7	88	2	17	17	20	0
Solyc03g116190.1.1	0	0	0	0	0	0	0	0	0	0	0	0	0
Solyc03g116200.1.1	1	0	52	0	0	0	313	0	2	15	2	13	0
Solyc03g116220.1.1	0	0	0	0	0	0	0	0	0	0	0	0	0
Solyc03g116230.2.1	0	0	0	0	0	0	0	0	0	0	0	0	0
Solyc03g116270.2.1	0	0	0	0	0	0	0	0	0	0	0	0	0
Solyc03g116280.2.1	0	0	0	0	0	0	0	0	0	0	0	0	0
Solyc03g116290.1.1	0	0	0	0	0	0	0	0	0	0	0	0	0
Solyc03g116320.2.1	8	50	4	46	4	41	290	59	48	44	3	17	1
Solyc03g116330.2.1	7	32	2	24	3	38	32	42	13	55	16	55	5
Solyc03g116340.2.1	5	85	37	64	12	202	122	121	9	102	33	158	8
Solyc03g116350.2.1	4	83	9	68	3	57	18	109	7	96	14	56	1
Solyc03g116360.1.1	0	0	0	0	0	0	0	0	0	0	0	0	0
Solyc03g116370.2.1	0	0	0	0	0	0	0	0	0	0	0	0	0
Solyc03g116380.2.1	7	18	0	13	3	50	23	74	8	81	20	59	0
Solyc03g116390.2.1	6	54	0	6	0	5469	1100	2267	92	12	82	10	1
Solyc03g116400.2.1	0	0	0	0	0	0	0	0	0	0	0	0	0
Solyc03g116460.2.1	3	6	0	7	0	77	27	74	0	53	11	30	0
Solyc03g116470.1.1	0	0	0	0	0	0	0	0	0	0	0	0	0
Solyc03g116480.1.1	7	18	0	9	0	494	194	383	5	28	8	19	0
Solyc03g116490.1.1	2	26	1	36	3	212	20	139	8	25	12	22	0
Solyc03g116500.2.1	0	0	0	0	0	0	0	0	0	0	0	0	0
Solyc03g116510.1.1	0	0	0	0	0	0	0	0	0	0	0	0	0
Solyc03g116530.2.1	3	3	0	0	0	177	35	108	10	6	1	10	4
Solyc03g116540.1.1	0	0	0	0	0	0	0	0	0	0	0	0	0
Solyc03g116550.2.1	5	35	3	19	1	48	35	47	8	88	30	35	3
Solyc03g116560.1.1	2	1	0	8	0	2	2	12	1	18	6	3	0
Solyc03g116570.2.1	7	197	18	96	3	125	59	151	7	208	18	137	1
Solyc03g116590.2.1	1	28	0	5	0	31	14	37	5	15	0	8	0
Solyc03g116600.2.1	2	30	2	8	4	65	17	65	4	26	6	36	1
Solyc03g116610.2.1	0	0	0	0	0	0	0	0	0	0	0	0	0
Solyc03g116620.2.1	0	0	0	0	0	0	0	0	0	0	0	0	0

Solyc03g116630.2.1	2	3	1	12	0	89	7	56	0	12	11	12	0
Solyc03g116640.2.1	0	0	0	0	0	0	0	0	0	0	0	0	0
Solyc03g116660.2.1	1	2	0	0	0	4	8	16	1	6	0	5	0
Solyc03g116670.2.1	5	684	19	681	11	2141	236	2348	14	695	40	733	4
Solyc03g116680.2.1	27	183	25	193	6	758	415	858	113	192	70	126	8
Solyc03g116690.2.1	0	0	0	0	0	0	0	0	0	0	0	0	0
Solyc03g116700.2.1	6	193	2	46	0	334	76	350	116	4	1	3	0
Solyc03g116710.2.1	0	0	0	0	0	0	0	0	0	0	0	0	0
Solyc03g116730.2.1	4	216	34	111	0	30	45	5	5	24	2	17	1
Solyc03g116740.2.1	4	35	2	7	9	50	50	40	39	123	21	18	0
Solyc03g116750.2.1	14	91	10	81	7	454	194	502	34	355	78	360	11
Solyc03g116760.2.1	6	51	2	25	3	61	53	133	10	93	17	80	0
Solyc03g116780.2.1	7	56	9	51	2	141	57	148	17	146	15	78	2
Solyc03g116790.2.1	5	22	4	10	0	30	34	20	13	22	4	6	1
Solyc03g116800.2.1	6	160	5	75	1	38	24	44	1	87	12	116	1
Solyc03g116810.2.1	5	733	39	474	6	326	243	1161	89	1675	73	956	2
Solyc03g116820.2.1	5	23	1	20	1	32	17	36	3	42	8	19	0
Solyc03g116830.2.1	2	11	0	10	0	28	20	28	1	19	3	14	0
Solyc03g116840.1.1	0	0	0	0	0	0	0	0	0	0	0	0	0
Solyc03g116850.2.1	0	0	0	0	0	0	0	0	0	0	0	0	0
Solyc03g116860.2.1	0	0	0	0	0	0	0	0	0	0	0	0	0
Solyc03g116870.2.1	6	160	12	82	0	8	20	24	1	33	7	23	2
Solyc03g116880.2.1	1	6	0	0	0	1	3	5	0	18	0	8	0
Solyc03g116890.2.1	2	31	2	16	0	84	0	8	1	6	7	8	0
Solyc03g116900.2.1	6	34	6	29	0	612	108	158	6	61	63	52	0
Solyc03g116910.2.1	0	0	0	0	0	0	0	0	0	0	0	0	0
Solyc03g116920.1.1	0	0	0	0	0	0	0	0	0	0	0	0	0
Solyc03g116930.2.1	4	11	5	13	0	51	93	78	17	35	19	64	2
Solyc03g116950.1.1	0	0	0	0	0	0	0	0	0	0	0	0	0
Solyc03g116970.1.1	0	0	0	0	0	0	0	0	0	0	0	0	0
Solyc03g116990.1.1	0	0	0	0	0	0	0	0	0	0	0	0	0
Solyc03g117000.2.1	0	0	0	0	0	0	0	0	0	0	0	0	0
Solyc03g117010.2.1	9	34	5	19	1	48	48	49	0	72	10	67	0
Solyc03g117020.2.1	5	95	6	59	8	340	86	305	29	133	42	54	1
Solyc03g117030.1.1	2	28	3	23	6	59	41	39	3	21	11	23	1
Solyc03g117040.2.1	5	143	8	73	17	120	49	240	21	254	45	242	0
Solyc03g117050.2.1	4	64	6	36	0	38	19	44	4	185	13	130	1
Solyc03g117060.2.1	8	74	6	39	5	184	76	189	17	245	52	240	10
Solyc03g117070.1.1	0	0	0	0	0	0	0	0	0	0	0	0	0
Solyc03g117080.2.1	1	9	0	0	0	23	1	23	0	16	0	12	0
Solyc03g117090.2.1	1	0	0	1	0	2	2	1	0	5	2	6	0

Solyc03g117100.2.1	0	0	0	0	0	0	0	0	0	0	0	0	0
Solyc03g117110.2.1	1	3	0	3	0	5	4	9	1	24	1	26	0
Solyc03g117120.2.1	16	58	4	15	0	99	64	135	14	111	45	114	1
Solyc03g117130.2.1	0	0	0	0	0	0	0	0	0	0	0	0	0
Solyc03g117150.2.1	0	0	0	0	0	0	0	0	0	0	0	0	0
Solyc03g117170.1.1	0	0	0	0	0	0	0	0	0	0	0	0	0
Solyc03g117180.2.1	3	13	1	14	1	2	8	2	1	12	13	21	0
Solyc03g117190.1.1	0	0	0	0	0	0	0	0	0	0	0	0	0
Solyc03g117200.2.1	4	14	0	9	0	32	31	18	10	9	6	6	0
Solyc03g117210.2.1	2	5	2	0	0	8	14	7	0	0	0	8	0
Solyc03g117250.2.1	5	112	16	24	0	9	16	24	0	0	3	0	0
Solyc03g117260.1.1	0	0	0	0	0	0	0	0	0	0	0	0	0
Solyc03g117270.1.1	10	109	18	59	14	59	31	16	8	22	6	18	1
Solyc03g117280.2.1	12	172	6	121	0	241	83	234	5	329	45	652	5
Solyc03g117290.1.1	3	7	2	2	0	74	37	95	13	21	3	17	0
Solyc03g117300.2.1	3	10	2	7	0	14	16	36	3	86	6	98	2
Solyc03g117310.1.1	0	0	0	0	0	0	0	0	0	0	0	0	0
Solyc03g117320.2.1	2	17	0	6	0	24	6	18	2	25	6	31	0
Solyc03g117330.2.1	1	0	4	0	0	2	9	7	0	2	0	0	0
Solyc03g117340.1.1	1	10	0	1	0	12	3	1	0	5	1	6	0
Solyc03g117350.1.1	3	5	1	11	0	5	14	16	3	4	0	12	0
Solyc03g117360.2.1	13	220	42	161	15	135	223	307	45	277	23	304	4
Solyc03g117370.2.1	4	5	2	3	0	20	20	22	1	10	6	2	0
Solyc03g117380.2.1	0	0	0	0	0	0	0	0	0	0	0	0	0
Solyc03g117390.1.1	0	0	0	0	0	0	0	0	0	0	0	0	0
Solyc03g117400.2.1	1	4	1	0	0	1	3	4	0	1	2	1	2
Solyc03g117410.2.1	0	0	0	0	0	0	0	0	0	0	0	0	0
Solyc03g117420.2.1	9	44	28	83	20	528	699	410	62	725	95	402	9
Solyc03g117430.2.1	16	268	34	245	4	446	642	695	33	442	114	309	12
Solyc03g117440.2.1	5	36	4	14	0	52	36	47	1	14	4	37	0
Solyc03g117470.2.1	2	28	3	6	0	42	6	49	1	125	6	24	0
Solyc03g117480.2.1	9	48	13	54	7	203	197	401	66	308	404	167	4
Solyc03g117490.2.1	13	80	4	39	7	137	240	286	45	216	12	75	1
Solyc03g117500.1.1	1	11	0	6	0	17	1	13	1	19	9	9	0
Solyc03g117510.2.1	0	0	0	0	0	0	0	0	0	0	0	0	0
Solyc03g117520.1.1	4	22	2	20	0	13	19	21	1	54	10	25	0
Solyc03g117530.1.1	2	9	2	1	0	3	0	9	1	17	11	12	0
Solyc03g117540.2.1	4	4	6	11	1	24	39	24	0	26	7	50	0
Solyc03g117550.1.1	0	0	0	0	0	0	0	0	0	0	0	0	0
Solyc03g117560.2.1	0	0	0	0	0	0	0	0	0	0	0	0	0
Solyc03g117570.2.1	0	0	0	0	0	0	0	0	0	0	0	0	0

Solyc03g117580.2.1	1	1	0	0	0	3	5	2	1	8	1	2	0
Solyc03g117590.2.1	7	1108	45	991	18	450	162	659	9	63	38	58	1
Solyc03g117600.2.1	14	181	23	90	4	237	236	366	163	30	111	24	1
Solyc03g117610.1.1	0	0	0	0	0	0	0	0	0	0	0	0	0
Solyc03g117630.1.1	1	1	0	4	0	0	0	1	0	4	10	1	0
Solyc03g117640.1.1	0	0	0	0	0	0	0	0	0	0	0	0	0
Solyc03g117650.2.1	1	5	0	0	0	14	4	14	0	6	0	5	0
Solyc03g117660.2.1	3	1	0	0	0	7	5	12	2	10	5	8	0
Solyc03g117670.2.1	7	94	12	47	0	90	76	116	10	104	12	60	2
Solyc03g117680.2.1	19	167	16	143	6	226	239	433	30	195	58	133	1
Solyc03g117690.2.1	1	12	3	3	0	1	0	4	0	1	5	0	0
Solyc03g117720.2.1	1	2	2	10	4	16	10	10	11	10	8	0	0
Solyc03g117730.2.1	0	0	0	0	0	0	0	0	0	0	0	0	0
Solyc03g117740.2.1	4	123	13	72	0	54	41	17	3	47	8	86	0
Solyc03g117750.2.1	1	2	3	3	0	1	1	7	0	4	0	4	0
Solyc03g117760.2.1	1	23	0	22	0	13	6	4	0	18	0	13	0
Solyc03g117770.2.1	11	1120	80	714	40	3281	1385	3481	266	2062	425	1327	33
Solyc03g117780.2.1	4	39	2	13	0	49	20	48	8	55	5	39	0
Solyc03g117790.1.1	0	0	0	0	0	0	0	0	0	0	0	0	0
Solyc03g117800.2.1	17	94	1	3	3	1097	394	196	543	20	7	1	4
Solyc03g117810.2.1	4	55	11	28	0	40	34	51	0	109	6	163	0
Solyc03g117820.1.1	2	6	0	5	0	2	12	8	0	4	1	1	0
Solyc03g117830.1.1	0	0	0	0	0	0	0	0	0	0	0	0	0
Solyc03g117840.2.1	0	0	0	0	0	0	0	0	0	0	0	0	0
Solyc03g117850.2.1	5	161	21	70	0	58	40	52	0	43	17	40	0
Solyc03g117860.2.1	0	0	0	0	0	0	0	0	0	0	0	0	0
Solyc03g117870.2.1	24	578	129	405	34	787	432	846	160	590	398	364	21
Solyc03g117890.2.1	9	759	142	392	20	3787	3722	1737	19	167	68	218	6
Solyc03g117900.2.1	0	0	0	0	0	0	0	0	0	0	0	0	0
Solyc03g117910.2.1	0	0	0	0	0	0	0	0	0	0	0	0	0
Solyc03g117930.2.1	5	10	0	7	0	8	19	15	7	29	12	14	0
Solyc03g117940.2.1	6	86	12	71	5	417	161	288	19	325	53	217	2
Solyc03g117950.2.1	23	338	51	230	1	1456	945	1383	94	212	50	127	7
Solyc03g117960.2.1	0	0	0	0	0	0	0	0	0	0	0	0	0
Solyc03g117970.2.1	3	7	0	4	0	5	4	12	2	34	14	3	0
Solyc03g117980.2.1	16	703	308	876	39	293	175	330	44	407	63	675	20
Solyc03g117990.1.1	0	0	0	0	0	0	0	0	0	0	0	0	0
Solyc03g118000.1.1	0	0	0	0	0	0	0	0	0	0	0	0	0
Solyc03g118010.2.1	14	106	13	50	17	200	131	219	33	157	68	93	1
Solyc03g118020.2.1	15	420	29	252	13	1713	247	2022	91	659	246	542	3
Solyc03g118030.2.1	2	9	0	3	0	14	17	30	1	13	0	20	0

Solyc03g118040.2.1	20	1388	127	654	85	3290	974	4051	372	1334	1514	1227	24
Solyc03g118050.2.1	0	0	0	0	0	0	0	0	0	0	0	0	0
Solyc03g118070.2.1	3	10	0	13	0	15	17	17	4	17	9	25	0
Solyc03g118080.2.1	0	0	0	0	0	0	0	0	0	0	0	0	0
Solyc03g118090.2.1	0	0	0	0	0	0	0	0	0	0	0	0	0
Solyc03g118100.2.1	0	0	0	0	0	0	0	0	0	0	0	0	0
Solyc03g118110.2.1	1	15	5	5	6	36	25	23	1	92	15	136	4
Solyc03g118120.2.1	7	51	2	23	0	5	15	1	0	265	19	189	1
Solyc03g118130.2.1	5	547	157	350	12	707	457	231	24	194	197	282	9
Solyc03g118140.2.1	0	0	0	0	0	0	0	0	0	0	0	0	0
Solyc03g118150.2.1	3	18	1	10	2	8	12	6	3	10	2	9	0
Solyc03g118160.1.1	0	0	0	0	0	0	0	0	0	0	0	0	0
Solyc03g118170.2.1	9	222	16	154	4	333	416	406	28	546	68	417	7
Solyc03g118180.2.1	0	0	0	0	0	0	0	0	0	0	0	0	0
Solyc03g118190.2.1	0	0	0	0	0	0	0	0	0	0	0	0	0
Solyc03g118200.2.1	2	7	0	5	0	13	2	13	0	10	5	8	0
Solyc03g118210.2.1	0	0	0	0	0	0	0	0	0	0	0	0	0
Solyc03g118220.1.1	0	0	0	0	0	0	0	0	0	0	0	0	0
Solyc03g118240.2.1	7	245	19	130	0	18	50	42	1	50	20	68	1
Solyc03g118250.2.1	1	4	1	1	0	7	5	7	0	13	2	10	0
Solyc03g118260.1.1	0	0	0	0	0	0	0	0	0	0	0	0	0
Solyc03g118270.1.1	9	147	11	116	4	753	69	310	11	280	73	248	0
Solyc03g118280.1.1	1	4	0	1	0	12	6	7	0	2	2	11	0
Solyc03g118290.2.1	20	631	50	543	17	954	471	1391	50	913	111	751	19
Solyc03g118300.1.1	2	22	2	34	2	26	26	40	3	17	2	36	0
Solyc03g118310.2.1	0	0	0	0	0	0	0	0	0	0	0	0	0
Solyc03g118320.1.1	0	0	0	0	0	0	0	0	0	0	0	0	0
Solyc03g118330.1.1	0	0	0	0	0	0	0	0	0	0	0	0	0
Solyc03g118340.2.1	21	113	19	74	8	1527	478	659	20	207	141	117	3
Solyc03g118350.2.1	2	10	0	12	0	9	4	21	0	15	3	14	0
Solyc03g118370.2.1	0	0	0	0	0	0	0	0	0	0	0	0	0
Solyc03g118380.1.1	4	31	7	14	5	23	15	31	4	74	16	31	0
Solyc03g118390.2.1	17	134	17	77	3	548	193	457	23	159	55	169	3
Solyc03g118410.2.1	4	94	11	41	0	6	11	10	0	45	23	85	0
Solyc03g118420.2.1	22	179	27	161	33	1192	546	691	104	229	69	131	2
Solyc03g118430.2.1	21	372	104	327	11	500	470	822	35	298	63	221	13
Solyc03g118450.1.1	0	0	0	0	0	0	0	0	0	0	0	0	0
Solyc03g118470.2.1	1	11	3	2	3	0	2	1	3	2	0	5	0
Solyc03g118480.2.1	0	0	0	0	0	0	0	0	0	0	0	0	0
Solyc03g118490.2.1	0	0	0	0	0	0	0	0	0	0	0	0	0
Solyc03g118500.2.1	4	5	0	2	1	18	20	8	7	27	0	8	2

Solyc03g118510.2.1	9	81	4	73	19	2	6	11	9	269	78	172	57
Solyc03g118520.2.1	1	2	1	0	11	2	7	10	6	6	1	3	0
Solyc03g118530.2.1	11	57	4	43	0	106	125	199	3	109	21	69	2
Solyc03g118540.2.1	3	12	4	11	5	8	51	11	12	1	8	1	0
Solyc03g118550.2.1	6	90	25	9	0	42	117	57	12	99	27	51	7
Solyc03g118570.2.1	2	0	1	2	0	16	9	8	0	8	3	13	0
Solyc03g118610.2.1	4	16	2	16	0	49	18	40	5	17	7	44	1
Solyc03g118620.2.1	26	133	30	111	7	345	217	334	67	136	31	86	2
Solyc03g118630.2.1	0	0	0	0	0	0	0	0	0	0	0	0	0
Solyc03g118640.2.1	17	36	7	45	6	101	233	108	26	296	61	222	14
Solyc03g118650.2.1	7	93	2	48	5	182	84	243	8	284	43	165	4
Solyc03g118660.2.1	2	12	0	4	2	46	24	28	0	25	7	21	0
Solyc03g118670.2.1	18	86	12	66	25	202	137	268	46	167	83	163	5
Solyc03g118680.2.1	8	28	1	20	5	37	30	66	16	46	15	45	3
Solyc03g118690.2.1	11	94	8	73	8	248	288	434	45	110	61	164	3
Solyc03g118700.2.1	1	0	0	0	0	0	0	1	0	4	4	7	0
Solyc03g118710.2.1	3	186	10	61	8	753	49	157	35	129	21	96	1
Solyc03g118720.2.1	0	0	0	0	0	0	0	0	0	0	0	0	0
Solyc03g118740.2.1	1	14	0	11	0	17	0	7	0	8	1	8	0
Solyc03g118750.2.1	1	2	0	6	0	4	5	3	0	3	0	11	0
Solyc03g118760.2.1	0	0	0	0	0	0	0	0	0	0	0	0	0
Solyc03g118770.2.1	0	0	0	0	0	0	0	0	0	0	0	0	0
Solyc03g118780.2.1	0	0	0	0	0	0	0	0	0	0	0	0	0
Solyc03g118790.1.1	2	0	0	0	0	20	10	20	1	5	0	2	0
Solyc03g118800.2.1	10	188	16	94	8	436	167	522	48	395	46	231	0
Solyc03g118810.1.1	2	207	31	81	22	693	34	319	12	6	1	53	2
Solyc03g118820.2.1	2	8	1	6	0	40	7	31	2	36	0	18	0
Solyc03g118830.2.1	2	12	2	3	0	5	8	2	8	10	0	4	3
Solyc03g118840.2.1	6	202	12	119	1	152	18	183	7	394	28	235	1
Solyc03g118850.2.1	0	0	0	0	0	0	0	0	0	0	0	0	0
Solyc03g118860.2.1	6	12	2	26	0	17	16	25	4	48	8	44	2
Solyc03g118870.2.1	0	0	0	0	0	0	0	0	0	0	0	0	0
Solyc03g118890.2.1	6	23	2	14	0	60	24	38	2	18	3	22	1
Solyc03g118910.1.1	13	131	13	90	2	262	106	456	46	347	85	284	4
Solyc03g118920.2.1	17	77	16	64	5	176	97	212	20	180	30	114	1
Solyc03g118960.1.1	0	0	0	0	0	0	0	0	0	0	0	0	0
Solyc03g118970.2.1	7	60	18	40	0	125	38	50	3	55	7	52	0
Solyc03g118980.2.1	0	0	0	0	0	0	0	0	0	0	0	0	0
Solyc03g118990.1.1	0	0	0	0	0	0	0	0	0	0	0	0	0
Solyc03g119010.2.1	2	133	10	66	1	96	70	86	20	495	103	307	26
Solyc03g119020.2.1	0	0	0	0	0	0	0	0	0	0	0	0	0

Solyc03g119030.2.1	12	68	5	48	10	188	88	151	12	114	16	73	10
Solyc03g119040.2.1	4	104	22	60	12	174	76	271	29	250	128	271	4
Solyc03g119050.2.1	2	2	0	13	5	4	6	5	2	9	3	5	0
Solyc03g119060.2.1	15	245	37	266	10	158	458	676	9	266	25	219	5
Solyc03g119070.2.1	0	0	0	0	0	0	0	0	0	0	0	0	0
Solyc03g119080.2.1	18	667	74	433	2	140	204	440	0	1815	63	1219	1
Solyc03g119090.2.1	14	83	9	101	5	208	81	369	30	363	79	318	3
Solyc03g119110.2.1	2	35	3	9	0	1	3	11	0	12	0	9	0
Solyc03g119120.2.1	0	0	0	0	0	0	0	0	0	0	0	0	0
Solyc03g119130.2.1	22	769	41	463	14	792	313	1169	62	778	179	580	16
Solyc03g119140.2.1	12	137	19	141	1	101	97	160	17	177	23	107	4
Solyc03g119150.2.1	6	249	103	222	5	5	136	32	1	53	8	43	0
Solyc03g119160.2.1	2	8	0	2	1	7	1	11	0	6	6	5	0
Solyc03g119170.2.1	3	11	0	13	0	48	17	48	11	33	13	20	0
Solyc03g119180.1.1	0	0	0	0	0	0	0	0	0	0	0	0	0
Solyc03g119190.2.1	0	0	0	0	0	0	0	0	0	0	0	0	0
Solyc03g119200.2.1	1	2	0	10	0	17	4	40	0	13	0	8	0
Solyc03g119210.1.1	1	49	1	9	0	41	12	42	0	3	0	1	0
Solyc03g119220.2.1	0	0	0	0	0	0	0	0	0	0	0	0	0
Solyc03g119230.1.1	0	0	0	0	0	0	0	0	0	0	0	0	0
Solyc03g119240.2.1	0	0	0	0	0	0	0	0	0	0	0	0	0
Solyc03g119250.2.1	9	309	87	187	35	37	3	64	4	22	0	181	31
Solyc03g119260.2.1	0	0	0	0	0	0	0	0	0	0	0	0	0
Solyc03g119270.1.1	9	48	3	48	3	98	36	89	0	101	4	89	0
Solyc03g119280.2.1	4	38	14	12	2	38	44	48	0	21	16	32	0
Solyc03g119290.2.1	5	39	4	6	1	44	43	47	6	60	6	79	0
Solyc03g119300.2.1	14	47	4	56	0	198	94	238	7	23	13	22	0
Solyc03g119310.2.1	0	0	0	0	0	0	0	0	0	0	0	0	0
Solyc03g119320.1.1	0	0	0	0	0	0	0	0	0	0	0	0	0
Solyc03g119330.2.1	7	76	8	22	5	136	103	130	22	193	30	66	0
Solyc03g119340.2.1	1	6	5	5	1	8	2	21	0	4	1	9	0
Solyc03g119350.1.1	0	0	0	0	0	0	0	0	0	0	0	0	0
Solyc03g119360.2.1	4	139	3	49	4	282	66	252	12	377	91	377	5
Solyc03g119370.1.1	0	0	0	0	0	0	0	0	0	0	0	0	0
Solyc03g119380.1.1	2	26	0	16	0	29	10	22	3	22	3	56	0
Solyc03g119390.2.1	3	5	2	3	0	6	20	4	0	0	0	0	0
Solyc03g119400.2.1	14	77	4	75	7	104	105	326	30	255	32	246	2
Solyc03g119420.2.1	1	5	0	2	0	8	4	6	0	4	2	7	1
Solyc03g119430.2.1	2	5	0	8	0	14	9	11	0	17	3	9	0
Solyc03g119440.2.1	13	85	14	55	10	165	93	178	13	65	10	68	5
Solyc03g119450.2.1	19	128	14	31	2	125	210	254	11	151	40	102	1

Solyc03g119460.2.1	7	43	9	40	0	111	80	165	15	159	24	117	2
Solyc03g119470.1.1	0	0	0	0	0	0	0	0	0	0	0	0	0
Solyc03g119480.2.1	5	18	0	14	1	18	21	25	6	61	20	23	2
Solyc03g119490.2.1	0	0	0	0	0	0	0	0	0	0	0	0	0
Solyc03g119500.1.1	0	0	0	0	0	0	0	0	0	0	0	0	0
Solyc03g119510.2.1	0	0	0	0	0	0	0	0	0	0	0	0	0
Solyc03g119520.2.1	7	113	7	37	0	75	202	77	9	88	37	80	11
Solyc03g119530.2.1	7	53	4	19	4	112	81	25	2	396	81	276	16
Solyc03g119540.2.1	12	349	34	328	0	173	70	97	4	40	10	80	1
Solyc03g119550.1.1	0	0	0	0	0	0	0	0	0	0	0	0	0
Solyc03g119570.1.1	2	6	1	2	0	16	9	3	0	2	2	6	0
Solyc03g119580.1.1	1	0	2	0	0	8	1	3	0	2	1	2	0
Solyc03g119590.1.1	0	0	0	0	0	0	0	0	0	0	0	0	0
Solyc03g119600.1.1	2	18	4	1	0	5	2	1	0	49	4	36	1
Solyc03g119610.1.1	13	299	34	123	21	935	609	693	319	1106	82	386	10
Solyc03g119630.2.1	0	0	0	0	0	0	0	0	0	0	0	0	0
Solyc03g119640.2.1	4	10	1	35	0	44	21	30	1	2	0	13	1
Solyc03g119650.2.1	14	115	13	65	10	344	142	360	17	212	32	179	1
Solyc03g119660.1.1	1	0	0	0	0	6	5	10	0	1	0	0	0
Solyc03g119680.1.1	0	0	0	0	0	0	0	0	0	0	0	0	0
Solyc03g119690.1.1	17	833	56	464	189	109	282	342	124	1753	227	926	83
Solyc03g119700.2.1	2	172	40	95	2	27	135	38	7	64	15	77	8
Solyc03g119710.2.1	1	11	0	3	0	9	7	9	0	15	0	9	0
Solyc03g119720.2.1	11	19	6	5	0	28	117	51	10	35	21	40	2
Solyc03g119730.2.1	7	23	9	20	1	23	21	53	5	50	26	45	5
Solyc03g119740.2.1	1	11	0	6	0	6	0	6	0	1	0	1	0
Solyc03g119750.2.1	7	50	6	27	7	62	58	73	6	71	12	31	0
Solyc03g119760.2.1	6	24	4	23	0	33	18	36	0	41	5	27	0
Solyc03g119770.2.1	0	0	0	0	0	0	0	0	0	0	0	0	0
Solyc03g119780.2.1	24	109	17	77	4	200	271	249	51	268	80	292	10
Solyc03g119790.2.1	5	16	2	22	0	26	32	56	8	23	10	40	0
Solyc03g119810.1.1	1	0	0	2	0	50	6	8	0	2	1	1	0
Solyc03g119820.1.1	0	0	0	0	0	0	0	0	0	0	0	0	0
Solyc03g119830.2.1	1	1	0	1	0	4	3	13	2	1	1	0	0
Solyc03g119840.2.1	0	0	0	0	0	0	0	0	0	0	0	0	0
Solyc03g119850.1.1	0	0	0	0	0	0	0	0	0	0	0	0	0
Solyc03g119860.2.1	2	55	6	30	0	1	4	0	0	6	3	2	0
Solyc03g119870.2.1	8	21	2	5	5	42	30	53	2	38	18	23	1
Solyc03g119880.2.1	0	0	0	0	0	0	0	0	0	0	0	0	0
Solyc03g119890.2.1	3	23	1	9	1	23	22	16	2	19	4	15	0
Solyc03g119920.1.1	10	27	1	16	5	35	75	49	6	72	30	59	0

Solyc03g119930.1.1	7	81	11	38	4	53	26	43	4	42	0	58	2
Solyc03g119940.2.1	0	0	0	0	0	0	0	0	0	0	0	0	0
Solyc03g119950.1.1	0	0	0	0	0	0	0	0	0	0	0	0	0
Solyc03g119960.2.1	0	0	0	0	0	0	0	0	0	0	0	0	0
Solyc03g119970.2.1	10	148	5	82	4	561	75	393	15	94	0	26	0
Solyc03g119980.2.1	11	89	11	67	8	247	167	199	102	109	150	66	8
Solyc03g119990.2.1	0	0	0	0	0	0	0	0	0	0	0	0	0
Solyc03g120000.2.1	9	11	4	13	0	46	42	36	3	64	12	44	1
Solyc03g120010.2.1	10	359	85	292	26	717	630	577	81	975	253	818	19
Solyc03g120020.2.1	1	5	3	0	0	0	3	2	2	0	0	0	0
Solyc03g120040.2.1	2	8	0	0	0	20	5	14	0	5	14	5	4
Solyc03g120060.2.1	0	0	0	0	0	0	0	0	0	0	0	0	0
Solyc03g120070.2.1	1	6	1	2	3	12	21	0	4	11	9	0	1
Solyc03g120080.2.1	2	8	1	8	0	10	6	5	0	12	2	3	0
Solyc03g120090.1.1	5	94	3	38	0	55	25	64	0	177	16	178	2
Solyc03g120100.2.1	0	0	0	0	0	0	0	0	0	0	0	0	0
Solyc03g120110.2.1	0	0	0	0	0	0	0	0	0	0	0	0	0
Solyc03g120120.2.1	5	8	0	3	0	15	23	4	1	20	2	36	0
Solyc03g120130.2.1	0	0	0	0	0	0	0	0	0	0	0	0	0
Solyc03g120140.2.1	1	0	1	3	0	2	4	7	1	5	0	1	0
Solyc03g120150.2.1	8	22	0	12	0	30	42	50	2	37	12	40	0
Solyc03g120160.1.1	3	4	0	15	0	0	6	0	1	0	5	2	2
Solyc03g120170.1.1	0	0	0	0	0	0	0	0	0	0	0	0	0
Solyc03g120180.1.1	0	0	0	0	0	0	0	0	0	0	0	0	0
Solyc03g120190.2.1	0	0	0	0	0	0	0	0	0	0	0	0	0
Solyc03g120200.1.1	0	0	0	0	0	0	0	0	0	0	0	0	0
Solyc03g120230.2.1	7	20	13	22	0	31	91	63	2	39	18	35	2
Solyc03g120240.2.1	3	24	4	9	6	70	76	44	13	46	6	41	0
Solyc03g120250.2.1	0	0	0	0	0	0	0	0	0	0	0	0	0
Solyc03g120260.2.1	5	19	0	11	0	212	18	106	41	11	0	0	0
Solyc03g120270.2.1	7	108	6	57	8	266	95	309	35	272	44	210	1
Solyc03g120280.1.1	27	100	24	86	19	335	317	470	77	318	114	284	14
Solyc03g120290.2.1	0	0	0	0	0	0	0	0	0	0	0	0	0
Solyc03g120300.2.1	15	126	14	86	5	216	86	221	32	196	44	153	1
Solyc03g120310.2.1	11	38	5	27	8	29	48	33	16	354	64	321	7
Solyc03g120320.1.1	3	23	3	9	0	16	0	8	6	13	10	4	0
Solyc03g120330.1.1	0	0	0	0	0	0	0	0	0	0	0	0	0
Solyc03g120340.2.1	1	2	1	1	0	4	7	2	0	6	4	11	0
Solyc03g120350.2.1	8	47	8	45	1	91	35	135	11	138	20	177	4
Solyc03g120360.1.1	0	0	0	0	0	0	0	0	0	0	0	0	0
Solyc03g120380.2.1	1	7	1	1	4	0	5	0	0	7	0	1	0

Solyc03g120390.2.1	3	5	1	1	0	171	60	100	9	499	21	228	8
Solyc03g120410.1.1	2	13	1	8	6	4	16	7	3	10	1	12	0
Solyc03g120420.2.1	2	3	17	3	3	45	346	61	22	166	273	91	5
Solyc03g120430.2.1	9	501	54	350	1	115	241	90	5	218	29	199	9
Solyc03g120450.2.1	8	154	7	51	8	283	200	267	38	123	24	83	6
Solyc03g120460.2.1	4	1	0	4	0	17	10	5	0	3	1	25	0
Solyc03g120470.2.1	18	1606	46	461	44	175	211	354	53	93	18	114	0
Solyc03g120480.2.1	2	9	1	7	1	8	18	36	3	20	3	12	0
Solyc03g120490.2.1	9	46	10	29	4	87	62	137	7	117	28	100	2
Solyc03g120500.2.1	7	41	4	35	1	32	34	61	4	206	35	166	8
Solyc03g120510.1.1	5	40	2	34	0	30	31	50	2	86	9	50	1
Solyc03g120520.1.1	0	0	0	0	0	0	0	0	0	0	0	0	0
Solyc03g120530.2.1	0	0	0	0	0	0	0	0	0	0	0	0	0
Solyc03g120540.2.1	3	13	2	11	0	50	22	36	0	27	8	33	0
Solyc03g120550.2.1	18	826	57	367	32	1023	288	784	82	317	119	300	18
Solyc03g120560.2.1	7	34	0	25	0	29	4	60	0	66	29	72	0
Solyc03g120570.2.1	0	0	0	0	0	0	0	0	0	0	0	0	0
Solyc03g120590.2.1	0	0	0	0	0	0	0	0	0	0	0	0	0
Solyc03g120610.2.1	0	0	0	0	0	0	0	0	0	0	0	0	0
Solyc03g120620.2.1	10	55	0	32	5	17	24	48	18	110	22	110	0
Solyc03g120630.2.1	2	349	16	143	6	152	120	116	2	411	12	240	0
Solyc03g120640.2.1	4	1689	90	807	5	95	147	118	7	369	101	235	2
Solyc03g120650.2.1	0	0	0	0	0	0	0	0	0	0	0	0	0
Solyc03g120660.2.1	0	0	0	0	0	0	0	0	0	0	0	0	0
Solyc03g120670.2.1	14	339	44	185	7	89	262	201	33	247	121	235	12
Solyc03g120680.2.1	0	0	0	0	0	0	0	0	0	0	0	0	0
Solyc03g120690.2.1	0	0	0	0	0	0	0	0	0	0	0	0	0
Solyc03g120700.2.1	14	54	6	26	3	69	37	124	7	113	26	93	0
Solyc03g120710.2.1	4	188	5	43	7	53	27	138	19	530	26	442	7
Solyc03g120720.2.1	12	53	4	32	15	143	83	155	33	124	20	122	2
Solyc03g120730.2.1	13	74	11	70	11	133	350	87	53	233	27	205	6
Solyc03g120740.1.1	2	11	2	8	1	29	45	15	8	72	41	28	1
Solyc03g120750.2.1	4	106	4	36	1	41	50	68	12	76	26	59	2
Solyc03g120760.2.1	5	14	5	8	4	19	157	32	24	25	42	13	3
Solyc03g120770.2.1	5	16	0	6	0	67	42	76	11	22	5	29	4
Solyc03g120780.2.1	1	3	5	0	4	60	10	16	6	13	5	15	0
Solyc03g120790.1.1	0	0	0	0	0	0	0	0	0	0	0	0	0
Solyc03g120810.2.1	3	12	3	14	0	9	10	15	1	12	3	17	0
Solyc03g120820.2.1	1	4	0	2	0	6	13	0	0	6	8	4	0
Solyc03g120830.2.1	0	0	0	0	0	0	0	0	0	0	0	0	0
Solyc03g120840.1.1	3	19	1	2	0	118	18	72	1	16	7	6	0

Solyc03g120850.2.1	11	217	26	110	5	189	206	329	4	155	52	252	6
Solyc03g120870.2.1	0	0	0	0	0	0	0	0	0	0	0	0	0
Solyc03g120880.2.1	11	57	4	38	4	87	116	171	14	217	57	158	1
Solyc03g120890.2.1	3	26	3	6	0	4	6	1	0	53	8	27	0
Solyc03g120900.1.1	7	12	2	15	0	168	45	168	14	0	20	2	6
Solyc03g120910.2.1	9	40	2	36	0	33	23	185	0	136	8	84	3
Solyc03g120930.1.1	0	0	0	0	0	0	0	0	0	0	0	0	0
Solyc03g120940.2.1	1	15	2	1	0	1	8	8	0	22	0	7	0
Solyc03g120970.2.1	0	0	0	0	0	0	0	0	0	0	0	0	0
Solyc03g120980.2.1	23	97	19	77	0	984	509	1600	357	118	29	50	0
Solyc03g120990.2.1	0	0	0	0	0	0	0	0	0	0	0	0	0
Solyc03g121010.2.1	0	0	0	0	0	0	0	0	0	0	0	0	0
Solyc03g121020.2.1	11	114	7	106	0	300	139	351	29	169	29	83	1
Solyc03g121030.1.1	2	5	1	0	3	7	10	10	2	21	0	44	6
Solyc03g121040.2.1	10	5	3	3	0	46	52	91	11	61	23	34	1
Solyc03g121050.2.1	6	32	2	15	0	29	22	25	1	238	48	193	1
Solyc03g121060.2.1	3	23	0	10	0	15	8	38	0	1	2	11	0
Solyc03g121070.2.1	2	56	3	33	1	57	32	60	9	91	15	29	0
Solyc03g121080.2.1	0	0	0	0	0	0	0	0	0	0	0	0	0
Solyc03g121090.2.1	5	203	147	327	46	157	709	264	21	123	9	275	22
Solyc03g121100.2.1	1	3	3	0	0	1	2	4	2	0	0	0	0
Solyc03g121110.2.1	1	5	0	0	0	9	18	4	0	15	0	7	0
Solyc03g121120.2.1	3	10	2	7	0	7	31	16	2	9	7	10	0
Solyc03g121130.2.1	1	5	1	0	0	0	4	4	1	2	0	6	0
Solyc03g121140.2.1	0	0	0	0	0	0	0	0	0	0	0	0	0
Solyc03g121170.2.1	0	0	0	0	0	0	0	0	0	0	0	0	0
Solyc03g121180.2.1	12	530	26	6	65	96	121	11	204	101	214	20	206
Solyc03g121210.2.1	1	1	1	1	0	5	12	0	0	4	1	4	0
Solyc03g121240.1.1	3	2	1	0	2	8	37	5	2	0	0	2	0
Solyc03g121250.2.1	1	0	0	0	0	0	0	1	0	15	0	5	0
Solyc03g121260.2.1	2	10	1	7	0	2	14	4	0	5	0	2	0
Solyc03g121270.2.1	8	84	9	39	2	127	162	179	12	238	42	272	0
Solyc03g121280.2.1	2	3	0	0	0	43	8	35	1	16	1	15	0
Solyc03g121290.2.1	8	31	4	23	0	111	61	84	11	38	28	67	0
Solyc03g121300.2.1	2	67	3	40	0	19	2	56	0	47	4	26	0
Solyc03g121310.2.1	6	67	6	43	7	102	104	156	14	248	18	222	3
Solyc03g121320.2.1	3	9	8	10	0	8	20	17	3	23	5	14	0
Solyc03g121330.2.1	2	74	5	48	5	168	49	167	26	227	70	201	0
Solyc03g121340.1.1	0	0	0	0	0	0	0	0	0	0	0	0	0
Solyc03g121350.2.1	0	0	0	0	0	0	0	0	0	0	0	0	0
Solyc03g121360.1.1	6	266	27	165	10	241	73	132	10	328	36	368	13

Solyc03g121370.2.1	2	21	2	12	1	27	12	42	6	79	5	46	0
Solyc03g121380.2.1	4	7	0	11	0	16	7	13	0	14	8	18	0
Solyc03g121400.1.1	2	13	6	12	2	56	8	13	1	1	5	20	0
Solyc03g121410.2.1	6	24	1	11	1	117	52	108	7	38	18	25	1
Solyc03g121420.2.1	2	5	4	2	0	33	10	14	0	0	0	0	0
Solyc03g121440.2.1	6	58	5	31	0	9	11	39	1	79	9	23	0
Solyc03g121450.2.1	2	1	1	0	1	13	16	5	3	8	2	16	0
Solyc03g121460.1.1	8	118	11	21	6	143	32	114	60	194	29	87	0
Solyc03g121480.2.1	6	58	12	50	1	37	221	61	15	133	24	68	9
Solyc03g121490.2.1	0	0	0	0	0	0	0	0	0	0	0	0	0
Solyc03g121500.2.1	5	30	1	23	2	78	63	52	12	93	17	100	0
Solyc03g121510.2.1	0	0	0	0	0	0	0	0	0	0	0	0	0
Solyc03g121520.2.1	2	1	0	0	0	0	6	3	0	4	0	6	0
Solyc03g121530.2.1	11	84	4	48	0	75	90	117	1	60	14	51	2
Solyc03g121540.2.1	12	10	3	14	0	60	1688	197	9	52	88	36	1
Solyc03g121550.2.1	0	0	0	0	0	0	0	0	0	0	0	0	0
Solyc03g121560.2.1	2	8	3	4	0	5	16	18	1	21	2	2	0
Solyc03g121570.2.1	8	34	2	28	7	86	74	152	13	91	18	85	0
Solyc03g121580.2.1	5	32	6	12	9	18	53	36	10	59	28	48	4
Solyc03g121590.2.1	18	186	16	146	8	639	159	692	62	413	56	454	11
Solyc03g121600.2.1	0	0	0	0	0	0	0	0	0	0	0	0	0
Solyc03g121610.2.1	3	6	1	13	0	5	7	7	1	45	4	17	0
Solyc03g121620.1.1	9	248	39	165	33	211	135	63	27	97	90	211	25
Solyc03g121630.2.1	8	14	1	19	0	62	39	75	2	35	14	41	0
Solyc03g121640.2.1	4	11	5	6	0	9	25	46	3	68	12	42	0
Solyc03g121650.2.1	2	9	0	0	0	6	13	40	9	23	3	7	0
Solyc03g121660.2.1	12	454	64	358	1	605	310	475	1	175	107	113	0
Solyc03g121670.2.1	3	10	1	4	0	49	3	13	3	8	1	19	0
Solyc03g121680.1.1	1	2	7	1	46	6	74	0	54	0	1	0	0
Solyc03g121700.2.1	11	37	0	51	6	136	86	184	40	207	49	146	0
Solyc03g121710.2.1	8	121	8	47	1	268	103	221	28	219	35	73	1
Solyc03g121720.2.1	9	151	11	75	3	63	125	69	5	107	18	120	1
Solyc03g121730.2.1	10	107	22	86	2	121	243	195	21	99	26	88	1
Solyc03g121760.2.1	0	0	0	0	0	0	0	0	0	0	0	0	0
Solyc03g121780.1.1	0	0	0	0	0	0	0	0	0	0	0	0	0
Solyc03g121790.2.1	0	0	0	0	0	0	0	0	0	0	0	0	0
Solyc03g121800.2.1	2	15	5	8	0	10	16	10	2	8	0	7	0
Solyc03g121810.2.1	0	0	0	0	0	0	0	0	0	0	0	0	0
Solyc03g121820.2.1	7	58	11	33	3	94	78	94	1	218	35	155	5
Solyc03g121830.1.1	7	50	7	52	8	122	69	155	29	142	30	117	1
Solyc03g121840.2.1	6	3	0	3	0	28	16	22	1	62	11	66	1

Solyc03g121850.2.1	0	0	0	0	0	0	0	0	0	0	0	0	0
Solyc03g121860.1.1	2	45	8	28	2	57	21	41	4	292	16	59	0
Solyc03g121870.2.1	0	0	0	0	0	0	0	0	0	0	0	0	0
Solyc03g121880.2.1	17	350	32	156	41	2401	1568	2407	217	254	88	186	12
Solyc03g121890.1.1	12	210	21	88	18	125	153	49	14	152	44	123	11
Solyc03g121900.1.1	0	0	0	0	0	0	0	0	0	0	0	0	0
Solyc03g121910.1.1	17	530	104	241	21	457	384	534	38	331	103	266	4
Solyc03g121920.2.1	6	19	17	10	12	56	85	44	9	143	23	111	1
Solyc03g121930.2.1	1	4	1	0	0	9	4	10	1	5	0	0	0
Solyc03g121940.2.1	0	0	0	0	0	0	0	0	0	0	0	0	0
Solyc03g121950.2.1	13	89	5	53	14	180	146	163	30	208	50	144	1
Solyc03g121960.2.1	14	638	67	280	10	888	524	1066	129	207	151	133	1
Solyc03g121970.1.1	1	4	0	2	0	12	11	7	0	12	0	8	0
Solyc03g121980.2.1	4	36	6	22	6	85	92	92	6	165	66	204	7
Solyc03g121990.2.1	21	143	16	111	10	457	95	311	32	316	36	229	1
Solyc03g122000.2.1	0	0	0	0	0	0	0	0	0	0	0	0	0
Solyc03g122020.2.1	10	73	17	49	5	116	116	249	25	80	34	76	1
Solyc03g122030.1.1	4	28	2	3	1	19	39	25	4	48	17	20	1
Solyc03g122040.2.1	7	30	10	30	1	41	41	42	5	71	25	50	4
Solyc03g122060.1.1	0	0	0	0	0	0	0	0	0	0	0	0	0
Solyc03g122070.1.1	0	0	0	0	0	0	0	0	0	0	0	0	0
Solyc03g122080.2.1	0	0	0	0	0	0	0	0	0	0	0	0	0
Solyc03g122090.2.1	3	9	0	6	0	49	12	43	8	32	8	42	0
Solyc03g122100.1.1	0	0	0	0	0	0	0	0	0	0	0	0	0
Solyc03g122110.1.1	0	0	0	0	0	0	0	0	0	0	0	0	0
Solyc03g122120.2.1	15	40	2	33	4	191	33	223	5	139	93	118	0
Solyc03g122130.2.1	1	0	0	0	0	1	6	5	1	8	77	0	0
Solyc03g122140.2.1	0	0	0	0	0	0	0	0	0	0	0	0	0
Solyc03g122170.2.1	0	0	0	0	0	0	0	0	0	0	0	0	0
Solyc03g122180.2.1	3	8	1	4	0	19	16	26	3	50	10	42	1
Solyc03g122190.2.1	5	174	18	70	3	59	18	39	2	49	10	43	2
Solyc03g122240.2.1	8	33	3	64	11	119	41	58	12	93	16	63	0
Solyc03g122250.2.1	6	77	7	17	8	20	12	67	1	85	27	90	0
Solyc03g122260.2.1	2	37	6	24	3	68	22	64	5	101	14	56	4
Solyc03g122270.2.1	17	121	8	107	11	212	126	253	16	801	64	417	3
Solyc03g122280.2.1	0	0	0	0	0	0	0	0	0	0	0	0	0
Solyc03g122290.2.1	6	48	6	27	0	326	30	204	10	175	5	26	0
Solyc03g122300.1.1	2	27	1	18	0	15	9	29	0	14	19	29	6
Solyc03g122310.2.1	14	177	9	133	8	2277	2757	1581	173	486	238	410	7
Solyc03g122340.2.1	28	796	264	685	52	543	108	225	7	45	57	332	22
Solyc03g122350.2.1	4	39	22	39	0	84	17	130	6	17	11	31	1

Solyc03g122360.2.1	3	13	1	12	0	3	0	19	0	12	10	0	0
Solyc03g122370.2.1	6	15	8	28	0	30	78	63	6	49	11	36	2
Solyc03g123370.2.1	14	592	53	343	14	1312	229	802	48	187	72	144	6
Solyc03g123380.1.1	0	0	0	0	0	0	0	0	0	0	0	0	0
Solyc03g123390.2.1	7	563	37	223	1	10	3	26	0	23	34	12	0
Solyc03g123400.1.1	0	0	0	0	0	0	0	0	0	0	0	0	0
Solyc03g123410.1.1	5	893	53	241	2	1	8	0	0	3	0	0	0
Solyc03g123420.1.1	2	4	1	4	0	1	3	10	3	10	8	7	0
Solyc03g123430.2.1	0	0	0	0	0	0	0	0	0	0	0	0	0
Solyc03g123440.2.1	5	8	3	15	0	43	24	39	3	12	5	17	0
Solyc03g123460.2.1	1	15	0	3	0	0	8	4	0	14	1	17	1
Solyc03g123490.1.1	21	281	31	181	5	92	69	220	2	1203	250	718	12
Solyc03g123500.2.1	10	163	12	49	2	478	150	502	29	184	62	162	4
Solyc03g123520.2.1	16	146	21	111	21	158	137	225	25	114	33	99	3
Solyc03g123530.2.1	8	12	0	17	1	91	35	115	3	69	18	72	0
Solyc03g123540.2.1	0	0	0	0	0	0	0	0	0	0	0	0	0
Solyc03g123560.2.1	4	201	17	71	6	34	8	9	1	80	50	77	1
Solyc03g123570.2.1	34	154	31	158	14	356	274	273	19	159	64	212	12
Solyc03g123580.2.1	1	4	0	1	0	0	4	4	0	0	0	1	0
Solyc03g123590.2.1	0	0	0	0	0	0	0	0	0	0	0	0	0
Solyc03g123600.2.1	0	0	0	0	0	0	0	0	0	0	0	0	0
Solyc03g123610.2.1	20	274	18	250	13	717	421	652	62	585	91	719	2
Solyc03g123620.2.1	7	208	22	112	12	4	10	12	0	18	13	60	0
Solyc03g123630.2.1	12	1172	8	495	10	255	187	744	23	3747	59	1902	3
Solyc03g123640.2.1	14	73	22	76	2	389	172	215	37	299	32	167	5
Solyc03g123650.1.1	0	0	0	0	0	0	0	0	0	0	0	0	0
Solyc03g123660.2.1	3	43	3	26	0	77	37	89	15	78	6	37	1
Solyc03g123670.2.1	0	0	0	0	0	0	0	0	0	0	0	0	0
Solyc03g123680.1.1	0	0	0	0	0	0	0	0	0	0	0	0	0
Solyc03g123700.2.1	4	33	5	24	1	34	19	21	1	38	3	23	0
Solyc03g123710.2.1	1	2	0	0	0	0	2	2	0	10	3	13	0
Solyc03g123720.2.1	4	45	5	18	2	31	23	44	8	112	19	92	0
Solyc03g123730.2.1	3	27	2	16	3	32	8	32	7	19	3	24	0
Solyc03g123740.2.1	18	192	16	106	13	529	234	736	186	264	41	136	1
Solyc03g123750.2.1	15	17	35	15	7	86	426	83	19	168	57	184	19
Solyc03g123760.2.1	8	82	11	64	0	88	108	299	10	87	23	74	0
Solyc03g123780.2.1	1	7	1	4	0	2	5	0	0	0	2	2	0
Solyc03g123790.2.1	0	0	0	0	0	0	0	0	0	0	0	0	0
Solyc03g123800.1.1	14	213	23	101	8	151	58	163	8	359	64	245	13
Solyc03g123830.2.1	13	331	55	278	51	143	163	345	54	2036	265	1658	16
Solyc03g123840.2.1	2	15	3	9	3	98	77	63	8	87	17	32	4

Solyc03g123850.2.1	0	0	0	0	0	0	0	0	0	0	0	0	0
Solyc03g123860.2.1	5	18	0	18	0	112	23	46	1	42	6	16	0
Solyc03g123870.2.1	11	75	15	55	19	155	108	178	15	80	29	67	2
Solyc03g123880.2.1	9	151	12	58	3	134	101	192	6	174	38	107	5
Solyc03g123890.2.1	3	9	1	0	0	26	15	11	0	12	2	9	0
Solyc03g123900.2.1	6	86	5	64	4	154	47	243	12	362	17	200	0
Solyc03g123910.2.1	8	103	32	56	3	122	273	156	30	274	57	252	4
Solyc03g123920.2.1	5	38	1	20	1	19	21	59	2	90	21	50	0
Solyc03g123930.1.1	0	0	0	0	0	0	0	0	0	0	0	0	0
Solyc03g123940.1.1	0	0	0	0	0	0	0	0	0	0	0	0	0
Solyc03g123950.2.1	1	3	0	1	0	0	5	12	0	7	1	6	0
Solyc03g123960.2.1	4	20	4	5	1	27	51	53	13	35	26	21	0
Solyc03g123980.2.1	13	69	12	57	5	219	254	312	43	157	56	134	5
Solyc03g123990.2.1	1	3	0	0	0	6	6	1	0	2	0	6	0
Solyc03g124000.1.1	0	0	0	0	0	0	0	0	0	0	0	0	0
Solyc03g124010.2.1	1	0	0	1	0	8	7	2	0	5	0	6	0
Solyc03g124020.1.1	0	0	0	0	0	0	0	0	0	0	0	0	0
Solyc03g124030.2.1	5	68	15	35	0	43	110	41	3	47	10	47	6
Solyc03g124040.2.1	3	17	2	5	0	56	24	103	3	52	1	20	4
Solyc03g124050.2.1	0	0	0	0	0	0	0	0	0	0	0	0	0
Solyc03g124060.2.1	0	0	0	0	0	0	0	0	0	0	0	0	0
Solyc03g124110.1.1	5	288	23	93	10	29	1	0	1	1	0	1	0
Solyc04g005010.2.1	3	9	0	11	0	12	19	16	7	5	0	3	0
Solyc04g005020.2.1	0	0	0	0	0	0	0	0	0	0	0	0	0
Solyc04g005030.2.1	3	46	3	81	0	219	52	276	4	124	7	101	3
Solyc04g005040.1.1	2	62	6	20	3	9	0	1	0	6	3	27	1
Solyc04g005050.1.1	4	103	11	97	5	10	66	23	5	43	14	120	2
Solyc04g005060.2.1	1	6	2	1	4	36	43	8	5	10	5	11	2
Solyc04g005070.2.1	10	892	21	240	0	14	6	66	1	123	44	19	1
Solyc04g005080.2.1	7	34	4	32	2	84	82	123	9	101	22	96	0
Solyc04g005090.2.1	5	177	11	59	1	14	51	14	1	33	15	38	0
Solyc04g005100.2.1	4	357	36	163	4	49	6	85	1	933	44	833	28
Solyc04g005120.2.1	5	56	5	20	0	29	52	48	3	34	15	42	0
Solyc04g005130.2.1	0	0	0	0	0	0	0	0	0	0	0	0	0
Solyc04g005140.2.1	0	0	0	0	0	0	0	0	0	0	0	0	0
Solyc04g005160.1.1	3	31	5	12	0	35	11	27	0	25	5	34	0
Solyc04g005180.2.1	2	9	1	39	0	16	7	35	0	23	6	3	0
Solyc04g005190.2.1	2	5	1	11	0	12	4	7	1	12	1	1	0
Solyc04g005200.2.1	0	0	0	0	0	0	0	0	0	0	0	0	0
Solyc04g005230.2.1	0	0	0	0	0	0	0	0	0	0	0	0	0
Solyc04g005250.2.1	8	8	0	9	0	128	64	66	6	38	2	40	5

Solyc04g005260.1.1	0	0	0	0	0	0	0	0	0	0	0	0	0
Solyc04g005270.1.1	14	171	10	182	15	530	271	1146	18	838	58	705	7
Solyc04g005280.2.1	9	37	2	30	0	113	35	99	1	59	13	35	3
Solyc04g005290.2.1	5	8	0	3	0	18	43	23	3	22	16	16	0
Solyc04g005320.2.1	0	0	0	0	0	0	0	0	0	0	0	0	0
Solyc04g005330.2.1	10	93	40	59	0	303	302	364	33	199	63	259	2
Solyc04g005340.2.1	9	845	221	342	71	3115	2641	1429	1057	1568	281	1344	22
Solyc04g005350.2.1	1	12	1	6	0	17	2	17	2	24	5	33	1
Solyc04g005380.2.1	9	3	0	10	2	505	85	162	7	15	6	6	0
Solyc04g005410.1.1	0	0	0	0	0	0	0	0	0	0	0	0	0
Solyc04g005420.2.1	7	58	12	75	2	432	249	375	25	123	46	64	0
Solyc04g005430.2.1	4	20	5	2	0	40	24	20	1	53	10	39	0
Solyc04g005440.1.1	0	0	0	0	0	0	0	0	0	0	0	0	0
Solyc04g005450.2.1	17	235	16	132	18	456	322	779	37	741	78	558	2
Solyc04g005460.1.1	0	0	0	0	0	0	0	0	0	0	0	0	0
Solyc04g005470.2.1	0	0	0	0	0	0	0	0	0	0	0	0	0
Solyc04g005480.1.1	4	163	23	66	4	132	4	29	1	45	7	43	3
Solyc04g005490.2.1	8	66	4	22	7	134	51	78	15	78	42	128	1
Solyc04g005500.2.1	16	40	6	29	4	37	52	58	9	130	33	106	0
Solyc04g005510.2.1	1	10	0	8	4	20	5	3	0	25	16	15	0
Solyc04g005520.2.1	1	2	1	2	0	1	7	9	0	7	0	0	0
Solyc04g005530.2.1	11	84	16	50	13	121	88	199	19	205	36	95	8
Solyc04g005540.2.1	0	0	0	0	0	0	0	0	0	0	0	0	0
Solyc04g005550.1.1	0	0	0	0	0	0	0	0	0	0	0	0	0
Solyc04g005560.2.1	1	1	0	6	0	0	4	1	0	4	0	2	0
Solyc04g005570.2.1	2	3	0	4	0	19	4	13	0	21	0	4	0
Solyc04g005580.1.1	0	0	0	0	0	0	0	0	0	0	0	0	0
Solyc04g005590.2.1	1	13	0	5	0	14	2	22	0	14	1	23	1
Solyc04g005610.2.1	5	7	5	32	0	163	74	291	0	11	0	2	0
Solyc04g005630.2.1	6	41	5	36	6	56	37	105	1	35	12	30	1
Solyc04g005650.1.1	5	130	16	118	2	284	134	361	17	285	23	157	1
Solyc04g005670.1.1	18	480	81	566	14	2891	363	1252	71	326	190	339	2
Solyc04g005680.2.1	1	22	3	2	0	14	12	48	1	64	4	18	0
Solyc04g005690.2.1	10	80	7	52	1	163	102	177	8	99	32	78	11
Solyc04g005700.2.1	0	0	0	0	0	0	0	0	0	0	0	0	0
Solyc04g005730.2.1	0	0	0	0	0	0	0	0	0	0	0	0	0
Solyc04g005770.1.1	0	0	0	0	0	0	0	0	0	0	0	0	0
Solyc04g005780.1.1	0	0	0	0	0	0	0	0	0	0	0	0	0
Solyc04g005790.2.1	1	0	0	1	0	5	18	8	3	3	0	0	0
Solyc04g005800.2.1	3	25	2	18	3	171	19	45	2	1	0	2	0
Solyc04g005810.2.1	0	0	0	0	0	0	0	0	0	0	0	0	0

Solyc04g005820.2.1	1	10	1	6	0	9	3	4	1	3	3	9	0
Solyc04g005830.2.1	1	3	0	0	0	0	0	2	0	4	0	8	0
Solyc04g005860.2.1	15	116	2	77	9	513	190	418	46	301	23	135	4
Solyc04g005900.2.1	0	0	0	0	0	0	0	0	0	0	0	0	0
Solyc04g005910.2.1	10	29	3	23	0	89	86	143	0	20	4	23	0
Solyc04g005920.1.1	0	0	0	0	0	0	0	0	0	0	0	0	0
Solyc04g005930.1.1	0	0	0	0	0	0	0	0	0	0	0	0	0
Solyc04g006930.2.1	0	0	0	0	0	0	0	0	0	0	0	0	0
Solyc04g006940.2.1	0	0	0	0	0	0	0	0	0	0	0	0	0
Solyc04g006960.2.1	10	72	1	25	0	284	88	266	1	29	0	22	0
Solyc04g006970.2.1	26	327	113	308	9	480	672	592	61	3923	2113	3248	133
Solyc04g006980.1.1	0	0	0	0	0	0	0	0	0	0	0	0	0
Solyc04g006990.2.1	0	0	0	0	0	0	0	0	0	0	0	0	0
Solyc04g007000.1.1	8	22	1	12	0	154	131	69	0	34	0	0	0
Solyc04g007010.2.1	0	0	0	0	0	0	0	0	0	0	0	0	0
Solyc04g007020.2.1	6	138	12	101	3	399	68	247	26	292	45	163	0
Solyc04g007030.2.1	0	0	0	0	0	0	0	0	0	0	0	0	0
Solyc04g007050.2.1	0	0	0	0	0	0	0	0	0	0	0	0	0
Solyc04g007060.2.1	14	152	11	76	3	149	116	307	9	313	32	133	6
Solyc04g007070.2.1	15	178	6	145	5	211	119	112	1	74	3	57	1
Solyc04g007080.2.1	0	0	0	0	0	0	0	0	0	0	0	0	0
Solyc04g007090.1.1	0	0	0	0	0	0	0	0	0	0	0	0	0
Solyc04g007100.2.1	1	0	0	0	0	1	3	4	0	6	1	8	0
Solyc04g007110.2.1	1	3	0	1	0	0	2	1	0	1	2	2	0
Solyc04g007120.2.1	10	325	27	268	39	1032	707	1193	147	837	241	630	17
Solyc04g007130.1.1	2	10	1	5	0	13	3	4	2	4	8	13	0
Solyc04g007140.1.1	0	0	0	0	0	0	0	0	0	0	0	0	0
Solyc04g007150.2.1	2	7	0	8	0	0	1	17	0	11	1	16	0
Solyc04g007160.1.1	7	35	2	44	2	34	3	73	2	180	14	155	0
Solyc04g007170.2.1	0	0	0	0	0	0	0	0	0	0	0	0	0
Solyc04g007180.1.1	3	3	0	6	0	20	6	16	0	17	6	11	0
Solyc04g007210.2.1	6	126	6	73	0	14	51	9	1	32	8	43	1
Solyc04g007220.2.1	4	32	2	33	2	75	19	68	1	72	15	64	0
Solyc04g007230.2.1	8	91	6	28	1	184	50	161	11	149	16	86	0
Solyc04g007240.1.1	0	0	0	0	0	0	0	0	0	0	0	0	0
Solyc04g007250.1.1	4	5	2	1	0	7	59	1	3	0	0	6	1
Solyc04g007260.2.1	3	9	0	6	6	24	37	13	8	18	3	12	4
Solyc04g007270.2.1	15	85	22	109	17	164	217	280	92	213	73	167	5
Solyc04g007280.2.1	27	141	6	153	11	239	173	439	65	318	97	255	4
Solyc04g007290.2.1	0	0	0	0	0	0	0	0	0	0	0	0	0
Solyc04g007300.2.1	0	0	0	0	0	0	0	0	0	0	0	0	0

Solyc04g007320.1.1	0	0	0	0	0	0	0	0	0	0	0	0	0
Solyc04g007330.1.1	0	0	0	0	0	0	0	0	0	0	0	0	0
Solyc04g007340.2.1	2	3	3	2	0	36	10	69	5	27	2	14	0
Solyc04g007350.2.1	2	17	0	8	0	22	2	14	1	16	6	9	0
Solyc04g007370.2.1	4	28	1	2	1	11	13	24	3	17	7	25	0
Solyc04g007380.1.1	0	0	0	0	0	0	0	0	0	0	0	0	0
Solyc04g007390.2.1	0	0	0	0	0	0	0	0	0	0	0	0	0
Solyc04g007400.2.1	1	29	0	19	0	7	3	31	0	10	1	11	0
Solyc04g007410.2.1	2	15	0	4	0	1	7	0	0	11	2	14	1
Solyc04g007420.2.1	0	0	0	0	0	0	0	0	0	0	0	0	0
Solyc04g007450.2.1	10	48	16	48	0	351	360	330	17	84	36	106	5
Solyc04g007460.2.1	16	98	6	134	8	220	81	320	21	255	26	200	0
Solyc04g007470.2.1	6	1	2	0	0	17	45	21	3	40	2	24	0
Solyc04g007480.1.1	0	0	0	0	0	0	0	0	0	0	0	0	0
Solyc04g007490.2.1	0	0	0	0	0	0	0	0	0	0	0	0	0
Solyc04g007500.1.1	4	139	11	115	2	27	20	167	0	144	0	270	3
Solyc04g007510.2.1	1	1	0	2	0	3	7	2	0	0	0	0	0
Solyc04g007520.2.1	4	17	4	6	0	50	72	59	2	49	6	20	6
Solyc04g007530.2.1	5	9	1	3	5	33	55	8	68	2	21	0	12
Solyc04g007540.1.1	3	14	3	20	0	8	26	37	4	61	9	76	0
Solyc04g007550.2.1	15	572	45	357	42	2199	333	2328	214	985	191	1135	16
Solyc04g007560.2.1	22	147	20	141	11	345	184	469	109	414	70	347	9
Solyc04g007580.1.1	6	297	46	171	6	29	150	29	2	11	4	47	2
Solyc04g007600.2.1	1	1	0	0	0	1	6	1	0	16	0	16	0
Solyc04g007610.2.1	6	66	4	52	1	450	25	220	10	76	23	86	5
Solyc04g007620.1.1	0	0	0	0	0	0	0	0	0	0	0	0	0
Solyc04g007630.1.1	3	20	1	14	0	30	37	17	12	1	0	0	0
Solyc04g007640.2.1	5	101	7	53	2	29	17	43	3	96	9	42	0
Solyc04g007650.2.1	4	0	4	2	2	2	9	0	0	32	15	61	1
Solyc04g007660.1.1	7	22	5	17	0	22	22	25	8	44	21	32	0
Solyc04g007670.1.1	0	0	0	0	0	0	0	0	0	0	0	0	0
Solyc04g007690.2.1	0	0	0	0	0	0	0	0	0	0	0	0	0
Solyc04g007700.2.1	2	15	0	13	0	9	13	13	1	26	2	21	0
Solyc04g007710.2.1	5	50	13	27	5	98	86	98	28	35	14	16	4
Solyc04g007720.2.1	1	1	0	0	0	0	3	3	0	4	0	1	0
Solyc04g007730.2.1	4	26	1	14	0	53	10	17	2	24	15	12	0
Solyc04g007750.2.1	0	0	0	0	0	0	0	0	0	0	0	0	0
Solyc04g007760.2.1	0	0	0	0	0	0	0	0	0	0	0	0	0
Solyc04g007770.2.1	10	18	1	41	3	45	0	103	5	17489	8159	30612	1224
Solyc04g007780.2.1	1	14	0	9	0	2	1	1	0	9	4	12	0
Solyc04g007790.2.1	1	456	3	235	0	21	2	28	0	0	4	1	0

Solyc04g007800.2.1	4	156	20	56	0	62	34	61	0	4	0	16	0
Solyc04g007810.1.1	0	0	0	0	0	0	0	0	0	0	0	0	0
Solyc04g007820.2.1	4	52	5	5	38	3	10	6	35	1	0	0	0
Solyc04g007830.2.1	1	3	3	0	7	3	3	0	7	0	0	0	0
Solyc04g007840.2.1	3	19	1	5	0	13	8	12	1	49	5	7	0
Solyc04g007850.2.1	2	24	3	20	0	189	71	118	6	47	6	35	1
Solyc04g007860.2.1	13	193	27	156	2	572	144	593	58	714	83	663	1
Solyc04g007870.2.1	13	102	16	115	2	58	33	55	7	246	32	330	3
Solyc04g007880.2.1	3	108	5	109	0	82	9	35	3	39	6	95	1
Solyc04g007890.2.1	0	0	0	0	0	0	0	0	0	0	0	0	0
Solyc04g007900.2.1	8	71	6	33	1	52	27	52	3	58	13	43	0
Solyc04g007910.2.1	1	2	1	0	0	4	3	1	0	1	2	8	0
Solyc04g007940.2.1	0	0	0	0	0	0	0	0	0	0	0	0	0
Solyc04g007950.2.1	1	14	0	6	0	0	1	1	4	6	4	6	0
Solyc04g007960.2.1	7	103	19	57	0	184	206	97	1	5	2	9	0
Solyc04g007970.2.1	2	100	8	39	2	63	35	71	7	291	18	230	0
Solyc04g007980.2.1	7	212	7	111	1	2242	379	3027	29	82	7	38	9
Solyc04g007990.1.1	4	12	0	7	0	116	84	77	4	1	0	3	0
Solyc04g008010.2.1	8	42	5	15	0	45	48	50	11	95	18	141	4
Solyc04g008020.2.1	7	58	8	61	2	32	74	72	3	174	18	124	2
Solyc04g008030.1.1	2	0	0	2	0	10	7	9	1	7	4	4	0
Solyc04g008040.2.1	1	8	0	4	0	0	4	2	1	4	0	16	0
Solyc04g008050.2.1	11	57	4	42	7	100	64	175	12	132	27	162	2
Solyc04g008060.2.1	2	23	5	13	0	8	6	13	0	0	3	0	0
Solyc04g008100.1.1	1	6	3	16	4	2	1	0	1	4	0	13	0
Solyc04g008110.2.1	6	8	2	6	2	5	17	41	4	62	15	43	16
Solyc04g008120.1.1	0	0	0	0	0	0	0	0	0	0	0	0	0
Solyc04g008140.1.1	0	0	0	0	0	0	0	0	0	0	0	0	0
Solyc04g008170.1.1	0	0	0	0	0	0	0	0	0	0	0	0	0
Solyc04g008200.2.1	0	0	0	0	0	0	0	0	0	0	0	0	0
Solyc04g008210.1.1	15	1056	75	608	1	734	333	567	1	44	6	56	2
Solyc04g008220.2.1	0	0	0	0	0	0	0	0	0	0	0	0	0
Solyc04g008230.2.1	0	0	0	0	0	0	0	0	0	0	0	0	0
Solyc04g008240.2.1	11	97	13	64	6	194	127	178	12	191	68	87	7
Solyc04g008250.1.1	0	0	0	0	0	0	0	0	0	0	0	0	0
Solyc04g008260.2.1	4	16	2	7	6	16	22	12	6	12	12	4	1
Solyc04g008270.2.1	0	0	0	0	0	0	0	0	0	0	0	0	0
Solyc04g008280.2.1	2	60	0	6	0	38	15	49	2	51	6	34	0
Solyc04g008290.2.1	10	146	9	79	8	79	79	121	10	83	7	52	0
Solyc04g008300.1.1	7	19	3	28	0	64	40	82	6	86	10	64	0
Solyc04g008310.1.1	1	5	1	1	0	6	5	8	3	7	0	10	0

Solyc04g008330.1.1	1	6	1	4	0	7	1	1	0	0	2	2	0
Solyc04g008340.2.1	15	181	9	184	22	901	292	892	35	350	107	298	4
Solyc04g008360.2.1	0	0	0	0	0	0	0	0	0	0	0	0	0
Solyc04g008370.1.1	0	0	0	0	0	0	0	0	0	0	0	0	0
Solyc04g008380.2.1	3	76	4	24	0	102	68	106	22	78	18	39	2
Solyc04g008400.1.1	0	0	0	0	0	0	0	0	0	0	0	0	0
Solyc04g008410.1.1	0	0	0	0	0	0	0	0	0	0	0	0	0
Solyc04g008430.1.1	1	6	0	6	0	0	2	10	0	3	2	1	0
Solyc04g008450.2.1	0	0	0	0	0	0	0	0	0	0	0	0	0
Solyc04g008460.2.1	5	166	3	109	5	281	83	323	22	658	95	380	5
Solyc04g008480.1.1	0	0	0	0	0	0	0	0	0	0	0	0	0
Solyc04g008490.1.1	2	54	1	21	0	42	22	90	5	91	15	67	0
Solyc04g008500.2.1	1	7	0	0	0	9	3	9	0	1	1	0	0
Solyc04g008510.2.1	8	80	11	36	4	42	64	108	18	151	25	98	0
Solyc04g008520.2.1	9	210	14	136	5	215	185	336	15	215	42	194	2
Solyc04g008540.2.1	2	13	1	12	0	23	23	38	2	38	6	21	0
Solyc04g008550.2.1	14	91	12	83	11	178	149	228	18	137	62	147	10
Solyc04g008560.2.1	4	11	0	7	2	20	11	16	1	8	8	20	0
Solyc04g008570.2.1	10	43	17	48	0	38	39	37	4	12	21	13	0
Solyc04g008580.2.1	1	1	0	5	0	8	4	10	1	12	10	6	0
Solyc04g008590.2.1	7	88	8	69	25	291	136	234	54	183	124	126	11
Solyc04g008600.2.1	0	0	0	0	0	0	0	0	0	0	0	0	0
Solyc04g008610.2.1	12	17	1	20	3	92	111	119	21	76	35	51	8
Solyc04g008620.2.1	9	73	9	31	3	230	56	134	2	119	28	116	0
Solyc04g008630.2.1	10	34	4	18	3	71	69	99	9	23	18	39	1
Solyc04g008640.2.1	5	22	4	8	0	101	43	80	9	82	7	50	0
Solyc04g008650.2.1	0	0	0	0	0	0	0	0	0	0	0	0	0
Solyc04g008660.2.1	4	25	3	6	2	43	16	28	6	57	3	43	0
Solyc04g008670.1.1	0	0	0	0	0	0	0	0	0	0	0	0	0
Solyc04g008680.2.1	24	216	24	237	16	533	379	741	67	397	95	295	11
Solyc04g008690.2.1	2	5	2	9	3	7	8	23	0	19	8	61	1
Solyc04g008700.2.1	5	13	4	21	4	6	28	50	24	25	14	15	14
Solyc04g008710.2.1	8	140	37	83	1	109	257	218	13	243	94	278	9
Solyc04g008720.2.1	0	0	0	0	0	0	0	0	0	0	0	0	0
Solyc04g008730.2.1	3	23	2	11	0	17	27	36	0	129	1	54	0
Solyc04g008740.2.1	7	313	9	156	3	429	104	559	28	630	64	602	6
Solyc04g008750.2.1	0	0	0	0	0	0	0	0	0	0	0	0	0
Solyc04g008760.1.1	4	38	2	21	1	54	17	57	1	53	7	37	0
Solyc04g008770.2.1	1	7	1	2	0	0	3	7	0	1	0	3	0
Solyc04g008780.2.1	0	0	0	0	0	0	0	0	0	0	0	0	0
Solyc04g008790.1.1	1	4	1	2	0	2	3	9	0	3	0	3	0

Solyc04g008800.2.1	4	15	1	2	0	42	18	37	5	42	3	22	0
Solyc04g008810.2.1	1	76	9	14	3	103	68	124	22	209	66	170	3
Solyc04g008820.2.1	1	7	0	0	0	0	2	5	0	10	4	3	0
Solyc04g008830.1.1	1	5	0	0	2	9	3	2	1	0	2	0	0
Solyc04g008840.2.1	10	26	3	39	13	40	64	78	8	113	20	106	4
Solyc04g008850.1.1	0	0	0	0	0	0	0	0	0	0	0	0	0
Solyc04g008860.2.1	3	15	0	10	0	15	9	30	0	7	2	34	0
Solyc04g008870.2.1	1	4	0	0	0	0	5	3	3	1	3	5	0
Solyc04g008880.2.1	0	0	0	0	0	0	0	0	0	0	0	0	0
Solyc04g008890.2.1	1	81	49	27	18	262	2208	158	57	87	370	27	12
Solyc04g008900.2.1	0	0	0	0	0	0	0	0	0	0	0	0	0
Solyc04g008910.1.1	0	0	0	0	0	0	0	0	0	0	0	0	0
Solyc04g008940.2.1	0	0	0	0	0	0	0	0	0	0	0	0	0
Solyc04g008950.2.1	0	0	0	0	0	0	0	0	0	0	0	0	0
Solyc04g008960.2.1	1	7	0	5	0	3	2	4	0	34	0	13	0
Solyc04g008970.2.1	0	0	0	0	0	0	0	0	0	0	0	0	0
Solyc04g008980.2.1	10	53	6	72	4	159	96	196	11	634	57	410	5
Solyc04g008990.2.1	2	11	2	8	7	31	20	18	2	14	0	6	2
Solyc04g009000.1.1	0	0	0	0	0	0	0	0	0	0	0	0	0
Solyc04g009010.1.1	1	3	0	2	0	6	2	1	0	6	1	5	0
Solyc04g009020.2.1	8	42	22	22	1	12	89	8	1	23	26	58	1
Solyc04g009030.2.1	5	6447	977	2723	6	257	792	350	4	955	266	1025	3
Solyc04g009040.2.1	0	0	0	0	0	0	0	0	0	0	0	0	0
Solyc04g009050.2.1	4	56	11	18	0	51	24	33	2	18	8	18	0
Solyc04g009060.2.1	7	47	9	30	2	100	61	97	16	98	31	85	1
Solyc04g009070.1.1	0	0	0	0	0	0	0	0	0	0	0	0	0
Solyc04g009110.1.1	0	0	0	0	0	0	0	0	0	0	0	0	0
Solyc04g009130.2.1	0	0	0	0	0	0	0	0	0	0	0	0	0
Solyc04g009140.2.1	0	0	0	0	0	0	0	0	0	0	0	0	0
Solyc04g009150.1.1	0	0	0	0	0	0	0	0	0	0	0	0	0
Solyc04g009160.2.1	4	13	0	0	0	13	15	34	5	23	5	10	2
Solyc04g009170.1.1	1	1	0	0	0	4	4	6	0	8	3	0	0
Solyc04g009180.1.1	4	109	5	75	7	37	18	46	11	34	5	16	0
Solyc04g009190.2.1	0	0	0	0	0	0	0	0	0	0	0	0	0
Solyc04g009200.2.1	12	113	24	135	0	131	181	159	15	209	104	208	12
Solyc04g009210.1.1	0	0	0	0	0	0	0	0	0	0	0	0	0
Solyc04g009220.1.1	0	0	0	0	0	0	0	0	0	0	0	0	0
Solyc04g009230.2.1	4	58	3	33	1	189	16	103	1	121	3	85	1
Solyc04g009240.1.1	0	0	0	0	0	0	0	0	0	0	0	0	0
Solyc04g009250.1.1	0	0	0	0	0	0	0	0	0	0	0	0	0
Solyc04g009280.1.1	0	0	0	0	0	0	0	0	0	0	0	0	0

Solyc04g009290.1.1	0	0	0	0	0	0	0	0	0	0	0	0	0
Solyc04g009300.2.1	7	37	4	8	0	76	39	121	5	35	8	51	5
Solyc04g009310.2.1	3	26	2	4	0	42	13	24	4	28	9	12	0
Solyc04g009320.2.1	0	0	0	0	0	0	0	0	0	0	0	0	0
Solyc04g009330.2.1	1	0	0	0	0	7	8	3	0	13	0	17	0
Solyc04g009340.2.1	6	37	4	49	1	123	57	136	10	88	25	46	0
Solyc04g009350.2.1	6	33	4	15	0	35	29	66	5	141	7	106	4
Solyc04g009360.2.1	4	8	1	8	0	30	24	31	2	24	3	13	0
Solyc04g009370.2.1	2	53	4	24	0	37	76	25	2	2	5	2	3
Solyc04g009380.1.1	2	22	3	7	1	11	10	13	2	10	2	1	0
Solyc04g009400.2.1	0	0	0	0	0	0	0	0	0	0	0	0	0
Solyc04g009410.2.1	5	177	21	91	9	248	140	323	22	296	35	217	8
Solyc04g009420.2.1	3	17	1	14	0	3	7	8	1	14	8	17	1
Solyc04g009430.2.1	1	8	3	1	0	12	5	2	2	10	3	8	0
Solyc04g009440.2.1	4	149	16	41	0	115	5	22	4	7	0	5	0
Solyc04g009450.1.1	0	0	0	0	0	0	0	0	0	0	0	0	0
Solyc04g009470.2.1	1	6	0	10	0	19	5	11	0	13	0	0	0
Solyc04g009480.1.1	0	0	0	0	0	0	0	0	0	0	0	0	0
Solyc04g009500.2.1	8	53	14	45	0	94	50	170	14	99	12	85	1
Solyc04g009510.2.1	11	68	16	54	14	31	78	74	5	183	27	128	1
Solyc04g009520.2.1	0	0	0	0	0	0	0	0	0	0	0	0	0
Solyc04g009530.2.1	2	19	3	8	1	16	27	25	1	6	6	12	0
Solyc04g009540.1.1	3	94	0	23	0	29	37	62	0	28	1	23	2
Solyc04g009550.2.1	2	8	2	0	0	6	6	16	3	16	4	9	0
Solyc04g009560.2.1	5	38	11	26	5	6	35	29	11	102	25	66	1
Solyc04g009570.2.1	3	15	0	7	1	17	19	18	2	19	5	11	0
Solyc04g009580.2.1	6	70	6	20	0	34	42	49	8	177	5	81	0
Solyc04g009600.2.1	0	0	0	0	0	0	0	0	0	0	0	0	0
Solyc04g009610.2.1	0	0	0	0	0	0	0	0	0	0	0	0	0
Solyc04g009620.2.1	11	38	3	25	2	53	70	111	14	163	42	107	3
Solyc04g009630.2.1	23	189	67	155	3	278	198	604	21	172	57	130	3
Solyc04g009640.2.1	1	1	0	1	0	6	5	8	0	0	0	3	0
Solyc04g009650.2.1	6	23	1	24	0	41	30	72	8	95	13	86	2
Solyc04g009660.2.1	0	0	0	0	0	0	0	0	0	0	0	0	0
Solyc04g009700.2.1	7	23	6	6	3	40	31	47	7	27	14	31	3
Solyc04g009720.1.1	1	1	1	0	0	2	4	1	0	5	2	2	2
Solyc04g009730.2.1	0	0	0	0	0	0	0	0	0	0	0	0	0
Solyc04g009740.2.1	9	69	13	46	5	55	58	101	2	256	13	185	3
Solyc04g009770.2.1	10	1176	135	698	46	2199	902	1652	225	2561	514	1469	39
Solyc04g009790.2.1	1	2	0	6	0	1	0	1	0	2	4	9	0
Solyc04g009800.2.1	3	54	1	19	0	103	22	67	3	50	9	34	0

Solyc04g009810.2.1	6	27	1	48	0	26	45	62	11	60	23	93	0
Solyc04g009820.2.1	1	1	1	0	0	0	0	1	0	11	5	3	0
Solyc04g009830.2.1	2	1	1	0	0	3	2	1	0	2	4	4	0
Solyc04g009840.2.1	2	4	0	0	0	3	4	6	0	3	0	5	0
Solyc04g009850.2.1	5	11	0	13	0	38	17	39	3	23	6	27	0
Solyc04g009860.2.1	0	0	0	0	0	0	0	0	0	0	0	0	0
Solyc04g009900.2.1	7	59	5	23	17	361	83	106	57	6	17	16	1
Solyc04g009910.2.1	6	4	1	7	0	261	208	406	10	77	4	20	1
Solyc04g009920.2.1	5	35	3	17	6	51	34	48	3	59	8	23	0
Solyc04g009940.2.1	8	41	4	8	0	113	63	127	8	43	17	70	5
Solyc04g009950.2.1	20	155	17	178	3	233	150	380	22	352	98	317	1
Solyc04g009960.2.1	2	47	0	39	0	238	84	55	0	11	176	9	0
Solyc04g009970.2.1	5	7	2	9	0	24	54	50	13	21	8	16	0
Solyc04g009990.2.1	12	42	5	35	4	96	61	102	12	70	19	82	0
Solyc04g010000.2.1	1	0	1	0	12	0	19	0	13	4	3	0	13
Solyc04g010020.2.1	0	0	0	0	0	0	0	0	0	0	0	0	0
Solyc04g010030.1.1	0	0	0	0	0	0	0	0	0	0	0	0	0
Solyc04g010040.2.1	0	0	0	0	0	0	0	0	0	0	0	0	0
Solyc04g010050.2.1	0	0	0	0	0	0	0	0	0	0	0	0	0
Solyc04g010060.2.1	2	16	2	6	0	13	16	5	3	7	7	1	0
Solyc04g010070.2.1	9	58	3	45	1	77	46	121	21	67	30	54	0
Solyc04g010080.2.1	7	64	6	19	1	80	30	97	9	112	15	58	0
Solyc04g010090.2.1	0	0	0	0	0	0	0	0	0	0	0	0	0
Solyc04g010100.1.1	1	27	0	12	0	39	9	13	1	43	3	19	0
Solyc04g010110.2.1	5	9	3	15	0	30	26	59	3	66	11	60	1
Solyc04g010120.2.1	5	58	2	36	2	71	39	112	8	151	11	91	0
Solyc04g010130.1.1	0	0	0	0	0	0	0	0	0	0	0	0	0
Solyc04g010140.1.1	0	0	0	0	0	0	0	0	0	0	0	0	0
Solyc04g010180.2.1	2	8	0	13	1	34	8	38	2	11	0	7	0
Solyc04g010190.1.1	3	178	10	135	0	17	13	35	0	96	2	100	0
Solyc04g010200.1.1	2	3	4	1	19	13	40	2	76	22	10	0	1
Solyc04g010210.1.1	0	0	0	0	0	0	0	0	0	0	0	0	0
Solyc04g010220.2.1	4	25	3	16	0	48	19	95	1	91	15	85	2
Solyc04g010230.2.1	3	9	1	0	0	14	2	22	1	42	4	19	0
Solyc04g010240.2.1	0	0	0	0	0	0	0	0	0	0	0	0	0
Solyc04g010250.2.1	8	121	22	77	48	118	224	378	57	971	19	639	2
Solyc04g010260.2.1	10	81	20	88	8	291	129	258	22	285	101	212	4
Solyc04g010290.2.1	4	42	2	34	1	24	55	38	5	106	11	46	0
Solyc04g010300.2.1	0	0	0	0	0	0	0	0	0	0	0	0	0
Solyc04g010310.2.1	12	45	6	51	4	83	47	114	8	75	26	58	0
Solyc04g010320.1.1	0	0	0	0	0	0	0	0	0	0	0	0	0

Solyc04g011350.2.1	11	41	4	18	1	59	74	69	40	34	23	21	3
Solyc04g011360.2.1	5	75	3	42	4	101	92	129	14	135	29	144	7
Solyc04g011370.2.1	3	90	11	27	2	110	203	78	30	221	92	94	3
Solyc04g011380.2.1	12	51	8	43	1	105	65	137	12	150	34	105	1
Solyc04g011390.1.1	3	187	2	45	3	62	14	39	5	266	71	197	0
Solyc04g011400.2.1	7	285	31	167	11	457	336	585	75	1317	73	1051	51
Solyc04g011420.2.1	1	4	0	0	0	8	0	17	0	26	5	3	0
Solyc04g011430.2.1	0	0	0	0	0	0	0	0	0	0	0	0	0
Solyc04g011440.2.1	19	85	14	89	17	476	65	334	13	351	717	540	5
Solyc04g011460.1.1	5	45	3	39	4	108	114	107	20	59	15	41	6
Solyc04g011500.2.1	4	326	60	171	11	132	223	153	16	388	99	562	13
Solyc04g011510.2.1	6	652	29	306	9	272	101	376	39	1248	117	1272	11
Solyc04g011520.2.1	5	47	9	23	0	144	40	84	12	22	5	28	0
Solyc04g011530.2.1	2	8	1	15	0	202	7	144	1	22	1	33	0
Solyc04g011540.2.1	2	11	1	3	4	23	1	26	1	0	6	0	0
Solyc04g011550.2.1	4	95	2	62	1	91	32	162	5	509	18	385	1
Solyc04g011560.2.1	7	96	5	64	11	139	55	177	18	171	13	61	1
Solyc04g011570.2.1	0	0	0	0	0	0	0	0	0	0	0	0	0
Solyc04g011580.2.1	12	38	2	54	1	28	83	172	7	232	54	188	5
Solyc04g011600.2.1	0	0	0	0	0	0	0	0	0	0	0	0	0
Solyc04g011610.1.1	7	95	8	98	18	105	80	156	33	162	37	92	12
Solyc04g011620.2.1	5	26	8	11	3	66	83	54	9	66	34	58	7
Solyc04g011630.1.1	1	35	1	14	0	47	6	40	1	29	0	35	0
Solyc04g011640.2.1	0	0	0	0	0	0	0	0	0	0	0	0	0
Solyc04g011650.2.1	11	10	0	8	0	86	32	61	5	30	5	21	0
Solyc04g011670.2.1	2	7	3	19	0	11	26	50	0	23	6	8	0
Solyc04g011680.1.1	0	0	0	0	0	0	0	0	0	0	0	0	0
Solyc04g011690.2.1	1	18	1	29	0	10	6	9	0	0	0	0	0
Solyc04g011700.1.1	6	17	0	5	0	26	7	29	2	14	14	12	0
Solyc04g011770.2.1	2	0	0	0	0	2	5	4	0	9	0	6	0
Solyc04g011810.1.1	0	0	0	0	0	0	0	0	0	0	0	0	0
Solyc04g011830.1.1	0	0	0	0	0	0	0	0	0	0	0	0	0
Solyc04g011860.1.1	0	0	0	0	0	0	0	0	0	0	0	0	0
Solyc04g011880.1.1	0	0	0	0	0	0	0	0	0	0	0	0	0
Solyc04g011900.2.1	8	123	6	42	0	15	50	28	2	43	5	23	0
Solyc04g011910.2.1	0	0	0	0	0	0	0	0	0	0	0	0	0
Solyc04g011980.1.1	0	0	0	0	0	0	0	0	0	0	0	0	0
Solyc04g011990.1.1	0	0	0	0	0	0	0	0	0	0	0	0	0
Solyc04g012010.2.1	0	0	0	0	0	0	0	0	0	0	0	0	0
Solyc04g012020.1.1	0	0	0	0	0	0	0	0	0	0	0	0	0
Solyc04g012030.2.1	1	1	0	3	0	7	7	9	2	7	0	2	0

Solyc04g012040.2.1	5	19	1	15	0	24	21	32	2	12	7	13	0
Solyc04g012050.2.1	1	0	0	0	0	11	4	5	0	0	0	0	0
Solyc04g012060.2.1	4	22	2	1	0	2	7	22	2	9	11	21	0
Solyc04g012090.1.1	0	0	0	0	0	0	0	0	0	0	0	0	0
Solyc04g012100.1.1	0	0	0	0	0	0	0	0	0	0	0	0	0
Solyc04g012120.2.1	3	91	33	44	32	89	567	115	98	80	139	155	22
Solyc04g012140.1.1	3	5	1	2	0	59	22	17	3	12	2	10	0
Solyc04g012150.2.1	0	0	0	0	0	0	0	0	0	0	0	0	0
Solyc04g012160.2.1	7	77	5	45	1	922	280	485	103	42	9	64	0
Solyc04g012170.2.1	26	106	24	120	10	250	318	371	44	267	59	276	9
Solyc04g012180.2.1	5	75	9	34	3	58	83	111	20	135	19	91	1
Solyc04g012190.1.1	0	0	0	0	0	0	0	0	0	0	0	0	0
Solyc04g014210.2.1	15	91	11	79	22	211	161	225	25	223	36	202	4
Solyc04g014220.1.1	1	1	0	0	0	15	5	0	3	0	0	1	0
Solyc04g014230.1.1	0	0	0	0	0	0	0	0	0	0	0	0	0
Solyc04g014240.1.1	3	14	0	7	0	11	17	22	2	11	5	16	0
Solyc04g014250.2.1	3	20	4	2	4	36	101	68	9	124	33	72	13
Solyc04g014260.1.1	0	0	0	0	0	0	0	0	0	0	0	0	0
Solyc04g014270.2.1	4	33	0	18	6	104	35	63	11	65	3	27	3
Solyc04g014280.1.1	0	0	0	0	0	0	0	0	0	0	0	0	0
Solyc04g014290.1.1	0	0	0	0	0	0	0	0	0	0	0	0	0
Solyc04g014310.1.1	0	0	0	0	0	0	0	0	0	0	0	0	0
Solyc04g014320.1.1	0	0	0	0	0	0	0	0	0	0	0	0	0
Solyc04g014330.1.1	0	0	0	0	0	0	0	0	0	0	0	0	0
Solyc04g014350.1.1	0	0	0	0	0	0	0	0	0	0	0	0	0
Solyc04g014360.2.1	0	0	0	0	0	0	0	0	0	0	0	0	0
Solyc04g014370.2.1	4	5	1	3	1	32	41	23	33	13	10	2	4
Solyc04g014380.2.1	0	0	0	0	0	0	0	0	0	0	0	0	0
Solyc04g014390.2.1	2	51	3	18	8	161	106	95	10	146	25	113	12
Solyc04g014400.2.1	36	802	34	725	0	960	195	1023	1	22	1	3	0
Solyc04g014410.2.1	0	0	0	0	0	0	0	0	0	0	0	0	0
Solyc04g014420.2.1	0	0	0	0	0	0	0	0	0	0	0	0	0
Solyc04g014440.2.1	0	0	0	0	0	0	0	0	0	0	0	0	0
Solyc04g014450.1.1	0	0	0	0	0	0	0	0	0	0	0	0	0
Solyc04g014460.2.1	5	173	6	88	1	174	63	204	17	214	48	200	4
Solyc04g014470.2.1	0	0	0	0	0	0	0	0	0	0	0	0	0
Solyc04g014480.2.1	5	17	0	7	0	28	21	35	0	98	7	120	0
Solyc04g014490.1.1	2	68	5	29	6	110	53	112	30	222	32	116	1
Solyc04g014500.2.1	8	25	2	54	0	164	47	133	6	73	19	80	0
Solyc04g014510.2.1	0	0	0	0	0	0	0	0	0	0	0	0	0
Solyc04g014530.1.1	8	28	1	22	0	186	60	99	1	30	0	50	0

Solyc04g014540.1.1	2	16	0	6	0	6	4	11	1	34	6	17	0
Solyc04g014550.2.1	0	0	0	0	0	0	0	0	0	0	0	0	0
Solyc04g014560.2.1	13	133	15	155	4	265	170	377	42	259	56	108	4
Solyc04g014570.2.1	13	261	31	119	17	306	111	338	42	797	149	606	12
Solyc04g014600.2.1	2	7	7	5	0	52	38	26	1	169	71	257	0
Solyc04g014610.2.1	0	0	0	0	0	0	0	0	0	0	0	0	0
Solyc04g014640.1.1	0	0	0	0	0	0	0	0	0	0	0	0	0
Solyc04g014650.2.1	0	0	0	0	0	0	0	0	0	0	0	0	0
Solyc04g014670.2.1	4	19	2	28	2	69	48	48	9	66	14	48	3
Solyc04g014680.2.1	5	200	3	73	4	27	14	42	1	283	31	288	3
Solyc04g014690.2.1	0	0	0	0	0	0	0	0	0	0	0	0	0
Solyc04g014700.2.1	1	6	2	1	0	3	15	11	1	27	4	18	0
Solyc04g014710.2.1	3	28	0	26	0	27	8	32	2	85	8	69	0
Solyc04g014720.2.1	4	131	43	44	25	171	280	259	63	346	579	378	44
Solyc04g014730.2.1	9	22	7	4	0	45	117	70	5	130	31	88	9
Solyc04g014740.1.1	3	25	4	19	0	74	44	40	3	69	22	52	0
Solyc04g014760.2.1	1	22	0	7	2	45	29	21	0	52	4	60	0
Solyc04g014770.1.1	0	0	0	0	0	0	0	0	0	0	0	0	0
Solyc04g014800.2.1	0	0	0	0	0	0	0	0	0	0	0	0	0
Solyc04g014810.2.1	7	129	6	55	5	103	23	121	8	107	26	65	3
Solyc04g014820.2.1	1	5	1	1	0	8	9	2	0	17	0	9	0
Solyc04g014830.1.1	0	0	0	0	0	0	0	0	0	0	0	0	0
Solyc04g014850.2.1	0	0	0	0	0	0	0	0	0	0	0	0	0
Solyc04g014870.2.1	6	28	0	29	1	114	42	135	19	51	16	59	2
Solyc04g014900.2.1	2	18	5	32	3	98	4	40	3	15	0	20	0
Solyc04g014960.2.1	0	0	0	0	0	0	0	0	0	0	0	0	0
Solyc04g014970.1.1	0	0	0	0	0	0	0	0	0	0	0	0	0
Solyc04g014990.1.1	0	0	0	0	0	0	0	0	0	0	0	0	0
Solyc04g015010.2.1	0	0	0	0	0	0	0	0	0	0	0	0	0
Solyc04g015020.2.1	17	273	26	145	18	323	160	412	72	409	464	253	31
Solyc04g015030.2.1	0	0	0	0	0	0	0	0	0	0	0	0	0
Solyc04g015040.2.1	3	55	2	35	0	23	13	30	0	26	2	19	0
Solyc04g015050.2.1	6	33	2	36	10	47	41	55	6	82	16	41	2
Solyc04g015070.2.1	1	2	2	2	0	0	1	8	0	8	2	9	0
Solyc04g015080.2.1	0	0	0	0	0	0	0	0	0	0	0	0	0
Solyc04g015090.1.1	0	0	0	0	0	0	0	0	0	0	0	0	0
Solyc04g015100.2.1	10	105	3	53	3	634	109	483	17	142	66	112	3
Solyc04g015120.2.1	0	0	0	0	0	0	0	0	0	0	0	0	0
Solyc04g015130.2.1	7	93	13	91	7	92	90	252	19	122	16	132	3
Solyc04g015140.1.1	0	0	0	0	0	0	0	0	0	0	0	0	0
Solyc04g015150.2.1	4	50	2	24	0	36	70	54	3	33	5	44	0

Solyc04g015160.2.1	5	13	7	15	2	51	56	25	13	24	9	9	4
Solyc04g015170.2.1	12	50	4	29	2	73	54	101	10	71	38	52	0
Solyc04g015180.2.1	1	11	0	30	0	37	12	44	3	93	19	145	2
Solyc04g015190.2.1	5	18	0	8	6	24	27	47	8	82	9	22	1
Solyc04g015200.2.1	10	14	1	11	1	195	43	214	11	31	15	22	0
Solyc04g015210.2.1	0	0	0	0	0	0	0	0	0	0	0	0	0
Solyc04g015270.2.1	12	275	13	133	26	164	128	307	22	346	77	273	18
Solyc04g015280.2.1	0	0	0	0	0	0	0	0	0	0	0	0	0
Solyc04g015290.2.1	2	4	0	4	0	12	15	19	0	18	1	18	0
Solyc04g015300.2.1	1	10	4	10	0	17	16	28	0	20	5	31	0
Solyc04g015330.2.1	2	16	1	4	0	3	17	22	0	12	2	5	0
Solyc04g015340.2.1	6	207	23	54	3	38	76	57	9	181	36	87	0
Solyc04g015350.2.1	12	39	2	34	6	137	77	160	13	134	13	58	1
Solyc04g015360.2.1	11	484	35	332	25	85	55	107	22	21	13	60	0
Solyc04g015370.2.1	3	51	3	49	0	119	10	70	4	427	21	232	3
Solyc04g015400.2.1	0	0	0	0	0	0	0	0	0	0	0	0	0
Solyc04g015430.2.1	0	0	0	0	0	0	0	0	0	0	0	0	0
Solyc04g015440.2.1	10	43	9	32	0	6	80	45	4	28	6	12	0
Solyc04g015450.2.1	15	549	42	383	2	225	133	313	1	388	45	235	1
Solyc04g015460.2.1	0	0	0	0	0	0	0	0	0	0	0	0	0
Solyc04g015470.2.1	0	0	0	0	0	0	0	0	0	0	0	0	0
Solyc04g015480.2.1	6	71	3	49	1	54	61	113	1	32	10	47	0
Solyc04g015490.2.1	11	187	21	118	4	71	144	73	1	59	24	83	2
Solyc04g015510.1.1	0	0	0	0	0	0	0	0	0	0	0	0	0
Solyc04g015520.2.1	3	14	1	28	0	260	115	263	12	8	4	6	0
Solyc04g015530.1.1	0	0	0	0	0	0	0	0	0	0	0	0	0
Solyc04g015560.2.1	6	49	1	37	2	100	59	87	5	259	8	251	0
Solyc04g015570.2.1	1	4	1	3	0	1	2	2	1	10	3	2	0
Solyc04g015580.2.1	1	2	0	0	0	4	3	4	0	0	0	4	0
Solyc04g015590.2.1	0	0	0	0	0	0	0	0	0	0	0	0	0
Solyc04g015600.2.1	3	5	0	4	1	1	47	13	8	7	12	4	0
Solyc04g015610.2.1	0	0	0	0	0	0	0	0	0	0	0	0	0
Solyc04g015620.2.1	0	0	0	0	0	0	0	0	0	0	0	0	0
Solyc04g015630.2.1	0	0	0	0	0	0	0	0	0	0	0	0	0
Solyc04g015640.1.1	0	0	0	0	0	0	0	0	0	0	0	0	0
Solyc04g015680.2.1	10	194	7	140	4	331	77	427	15	449	42	331	1
Solyc04g015690.2.1	4	13	6	3	2	33	17	27	2	25	8	18	0
Solyc04g015710.2.1	0	0	0	0	0	0	0	0	0	0	0	0	0
Solyc04g015740.2.1	12	103	11	41	7	85	58	129	29	115	53	108	0
Solyc04g015750.2.1	21	4756	1188	3002	62	108	142	138	15	310	619	449	8
Solyc04g015760.2.1	3	16	3	3	0	9	13	48	6	32	2	29	0

Solyc04g015830.2.1	18	394	56	384	24	1301	814	1223	90	1443	99	880	23
Solyc04g015850.2.1	3	65	3	16	2	5	14	26	0	104	0	58	0
Solyc04g015940.2.1	5	11	6	15	0	34	25	47	1	46	10	48	0
Solyc04g015950.1.1	2	12	2	21	0	10	14	28	0	25	2	47	0
Solyc04g015960.2.1	0	0	0	0	0	0	0	0	0	0	0	0	0
Solyc04g015970.2.1	17	70	21	52	0	99	151	177	7	81	18	67	1
Solyc04g015980.2.1	0	0	0	0	0	0	0	0	0	0	0	0	0
Solyc04g016000.2.1	1	0	0	0	0	8	7	3	2	0	0	0	0
Solyc04g016010.2.1	0	0	0	0	0	0	0	0	0	0	0	0	0
Solyc04g016050.2.1	0	0	0	0	0	0	0	0	0	0	0	0	0
Solyc04g016060.1.1	0	0	0	0	0	0	0	0	0	0	0	0	0
Solyc04g016080.1.1	0	0	0	0	0	0	0	0	0	0	0	0	0
Solyc04g016130.2.1	6	27	6	22	3	53	47	77	4	79	12	98	6
Solyc04g016160.2.1	0	0	0	0	0	0	0	0	0	0	0	0	0
Solyc04g016180.2.1	1	2	0	0	0	14	24	37	5	2	0	0	0
Solyc04g016190.1.1	0	0	0	0	0	0	0	0	0	0	0	0	0
Solyc04g016200.1.1	0	0	0	0	0	0	0	0	0	0	0	0	0
Solyc04g016210.2.1	2	9	6	8	0	0	2	1	0	12	11	1	0
Solyc04g016220.2.1	0	0	0	0	0	0	0	0	0	0	0	0	0
Solyc04g016230.2.1	1	1	0	5	0	10	0	0	0	0	0	0	0
Solyc04g016240.2.1	6	50	2	31	0	44	44	102	2	149	22	74	1
Solyc04g016250.2.1	0	0	0	0	0	0	0	0	0	0	0	0	0
Solyc04g016260.2.1	11	130	11	101	6	54	51	77	11	32	14	53	0
Solyc04g016270.1.1	2	141	2	81	0	227	2	49	0	86	0	37	0
Solyc04g016280.2.1	0	0	0	0	0	0	0	0	0	0	0	0	0
Solyc04g016290.2.1	0	0	0	0	0	0	0	0	0	0	0	0	0
Solyc04g016330.2.1	7	24	0	0	5	36	30	13	24	131	24	73	12
Solyc04g016350.1.1	1	6	0	2	0	0	10	5	1	2	2	1	0
Solyc04g016360.2.1	5	79	1	32	1	196	99	247	9	491	33	250	0
Solyc04g016370.2.1	7	24	3	15	0	34	33	47	5	17	8	32	0
Solyc04g016380.2.1	4	39	4	10	0	108	41	129	10	45	26	38	3
Solyc04g016390.1.1	0	0	0	0	0	0	0	0	0	0	0	0	0
Solyc04g016400.2.1	0	0	0	0	0	0	0	0	0	0	0	0	0
Solyc04g016410.2.1	12	25	5	18	6	102	80	83	23	48	20	63	0
Solyc04g016420.2.1	2	0	0	1	0	126	20	172	2	3	5	6	0
Solyc04g016430.2.1	0	0	0	0	0	0	0	0	0	0	0	0	0
Solyc04g016440.2.1	0	0	0	0	0	0	0	0	0	0	0	0	0
Solyc04g016470.2.1	6	2	1	1	1	82	41	32	4	18	138	3	2
Solyc04g016480.2.1	2	10	0	10	0	2	28	6	2	11	2	5	0
Solyc04g016490.2.1	0	0	0	0	0	0	0	0	0	0	0	0	0
Solyc04g016510.2.1	7	40	6	19	0	90	84	81	6	139	23	45	0

Solyc04g016520.2.1	4	28	1	12	0	57	11	82	5	65	11	88	0
Solyc04g016530.2.1	2	7	0	3	1	11	16	19	3	15	9	18	1
Solyc04g016540.1.1	1	1	0	0	0	12	4	3	0	4	2	1	0
Solyc04g016550.2.1	2	8	0	7	0	0	12	2	0	13	0	7	0
Solyc04g016560.1.1	0	0	0	0	0	0	0	0	0	0	0	0	0
Solyc04g016590.2.1	0	0	0	0	0	0	0	0	0	0	0	0	0
Solyc04g017610.2.1	0	0	0	0	0	0	0	0	0	0	0	0	0
Solyc04g017620.2.1	2	14	4	5	7	45	7	22	8	18	4	11	1
Solyc04g017650.2.1	0	0	0	0	0	0	0	0	0	0	0	0	0
Solyc04g017670.2.1	0	0	0	0	0	0	0	0	0	0	0	0	0
Solyc04g017680.2.1	4	6	3	4	0	26	29	35	10	25	15	11	0
Solyc04g017690.2.1	8	825	164	638	51	2936	3229	2755	184	1931	317	877	13
Solyc04g017700.2.1	2	1	3	1	0	0	4	4	0	7	0	1	0
Solyc04g017710.2.1	6	141	6	95	8	263	125	290	28	213	51	123	3
Solyc04g017720.2.1	0	0	0	0	0	0	0	0	0	0	0	0	0
Solyc04g017750.2.1	22	144	12	97	12	134	156	179	38	122	20	76	0
Solyc04g018090.2.1	0	0	0	0	0	0	0	0	0	0	0	0	0
Solyc04g018100.2.1	1	1	2	12	0	68	7	34	0	6	2	0	0
Solyc04g018110.1.1	3	635	23	325	30	323	89	199	40	216	39	133	8
Solyc04g018140.1.1	0	0	0	0	0	0	0	0	0	0	0	0	0
Solyc04g024340.2.1	3	7	0	0	0	23	11	15	2	13	5	9	1
Solyc04g024420.2.1	0	0	0	0	0	0	0	0	0	0	0	0	0
Solyc04g024430.2.1	0	0	0	0	0	0	0	0	0	0	0	0	0
Solyc04g024500.2.1	1	11	0	1	0	18	5	11	0	22	1	7	0
Solyc04g024510.2.1	1	5	3	12	0	32	3	34	5	45	8	29	1
Solyc04g024520.2.1	7	37	9	34	0	33	63	56	17	33	11	24	5
Solyc04g024530.2.1	4	13	1	15	2	26	29	40	2	17	11	25	0
Solyc04g024590.2.1	4	33	4	14	0	76	50	79	6	76	9	33	0
Solyc04g024710.2.1	4	8	2	1	0	13	14	23	6	10	3	13	0
Solyc04g024780.1.1	0	0	0	0	0	0	0	0	0	0	0	0	0
Solyc04g024840.2.1	0	0	0	0	0	0	0	0	0	0	0	0	0
Solyc04g024940.2.1	6	27	7	15	1	119	39	69	9	70	7	37	1
Solyc04g025040.1.1	14	3506	67	1892	6	781	92	842	3	401	62	221	1
Solyc04g025120.1.1	0	0	0	0	0	0	0	0	0	0	0	0	0
Solyc04g025160.2.1	4	21	5	9	1	17	32	21	2	30	8	21	7
Solyc04g025170.2.1	2	16	0	15	0	48	4	31	1	39	5	35	0
Solyc04g025180.2.1	0	0	0	0	0	0	0	0	0	0	0	0	0
Solyc04g025200.2.1	0	0	0	0	0	0	0	0	0	0	0	0	0
Solyc04g025210.2.1	3	10	2	1	2	13	28	11	4	21	1	9	0
Solyc04g025260.2.1	7	36	3	32	1	96	91	127	19	80	20	65	3
Solyc04g025280.2.1	0	0	0	0	0	0	0	0	0	0	0	0	0

Solyc04g025290.2.1	5	54	4	18	5	39	33	45	0	37	8	12	0
Solyc04g025300.2.1	6	30	8	46	5	226	212	187	15	107	57	90	9
Solyc04g025370.1.1	0	0	0	0	0	0	0	0	0	0	0	0	0
Solyc04g025430.1.1	0	0	0	0	0	0	0	0	0	0	0	0	0
Solyc04g025440.2.1	0	0	0	0	0	0	0	0	0	0	0	0	0
Solyc04g025450.1.1	0	0	0	0	0	0	0	0	0	0	0	0	0
Solyc04g025530.2.1	0	0	0	0	0	0	0	0	0	0	0	0	0
Solyc04g025540.2.1	8	67	7	24	8	94	135	180	33	61	30	35	5
Solyc04g025560.2.1	0	0	0	0	0	0	0	0	0	0	0	0	0
Solyc04g025610.1.1	0	0	0	0	0	0	0	0	0	0	0	0	0
Solyc04g025650.2.1	5	260	27	134	5	8	9	25	5	4	36	36	10
Solyc04g025670.2.1	0	0	0	0	0	0	0	0	0	0	0	0	0
Solyc04g025870.2.1	11	79	9	31	6	117	72	193	19	176	16	65	0
Solyc04g025880.2.1	0	0	0	0	0	0	0	0	0	0	0	0	0
Solyc04g025940.2.1	21	84	18	77	7	521	322	693	40	322	42	152	1
Solyc04g025990.2.1	11	576	139	280	30	234	804	395	36	424	115	352	6
Solyc04g026020.2.1	1	5	0	8	0	0	4	0	0	7	0	4	0
Solyc04g026030.2.1	2	29	1	21	0	49	38	46	0	11	11	23	0
Solyc04g026100.1.1	0	0	0	0	0	0	0	0	0	0	0	0	0
Solyc04g026110.2.1	0	0	0	0	0	0	0	0	0	0	0	0	0
Solyc04g026200.2.1	0	0	0	0	0	0	0	0	0	0	0	0	0
Solyc04g026270.2.1	2	4	0	7	0	2	18	14	2	12	11	35	3
Solyc04g026280.2.1	2	5	1	11	0	8	8	8	1	14	4	21	0
Solyc04g026300.1.1	0	0	0	0	0	0	0	0	0	0	0	0	0
Solyc04g026350.2.1	3	66	10	45	9	116	105	234	19	88	50	86	7
Solyc04g026360.2.1	0	0	0	0	0	0	0	0	0	0	0	0	0
Solyc04g026380.2.1	4	29	3	7	1	15	92	38	7	21	22	35	3
Solyc04g028560.2.1	3	12	0	4	0	17	11	10	4	19	10	14	0
Solyc04g028570.1.1	1	2	0	0	0	3	9	5	0	0	0	5	0
Solyc04g028580.1.1	1	0	5	0	0	8	0	4	0	2	2	0	0
Solyc04g039620.2.1	1	3	0	7	0	12	8	7	1	2	0	8	0
Solyc04g039630.1.1	0	0	0	0	0	0	0	0	0	0	0	0	0
Solyc04g039640.2.1	0	0	0	0	0	0	0	0	0	0	0	0	0
Solyc04g039650.2.1	0	0	0	0	0	0	0	0	0	0	0	0	0
Solyc04g039670.2.1	0	0	0	0	0	0	0	0	0	0	0	0	0
Solyc04g039730.2.1	1	1	1	0	0	3	1	8	0	2	2	9	0
Solyc04g039760.1.1	0	0	0	0	0	0	0	0	0	0	0	0	0
Solyc04g039900.1.1	0	0	0	0	0	0	0	0	0	0	0	0	0
Solyc04g039930.2.1	0	0	0	0	0	0	0	0	0	0	0	0	0
Solyc04g039940.1.1	0	0	0	0	0	0	0	0	0	0	0	0	0
Solyc04g039950.2.1	14	46	11	29	6	101	79	152	12	109	24	104	1

Solyc04g039980.2.1	0	0	0	0	0	0	0	0	0	0	0	0	0
Solyc04g039990.2.1	0	0	0	0	0	0	0	0	0	0	0	0	0
Solyc04g040000.2.1	0	0	0	0	0	0	0	0	0	0	0	0	0
Solyc04g040070.1.1	0	0	0	0	0	0	0	0	0	0	0	0	0
Solyc04g040090.2.1	0	0	0	0	0	0	0	0	0	0	0	0	0
Solyc04g040110.2.1	2	17	2	11	4	5	8	8	1	24	0	5	0
Solyc04g040120.1.1	0	0	0	0	0	0	0	0	0	0	0	0	0
Solyc04g040130.1.1	0	0	0	0	0	0	0	0	0	0	0	0	0
Solyc04g040160.2.1	4	62	8	50	2	0	10	18	1	24	6	43	1
Solyc04g040170.2.1	0	0	0	0	0	0	0	0	0	0	0	0	0
Solyc04g040180.2.1	3	42	0	15	0	743	38	369	4	30	11	22	0
Solyc04g040190.1.1	6	106	3	49	1	43	29	92	0	7	4	28	0
Solyc04g040200.2.1	4	29	2	17	0	29	30	36	2	53	6	91	0
Solyc04g040210.2.1	1	39	4	15	0	22	18	43	0	21	1	78	0
Solyc04g040220.2.1	0	0	0	0	0	0	0	0	0	0	0	0	0
Solyc04g040230.1.1	0	0	0	0	0	0	0	0	0	0	0	0	0
Solyc04g045300.1.1	1	1	0	0	0	4	4	0	1	2	1	3	0
Solyc04g045310.2.1	0	0	0	0	0	0	0	0	0	0	0	0	0
Solyc04g045340.2.1	8	125	3	87	7	155	135	156	16	402	58	411	0
Solyc04g045430.2.1	6	40	4	20	12	68	37	81	3	91	8	45	2
Solyc04g045470.2.1	3	13	4	7	0	19	9	8	2	44	15	51	0
Solyc04g045480.2.1	5	69	2	42	2	179	69	143	17	140	15	110	0
Solyc04g045520.2.1	0	0	0	0	0	0	0	0	0	0	0	0	0
Solyc04g045530.2.1	0	0	0	0	0	0	0	0	0	0	0	0	0
Solyc04g045560.2.1	0	0	0	0	0	0	0	0	0	0	0	0	0
Solyc04g045580.2.1	2	17	2	5	0	0	26	8	0	18	0	17	0
Solyc04g045590.2.1	3	17	0	14	1	49	12	51	4	20	3	17	0
Solyc04g045600.2.1	3	33	3	15	2	3	27	4	1	35	7	32	0
Solyc04g045620.2.1	0	0	0	0	0	0	0	0	0	0	0	0	0
Solyc04g045630.2.1	4	30	4	35	0	120	22	78	3	139	12	57	0
Solyc04g045650.2.1	3	9	1	3	0	32	10	11	4	17	3	8	0
Solyc04g045660.2.1	2	1	0	4	1	21	26	18	1	7	2	4	0
Solyc04g047670.2.1	0	0	0	0	0	0	0	0	0	0	0	0	0
Solyc04g047680.1.1	0	0	0	0	0	0	0	0	0	0	0	0	0
Solyc04g047690.2.1	0	0	0	0	0	0	0	0	0	0	0	0	0
Solyc04g047720.2.1	4	26	1	15	2	92	67	63	5	81	15	43	1
Solyc04g047750.2.1	0	0	0	0	0	0	0	0	0	0	0	0	0
Solyc04g047770.2.1	16	135	8	51	11	297	119	317	23	279	43	188	0
Solyc04g048900.2.1	2	45	3	29	0	30	5	64	0	21	4	2	0
Solyc04g048950.2.1	1	7	0	0	0	24	5	18	0	35	1	15	0
Solyc04g049010.2.1	9	57	3	36	16	159	107	157	13	186	27	106	14

Solyc04g049070.2.1	18	207	27	95	16	664	387	786	82	322	89	286	4
Solyc04g049080.2.1	2	8	0	0	0	3	12	18	0	23	0	11	0
Solyc04g049090.2.1	3	36	0	1	4	207	16	82	44	0	0	0	0
Solyc04g049120.2.1	25	188	18	204	21	342	320	520	59	260	86	255	5
Solyc04g049130.2.1	5	18	11	22	2	24	122	50	3	35	20	12	1
Solyc04g049140.2.1	4	13	4	2	0	23	23	26	5	19	4	16	1
Solyc04g049150.1.1	0	0	0	0	0	0	0	0	0	0	0	0	0
Solyc04g049160.2.1	1	0	0	0	0	8	7	13	0	9	1	5	0
Solyc04g049180.2.1	4	50	10	18	1	63	42	89	33	186	38	107	8
Solyc04g049330.2.1	2	250	17	87	17	641	172	479	59	524	321	514	4
Solyc04g049340.2.1	0	0	0	0	0	0	0	0	0	0	0	0	0
Solyc04g049350.2.1	5	87	11	48	2	168	64	215	26	104	27	50	1
Solyc04g049360.2.1	7	47	1	68	0	40	24	75	1	85	16	80	2
Solyc04g049380.2.1	8	82	36	35	7	48	312	74	20	48	108	37	5
Solyc04g049390.2.1	9	150	27	92	8	74	205	110	18	58	44	35	2
Solyc04g049410.2.1	2	15	0	9	11	21	10	12	4	24	12	11	0
Solyc04g049450.2.1	16	154	36	74	8	528	273	433	79	238	175	224	10
Solyc04g049490.2.1	8	21	3	10	0	141	61	145	52	128	19	55	2
Solyc04g049550.2.1	1	3	3	0	0	6	1	3	2	4	5	0	0
Solyc04g049580.2.1	1	0	0	2	0	8	0	7	0	6	4	5	0
Solyc04g049620.2.1	0	0	0	0	0	0	0	0	0	0	0	0	0
Solyc04g049630.2.1	7	16	1	4	0	29	29	52	3	61	15	51	1
Solyc04g049660.1.1	1	11	0	7	0	13	6	16	2	17	3	14	0
Solyc04g049670.2.1	5	239	25	287	18	280	142	421	55	483	70	304	23
Solyc04g049680.2.1	3	13	3	25	3	14	26	39	8	25	10	40	5
Solyc04g049690.2.1	3	24	1	3	0	32	17	38	1	51	17	16	2
Solyc04g049710.2.1	3	22	2	21	0	40	13	37	2	49	8	30	0
Solyc04g049720.2.1	1	1	0	0	0	0	15	3	0	18	0	3	0
Solyc04g049760.2.1	7	16	5	11	1	30	23	39	6	88	12	50	2
Solyc04g049770.2.1	3	77	2	31	0	120	58	132	11	141	25	120	2
Solyc04g049790.2.1	1	5	1	1	0	4	4	10	0	26	0	12	0
Solyc04g049800.2.1	2	4	2	3	4	3	9	22	3	2	0	6	0
Solyc04g049840.2.1	7	4	7	4	0	27	23	23	3	12	19	54	0
Solyc04g049850.2.1	9	46	8	12	3	33	54	76	3	71	29	55	2
Solyc04g049860.1.1	0	0	0	0	0	0	0	0	0	0	0	0	0
Solyc04g049890.2.1	2	4	0	0	0	0	4	2	0	3	0	6	0
Solyc04g049910.2.1	1	7	1	5	0	6	2	6	0	25	9	14	0
Solyc04g049930.2.1	23	97	15	161	2	262	153	441	31	211	73	159	0
Solyc04g049940.2.1	2	16	2	8	3	46	20	40	9	22	10	21	0
Solyc04g049950.1.1	7	47	14	36	8	111	72	132	18	113	38	72	6
Solyc04g049970.2.1	3	5	1	3	0	32	11	17	6	5	1	9	1

Solyc04g050010.1.1	0	0	0	0	0	0	0	0	0	0	0	0	0
Solyc04g050040.2.1	7	42	8	27	2	42	64	73	4	67	5	33	0
Solyc04g050050.2.1	0	0	0	0	0	0	0	0	0	0	0	0	0
Solyc04g050140.2.1	4	16	1	5	0	24	38	16	4	4	3	11	0
Solyc04g050150.2.1	9	24	1	13	2	62	66	96	9	39	19	33	5
Solyc04g050160.2.1	1	3	1	4	0	8	7	12	1	1	0	14	0
Solyc04g050170.2.1	3	7	6	11	0	2	11	12	0	8	2	9	0
Solyc04g050190.2.1	7	44	8	17	6	57	44	94	10	74	13	100	0
Solyc04g050200.1.1	0	0	0	0	0	0	0	0	0	0	0	0	0
Solyc04g050240.2.1	2	22	3	8	0	34	34	28	10	21	11	42	0
Solyc04g050270.1.1	3	38	3	22	1	66	66	44	5	71	8	91	0
Solyc04g050280.1.1	0	0	0	0	0	0	0	0	0	0	0	0	0
Solyc04g050300.1.1	0	0	0	0	0	0	0	0	0	0	0	0	0
Solyc04g050310.2.1	1	8	0	7	3	0	1	36	0	8	5	16	0
Solyc04g050440.2.1	1	3	2	7	0	0	0	0	0	1	0	0	0
Solyc04g050470.2.1	2	58	0	9	0	27	41	14	0	34	8	31	0
Solyc04g050480.2.1	1	10	2	5	0	33	5	26	0	65	0	56	0
Solyc04g050490.2.1	4	60	3	0	6	13	54	15	38	55	3	14	24
Solyc04g050510.2.1	9	41	13	42	2	84	93	103	18	100	26	167	1
Solyc04g050520.2.1	8	14	3	17	0	116	42	141	11	45	20	74	0
Solyc04g050540.2.1	7	25	4	17	5	56	97	69	2	55	8	28	4
Solyc04g050550.1.1	1	1	0	1	0	0	4	3	0	4	0	10	0
Solyc04g050560.2.1	0	0	0	0	0	0	0	0	0	0	0	0	0
Solyc04g050570.2.1	0	0	0	0	0	0	0	0	0	0	0	0	0
Solyc04g050620.2.1	12	462	24	174	41	247	309	421	23	1478	27	488	16
Solyc04g050690.1.1	0	0	0	0	0	0	0	0	0	0	0	0	0
Solyc04g050710.1.1	0	0	0	0	0	0	0	0	0	0	0	0	0
Solyc04g050720.2.1	0	0	0	0	0	0	0	0	0	0	0	0	0
Solyc04g050730.2.1	0	0	0	0	0	0	0	0	0	0	0	0	0
Solyc04g050760.2.1	5	2	5	4	7	18	13	16	2	11	18	19	0
Solyc04g050780.1.1	0	0	0	0	0	0	0	0	0	0	0	0	0
Solyc04g050790.2.1	0	0	0	0	0	0	0	0	0	0	0	0	0
Solyc04g050820.1.1	0	0	0	0	0	0	0	0	0	0	0	0	0
Solyc04g050860.1.1	0	0	0	0	0	0	0	0	0	0	0	0	0
Solyc04g050920.2.1	4	33	5	13	4	0	34	21	1	122	6	28	2
Solyc04g050930.2.1	7	81	20	61	6	3	84	41	9	59	22	79	0
Solyc04g050960.2.1	12	49	14	60	0	28	28	97	4	181	30	150	2
Solyc04g050990.2.1	1	0	0	0	0	7	3	3	0	2	2	2	0
Solyc04g051110.1.1	0	0	0	0	0	0	0	0	0	0	0	0	0
Solyc04g051120.2.1	5	26	4	22	0	86	37	91	7	71	15	18	0
Solyc04g051150.2.1	1	2	3	1	1	5	10	2	3	7	1	1	0

Solyc04g051180.1.1	0	0	0	0	0	0	0	0	0	0	0	0	0
Solyc04g051190.2.1	11	100	19	54	6	189	87	225	9	48	17	35	0
Solyc04g051200.1.1	7	19	3	10	3	33	36	85	6	22	12	37	2
Solyc04g051280.2.1	6	1	2	0	0	114	1504	42	46	5	96	4	0
Solyc04g051310.2.1	7	66	7	33	0	136	67	213	21	83	19	94	3
Solyc04g051340.2.1	3	43	6	7	0	49	42	31	4	43	5	62	0
Solyc04g051350.2.1	3	9	1	6	0	0	5	5	4	3	2	6	0
Solyc04g051360.2.1	0	0	0	0	0	0	0	0	0	0	0	0	0
Solyc04g051370.2.1	8	262	26	224	11	3293	589	2102	120	573	174	441	9
Solyc04g051390.2.1	0	0	0	0	0	0	0	0	0	0	0	0	0
Solyc04g051400.1.1	0	0	0	0	0	0	0	0	0	0	0	0	0
Solyc04g051410.2.1	1	4	0	0	0	13	3	5	0	4	1	1	0
Solyc04g051450.2.1	3	12	0	14	0	12	22	24	4	9	2	22	0
Solyc04g051460.2.1	0	0	0	0	0	0	0	0	0	0	0	0	0
Solyc04g051470.1.1	3	28	2	76	4	54	1	21	0	364	16	268	3
Solyc04g051480.1.1	0	0	0	0	0	0	0	0	0	0	0	0	0
Solyc04g051490.2.1	0	0	0	0	0	0	0	0	0	0	0	0	0
Solyc04g051500.2.1	1	38	3	31	0	78	7	58	1	12	3	6	0
Solyc04g051510.1.1	21	299	27	195	19	262	277	379	20	307	53	170	0
Solyc04g051540.2.1	0	0	0	0	0	0	0	0	0	0	0	0	0
Solyc04g051550.1.1	1	6	0	0	0	5	15	8	0	1	0	3	0
Solyc04g051560.1.1	1	0	0	2	0	1	2	14	0	10	0	4	0
Solyc04g051570.2.1	13	81	3	62	9	231	78	179	9	131	18	104	3
Solyc04g051580.2.1	8	72	9	44	0	31	92	61	2	95	13	57	4
Solyc04g051600.2.1	0	0	0	0	0	0	0	0	0	0	0	0	0
Solyc04g051610.2.1	1	7	3	13	0	36	63	59	4	5	0	5	0
Solyc04g051640.2.1	0	0	0	0	0	0	0	0	0	0	0	0	0
Solyc04g051650.2.1	0	0	0	0	0	0	0	0	0	0	0	0	0
Solyc04g051660.2.1	1	0	7	8	0	40	64	64	2	20	12	21	0
Solyc04g051670.2.1	9	14	4	29	3	170	76	173	12	81	34	104	6
Solyc04g051680.2.1	0	0	0	0	0	0	0	0	0	0	0	0	0
Solyc04g051690.2.1	0	0	0	0	0	0	0	0	0	0	0	0	0
Solyc04g051700.2.1	5	43	3	30	0	54	25	49	12	60	5	59	0
Solyc04g051710.1.1	2	39	1	16	1	123	42	43	4	55	13	61	5
Solyc04g051730.1.1	0	0	0	0	0	0	0	0	0	0	0	0	0
Solyc04g051780.2.1	7	24	7	35	6	34	42	51	7	82	9	87	0
Solyc04g051800.2.1	11	1984	242	1298	16	2366	208	1404	28	296	89	233	2
Solyc04g051820.2.1	3	522	64	222	55	383	25	112	6	318	52	361	4
Solyc04g051830.2.1	3	4	1	2	0	7	12	29	5	28	14	26	1
Solyc04g051850.2.1	4	275	31	126	9	195	325	302	14	438	94	401	11
Solyc04g051860.2.1	4	16	0	12	4	51	37	51	4	16	14	14	0

Solyc04g051870.1.1	1	5	1	1	0	0	7	3	0	3	0	9	0
Solyc04g051880.1.1	0	0	0	0	0	0	0	0	0	0	0	0	0
Solyc04g053000.1.1	0	0	0	0	0	0	0	0	0	0	0	0	0
Solyc04g053050.2.1	0	0	0	0	0	0	0	0	0	0	0	0	0
Solyc04g053080.2.1	6	187	11	202	2	191	112	290	31	656	57	365	3
Solyc04g053110.1.1	0	0	0	0	0	0	0	0	0	0	0	0	0
Solyc04g053120.2.1	10	63	6	26	3	77	68	127	3	216	34	257	17
Solyc04g053130.2.1	4	205	65	112	15	712	226	746	10	270	57	321	8
Solyc04g054140.2.1	17	93	8	90	17	273	158	257	35	188	72	167	9
Solyc04g054150.1.1	0	0	0	0	0	0	0	0	0	0	0	0	0
Solyc04g054170.2.1	4	20	0	18	0	54	13	48	1	17	11	16	4
Solyc04g054180.2.1	2	1	1	11	0	25	9	17	0	19	2	15	0
Solyc04g054190.2.1	9	308	93	326	4	120	34	152	6	87	70	102	5
Solyc04g054200.2.1	4	8	0	7	1	12	8	23	3	10	9	9	0
Solyc04g054220.2.1	0	0	0	0	0	0	0	0	0	0	0	0	0
Solyc04g054250.2.1	0	0	0	0	0	0	0	0	0	0	0	0	0
Solyc04g054260.2.1	0	0	0	0	0	0	0	0	0	0	0	0	0
Solyc04g054270.2.1	1	3	0	0	1	0	4	7	0	4	3	4	0
Solyc04g054290.1.1	0	0	0	0	0	0	0	0	0	0	0	0	0
Solyc04g054310.2.1	13	57	12	35	2	149	271	181	5	196	51	96	10
Solyc04g054320.2.1	9	80	6	53	1	134	85	230	10	29	4	26	0
Solyc04g054330.2.1	2	26	1	13	0	23	15	50	4	17	3	14	0
Solyc04g054340.1.1	2	2	1	6	0	1	84	10	2	26	1	7	0
Solyc04g054370.1.1	11	204	2	117	5	459	458	787	61	235	38	108	2
Solyc04g054400.2.1	4	21	2	24	0	34	19	64	2	51	20	55	0
Solyc04g054410.2.1	6	18	1	11	0	52	20	31	5	12	1	12	0
Solyc04g054460.2.1	3	31	7	15	2	76	16	49	2	49	7	38	0
Solyc04g054470.2.1	19	89	11	71	3	248	411	323	77	114	188	191	7
Solyc04g054480.2.1	32	1847	59	1247	32	600	368	1233	32	8305	392	6666	19
Solyc04g054490.2.1	5	51	7	31	4	112	197	114	11	27	11	41	0
Solyc04g054500.2.1	1	31	0	9	0	0	4	1	0	35	0	19	0
Solyc04g054510.2.1	0	0	0	0	0	0	0	0	0	0	0	0	0
Solyc04g054520.2.1	0	0	0	0	0	0	0	0	0	0	0	0	0
Solyc04g054690.2.1	1	4	7	11	0	0	0	5	0	0	0	5	0
Solyc04g054700.2.1	6	18	3	24	0	39	43	44	8	66	13	54	1
Solyc04g054710.2.1	10	186	14	79	3	428	89	557	35	172	44	131	0
Solyc04g054730.2.1	0	0	0	0	0	0	0	0	0	0	0	0	0
Solyc04g054740.2.1	3	653	37	399	0	72	18	72	2	96	19	91	2
Solyc04g054760.2.1	0	0	0	0	0	0	0	0	0	0	0	0	0
Solyc04g054800.2.1	0	0	0	0	0	0	0	0	0	0	0	0	0
Solyc04g054810.2.1	4	685	25	400	62	398	105	393	34	1544	351	1990	20

Solyc04g054820.2.1	0	0	0	0	0	0	0	0	0	0	0	0	0
Solyc04g054830.2.1	0	0	0	0	0	0	0	0	0	0	0	0	0
Solyc04g054880.2.1	3	7	0	5	1	3	15	6	5	32	3	28	14
Solyc04g054890.2.1	10	28	7	23	8	96	84	82	12	32	49	17	1
Solyc04g054900.2.1	3	7	1	1	0	15	21	11	2	23	4	4	0
Solyc04g054910.2.1	6	306	45	272	32	331	291	522	88	232	38	182	26
Solyc04g054930.2.1	3	35	8	15	2	87	37	56	3	32	1	11	3
Solyc04g054950.2.1	0	0	0	0	0	0	0	0	0	0	0	0	0
Solyc04g054980.2.1	12	719	13	341	12	283	601	419	169	1220	99	1487	15
Solyc04g054990.2.1	6	81	10	28	15	115	563	198	146	73	112	46	8
Solyc04g055000.2.1	0	0	0	0	0	0	0	0	0	0	0	0	0
Solyc04g055010.2.1	4	19	1	18	3	30	13	25	5	57	9	20	0
Solyc04g055030.1.1	0	0	0	0	0	0	0	0	0	0	0	0	0
Solyc04g055050.1.1	0	0	0	0	0	0	0	0	0	0	0	0	0
Solyc04g055080.2.1	0	0	0	0	0	0	0	0	0	0	0	0	0
Solyc04g055090.1.1	2	10	1	5	0	2	0	1	0	13	11	17	5
Solyc04g055100.1.1	0	0	0	0	0	0	0	0	0	0	0	0	0
Solyc04g055110.2.1	2	6	0	4	0	6	2	18	0	24	10	19	0
Solyc04g055120.2.1	21	144	17	142	31	575	388	635	29	201	77	278	7
Solyc04g055160.2.1	3	15	0	5	2	17	7	11	1	18	26	13	0
Solyc04g055170.2.1	4	90	4	76	3	470	78	451	35	818	96	537	23
Solyc04g055180.2.1	5	17	3	7	0	32	18	36	4	31	7	11	0
Solyc04g055190.2.1	0	0	0	0	0	0	0	0	0	0	0	0	0
Solyc04g055200.2.1	5	58	1	48	3	263	5	196	1	328	22	186	0
Solyc04g055210.2.1	1	0	0	0	0	2	1	6	0	7	4	3	1
Solyc04g055220.1.1	0	0	0	0	0	0	0	0	0	0	0	0	0
Solyc04g055250.2.1	0	0	0	0	0	0	0	0	0	0	0	0	0
Solyc04g055260.2.1	0	0	0	0	0	0	0	0	0	0	0	0	0
Solyc04g056260.1.1	0	0	0	0	0	0	0	0	0	0	0	0	0
Solyc04g056270.2.1	20	175	15	172	9	217	158	456	35	293	57	232	1
Solyc04g056280.2.1	9	61	1	42	0	55	50	128	16	147	16	102	2
Solyc04g056290.2.1	4	13	0	2	0	33	21	26	4	20	5	8	0
Solyc04g056310.2.1	0	0	0	0	0	0	0	0	0	0	0	0	0
Solyc04g056320.1.1	2	10	6	6	0	14	0	17	0	43	3	24	0
Solyc04g056330.1.1	3	26	4	18	0	18	13	7	4	10	3	18	2
Solyc04g056340.2.1	1	2	3	2	0	4	8	1	0	1	1	14	0
Solyc04g056350.1.1	2	89	4	26	8	120	63	105	12	300	29	225	3
Solyc04g056370.2.1	1	0	0	0	0	1	0	0	0	14	5	13	0
Solyc04g056380.2.1	0	0	0	0	0	0	0	0	0	0	0	0	0
Solyc04g056390.2.1	8	637	16	130	5	267	93	456	8	253	52	237	0
Solyc04g056400.2.1	0	0	0	0	0	0	0	0	0	0	0	0	0

Solyc04g056410.2.1	0	0	0	0	0	0	0	0	0	0	0	0	0
Solyc04g056430.2.1	3	16	0	16	0	14	16	16	3	26	7	23	0
Solyc04g056450.2.1	1	0	1	0	10	9	10	2	0	0	1	0	0
Solyc04g056530.1.1	0	0	0	0	0	0	0	0	0	0	0	0	0
Solyc04g056540.2.1	8	16	1	18	0	69	54	108	1	63	4	23	0
Solyc04g056560.2.1	5	59	5	55	4	56	26	58	1	47	26	43	1
Solyc04g056570.2.1	0	0	0	0	0	0	0	0	0	0	0	0	0
Solyc04g056580.2.1	7	379	45	237	0	114	266	212	9	241	36	257	6
Solyc04g056590.2.1	2	6	0	3	0	3	0	10	0	11	7	4	0
Solyc04g056600.2.1	3	6	4	0	0	28	11	13	3	13	6	18	5
Solyc04g056610.2.1	1	0	1	0	0	4	7	2	2	4	1	0	0
Solyc04g056630.2.1	9	106	17	128	14	130	120	176	34	233	51	172	3
Solyc04g056640.1.1	3	1	0	1	0	0	7	4	0	10	0	17	0
Solyc04g056650.2.1	6	36	2	17	1	96	45	72	2	53	13	51	0
Solyc04g057780.2.1	0	0	0	0	0	0	0	0	0	0	0	0	0
Solyc04g057870.2.1	5	71	8	57	1	44	59	91	1	55	7	65	1
Solyc04g057880.2.1	7	17	17	41	3	218	246	116	33	47	19	43	0
Solyc04g057890.2.1	5	88	3	61	1	147	25	130	5	119	45	103	7
Solyc04g057930.2.1	17	106	6	109	33	767	243	329	27	453	90	304	9
Solyc04g057940.2.1	0	0	0	0	0	0	0	0	0	0	0	0	0
Solyc04g057950.1.1	0	0	0	0	0	0	0	0	0	0	0	0	0
Solyc04g057960.2.1	17	54	31	51	5	95	473	180	56	109	37	77	9
Solyc04g057970.2.1	1	0	0	1	0	0	2	2	1	6	0	2	0
Solyc04g057980.2.1	7	188	51	86	0	8	48	26	0	1	8	54	0
Solyc04g057990.2.1	10	22	2	22	0	65	38	45	7	37	20	17	1
Solyc04g058000.1.1	0	0	0	0	0	0	0	0	0	0	0	0	0
Solyc04g058060.1.1	2	136	23	54	12	139	118	154	41	171	115	94	17
Solyc04g058070.2.1	6	18	1	2	1	29	26	33	2	82	9	37	0
Solyc04g058080.2.1	0	0	0	0	0	0	0	0	0	0	0	0	0
Solyc04g058090.2.1	13	66	10	34	5	145	156	226	16	164	79	146	12
Solyc04g058100.2.1	0	0	0	0	0	0	0	0	0	0	0	0	0
Solyc04g058110.2.1	0	0	0	0	0	0	0	0	0	0	0	0	0
Solyc04g058130.2.1	1	2	0	2	0	10	3	6	0	6	1	2	0
Solyc04g058140.2.1	8	39	4	35	9	96	85	100	10	71	27	48	1
Solyc04g058150.2.1	1	686	95	307	0	37	609	65	2	219	50	304	0
Solyc04g058160.1.1	0	0	0	0	0	0	0	0	0	0	0	0	0
Solyc04g058170.1.1	0	0	0	0	0	0	0	0	0	0	0	0	0
Solyc04g058180.2.1	3	0	0	0	0	0	1	1	0	29	20	29	0
Solyc04g058190.2.1	0	0	0	0	0	0	0	0	0	0	0	0	0
Solyc04g058200.2.1	0	0	0	0	0	0	0	0	0	0	0	0	0
Solyc04g063200.1.1	0	0	0	0	0	0	0	0	0	0	0	0	0

Solyc04g063210.2.1	4	77	1	26	0	248	13	191	0	34	6	24	0
Solyc04g063230.2.1	22	541	37	314	26	92	131	368	18	1263	158	1313	11
Solyc04g063240.2.1	3	107	6	46	1	0	5	8	0	8	10	9	0
Solyc04g063270.1.1	0	0	0	0	0	0	0	0	0	0	0	0	0
Solyc04g063280.2.1	10	207	12	132	2	751	229	455	57	331	114	265	24
Solyc04g063290.2.1	10	527	21	232	6	1283	239	1256	61	913	199	637	7
Solyc04g063300.1.1	0	0	0	0	0	0	0	0	0	0	0	0	0
Solyc04g063320.1.1	0	0	0	0	0	0	0	0	0	0	0	0	0
Solyc04g063340.1.1	0	0	0	0	0	0	0	0	0	0	0	0	0
Solyc04g063350.2.1	13	51	10	30	56	1035	1313	1099	75	106	27	16	0
Solyc04g063360.2.1	21	45	5	67	10	165	185	286	20	130	42	105	4
Solyc04g063370.1.1	0	0	0	0	0	0	0	0	0	0	0	0	0
Solyc04g063380.2.1	7	40	5	34	4	266	187	341	16	80	24	64	0
Solyc04g063390.2.1	0	0	0	0	0	0	0	0	0	0	0	0	0
Solyc04g063400.1.1	0	0	0	0	0	0	0	0	0	0	0	0	0
Solyc04g064470.1.1	0	0	0	0	0	0	0	0	0	0	0	0	0
Solyc04g064480.2.1	2	1	0	0	0	1	19	4	3	2	0	0	0
Solyc04g064490.2.1	10	64	8	33	3	36	37	85	6	144	28	98	0
Solyc04g064500.2.1	9	212	13	102	17	329	196	423	34	219	56	123	3
Solyc04g064510.2.1	1	34	1	20	0	45	10	67	0	55	8	30	0
Solyc04g064530.1.1	11	176	26	106	5	85	36	67	12	35	4	25	0
Solyc04g064540.2.1	0	0	0	0	0	0	0	0	0	0	0	0	0
Solyc04g064550.1.1	12	213	23	169	9	157	155	312	26	69	23	56	1
Solyc04g064560.2.1	2	2	1	0	0	11	3	13	0	2	4	4	0
Solyc04g064570.2.1	7	36	2	19	2	70	59	99	6	124	36	117	2
Solyc04g064590.1.1	2	7	4	1	3	5	7	3	0	2	0	9	3
Solyc04g064610.2.1	0	0	0	0	0	0	0	0	0	0	0	0	0
Solyc04g064620.2.1	0	0	0	0	0	0	0	0	0	0	0	0	0
Solyc04g064630.2.1	0	0	0	0	0	0	0	0	0	0	0	0	0
Solyc04g064640.2.1	0	0	0	0	0	0	0	0	0	0	0	0	0
Solyc04g064650.1.1	0	0	0	0	0	0	0	0	0	0	0	0	0
Solyc04g064660.1.1	0	0	0	0	0	0	0	0	0	0	0	0	0
Solyc04g064670.2.1	4	44	4	51	0	9	25	9	0	23	19	47	0
Solyc04g064680.2.1	1	11	0	12	1	19	4	19	0	20	2	28	0
Solyc04g064690.2.1	1	0	0	0	0	0	0	0	0	9	6	8	0
Solyc04g064700.2.1	13	212	12	170	10	520	363	635	99	436	74	291	3
Solyc04g064710.2.1	4	0	0	0	0	38	9	36	18	0	14	0	0
Solyc04g064720.2.1	1	18	0	16	0	2	3	10	0	5	1	7	0
Solyc04g064740.2.1	4	23	2	6	1	114	41	89	19	3	1	0	0
Solyc04g064750.1.1	0	0	0	0	0	0	0	0	0	0	0	0	0
Solyc04g064760.2.1	1	4	0	0	0	0	1	10	0	0	5	0	0

Solyc04g064770.1.1	0	0	0	0	0	0	0	0	0	0	0	0	0
Solyc04g064790.2.1	4	28	11	39	1	87	97	79	1	52	34	58	1
Solyc04g064800.2.1	5	36	4	37	0	114	53	69	11	118	29	44	4
Solyc04g064810.2.1	6	70	9	27	4	143	35	132	21	70	19	93	1
Solyc04g064820.2.1	9	59	7	38	0	118	104	134	38	130	15	145	0
Solyc04g064830.2.1	0	0	0	0	0	0	0	0	0	0	0	0	0
Solyc04g064850.2.1	2	11	0	6	0	22	26	31	7	21	0	12	0
Solyc04g064860.1.1	0	0	0	0	0	0	0	0	0	0	0	0	0
Solyc04g064870.2.1	5	59	6	29	0	41	74	59	0	16	11	7	0
Solyc04g064880.2.1	0	0	0	0	0	0	0	0	0	0	0	0	0
Solyc04g064890.2.1	0	0	0	0	0	0	0	0	0	0	0	0	0
Solyc04g064900.1.1	0	0	0	0	0	0	0	0	0	0	0	0	0
Solyc04g064930.2.1	6	156	8	64	0	13	28	35	0	39	14	65	3
Solyc04g064940.2.1	0	0	0	0	0	0	0	0	0	0	0	0	0
Solyc04g070960.1.1	0	0	0	0	0	0	0	0	0	0	0	0	0
Solyc04g070970.2.1	0	0	0	0	0	0	0	0	0	0	0	0	0
Solyc04g070980.2.1	16	1568	98	301	8	1228	110	1029	17	555	106	290	2
Solyc04g070990.2.1	6	45	1	29	7	58	33	91	8	94	10	68	0
Solyc04g071010.2.1	6	40	6	40	0	75	30	97	6	36	19	23	1
Solyc04g071030.1.1	5	84	16	52	3	25	13	35	9	34	5	56	0
Solyc04g071040.2.1	10	123	12	44	5	222	65	127	21	175	36	76	4
Solyc04g071050.2.1	1	2	0	1	0	11	6	11	0	2	1	1	0
Solyc04g071070.2.1	15	237	79	62	114	76	1458	182	185	22	118	20	5
Solyc04g071080.1.1	0	0	0	0	0	0	0	0	0	0	0	0	0
Solyc04g071090.2.1	0	0	0	0	0	0	0	0	0	0	0	0	0
Solyc04g071100.1.1	0	0	0	0	0	0	0	0	0	0	0	0	0
Solyc04g071120.2.1	4	3	1	4	0	6	25	14	1	20	0	4	0
Solyc04g071130.1.1	0	0	0	0	0	0	0	0	0	0	0	0	0
Solyc04g071140.2.1	9	273	20	258	0	191	235	480	12	829	42	366	7
Solyc04g071150.2.1	0	0	0	0	0	0	0	0	0	0	0	0	0
Solyc04g071160.2.1	5	51	9	16	4	99	67	116	12	134	15	116	0
Solyc04g071180.2.1	0	0	0	0	0	0	0	0	0	0	0	0	0
Solyc04g071260.2.1	0	0	0	0	0	0	0	0	0	0	0	0	0
Solyc04g071280.1.1	0	0	0	0	0	0	0	0	0	0	0	0	0
Solyc04g071290.2.1	1	5	1	0	0	12	6	14	1	2	0	3	0
Solyc04g071310.2.1	2	7	0	2	0	8	19	19	0	16	5	6	0
Solyc04g071320.2.1	17	103	9	59	6	110	93	165	23	117	51	61	3
Solyc04g071330.2.1	0	0	0	0	0	0	0	0	0	0	0	0	0
Solyc04g071340.2.1	5	277	57	169	0	8	41	17	0	99	86	152	2
Solyc04g071350.2.1	24	181	26	117	12	592	322	622	74	302	117	247	2
Solyc04g071360.2.1	8	108	7	50	9	44	29	39	22	261	22	194	0

Solyc04g071400.2.1	0	0	0	0	0	0	0	0	0	0	0	0	0
Solyc04g071420.2.1	2	14	3	13	0	19	6	13	1	14	1	14	0
Solyc04g071430.1.1	0	0	0	0	0	0	0	0	0	0	0	0	0
Solyc04g071440.2.1	6	40	14	25	6	53	38	53	20	87	14	75	2
Solyc04g071470.2.1	0	0	0	0	0	0	0	0	0	0	0	0	0
Solyc04g071480.1.1	5	439	119	241	14	114	25	70	7	76	22	114	11
Solyc04g071490.2.1	0	0	0	0	0	0	0	0	0	0	0	0	0
Solyc04g071500.2.1	0	0	0	0	0	0	0	0	0	0	0	0	0
Solyc04g071510.2.1	1	4	0	0	0	4	0	9	0	10	0	6	0
Solyc04g071540.2.1	5	30	1	19	0	21	93	46	11	21	2	13	10
Solyc04g071550.2.1	3	17	1	6	0	31	15	20	0	19	7	15	2
Solyc04g071560.2.1	6	88	4	21	0	37	77	67	7	18	12	18	0
Solyc04g071570.2.1	6	16	1	11	4	27	21	32	5	33	6	31	0
Solyc04g071580.2.1	1	1	0	0	0	3	0	1	2	1	3	22	7
Solyc04g071590.1.1	0	0	0	0	0	0	0	0	0	0	0	0	0
Solyc04g071600.2.1	0	0	0	0	0	0	0	0	0	0	0	0	0
Solyc04g071610.2.1	2	121	6	91	6	827	346	797	152	4128	269	4602	58
Solyc04g071620.2.1	12	1384	136	363	504	12471	13389	23953	4215	805	272	209	26
Solyc04g071630.2.1	0	0	0	0	0	0	0	0	0	0	0	0	0
Solyc04g071650.2.1	19	1367	40	474	113	121	233	305	169	2615	438	1127	10
Solyc04g071660.2.1	1	1	0	1	0	4	7	8	1	1	8	3	0
Solyc04g071670.1.1	1	132	2	603	46	374	26	113	14	67	10	28	0
Solyc04g071680.1.1	0	0	0	0	0	0	0	0	0	0	0	0	0
Solyc04g071700.2.1	3	11	0	7	3	10	21	34	0	5	2	4	0
Solyc04g071710.2.1	2	19	4	18	0	26	24	28	13	17	31	9	1
Solyc04g071730.2.1	3	8	0	18	0	31	13	42	0	14	4	35	0
Solyc04g071750.2.1	4	27	1	10	0	51	35	34	0	22	3	18	1
Solyc04g071770.2.1	12	1	0	4	0	833	174	330	29	5	82	3	0
Solyc04g071780.2.1	3	7	0	16	0	378	5	258	2	5	11	0	0
Solyc04g071790.1.1	0	0	0	0	0	0	0	0	0	0	0	0	0
Solyc04g071800.2.1	3	462	24	739	3	42	10	84	0	741	61	1418	5
Solyc04g071820.2.1	0	0	0	0	0	0	0	0	0	0	0	0	0
Solyc04g071860.2.1	3	93	1	91	2	129	44	102	18	82	5	77	0
Solyc04g071870.1.1	8	58	1	47	1	82	46	72	5	55	16	47	0
Solyc04g071880.2.1	13	85	12	47	7	170	206	269	45	161	73	140	4
Solyc04g071890.2.1	8	401	11	120	17	82	106	131	62	255	4	328	10
Solyc04g071900.2.1	5	191	6	27	19	222	56	164	22	18	5	46	0
Solyc04g071930.2.1	6	181	21	78	1	47	73	80	3	100	15	107	1
Solyc04g071940.2.1	7	165	2	49	0	138	72	123	2	70	27	41	0
Solyc04g071960.2.1	4	23	5	7	0	0	15	6	0	2	10	4	0
Solyc04g071970.2.1	3	26	8	25	0	45	53	53	1	61	1	33	1

Solyc04g071980.2.1	5	24	4	5	0	109	32	125	11	52	9	55	0
Solyc04g071990.2.1	23	136	20	66	8	627	799	1338	97	435	36	334	9
Solyc04g072000.2.1	1	3	0	3	1	24	8	37	3	0	0	0	0
Solyc04g072010.2.1	6	34	6	4	3	19	12	20	0	36	10	30	3
Solyc04g072020.2.1	1	6	0	0	0	2	4	2	1	0	0	5	0
Solyc04g072030.1.1	6	51	1	64	0	38	10	84	3	179	8	140	0
Solyc04g072050.2.1	3	42	6	17	1	3	18	6	9	70	19	74	11
Solyc04g072060.2.1	1	10	2	3	0	15	10	10	4	11	2	7	0
Solyc04g072070.2.1	0	0	0	0	0	0	0	0	0	0	0	0	0
Solyc04g072110.2.1	3	63	2	24	0	209	2	121	5	169	10	96	0
Solyc04g072120.2.1	5	100	9	54	0	298	50	203	4	257	14	179	1
Solyc04g072140.2.1	2	8	1	4	0	3	8	15	0	60	7	61	0
Solyc04g072150.1.1	0	0	0	0	0	0	0	0	0	0	0	0	0
Solyc04g072160.2.1	6	12	4	13	5	61	36	14	3	135	171	278	4
Solyc04g072170.1.1	0	0	0	0	0	0	0	0	0	0	0	0	0
Solyc04g072180.1.1	0	0	0	0	0	0	0	0	0	0	0	0	0
Solyc04g072190.2.1	0	0	0	0	0	0	0	0	0	0	0	0	0
Solyc04g072200.1.1	7	12	5	3	1	14	28	35	5	21	18	20	0
Solyc04g072220.2.1	13	112	14	82	10	141	70	163	29	126	41	157	0
Solyc04g072230.2.1	5	27	3	11	0	24	12	32	2	29	7	29	0
Solyc04g072240.2.1	6	99	4	63	3	98	55	110	2	74	1	49	0
Solyc04g072250.2.1	0	0	0	0	0	0	0	0	0	0	0	0	0
Solyc04g072260.1.1	14	13	3	33	2	100	79	101	9	56	22	47	0
Solyc04g072270.1.1	0	0	0	0	0	0	0	0	0	0	0	0	0
Solyc04g072280.2.1	2	0	0	0	0	43	9	45	0	0	0	0	0
Solyc04g072290.2.1	11	83	6	24	5	214	65	189	23	149	20	178	0
Solyc04g072310.2.1	0	0	0	0	0	0	0	0	0	0	0	0	0
Solyc04g072360.1.1	0	0	0	0	0	0	0	0	0	0	0	0	0
Solyc04g072380.2.1	0	0	0	0	0	0	0	0	0	0	0	0	0
Solyc04g072390.2.1	10	45	8	38	2	101	60	207	15	125	28	80	1
Solyc04g072400.2.1	5	15	9	14	0	45	182	94	4	27	19	44	0
Solyc04g072410.2.1	2	23	4	12	0	28	61	49	11	58	27	35	2
Solyc04g072440.2.1	2	7	0	4	6	10	19	9	13	17	4	4	2
Solyc04g072450.2.1	10	233	27	81	10	134	146	225	46	342	76	160	21
Solyc04g072460.2.1	4	10	2	5	0	59	43	38	0	55	2	46	0
Solyc04g072470.2.1	0	0	0	0	0	0	0	0	0	0	0	0	0
Solyc04g072490.2.1	0	0	0	0	0	0	0	0	0	0	0	0	0
Solyc04g072500.1.1	0	0	0	0	0	0	0	0	0	0	0	0	0
Solyc04g072510.2.1	1	11	0	0	0	4	4	7	1	14	0	11	0
Solyc04g072520.1.1	1	3	0	2	3	3	3	4	1	1	1	0	0
Solyc04g072530.2.1	0	0	0	0	0	0	0	0	0	0	0	0	0

Solyc04g072540.2.1	3	6	2	0	2	9	16	7	0	2	3	7	0
Solyc04g072550.1.1	0	0	0	0	0	0	0	0	0	0	0	0	0
Solyc04g072560.2.1	17	302	24	192	12	662	393	568	143	447	76	568	9
Solyc04g072570.2.1	9	58	11	42	20	41	28	72	9	115	15	83	9
Solyc04g072580.1.1	0	0	0	0	0	0	0	0	0	0	0	0	0
Solyc04g072590.1.1	0	0	0	0	0	0	0	0	0	0	0	0	0
Solyc04g072600.1.1	0	0	0	0	0	0	0	0	0	0	0	0	0
Solyc04g072620.1.1	0	0	0	0	0	0	0	0	0	0	0	0	0
Solyc04g072630.2.1	20	76	9	68	10	83	105	119	23	117	40	148	4
Solyc04g072640.2.1	1	18	0	8	0	26	6	19	1	16	0	11	1
Solyc04g072660.2.1	4	122	19	52	15	384	314	282	46	396	212	274	23
Solyc04g072670.2.1	23	243	31	128	11	401	266	631	60	324	68	331	2
Solyc04g072690.2.1	0	0	0	0	0	0	0	0	0	0	0	0	0
Solyc04g072700.2.1	3	6	0	0	0	78	47	48	3	0	0	0	0
Solyc04g072720.2.1	0	0	0	0	0	0	0	0	0	0	0	0	0
Solyc04g072740.2.1	8	235	38	168	0	124	28	292	0	9	0	1	0
Solyc04g072760.2.1	2	1	0	2	0	8	8	2	24	0	3	0	1
Solyc04g072770.2.1	2	12	1	4	0	6	7	12	0	13	1	19	0
Solyc04g072780.2.1	4	340	10	275	11	358	129	443	2	852	38	701	13
Solyc04g072790.1.1	0	0	0	0	0	0	0	0	0	0	0	0	0
Solyc04g072800.2.1	0	0	0	0	0	0	0	0	0	0	0	0	0
Solyc04g072810.2.1	10	228	21	135	17	489	443	351	31	818	41	466	4
Solyc04g072820.1.1	2	8	4	9	4	18	20	13	1	8	9	25	0
Solyc04g072830.2.1	13	40	2	53	8	98	66	118	18	62	32	57	0
Solyc04g072840.2.1	3	4	4	3	0	11	14	52	2	12	6	3	0
Solyc04g072850.2.1	13	109	12	81	16	5	35	51	1	514	67	332	6
Solyc04g072860.2.1	3	3	1	3	0	25	19	13	4	8	1	7	0
Solyc04g072870.1.1	0	0	0	0	0	0	0	0	0	0	0	0	0
Solyc04g072880.2.1	2	23	7	14	1	74	18	36	13	32	6	28	0
Solyc04g072890.2.1	18	80	14	54	25	1021	344	248	201	769	224	869	15
Solyc04g072900.1.1	8	660	34	383	38	287	78	134	24	167	24	79	9
Solyc04g072910.2.1	7	64	3	14	9	139	72	190	29	64	27	46	4
Solyc04g072920.2.1	2	10	1	1	0	35	20	8	0	16	3	31	0
Solyc04g072930.1.1	4	15	1	18	2	27	37	26	3	42	12	27	0
Solyc04g072940.2.1	0	0	0	0	0	0	0	0	0	0	0	0	0
Solyc04g073940.2.1	1	1	0	0	0	9	7	10	0	0	0	0	0
Solyc04g073950.1.1	5	24	2	19	0	97	38	45	24	53	80	28	7
Solyc04g073960.2.1	2	3	1	3	1	5	4	8	7	10	1	14	0
Solyc04g073970.2.1	5	27	1	9	3	24	20	24	6	66	24	35	1
Solyc04g073980.2.1	0	0	0	0	0	0	0	0	0	0	0	0	0
Solyc04g073990.2.1	8	153	15	86	10	904	293	925	79	537	99	1004	32

Solyc04g074000.2.1	0	0	0	0	0	0	0	0	0	0	0	0	0
Solyc04g074020.2.1	0	0	0	0	0	0	0	0	0	0	0	0	0
Solyc04g074030.2.1	0	0	0	0	0	0	0	0	0	0	0	0	0
Solyc04g074040.2.1	5	190	17	125	4	242	187	383	24	356	54	376	9
Solyc04g074050.2.1	0	0	0	0	0	0	0	0	0	0	0	0	0
Solyc04g074070.1.1	0	0	0	0	0	0	0	0	0	0	0	0	0
Solyc04g074080.2.1	1	1	0	0	2	3	1	0	0	0	2	0	2
Solyc04g074090.2.1	10	56	4	16	0	82	35	113	10	107	17	109	2
Solyc04g074100.2.1	3	18	0	8	2	50	8	30	2	7	4	8	0
Solyc04g074110.2.1	1	3	0	0	0	0	0	0	2	4	5	8	1
Solyc04g074120.2.1	0	0	0	0	0	0	0	0	0	0	0	0	0
Solyc04g074130.2.1	1	5	3	5	0	0	0	2	0	1	0	2	0
Solyc04g074140.2.1	9	11	1	11	0	70	64	95	4	100	15	27	0
Solyc04g074150.1.1	3	7	2	4	0	15	10	6	1	20	18	41	0
Solyc04g074160.1.1	0	0	0	0	0	0	0	0	0	0	0	0	0
Solyc04g074180.2.1	14	239	37	234	9	194	146	343	4	124	94	136	4
Solyc04g074190.2.1	0	0	0	0	0	0	0	0	0	0	0	0	0
Solyc04g074220.2.1	2	22	1	6	0	9	12	17	0	6	2	6	0
Solyc04g074230.2.1	4	283	6	116	4	914	161	1366	44	436	88	309	15
Solyc04g074240.2.1	4	173	32	136	12	251	192	205	31	237	63	248	9
Solyc04g074250.1.1	0	0	0	0	0	0	0	0	0	0	0	0	0
Solyc04g074270.2.1	9	213	30	109	13	755	71	343	27	85	32	90	1
Solyc04g074280.1.1	1	0	0	0	0	6	2	1	1	0	2	5	0
Solyc04g074290.2.1	10	161	17	122	32	92	62	90	26	222	52	265	5
Solyc04g074300.2.1	5	598	32	224	42	1435	399	1296	95	1212	333	1128	15
Solyc04g074310.2.1	0	0	0	0	0	0	0	0	0	0	0	0	0
Solyc04g074330.2.1	0	0	0	0	0	0	0	0	0	0	0	0	0
Solyc04g074340.2.1	0	0	0	0	0	0	0	0	0	0	0	0	0
Solyc04g074350.2.1	0	0	0	0	0	0	0	0	0	0	0	0	0
Solyc04g074400.1.1	0	0	0	0	0	0	0	0	0	0	0	0	0
Solyc04g074410.1.1	3	243	8	63	21	0	6	10	0	16	0	79	6
Solyc04g074420.1.1	2	704	29	288	35	63	5	74	7	108	4	207	22
Solyc04g074430.1.1	4	272	34	60	41	7	7	18	5	34	3	19	10
Solyc04g074440.1.1	7	278	69	117	65	4	1	1	13	37	2	79	12
Solyc04g074450.1.1	6	756	65	142	29	3	6	5	0	31	1	88	2
Solyc04g074470.1.1	3	40	8	4	7	6	63	4	106	1	3	5	13
Solyc04g074480.2.1	5	106	6	63	3	529	114	495	43	98	13	109	2
Solyc04g074490.2.1	13	180	21	143	7	1015	138	396	19	314	24	226	1
Solyc04g074500.2.1	10	27	7	2	0	60	64	70	11	31	15	23	0
Solyc04g074510.2.1	4	350	32	240	30	319	146	345	24	461	72	363	3
Solyc04g074520.1.1	9	75	10	56	1	143	68	118	4	89	6	63	1

Solyc04g074530.2.1	4	12	3	14	0	29	8	53	1	38	4	26	1
Solyc04g074540.2.1	0	0	0	0	0	0	0	0	0	0	0	0	0
Solyc04g074550.2.1	3	73	3	43	4	347	32	255	6	76	28	126	7
Solyc04g074560.1.1	1	6	2	13	0	5	4	13	4	15	17	15	4
Solyc04g074570.1.1	2	24	3	29	4	58	54	51	12	14	16	37	0
Solyc04g074580.1.1	5	488	3	140	26	366	122	346	34	355	88	198	7
Solyc04g074590.2.1	0	0	0	0	0	0	0	0	0	0	0	0	0
Solyc04g074630.2.1	1	1	5	20	0	7	11	7	1	19	0	2	0
Solyc04g074640.2.1	5	120	21	55	0	5	9	2	0	2	16	0	0
Solyc04g074650.2.1	0	0	0	0	0	0	0	0	0	0	0	0	0
Solyc04g074660.1.1	1	1	0	2	0	10	3	6	0	3	3	1	0
Solyc04g074670.2.1	5	10	0	5	5	57	12	32	2	40	29	23	0
Solyc04g074680.1.1	3	56	42	50	11	14	3	15	1	0	0	13	0
Solyc04g074690.2.1	0	0	0	0	0	0	0	0	0	0	0	0	0
Solyc04g074700.2.1	0	0	0	0	0	0	0	0	0	0	0	0	0
Solyc04g074710.2.1	0	0	0	0	0	0	0	0	0	0	0	0	0
Solyc04g074720.2.1	4	12	1	8	0	17	10	25	1	29	10	8	0
Solyc04g074730.1.1	7	560	27	327	11	3079	494	1086	58	612	80	485	3
Solyc04g074740.2.1	3	39	2	26	0	14	4	8	0	4	9	1	0
Solyc04g074750.2.1	3	18	0	4	0	0	27	13	0	13	5	4	0
Solyc04g074760.2.1	0	0	0	0	0	0	0	0	0	0	0	0	0
Solyc04g074770.2.1	1	4	1	2	1	2	5	5	1	3	1	0	4
Solyc04g074790.1.1	1	2	1	0	0	1	8	1	2	1	0	6	0
Solyc04g074800.2.1	4	21	2	12	0	25	16	21	2	88	14	32	1
Solyc04g074820.1.1	0	0	0	0	0	0	0	0	0	0	0	0	0
Solyc04g074830.1.1	0	0	0	0	0	0	0	0	0	0	0	0	0
Solyc04g074840.2.1	0	0	0	0	0	0	0	0	0	0	0	0	0
Solyc04g074850.2.1	15	48	3	12	2	329	91	233	0	84	165	59	0
Solyc04g074860.2.1	3	3	0	2	0	10	11	7	0	15	12	14	1
Solyc04g074870.2.1	4	32	1	6	8	5	24	15	8	30	5	30	3
Solyc04g074890.2.1	0	0	0	0	0	0	0	0	0	0	0	0	0
Solyc04g074900.2.1	0	0	0	0	0	0	0	0	0	0	0	0	0
Solyc04g074910.2.1	1	93	79	46	45	141	772	134	59	283	222	168	31
Solyc04g074920.2.1	0	0	0	0	0	0	0	0	0	0	0	0	0
Solyc04g074930.2.1	0	0	0	0	0	0	0	0	0	0	0	0	0
Solyc04g074940.2.1	2	30	1	7	0	21	12	28	6	32	11	55	0
Solyc04g074950.2.1	5	63	30	9	12	24	11	4	10	0	12	21	0
Solyc04g074960.2.1	1	3	1	5	1	0	3	1	5	3	5	0	0
Solyc04g074980.2.1	10	74	11	87	5	46	19	58	4	61	31	52	6
Solyc04g074990.2.1	1	2	0	0	0	17	5	5	0	4	1	0	0
Solyc04g075000.1.1	0	0	0	0	0	0	0	0	0	0	0	0	0

Solyc04g076010.2.1	7	64	14	64	5	173	79	203	12	190	32	226	5
Solyc04g076020.2.1	9	48	1	17	4	52	104	92	15	161	25	175	12
Solyc04g076030.1.1	13	196	10	117	38	412	260	511	79	233	74	194	49
Solyc04g076040.2.1	2	23	1	11	0	18	10	14	0	14	6	16	0
Solyc04g076050.2.1	9	77	2	33	3	312	50	166	6	261	59	206	0
Solyc04g076060.2.1	4	29	2	14	2	46	88	136	6	242	21	208	2
Solyc04g076070.1.1	4	15	1	8	1	38	36	48	4	57	13	36	8
Solyc04g076080.2.1	1	6	1	13	0	7	2	4	1	7	1	7	0
Solyc04g076090.2.1	10	222	11	117	21	133	108	148	28	136	43	100	0
Solyc04g076100.2.1	2	1	1	1	0	3	6	1	0	5	4	16	0
Solyc04g076110.2.1	3	27	1	29	0	28	24	33	4	56	13	21	0
Solyc04g076120.2.1	0	0	0	0	0	0	0	0	0	0	0	0	0
Solyc04g076130.2.1	0	0	0	0	0	0	0	0	0	0	0	0	0
Solyc04g076140.2.1	0	0	0	0	0	0	0	0	0	0	0	0	0
Solyc04g076150.2.1	0	0	0	0	0	0	0	0	0	0	0	0	0
Solyc04g076180.2.1	4	18	1	10	0	6	19	29	1	28	15	25	0
Solyc04g076190.1.1	0	0	0	0	0	0	0	0	0	0	0	0	0
Solyc04g076200.2.1	2	39	4	10	0	18	36	36	3	67	8	29	4
Solyc04g076210.2.1	29	364	66	346	18	742	286	677	74	368	212	323	7
Solyc04g076220.2.1	2	6	1	9	0	25	6	15	0	5	4	1	0
Solyc04g076230.2.1	8	118	14	49	1	247	89	257	16	231	30	172	2
Solyc04g076250.2.1	0	0	0	0	0	0	0	0	0	0	0	0	0
Solyc04g076260.1.1	0	0	0	0	0	0	0	0	0	0	0	0	0
Solyc04g076270.1.1	8	83	5	37	13	80	87	135	15	181	31	78	1
Solyc04g076280.2.1	4	68	6	16	0	115	10	54	1	0	1	0	0
Solyc04g076290.2.1	4	13	3	9	2	24	33	34	5	113	12	76	4
Solyc04g076300.2.1	22	135	33	120	2	359	383	543	18	51	9	31	0
Solyc04g076310.2.1	0	0	0	0	0	0	0	0	0	0	0	0	0
Solyc04g076320.2.1	6	34	4	30	0	66	44	88	6	50	17	43	0
Solyc04g076330.1.1	0	0	0	0	0	0	0	0	0	0	0	0	0
Solyc04g076340.2.1	1	26	0	1	0	15	13	22	1	16	0	16	0
Solyc04g076350.1.1	0	0	0	0	0	0	0	0	0	0	0	0	0
Solyc04g076360.2.1	1	0	0	1	0	16	9	8	0	4	4	13	0
Solyc04g076370.2.1	2	0	1	0	0	3	9	6	2	9	1	2	0
Solyc04g076380.2.1	18	494	76	340	38	1092	644	1176	146	1102	197	698	6
Solyc04g076390.2.1	11	63	8	36	1	40	65	62	2	64	16	41	0
Solyc04g076400.2.1	0	0	0	0	0	0	0	0	0	0	0	0	0
Solyc04g076410.2.1	2	11	0	1	0	11	2	7	2	2	3	9	0
Solyc04g076420.2.1	7	36	8	7	1	89	67	75	19	68	44	35	3
Solyc04g076430.2.1	9	35	1	18	1	54	45	74	14	51	15	40	0
Solyc04g076450.1.1	2	4	0	0	0	4	2	10	0	4	1	15	0

Solyc04g076460.2.1	2	3	1	0	2	7	5	10	0	6	8	12	0
Solyc04g076470.1.1	5	58	2	23	0	66	22	60	4	55	6	75	0
Solyc04g076480.2.1	0	0	0	0	0	0	0	0	0	0	0	0	0
Solyc04g076490.2.1	0	0	0	0	0	0	0	0	0	0	0	0	0
Solyc04g076500.2.1	3	3	0	0	0	20	10	32	0	7	4	14	0
Solyc04g076510.2.1	17	65	12	44	2	206	126	256	10	125	26	162	5
Solyc04g076520.1.1	10	92	17	92	6	123	63	252	9	133	27	108	7
Solyc04g076530.2.1	12	80	4	73	0	111	73	118	3	185	9	159	3
Solyc04g076540.2.1	18	64	11	84	10	277	117	167	19	283	49	189	0
Solyc04g076550.2.1	1	2	1	1	0	0	6	19	0	7	3	7	0
Solyc04g076560.2.1	1	1	2	0	0	3	13	15	0	12	0	2	0
Solyc04g076570.2.1	1	2	0	0	0	6	4	4	0	1	1	0	0
Solyc04g076580.2.1	3	21	5	19	0	21	42	42	12	29	6	7	1
Solyc04g076600.1.1	0	0	0	0	0	0	0	0	0	0	0	0	0
Solyc04g076610.1.1	9	9	4	3	0	48	28	93	36	34	3	15	1
Solyc04g076620.2.1	60	604	106	488	83	1330	1237	2063	196	1701	347	1186	29
Solyc04g076630.2.1	0	0	0	0	0	0	0	0	0	0	0	0	0
Solyc04g076650.1.1	0	0	0	0	0	0	0	0	0	0	0	0	0
Solyc04g076660.2.1	7	15	4	10	3	44	23	54	3	8	11	6	0
Solyc04g076670.2.1	3	41	2	33	0	68	52	92	7	27	23	29	1
Solyc04g076690.2.1	2	7	0	3	0	71	29	28	2	13	7	12	0
Solyc04g076700.2.1	0	0	0	0	0	0	0	0	0	0	0	0	0
Solyc04g076710.2.1	0	0	0	0	0	0	0	0	0	0	0	0	0
Solyc04g076740.2.1	6	21	3	30	0	5	79	48	2	74	7	56	2
Solyc04g076750.1.1	0	0	0	0	0	0	0	0	0	0	0	0	0
Solyc04g076770.2.1	0	0	0	0	0	0	0	0	0	0	0	0	0
Solyc04g076780.2.1	7	193	3	107	0	87	58	112	3	597	15	380	1
Solyc04g076790.2.1	7	117	16	113	8	116	77	145	23	691	68	360	3
Solyc04g076800.2.1	2	3	2	0	2	4	3	3	1	3	0	2	0
Solyc04g076810.2.1	6	51	1	31	0	99	39	233	6	164	10	80	0
Solyc04g076820.1.1	22	401	50	202	29	1256	1209	1428	408	424	171	301	22
Solyc04g076830.2.1	4	73	4	22	3	203	105	187	11	19	9	7	0
Solyc04g076850.2.1	10	696	42	491	16	530	245	981	40	1188	205	1127	12
Solyc04g076860.2.1	2	1	0	0	0	11	1	9	0	5	11	9	0
Solyc04g076870.2.1	14	499	60	251	16	58	50	152	10	202	95	302	1
Solyc04g076880.2.1	7	128	5	34	3	143	580	347	43	144	16	170	16
Solyc04g076890.2.1	4	57	12	25	0	169	77	79	12	75	17	84	3
Solyc04g076900.1.1	1	5	0	1	0	8	6	10	0	8	0	5	0
Solyc04g076910.2.1	1	3	0	3	0	6	2	18	1	5	4	0	0
Solyc04g076920.2.1	2	14	0	7	0	38	6	15	2	43	10	23	0
Solyc04g076940.2.1	0	0	0	0	0	0	0	0	0	0	0	0	0

Solyc04g076950.2.1	1	6	0	2	0	4	5	2	0	0	0	2	0
Solyc04g076960.2.1	3	24	1	4	0	74	26	38	0	21	11	12	1
Solyc04g076970.2.1	0	0	0	0	0	0	0	0	0	0	0	0	0
Solyc04g076980.2.1	6	63	15	58	12	84	124	107	28	88	15	121	3
Solyc04g076990.2.1	11	171	15	179	1	450	154	297	41	196	70	122	5
Solyc04g077000.2.1	2	1	0	4	0	13	10	21	2	4	5	4	0
Solyc04g077010.2.1	17	252	52	150	0	247	443	684	3	123	22	84	2
Solyc04g077020.2.1	8	3050	285	1450	104	1115	978	2231	61	9328	599	6477	38
Solyc04g077030.2.1	4	13	4	7	0	38	50	48	5	27	11	29	1
Solyc04g077040.2.1	14	81	7	63	6	207	93	151	7	175	32	145	11
Solyc04g077050.2.1	11	119	4	40	0	683	197	645	6	72	5	99	2
Solyc04g077060.2.1	6	28	6	13	0	66	41	71	4	83	33	76	4
Solyc04g077070.1.1	0	0	0	0	0	0	0	0	0	0	0	0	0
Solyc04g077100.2.1	0	0	0	0	0	0	0	0	0	0	0	0	0
Solyc04g077120.2.1	7	79	8	72	7	175	65	107	8	44	8	39	2
Solyc04g077130.1.1	4	50	13	16	0	7	51	21	1	16	11	31	0
Solyc04g077140.2.1	0	0	0	0	0	0	0	0	0	0	0	0	0
Solyc04g077150.2.1	0	0	0	0	0	0	0	0	0	0	0	0	0
Solyc04g077160.1.1	4	54	5	9	3	40	14	41	8	144	12	150	2
Solyc04g077170.2.1	0	0	0	0	0	0	0	0	0	0	0	0	0
Solyc04g077180.1.1	0	0	0	0	0	0	0	0	0	0	0	0	0
Solyc04g077190.2.1	1	6	1	6	0	6	23	19	0	3	0	1	0
Solyc04g077210.2.1	0	0	0	0	0	0	0	0	0	0	0	0	0
Solyc04g077220.2.1	1	1	0	0	0	30	7	4	0	0	1	3	0
Solyc04g077230.1.1	0	0	0	0	0	0	0	0	0	0	0	0	0
Solyc04g077240.2.1	0	0	0	0	0	0	0	0	0	0	0	0	0
Solyc04g077270.2.1	6	98	25	98	0	4	0	10	0	3	0	1	0
Solyc04g077280.2.1	0	0	0	0	0	0	0	0	0	0	0	0	0
Solyc04g077290.1.1	0	0	0	0	0	0	0	0	0	0	0	0	0
Solyc04g077300.2.1	0	0	0	0	0	0	0	0	0	0	0	0	0
Solyc04g077310.2.1	0	0	0	0	0	0	0	0	0	0	0	0	0
Solyc04g077320.1.1	0	0	0	0	0	0	0	0	0	0	0	0	0
Solyc04g077330.2.1	0	0	0	0	0	0	0	0	0	0	0	0	0
Solyc04g077340.2.1	3	23	13	9	1	2	1	0	0	0	0	5	0
Solyc04g077350.1.1	0	0	0	0	0	0	0	0	0	0	0	0	0
Solyc04g077360.2.1	5	20	4	12	2	20	19	7	11	2	12	2	0
Solyc04g077370.1.1	0	0	0	0	0	0	0	0	0	0	0	0	0
Solyc04g077390.2.1	0	0	0	0	0	0	0	0	0	0	0	0	0
Solyc04g077400.2.1	0	0	0	0	0	0	0	0	0	0	0	0	0
Solyc04g077420.2.1	1	8	2	0	0	10	3	11	0	8	0	2	0
Solyc04g077430.2.1	2	9	0	9	3	13	27	22	4	3	13	21	0

Solyc04g077440.2.1	7	450	52	198	31	59	119	94	25	345	181	208	2
Solyc04g077450.2.1	0	0	0	0	0	0	0	0	0	0	0	0	0
Solyc04g077460.2.1	0	0	0	0	0	0	0	0	0	0	0	0	0
Solyc04g077470.2.1	5	383	36	131	33	83	40	28	0	215	47	127	0
Solyc04g077480.2.1	0	0	0	0	0	0	0	0	0	0	0	0	0
Solyc04g077490.2.1	0	0	0	0	0	0	0	0	0	0	0	0	0
Solyc04g077500.2.1	9	378	171	303	0	41	87	70	4	43	32	115	2
Solyc04g077510.2.1	2	2	0	0	0	2	0	7	0	0	12	0	0
Solyc04g077520.2.1	2	24	0	7	0	97	14	48	2	40	6	18	1
Solyc04g077540.2.1	0	0	0	0	0	0	0	0	0	0	0	0	0
Solyc04g077560.2.1	8	29	2	10	1	38	28	64	3	55	20	25	0
Solyc04g077570.1.1	0	0	0	0	0	0	0	0	0	0	0	0	0
Solyc04g077580.2.1	0	0	0	0	0	0	0	0	0	0	0	0	0
Solyc04g077590.2.1	7	86	9	45	8	52	35	69	9	26	16	47	3
Solyc04g077600.2.1	6	17	1	18	4	43	31	30	6	82	16	41	0
Solyc04g077610.2.1	6	42	6	29	11	11	15	17	2	53	4	38	12
Solyc04g077620.1.1	1	12	0	6	0	11	4	2	0	7	0	0	0
Solyc04g077630.2.1	0	0	0	0	0	0	0	0	0	0	0	0	0
Solyc04g077640.2.1	0	0	0	0	0	0	0	0	0	0	0	0	0
Solyc04g077650.2.1	13	12	13	27	0	1964	1007	1427	48	715	772	279	0
Solyc04g077660.2.1	2	6	0	5	0	8	1	4	7	6	3	4	0
Solyc04g077670.2.1	1	39	0	14	0	18	16	27	2	123	21	77	0
Solyc04g077680.2.1	4	24	1	24	1	39	6	34	1	26	3	33	0
Solyc04g077690.2.1	0	0	0	0	0	0	0	0	0	0	0	0	0
Solyc04g077740.1.1	2	0	0	0	0	91	21	21	0	9	7	6	0
Solyc04g077760.1.1	0	0	0	0	0	0	0	0	0	0	0	0	0
Solyc04g077770.2.1	0	0	0	0	0	0	0	0	0	0	0	0	0
Solyc04g077780.2.1	1	26	1	19	0	0	14	7	0	77	3	71	1
Solyc04g077790.2.1	7	21	4	10	0	41	60	43	3	98	24	98	1
Solyc04g077810.1.1	0	0	0	0	0	0	0	0	0	0	0	0	0
Solyc04g077820.2.1	9	43	6	14	5	97	45	103	5	66	19	44	0
Solyc04g077830.2.1	0	0	0	0	0	0	0	0	0	0	0	0	0
Solyc04g077840.2.1	0	0	0	0	0	0	0	0	0	0	0	0	0
Solyc04g077850.2.1	5	24	4	19	0	27	24	21	10	21	8	12	0
Solyc04g077860.2.1	2	1	4	1	1	3	15	5	4	6	4	0	0
Solyc04g077870.2.1	10	44	1	31	3	99	78	224	10	37	11	8	0
Solyc04g077920.2.1	7	103	8	57	6	261	64	198	24	70	34	56	4
Solyc04g077930.2.1	9	13	2	18	0	86	39	98	4	77	23	70	0
Solyc04g077940.2.1	18	42	9	38	3	114	85	147	23	178	24	144	1
Solyc04g077950.2.1	2	2	2	0	0	7	1	3	0	3	2	3	0
Solyc04g077970.2.1	6	198	17	82	7	301	152	402	39	822	167	536	12

Solyc04g077980.1.1	14	1371	390	1116	247	239	345	62	174	268	7	306	50
Solyc04g077990.2.1	0	0	0	0	0	0	0	0	0	0	0	0	0
Solyc04g078000.2.1	2	12	0	1	0	24	9	10	0	1	8	0	0
Solyc04g078040.2.1	0	0	0	0	0	0	0	0	0	0	0	0	0
Solyc04g078050.2.1	7	15	0	10	3	44	33	46	17	30	5	17	1
Solyc04g078060.1.1	1	6	0	4	0	57	21	35	3	18	9	22	0
Solyc04g078070.2.1	0	0	0	0	0	0	0	0	0	0	0	0	0
Solyc04g078090.2.1	20	31	7	38	12	254	507	441	50	156	84	112	3
Solyc04g078100.1.1	0	0	0	0	0	0	0	0	0	0	0	0	0
Solyc04g078110.1.1	5	94	19	114	4	15	7	73	0	44	7	11	0
Solyc04g078120.2.1	1	0	0	0	0	0	4	0	1	4	0	7	0
Solyc04g078130.2.1	5	26	8	9	2	29	17	33	0	38	8	36	2
Solyc04g078140.2.1	3	26	0	12	0	1	0	3	0	0	7	0	0
Solyc04g078150.2.1	0	0	0	0	0	0	0	0	0	0	0	0	0
Solyc04g078160.2.1	3	10	1	9	3	105	9	47	1	27	2	23	1
Solyc04g078190.2.1	1	3	0	5	0	5	9	3	0	3	0	0	2
Solyc04g078200.2.1	2	176	86	116	43	36	393	73	4	439	152	326	10
Solyc04g078210.1.1	0	0	0	0	0	0	0	0	0	0	0	0	0
Solyc04g078250.2.1	6	9	0	22	0	29	27	23	5	30	5	30	0
Solyc04g078270.2.1	0	0	0	0	0	0	0	0	0	0	0	0	0
Solyc04g078290.2.1	6	97	6	109	0	422	70	380	115	31	2	8	1
Solyc04g078310.2.1	0	0	0	0	0	0	0	0	0	0	0	0	0
Solyc04g078320.2.1	9	74	4	28	0	143	113	123	19	103	21	72	0
Solyc04g078340.2.1	0	0	0	0	0	0	0	0	0	0	0	0	0
Solyc04g078370.2.1	0	0	0	0	0	0	0	0	0	0	0	0	0
Solyc04g078380.1.1	4	64	1	36	0	41	9	96	2	88	3	86	0
Solyc04g078390.1.1	2	35	7	26	1	34	5	39	2	88	2	38	1
Solyc04g078400.2.1	6	32	2	33	6	56	35	71	2	147	24	197	2
Solyc04g078420.1.1	9	237	8	114	1	351	131	163	25	205	25	128	2
Solyc04g078450.2.1	1	0	0	0	0	152	37	35	13	3	5	1	0
Solyc04g078460.2.1	5	167	0	104	0	14	7	6	0	95	38	18	0
Solyc04g078470.2.1	0	0	0	0	0	0	0	0	0	0	0	0	0
Solyc04g078480.2.1	4	38	0	29	0	178	29	70	1	25	1	13	0
Solyc04g078490.1.1	6	71	7	40	1	82	31	97	12	78	14	63	4
Solyc04g078500.1.1	0	0	0	0	0	0	0	0	0	0	0	0	0
Solyc04g078510.2.1	3	38	3	14	11	48	14	44	2	35	11	48	0
Solyc04g078520.2.1	0	0	0	0	0	0	0	0	0	0	0	0	0
Solyc04g078540.2.1	13	131	6	72	3	146	78	180	18	165	23	74	5
Solyc04g078550.2.1	8	132	37	79	12	52	23	57	9	24	12	35	0
Solyc04g078560.1.1	0	0	0	0	0	0	0	0	0	0	0	0	0
Solyc04g078590.2.1	0	0	0	0	0	0	0	0	0	0	0	0	0

Solyc04g078600.2.1	3	46	0	25	1	55	14	55	0	94	13	107	0
Solyc04g078610.2.1	17	300	25	253	27	115	244	223	69	373	94	516	4
Solyc04g078620.2.1	1	10	0	0	0	11	4	16	0	14	0	15	0
Solyc04g078630.2.1	2	5	3	3	2	10	10	16	4	2	0	1	0
Solyc04g078640.1.1	4	4	1	5	0	110	30	73	1	15	4	11	0
Solyc04g078650.2.1	0	0	0	0	0	0	0	0	0	0	0	0	0
Solyc04g078660.1.1	0	0	0	0	0	0	0	0	0	0	0	0	0
Solyc04g078670.2.1	1	0	0	0	0	0	6	1	1	4	0	3	0
Solyc04g078680.2.1	12	94	12	76	11	164	109	133	48	75	44	93	4
Solyc04g078690.2.1	5	39	2	20	1	44	24	53	4	6	6	6	0
Solyc04g078700.2.1	9	98	8	31	4	75	88	112	18	61	17	46	2
Solyc04g078710.1.1	0	0	0	0	0	0	0	0	0	0	0	0	0
Solyc04g078730.1.1	0	0	0	0	0	0	0	0	0	0	0	0	0
Solyc04g078740.2.1	10	71	3	75	2	97	96	86	4	2	4	3	0
Solyc04g078750.2.1	4	147	1	72	1	9	1	20	1	52	13	40	0
Solyc04g078760.2.1	9	99	12	50	2	145	65	112	8	59	34	50	1
Solyc04g078770.2.1	0	0	0	0	0	0	0	0	0	0	0	0	0
Solyc04g078780.2.1	5	8	5	4	5	48	48	34	7	27	19	11	0
Solyc04g078790.2.1	0	0	0	0	0	0	0	0	0	0	0	0	0
Solyc04g078800.2.1	0	0	0	0	0	0	0	0	0	0	0	0	0
Solyc04g078810.2.1	2	15	1	0	0	10	1	15	1	3	12	4	0
Solyc04g078820.2.1	6	52	1	68	4	174	107	272	69	130	19	120	10
Solyc04g078830.2.1	13	88	9	48	3	136	99	151	4	81	26	88	9
Solyc04g078840.2.1	12	52	6	59	7	1396	464	1065	133	81	28	44	0
Solyc04g078850.2.1	5	21	1	3	2	47	52	88	3	8	17	16	0
Solyc04g078860.2.1	1	0	0	1	0	1	4	1	0	3	0	3	0
Solyc04g078870.2.1	8	65	3	47	4	182	68	72	13	49	4	43	7
Solyc04g078880.2.1	8	75	1	30	9	35	47	71	13	150	21	105	5
Solyc04g078890.2.1	7	18	6	4	3	25	22	22	3	20	2	16	1
Solyc04g078900.2.1	1	10	1	11	0	5	12	9	0	7	0	24	0
Solyc04g078910.2.1	6	83	9	73	0	92	49	66	2	140	2	91	0
Solyc04g078920.2.1	2	26	2	3	0	109	17	84	2	71	4	64	7
Solyc04g078930.2.1	0	0	0	0	0	0	0	0	0	0	0	0	0
Solyc04g078940.2.1	1	0	0	5	0	6	6	5	1	0	0	0	0
Solyc04g078950.1.1	4	35	7	25	1	32	27	56	20	21	16	18	0
Solyc04g078960.1.1	0	0	0	0	0	0	0	0	0	0	0	0	0
Solyc04g078970.1.1	4	12	3	5	0	21	34	29	2	4	0	13	0
Solyc04g078980.2.1	0	0	0	0	0	0	0	0	0	0	0	0	0
Solyc04g078990.2.1	0	0	0	0	0	0	0	0	0	0	0	0	0
Solyc04g079000.2.1	3	26	2	21	0	20	37	38	1	17	2	31	0
Solyc04g079030.2.1	0	0	0	0	0	0	0	0	0	0	0	0	0

Solyc04g079040.2.1	2	12	2	0	3	0	23	1	1	0	0	4	0
Solyc04g079050.1.1	11	280	28	194	23	5	69	2	20	56	114	53	4
Solyc04g079060.2.1	5	100	5	72	1	305	35	295	2	143	11	163	0
Solyc04g079070.2.1	0	0	0	0	0	0	0	0	0	0	0	0	0
Solyc04g079090.2.1	9	48	8	19	8	116	131	160	21	134	17	54	5
Solyc04g079100.2.1	12	33	8	35	12	78	75	148	11	117	22	102	1
Solyc04g079110.1.1	3	17	7	24	0	19	30	5	0	1	0	3	0
Solyc04g079120.2.1	5	53	4	20	5	44	11	20	5	10	20	6	0
Solyc04g079140.2.1	3	8	3	5	1	6	30	25	1	13	14	8	3
Solyc04g079150.1.1	0	0	0	0	0	0	0	0	0	0	0	0	0
Solyc04g079160.2.1	3	11	0	9	0	57	26	33	7	31	9	21	8
Solyc04g079170.2.1	1	1	0	0	0	4	2	11	1	12	3	9	0
Solyc04g079180.2.1	4	84	8	59	6	243	129	223	11	451	48	377	3
Solyc04g079190.2.1	1	9	0	7	0	10	6	61	3	6	0	5	0
Solyc04g079200.2.1	1	19	2	6	0	67	32	26	1	13	11	8	1
Solyc04g079210.2.1	0	0	0	0	0	0	0	0	0	0	0	0	0
Solyc04g079220.1.1	0	0	0	0	0	0	0	0	0	0	0	0	0
Solyc04g079230.2.1	0	0	0	0	0	0	0	0	0	0	0	0	0
Solyc04g079240.2.1	0	0	0	0	0	0	0	0	0	0	0	0	0
Solyc04g079250.2.1	0	0	0	0	0	0	0	0	0	0	0	0	0
Solyc04g079260.2.1	0	0	0	0	0	0	0	0	0	0	0	0	0
Solyc04g079270.2.1	10	168	4	140	3	1015	137	480	12	498	91	408	3
Solyc04g079280.2.1	0	0	0	0	0	0	0	0	0	0	0	0	0
Solyc04g079290.2.1	0	0	0	0	0	0	0	0	0	0	0	0	0
Solyc04g079300.2.1	15	25	0	28	3	891	205	810	76	35	28	24	0
Solyc04g079310.2.1	5	32	1	37	5	105	33	160	8	62	5	68	2
Solyc04g079320.2.1	6	46	1	19	0	57	24	62	1	70	13	78	3
Solyc04g079340.2.1	5	31	0	19	0	3	22	15	0	38	15	49	1
Solyc04g079350.1.1	1	5	1	8	3	17	4	0	1	5	0	2	0
Solyc04g079360.1.1	0	0	0	0	0	0	0	0	0	0	0	0	0
Solyc04g079380.1.1	0	0	0	0	0	0	0	0	0	0	0	0	0
Solyc04g079390.2.1	1	1	1	0	0	6	6	4	2	0	0	1	0
Solyc04g079400.2.1	4	24	1	8	0	60	17	24	2	1	7	2	1
Solyc04g079410.2.1	0	0	0	0	0	0	0	0	0	0	0	0	0
Solyc04g079420.2.1	19	375	67	331	8	1183	359	1053	110	103	60	95	5
Solyc04g079430.2.1	9	95	13	60	7	141	95	102	14	235	87	254	3
Solyc04g079440.2.1	10	87	6	78	1	208	76	261	24	195	25	159	4
Solyc04g079450.2.1	0	0	0	0	0	0	0	0	0	0	0	0	0
Solyc04g079470.2.1	1	0	0	0	0	64	5	25	10	3	0	1	0
Solyc04g079480.2.1	1	5	1	2	0	3	2	3	0	0	1	0	0
Solyc04g079520.2.1	0	0	0	0	0	0	0	0	0	0	0	0	0

Solyc04g079530.2.1	0	0	0	0	0	0	0	0	0	0	0	0	0
Solyc04g079540.2.1	9	39	2	25	3	69	54	65	14	79	21	52	1
Solyc04g079550.2.1	5	200	17	74	4	146	113	246	14	213	40	136	7
Solyc04g079560.2.1	4	29	5	18	1	7	12	44	0	129	19	90	4
Solyc04g079570.1.1	0	0	0	0	0	0	0	0	0	0	0	0	0
Solyc04g079590.2.1	4	13	2	14	3	4	14	31	5	24	24	46	0
Solyc04g079600.2.1	0	0	0	0	0	0	0	0	0	0	0	0	0
Solyc04g079640.2.1	1	16	1	29	0	19	4	19	1	15	3	15	0
Solyc04g079660.2.1	2	2	0	0	0	31	65	102	16	0	0	0	0
Solyc04g079680.2.1	1	0	0	3	0	7	5	6	0	0	1	1	0
Solyc04g079690.2.1	2	6	1	4	0	15	10	15	1	21	4	11	1
Solyc04g079700.2.1	2	12	2	8	0	6	4	0	0	0	2	0	0
Solyc04g079710.2.1	4	4	3	0	0	11	12	20	18	8	0	3	0
Solyc04g079720.2.1	12	238	4	222	4	235	51	300	7	531	27	272	2
Solyc04g079730.1.1	4	17	11	4	4	4	4	3	2	5	1	3	0
Solyc04g079740.2.1	2	6	1	0	0	2	25	14	1	5	1	1	0
Solyc04g079750.2.1	2	1	0	2	0	5	6	5	0	3	0	4	0
Solyc04g079760.2.1	0	0	0	0	0	0	0	0	0	0	0	0	0
Solyc04g079770.2.1	2	5	2	0	0	0	2	7	0	2	1	9	0
Solyc04g079780.2.1	0	0	0	0	0	0	0	0	0	0	0	0	0
Solyc04g079790.2.1	7	146	9	71	5	76	120	58	6	108	19	101	2
Solyc04g079800.2.1	0	0	0	0	0	0	0	0	0	0	0	0	0
Solyc04g079810.1.1	1	2	0	0	0	3	6	6	0	10	1	9	0
Solyc04g079820.2.1	0	0	0	0	0	0	0	0	0	0	0	0	0
Solyc04g079830.2.1	9	160	20	56	2	182	118	241	45	13	2	8	0
Solyc04g079840.2.1	3	8	1	2	0	28	20	17	6	3	1	3	0
Solyc04g079850.1.1	1	0	0	0	0	5	3	4	1	6	1	3	0
Solyc04g079860.1.1	2	6	4	14	0	3	4	6	1	5	2	3	0
Solyc04g079870.1.1	0	0	0	0	0	0	0	0	0	0	0	0	0
Solyc04g079880.2.1	1	5	1	3	0	2	1	3	0	9	0	5	0
Solyc04g079890.2.1	0	0	0	0	0	0	0	0	0	0	0	0	0
Solyc04g079900.2.1	10	72	8	38	1	54	34	120	13	289	26	214	1
Solyc04g079910.2.1	0	0	0	0	0	0	0	0	0	0	0	0	0
Solyc04g079920.2.1	9	30	6	43	0	111	78	126	13	55	27	48	9
Solyc04g079930.2.1	5	8	0	5	2	9	31	25	4	30	6	29	0
Solyc04g079940.2.1	9	165	4	110	5	1053	80	500	11	199	8	188	1
Solyc04g079950.2.1	2	1	0	0	0	5	8	10	8	0	1	4	0
Solyc04g079960.1.1	4	21	0	47	0	51	27	39	0	1	9	13	5
Solyc04g079970.2.1	3	832	51	543	8	649	247	947	38	4970	216	4025	41
Solyc04g079980.2.1	5	62	1	39	4	46	53	70	2	88	4	36	3
Solyc04g079990.2.1	2	4	0	0	0	0	7	1	2	10	3	2	2

Solyc04g080000.1.1	1	4	1	5	0	5	15	9	3	8	2	12	0
Solyc04g080010.2.1	2	44	8	14	0	0	0	3	0	12	1	2	0
Solyc04g080020.2.1	7	83	12	63	17	80	69	131	13	162	31	91	8
Solyc04g080030.2.1	0	0	0	0	0	0	0	0	0	0	0	0	0
Solyc04g080040.2.1	0	0	0	0	0	0	0	0	0	0	0	0	0
Solyc04g080050.2.1	2	9	0	5	0	23	9	30	0	24	4	4	0
Solyc04g080060.2.1	0	0	0	0	0	0	0	0	0	0	0	0	0
Solyc04g080070.1.1	0	0	0	0	0	0	0	0	0	0	0	0	0
Solyc04g080080.2.1	9	158	17	92	8	266	105	295	14	615	31	475	19
Solyc04g080090.2.1	2	8	1	0	0	8	5	9	0	2	0	1	0
Solyc04g080100.2.1	2	16	1	20	0	14	12	28	1	37	0	40	0
Solyc04g080110.2.1	0	0	0	0	0	0	0	0	0	0	0	0	0
Solyc04g080120.1.1	2	5	1	0	0	0	2	2	0	5	1	3	0
Solyc04g080130.2.1	4	18	0	19	0	4	3	24	0	25	9	33	0
Solyc04g080140.2.1	5	24	2	27	11	81	58	103	10	184	35	127	16
Solyc04g080160.2.1	3	12	4	5	0	67	45	51	7	9	12	33	0
Solyc04g080170.2.1	5	9	2	3	0	40	76	60	15	33	3	31	0
Solyc04g080180.1.1	9	61	1	17	0	159	51	126	6	164	33	101	0
Solyc04g080190.2.1	4	90	1	44	0	293	12	139	1	155	4	82	0
Solyc04g080200.2.1	0	0	0	0	0	0	0	0	0	0	0	0	0
Solyc04g080210.1.1	0	0	0	0	0	0	0	0	0	0	0	0	0
Solyc04g080220.2.1	0	0	0	0	0	0	0	0	0	0	0	0	0
Solyc04g080230.2.1	5	65	4	51	7	7	20	26	2	83	8	60	3
Solyc04g080240.2.1	26	147	19	108	4	289	383	669	43	341	80	291	0
Solyc04g080260.2.1	10	90	21	60	10	85	343	267	32	176	37	182	4
Solyc04g080270.2.1	0	0	0	0	0	0	0	0	0	0	0	0	0
Solyc04g080280.2.1	2	85	14	46	13	47	153	104	27	124	62	110	8
Solyc04g080290.2.1	9	208	20	60	25	96	91	83	49	162	200	116	11
Solyc04g080300.2.1	7	103	15	56	5	160	36	91	8	69	29	67	0
Solyc04g080310.1.1	1	1	1	0	0	20	3	9	0	1	2	0	0
Solyc04g080340.2.1	16	114	9	78	2	357	121	353	41	201	55	132	1
Solyc04g080350.2.1	3	7	0	4	0	11	12	19	0	7	4	9	1
Solyc04g080360.2.1	13	105	8	88	7	60	39	151	13	183	56	177	5
Solyc04g080370.2.1	7	85	2	64	7	177	82	188	15	100	25	74	1
Solyc04g080380.2.1	0	0	0	0	0	0	0	0	0	0	0	0	0
Solyc04g080390.2.1	4	35	1	25	0	18	19	27	0	36	7	27	2
Solyc04g080420.2.1	0	0	0	0	0	0	0	0	0	0	0	0	0
Solyc04g080430.2.1	8	126	13	84	2	158	60	151	8	29	23	38	4
Solyc04g080440.2.1	3	18	1	6	1	20	19	35	4	12	11	21	1
Solyc04g080450.1.1	6	152	9	69	1	49	35	111	0	198	21	250	0
Solyc04g080460.2.1	4	38	3	17	3	31	16	45	10	97	0	37	12

Solyc04g080470.2.1	6	42	11	25	3	20	28	26	1	24	8	16	2
Solyc04g080490.2.1	0	0	0	0	0	0	0	0	0	0	0	0	0
Solyc04g080520.1.1	0	0	0	0	0	0	0	0	0	0	0	0	0
Solyc04g080540.2.1	0	0	0	0	0	0	0	0	0	0	0	0	0
Solyc04g080550.2.1	9	98	1	66	0	45	17	60	0	15	99	3	0
Solyc04g080570.2.1	10	106	9	56	3	132	86	251	13	429	42	221	1
Solyc04g080580.2.1	3	15	3	9	0	6	4	19	1	27	1	51	1
Solyc04g080590.2.1	14	424	34	194	13	994	234	821	107	915	78	580	1
Solyc04g080600.2.1	14	73	6	40	0	171	78	129	17	21	14	9	0
Solyc04g080610.2.1	4	22	0	7	5	18	29	85	6	82	12	37	3
Solyc04g080620.2.1	0	0	0	0	0	0	0	0	0	0	0	0	0
Solyc04g080630.2.1	1	186	3	87	1	19	21	32	1	130	5	102	0
Solyc04g080640.1.1	1	2	0	3	0	2	5	17	1	1	0	6	0
Solyc04g080650.2.1	0	0	0	0	0	0	0	0	0	0	0	0	0
Solyc04g080660.2.1	0	0	0	0	0	0	0	0	0	0	0	0	0
Solyc04g080670.2.1	4	17	1	15	1	26	12	26	0	46	13	63	1
Solyc04g080680.2.1	1	4	0	3	0	19	2	10	2	24	1	8	0
Solyc04g080700.2.1	6	120	6	107	5	48	231	254	10	361	13	275	10
Solyc04g080710.2.1	2	3	2	3	0	19	6	29	0	0	0	0	0
Solyc04g080730.2.1	7	135	9	74	2	128	69	173	12	188	18	162	0
Solyc04g080740.1.1	2	27	4	35	5	53	42	59	3	74	33	30	0
Solyc04g080750.2.1	4	7	2	0	5	45	71	0	30	0	1	0	4
Solyc04g080770.2.1	8	126	9	114	3	354	190	276	6	343	15	130	9
Solyc04g080790.2.1	9	62	0	6	2	172	55	158	20	4	0	5	0
Solyc04g080800.2.1	2	1	1	9	0	4	7	4	0	3	0	3	0
Solyc04g080810.2.1	1	2	0	1	0	13	4	3	1	3	0	16	0
Solyc04g080820.2.1	0	0	0	0	0	0	0	0	0	0	0	0	0
Solyc04g080830.2.1	9	13	7	27	1	24	71	35	1	38	8	9	0
Solyc04g080840.1.1	0	0	0	0	0	0	0	0	0	0	0	0	0
Solyc04g080850.2.1	3	861	58	312	106	3002	1386	1574	566	471	294	373	51
Solyc04g080860.1.1	2	152	16	78	0	49	50	88	2	38	14	54	3
Solyc04g080870.1.1	0	0	0	0	0	0	0	0	0	0	0	0	0
Solyc04g080880.2.1	16	155	26	89	14	561	177	554	41	260	89	203	9
Solyc04g080890.2.1	0	0	0	0	0	0	0	0	0	0	0	0	0
Solyc04g080900.2.1	7	32	8	25	6	46	27	47	1	19	15	10	0
Solyc04g080920.1.1	0	0	0	0	0	0	0	0	0	0	0	0	0
Solyc04g080930.2.1	4	45	1	42	0	33	25	56	2	58	4	91	0
Solyc04g080940.2.1	5	81	10	57	11	30	24	43	1	60	19	39	0
Solyc04g080950.2.1	0	0	0	0	0	0	0	0	0	0	0	0	0
Solyc04g080960.2.1	15	5601	398	4806	262	5138	5423	8541	783	6195	789	2222	110
Solyc04g080970.2.1	0	0	0	0	0	0	0	0	0	0	0	0	0

Solyc04g080980.2.1	17	240	35	163	31	845	301	1062	57	571	164	420	16
Solyc04g081000.2.1	0	0	0	0	0	0	0	0	0	0	0	0	0
Solyc04g081020.2.1	0	0	0	0	0	0	0	0	0	0	0	0	0
Solyc04g081030.2.1	4	31	5	8	1	27	21	70	3	59	8	76	0
Solyc04g081050.1.1	11	90	6	53	0	99	71	121	15	181	53	107	0
Solyc04g081060.2.1	0	0	0	0	0	0	0	0	0	0	0	0	0
Solyc04g081070.2.1	19	122	13	94	0	269	172	412	20	279	48	209	3
Solyc04g081080.1.1	0	0	0	0	0	0	0	0	0	0	0	0	0
Solyc04g081090.2.1	3	200	31	177	30	446	399	624	73	507	77	460	7
Solyc04g081100.2.1	12	23	3	24	0	48	46	81	2	51	25	48	0
Solyc04g081140.1.1	0	0	0	0	0	0	0	0	0	0	0	0	0
Solyc04g081150.2.1	0	0	0	0	0	0	0	0	0	0	0	0	0
Solyc04g081160.2.1	0	0	0	0	0	0	0	0	0	0	0	0	0
Solyc04g081170.2.1	8	66	3	45	0	113	104	160	19	83	49	100	0
Solyc04g081190.2.1	9	24	2	24	3	37	18	32	13	107	57	69	7
Solyc04g081200.2.1	10	87	7	40	1	127	55	114	11	158	38	89	4
Solyc04g081210.2.1	20	94	20	94	30	141	110	269	24	290	51	256	12
Solyc04g081220.2.1	2	2	0	2	0	5	7	5	3	9	5	5	0
Solyc04g081230.2.1	17	150	16	83	17	485	400	426	49	313	33	327	0
Solyc04g081240.2.1	5	8	1	13	0	13	4	26	3	17	16	10	0
Solyc04g081250.1.1	0	0	0	0	0	0	0	0	0	0	0	0	0
Solyc04g081260.2.1	10	162	12	110	1	48	22	114	6	157	28	133	3
Solyc04g081270.1.1	0	0	0	0	0	0	0	0	0	0	0	0	0
Solyc04g081280.2.1	9	46	6	16	4	97	40	119	17	61	33	34	3
Solyc04g081290.2.1	3	41	0	23	5	86	19	50	1	5	2	3	5
Solyc04g081300.2.1	9	300	29	158	84	16	21	41	3	1773	99	1461	23
Solyc04g081310.2.1	2	11	0	10	0	14	3	5	1	7	6	8	0
Solyc04g081320.2.1	5	134	3	37	0	9	22	16	0	34	4	60	0
Solyc04g081330.2.1	7	365	45	251	7	111	113	191	4	220	52	202	7
Solyc04g081340.2.1	6	42	1	18	3	95	50	114	9	61	8	46	0
Solyc04g081350.2.1	0	0	0	0	0	0	0	0	0	0	0	0	0
Solyc04g081360.2.1	2	6	0	12	3	2	15	5	0	4	5	5	1
Solyc04g081370.2.1	5	57	3	21	1	22	20	5	13	33	7	17	1
Solyc04g081390.2.1	0	0	0	0	0	0	0	0	0	0	0	0	0
Solyc04g081400.2.1	8	28	2	33	2	28	12	22	7	91	31	91	0
Solyc04g081410.2.1	0	0	0	0	0	0	0	0	0	0	0	0	0
Solyc04g081420.2.1	5	24	3	14	0	30	27	38	1	21	19	23	0
Solyc04g081430.2.1	2	38	4	15	1	61	24	93	5	60	5	51	0
Solyc04g081440.2.1	12	444	21	432	15	396	341	769	51	1275	74	909	8
Solyc04g081450.2.1	0	0	0	0	0	0	0	0	0	0	0	0	0
Solyc04g081460.2.1	0	0	0	0	0	0	0	0	0	0	0	0	0

Solyc04g081470.2.1	4	10	2	6	0	8	15	25	1	39	3	15	1
Solyc04g081480.1.1	2	4	0	3	0	12	9	11	0	0	0	1	0
Solyc04g081490.2.1	6	1866	90	1066	71	478	789	779	82	4479	579	3387	53
Solyc04g081500.2.1	9	39	2	38	0	40	61	52	9	121	14	86	6
Solyc04g081510.2.1	8	55	12	47	3	455	76	99	21	51	27	47	3
Solyc04g081520.1.1	0	0	0	0	0	0	0	0	0	0	0	0	0
Solyc04g081530.1.1	4	118	12	25	1	248	63	66	19	12	5	30	0
Solyc04g081540.2.1	2	15	0	1	0	32	14	31	1	13	3	7	0
Solyc04g081550.2.1	8	51	10	29	1	112	21	64	4	66	8	42	0
Solyc04g081570.2.1	17	895	26	486	9	1478	179	1881	35	864	342	814	20
Solyc04g081580.2.1	2	5	1	16	0	58	12	61	2	4	4	19	0
Solyc04g081590.2.1	12	94	13	39	0	64	87	295	2	85	21	25	0
Solyc04g081630.1.1	0	0	0	0	0	0	0	0	0	0	0	0	0
Solyc04g081650.2.1	0	0	0	0	0	0	0	0	0	0	0	0	0
Solyc04g081660.1.1	0	0	0	0	0	0	0	0	0	0	0	0	0
Solyc04g081670.2.1	0	0	0	0	0	0	0	0	0	0	0	0	0
Solyc04g081680.2.1	0	0	0	0	0	0	0	0	0	0	0	0	0
Solyc04g081690.1.1	0	0	0	0	0	0	0	0	0	0	0	0	0
Solyc04g081700.2.1	2	5	1	8	0	3	4	4	0	0	2	0	0
Solyc04g081730.2.1	8	243	13	182	25	542	53	286	26	185	59	326	5
Solyc04g081740.2.1	4	18	0	15	0	52	16	32	5	54	8	72	0
Solyc04g081750.2.1	12	64	7	46	4	201	85	207	17	188	51	234	2
Solyc04g081760.2.1	0	0	0	0	0	0	0	0	0	0	0	0	0
Solyc04g081770.2.1	15	50	4	6	12	336	187	55	253	38	5	10	3
Solyc04g081790.2.1	1	11	0	0	2	0	7	0	2	11	1	0	10
Solyc04g081800.1.1	0	0	0	0	0	0	0	0	0	0	0	0	0
Solyc04g081810.2.1	0	0	0	0	0	0	0	0	0	0	0	0	0
Solyc04g081820.2.1	10	63	22	51	7	128	100	212	17	194	40	190	2
Solyc04g081840.2.1	0	0	0	0	0	0	0	0	0	0	0	0	0
Solyc04g081850.2.1	1	0	0	1	3	7	22	7	1	5	5	9	0
Solyc04g081870.2.1	4	35	20	35	17	7	64	12	18	8	12	1	2
Solyc04g081880.2.1	5	340	15	229	4	500	284	582	41	423	29	208	2
Solyc04g081890.1.1	1	5	1	0	0	2	15	8	3	6	7	4	0
Solyc04g081900.2.1	11	113	18	64	11	5868	2267	4458	242	230	126	142	0
Solyc04g081910.2.1	6	51	8	21	0	109	18	84	5	135	18	83	0
Solyc04g081920.2.1	3	8	0	5	0	9	16	9	1	18	4	11	0
Solyc04g081930.2.1	1	0	0	0	0	2	4	0	3	10	2	4	0
Solyc04g081940.2.1	1	8	2	1	0	8	7	2	5	6	1	12	0
Solyc04g081950.2.1	2	2	0	3	0	2	7	2	0	8	1	4	0
Solyc04g081960.1.1	3	6	1	4	0	44	30	38	15	2	1	7	1
Solyc04g081970.2.1	3	394	33	172	0	804	383	682	28	125	42	153	3

Solyc04g081980.2.1	0	0	0	0	0	0	0	0	0	0	0	0	0
Solyc04g081990.2.1	1	2	0	2	0	1	5	3	0	2	0	0	0
Solyc04g082000.2.1	0	0	0	0	0	0	0	0	0	0	0	0	0
Solyc04g082010.1.1	3	2648	573	866	0	31	1090	60	3	706	113	173	8
Solyc04g082020.2.1	6	80	7	66	0	137	64	171	3	54	12	51	2
Solyc04g082030.1.1	5	69	41	41	0	79	126	119	28	7	65	11	0
Solyc04g082040.2.1	7	18	2	9	0	52	77	62	10	22	6	17	1
Solyc04g082050.2.1	9	43	7	28	2	290	83	960	5	996	67	561	11
Solyc04g082060.2.1	2	4	0	10	0	2	8	7	1	27	1	46	0
Solyc04g082070.2.1	4	26	4	21	0	29	29	113	6	23	11	43	0
Solyc04g082090.2.1	7	21	11	15	3	152	87	93	20	140	45	119	7
Solyc04g082110.2.1	0	0	0	0	0	0	0	0	0	0	0	0	0
Solyc04g082120.2.1	16	55	9	32	5	257	18	339	15	19	104	2	2
Solyc04g082130.2.1	2	11	1	3	0	14	15	24	0	34	9	14	0
Solyc04g082140.2.1	5	348	2	135	22	18	42	72	49	57	27	62	0
Solyc04g082150.1.1	0	0	0	0	0	0	0	0	0	0	0	0	0
Solyc04g082160.2.1	8	88	5	47	2	136	75	138	20	158	39	133	1
Solyc04g082180.2.1	0	0	0	0	0	0	0	0	0	0	0	0	0
Solyc04g082200.2.1	7	2944	125	1428	228	23400	8769	18849	4676	2732	901	1918	143
Solyc04g082210.2.1	3	10	1	14	0	37	15	51	1	153	1	56	0
Solyc04g082220.1.1	0	0	0	0	0	0	0	0	0	0	0	0	0
Solyc04g082250.2.1	24	1992	445	1512	49	729	2125	1000	78	708	592	834	19
Solyc04g082260.2.1	10	134	12	94	5	325	198	503	46	209	41	204	1
Solyc04g082270.2.1	0	0	0	0	0	0	0	0	0	0	0	0	0
Solyc04g082280.2.1	1	7	0	10	0	15	6	25	0	17	0	37	0
Solyc04g082290.2.1	1	2	1	0	0	0	3	1	0	6	0	10	0
Solyc04g082300.2.1	6	12	1	9	1	24	19	37	2	78	7	66	0
Solyc04g082310.2.1	4	28	1	11	0	21	17	31	0	15	5	11	0
Solyc04g082320.2.1	0	0	0	0	0	0	0	0	0	0	0	0	0
Solyc04g082330.2.1	6	17	0	21	0	61	21	63	3	84	16	80	3
Solyc04g082340.2.1	3	14	0	11	0	28	18	31	2	38	5	11	0
Solyc04g082350.1.1	0	0	0	0	0	0	0	0	0	0	0	0	0
Solyc04g082360.1.1	6	64	4	41	5	236	13	155	2	199	2	122	0
Solyc04g082380.2.1	1	0	4	2	0	3	5	9	3	3	0	0	0
Solyc04g082390.2.1	1	5	0	0	0	0	6	11	2	3	1	7	0
Solyc04g082400.2.1	20	136	12	63	2	951	208	827	7	280	50	216	0
Solyc04g082410.2.1	8	49	1	62	0	34	29	76	0	134	11	86	7
Solyc04g082420.2.1	3	28	2	23	0	16	62	47	2	61	16	46	1
Solyc04g082430.2.1	0	0	0	0	0	0	0	0	0	0	0	0	0
Solyc04g082440.2.1	0	0	0	0	0	0	0	0	0	0	0	0	0
Solyc04g082450.2.1	1	7	0	0	0	7	9	8	0	19	0	6	0

Solyc04g082460.2.1	8	9	3	5	9	64	176	65	29	58	78	45	13
Solyc04g082470.1.1	1	4	0	7	0	10	2	6	0	7	2	19	0
Solyc04g082480.2.1	4	119	15	68	8	30	10	54	2	33	12	51	0
Solyc04g082490.2.1	8	593	14	218	4	976	109	455	2	658	67	358	6
Solyc04g082500.2.1	0	0	0	0	0	0	0	0	0	0	0	0	0
Solyc04g082510.2.1	4	33	1	10	1	20	15	28	12	14	23	7	3
Solyc04g082520.1.1	0	0	0	0	0	0	0	0	0	0	0	0	0
Solyc04g082530.2.1	0	0	0	0	0	0	0	0	0	0	0	0	0
Solyc04g082540.1.1	0	0	0	0	0	0	0	0	0	0	0	0	0
Solyc04g082560.2.1	25	166	31	161	18	303	244	441	60	397	56	303	2
Solyc04g082570.2.1	10	101	5	66	15	193	77	186	19	64	7	31	1
Solyc04g082580.2.1	11	115	9	38	14	114	95	176	24	108	19	70	9
Solyc04g082590.2.1	0	0	0	0	0	0	0	0	0	0	0	0	0
Solyc04g082600.2.1	7	24	2	8	3	49	43	51	1	138	4	46	0
Solyc04g082610.2.1	9	26	3	18	9	21	56	50	2	69	18	91	1
Solyc04g082620.2.1	5	79	1	108	6	526	84	266	14	207	12	166	0
Solyc04g082630.2.1	17	2886	315	1734	38	157	118	379	22	988	570	1290	26
Solyc04g082640.2.1	3	44	3	13	2	140	29	46	1	324	12	192	0
Solyc04g082650.2.1	0	0	0	0	0	0	0	0	0	0	0	0	0
Solyc04g082660.2.1	5	25	4	14	0	1	21	9	1	0	0	7	0
Solyc04g082670.2.1	10	92	8	57	11	192	61	331	23	298	48	249	4
Solyc04g082680.2.1	2	25	2	8	0	31	11	24	0	16	2	19	1
Solyc04g082690.1.1	1	1	0	5	0	2	9	7	0	19	5	16	0
Solyc04g082700.2.1	14	190	13	197	4	623	100	606	18	229	104	192	10
Solyc04g082710.2.1	12	227	2	83	0	131	72	270	0	6	2	9	0
Solyc04g082720.2.1	2	7	4	2	0	12	6	14	0	46	17	9	1
Solyc04g082740.2.1	1	18	0	0	0	16	11	13	0	4	0	0	0
Solyc04g082750.2.1	0	0	0	0	0	0	0	0	0	0	0	0	0
Solyc04g082760.2.1	9	49	12	37	8	83	79	140	19	57	11	48	2
Solyc04g082770.2.1	6	61	3	37	1	83	129	130	25	118	41	154	6
Solyc04g082780.2.1	10	157	17	73	3	90	271	170	26	348	164	317	10
Solyc04g082790.2.1	5	41	4	19	1	39	35	58	12	51	13	34	2
Solyc04g082800.2.1	0	0	0	0	0	0	0	0	0	0	0	0	0
Solyc04g082810.2.1	0	0	0	0	0	0	0	0	0	0	0	0	0
Solyc04g082820.2.1	4	15	0	29	0	28	13	39	2	13	6	6	0
Solyc04g082830.2.1	7	17	1	1	5	245	68	163	20	84	11	73	0
Solyc04g082840.2.1	0	0	0	0	0	0	0	0	0	0	0	0	0
Solyc04g082860.1.1	0	0	0	0	0	0	0	0	0	0	0	0	0
Solyc04g082870.1.1	0	0	0	0	0	0	0	0	0	0	0	0	0
Solyc04g082880.2.1	9	22	1	11	7	42	34	77	5	576	96	434	5
Solyc04g082890.2.1	7	100	14	61	13	160	179	197	73	254	63	116	5

Solyc04g082900.2.1	0	0	0	0	0	0	0	0	0	0	0	0	0
Solyc04g082910.1.1	3	19	8	23	0	24	21	18	0	10	5	10	1
Solyc04g082920.2.1	1	9	0	3	0	2	20	11	0	19	5	30	0
Solyc04g082930.1.1	1	47	4	36	5	10	17	18	0	125	5	117	0
Solyc04g082940.2.1	0	0	0	0	0	0	0	0	0	0	0	0	0
Solyc04g082950.2.1	0	0	0	0	0	0	0	0	0	0	0	0	0
Solyc04g082960.1.1	7	63	29	73	3	7	13	13	6	58	11	119	8
Solyc04g082970.2.1	1	25	0	3	1	0	0	2	2	8	0	2	0
Solyc04g082980.1.1	5	16	1	1	0	33	22	36	3	17	5	20	0
Solyc04g082990.2.1	0	0	0	0	0	0	0	0	0	0	0	0	0
Solyc04g083010.2.1	7	114	5	94	5	186	74	167	10	100	29	144	4
Solyc04g083140.1.1	0	0	0	0	0	0	0	0	0	0	0	0	0
Solyc04g083150.1.1	10	21	5	8	3	56	63	35	3	38	6	45	0
Solyc04g083160.1.1	1	2	0	2	0	4	5	3	0	0	0	0	0
Solyc05g005000.2.1	0	0	0	0	0	0	0	0	0	0	0	0	0
Solyc05g005010.2.1	0	0	0	0	0	0	0	0	0	0	0	0	0
Solyc05g005020.2.1	25	375	38	259	12	326	320	562	9	531	98	456	2
Solyc05g005030.2.1	0	0	0	0	0	0	0	0	0	0	0	0	0
Solyc05g005050.2.1	0	0	0	0	0	0	0	0	0	0	0	0	0
Solyc05g005060.2.1	0	0	0	0	0	0	0	0	0	0	0	0	0
Solyc05g005070.2.1	3	75	8	47	2	29	13	43	7	12	0	33	0
Solyc05g005080.2.1	14	798	79	613	84	65	165	200	49	3254	511	3622	31
Solyc05g005100.2.1	2	13	7	13	1	10	9	30	0	38	2	11	0
Solyc05g005110.1.1	2	2	0	4	1	10	11	13	1	14	0	18	0
Solyc05g005130.1.1	5	4	0	4	0	67	15	42	1	28	1	17	0
Solyc05g005140.2.1	0	0	0	0	0	0	0	0	0	0	0	0	0
Solyc05g005150.1.1	9	81	98	149	3	120	34	66	29	20	3	23	0
Solyc05g005160.2.1	5	266	65	110	66	673	491	406	157	433	377	529	16
Solyc05g005170.2.1	0	0	0	0	0	0	0	0	0	0	0	0	0
Solyc05g005180.2.1	4	19	8	8	0	8	21	14	0	15	4	18	2
Solyc05g005190.2.1	2	74	4	27	3	63	29	41	4	123	42	120	3
Solyc05g005200.2.1	3	42	1	15	0	85	63	30	5	129	10	107	0
Solyc05g005210.2.1	2	15	1	21	0	7	4	15	2	4	4	10	0
Solyc05g005220.1.1	0	0	0	0	0	0	0	0	0	0	0	0	0
Solyc05g005230.2.1	0	0	0	0	0	0	0	0	0	0	0	0	0
Solyc05g005240.1.1	0	0	0	0	0	0	0	0	0	0	0	0	0
Solyc05g005250.2.1	11	44	3	29	4	234	126	191	13	164	71	75	1
Solyc05g005260.1.1	9	538	217	504	39	374	120	98	43	164	10	314	4
Solyc05g005280.2.1	2	23	0	9	0	70	5	25	4	9	3	7	0
Solyc05g005290.2.1	1	9	0	2	0	51	0	39	0	50	0	51	0
Solyc05g005300.2.1	0	0	0	0	0	0	0	0	0	0	0	0	0

Solyc05g005310.1.1	3	3	0	13	0	21	4	28	3	12	9	14	0
Solyc05g005320.1.1	1	4	0	0	0	13	6	14	0	2	1	11	0
Solyc05g005330.2.1	4	16	1	25	8	16	23	34	1	14	0	20	0
Solyc05g005340.2.1	1	0	0	0	2	4	15	3	15	2	7	1	5
Solyc05g005440.1.1	0	0	0	0	0	0	0	0	0	0	0	0	0
Solyc05g005460.2.1	11	166	13	85	12	202	439	281	410	101	33	119	21
Solyc05g005480.2.1	10	771	150	520	36	395	124	739	32	117	212	172	12
Solyc05g005490.2.1	8	1000	42	186	13	959	557	1982	42	375	85	86	2
Solyc05g005500.1.1	0	0	0	0	0	0	0	0	0	0	0	0	0
Solyc05g005510.2.1	1	84	3	61	6	167	16	289	19	107	13	82	3
Solyc05g005540.2.1	0	0	0	0	0	0	0	0	0	0	0	0	0
Solyc05g005550.2.1	0	0	0	0	0	0	0	0	0	0	0	0	0
Solyc05g005560.2.1	11	249	23	105	13	35	22	66	3	4524	162	7569	14
Solyc05g005570.2.1	2	7	7	0	0	1	0	0	0	30	0	34	0
Solyc05g005580.2.1	0	0	0	0	0	0	0	0	0	0	0	0	0
Solyc05g005590.2.1	2	5	7	1	0	4	76	10	5	4	0	5	0
Solyc05g005600.1.1	0	0	0	0	0	0	0	0	0	0	0	0	0
Solyc05g005610.2.1	0	0	0	0	0	0	0	0	0	0	0	0	0
Solyc05g005620.2.1	16	141	20	113	9	252	158	393	25	297	67	194	3
Solyc05g005630.2.1	2	2	0	1	0	18	12	7	0	0	0	2	0
Solyc05g005640.2.1	13	34	3	16	0	55	80	106	12	73	18	67	2
Solyc05g005650.1.1	0	0	0	0	0	0	0	0	0	0	0	0	0
Solyc05g005670.1.1	0	0	0	0	0	0	0	0	0	0	0	0	0
Solyc05g005680.2.1	0	0	0	0	0	0	0	0	0	0	0	0	0
Solyc05g005690.1.1	3	123	2	42	0	66	21	124	9	93	22	98	0
Solyc05g005700.2.1	5	15	0	20	2	80	30	84	7	318	13	260	0
Solyc05g005710.2.1	10	254	19	132	4	410	157	621	21	696	130	666	1
Solyc05g005720.2.1	2	2	0	1	0	25	1	55	1	169	10	285	0
Solyc05g005730.2.1	11	38	2	5	0	82	36	104	7	82	16	68	0
Solyc05g005740.2.1	17	150	14	112	6	176	254	216	7	51	9	16	0
Solyc05g005750.2.1	9	46	11	48	4	191	322	183	32	104	10	51	5
Solyc05g005760.2.1	6	79	6	23	0	1	6	7	0	148	41	135	0
Solyc05g005770.2.1	5	17	0	11	3	16	25	24	3	25	10	29	0
Solyc05g005780.2.1	7	40	2	24	4	80	72	100	9	171	14	142	1
Solyc05g005790.2.1	0	0	0	0	0	0	0	0	0	0	0	0	0
Solyc05g005800.2.1	12	87	20	58	12	129	167	263	23	316	91	327	8
Solyc05g005810.2.1	2	11	1	2	0	4	0	1	0	1	4	3	0
Solyc05g005830.2.1	0	0	0	0	0	0	0	0	0	0	0	0	0
Solyc05g005860.1.1	7	34	4	26	3	23	33	64	2	34	12	28	0
Solyc05g005870.2.1	0	0	0	0	0	0	0	0	0	0	0	0	0
Solyc05g005880.2.1	3	641	76	210	3	105	504	155	2	184	32	226	1

Solyc05g005890.2.1	0	0	0	0	0	0	0	0	0	0	0	0	0
Solyc05g005900.2.1	2	25	6	9	0	10	8	15	0	15	1	12	0
Solyc05g005910.2.1	0	0	0	0	0	0	0	0	0	0	0	0	0
Solyc05g005920.2.1	0	0	0	0	0	0	0	0	0	0	0	0	0
Solyc05g005930.1.1	0	0	0	0	0	0	0	0	0	0	0	0	0
Solyc05g005940.2.1	3	5	1	1	0	0	2	2	0	102	10	115	0
Solyc05g005950.2.1	13	225	17	126	3	80	95	76	2	307	69	148	0
Solyc05g005960.2.1	7	279	10	242	0	83	38	288	0	570	72	259	3
Solyc05g005970.2.1	0	0	0	0	0	0	0	0	0	0	0	0	0
Solyc05g005980.2.1	3	88	25	26	0	12	5	7	5	55	17	27	0
Solyc05g005990.2.1	4	56	4	5	1	22	28	29	0	30	19	61	0
Solyc05g006000.2.1	0	0	0	0	0	0	0	0	0	0	0	0	0
Solyc05g006010.2.1	0	0	0	0	0	0	0	0	0	0	0	0	0
Solyc05g006020.2.1	0	0	0	0	0	0	0	0	0	0	0	0	0
Solyc05g006030.2.1	6	77	3	22	0	13	27	50	0	93	28	92	1
Solyc05g006040.2.1	0	0	0	0	0	0	0	0	0	0	0	0	0
Solyc05g006050.2.1	4	45	2	57	2	11	32	31	4	20	5	24	0
Solyc05g006070.2.1	5	31	0	21	0	29	32	66	0	25	2	10	0
Solyc05g006080.1.1	2	49	3	1	0	23	2	40	2	51	5	44	2
Solyc05g006090.2.1	3	25	1	22	8	80	76	69	2	124	28	31	0
Solyc05g006100.2.1	0	0	0	0	0	0	0	0	0	0	0	0	0
Solyc05g006110.2.1	7	35	8	25	0	28	98	36	0	15	7	10	2
Solyc05g006120.2.1	3	9	1	0	0	18	17	17	1	0	0	0	0
Solyc05g006130.2.1	11	14	0	10	0	42	79	81	9	38	6	36	0
Solyc05g006160.2.1	2	4	0	12	0	2	3	0	0	2	20	5	0
Solyc05g006170.2.1	6	21	1	21	0	53	39	54	0	78	8	50	1
Solyc05g006180.2.1	0	0	0	0	0	0	0	0	0	0	0	0	0
Solyc05g006200.2.1	1	1	0	1	0	1	2	3	0	4	3	1	0
Solyc05g006210.2.1	0	0	0	0	0	0	0	0	0	0	0	0	0
Solyc05g006220.2.1	7	32	3	10	0	66	29	26	1	21	19	20	0
Solyc05g006240.2.1	8	109	11	67	1	84	50	136	6	133	34	85	5
Solyc05g006310.1.1	0	0	0	0	0	0	0	0	0	0	0	0	0
Solyc05g006320.2.1	10	44	12	22	14	103	78	119	13	108	26	69	0
Solyc05g006330.1.1	0	0	0	0	0	0	0	0	0	0	0	0	0
Solyc05g006340.2.1	10	82	8	33	12	60	80	165	42	149	55	86	13
Solyc05g006390.1.1	0	0	0	0	0	0	0	0	0	0	0	0	0
Solyc05g006400.2.1	6	140	13	49	1	167	68	253	20	498	43	288	10
Solyc05g006410.2.1	1	3	0	10	0	10	4	2	0	4	0	10	0
Solyc05g006420.2.1	0	0	0	0	0	0	0	0	0	0	0	0	0
Solyc05g006430.1.1	0	0	0	0	0	0	0	0	0	0	0	0	0
Solyc05g006440.2.1	9	255	27	122	2	48	171	82	0	158	28	152	1

Solyc05g006450.1.1	0	0	0	0	0	0	0	0	0	0	0	0	0
Solyc05g006460.1.1	0	0	0	0	0	0	0	0	0	0	0	0	0
Solyc05g006470.2.1	0	0	0	0	0	0	0	0	0	0	0	0	0
Solyc05g006490.2.1	27	203	23	134	14	346	480	976	49	245	19	105	0
Solyc05g006500.2.1	0	0	0	0	0	0	0	0	0	0	0	0	0
Solyc05g006510.1.1	0	0	0	0	0	0	0	0	0	0	0	0	0
Solyc05g006520.2.1	6	84	14	37	8	171	114	172	18	126	47	87	0
Solyc05g006530.2.1	9	21	14	34	1	119	122	174	15	25	10	16	3
Solyc05g006540.2.1	25	125	10	73	6	154	259	205	38	153	82	116	9
Solyc05g006550.2.1	0	0	0	0	0	0	0	0	0	0	0	0	0
Solyc05g006560.1.1	5	23	1	26	1	20	17	17	2	29	3	15	0
Solyc05g006570.1.1	1	5	2	0	2	3	7	1	2	6	15	1	1
Solyc05g006580.2.1	3	35	1	7	0	30	9	48	2	103	0	64	0
Solyc05g006590.2.1	2	12	0	1	2	20	11	25	1	4	1	12	0
Solyc05g006620.2.1	10	35	7	45	9	44	65	83	7	90	7	52	0
Solyc05g006630.2.1	10	61	6	54	3	102	76	149	26	67	6	56	6
Solyc05g006640.2.1	7	30	0	13	1	134	15	87	2	14	8	26	0
Solyc05g006650.2.1	2	72	0	15	1	5	3	25	0	65	3	16	0
Solyc05g006660.2.1	7	156	4	189	3	314	49	207	6	85	28	62	0
Solyc05g006670.1.1	0	0	0	0	0	0	0	0	0	0	0	0	0
Solyc05g006680.2.1	4	38	4	18	0	33	43	35	5	10	3	18	2
Solyc05g006690.2.1	0	0	0	0	0	0	0	0	0	0	0	0	0
Solyc05g006700.2.1	0	0	0	0	0	0	0	0	0	0	0	0	0
Solyc05g006710.2.1	0	0	0	0	0	0	0	0	0	0	0	0	0
Solyc05g006720.1.1	0	0	0	0	0	0	0	0	0	0	0	0	0
Solyc05g006730.2.1	2	12	8	0	3	6	12	0	8	0	0	0	0
Solyc05g006740.2.1	0	0	0	0	0	0	0	0	0	0	0	0	0
Solyc05g006760.2.1	10	32	3	14	1	95	130	110	11	44	4	35	2
Solyc05g006770.2.1	0	0	0	0	0	0	0	0	0	0	0	0	0
Solyc05g006780.2.1	3	45	4	16	0	36	17	31	7	21	2	21	1
Solyc05g006790.1.1	0	0	0	0	0	0	0	0	0	0	0	0	0
Solyc05g006800.1.1	1	0	0	1	0	6	4	7	1	7	0	3	0
Solyc05g006810.2.1	0	0	0	0	0	0	0	0	0	0	0	0	0
Solyc05g006820.2.1	2	9	2	21	5	11	6	20	1	34	3	57	0
Solyc05g006830.2.1	0	0	0	0	0	0	0	0	0	0	0	0	0
Solyc05g006850.2.1	3	110	2	82	0	193	68	393	0	28	4	23	0
Solyc05g006860.2.1	1	3	0	0	0	14	3	7	0	0	2	0	0
Solyc05g006870.2.1	1	20	0	41	0	110	21	276	0	60	4	78	0
Solyc05g006880.1.1	3	21	1	14	1	26	5	34	6	32	5	15	0
Solyc05g006890.1.1	0	0	0	0	0	0	0	0	0	0	0	0	0
Solyc05g006900.1.1	4	23	5	30	0	22	22	33	3	78	15	43	0

Solyc05g006910.2.1	2	2	1	1	0	46	20	51	1	13	0	8	0
Solyc05g006920.2.1	8	156	16	107	0	259	25	144	0	225	21	364	3
Solyc05g006930.2.1	0	0	0	0	0	0	0	0	0	0	0	0	0
Solyc05g006940.2.1	3	23	5	18	3	124	59	79	4	55	4	50	0
Solyc05g006950.2.1	11	81	13	41	8	103	162	159	24	163	41	156	2
Solyc05g006960.2.1	1	5	2	8	0	28	10	15	2	1	1	6	0
Solyc05g006970.2.1	10	20	2	16	4	40	191	55	0	27	5	14	0
Solyc05g006980.2.1	12	26	13	22	0	328	808	282	22	59	46	37	1
Solyc05g006990.2.1	2	14	0	18	0	3	15	72	0	1	0	3	0
Solyc05g007000.2.1	0	0	0	0	0	0	0	0	0	0	0	0	0
Solyc05g007010.1.1	0	0	0	0	0	0	0	0	0	0	0	0	0
Solyc05g007020.2.1	17	357	56	288	7	746	260	855	34	301	64	227	7
Solyc05g007030.2.1	14	70	6	56	2	214	98	269	19	146	34	156	2
Solyc05g007040.1.1	0	0	0	0	0	0	0	0	0	0	0	0	0
Solyc05g007050.2.1	9	71	6	41	5	148	78	182	23	50	31	37	1
Solyc05g007060.2.1	8	127	1	70	1	760	138	1373	21	408	15	270	2
Solyc05g007070.2.1	23	153	10	141	14	1292	787	1356	39	1029	108	640	1
Solyc05g007080.2.1	0	0	0	0	0	0	0	0	0	0	0	0	0
Solyc05g007090.2.1	1	9	0	3	0	2	9	4	0	0	4	1	0
Solyc05g007100.2.1	3	10	1	9	1	3	21	23	1	8	3	10	0
Solyc05g007120.2.1	21	405	5	233	13	131	126	295	8	814	365	823	13
Solyc05g007130.2.1	1	7	0	0	0	7	5	6	0	16	1	26	0
Solyc05g007140.2.1	8	158	16	142	33	79	55	93	25	32	14	62	8
Solyc05g007150.2.1	3	9	0	6	0	35	9	53	7	0	7	1	0
Solyc05g007160.2.1	5	9	1	23	2	15	17	21	0	11	6	41	0
Solyc05g007170.2.1	0	0	0	0	0	0	0	0	0	0	0	0	0
Solyc05g007180.2.1	1	4	0	9	0	0	0	1	0	35	3	3	0
Solyc05g007190.2.1	2	22	1	27	0	138	27	31	5	262	7	208	1
Solyc05g007200.2.1	3	30	0	22	0	27	17	27	4	55	8	74	0
Solyc05g007220.2.1	6	40	2	13	1	75	19	80	5	50	36	58	1
Solyc05g007230.2.1	0	0	0	0	0	0	0	0	0	0	0	0	0
Solyc05g007240.1.1	4	320	31	224	15	413	83	458	20	780	75	457	8
Solyc05g007250.2.1	10	387	17	126	16	605	178	637	48	987	169	834	11
Solyc05g007260.2.1	11	140	10	61	2	194	74	318	17	299	40	275	5
Solyc05g007270.2.1	7	38	3	11	3	41	53	23	10	70	25	38	4
Solyc05g007280.2.1	0	0	0	0	0	0	0	0	0	0	0	0	0
Solyc05g007290.1.1	0	0	0	0	0	0	0	0	0	0	0	0	0
Solyc05g007300.2.1	0	0	0	0	0	0	0	0	0	0	0	0	0
Solyc05g007350.1.1	0	0	0	0	0	0	0	0	0	0	0	0	0
Solyc05g007420.1.1	1	2	0	13	0	2	2	2	0	0	0	0	0
Solyc05g007430.2.1	1	0	0	0	0	4	12	12	0	10	0	5	0

Solyc05g007440.2.1	4	230	2	245	1	2208	29	1308	20	417	14	370	1
Solyc05g007450.2.1	0	0	0	0	0	0	0	0	0	0	0	0	0
Solyc05g007460.2.1	4	53	5	51	6	122	71	115	26	34	9	24	2
Solyc05g007470.2.1	13	94	19	103	7	32	109	148	9	24	17	7	0
Solyc05g007480.1.1	7	29	5	16	3	36	46	97	5	89	0	30	0
Solyc05g007490.2.1	5	50	5	40	0	77	45	157	0	68	53	88	1
Solyc05g007500.2.1	5	33	3	14	0	14	14	29	1	15	9	21	0
Solyc05g007510.2.1	14	40	7	76	4	41	90	73	3	219	30	206	2
Solyc05g007520.1.1	0	0	0	0	0	0	0	0	0	0	0	0	0
Solyc05g007560.2.1	5	301	13	151	14	433	136	567	35	277	107	232	8
Solyc05g007570.2.1	6	22	8	10	1	68	25	79	2	10	0	14	1
Solyc05g007580.1.1	0	0	0	0	0	0	0	0	0	0	0	0	0
Solyc05g007590.2.1	1	6	0	5	0	0	1	7	1	19	4	2	0
Solyc05g007600.1.1	0	0	0	0	0	0	0	0	0	0	0	0	0
Solyc05g007610.2.1	0	0	0	0	0	0	0	0	0	0	0	0	0
Solyc05g007630.2.1	0	0	0	0	0	0	0	0	0	0	0	0	0
Solyc05g007640.2.1	0	0	0	0	0	0	0	0	0	0	0	0	0
Solyc05g007660.2.1	4	86	6	64	3	182	87	190	12	132	54	104	0
Solyc05g007670.2.1	8	121	1	54	4	221	109	232	15	155	19	111	0
Solyc05g007680.1.1	7	43	6	26	0	25	38	23	1	23	14	12	0
Solyc05g007690.1.1	0	0	0	0	0	0	0	0	0	0	0	0	0
Solyc05g007720.1.1	0	0	0	0	0	0	0	0	0	0	0	0	0
Solyc05g007730.2.1	0	0	0	0	0	0	0	0	0	0	0	0	0
Solyc05g007740.1.1	3	13	3	2	0	68	31	82	1	23	8	20	0
Solyc05g007750.2.1	1	0	1	0	0	3	5	3	0	3	1	7	0
Solyc05g007760.1.1	0	0	0	0	0	0	0	0	0	0	0	0	0
Solyc05g007770.2.1	6	25	1	27	0	295	69	140	6	8	58	3	0
Solyc05g007780.2.1	3	95	28	37	0	9	88	21	0	0	17	5	0
Solyc05g007790.2.1	1	27	0	9	0	2	8	10	0	1	13	2	0
Solyc05g007800.2.1	0	0	0	0	0	0	0	0	0	0	0	0	0
Solyc05g007810.2.1	12	47	9	50	1	145	70	209	21	111	51	121	6
Solyc05g007820.2.1	4	14	6	21	2	12	43	45	13	33	6	24	0
Solyc05g007830.2.1	2	11	6	3	2	13	1	42	0	6	3	9	1
Solyc05g007840.2.1	3	11	0	4	7	15	26	21	1	12	9	9	0
Solyc05g007850.1.1	0	0	0	0	0	0	0	0	0	0	0	0	0
Solyc05g007880.2.1	5	46	3	37	0	29	18	14	6	2	4	39	0
Solyc05g007890.2.1	5	19	0	10	0	48	32	20	0	3	3	6	0
Solyc05g007900.2.1	3	17	6	7	1	13	69	14	11	75	18	85	0
Solyc05g007910.2.1	2	13	1	9	0	13	57	13	1	7	1	14	0
Solyc05g007930.2.1	3	13	1	3	0	18	12	21	2	4	4	3	0
Solyc05g007940.2.1	9	72	0	42	0	915	172	234	21	2	2	3	0

Solyc05g007950.2.1	9	3	0	6	0	303	64	729	34	2	3	1	0
Solyc05g007960.2.1	6	14	3	12	0	52	34	27	1	27	6	27	4
Solyc05g007970.2.1	2	46	6	25	0	106	89	123	5	46	16	31	0
Solyc05g007980.2.1	11	210	24	220	0	224	103	376	1	7	1	2	0
Solyc05g007990.2.1	1	2	0	0	0	11	2	4	0	0	5	0	0
Solyc05g008000.2.1	1	5	1	7	6	13	6	2	4	2	1	1	0
Solyc05g008010.2.1	1	11	0	5	0	9	1	21	1	85	4	37	0
Solyc05g008020.2.1	0	0	0	0	0	0	0	0	0	0	0	0	0
Solyc05g008030.1.1	0	0	0	0	0	0	0	0	0	0	0	0	0
Solyc05g008040.2.1	5	13	2	5	0	31	24	47	0	18	6	37	0
Solyc05g008060.2.1	13	210	12	144	6	163	60	128	9	88	25	116	2
Solyc05g008070.2.1	0	0	0	0	0	0	0	0	0	0	0	0	0
Solyc05g008080.1.1	0	0	0	0	0	0	0	0	0	0	0	0	0
Solyc05g008090.2.1	9	60	9	62	7	46	53	180	15	46	15	68	0
Solyc05g008100.1.1	7	175	23	100	7	108	300	283	72	160	24	175	1
Solyc05g008110.2.1	5	66	2	58	1	206	70	182	19	133	7	86	0
Solyc05g008120.2.1	4	16	0	7	0	0	9	0	1	2	14	6	0
Solyc05g008130.2.1	2	48	0	14	0	187	10	74	0	94	1	44	0
Solyc05g008140.2.1	2	21	3	18	0	7	5	17	1	24	8	40	0
Solyc05g008180.2.1	6	90	20	30	2	302	307	166	59	116	30	60	1
Solyc05g008190.2.1	0	0	0	0	0	0	0	0	0	0	0	0	0
Solyc05g008200.2.1	23	183	14	96	13	209	141	266	40	516	137	515	5
Solyc05g008210.2.1	0	0	0	0	0	0	0	0	0	0	0	0	0
Solyc05g008220.2.1	1	0	0	0	0	9	1	10	5	0	1	0	0
Solyc05g008230.2.1	10	119	7	84	2	78	36	139	3	262	23	200	2
Solyc05g008250.1.1	0	0	0	0	0	0	0	0	0	0	0	0	0
Solyc05g008260.2.1	20	86	18	112	16	142	297	220	33	435	58	256	13
Solyc05g008270.2.1	2	21	2	22	0	10	6	7	1	29	1	24	0
Solyc05g008290.2.1	13	304	20	255	0	148	24	456	0	167	24	291	1
Solyc05g008300.1.1	0	0	0	0	0	0	0	0	0	0	0	0	0
Solyc05g008310.2.1	2	4	0	0	0	7	0	7	0	6	2	1	1
Solyc05g008320.1.1	0	0	0	0	0	0	0	0	0	0	0	0	0
Solyc05g008330.1.1	0	0	0	0	0	0	0	0	0	0	0	0	0
Solyc05g008340.2.1	1	8	0	0	0	5	0	9	2	24	6	8	0
Solyc05g008350.2.1	16	168	35	153	0	94	172	197	1	136	20	183	6
Solyc05g008370.1.1	1	45	1	19	0	39	1	16	0	11	5	2	0
Solyc05g008380.2.1	8	30	6	23	2	385	255	471	60	141	23	166	4
Solyc05g008390.2.1	5	9	0	13	0	295	97	194	5	205	5	81	0
Solyc05g008400.2.1	4	65	10	11	0	28	49	95	3	23	4	11	0
Solyc05g008410.1.1	0	0	0	0	0	0	0	0	0	0	0	0	0
Solyc05g008420.2.1	3	5	2	3	0	24	16	11	0	14	3	19	0

Solyc05g008430.2.1	11	102	11	75	4	221	117	243	27	188	30	200	0
Solyc05g008440.1.1	7	54	8	26	2	64	54	83	12	179	14	94	5
Solyc05g008450.2.1	7	162	15	122	0	99	57	174	6	283	27	338	1
Solyc05g008460.2.1	9	584	83	326	20	1381	834	2045	214	1002	349	802	7
Solyc05g008480.2.1	1	6	0	2	0	1	6	5	0	3	1	2	0
Solyc05g008510.2.1	1	0	1	0	0	8	3	11	0	0	1	0	0
Solyc05g008520.2.1	7	26	5	8	3	59	65	56	11	72	31	53	4
Solyc05g008530.2.1	10	588	62	416	9	699	570	600	40	298	90	405	4
Solyc05g008540.2.1	0	0	0	0	0	0	0	0	0	0	0	0	0
Solyc05g008560.1.1	0	0	0	0	0	0	0	0	0	0	0	0	0
Solyc05g008580.2.1	1	17	0	0	0	4	0	10	0	1	8	6	0
Solyc05g008590.2.1	3	9	2	7	1	11	8	44	0	13	4	6	0
Solyc05g008600.2.1	13	446	7	273	19	852	96	1000	60	2424	354	2169	31
Solyc05g008610.2.1	7	108	7	73	4	190	52	327	11	83	40	199	10
Solyc05g008620.2.1	0	0	0	0	0	0	0	0	0	0	0	0	0
Solyc05g008630.2.1	17	129	11	78	6	339	217	533	28	202	70	186	7
Solyc05g008640.1.1	3	3	0	0	0	235	58	41	4	22	2	1	0
Solyc05g008670.2.1	0	0	0	0	0	0	0	0	0	0	0	0	0
Solyc05g008690.1.1	0	0	0	0	0	0	0	0	0	0	0	0	0
Solyc05g008750.2.1	4	6	0	7	0	19	21	26	4	10	19	19	0
Solyc05g008760.2.1	7	104	15	46	3	78	131	148	34	65	26	69	8
Solyc05g008770.2.1	0	0	0	0	0	0	0	0	0	0	0	0	0
Solyc05g008780.2.1	17	137	9	61	5	209	85	282	30	288	57	145	2
Solyc05g008790.2.1	6	22	3	24	0	48	34	42	3	57	8	36	2
Solyc05g008800.2.1	2	1	0	7	0	7	9	5	0	1	1	5	0
Solyc05g008810.2.1	3	88	10	77	4	20	10	86	8	148	2	167	2
Solyc05g008820.2.1	0	0	0	0	0	0	0	0	0	0	0	0	0
Solyc05g008830.2.1	16	289	33	214	56	398	387	577	104	799	254	580	58
Solyc05g008860.2.1	2	8	0	6	0	9	11	15	0	4	7	2	1
Solyc05g008870.2.1	1	3	0	0	0	6	8	2	0	1	0	9	0
Solyc05g008890.2.1	2	5	0	3	0	0	5	2	0	1	0	1	0
Solyc05g008900.2.1	2	23	0	12	0	53	3	62	0	66	5	30	0
Solyc05g008920.2.1	5	69	7	76	0	256	34	227	18	79	40	115	2
Solyc05g008930.2.1	5	20	5	3	2	6	17	13	5	15	5	16	4
Solyc05g008940.2.1	2	10	3	15	5	2	0	4	0	4	6	1	0
Solyc05g008950.2.1	0	0	0	0	0	0	0	0	0	0	0	0	0
Solyc05g008960.2.1	0	0	0	0	0	0	0	0	0	0	0	0	0
Solyc05g008970.1.1	0	0	0	0	0	0	0	0	0	0	0	0	0
Solyc05g008980.2.1	0	0	0	0	0	0	0	0	0	0	0	0	0
Solyc05g008990.2.1	0	0	0	0	0	0	0	0	0	0	0	0	0
Solyc05g009000.2.1	3	13	1	14	0	45	17	18	8	6	0	8	0

Solyc05g009010.1.1	2	19	5	17	1	24	11	9	3	5	0	8	0
Solyc05g009020.2.1	4	21	1	19	2	37	15	39	2	24	12	16	0
Solyc05g009030.2.1	7	143	6	37	29	177	139	325	10	126	41	87	1
Solyc05g009040.2.1	0	0	0	0	0	0	0	0	0	0	0	0	0
Solyc05g009050.2.1	0	0	0	0	0	0	0	0	0	0	0	0	0
Solyc05g009060.2.1	0	0	0	0	0	0	0	0	0	0	0	0	0
Solyc05g009070.2.1	6	88	5	17	4	50	14	27	13	115	39	93	9
Solyc05g009080.2.1	5	74	12	70	0	17	20	38	1	23	16	19	0
Solyc05g009090.1.1	0	0	0	0	0	0	0	0	0	0	0	0	0
Solyc05g009100.2.1	0	0	0	0	0	0	0	0	0	0	0	0	0
Solyc05g009120.2.1	0	0	0	0	0	0	0	0	0	0	0	0	0
Solyc05g009130.2.1	0	0	0	0	0	0	0	0	0	0	0	0	0
Solyc05g009140.2.1	4	11	1	7	0	72	25	40	2	22	2	19	1
Solyc05g009150.2.1	13	55	19	60	4	67	90	132	15	109	17	83	0
Solyc05g009160.2.1	7	46	2	43	6	53	19	91	16	151	11	126	0
Solyc05g009170.1.1	0	0	0	0	0	0	0	0	0	0	0	0	0
Solyc05g009180.1.1	0	0	0	0	0	0	0	0	0	0	0	0	0
Solyc05g009190.2.1	5	54	1	25	1	205	22	126	2	128	18	43	1
Solyc05g009210.1.1	0	0	0	0	0	0	0	0	0	0	0	0	0
Solyc05g009220.2.1	13	103	21	78	26	229	234	262	37	395	124	364	19
Solyc05g009230.1.1	0	0	0	0	0	0	0	0	0	0	0	0	0
Solyc05g009240.2.1	0	0	0	0	0	0	0	0	0	0	0	0	0
Solyc05g009250.1.1	0	0	0	0	0	0	0	0	0	0	0	0	0
Solyc05g009270.2.1	7	46	10	5	33	1	19	1	8	36	0	0	0
Solyc05g009280.2.1	0	0	0	0	0	0	0	0	0	0	0	0	0
Solyc05g009290.2.1	7	91	8	43	10	201	106	175	14	298	34	225	2
Solyc05g009300.1.1	5	87	7	68	0	16	41	61	0	157	9	175	1
Solyc05g009310.2.1	8	59	0	11	0	2	142	68	1	18	2	34	1
Solyc05g009320.2.1	0	0	0	0	0	0	0	0	0	0	0	0	0
Solyc05g009330.2.1	0	0	0	0	0	0	0	0	0	0	0	0	0
Solyc05g009340.1.1	0	0	0	0	0	0	0	0	0	0	0	0	0
Solyc05g009350.2.1	2	6	4	1	0	10	3	10	0	25	4	13	0
Solyc05g009360.2.1	1	10	0	3	2	28	7	37	0	18	3	10	0
Solyc05g009370.2.1	3	15	1	9	0	2	13	8	0	18	5	22	0
Solyc05g009380.2.1	0	0	0	0	0	0	0	0	0	0	0	0	0
Solyc05g009390.2.1	3	2	0	1	0	1	2	4	1	6	3	6	0
Solyc05g009400.1.1	0	0	0	0	0	0	0	0	0	0	0	0	0
Solyc05g009420.1.1	5	24	1	31	7	105	43	56	10	63	16	45	9
Solyc05g009430.2.1	0	0	0	0	0	0	0	0	0	0	0	0	0
Solyc05g009450.1.1	2	6	0	5	0	21	11	18	6	7	5	4	0
Solyc05g009460.2.1	0	0	0	0	0	0	0	0	0	0	0	0	0

Solyc05g009470.2.1	16	430	51	142	36	143	210	131	37	352	79	300	16
Solyc05g009480.1.1	0	0	0	0	0	0	0	0	0	0	0	0	0
Solyc05g009500.2.1	7	275	52	198	0	328	20	235	0	121	6	99	0
Solyc05g009520.1.1	5	81	3	29	0	205	127	185	21	16	9	4	0
Solyc05g009530.2.1	8	67	0	22	1	57	51	109	5	87	8	58	1
Solyc05g009540.2.1	20	112	13	50	8	237	121	358	46	98	66	101	9
Solyc05g009550.2.1	5	29	3	12	4	38	10	41	12	81	19	180	3
Solyc05g009560.2.1	0	0	0	0	0	0	0	0	0	0	0	0	0
Solyc05g009570.2.1	7	229	24	97	0	29	97	78	0	60	1	91	0
Solyc05g009600.2.1	9	357	27	192	13	479	167	677	32	520	91	311	7
Solyc05g009610.1.1	3	45	3	54	0	44	16	10	0	16	0	8	0
Solyc05g009630.2.1	3	19	1	20	0	16	20	30	1	66	1	42	0
Solyc05g009650.2.1	2	63	23	89	7	20	4	8	0	3	0	14	0
Solyc05g009660.2.1	0	0	0	0	0	0	0	0	0	0	0	0	0
Solyc05g009670.2.1	1	3	1	1	0	3	8	4	1	2	2	7	0
Solyc05g009680.1.1	7	82	4	40	2	19	28	85	0	89	12	85	1
Solyc05g009690.2.1	6	128	7	104	2	296	113	266	9	156	37	85	1
Solyc05g009700.2.1	1	0	0	5	0	24	8	15	0	0	2	0	0
Solyc05g009710.2.1	5	9	0	1	0	23	8	10	3	15	9	25	1
Solyc05g009720.2.1	2	9	3	0	1	4	11	11	4	5	0	5	0
Solyc05g009730.1.1	0	0	0	0	0	0	0	0	0	0	0	0	0
Solyc05g009740.1.1	3	6	0	12	0	33	14	15	0	24	1	19	0
Solyc05g009760.1.1	1	3	2	7	0	5	0	9	0	0	1	0	0
Solyc05g009780.2.1	5	45	15	57	0	44	63	32	1	95	13	45	1
Solyc05g009790.1.1	12	1394	167	1063	74	140	60	258	10	152	6	106	0
Solyc05g009800.2.1	6	20	9	30	2	14	11	35	2	12	7	24	0
Solyc05g009820.2.1	4	127	13	40	13	1	0	15	0	124	10	82	0
Solyc05g009840.2.1	5	38	1	25	0	42	18	52	0	11	3	14	0
Solyc05g009850.2.1	9	35	3	39	3	96	21	110	8	69	23	88	0
Solyc05g009870.2.1	6	39	3	31	4	104	34	150	6	75	4	54	0
Solyc05g009880.2.1	1	5	0	2	0	4	4	9	0	0	0	5	0
Solyc05g009890.1.1	1	5	1	3	0	0	3	0	0	11	0	5	0
Solyc05g009900.1.1	0	0	0	0	0	0	0	0	0	0	0	0	0
Solyc05g009910.2.1	2	17	3	17	1	18	9	50	0	17	1	30	0
Solyc05g009920.2.1	7	18	1	30	0	43	30	65	1	33	6	39	0
Solyc05g009930.2.1	20	641	169	656	23	312	379	745	22	193	130	198	5
Solyc05g009940.2.1	8	32	3	6	0	47	51	70	5	50	3	34	3
Solyc05g009950.2.1	0	0	0	0	0	0	0	0	0	0	0	0	0
Solyc05g009980.2.1	17	596	70	481	39	1900	607	2075	114	2264	433	1748	24
Solyc05g009990.2.1	2	8	9	0	1	0	3	2	0	41	3	139	0
Solyc05g010000.1.1	0	0	0	0	0	0	0	0	0	0	0	0	0

Solyc05g010010.1.1	0	0	0	0	0	0	0	0	0	0	0	0	0
Solyc05g010020.2.1	0	0	0	0	0	0	0	0	0	0	0	0	0
Solyc05g010040.2.1	1	31	7	17	0	1	0	3	0	1	0	0	0
Solyc05g010050.2.1	3	10	0	5	0	8	12	9	3	2	2	10	0
Solyc05g010060.2.1	0	0	0	0	0	0	0	0	0	0	0	0	0
Solyc05g010070.2.1	2	5	0	3	0	11	8	4	0	7	0	6	0
Solyc05g010080.2.1	9	196	5	59	0	111	108	219	9	46	27	32	0
Solyc05g010100.2.1	12	45	14	51	4	192	85	171	14	155	23	85	0
Solyc05g010110.1.1	9	20	9	28	2	62	49	94	22	74	16	51	2
Solyc05g010120.2.1	9	21	8	40	4	65	27	44	11	31	15	30	3
Solyc05g010130.1.1	0	0	0	0	0	0	0	0	0	0	0	0	0
Solyc05g010140.2.1	8	20	5	17	1	24	20	32	10	26	18	27	0
Solyc05g010150.2.1	0	0	0	0	0	0	0	0	0	0	0	0	0
Solyc05g010160.2.1	9	10	5	12	0	52	32	56	6	110	32	87	0
Solyc05g010180.2.1	5	48	2	31	1	1	6	2	4	37	22	47	0
Solyc05g010240.2.1	2	1	2	1	0	4	1	1	0	5	0	5	0
Solyc05g010250.1.1	9	339	25	126	13	159	131	275	30	278	42	267	4
Solyc05g010260.2.1	5	39	5	5	0	99	36	91	14	115	24	74	1
Solyc05g010270.1.1	0	0	0	0	0	0	0	0	0	0	0	0	0
Solyc05g010280.2.1	3	49	3	29	6	49	38	77	3	43	2	25	1
Solyc05g010290.1.1	0	0	0	0	0	0	0	0	0	0	0	0	0
Solyc05g010300.2.1	3	9	1	13	3	46	7	29	2	34	23	27	0
Solyc05g010320.2.1	10	362	15	72	61	59	163	81	46	134	86	77	7
Solyc05g010330.2.1	0	0	0	0	0	0	0	0	0	0	0	0	0
Solyc05g010340.2.1	5	12	0	0	2	45	19	20	7	11	20	4	1
Solyc05g010350.2.1	0	0	0	0	0	0	0	0	0	0	0	0	0
Solyc05g010400.2.1	1	2	0	17	0	6	6	5	0	15	0	10	0
Solyc05g010420.1.1	5	598	106	245	1	2550	1925	2840	93	1125	546	847	24
Solyc05g010430.2.1	2	11	1	8	0	35	26	51	6	21	6	22	0
Solyc05g010450.1.1	5	45	3	25	0	158	114	169	3	39	15	24	0
Solyc05g010470.2.1	0	0	0	0	0	0	0	0	0	0	0	0	0
Solyc05g010480.2.1	3	6	0	1	0	10	12	12	0	5	0	22	0
Solyc05g010530.2.1	0	0	0	0	0	0	0	0	0	0	0	0	0
Solyc05g010540.2.1	0	0	0	0	0	0	0	0	0	0	0	0	0
Solyc05g010570.2.1	0	0	0	0	0	0	0	0	0	0	0	0	0
Solyc05g010590.2.1	9	65	10	28	1	29	22	39	6	16	10	7	1
Solyc05g010610.2.1	1	1	2	0	0	3	9	4	0	6	0	8	0
Solyc05g010620.1.1	0	0	0	0	0	0	0	0	0	0	0	0	0
Solyc05g010640.2.1	0	0	0	0	0	0	0	0	0	0	0	0	0
Solyc05g010650.2.1	0	0	0	0	0	0	0	0	0	0	0	0	0
Solyc05g010660.2.1	4	4	2	7	0	17	21	21	5	18	8	10	0

Solyc05g010670.2.1	11	163	41	170	3	315	420	589	34	361	217	542	14
Solyc05g010680.1.1	0	0	0	0	0	0	0	0	0	0	0	0	0
Solyc05g010690.1.1	0	0	0	0	0	0	0	0	0	0	0	0	0
Solyc05g010710.1.1	0	0	0	0	0	0	0	0	0	0	0	0	0
Solyc05g010720.2.1	0	0	0	0	0	0	0	0	0	0	0	0	0
Solyc05g010760.1.1	0	0	0	0	0	0	0	0	0	0	0	0	0
Solyc05g010770.2.1	0	0	0	0	0	0	0	0	0	0	0	0	0
Solyc05g010780.1.1	4	2	0	7	0	9	19	27	3	33	3	28	0
Solyc05g010790.1.1	0	0	0	0	0	0	0	0	0	0	0	0	0
Solyc05g010800.2.1	0	0	0	0	0	0	0	0	0	0	0	0	0
Solyc05g010810.1.1	14	289	30	334	18	409	268	701	33	1486	248	1313	8
Solyc05g011810.2.1	2	18	2	2	0	16	28	17	1	37	11	22	2
Solyc05g011820.1.1	0	0	0	0	0	0	0	0	0	0	0	0	0
Solyc05g011830.2.1	4	15	1	13	4	39	14	31	20	14	18	11	0
Solyc05g011840.2.1	0	0	0	0	0	0	0	0	0	0	0	0	0
Solyc05g011860.1.1	0	0	0	0	0	0	0	0	0	0	0	0	0
Solyc05g011890.1.1	0	0	0	0	0	0	0	0	0	0	0	0	0
Solyc05g011920.2.1	3	14	2	3	0	11	15	10	2	13	15	8	0
Solyc05g011930.2.1	3	1	1	0	3	8	14	14	3	9	6	4	0
Solyc05g011940.2.1	0	0	0	0	0	0	0	0	0	0	0	0	0
Solyc05g011960.2.1	0	0	0	0	0	0	0	0	0	0	0	0	0
Solyc05g011970.2.1	0	0	0	0	0	0	0	0	0	0	0	0	0
Solyc05g011980.2.1	5	69	17	36	5	33	37	34	19	519	47	408	21
Solyc05g011990.2.1	7	7	0	12	2	56	24	69	13	14	15	10	0
Solyc05g012000.2.1	0	0	0	0	0	0	0	0	0	0	0	0	0
Solyc05g012010.2.1	3	5	4	11	0	8	6	6	3	16	8	19	0
Solyc05g012020.2.1	0	0	0	0	0	0	0	0	0	0	0	0	0
Solyc05g012030.1.1	0	0	0	0	0	0	0	0	0	0	0	0	0
Solyc05g012040.2.1	3	7	2	2	0	5	19	11	8	14	4	3	0
Solyc05g012060.2.1	0	0	0	0	0	0	0	0	0	0	0	0	0
Solyc05g012070.2.1	3	51	3	19	12	187	66	51	23	128	20	93	2
Solyc05g012080.2.1	1	2	0	5	0	16	2	8	3	13	2	5	0
Solyc05g012090.2.1	1	2	0	0	0	2	2	10	1	7	2	0	0
Solyc05g012100.2.1	13	35	6	39	0	68	164	194	5	69	8	56	0
Solyc05g012110.2.1	6	87	32	61	18	166	376	324	47	130	15	52	4
Solyc05g012120.2.1	5	27	1	4	0	34	28	47	2	20	4	29	0
Solyc05g012130.2.1	16	113	16	88	5	313	105	266	25	140	34	116	6
Solyc05g012140.2.1	7	27	1	43	0	47	63	122	6	126	13	89	0
Solyc05g012150.2.1	5	14	7	12	5	15	67	25	12	19	15	33	0
Solyc05g012160.2.1	2	239	35	185	7	254	409	246	36	913	185	1991	65
Solyc05g012180.2.1	0	0	0	0	0	0	0	0	0	0	0	0	0

Solyc05g012190.2.1	3	1	0	2	0	7	20	15	0	17	1	15	0
Solyc05g012200.2.1	1	14	0	4	0	32	16	32	3	16	5	27	0
Solyc05g012210.2.1	8	24	4	15	5	433	221	346	42	6	1	2	0
Solyc05g012220.2.1	0	0	0	0	0	0	0	0	0	0	0	0	0
Solyc05g012230.2.1	6	52	6	35	0	76	27	13	9	2	1	6	0
Solyc05g012250.1.1	0	0	0	0	0	0	0	0	0	0	0	0	0
Solyc05g012260.2.1	10	64	2	31	0	197	100	261	16	161	38	145	2
Solyc05g012270.2.1	3	20	4	10	0	52	11	46	0	43	11	51	1
Solyc05g012280.2.1	0	0	0	0	0	0	0	0	0	0	0	0	0
Solyc05g012290.2.1	4	16	2	15	0	24	25	20	0	62	5	62	1
Solyc05g012300.1.1	0	0	0	0	0	0	0	0	0	0	0	0	0
Solyc05g012310.2.1	1	0	0	2	0	0	6	2	2	5	0	4	0
Solyc05g012320.1.1	0	0	0	0	0	0	0	0	0	0	0	0	0
Solyc05g012330.2.1	1	5	3	1	2	1	14	2	4	0	5	0	0
Solyc05g012340.2.1	2	11	1	1	1	0	14	15	1	21	2	28	0
Solyc05g012350.2.1	0	0	0	0	0	0	0	0	0	0	0	0	0
Solyc05g012360.2.1	0	0	0	0	0	0	0	0	0	0	0	0	0
Solyc05g012370.2.1	2	11	9	9	0	15	18	10	0	5	0	10	0
Solyc05g012380.2.1	4	35	3	52	6	12	7	7	0	37	3	34	2
Solyc05g012390.2.1	7	21	1	15	0	71	19	70	4	64	12	47	0
Solyc05g012400.2.1	1	15	0	18	0	12	4	25	0	13	2	18	1
Solyc05g012410.1.1	0	0	0	0	0	0	0	0	0	0	0	0	0
Solyc05g012430.1.1	0	0	0	0	0	0	0	0	0	0	0	0	0
Solyc05g012440.2.1	1	1	1	0	0	7	7	13	2	10	0	0	0
Solyc05g012450.2.1	7	28	2	24	0	22	24	30	3	31	24	31	0
Solyc05g012460.2.1	2	6	0	9	0	12	26	20	2	19	4	12	0
Solyc05g012480.2.1	10	140	24	109	17	320	331	526	89	172	37	144	2
Solyc05g012490.1.1	0	0	0	0	0	0	0	0	0	0	0	0	0
Solyc05g012500.2.1	7	30	4	4	15	37	20	43	15	74	20	66	7
Solyc05g012510.2.1	26	346	33	234	17	38	453	87	12	1718	190	1310	11
Solyc05g012520.2.1	0	0	0	0	0	0	0	0	0	0	0	0	0
Solyc05g012540.2.1	0	0	0	0	0	0	0	0	0	0	0	0	0
Solyc05g012550.2.1	0	0	0	0	0	0	0	0	0	0	0	0	0
Solyc05g012560.1.1	7	39	6	10	0	25	46	32	4	101	14	71	3
Solyc05g012570.2.1	0	0	0	0	0	0	0	0	0	0	0	0	0
Solyc05g012580.1.1	3	2157	47	1000	111	2393	1479	3809	323	1628	38	1026	19
Solyc05g012590.2.1	3	13	3	10	1	42	4	50	1	46	8	34	1
Solyc05g012600.2.1	4	85	17	30	0	17	137	41	6	42	20	57	0
Solyc05g012610.2.1	12	64	4	48	5	77	126	124	29	142	34	135	5
Solyc05g012620.2.1	8	73	9	56	2	25	56	44	3	21	19	34	0
Solyc05g012630.1.1	0	0	0	0	0	0	0	0	0	0	0	0	0

Solyc05g012640.2.1	4	18	3	36	0	40	10	32	3	88	16	10	0
Solyc05g012650.2.1	14	58	10	51	0	46	79	88	12	66	17	55	1
Solyc05g012660.1.1	0	0	0	0	0	0	0	0	0	0	0	0	0
Solyc05g012670.1.1	0	0	0	0	0	0	0	0	0	0	0	0	0
Solyc05g012680.2.1	0	0	0	0	0	0	0	0	0	0	0	0	0
Solyc05g012690.2.1	2	8	2	4	0	9	31	43	3	23	2	19	0
Solyc05g012700.2.1	9	86	5	42	4	58	101	143	0	44	3	33	2
Solyc05g012710.2.1	3	44	7	20	0	49	25	35	0	21	2	13	1
Solyc05g012720.2.1	0	0	0	0	0	0	0	0	0	0	0	0	0
Solyc05g012730.1.1	0	0	0	0	0	0	0	0	0	0	0	0	0
Solyc05g012740.1.1	0	0	0	0	0	0	0	0	0	0	0	0	0
Solyc05g012770.2.1	3	7	0	14	0	114	13	50	2	11	2	6	0
Solyc05g012780.2.1	0	0	0	0	0	0	0	0	0	0	0	0	0
Solyc05g012790.2.1	4	30	1	7	1	0	18	13	4	12	8	11	0
Solyc05g012800.1.1	3	11	2	5	0	7	16	7	0	42	4	24	0
Solyc05g012810.1.1	1	7	6	2	2	2	3	0	3	4	0	2	0
Solyc05g012820.1.1	0	0	0	0	0	0	0	0	0	0	0	0	0
Solyc05g012850.2.1	5	44	3	38	3	30	21	36	2	46	10	37	0
Solyc05g012890.1.1	1	5	2	2	0	1	3	11	3	0	0	0	0
Solyc05g012910.2.1	0	0	0	0	0	0	0	0	0	0	0	0	0
Solyc05g012920.1.1	2	13	0	1	0	22	16	43	0	43	1	10	5
Solyc05g012930.2.1	0	0	0	0	0	0	0	0	0	0	0	0	0
Solyc05g012940.2.1	1	1	0	0	0	10	16	2	3	1	0	0	0
Solyc05g012950.1.1	0	0	0	0	0	0	0	0	0	0	0	0	0
Solyc05g012970.2.1	0	0	0	0	0	0	0	0	0	0	0	0	0
Solyc05g013010.2.1	0	0	0	0	0	0	0	0	0	0	0	0	0
Solyc05g013030.1.1	14	302	62	179	18	1200	490	1197	95	549	155	262	9
Solyc05g013040.2.1	5	17	4	6	0	46	21	32	1	29	15	39	1
Solyc05g013050.2.1	3	36	0	3	0	55	26	82	2	63	24	57	0
Solyc05g013060.2.1	8	55	1	35	1	383	81	313	3	34	8	5	0
Solyc05g013070.2.1	11	157	13	87	10	243	148	344	64	183	35	106	6
Solyc05g013130.2.1	0	0	0	0	0	0	0	0	0	0	0	0	0
Solyc05g013140.2.1	0	0	0	0	0	0	0	0	0	0	0	0	0
Solyc05g013150.2.1	3	15	5	7	0	16	13	10	0	12	7	5	0
Solyc05g013160.2.1	18	149	33	105	1	121	204	163	9	152	21	104	1
Solyc05g013170.2.1	3	14	0	14	3	15	7	20	8	17	8	20	0
Solyc05g013180.2.1	1	5	0	3	0	6	6	11	0	0	0	3	0
Solyc05g013190.2.1	0	0	0	0	0	0	0	0	0	0	0	0	0
Solyc05g013200.1.1	0	0	0	0	0	0	0	0	0	0	0	0	0
Solyc05g013210.2.1	0	0	0	0	0	0	0	0	0	0	0	0	0
Solyc05g013220.2.1	3	16	0	0	8	12	50	8	50	2	3	3	0

Solyc05g013240.2.1	0	0	0	0	0	0	0	0	0	0	0	0	0
Solyc05g013250.1.1	0	0	0	0	0	0	0	0	0	0	0	0	0
Solyc05g013260.1.1	0	0	0	0	0	0	0	0	0	0	0	0	0
Solyc05g013280.2.1	5	30	0	18	0	31	32	78	3	32	1	22	0
Solyc05g013290.1.1	1	4	0	0	0	0	1	0	0	14	1	4	0
Solyc05g013300.1.1	3	31	0	16	0	17	22	34	6	72	9	27	0
Solyc05g013310.1.1	0	0	0	0	0	0	0	0	0	0	0	0	0
Solyc05g013320.1.1	5	19	1	8	0	11	20	16	3	49	11	59	0
Solyc05g013330.2.1	0	0	0	0	0	0	0	0	0	0	0	0	0
Solyc05g013340.2.1	3	24	4	22	0	63	22	49	19	15	6	32	0
Solyc05g013360.2.1	0	0	0	0	0	0	0	0	0	0	0	0	0
Solyc05g013370.1.1	0	0	0	0	0	0	0	0	0	0	0	0	0
Solyc05g013380.2.1	3	440	40	325	6	86	79	101	7	111	26	77	0
Solyc05g013390.1.1	2	4	0	5	1	13	1	12	0	14	1	2	0
Solyc05g013400.2.1	2	3	0	0	1	17	3	11	0	25	1	20	0
Solyc05g013410.2.1	0	0	0	0	0	0	0	0	0	0	0	0	0
Solyc05g013440.2.1	12	654	29	469	0	216	59	300	2	379	87	149	15
Solyc05g013450.2.1	7	73	8	57	0	13	116	104	0	6	50	1	2
Solyc05g013460.2.1	7	51	17	25	2	32	16	37	0	104	3	81	2
Solyc05g013470.2.1	0	0	0	0	0	0	0	0	0	0	0	0	0
Solyc05g013480.2.1	7	253	11	157	5	256	112	331	9	166	18	133	0
Solyc05g013490.2.1	10	29	0	21	5	71	61	87	8	59	19	71	1
Solyc05g013500.2.1	0	0	0	0	0	0	0	0	0	0	0	0	0
Solyc05g013510.2.1	4	2284	43	652	0	297	70	468	3	1232	57	590	0
Solyc05g013530.2.1	3	50	10	42	0	101	18	134	0	24	13	11	0
Solyc05g013540.1.1	3	2	5	0	8	7	61	0	18	0	0	0	0
Solyc05g013560.2.1	3	71	2	13	7	2	20	7	0	0	0	2	0
Solyc05g013570.2.1	6	106	18	53	4	186	42	61	0	13	2	10	0
Solyc05g013580.2.1	8	234	10	232	2	259	79	245	22	189	12	166	9
Solyc05g013630.1.1	6	3407	205	1605	0	1554	709	3075	1	327	128	358	1
Solyc05g013640.1.1	7	334	11	104	1	561	297	834	0	198	45	169	0
Solyc05g013650.2.1	0	0	0	0	0	0	0	0	0	0	0	0	0
Solyc05g013660.2.1	4	11	0	4	0	34	13	41	9	3	0	8	1
Solyc05g013670.2.1	13	72	12	74	0	31	127	135	12	93	15	44	4
Solyc05g013680.2.1	0	0	0	0	0	0	0	0	0	0	0	0	0
Solyc05g013690.2.1	1	25	2	23	0	1	3	5	0	11	1	2	0
Solyc05g013700.2.1	8	142	8	97	3	186	59	229	15	137	61	97	3
Solyc05g013710.2.1	0	0	0	0	0	0	0	0	0	0	0	0	0
Solyc05g013720.2.1	17	158	22	114	21	333	340	476	20	506	99	321	18
Solyc05g013730.2.1	5	69	2	24	0	41	28	70	7	181	4	149	0
Solyc05g013740.2.1	11	29	1	22	3	46	31	70	2	75	24	71	1

Solyc05g013750.2.1	17	110	20	178	13	396	106	326	20	27	19	27	7
Solyc05g013760.2.1	0	0	0	0	0	0	0	0	0	0	0	0	0
Solyc05g013770.2.1	3	9	0	6	0	23	4	25	3	9	4	8	0
Solyc05g013780.2.1	4	59	1	49	2	151	35	87	17	47	24	30	0
Solyc05g013790.2.1	2	5	1	11	0	18	9	17	0	8	0	13	0
Solyc05g013800.1.1	0	0	0	0	0	0	0	0	0	0	0	0	0
Solyc05g013810.2.1	7	15	8	21	0	83	109	59	22	4	15	4	0
Solyc05g013820.2.1	5	102	3	65	1	160	42	173	27	227	28	146	0
Solyc05g013850.2.1	11	137	3	97	0	55	56	52	0	124	2	78	0
Solyc05g013860.2.1	0	0	0	0	0	0	0	0	0	0	0	0	0
Solyc05g013870.2.1	1	10	0	20	0	2	7	4	0	5	0	1	0
Solyc05g013880.1.1	1	34	0	9	0	1	3	0	0	1	0	0	0
Solyc05g013910.2.1	1	228	0	124	0	677	22	393	7	1009	22	277	0
Solyc05g013940.2.1	3	35	5	15	1	32	34	23	5	61	7	34	2
Solyc05g013960.2.1	8	51	9	43	11	16	58	57	3	95	32	86	3
Solyc05g013970.2.1	17	30	13	38	6	65	79	113	36	81	37	56	2
Solyc05g013990.2.1	10	54	15	38	12	183	132	242	27	244	115	228	5
Solyc05g014000.2.1	6	145	7	57	20	7	21	15	17	581	64	397	13
Solyc05g014030.2.1	8	106	9	61	3	180	211	215	17	227	36	147	3
Solyc05g014050.2.1	18	120	19	74	3	386	242	389	26	245	23	229	10
Solyc05g014070.1.1	0	0	0	0	0	0	0	0	0	0	0	0	0
Solyc05g014100.1.1	1	1	0	2	0	2	3	4	0	3	0	2	0
Solyc05g014110.2.1	0	0	0	0	0	0	0	0	0	0	0	0	0
Solyc05g014120.1.1	4	22	5	13	0	53	29	47	6	69	19	54	0
Solyc05g014130.2.1	2	0	0	4	0	13	15	16	0	12	3	6	0
Solyc05g014140.1.1	0	0	0	0	0	0	0	0	0	0	0	0	0
Solyc05g014150.2.1	1	3	0	0	3	2	6	1	7	0	1	0	0
Solyc05g014160.2.1	2	5	1	1	0	10	15	11	3	8	1	12	0
Solyc05g014170.1.1	0	0	0	0	0	0	0	0	0	0	0	0	0
Solyc05g014190.2.1	4	48	22	45	6	52	66	39	6	2	28	8	2
Solyc05g014200.2.1	25	57	7	59	5	126	135	237	29	179	36	107	3
Solyc05g014210.2.1	0	0	0	0	0	0	0	0	0	0	0	0	0
Solyc05g014230.2.1	0	0	0	0	0	0	0	0	0	0	0	0	0
Solyc05g014240.2.1	0	0	0	0	0	0	0	0	0	0	0	0	0
Solyc05g014250.2.1	4	8	1	7	0	13	26	15	2	17	0	26	0
Solyc05g014260.2.1	5	150	3	117	5	82	58	131	5	62	0	15	0
Solyc05g014270.2.1	0	0	0	0	0	0	0	0	0	0	0	0	0
Solyc05g014280.2.1	0	0	0	0	0	0	0	0	0	0	0	0	0
Solyc05g014310.2.1	6	172	29	146	0	137	54	101	5	129	24	144	1
Solyc05g014320.2.1	0	0	0	0	0	0	0	0	0	0	0	0	0
Solyc05g014340.2.1	6	21	3	20	3	58	23	73	8	26	16	14	0

Solyc05g014370.2.1	0	0	0	0	0	0	0	0	0	0	0	0	0
Solyc05g014380.2.1	7	4	1	11	0	58	15	118	0	24	27	27	0
Solyc05g014390.2.1	1	4	0	1	0	20	0	13	0	2	5	0	0
Solyc05g014400.2.1	0	0	0	0	0	0	0	0	0	0	0	0	0
Solyc05g014410.1.1	0	0	0	0	0	0	0	0	0	0	0	0	0
Solyc05g014470.2.1	16	2208	249	1063	312	2419	2066	2962	606	6449	3088	5813	237
Solyc05g014490.1.1	5	11	5	3	0	27	23	49	0	33	1	36	0
Solyc05g014500.1.1	0	0	0	0	0	0	0	0	0	0	0	0	0
Solyc05g014510.2.1	7	115	18	123	6	24	22	91	2	28	1	12	0
Solyc05g014540.2.1	1	5	0	2	0	0	0	6	0	23	8	9	0
Solyc05g014550.1.1	1	3	0	0	0	12	0	4	0	0	0	0	0
Solyc05g014560.2.1	0	0	0	0	0	0	0	0	0	0	0	0	0
Solyc05g014590.2.1	0	0	0	0	0	0	0	0	0	0	0	0	0
Solyc05g014640.2.1	5	24	5	14	2	10	33	26	2	37	15	16	0
Solyc05g014650.2.1	7	16	2	14	4	17	33	24	5	48	11	95	3
Solyc05g014690.1.1	0	0	0	0	0	0	0	0	0	0	0	0	0
Solyc05g014700.2.1	0	0	0	0	0	0	0	0	0	0	0	0	0
Solyc05g014710.2.1	0	0	0	0	0	0	0	0	0	0	0	0	0
Solyc05g014720.2.1	0	0	0	0	0	0	0	0	0	0	0	0	0
Solyc05g014730.1.1	0	0	0	0	0	0	0	0	0	0	0	0	0
Solyc05g014740.1.1	0	0	0	0	0	0	0	0	0	0	0	0	0
Solyc05g014750.2.1	0	0	0	0	0	0	0	0	0	0	0	0	0
Solyc05g014760.2.1	13	194	18	124	21	255	111	217	23	159	36	105	3
Solyc05g014790.2.1	20	104	17	24	31	132	362	291	104	328	48	208	14
Solyc05g014970.2.1	8	19	3	14	1	62	58	39	4	46	2	48	0
Solyc05g014980.2.1	1	5	0	0	0	18	5	9	2	0	2	3	3
Solyc05g015040.2.1	0	0	0	0	0	0	0	0	0	0	0	0	0
Solyc05g015050.2.1	19	107	13	79	15	117	173	176	10	292	39	227	0
Solyc05g015060.2.1	0	0	0	0	0	0	0	0	0	0	0	0	0
Solyc05g015070.2.1	19	115	9	68	15	461	252	502	115	161	32	137	3
Solyc05g015150.2.1	3	27	3	26	2	7	11	8	1	14	5	2	0
Solyc05g015210.2.1	10	31	1	16	2	75	38	98	10	37	12	34	2
Solyc05g015220.2.1	3	20	3	8	0	61	27	79	3	58	4	39	0
Solyc05g015250.2.1	0	0	0	0	0	0	0	0	0	0	0	0	0
Solyc05g015310.2.1	3	10	3	0	0	0	42	15	2	14	3	15	0
Solyc05g015320.2.1	0	0	0	0	0	0	0	0	0	0	0	0	0
Solyc05g015330.1.1	0	0	0	0	0	0	0	0	0	0	0	0	0
Solyc05g015380.2.1	0	0	0	0	0	0	0	0	0	0	0	0	0
Solyc05g015390.2.1	14	660	65	478	7	1381	220	982	51	2571	631	2042	13
Solyc05g015420.2.1	4	40	4	11	7	36	25	29	5	90	6	25	0
Solyc05g015460.2.1	0	0	0	0	0	0	0	0	0	0	0	0	0

Solyc05g015470.1.1	0	0	0	0	0	0	0	0	0	0	0	0	0
Solyc05g015480.2.1	0	0	0	0	0	0	0	0	0	0	0	0	0
Solyc05g015490.2.1	8	116	21	6	53	430	290	192	380	13	40	1	14
Solyc05g015500.2.1	4	82	13	44	7	76	109	96	17	213	70	138	2
Solyc05g015510.2.1	6	40	1	37	0	56	47	90	8	79	18	103	1
Solyc05g015520.2.1	1	5	1	3	0	21	8	40	0	18	3	27	5
Solyc05g015530.2.1	0	0	0	0	0	0	0	0	0	0	0	0	0
Solyc05g015610.2.1	21	134	33	155	11	679	631	1031	41	382	29	216	3
Solyc05g015630.2.1	5	60	8	9	0	40	43	61	3	182	10	99	3
Solyc05g015640.2.1	8	26	2	8	0	75	64	97	15	89	18	67	0
Solyc05g015650.2.1	14	60	2	52	3	102	64	160	16	138	44	89	0
Solyc05g015710.2.1	6	50	12	32	3	59	143	143	12	99	12	67	5
Solyc05g015720.1.1	0	0	0	0	0	0	0	0	0	0	0	0	0
Solyc05g015750.2.1	2	0	0	0	0	0	0	0	0	91	7	82	0
Solyc05g015800.2.1	0	0	0	0	0	0	0	0	0	0	0	0	0
Solyc05g015820.2.1	2	4	0	1	1	15	4	17	1	2	5	7	0
Solyc05g015840.2.1	0	0	0	0	0	0	0	0	0	0	0	0	0
Solyc05g015850.2.1	0	0	0	0	0	0	0	0	0	0	0	0	0
Solyc05g015860.2.1	11	48	5	29	4	218	626	336	21	172	113	136	3
Solyc05g015880.2.1	2	27	4	19	0	2	13	1	0	0	0	0	0
Solyc05g015890.2.1	0	0	0	0	0	0	0	0	0	0	0	0	0
Solyc05g015920.2.1	14	75	16	48	0	174	86	193	16	86	24	101	0
Solyc05g015930.2.1	12	29	5	22	2	63	49	98	13	66	13	80	3
Solyc05g015950.2.1	4	11	2	5	8	49	29	41	5	22	3	17	2
Solyc05g015980.2.1	1	5	1	0	0	1	1	2	0	6	2	7	0
Solyc05g016030.2.1	5	29	1	17	0	15	50	28	13	27	5	47	0
Solyc05g016060.2.1	1	36	0	14	1	211	31	90	9	5	2	21	0
Solyc05g016180.2.1	0	0	0	0	0	0	0	0	0	0	0	0	0
Solyc05g016190.2.1	0	0	0	0	0	0	0	0	0	0	0	0	0
Solyc05g016230.2.1	0	0	0	0	0	0	0	0	0	0	0	0	0
Solyc05g016240.2.1	9	115	9	79	4	296	144	485	42	177	30	133	3
Solyc05g016250.2.1	0	0	0	0	0	0	0	0	0	0	0	0	0
Solyc05g016310.1.1	0	0	0	0	0	0	0	0	0	0	0	0	0
Solyc05g016330.2.1	1	5	0	0	0	3	11	1	2	1	0	5	0
Solyc05g016380.2.1	0	0	0	0	0	0	0	0	0	0	0	0	0
Solyc05g016390.2.1	0	0	0	0	0	0	0	0	0	0	0	0	0
Solyc05g016410.2.1	3	79	12	52	18	154	184	142	14	157	29	110	4
Solyc05g016440.2.1	8	37	4	42	1	151	80	100	18	62	40	102	2
Solyc05g016460.1.1	11	19	10	26	5	103	88	113	12	36	15	43	0
Solyc05g017760.2.1	12	238	32	71	10	61	30	115	11	412	216	192	21
Solyc05g017840.2.1	0	0	0	0	0	0	0	0	0	0	0	0	0

Solyc05g017860.2.1	5	20	3	16	0	72	27	38	2	25	4	42	0
Solyc05g017890.2.1	21	94	8	156	12	368	134	299	43	134	44	158	0
Solyc05g017900.2.1	2	1	2	0	0	2	4	7	2	2	0	2	0
Solyc05g017940.2.1	1	0	0	1	2	7	9	1	1	1	5	1	0
Solyc05g017950.2.1	6	87	3	58	1	39	36	92	8	31	23	76	0
Solyc05g017980.2.1	7	53	8	46	9	46	60	54	9	122	34	83	3
Solyc05g017990.2.1	12	86	15	89	0	104	164	272	11	149	46	93	2
Solyc05g018050.1.1	0	0	0	0	0	0	0	0	0	0	0	0	0
Solyc05g018060.1.1	2	5	0	1	0	37	12	19	3	0	0	4	0
Solyc05g018120.1.1	0	0	0	0	0	0	0	0	0	0	0	0	0
Solyc05g018130.2.1	5	30	12	17	0	18	16	20	4	27	14	28	0
Solyc05g018150.2.1	2	7	0	2	0	63	11	33	1	31	2	43	0
Solyc05g018180.2.1	4	20	3	21	0	20	18	24	1	17	2	30	0
Solyc05g018210.2.1	4	42	4	19	1	41	36	64	4	111	4	82	2
Solyc05g018230.2.1	6	73	7	53	0	29	30	94	1	6	1	9	0
Solyc05g018240.2.1	7	29	6	32	1	56	76	156	5	53	9	56	0
Solyc05g018300.2.1	7	47	2	41	3	36	25	64	1	46	12	37	3
Solyc05g018320.2.1	9	111	10	68	3	128	59	205	16	233	31	150	3
Solyc05g018340.2.1	1	3	0	4	0	7	10	7	5	11	0	5	0
Solyc05g018350.2.1	0	0	0	0	0	0	0	0	0	0	0	0	0
Solyc05g018370.1.1	0	0	0	0	0	0	0	0	0	0	0	0	0
Solyc05g018390.2.1	2	10	5	13	1	51	14	36	1	45	2	19	0
Solyc05g018410.1.1	0	0	0	0	0	0	0	0	0	0	0	0	0
Solyc05g018490.2.1	6	71	6	45	5	172	75	132	17	89	32	69	0
Solyc05g018500.2.1	8	59	5	26	7	76	51	106	25	49	13	41	1
Solyc05g018510.2.1	12	89	13	14	46	18	103	29	42	306	15	42	20
Solyc05g018520.2.1	6	41	4	44	1	89	69	130	7	87	35	69	2
Solyc05g018570.2.1	2	19	3	9	0	15	16	28	1	44	14	35	0
Solyc05g018590.2.1	2	39	6	34	1	14	66	89	14	40	18	34	3
Solyc05g018600.2.1	1	3	0	5	0	6	7	15	4	18	3	12	0
Solyc05g018610.1.1	3	23	1	2	0	12	19	33	4	19	6	15	0
Solyc05g018620.2.1	5	69	7	46	1	14	47	56	9	80	41	109	1
Solyc05g018630.1.1	1	14	1	15	1	22	10	8	0	22	7	16	0
Solyc05g018640.2.1	0	0	0	0	0	0	0	0	0	0	0	0	0
Solyc05g018650.2.1	10	328	55	211	16	274	445	511	25	279	74	220	9
Solyc05g018700.2.1	10	63	1	69	2	249	56	245	10	162	21	234	6
Solyc05g018730.2.1	0	0	0	0	0	0	0	0	0	0	0	0	0
Solyc05g018750.2.1	6	34	3	18	3	74	28	69	5	146	14	74	3
Solyc05g018760.1.1	3	13	1	34	0	308	36	135	7	419	18	199	1
Solyc05g018810.2.1	4	54	8	62	1	159	48	201	9	293	39	241	4
Solyc05g018820.1.1	0	0	0	0	0	0	0	0	0	0	0	0	0

Solyc05g018880.1.1	0	0	0	0	0	0	0	0	0	0	0	0	0
Solyc05g020020.2.1	3	11	0	16	0	25	27	36	0	42	0	63	0
Solyc05g021090.2.1	0	0	0	0	0	0	0	0	0	0	0	0	0
Solyc05g021100.2.1	5	60	5	49	3	122	73	165	19	49	11	44	0
Solyc05g021110.2.1	0	0	0	0	0	0	0	0	0	0	0	0	0
Solyc05g021390.2.1	0	0	0	0	0	0	0	0	0	0	0	0	0
Solyc05g021510.1.1	0	0	0	0	0	0	0	0	0	0	0	0	0
Solyc05g021530.1.1	2	2	9	7	4	19	27	1	2	4	6	11	0
Solyc05g021540.1.1	0	0	0	0	0	0	0	0	0	0	0	0	0
Solyc05g021570.1.1	0	0	0	0	0	0	0	0	0	0	0	0	0
Solyc05g021580.2.1	0	0	0	0	0	0	0	0	0	0	0	0	0
Solyc05g023680.2.1	0	0	0	0	0	0	0	0	0	0	0	0	0
Solyc05g023710.2.1	7	35	1	10	1	81	34	65	9	70	13	96	3
Solyc05g023740.2.1	9	107	15	85	8	321	284	318	49	195	47	153	2
Solyc05g023750.2.1	5	17	1	4	1	22	13	36	1	108	10	58	0
Solyc05g023760.2.1	1	8	0	3	0	4	2	3	0	0	1	8	0
Solyc05g023770.2.1	0	0	0	0	0	0	0	0	0	0	0	0	0
Solyc05g023800.2.1	0	0	0	0	0	0	0	0	0	0	0	0	0
Solyc05g023820.2.1	1	26	1	2	3	19	6	27	3	11	8	12	0
Solyc05g023830.1.1	0	0	0	0	0	0	0	0	0	0	0	0	0
Solyc05g023900.1.1	1	0	0	0	0	0	1	1	0	6	3	6	0
Solyc05g023910.1.1	0	0	0	0	0	0	0	0	0	0	0	0	0
Solyc05g024010.2.1	3	6	0	3	0	8	15	20	1	50	4	23	0
Solyc05g024020.1.1	0	0	0	0	0	0	0	0	0	0	0	0	0
Solyc05g024160.2.1	0	0	0	0	0	0	0	0	0	0	0	0	0
Solyc05g024230.1.1	0	0	0	0	0	0	0	0	0	0	0	0	0
Solyc05g024240.2.1	2	34	1	15	1	29	12	118	10	47	4	11	0
Solyc05g024260.2.1	9	42	12	6	6	2059	2599	1207	67	185	173	239	10
Solyc05g024290.2.1	5	62	3	31	0	166	53	199	12	100	11	47	0
Solyc05g024370.1.1	0	0	0	0	0	0	0	0	0	0	0	0	0
Solyc05g024380.1.1	0	0	0	0	0	0	0	0	0	0	0	0	0
Solyc05g024410.2.1	5	27	1	18	0	38	17	55	0	309	12	182	5
Solyc05g024430.2.1	2	136	6	54	0	2	15	12	2	66	12	99	2
Solyc05g025500.2.1	0	0	0	0	0	0	0	0	0	0	0	0	0
Solyc05g025510.2.1	14	86	4	47	6	187	82	204	7	285	40	265	4
Solyc05g025570.1.1	1	0	1	0	8	0	1	0	0	1	31	0	3
Solyc05g025590.2.1	2	7	0	6	0	6	10	15	1	3	2	2	0
Solyc05g025600.1.1	5	4360	313	2978	24	105	504	238	8	748	168	861	11
Solyc05g025650.2.1	0	0	0	0	0	0	0	0	0	0	0	0	0
Solyc05g025680.1.1	0	0	0	0	0	0	0	0	0	0	0	0	0
Solyc05g025690.1.1	0	0	0	0	0	0	0	0	0	0	0	0	0

Solyc05g025740.2.1	16	82	9	52	13	297	179	246	18	229	38	131	5
Solyc05g025750.2.1	1	4	2	0	0	7	5	15	0	11	1	3	0
Solyc05g025810.2.1	1	25	0	27	0	32	0	28	0	61	0	47	0
Solyc05g025820.2.1	0	0	0	0	0	0	0	0	0	0	0	0	0
Solyc05g025890.1.1	3	29	3	23	0	0	10	4	0	5	0	9	0
Solyc05g025900.2.1	3	24	4	11	0	52	31	67	2	22	11	21	0
Solyc05g025910.2.1	0	0	0	0	0	0	0	0	0	0	0	0	0
Solyc05g026020.1.1	0	0	0	0	0	0	0	0	0	0	0	0	0
Solyc05g026050.2.1	3	53	14	24	5	111	105	99	12	146	83	90	8
Solyc05g026060.1.1	1	0	2	3	0	3	2	3	1	4	1	0	0
Solyc05g026070.1.1	3	2	0	0	0	24	12	14	10	26	1	10	0
Solyc05g026080.2.1	0	0	0	0	0	0	0	0	0	0	0	0	0
Solyc05g026130.2.1	2	39	6	6	0	87	6	62	1	53	6	59	0
Solyc05g026140.2.1	5	28	0	15	3	15	47	25	2	46	7	44	0
Solyc05g026180.1.1	0	0	0	0	0	0	0	0	0	0	0	0	0
Solyc05g026240.1.1	6	75	9	25	10	22	70	41	17	129	51	136	1
Solyc05g026310.2.1	2	50	3	41	0	136	10	74	9	65	19	23	5
Solyc05g026360.2.1	0	0	0	0	0	0	0	0	0	0	0	0	0
Solyc05g026380.1.1	1	5	0	3	0	0	6	3	0	4	0	5	0
Solyc05g026420.1.1	1	2	0	0	0	1	4	10	1	10	3	2	0
Solyc05g026460.1.1	0	0	0	0	0	0	0	0	0	0	0	0	0
Solyc05g026470.1.1	0	0	0	0	0	0	0	0	0	0	0	0	0
Solyc05g026480.1.1	0	0	0	0	0	0	0	0	0	0	0	0	0
Solyc05g026490.2.1	1	11	3	13	0	0	2	0	0	0	0	0	0
Solyc05g026510.2.1	13	143	15	126	5	1510	952	1290	105	357	146	335	8
Solyc05g026540.2.1	9	225	26	198	4	1304	116	470	38	824	47	387	13
Solyc05g026550.2.1	1	84	5	22	0	11	58	25	2	7	0	12	0
Solyc05g026560.2.1	10	88	7	52	1	130	93	425	6	142	12	101	0
Solyc05g026570.2.1	2	9	6	19	0	0	23	2	0	9	0	15	0
Solyc05g026590.1.1	0	0	0	0	0	0	0	0	0	0	0	0	0
Solyc05g026600.2.1	0	0	0	0	0	0	0	0	0	0	0	0	0
Solyc05g032620.1.1	0	0	0	0	0	0	0	0	0	0	0	0	0
Solyc05g032660.2.1	2	53	11	18	0	24	69	21	1	68	15	21	0
Solyc05g032680.2.1	0	0	0	0	0	0	0	0	0	0	0	0	0
Solyc05g032690.2.1	1	1	0	0	0	2	0	4	1	3	0	0	1
Solyc05g032700.2.1	0	0	0	0	0	0	0	0	0	0	0	0	0
Solyc05g032750.2.1	13	62	3	59	0	97	83	119	6	204	28	131	1
Solyc05g032760.2.1	0	0	0	0	0	0	0	0	0	0	0	0	0
Solyc05g032770.2.1	0	0	0	0	0	0	0	0	0	0	0	0	0
Solyc05g032820.1.1	0	0	0	0	0	0	0	0	0	0	0	0	0
Solyc05g032830.1.1	0	0	0	0	0	0	0	0	0	0	0	0	0

Solyc05g032850.2.1	13	37	8	71	7	317	217	381	41	105	39	97	1
Solyc05g032880.2.1	0	0	0	0	0	0	0	0	0	0	0	0	0
Solyc05g039940.2.1	0	0	0	0	0	0	0	0	0	0	0	0	0
Solyc05g039950.1.1	0	0	0	0	0	0	0	0	0	0	0	0	0
Solyc05g040050.1.1	14	132	20	139	7	298	145	279	21	167	35	104	4
Solyc05g041130.1.1	0	0	0	0	0	0	0	0	0	0	0	0	0
Solyc05g041190.1.1	0	0	0	0	0	0	0	0	0	0	0	0	0
Solyc05g041200.2.1	17	272	17	134	1	1764	402	1960	20	202	60	146	2
Solyc05g041420.2.1	1	6	1	0	0	5	5	7	5	2	0	4	0
Solyc05g041430.2.1	1	49	1	15	0	0	3	5	2	29	3	44	0
Solyc05g041530.2.1	8	109	4	113	6	59	57	74	1	296	4	219	5
Solyc05g041540.2.1	9	23	3	8	9	379	151	301	22	45	50	23	2
Solyc05g041580.1.1	0	0	0	0	0	0	0	0	0	0	0	0	0
Solyc05g041750.1.1	0	0	0	0	0	0	0	0	0	0	0	0	0
Solyc05g041820.1.1	0	0	0	0	0	0	0	0	0	0	0	0	0
Solyc05g041830.2.1	1	1	0	0	0	6	4	12	0	7	3	7	0
Solyc05g041860.2.1	0	0	0	0	0	0	0	0	0	0	0	0	0
Solyc05g041870.1.1	0	0	0	0	0	0	0	0	0	0	0	0	0
Solyc05g041910.2.1	8	439	65	312	32	229	98	193	46	135	18	220	3
Solyc05g041920.2.1	0	0	0	0	0	0	0	0	0	0	0	0	0
Solyc05g041990.1.1	0	0	0	0	0	0	0	0	0	0	0	0	0
Solyc05g042030.2.1	6	120	17	69	5	143	159	168	24	285	66	217	3
Solyc05g042040.2.1	0	0	0	0	0	0	0	0	0	0	0	0	0
Solyc05g042050.2.1	3	9	0	9	0	16	13	13	0	10	1	10	0
Solyc05g042070.2.1	0	0	0	0	0	0	0	0	0	0	0	0	0
Solyc05g042120.2.1	0	0	0	0	0	0	0	0	0	0	0	0	0
Solyc05g042170.1.1	0	0	0	0	0	0	0	0	0	0	0	0	0
Solyc05g043190.2.1	0	0	0	0	0	0	0	0	0	0	0	0	0
Solyc05g043240.1.1	0	0	0	0	0	0	0	0	0	0	0	0	0
Solyc05g043330.2.1	0	0	0	0	0	0	0	0	0	0	0	0	0
Solyc05g043340.2.1	0	0	0	0	0	0	0	0	0	0	0	0	0
Solyc05g043350.2.1	3	7	1	7	2	8	16	8	7	12	12	35	0
Solyc05g043400.1.1	0	0	0	0	0	0	0	0	0	0	0	0	0
Solyc05g043420.1.1	0	0	0	0	0	0	0	0	0	0	0	0	0
Solyc05g043430.2.1	3	66	3	47	0	67	32	89	7	224	33	177	2
Solyc05g044470.1.1	0	0	0	0	0	0	0	0	0	0	0	0	0
Solyc05g044480.2.1	2	7	3	3	0	0	9	17	1	3	1	6	0
Solyc05g044610.1.1	27	65	7	57	10	349	123	315	14	235	24	162	0
Solyc05g044620.1.1	0	0	0	0	0	0	0	0	0	0	0	0	0
Solyc05g044630.2.1	3	66	5	15	0	56	45	40	0	16	5	27	0
Solyc05g045650.2.1	8	25	10	7	1	25	52	44	22	11	25	11	0

Solyc05g045740.1.1	0	0	0	0	0	0	0	0	0	0	0	0	0
Solyc05g045760.1.1	0	0	0	0	0	0	0	0	0	0	0	0	0
Solyc05g045780.1.1	0	0	0	0	0	0	0	0	0	0	0	0	0
Solyc05g045830.1.1	0	0	0	0	0	0	0	0	0	0	0	0	0
Solyc05g045840.1.1	0	0	0	0	0	0	0	0	0	0	0	0	0
Solyc05g045870.1.1	0	0	0	0	0	0	0	0	0	0	0	0	0
Solyc05g045900.1.1	0	0	0	0	0	0	0	0	0	0	0	0	0
Solyc05g045950.1.1	0	0	0	0	0	0	0	0	0	0	0	0	0
Solyc05g046010.2.1	0	0	0	0	0	0	0	0	0	0	0	0	0
Solyc05g046120.2.1	0	0	0	0	0	0	0	0	0	0	0	0	0
Solyc05g046130.2.1	1	1	0	0	0	1	0	2	0	3	0	7	0
Solyc05g046140.2.1	0	0	0	0	0	0	0	0	0	0	0	0	0
Solyc05g046190.1.1	0	0	0	0	0	0	0	0	0	0	0	0	0
Solyc05g046270.2.1	0	0	0	0	0	0	0	0	0	0	0	0	0
Solyc05g046280.1.1	0	0	0	0	0	0	0	0	0	0	0	0	0
Solyc05g046290.2.1	0	0	0	0	0	0	0	0	0	0	0	0	0
Solyc05g046300.2.1	6	59	32	37	12	53	529	62	53	33	35	42	14
Solyc05g046310.2.1	5	27	0	7	0	45	15	24	2	49	12	22	0
Solyc05g046320.1.1	0	0	0	0	0	0	0	0	0	0	0	0	0
Solyc05g046330.2.1	12	76	4	42	7	121	58	156	4	196	23	218	4
Solyc05g046390.2.1	2	194	2	60	0	244	51	206	5	394	23	231	0
Solyc05g047390.1.1	1	22	1	18	1	32	3	27	1	14	2	7	0
Solyc05g047420.2.1	3	10	0	14	0	20	14	41	2	23	2	41	0
Solyc05g047450.1.1	8	83	32	47	4	112	152	146	36	109	35	166	2
Solyc05g047460.2.1	16	119	15	112	1	56	96	155	60	125	78	96	1
Solyc05g047510.1.1	0	0	0	0	0	0	0	0	0	0	0	0	0
Solyc05g047520.2.1	4	19	4	13	0	75	19	68	12	38	6	38	0
Solyc05g047530.2.1	0	0	0	0	0	0	0	0	0	0	0	0	0
Solyc05g047540.2.1	0	0	0	0	0	0	0	0	0	0	0	0	0
Solyc05g047550.2.1	1	6	2	5	0	1	3	8	0	0	0	0	0
Solyc05g047560.1.1	1	0	0	0	0	2	16	4	0	2	0	0	0
Solyc05g047590.2.1	3	245	0	120	0	5	0	20	0	39	13	31	0
Solyc05g047600.2.1	0	0	0	0	0	0	0	0	0	0	0	0	0
Solyc05g047610.2.1	1	15	0	9	4	19	7	26	0	4	0	3	0
Solyc05g047640.2.1	0	0	0	0	0	0	0	0	0	0	0	0	0
Solyc05g047680.2.1	0	0	0	0	0	0	0	0	0	0	0	0	0
Solyc05g047690.2.1	1	0	0	0	3	0	6	0	17	0	2	0	12
Solyc05g047700.1.1	0	0	0	0	0	0	0	0	0	0	0	0	0
Solyc05g047710.2.1	0	0	0	0	0	0	0	0	0	0	0	0	0
Solyc05g048740.2.1	0	0	0	0	0	0	0	0	0	0	0	0	0
Solyc05g048750.2.1	3	2	2	4	0	17	6	16	0	8	7	12	0

Solyc05g048760.2.1	1	23	0	19	2	17	12	46	0	84	5	136	0
Solyc05g048770.2.1	0	0	0	0	0	0	0	0	0	0	0	0	0
Solyc05g048810.2.1	10	40	8	11	0	93	62	61	5	237	28	253	1
Solyc05g048820.2.1	0	0	0	0	0	0	0	0	0	0	0	0	0
Solyc05g048830.2.1	0	0	0	0	0	0	0	0	0	0	0	0	0
Solyc05g048850.2.1	0	0	0	0	0	0	0	0	0	0	0	0	0
Solyc05g048860.2.1	2	5	0	10	0	12	3	10	0	7	6	16	0
Solyc05g049880.2.1	1	30	4	46	1	21	3	41	1	126	3	114	1
Solyc05g049900.2.1	5	38	8	26	0	55	44	50	7	59	7	55	0
Solyc05g049940.2.1	2	2	2	2	0	5	3	6	3	13	7	11	1
Solyc05g049950.2.1	3	23	4	13	1	102	37	64	10	61	27	68	1
Solyc05g049960.2.1	2	1	0	0	0	2	3	3	0	12	1	25	0
Solyc05g049970.2.1	2	13	1	12	1	9	10	6	6	9	3	11	0
Solyc05g049980.2.1	3	41	0	43	0	97	46	378	0	273	11	119	0
Solyc05g049990.2.1	2	21	1	16	4	27	24	34	0	29	0	26	0
Solyc05g050000.2.1	5	2	0	8	0	27	29	12	2	8	22	16	0
Solyc05g050010.2.1	1	0	0	0	0	0	0	0	0	5	8	4	0
Solyc05g050020.2.1	6	19	4	10	0	43	47	30	3	67	7	83	0
Solyc05g050090.2.1	1	65	2	56	0	27	2	31	0	0	0	0	0
Solyc05g050110.2.1	4	40	17	26	0	8	124	14	4	1	8	4	0
Solyc05g050120.2.1	16	1168	145	729	12	1628	550	2627	24	3496	414	2364	14
Solyc05g050130.2.1	5	345	11	394	1	327	31	636	0	79	0	22	0
Solyc05g050200.2.1	3	260	2	116	0	338	25	362	8	252	29	150	3
Solyc05g050210.2.1	0	0	0	0	0	0	0	0	0	0	0	0	0
Solyc05g050220.2.1	2	0	0	0	0	271	8	47	1	16	2	3	0
Solyc05g050230.2.1	2	1547	55	1176	33	470	213	725	33	1324	87	1195	12
Solyc05g050280.2.1	16	160	41	127	7	298	346	316	16	12	49	12	0
Solyc05g050290.1.1	0	0	0	0	0	0	0	0	0	0	0	0	0
Solyc05g050320.2.1	0	0	0	0	0	0	0	0	0	0	0	0	0
Solyc05g050330.2.1	1	1	1	3	0	4	2	4	0	1	6	2	0
Solyc05g050340.2.1	0	0	0	0	0	0	0	0	0	0	0	0	0
Solyc05g050350.1.1	0	0	0	0	0	0	0	0	0	0	0	0	0
Solyc05g050360.2.1	3	9	7	10	1	18	16	9	10	0	2	0	0
Solyc05g050380.2.1	7	65	15	44	16	79	43	51	6	37	10	24	3
Solyc05g050390.2.1	11	62	4	54	7	180	115	163	20	201	31	93	3
Solyc05g050430.1.1	0	0	0	0	0	0	0	0	0	0	0	0	0
Solyc05g050490.2.1	1	2	0	0	0	3	5	3	0	0	0	2	0
Solyc05g050500.1.1	13	570	399	417	35	284	529	425	32	169	279	184	7
Solyc05g050520.2.1	0	0	0	0	0	0	0	0	0	0	0	0	0
Solyc05g050530.2.1	4	7	1	4	0	7	13	24	0	21	1	8	0
Solyc05g050540.2.1	0	0	0	0	0	0	0	0	0	0	0	0	0

Solyc05g050550.2.1	1	9	0	2	0	0	5	3	0	0	0	0	0
Solyc05g050560.1.1	11	38	10	44	2	210	43	76	16	14	11	10	5
Solyc05g050570.2.1	0	0	0	0	0	0	0	0	0	0	0	0	0
Solyc05g050580.2.1	4	32	5	16	0	46	33	48	8	146	15	85	0
Solyc05g050600.2.1	3	29	1	30	2	141	74	93	15	85	36	46	0
Solyc05g050610.1.1	1	3	1	6	0	17	10	24	3	3	3	7	0
Solyc05g050620.2.1	1	5	2	7	0	1	5	4	0	0	2	13	0
Solyc05g050630.2.1	8	39	8	49	0	63	50	102	6	51	9	66	0
Solyc05g050680.2.1	4	51	8	56	2	117	30	148	19	109	16	85	0
Solyc05g050700.1.1	0	0	0	0	0	0	0	0	0	0	0	0	0
Solyc05g050710.2.1	2	5	0	3	0	3	9	3	1	27	2	12	0
Solyc05g050720.2.1	0	0	0	0	0	0	0	0	0	0	0	0	0
Solyc05g050740.2.1	0	0	0	0	0	0	0	0	0	0	0	0	0
Solyc05g050750.1.1	0	0	0	0	0	0	0	0	0	0	0	0	0
Solyc05g050770.2.1	2	13	0	0	0	0	8	6	0	1	6	4	0
Solyc05g050780.2.1	0	0	0	0	0	0	0	0	0	0	0	0	0
Solyc05g050800.2.1	2	112	8	53	12	78	21	132	14	48	3	21	0
Solyc05g050810.2.1	0	0	0	0	0	0	0	0	0	0	0	0	0
Solyc05g050820.2.1	0	0	0	0	0	0	0	0	0	0	0	0	0
Solyc05g050830.1.1	5	6	0	1	2	0	6	2	1	93	7	77	0
Solyc05g050840.2.1	3	2	5	4	0	3	32	13	1	17	6	21	2
Solyc05g050850.2.1	0	0	0	0	0	0	0	0	0	0	0	0	0
Solyc05g050900.2.1	0	0	0	0	0	0	0	0	0	0	0	0	0
Solyc05g050910.2.1	0	0	0	0	0	0	0	0	0	0	0	0	0
Solyc05g050920.2.1	0	0	0	0	0	0	0	0	0	0	0	0	0
Solyc05g050930.1.1	4	23	1	22	2	35	26	31	6	53	13	63	4
Solyc05g050940.2.1	2	0	0	0	0	3	5	1	0	1	1	8	1
Solyc05g050950.2.1	0	0	0	0	0	0	0	0	0	0	0	0	0
Solyc05g050960.2.1	0	0	0	0	0	0	0	0	0	0	0	0	0
Solyc05g050970.2.1	16	218	13	125	24	372	124	844	56	873	117	614	8
Solyc05g050980.2.1	6	23	6	9	0	3	19	18	13	88	12	72	0
Solyc05g050990.1.1	0	0	0	0	0	0	0	0	0	0	0	0	0
Solyc05g051000.2.1	0	0	0	0	0	0	0	0	0	0	0	0	0
Solyc05g051010.2.1	0	0	0	0	0	0	0	0	0	0	0	0	0
Solyc05g051030.2.1	0	0	0	0	0	0	0	0	0	0	0	0	0
Solyc05g051040.2.1	5	52	3	31	1	81	44	88	7	70	32	41	2
Solyc05g051050.2.1	7	7	0	4	0	15	19	30	3	33	2	15	0
Solyc05g051060.2.1	2	18	0	7	2	34	2	15	1	39	11	36	1
Solyc05g051070.2.1	3	14	1	16	0	37	6	35	2	7	6	12	0
Solyc05g051180.1.1	0	0	0	0	0	0	0	0	0	0	0	0	0
Solyc05g051200.1.1	0	0	0	0	0	0	0	0	0	0	0	0	0

Solyc05g051210.2.1	0	0	0	0	0	0	0	0	0	0	0	0	0
Solyc05g051220.2.1	0	0	0	0	0	0	0	0	0	0	0	0	0
Solyc05g051230.2.1	2	94	3	83	4	50	79	171	9	152	11	236	0
Solyc05g051240.1.1	13	1094	20	335	25	150	32	96	27	181	70	109	6
Solyc05g051250.2.1	0	0	0	0	0	0	0	0	0	0	0	0	0
Solyc05g051260.2.1	0	0	0	0	0	0	0	0	0	0	0	0	0
Solyc05g051270.2.1	0	0	0	0	0	0	0	0	0	0	0	0	0
Solyc05g051280.2.1	3	24	1	13	0	20	4	9	0	10	5	6	0
Solyc05g051290.2.1	1	3	0	4	0	0	0	5	0	5	4	2	0
Solyc05g051310.1.1	0	0	0	0	0	0	0	0	0	0	0	0	0
Solyc05g051330.1.1	1	0	0	0	0	1	7	1	0	6	0	6	0
Solyc05g051340.1.1	1	0	0	0	0	8	6	6	1	4	0	4	0
Solyc05g051350.1.1	0	0	0	0	0	0	0	0	0	0	0	0	0
Solyc05g051360.1.1	0	0	0	0	0	0	0	0	0	0	0	0	0
Solyc05g051370.2.1	3	13	0	12	0	13	22	25	3	9	12	16	0
Solyc05g051380.2.1	0	0	0	0	0	0	0	0	0	0	0	0	0
Solyc05g051400.2.1	13	417	102	255	74	274	452	93	21	733	266	658	36
Solyc05g051410.2.1	0	0	0	0	0	0	0	0	0	0	0	0	0
Solyc05g051430.2.1	0	0	0	0	0	0	0	0	0	0	0	0	0
Solyc05g051450.1.1	0	0	0	0	0	0	0	0	0	0	0	0	0
Solyc05g051460.2.1	5	22	0	28	2	34	25	66	2	39	3	60	0
Solyc05g051490.2.1	6	10	6	19	0	44	25	51	15	16	23	16	0
Solyc05g051500.2.1	2	76	1	59	0	74	40	95	4	118	11	77	0
Solyc05g051510.2.1	0	0	0	0	0	0	0	0	0	0	0	0	0
Solyc05g051520.2.1	9	14	8	3	0	14	32	69	12	42	14	58	3
Solyc05g051530.2.1	1	12	0	9	0	4	0	8	0	0	0	0	0
Solyc05g051560.2.1	0	0	0	0	0	0	0	0	0	0	0	0	0
Solyc05g051570.2.1	6	31	0	16	3	53	5	54	3	130	24	126	0
Solyc05g051580.2.1	0	0	0	0	0	0	0	0	0	0	0	0	0
Solyc05g051590.2.1	4	11	4	9	4	21	59	30	8	24	9	26	2
Solyc05g051620.2.1	7	50	5	23	0	88	39	63	1	23	6	24	0
Solyc05g051630.2.1	3	4	0	3	0	28	7	6	1	5	5	8	0
Solyc05g051640.2.1	0	0	0	0	0	0	0	0	0	0	0	0	0
Solyc05g051660.1.1	11	316	45	145	138	687	481	367	681	119	133	132	30
Solyc05g051670.1.1	0	0	0	0	0	0	0	0	0	0	0	0	0
Solyc05g051680.2.1	1	3	1	2	0	2	6	0	0	17	2	2	0
Solyc05g051690.2.1	6	33	5	15	5	51	49	68	5	61	29	68	2
Solyc05g051700.2.1	5	148	6	56	0	103	34	118	1	85	12	53	0
Solyc05g051710.2.1	2	23	6	17	2	24	5	12	3	0	0	0	0
Solyc05g051720.1.1	0	0	0	0	0	0	0	0	0	0	0	0	0
Solyc05g051740.2.1	0	0	0	0	0	0	0	0	0	0	0	0	0

Solyc05g051750.2.1	10	304	35	203	0	129	386	172	0	229	28	261	0
Solyc05g051760.2.1	0	0	0	0	0	0	0	0	0	0	0	0	0
Solyc05g051770.2.1	5	29	1	11	0	47	48	51	1	35	7	20	0
Solyc05g051780.2.1	0	0	0	0	0	0	0	0	0	0	0	0	0
Solyc05g051790.2.1	0	0	0	0	0	0	0	0	0	0	0	0	0
Solyc05g051800.2.1	0	0	0	0	0	0	0	0	0	0	0	0	0
Solyc05g051820.2.1	0	0	0	0	0	0	0	0	0	0	0	0	0
Solyc05g051840.1.1	2	12	1	6	2	15	6	18	5	12	2	26	0
Solyc05g051850.2.1	9	1510	54	658	5	819	49	722	3	874	71	330	0
Solyc05g051860.2.1	1	3	1	0	0	3	7	4	0	0	0	0	0
Solyc05g051870.2.1	2	23	0	16	0	4	7	7	0	1	0	0	0
Solyc05g051880.2.1	0	0	0	0	0	0	0	0	0	0	0	0	0
Solyc05g051900.2.1	2	1	1	4	0	10	22	7	0	6	1	2	0
Solyc05g051910.1.1	0	0	0	0	0	0	0	0	0	0	0	0	0
Solyc05g051920.2.1	0	0	0	0	0	0	0	0	0	0	0	0	0
Solyc05g051970.2.1	0	0	0	0	0	0	0	0	0	0	0	0	0
Solyc05g051990.2.1	0	0	0	0	0	0	0	0	0	0	0	0	0
Solyc05g052000.2.1	0	0	0	0	0	0	0	0	0	0	0	0	0
Solyc05g052010.2.1	0	0	0	0	0	0	0	0	0	0	0	0	0
Solyc05g052020.2.1	10	25	8	23	7	139	171	65	14	143	27	90	8
Solyc05g052030.1.1	5	166	16	96	1	105	16	100	4	43	3	23	1
Solyc05g052040.1.1	5	607	66	505	38	34	9	82	2	79	5	245	4
Solyc05g052050.1.1	2	40	7	30	4	2	1	5	1	4	2	4	0
Solyc05g052070.2.1	2	8	0	5	0	18	10	27	2	52	17	42	0
Solyc05g052100.2.1	0	0	0	0	0	0	0	0	0	0	0	0	0
Solyc05g052130.2.1	0	0	0	0	0	0	0	0	0	0	0	0	0
Solyc05g052140.2.1	1	15	4	2	0	26	21	32	4	14	13	21	0
Solyc05g052150.2.1	1	9	1	0	0	7	11	25	0	21	1	17	0
Solyc05g052160.2.1	0	0	0	0	0	0	0	0	0	0	0	0	0
Solyc05g052170.2.1	2	28	2	4	0	17	9	34	2	22	1	12	0
Solyc05g052180.2.1	0	0	0	0	0	0	0	0	0	0	0	0	0
Solyc05g052190.2.1	4	9	2	4	0	15	18	11	5	37	7	15	0
Solyc05g052200.2.1	7	63	3	22	0	53	31	130	17	65	22	88	0
Solyc05g052210.2.1	1	2	0	0	0	5	1	2	0	2	4	8	0
Solyc05g052220.2.1	6	18	1	16	0	25	62	49	13	21	20	37	3
Solyc05g052240.2.1	7	453	8	180	52	164	80	66	21	385	30	293	7
Solyc05g052260.2.1	3	38	2	35	0	328	59	130	2	89	25	116	4
Solyc05g052270.1.1	7	57	6	24	6	174	113	220	34	40	36	69	2
Solyc05g052280.2.1	7	16	3	8	5	114	284	61	291	0	3	3	3
Solyc05g052290.1.1	0	0	0	0	0	0	0	0	0	0	0	0	0
Solyc05g052300.2.1	9	64	29	13	0	78	292	66	15	3	1	2	1

Solyc05g052310.2.1	3	81	2	32	2	72	69	127	9	112	13	111	0
Solyc05g052320.2.1	0	0	0	0	0	0	0	0	0	0	0	0	0
Solyc05g052340.2.1	1	1	0	1	0	6	7	4	0	0	0	0	0
Solyc05g052350.2.1	0	0	0	0	0	0	0	0	0	0	0	0	0
Solyc05g052360.1.1	0	0	0	0	0	0	0	0	0	0	0	0	0
Solyc05g052370.2.1	1	0	0	0	0	8	3	9	4	0	0	5	0
Solyc05g052400.2.1	1	4	0	0	0	1	8	2	3	0	0	0	0
Solyc05g052410.1.1	2	0	0	0	0	12	4	6	3	0	3	1	0
Solyc05g052450.2.1	0	0	0	0	0	0	0	0	0	0	0	0	0
Solyc05g052460.2.1	1	1	1	0	8	0	1	6	0	5	2	0	0
Solyc05g052470.2.1	4	93	6	88	3	365	108	457	8	279	47	259	3
Solyc05g052480.2.1	2	14	1	10	0	61	15	30	2	17	0	14	0
Solyc05g052490.2.1	6	21	3	13	1	25	24	39	6	16	13	24	0
Solyc05g052500.2.1	5	38	2	23	2	11	25	52	4	71	6	45	0
Solyc05g052510.2.1	14	209	23	117	10	684	257	662	49	753	74	510	2
Solyc05g052520.2.1	4	33	17	39	9	25	8	8	1	15	4	65	0
Solyc05g052550.1.1	6	571	46	410	27	59	15	45	2	95	5	244	9
Solyc05g052560.1.1	1	149	20	73	4	10	7	10	2	11	0	23	1
Solyc05g052570.2.1	15	2103	203	1820	146	232	84	208	21	311	24	698	17
Solyc05g052580.2.1	7	125	3	23	2	61	31	115	13	78	15	146	2
Solyc05g052590.1.1	0	0	0	0	0	0	0	0	0	0	0	0	0
Solyc05g052600.2.1	7	782	37	525	0	62	61	92	2	212	29	263	10
Solyc05g052610.2.1	10	26	2	15	0	21	51	48	5	67	15	46	0
Solyc05g052620.2.1	4	46	2	28	0	44	21	60	8	64	6	60	1
Solyc05g052630.2.1	24	677	47	632	17	552	483	845	69	1199	283	978	19
Solyc05g052640.2.1	2	7	0	4	0	28	29	21	3	11	4	17	0
Solyc05g052650.2.1	0	0	0	0	0	0	0	0	0	0	0	0	0
Solyc05g052660.1.1	0	0	0	0	0	0	0	0	0	0	0	0	0
Solyc05g052670.1.1	3	5	2	7	0	410	8	13	0	0	0	0	0
Solyc05g052680.1.1	9	3	15	17	18	146	31	280	14	0	19	2	4
Solyc05g052690.2.1	2	17	1	8	0	22	5	28	0	31	5	25	2
Solyc05g052700.2.1	4	17	1	8	1	146	63	56	12	1	12	5	0
Solyc05g052710.2.1	8	311	6	105	0	75	86	122	0	69	9	40	1
Solyc05g052720.1.1	1	7	0	0	0	0	9	0	0	17	0	6	0
Solyc05g052750.2.1	0	0	0	0	0	0	0	0	0	0	0	0	0
Solyc05g052760.2.1	14	311	34	270	19	1021	1056	1130	98	960	176	757	25
Solyc05g052780.2.1	5	126	3	61	3	192	69	161	9	216	25	109	3
Solyc05g052790.2.1	4	146	6	53	0	254	22	107	5	237	18	105	3
Solyc05g052800.2.1	4	187	9	78	5	133	48	170	17	474	37	498	16
Solyc05g052810.2.1	6	88	2	18	0	202	26	135	13	165	37	158	2
Solyc05g052820.2.1	1	4	0	1	0	1	1	3	2	11	1	2	0

Solyc05g052830.2.1	0	0	0	0	0	0	0	0	0	0	0	0	0
Solyc05g052840.1.1	0	0	0	0	0	0	0	0	0	0	0	0	0
Solyc05g052850.2.1	1	0	0	3	0	0	0	1	1	7	3	1	0
Solyc05g052860.2.1	2	4	0	3	0	1	0	3	0	0	8	0	0
Solyc05g052870.2.1	0	0	0	0	0	0	0	0	0	0	0	0	0
Solyc05g052880.2.1	0	0	0	0	0	0	0	0	0	0	0	0	0
Solyc05g052910.2.1	0	0	0	0	0	0	0	0	0	0	0	0	0
Solyc05g052920.2.1	4	28	4	38	0	25	93	42	5	39	23	43	1
Solyc05g052930.2.1	2	3	0	0	0	8	17	6	11	18	3	2	0
Solyc05g052940.2.1	1	1	0	0	0	0	4	10	1	7	2	1	0
Solyc05g052950.2.1	0	0	0	0	0	0	0	0	0	0	0	0	0
Solyc05g052960.2.1	6	46	6	29	6	136	55	109	13	227	17	136	1
Solyc05g052970.2.1	8	37	1	34	1	68	37	98	6	132	25	100	1
Solyc05g052980.2.1	17	475	32	228	13	1640	637	943	116	263	100	309	9
Solyc05g052990.2.1	6	26	0	21	2	29	33	34	1	23	0	19	1
Solyc05g053000.1.1	3	7	0	12	0	21	30	11	0	2	2	0	0
Solyc05g053010.1.1	2	27	1	11	0	1	6	4	0	4	6	1	0
Solyc05g053020.2.1	0	0	0	0	0	0	0	0	0	0	0	0	0
Solyc05g053040.2.1	3	11	2	8	2	19	5	11	0	66	2	52	0
Solyc05g053060.1.1	1	46	1	12	0	0	0	1	0	2	7	0	0
Solyc05g053070.2.1	2	264	32	68	15	2059	1656	1095	397	79	67	141	17
Solyc05g053080.2.1	0	0	0	0	0	0	0	0	0	0	0	0	0
Solyc05g053090.1.1	1	1	0	3	0	5	4	15	3	6	0	6	0
Solyc05g053100.2.1	14	80	42	55	6	14	255	56	6	161	23	90	7
Solyc05g053130.2.1	11	31	16	27	3	109	99	158	8	66	29	64	0
Solyc05g053140.2.1	15	209	8	78	15	575	104	455	45	331	37	169	1
Solyc05g053150.1.1	6	0	0	0	0	552	73	84	4	0	0	0	0
Solyc05g053160.2.1	1	0	0	0	0	27	20	8	1	0	21	1	0
Solyc05g053180.2.1	0	0	0	0	0	0	0	0	0	0	0	0	0
Solyc05g053190.2.1	4	8	3	0	0	14	14	12	0	5	1	17	1
Solyc05g053200.1.1	0	0	0	0	0	0	0	0	0	0	0	0	0
Solyc05g053210.2.1	9	567	61	273	5	1490	399	1550	51	728	94	260	1
Solyc05g053220.2.1	2	16	7	8	0	67	66	69	7	25	20	14	0
Solyc05g053230.2.1	9	155	6	112	1	124	55	205	13	130	16	84	0
Solyc05g053240.2.1	11	161	20	141	18	136	104	177	13	302	57	208	0
Solyc05g053250.2.1	4	63	5	53	2	237	14	259	3	157	6	166	1
Solyc05g053260.2.1	6	10	2	10	0	10	34	15	1	26	12	39	0
Solyc05g053270.1.1	2	2	0	0	0	16	5	2	0	2	4	5	0
Solyc05g053280.2.1	6	27	4	18	0	24	45	59	2	25	2	38	1
Solyc05g053290.2.1	5	30	9	14	2	121	6	43	4	25	5	37	0
Solyc05g053300.2.1	15	633	70	277	38	775	132	480	32	438	146	289	10

Solyc05g053310.2.1	3	9	8	8	0	11	17	19	0	27	9	20	1
Solyc05g053320.1.1	1	3	0	0	2	9	3	2	0	8	1	3	0
Solyc05g053330.2.1	0	0	0	0	0	0	0	0	0	0	0	0	0
Solyc05g053340.2.1	0	0	0	0	0	0	0	0	0	0	0	0	0
Solyc05g053350.2.1	0	0	0	0	0	0	0	0	0	0	0	0	0
Solyc05g053360.2.1	3	47	5	21	2	12	55	27	3	75	6	36	2
Solyc05g053370.2.1	0	0	0	0	0	0	0	0	0	0	0	0	0
Solyc05g053390.2.1	4	21	4	17	0	13	34	50	6	77	34	58	2
Solyc05g053400.1.1	0	0	0	0	0	0	0	0	0	0	0	0	0
Solyc05g053410.2.1	1	3	0	7	1	1	1	2	0	5	4	10	0
Solyc05g053430.2.1	0	0	0	0	0	0	0	0	0	0	0	0	0
Solyc05g053440.2.1	2	135	1	29	2	220	24	246	8	245	20	139	4
Solyc05g053470.2.1	9	56	7	34	15	182	62	208	16	198	86	153	4
Solyc05g053480.2.1	0	0	0	0	0	0	0	0	0	0	0	0	0
Solyc05g053490.2.1	3	8	3	1	8	9	8	16	5	15	4	4	0
Solyc05g053500.2.1	1	35	4	35	1	17	8	11	6	9	4	28	0
Solyc05g053530.1.1	0	0	0	0	0	0	0	0	0	0	0	0	0
Solyc05g053540.2.1	6	74	10	44	36	86	112	81	22	685	35	698	6
Solyc05g053550.2.1	14	3183	27	2278	203	86	133	192	138	6326	88	4359	1
Solyc05g053570.2.1	0	0	0	0	0	0	0	0	0	0	0	0	0
Solyc05g053590.2.1	1	13	0	19	0	31	5	84	0	143	3	95	0
Solyc05g053600.2.1	0	0	0	0	0	0	0	0	0	0	0	0	0
Solyc05g053610.2.1	0	0	0	0	0	0	0	0	0	0	0	0	0
Solyc05g053620.2.1	5	7	0	6	0	35	0	20	0	27	34	20	2
Solyc05g053640.1.1	0	0	0	0	0	0	0	0	0	0	0	0	0
Solyc05g053650.2.1	24	443	24	238	19	1414	493	1240	123	1369	182	687	9
Solyc05g053660.1.1	0	0	0	0	0	0	0	0	0	0	0	0	0
Solyc05g053670.2.1	1	88	2	18	5	36	39	69	15	113	20	99	4
Solyc05g053690.1.1	0	0	0	0	0	0	0	0	0	0	0	0	0
Solyc05g053710.2.1	7	22	10	25	2	93	85	82	17	51	23	70	0
Solyc05g053720.2.1	4	24	0	11	1	51	18	82	3	31	12	34	1
Solyc05g053730.2.1	2	11	0	3	0	15	13	37	6	19	8	5	0
Solyc05g053740.2.1	1	4	2	0	0	11	2	12	0	3	0	2	0
Solyc05g053760.2.1	8	121	50	146	14	443	233	336	42	42	21	34	4
Solyc05g053770.2.1	6	25	0	4	0	78	28	56	3	39	11	53	0
Solyc05g053780.2.1	5	286	10	137	11	797	133	942	58	976	56	894	5
Solyc05g053790.1.1	0	0	0	0	0	0	0	0	0	0	0	0	0
Solyc05g053800.2.1	8	14	2	23	0	23	38	61	5	55	17	63	1
Solyc05g053810.2.1	13	1619	82	854	57	1034	559	1408	156	6286	335	2450	32
Solyc05g053820.2.1	14	54	3	45	1	115	69	182	15	146	21	147	7
Solyc05g053830.2.1	8	104	4	74	8	276	84	266	20	281	34	257	0

Solyc05g053850.2.1	0	0	0	0	0	0	0	0	0	0	0	0	0
Solyc05g053860.2.1	0	0	0	0	0	0	0	0	0	0	0	0	0
Solyc05g053870.2.1	2	16	3	8	0	1	2	9	0	5	2	8	0
Solyc05g053880.2.1	3	5	1	6	0	23	3	15	2	3	4	8	0
Solyc05g053890.1.1	1	8	0	3	0	1	2	6	0	2	5	0	0
Solyc05g053900.2.1	2	4	0	6	0	16	2	7	0	3	6	0	0
Solyc05g053930.2.1	5	7	0	6	2	13	13	29	1	19	14	16	1
Solyc05g053940.2.1	1	39	5	15	1	65	66	83	15	161	36	127	0
Solyc05g053950.2.1	1	43	4	4	1	40	53	59	3	78	8	9	0
Solyc05g053960.2.1	0	0	0	0	0	0	0	0	0	0	0	0	0
Solyc05g053970.2.1	0	0	0	0	0	0	0	0	0	0	0	0	0
Solyc05g053980.1.1	0	0	0	0	0	0	0	0	0	0	0	0	0
Solyc05g053990.2.1	1	0	0	0	0	0	5	3	0	9	1	3	0
Solyc05g054000.2.1	0	0	0	0	0	0	0	0	0	0	0	0	0
Solyc05g054010.2.1	0	0	0	0	0	0	0	0	0	0	0	0	0
Solyc05g054020.1.1	0	0	0	0	0	0	0	0	0	0	0	0	0
Solyc05g054030.2.1	2	15	3	8	0	8	5	39	2	1	0	0	0
Solyc05g054040.2.1	0	0	0	0	0	0	0	0	0	0	0	0	0
Solyc05g054050.2.1	0	0	0	0	0	0	0	0	0	0	0	0	0
Solyc05g054060.2.1	0	0	0	0	0	0	0	0	0	0	0	0	0
Solyc05g054070.2.1	3	54	1	11	6	76	15	84	10	124	34	109	1
Solyc05g054080.2.1	4	20	3	18	4	24	42	67	9	28	24	11	0
Solyc05g054090.2.1	8	77	20	17	103	152	51	79	9	6888	70	1589	43
Solyc05g054100.2.1	3	42	2	27	1	119	24	79	16	51	40	59	0
Solyc05g054110.2.1	0	0	0	0	0	0	0	0	0	0	0	0	0
Solyc05g054120.1.1	1	25	1	10	4	3	4	6	0	3	1	3	0
Solyc05g054130.2.1	5	155	6	114	7	169	101	206	12	94	10	85	0
Solyc05g054140.2.1	5	11	1	9	4	142	20	72	7	51	14	35	0
Solyc05g054150.2.1	3	13	2	9	2	33	28	44	6	4	4	5	0
Solyc05g054170.2.1	2	6	5	10	0	9	4	8	0	9	0	10	0
Solyc05g054180.2.1	8	57	7	32	5	155	51	116	12	108	20	49	1
Solyc05g054190.2.1	3	41	0	13	0	1	24	28	0	4	2	9	0
Solyc05g054200.2.1	2	6	3	3	0	10	9	8	0	23	0	20	0
Solyc05g054210.2.1	18	372	17	208	15	36	47	116	4	861	123	668	14
Solyc05g054220.2.1	3	26	2	15	4	21	5	22	0	64	7	90	0
Solyc05g054230.2.1	1	1	0	4	0	3	5	3	3	0	0	0	0
Solyc05g054240.2.1	12	58	8	50	0	50	41	97	5	217	48	155	0
Solyc05g054250.1.1	0	0	0	0	0	0	0	0	0	0	0	0	0
Solyc05g054260.2.1	6	54	2	35	9	52	38	63	1	89	5	75	1
Solyc05g054310.2.1	5	16	2	16	9	50	35	46	7	25	13	46	1
Solyc05g054320.2.1	3	103	8	56	0	9	3	14	0	58	39	51	1

Solyc05g054330.2.1	1	48	0	13	2	26	0	7	1	27	6	5	0
Solyc05g054340.2.1	0	0	0	0	0	0	0	0	0	0	0	0	0
Solyc05g054350.2.1	1	27	0	24	0	16	0	20	0	172	4	108	0
Solyc05g054360.2.1	0	0	0	0	0	0	0	0	0	0	0	0	0
Solyc05g054370.2.1	17	121	10	76	9	832	377	1088	42	497	55	404	7
Solyc05g054380.1.1	0	0	0	0	0	0	0	0	0	0	0	0	0
Solyc05g054390.2.1	14	151	24	94	1	206	272	199	27	127	57	97	10
Solyc05g054400.2.1	1	14	0	5	0	13	0	29	0	25	0	27	0
Solyc05g054410.2.1	18	78	8	88	4	201	164	314	27	159	24	143	1
Solyc05g054420.2.1	3	7	0	1	0	9	41	30	10	5	19	7	0
Solyc05g054430.2.1	0	0	0	0	0	0	0	0	0	0	0	0	0
Solyc05g054440.2.1	0	0	0	0	0	0	0	0	0	0	0	0	0
Solyc05g054450.1.1	1	5	0	0	0	1	2	3	0	4	4	5	0
Solyc05g054460.2.1	0	0	0	0	0	0	0	0	0	0	0	0	0
Solyc05g054480.2.1	1	8	0	0	0	5	11	9	0	14	1	3	0
Solyc05g054490.2.1	11	256	24	96	40	280	416	210	356	278	78	172	1
Solyc05g054500.1.1	10	103	6	43	8	120	95	159	23	186	39	118	2
Solyc05g054510.1.1	0	0	0	0	0	0	0	0	0	0	0	0	0
Solyc05g054520.2.1	4	14	1	13	0	30	15	23	5	23	9	16	0
Solyc05g054530.1.1	1	7	0	4	0	2	1	9	0	0	2	0	0
Solyc05g054540.2.1	1	7	0	1	0	2	7	18	3	31	6	40	1
Solyc05g054550.2.1	2	11	4	7	0	10	15	13	2	38	1	0	0
Solyc05g054560.2.1	5	12	0	7	0	48	60	59	8	85	25	77	0
Solyc05g054570.2.1	11	70	8	74	7	378	343	514	39	149	91	122	0
Solyc05g054580.2.1	6	440	27	189	15	951	249	1092	81	1114	262	1052	17
Solyc05g054590.2.1	10	96	6	28	5	241	158	237	22	31	266	1	2
Solyc05g054600.2.1	3	15	1	15	4	18	40	22	0	31	5	28	0
Solyc05g054610.1.1	1	7	0	2	0	0	4	0	0	1	0	3	0
Solyc05g054620.2.1	7	79	4	54	3	26	62	51	11	649	35	464	4
Solyc05g054630.2.1	10	10	6	33	0	54	44	63	7	49	13	45	0
Solyc05g054640.2.1	12	113	14	72	15	261	355	564	69	352	114	268	1
Solyc05g054650.1.1	0	0	0	0	0	0	0	0	0	0	0	0	0
Solyc05g054660.1.1	0	0	0	0	0	0	0	0	0	0	0	0	0
Solyc05g054670.2.1	0	0	0	0	0	0	0	0	0	0	0	0	0
Solyc05g054680.1.1	2	28	1	5	2	9	16	4	0	7	3	5	0
Solyc05g054700.2.1	10	57	4	43	4	70	61	162	7	75	15	48	0
Solyc05g054710.2.1	8	48	5	18	1	54	90	138	5	67	2	42	1
Solyc05g054720.2.1	7	184	23	37	1	149	155	209	10	240	32	156	1
Solyc05g054730.2.1	7	64	3	48	2	78	36	65	4	194	15	166	0
Solyc05g054740.2.1	8	109	19	35	20	21	64	41	12	86	24	55	1
Solyc05g054750.2.1	0	0	0	0	0	0	0	0	0	0	0	0	0

Solyc05g054760.2.1	6	857	88	412	16	626	453	638	30	754	359	617	14
Solyc05g054770.2.1	0	0	0	0	0	0	0	0	0	0	0	0	0
Solyc05g054790.1.1	0	0	0	0	0	0	0	0	0	0	0	0	0
Solyc05g054800.1.1	15	332	67	253	41	383	180	431	79	342	95	284	17
Solyc05g054810.2.1	2	9	4	24	0	18	12	35	3	73	22	63	0
Solyc05g054820.1.1	1	6	0	5	0	1	4	5	0	0	0	8	0
Solyc05g054830.2.1	10	334	53	279	13	557	253	604	27	320	43	207	8
Solyc05g054840.2.1	1	2	0	4	0	12	6	5	0	3	0	5	0
Solyc05g054850.1.1	0	0	0	0	0	0	0	0	0	0	0	0	0
Solyc05g054860.1.1	13	130	4	100	3	203	74	86	4	87	24	91	3
Solyc05g054870.2.1	0	0	0	0	0	0	0	0	0	0	0	0	0
Solyc05g054880.2.1	0	0	0	0	0	0	0	0	0	0	0	0	0
Solyc05g054890.2.1	3	0	0	8	0	21	42	12	16	0	0	0	0
Solyc05g054900.2.1	0	0	0	0	0	0	0	0	0	0	0	0	0
Solyc05g054910.2.1	16	74	13	75	6	173	64	163	18	191	36	170	3
Solyc05g054920.2.1	8	271	12	162	14	254	116	292	25	1458	89	959	6
Solyc05g054930.2.1	0	0	0	0	0	0	0	0	0	0	0	0	0
Solyc05g054940.2.1	4	15	0	26	0	43	32	64	2	40	2	16	0
Solyc05g054950.2.1	5	9	1	5	0	31	17	41	1	8	5	28	3
Solyc05g054960.2.1	8	75	2	35	0	191	39	169	6	172	48	137	0
Solyc05g054970.2.1	13	97	11	130	10	195	82	250	10	180	34	134	0
Solyc05g054980.2.1	5	33	1	27	2	83	34	43	5	37	4	30	0
Solyc05g054990.2.1	1	3	0	1	0	1	0	12	5	8	4	6	0
Solyc05g055000.2.1	3	27	0	23	2	23	14	28	0	12	1	19	0
Solyc05g055010.2.1	2	5	2	4	0	17	2	9	0	17	14	16	1
Solyc05g055030.1.1	0	0	0	0	0	0	0	0	0	0	0	0	0
Solyc05g055040.2.1	7	35	0	11	0	35	34	86	6	20	5	24	7
Solyc05g055060.2.1	4	71	5	52	6	84	57	104	7	134	32	80	0
Solyc05g055070.2.1	7	27	5	19	2	64	55	43	6	38	14	31	0
Solyc05g055090.2.1	11	43	9	46	2	96	104	187	15	106	12	65	0
Solyc05g055100.1.1	0	0	0	0	0	0	0	0	0	0	0	0	0
Solyc05g055110.2.1	4	10	0	6	0	14	15	42	4	36	10	15	2
Solyc05g055120.2.1	3	18	0	9	0	18	7	17	3	50	3	36	1
Solyc05g055130.1.1	0	0	0	0	0	0	0	0	0	0	0	0	0
Solyc05g055160.2.1	8	1277	292	785	101	1233	1599	1342	137	4646	615	3116	83
Solyc05g055170.2.1	1	3	3	4	0	37	9	25	8	34	5	26	1
Solyc05g055200.2.1	0	0	0	0	0	0	0	0	0	0	0	0	0
Solyc05g055210.1.1	4	54	7	15	0	51	65	87	4	98	35	70	5
Solyc05g055220.1.1	0	0	0	0	0	0	0	0	0	0	0	0	0
Solyc05g055230.1.1	0	0	0	0	0	0	0	0	0	0	0	0	0
Solyc05g055240.2.1	3	17	0	17	1	43	6	27	0	38	10	19	0

Solyc05g055250.2.1	3	5	0	2	6	10	10	10	1	21	0	10	0
Solyc05g055260.2.1	2	10	2	4	0	13	4	42	2	28	8	20	1
Solyc05g055280.1.1	1	10	2	1	0	12	4	6	0	6	0	15	0
Solyc05g055300.1.1	5	44	2	14	0	32	8	54	7	31	17	42	0
Solyc05g055310.2.1	2	89	8	66	6	148	84	97	6	119	33	115	0
Solyc05g055330.2.1	3	27	0	0	2	213	18	4	13	11	4	1	1
Solyc05g055340.2.1	6	20	9	8	3	21	42	67	10	96	24	21	1
Solyc05g055350.2.1	5	46	6	28	2	102	75	88	2	48	7	48	1
Solyc05g055360.2.1	23	60	14	53	26	236	282	308	48	261	72	142	0
Solyc05g055380.1.1	0	0	0	0	0	0	0	0	0	0	0	0	0
Solyc05g055390.2.1	2	13	0	7	0	19	2	13	0	38	1	29	0
Solyc05g055400.2.1	7	71	9	1	18	115	69	42	156	737	35	115	28
Solyc05g055410.1.1	0	0	0	0	0	0	0	0	0	0	0	0	0
Solyc05g055420.2.1	10	62	6	26	9	55	34	84	9	59	40	64	1
Solyc05g055430.2.1	8	317	32	314	1	313	116	269	12	189	21	130	3
Solyc05g055440.1.1	3	154	5	46	2	93	91	117	24	158	49	95	2
Solyc05g055450.2.1	7	202	11	86	5	268	69	321	20	254	49	157	1
Solyc05g055460.1.1	1	39	2	30	0	12	8	5	0	0	0	0	0
Solyc05g055470.2.1	13	83	13	49	10	350	113	276	45	64	38	64	4
Solyc05g055480.2.1	8	60	7	25	1	84	53	74	2	49	14	44	13
Solyc05g055510.2.1	0	0	0	0	0	0	0	0	0	0	0	0	0
Solyc05g055520.1.1	0	0	0	0	0	0	0	0	0	0	0	0	0
Solyc05g055530.2.1	0	0	0	0	0	0	0	0	0	0	0	0	0
Solyc05g055540.1.1	0	0	0	0	0	0	0	0	0	0	0	0	0
Solyc05g055550.2.1	0	0	0	0	0	0	0	0	0	0	0	0	0
Solyc05g055600.2.1	8	73	11	115	2	397	232	301	29	206	48	190	1
Solyc05g055610.2.1	8	29	3	24	4	98	47	111	7	85	8	61	2
Solyc05g055630.2.1	1	9	0	3	0	0	2	3	1	0	2	5	0
Solyc05g055640.2.1	2	184	17	49	17	295	133	224	42	536	154	408	34
Solyc05g055680.2.1	0	0	0	0	0	0	0	0	0	0	0	0	0
Solyc05g055690.1.1	0	0	0	0	0	0	0	0	0	0	0	0	0
Solyc05g055700.2.1	4	15	3	11	9	26	33	21	12	17	14	6	2
Solyc05g055710.2.1	0	0	0	0	0	0	0	0	0	0	0	0	0
Solyc05g055730.2.1	0	0	0	0	0	0	0	0	0	0	0	0	0
Solyc05g055740.2.1	0	0	0	0	0	0	0	0	0	0	0	0	0
Solyc05g055750.2.1	0	0	0	0	0	0	0	0	0	0	0	0	0
Solyc05g055760.2.1	2	49	12	18	0	41	40	52	3	98	44	141	0
Solyc05g055770.2.1	4	391	53	269	22	845	626	1210	121	1266	173	617	19
Solyc05g055780.2.1	0	0	0	0	0	0	0	0	0	0	0	0	0
Solyc05g055790.2.1	6	102	7	71	7	74	27	80	2	44	9	17	0
Solyc05g055800.2.1	1	43	0	7	0	102	18	66	1	60	3	26	1

Solyc05g055810.2.1	3	40	2	17	0	85	33	66	7	4	1	0	0
Solyc05g055820.2.1	3	44	2	13	0	27	62	23	0	0	1	0	0
Solyc05g055830.2.1	4	3	0	2	0	20	10	24	3	6	6	4	0
Solyc05g055840.2.1	5	15	9	13	0	0	15	12	1	38	2	45	2
Solyc05g055850.2.1	4	191	36	62	4	41	61	73	2	53	16	117	5
Solyc05g055870.2.1	5	9	0	12	0	64	16	95	28	54	24	59	0
Solyc05g055880.2.1	0	0	0	0	0	0	0	0	0	0	0	0	0
Solyc05g055890.2.1	1	2	1	2	0	11	4	6	2	22	0	7	0
Solyc05g055910.2.1	14	48	5	54	0	278	308	305	12	139	52	90	0
Solyc05g055940.2.1	10	313	17	160	16	246	381	442	31	78	47	57	2
Solyc05g055950.1.1	0	0	0	0	0	0	0	0	0	0	0	0	0
Solyc05g055960.1.1	0	0	0	0	0	0	0	0	0	0	0	0	0
Solyc05g055970.2.1	0	0	0	0	0	0	0	0	0	0	0	0	0
Solyc05g055980.2.1	0	0	0	0	0	0	0	0	0	0	0	0	0
Solyc05g055990.2.1	5	45	17	25	3	1	51	3	2	3	1	1	2
Solyc05g056000.2.1	14	117	25	38	14	181	234	171	141	347	52	258	21
Solyc05g056010.2.1	7	42	5	44	3	32	48	92	5	58	11	41	0
Solyc05g056020.2.1	1	4	0	0	2	0	6	0	10	14	11	0	14
Solyc05g056030.2.1	11	31	7	22	2	24	41	55	12	195	33	69	2
Solyc05g056040.2.1	5	26	1	20	0	46	19	63	3	23	1	39	0
Solyc05g056050.2.1	1	82	8	32	0	0	0	1	0	0	0	0	0
Solyc05g056060.2.1	1	11	0	6	0	10	4	10	1	30	2	34	0
Solyc05g056070.2.1	2	871	130	662	2	21	69	22	3	37	68	87	3
Solyc05g056080.2.1	1	2	0	0	0	2	4	0	4	0	0	5	0
Solyc05g056090.2.1	4	16	1	5	0	24	26	52	4	46	23	38	0
Solyc05g056100.2.1	4	33	6	25	3	260	24	132	10	80	12	72	3
Solyc05g056110.2.1	3	20	3	10	4	39	72	54	9	36	8	24	0
Solyc05g056120.2.1	9	318	26	147	33	22	27	48	30	157	113	138	2
Solyc05g056130.2.1	7	110	12	74	3	132	156	417	20	322	42	190	2
Solyc05g056140.2.1	2	17	2	7	3	7	1	12	0	2	4	0	0
Solyc05g056150.2.1	5	14	2	8	0	26	24	29	1	26	2	19	0
Solyc05g056160.2.1	4	125	11	71	4	273	86	278	35	211	25	157	0
Solyc05g056170.2.1	14	101	5	82	4	160	84	310	84	50	24	0	0
Solyc05g056200.1.1	2	11	0	10	0	85	9	23	2	1	1	2	0
Solyc05g056210.2.1	28	107	14	99	10	327	247	403	33	245	66	211	7
Solyc05g056220.2.1	13	75	9	71	19	111	61	174	10	233	35	158	4
Solyc05g056230.2.1	6	149	17	108	4	245	106	273	13	284	56	334	1
Solyc05g056240.2.1	1	15	1	7	1	20	21	15	3	79	7	44	0
Solyc05g056250.2.1	6	87	4	50	9	192	77	281	16	255	57	132	8
Solyc05g056260.2.1	7	49	10	32	2	42	72	103	2	44	7	79	3
Solyc05g056270.2.1	5	23	5	1	0	36	27	58	1	46	9	68	0

Solyc05g056280.2.1	6	32	3	28	1	38	45	86	9	64	16	50	0
Solyc05g056290.2.1	12	127	17	122	10	480	190	383	13	276	30	219	2
Solyc05g056300.2.1	2	251	26	74	1	8	16	13	0	27	11	33	0
Solyc05g056310.2.1	15	121	26	42	21	200	85	373	25	401	126	378	7
Solyc05g056320.2.1	11	237	31	188	11	279	110	301	37	209	71	221	3
Solyc05g056330.2.1	1	4	1	1	0	0	5	0	0	6	3	3	0
Solyc05g056340.2.1	13	111	10	54	3	191	121	260	21	162	59	125	2
Solyc05g056350.2.1	14	159	17	140	9	276	201	391	35	228	77	201	11
Solyc05g056370.2.1	14	123	27	100	3	50	159	171	13	128	21	103	0
Solyc05g056380.2.1	0	0	0	0	0	0	0	0	0	0	0	0	0
Solyc05g056390.2.1	2	28	0	7	0	16	11	17	5	46	9	47	0
Solyc05g056400.2.1	0	0	0	0	0	0	0	0	0	0	0	0	0
Solyc05g056420.2.1	0	0	0	0	0	0	0	0	0	0	0	0	0
Solyc05g056430.1.1	0	0	0	0	0	0	0	0	0	0	0	0	0
Solyc05g056440.1.1	0	0	0	0	0	0	0	0	0	0	0	0	0
Solyc05g056450.2.1	2	9	0	15	0	5	17	7	0	0	0	0	0
Solyc05g056460.2.1	7	42	6	26	0	115	28	126	11	96	21	34	0
Solyc05g056470.1.1	0	0	0	0	0	0	0	0	0	0	0	0	0
Solyc05g056480.2.1	8	19	1	34	0	171	120	291	0	44	2	41	0
Solyc05g056490.2.1	8	81	10	34	3	75	57	97	11	256	23	158	8
Solyc05g056500.1.1	0	0	0	0	0	0	0	0	0	0	0	0	0
Solyc05g056510.2.1	6	96	9	40	8	229	78	214	18	322	30	282	4
Solyc05g056520.2.1	1	0	0	4	0	3	3	4	0	5	1	2	0
Solyc05g056530.2.1	0	0	0	0	0	0	0	0	0	0	0	0	0
Solyc05g056540.2.1	11	54	5	79	2	76	61	122	2	155	14	140	1
Solyc05g056550.2.1	2	2	0	0	0	5	5	7	0	1	0	1	0
Solyc05g056560.2.1	1	0	0	0	0	4	6	8	0	1	3	4	6
Solyc05g056570.2.1	1	2	0	3	0	4	5	5	4	5	2	8	0
Solyc05g056580.2.1	15	295	32	250	21	302	274	520	65	553	145	535	2
Solyc05g056590.2.1	2	6	0	7	0	60	10	20	1	16	0	3	0
Solyc05g056620.1.1	2	0	0	0	0	0	0	0	0	19	19	13	0
Solyc06g005050.2.1	0	0	0	0	0	0	0	0	0	0	0	0	0
Solyc06g005060.2.1	10	931	196	611	251	2156	1204	3407	772	1948	1082	2281	614
Solyc06g005070.1.1	2	6	0	5	0	10	30	17	0	0	0	0	0
Solyc06g005080.2.1	11	75	11	52	7	205	108	246	11	217	42	244	2
Solyc06g005090.2.1	0	0	0	0	0	0	0	0	0	0	0	0	0
Solyc06g005110.2.1	5	11	1	12	0	7	12	19	2	28	0	22	9
Solyc06g005130.2.1	0	0	0	0	0	0	0	0	0	0	0	0	0
Solyc06g005140.2.1	1	2	0	0	0	3	0	2	0	2	3	6	0
Solyc06g005150.2.1	2	175	1	112	12	136	14	110	0	588	24	235	1
Solyc06g005160.2.1	4	1114	85	464	26	511	129	433	33	1251	278	747	31

Solyc06g005170.2.1	2	244	44	149	13	17	11	22	4	37	0	157	9
Solyc06g005180.1.1	0	0	0	0	0	0	0	0	0	0	0	0	0
Solyc06g005190.2.1	26	97	21	70	5	316	177	290	40	256	58	211	7
Solyc06g005200.2.1	4	9	1	2	2	22	17	13	3	16	17	18	0
Solyc06g005210.1.1	0	0	0	0	0	0	0	0	0	0	0	0	0
Solyc06g005220.2.1	0	0	0	0	0	0	0	0	0	0	0	0	0
Solyc06g005230.1.1	2	43	2	24	0	23	1	19	0	46	10	37	1
Solyc06g005250.2.1	1	24	0	9	0	160	9	60	4	13	8	13	0
Solyc06g005260.2.1	4	62	138	18	45	88	478	52	170	22	61	37	6
Solyc06g005290.2.1	3	14	1	17	2	18	24	25	10	9	0	9	1
Solyc06g005300.2.1	0	0	0	0	0	0	0	0	0	0	0	0	0
Solyc06g005310.2.1	0	0	0	0	0	0	0	0	0	0	0	0	0
Solyc06g005330.2.1	0	0	0	0	0	0	0	0	0	0	0	0	0
Solyc06g005340.2.1	0	0	0	0	0	0	0	0	0	0	0	0	0
Solyc06g005350.2.1	1	6	7	1	3	0	41	7	4	5	5	2	3
Solyc06g005360.2.1	3	261	20	137	3	354	161	280	25	426	59	381	10
Solyc06g005370.2.1	6	26	3	12	2	47	45	38	3	37	17	23	1
Solyc06g005380.2.1	1	0	0	0	0	9	4	5	0	0	0	0	0
Solyc06g005390.1.1	0	0	0	0	0	0	0	0	0	0	0	0	0
Solyc06g005410.2.1	0	0	0	0	0	0	0	0	0	0	0	0	0
Solyc06g005420.1.1	4	145	1	57	3	81	43	235	29	223	24	121	1
Solyc06g005430.1.1	2	15	0	9	0	11	5	31	1	16	0	17	0
Solyc06g005450.1.1	1	3	0	1	0	1	0	4	0	16	5	8	0
Solyc06g005470.2.1	0	0	0	0	0	0	0	0	0	0	0	0	0
Solyc06g005480.2.1	2	11	2	6	2	29	13	25	3	12	0	19	1
Solyc06g005490.2.1	5	22	0	11	0	58	36	37	4	95	18	44	0
Solyc06g005500.2.1	15	226	39	195	11	92	104	127	30	136	26	166	7
Solyc06g005520.2.1	0	0	0	0	0	0	0	0	0	0	0	0	0
Solyc06g005560.2.1	3	35	0	33	4	4	4	18	3	3	1	6	0
Solyc06g005570.2.1	11	77	8	36	5	107	70	148	15	101	25	71	0
Solyc06g005590.2.1	4	44	2	31	0	46	8	36	0	83	7	65	0
Solyc06g005600.2.1	6	19	10	31	2	42	50	34	10	59	12	51	1
Solyc06g005610.1.1	0	0	0	0	0	0	0	0	0	0	0	0	0
Solyc06g005620.2.1	0	0	0	0	0	0	0	0	0	0	0	0	0
Solyc06g005630.1.1	0	0	0	0	0	0	0	0	0	0	0	0	0
Solyc06g005650.2.1	2	40	10	53	0	8	9	8	7	26	3	28	2
Solyc06g005670.2.1	22	117	24	92	14	381	393	451	86	231	58	174	5
Solyc06g005680.2.1	3	7	0	6	0	16	24	29	6	41	2	31	0
Solyc06g005690.1.1	0	0	0	0	0	0	0	0	0	0	0	0	0
Solyc06g005700.1.1	0	0	0	0	0	0	0	0	0	0	0	0	0
Solyc06g005710.2.1	8	309	96	216	1	3	41	34	1	24	17	31	0

Solyc06g005720.2.1	0	0	0	0	0	0	0	0	0	0	0	0	0
Solyc06g005750.2.1	6	965	66	309	15	29	11	45	7	51	402	8	0
Solyc06g005770.1.1	0	0	0	0	0	0	0	0	0	0	0	0	0
Solyc06g005790.2.1	2	30	1	35	0	19	14	44	0	55	5	34	0
Solyc06g005800.2.1	5	46	2	20	0	57	53	79	8	39	9	36	2
Solyc06g005810.2.1	1	8	0	0	0	10	8	4	0	4	3	6	0
Solyc06g005820.2.1	2	14	1	7	3	0	2	4	0	0	3	0	0
Solyc06g005840.2.1	1	3	0	0	0	5	4	12	2	10	1	4	0
Solyc06g005850.2.1	0	0	0	0	0	0	0	0	0	0	0	0	0
Solyc06g005860.1.1	3	17	2	15	0	7	22	24	1	35	5	26	0
Solyc06g005890.2.1	2	1	0	4	0	1	0	3	2	9	6	6	0
Solyc06g005900.2.1	0	0	0	0	0	0	0	0	0	0	0	0	0
Solyc06g005910.2.1	0	0	0	0	0	0	0	0	0	0	0	0	0
Solyc06g005940.2.1	29	995	44	554	28	6095	1266	4878	256	1296	1073	1444	24
Solyc06g005950.2.1	17	154	25	151	5	88	201	229	25	119	43	94	4
Solyc06g005970.2.1	13	33	15	37	13	152	399	192	16	319	67	259	2
Solyc06g005980.2.1	0	0	0	0	0	0	0	0	0	0	0	0	0
Solyc06g005990.2.1	4	7	2	7	1	21	13	21	4	25	3	16	0
Solyc06g006000.2.1	5	181	2	50	2	32	100	206	62	1174	0	335	9
Solyc06g006010.1.1	0	0	0	0	0	0	0	0	0	0	0	0	0
Solyc06g006040.1.1	0	0	0	0	0	0	0	0	0	0	0	0	0
Solyc06g006060.1.1	0	0	0	0	0	0	0	0	0	0	0	0	0
Solyc06g006080.2.1	19	2179	54	1518	18	637	451	1207	6	3124	170	2264	22
Solyc06g006090.1.1	0	0	0	0	0	0	0	0	0	0	0	0	0
Solyc06g006100.2.1	8	80	2	51	0	98	50	348	22	370	20	344	8
Solyc06g006110.2.1	0	0	0	0	0	0	0	0	0	0	0	0	0
Solyc06g007110.2.1	16	134	15	94	1	133	76	183	14	143	11	104	3
Solyc06g007120.2.1	6	29	1	3	1	46	28	44	3	54	17	40	0
Solyc06g007130.2.1	1	6	0	0	0	0	3	2	0	3	9	12	0
Solyc06g007140.2.1	0	0	0	0	0	0	0	0	0	0	0	0	0
Solyc06g007150.2.1	2	6	0	0	0	10	10	17	0	0	4	10	0
Solyc06g007160.2.1	8	134	18	61	2	6	54	22	0	2	0	0	1
Solyc06g007170.2.1	0	0	0	0	0	0	0	0	0	0	0	0	0
Solyc06g007180.2.1	18	29	10	5	3	1194	2665	1875	14	15	77	2	0
Solyc06g007190.2.1	9	259	77	138	7	255	2	31	4	21	1	90	5
Solyc06g007200.2.1	6	24	3	9	0	65	39	91	7	79	19	82	0
Solyc06g007240.2.1	4	54	1	14	0	20	24	28	4	29	6	14	0
Solyc06g007250.2.1	3	50	2	26	2	149	64	66	1	96	20	53	0
Solyc06g007260.1.1	0	0	0	0	0	0	0	0	0	0	0	0	0
Solyc06g007280.2.1	0	0	0	0	0	0	0	0	0	0	0	0	0
Solyc06g007300.1.1	0	0	0	0	0	0	0	0	0	0	0	0	0

Solyc06g007310.2.1	3	15	3	16	0	12	20	16	0	12	4	45	0
Solyc06g007320.2.1	22	234	23	208	13	943	538	1133	152	568	208	577	15
Solyc06g007330.2.1	0	0	0	0	0	0	0	0	0	0	0	0	0
Solyc06g007340.2.1	5	116	3	41	14	117	47	123	15	144	42	121	1
Solyc06g007350.2.1	2	23	0	18	2	3	14	2	0	24	0	18	0
Solyc06g007360.2.1	2	21	0	9	0	3	12	28	1	40	13	32	2
Solyc06g007370.1.1	0	0	0	0	0	0	0	0	0	0	0	0	0
Solyc06g007430.1.1	10	41	5	49	0	183	104	239	16	167	28	65	2
Solyc06g007440.2.1	12	240	22	99	25	97	179	29	12	6	4	19	1
Solyc06g007460.2.1	0	0	0	0	0	0	0	0	0	0	0	0	0
Solyc06g007470.2.1	5	184	4	92	0	227	42	227	2	989	34	810	10
Solyc06g007480.2.1	1	5	0	7	0	17	4	5	3	3	0	1	0
Solyc06g007510.2.1	2	1374	278	1112	144	2159	1874	1627	211	2723	443	1476	110
Solyc06g007520.2.1	3	114	9	70	3	293	60	187	22	391	60	321	6
Solyc06g007530.2.1	1	1	1	0	0	0	3	0	0	9	0	11	0
Solyc06g007540.2.1	0	0	0	0	0	0	0	0	0	0	0	0	0
Solyc06g007570.2.1	3	71	3	28	7	146	61	171	22	101	37	136	0
Solyc06g007580.1.1	4	4	1	7	0	10	56	8	3	17	0	20	0
Solyc06g007590.2.1	7	50	5	27	0	68	49	78	3	67	22	37	0
Solyc06g007600.2.1	0	0	0	0	0	0	0	0	0	0	0	0	0
Solyc06g007620.2.1	3	34	2	16	1	3	16	5	7	16	9	71	0
Solyc06g007630.1.1	2	7	1	4	0	9	9	6	2	13	0	21	0
Solyc06g007640.2.1	22	341	33	238	22	668	272	671	60	422	119	363	20
Solyc06g007660.1.1	1	6	1	14	0	24	2	15	0	31	0	9	0
Solyc06g007670.2.1	3	277	18	86	19	523	302	659	109	950	227	1269	32
Solyc06g007680.1.1	5	19	4	30	1	10	30	22	2	34	3	16	0
Solyc06g007690.1.1	0	0	0	0	0	0	0	0	0	0	0	0	0
Solyc06g007700.2.1	7	130	17	106	10	329	141	345	19	400	105	383	12
Solyc06g007710.2.1	6	34	5	17	0	82	22	119	7	89	13	65	1
Solyc06g007730.1.1	0	0	0	0	0	0	0	0	0	0	0	0	0
Solyc06g007740.1.1	0	0	0	0	0	0	0	0	0	0	0	0	0
Solyc06g007760.2.1	5	150	1	44	0	43	25	73	0	62	4	63	0
Solyc06g007770.2.1	0	0	0	0	0	0	0	0	0	0	0	0	0
Solyc06g007780.2.1	5	6	4	12	0	10	23	32	8	18	15	13	0
Solyc06g007790.1.1	0	0	0	0	0	0	0	0	0	0	0	0	0
Solyc06g007800.2.1	3	11	2	5	0	14	21	24	5	22	3	24	0
Solyc06g007830.1.1	0	0	0	0	0	0	0	0	0	0	0	0	0
Solyc06g007850.2.1	0	0	0	0	0	0	0	0	0	0	0	0	0
Solyc06g007860.1.1	0	0	0	0	0	0	0	0	0	0	0	0	0
Solyc06g007870.2.1	4	19	0	13	2	48	10	44	5	57	15	64	0
Solyc06g007890.2.1	0	0	0	0	0	0	0	0	0	0	0	0	0

Solyc06g007910.2.1	1	6	0	3	1	0	19	3	2	4	10	0	1
Solyc06g007930.2.1	6	54	3	26	1	47	36	66	19	40	17	40	0
Solyc06g007940.1.1	0	0	0	0	0	0	0	0	0	0	0	0	0
Solyc06g007970.2.1	0	0	0	0	0	0	0	0	0	0	0	0	0
Solyc06g007980.2.1	11	234	13	109	2	149	66	170	3	410	44	394	2
Solyc06g007990.2.1	3	13	6	2	0	3	49	19	0	6	14	10	0
Solyc06g008010.2.1	5	113	26	56	14	210	337	139	31	144	61	79	6
Solyc06g008020.2.1	1	13	0	3	0	0	5	9	3	1	5	2	0
Solyc06g008030.2.1	17	88	16	58	6	196	130	157	7	215	5	65	0
Solyc06g008040.2.1	13	92	12	57	8	45	57	106	15	155	21	154	4
Solyc06g008050.2.1	0	0	0	0	0	0	0	0	0	0	0	0	0
Solyc06g008080.1.1	0	0	0	0	0	0	0	0	0	0	0	0	0
Solyc06g008090.2.1	10	18	4	15	2	47	25	56	4	149	23	107	0
Solyc06g008100.2.1	1	9	0	0	0	2	16	7	11	2	0	0	0
Solyc06g008110.2.1	10	47	12	32	9	104	104	161	31	113	50	68	5
Solyc06g008120.1.1	2	23	9	11	3	31	51	21	8	17	20	20	2
Solyc06g008130.2.1	7	29	1	14	0	31	31	44	4	61	9	32	1
Solyc06g008150.2.1	1	3	3	0	0	5	0	1	0	1	1	3	0
Solyc06g008160.2.1	2	23	4	15	0	2	5	8	0	12	3	9	0
Solyc06g008170.2.1	2	45	0	28	0	35	22	73	7	120	12	238	3
Solyc06g008180.1.1	0	0	0	0	0	0	0	0	0	0	0	0	0
Solyc06g008190.1.1	0	0	0	0	0	0	0	0	0	0	0	0	0
Solyc06g008200.2.1	9	62	1	67	4	207	80	194	10	206	6	155	1
Solyc06g008210.2.1	0	0	0	0	0	0	0	0	0	0	0	0	0
Solyc06g008220.2.1	8	88	8	55	2	54	154	107	5	178	20	165	0
Solyc06g008230.2.1	13	60	13	30	3	119	84	158	12	90	19	66	0
Solyc06g008250.2.1	3	22	0	22	0	40	11	37	2	51	2	54	0
Solyc06g008260.2.1	3	94	0	28	0	76	14	99	6	299	43	142	1
Solyc06g008270.2.1	0	0	0	0	0	0	0	0	0	0	0	0	0
Solyc06g008280.2.1	2	8	0	0	0	2	10	9	0	11	0	6	0
Solyc06g008290.2.1	0	0	0	0	0	0	0	0	0	0	0	0	0
Solyc06g008300.2.1	19	147	68	127	2	21	82	66	6	104	91	3	2
Solyc06g008310.2.1	10	40	5	31	2	94	67	93	13	66	36	90	0
Solyc06g008320.2.1	3	62	17	49	3	165	305	118	42	91	55	33	4
Solyc06g008330.1.1	0	0	0	0	0	0	0	0	0	0	0	0	0
Solyc06g008340.2.1	7	107	9	76	0	142	85	166	24	133	64	75	5
Solyc06g008350.2.1	2	14	0	1	0	10	6	15	0	25	6	31	1
Solyc06g008360.2.1	0	0	0	0	0	0	0	0	0	0	0	0	0
Solyc06g008380.1.1	0	0	0	0	0	0	0	0	0	0	0	0	0
Solyc06g008450.2.1	0	0	0	0	0	0	0	0	0	0	0	0	0
Solyc06g008490.2.1	11	32	4	17	0	84	77	118	12	55	17	40	0

Solyc06g008500.2.1	1	6	1	0	0	0	0	3	0	0	1	1	0
Solyc06g008510.2.1	0	0	0	0	0	0	0	0	0	0	0	0	0
Solyc06g008520.2.1	2	17	4	6	4	43	6	47	3	52	5	46	0
Solyc06g008530.1.1	0	0	0	0	0	0	0	0	0	0	0	0	0
Solyc06g008560.1.1	0	0	0	0	0	0	0	0	0	0	0	0	0
Solyc06g008580.2.1	2	24	0	5	0	0	12	0	8	22	1	13	0
Solyc06g008590.2.1	1	22	3	7	0	0	4	0	1	40	0	30	4
Solyc06g008600.2.1	3	10	0	20	0	32	16	24	1	31	11	17	2
Solyc06g008610.2.1	7	59	4	58	3	96	68	200	25	372	34	269	10
Solyc06g008620.1.1	0	0	0	0	0	0	0	0	0	0	0	0	0
Solyc06g008690.1.1	0	0	0	0	0	0	0	0	0	0	0	0	0
Solyc06g008700.2.1	7	28	4	6	0	81	50	114	16	40	24	57	4
Solyc06g008710.1.1	0	0	0	0	0	0	0	0	0	0	0	0	0
Solyc06g008720.2.1	20	97	14	67	11	192	207	276	36	155	36	124	3
Solyc06g008730.2.1	7	19	3	2	0	30	39	71	7	44	10	43	1
Solyc06g008740.2.1	6	24	4	25	4	8	15	29	0	49	22	60	0
Solyc06g008760.1.1	0	0	0	0	0	0	0	0	0	0	0	0	0
Solyc06g008770.1.1	0	0	0	0	0	0	0	0	0	0	0	0	0
Solyc06g008790.2.1	0	0	0	0	0	0	0	0	0	0	0	0	0
Solyc06g008800.1.1	0	0	0	0	0	0	0	0	0	0	0	0	0
Solyc06g008810.2.1	0	0	0	0	0	0	0	0	0	0	0	0	0
Solyc06g008820.2.1	2	2	0	12	1	8	8	10	4	6	4	23	0
Solyc06g008840.2.1	0	0	0	0	0	0	0	0	0	0	0	0	0
Solyc06g008850.2.1	12	143	6	148	12	289	148	380	24	425	47	298	0
Solyc06g008860.2.1	5	73	7	38	0	97	75	143	13	73	15	91	1
Solyc06g008870.2.1	4	38	6	32	2	192	33	134	8	10	9	23	0
Solyc06g008880.2.1	9	61	5	65	4	48	94	134	5	90	21	110	10
Solyc06g008890.2.1	0	0	0	0	0	0	0	0	0	0	0	0	0
Solyc06g008900.2.1	2	3	0	7	0	9	4	10	1	4	1	20	0
Solyc06g008920.2.1	22	39	1	35	1	313	45	316	2	73	285	26	0
Solyc06g008930.2.1	0	0	0	0	0	0	0	0	0	0	0	0	0
Solyc06g008940.2.1	3	6	1	3	1	18	16	17	5	21	24	36	0
Solyc06g008960.2.1	12	11	5	13	2	29	45	48	7	65	18	55	4
Solyc06g008970.2.1	2	3	0	6	0	3	9	11	2	9	2	4	0
Solyc06g008980.2.1	0	0	0	0	0	0	0	0	0	0	0	0	0
Solyc06g009020.2.1	6	231	21	149	12	400	243	222	44	315	138	337	9
Solyc06g009050.2.1	6	77	9	50	0	661	360	588	21	143	286	29	5
Solyc06g009060.2.1	5	106	8	66	2	118	86	115	21	133	35	100	0
Solyc06g009070.2.1	2	8	0	0	2	16	20	0	3	101	7	91	5
Solyc06g009080.1.1	0	0	0	0	0	0	0	0	0	0	0	0	0
Solyc06g009110.2.1	2	30	2	25	0	19	3	27	4	1	9	1	0

Solyc06g009120.2.1	4	55	6	22	1	31	23	24	10	27	23	46	0
Solyc06g009130.2.1	6	28	8	13	0	11	50	30	1	92	33	40	0
Solyc06g009140.2.1	2	368	310	1324	103	1496	2246	1462	128	384	338	579	29
Solyc06g009150.1.1	2	10	1	12	0	1	23	4	0	0	2	0	0
Solyc06g009160.2.1	6	25	3	12	0	10	50	42	5	35	10	28	0
Solyc06g009170.2.1	6	87	4	75	5	276	153	176	15	235	24	65	0
Solyc06g009190.2.1	19	264	61	25	88	18	289	80	176	3568	3	1759	2
Solyc06g009200.2.1	0	0	0	0	0	0	0	0	0	0	0	0	0
Solyc06g009210.2.1	2	55	4	36	1	185	31	225	6	147	36	140	1
Solyc06g009220.2.1	12	180	17	105	1	225	65	242	11	269	16	182	1
Solyc06g009240.2.1	0	0	0	0	0	0	0	0	0	0	0	0	0
Solyc06g009260.2.1	0	0	0	0	0	0	0	0	0	0	0	0	0
Solyc06g009270.2.1	6	86	8	36	8	221	53	165	24	35	9	21	18
Solyc06g009280.1.1	0	0	0	0	0	0	0	0	0	0	0	0	0
Solyc06g009290.2.1	0	0	0	0	0	0	0	0	0	0	0	0	0
Solyc06g009330.2.1	1	4	0	0	0	0	6	5	0	0	0	0	0
Solyc06g009340.2.1	1	35	3	9	1	6	6	13	0	67	4	45	0
Solyc06g009350.2.1	5	18	2	12	1	86	127	57	10	96	29	37	2
Solyc06g009360.1.1	6	18	1	3	4	70	31	49	3	64	8	35	0
Solyc06g009370.2.1	3	8	0	10	0	49	18	55	0	35	0	18	0
Solyc06g009380.2.1	7	88	8	51	1	568	189	552	9	164	57	113	0
Solyc06g009390.2.1	4	3	1	2	0	70	23	44	1	9	3	5	0
Solyc06g009400.2.1	4	52	3	32	1	42	55	101	2	90	16	58	4
Solyc06g009490.2.1	1	3	0	2	0	0	3	0	0	12	5	2	0
Solyc06g009500.1.1	0	0	0	0	0	0	0	0	0	0	0	0	0
Solyc06g009510.1.1	0	0	0	0	0	0	0	0	0	0	0	0	0
Solyc06g009520.2.1	11	129	18	89	1	93	116	252	17	128	33	170	4
Solyc06g009530.2.1	30	128	23	89	25	610	322	634	77	179	103	140	8
Solyc06g009620.1.1	0	0	0	0	0	0	0	0	0	0	0	0	0
Solyc06g009630.1.1	7	746	52	253	8	256	306	475	10	932	83	532	5
Solyc06g009640.1.1	4	79	9	53	33	103	97	29	28	83	68	129	16
Solyc06g009650.2.1	12	78	10	68	2	219	110	248	26	179	25	191	1
Solyc06g009660.2.1	8	65	7	58	3	78	110	87	27	114	45	90	1
Solyc06g009670.2.1	0	0	0	0	0	0	0	0	0	0	0	0	0
Solyc06g009680.2.1	0	0	0	0	0	0	0	0	0	0	0	0	0
Solyc06g009690.1.1	0	0	0	0	0	0	0	0	0	0	0	0	0
Solyc06g009750.2.1	14	200	14	147	5	372	128	453	22	619	54	306	6
Solyc06g009770.1.1	6	19	1	2	7	24	26	4	58	216	14	28	37
Solyc06g009780.2.1	1	8	0	2	0	5	2	5	0	2	2	1	0
Solyc06g009790.2.1	0	0	0	0	0	0	0	0	0	0	0	0	0
Solyc06g009800.1.1	0	0	0	0	0	0	0	0	0	0	0	0	0

Solyc06g009810.2.1	0	0	0	0	0	0	0	0	0	0	0	0	0
Solyc06g009820.2.1	0	0	0	0	0	0	0	0	0	0	0	0	0
Solyc06g009830.2.1	0	0	0	0	0	0	0	0	0	0	0	0	0
Solyc06g009900.1.1	0	0	0	0	0	0	0	0	0	0	0	0	0
Solyc06g009960.1.1	0	0	0	0	0	0	0	0	0	0	0	0	0
Solyc06g009970.2.1	10	395	31	219	43	567	219	844	173	1339	189	1343	233
Solyc06g009990.1.1	0	0	0	0	0	0	0	0	0	0	0	0	0
Solyc06g010000.2.1	10	29	10	49	6	136	75	214	18	120	12	33	1
Solyc06g010010.1.1	0	0	0	0	0	0	0	0	0	0	0	0	0
Solyc06g010030.2.1	0	0	0	0	0	0	0	0	0	0	0	0	0
Solyc06g010060.1.1	0	0	0	0	0	0	0	0	0	0	0	0	0
Solyc06g010170.2.1	0	0	0	0	0	0	0	0	0	0	0	0	0
Solyc06g010200.2.1	26	148	15	141	6	510	263	673	26	306	35	210	4
Solyc06g010210.2.1	0	0	0	0	0	0	0	0	0	0	0	0	0
Solyc06g010220.1.1	0	0	0	0	0	0	0	0	0	0	0	0	0
Solyc06g010230.2.1	0	0	0	0	0	0	0	0	0	0	0	0	0
Solyc06g010240.1.1	0	0	0	0	0	0	0	0	0	0	0	0	0
Solyc06g010250.2.1	0	0	0	0	0	0	0	0	0	0	0	0	0
Solyc06g010260.2.1	0	0	0	0	0	0	0	0	0	0	0	0	0
Solyc06g011280.2.1	4	132	13	100	5	412	81	306	43	372	128	358	8
Solyc06g011350.2.1	6	20	8	19	5	103	102	62	0	33	16	19	1
Solyc06g011370.2.1	0	0	0	0	0	0	0	0	0	0	0	0	0
Solyc06g011380.2.1	0	0	0	0	0	0	0	0	0	0	0	0	0
Solyc06g011400.2.1	0	0	0	0	0	0	0	0	0	0	0	0	0
Solyc06g011440.1.1	0	0	0	0	0	0	0	0	0	0	0	0	0
Solyc06g011450.1.1	13	157	10	110	16	181	97	181	16	147	13	68	0
Solyc06g011470.2.1	0	0	0	0	0	0	0	0	0	0	0	0	0
Solyc06g011530.2.1	2	25	1	23	0	28	17	19	3	30	10	82	0
Solyc06g011540.2.1	2	10	3	2	0	7	11	8	1	25	9	21	1
Solyc06g011550.2.1	3	25	2	24	0	4	6	12	0	17	0	16	0
Solyc06g011560.2.1	2	7	1	4	2	18	29	19	8	14	23	8	3
Solyc06g011570.2.1	2	3	0	0	0	0	7	0	0	6	0	7	0
Solyc06g011580.1.1	0	0	0	0	0	0	0	0	0	0	0	0	0
Solyc06g011590.1.1	1	0	0	0	0	0	6	0	0	4	0	4	0
Solyc06g016660.2.1	0	0	0	0	0	0	0	0	0	0	0	0	0
Solyc06g016750.2.1	5	21	4	18	0	15	36	40	3	37	3	17	0
Solyc06g016760.1.1	0	0	0	0	0	0	0	0	0	0	0	0	0
Solyc06g016770.2.1	3	6	5	5	0	4	21	13	10	9	1	1	0
Solyc06g016790.1.1	1	5	0	0	0	6	1	2	0	5	2	10	0
Solyc06g016800.1.1	0	0	0	0	0	0	0	0	0	0	0	0	0
Solyc06g017860.1.1	0	0	0	0	0	0	0	0	0	0	0	0	0

Solyc06g017970.2.1	13	76	11	45	6	159	113	274	21	147	59	104	4
Solyc06g018050.2.1	5	14	2	13	4	13	25	25	2	43	20	12	0
Solyc06g018100.2.1	3	18	0	6	0	0	15	8	0	35	4	4	0
Solyc06g018110.2.1	0	0	0	0	0	0	0	0	0	0	0	0	0
Solyc06g019170.2.1	1	14	1	6	0	36	81	47	0	15	3	8	0
Solyc06g019200.2.1	8	54	10	23	0	11	157	28	2	33	10	20	2
Solyc06g024200.1.1	0	0	0	0	0	0	0	0	0	0	0	0	0
Solyc06g024210.1.1	0	0	0	0	0	0	0	0	0	0	0	0	0
Solyc06g024220.1.1	0	0	0	0	0	0	0	0	0	0	0	0	0
Solyc06g024230.1.1	0	0	0	0	0	0	0	0	0	0	0	0	0
Solyc06g024240.1.1	0	0	0	0	0	0	0	0	0	0	0	0	0
Solyc06g024250.1.1	1	3	0	3	1	2	0	1	0	1	4	2	2
Solyc06g024260.1.1	0	0	0	0	0	0	0	0	0	0	0	0	0
Solyc06g024290.1.1	0	0	0	0	0	0	0	0	0	0	0	0	0
Solyc06g024300.1.1	1	0	3	0	48	1	19	0	24	0	129	0	13
Solyc06g024340.1.1	0	0	0	0	0	0	0	0	0	0	0	0	0
Solyc06g024350.1.1	1	44	0	88	5	7	0	22	1	68	6	28	3
Solyc06g024360.1.1	0	0	0	0	0	0	0	0	0	0	0	0	0
Solyc06g024370.1.1	2	3	0	1	12	0	5	1	2	0	11	0	3
Solyc06g024380.1.1	0	0	0	0	0	0	0	0	0	0	0	0	0
Solyc06g030470.2.1	1	18	0	4	0	2	5	19	0	6	0	1	0
Solyc06g030490.2.1	0	0	0	0	0	0	0	0	0	0	0	0	0
Solyc06g030510.2.1	0	0	0	0	0	0	0	0	0	0	0	0	0
Solyc06g030530.2.1	1	2	2	2	0	0	21	8	6	10	4	18	0
Solyc06g030540.2.1	1	11	2	4	0	6	14	20	0	27	0	26	0
Solyc06g030560.1.1	0	0	0	0	0	0	0	0	0	0	0	0	0
Solyc06g030570.2.1	3	19	5	8	0	37	42	30	1	32	3	30	0
Solyc06g030580.2.1	1	9	2	2	0	27	23	34	1	8	13	18	0
Solyc06g030590.1.1	0	0	0	0	0	0	0	0	0	0	0	0	0
Solyc06g031680.2.1	2	1	3	5	2	27	139	45	29	19	42	33	0
Solyc06g031690.2.1	2	30	0	4	4	33	123	76	20	76	10	36	3
Solyc06g031710.2.1	0	0	0	0	0	0	0	0	0	0	0	0	0
Solyc06g033850.2.1	0	0	0	0	0	0	0	0	0	0	0	0	0
Solyc06g033860.1.1	0	0	0	0	0	0	0	0	0	0	0	0	0
Solyc06g033920.1.1	0	0	0	0	0	0	0	0	0	0	0	0	0
Solyc06g034020.2.1	4	176	60	145	33	337	435	205	29	538	73	325	4
Solyc06g034040.1.1	0	0	0	0	0	0	0	0	0	0	0	0	0
Solyc06g034100.2.1	3	17	9	14	6	49	106	50	13	26	43	32	5
Solyc06g034110.2.1	7	86	2	32	0	108	23	75	1	56	4	21	0
Solyc06g034120.2.1	9	101	13	107	4	272	204	407	30	417	49	310	5
Solyc06g034140.1.1	0	0	0	0	0	0	0	0	0	0	0	0	0

Solyc06g034150.2.1	1	5	0	0	0	0	4	3	0	5	1	1	0
Solyc06g034160.2.1	4	106	8	68	3	212	132	200	18	125	40	110	1
Solyc06g034220.2.1	4	21	1	21	0	41	14	72	4	54	12	10	0
Solyc06g034230.1.1	1	0	0	5	0	1	0	7	2	5	4	4	0
Solyc06g034290.2.1	13	31	12	36	3	150	120	461	3	25	11	3	0
Solyc06g034320.1.1	1	4	2	3	0	1	1	18	0	17	2	4	0
Solyc06g034330.2.1	14	129	10	90	4	153	103	198	13	301	66	163	5
Solyc06g034340.1.1	0	0	0	0	0	0	0	0	0	0	0	0	0
Solyc06g034360.1.1	0	0	0	0	0	0	0	0	0	0	0	0	0
Solyc06g034370.1.1	2	2	3	0	0	44	25	97	2	1	0	0	1
Solyc06g034380.1.1	0	0	0	0	0	0	0	0	0	0	0	0	0
Solyc06g034390.1.1	1	0	0	3	3	0	8	0	0	5	6	5	4
Solyc06g034410.2.1	1	0	0	0	0	40	3	13	1	0	0	0	0
Solyc06g035430.2.1	0	0	0	0	0	0	0	0	0	0	0	0	0
Solyc06g035440.2.1	0	0	0	0	0	0	0	0	0	0	0	0	0
Solyc06g035450.2.1	6	210	114	120	37	665	1487	589	103	1339	547	942	62
Solyc06g035460.2.1	12	410	68	242	62	570	507	689	102	2055	567	1623	38
Solyc06g035520.2.1	8	12	30	11	26	39	24	37	11	2	0	6	0
Solyc06g035530.2.1	0	0	0	0	0	0	0	0	0	0	0	0	0
Solyc06g035560.1.1	0	0	0	0	0	0	0	0	0	0	0	0	0
Solyc06g035570.1.1	0	0	0	0	0	0	0	0	0	0	0	0	0
Solyc06g035580.2.1	18	51	12	0	37	3	18	7	10	827	131	22	140
Solyc06g035600.2.1	0	0	0	0	0	0	0	0	0	0	0	0	0
Solyc06g035620.2.1	0	0	0	0	0	0	0	0	0	0	0	0	0
Solyc06g035630.1.1	0	0	0	0	0	0	0	0	0	0	0	0	0
Solyc06g035690.2.1	8	110	1	63	2	395	47	209	17	114	47	184	5
Solyc06g035700.1.1	3	65	12	27	8	27	0	5	0	0	0	0	0
Solyc06g035720.2.1	5	34	1	30	6	89	28	97	19	484	30	278	1
Solyc06g035760.2.1	1	2	0	4	0	0	0	2	0	8	6	7	3
Solyc06g035790.2.1	1	0	7	0	0	0	0	0	0	8	11	8	0
Solyc06g035840.1.1	0	0	0	0	0	0	0	0	0	0	0	0	0
Solyc06g035850.1.1	0	0	0	0	0	0	0	0	0	0	0	0	0
Solyc06g035860.1.1	0	0	0	0	0	0	0	0	0	0	0	0	0
Solyc06g035870.2.1	4	178	8	56	1	237	159	231	23	295	37	216	5
Solyc06g035920.2.1	2	29	0	21	0	11	18	73	4	67	3	73	1
Solyc06g035940.2.1	0	0	0	0	0	0	0	0	0	0	0	0	0
Solyc06g035960.2.1	7	42	20	31	1	486	9	182	6	9	9	10	0
Solyc06g035970.2.1	4	222	30	129	21	308	149	304	27	1090	139	859	3
Solyc06g035990.2.1	3	8	3	2	0	8	29	37	1	6	6	4	0
Solyc06g036000.2.1	2	11	0	8	0	23	20	21	3	2	4	4	0
Solyc06g036050.2.1	2	126	11	46	10	123	59	121	12	299	108	140	5

Solyc06g036060.2.1	5	1	2	16	0	8	9	5	1	41	16	55	4
Solyc06g036070.2.1	2	12	0	0	0	9	13	10	4	2	2	3	1
Solyc06g036080.2.1	5	22	6	23	1	62	33	57	6	28	10	25	0
Solyc06g036100.2.1	3	13	0	22	2	5	17	7	4	99	5	87	2
Solyc06g036110.1.1	11	260	50	121	5	2657	174	1682	272	29	99	36	13
Solyc06g036130.2.1	8	14	2	8	2	85	35	66	9	57	46	40	1
Solyc06g036170.1.1	15	112	49	103	13	219	100	185	11	345	50	207	8
Solyc06g036190.1.1	0	0	0	0	0	0	0	0	0	0	0	0	0
Solyc06g036240.1.1	1	0	0	0	0	5	5	4	0	0	0	0	0
Solyc06g036250.1.1	0	0	0	0	0	0	0	0	0	0	0	0	0
Solyc06g036260.2.1	5	225	20	118	4	478	149	545	4	61	36	75	0
Solyc06g036290.2.1	7	12	1	1	6	42	11	35	0	7	125	53	0
Solyc06g036300.2.1	2	2	0	4	0	7	0	9	0	23	7	11	0
Solyc06g036310.2.1	1	16	2	19	0	0	2	2	0	10	0	39	0
Solyc06g036330.1.1	10	118	19	110	0	174	114	224	14	102	22	110	2
Solyc06g036340.2.1	4	28	2	20	0	5	16	30	2	27	5	16	1
Solyc06g036350.2.1	4	93	13	37	1	123	73	117	28	514	70	298	6
Solyc06g036410.1.1	0	0	0	0	0	0	0	0	0	0	0	0	0
Solyc06g036420.1.1	0	0	0	0	0	0	0	0	0	0	0	0	0
Solyc06g036430.2.1	2	17	4	3	12	8	34	8	3	32	38	45	11
Solyc06g036440.1.1	0	0	0	0	0	0	0	0	0	0	0	0	0
Solyc06g036470.1.1	2	0	3	0	0	7	21	5	22	0	1	1	0
Solyc06g036490.1.1	0	0	0	0	0	0	0	0	0	0	0	0	0
Solyc06g036520.1.1	1	9	0	9	1	15	8	16	2	19	3	112	0
Solyc06g036530.1.1	0	0	0	0	0	0	0	0	0	0	0	0	0
Solyc06g036540.1.1	1	19	3	10	1	49	85	66	12	54	12	35	3
Solyc06g036580.2.1	13	77	13	58	2	187	62	257	20	96	39	112	0
Solyc06g036590.1.1	4	23	0	23	4	53	29	64	3	16	6	19	2
Solyc06g036620.1.1	0	0	0	0	0	0	0	0	0	0	0	0	0
Solyc06g036640.2.1	10	72	2	52	8	265	123	354	4	40	1	35	1
Solyc06g036720.2.1	8	41	7	41	0	142	113	116	10	119	58	137	1
Solyc06g036770.1.1	0	0	0	0	0	0	0	0	0	0	0	0	0
Solyc06g036780.2.1	2	3	0	0	4	0	3	2	1	10	9	3	1
Solyc06g042950.2.1	1	7	2	9	4	0	0	6	0	3	3	6	0
Solyc06g042960.1.1	1	2	1	1	0	6	5	11	1	13	0	10	0
Solyc06g042970.1.1	0	0	0	0	0	0	0	0	0	0	0	0	0
Solyc06g042980.2.1	0	0	0	0	0	0	0	0	0	0	0	0	0
Solyc06g042990.1.1	0	0	0	0	0	0	0	0	0	0	0	0	0
Solyc06g043020.1.1	1	2	1	0	9	0	4	0	0	1	1	0	1
Solyc06g043030.2.1	8	37	6	12	2	25	51	81	13	141	30	57	2
Solyc06g043060.1.1	0	0	0	0	0	0	0	0	0	0	0	0	0

Solyc06g043070.2.1	0	0	0	0	0	0	0	0	0	0	0	0	0
Solyc06g043140.2.1	0	0	0	0	0	0	0	0	0	0	0	0	0
Solyc06g043150.2.1	0	0	0	0	0	0	0	0	0	0	0	0	0
Solyc06g043160.1.1	0	0	0	0	0	0	0	0	0	0	0	0	0
Solyc06g043170.2.1	9	86	7	67	6	126	90	195	19	118	39	103	0
Solyc06g043270.1.1	0	0	0	0	0	0	0	0	0	0	0	0	0
Solyc06g048410.2.1	11	495	84	375	8	550	132	387	2	554	56	782	0
Solyc06g048420.1.1	0	0	0	0	0	0	0	0	0	0	0	0	0
Solyc06g048430.2.1	0	0	0	0	0	0	0	0	0	0	0	0	0
Solyc06g048440.2.1	1	0	0	0	0	0	0	1	0	5	544	3	3
Solyc06g048450.2.1	0	0	0	0	0	0	0	0	0	0	0	0	0
Solyc06g048460.1.1	2	0	2	1	0	3	14	5	3	6	9	9	1
Solyc06g048470.2.1	14	71	11	18	6	7	36	30	5	216	47	132	0
Solyc06g048480.2.1	4	24	1	7	2	23	18	29	5	18	5	29	0
Solyc06g048510.2.1	8	45	3	38	7	56	67	102	7	110	33	83	2
Solyc06g048520.2.1	3	33	4	3	0	47	14	21	3	33	5	34	0
Solyc06g048530.2.1	0	0	0	0	0	0	0	0	0	0	0	0	0
Solyc06g048540.2.1	6	99	9	42	2	94	86	140	1	130	8	85	1
Solyc06g048570.2.1	2	8	0	2	0	2	3	8	1	8	0	1	0
Solyc06g048590.2.1	3	7	3	3	1	8	21	13	1	29	8	15	0
Solyc06g048610.2.1	1	8	1	0	1	12	18	33	5	6	13	4	0
Solyc06g048620.2.1	0	0	0	0	0	0	0	0	0	0	0	0	0
Solyc06g048630.2.1	0	0	0	0	0	0	0	0	0	0	0	0	0
Solyc06g048700.1.1	0	0	0	0	0	0	0	0	0	0	0	0	0
Solyc06g048730.2.1	7	164	21	101	1	159	149	255	12	50	24	46	0
Solyc06g048750.2.1	0	0	0	0	0	0	0	0	0	0	0	0	0
Solyc06g048810.2.1	4	42	2	27	3	55	32	41	8	39	27	68	1
Solyc06g048820.1.1	3	119	4	14	0	6	6	9	0	51	6	40	0
Solyc06g048840.2.1	2	5	0	0	0	23	0	3	2	0	763	1	3
Solyc06g048890.2.1	8	26	4	32	0	39	57	63	6	71	16	50	1
Solyc06g048900.1.1	0	0	0	0	0	0	0	0	0	0	0	0	0
Solyc06g048920.2.1	0	0	0	0	0	0	0	0	0	0	0	0	0
Solyc06g048930.2.1	0	0	0	0	0	0	0	0	0	0	0	0	0
Solyc06g048940.2.1	11	72	6	80	5	197	61	309	13	72	18	88	0
Solyc06g048950.2.1	3	12	0	7	0	5	9	4	1	9	11	1	0
Solyc06g048960.2.1	1	0	0	0	0	2	4	4	0	2	0	1	0
Solyc06g048970.2.1	1	1	3	1	5	3	146	3	8	4	12	0	1
Solyc06g049020.1.1	4	244	27	214	14	25	6	24	7	88	11	64	1
Solyc06g049030.2.1	5	26	4	5	0	60	92	36	33	22	3	14	1
Solyc06g049040.2.1	4	24	6	11	8	3	26	5	9	18	3	9	0
Solyc06g049050.2.1	0	0	0	0	0	0	0	0	0	0	0	0	0

Solyc06g049060.2.1	2	2	0	2	0	5	7	3	2	3	3	3	0
Solyc06g049070.2.1	4	46	6	4	2	69	34	102	10	84	9	73	0
Solyc06g049080.2.1	6	168	5	79	2	241	80	223	9	335	34	194	0
Solyc06g049090.2.1	0	0	0	0	0	0	0	0	0	0	0	0	0
Solyc06g049100.2.1	0	0	0	0	0	0	0	0	0	0	0	0	0
Solyc06g050120.2.1	2	81	14	65	9	183	102	330	19	311	76	214	3
Solyc06g050130.2.1	8	75	1	22	0	128	141	133	19	89	8	47	3
Solyc06g050140.1.1	0	0	0	0	0	0	0	0	0	0	0	0	0
Solyc06g050160.2.1	0	0	0	0	0	0	0	0	0	0	0	0	0
Solyc06g050170.2.1	31	317	21	121	56	74	164	246	50	915	365	566	75
Solyc06g050210.2.1	3	6	0	1	4	7	4	21	3	5	5	20	1
Solyc06g050220.2.1	0	0	0	0	0	0	0	0	0	0	0	0	0
Solyc06g050230.2.1	4	16	4	2	0	11	25	25	3	19	7	25	0
Solyc06g050250.1.1	4	14	0	1	3	85	32	42	5	1	1	3	0
Solyc06g050300.1.1	7	20	2	23	1	145	39	88	5	35	15	36	0
Solyc06g050310.2.1	3	66	4	45	0	33	52	93	1	49	5	48	0
Solyc06g050320.2.1	0	0	0	0	0	0	0	0	0	0	0	0	0
Solyc06g050340.2.1	0	0	0	0	0	0	0	0	0	0	0	0	0
Solyc06g050350.2.1	4	21	4	4	0	4	12	27	17	42	0	20	0
Solyc06g050370.1.1	2	34	12	15	19	16	25	6	28	34	6	21	1
Solyc06g050430.2.1	0	0	0	0	0	0	0	0	0	0	0	0	0
Solyc06g050440.2.1	6	26	0	5	0	37	111	31	55	1	8	3	0
Solyc06g050500.2.1	1	7	0	15	0	6	2	1	0	18	5	2	0
Solyc06g050510.2.1	17	121	11	79	1	110	99	210	23	273	49	177	8
Solyc06g050520.1.1	0	0	0	0	0	0	0	0	0	0	0	0	0
Solyc06g050530.2.1	0	0	0	0	0	0	0	0	0	0	0	0	0
Solyc06g050550.2.1	12	103	14	82	0	161	94	278	29	340	35	282	5
Solyc06g050560.2.1	0	0	0	0	0	0	0	0	0	0	0	0	0
Solyc06g050570.1.1	3	65	2	51	7	63	29	83	9	126	4	74	0
Solyc06g050590.2.1	5	43	6	24	8	79	36	136	16	159	42	102	3
Solyc06g050600.2.1	0	0	0	0	0	0	0	0	0	0	0	0	0
Solyc06g050610.1.1	0	0	0	0	0	0	0	0	0	0	0	0	0
Solyc06g050620.2.1	6	60	9	30	0	23	41	36	7	20	9	25	0
Solyc06g050630.2.1	3	12	4	9	3	14	19	3	3	4	1	52	0
Solyc06g050690.2.1	0	0	0	0	0	0	0	0	0	0	0	0	0
Solyc06g050700.2.1	2	18	1	10	0	2	6	39	0	59	0	25	0
Solyc06g050710.2.1	0	0	0	0	0	0	0	0	0	0	0	0	0
Solyc06g050720.2.1	7	59	1	37	1	135	38	90	10	121	8	89	0
Solyc06g050730.2.1	0	0	0	0	0	0	0	0	0	0	0	0	0
Solyc06g050760.1.1	5	34	6	9	3	9	8	3	2	154	23	105	1
Solyc06g050770.2.1	6	90	30	45	9	223	652	249	113	208	153	134	6

Solyc06g050780.1.1	0	0	0	0	0	0	0	0	0	0	0	0	0
Solyc06g050790.2.1	5	4	0	6	0	46	50	39	2	13	4	8	0
Solyc06g050800.2.1	8	11	3	28	2	384	244	417	70	34	16	26	0
Solyc06g050810.2.1	2	19	2	11	0	12	4	49	5	2	0	0	0
Solyc06g050820.1.1	0	0	0	0	0	0	0	0	0	0	0	0	0
Solyc06g050840.2.1	0	0	0	0	0	0	0	0	0	0	0	0	0
Solyc06g050860.2.1	0	0	0	0	0	0	0	0	0	0	0	0	0
Solyc06g050880.2.1	5	20	4	16	7	173	86	111	10	40	14	39	0
Solyc06g050900.2.1	2	15	7	6	4	0	0	0	0	0	1	2	1
Solyc06g050910.1.1	0	0	0	0	0	0	0	0	0	0	0	0	0
Solyc06g050920.2.1	1	15	0	3	0	1	1	17	0	0	0	0	0
Solyc06g050930.2.1	7	21	3	19	1	96	60	157	10	32	1	22	0
Solyc06g050940.1.1	0	0	0	0	0	0	0	0	0	0	0	0	0
Solyc06g050950.2.1	0	0	0	0	0	0	0	0	0	0	0	0	0
Solyc06g050980.2.1	5	1327	61	900	6	1425	383	870	9	468	140	938	10
Solyc06g050990.2.1	4	23	2	48	0	351	95	173	24	99	12	106	0
Solyc06g051010.1.1	3	74	8	47	11	24	9	15	4	1	0	2	0
Solyc06g051020.2.1	23	89	11	58	21	494	735	827	263	274	49	226	5
Solyc06g051030.2.1	16	204	13	129	1	115	67	126	15	87	23	165	10
Solyc06g051040.2.1	0	0	0	0	0	0	0	0	0	0	0	0	0
Solyc06g051060.2.1	0	0	0	0	0	0	0	0	0	0	0	0	0
Solyc06g051080.2.1	13	162	34	81	1	204	131	208	6	37	16	48	4
Solyc06g051090.1.1	0	0	0	0	0	0	0	0	0	0	0	0	0
Solyc06g051120.2.1	6	27	5	22	0	44	16	45	7	40	19	27	0
Solyc06g051130.1.1	0	0	0	0	0	0	0	0	0	0	0	0	0
Solyc06g051150.1.1	0	0	0	0	0	0	0	0	0	0	0	0	0
Solyc06g051160.2.1	1	26	0	9	2	77	21	62	2	49	3	51	0
Solyc06g051170.2.1	0	0	0	0	0	0	0	0	0	0	0	0	0
Solyc06g051190.1.1	0	0	0	0	0	0	0	0	0	0	0	0	0
Solyc06g051200.2.1	7	155	8	98	2	44	55	85	4	88	14	97	2
Solyc06g051210.2.1	7	56	14	37	8	71	38	49	6	71	11	55	0
Solyc06g051230.2.1	0	0	0	0	0	0	0	0	0	0	0	0	0
Solyc06g051250.1.1	0	0	0	0	0	0	0	0	0	0	0	0	0
Solyc06g051260.2.1	1	27	3	32	0	25	2	18	0	35	9	28	0
Solyc06g051270.2.1	10	183	18	148	14	97	153	260	15	801	30	738	9
Solyc06g051280.1.1	0	0	0	0	0	0	0	0	0	0	0	0	0
Solyc06g051300.2.1	0	0	0	0	0	0	0	0	0	0	0	0	0
Solyc06g051310.2.1	28	291	44	202	40	490	365	666	108	665	158	608	19
Solyc06g051320.2.1	0	0	0	0	0	0	0	0	0	0	0	0	0
Solyc06g051350.2.1	3	4	0	0	0	2	21	10	0	3	0	8	0
Solyc06g051360.2.1	5	19	2	37	0	184	27	182	48	120	10	77	1

Solyc06g051380.1.1	1	3	0	0	0	4	3	4	0	4	1	2	0
Solyc06g051390.2.1	22	59	21	35	12	205	161	162	19	133	36	136	5
Solyc06g051400.2.1	3	108	20	36	6	40	32	35	7	19	25	31	0
Solyc06g051410.2.1	4	25	1	20	0	11	21	36	0	31	10	24	0
Solyc06g051420.2.1	0	0	0	0	0	0	0	0	0	0	0	0	0
Solyc06g051430.2.1	6	44	2	26	12	69	101	98	17	95	18	76	9
Solyc06g051440.2.1	4	28	6	29	0	61	12	34	4	40	6	26	0
Solyc06g051450.1.1	1	6	0	1	0	18	15	10	0	31	2	3	0
Solyc06g051460.2.1	2	10	1	12	0	2	9	6	1	7	7	6	0
Solyc06g051500.2.1	0	0	0	0	0	0	0	0	0	0	0	0	0
Solyc06g051510.2.1	5	14	0	21	0	18	29	25	4	43	16	45	1
Solyc06g051520.2.1	3	12	5	6	2	26	32	19	9	19	32	26	0
Solyc06g051540.2.1	6	9	5	3	12	40	47	25	5	26	16	23	0
Solyc06g051570.2.1	4	7	1	3	0	10	16	18	1	9	0	8	1
Solyc06g051620.2.1	6	14	8	0	3	126	78	211	28	92	8	72	2
Solyc06g051630.2.1	4	3	0	0	0	12	16	11	0	12	1	16	3
Solyc06g051650.2.1	2	70	2	42	0	108	25	91	4	100	13	58	0
Solyc06g051660.1.1	3	9	0	0	4	13	22	18	1	47	0	21	0
Solyc06g051680.1.1	0	0	0	0	0	0	0	0	0	0	0	0	0
Solyc06g051720.2.1	5	38	2	13	1	89	25	81	11	39	10	19	3
Solyc06g051730.2.1	1	2	1	0	1	19	9	11	7	16	0	13	0
Solyc06g051740.1.1	1	3	0	0	0	1	9	3	0	6	1	2	0
Solyc06g051750.2.1	8	66	12	21	0	131	38	266	1	74	26	56	0
Solyc06g051760.2.1	3	5	0	1	0	11	11	22	0	27	6	16	0
Solyc06g051780.2.1	4	8	2	7	2	15	13	16	1	13	13	6	0
Solyc06g051790.2.1	8	46	4	31	0	87	88	110	12	89	23	53	0
Solyc06g051800.2.1	3	9	1	6	1	19	46	26	26	19	133	9	0
Solyc06g051810.2.1	10	235	10	96	5	388	92	418	25	350	50	269	3
Solyc06g051820.2.1	0	0	0	0	0	0	0	0	0	0	0	0	0
Solyc06g051830.1.1	0	0	0	0	0	0	0	0	0	0	0	0	0
Solyc06g051840.1.1	0	0	0	0	0	0	0	0	0	0	0	0	0
Solyc06g051850.1.1	0	0	0	0	0	0	0	0	0	0	0	0	0
Solyc06g051860.1.1	0	0	0	0	0	0	0	0	0	0	0	0	0
Solyc06g051900.2.1	4	21	1	17	1	16	27	14	0	15	6	16	2
Solyc06g051920.2.1	2	14	0	10	1	16	6	32	3	24	2	24	0
Solyc06g051930.2.1	13	246	75	210	10	1205	372	355	15	134	88	221	0
Solyc06g051940.2.1	10	91	2	9	0	897	129	267	13	133	49	64	0
Solyc06g051950.2.1	0	0	0	0	0	0	0	0	0	0	0	0	0
Solyc06g051960.2.1	3	146	7	56	6	3	12	3	1	13	4	9	0
Solyc06g051970.2.1	0	0	0	0	0	0	0	0	0	0	0	0	0
Solyc06g051980.2.1	6	10	2	13	5	33	34	30	7	68	18	47	5

Solyc06g051990.2.1	0	0	0	0	0	0	0	0	0	0	0	0	0
Solyc06g052000.1.1	0	0	0	0	0	0	0	0	0	0	0	0	0
Solyc06g052010.1.1	0	0	0	0	0	0	0	0	0	0	0	0	0
Solyc06g052020.2.1	0	0	0	0	0	0	0	0	0	0	0	0	0
Solyc06g052030.2.1	16	324	45	470	23	999	354	995	62	501	108	551	19
Solyc06g052050.2.1	0	0	0	0	0	0	0	0	0	0	0	0	0
Solyc06g052060.2.1	0	0	0	0	0	0	0	0	0	0	0	0	0
Solyc06g052070.2.1	1	4	0	14	0	0	0	8	0	2	0	3	0
Solyc06g052080.2.1	4	23	0	22	1	49	38	51	3	39	15	31	0
Solyc06g053140.2.1	10	655	82	375	0	80	105	90	0	135	109	186	0
Solyc06g053150.1.1	1	2	1	0	0	10	2	9	2	0	0	1	0
Solyc06g053160.2.1	8	97	10	76	8	239	146	263	24	126	43	107	7
Solyc06g053170.2.1	1	13	1	8	5	41	52	22	32	6	3	2	2
Solyc06g053180.2.1	1	17	11	2	1	27	27	27	18	34	8	5	0
Solyc06g053190.2.1	5	12	1	3	0	41	35	49	2	32	5	30	0
Solyc06g053200.2.1	7	88	6	54	1	194	131	256	5	217	41	150	4
Solyc06g053210.2.1	3	1	2	0	0	49	59	36	6	9	0	4	0
Solyc06g053220.2.1	8	34	2	18	1	326	212	241	15	63	8	25	0
Solyc06g053230.2.1	12	58	2	66	3	170	53	192	4	197	35	129	1
Solyc06g053240.2.1	0	0	0	0	0	0	0	0	0	0	0	0	0
Solyc06g053260.1.1	3	161	13	20	43	287	29	23	38	1	4	1	0
Solyc06g053290.1.1	1	10	2	4	0	0	9	5	2	9	3	0	0
Solyc06g053300.2.1	5	51	5	22	0	53	32	63	1	68	10	70	0
Solyc06g053310.2.1	6	204	5	104	2	250	83	299	16	122	30	91	1
Solyc06g053320.2.1	3	16	4	24	1	22	21	38	3	45	8	30	0
Solyc06g053330.2.1	6	19	3	11	4	101	24	99	12	77	16	56	0
Solyc06g053340.2.1	9	15	1	32	0	57	24	96	5	49	47	38	1
Solyc06g053350.2.1	4	8	4	3	1	46	25	20	6	25	6	17	0
Solyc06g053360.2.1	3	12	0	13	11	37	60	17	25	6	75	2	0
Solyc06g053370.1.1	2	2	0	0	0	1	0	1	0	4	3	5	0
Solyc06g053380.2.1	1	7	0	0	3	4	20	1	0	5	0	0	0
Solyc06g053400.2.1	9	47	3	40	0	113	61	222	3	84	30	92	1
Solyc06g053420.2.1	0	0	0	0	0	0	0	0	0	0	0	0	0
Solyc06g053430.2.1	3	19	0	2	1	29	10	19	0	20	3	19	0
Solyc06g053440.2.1	0	0	0	0	0	0	0	0	0	0	0	0	0
Solyc06g053450.2.1	21	543	43	379	26	856	735	1158	97	911	195	633	11
Solyc06g053460.1.1	3	10	5	20	2	11	11	30	4	40	16	37	3
Solyc06g053470.2.1	2	32	2	25	0	78	54	95	16	55	10	35	0
Solyc06g053480.2.1	8	421	35	244	14	181	94	219	24	389	75	374	5
Solyc06g053490.2.1	1	1	0	1	0	4	0	3	1	0	7	1	0
Solyc06g053500.2.1	0	0	0	0	0	0	0	0	0	0	0	0	0

Solyc06g053510.2.1	0	0	0	0	0	0	0	0	0	0	0	0	0
Solyc06g053530.2.1	4	47	4	16	4	13	21	84	8	77	14	36	2
Solyc06g053550.2.1	0	0	0	0	0	0	0	0	0	0	0	0	0
Solyc06g053560.2.1	1	12	0	1	0	8	9	23	1	10	0	0	0
Solyc06g053570.2.1	10	32	10	35	1	68	97	144	22	49	13	58	2
Solyc06g053580.2.1	2	7	1	2	0	7	1	20	2	9	4	7	0
Solyc06g053590.2.1	5	86	12	73	2	139	114	255	29	97	25	81	1
Solyc06g053600.2.1	12	215	34	72	0	60	101	87	3	68	13	97	0
Solyc06g053610.2.1	2	14	2	1	0	27	1	4	0	4	4	8	0
Solyc06g053620.2.1	2	19	2	15	0	0	0	3	2	7	15	0	0
Solyc06g053630.2.1	5	141	13	77	3	136	52	108	4	94	16	115	0
Solyc06g053640.1.1	0	0	0	0	0	0	0	0	0	0	0	0	0
Solyc06g053650.1.1	0	0	0	0	0	0	0	0	0	0	0	0	0
Solyc06g053660.2.1	2	4	2	0	0	9	15	5	0	21	4	10	0
Solyc06g053670.1.1	5	106	5	76	6	203	127	235	37	164	15	36	6
Solyc06g053700.1.1	0	0	0	0	0	0	0	0	0	0	0	0	0
Solyc06g053710.2.1	18	67	7	59	10	1584	418	694	37	910	455	315	9
Solyc06g053730.1.1	1	8	0	6	4	9	6	0	2	3	0	0	0
Solyc06g053740.2.1	10	46	2	15	1	247	52	126	9	165	25	166	5
Solyc06g053750.2.1	18	30	4	48	6	93	120	173	14	159	38	141	6
Solyc06g053760.2.1	0	0	0	0	0	0	0	0	0	0	0	0	0
Solyc06g053770.2.1	0	0	0	0	0	0	0	0	0	0	0	0	0
Solyc06g053780.2.1	6	21	1	33	0	22	37	55	0	18	4	20	0
Solyc06g053790.2.1	12	231	30	146	8	689	398	538	57	653	317	353	8
Solyc06g053800.1.1	9	63	2	44	4	159	65	222	17	111	46	161	2
Solyc06g053810.2.1	9	67	14	36	0	26	95	42	1	26	16	16	0
Solyc06g053820.2.1	4	140	6	65	8	342	92	235	7	395	32	375	0
Solyc06g053830.2.1	2	17	6	9	0	14	25	8	5	1	2	5	0
Solyc06g053840.2.1	4	83	2	41	0	44	12	28	0	155	5	117	0
Solyc06g053850.2.1	1	0	0	0	0	9	4	3	0	0	0	2	0
Solyc06g053860.2.1	2	7	2	13	0	25	11	23	2	16	4	32	2
Solyc06g053870.2.1	0	0	0	0	0	0	0	0	0	0	0	0	0
Solyc06g053900.2.1	0	0	0	0	0	0	0	0	0	0	0	0	0
Solyc06g053910.2.1	12	52	10	69	4	116	130	134	19	104	66	137	1
Solyc06g053920.2.1	9	23	7	16	1	44	44	43	16	32	14	17	0
Solyc06g053930.2.1	0	0	0	0	0	0	0	0	0	0	0	0	0
Solyc06g053950.1.1	0	0	0	0	0	0	0	0	0	0	0	0	0
Solyc06g053960.2.1	1	4	0	5	0	30	0	36	0	444	3	262	0
Solyc06g053980.2.1	13	435	6	231	0	149	114	183	14	97	33	98	0
Solyc06g054010.2.1	1	5	1	6	0	6	3	16	0	5	0	0	0
Solyc06g054020.2.1	13	89	6	65	2	179	180	220	4	454	43	256	0

Solyc06g054030.2.1	0	0	0	0	0	0	0	0	0	0	0	0	0
Solyc06g054050.1.1	0	0	0	0	0	0	0	0	0	0	0	0	0
Solyc06g054060.1.1	0	0	0	0	0	0	0	0	0	0	0	0	0
Solyc06g054070.2.1	0	0	0	0	0	0	0	0	0	0	0	0	0
Solyc06g054080.2.1	7	49	14	27	4	63	75	73	10	108	12	69	1
Solyc06g054090.1.1	0	0	0	0	0	0	0	0	0	0	0	0	0
Solyc06g054120.1.1	0	0	0	0	0	0	0	0	0	0	0	0	0
Solyc06g054130.1.1	0	0	0	0	0	0	0	0	0	0	0	0	0
Solyc06g054140.2.1	0	0	0	0	0	0	0	0	0	0	0	0	0
Solyc06g054150.1.1	0	0	0	0	0	0	0	0	0	0	0	0	0
Solyc06g054220.2.1	0	0	0	0	0	0	0	0	0	0	0	0	0
Solyc06g054240.2.1	3	13	2	8	0	4	10	18	0	12	12	5	0
Solyc06g054250.2.1	5	23	2	34	3	26	31	73	1	73	9	45	0
Solyc06g054260.1.1	9	8908	1277	6157	66	745	3678	1337	133	1335	1067	1028	35
Solyc06g054270.2.1	2	36	2	25	0	1	1	7	3	0	0	3	0
Solyc06g054310.1.1	0	0	0	0	0	0	0	0	0	0	0	0	0
Solyc06g054320.1.1	0	0	0	0	0	0	0	0	0	0	0	0	0
Solyc06g054330.2.1	1	4	0	1	5	42	16	15	6	14	6	15	0
Solyc06g054340.1.1	0	0	0	0	0	0	0	0	0	0	0	0	0
Solyc06g054370.1.1	0	0	0	0	0	0	0	0	0	0	0	0	0
Solyc06g054380.2.1	1	3	1	0	0	11	8	4	4	7	0	11	0
Solyc06g054390.2.1	0	0	0	0	0	0	0	0	0	0	0	0	0
Solyc06g054400.2.1	10	18	1	11	4	42	59	72	14	50	27	24	0
Solyc06g054410.2.1	1	10	0	3	0	12	1	13	1	14	0	1	2
Solyc06g054420.2.1	6	155	11	107	16	174	131	206	14	283	102	249	7
Solyc06g054430.1.1	1	2	1	2	0	3	13	5	2	11	6	2	2
Solyc06g054440.2.1	4	18	16	13	0	23	34	45	6	24	6	11	0
Solyc06g054450.1.1	0	0	0	0	0	0	0	0	0	0	0	0	0
Solyc06g054460.1.1	0	0	0	0	0	0	0	0	0	0	0	0	0
Solyc06g054470.1.1	1	2	2	0	0	6	4	0	0	13	0	10	2
Solyc06g054480.2.1	1	1	0	3	1	0	11	2	17	4	19	7	2
Solyc06g054490.2.1	4	39	4	13	0	40	17	77	5	87	7	86	2
Solyc06g054510.2.1	6	404	26	236	4	700	235	599	53	2570	91	2123	11
Solyc06g054530.2.1	1	3	2	0	0	4	7	10	0	3	2	10	0
Solyc06g054540.2.1	3	7	0	8	0	12	82	23	4	2	0	1	1
Solyc06g054550.2.1	0	0	0	0	0	0	0	0	0	0	0	0	0
Solyc06g054560.2.1	12	28	9	39	8	65	47	83	29	26	38	27	8
Solyc06g054570.1.1	0	0	0	0	0	0	0	0	0	0	0	0	0
Solyc06g054580.2.1	1	1	0	0	0	8	6	7	0	1	0	0	0
Solyc06g054590.2.1	0	0	0	0	0	0	0	0	0	0	0	0	0
Solyc06g054600.2.1	1	1	0	0	0	8	1	7	0	4	2	6	0

Solyc06g054610.1.1	0	0	0	0	0	0	0	0	0	0	0	0	0
Solyc06g054620.2.1	4	9	3	6	0	32	9	23	4	5	3	13	0
Solyc06g054630.1.1	0	0	0	0	0	0	0	0	0	0	0	0	0
Solyc06g054640.1.1	0	0	0	0	0	0	0	0	0	0	0	0	0
Solyc06g054650.2.1	7	37	1	22	0	88	21	94	6	109	15	90	2
Solyc06g054660.1.1	0	0	0	0	0	0	0	0	0	0	0	0	0
Solyc06g054670.1.1	0	0	0	0	0	0	0	0	0	0	0	0	0
Solyc06g054690.2.1	9	126	10	79	14	101	42	127	8	176	19	105	0
Solyc06g059710.2.1	0	0	0	0	0	0	0	0	0	0	0	0	0
Solyc06g059720.2.1	1	0	0	0	0	0	1	0	4	3	119	0	0
Solyc06g059730.1.1	0	0	0	0	0	0	0	0	0	0	0	0	0
Solyc06g059740.2.1	10	60	0	25	0	156	12	121	0	10466	236	3005	1
Solyc06g059750.2.1	14	78	6	89	6	136	75	212	13	148	23	104	1
Solyc06g059760.2.1	10	104	8	72	5	133	74	239	18	230	13	147	0
Solyc06g059800.2.1	3	50	5	33	10	6	1	20	4	32	10	45	11
Solyc06g059810.1.1	0	0	0	0	0	0	0	0	0	0	0	0	0
Solyc06g059840.2.1	8	8	1	0	17	26	40	9	72	22	26	2	9
Solyc06g059850.1.1	0	0	0	0	0	0	0	0	0	0	0	0	0
Solyc06g059860.2.1	7	45	5	23	6	478	95	311	40	12	12	14	0
Solyc06g059870.1.1	1	9	0	9	0	0	4	10	0	52	0	28	0
Solyc06g059960.2.1	11	33	8	20	3	46	40	58	6	58	36	62	3
Solyc06g059980.2.1	1	2	0	3	0	6	3	2	0	0	0	8	0
Solyc06g060010.2.1	0	0	0	0	0	0	0	0	0	0	0	0	0
Solyc06g060050.1.1	0	0	0	0	0	0	0	0	0	0	0	0	0
Solyc06g060060.2.1	4	23	3	17	0	8	15	17	0	9	4	15	0
Solyc06g060080.2.1	1	10	1	0	0	24	37	16	1	9	5	15	0
Solyc06g060090.2.1	7	53	7	44	2	117	24	66	8	76	7	64	0
Solyc06g060100.2.1	0	0	0	0	0	0	0	0	0	0	0	0	0
Solyc06g060110.2.1	12	568	121	448	12	1173	337	1288	1	61	24	208	2
Solyc06g060120.2.1	11	57	4	30	0	130	92	208	3	93	25	48	0
Solyc06g060130.2.1	9	30	4	9	3	100	55	128	7	165	16	126	1
Solyc06g060140.2.1	0	0	0	0	0	0	0	0	0	0	0	0	0
Solyc06g060150.2.1	3	23	4	2	0	35	11	64	1	92	13	36	1
Solyc06g060160.1.1	0	0	0	0	0	0	0	0	0	0	0	0	0
Solyc06g060170.2.1	4	6	5	4	0	46	11	20	1	2	8	2	0
Solyc06g060230.2.1	10	371	23	266	8	985	137	382	19	71	36	62	0
Solyc06g060250.2.1	7	92	16	105	5	250	207	318	11	228	14	191	0
Solyc06g060260.2.1	7	142	15	80	1	327	91	404	12	329	51	365	4
Solyc06g060270.2.1	0	0	0	0	0	0	0	0	0	0	0	0	0
Solyc06g060280.2.1	0	0	0	0	0	0	0	0	0	0	0	0	0
Solyc06g060290.2.1	7	227	29	147	9	1324	211	1019	39	442	118	345	11

Solyc06g060300.1.1	0	0	0	0	0	0	0	0	0	0	0	0	0
Solyc06g060310.2.1	6	106	17	72	0	8	5	14	2	5	18	24	0
Solyc06g060320.2.1	0	0	0	0	0	0	0	0	0	0	0	0	0
Solyc06g060340.2.1	7	2522	210	1459	5	11	40	37	4	102	260	229	0
Solyc06g060350.2.1	3	5	1	8	0	12	13	8	4	18	3	7	0
Solyc06g060360.2.1	0	0	0	0	0	0	0	0	0	0	0	0	0
Solyc06g060370.2.1	2	24	1	11	0	76	6	40	9	56	6	36	0
Solyc06g060380.2.1	2	13	2	14	0	11	4	18	0	6	5	6	0
Solyc06g060390.2.1	5	18	0	14	0	46	31	43	1	30	8	40	0
Solyc06g060400.2.1	4	122	6	79	3	197	57	291	18	529	84	364	0
Solyc06g060410.2.1	0	0	0	0	0	0	0	0	0	0	0	0	0
Solyc06g060420.2.1	12	46	4	37	1	106	106	92	12	112	36	70	0
Solyc06g060460.2.1	6	34	2	6	0	29	22	36	1	62	24	56	0
Solyc06g060480.1.1	0	0	0	0	0	0	0	0	0	0	0	0	0
Solyc06g060490.2.1	4	70	9	28	0	274	86	168	12	101	23	44	2
Solyc06g060510.1.1	1	8	0	0	0	0	9	1	1	3	0	8	0
Solyc06g060520.1.1	1	17	1	12	0	12	1	8	0	5	1	9	0
Solyc06g060530.1.1	0	0	0	0	0	0	0	0	0	0	0	0	0
Solyc06g060550.2.1	0	0	0	0	0	0	0	0	0	0	0	0	0
Solyc06g060560.2.1	0	0	0	0	0	0	0	0	0	0	0	0	0
Solyc06g060570.2.1	0	0	0	0	0	0	0	0	0	0	0	0	0
Solyc06g060590.2.1	3	19	1	5	0	8	28	24	0	0	0	0	0
Solyc06g060610.2.1	9	59	2	9	3	82	50	67	8	55	10	20	1
Solyc06g060620.2.1	15	435	54	551	1	2139	52	3379	0	157	13	74	0
Solyc06g060630.1.1	0	0	0	0	0	0	0	0	0	0	0	0	0
Solyc06g060640.1.1	0	0	0	0	0	0	0	0	0	0	0	0	0
Solyc06g060670.2.1	6	1	1	8	2	30	16	21	2	26	16	12	1
Solyc06g060680.1.1	5	40	3	19	0	27	14	30	9	5	0	6	0
Solyc06g060690.2.1	3	29	0	19	0	5	12	16	3	13	7	40	1
Solyc06g060700.1.1	5	11	2	3	6	3	27	17	6	3	17	2	0
Solyc06g060710.2.1	1	0	0	0	0	0	2	2	0	4	2	4	0
Solyc06g060720.2.1	5	85	1	62	4	124	33	239	16	109	19	93	2
Solyc06g060730.2.1	5	12	1	8	0	30	23	35	1	44	13	10	0
Solyc06g060740.1.1	0	0	0	0	0	0	0	0	0	0	0	0	0
Solyc06g060750.2.1	2	3	1	0	0	4	7	8	0	9	6	2	0
Solyc06g060760.2.1	3	48	0	7	3	23	31	32	15	1	1	0	0
Solyc06g060770.2.1	2	5	3	15	0	2	11	5	0	0	0	0	0
Solyc06g060780.2.1	1	4	0	1	0	13	11	19	5	14	1	16	0
Solyc06g060790.1.1	10	229	29	106	17	405	206	450	30	247	93	287	8
Solyc06g060800.2.1	3	12	5	12	1	14	19	41	2	17	3	5	0
Solyc06g060810.2.1	3	4	0	2	0	15	6	10	0	26	1	37	0

Solyc06g060820.1.1	2	0	0	2	0	6	6	3	1	0	0	1	0
Solyc06g060830.2.1	3	49	11	31	7	0	3	0	0	10	3	3	0
Solyc06g060840.1.1	2	0	0	0	0	16	37	19	0	1	12	0	0
Solyc06g060850.2.1	5	75	12	48	12	136	147	166	10	160	38	165	16
Solyc06g060860.1.1	0	0	0	0	0	0	0	0	0	0	0	0	0
Solyc06g060870.1.1	0	0	0	0	0	0	0	0	0	0	0	0	0
Solyc06g060880.2.1	2	17	2	9	0	2	7	7	0	1	2	3	0
Solyc06g060890.2.1	1	1	0	3	0	3	3	4	2	0	1	1	0
Solyc06g060910.1.1	11	1	1	0	1	346	137	188	46	36	8	15	0
Solyc06g060920.2.1	1	33	1	14	0	30	19	37	3	30	1	28	0
Solyc06g060940.1.1	5	19	1	7	0	56	36	40	11	23	7	38	0
Solyc06g060950.1.1	0	0	0	0	0	0	0	0	0	0	0	0	0
Solyc06g060960.1.1	9	17	1	26	0	25	37	55	1	42	20	9	1
Solyc06g060970.1.1	1	0	0	0	0	194	12	38	0	0	0	0	0
Solyc06g060990.2.1	8	23	3	26	3	31	15	45	5	71	15	31	0
Solyc06g061000.2.1	6	246	16	144	9	288	165	344	18	692	98	615	9
Solyc06g061010.2.1	1	0	0	0	0	23	6	2	0	1	0	2	1
Solyc06g061020.2.1	11	347	33	268	10	193	39	239	10	480	270	527	11
Solyc06g061030.2.1	19	93	9	63	18	322	89	359	32	88	41	65	6
Solyc06g061040.2.1	0	0	0	0	0	0	0	0	0	0	0	0	0
Solyc06g061050.2.1	0	0	0	0	0	0	0	0	0	0	0	0	0
Solyc06g061070.2.1	1	259	15	71	0	25	44	42	1	51	6	62	0
Solyc06g061080.2.1	0	0	0	0	0	0	0	0	0	0	0	0	0
Solyc06g061090.2.1	0	0	0	0	0	0	0	0	0	0	0	0	0
Solyc06g061100.2.1	1	0	0	0	0	7	6	0	24	0	0	0	0
Solyc06g061110.1.1	0	0	0	0	0	0	0	0	0	0	0	0	0
Solyc06g061120.2.1	8	33	3	35	3	47	44	61	7	107	7	126	2
Solyc06g061130.2.1	0	0	0	0	0	0	0	0	0	0	0	0	0
Solyc06g061140.2.1	1	3	0	0	0	0	5	6	2	3	0	2	0
Solyc06g061150.2.1	3	18	7	12	1	28	54	39	9	44	13	58	10
Solyc06g061170.2.1	2	10	4	1	0	25	16	73	16	43	0	19	0
Solyc06g061180.1.1	1	17	0	3	0	1	10	10	1	17	1	9	0
Solyc06g061190.1.1	3	14	7	0	0	22	39	14	4	7	5	12	0
Solyc06g061200.1.1	7	1826	65	1196	0	270	469	99	1	2528	237	2628	8
Solyc06g061230.2.1	5	167	48	139	102	711	715	1332	24	17	0	33	2
Solyc06g061240.2.1	3	32	3	56	4	124	24	111	2	65	11	52	0
Solyc06g061250.1.1	1	22	2	9	0	81	11	32	1	2	0	5	1
Solyc06g061280.2.1	12	10	6	8	5	440	352	154	357	0	15	0	7
Solyc06g062280.2.1	8	151	9	123	3	262	181	432	22	287	33	326	3
Solyc06g062290.1.1	0	0	0	0	0	0	0	0	0	0	0	0	0
Solyc06g062310.2.1	0	0	0	0	0	0	0	0	0	0	0	0	0

Solyc06g062320.1.1	1	0	0	0	0	2	3	1	0	3	0	3	0
Solyc06g062330.1.1	0	0	0	0	0	0	0	0	0	0	0	0	0
Solyc06g062350.2.1	3	7	0	3	0	20	3	19	1	7	4	8	0
Solyc06g062360.2.1	1	3	1	1	0	2	6	12	0	19	5	21	0
Solyc06g062370.2.1	7	99	30	49	1	6	42	70	0	4	108	4	0
Solyc06g062380.2.1	0	0	0	0	0	0	0	0	0	0	0	0	0
Solyc06g062400.1.1	3	3	1	0	0	18	10	9	2	8	6	9	0
Solyc06g062420.2.1	0	0	0	0	0	0	0	0	0	0	0	0	0
Solyc06g062430.2.1	5	1	1	3	0	86	77	64	5	7	8	2	0
Solyc06g062440.2.1	2	8	1	2	0	7	20	10	0	12	0	6	0
Solyc06g062460.2.1	1	3	0	0	0	50	4	9	0	0	0	0	0
Solyc06g062480.2.1	5	7	1	0	0	11	8	15	1	14	11	20	0
Solyc06g062490.2.1	0	0	0	0	0	0	0	0	0	0	0	0	0
Solyc06g062500.2.1	3	9	0	7	2	22	3	9	0	99	5	31	1
Solyc06g062510.2.1	5	277	20	145	5	373	171	380	37	582	87	290	10
Solyc06g062520.1.1	0	0	0	0	0	0	0	0	0	0	0	0	0
Solyc06g062530.2.1	4	28	2	32	0	51	27	103	11	14	4	16	2
Solyc06g062580.2.1	3	28	2	30	8	13	9	23	0	13	1	4	1
Solyc06g062590.2.1	8	91	11	102	12	182	120	283	46	258	41	249	7
Solyc06g062600.2.1	10	15	11	2	1	75	153	23	89	37	0	2	0
Solyc06g062610.1.1	0	0	0	0	0	0	0	0	0	0	0	0	0
Solyc06g062620.2.1	0	0	0	0	0	0	0	0	0	0	0	0	0
Solyc06g062630.2.1	0	0	0	0	0	0	0	0	0	0	0	0	0
Solyc06g062660.2.1	7	22	6	31	0	112	64	94	1	75	12	100	1
Solyc06g062670.2.1	6	44	17	38	0	149	65	174	0	52	15	46	0
Solyc06g062680.1.1	0	0	0	0	0	0	0	0	0	0	0	0	0
Solyc06g062690.2.1	1	2	0	0	0	0	0	4	0	6	5	15	0
Solyc06g062700.2.1	1	0	0	0	0	4	3	1	0	2	0	5	2
Solyc06g062760.2.1	3	8	0	2	0	35	16	36	1	18	7	7	0
Solyc06g062770.2.1	0	0	0	0	0	0	0	0	0	0	0	0	0
Solyc06g062780.2.1	9	40	7	23	8	95	61	89	82	95	19	48	0
Solyc06g062790.2.1	7	56	13	42	1	221	180	182	27	81	42	83	1
Solyc06g062800.2.1	0	0	0	0	0	0	0	0	0	0	0	0	0
Solyc06g062810.1.1	5	29	4	21	8	46	36	79	11	99	23	133	1
Solyc06g062820.2.1	0	0	0	0	0	0	0	0	0	0	0	0	0
Solyc06g062840.2.1	9	26	5	15	2	8	110	47	9	134	53	113	3
Solyc06g062850.2.1	3	16	0	9	0	34	6	49	3	22	7	18	0
Solyc06g062860.2.1	9	31	8	37	5	51	85	60	12	59	33	53	0
Solyc06g062880.1.1	0	0	0	0	0	0	0	0	0	0	0	0	0
Solyc06g062890.2.1	0	0	0	0	0	0	0	0	0	0	0	0	0
Solyc06g062900.2.1	1	16	0	6	0	14	6	8	3	12	2	18	0

Solyc06g062910.2.1	0	0	0	0	0	0	0	0	0	0	0	0	0
Solyc06g062920.2.1	3	43	8	46	0	0	10	2	0	9	0	75	5
Solyc06g062940.2.1	3	227	9	101	1	108	49	198	19	170	15	81	1
Solyc06g062950.1.1	33	1350	84	844	24	1141	1592	1639	58	1150	200	731	24
Solyc06g062960.1.1	5	41	3	21	0	56	40	65	4	4	4	5	0
Solyc06g062980.2.1	0	0	0	0	0	0	0	0	0	0	0	0	0
Solyc06g062990.1.1	4	29	6	17	0	27	9	27	1	88	3	60	0
Solyc06g063000.2.1	7	105	17	103	7	159	75	233	18	433	91	301	4
Solyc06g063010.2.1	9	34	2	47	3	277	74	279	11	109	40	92	3
Solyc06g063020.2.1	2	3	0	0	0	2	4	5	0	2	0	2	0
Solyc06g063030.2.1	0	0	0	0	0	0	0	0	0	0	0	0	0
Solyc06g063040.1.1	1	0	0	0	0	0	3	1	1	9	2	5	0
Solyc06g063050.2.1	0	0	0	0	0	0	0	0	0	0	0	0	0
Solyc06g063060.2.1	0	0	0	0	0	0	0	0	0	0	0	0	0
Solyc06g063070.2.1	20	2949	574	2406	66	4442	2332	5421	215	7598	2687	5634	134
Solyc06g063090.2.1	7	18	3	23	0	188	62	150	33	32	19	53	3
Solyc06g063100.2.1	4	19	4	23	0	82	58	60	4	72	10	56	4
Solyc06g063110.1.1	0	0	0	0	0	0	0	0	0	0	0	0	0
Solyc06g063120.2.1	0	0	0	0	0	0	0	0	0	0	0	0	0
Solyc06g063130.2.1	0	0	0	0	0	0	0	0	0	0	0	0	0
Solyc06g063140.2.1	3	165	7	78	6	206	98	393	30	370	31	174	8
Solyc06g063150.2.1	0	0	0	0	0	0	0	0	0	0	0	0	0
Solyc06g063160.1.1	0	0	0	0	0	0	0	0	0	0	0	0	0
Solyc06g063170.2.1	1	1	3	6	0	7	7	11	9	16	1	20	0
Solyc06g063180.1.1	0	0	0	0	0	0	0	0	0	0	0	0	0
Solyc06g063190.2.1	2	11	2	3	0	8	3	3	0	0	0	0	0
Solyc06g063200.1.1	0	0	0	0	0	0	0	0	0	0	0	0	0
Solyc06g063210.2.1	0	0	0	0	0	0	0	0	0	0	0	0	0
Solyc06g063220.2.1	1	36	7	14	0	19	14	26	2	31	16	58	0
Solyc06g063230.2.1	4	70	2	40	0	43	30	58	0	116	4	84	1
Solyc06g063240.2.1	22	803	56	388	61	366	173	535	42	4102	281	2728	20
Solyc06g063250.2.1	5	44	7	45	2	107	29	101	13	58	14	54	0
Solyc06g063260.2.1	2	7	0	1	0	6	12	9	1	0	1	0	0
Solyc06g063270.2.1	0	0	0	0	0	0	0	0	0	0	0	0	0
Solyc06g063280.1.1	0	0	0	0	0	0	0	0	0	0	0	0	0
Solyc06g063290.2.1	4	13	2	6	2	21	22	64	0	36	3	23	0
Solyc06g063300.2.1	8	26	2	20	2	144	221	71	9	124	17	40	1
Solyc06g063310.1.1	2	184	4	141	0	164	58	237	17	151	12	199	6
Solyc06g063320.2.1	9	60	8	42	5	62	62	96	11	163	20	138	0
Solyc06g063330.2.1	0	0	0	0	0	0	0	0	0	0	0	0	0
Solyc06g063340.2.1	1	1	0	0	0	4	2	0	0	2	5	7	0

Solyc06g063360.2.1	0	0	0	0	0	0	0	0	0	0	0	0	0
Solyc06g063370.2.1	5	7230	878	2859	14	71	155	72	11	778	505	727	23
Solyc06g063410.2.1	3	19	6	6	0	66	29	47	2	15	3	18	2
Solyc06g063420.2.1	15	18	4	34	4	102	99	135	6	87	15	77	0
Solyc06g064430.1.1	1	2	0	0	0	0	2	0	0	15	2	8	0
Solyc06g064440.2.1	0	0	0	0	0	0	0	0	0	0	0	0	0
Solyc06g064450.2.1	5	82	9	68	7	157	84	92	17	24	21	20	2
Solyc06g064460.2.1	2	26	3	4	0	2	17	15	1	58	2	54	0
Solyc06g064470.2.1	3	111	6	37	0	331	49	316	25	547	40	330	2
Solyc06g064490.2.1	2	3	1	3	5	1	24	6	3	19	21	17	2
Solyc06g064500.2.1	0	0	0	0	0	0	0	0	0	0	0	0	0
Solyc06g064510.2.1	0	0	0	0	0	0	0	0	0	0	0	0	0
Solyc06g064550.2.1	6	68	121	50	5	4	3	1	0	36	37	57	2
Solyc06g064560.2.1	0	0	0	0	0	0	0	0	0	0	0	0	0
Solyc06g064570.2.1	2	19	3	18	1	6	6	13	0	10	13	3	0
Solyc06g064580.1.1	0	0	0	0	0	0	0	0	0	0	0	0	0
Solyc06g064590.1.1	0	0	0	0	0	0	0	0	0	0	0	0	0
Solyc06g064600.1.1	0	0	0	0	0	0	0	0	0	0	0	0	0
Solyc06g064610.1.1	0	0	0	0	0	0	0	0	0	0	0	0	0
Solyc06g064630.2.1	3	120	2	62	2	188	28	161	3	376	40	291	5
Solyc06g064640.2.1	1	6	0	0	0	3	1	9	0	19	0	3	0
Solyc06g064650.2.1	0	0	0	0	0	0	0	0	0	0	0	0	0
Solyc06g064660.2.1	0	0	0	0	0	0	0	0	0	0	0	0	0
Solyc06g064680.1.1	0	0	0	0	0	0	0	0	0	0	0	0	0
Solyc06g064750.1.1	0	0	0	0	0	0	0	0	0	0	0	0	0
Solyc06g064810.2.1	1	13	0	15	0	3	14	12	0	6	1	20	0
Solyc06g064820.2.1	0	0	0	0	0	0	0	0	0	0	0	0	0
Solyc06g064830.2.1	5	45	2	24	0	75	35	176	7	113	18	91	1
Solyc06g064840.2.1	0	0	0	0	0	0	0	0	0	0	0	0	0
Solyc06g064850.1.1	0	0	0	0	0	0	0	0	0	0	0	0	0
Solyc06g064860.1.1	0	0	0	0	0	0	0	0	0	0	0	0	0
Solyc06g064870.2.1	10	47	0	15	0	43	50	46	17	18	12	20	4
Solyc06g064880.2.1	0	0	0	0	0	0	0	0	0	0	0	0	0
Solyc06g064890.2.1	3	43	4	35	3	52	33	79	3	74	9	43	0
Solyc06g064910.2.1	21	168	17	104	17	239	200	340	28	379	71	272	7
Solyc06g064940.2.1	17	407	73	386	25	186	192	375	94	538	113	413	15
Solyc06g065010.2.1	0	0	0	0	0	0	0	0	0	0	0	0	0
Solyc06g065020.2.1	16	543	80	489	46	1902	1736	1387	37	1713	114	1149	14
Solyc06g065030.2.1	1	6	0	1	0	8	5	13	0	6	1	9	0
Solyc06g065040.2.1	0	0	0	0	0	0	0	0	0	0	0	0	0
Solyc06g065050.1.1	0	0	0	0	0	0	0	0	0	0	0	0	0

Solyc06g065060.1.1	0	0	0	0	0	0	0	0	0	0	0	0	0
Solyc06g065100.2.1	5	9	1	10	7	15	52	16	4	46	15	18	1
Solyc06g065170.2.1	14	114	19	70	2	182	134	260	38	121	62	112	7
Solyc06g065180.2.1	3	33	48	23	25	96	365	48	72	108	144	42	35
Solyc06g065190.1.1	0	0	0	0	0	0	0	0	0	0	0	0	0
Solyc06g065200.2.1	0	0	0	0	0	0	0	0	0	0	0	0	0
Solyc06g065210.2.1	3	37	9	30	21	24	62	97	18	94	6	89	0
Solyc06g065230.2.1	1	0	1	0	2	0	12	1	9	5	4	2	9
Solyc06g065240.2.1	6	51	2	37	4	451	128	136	18	214	91	197	5
Solyc06g065250.1.1	6	41	4	19	12	63	44	100	25	80	16	78	1
Solyc06g065260.2.1	9	55	1	47	0	64	72	190	3	146	25	88	0
Solyc06g065270.2.1	11	123	57	65	0	226	589	274	24	380	43	402	7
Solyc06g065290.2.1	0	0	0	0	0	0	0	0	0	0	0	0	0
Solyc06g065300.2.1	0	0	0	0	0	0	0	0	0	0	0	0	0
Solyc06g065310.2.1	0	0	0	0	0	0	0	0	0	0	0	0	0
Solyc06g065320.2.1	9	54	3	28	1	89	45	129	13	96	17	107	0
Solyc06g065330.2.1	4	45	2	19	1	49	22	48	6	113	20	78	5
Solyc06g065340.1.1	0	0	0	0	0	0	0	0	0	0	0	0	0
Solyc06g065350.2.1	0	0	0	0	0	0	0	0	0	0	0	0	0
Solyc06g065360.2.1	3	8	1	3	0	16	10	9	8	7	12	9	0
Solyc06g065370.2.1	4	148	35	74	10	340	408	424	93	302	128	220	27
Solyc06g065380.2.1	0	0	0	0	0	0	0	0	0	0	0	0	0
Solyc06g065390.2.1	3	47	3	37	0	21	30	40	0	42	3	43	0
Solyc06g065400.2.1	1	6	1	0	0	7	3	6	1	18	15	15	2
Solyc06g065410.2.1	0	0	0	0	0	0	0	0	0	0	0	0	0
Solyc06g065440.1.1	4	79	3	21	2	9	15	26	0	172	5	103	0
Solyc06g065450.2.1	0	0	0	0	0	0	0	0	0	0	0	0	0
Solyc06g065460.2.1	9	42	3	33	12	111	76	107	15	111	13	71	5
Solyc06g065470.2.1	4	61	2	18	0	157	21	120	6	84	14	48	0
Solyc06g065480.2.1	1	2	2	8	0	3	3	1	0	15	1	17	0
Solyc06g065490.2.1	3	22	7	3	0	1	20	2	0	8	3	9	2
Solyc06g065500.2.1	6	35	10	37	3	163	101	90	12	64	29	88	1
Solyc06g065510.2.1	2	11	1	8	1	0	11	16	0	18	4	10	0
Solyc06g065520.2.1	9	60	1	62	3	176	35	302	2	161	18	135	0
Solyc06g065530.2.1	0	0	0	0	0	0	0	0	0	0	0	0	0
Solyc06g065540.2.1	27	248	21	88	10	318	121	244	25	237	47	158	11
Solyc06g065560.1.1	4	63	24	21	0	111	44	81	37	35	29	27	0
Solyc06g065570.2.1	3	41	2	23	0	192	87	198	6	56	204	36	2
Solyc06g065580.2.1	14	200	11	77	27	2116	141	789	21	1726	41	184	2
Solyc06g065590.2.1	6	169	12	128	9	514	92	417	42	962	70	305	6
Solyc06g065610.2.1	3	9	1	0	0	14	9	23	2	29	8	20	0

Solyc06g065620.1.1	0	0	0	0	0	0	0	0	0	0	0	0	0
Solyc06g065630.2.1	0	0	0	0	0	0	0	0	0	0	0	0	0
Solyc06g065640.2.1	19	170	25	125	20	215	226	270	42	273	56	228	0
Solyc06g065650.2.1	5	45	4	23	1	6	12	30	0	108	16	74	0
Solyc06g065660.1.1	0	0	0	0	0	0	0	0	0	0	0	0	0
Solyc06g065670.2.1	15	25	10	7	21	4	37	3	21	153	20	58	13
Solyc06g065680.2.1	0	0	0	0	0	0	0	0	0	0	0	0	0
Solyc06g065690.2.1	1	6	4	4	1	0	2	10	0	8	3	4	0
Solyc06g065700.2.1	8	34	15	21	4	87	90	77	20	61	42	62	19
Solyc06g065710.2.1	29	240	36	214	22	723	302	792	106	418	151	309	6
Solyc06g065720.1.1	15	103	11	62	15	257	81	215	32	119	43	107	4
Solyc06g065730.2.1	29	110	13	105	8	201	150	387	26	174	45	200	1
Solyc06g065740.2.1	0	0	0	0	0	0	0	0	0	0	0	0	0
Solyc06g065790.1.1	0	0	0	0	0	0	0	0	0	0	0	0	0
Solyc06g065800.2.1	1	10	0	0	0	2	4	15	0	14	1	2	0
Solyc06g065820.2.1	0	0	0	0	0	0	0	0	0	0	0	0	0
Solyc06g065840.2.1	17	62	8	41	5	205	100	241	26	143	39	136	10
Solyc06g065900.2.1	1	15	1	5	3	2	3	1	0	6	0	5	0
Solyc06g065910.2.1	0	0	0	0	0	0	0	0	0	0	0	0	0
Solyc06g065920.2.1	2	10	6	13	1	1	46	13	19	21	9	38	4
Solyc06g065940.2.1	1	1	0	2	0	0	1	1	0	7	3	1	0
Solyc06g065950.2.1	7	233	20	186	23	646	513	454	63	282	74	164	3
Solyc06g065960.1.1	0	0	0	0	0	0	0	0	0	0	0	0	0
Solyc06g065970.1.1	1	67	2	9	0	0	3	1	0	7	3	0	0
Solyc06g065980.2.1	2	52	1	20	0	60	41	102	4	73	6	27	1
Solyc06g065990.1.1	5	521	116	289	3	53	69	56	2	22	44	40	4
Solyc06g066000.1.1	5	1304	161	855	18	238	279	421	11	247	44	247	7
Solyc06g066010.2.1	11	41	3	40	1	417	1019	389	92	18	9	24	0
Solyc06g066020.2.1	1	3	0	0	0	1	9	1	3	0	3	0	0
Solyc06g066030.2.1	14	294	44	207	26	317	265	534	41	958	171	965	9
Solyc06g066050.2.1	0	0	0	0	0	0	0	0	0	0	0	0	0
Solyc06g066060.2.1	5	96	14	37	13	378	130	331	29	284	64	180	3
Solyc06g066070.1.1	0	0	0	0	0	0	0	0	0	0	0	0	0
Solyc06g066080.1.1	0	0	0	0	0	0	0	0	0	0	0	0	0
Solyc06g066090.2.1	3	7	1	5	2	54	20	19	2	5	9	10	0
Solyc06g066100.2.1	0	0	0	0	0	0	0	0	0	0	0	0	0
Solyc06g066160.2.1	0	0	0	0	0	0	0	0	0	0	0	0	0
Solyc06g066170.2.1	6	34	2	7	0	53	25	31	11	1	34	1	3
Solyc06g066180.2.1	0	0	0	0	0	0	0	0	0	0	0	0	0
Solyc06g066190.2.1	3	32	2	12	0	56	10	33	6	32	5	9	0
Solyc06g066210.1.1	3	4	3	0	7	7	40	10	4	9	10	25	1

Solyc06g066240.2.1	0	0	0	0	0	0	0	0	0	0	0	0	0
Solyc06g066280.2.1	0	0	0	0	0	0	0	0	0	0	0	0	0
Solyc06g066290.2.1	11	11	12	2	0	90	229	120	42	16	14	23	0
Solyc06g066300.1.1	12	36	5	20	0	43	55	74	6	114	24	76	0
Solyc06g066310.2.1	2	6	0	0	6	6	3	15	0	16	3	13	0
Solyc06g066320.2.1	13	297	21	185	15	485	233	549	68	445	96	493	12
Solyc06g066330.2.1	5	20	2	12	3	30	23	30	10	44	4	32	6
Solyc06g066340.2.1	2	15	1	2	0	9	9	23	1	3	4	3	0
Solyc06g066370.2.1	20	887	181	747	75	645	214	285	61	78	62	216	27
Solyc06g066380.1.1	1	1	0	0	0	2	8	4	0	7	0	8	0
Solyc06g066400.2.1	1	7	1	0	0	6	12	3	2	3	1	1	0
Solyc06g066420.2.1	1	4	0	18	0	1	20	10	0	0	0	0	0
Solyc06g066430.2.1	4	14	5	22	1	7	21	33	6	40	21	30	2
Solyc06g066440.2.1	6	19	5	20	0	132	86	140	9	27	5	33	1
Solyc06g066460.2.1	11	43	16	37	4	227	231	116	38	107	64	88	2
Solyc06g066470.2.1	17	67	6	73	13	300	138	174	43	168	43	88	9
Solyc06g066490.1.1	1	9	1	7	0	0	3	13	0	0	0	0	0
Solyc06g066500.1.1	0	0	0	0	0	0	0	0	0	0	0	0	0
Solyc06g066510.1.1	0	0	0	0	0	0	0	0	0	0	0	0	0
Solyc06g066520.1.1	0	0	0	0	0	0	0	0	0	0	0	0	0
Solyc06g066530.1.1	0	0	0	0	0	0	0	0	0	0	0	0	0
Solyc06g066540.1.1	0	0	0	0	0	0	0	0	0	0	0	0	0
Solyc06g066550.2.1	10	8	0	8	3	39	54	49	3	31	10	48	0
Solyc06g066560.1.1	0	0	0	0	0	0	0	0	0	0	0	0	0
Solyc06g066570.2.1	5	35	4	29	1	31	15	69	5	61	8	50	1
Solyc06g066580.2.1	1	0	0	0	0	8	6	8	0	2	0	1	0
Solyc06g066600.2.1	13	69	3	52	1	934	138	401	24	85	28	118	2
Solyc06g066610.2.1	14	56	6	23	2	87	67	109	15	122	21	86	1
Solyc06g066620.2.1	10	447	100	204	6	83	114	110	2	238	36	303	4
Solyc06g066630.1.1	0	0	0	0	0	0	0	0	0	0	0	0	0
Solyc06g066640.2.1	6	939	139	377	1	19	234	29	2	56	49	19	4
Solyc06g066650.2.1	0	0	0	0	0	0	0	0	0	0	0	0	0
Solyc06g066660.2.1	0	0	0	0	0	0	0	0	0	0	0	0	0
Solyc06g066670.2.1	0	0	0	0	0	0	0	0	0	0	0	0	0
Solyc06g066680.2.1	2	0	0	0	0	11	14	18	0	1	0	4	0
Solyc06g066700.1.1	0	0	0	0	0	0	0	0	0	0	0	0	0
Solyc06g066730.2.1	9	160	2	78	10	149	61	237	19	456	42	509	0
Solyc06g066740.1.1	4	21	1	17	1	18	8	37	2	24	18	32	0
Solyc06g066760.2.1	3	45	5	20	9	57	39	73	3	60	21	57	3
Solyc06g066770.1.1	1	7	0	4	0	3	4	0	0	2	0	0	0
Solyc06g066780.2.1	4	17	0	11	0	52	39	41	3	32	7	14	2

Solyc06g066790.2.1	8	35	5	29	7	128	42	161	26	55	14	46	4
Solyc06g066800.1.1	2	34	6	14	0	15	2	4	1	14	1	21	1
Solyc06g066810.2.1	5	16	2	13	0	10	12	31	3	28	10	27	0
Solyc06g066820.2.1	0	0	0	0	0	0	0	0	0	0	0	0	0
Solyc06g067870.2.1	0	0	0	0	0	0	0	0	0	0	0	0	0
Solyc06g067880.2.1	5	154	13	73	4	168	85	145	25	385	62	375	3
Solyc06g067890.2.1	1	0	0	3	1	9	2	13	4	5	2	0	0
Solyc06g067900.1.1	0	0	0	0	0	0	0	0	0	0	0	0	0
Solyc06g067910.2.1	1	6	0	2	2	1	2	2	0	0	0	0	0
Solyc06g067920.2.1	15	159	12	69	5	246	81	334	19	160	61	167	3
Solyc06g067940.2.1	5	142	23	60	10	204	79	266	16	139	41	106	2
Solyc06g067950.2.1	2	9	0	4	0	6	7	21	0	74	6	25	0
Solyc06g067960.2.1	4	52	4	16	0	118	41	97	12	57	13	63	1
Solyc06g067970.1.1	4	7	0	10	5	16	32	54	7	51	5	31	0
Solyc06g067980.2.1	2	140	0	7	5	2889	37	765	46	9	3	3	0
Solyc06g067990.2.1	5	11	1	13	2	29	32	22	0	35	2	16	0
Solyc06g068010.2.1	6	68	12	61	2	38	95	64	7	109	17	45	3
Solyc06g068020.1.1	1	0	1	0	0	4	6	15	1	10	1	0	0
Solyc06g068030.2.1	2	17	0	2	1	10	26	11	2	98	10	65	0
Solyc06g068040.2.1	0	0	0	0	0	0	0	0	0	0	0	0	0
Solyc06g068050.2.1	2	2	1	0	3	14	23	12	6	14	9	20	0
Solyc06g068060.2.1	0	0	0	0	0	0	0	0	0	0	0	0	0
Solyc06g068080.2.1	4	37	1	12	0	135	33	45	7	118	31	76	2
Solyc06g068090.2.1	12	237	16	143	13	1336	140	1726	68	285	68	227	3
Solyc06g068120.2.1	3	5	2	8	0	11	30	18	1	43	3	42	1
Solyc06g068130.2.1	2	2	0	0	0	29	21	33	0	23	0	4	0
Solyc06g068140.2.1	24	422	24	268	17	510	255	667	35	728	74	406	25
Solyc06g068160.2.1	3	2	0	0	0	31	11	4	2	11	2	35	0
Solyc06g068190.1.1	0	0	0	0	0	0	0	0	0	0	0	0	0
Solyc06g068200.1.1	0	0	0	0	0	0	0	0	0	0	0	0	0
Solyc06g068210.1.1	6	8	2	11	2	9	20	16	2	43	8	37	0
Solyc06g068220.2.1	7	272	55	164	2	23	61	82	1	44	12	51	1
Solyc06g068230.2.1	27	468	125	520	48	947	251	906	23	205	143	331	7
Solyc06g068240.2.1	0	0	0	0	0	0	0	0	0	0	0	0	0
Solyc06g068270.2.1	0	0	0	0	0	0	0	0	0	0	0	0	0
Solyc06g068280.2.1	8	93	7	65	0	133	50	115	7	159	11	81	0
Solyc06g068290.1.1	0	0	0	0	0	0	0	0	0	0	0	0	0
Solyc06g068300.2.1	0	0	0	0	0	0	0	0	0	0	0	0	0
Solyc06g068310.1.1	0	0	0	0	0	0	0	0	0	0	0	0	0
Solyc06g068320.2.1	8	69	12	30	0	68	72	151	16	218	63	201	5
Solyc06g068330.1.1	3	5	0	2	0	11	4	7	3	6	2	7	1

Solyc06g068360.2.1	0	0	0	0	0	0	0	0	0	0	0	0	0
Solyc06g068370.2.1	0	0	0	0	0	0	0	0	0	0	0	0	0
Solyc06g068380.2.1	5	32	2	12	0	25	29	45	0	100	6	65	0
Solyc06g068390.1.1	0	0	0	0	0	0	0	0	0	0	0	0	0
Solyc06g068400.2.1	3	14	3	7	5	47	50	41	9	60	61	41	4
Solyc06g068410.2.1	3	27	0	16	3	23	33	28	7	50	8	44	0
Solyc06g068430.2.1	0	0	0	0	0	0	0	0	0	0	0	0	0
Solyc06g068440.2.1	3	37	5	35	0	298	12	368	0	41	4	10	0
Solyc06g068450.1.1	7	89	10	70	1	374	198	574	11	157	7	144	2
Solyc06g068460.2.1	11	1056	183	510	76	127	51	38	2	118	17	215	9
Solyc06g068470.2.1	1	1	0	0	1	0	4	0	0	23	9	31	0
Solyc06g068480.2.1	5	98	14	29	4	43	62	54	6	36	8	32	5
Solyc06g068490.2.1	10	33	6	45	1	98	43	90	3	106	24	70	1
Solyc06g068500.2.1	5	49	1	29	0	58	268	231	4	840	31	235	6
Solyc06g068510.1.1	0	0	0	0	0	0	0	0	0	0	0	0	0
Solyc06g068520.2.1	3	77	5	52	0	224	38	130	0	91	81	178	0
Solyc06g068540.2.1	3	20	5	11	0	54	32	93	4	8	9	29	1
Solyc06g068550.2.1	0	0	0	0	0	0	0	0	0	0	0	0	0
Solyc06g068560.2.1	1	3	0	5	0	5	4	1	0	3	0	2	0
Solyc06g068570.2.1	0	0	0	0	0	0	0	0	0	0	0	0	0
Solyc06g068590.2.1	0	0	0	0	0	0	0	0	0	0	0	0	0
Solyc06g068600.2.1	0	0	0	0	0	0	0	0	0	0	0	0	0
Solyc06g068610.1.1	0	0	0	0	0	0	0	0	0	0	0	0	0
Solyc06g068620.2.1	3	78	5	38	4	170	58	226	20	75	22	48	4
Solyc06g068630.2.1	2	18	0	5	0	52	23	47	2	15	7	9	0
Solyc06g068650.2.1	10	209	13	212	0	134	67	295	39	21	10	30	0
Solyc06g068670.2.1	5	475	45	547	28	656	1193	480	37	433	94	404	4
Solyc06g068680.2.1	0	0	0	0	0	0	0	0	0	0	0	0	0
Solyc06g068690.2.1	0	0	0	0	0	0	0	0	0	0	0	0	0
Solyc06g068700.2.1	0	0	0	0	0	0	0	0	0	0	0	0	0
Solyc06g068710.2.1	1	1	0	0	0	1	0	5	0	6	4	1	0
Solyc06g068720.2.1	9	60	1	44	7	270	52	174	20	48	16	51	0
Solyc06g068740.2.1	4	25	0	8	0	192	56	110	2	76	17	65	0
Solyc06g068750.2.1	8	87	3	53	1	33	47	49	1	83	4	116	1
Solyc06g068760.2.1	2	75	3	39	0	11	11	61	0	32	5	31	0
Solyc06g068770.2.1	1	14	0	3	0	9	4	17	0	4	1	2	0
Solyc06g068780.2.1	0	0	0	0	0	0	0	0	0	0	0	0	0
Solyc06g068800.2.1	6	24	2	0	5	51	32	55	81	39	12	11	7
Solyc06g068810.2.1	0	0	0	0	0	0	0	0	0	0	0	0	0
Solyc06g068820.2.1	2	17	0	9	0	30	7	23	2	16	0	13	0
Solyc06g068840.2.1	9	34	6	39	1	110	56	95	4	50	24	40	0

Solyc06g068850.2.1	10	10	8	28	8	59	54	112	5	117	14	110	1
Solyc06g068860.2.1	25	104	14	91	15	370	782	473	40	451	83	470	29
Solyc06g068870.2.1	13	79	9	44	9	231	122	224	22	242	74	203	2
Solyc06g068880.2.1	7	18	4	9	8	12	10	25	13	74	84	63	0
Solyc06g068900.2.1	0	0	0	0	0	0	0	0	0	0	0	0	0
Solyc06g068910.2.1	0	0	0	0	0	0	0	0	0	0	0	0	0
Solyc06g068920.2.1	7	23	4	19	2	94	59	92	6	69	25	118	0
Solyc06g068940.2.1	0	0	0	0	0	0	0	0	0	0	0	0	0
Solyc06g068950.2.1	0	0	0	0	0	0	0	0	0	0	0	0	0
Solyc06g068960.1.1	1	135	7	46	1	106	1	226	4	2	3	4	0
Solyc06g068970.2.1	3	54	7	31	0	0	7	13	2	40	17	31	0
Solyc06g068980.2.1	10	57	7	52	2	28	29	64	1	40	16	48	0
Solyc06g068990.2.1	0	0	0	0	0	0	0	0	0	0	0	0	0
Solyc06g069000.2.1	0	0	0	0	0	0	0	0	0	0	0	0	0
Solyc06g069010.2.1	11	207	10	96	8	348	130	472	32	517	115	628	11
Solyc06g069020.2.1	13	133	16	72	19	468	216	536	133	493	115	350	11
Solyc06g069030.2.1	6	113	20	58	0	109	44	121	4	61	5	59	4
Solyc06g069040.2.1	5	20	8	3	3	56	137	24	54	1	10	0	0
Solyc06g069070.1.1	2	33	1	22	0	194	80	606	0	6	0	1	0
Solyc06g069080.2.1	0	0	0	0	0	0	0	0	0	0	0	0	0
Solyc06g069090.2.1	3	106	11	63	5	535	135	430	60	266	77	273	0
Solyc06g069120.2.1	1	45	1	36	0	60	21	72	0	279	24	203	1
Solyc06g069130.2.1	0	0	0	0	0	0	0	0	0	0	0	0	0
Solyc06g069150.1.1	1	13	0	1	0	62	3	24	0	4	0	0	0
Solyc06g069170.2.1	5	731	141	459	11	576	1219	952	31	428	196	449	10
Solyc06g069180.2.1	5	36	1	39	4	145	58	110	10	98	49	57	6
Solyc06g069190.2.1	10	116	21	139	30	90	102	187	10	254	40	221	10
Solyc06g069210.2.1	13	65	7	53	2	163	155	182	27	160	51	156	2
Solyc06g069220.1.1	0	0	0	0	0	0	0	0	0	0	0	0	0
Solyc06g069230.2.1	0	0	0	0	0	0	0	0	0	0	0	0	0
Solyc06g069240.1.1	0	0	0	0	0	0	0	0	0	0	0	0	0
Solyc06g069270.1.1	0	0	0	0	0	0	0	0	0	0	0	0	0
Solyc06g069280.2.1	3	3	0	1	0	5	8	12	1	16	5	13	0
Solyc06g069290.2.1	0	0	0	0	0	0	0	0	0	0	0	0	0
Solyc06g069300.1.1	0	0	0	0	0	0	0	0	0	0	0	0	0
Solyc06g069310.2.1	0	0	0	0	0	0	0	0	0	0	0	0	0
Solyc06g069330.2.1	6	24	1	5	2	28	46	60	17	60	3	17	3
Solyc06g069350.1.1	0	0	0	0	0	0	0	0	0	0	0	0	0
Solyc06g069360.2.1	6	37	13	23	1	96	94	72	12	63	30	63	6
Solyc06g069370.2.1	1	4	0	8	0	3	0	4	0	3	4	8	0
Solyc06g069380.2.1	5	37	4	11	8	46	44	40	40	10	13	8	0

Solyc06g069390.2.1	2	13	1	3	0	32	15	15	0	33	3	14	1
Solyc06g069400.2.1	0	0	0	0	0	0	0	0	0	0	0	0	0
Solyc06g069410.2.1	5	36	3	29	0	66	18	59	6	41	8	21	0
Solyc06g069420.2.1	3	19	7	20	3	20	57	10	13	8	0	3	0
Solyc06g069430.2.1	11	56	33	21	25	249	401	114	72	293	178	187	3
Solyc06g069440.2.1	29	165	26	153	1	389	266	616	57	234	74	246	5
Solyc06g069450.2.1	2	19	0	1	0	0	11	20	1	14	3	10	0
Solyc06g069460.1.1	0	0	0	0	0	0	0	0	0	0	0	0	0
Solyc06g069470.2.1	5	46	10	48	7	296	69	528	26	153	41	156	0
Solyc06g069480.2.1	29	96	12	47	12	249	304	310	41	172	56	134	13
Solyc06g069490.2.1	4	27	0	38	0	29	51	71	22	52	21	95	1
Solyc06g069500.2.1	16	65	12	43	6	150	67	244	10	103	26	98	12
Solyc06g069510.2.1	1	0	0	0	0	0	0	5	0	17	0	2	0
Solyc06g069520.2.1	0	0	0	0	0	0	0	0	0	0	0	0	0
Solyc06g069530.2.1	1	17	2	5	0	21	14	23	4	39	3	9	0
Solyc06g069550.1.1	0	0	0	0	0	0	0	0	0	0	0	0	0
Solyc06g069560.1.1	10	174	20	86	8	324	104	492	52	150	34	107	1
Solyc06g069570.2.1	2	52	20	53	0	78	35	89	26	46	1	50	2
Solyc06g069580.2.1	2	3	1	1	0	5	4	5	1	5	6	6	0
Solyc06g069600.1.1	0	0	0	0	0	0	0	0	0	0	0	0	0
Solyc06g069620.1.1	0	0	0	0	0	0	0	0	0	0	0	0	0
Solyc06g069630.2.1	2	6	1	2	0	2	5	15	0	8	0	8	0
Solyc06g069650.2.1	0	0	0	0	0	0	0	0	0	0	0	0	0
Solyc06g069660.1.1	0	0	0	0	0	0	0	0	0	0	0	0	0
Solyc06g069670.2.1	0	0	0	0	0	0	0	0	0	0	0	0	0
Solyc06g069680.1.1	0	0	0	0	0	0	0	0	0	0	0	0	0
Solyc06g069690.2.1	10	17	2	14	11	245	85	168	16	32	23	16	4
Solyc06g069700.2.1	0	0	0	0	0	0	0	0	0	0	0	0	0
Solyc06g069710.2.1	4	0	0	0	0	158	20	136	18	0	7	0	0
Solyc06g069720.2.1	0	0	0	0	0	0	0	0	0	0	0	0	0
Solyc06g069730.2.1	8	454	171	94	1	1	2	5	0	6	5	1	0
Solyc06g069740.1.1	0	0	0	0	0	0	0	0	0	0	0	0	0
Solyc06g069760.2.1	19	135	27	232	18	163	99	118	18	113	49	121	0
Solyc06g069770.2.1	4	23	3	20	2	102	53	93	4	177	22	72	0
Solyc06g069780.2.1	4	38	5	20	1	55	61	32	4	42	18	47	0
Solyc06g069790.2.1	0	0	0	0	0	0	0	0	0	0	0	0	0
Solyc06g069820.2.1	0	0	0	0	0	0	0	0	0	0	0	0	0
Solyc06g069850.2.1	0	0	0	0	0	0	0	0	0	0	0	0	0
Solyc06g069860.2.1	4	691	53	347	11	1530	218	1070	65	3483	286	2832	27
Solyc06g069880.1.1	0	0	0	0	0	0	0	0	0	0	0	0	0
Solyc06g069890.2.1	7	38	3	2	6	116	29	174	16	144	14	144	1

Solyc06g070900.2.1	0	0	0	0	0	0	0	0	0	0	0	0	0
Solyc06g070920.2.1	0	0	0	0	0	0	0	0	0	0	0	0	0
Solyc06g070940.2.1	0	0	0	0	0	0	0	0	0	0	0	0	0
Solyc06g070950.1.1	0	0	0	0	0	0	0	0	0	0	0	0	0
Solyc06g070960.1.1	0	0	0	0	0	0	0	0	0	0	0	0	0
Solyc06g070970.2.1	0	0	0	0	0	0	0	0	0	0	0	0	0
Solyc06g070980.2.1	7	644	49	423	24	663	333	766	48	3094	297	3381	17
Solyc06g070990.2.1	0	0	0	0	0	0	0	0	0	0	0	0	0
Solyc06g071000.2.1	16	172	22	134	22	2522	5139	2909	103	557	156	623	26
Solyc06g071010.2.1	3	20	0	8	3	91	21	63	1	23	3	11	0
Solyc06g071020.2.1	0	0	0	0	0	0	0	0	0	0	0	0	0
Solyc06g071030.2.1	1	5	0	3	0	0	5	1	0	0	0	0	0
Solyc06g071040.2.1	12	48	1	36	2	166	80	119	16	97	14	60	1
Solyc06g071050.2.1	9	188	49	233	9	65	188	112	58	272	11	170	10
Solyc06g071060.1.1	0	0	0	0	0	0	0	0	0	0	0	0	0
Solyc06g071070.1.1	3	32	9	27	5	26	98	19	0	35	2	16	0
Solyc06g071080.2.1	0	0	0	0	0	0	0	0	0	0	0	0	0
Solyc06g071090.1.1	0	0	0	0	0	0	0	0	0	0	0	0	0
Solyc06g071100.2.1	12	1040	133	724	70	952	1311	1565	109	682	323	378	10
Solyc06g071110.2.1	2	6	0	9	0	5	6	10	5	9	2	6	0
Solyc06g071120.2.1	17	62	6	31	1	87	80	160	22	85	27	106	0
Solyc06g071140.2.1	5	45	1	47	3	39	42	115	16	105	19	37	0
Solyc06g071150.2.1	0	0	0	0	0	0	0	0	0	0	0	0	0
Solyc06g071160.2.1	4	22	3	18	4	14	8	30	3	40	8	36	3
Solyc06g071170.2.1	7	160	18	128	3	357	66	435	21	279	43	158	0
Solyc06g071180.2.1	4	3	0	0	0	24	19	40	0	18	2	5	0
Solyc06g071200.2.1	12	24	3	36	3	132	98	104	4	153	25	115	0
Solyc06g071210.2.1	11	59	8	74	12	231	119	240	6	123	18	72	3
Solyc06g071220.1.1	0	0	0	0	0	0	0	0	0	0	0	0	0
Solyc06g071230.2.1	12	203	43	120	18	720	327	545	66	703	132	395	27
Solyc06g071250.2.1	0	0	0	0	0	0	0	0	0	0	0	0	0
Solyc06g071260.2.1	3	8	0	5	3	8	22	9	1	6	1	11	0
Solyc06g071270.2.1	7	48	5	16	2	50	31	59	3	56	14	40	0
Solyc06g071280.2.1	6	85	15	97	2	88	46	94	2	28	0	38	0
Solyc06g071290.2.1	6	69	1	37	1	8	30	33	3	209	4	155	0
Solyc06g071300.1.1	0	0	0	0	0	0	0	0	0	0	0	0	0
Solyc06g071310.2.1	5	35	3	8	2	16	34	24	0	57	12	37	1
Solyc06g071320.2.1	0	0	0	0	0	0	0	0	0	0	0	0	0
Solyc06g071330.2.1	1	1	0	0	0	0	2	2	0	66	9	82	2
Solyc06g071350.2.1	29	172	33	217	22	578	534	868	74	291	133	429	5
Solyc06g071370.1.1	4	23	1	24	0	20	15	56	1	39	9	35	1

Solyc06g071380.2.1	2	7	1	3	0	8	11	30	3	31	6	12	0
Solyc06g071390.2.1	1	1	0	0	0	2	7	3	2	6	0	2	5
Solyc06g071400.2.1	0	0	0	0	0	0	0	0	0	0	0	0	0
Solyc06g071410.2.1	2	9	4	1	0	7	38	8	2	25	4	3	1
Solyc06g071420.2.1	7	23	7	29	7	44	50	49	2	68	19	45	7
Solyc06g071430.2.1	15	195	41	219	5	227	474	436	43	182	118	136	5
Solyc06g071440.2.1	4	13	6	4	2	34	24	35	6	13	4	17	0
Solyc06g071450.2.1	0	0	0	0	0	0	0	0	0	0	0	0	0
Solyc06g071460.2.1	0	0	0	0	0	0	0	0	0	0	0	0	0
Solyc06g071470.2.1	12	147	13	139	6	443	237	478	29	374	104	235	11
Solyc06g071480.2.1	0	0	0	0	0	0	0	0	0	0	0	0	0
Solyc06g071490.1.1	21	96	22	106	9	177	82	272	16	247	94	126	6
Solyc06g071500.2.1	21	145	32	97	2	2188	436	1968	8	771	55	627	0
Solyc06g071510.2.1	3	4	0	8	0	19	11	31	6	21	17	40	1
Solyc06g071530.2.1	2	185	2	53	3	319	42	233	12	247	9	133	2
Solyc06g071540.2.1	0	0	0	0	0	0	0	0	0	0	0	0	0
Solyc06g071550.2.1	7	26	6	26	11	37	55	96	14	33	16	11	2
Solyc06g071560.2.1	3	11	2	6	3	15	19	16	0	3	1	0	0
Solyc06g071570.2.1	0	0	0	0	0	0	0	0	0	0	0	0	0
Solyc06g071580.2.1	0	0	0	0	0	0	0	0	0	0	0	0	0
Solyc06g071590.2.1	0	0	0	0	0	0	0	0	0	0	0	0	0
Solyc06g071600.2.1	1	0	0	0	0	3	4	4	0	2	1	2	0
Solyc06g071620.2.1	0	0	0	0	0	0	0	0	0	0	0	0	0
Solyc06g071630.1.1	0	0	0	0	0	0	0	0	0	0	0	0	0
Solyc06g071640.2.1	0	0	0	0	0	0	0	0	0	0	0	0	0
Solyc06g071660.1.1	0	0	0	0	0	0	0	0	0	0	0	0	0
Solyc06g071670.1.1	2	12	2	9	0	11	10	19	0	26	0	22	0
Solyc06g071680.2.1	8	91	4	116	4	297	61	268	11	225	73	198	0
Solyc06g071690.2.1	0	0	0	0	0	0	0	0	0	0	0	0	0
Solyc06g071720.1.1	2	101	5	51	1	124	21	121	14	693	23	333	18
Solyc06g071730.2.1	0	0	0	0	0	0	0	0	0	0	0	0	0
Solyc06g071740.1.1	0	0	0	0	0	0	0	0	0	0	0	0	0
Solyc06g071750.2.1	7	16	2	9	0	529	18	93	2	7	72	53	0
Solyc06g071770.2.1	31	145	17	117	1	691	543	918	75	551	139	354	11
Solyc06g071780.2.1	1	2	0	18	0	0	0	2	0	2	13	18	0
Solyc06g071790.2.1	15	1612	221	1277	7	416	794	802	14	598	148	913	2
Solyc06g071800.2.1	5	19	2	25	0	79	60	69	11	53	12	95	1
Solyc06g071810.1.1	17	340	99	427	53	373	3	195	9	93	3	158	6
Solyc06g071820.2.1	13	159	66	134	20	514	323	444	44	1946	334	1290	13
Solyc06g071830.2.1	0	0	0	0	0	0	0	0	0	0	0	0	0
Solyc06g071850.2.1	3	12	1	13	0	28	44	17	5	14	8	13	2

Solyc06g071860.2.1	19	43	8	33	11	208	127	303	25	158	84	131	14
Solyc06g071870.2.1	1	139	3	106	24	985	251	963	230	99	44	159	27
Solyc06g071880.2.1	3	48	3	14	0	93	14	81	3	96	9	160	3
Solyc06g071890.2.1	0	0	0	0	0	0	0	0	0	0	0	0	0
Solyc06g071900.2.1	2	8	0	11	0	83	48	117	11	31	3	18	0
Solyc06g071910.2.1	5	66	13	37	0	146	115	282	9	154	33	109	2
Solyc06g071920.2.1	3	504	222	285	26	370	385	392	50	682	367	578	31
Solyc06g071930.1.1	0	0	0	0	0	0	0	0	0	0	0	0	0
Solyc06g071950.1.1	2	3	0	5	0	0	1	0	0	7	4	2	0
Solyc06g071960.2.1	3	54	9	30	7	21	19	22	2	36	10	20	1
Solyc06g071980.2.1	1	4	0	0	0	0	4	7	0	4	0	3	0
Solyc06g071990.2.1	2	8	2	7	2	4	10	15	9	46	2	28	6
Solyc06g072010.1.1	0	0	0	0	0	0	0	0	0	0	0	0	0
Solyc06g072020.1.1	0	0	0	0	0	0	0	0	0	0	0	0	0
Solyc06g072030.1.1	1	7	4	1	0	15	1	17	0	12	0	10	0
Solyc06g072040.1.1	6	346	38	216	11	210	274	456	28	529	79	409	11
Solyc06g072050.2.1	12	114	10	95	6	251	114	255	18	152	28	94	0
Solyc06g072070.1.1	0	0	0	0	0	0	0	0	0	0	0	0	0
Solyc06g072080.2.1	0	0	0	0	0	0	0	0	0	0	0	0	0
Solyc06g072110.2.1	11	102	19	76	4	263	195	190	12	279	34	130	22
Solyc06g072120.2.1	7	922	60	636	44	2235	522	1912	214	2001	429	1566	21
Solyc06g072130.2.1	14	3	0	1	0	813	32	719	1	13	257	2	0
Solyc06g072140.2.1	5	31	4	13	0	70	15	80	6	77	10	78	0
Solyc06g072160.2.1	1	3	0	3	0	2	4	9	0	7	1	1	0
Solyc06g072170.2.1	1	2	0	3	0	7	3	13	0	4	0	1	0
Solyc06g072220.1.1	0	0	0	0	0	0	0	0	0	0	0	0	0
Solyc06g072230.1.1	1	15	2	6	0	22	11	22	0	0	0	0	0
Solyc06g072240.1.1	0	0	0	0	0	0	0	0	0	0	0	0	0
Solyc06g072250.2.1	2	2	3	2	0	6	9	6	0	9	9	16	0
Solyc06g072270.2.1	4	3	0	2	0	13	8	31	1	12	7	9	0
Solyc06g072280.2.1	1	19	2	6	0	57	14	18	3	37	1	24	0
Solyc06g072290.2.1	0	0	0	0	0	0	0	0	0	0	0	0	0
Solyc06g072300.2.1	25	333	33	261	51	724	239	703	61	671	82	538	7
Solyc06g072310.2.1	0	0	0	0	0	0	0	0	0	0	0	0	0
Solyc06g072320.2.1	0	0	0	0	0	0	0	0	0	0	0	0	0
Solyc06g072330.2.1	0	0	0	0	0	0	0	0	0	0	0	0	0
Solyc06g072340.2.1	5	15	2	20	0	36	28	38	8	7	1	3	0
Solyc06g072350.2.1	4	1020	198	277	0	152	78	210	0	7	0	2	1
Solyc06g072360.2.1	0	0	0	0	0	0	0	0	0	0	0	0	0
Solyc06g072370.2.1	2	3	1	0	0	15	6	3	0	7	5	6	1
Solyc06g072380.2.1	8	37	4	23	0	142	193	259	35	53	29	59	4

Solyc06g072420.2.1	0	0	0	0	0	0	0	0	0	0	0	0	0
Solyc06g072430.1.1	6	38	7	13	0	42	7	15	1	31	57	41	0
Solyc06g072440.2.1	2	15	0	15	0	10	12	9	4	36	13	29	1
Solyc06g072460.1.1	2	4	0	4	0	20	12	3	0	1	0	3	0
Solyc06g072470.2.1	1	17	1	0	0	2	3	7	0	7	0	2	0
Solyc06g072490.2.1	2	35	6	24	5	82	44	103	20	103	57	143	4
Solyc06g072500.2.1	0	0	0	0	0	0	0	0	0	0	0	0	0
Solyc06g072510.2.1	0	0	0	0	0	0	0	0	0	0	0	0	0
Solyc06g072520.1.1	0	0	0	0	0	0	0	0	0	0	0	0	0
Solyc06g072530.2.1	0	0	0	0	0	0	0	0	0	0	0	0	0
Solyc06g072550.2.1	0	0	0	0	0	0	0	0	0	0	0	0	0
Solyc06g072570.2.1	1	21	2	12	1	29	20	43	1	15	1	12	0
Solyc06g072580.2.1	7	92	21	106	7	171	133	156	25	167	21	64	4
Solyc06g072600.2.1	1	3	0	15	0	12	1	13	0	14	0	6	0
Solyc06g072610.2.1	7	32	7	11	0	83	48	43	30	30	10	44	1
Solyc06g072620.2.1	0	0	0	0	0	0	0	0	0	0	0	0	0
Solyc06g072630.2.1	0	0	0	0	0	0	0	0	0	0	0	0	0
Solyc06g072640.1.1	0	0	0	0	0	0	0	0	0	0	0	0	0
Solyc06g072650.1.1	1	1	0	0	0	94	12	24	0	0	0	0	0
Solyc06g072660.2.1	1	1	0	0	0	0	2	5	0	5	3	3	0
Solyc06g072670.2.1	3	1	0	0	0	35	0	129	0	0	19	2	6
Solyc06g072680.2.1	3	0	0	0	0	24	19	55	3	0	15	0	0
Solyc06g072690.1.1	2	6	0	1	0	0	7	6	1	15	0	5	0
Solyc06g072700.2.1	4	46	1	55	0	16	9	9	0	79	12	62	0
Solyc06g072710.2.1	9	165	28	91	1	25	51	57	1	35	9	37	0
Solyc06g072720.2.1	2	10	0	6	0	27	8	16	1	10	1	6	0
Solyc06g072730.2.1	0	0	0	0	0	0	0	0	0	0	0	0	0
Solyc06g072740.1.1	3	11	4	8	1	61	7	29	1	9	7	5	0
Solyc06g072750.2.1	1	2	2	0	0	2	6	7	0	2	0	3	0
Solyc06g072760.2.1	0	0	0	0	0	0	0	0	0	0	0	0	0
Solyc06g072770.2.1	0	0	0	0	0	0	0	0	0	0	0	0	0
Solyc06g072780.1.1	0	0	0	0	0	0	0	0	0	0	0	0	0
Solyc06g072790.2.1	10	59	6	46	9	72	72	136	20	92	34	62	0
Solyc06g072800.2.1	2	20	4	12	0	15	31	25	5	39	10	7	0
Solyc06g072810.2.1	0	0	0	0	0	0	0	0	0	0	0	0	0
Solyc06g072820.2.1	0	0	0	0	0	0	0	0	0	0	0	0	0
Solyc06g072830.2.1	0	0	0	0	0	0	0	0	0	0	0	0	0
Solyc06g072840.2.1	1	7	0	1	0	11	3	7	0	302	9	300	3
Solyc06g072870.1.1	0	0	0	0	0	0	0	0	0	0	0	0	0
Solyc06g072890.1.1	0	0	0	0	0	0	0	0	0	0	0	0	0
Solyc06g072900.2.1	0	0	0	0	0	0	0	0	0	0	0	0	0

Solyc06g072930.2.1	1	19	0	8	0	22	3	14	0	20	0	16	0
Solyc06g072960.1.1	0	0	0	0	0	0	0	0	0	0	0	0	0
Solyc06g072970.2.1	1	0	0	2	0	1	2	2	0	8	0	2	0
Solyc06g072980.2.1	9	23	7	21	0	62	68	109	14	55	14	44	0
Solyc06g072990.1.1	0	0	0	0	0	0	0	0	0	0	0	0	0
Solyc06g073010.2.1	0	0	0	0	0	0	0	0	0	0	0	0	0
Solyc06g073020.2.1	1	8	1	0	0	9	10	4	0	10	0	4	0
Solyc06g073030.1.1	0	0	0	0	0	0	0	0	0	0	0	0	0
Solyc06g073050.2.1	12	353	174	228	54	147	70	93	42	98	66	203	33
Solyc06g073060.2.1	11	135	3	25	2	590	29	581	5	270	44	197	0
Solyc06g073070.1.1	0	0	0	0	0	0	0	0	0	0	0	0	0
Solyc06g073080.2.1	0	0	0	0	0	0	0	0	0	0	0	0	0
Solyc06g073090.2.1	9	327	22	246	7	99	522	289	8	317	17	170	6
Solyc06g073110.2.1	8	39	4	31	1	78	37	54	1	69	29	68	1
Solyc06g073120.2.1	2	10	2	4	1	25	7	23	3	8	2	13	0
Solyc06g073130.2.1	1	0	1	0	1	1	18	20	3	35	2	19	0
Solyc06g073140.1.1	0	0	0	0	0	0	0	0	0	0	0	0	0
Solyc06g073150.2.1	0	0	0	0	0	0	0	0	0	0	0	0	0
Solyc06g073160.2.1	0	0	0	0	0	0	0	0	0	0	0	0	0
Solyc06g073170.1.1	1	21	4	14	0	7	2	2	2	65	0	25	0
Solyc06g073180.2.1	5	559	33	415	28	249	69	403	28	126	124	184	1
Solyc06g073190.2.1	5	294	11	146	19	262	299	536	59	2261	72	1418	34
Solyc06g073200.2.1	1	1	0	3	0	4	4	9	0	2	0	1	0
Solyc06g073210.2.1	1	4	0	1	0	5	6	12	0	1	1	9	0
Solyc06g073230.2.1	1	4	0	2	0	9	4	6	0	10	0	1	0
Solyc06g073240.2.1	4	12	1	25	0	30	18	21	2	54	5	64	1
Solyc06g073260.2.1	10	4823	281	2046	0	36	137	191	2	73	30	86	1
Solyc06g073270.2.1	0	0	0	0	0	0	0	0	0	0	0	0	0
Solyc06g073280.2.1	6	114	8	44	0	108	57	184	2	235	35	233	3
Solyc06g073290.1.1	11	954	84	659	3	19	51	28	10	58	131	65	6
Solyc06g073300.1.1	1	131	5	47	3	203	22	199	9	819	22	427	2
Solyc06g073310.2.1	8	430	22	163	20	992	235	1557	49	1062	114	1069	10
Solyc06g073320.2.1	15	9019	805	6530	95	1543	201	1666	23	3298	410	3069	14
Solyc06g073330.2.1	11	109	19	78	13	270	157	320	29	450	87	364	12
Solyc06g073340.2.1	10	59	7	62	5	86	51	154	23	154	44	192	10
Solyc06g073350.2.1	0	0	0	0	0	0	0	0	0	0	0	0	0
Solyc06g073360.2.1	2	34	2	1	4	48	35	28	1	98	36	266	17
Solyc06g073370.2.1	1	92	1	46	0	136	24	175	5	130	11	143	0
Solyc06g073380.2.1	1	3	0	3	3	11	2	0	0	2	2	2	1
Solyc06g073390.2.1	7	20	5	9	0	44	35	45	5	67	27	64	4
Solyc06g073400.2.1	2	9	0	0	1	15	9	13	0	14	0	9	0

Solyc06g073410.2.1	1	12	2	0	6	33	44	7	9	9	10	15	2
Solyc06g073420.2.1	4	16	1	12	2	28	33	30	3	10	0	4	0
Solyc06g073430.2.1	1	28	0	17	0	59	8	56	2	121	18	89	0
Solyc06g073450.2.1	7	431	8	201	8	255	320	270	53	461	72	400	11
Solyc06g073460.2.1	4	242	4	132	1	337	142	355	40	291	45	173	3
Solyc06g073470.2.1	4	30	0	24	3	30	13	54	1	59	5	50	1
Solyc06g073490.1.1	5	9	0	6	0	23	11	29	4	11	10	13	0
Solyc06g073500.2.1	8	8	0	4	0	58	31	101	3	26	8	30	0
Solyc06g073510.2.1	2	1	2	6	3	0	12	3	0	12	1	11	0
Solyc06g073520.2.1	2	9	4	2	4	7	24	25	10	17	4	4	14
Solyc06g073530.1.1	0	0	0	0	0	0	0	0	0	0	0	0	0
Solyc06g073540.2.1	16	227	21	379	24	119	87	290	23	1040	218	1082	12
Solyc06g073550.2.1	14	74	8	63	10	155	55	257	6	209	75	248	0
Solyc06g073560.2.1	0	0	0	0	0	0	0	0	0	0	0	0	0
Solyc06g073580.2.1	0	0	0	0	0	0	0	0	0	0	0	0	0
Solyc06g073600.2.1	6	60	3	64	0	158	76	185	20	90	19	76	1
Solyc06g073610.2.1	0	0	0	0	0	0	0	0	0	0	0	0	0
Solyc06g073620.2.1	1	24	0	15	0	8	9	19	0	51	0	42	0
Solyc06g073630.2.1	16	207	15	195	15	638	177	654	34	379	66	272	8
Solyc06g073640.2.1	0	0	0	0	0	0	0	0	0	0	0	0	0
Solyc06g073650.1.1	4	5	0	8	1	18	11	43	2	33	6	10	0
Solyc06g073660.2.1	0	0	0	0	0	0	0	0	0	0	0	0	0
Solyc06g073670.2.1	9	48	8	55	13	68	101	92	11	148	35	105	7
Solyc06g073680.2.1	0	0	0	0	0	0	0	0	0	0	0	0	0
Solyc06g073690.2.1	0	0	0	0	0	0	0	0	0	0	0	0	0
Solyc06g073700.2.1	1	30	1	10	0	19	23	33	10	42	19	22	0
Solyc06g073720.1.1	10	216	35	197	33	528	454	588	68	590	98	379	5
Solyc06g073730.1.1	20	518	23	518	13	1243	389	1554	15	577	262	435	5
Solyc06g073740.2.1	14	101	7	89	3	696	270	1269	2	715	104	746	0
Solyc06g073750.2.1	0	0	0	0	0	0	0	0	0	0	0	0	0
Solyc06g073760.2.1	1	2	0	0	0	4	1	8	0	0	8	0	0
Solyc06g073780.2.1	26	96	28	66	13	120	319	243	58	230	66	215	6
Solyc06g073790.2.1	0	0	0	0	0	0	0	0	0	0	0	0	0
Solyc06g073800.2.1	0	0	0	0	0	0	0	0	0	0	0	0	0
Solyc06g073810.2.1	5	17	1	9	4	32	19	41	6	20	5	9	0
Solyc06g073820.1.1	0	0	0	0	0	0	0	0	0	0	0	0	0
Solyc06g073830.1.1	0	0	0	0	0	0	0	0	0	0	0	0	0
Solyc06g073840.2.1	13	105	12	54	2	400	282	358	65	159	42	85	3
Solyc06g073850.1.1	3	45	10	33	10	12	2	10	2	1	2	3	0
Solyc06g073860.1.1	4	15	1	22	1	83	55	46	2	33	8	17	0
Solyc06g073870.2.1	3	19	2	8	0	9	7	21	0	42	3	59	1

Solyc06g073880.2.1	5	35	4	28	0	86	19	53	4	19	4	36	0
Solyc06g073890.2.1	2	30	0	34	0	145	15	63	4	41	4	34	0
Solyc06g073900.2.1	0	0	0	0	0	0	0	0	0	0	0	0	0
Solyc06g073910.2.1	5	1	8	2	4	0	14	2	2	85	43	40	7
Solyc06g073920.2.1	1	12	0	2	0	23	5	12	0	364	10	288	0
Solyc06g073930.2.1	3	8	1	13	0	17	21	10	1	1	7	7	0
Solyc06g073940.2.1	0	0	0	0	0	0	0	0	0	0	0	0	0
Solyc06g073950.2.1	2	21	1	20	0	37	32	35	1	24	4	15	0
Solyc06g073960.2.1	12	117	14	69	9	189	143	188	31	169	39	76	0
Solyc06g073970.1.1	12	94	9	49	6	265	160	361	59	461	58	383	15
Solyc06g073980.2.1	1	0	0	3	1	0	4	0	0	4	0	5	3
Solyc06g073990.1.1	4	11	1	1	0	250	70	72	61	3	6	4	5
Solyc06g074000.1.1	6	148	7	85	15	14	36	7	3	208	144	317	0
Solyc06g074010.2.1	0	0	0	0	0	0	0	0	0	0	0	0	0
Solyc06g074020.2.1	0	0	0	0	0	0	0	0	0	0	0	0	0
Solyc06g074030.1.1	7	958	50	827	21	20	0	16	1	300	10	665	14
Solyc06g074040.1.1	0	0	0	0	0	0	0	0	0	0	0	0	0
Solyc06g074050.1.1	2	19	1	14	0	2	4	5	0	16	8	7	1
Solyc06g074070.2.1	7	45	8	47	2	24	13	17	6	306	23	196	6
Solyc06g074080.2.1	3	16	0	7	1	33	30	34	5	51	2	23	0
Solyc06g074090.2.1	3	397	29	83	3	110	17	170	0	81	224	41	4
Solyc06g074110.2.1	4	12	2	9	0	25	28	36	3	16	2	1	0
Solyc06g074120.2.1	20	278	30	361	21	597	301	912	81	524	40	503	8
Solyc06g074130.2.1	4	11	0	10	0	6	20	30	2	16	5	12	0
Solyc06g074140.1.1	0	0	0	0	0	0	0	0	0	0	0	0	0
Solyc06g074170.2.1	0	0	0	0	0	0	0	0	0	0	0	0	0
Solyc06g074180.1.1	0	0	0	0	0	0	0	0	0	0	0	0	0
Solyc06g074190.2.1	2	6	0	5	0	2	4	38	0	2	1	4	0
Solyc06g074200.2.1	2	3117	736	1005	9	12	159	30	5	319	230	399	33
Solyc06g074210.2.1	3	32	2	10	3	57	17	95	10	53	4	35	0
Solyc06g074220.2.1	18	56	28	44	5	383	202	281	59	278	45	138	6
Solyc06g074240.1.1	0	0	0	0	0	0	0	0	0	0	0	0	0
Solyc06g074250.2.1	4	46	0	37	6	26	15	40	1	39	7	53	0
Solyc06g074260.2.1	11	112	5	10	8	87	16	58	5	5917	91	817	252
Solyc06g074280.2.1	0	0	0	0	0	0	0	0	0	0	0	0	0
Solyc06g074290.1.1	4	19	0	1	2	35	25	50	0	339	243	294	4
Solyc06g074300.2.1	3	102	2	49	2	198	27	189	6	309	24	238	1
Solyc06g074310.2.1	4	12	1	12	0	17	16	26	0	18	7	23	0
Solyc06g074320.2.1	0	0	0	0	0	0	0	0	0	0	0	0	0
Solyc06g074330.2.1	8	27	3	18	0	39	37	47	1	30	6	37	5
Solyc06g074360.2.1	0	0	0	0	0	0	0	0	0	0	0	0	0

Solyc06g074370.1.1	3	37	11	46	5	10	56	19	14	82	16	16	0
Solyc06g074380.2.1	2	16	0	0	0	0	12	7	7	21	4	5	0
Solyc06g074390.2.1	3	16	6	0	5	40	6	3	49	4	2	0	0
Solyc06g074400.2.1	4	9	1	4	0	75	25	125	11	54	19	63	0
Solyc06g074410.2.1	0	0	0	0	0	0	0	0	0	0	0	0	0
Solyc06g074420.1.1	2	4	2	1	0	61	10	36	0	0	0	0	0
Solyc06g074430.2.1	4	82	1	42	5	123	32	165	19	108	35	192	2
Solyc06g074440.2.1	0	0	0	0	0	0	0	0	0	0	0	0	0
Solyc06g074450.1.1	12	40	3	38	0	138	43	91	14	122	28	52	0
Solyc06g074460.2.1	0	0	0	0	0	0	0	0	0	0	0	0	0
Solyc06g074470.2.1	0	0	0	0	0	0	0	0	0	0	0	0	0
Solyc06g074480.2.1	6	13	3	13	1	54	27	59	1	24	15	25	1
Solyc06g074490.1.1	0	0	0	0	0	0	0	0	0	0	0	0	0
Solyc06g074500.1.1	4	13	0	1	4	33	53	4	22	2	0	0	0
Solyc06g074510.2.1	4	51	5	38	0	15	15	12	0	11	6	18	0
Solyc06g074530.1.1	3	83	13	71	0	9	10	30	0	35	0	141	1
Solyc06g074540.2.1	2	6	1	5	10	2	13	2	3	0	7	0	3
Solyc06g074550.2.1	0	0	0	0	0	0	0	0	0	0	0	0	0
Solyc06g074560.1.1	0	0	0	0	0	0	0	0	0	0	0	0	0
Solyc06g074580.1.1	0	0	0	0	0	0	0	0	0	0	0	0	0
Solyc06g074620.2.1	4	30	9	38	4	12	19	17	10	45	2	215	2
Solyc06g074630.2.1	2	6	2	0	5	6	26	11	1	39	13	22	1
Solyc06g074640.1.1	0	0	0	0	0	0	0	0	0	0	0	0	0
Solyc06g074650.2.1	14	92	10	73	4	178	59	184	14	116	27	107	2
Solyc06g074670.2.1	5	224	48	65	28	76	31	29	0	104	36	187	12
Solyc06g074680.2.1	1	0	0	0	0	5	8	5	0	0	0	4	0
Solyc06g074690.2.1	4	11	6	5	7	26	16	28	7	18	7	8	0
Solyc06g074700.2.1	1	2	0	1	0	2	6	4	0	5	2	1	0
Solyc06g074710.1.1	5	3	0	2	1	874	58	367	15	44	29	39	0
Solyc06g074720.2.1	2	14	1	26	0	49	10	42	0	168	22	69	2
Solyc06g074730.2.1	0	0	0	0	0	0	0	0	0	0	0	0	0
Solyc06g074740.2.1	15	77	3	77	3	440	112	526	16	182	7	157	2
Solyc06g074750.1.1	0	0	0	0	0	0	0	0	0	0	0	0	0
Solyc06g074760.1.1	0	0	0	0	0	0	0	0	0	0	0	0	0
Solyc06g074770.2.1	0	0	0	0	0	0	0	0	0	0	0	0	0
Solyc06g074780.1.1	3	61	0	13	0	62	23	72	3	95	9	54	2
Solyc06g074790.1.1	7	112	1	27	0	17	62	29	10	157	24	189	2
Solyc06g074800.1.1	0	0	0	0	0	0	0	0	0	0	0	0	0
Solyc06g074810.2.1	6	11	3	4	0	26	35	18	5	35	6	21	0
Solyc06g074820.2.1	7	1994	69	627	17	245	101	409	4	904	94	629	5
Solyc06g074840.2.1	3	29	4	18	3	54	21	43	8	36	4	21	1

Solyc06g074850.2.1	2	12	0	6	0	7	7	8	0	1	3	0	0
Solyc06g074870.1.1	0	0	0	0	0	0	0	0	0	0	0	0	0
Solyc06g074890.1.1	5	81	12	51	0	6	32	19	0	39	1	33	0
Solyc06g074900.1.1	2	8	4	4	0	2	17	9	2	18	0	3	0
Solyc06g074940.2.1	3	9	0	13	0	162	5	65	4	29	7	36	0
Solyc06g074960.2.1	3	15	1	17	5	25	15	22	2	24	6	16	0
Solyc06g074970.1.1	3	5	2	5	0	5	7	16	0	24	11	21	0
Solyc06g074980.2.1	9	303	20	335	32	1032	441	1593	110	701	163	545	12
Solyc06g074990.1.1	0	0	0	0	0	0	0	0	0	0	0	0	0
Solyc06g075000.2.1	2	4	0	3	0	29	11	23	1	13	1	12	0
Solyc06g075010.2.1	8	81	6	31	10	88	80	127	5	213	51	164	18
Solyc06g075020.2.1	0	0	0	0	0	0	0	0	0	0	0	0	0
Solyc06g075030.1.1	1	3	2	4	0	0	0	1	0	1	0	2	0
Solyc06g075080.2.1	5	44	3	25	2	25	16	19	0	65	20	58	1
Solyc06g075090.2.1	1	10	1	1	0	0	4	2	1	0	0	0	0
Solyc06g075100.2.1	0	0	0	0	0	0	0	0	0	0	0	0	0
Solyc06g075110.2.1	8	55	7	33	0	157	105	119	33	127	35	119	6
Solyc06g075130.2.1	0	0	0	0	0	0	0	0	0	0	0	0	0
Solyc06g075140.2.1	0	0	0	0	0	0	0	0	0	0	0	0	0
Solyc06g075150.2.1	0	0	0	0	0	0	0	0	0	0	0	0	0
Solyc06g075160.2.1	11	169	17	98	32	188	97	139	44	75	15	37	0
Solyc06g075170.1.1	3	47	1	8	0	19	19	19	1	23	6	21	0
Solyc06g075180.1.1	5	205	16	81	15	208	66	183	40	517	53	399	78
Solyc06g075190.1.1	4	42	7	42	5	39	11	16	1	75	13	63	0
Solyc06g075220.1.1	2	23	3	16	0	12	11	43	0	6	3	1	0
Solyc06g075250.2.1	7	69	2	59	0	91	50	80	0	129	26	132	0
Solyc06g075310.2.1	1	0	0	1	0	4	5	2	2	1	0	3	0
Solyc06g075320.2.1	0	0	0	0	0	0	0	0	0	0	0	0	0
Solyc06g075330.1.1	2	2	0	0	0	29	12	0	14	1	0	0	0
Solyc06g075340.2.1	18	190	26	202	6	346	297	830	40	894	112	521	8
Solyc06g075350.1.1	0	0	0	0	0	0	0	0	0	0	0	0	0
Solyc06g075360.2.1	6	61	2	55	3	196	72	237	24	216	13	279	1
Solyc06g075370.2.1	3	24	5	16	0	83	20	97	1	16	0	13	0
Solyc06g075390.2.1	2	17	4	8	0	8	10	19	4	47	9	23	2
Solyc06g075400.2.1	3	8	0	5	0	24	11	42	4	20	3	5	0
Solyc06g075410.2.1	0	0	0	0	0	0	0	0	0	0	0	0	0
Solyc06g075420.1.1	0	0	0	0	0	0	0	0	0	0	0	0	0
Solyc06g075440.1.1	0	0	0	0	0	0	0	0	0	0	0	0	0
Solyc06g075470.1.1	0	0	0	0	0	0	0	0	0	0	0	0	0
Solyc06g075480.2.1	0	0	0	0	0	0	0	0	0	0	0	0	0
Solyc06g075500.2.1	0	0	0	0	0	0	0	0	0	0	0	0	0

Solyc06g075510.2.1	0	0	0	0	0	0	0	0	0	0	0	0	0
Solyc06g075520.2.1	0	0	0	0	0	0	0	0	0	0	0	0	0
Solyc06g075530.1.1	0	0	0	0	0	0	0	0	0	0	0	0	0
Solyc06g075540.2.1	1	19	3	7	0	71	3	34	1	81	0	38	0
Solyc06g075550.2.1	1	3	0	4	0	48	4	16	1	20	8	14	0
Solyc06g075570.1.1	0	0	0	0	0	0	0	0	0	0	0	0	0
Solyc06g075580.2.1	2	11	1	3	0	1	3	3	1	18	8	14	1
Solyc06g075590.2.1	7	51	7	28	5	125	97	99	12	123	22	112	4
Solyc06g075600.2.1	0	0	0	0	0	0	0	0	0	0	0	0	0
Solyc06g075610.1.1	22	392	199	326	31	367	210	136	22	15	31	73	5
Solyc06g075620.2.1	1	5	0	0	0	16	3	18	0	2	0	3	0
Solyc06g075630.2.1	0	0	0	0	0	0	0	0	0	0	0	0	0
Solyc06g075640.1.1	0	0	0	0	0	0	0	0	0	0	0	0	0
Solyc06g075650.2.1	2	50	5	8	2	9	28	10	8	0	0	0	0
Solyc06g075660.2.1	3	15	8	31	0	60	50	50	0	1	2	0	0
Solyc06g075670.1.1	0	0	0	0	0	0	0	0	0	0	0	0	0
Solyc06g075680.1.1	0	0	0	0	0	0	0	0	0	0	0	0	0
Solyc06g075690.2.1	2	47	3	26	1	24	4	10	0	0	1	29	0
Solyc06g075780.1.1	3	85	11	54	0	26	8	29	3	7	2	30	0
Solyc06g075790.2.1	9	33	7	46	3	19	25	42	1	111	12	75	1
Solyc06g075800.1.1	7	121	18	73	10	57	267	112	47	77	143	133	16
Solyc06g075810.2.1	1	31	1	13	0	59	18	53	5	75	30	50	4
Solyc06g075830.1.1	0	0	0	0	0	0	0	0	0	0	0	0	0
Solyc06g075850.1.1	0	0	0	0	0	0	0	0	0	0	0	0	0
Solyc06g075930.1.1	0	0	0	0	0	0	0	0	0	0	0	0	0
Solyc06g075960.1.1	0	0	0	0	0	0	0	0	0	0	0	0	0
Solyc06g075980.2.1	7	28	1	13	1	78	34	49	4	155	5	80	0
Solyc06g075990.2.1	2	0	0	0	2	35	25	10	0	0	1	0	1
Solyc06g076010.2.1	0	0	0	0	0	0	0	0	0	0	0	0	0
Solyc06g076020.2.1	11	319	29	228	8	1373	69	326	35	40	60	104	1
Solyc06g076030.2.1	0	0	0	0	0	0	0	0	0	0	0	0	0
Solyc06g076040.2.1	15	152	43	97	7	63	40	153	11	288	31	599	3
Solyc06g076050.2.1	13	90	16	54	12	53	43	28	22	140	49	229	22
Solyc06g076060.1.1	0	0	0	0	0	0	0	0	0	0	0	0	0
Solyc06g076080.1.1	4	71	10	17	7	1	1	1	7	1	0	43	3
Solyc06g076090.2.1	0	0	0	0	0	0	0	0	0	0	0	0	0
Solyc06g076100.2.1	8	22	3	24	2	26	14	31	9	29	10	11	1
Solyc06g076110.1.1	0	0	0	0	0	0	0	0	0	0	0	0	0
Solyc06g076120.2.1	0	0	0	0	0	0	0	0	0	0	0	0	0
Solyc06g076130.2.1	7	27	12	47	6	67	25	50	8	1	5	12	0
Solyc06g076140.2.1	0	0	0	0	0	0	0	0	0	0	0	0	0

Solyc06g076150.2.1	15	102	17	75	15	273	195	424	97	253	78	224	20
Solyc06g076160.2.1	3	5	0	1	3	12	30	19	41	2	9	1	1
Solyc06g076170.2.1	0	0	0	0	0	0	0	0	0	0	0	0	0
Solyc06g076220.2.1	1	5	0	0	2	0	0	0	0	0	0	1	0
Solyc06g076230.1.1	0	0	0	0	0	0	0	0	0	0	0	0	0
Solyc06g076250.2.1	3	38	7	28	0	57	28	53	0	22	3	28	1
Solyc06g076260.2.1	0	0	0	0	0	0	0	0	0	0	0	0	0
Solyc06g076270.2.1	0	0	0	0	0	0	0	0	0	0	0	0	0
Solyc06g076280.1.1	6	62	15	104	1	37	66	63	5	27	18	52	1
Solyc06g076290.1.1	0	0	0	0	0	0	0	0	0	0	0	0	0
Solyc06g076300.2.1	3	13	0	5	0	12	5	7	5	9	14	28	3
Solyc06g076320.1.1	0	0	0	0	0	0	0	0	0	0	0	0	0
Solyc06g076330.2.1	0	0	0	0	0	0	0	0	0	0	0	0	0
Solyc06g076340.2.1	15	87	12	105	8	433	209	563	34	204	80	194	7
Solyc06g076350.2.1	8	17	0	7	2	49	57	95	7	194	11	94	2
Solyc06g076360.2.1	19	196	9	93	6	308	95	499	23	532	48	368	3
Solyc06g076390.2.1	2	21	1	40	0	58	23	68	3	23	12	28	2
Solyc06g076400.2.1	12	287	8	132	3	2707	450	1008	59	96	24	102	0
Solyc06g076410.2.1	4	47	7	32	0	20	10	14	1	173	28	152	2
Solyc06g076420.1.1	0	0	0	0	0	0	0	0	0	0	0	0	0
Solyc06g076430.2.1	4	19	5	6	0	18	45	20	5	52	14	40	0
Solyc06g076440.1.1	1	8	1	3	1	5	4	3	0	7	0	4	0
Solyc06g076450.2.1	0	0	0	0	0	0	0	0	0	0	0	0	0
Solyc06g076460.2.1	2	30	2	12	0	34	12	43	0	67	9	26	0
Solyc06g076470.2.1	3	80	7	46	0	4	7	9	0	3	0	3	0
Solyc06g076480.2.1	6	262	18	157	2	500	87	637	2	188	4	180	0
Solyc06g076490.2.1	7	64	4	74	10	186	22	180	9	103	15	46	0
Solyc06g076510.2.1	2	6	1	0	0	10	12	8	3	21	6	21	1
Solyc06g076520.1.1	2	68	1	77	0	106	7	65	1	471	49	291	0
Solyc06g076540.1.1	0	0	0	0	0	0	0	0	0	0	0	0	0
Solyc06g076560.1.1	4	18	4	17	0	5	2	9	0	63	17	80	0
Solyc06g076570.1.1	4	107	4	69	0	35	0	63	0	607	22	442	0
Solyc06g076580.1.1	1	2	1	2	0	0	0	0	0	30	4	18	0
Solyc06g076590.2.1	4	10	0	11	0	15	20	24	0	22	6	15	1
Solyc06g076600.1.1	0	0	0	0	0	0	0	0	0	0	0	0	0
Solyc06g076610.2.1	2	5	1	0	0	5	6	4	0	9	3	8	0
Solyc06g076620.2.1	2	10	1	1	0	5	5	16	1	11	4	4	0
Solyc06g076630.2.1	12	101	7	26	0	50	85	108	0	52	52	49	1
Solyc06g076640.2.1	8	331	20	109	6	157	88	380	12	557	175	398	1
Solyc06g076650.2.1	6	21	3	6	3	48	36	64	7	59	17	37	0
Solyc06g076660.2.1	3	13	3	10	2	4	16	20	4	12	24	23	2

Solyc06g076670.2.1	15	149	26	65	6	707	256	604	43	231	78	177	5
Solyc06g076680.2.1	0	0	0	0	0	0	0	0	0	0	0	0	0
Solyc06g076690.2.1	1	5	0	4	0	6	8	9	1	9	1	0	0
Solyc06g076700.1.1	0	0	0	0	0	0	0	0	0	0	0	0	0
Solyc06g076710.1.1	0	0	0	0	0	0	0	0	0	0	0	0	0
Solyc06g076740.1.1	0	0	0	0	0	0	0	0	0	0	0	0	0
Solyc06g076750.2.1	14	203	16	258	2	611	137	723	6	507	41	246	1
Solyc06g076760.1.1	0	0	0	0	0	0	0	0	0	0	0	0	0
Solyc06g076770.2.1	4	114	29	43	1	72	230	116	9	240	35	201	1
Solyc06g076780.2.1	13	55	2	37	0	241	128	295	27	153	57	122	4
Solyc06g076790.1.1	0	0	0	0	0	0	0	0	0	0	0	0	0
Solyc06g076800.2.1	0	0	0	0	0	0	0	0	0	0	0	0	0
Solyc06g076820.1.1	18	216	12	133	13	63	108	113	45	387	48	186	2
Solyc06g076850.2.1	13	658	67	415	17	288	760	668	28	291	58	242	0
Solyc06g076860.2.1	0	0	0	0	0	0	0	0	0	0	0	0	0
Solyc06g076870.2.1	0	0	0	0	0	0	0	0	0	0	0	0	0
Solyc06g076880.2.1	3	12	4	10	0	36	32	30	1	93	9	75	0
Solyc06g076890.1.1	4	39	1	17	3	36	12	33	5	62	4	51	1
Solyc06g076900.2.1	1	1	0	0	0	7	3	12	4	1	2	5	0
Solyc06g076910.1.1	0	0	0	0	0	0	0	0	0	0	0	0	0
Solyc06g076930.1.1	0	0	0	0	0	0	0	0	0	0	0	0	0
Solyc06g076940.2.1	2	32	2	17	0	32	44	45	8	80	17	35	0
Solyc06g076950.2.1	2	0	1	5	0	6	5	2	1	1	0	4	0
Solyc06g076960.1.1	2	1	0	0	2	3	6	5	0	23	6	7	3
Solyc06g076970.2.1	4	250	15	100	1	550	194	545	39	592	69	620	4
Solyc06g081980.1.1	7	593	46	181	1	138	77	168	14	203	103	168	4
Solyc06g082000.2.1	0	0	0	0	0	0	0	0	0	0	0	0	0
Solyc06g082010.2.1	19	262	44	322	20	1780	930	1113	76	400	48	262	14
Solyc06g082030.2.1	0	0	0	0	0	0	0	0	0	0	0	0	0
Solyc06g082040.1.1	0	0	0	0	0	0	0	0	0	0	0	0	0
Solyc06g082050.1.1	0	0	0	0	0	0	0	0	0	0	0	0	0
Solyc06g082060.2.1	0	0	0	0	0	0	0	0	0	0	0	0	0
Solyc06g082070.2.1	4	22	4	21	1	8	13	6	0	39	18	18	0
Solyc06g082080.2.1	2	3	1	0	0	5	0	2	0	0	5	2	0
Solyc06g082090.2.1	8	362	30	201	13	934	201	833	64	652	113	515	11
Solyc06g082100.2.1	28	196	31	217	20	538	439	974	52	470	139	415	8
Solyc06g082120.2.1	4	76	9	33	10	218	127	119	22	296	65	167	0
Solyc06g082140.2.1	0	0	0	0	0	0	0	0	0	0	0	0	0
Solyc06g082150.2.1	16	114	28	57	6	122	140	136	23	59	15	43	1
Solyc06g082160.2.1	3	9	6	6	0	6	28	27	3	25	4	3	0
Solyc06g082170.2.1	0	0	0	0	0	0	0	0	0	0	0	0	0

Solyc06g082180.1.1	4	26	0	17	0	16	18	19	3	22	19	34	3
Solyc06g082190.2.1	0	0	0	0	0	0	0	0	0	0	0	0	0
Solyc06g082200.2.1	5	19	2	8	0	16	39	44	3	62	7	36	1
Solyc06g082210.1.1	4	15	1	12	0	19	2	8	0	85	18	33	0
Solyc06g082220.2.1	10	257	18	110	14	435	128	442	46	269	98	155	0
Solyc06g082230.2.1	0	0	0	0	0	0	0	0	0	0	0	0	0
Solyc06g082240.2.1	0	0	0	0	0	0	0	0	0	0	0	0	0
Solyc06g082270.1.1	5	49	3	22	0	83	31	73	14	70	13	43	0
Solyc06g082280.2.1	9	97	8	52	0	111	24	98	1	146	18	85	2
Solyc06g082290.2.1	3	17	3	6	0	40	8	30	6	22	6	33	0
Solyc06g082300.2.1	1	7	1	9	0	3	3	10	0	4	0	1	0
Solyc06g082320.2.1	0	0	0	0	0	0	0	0	0	0	0	0	0
Solyc06g082360.2.1	6	47	9	12	13	106	80	78	7	132	51	94	4
Solyc06g082370.2.1	8	21	3	13	0	67	49	123	13	120	14	76	1
Solyc06g082380.2.1	3	43	2	34	5	75	69	40	3	25	3	14	1
Solyc06g082390.2.1	9	77	17	59	2	171	83	166	5	280	58	245	3
Solyc06g082410.1.1	0	0	0	0	0	0	0	0	0	0	0	0	0
Solyc06g082440.1.1	3	21	5	5	11	77	91	39	131	16	7	21	8
Solyc06g082460.1.1	1	0	0	0	0	0	6	0	0	30	8	9	0
Solyc06g082470.2.1	17	42	6	62	0	287	215	478	19	112	56	73	1
Solyc06g082480.2.1	6	7	3	33	0	76	63	71	4	3	5	6	0
Solyc06g082490.2.1	3	39	1	21	3	70	16	46	5	104	5	46	0
Solyc06g082500.2.1	2	11	0	2	0	15	3	20	0	50	8	14	0
Solyc06g082510.2.1	7	61	1	72	0	131	21	115	5	275	11	256	4
Solyc06g082520.2.1	11	38	2	46	2	127	76	96	14	62	27	43	3
Solyc06g082530.1.1	1	0	0	4	0	10	4	0	0	0	0	0	0
Solyc06g082560.1.1	0	0	0	0	0	0	0	0	0	0	0	0	0
Solyc06g082570.1.1	3	19	6	0	16	52	6	0	3	6	1	1	1
Solyc06g082580.2.1	8	204	26	77	26	320	133	300	46	368	86	213	1
Solyc06g082590.1.1	1	18	10	39	4	8	6	14	2	2	2	3	0
Solyc06g082600.2.1	2	215	0	73	0	125	51	166	0	242	23	210	0
Solyc06g082610.2.1	2	6	1	6	0	1	2	5	0	29	3	23	0
Solyc06g082620.1.1	4	191	18	82	27	109	151	210	33	996	274	1047	52
Solyc06g082630.2.1	1	17	1	2	0	50	74	42	16	55	9	28	0
Solyc06g082650.2.1	0	0	0	0	0	0	0	0	0	0	0	0	0
Solyc06g082660.2.1	2	11	3	16	5	14	59	32	21	22	14	4	1
Solyc06g082670.2.1	1	9	1	2	1	7	0	21	0	20	6	15	0
Solyc06g082680.2.1	2	45	0	28	0	57	22	81	1	93	6	54	0
Solyc06g082700.1.1	0	0	0	0	0	0	0	0	0	0	0	0	0
Solyc06g082710.2.1	6	31	2	16	0	63	16	53	1	61	25	47	7
Solyc06g082730.2.1	0	0	0	0	0	0	0	0	0	0	0	0	0

Solyc06g082750.2.1	4	233	2	174	1	301	44	195	0	101	4	148	2
Solyc06g082760.2.1	1	19	2	6	0	1	48	9	1	5	10	15	0
Solyc06g082770.2.1	0	0	0	0	0	0	0	0	0	0	0	0	0
Solyc06g082780.2.1	4	6	9	4	9	66	11	16	0	5	1	4	0
Solyc06g082800.2.1	5	16	1	9	3	24	16	28	4	59	6	34	1
Solyc06g082810.2.1	0	0	0	0	0	0	0	0	0	0	0	0	0
Solyc06g082830.2.1	4	110	5	45	6	83	60	126	5	454	28	336	1
Solyc06g082840.2.1	12	108	12	268	9	621	207	423	7	580	35	295	9
Solyc06g082850.2.1	0	0	0	0	0	0	0	0	0	0	0	0	0
Solyc06g082860.2.1	0	0	0	0	0	0	0	0	0	0	0	0	0
Solyc06g082870.2.1	8	230	26	144	17	326	235	591	49	821	189	760	30
Solyc06g082880.1.1	0	0	0	0	0	0	0	0	0	0	0	0	0
Solyc06g082890.2.1	1	10	0	0	0	2	3	5	0	8	2	3	0
Solyc06g082900.2.1	2	4	0	2	0	16	13	14	1	10	1	5	0
Solyc06g082910.2.1	7	90	6	54	0	7	16	35	0	79	19	46	0
Solyc06g082920.2.1	16	244	33	161	28	530	442	601	108	118	86	131	3
Solyc06g082930.2.1	13	213	22	137	18	295	183	279	45	446	141	411	11
Solyc06g082940.2.1	7	8843	608	4458	36	115	1051	215	10	1188	449	1169	49
Solyc06g082950.2.1	8	2064	382	1324	16	46	175	65	2	251	75	186	3
Solyc06g082960.2.1	0	0	0	0	0	0	0	0	0	0	0	0	0
Solyc06g082970.2.1	4	151	7	49	0	7	36	24	2	8	5	49	1
Solyc06g082980.2.1	10	2415	109	1455	12	226	201	1045	28	813	620	475	8
Solyc06g082990.2.1	1	0	0	0	0	0	3	0	0	5	0	5	0
Solyc06g083000.1.1	0	0	0	0	0	0	0	0	0	0	0	0	0
Solyc06g083010.1.1	1	3	2	0	0	11	8	5	0	5	2	11	0
Solyc06g083020.1.1	0	0	0	0	0	0	0	0	0	0	0	0	0
Solyc06g083030.2.1	3	19	3	4	6	6	13	18	6	4	15	3	1
Solyc06g083040.2.1	8	272	4	220	2	84	134	114	1	2	2	2	0
Solyc06g083050.2.1	0	0	0	0	0	0	0	0	0	0	0	0	0
Solyc06g083060.2.1	0	0	0	0	0	0	0	0	0	0	0	0	0
Solyc06g083070.2.1	7	137	7	58	5	0	23	16	1	71	4	57	0
Solyc06g083080.2.1	4	29	2	3	0	7	25	6	0	12	3	20	0
Solyc06g083090.2.1	0	0	0	0	0	0	0	0	0	0	0	0	0
Solyc06g083100.1.1	0	0	0	0	0	0	0	0	0	0	0	0	0
Solyc06g083110.1.1	6	83	8	54	0	44	63	189	2	69	6	126	3
Solyc06g083120.1.1	0	0	0	0	0	0	0	0	0	0	0	0	0
Solyc06g083130.2.1	0	0	0	0	0	0	0	0	0	0	0	0	0
Solyc06g083140.2.1	0	0	0	0	0	0	0	0	0	0	0	0	0
Solyc06g083150.2.1	5	25	10	10	1	15	35	22	1	40	18	57	3
Solyc06g083170.2.1	0	0	0	0	0	0	0	0	0	0	0	0	0
Solyc06g083180.2.1	8	330	16	104	5	447	66	544	32	360	61	375	10

Solyc06g083190.2.1	22	462	28	382	8	1428	517	1708	67	730	203	639	8
Solyc06g083200.1.1	0	0	0	0	0	0	0	0	0	0	0	0	0
Solyc06g083210.2.1	9	29	10	15	2	83	120	79	5	38	13	45	4
Solyc06g083220.2.1	18	21	9	22	1	101	138	136	53	84	70	117	4
Solyc06g083230.2.1	4	25	7	31	1	28	8	34	2	12	7	17	0
Solyc06g083250.2.1	4	6	3	6	0	3	6	13	1	7	0	16	0
Solyc06g083260.2.1	3	31	3	19	2	159	73	133	11	73	11	59	2
Solyc06g083270.2.1	2	0	0	4	0	11	4	13	0	0	5	0	0
Solyc06g083280.1.1	0	0	0	0	0	0	0	0	0	0	0	0	0
Solyc06g083290.1.1	0	0	0	0	0	0	0	0	0	0	0	0	0
Solyc06g083300.2.1	14	426	28	391	15	519	253	714	84	2034	159	1386	14
Solyc06g083310.2.1	9	127	11	104	18	310	92	188	30	339	76	289	11
Solyc06g083330.2.1	0	0	0	0	0	0	0	0	0	0	0	0	0
Solyc06g083340.2.1	1	174	2	40	2	146	15	162	4	159	6	105	2
Solyc06g083350.2.1	0	0	0	0	0	0	0	0	0	0	0	0	0
Solyc06g083360.2.1	2	127	5	54	0	152	24	182	6	202	22	203	1
Solyc06g083370.2.1	0	0	0	0	0	0	0	0	0	0	0	0	0
Solyc06g083380.2.1	4	12	1	12	7	25	30	23	9	27	19	26	3
Solyc06g083390.2.1	5	264	83	208	21	151	59	158	6	105	11	73	5
Solyc06g083410.2.1	0	0	0	0	0	0	0	0	0	0	0	0	0
Solyc06g083420.1.1	19	57	7	59	4	261	185	339	31	136	32	97	4
Solyc06g083430.1.1	1	1	0	0	0	2	0	4	0	5	5	3	0
Solyc06g083440.2.1	1	24	9	10	14	24	55	24	7	116	97	54	15
Solyc06g083460.2.1	13	122	13	101	13	213	170	284	16	429	86	284	2
Solyc06g083470.2.1	0	0	0	0	0	0	0	0	0	0	0	0	0
Solyc06g083500.2.1	0	0	0	0	0	0	0	0	0	0	0	0	0
Solyc06g083510.2.1	5	20	2	11	1	67	30	43	2	44	6	37	1
Solyc06g083520.2.1	5	19	2	13	4	41	32	89	13	32	17	28	0
Solyc06g083530.2.1	2	85	4	35	6	91	27	90	18	198	54	160	3
Solyc06g083540.2.1	1	16	1	1	0	31	1	24	0	31	4	21	3
Solyc06g083550.1.1	4	27	2	31	0	45	6	13	3	23	0	7	0
Solyc06g083570.2.1	0	0	0	0	0	0	0	0	0	0	0	0	0
Solyc06g083580.2.1	6	56	0	45	7	3	2	1	0	304	41	239	0
Solyc06g083620.2.1	12	402	27	253	20	879	452	976	132	919	131	602	8
Solyc06g083630.2.1	5	46	5	25	2	107	35	81	1	69	11	42	0
Solyc06g083640.2.1	3	7	1	4	0	4	17	17	3	9	9	17	0
Solyc06g083650.2.1	2	36	1	5	10	6	14	3	7	5	0	0	0
Solyc06g083660.2.1	12	192	17	112	47	44	82	46	21	311	66	330	4
Solyc06g083670.2.1	5	33	6	34	0	110	7	93	0	73	0	28	0
Solyc06g083680.2.1	1	111	10	80	0	5	4	32	0	26	19	18	0
Solyc06g083690.2.1	6	245	5	123	0	169	85	164	4	146	18	152	4

Solyc06g083700.2.1	0	0	0	0	0	0	0	0	0	0	0	0	0
Solyc06g083710.1.1	0	0	0	0	0	0	0	0	0	0	0	0	0
Solyc06g083720.1.1	0	0	0	0	0	0	0	0	0	0	0	0	0
Solyc06g083730.2.1	5	57	14	25	7	204	65	142	12	133	55	116	1
Solyc06g083740.1.1	2	4	2	2	0	12	0	28	3	12	11	14	1
Solyc06g083750.2.1	1	3	0	4	0	16	2	11	0	7	2	7	0
Solyc06g083760.2.1	4	20	3	10	4	12	22	24	6	9	3	15	3
Solyc06g083770.2.1	3	26	5	9	2	14	11	29	0	33	0	12	0
Solyc06g083780.2.1	1	31	0	22	0	26	14	41	5	54	25	74	6
Solyc06g083790.2.1	8	106	15	74	7	279	140	301	27	326	66	284	1
Solyc06g083800.2.1	0	0	0	0	0	0	0	0	0	0	0	0	0
Solyc06g083810.2.1	3	43	1	27	9	40	20	78	3	72	11	135	0
Solyc06g083820.2.1	4	100	2	41	4	110	18	158	2	73	14	71	0
Solyc06g083830.2.1	10	106	3	45	2	268	95	244	8	223	21	159	0
Solyc06g083840.2.1	0	0	0	0	0	0	0	0	0	0	0	0	0
Solyc06g083850.2.1	0	0	0	0	0	0	0	0	0	0	0	0	0
Solyc06g083860.2.1	1	10	0	2	0	2	4	1	0	16	0	7	0
Solyc06g083870.2.1	7	91	3	51	5	146	41	188	14	111	17	91	1
Solyc06g083880.2.1	0	0	0	0	0	0	0	0	0	0	0	0	0
Solyc06g083890.2.1	5	19	1	26	0	30	24	38	0	40	5	31	0
Solyc06g083900.2.1	0	0	0	0	0	0	0	0	0	0	0	0	0
Solyc06g083920.2.1	0	0	0	0	0	0	0	0	0	0	0	0	0
Solyc06g083930.1.1	0	0	0	0	0	0	0	0	0	0	0	0	0
Solyc06g083960.1.1	0	0	0	0	0	0	0	0	0	0	0	0	0
Solyc06g083980.1.1	8	89	14	55	11	343	79	229	29	121	33	70	1
Solyc06g083990.2.1	1	1	0	1	0	3	4	0	1	5	0	6	0
Solyc06g084000.2.1	3	52	5	34	3	131	29	148	11	108	7	75	0
Solyc06g084010.2.1	5	50	8	39	1	209	34	390	9	145	9	115	2
Solyc06g084020.2.1	7	174	6	87	18	188	176	378	43	1075	153	998	31
Solyc06g084030.2.1	3	13	1	0	0	16	7	15	0	127	5	49	0
Solyc06g084040.2.1	0	0	0	0	0	0	0	0	0	0	0	0	0
Solyc06g084050.2.1	5	202	30	83	0	0	19	7	0	27	17	51	0
Solyc06g084060.2.1	7	7	0	19	1	20	16	35	4	20	12	28	0
Solyc06g084070.2.1	0	0	0	0	0	0	0	0	0	0	0	0	0
Solyc06g084080.2.1	0	0	0	0	0	0	0	0	0	0	0	0	0
Solyc06g084090.2.1	1	3	0	0	0	0	0	3	0	19	4	13	0
Solyc06g084100.2.1	0	0	0	0	0	0	0	0	0	0	0	0	0
Solyc06g084120.2.1	0	0	0	0	0	0	0	0	0	0	0	0	0
Solyc06g084130.2.1	0	0	0	0	0	0	0	0	0	0	0	0	0
Solyc06g084140.2.1	6	65	31	35	1	6	4	7	0	13	1	7	0
Solyc06g084150.2.1	4	18	1	4	4	48	16	17	6	99	6	23	0

Solyc06g084160.2.1	4	73	9	50	0	67	40	111	7	87	37	44	3
Solyc06g084170.2.1	2	4	0	0	0	24	27	7	19	8	5	0	0
Solyc06g084180.2.1	0	0	0	0	0	0	0	0	0	0	0	0	0
Solyc06g084190.2.1	2	4	3	0	9	43	66	6	58	5	5	0	0
Solyc06g084200.1.1	4	27	1	30	0	93	35	103	3	71	10	54	0
Solyc06g084210.2.1	14	113	14	76	2	203	210	341	47	348	92	365	10
Solyc06g084220.1.1	0	0	0	0	0	0	0	0	0	0	0	0	0
Solyc06g084230.2.1	2	64	6	23	0	105	24	122	6	237	20	96	4
Solyc06g084240.1.1	0	0	0	0	0	0	0	0	0	0	0	0	0
Solyc06g084250.2.1	5	8	2	13	0	0	34	11	2	49	14	63	2
Solyc06g084260.2.1	2	3	2	1	0	18	29	11	1	6	4	11	3
Solyc06g084270.2.1	8	111	18	79	6	165	207	205	17	180	18	129	0
Solyc06g084290.2.1	6	46	1	30	2	16	13	39	6	88	21	43	6
Solyc06g084300.1.1	0	0	0	0	0	0	0	0	0	0	0	0	0
Solyc06g084310.2.1	0	0	0	0	0	0	0	0	0	0	0	0	0
Solyc06g084320.1.1	5	16	4	13	2	48	46	39	5	26	1	18	1
Solyc06g084350.2.1	0	0	0	0	0	0	0	0	0	0	0	0	0
Solyc06g084360.2.1	3	61	3	72	0	60	64	101	2	122	7	118	4
Solyc06g084370.1.1	0	0	0	0	0	0	0	0	0	0	0	0	0
Solyc06g084380.2.1	0	0	0	0	0	0	0	0	0	0	0	0	0
Solyc06g084390.2.1	1	7	0	1	1	40	4	30	1	46	10	31	0
Solyc06g084400.2.1	7	33	8	6	4	11	92	29	14	56	74	71	4
Solyc06g084410.2.1	6	69	6	44	6	101	73	137	8	76	22	91	2
Solyc06g084420.1.1	0	0	0	0	0	0	0	0	0	0	0	0	0
Solyc06g084430.2.1	1	21	1	20	0	12	3	2	0	6	2	41	0
Solyc06g084440.2.1	8	172	12	96	11	551	102	319	27	345	48	252	5
Solyc06g084450.2.1	0	0	0	0	0	0	0	0	0	0	0	0	0
Solyc06g084460.2.1	5	42	0	18	0	20	20	32	0	42	3	20	0
Solyc06g084470.2.1	1	13	0	8	0	0	7	6	0	9	0	18	0
Solyc06g084480.2.1	3	14	0	5	0	4	27	7	0	9	2	8	0
Solyc06g084500.2.1	3	27	4	20	1	46	41	68	18	44	12	29	5
Solyc06g084510.2.1	3	69	6	45	0	69	47	75	12	81	31	106	0
Solyc06g084520.2.1	15	112	17	102	13	209	112	278	52	217	73	232	4
Solyc06g084530.2.1	12	50	10	50	0	118	131	224	18	113	23	57	1
Solyc06g084540.2.1	1	9	0	2	0	0	12	6	2	43	17	8	0
Solyc06g084550.2.1	2	3	0	0	0	6	5	8	0	19	0	14	0
Solyc06g084570.2.1	9	38	3	11	0	73	63	111	6	45	13	26	1
Solyc06g084580.1.1	0	0	0	0	0	0	0	0	0	0	0	0	0
Solyc06g084590.2.1	5	17	2	13	2	70	29	53	6	30	11	20	0
Solyc06g084600.1.1	1	3	0	0	0	1	6	3	0	0	0	1	0
Solyc06g084610.2.1	2	29	3	3	0	8	28	9	3	1	3	3	0

Solyc06g084620.1.1	0	0	0	0	0	0	0	0	0	0	0	0	0
Solyc06g084760.1.1	0	0	0	0	0	0	0	0	0	0	0	0	0
Solyc06g084820.1.1	4	26	4	14	8	14	23	16	23	0	0	0	0
Solyc07g005000.2.1	5	31	1	21	2	43	37	51	0	44	11	33	0
Solyc07g005010.2.1	8	51	12	52	4	30	43	80	4	12	16	16	0
Solyc07g005020.2.1	2	11	2	1	0	1	4	5	1	15	10	15	0
Solyc07g005030.2.1	11	38	2	29	0	43	113	81	17	103	7	80	0
Solyc07g005040.2.1	0	0	0	0	0	0	0	0	0	0	0	0	0
Solyc07g005050.2.1	2	47	3	8	0	65	41	58	6	100	16	51	1
Solyc07g005060.2.1	1	2	0	4	0	3	3	7	3	13	1	7	0
Solyc07g005090.2.1	1	8	0	2	0	4	6	10	0	0	0	0	0
Solyc07g005100.2.1	2	1	0	2	0	21	0	15	0	3	7	4	0
Solyc07g005110.2.1	0	0	0	0	0	0	0	0	0	0	0	0	0
Solyc07g005120.2.1	2	19	0	20	0	33	16	37	0	13	0	13	0
Solyc07g005130.2.1	3	7	1	0	0	8	13	26	3	6	2	0	0
Solyc07g005140.2.1	1	4	1	5	0	8	3	8	0	15	1	6	0
Solyc07g005150.1.1	0	0	0	0	0	0	0	0	0	0	0	0	0
Solyc07g005170.1.1	0	0	0	0	0	0	0	0	0	0	0	0	0
Solyc07g005180.2.1	1	25	1	32	6	50	1	38	0	56	0	27	3
Solyc07g005190.2.1	2	15	0	3	2	5	4	7	0	2	11	0	0
Solyc07g005200.2.1	0	0	0	0	0	0	0	0	0	0	0	0	0
Solyc07g005210.2.1	1	50	7	41	0	30	67	46	2	31	3	12	0
Solyc07g005220.1.1	0	0	0	0	0	0	0	0	0	0	0	0	0
Solyc07g005230.2.1	2	14	6	9	0	15	11	24	5	29	8	29	0
Solyc07g005240.2.1	2	3	0	6	0	7	8	5	0	4	2	5	0
Solyc07g005270.2.1	0	0	0	0	0	0	0	0	0	0	0	0	0
Solyc07g005280.2.1	0	0	0	0	0	0	0	0	0	0	0	0	0
Solyc07g005290.1.1	0	0	0	0	0	0	0	0	0	0	0	0	0
Solyc07g005300.1.1	0	0	0	0	0	0	0	0	0	0	0	0	0
Solyc07g005310.1.1	0	0	0	0	0	0	0	0	0	0	0	0	0
Solyc07g005320.2.1	27	199	22	113	9	466	290	611	45	239	84	181	4
Solyc07g005330.2.1	16	383	23	239	39	1801	258	2109	148	174	100	182	10
Solyc07g005340.1.1	16	182	17	155	12	483	205	434	75	334	75	175	4
Solyc07g005360.2.1	13	66	4	46	11	177	107	126	23	91	30	43	1
Solyc07g005370.2.1	5	208	21	58	1	7	0	0	0	1	0	2	0
Solyc07g005390.2.1	9	2866	317	1912	168	1479	154	987	69	398	378	458	15
Solyc07g005400.2.1	1	3	0	3	0	2	0	2	0	1	10	5	0
Solyc07g005410.2.1	0	0	0	0	0	0	0	0	0	0	0	0	0
Solyc07g005420.1.1	0	0	0	0	0	0	0	0	0	0	0	0	0
Solyc07g005430.2.1	2	136	3	66	0	885	48	165	8	466	16	247	4
Solyc07g005440.1.1	20	216	30	130	0	537	137	374	5	547	134	409	1

Solyc07g005450.2.1	1	7	2	2	0	57	15	34	1	10	0	25	0
Solyc07g005460.2.1	1	11	1	12	0	130	5	62	0	11	2	27	0
Solyc07g005470.2.1	1	2	0	0	0	6	3	4	0	0	0	0	0
Solyc07g005480.2.1	11	30	0	23	0	164	127	151	17	64	26	80	3
Solyc07g005490.2.1	0	0	0	0	0	0	0	0	0	0	0	0	0
Solyc07g005500.2.1	8	7	2	2	4	63	61	34	17	10	19	10	0
Solyc07g005510.2.1	8	440	51	221	3	56	29	96	2	86	75	45	1
Solyc07g005520.1.1	0	0	0	0	0	0	0	0	0	0	0	0	0
Solyc07g005530.2.1	22	416	61	306	28	488	278	573	40	2780	309	1690	19
Solyc07g005540.1.1	3	32	3	2	1	17	51	23	10	0	0	2	2
Solyc07g005550.2.1	9	121	27	86	4	413	334	379	21	88	84	83	0
Solyc07g005560.2.1	3	208	2	61	0	153	29	254	5	293	12	330	1
Solyc07g005570.2.1	9	67	10	63	2	53	111	102	3	94	9	70	9
Solyc07g005580.2.1	4	9	4	9	0	42	19	54	3	5	0	15	0
Solyc07g005590.2.1	15	107	7	84	9	123	144	156	65	126	47	81	1
Solyc07g005600.2.1	8	205	13	136	18	401	102	397	45	314	67	265	0
Solyc07g005610.2.1	1	1	1	1	0	2	5	12	0	2	5	4	0
Solyc07g005620.2.1	1	3	0	1	0	22	9	13	3	2	2	1	0
Solyc07g005640.2.1	1	27	3	6	2	22	24	60	16	17	17	6	0
Solyc07g005650.2.1	1	5	0	2	0	8	8	3	3	0	0	0	0
Solyc07g005660.2.1	5	16	6	17	0	23	40	59	5	123	46	28	3
Solyc07g005680.2.1	0	0	0	0	0	0	0	0	0	0	0	0	0
Solyc07g005690.2.1	7	70	4	55	0	191	27	141	5	152	14	118	0
Solyc07g005700.2.1	11	65	5	25	2	73	48	118	10	194	26	175	14
Solyc07g005710.2.1	4	10	4	3	1	18	14	35	0	45	8	15	0
Solyc07g005720.2.1	0	0	0	0	0	0	0	0	0	0	0	0	0
Solyc07g005730.2.1	2	19	0	2	0	7	18	16	0	33	9	19	1
Solyc07g005740.1.1	0	0	0	0	0	0	0	0	0	0	0	0	0
Solyc07g005750.2.1	6	7	0	11	0	13	27	19	10	24	5	13	0
Solyc07g005760.2.1	16	613	32	280	5	715	345	506	17	584	267	283	8
Solyc07g005770.2.1	0	0	0	0	0	0	0	0	0	0	0	0	0
Solyc07g005790.2.1	0	0	0	0	0	0	0	0	0	0	0	0	0
Solyc07g005800.2.1	3	0	3	0	0	9	4	3	0	3	0	8	0
Solyc07g005810.2.1	18	196	28	178	17	462	353	513	89	702	190	689	16
Solyc07g005820.2.1	0	0	0	0	0	0	0	0	0	0	0	0	0
Solyc07g005830.2.1	8	434	37	234	9	115	267	287	4	250	47	122	3
Solyc07g005840.2.1	8	75	3	63	0	61	57	78	2	1	14	14	1
Solyc07g005850.1.1	0	0	0	0	0	0	0	0	0	0	0	0	0
Solyc07g005860.2.1	5	13	0	13	0	46	29	74	1	30	14	7	0
Solyc07g005870.2.1	1	4	0	0	0	1	2	2	0	1	0	3	0
Solyc07g005880.2.1	10	65	5	46	0	141	54	174	17	115	23	112	1

Solyc07g005890.2.1	6	7	0	2	0	31	18	18	0	22	2	24	0
Solyc07g005900.2.1	2	5	3	5	0	2	37	1	13	52	2	0	3
Solyc07g005910.2.1	0	0	0	0	0	0	0	0	0	0	0	0	0
Solyc07g005920.1.1	0	0	0	0	0	0	0	0	0	0	0	0	0
Solyc07g005930.2.1	4	10	0	4	0	4	14	23	0	21	8	15	2
Solyc07g005940.2.1	7	269	41	206	26	531	448	804	56	742	122	532	8
Solyc07g005950.2.1	5	27	2	28	0	11	19	33	0	32	4	39	0
Solyc07g005960.2.1	9	24	9	35	1	23	151	36	4	41	24	104	1
Solyc07g005970.2.1	14	99	19	100	6	198	159	273	23	132	15	75	4
Solyc07g005990.2.1	11	82	10	66	3	181	99	199	25	180	32	114	5
Solyc07g006000.2.1	8	177	11	77	1	19	52	45	0	33	6	24	1
Solyc07g006010.2.1	1	10	0	0	0	10	0	24	1	29	3	40	0
Solyc07g006020.2.1	0	0	0	0	0	0	0	0	0	0	0	0	0
Solyc07g006030.2.1	30	479	185	425	0	58	190	237	5	199	54	299	0
Solyc07g006040.2.1	1	10	2	3	0	0	10	2	0	5	0	0	0
Solyc07g006050.1.1	0	0	0	0	0	0	0	0	0	0	0	0	0
Solyc07g006060.2.1	0	0	0	0	0	0	0	0	0	0	0	0	0
Solyc07g006070.1.1	8	94	5	24	2	184	46	106	2	119	28	87	3
Solyc07g006100.2.1	0	0	0	0	0	0	0	0	0	0	0	0	0
Solyc07g006110.2.1	0	0	0	0	0	0	0	0	0	0	0	0	0
Solyc07g006120.2.1	3	28	1	16	2	41	41	48	2	60	6	19	3
Solyc07g006130.1.1	10	16	7	18	1	321	157	206	51	44	51	36	2
Solyc07g006140.2.1	11	88	19	55	3	107	259	199	173	25	13	55	4
Solyc07g006150.2.1	1	6	0	5	0	13	7	6	2	5	0	10	0
Solyc07g006160.2.1	9	51	1	28	3	181	112	182	8	49	27	23	1
Solyc07g006170.2.1	0	0	0	0	0	0	0	0	0	0	0	0	0
Solyc07g006180.2.1	4	16	0	6	0	20	19	24	1	17	13	19	0
Solyc07g006190.2.1	8	20	1	30	4	42	19	58	8	42	12	49	0
Solyc07g006200.2.1	1	10	0	2	1	14	2	20	0	17	0	8	0
Solyc07g006220.1.1	14	640	53	423	59	237	145	266	42	1167	173	1286	11
Solyc07g006230.1.1	0	0	0	0	0	0	0	0	0	0	0	0	0
Solyc07g006240.2.1	0	0	0	0	0	0	0	0	0	0	0	0	0
Solyc07g006250.2.1	0	0	0	0	0	0	0	0	0	0	0	0	0
Solyc07g006260.2.1	8	57	4	27	3	110	33	97	0	74	8	28	2
Solyc07g006270.2.1	3	75	6	35	6	892	126	300	8	352	44	164	0
Solyc07g006280.2.1	8	1003	169	484	87	409	248	362	44	595	355	954	54
Solyc07g006300.2.1	6	125	7	5	40	324	330	89	333	114	24	19	20
Solyc07g006320.2.1	0	0	0	0	0	0	0	0	0	0	0	0	0
Solyc07g006330.1.1	0	0	0	0	0	0	0	0	0	0	0	0	0
Solyc07g006340.2.1	4	34	0	5	0	3	18	10	4	21	3	15	0
Solyc07g006350.2.1	8	68	7	23	5	99	86	122	18	79	10	69	5

Solyc07g006360.1.1	6	46	3	29	8	107	26	38	9	11	6	2	1
Solyc07g006370.1.1	4	20	1	12	0	195	12	257	0	82	0	66	0
Solyc07g006380.2.1	0	0	0	0	0	0	0	0	0	0	0	0	0
Solyc07g006400.1.1	5	24	8	11	0	60	69	46	10	52	26	91	5
Solyc07g006410.1.1	1	13	0	14	0	4	0	14	0	0	4	1	0
Solyc07g006420.1.1	0	0	0	0	0	0	0	0	0	0	0	0	0
Solyc07g006430.1.1	0	0	0	0	0	0	0	0	0	0	0	0	0
Solyc07g006480.2.1	0	0	0	0	0	0	0	0	0	0	0	0	0
Solyc07g006490.2.1	8	106	12	34	3	39	64	61	6	49	13	25	1
Solyc07g006500.2.1	16	21	5	11	5	271	257	370	52	84	123	18	0
Solyc07g006510.2.1	10	70	6	59	0	224	75	259	22	50	22	47	0
Solyc07g006520.2.1	5	78	6	52	1	18	65	108	0	31	10	74	0
Solyc07g006530.2.1	5	16	1	5	1	36	23	52	7	17	5	30	2
Solyc07g006540.2.1	23	363	42	242	99	654	535	455	171	178	73	151	5
Solyc07g006560.2.1	7	154	15	42	7	7	82	42	7	22	82	3	1
Solyc07g006570.2.1	13	452	25	195	1	46	140	283	0	1362	471	425	12
Solyc07g006580.2.1	6	82	12	28	2	19	128	26	8	102	18	63	0
Solyc07g006590.1.1	1	33	4	33	0	36	6	69	0	16	2	19	0
Solyc07g006600.1.1	4	24	1	11	0	45	27	110	0	47	11	9	0
Solyc07g006610.2.1	0	0	0	0	0	0	0	0	0	0	0	0	0
Solyc07g006620.2.1	0	0	0	0	0	0	0	0	0	0	0	0	0
Solyc07g006630.2.1	2	52	0	69	5	11	0	11	1	7	11	3	0
Solyc07g006640.2.1	1	3	0	1	0	3	3	4	1	20	2	1	0
Solyc07g006650.2.1	9	100	12	87	4	338	217	502	18	347	74	222	0
Solyc07g006670.1.1	0	0	0	0	0	0	0	0	0	0	0	0	0
Solyc07g006680.1.1	0	0	0	0	0	0	0	0	0	0	0	0	0
Solyc07g006690.2.1	6	11	4	7	2	31	26	28	1	20	0	18	0
Solyc07g006700.1.1	0	0	0	0	0	0	0	0	0	0	0	0	0
Solyc07g006710.1.1	0	0	0	0	0	0	0	0	0	0	0	0	0
Solyc07g006730.2.1	4	8	6	3	2	26	4	5	0	153	20	172	1
Solyc07g006760.2.1	14	115	13	76	17	702	163	503	21	239	52	205	2
Solyc07g006770.2.1	3	6	0	0	2	60	19	16	27	3	3	0	1
Solyc07g006780.2.1	1	18	0	23	0	65	19	37	0	37	1	29	0
Solyc07g006790.2.1	5	69	5	45	6	126	33	144	10	122	24	59	1
Solyc07g006810.2.1	7	22	9	23	0	17	33	36	0	24	6	29	1
Solyc07g006820.2.1	17	81	3	78	3	336	136	318	29	161	44	144	5
Solyc07g006830.2.1	10	129	10	103	2	180	90	222	23	244	44	121	9
Solyc07g006850.1.1	0	0	0	0	0	0	0	0	0	0	0	0	0
Solyc07g006860.2.1	6	7	0	3	0	202	206	136	4	0	0	0	0
Solyc07g006870.2.1	1	4	0	0	0	16	7	3	2	0	0	0	0
Solyc07g006880.1.1	5	1	0	0	0	0	0	13	0	209	67	104	1

Solyc07g006890.1.1	13	574	67	252	20	743	57	361	7	56	15	183	18
Solyc07g006910.2.1	2	12	1	7	1	3	8	14	1	28	10	29	0
Solyc07g006920.2.1	0	0	0	0	0	0	0	0	0	0	0	0	0
Solyc07g006930.1.1	0	0	0	0	0	0	0	0	0	0	0	0	0
Solyc07g006950.2.1	1	1	0	1	0	2	7	11	1	6	0	11	0
Solyc07g006960.2.1	1	14	0	8	0	11	3	31	0	41	0	24	0
Solyc07g006970.2.1	10	21	0	18	5	65	89	54	6	67	19	50	4
Solyc07g006990.1.1	0	0	0	0	0	0	0	0	0	0	0	0	0
Solyc07g007000.1.1	0	0	0	0	0	0	0	0	0	0	0	0	0
Solyc07g007010.1.1	0	0	0	0	0	0	0	0	0	0	0	0	0
Solyc07g007020.2.1	0	0	0	0	0	0	0	0	0	0	0	0	0
Solyc07g007030.1.1	11	116	14	39	4	89	34	97	7	335	70	288	1
Solyc07g007040.2.1	29	209	34	230	13	483	490	547	85	395	112	416	9
Solyc07g007050.1.1	0	0	0	0	0	0	0	0	0	0	0	0	0
Solyc07g007060.1.1	9	158	7	225	0	729	160	1573	10	206	42	134	2
Solyc07g007110.2.1	4	54	11	38	5	68	116	66	7	71	21	62	2
Solyc07g007120.2.1	12	529	59	365	20	1182	1309	1834	166	923	132	515	12
Solyc07g007130.1.1	4	185	37	207	43	402	72	135	13	135	23	110	8
Solyc07g007140.2.1	9	34	2	21	0	147	43	184	7	49	22	22	0
Solyc07g007150.1.1	3	23	2	15	0	11	8	27	0	67	13	37	0
Solyc07g007160.2.1	6	107	8	58	0	72	48	102	2	24	6	28	0
Solyc07g007170.2.1	1	40	2	31	2	34	4	16	1	7	0	11	0
Solyc07g007180.2.1	7	41	10	21	0	78	273	113	4	35	11	32	0
Solyc07g007190.2.1	0	0	0	0	0	0	0	0	0	0	0	0	0
Solyc07g007200.2.1	0	0	0	0	0	0	0	0	0	0	0	0	0
Solyc07g007210.2.1	12	78	4	81	6	185	84	306	23	293	48	280	19
Solyc07g007220.2.1	10	143	32	99	2	153	41	118	13	62	4	66	3
Solyc07g007230.2.1	14	121	8	80	0	303	160	338	14	205	25	187	2
Solyc07g007240.2.1	0	0	0	0	0	0	0	0	0	0	0	0	0
Solyc07g007250.2.1	7	1009	202	563	0	88	988	350	0	116	330	110	166
Solyc07g007260.2.1	1	22	1	9	0	6	0	0	0	9	8	3	0
Solyc07g007270.2.1	0	0	0	0	0	0	0	0	0	0	0	0	0
Solyc07g007280.2.1	1	17	1	18	0	1	7	2	0	0	0	11	0
Solyc07g007300.2.1	3	17	2	19	0	28	12	13	7	18	1	15	0
Solyc07g007310.2.1	8	123	4	74	2	99	53	87	2	80	8	76	0
Solyc07g007330.1.1	10	200	73	184	12	802	368	869	12	490	31	285	17
Solyc07g007340.2.1	6	14	1	10	0	112	22	221	0	76	2	31	0
Solyc07g007350.1.1	3	53	0	24	0	83	5	88	0	61	1	12	2
Solyc07g007360.1.1	4	30	0	18	0	40	9	80	6	34	23	35	1
Solyc07g007370.2.1	9	33	4	19	2	65	62	77	7	42	10	33	2
Solyc07g007380.2.1	8	70	0	48	10	125	46	157	17	31	25	45	0

Solyc07g007390.2.1	16	118	12	67	8	318	83	329	26	136	71	86	6
Solyc07g007400.2.1	2	0	0	0	0	5	4	7	1	161	13	112	1
Solyc07g007410.2.1	1	0	0	0	0	0	0	5	0	10	4	2	0
Solyc07g007420.2.1	0	0	0	0	0	0	0	0	0	0	0	0	0
Solyc07g007430.2.1	9	51	4	42	4	117	52	106	5	125	18	70	0
Solyc07g007440.1.1	0	0	0	0	0	0	0	0	0	0	0	0	0
Solyc07g007450.2.1	0	0	0	0	0	0	0	0	0	0	0	0	0
Solyc07g007470.1.1	0	0	0	0	0	0	0	0	0	0	0	0	0
Solyc07g007500.2.1	7	217	6	78	5	247	104	366	19	228	13	150	1
Solyc07g007510.2.1	4	75	0	41	0	90	22	122	8	120	12	78	0
Solyc07g007550.2.1	5	7	2	2	0	9	24	8	10	88	23	118	0
Solyc07g007560.2.1	10	67	19	26	10	48	36	40	8	51	46	40	2
Solyc07g007570.1.1	0	0	0	0	0	0	0	0	0	0	0	0	0
Solyc07g007590.1.1	5	141	26	245	48	79	34	59	20	92	32	299	0
Solyc07g007600.2.1	18	1925	160	1145	85	1417	1030	2194	202	3610	243	1986	42
Solyc07g007610.2.1	0	0	0	0	0	0	0	0	0	0	0	0	0
Solyc07g007620.2.1	4	143	26	77	9	52	53	59	32	7	3	4	0
Solyc07g007630.2.1	4	23	3	25	0	46	44	74	10	23	15	33	0
Solyc07g007640.1.1	0	0	0	0	0	0	0	0	0	0	0	0	0
Solyc07g007650.1.1	8	57	7	35	5	186	102	174	17	80	33	63	0
Solyc07g007660.1.1	10	127	23	105	0	226	116	234	42	72	35	23	2
Solyc07g007670.2.1	13	344	25	210	3	552	213	833	28	170	52	110	13
Solyc07g007680.2.1	14	28	2	22	10	229	116	465	11	81	25	105	1
Solyc07g007690.2.1	3	22	1	11	0	10	11	21	0	0	1	9	0
Solyc07g007700.1.1	8	75	2	39	0	244	106	212	7	102	26	67	2
Solyc07g007710.2.1	4	0	0	0	0	118	25	39	0	0	17	0	0
Solyc07g007730.2.1	0	0	0	0	0	0	0	0	0	0	0	0	0
Solyc07g007740.1.1	0	0	0	0	0	0	0	0	0	0	0	0	0
Solyc07g007750.2.1	4	9	27	8	0	6	1089	15	0	1012	179	1458	17
Solyc07g007760.2.1	5	1287	7	558	2	3890	1719	6621	2	7042	15	6408	0
Solyc07g007770.1.1	0	0	0	0	0	0	0	0	0	0	0	0	0
Solyc07g007780.2.1	17	181	22	119	4	236	116	302	17	266	38	209	4
Solyc07g007790.2.1	9	109	17	135	9	524	171	551	39	473	109	552	10
Solyc07g007800.2.1	7	35	0	1	3	117	154	13	155	43	10	12	0
Solyc07g007810.2.1	6	18	2	8	1	48	21	33	4	24	7	23	0
Solyc07g007820.2.1	16	244	13	142	5	351	88	243	32	191	48	145	5
Solyc07g007830.2.1	8	81	6	61	9	151	47	186	14	263	35	155	3
Solyc07g007840.1.1	1	2	0	1	0	8	0	10	0	3	0	13	0
Solyc07g007850.2.1	1	2	1	5	0	11	3	23	1	2	0	9	0
Solyc07g007870.2.1	7	154	19	155	24	230	91	296	25	303	56	287	17
Solyc07g007890.2.1	12	52	4	49	1	234	47	230	18	108	27	69	3

Solyc07g007920.1.1	0	0	0	0	0	0	0	0	0	0	0	0	0
Solyc07g007930.2.1	22	51	8	38	2	316	2503	515	30	645	240	375	19
Solyc07g007940.2.1	0	0	0	0	0	0	0	0	0	0	0	0	0
Solyc07g007950.1.1	5	58	13	48	2	19	13	30	0	14	3	25	1
Solyc07g007960.2.1	4	23	4	10	4	39	25	57	11	40	15	23	2
Solyc07g007970.2.1	2	23	0	0	0	36	4	14	0	21	1	16	0
Solyc07g007980.2.1	1	29	4	19	0	1	3	2	6	17	5	20	4
Solyc07g007990.2.1	0	0	0	0	0	0	0	0	0	0	0	0	0
Solyc07g008000.2.1	8	25	9	29	1	58	37	46	12	97	26	48	7
Solyc07g008010.2.1	0	0	0	0	0	0	0	0	0	0	0	0	0
Solyc07g008020.2.1	1	5	1	2	0	3	4	5	0	0	0	0	0
Solyc07g008030.1.1	1	1	1	3	0	16	2	8	0	5	1	1	0
Solyc07g008040.2.1	22	76	14	72	18	237	251	263	32	182	77	121	4
Solyc07g008050.2.1	7	20	3	15	1	45	51	45	17	29	23	33	3
Solyc07g008060.1.1	17	91	22	59	13	111	106	128	42	176	81	137	5
Solyc07g008070.1.1	0	0	0	0	0	0	0	0	0	0	0	0	0
Solyc07g008080.2.1	3	15	3	8	0	29	36	21	2	39	6	34	9
Solyc07g008090.2.1	0	0	0	0	0	0	0	0	0	0	0	0	0
Solyc07g008100.2.1	2	8	0	4	0	13	6	11	2	74	11	46	5
Solyc07g008110.2.1	0	0	0	0	0	0	0	0	0	0	0	0	0
Solyc07g008120.2.1	0	0	0	0	0	0	0	0	0	0	0	0	0
Solyc07g008130.2.1	0	0	0	0	0	0	0	0	0	0	0	0	0
Solyc07g008140.2.1	10	97	2	59	3	1122	224	537	82	47	6	0	1
Solyc07g008150.2.1	9	81	5	58	2	61	60	96	11	73	20	52	0
Solyc07g008160.2.1	14	37	6	36	4	131	73	106	8	176	20	83	2
Solyc07g008170.2.1	4	12	2	22	1	47	29	41	12	17	18	14	1
Solyc07g008180.2.1	3	32	1	12	6	32	29	31	3	102	22	93	1
Solyc07g008190.2.1	11	72	8	77	7	257	79	225	25	108	25	104	1
Solyc07g008200.2.1	0	0	0	0	0	0	0	0	0	0	0	0	0
Solyc07g008210.2.1	2	28	0	65	0	225	8	114	0	82	3	54	0
Solyc07g008240.2.1	0	0	0	0	0	0	0	0	0	0	0	0	0
Solyc07g008250.2.1	7	150	4	209	1	7023	445	8680	31	132	72	86	0
Solyc07g008260.2.1	0	0	0	0	0	0	0	0	0	0	0	0	0
Solyc07g008270.2.1	1	7	1	8	1	9	12	22	1	1	8	2	0
Solyc07g008280.2.1	3	8	2	12	0	47	28	22	15	1	5	1	0
Solyc07g008290.2.1	0	0	0	0	0	0	0	0	0	0	0	0	0
Solyc07g008310.2.1	5	24	6	16	0	29	63	43	1	59	4	33	0
Solyc07g008320.2.1	14	136	14	85	5	377	83	345	28	160	61	139	4
Solyc07g008330.2.1	11	56	7	30	0	108	60	156	21	66	41	92	2
Solyc07g008340.2.1	12	153	19	97	8	606	245	552	83	369	66	318	5
Solyc07g008350.2.1	6	58	5	15	10	84	28	72	12	170	17	72	2

Solyc07g008360.1.1	0	0	0	0	0	0	0	0	0	0	0	0	0
Solyc07g008370.2.1	7	212	18	124	22	386	151	472	51	460	95	445	13
Solyc07g008390.1.1	0	0	0	0	0	0	0	0	0	0	0	0	0
Solyc07g008410.2.1	1	0	0	0	0	9	2	17	0	2	3	2	0
Solyc07g008430.1.1	0	0	0	0	0	0	0	0	0	0	0	0	0
Solyc07g008440.2.1	0	0	0	0	0	0	0	0	0	0	0	0	0
Solyc07g008450.2.1	9	31	2	18	0	47	65	99	12	59	17	97	0
Solyc07g008460.2.1	3	14	2	11	2	23	26	19	3	30	5	14	0
Solyc07g008470.2.1	0	0	0	0	0	0	0	0	0	0	0	0	0
Solyc07g008480.1.1	0	0	0	0	0	0	0	0	0	0	0	0	0
Solyc07g008490.2.1	6	32	4	23	4	104	41	99	7	45	22	49	4
Solyc07g008520.2.1	7	2	0	6	0	29	92	69	8	2	62	2	3
Solyc07g008530.1.1	4	10	6	8	0	5	36	21	3	23	7	7	0
Solyc07g008540.2.1	11	188	8	129	5	84	98	163	1	63	2	70	1
Solyc07g008550.2.1	0	0	0	0	0	0	0	0	0	0	0	0	0
Solyc07g008560.2.1	4	11	0	10	0	12	6	36	0	32	19	28	0
Solyc07g008570.2.1	1	46	1	17	0	0	0	1	2	0	13	2	0
Solyc07g008590.1.1	2	11	5	12	1	11	4	18	1	18	1	20	0
Solyc07g008600.1.1	0	0	0	0	0	0	0	0	0	0	0	0	0
Solyc07g008610.1.1	7	79	19	40	2	159	238	199	35	149	71	118	4
Solyc07g008620.1.1	0	0	0	0	0	0	0	0	0	0	0	0	0
Solyc07g008630.1.1	0	0	0	0	0	0	0	0	0	0	0	0	0
Solyc07g008640.1.1	0	0	0	0	0	0	0	0	0	0	0	0	0
Solyc07g008650.2.1	0	0	0	0	0	0	0	0	0	0	0	0	0
Solyc07g008660.2.1	2	11	0	3	0	14	1	12	1	10	7	6	0
Solyc07g008670.2.1	1	22	5	15	4	4	3	13	1	26	8	12	0
Solyc07g008700.2.1	2	8	2	11	1	22	9	31	4	30	9	20	0
Solyc07g008710.2.1	0	0	0	0	0	0	0	0	0	0	0	0	0
Solyc07g008720.2.1	2	315	12	180	9	259	104	330	22	739	80	582	21
Solyc07g008730.1.1	1	2	0	0	0	0	8	4	0	4	1	1	0
Solyc07g008750.2.1	2	19	2	5	2	56	24	45	2	111	7	117	3
Solyc07g008760.2.1	3	4	0	3	0	16	33	15	3	28	4	25	0
Solyc07g008770.2.1	3	57	4	14	0	71	66	167	3	121	3	42	0
Solyc07g008780.2.1	9	50	7	38	1	126	110	147	16	104	50	58	5
Solyc07g008800.2.1	1	5	0	6	0	5	5	5	4	11	2	17	0
Solyc07g008820.2.1	0	0	0	0	0	0	0	0	0	0	0	0	0
Solyc07g008840.2.1	15	134	16	95	14	214	88	312	15	216	57	172	4
Solyc07g008850.2.1	0	0	0	0	0	0	0	0	0	0	0	0	0
Solyc07g008860.2.1	8	28	3	20	0	111	47	117	10	44	17	63	2
Solyc07g008880.2.1	30	409	98	424	29	1551	822	1821	123	1711	394	1336	20
Solyc07g008890.1.1	0	0	0	0	0	0	0	0	0	0	0	0	0

Solyc07g008900.2.1	8	42	4	32	5	35	31	45	4	466	62	485	3
Solyc07g008910.2.1	0	0	0	0	0	0	0	0	0	0	0	0	0
Solyc07g008920.2.1	3	4	1	0	0	27	24	7	27	1	0	1	0
Solyc07g008930.1.1	0	0	0	0	0	0	0	0	0	0	0	0	0
Solyc07g008940.2.1	3	11	0	11	2	20	22	20	5	20	10	48	6
Solyc07g008950.2.1	12	109	7	45	10	322	119	328	36	231	52	255	8
Solyc07g008970.2.1	1	8	0	7	0	70	0	22	0	42	4	26	0
Solyc07g009000.1.1	0	0	0	0	0	0	0	0	0	0	0	0	0
Solyc07g009130.2.1	22	93	11	119	0	410	185	637	0	143	43	119	0
Solyc07g009140.2.1	5	116	1	43	3	229	66	280	25	202	32	149	1
Solyc07g009150.2.1	5	87	22	60	7	45	130	46	8	42	50	54	3
Solyc07g009160.2.1	3	17	6	2	1	49	23	24	3	15	10	24	2
Solyc07g009170.2.1	3	5	0	6	0	10	10	7	0	13	7	2	0
Solyc07g009180.1.1	0	0	0	0	0	0	0	0	0	0	0	0	0
Solyc07g009220.1.1	0	0	0	0	0	0	0	0	0	0	0	0	0
Solyc07g009230.2.1	1	4	0	2	0	2	0	9	0	0	15	0	0
Solyc07g009320.2.1	5	28	3	13	0	52	30	92	7	22	6	29	0
Solyc07g009330.2.1	5	261	27	124	22	247	280	286	87	503	184	507	9
Solyc07g009340.2.1	0	0	0	0	0	0	0	0	0	0	0	0	0
Solyc07g009350.2.1	17	45	11	26	3	139	173	140	36	175	50	172	15
Solyc07g009360.1.1	0	0	0	0	0	0	0	0	0	0	0	0	0
Solyc07g009380.2.1	8	985	65	428	42	23	155	11	1	105	0	10	0
Solyc07g009410.2.1	0	0	0	0	0	0	0	0	0	0	0	0	0
Solyc07g009440.1.1	2	2	1	8	1	5	5	11	0	0	0	0	0
Solyc07g009460.2.1	2	8	0	0	0	0	8	7	0	27	0	24	0
Solyc07g009490.2.1	9	71	10	51	1	69	70	101	18	123	23	120	2
Solyc07g009500.1.1	0	0	0	0	0	0	0	0	0	0	0	0	0
Solyc07g009510.1.1	0	0	0	0	0	0	0	0	0	0	0	0	0
Solyc07g009530.1.1	0	0	0	0	0	0	0	0	0	0	0	0	0
Solyc07g014590.2.1	6	38	6	35	0	6	19	9	0	120	11	59	0
Solyc07g014610.2.1	6	19	1	13	2	51	32	37	2	117	12	108	0
Solyc07g014620.1.1	1	1	0	0	0	0	3	2	1	10	1	9	0
Solyc07g014640.2.1	3	9	0	0	0	11	7	7	3	15	9	27	0
Solyc07g014650.2.1	0	0	0	0	0	0	0	0	0	0	0	0	0
Solyc07g014670.2.1	16	31	11	52	4	174	235	196	13	123	8	82	2
Solyc07g014680.2.1	0	0	0	0	0	0	0	0	0	0	0	0	0
Solyc07g014690.2.1	2	1	0	2	0	10	10	8	0	0	0	0	0
Solyc07g014700.2.1	16	173	14	105	0	343	107	436	19	468	42	475	1
Solyc07g014730.2.1	3	29	0	19	0	3	48	4	0	14	0	9	0
Solyc07g014740.2.1	0	0	0	0	0	0	0	0	0	0	0	0	0
Solyc07g015800.1.1	14	57	15	40	5	63	77	69	7	149	20	85	6

Solyc07g015860.2.1	4	34	7	32	0	11	33	14	3	60	2	53	3
Solyc07g015910.2.1	0	0	0	0	0	0	0	0	0	0	0	0	0
Solyc07g015970.1.1	2	4	0	15	0	16	8	4	1	35	15	18	0
Solyc07g015980.2.1	1	1	0	0	0	0	9	2	0	7	0	2	0
Solyc07g016150.2.1	3	40	0	14	0	155	27	108	6	95	12	120	2
Solyc07g016170.2.1	2	7	0	2	0	36	8	4	0	26	10	14	0
Solyc07g016180.2.1	2	8	0	16	0	4	5	1	0	10	4	11	0
Solyc07g016200.2.1	5	217	4	68	16	263	61	272	24	262	42	200	2
Solyc07g016210.1.1	0	0	0	0	0	0	0	0	0	0	0	0	0
Solyc07g017220.2.1	6	136	11	143	4	200	52	162	9	64	13	58	4
Solyc07g017230.2.1	0	0	0	0	0	0	0	0	0	0	0	0	0
Solyc07g017240.1.1	0	0	0	0	0	0	0	0	0	0	0	0	0
Solyc07g017250.2.1	0	0	0	0	0	0	0	0	0	0	0	0	0
Solyc07g017260.2.1	0	0	0	0	0	0	0	0	0	0	0	0	0
Solyc07g017290.2.1	0	0	0	0	0	0	0	0	0	0	0	0	0
Solyc07g017400.2.1	9	103	12	47	0	139	65	213	20	117	23	116	2
Solyc07g017410.2.1	0	0	0	0	0	0	0	0	0	0	0	0	0
Solyc07g017430.2.1	17	175	15	93	11	246	200	237	26	189	21	110	7
Solyc07g017490.2.1	8	136	29	65	29	272	745	360	57	226	85	157	1
Solyc07g017500.2.1	15	121	26	111	6	197	230	224	34	205	48	212	5
Solyc07g017510.2.1	40	401	42	446	10	2281	2162	5378	65	2057	254	2155	9
Solyc07g017520.2.1	0	0	0	0	0	0	0	0	0	0	0	0	0
Solyc07g017530.2.1	0	0	0	0	0	0	0	0	0	0	0	0	0
Solyc07g017540.2.1	0	0	0	0	0	0	0	0	0	0	0	0	0
Solyc07g017580.1.1	0	0	0	0	0	0	0	0	0	0	0	0	0
Solyc07g017600.2.1	19	264	10	116	14	349	40	203	73	1524	99	579	5
Solyc07g017610.2.1	41	261	17	159	15	3549	2110	2894	79	1813	177	1397	15
Solyc07g017640.2.1	4	2	1	3	0	7	21	21	2	22	10	25	0
Solyc07g017650.2.1	0	0	0	0	0	0	0	0	0	0	0	0	0
Solyc07g017680.1.1	2	2	0	4	0	1	1	0	0	5	1	0	0
Solyc07g017730.2.1	0	0	0	0	0	0	0	0	0	0	0	0	0
Solyc07g017740.2.1	1	5	0	4	0	7	2	10	0	4	2	8	1
Solyc07g017750.2.1	9	147	17	47	2	182	136	223	24	321	46	107	8
Solyc07g017760.1.1	11	32	14	28	3	52	114	74	6	26	20	51	3
Solyc07g017770.2.1	3	14	0	3	0	31	29	18	0	86	7	124	3
Solyc07g017780.2.1	20	1369	204	1682	42	6142	1410	6500	318	966	172	831	3
Solyc07g017800.2.1	2	209	27	122	6	71	59	91	1	30	9	40	2
Solyc07g017860.2.1	1	8	0	0	0	7	6	3	0	14	4	5	0
Solyc07g017880.2.1	0	0	0	0	0	0	0	0	0	0	0	0	0
Solyc07g017900.2.1	5	118	3	41	1	193	90	222	12	321	43	201	2
Solyc07g017940.1.1	10	52	3	31	2	111	40	82	9	105	40	88	0

Solyc07g017950.2.1	0	0	0	0	0	0	0	0	0	0	0	0	0
Solyc07g017960.2.1	2	15	1	14	0	9	58	25	22	36	25	23	0
Solyc07g017980.1.1	0	0	0	0	0	0	0	0	0	0	0	0	0
Solyc07g017990.2.1	0	0	0	0	0	0	0	0	0	0	0	0	0
Solyc07g018000.2.1	7	29	9	35	7	35	53	47	1	45	9	25	6
Solyc07g018010.2.1	0	0	0	0	0	0	0	0	0	0	0	0	0
Solyc07g018070.2.1	0	0	0	0	0	0	0	0	0	0	0	0	0
Solyc07g018090.2.1	5	20	3	16	1	39	45	50	4	21	8	18	0
Solyc07g018100.2.1	5	16	3	21	1	21	28	36	1	26	2	6	0
Solyc07g018140.2.1	0	0	0	0	0	0	0	0	0	0	0	0	0
Solyc07g018190.2.1	0	0	0	0	0	0	0	0	0	0	0	0	0
Solyc07g018200.1.1	0	0	0	0	0	0	0	0	0	0	0	0	0
Solyc07g018210.2.1	0	0	0	0	0	0	0	0	0	0	0	0	0
Solyc07g018220.2.1	0	0	0	0	0	0	0	0	0	0	0	0	0
Solyc07g018240.1.1	0	0	0	0	0	0	0	0	0	0	0	0	0
Solyc07g018250.1.1	0	0	0	0	0	0	0	0	0	0	0	0	0
Solyc07g018270.2.1	9	81	4	43	3	167	51	100	3	81	23	86	0
Solyc07g018290.2.1	0	0	0	0	0	0	0	0	0	0	0	0	0
Solyc07g018300.2.1	0	0	0	0	0	0	0	0	0	0	0	0	0
Solyc07g018340.2.1	4	5	4	1	0	23	9	23	7	26	5	19	0
Solyc07g018350.2.1	12	64	13	50	3	224	181	162	14	168	33	83	5
Solyc07g018360.2.1	18	429	63	257	25	271	624	671	48	312	90	243	38
Solyc07g018400.2.1	0	0	0	0	0	0	0	0	0	0	0	0	0
Solyc07g019430.1.1	0	0	0	0	0	0	0	0	0	0	0	0	0
Solyc07g019440.2.1	10	100	5	68	7	287	108	268	13	203	52	166	1
Solyc07g019460.2.1	16	839	81	381	30	359	117	262	80	271	212	326	10
Solyc07g019510.2.1	0	0	0	0	0	0	0	0	0	0	0	0	0
Solyc07g019530.2.1	3	25	7	9	0	18	14	34	6	38	3	27	0
Solyc07g019650.2.1	1	2	1	3	0	9	5	7	0	2	0	1	0
Solyc07g019670.2.1	20	199	10	138	6	1327	159	932	46	539	617	1041	16
Solyc07g020710.2.1	5	24	3	8	6	41	18	40	0	59	11	29	1
Solyc07g020790.2.1	6	32	7	11	0	94	45	82	2	65	39	133	2
Solyc07g020800.2.1	0	0	0	0	0	0	0	0	0	0	0	0	0
Solyc07g020860.2.1	2	394	43	159	45	338	276	601	64	685	178	677	26
Solyc07g020870.1.1	0	0	0	0	0	0	0	0	0	0	0	0	0
Solyc07g020960.2.1	3	22	2	13	0	29	14	26	0	18	2	24	1
Solyc07g021020.2.1	0	0	0	0	0	0	0	0	0	0	0	0	0
Solyc07g021120.1.1	0	0	0	0	0	0	0	0	0	0	0	0	0
Solyc07g021180.1.1	0	0	0	0	0	0	0	0	0	0	0	0	0
Solyc07g021320.2.1	0	0	0	0	0	0	0	0	0	0	0	0	0
Solyc07g021330.1.1	0	0	0	0	0	0	0	0	0	0	0	0	0

Solyc07g021340.2.1	1	10	4	5	1	1	19	9	1	5	4	2	0
Solyc07g021540.2.1	5	154	17	78	2	65	60	168	19	350	22	232	9
Solyc07g021550.2.1	3	14	0	12	1	9	6	25	1	51	0	37	0
Solyc07g021610.1.1	0	0	0	0	0	0	0	0	0	0	0	0	0
Solyc07g021620.1.1	6	39	5	20	0	43	44	57	1	39	3	28	0
Solyc07g021630.2.1	2	0	0	2	0	16	139	24	2	0	7	0	0
Solyc07g021650.1.1	0	0	0	0	0	0	0	0	0	0	0	0	0
Solyc07g021680.1.1	0	0	0	0	0	0	0	0	0	0	0	0	0
Solyc07g021700.2.1	5	59	1	31	3	5	18	16	7	27	4	23	0
Solyc07g021710.2.1	0	0	0	0	0	0	0	0	0	0	0	0	0
Solyc07g021750.1.1	4	27	8	8	2	11	11	17	2	53	15	46	0
Solyc07g022760.2.1	14	81	12	72	10	240	239	207	21	160	34	145	2
Solyc07g022770.1.1	1	6	1	3	0	1	2	6	0	6	1	3	0
Solyc07g022780.2.1	3	6	2	5	3	15	35	64	9	17	9	9	4
Solyc07g022790.2.1	4	18	3	17	2	46	42	78	6	59	26	44	0
Solyc07g022830.2.1	2	9	1	15	0	1	10	5	0	1	0	42	0
Solyc07g022860.2.1	5	17	4	18	3	34	60	55	14	71	14	60	0
Solyc07g022880.2.1	0	0	0	0	0	0	0	0	0	0	0	0	0
Solyc07g022900.2.1	0	0	0	0	0	0	0	0	0	0	0	0	0
Solyc07g022910.2.1	22	128	30	112	3	328	119	301	24	160	32	87	0
Solyc07g022920.2.1	6	19	1	16	0	30	51	32	3	61	14	72	0
Solyc07g023990.1.1	0	0	0	0	0	0	0	0	0	0	0	0	0
Solyc07g024000.2.1	5	53	5	27	0	180	139	151	8	68	23	70	0
Solyc07g024010.1.1	0	0	0	0	0	0	0	0	0	0	0	0	0
Solyc07g024020.1.1	0	0	0	0	0	0	0	0	0	0	0	0	0
Solyc07g024070.1.1	0	0	0	0	0	0	0	0	0	0	0	0	0
Solyc07g025110.1.1	0	0	0	0	0	0	0	0	0	0	0	0	0
Solyc07g025140.2.1	0	0	0	0	0	0	0	0	0	0	0	0	0
Solyc07g025160.2.1	0	0	0	0	0	0	0	0	0	0	0	0	0
Solyc07g025170.2.1	0	0	0	0	0	0	0	0	0	0	0	0	0
Solyc07g025200.1.1	0	0	0	0	0	0	0	0	0	0	0	0	0
Solyc07g025210.1.1	4	14	0	8	1	26	28	23	5	26	8	16	2
Solyc07g025230.1.1	0	0	0	0	0	0	0	0	0	0	0	0	0
Solyc07g025250.1.1	0	0	0	0	0	0	0	0	0	0	0	0	0
Solyc07g025370.2.1	5	33	3	20	11	70	84	131	11	54	24	48	5
Solyc07g025380.2.1	0	0	0	0	0	0	0	0	0	0	0	0	0
Solyc07g025390.2.1	1	5	0	1	0	1	8	14	1	11	2	8	0
Solyc07g025400.2.1	1	4	0	0	0	4	9	4	0	2	0	2	0
Solyc07g025510.2.1	2	11	3	7	0	5	5	3	2	42	5	40	0
Solyc07g025520.2.1	3	32	3	29	0	12	23	27	0	15	7	0	1
Solyc07g025530.1.1	1	10	0	0	0	1	7	5	0	4	0	0	0

Solyc07g026610.1.1	0	0	0	0	0	0	0	0	0	0	0	0	0
Solyc07g026640.1.1	0	0	0	0	0	0	0	0	0	0	0	0	0
Solyc07g026650.2.1	0	0	0	0	0	0	0	0	0	0	0	0	0
Solyc07g026660.2.1	0	0	0	0	0	0	0	0	0	0	0	0	0
Solyc07g026670.2.1	1	0	0	1	0	7	3	3	1	4	1	1	0
Solyc07g026680.2.1	0	0	0	0	0	0	0	0	0	0	0	0	0
Solyc07g026730.1.1	0	0	0	0	0	0	0	0	0	0	0	0	0
Solyc07g026740.1.1	0	0	0	0	0	0	0	0	0	0	0	0	0
Solyc07g026760.1.1	0	0	0	0	0	0	0	0	0	0	0	0	0
Solyc07g026770.2.1	2	48	2	6	0	44	19	99	4	138	9	122	0
Solyc07g026810.2.1	15	50	9	29	7	126	103	209	19	109	54	106	5
Solyc07g026930.1.1	0	0	0	0	0	0	0	0	0	0	0	0	0
Solyc07g026950.2.1	0	0	0	0	0	0	0	0	0	0	0	0	0
Solyc07g026960.2.1	4	100	0	101	0	185	7	224	1	204	3	141	1
Solyc07g027020.2.1	8	21	7	15	3	46	67	111	4	66	21	55	2
Solyc07g032080.2.1	0	0	0	0	0	0	0	0	0	0	0	0	0
Solyc07g032090.2.1	2	13	1	13	0	39	53	41	5	27	13	17	0
Solyc07g032100.2.1	8	39	1	60	8	100	82	196	6	134	49	107	6
Solyc07g032110.2.1	3	12	5	2	0	20	54	25	4	21	3	11	0
Solyc07g032170.2.1	1	29	5	13	5	30	25	30	2	169	24	135	2
Solyc07g032180.2.1	1	22	3	9	6	47	18	28	6	38	6	17	0
Solyc07g032220.2.1	0	0	0	0	0	0	0	0	0	0	0	0	0
Solyc07g032230.2.1	19	389	99	396	38	1012	437	829	80	313	106	447	12
Solyc07g032240.2.1	1	4	0	0	0	2	8	10	6	14	0	4	0
Solyc07g032250.2.1	2	20	2	3	0	46	34	62	3	77	12	46	1
Solyc07g032260.2.1	1	9	1	0	1	31	4	17	0	11	0	0	0
Solyc07g032380.2.1	5	124	24	46	2	72	50	30	1	38	17	76	8
Solyc07g032480.2.1	6	9	0	11	7	55	31	47	7	36	5	22	2
Solyc07g032490.2.1	0	0	0	0	0	0	0	0	0	0	0	0	0
Solyc07g032510.2.1	0	0	0	0	0	0	0	0	0	0	0	0	0
Solyc07g032710.2.1	0	0	0	0	0	0	0	0	0	0	0	0	0
Solyc07g032730.2.1	0	0	0	0	0	0	0	0	0	0	0	0	0
Solyc07g032740.2.1	2	12	0	5	0	108	10	61	2	82	5	64	4
Solyc07g037950.1.1	1	1	0	0	4	6	6	4	0	8	1	0	0
Solyc07g037960.1.1	4	18	0	6	0	12	18	27	0	11	3	12	0
Solyc07g038100.2.1	3	3	2	8	2	5	12	4	0	9	2	6	0
Solyc07g038120.1.1	0	0	0	0	0	0	0	0	0	0	0	0	0
Solyc07g038160.2.1	1	4	0	0	0	7	6	4	0	0	2	0	0
Solyc07g038190.2.1	0	0	0	0	0	0	0	0	0	0	0	0	0
Solyc07g039190.2.1	6	58	9	33	0	9	39	21	0	19	4	20	5
Solyc07g039200.2.1	4	86	4	47	0	380	87	283	20	378	54	263	7

Solyc07g039210.2.1	2	16	0	20	0	33	14	58	1	39	2	43	0
Solyc07g039280.1.1	0	0	0	0	0	0	0	0	0	0	0	0	0
Solyc07g039290.1.1	0	0	0	0	0	0	0	0	0	0	0	0	0
Solyc07g039300.1.1	0	0	0	0	0	0	0	0	0	0	0	0	0
Solyc07g039310.1.1	0	0	0	0	0	0	0	0	0	0	0	0	0
Solyc07g039320.2.1	0	0	0	0	0	0	0	0	0	0	0	0	0
Solyc07g039330.2.1	2	14	1	5	0	15	23	17	1	39	9	65	0
Solyc07g039340.2.1	8	60	4	24	8	71	112	87	9	39	21	23	2
Solyc07g039370.1.1	0	0	0	0	0	0	0	0	0	0	0	0	0
Solyc07g039380.2.1	0	0	0	0	0	0	0	0	0	0	0	0	0
Solyc07g039420.1.1	0	0	0	0	0	0	0	0	0	0	0	0	0
Solyc07g039450.2.1	2	6	0	10	0	37	5	19	0	30	4	16	0
Solyc07g039500.2.1	7	20	4	15	0	35	45	33	5	18	6	20	0
Solyc07g039510.2.1	1	0	1	5	1	0	10	0	0	1	1	1	2
Solyc07g039550.2.1	3	10	1	16	1	14	10	13	2	6	3	20	0
Solyc07g039570.2.1	0	0	0	0	0	0	0	0	0	0	0	0	0
Solyc07g040680.2.1	3	10	1	8	1	14	13	12	1	28	23	37	0
Solyc07g040690.2.1	3	4	2	4	0	7	11	8	2	22	12	6	2
Solyc07g040710.2.1	0	0	0	0	0	0	0	0	0	0	0	0	0
Solyc07g040720.2.1	1	5	0	0	4	11	2	5	0	1	1	0	0
Solyc07g040730.1.1	4	58	1	8	5	17	14	21	6	211	13	86	2
Solyc07g040740.2.1	1	3	2	1	0	0	0	4	0	3	2	0	0
Solyc07g040750.1.1	1	4	0	3	0	1	9	5	1	0	0	0	0
Solyc07g040780.2.1	0	0	0	0	0	0	0	0	0	0	0	0	0
Solyc07g040790.2.1	0	0	0	0	0	0	0	0	0	0	0	0	0
Solyc07g040890.1.1	0	0	0	0	0	0	0	0	0	0	0	0	0
Solyc07g040910.2.1	1	7	1	0	0	4	0	7	0	2	3	1	0
Solyc07g040920.2.1	1	6	1	11	1	19	3	25	2	17	6	12	0
Solyc07g040940.2.1	9	29	3	26	12	50	35	79	9	118	16	97	2
Solyc07g040950.2.1	3	201	27	110	4	53	62	59	2	26	14	46	0
Solyc07g040960.1.1	16	1453	333	1163	84	94	12	87	2	259	0	624	33
Solyc07g040970.1.1	0	0	0	0	0	0	0	0	0	0	0	0	0
Solyc07g040980.2.1	15	59	7	25	3	160	125	186	25	135	40	128	3
Solyc07g040990.2.1	16	118	25	96	12	2140	848	1625	146	283	49	131	2
Solyc07g041000.2.1	0	0	0	0	0	0	0	0	0	0	0	0	0
Solyc07g041010.2.1	2	37	1	11	2	38	46	53	4	203	11	100	0
Solyc07g041020.2.1	2	6	0	0	3	4	3	3	0	3	2	6	0
Solyc07g041070.2.1	1	1	0	4	0	4	6	1	0	3	2	0	0
Solyc07g041080.2.1	1	6	0	10	0	12	4	31	0	6	0	8	0
Solyc07g041150.2.1	24	319	40	253	30	799	340	621	76	520	140	389	3
Solyc07g041160.2.1	13	140	9	138	2	342	57	343	28	332	37	319	7

Solyc07g041180.2.1	0	0	0	0	0	0	0	0	0	0	0	0	0
Solyc07g041190.2.1	0	0	0	0	0	0	0	0	0	0	0	0	0
Solyc07g041200.2.1	3	11	2	17	0	18	11	16	3	33	9	24	0
Solyc07g041210.2.1	0	0	0	0	0	0	0	0	0	0	0	0	0
Solyc07g041230.2.1	1	1	0	0	0	4	2	6	2	0	1	0	0
Solyc07g041280.2.1	2	0	0	0	0	2	43	4	3	9	15	3	2
Solyc07g041290.1.1	0	0	0	0	0	0	0	0	0	0	0	0	0
Solyc07g041310.2.1	3	87	2	39	7	226	47	186	3	806	45	300	2
Solyc07g041330.1.1	4	13	7	8	10	14	42	26	3	11	13	22	1
Solyc07g041340.1.1	2	18	2	30	2	7	5	10	0	30	2	19	5
Solyc07g041490.1.1	7	90	16	40	9	139	115	231	16	168	54	151	4
Solyc07g041500.2.1	0	0	0	0	0	0	0	0	0	0	0	0	0
Solyc07g041510.1.1	3	5	5	6	0	18	23	34	4	29	5	53	0
Solyc07g041520.2.1	0	0	0	0	0	0	0	0	0	0	0	0	0
Solyc07g041550.2.1	3	13	1	11	0	29	24	15	1	35	9	18	0
Solyc07g041640.2.1	0	0	0	0	0	0	0	0	0	0	0	0	0
Solyc07g041660.2.1	13	88	9	78	4	159	246	199	38	130	15	81	7
Solyc07g041720.1.1	3	574	14	404	0	1	0	1	0	1	0	19	0
Solyc07g041730.2.1	3	36	5	14	0	11	9	9	2	23	4	38	2
Solyc07g041750.2.1	6	47	3	20	4	105	52	152	11	244	35	151	2
Solyc07g041760.2.1	0	0	0	0	0	0	0	0	0	0	0	0	0
Solyc07g041780.2.1	0	0	0	0	0	0	0	0	0	0	0	0	0
Solyc07g041830.2.1	6	57	5	93	0	134	40	187	2	37	2	12	1
Solyc07g041870.2.1	9	26	1	28	0	71	43	125	12	59	21	87	6
Solyc07g041900.2.1	11	2846	206	1118	108	22434	8713	17414	871	6740	1607	5463	119
Solyc07g041910.2.1	0	0	0	0	0	0	0	0	0	0	0	0	0
Solyc07g041920.2.1	12	1498	77	840	0	4	79	2	0	1	32	1	0
Solyc07g041930.2.1	3	13	1	5	2	24	14	32	2	21	2	26	0
Solyc07g041940.2.1	0	0	0	0	0	0	0	0	0	0	0	0	0
Solyc07g041970.2.1	22	98	5	62	8	68	12	134	4	528	275	541	10
Solyc07g041980.2.1	0	0	0	0	0	0	0	0	0	0	0	0	0
Solyc07g042000.2.1	1	1	0	0	0	5	3	4	0	2	2	2	0
Solyc07g042010.2.1	0	0	0	0	0	0	0	0	0	0	0	0	0
Solyc07g042020.2.1	5	22	5	6	0	35	65	36	13	17	14	27	2
Solyc07g042030.1.1	3	7	1	10	0	36	37	26	6	3	4	9	0
Solyc07g042080.2.1	9	26	7	11	2	84	105	67	53	35	5	37	6
Solyc07g042090.2.1	0	0	0	0	0	0	0	0	0	0	0	0	0
Solyc07g042130.2.1	2	5	1	0	0	18	4	15	2	2	0	3	0
Solyc07g042150.2.1	5	43	5	16	4	26	74	55	5	56	22	53	2
Solyc07g042160.2.1	3	10	0	12	0	28	8	19	1	49	16	26	3
Solyc07g042170.2.1	11	842	76	364	20	1694	188	452	51	179	68	164	8

Solyc07g042180.2.1	10	312	33	269	10	579	339	729	39	560	140	509	3
Solyc07g042190.2.1	6	58	5	42	2	123	140	198	10	268	81	655	7
Solyc07g042220.1.1	0	0	0	0	0	0	0	0	0	0	0	0	0
Solyc07g042230.1.1	4	2	17	1	21	277	117	75	85	0	32	1	0
Solyc07g042250.2.1	7	323	25	146	2	426	85	537	11	584	92	623	2
Solyc07g042260.2.1	3	29	3	11	6	2	19	6	2	18	11	30	0
Solyc07g042270.2.1	4	23	3	3	2	28	9	44	0	25	13	14	4
Solyc07g042280.2.1	0	0	0	0	0	0	0	0	0	0	0	0	0
Solyc07g042300.1.1	0	0	0	0	0	0	0	0	0	0	0	0	0
Solyc07g042330.1.1	0	0	0	0	0	0	0	0	0	0	0	0	0
Solyc07g042380.2.1	21	79	15	77	4	198	135	215	25	291	101	303	2
Solyc07g042390.1.1	3	21	2	1	4	0	14	1	14	70	23	76	16
Solyc07g042400.2.1	0	0	0	0	0	0	0	0	0	0	0	0	0
Solyc07g042410.1.1	0	0	0	0	0	0	0	0	0	0	0	0	0
Solyc07g042430.1.1	0	0	0	0	0	0	0	0	0	0	0	0	0
Solyc07g042440.2.1	0	0	0	0	0	0	0	0	0	0	0	0	0
Solyc07g042450.2.1	2	4	0	6	0	6	4	6	0	12	4	11	0
Solyc07g042460.1.1	1	0	0	0	0	2	2	4	0	4	4	0	2
Solyc07g042470.2.1	0	0	0	0	0	0	0	0	0	0	0	0	0
Solyc07g042490.1.1	1	61	1	2	0	141	11	64	1	2860	7	209	4
Solyc07g042500.2.1	25	63	3	52	3	387	131	525	10	99	29	107	0
Solyc07g042510.2.1	0	0	0	0	0	0	0	0	0	0	0	0	0
Solyc07g042520.2.1	7	172	7	103	0	48	29	53	0	6	3	0	0
Solyc07g042540.2.1	0	0	0	0	0	0	0	0	0	0	0	0	0
Solyc07g042550.2.1	24	189	69	73	61	3831	3955	2675	630	1203	1215	546	106
Solyc07g042560.2.1	0	0	0	0	0	0	0	0	0	0	0	0	0
Solyc07g042570.2.1	4	42	5	41	1	58	45	108	2	93	25	64	0
Solyc07g042580.2.1	0	0	0	0	0	0	0	0	0	0	0	0	0
Solyc07g042590.2.1	13	82	17	46	2	88	27	66	7	112	34	96	0
Solyc07g042620.2.1	0	0	0	0	0	0	0	0	0	0	0	0	0
Solyc07g042630.2.1	8	110	10	15	6	115	50	25	14	0	1	0	0
Solyc07g042680.2.1	3	55	4	38	10	63	49	71	7	76	17	75	3
Solyc07g042690.2.1	0	0	0	0	0	0	0	0	0	0	0	0	0
Solyc07g042700.2.1	0	0	0	0	0	0	0	0	0	0	0	0	0
Solyc07g042710.2.1	0	0	0	0	0	0	0	0	0	0	0	0	0
Solyc07g042720.1.1	0	0	0	0	0	0	0	0	0	0	0	0	0
Solyc07g042730.2.1	0	0	0	0	0	0	0	0	0	0	0	0	0
Solyc07g042740.2.1	0	0	0	0	0	0	0	0	0	0	0	0	0
Solyc07g042750.2.1	2	8	3	3	0	2	13	12	0	16	6	11	0
Solyc07g042760.1.1	0	0	0	0	0	0	0	0	0	0	0	0	0
Solyc07g042800.2.1	2	5	1	1	0	6	8	9	2	6	0	0	0

Solyc07g042810.1.1	0	0	0	0	0	0	0	0	0	0	0	0	0
Solyc07g042820.2.1	0	0	0	0	0	0	0	0	0	0	0	0	0
Solyc07g042830.2.1	4	19	1	14	4	22	10	41	2	16	12	12	1
Solyc07g042840.1.1	0	0	0	0	0	0	0	0	0	0	0	0	0
Solyc07g042850.2.1	0	0	0	0	0	0	0	0	0	0	0	0	0
Solyc07g042890.2.1	6	127	7	118	5	267	94	252	15	164	64	205	0
Solyc07g042900.2.1	6	132	11	144	5	519	80	249	8	262	21	242	4
Solyc07g042910.2.1	6	44	7	22	2	69	71	123	14	117	17	56	1
Solyc07g042930.2.1	4	13	0	4	1	29	29	24	3	32	7	8	0
Solyc07g042950.1.1	0	0	0	0	0	0	0	0	0	0	0	0	0
Solyc07g042960.1.1	0	0	0	0	0	0	0	0	0	0	0	0	0
Solyc07g042970.2.1	1	0	0	0	0	0	2	0	5	0	2	0	0
Solyc07g042980.2.1	0	0	0	0	0	0	0	0	0	0	0	0	0
Solyc07g042990.1.1	0	0	0	0	0	0	0	0	0	0	0	0	0
Solyc07g043000.2.1	0	0	0	0	0	0	0	0	0	0	0	0	0
Solyc07g043050.1.1	35	315	74	249	16	134	616	239	61	110	88	56	2
Solyc07g043060.1.1	0	0	0	0	0	0	0	0	0	0	0	0	0
Solyc07g043120.1.1	0	0	0	0	0	0	0	0	0	0	0	0	0
Solyc07g043130.2.1	10	760	42	551	59	121	42	60	12	116	60	149	5
Solyc07g043150.1.1	0	0	0	0	0	0	0	0	0	0	0	0	0
Solyc07g043160.1.1	23	321	52	357	10	40	309	179	10	299	58	280	5
Solyc07g043170.2.1	0	0	0	0	0	0	0	0	0	0	0	0	0
Solyc07g043190.1.1	0	0	0	0	0	0	0	0	0	0	0	0	0
Solyc07g043230.2.1	6	179	16	219	1	329	29	372	24	17	2	2	1
Solyc07g043240.2.1	0	0	0	0	0	0	0	0	0	0	0	0	0
Solyc07g043250.1.1	1	31	6	25	0	68	2	28	6	13	2	27	0
Solyc07g043260.2.1	11	54	2	66	1	123	83	143	9	74	10	38	0
Solyc07g043270.2.1	14	113	4	87	1	271	105	253	22	294	36	211	0
Solyc07g043310.2.1	14	138	3	143	4	2646	458	1785	99	456	68	319	6
Solyc07g043320.2.1	32	461	43	357	10	246	256	455	36	1607	302	1531	37
Solyc07g043330.1.1	11	115	13	44	2	114	75	135	1	101	11	103	0
Solyc07g043360.1.1	4	344	18	124	15	360	92	387	37	523	103	496	7
Solyc07g043390.2.1	19	6674	145	2686	8	1014	20	1037	0	331	262	22	3
Solyc07g043400.1.1	1	348	6	80	0	28	1	22	0	13	8	5	0
Solyc07g043410.1.1	0	0	0	0	0	0	0	0	0	0	0	0	0
Solyc07g043420.2.1	15	14986	650	5481	38	677	306	2137	5	2278	1591	607	8
Solyc07g043460.2.1	15	1555	149	668	10	260	85	497	0	163	449	49	0
Solyc07g043480.1.1	23	1530	30	766	5	46	67	178	1	6	117	0	0
Solyc07g043490.1.1	27	2447	44	707	2	298	77	440	5	128	489	105	2
Solyc07g043500.1.1	12	644	14	195	8	225	31	421	8	65	93	62	0
Solyc07g043510.2.1	1	5	1	6	0	5	4	12	3	6	3	10	0

Solyc07g043550.2.1	2	9	0	6	8	19	14	23	9	2	4	1	0
Solyc07g043560.2.1	17	55	6	29	4	224	113	215	58	79	84	97	3
Solyc07g043570.2.1	5	205	3	91	2	944	45	419	7	161	16	93	0
Solyc07g043580.2.1	7	212	18	142	6	69	57	114	11	80	6	95	0
Solyc07g043590.2.1	11	307	36	196	22	1065	474	523	103	190	129	119	1
Solyc07g043600.2.1	15	120	9	68	0	102	137	195	3	148	19	118	3
Solyc07g043610.2.1	15	134	36	103	36	263	256	218	9	353	152	244	15
Solyc07g043620.2.1	14	70	7	45	2	82	53	186	11	125	73	155	8
Solyc07g043640.2.1	0	0	0	0	0	0	0	0	0	0	0	0	0
Solyc07g043710.2.1	0	0	0	0	0	0	0	0	0	0	0	0	0
Solyc07g044750.2.1	0	0	0	0	0	0	0	0	0	0	0	0	0
Solyc07g044760.2.1	10	101	7	69	4	245	100	270	24	516	82	419	2
Solyc07g044780.2.1	1	1	3	7	2	12	10	10	2	0	2	1	0
Solyc07g044810.2.1	4	17	1	7	3	27	23	35	9	43	16	38	3
Solyc07g044830.2.1	0	0	0	0	0	0	0	0	0	0	0	0	0
Solyc07g044840.2.1	20	377	50	261	26	869	434	1293	124	1411	260	1151	13
Solyc07g044850.2.1	11	221	17	150	11	427	324	519	38	454	75	340	13
Solyc07g044860.2.1	2	1394	141	912	8	30	220	78	12	239	239	460	13
Solyc07g044880.2.1	0	0	0	0	0	0	0	0	0	0	0	0	0
Solyc07g044910.1.1	0	0	0	0	0	0	0	0	0	0	0	0	0
Solyc07g044930.2.1	1	1	0	0	0	5	5	3	0	5	5	3	0
Solyc07g044940.1.1	0	0	0	0	0	0	0	0	0	0	0	0	0
Solyc07g044960.1.1	8	35	2	16	19	59	57	42	15	13	0	5	0
Solyc07g044970.1.1	0	0	0	0	0	0	0	0	0	0	0	0	0
Solyc07g044980.2.1	14	211	24	158	12	313	144	596	28	113	8	112	0
Solyc07g044990.2.1	1	7	0	3	0	11	7	1	0	1	2	3	0
Solyc07g045000.2.1	0	0	0	0	0	0	0	0	0	0	0	0	0
Solyc07g045010.2.1	0	0	0	0	0	0	0	0	0	0	0	0	0
Solyc07g045030.2.1	0	0	0	0	0	0	0	0	0	0	0	0	0
Solyc07g045050.2.1	9	120	12	61	10	28	126	85	17	108	30	65	4
Solyc07g045070.2.1	6	53	15	44	11	49	211	151	11	188	36	130	5
Solyc07g045080.2.1	0	0	0	0	0	0	0	0	0	0	0	0	0
Solyc07g045090.2.1	0	0	0	0	0	0	0	0	0	0	0	0	0
Solyc07g045100.1.1	4	146	15	109	0	21	6	9	3	9	5	29	0
Solyc07g045140.2.1	6	2	0	6	3	70	15	25	8	0	22	2	0
Solyc07g045150.2.1	8	90	1	40	2	153	45	117	3	64	20	77	0
Solyc07g045160.2.1	6	40	2	29	1	95	12	42	10	25	12	4	0
Solyc07g045170.2.1	2	4	0	8	0	11	20	4	4	18	8	35	0
Solyc07g045180.2.1	9	22	0	6	0	36	62	57	3	38	4	51	2
Solyc07g045190.1.1	4	61	3	48	0	220	90	146	6	86	25	74	1
Solyc07g045210.1.1	4	107	5	67	5	48	36	75	4	138	41	135	0

Solyc07g045220.2.1	0	0	0	0	0	0	0	0	0	0	0	0	0
Solyc07g045230.2.1	7	31	4	30	2	35	38	59	6	45	37	71	2
Solyc07g045240.2.1	3	56	6	22	8	121	68	146	9	68	9	76	0
Solyc07g045250.1.1	3	96	1	39	4	59	20	47	2	113	20	38	4
Solyc07g045290.2.1	10	60	6	25	14	138	72	163	26	136	48	179	7
Solyc07g045300.2.1	2	25	3	9	0	21	14	22	3	10	1	10	0
Solyc07g045310.2.1	10	96	27	48	0	70	211	121	3	51	16	30	5
Solyc07g045320.2.1	0	0	0	0	0	0	0	0	0	0	0	0	0
Solyc07g045330.2.1	5	40	5	56	0	156	130	113	17	123	26	149	1
Solyc07g045340.2.1	2	16	0	12	0	21	7	23	0	79	5	30	0
Solyc07g045350.2.1	3	26	0	13	0	33	36	63	3	86	15	43	1
Solyc07g045370.2.1	4	15	2	10	0	36	24	32	1	24	11	40	0
Solyc07g045380.2.1	4	3	0	10	0	7	10	9	2	16	9	3	5
Solyc07g045390.1.1	0	0	0	0	0	0	0	0	0	0	0	0	0
Solyc07g045400.1.1	0	0	0	0	0	0	0	0	0	0	0	0	0
Solyc07g045410.1.1	0	0	0	0	0	0	0	0	0	0	0	0	0
Solyc07g045420.2.1	13	375	45	380	8	879	173	540	16	264	58	210	1
Solyc07g045440.1.1	13	1043	22	595	66	48	32	146	7	2735	392	1447	17
Solyc07g045450.1.1	0	0	0	0	0	0	0	0	0	0	0	0	0
Solyc07g045460.2.1	0	0	0	0	0	0	0	0	0	0	0	0	0
Solyc07g045470.1.1	6	38	9	39	5	63	22	63	11	48	10	28	0
Solyc07g045480.2.1	6	14	11	29	1	82	70	101	17	30	11	28	1
Solyc07g045510.2.1	0	0	0	0	0	0	0	0	0	0	0	0	0
Solyc07g045520.2.1	2	17	1	20	0	38	27	26	2	19	2	12	0
Solyc07g045530.1.1	1	10	7	5	0	1	1	10	4	0	0	2	0
Solyc07g045540.2.1	6	114	25	117	1	29	78	58	3	67	30	68	0
Solyc07g045570.1.1	0	0	0	0	0	0	0	0	0	0	0	0	0
Solyc07g047610.2.1	1	4	0	1	0	8	0	6	0	8	4	22	0
Solyc07g047620.1.1	2	0	0	0	0	7	20	8	4	11	5	3	0
Solyc07g047670.2.1	14	259	18	164	6	387	154	464	46	933	90	614	10
Solyc07g047680.1.1	0	0	0	0	0	0	0	0	0	0	0	0	0
Solyc07g047690.1.1	9	36	5	15	2	47	38	82	3	16	11	22	0
Solyc07g047700.2.1	1	8	1	5	0	20	9	19	0	15	0	15	0
Solyc07g047710.2.1	0	0	0	0	0	0	0	0	0	0	0	0	0
Solyc07g047720.2.1	6	26	1	27	0	43	24	29	8	73	15	53	0
Solyc07g047730.2.1	0	0	0	0	0	0	0	0	0	0	0	0	0
Solyc07g047740.2.1	0	0	0	0	0	0	0	0	0	0	0	0	0
Solyc07g047750.2.1	0	0	0	0	0	0	0	0	0	0	0	0	0
Solyc07g047760.1.1	8	21	3	0	0	29	41	57	14	61	20	45	1
Solyc07g047770.2.1	16	164	13	124	6	117	103	105	15	341	39	261	8
Solyc07g047780.2.1	6	62	7	52	4	113	44	105	12	61	18	39	0

Solyc07g047790.2.1	13	41	0	36	0	211	46	288	17	202	61	277	11
Solyc07g047800.2.1	7	372	46	190	7	1179	1388	887	126	505	538	581	4
Solyc07g047820.1.1	0	0	0	0	0	0	0	0	0	0	0	0	0
Solyc07g047830.2.1	10	39	10	23	4	39	61	54	10	49	16	33	4
Solyc07g047840.2.1	2	22	1	9	0	2	9	19	0	10	2	9	0
Solyc07g047850.2.1	13	11677	1207	5452	31	124	201	65	13	199	1069	172	29
Solyc07g047860.2.1	11	19	3	34	2	67	46	102	5	67	18	65	3
Solyc07g047870.1.1	0	0	0	0	0	0	0	0	0	0	0	0	0
Solyc07g047880.2.1	0	0	0	0	0	0	0	0	0	0	0	0	0
Solyc07g047890.2.1	0	0	0	0	0	0	0	0	0	0	0	0	0
Solyc07g047900.2.1	1	1	0	1	0	7	0	3	0	4	6	4	0
Solyc07g047940.2.1	0	0	0	0	0	0	0	0	0	0	0	0	0
Solyc07g047950.1.1	10	115	18	155	7	120	88	129	4	123	32	77	0
Solyc07g047960.2.1	10	10	1	7	0	49	46	30	9	39	7	45	1
Solyc07g047970.2.1	4	38	5	20	3	48	39	83	12	59	14	55	0
Solyc07g047980.1.1	11	191	19	152	7	196	88	189	23	220	47	221	16
Solyc07g047990.1.1	0	0	0	0	0	0	0	0	0	0	0	0	0
Solyc07g048000.2.1	0	0	0	0	0	0	0	0	0	0	0	0	0
Solyc07g048010.2.1	1	3	0	0	0	2	0	8	0	4	1	4	0
Solyc07g048030.2.1	8	84	14	42	1	121	79	119	28	109	59	137	7
Solyc07g048040.1.1	6	30	3	31	7	141	31	159	12	63	15	51	0
Solyc07g048050.1.1	0	0	0	0	0	0	0	0	0	0	0	0	0
Solyc07g048060.1.1	0	0	0	0	0	0	0	0	0	0	0	0	0
Solyc07g048070.2.1	2	6	1	5	0	25	6	24	4	0	0	0	0
Solyc07g048080.2.1	7	83	9	47	4	84	55	88	5	72	36	49	0
Solyc07g048090.1.1	3	78	8	29	1	49	16	64	0	44	7	9	0
Solyc07g048100.1.1	0	0	0	0	0	0	0	0	0	0	0	0	0
Solyc07g048110.2.1	0	0	0	0	0	0	0	0	0	0	0	0	0
Solyc07g048120.2.1	2	3	0	6	0	16	19	22	5	3	5	15	0
Solyc07g049140.2.1	0	0	0	0	0	0	0	0	0	0	0	0	0
Solyc07g049150.2.1	7	55	5	70	1	250	81	148	12	258	12	196	11
Solyc07g049160.2.1	4	17	6	3	0	15	47	44	6	62	17	54	9
Solyc07g049180.2.1	5	31	10	14	3	68	12	86	2	38	5	30	2
Solyc07g049190.2.1	0	0	0	0	0	0	0	0	0	0	0	0	0
Solyc07g049200.2.1	4	8	0	7	1	22	18	27	5	14	4	31	0
Solyc07g049210.2.1	3	26	3	24	0	11	46	20	4	178	9	69	0
Solyc07g049220.2.1	5	25	0	23	1	35	18	52	3	30	17	20	0
Solyc07g049230.1.1	2	22	1	9	0	5	39	30	0	22	4	20	0
Solyc07g049260.2.1	4	42	2	50	0	198	28	95	7	52	25	47	4
Solyc07g049270.2.1	1	1	0	0	4	9	5	1	1	0	2	4	0
Solyc07g049280.2.1	0	0	0	0	0	0	0	0	0	0	0	0	0

Solyc07g049290.2.1	12	170	51	121	19	144	395	407	26	183	85	185	9
Solyc07g049300.2.1	1	2	0	9	0	0	0	0	0	22	13	12	0
Solyc07g049310.2.1	8	29	10	16	0	4	84	14	6	43	6	38	2
Solyc07g049320.2.1	1	6	1	6	0	0	26	15	0	0	5	4	0
Solyc07g049350.2.1	17	92	7	92	4	355	180	278	74	253	20	217	4
Solyc07g049360.2.1	1	64	7	52	0	211	207	139	30	82	17	119	1
Solyc07g049370.2.1	2	27	1	15	0	35	11	24	8	48	7	16	0
Solyc07g049390.1.1	4	42	10	23	2	24	18	21	15	28	5	6	1
Solyc07g049400.1.1	0	0	0	0	0	0	0	0	0	0	0	0	0
Solyc07g049410.2.1	2	3	4	0	0	7	17	10	0	17	13	3	0
Solyc07g049430.2.1	0	0	0	0	0	0	0	0	0	0	0	0	0
Solyc07g049440.2.1	10	308	9	2	24	2603	315	173	882	358	64	38	41
Solyc07g049450.2.1	4	143	5	62	4	600	130	424	27	324	220	385	1
Solyc07g049460.2.1	0	0	0	0	0	0	0	0	0	0	0	0	0
Solyc07g049470.2.1	0	0	0	0	0	0	0	0	0	0	0	0	0
Solyc07g049480.2.1	5	38	3	12	5	58	35	88	10	57	22	40	0
Solyc07g049490.1.1	3	40	5	20	3	73	23	52	15	78	15	81	0
Solyc07g049500.2.1	2	5	0	9	4	13	9	12	0	19	5	17	0
Solyc07g049530.2.1	5	229	4	137	0	912	71	482	1	148	520	107	0
Solyc07g049550.2.1	1	14	0	4	0	11	3	10	1	13	41	3	0
Solyc07g049560.2.1	4	24	5	7	0	27	3	13	3	8	0	10	0
Solyc07g049570.2.1	0	0	0	0	0	0	0	0	0	0	0	0	0
Solyc07g049580.2.1	2	14	1	9	10	54	4	29	0	33	4	10	1
Solyc07g049590.2.1	4	5	1	6	0	14	12	16	1	34	7	25	0
Solyc07g049600.2.1	0	0	0	0	0	0	0	0	0	0	0	0	0
Solyc07g049610.1.1	3	20	2	11	2	4	8	3	0	10	0	10	0
Solyc07g049630.1.1	2	75	11	34	3	28	24	47	3	150	6	130	7
Solyc07g049660.2.1	15	442	0	456	0	113	0	308	2	98	367	92	1
Solyc07g049680.2.1	0	0	0	0	0	0	0	0	0	0	0	0	0
Solyc07g049690.2.1	3	76	6	38	0	83	40	153	2	71	18	30	0
Solyc07g049700.1.1	0	0	0	0	0	0	0	0	0	0	0	0	0
Solyc07g049710.2.1	0	0	0	0	0	0	0	0	0	0	0	0	0
Solyc07g049720.2.1	3	29	0	53	1	137	50	54	5	43	10	90	4
Solyc07g049730.2.1	5	73	2	14	0	46	11	86	1	218	7	124	0
Solyc07g049740.2.1	0	0	0	0	0	0	0	0	0	0	0	0	0
Solyc07g049750.2.1	12	48	7	72	0	60	66	53	7	156	28	106	3
Solyc07g051820.2.1	0	0	0	0	0	0	0	0	0	0	0	0	0
Solyc07g051840.2.1	5	28	2	22	8	23	10	41	3	3	26	13	0
Solyc07g051850.2.1	12	485	59	349	22	650	949	929	209	1569	307	1008	24
Solyc07g051860.1.1	0	0	0	0	0	0	0	0	0	0	0	0	0
Solyc07g051870.1.1	0	0	0	0	0	0	0	0	0	0	0	0	0

Solyc07g051880.1.1	2	0	0	0	0	0	1	0	0	4	4	4	0
Solyc07g051890.1.1	0	0	0	0	0	0	0	0	0	0	0	0	0
Solyc07g051920.1.1	0	0	0	0	0	0	0	0	0	0	0	0	0
Solyc07g051930.1.1	2	4	0	0	0	15	18	7	8	0	1	0	0
Solyc07g051950.2.1	0	0	0	0	0	0	0	0	0	0	0	0	0
Solyc07g052060.2.1	0	0	0	0	0	0	0	0	0	0	0	0	0
Solyc07g052070.1.1	0	0	0	0	0	0	0	0	0	0	0	0	0
Solyc07g052100.2.1	0	0	0	0	0	0	0	0	0	0	0	0	0
Solyc07g052110.2.1	5	15	2	13	0	21	53	50	8	68	16	84	0
Solyc07g052140.2.1	0	0	0	0	0	0	0	0	0	0	0	0	0
Solyc07g052180.1.1	0	0	0	0	0	0	0	0	0	0	0	0	0
Solyc07g052210.2.1	0	0	0	0	0	0	0	0	0	0	0	0	0
Solyc07g052220.1.1	3	73	10	8	15	0	15	18	108	0	18	3	17
Solyc07g052230.2.1	7	11	3	14	17	29	17	9	66	153	25	74	4
Solyc07g052240.2.1	4	38	2	14	1	0	1	5	0	63	48	37	6
Solyc07g052270.2.1	0	0	0	0	0	0	0	0	0	0	0	0	0
Solyc07g052290.1.1	13	198	27	135	1	61	243	237	8	80	27	57	5
Solyc07g052310.1.1	0	0	0	0	0	0	0	0	0	0	0	0	0
Solyc07g052320.2.1	2	13	4	5	1	4	9	11	6	2	4	2	0
Solyc07g052340.2.1	3	5	1	1	3	11	6	7	5	13	1	6	0
Solyc07g052350.2.1	18	743	89	438	48	2242	1230	1965	125	1110	329	810	20
Solyc07g052380.2.1	0	0	0	0	0	0	0	0	0	0	0	0	0
Solyc07g052390.2.1	4	18	3	3	8	17	22	31	8	166	25	97	18
Solyc07g052400.2.1	5	23	3	25	0	8	29	13	3	13	4	20	0
Solyc07g052420.2.1	1	1	0	2	0	9	2	2	0	5	3	4	0
Solyc07g052430.2.1	2	13	0	4	0	1	5	2	0	13	8	6	0
Solyc07g052440.2.1	0	0	0	0	0	0	0	0	0	0	0	0	0
Solyc07g052460.2.1	0	0	0	0	0	0	0	0	0	0	0	0	0
Solyc07g052470.2.1	0	0	0	0	0	0	0	0	0	0	0	0	0
Solyc07g052480.2.1	12	957	8	437	0	588	153	883	0	126	154	61	0
Solyc07g052490.2.1	0	0	0	0	0	0	0	0	0	0	0	0	0
Solyc07g052510.2.1	4	11	1	8	0	15	34	6	0	3	0	7	0
Solyc07g052570.2.1	3	4	0	0	0	23	3	12	3	12	10	8	0
Solyc07g052580.1.1	0	0	0	0	0	0	0	0	0	0	0	0	0
Solyc07g052590.1.1	1	2	0	3	0	3	1	0	0	3	2	3	0
Solyc07g052600.2.1	4	65	7	37	11	184	77	152	9	84	21	83	0
Solyc07g052610.2.1	4	66	23	40	1	101	52	56	0	6	19	23	3
Solyc07g052620.1.1	2	0	0	0	0	38	15	12	1	0	0	0	0
Solyc07g052640.2.1	1	1	0	2	0	7	5	3	0	3	3	4	0
Solyc07g052650.1.1	0	0	0	0	0	0	0	0	0	0	0	0	0
Solyc07g052670.2.1	0	0	0	0	0	0	0	0	0	0	0	0	0

Solyc07g052680.1.1	0	0	0	0	0	0	0	0	0	0	0	0	0
Solyc07g052690.2.1	8	82	4	47	0	34	36	77	1	21	17	34	0
Solyc07g052700.2.1	0	0	0	0	0	0	0	0	0	0	0	0	0
Solyc07g052710.1.1	0	0	0	0	0	0	0	0	0	0	0	0	0
Solyc07g052720.2.1	1	8	5	1	0	9	11	0	0	89	0	11	0
Solyc07g052730.2.1	0	0	0	0	0	0	0	0	0	0	0	0	0
Solyc07g052740.2.1	4	1	0	0	0	10	7	19	0	20	0	13	0
Solyc07g052760.1.1	0	0	0	0	0	0	0	0	0	0	0	0	0
Solyc07g052770.2.1	0	0	0	0	0	0	0	0	0	0	0	0	0
Solyc07g052780.2.1	0	0	0	0	0	0	0	0	0	0	0	0	0
Solyc07g052790.1.1	0	0	0	0	0	0	0	0	0	0	0	0	0
Solyc07g052830.2.1	0	0	0	0	0	0	0	0	0	0	0	0	0
Solyc07g052850.1.1	0	0	0	0	0	0	0	0	0	0	0	0	0
Solyc07g052870.1.1	0	0	0	0	0	0	0	0	0	0	0	0	0
Solyc07g052880.2.1	0	0	0	0	0	0	0	0	0	0	0	0	0
Solyc07g052900.1.1	1	22	1	9	4	11	9	57	3	31	3	14	0
Solyc07g052910.2.1	0	0	0	0	0	0	0	0	0	0	0	0	0
Solyc07g052920.2.1	2	5	0	3	2	18	15	14	12	6	9	9	0
Solyc07g052930.2.1	0	0	0	0	0	0	0	0	0	0	0	0	0
Solyc07g052940.2.1	29	123	13	108	10	265	190	438	35	204	69	165	1
Solyc07g052950.2.1	1	171	1	66	18	58	12	0	9	0	0	0	0
Solyc07g052960.1.1	2	0	0	0	0	0	0	0	0	10	16	6	0
Solyc07g052970.2.1	0	0	0	0	0	0	0	0	0	0	0	0	0
Solyc07g052980.2.1	8	523	34	229	93	8	134	0	4	88	77	91	27
Solyc07g052990.1.1	0	0	0	0	0	0	0	0	0	0	0	0	0
Solyc07g053000.1.1	0	0	0	0	0	0	0	0	0	0	0	0	0
Solyc07g053010.1.1	12	62	6	50	0	162	46	114	0	22	9	21	0
Solyc07g053020.1.1	16	134	28	132	13	60	139	64	2	89	25	93	4
Solyc07g053030.2.1	0	0	0	0	0	0	0	0	0	0	0	0	0
Solyc07g053050.1.1	1	41	8	32	3	0	3	11	3	5	0	8	0
Solyc07g053060.2.1	9	30	3	11	4	60	54	43	4	45	17	52	0
Solyc07g053080.2.1	0	0	0	0	0	0	0	0	0	0	0	0	0
Solyc07g053090.2.1	0	0	0	0	0	0	0	0	0	0	0	0	0
Solyc07g053100.2.1	0	0	0	0	0	0	0	0	0	0	0	0	0
Solyc07g053120.2.1	0	0	0	0	0	0	0	0	0	0	0	0	0
Solyc07g053130.2.1	0	0	0	0	0	0	0	0	0	0	0	0	0
Solyc07g053140.2.1	1	8	4	2	0	8	1	0	2	3	0	2	0
Solyc07g053150.2.1	4	16	1	8	0	12	18	27	0	15	5	12	1
Solyc07g053160.2.1	2	7	3	8	0	21	12	17	2	9	10	9	0
Solyc07g053170.2.1	14	122	15	108	10	236	161	237	16	98	40	122	3
Solyc07g053180.2.1	7	105	11	53	0	236	54	148	10	76	13	88	0

Solyc07g053190.2.1	17	104	3	90	7	326	70	250	10	226	40	196	0
Solyc07g053200.2.1	4	25	5	7	5	178	18	173	26	3	0	1	0
Solyc07g053210.2.1	0	0	0	0	0	0	0	0	0	0	0	0	0
Solyc07g053220.1.1	23	236	24	183	9	479	110	368	17	174	31	128	9
Solyc07g053230.2.1	0	0	0	0	0	0	0	0	0	0	0	0	0
Solyc07g053250.2.1	0	0	0	0	0	0	0	0	0	0	0	0	0
Solyc07g053260.2.1	4	503	43	430	31	971	624	1458	118	879	165	864	15
Solyc07g053270.2.1	14	80	9	35	0	1311	331	645	57	8	8	8	2
Solyc07g053280.2.1	15	760	170	620	339	1274	1117	1619	255	2240	491	2257	28
Solyc07g053290.2.1	2	14	0	18	1	6	12	20	0	28	0	9	0
Solyc07g053310.2.1	9	33	1	18	2	86	27	105	14	185	13	110	0
Solyc07g053320.2.1	10	20	1	12	0	86	68	172	12	51	18	50	0
Solyc07g053340.2.1	0	0	0	0	0	0	0	0	0	0	0	0	0
Solyc07g053350.2.1	0	0	0	0	0	0	0	0	0	0	0	0	0
Solyc07g053360.2.1	23	3	0	9	0	495	564	273	25	12	132	17	1
Solyc07g053380.1.1	0	0	0	0	0	0	0	0	0	0	0	0	0
Solyc07g053390.1.1	0	0	0	0	0	0	0	0	0	0	0	0	0
Solyc07g053410.2.1	3	39	19	16	0	18	50	15	0	2	0	0	0
Solyc07g053420.2.1	0	0	0	0	0	0	0	0	0	0	0	0	0
Solyc07g053430.2.1	1	28	5	21	0	34	4	46	3	41	3	19	0
Solyc07g053440.2.1	15	83	10	71	8	151	157	254	38	110	29	86	1
Solyc07g053450.2.1	0	0	0	0	0	0	0	0	0	0	0	0	0
Solyc07g053470.2.1	8	36	4	38	3	144	53	219	6	129	21	145	1
Solyc07g053480.2.1	5	100	8	25	15	65	32	126	13	87	6	115	4
Solyc07g053490.2.1	5	11	0	16	0	29	34	17	0	11	10	13	0
Solyc07g053520.1.1	0	0	0	0	0	0	0	0	0	0	0	0	0
Solyc07g053530.1.1	1	1	0	0	0	13	1	4	0	0	3	0	0
Solyc07g053540.1.1	4	165	13	68	33	2	1	0	0	138	6	78	0
Solyc07g053550.1.1	9	40	19	38	3	31	5	13	2	26	16	26	1
Solyc07g053560.2.1	0	0	0	0	0	0	0	0	0	0	0	0	0
Solyc07g053570.2.1	0	0	0	0	0	0	0	0	0	0	0	0	0
Solyc07g053580.1.1	2	1	3	4	3	9	5	19	3	16	10	30	0
Solyc07g053590.2.1	0	0	0	0	0	0	0	0	0	0	0	0	0
Solyc07g053600.2.1	7	35	2	19	19	38	17	19	33	15	3	4	0
Solyc07g053610.2.1	18	99	35	67	6	122	110	165	32	198	55	184	7
Solyc07g053620.2.1	0	0	0	0	0	0	0	0	0	0	0	0	0
Solyc07g053630.2.1	5	150	14	154	1	61	38	151	0	10	0	6	0
Solyc07g053640.1.1	8	48	6	28	15	21	85	87	49	14	11	21	1
Solyc07g053650.2.1	15	297	31	185	28	743	420	770	83	693	155	567	8
Solyc07g053660.2.1	4	8	0	8	1	32	26	43	6	21	17	51	2
Solyc07g053670.2.1	3	27	0	4	0	17	8	20	1	35	0	14	0

Solyc07g053690.2.1	2	76	20	25	2	45	113	96	24	56	31	33	0
Solyc07g053700.2.1	0	0	0	0	0	0	0	0	0	0	0	0	0
Solyc07g053710.2.1	7	38	2	32	0	101	58	127	8	110	9	83	0
Solyc07g053720.2.1	10	102	15	58	0	193	85	282	5	150	47	118	11
Solyc07g053730.2.1	10	16	1	15	0	22	51	48	8	48	7	36	0
Solyc07g053740.1.1	12	528	34	290	37	388	142	251	50	234	17	267	5
Solyc07g053750.1.1	3	2	0	0	0	20	10	15	2	2	12	0	0
Solyc07g053760.2.1	3	44	8	21	9	25	35	17	7	5	1	9	5
Solyc07g053790.1.1	0	0	0	0	0	0	0	0	0	0	0	0	0
Solyc07g053800.2.1	8	36	3	32	0	56	15	44	3	16	15	10	1
Solyc07g053810.2.1	0	0	0	0	0	0	0	0	0	0	0	0	0
Solyc07g053820.2.1	0	0	0	0	0	0	0	0	0	0	0	0	0
Solyc07g053830.2.1	6	181	50	188	1	640	230	1231	59	1235	313	688	30
Solyc07g053840.1.1	0	0	0	0	0	0	0	0	0	0	0	0	0
Solyc07g053850.2.1	1	2	2	8	0	7	10	11	3	15	1	4	0
Solyc07g053860.2.1	5	18	1	5	0	16	15	53	3	31	6	12	0
Solyc07g053870.2.1	1	2	0	0	0	0	4	12	0	8	0	0	0
Solyc07g053890.2.1	8	10	0	4	3	156	153	50	238	0	0	0	0
Solyc07g053900.2.1	0	0	0	0	0	0	0	0	0	0	0	0	0
Solyc07g053910.2.1	29	276	12	212	11	656	301	899	130	233	40	174	14
Solyc07g053920.2.1	3	21	1	11	7	14	12	8	2	10	6	17	2
Solyc07g053930.1.1	0	0	0	0	0	0	0	0	0	0	0	0	0
Solyc07g053940.1.1	0	0	0	0	0	0	0	0	0	0	0	0	0
Solyc07g053950.1.1	0	0	0	0	0	0	0	0	0	0	0	0	0
Solyc07g053960.2.1	9	41	12	32	2	77	60	59	15	58	30	55	4
Solyc07g053970.2.1	19	169	24	96	16	206	359	487	215	220	8	117	2
Solyc07g053980.2.1	26	168	20	116	10	628	339	547	31	116	42	151	0
Solyc07g053990.2.1	7	84	18	83	0	140	97	232	38	79	28	66	6
Solyc07g054000.2.1	0	0	0	0	0	0	0	0	0	0	0	0	0
Solyc07g054030.1.1	0	0	0	0	0	0	0	0	0	0	0	0	0
Solyc07g054060.2.1	0	0	0	0	0	0	0	0	0	0	0	0	0
Solyc07g054070.2.1	3	4	0	0	2	4	19	5	1	5	9	10	0
Solyc07g054080.1.1	13	142	11	127	6	462	522	806	25	396	22	244	27
Solyc07g054090.2.1	2	1	1	0	0	3	16	7	2	7	5	3	0
Solyc07g054100.1.1	1	3	0	0	1	1	2	2	0	5	4	2	0
Solyc07g054110.2.1	0	0	0	0	0	0	0	0	0	0	0	0	0
Solyc07g054120.1.1	0	0	0	0	0	0	0	0	0	0	0	0	0
Solyc07g054140.2.1	2	15	0	3	5	9	22	8	6	53	6	70	0
Solyc07g054160.2.1	8	58	5	35	2	88	43	103	12	93	20	69	0
Solyc07g054170.2.1	0	0	0	0	0	0	0	0	0	0	0	0	0
Solyc07g054180.2.1	0	0	0	0	0	0	0	0	0	0	0	0	0

Solyc07g054190.2.1	5	26	2	20	0	73	50	64	3	74	20	54	1
Solyc07g054200.2.1	21	62	8	79	13	123	130	236	21	128	64	88	1
Solyc07g054210.2.1	8	494	70	281	10	86	140	138	21	252	229	376	1
Solyc07g054220.1.1	2	9	0	7	0	40	11	17	3	3	3	12	0
Solyc07g054230.2.1	0	0	0	0	0	0	0	0	0	0	0	0	0
Solyc07g054270.2.1	7	48	29	35	4	287	207	188	54	114	115	174	4
Solyc07g054280.1.1	0	0	0	0	0	0	0	0	0	0	0	0	0
Solyc07g054290.1.1	8	242	26	122	0	36	38	81	4	60	14	55	0
Solyc07g054300.2.1	2	5	0	6	3	31	13	33	9	2	6	9	2
Solyc07g054310.1.1	7	22	0	0	0	4	0	10	0	1774	65	19	134
Solyc07g054320.2.1	8	73	3	51	8	88	43	97	9	152	24	116	0
Solyc07g054330.2.1	1	4	2	1	0	6	12	5	0	16	0	13	1
Solyc07g054340.1.1	0	0	0	0	0	0	0	0	0	0	0	0	0
Solyc07g054370.2.1	2	7	1	7	1	20	25	11	3	4	5	8	0
Solyc07g054400.1.1	0	0	0	0	0	0	0	0	0	0	0	0	0
Solyc07g054410.1.1	0	0	0	0	0	0	0	0	0	0	0	0	0
Solyc07g054420.2.1	2	13	1	7	0	3	5	5	0	16	2	4	1
Solyc07g054430.2.1	3	11	0	15	1	2	15	10	1	17	0	3	0
Solyc07g054440.2.1	8	27	1	9	3	58	55	65	2	55	24	24	1
Solyc07g054450.2.1	7	21	1	14	8	60	36	38	0	119	95	88	1
Solyc07g054460.2.1	8	26	0	29	6	112	32	210	10	102	25	116	5
Solyc07g054470.1.1	1	13	1	8	0	205	4	66	2	2	0	1	0
Solyc07g054480.1.1	1	3	1	0	0	5	4	11	0	0	2	1	2
Solyc07g054490.2.1	4	64	1	7	5	20	32	24	8	118	11	68	0
Solyc07g054500.2.1	8	64	3	24	5	91	192	140	14	15	5	23	0
Solyc07g054540.2.1	4	73	5	57	1	84	67	59	11	35	35	35	0
Solyc07g054560.1.1	0	0	0	0	0	0	0	0	0	0	0	0	0
Solyc07g054580.2.1	0	0	0	0	0	0	0	0	0	0	0	0	0
Solyc07g054600.2.1	0	0	0	0	0	0	0	0	0	0	0	0	0
Solyc07g054610.2.1	0	0	0	0	0	0	0	0	0	0	0	0	0
Solyc07g054620.2.1	0	0	0	0	0	0	0	0	0	0	0	0	0
Solyc07g054670.2.1	0	0	0	0	0	0	0	0	0	0	0	0	0
Solyc07g054680.2.1	1	2	0	3	1	6	6	6	0	22	1	21	0
Solyc07g054690.1.1	0	0	0	0	0	0	0	0	0	0	0	0	0
Solyc07g054720.1.1	0	0	0	0	0	0	0	0	0	0	0	0	0
Solyc07g054730.1.1	5	338	90	342	77	377	3506	881	204	488	232	244	14
Solyc07g054740.2.1	0	0	0	0	0	0	0	0	0	0	0	0	0
Solyc07g054760.1.1	2	15	2	4	0	75	4	21	0	161	5	124	2
Solyc07g054770.1.1	0	0	0	0	0	0	0	0	0	0	0	0	0
Solyc07g054780.1.1	4	99	5	18	5	135	13	69	1	1314	119	745	98
Solyc07g054790.1.1	0	0	0	0	0	0	0	0	0	0	0	0	0

Solyc07g054820.2.1	0	0	0	0	0	0	0	0	0	0	0	0	0
Solyc07g054830.2.1	3	22	2	41	1	110	12	86	4	24	8	31	0
Solyc07g054840.2.1	0	0	0	0	0	0	0	0	0	0	0	0	0
Solyc07g054850.2.1	7	117	15	63	5	168	31	444	1	73	39	97	0
Solyc07g054860.1.1	11	0	0	0	0	3	0	5	0	125	110	282	0
Solyc07g054880.2.1	0	0	0	0	0	0	0	0	0	0	0	0	0
Solyc07g054900.2.1	0	0	0	0	0	0	0	0	0	0	0	0	0
Solyc07g054980.1.1	0	0	0	0	0	0	0	0	0	0	0	0	0
Solyc07g055020.2.1	0	0	0	0	0	0	0	0	0	0	0	0	0
Solyc07g055030.1.1	1	2	0	0	0	0	1	0	2	9	3	10	0
Solyc07g055040.1.1	0	0	0	0	0	0	0	0	0	0	0	0	0
Solyc07g055050.2.1	13	295	15	115	5	23	176	112	6	288	5	260	1
Solyc07g055060.2.1	18	359	104	296	100	1538	290	837	68	491	94	441	9
Solyc07g055080.2.1	2	8	0	5	0	12	5	17	0	8	2	5	0
Solyc07g055100.2.1	0	0	0	0	0	0	0	0	0	0	0	0	0
Solyc07g055120.2.1	7	6	1	3	4	48	41	98	1	20	10	14	0
Solyc07g055130.2.1	2	19	1	10	4	17	12	13	2	14	9	2	0
Solyc07g055140.2.1	3	5	1	10	0	5	9	8	2	16	6	18	0
Solyc07g055150.2.1	6	21	1	8	0	32	27	38	2	41	7	31	0
Solyc07g055160.2.1	0	0	0	0	0	0	0	0	0	0	0	0	0
Solyc07g055170.1.1	0	0	0	0	0	0	0	0	0	0	0	0	0
Solyc07g055180.2.1	0	0	0	0	0	0	0	0	0	0	0	0	0
Solyc07g055190.2.1	0	0	0	0	0	0	0	0	0	0	0	0	0
Solyc07g055200.2.1	3	57	6	51	2	27	19	83	7	111	31	98	2
Solyc07g055210.2.1	10	164	5	87	22	158	97	275	31	467	139	421	8
Solyc07g055220.1.1	0	0	0	0	0	0	0	0	0	0	0	0	0
Solyc07g055230.2.1	0	0	0	0	0	0	0	0	0	0	0	0	0
Solyc07g055240.1.1	0	0	0	0	0	0	0	0	0	0	0	0	0
Solyc07g055250.2.1	1	6	0	0	0	7	5	8	0	32	0	21	0
Solyc07g055260.2.1	4	320	22	229	1	182	58	142	0	29	22	11	0
Solyc07g055270.1.1	0	0	0	0	0	0	0	0	0	0	0	0	0
Solyc07g055280.2.1	0	0	0	0	0	0	0	0	0	0	0	0	0
Solyc07g055290.2.1	3	24	2	18	0	30	25	38	0	46	8	66	1
Solyc07g055300.2.1	9	118	4	70	4	114	53	188	10	134	23	76	4
Solyc07g055310.1.1	0	0	0	0	0	0	0	0	0	0	0	0	0
Solyc07g055320.2.1	10	1412	205	1120	25	463	853	997	46	671	213	703	14
Solyc07g055330.2.1	4	20	1	7	0	47	34	32	0	5	0	3	0
Solyc07g055340.2.1	5	37	3	36	2	27	19	37	0	22	11	31	2
Solyc07g055440.1.1	0	0	0	0	0	0	0	0	0	0	0	0	0
Solyc07g055470.2.1	8	61	5	43	0	99	137	191	3	26	9	27	0
Solyc07g055480.1.1	0	0	0	0	0	0	0	0	0	0	0	0	0

Solyc07g055490.2.1	0	0	0	0	0	0	0	0	0	0	0	0	0
Solyc07g055530.2.1	2	5	0	3	0	33	7	19	2	9	0	11	0
Solyc07g055540.2.1	1	14	0	4	0	42	9	78	3	41	0	19	0
Solyc07g055550.1.1	0	0	0	0	0	0	0	0	0	0	0	0	0
Solyc07g055560.2.1	1	12	0	5	0	155	0	105	0	0	0	0	0
Solyc07g055630.2.1	0	0	0	0	0	0	0	0	0	0	0	0	0
Solyc07g055640.1.1	0	0	0	0	0	0	0	0	0	0	0	0	0
Solyc07g055650.1.1	0	0	0	0	0	0	0	0	0	0	0	0	0
Solyc07g055660.2.1	3	91	5	48	0	62	53	88	1	29	13	62	0
Solyc07g055670.1.1	0	0	0	0	0	0	0	0	0	0	0	0	0
Solyc07g055690.1.1	1	2	1	9	0	9	4	2	0	2	0	0	0
Solyc07g055700.2.1	3	13	1	11	0	8	13	18	0	21	2	15	0
Solyc07g055710.2.1	0	0	0	0	0	0	0	0	0	0	0	0	0
Solyc07g055720.2.1	5	101	0	36	1	4	60	27	0	13	4	13	0
Solyc07g055740.1.1	1	6	4	0	4	0	0	0	1	0	0	0	0
Solyc07g055750.2.1	0	0	0	0	0	0	0	0	0	0	0	0	0
Solyc07g055760.1.1	0	0	0	0	0	0	0	0	0	0	0	0	0
Solyc07g055770.1.1	0	0	0	0	0	0	0	0	0	0	0	0	0
Solyc07g055780.2.1	0	0	0	0	0	0	0	0	0	0	0	0	0
Solyc07g055810.2.1	1	11	3	5	0	4	2	0	0	0	0	0	0
Solyc07g055830.2.1	7	87	10	56	11	155	45	231	13	151	9	93	3
Solyc07g055840.2.1	12	29	2	10	5	195	131	84	4	20	17	17	0
Solyc07g055850.2.1	0	0	0	0	0	0	0	0	0	0	0	0	0
Solyc07g055860.2.1	9	85	3	68	0	214	48	228	8	149	33	160	0
Solyc07g055870.2.1	0	0	0	0	0	0	0	0	0	0	0	0	0
Solyc07g055880.1.1	0	0	0	0	0	0	0	0	0	0	0	0	0
Solyc07g055900.1.1	1	4	1	4	0	6	3	5	0	9	0	0	0
Solyc07g055910.2.1	0	0	0	0	0	0	0	0	0	0	0	0	0
Solyc07g055920.2.1	1	0	0	0	0	0	0	1	0	161	26	59	4
Solyc07g055930.2.1	7	44	4	32	5	48	33	55	27	33	22	12	7
Solyc07g055940.1.1	1	3	1	0	0	6	2	7	1	2	1	0	0
Solyc07g055950.2.1	1	10	0	3	4	0	0	0	2	0	2	0	0
Solyc07g055980.2.1	2	8	1	13	0	29	17	24	0	31	0	24	1
Solyc07g055990.2.1	1	5	0	0	0	0	5	0	0	0	0	6	0
Solyc07g056000.2.1	8	605	72	192	41	4	4	11	2	25	0	256	6
Solyc07g056010.2.1	7	38	8	27	0	92	15	114	5	138	25	84	0
Solyc07g056020.2.1	30	723	166	549	6	292	695	653	28	213	149	262	9
Solyc07g056030.2.1	9	90	1	42	7	211	58	180	12	182	40	187	1
Solyc07g056040.2.1	1	10	0	2	1	7	1	7	6	8	0	2	0
Solyc07g056050.1.1	14	270	35	168	13	496	232	572	28	298	77	268	0
Solyc07g056060.2.1	0	0	0	0	0	0	0	0	0	0	0	0	0

Solyc07g056070.1.1	1	17	2	20	0	45	18	27	3	18	1	6	3
Solyc07g056080.1.1	0	0	0	0	0	0	0	0	0	0	0	0	0
Solyc07g056110.2.1	7	28	0	28	1	118	17	107	14	52	12	46	1
Solyc07g056120.2.1	3	17	5	17	0	43	12	39	3	28	8	16	1
Solyc07g056140.2.1	12	1295	74	698	14	317	149	614	10	1816	295	1333	34
Solyc07g056150.2.1	3	43	5	23	3	111	70	110	28	84	19	19	0
Solyc07g056160.2.1	0	0	0	0	0	0	0	0	0	0	0	0	0
Solyc07g056170.2.1	0	0	0	0	0	0	0	0	0	0	0	0	0
Solyc07g056180.1.1	0	0	0	0	0	0	0	0	0	0	0	0	0
Solyc07g056190.2.1	5	115	76	126	12	135	26	42	28	1	0	10	8
Solyc07g056200.2.1	6	50	47	28	16	88	385	46	140	6	7	1	2
Solyc07g056210.2.1	0	0	0	0	0	0	0	0	0	0	0	0	0
Solyc07g056220.2.1	7	44	8	52	1	83	83	121	9	168	19	111	7
Solyc07g056230.2.1	0	0	0	0	0	0	0	0	0	0	0	0	0
Solyc07g056240.2.1	8	263	74	120	8	29	46	44	4	62	64	133	1
Solyc07g056250.2.1	3	13	6	6	0	10	1	7	0	1	2	14	0
Solyc07g056260.2.1	11	54	5	57	0	294	111	196	13	251	16	201	0
Solyc07g056270.2.1	2	8	0	5	0	10	16	6	0	13	5	12	0
Solyc07g056280.2.1	0	0	0	0	0	0	0	0	0	0	0	0	0
Solyc07g056310.2.1	0	0	0	0	0	0	0	0	0	0	0	0	0
Solyc07g056320.2.1	0	0	0	0	0	0	0	0	0	0	0	0	0
Solyc07g056330.2.1	0	0	0	0	0	0	0	0	0	0	0	0	0
Solyc07g056340.2.1	11	55	4	63	4	170	102	325	12	102	19	59	1
Solyc07g056350.2.1	9	184	9	77	8	134	85	123	11	377	29	213	3
Solyc07g056370.2.1	9	173	10	75	21	886	98	735	39	214	45	171	4
Solyc07g056390.2.1	6	21	6	9	1	70	26	53	7	20	21	6	0
Solyc07g056400.1.1	1	10	1	2	2	0	3	0	2	1	0	3	0
Solyc07g056410.2.1	7	0	0	7	0	82	40	43	0	0	1	2	0
Solyc07g056420.2.1	4	3349	34	1141	2	2016	43	866	8	438	194	358	0
Solyc07g056430.2.1	3	7	1	1	10	18	26	9	3	7	109	2	0
Solyc07g056440.2.1	5	12	11	11	2	47	35	21	6	12	49	23	5
Solyc07g056460.2.1	0	0	0	0	0	0	0	0	0	0	0	0	0
Solyc07g056470.2.1	1	1	0	3	0	17	3	3	0	2	1	0	0
Solyc07g056480.2.1	6	164	9	38	4	93	73	50	3	54	21	19	0
Solyc07g056490.2.1	3	55	6	26	7	46	51	48	2	112	35	81	8
Solyc07g056500.2.1	7	57	10	21	6	181	28	64	0	57	3	97	0
Solyc07g056510.2.1	0	0	0	0	0	0	0	0	0	0	0	0	0
Solyc07g056520.2.1	7	17	6	9	0	60	37	75	6	49	23	37	0
Solyc07g056530.2.1	1	3	1	4	0	0	5	1	0	5	1	2	0
Solyc07g056540.2.1	4	3739	599	2823	14	791	1848	926	13	161	123	117	1
Solyc07g056550.2.1	7	23	2	40	0	74	27	138	5	77	28	105	2

Solyc07g056570.1.1	24	515	53	608	5	10469	2312	5729	49	535	249	1131	28
Solyc07g056580.2.1	3	16	1	7	2	17	17	27	0	69	5	45	0
Solyc07g056590.2.1	3	15	2	16	0	43	36	58	2	34	5	26	0
Solyc07g056610.1.1	0	0	0	0	0	0	0	0	0	0	0	0	0
Solyc07g056630.2.1	8	81	1	49	3	223	150	126	13	247	57	129	3
Solyc07g056640.1.1	1	95	12	285	0	72	18	52	0	134	4	58	0
Solyc07g056650.2.1	1	5	1	3	0	1	4	0	1	9	0	0	0
Solyc07g056660.2.1	12	108	20	171	6	183	135	255	12	174	24	121	1
Solyc07g056670.2.1	2	2	2	3	0	1	1	5	0	3	4	6	0
Solyc07g061720.2.1	0	0	0	0	0	0	0	0	0	0	0	0	0
Solyc07g061730.2.1	0	0	0	0	0	0	0	0	0	0	0	0	0
Solyc07g061740.2.1	0	0	0	0	0	0	0	0	0	0	0	0	0
Solyc07g061780.2.1	11	62	7	28	5	95	81	182	13	82	29	63	6
Solyc07g061790.2.1	7	1071	66	447	2	1098	275	1017	4	476	181	1057	6
Solyc07g061800.2.1	4	123	9	64	0	4	4	6	0	1	210	0	2
Solyc07g061810.1.1	0	0	0	0	0	0	0	0	0	0	0	0	0
Solyc07g061890.1.1	0	0	0	0	0	0	0	0	0	0	0	0	0
Solyc07g061900.2.1	8	257	18	126	0	81	120	154	3	56	20	69	0
Solyc07g061910.1.1	0	0	0	0	0	0	0	0	0	0	0	0	0
Solyc07g061920.2.1	7	7	2	3	0	45	39	32	2	48	2	34	0
Solyc07g061930.1.1	1	14	2	15	0	8	1	7	0	28	8	7	2
Solyc07g061940.2.1	8	176	16	139	1	160	69	209	3	568	77	534	0
Solyc07g061950.2.1	11	42	12	34	6	194	47	67	12	115	24	52	4
Solyc07g061960.2.1	4	15	2	13	2	21	22	37	9	40	9	25	0
Solyc07g061970.2.1	3	108	16	29	2	60	90	120	17	195	14	111	1
Solyc07g061990.2.1	3	93	21	28	1	37	143	98	11	17	16	16	0
Solyc07g062020.1.1	0	0	0	0	0	0	0	0	0	0	0	0	0
Solyc07g062030.2.1	6	25	3	23	0	43	173	61	6	29	3	74	1
Solyc07g062040.2.1	2	0	0	0	0	4	6	7	0	6	3	0	0
Solyc07g062050.2.1	1	1	0	0	0	0	1	0	0	11	6	7	0
Solyc07g062060.2.1	3	45	8	23	2	18	49	58	3	52	15	29	0
Solyc07g062070.2.1	3	7	0	8	0	13	15	9	2	23	4	3	2
Solyc07g062080.2.1	7	92	9	56	7	137	37	112	13	69	26	79	1
Solyc07g062100.2.1	0	0	0	0	0	0	0	0	0	0	0	0	0
Solyc07g062110.2.1	5	26	1	8	1	57	27	60	3	50	25	50	0
Solyc07g062120.2.1	1	5	0	4	0	1	6	6	0	2	1	3	0
Solyc07g062130.2.1	17	819	94	484	120	1766	339	725	105	343	138	350	6
Solyc07g062140.2.1	4	82	5	49	0	18	12	62	1	203	3	234	0
Solyc07g062150.2.1	0	0	0	0	0	0	0	0	0	0	0	0	0
Solyc07g062160.2.1	9	99	7	67	0	140	52	140	13	152	19	66	1
Solyc07g062170.1.1	0	0	0	0	0	0	0	0	0	0	0	0	0

Solyc07g062180.2.1	8	83	8	18	0	162	89	146	11	60	17	55	1
Solyc07g062190.2.1	4	477	30	259	8	179	143	278	3	300	18	223	3
Solyc07g062210.2.1	2	7	0	8	0	11	9	10	0	0	0	0	0
Solyc07g062220.2.1	4	18	2	6	0	101	26	101	3	23	6	18	0
Solyc07g062250.2.1	13	125	16	93	2	153	55	147	18	165	12	143	5
Solyc07g062260.2.1	3	28	1	6	2	19	38	33	2	7	5	6	0
Solyc07g062270.2.1	4	12	3	2	2	3	22	8	3	18	6	11	1
Solyc07g062290.2.1	0	0	0	0	0	0	0	0	0	0	0	0	0
Solyc07g062300.2.1	6	22	5	17	1	14	19	16	2	39	7	30	0
Solyc07g062310.2.1	6	50	10	30	4	20	10	13	2	11	9	9	0
Solyc07g062390.2.1	3	15	0	17	0	9	8	6	1	25	4	11	0
Solyc07g062400.2.1	2	10	1	4	9	32	37	41	2	32	4	45	0
Solyc07g062410.2.1	5	13	9	6	0	28	19	37	3	24	14	29	0
Solyc07g062430.2.1	1	6	0	3	0	0	9	5	0	0	8	6	0
Solyc07g062440.1.1	8	13	2	11	1	65	68	83	1	30	21	19	2
Solyc07g062450.2.1	1	12	1	6	3	26	9	15	2	30	5	11	0
Solyc07g062460.2.1	0	0	0	0	0	0	0	0	0	0	0	0	0
Solyc07g062470.2.1	0	0	0	0	0	0	0	0	0	0	0	0	0
Solyc07g062480.1.1	5	21	2	4	1	304	796	98	2	3	60	0	5
Solyc07g062490.1.1	11	148	1	46	0	97	606	263	13	41	25	18	3
Solyc07g062500.2.1	19	130	10	86	1	1274	249	1341	29	97	227	55	3
Solyc07g062510.2.1	0	0	0	0	0	0	0	0	0	0	0	0	0
Solyc07g062520.2.1	5	36	11	12	0	48	23	55	2	46	33	11	0
Solyc07g062530.2.1	20	8	9	15	6	69	832	256	52	5472	1200	3889	41
Solyc07g062540.2.1	3	4	4	9	2	39	20	49	6	12	14	23	0
Solyc07g062550.2.1	1	1	0	0	0	0	3	1	0	11	0	13	0
Solyc07g062560.2.1	0	0	0	0	0	0	0	0	0	0	0	0	0
Solyc07g062570.2.1	5	202	23	92	6	267	177	318	42	323	77	207	17
Solyc07g062580.2.1	7	81	5	16	5	50	28	43	9	105	12	47	1
Solyc07g062590.2.1	0	0	0	0	0	0	0	0	0	0	0	0	0
Solyc07g062600.2.1	9	54	6	21	5	93	64	125	8	91	26	69	7
Solyc07g062610.2.1	5	64	7	55	3	132	58	182	5	132	12	70	2
Solyc07g062620.2.1	5	38	0	12	4	12	32	26	7	20	5	19	1
Solyc07g062630.1.1	0	0	0	0	0	0	0	0	0	0	0	0	0
Solyc07g062640.2.1	16	35	5	45	1	76	56	142	3	104	22	70	4
Solyc07g062650.2.1	9	598	64	319	46	1007	391	1290	139	786	206	656	15
Solyc07g062660.2.1	12	55	10	60	3	97	45	154	15	111	37	59	1
Solyc07g062670.1.1	0	0	0	0	0	0	0	0	0	0	0	0	0
Solyc07g062680.1.1	1	10	2	4	0	0	3	7	0	11	0	1	0
Solyc07g062690.1.1	1	6	4	4	3	45	8	14	4	0	1	0	0
Solyc07g062700.2.1	12	900	64	577	42	6090	403	5985	478	512	44	506	21

Solyc07g062710.2.1	0	0	0	0	0	0	0	0	0	0	0	0	0
Solyc07g062720.1.1	0	0	0	0	0	0	0	0	0	0	0	0	0
Solyc07g062730.1.1	10	78	13	38	8	19	61	40	5	38	7	23	6
Solyc07g062740.2.1	8	18	5	5	2	78	126	62	22	28	9	15	0
Solyc07g062750.2.1	1	1	0	1	0	0	30	1	1	3	13	2	2
Solyc07g062760.1.1	3	7	1	4	0	3	22	4	3	4	5	7	0
Solyc07g062770.2.1	3	6	0	10	0	4	12	11	3	14	8	20	1
Solyc07g062780.2.1	1	6	1	2	0	0	10	3	0	4	1	2	1
Solyc07g062810.2.1	0	0	0	0	0	0	0	0	0	0	0	0	0
Solyc07g062820.1.1	3	7	3	2	0	17	20	22	1	26	2	18	0
Solyc07g062840.2.1	0	0	0	0	0	0	0	0	0	0	0	0	0
Solyc07g062850.2.1	7	15	0	15	0	129	97	110	3	35	11	46	0
Solyc07g062860.2.1	2	13	0	13	0	135	10	77	0	29	11	8	0
Solyc07g062870.2.1	2	141	9	52	0	11	25	39	1	47	11	38	1
Solyc07g062880.2.1	0	0	0	0	0	0	0	0	0	0	0	0	0
Solyc07g062890.2.1	0	0	0	0	0	0	0	0	0	0	0	0	0
Solyc07g062910.2.1	2	6	5	11	1	13	9	17	3	13	3	24	0
Solyc07g062920.2.1	8	33	3	10	3	82	61	81	11	56	19	60	6
Solyc07g062930.2.1	0	0	0	0	0	0	0	0	0	0	0	0	0
Solyc07g062940.2.1	14	71	12	48	15	125	44	136	9	115	23	92	0
Solyc07g062950.2.1	0	0	0	0	0	0	0	0	0	0	0	0	0
Solyc07g062960.1.1	0	0	0	0	0	0	0	0	0	0	0	0	0
Solyc07g062970.2.1	8	60	3	40	0	477	330	664	15	241	33	113	0
Solyc07g062980.2.1	0	0	0	0	0	0	0	0	0	0	0	0	0
Solyc07g062990.1.1	0	0	0	0	0	0	0	0	0	0	0	0	0
Solyc07g063000.2.1	1	3	0	0	0	15	9	8	1	0	0	0	0
Solyc07g063010.2.1	10	58	9	25	19	70	148	98	37	279	87	209	11
Solyc07g063020.1.1	0	0	0	0	0	0	0	0	0	0	0	0	0
Solyc07g063040.2.1	9	110	42	73	13	297	237	200	73	532	165	390	13
Solyc07g063050.1.1	2	31	2	40	0	28	4	43	2	241	6	137	0
Solyc07g063070.1.1	4	26	7	11	0	9	13	25	6	15	4	16	0
Solyc07g063080.2.1	0	0	0	0	0	0	0	0	0	0	0	0	0
Solyc07g063100.2.1	10	350	36	243	36	812	529	1078	158	727	280	506	13
Solyc07g063110.2.1	4	28	1	13	0	57	25	76	17	25	8	11	2
Solyc07g063120.2.1	4	29	9	16	0	9	24	27	4	43	4	44	0
Solyc07g063130.2.1	5	22	1	25	0	24	18	24	3	42	17	39	1
Solyc07g063140.1.1	1	19	0	10	0	9	4	30	0	27	0	17	0
Solyc07g063150.2.1	2	27	0	12	0	89	53	71	17	32	35	15	0
Solyc07g063160.2.1	2	33	0	8	0	65	14	75	2	38	1	26	0
Solyc07g063170.1.1	1	8	1	2	1	6	1	5	1	7	5	12	6
Solyc07g063180.2.1	0	0	0	0	0	0	0	0	0	0	0	0	0

Solyc07g063190.2.1	8	720	75	461	20	188	597	548	22	616	94	472	8
Solyc07g063200.1.1	4	29	28	22	9	14	50	23	7	28	11	26	0
Solyc07g063220.2.1	2	5	0	0	0	22	6	22	0	19	5	7	0
Solyc07g063230.2.1	2	10	0	3	0	2	8	7	2	27	8	41	0
Solyc07g063240.2.1	9	43	3	53	4	66	27	59	4	86	29	110	3
Solyc07g063250.2.1	0	0	0	0	0	0	0	0	0	0	0	0	0
Solyc07g063260.2.1	6	27	4	20	3	51	45	50	11	36	5	36	0
Solyc07g063270.2.1	11	82	42	76	13	168	332	260	35	454	241	283	19
Solyc07g063290.2.1	1	4	0	0	0	1	1	5	0	13	4	3	0
Solyc07g063300.2.1	5	14	0	6	0	5	19	12	4	19	16	33	0
Solyc07g063310.2.1	7	13	0	24	0	23	29	62	0	90	10	73	1
Solyc07g063320.2.1	0	0	0	0	0	0	0	0	0	0	0	0	0
Solyc07g063330.2.1	10	47	4	43	3	46	24	60	17	102	55	75	2
Solyc07g063340.2.1	6	82	1	38	3	89	22	121	4	776	47	763	1
Solyc07g063350.2.1	8	55	6	30	10	11	17	11	2	98	37	122	0
Solyc07g063390.2.1	19	68	19	35	9	233	266	256	10	184	69	190	19
Solyc07g063410.2.1	14	23	1	32	1	1485	690	640	58	80	12	46	0
Solyc07g063420.2.1	6	1	0	2	0	23	23	24	3	147	11	154	0
Solyc07g063430.2.1	9	946	125	730	8	477	505	455	8	737	130	659	20
Solyc07g063440.2.1	11	61	8	69	8	175	63	201	8	220	27	163	2
Solyc07g063450.1.1	0	0	0	0	0	0	0	0	0	0	0	0	0
Solyc07g063460.1.1	8	137	11	81	0	33	31	50	19	146	37	87	12
Solyc07g063470.1.1	0	0	0	0	0	0	0	0	0	0	0	0	0
Solyc07g063480.2.1	1	19	0	1	1	4	4	6	1	8	8	13	0
Solyc07g063490.2.1	0	0	0	0	0	0	0	0	0	0	0	0	0
Solyc07g063500.2.1	0	0	0	0	0	0	0	0	0	0	0	0	0
Solyc07g063510.2.1	2	6	1	11	0	11	6	8	0	7	1	7	0
Solyc07g063520.2.1	4	60	4	39	1	497	93	331	40	181	18	158	0
Solyc07g063530.2.1	10	142	8	86	0	115	127	166	7	148	35	85	4
Solyc07g063540.2.1	2	21	0	0	0	5	11	18	2	26	9	15	0
Solyc07g063550.2.1	5	50	30	41	18	90	81	72	35	156	55	103	1
Solyc07g063560.2.1	0	0	0	0	0	0	0	0	0	0	0	0	0
Solyc07g063570.2.1	6	86	7	34	1	133	95	119	1	61	8	59	0
Solyc07g063580.2.1	0	0	0	0	0	0	0	0	0	0	0	0	0
Solyc07g063590.2.1	4	12	0	10	0	15	12	28	0	11	1	3	0
Solyc07g063600.2.1	3	1150	130	517	2	1	7	1	1	47	116	12	1
Solyc07g063610.2.1	4	155	8	57	0	32	67	43	10	94	9	46	1
Solyc07g063620.2.1	5	35	0	4	0	43	53	33	29	25	20	18	0
Solyc07g063630.2.1	5	81	5	56	4	129	73	111	23	123	36	99	0
Solyc07g063640.1.1	1	0	0	0	1	5	2	4	1	6	5	1	1
Solyc07g063650.2.1	8	38	6	39	0	96	58	144	25	34	12	46	0

Solyc07g063680.2.1	5	148	16	101	13	368	182	386	45	328	33	246	0
Solyc07g063690.1.1	1	25	3	4	0	1	0	0	0	2	10	0	0
Solyc07g063700.1.1	0	0	0	0	0	0	0	0	0	0	0	0	0
Solyc07g063710.1.1	0	0	0	0	0	0	0	0	0	0	0	0	0
Solyc07g063720.1.1	0	0	0	0	0	0	0	0	0	0	0	0	0
Solyc07g063740.2.1	5	85	2	17	0	5	8	12	0	557	17	278	5
Solyc07g063750.2.1	0	0	0	0	0	0	0	0	0	0	0	0	0
Solyc07g063770.2.1	1	2	0	0	0	0	4	7	0	2	0	0	0
Solyc07g063820.2.1	0	0	0	0	0	0	0	0	0	0	0	0	0
Solyc07g063830.2.1	0	0	0	0	0	0	0	0	0	0	0	0	0
Solyc07g063850.2.1	5	24	0	8	3	131	26	137	1	3	1	0	0
Solyc07g063860.2.1	3	51	2	36	0	1	9	11	0	68	6	27	0
Solyc07g063870.2.1	14	559	45	315	20	816	404	1131	104	1499	97	1022	10
Solyc07g063880.2.1	3	0	1	0	9	2	101	3	25	19	3	1	2
Solyc07g063890.2.1	5	153	2	66	7	194	33	322	14	1053	42	596	9
Solyc07g063900.2.1	4	89	7	37	5	295	193	236	23	165	70	151	7
Solyc07g063910.2.1	5	58	3	5	5	124	365	21	39	0	2	2	0
Solyc07g063920.2.1	0	0	0	0	0	0	0	0	0	0	0	0	0
Solyc07g063930.2.1	5	4	0	1	0	102	11	269	0	110	32	29	0
Solyc07g063940.1.1	10	95	23	48	4	151	27	92	2	26	13	49	0
Solyc07g063950.2.1	1	5	2	0	1	2	4	2	2	0	0	3	0
Solyc07g063960.2.1	4	322	30	129	3	54	117	108	3	78	22	51	3
Solyc07g063970.2.1	0	0	0	0	0	0	0	0	0	0	0	0	0
Solyc07g064010.2.1	0	0	0	0	0	0	0	0	0	0	0	0	0
Solyc07g064020.2.1	0	0	0	0	0	0	0	0	0	0	0	0	0
Solyc07g064030.2.1	2	5	3	2	0	17	6	9	1	2	3	0	0
Solyc07g064040.2.1	8	249	40	163	9	208	283	323	51	264	68	235	4
Solyc07g064050.2.1	0	0	0	0	0	0	0	0	0	0	0	0	0
Solyc07g064060.2.1	21	88	11	99	3	195	366	320	45	208	61	150	1
Solyc07g064080.2.1	7	22	2	4	6	57	42	45	5	50	9	61	0
Solyc07g064090.2.1	2	6	3	9	3	47	40	23	1	29	2	15	2
Solyc07g064100.1.1	2	13	2	11	0	26	12	22	0	6	0	1	1
Solyc07g064110.2.1	8	18	3	9	0	53	43	47	0	44	7	25	1
Solyc07g064120.1.1	0	0	0	0	0	0	0	0	0	0	0	0	0
Solyc07g064130.1.1	16	17962	2993	10514	2392	45375	24260	49467	7108	22166	10018	11958	429
Solyc07g064140.1.1	0	0	0	0	0	0	0	0	0	0	0	0	0
Solyc07g064150.2.1	2	219	17	118	12	122	143	164	14	630	218	391	34
Solyc07g064160.2.1	11	13585	709	7394	167	1171	403	1462	87	42276	710	34886	42
Solyc07g064170.2.1	19	3	2	18	0	27	2	62	0	9548	1441	16035	17
Solyc07g064180.2.1	19	4	5	7	1	4	7	8	0	1622	1158	2489	16
Solyc07g064190.1.1	0	0	0	0	0	0	0	0	0	0	0	0	0

Solyc07g064210.2.1	0	0	0	0	0	0	0	0	0	0	0	0	0
Solyc07g064220.1.1	0	0	0	0	0	0	0	0	0	0	0	0	0
Solyc07g064230.2.1	0	0	0	0	0	0	0	0	0	0	0	0	0
Solyc07g064240.2.1	2	24	5	10	0	9	19	12	0	13	0	8	0
Solyc07g064250.2.1	6	132	6	81	10	314	146	368	30	258	82	234	6
Solyc07g064260.2.1	0	0	0	0	0	0	0	0	0	0	0	0	0
Solyc07g064270.2.1	5	66	1	49	7	60	12	93	21	403	36	524	3
Solyc07g064280.2.1	6	23	4	27	4	74	35	50	0	114	19	131	4
Solyc07g064290.2.1	4	57	6	22	3	63	15	90	6	64	12	15	0
Solyc07g064300.2.1	6	21	0	22	1	51	22	42	4	70	9	50	0
Solyc07g064310.1.1	5	30	2	26	1	65	7	55	0	44	9	26	0
Solyc07g064320.1.1	0	0	0	0	0	0	0	0	0	0	0	0	0
Solyc07g064330.2.1	15	41	7	28	14	47	137	148	4	50	21	52	3
Solyc07g064340.2.1	0	0	0	0	0	0	0	0	0	0	0	0	0
Solyc07g064350.1.1	9	46	2	20	0	55	20	95	8	54	22	47	1
Solyc07g064360.1.1	0	0	0	0	0	0	0	0	0	0	0	0	0
Solyc07g064400.1.1	0	0	0	0	0	0	0	0	0	0	0	0	0
Solyc07g064410.1.1	0	0	0	0	0	0	0	0	0	0	0	0	0
Solyc07g064420.2.1	2	8	0	10	0	9	8	18	0	3	2	7	0
Solyc07g064440.2.1	7	25	4	43	5	129	89	150	16	23	22	52	2
Solyc07g064450.2.1	0	0	0	0	0	0	0	0	0	0	0	0	0
Solyc07g064470.2.1	0	0	0	0	0	0	0	0	0	0	0	0	0
Solyc07g064480.1.1	0	0	0	0	0	0	0	0	0	0	0	0	0
Solyc07g064490.2.1	2	9	1	1	0	9	5	11	0	17	2	1	0
Solyc07g064500.2.1	13	109	10	56	11	266	90	377	5	373	30	358	1
Solyc07g064510.2.1	7	212	13	172	10	888	177	609	48	827	135	555	13
Solyc07g064520.2.1	1	2	0	5	0	1	5	6	1	3	1	5	1
Solyc07g064550.2.1	2	2	0	3	0	7	10	11	0	4	6	5	0
Solyc07g064560.2.1	7	40	3	48	1	122	58	229	16	84	14	62	0
Solyc07g064570.2.1	2	4	3	8	0	1	17	14	1	34	1	22	0
Solyc07g064580.2.1	5	33	2	8	4	42	15	24	1	29	11	38	3
Solyc07g064590.2.1	3	24	1	8	4	32	12	15	3	46	7	26	0
Solyc07g064600.2.1	6	77	9	26	2	501	1506	311	5	4	6	7	0
Solyc07g064610.2.1	5	136	8	94	1	162	61	166	11	151	30	99	0
Solyc07g064620.1.1	0	0	0	0	0	0	0	0	0	0	0	0	0
Solyc07g064630.2.1	4	9	4	19	0	58	32	22	8	42	8	27	0
Solyc07g064640.2.1	1	0	0	0	0	2	0	0	1	11	0	6	0
Solyc07g064650.1.1	0	0	0	0	0	0	0	0	0	0	0	0	0
Solyc07g064660.1.1	0	0	0	0	0	0	0	0	0	0	0	0	0
Solyc07g064670.2.1	4	80	1	34	5	52	14	50	3	243	10	88	0
Solyc07g064680.2.1	1	8	1	3	1	4	4	11	2	19	15	7	0

Solyc07g064690.1.1	2	45	4	7	0	32	5	19	2	52	12	25	0
Solyc07g064700.2.1	13	40	7	56	1	153	67	149	10	114	41	93	1
Solyc07g064710.2.1	0	0	0	0	0	0	0	0	0	0	0	0	0
Solyc07g064720.2.1	2	1	3	1	0	4	0	3	0	0	1	0	0
Solyc07g064730.1.1	0	0	0	0	0	0	0	0	0	0	0	0	0
Solyc07g064740.2.1	3	3	2	11	0	23	34	41	4	57	4	27	0
Solyc07g064750.1.1	0	0	0	0	0	0	0	0	0	0	0	0	0
Solyc07g064760.1.1	0	0	0	0	0	0	0	0	0	0	0	0	0
Solyc07g064790.2.1	0	0	0	0	0	0	0	0	0	0	0	0	0
Solyc07g064800.2.1	6	27	4	13	0	87	42	99	42	112	29	104	3
Solyc07g064810.2.1	2	1	0	0	1	3	2	6	2	13	5	25	0
Solyc07g064820.1.1	1	9	7	4	0	2	0	1	0	0	0	1	0
Solyc07g064830.2.1	2	4	0	0	0	5	6	14	0	5	3	6	0
Solyc07g064840.1.1	3	3	1	2	0	4	11	15	3	8	8	9	0
Solyc07g064850.2.1	6	14	10	11	1	18	39	32	11	17	2	6	0
Solyc07g064860.1.1	0	0	0	0	0	0	0	0	0	0	0	0	0
Solyc07g064870.2.1	0	0	0	0	0	0	0	0	0	0	0	0	0
Solyc07g064880.2.1	1	13	1	18	2	20	23	12	1	23	0	26	1
Solyc07g064890.1.1	4	309	34	180	27	444	308	373	70	650	175	356	6
Solyc07g064900.2.1	8	150	10	66	4	343	111	359	24	186	138	211	0
Solyc07g064910.2.1	6	16	0	25	0	27	32	53	5	68	21	154	1
Solyc07g064920.2.1	1	7	0	5	0	3	8	11	1	13	0	5	0
Solyc07g064930.2.1	4	27	3	5	3	93	44	69	10	37	10	23	0
Solyc07g064940.2.1	8	155	22	96	1	93	171	143	3	81	10	31	0
Solyc07g064950.2.1	8	66	7	29	1	66	76	110	3	56	5	42	0
Solyc07g064960.1.1	0	0	0	0	0	0	0	0	0	0	0	0	0
Solyc07g064970.2.1	10	117	10	72	2	81	102	231	4	293	83	265	3
Solyc07g064980.2.1	16	134	17	121	7	288	99	450	55	221	60	234	3
Solyc07g064990.2.1	0	0	0	0	0	0	0	0	0	0	0	0	0
Solyc07g065000.2.1	7	35	2	15	0	99	29	48	9	35	10	41	3
Solyc07g065010.2.1	15	76	6	28	5	169	325	370	28	250	36	160	3
Solyc07g065020.2.1	1	2	0	5	0	12	2	26	1	28	0	26	0
Solyc07g065030.2.1	2	60	3	40	1	59	43	117	13	172	10	114	1
Solyc07g065040.2.1	0	0	0	0	0	0	0	0	0	0	0	0	0
Solyc07g065050.2.1	7	68	17	47	8	42	78	101	6	35	20	35	3
Solyc07g065060.1.1	0	0	0	0	0	0	0	0	0	0	0	0	0
Solyc07g065070.1.1	0	0	0	0	0	0	0	0	0	0	0	0	0
Solyc07g065080.2.1	21	230	14	85	2	146	77	135	23	154	70	168	6
Solyc07g065090.1.1	16	256	13	84	4	303	37	217	9	459	170	429	17
Solyc07g065110.1.1	0	0	0	0	0	0	0	0	0	0	0	0	0
Solyc07g065120.2.1	20	361	60	198	26	159	173	179	29	307	112	176	7

Solyc07g065130.2.1	0	0	0	0	0	0	0	0	0	0	0	0	0
Solyc07g065140.2.1	5	19	1	6	3	23	15	16	0	27	6	9	1
Solyc07g065160.2.1	0	0	0	0	0	0	0	0	0	0	0	0	0
Solyc07g065170.2.1	3	66	6	12	2	93	53	138	19	168	55	140	14
Solyc07g065180.2.1	2	15	0	31	0	24	11	23	1	22	0	36	0
Solyc07g065200.2.1	4	42	2	27	3	17	24	40	7	75	9	39	1
Solyc07g065210.2.1	0	0	0	0	0	0	0	0	0	0	0	0	0
Solyc07g065220.1.1	2	26	2	65	0	52	21	55	6	279	4	143	0
Solyc07g065230.1.1	8	156	12	139	11	158	219	378	37	490	80	221	2
Solyc07g065250.2.1	1	2	0	0	0	3	3	2	1	1	0	1	0
Solyc07g065260.2.1	12	27	6	38	3	51	57	88	8	115	9	94	0
Solyc07g065270.1.1	14	264	9	160	10	179	85	155	31	213	32	165	0
Solyc07g065280.2.1	4	48	4	30	0	27	60	65	10	65	25	37	0
Solyc07g065290.2.1	0	0	0	0	0	0	0	0	0	0	0	0	0
Solyc07g065310.2.1	4	19	2	9	6	33	25	23	1	31	11	26	1
Solyc07g065320.2.1	8	60	7	40	0	83	25	73	0	8	5	10	0
Solyc07g065330.2.1	0	0	0	0	0	0	0	0	0	0	0	0	0
Solyc07g065340.1.1	2	6	2	7	0	154	17	32	5	6	23	10	0
Solyc07g065370.2.1	12	142	3	77	11	304	56	196	12	242	26	233	7
Solyc07g065380.2.1	12	318	41	276	10	444	56	381	20	497	72	462	1
Solyc07g065400.2.1	0	0	0	0	0	0	0	0	0	0	0	0	0
Solyc07g065410.1.1	1	11	0	1	0	28	0	2	1	6	6	7	0
Solyc07g065420.1.1	4	3	2	0	0	13	48	10	1	2	4	7	0
Solyc07g065430.2.1	0	0	0	0	0	0	0	0	0	0	0	0	0
Solyc07g065450.2.1	1	1	2	1	0	0	6	9	2	4	0	5	0
Solyc07g065460.1.1	2	39	2	17	1	18	26	48	1	38	33	22	0
Solyc07g065470.2.1	2	1	1	0	0	2	8	3	0	12	3	11	0
Solyc07g065480.2.1	1	5	0	1	0	7	8	9	0	50	1	41	0
Solyc07g065490.2.1	12	251	18	232	13	412	138	587	47	366	84	361	2
Solyc07g065500.1.1	10	258	21	101	4	50	80	89	12	135	11	88	7
Solyc07g065510.2.1	1	17	1	18	0	17	4	27	0	22	1	19	0
Solyc07g065520.2.1	1	29	1	14	2	37	7	30	2	54	2	15	0
Solyc07g065530.1.1	0	0	0	0	0	0	0	0	0	0	0	0	0
Solyc07g065540.1.1	5	0	0	0	0	0	0	0	1	138	43	151	1
Solyc07g065550.2.1	0	0	0	0	0	0	0	0	0	0	0	0	0
Solyc07g065570.1.1	0	0	0	0	0	0	0	0	0	0	0	0	0
Solyc07g065590.2.1	3	3	2	6	0	46	57	40	5	28	2	13	1
Solyc07g065600.2.1	0	0	0	0	0	0	0	0	0	0	0	0	0
Solyc07g065610.1.1	0	0	0	0	0	0	0	0	0	0	0	0	0
Solyc07g065620.2.1	8	22	1	6	0	81	32	85	7	57	5	73	0
Solyc07g065630.2.1	68	410	59	279	27	1415	938	1610	104	628	229	560	16

Solyc07g065640.2.1	2	23	2	10	2	24	19	23	2	33	4	31	0
Solyc07g065650.2.1	4	41	3	20	1	166	35	131	8	54	7	44	0
Solyc07g065660.2.1	4	8	7	5	3	35	11	17	0	6	8	5	3
Solyc07g065670.1.1	0	0	0	0	0	0	0	0	0	0	0	0	0
Solyc07g065680.2.1	22	61	13	39	14	169	164	201	35	174	50	114	2
Solyc07g065690.2.1	1	5	2	0	0	6	5	4	0	12	0	6	0
Solyc07g065700.2.1	4	71	4	54	14	201	43	161	3	113	19	109	2
Solyc07g065710.2.1	11	131	23	154	8	140	103	248	39	201	33	132	0
Solyc07g065720.2.1	1	3	1	1	0	5	1	5	1	1	3	4	1
Solyc07g065730.1.1	0	0	0	0	0	0	0	0	0	0	0	0	0
Solyc07g065740.2.1	0	0	0	0	0	0	0	0	0	0	0	0	0
Solyc07g065760.1.1	2	3	0	1	0	4	0	8	0	4	6	3	0
Solyc07g065790.2.1	5	35	4	15	6	54	21	36	11	53	17	22	0
Solyc07g065800.2.1	6	35	1	21	1	136	22	103	3	82	2	82	0
Solyc07g065810.2.1	0	0	0	0	0	0	0	0	0	0	0	0	0
Solyc07g065820.2.1	3	44	5	24	0	37	79	40	7	26	13	20	0
Solyc07g065840.2.1	3	2813	138	3807	48	3495	586	4320	80	26016	542	22629	35
Solyc07g065850.2.1	1	6	0	4	0	14	0	8	0	3	6	5	0
Solyc07g065860.2.1	3	2	0	7	0	15	5	12	0	2	4	6	0
Solyc07g065870.2.1	0	0	0	0	0	0	0	0	0	0	0	0	0
Solyc07g065880.2.1	0	0	0	0	0	0	0	0	0	0	0	0	0
Solyc07g065890.2.1	2	12	0	2	0	11	6	25	0	18	3	22	1
Solyc07g065900.2.1	10	279	107	258	3	6	132	3	0	2	9	5	1
Solyc07g065910.1.1	0	0	0	0	0	0	0	0	0	0	0	0	0
Solyc07g065920.2.1	5	12	2	1	4	18	17	5	2	127	5	70	2
Solyc07g065930.2.1	9	29	3	8	0	154	79	167	44	27	17	9	0
Solyc07g065940.1.1	0	0	0	0	0	0	0	0	0	0	0	0	0
Solyc07g065950.2.1	22	193	43	135	20	428	345	750	45	528	85	322	9
Solyc07g065960.1.1	2	9	2	0	0	9	6	10	11	9	5	1	1
Solyc07g065970.1.1	7	95	12	30	9	83	68	47	8	15	11	2	1
Solyc07g065980.2.1	28	190	29	86	5	1424	1140	1566	36	211	138	154	23
Solyc07g065990.1.1	4	2	0	0	0	40	31	33	1	15	12	1	0
Solyc07g066010.2.1	6	89	2	68	0	29	29	62	0	26	6	20	0
Solyc07g066020.2.1	3	52	3	43	0	16	16	53	2	34	1	9	0
Solyc07g066030.2.1	14	116	14	82	24	413	139	293	89	138	46	98	36
Solyc07g066040.1.1	0	0	0	0	0	0	0	0	0	0	0	0	0
Solyc07g066050.2.1	0	0	0	0	0	0	0	0	0	0	0	0	0
Solyc07g066060.2.1	4	16	1	12	0	42	24	35	13	17	3	26	0
Solyc07g066070.2.1	6	124	11	74	11	92	185	259	36	270	20	148	7
Solyc07g066080.2.1	1	1	0	5	0	34	4	26	1	0	0	4	0
Solyc07g066090.2.1	8	14	1	25	0	78	32	82	5	44	32	56	0

Solyc07g066100.2.1	3	53	3	38	0	36	13	27	4	36	12	25	0
Solyc07g066110.1.1	0	0	0	0	0	0	0	0	0	0	0	0	0
Solyc07g066120.2.1	6	39	3	18	1	36	47	96	5	48	19	41	0
Solyc07g066150.1.1	5	3213	353	1356	14	43	346	87	13	284	238	356	29
Solyc07g066160.1.1	0	0	0	0	0	0	0	0	0	0	0	0	0
Solyc07g066170.2.1	10	42	3	14	4	101	44	65	13	52	21	47	1
Solyc07g066180.2.1	0	0	0	0	0	0	0	0	0	0	0	0	0
Solyc07g066190.1.1	0	0	0	0	0	0	0	0	0	0	0	0	0
Solyc07g066200.2.1	22	125	8	97	7	196	74	283	11	126	49	167	1
Solyc07g066210.2.1	5	10	9	6	1	30	19	35	1	25	4	31	2
Solyc07g066220.2.1	16	141	41	192	18	243	299	407	35	260	85	232	3
Solyc07g066230.2.1	1	1	0	7	0	14	0	3	0	18	4	8	0
Solyc07g066240.2.1	2	5	0	8	0	12	7	22	1	3	0	4	1
Solyc07g066260.2.1	10	112	12	74	10	886	69	221	28	60	33	58	1
Solyc07g066270.2.1	4	29	2	29	0	79	65	97	1	43	4	36	0
Solyc07g066280.2.1	2	5	0	5	0	9	11	15	0	10	1	8	0
Solyc07g066290.2.1	2	3	0	3	0	7	9	7	0	6	4	5	0
Solyc07g066300.2.1	4	9	3	6	0	4	23	21	1	12	3	10	0
Solyc07g066310.2.1	5	10216	787	6807	67	586	4711	732	39	1569	919	1899	38
Solyc07g066320.2.1	2	10	1	16	0	36	30	40	2	14	4	16	0
Solyc07g066330.2.1	3	2	0	1	0	33	9	28	0	1	0	0	0
Solyc07g066340.2.1	1	0	1	0	0	8	17	4	0	0	3	12	0
Solyc07g066350.1.1	0	0	0	0	0	0	0	0	0	0	0	0	0
Solyc07g066360.1.1	1	1	0	0	2	17	23	2	8	1	5	1	0
Solyc07g066370.2.1	3	10	0	0	0	9	6	9	0	65	8	44	0
Solyc07g066380.2.1	0	0	0	0	0	0	0	0	0	0	0	0	0
Solyc07g066390.1.1	2	8	0	3	0	10	28	3	0	4	0	12	0
Solyc07g066400.1.1	0	0	0	0	0	0	0	0	0	0	0	0	0
Solyc07g066410.2.1	8	15	3	7	0	60	30	83	10	37	13	27	0
Solyc07g066420.2.1	11	45	3	18	7	107	76	97	28	133	36	88	4
Solyc07g066430.2.1	3	70	0	51	0	72	16	50	0	280	2	277	0
Solyc07g066440.2.1	5	29	4	19	2	66	38	101	1	60	10	59	0
Solyc07g066450.2.1	4	10	2	25	0	20	20	23	3	14	22	18	6
Solyc07g066460.2.1	2	23	1	11	0	40	59	47	5	29	2	16	1
Solyc07g066470.2.1	6	154	15	115	0	221	243	373	28	271	54	250	3
Solyc07g066480.2.1	9	27	7	15	2	74	60	58	10	21	9	19	3
Solyc07g066520.2.1	3	20	4	7	0	75	27	65	2	26	3	15	0
Solyc07g066530.2.1	0	0	0	0	0	0	0	0	0	0	0	0	0
Solyc07g066540.1.1	0	0	0	0	0	0	0	0	0	0	0	0	0
Solyc07g066550.2.1	0	0	0	0	0	0	0	0	0	0	0	0	0
Solyc07g066560.1.1	1	45	11	40	1	16	7	14	4	9	1	2	0

Solyc07g066570.2.1	3	23	0	6	5	52	41	15	29	15	6	4	9
Solyc07g066580.2.1	5	111	20	57	18	197	161	294	35	199	43	151	6
Solyc07g066600.2.1	3	412	46	129	14	416	247	794	89	736	121	799	5
Solyc07g066610.2.1	3	1033	165	685	12	151	98	276	13	280	62	263	1
Solyc07g066620.2.1	3	21	2	9	0	1	16	12	0	27	3	33	0
Solyc07g066630.2.1	3	44	3	8	1	32	18	41	0	23	4	32	0
Solyc07g066640.2.1	0	0	0	0	0	0	0	0	0	0	0	0	0
Solyc07g066650.2.1	2	26	3	34	1	58	28	47	4	93	19	47	0
Solyc07g066660.2.1	3	12	0	5	0	11	22	9	2	12	2	17	1
Solyc07g066670.2.1	5	20	5	18	0	45	53	94	5	61	22	102	2
Solyc08g005000.2.1	0	0	0	0	0	0	0	0	0	0	0	0	0
Solyc08g005010.2.1	8	344	34	250	5	86	69	98	8	65	37	40	9
Solyc08g005020.2.1	11	143	10	100	5	239	56	311	25	233	50	137	1
Solyc08g005030.1.1	2	8	0	14	0	10	7	11	8	20	0	25	1
Solyc08g005050.2.1	8	299	78	212	17	141	98	87	13	163	48	227	2
Solyc08g005060.2.1	2	19	0	6	4	26	15	23	0	44	3	15	0
Solyc08g005070.2.1	14	169	21	123	12	267	159	277	22	948	120	784	8
Solyc08g005090.1.1	1	4	0	0	0	0	6	14	2	3	0	3	3
Solyc08g005100.2.1	0	0	0	0	0	0	0	0	0	0	0	0	0
Solyc08g005120.2.1	5	7	0	3	0	73	21	71	9	2	0	1	0
Solyc08g005130.1.1	0	0	0	0	0	0	0	0	0	0	0	0	0
Solyc08g005140.2.1	5	10	3	3	4	25	32	22	2	30	24	25	4
Solyc08g005150.2.1	2	2	0	0	0	8	8	11	0	5	0	10	0
Solyc08g005160.1.1	0	0	0	0	0	0	0	0	0	0	0	0	0
Solyc08g005170.2.1	5	25	4	24	4	78	57	89	11	53	15	27	2
Solyc08g005190.2.1	9	36	8	32	6	78	81	134	15	71	27	29	0
Solyc08g005200.2.1	6	23	2	24	6	36	65	43	11	40	14	55	0
Solyc08g005210.2.1	1	3	0	0	0	2	6	5	0	0	1	6	0
Solyc08g005220.2.1	8	62	12	37	0	28	21	43	0	21	6	17	0
Solyc08g005230.2.1	6	282	43	131	23	283	240	413	74	692	106	515	5
Solyc08g005240.1.1	0	0	0	0	0	0	0	0	0	0	0	0	0
Solyc08g005250.2.1	0	0	0	0	0	0	0	0	0	0	0	0	0
Solyc08g005260.1.1	0	0	0	0	0	0	0	0	0	0	0	0	0
Solyc08g005270.2.1	21	259	44	217	30	703	484	670	69	596	159	459	7
Solyc08g005280.1.1	0	0	0	0	0	0	0	0	0	0	0	0	0
Solyc08g005290.2.1	1	5	0	2	0	1	4	7	0	2	0	6	0
Solyc08g005300.1.1	15	62	0	52	7	107	50	126	23	59	18	41	0
Solyc08g005310.2.1	2	7	0	10	0	30	30	18	6	44	8	19	4
Solyc08g005320.2.1	1	0	0	0	0	4	0	0	5	0	3	0	0
Solyc08g005330.1.1	0	0	0	0	0	0	0	0	0	0	0	0	0
Solyc08g005350.2.1	0	0	0	0	0	0	0	0	0	0	0	0	0

Solyc08g005390.1.1	2	4	0	0	0	46	4	25	0	13	0	14	0
Solyc08g005400.2.1	0	0	0	0	0	0	0	0	0	0	0	0	0
Solyc08g005420.2.1	1	1	1	0	0	0	3	0	1	8	2	6	4
Solyc08g005430.2.1	6	14	3	6	7	18	27	35	1	27	5	25	1
Solyc08g005440.2.1	2	8	1	16	0	9	14	11	0	0	0	1	0
Solyc08g005450.1.1	7	257	6	257	6	249	87	272	24	1106	24	484	1
Solyc08g005460.2.1	0	0	0	0	0	0	0	0	0	0	0	0	0
Solyc08g005470.2.1	6	57	13	47	4	112	96	149	18	143	26	146	0
Solyc08g005480.2.1	1	7	3	3	0	44	8	62	5	24	3	15	0
Solyc08g005490.2.1	13	402	68	243	44	102	223	257	110	1191	109	1179	19
Solyc08g005510.1.1	0	0	0	0	0	0	0	0	0	0	0	0	0
Solyc08g005520.2.1	1	5	0	0	0	4	3	1	0	12	6	0	3
Solyc08g005530.2.1	0	0	0	0	0	0	0	0	0	0	0	0	0
Solyc08g005540.1.1	0	0	0	0	0	0	0	0	0	0	0	0	0
Solyc08g005560.2.1	15	45	9	49	0	48	103	134	0	395	24	398	1
Solyc08g005570.2.1	4	12	0	1	0	13	8	19	0	22	5	27	0
Solyc08g005580.2.1	0	0	0	0	0	0	0	0	0	0	0	0	0
Solyc08g005590.2.1	3	32	0	28	0	108	7	64	2	37	12	38	0
Solyc08g005610.2.1	1	67	9	17	8	0	4	0	7	0	0	0	0
Solyc08g005630.2.1	0	0	0	0	0	0	0	0	0	0	0	0	0
Solyc08g005640.2.1	0	0	0	0	0	0	0	0	0	0	0	0	0
Solyc08g005650.2.1	0	0	0	0	0	0	0	0	0	0	0	0	0
Solyc08g005670.2.1	0	0	0	0	0	0	0	0	0	0	0	0	0
Solyc08g005680.2.1	0	0	0	0	0	0	0	0	0	0	0	0	0
Solyc08g005690.1.1	0	0	0	0	0	0	0	0	0	0	0	0	0
Solyc08g005710.2.1	0	0	0	0	0	0	0	0	0	0	0	0	0
Solyc08g005720.2.1	0	0	0	0	0	0	0	0	0	0	0	0	0
Solyc08g005770.2.1	0	0	0	0	0	0	0	0	0	0	0	0	0
Solyc08g005780.2.1	4	14	0	4	2	2	20	24	5	20	4	13	0
Solyc08g005800.2.1	7	500	14	223	17	117	101	295	29	1026	21	1050	0
Solyc08g005860.2.1	13	49	2	31	0	189	155	135	5	31	5	26	0
Solyc08g005880.2.1	0	0	0	0	0	0	0	0	0	0	0	0	0
Solyc08g005900.2.1	2	7	0	5	0	19	8	15	8	7	3	6	0
Solyc08g005910.2.1	6	139	4	64	3	366	69	281	28	365	25	280	1
Solyc08g005920.2.1	0	0	0	0	0	0	0	0	0	0	0	0	0
Solyc08g005930.1.1	2	7	3	0	6	0	11	1	0	0	1	5	1
Solyc08g005940.1.1	0	0	0	0	0	0	0	0	0	0	0	0	0
Solyc08g005950.2.1	0	0	0	0	0	0	0	0	0	0	0	0	0
Solyc08g005960.1.1	3	439	25	72	3	7	244	34	0	302	55	35	3
Solyc08g005970.2.1	4	5	0	11	0	46	15	48	6	111	28	139	3
Solyc08g005980.2.1	4	130	7	126	0	39	12	92	2	162	1	75	0

Solyc08g005990.2.1	2	7	0	5	2	6	10	3	0	10	5	13	0
Solyc08g006000.2.1	2	120	0	93	0	253	31	167	13	200	24	184	1
Solyc08g006010.2.1	4	6	1	5	0	18	18	15	8	10	1	5	0
Solyc08g006030.2.1	3	24	2	12	0	19	12	18	2	24	4	5	0
Solyc08g006040.2.1	7	206	18	104	19	413	176	459	57	368	134	362	16
Solyc08g006050.1.1	2	2	0	0	0	5	3	4	1	0	1	4	0
Solyc08g006060.2.1	3	26	3	14	0	47	9	76	2	94	13	62	0
Solyc08g006070.2.1	1	11	0	0	3	7	1	4	0	20	3	8	0
Solyc08g006080.1.1	4	6	0	10	0	3	8	14	1	75	7	17	0
Solyc08g006090.2.1	3	24	0	8	0	36	44	67	4	30	14	23	1
Solyc08g006110.2.1	10	93	4	52	3	203	53	289	2	289	44	103	9
Solyc08g006120.2.1	0	0	0	0	0	0	0	0	0	0	0	0	0
Solyc08g006150.2.1	10	67	8	35	7	116	81	88	17	255	75	124	4
Solyc08g006160.2.1	9	377	57	225	3	99	65	63	7	69	61	124	0
Solyc08g006170.1.1	0	0	0	0	0	0	0	0	0	0	0	0	0
Solyc08g006180.2.1	0	0	0	0	0	0	0	0	0	0	0	0	0
Solyc08g006210.1.1	0	0	0	0	0	0	0	0	0	0	0	0	0
Solyc08g006220.2.1	0	0	0	0	0	0	0	0	0	0	0	0	0
Solyc08g006230.2.1	0	0	0	0	0	0	0	0	0	0	0	0	0
Solyc08g006250.1.1	5	25	2	6	0	13	9	13	1	34	5	18	2
Solyc08g006260.1.1	0	0	0	0	0	0	0	0	0	0	0	0	0
Solyc08g006270.1.1	0	0	0	0	0	0	0	0	0	0	0	0	0
Solyc08g006290.1.1	0	0	0	0	0	0	0	0	0	0	0	0	0
Solyc08g006310.2.1	8	39	3	45	28	15	12	14	3	92	35	41	0
Solyc08g006320.2.1	9	390	105	409	10	344	37	200	11	109	34	273	9
Solyc08g006330.2.1	5	11	5	9	0	208	35	124	0	7	16	12	0
Solyc08g006350.2.1	1	8	0	1	0	0	5	7	0	7	0	6	0
Solyc08g006360.1.1	0	0	0	0	0	0	0	0	0	0	0	0	0
Solyc08g006370.1.1	0	0	0	0	0	0	0	0	0	0	0	0	0
Solyc08g006380.2.1	0	0	0	0	0	0	0	0	0	0	0	0	0
Solyc08g006410.2.1	0	0	0	0	0	0	0	0	0	0	0	0	0
Solyc08g006420.2.1	22	114	16	59	3	200	122	257	27	133	46	167	0
Solyc08g006430.2.1	24	313	18	241	1	164	408	167	20	220	147	149	7
Solyc08g006440.2.1	0	0	0	0	0	0	0	0	0	0	0	0	0
Solyc08g006460.2.1	16	129	23	74	2	275	181	335	21	186	64	97	1
Solyc08g006470.2.1	2	0	0	3	0	2	1	5	0	7	0	0	0
Solyc08g006480.2.1	4	40	9	36	3	120	50	75	8	54	6	27	0
Solyc08g006500.2.1	1	2	0	2	0	0	6	5	0	4	0	0	0
Solyc08g006510.2.1	5	20	0	14	3	78	16	90	9	45	3	32	0
Solyc08g006520.1.1	0	0	0	0	0	0	0	0	0	0	0	0	0
Solyc08g006530.2.1	8	331	51	343	29	167	131	269	26	67	62	83	13

Solyc08g006540.2.1	6	119	31	73	0	8	56	19	3	26	6	41	1
Solyc08g006550.2.1	0	0	0	0	0	0	0	0	0	0	0	0	0
Solyc08g006560.2.1	1	3	3	0	11	0	4	1	10	0	0	0	0
Solyc08g006570.2.1	0	0	0	0	0	0	0	0	0	0	0	0	0
Solyc08g006580.2.1	0	0	0	0	0	0	0	0	0	0	0	0	0
Solyc08g006590.1.1	2	45	2	10	0	15	17	11	0	9	0	10	0
Solyc08g006600.2.1	3	10	9	31	0	23	14	23	2	17	5	30	0
Solyc08g006610.2.1	0	0	0	0	0	0	0	0	0	0	0	0	0
Solyc08g006620.2.1	2	16	0	13	0	27	12	29	2	13	1	12	0
Solyc08g006630.2.1	0	0	0	0	0	0	0	0	0	0	0	0	0
Solyc08g006640.2.1	9	147	6	81	0	46	50	52	5	170	21	165	3
Solyc08g006650.2.1	2	20	1	19	4	17	68	34	10	27	40	58	1
Solyc08g006660.1.1	6	32	4	7	0	44	19	41	10	45	13	16	1
Solyc08g006670.2.1	5	31	9	9	1	35	28	41	4	63	10	24	1
Solyc08g006680.2.1	1	13	2	4	2	3	4	18	0	2	0	10	0
Solyc08g006690.1.1	0	0	0	0	0	0	0	0	0	0	0	0	0
Solyc08g006700.2.1	0	0	0	0	0	0	0	0	0	0	0	0	0
Solyc08g006710.2.1	0	0	0	0	0	0	0	0	0	0	0	0	0
Solyc08g006720.2.1	5	335	33	124	6	126	113	161	11	181	21	182	6
Solyc08g006730.1.1	8	7	1	6	2	218	241	162	398	0	11	0	0
Solyc08g006740.2.1	2	2	0	0	0	11	16	25	20	0	0	0	0
Solyc08g006750.2.1	3	1	0	0	0	22	14	22	7	0	0	0	0
Solyc08g006760.2.1	1	9	1	9	0	1	7	0	0	6	1	4	0
Solyc08g006770.2.1	8	96	0	40	0	480	77	577	1	701	0	581	0
Solyc08g006780.2.1	10	297	20	104	8	409	349	665	11	195	85	159	1
Solyc08g006790.2.1	0	0	0	0	0	0	0	0	0	0	0	0	0
Solyc08g006800.1.1	0	0	0	0	0	0	0	0	0	0	0	0	0
Solyc08g006820.2.1	10	47	2	14	4	57	18	69	7	144	34	123	11
Solyc08g006830.2.1	0	0	0	0	0	0	0	0	0	0	0	0	0
Solyc08g006840.2.1	5	11	1	14	3	43	12	46	10	20	13	24	0
Solyc08g006870.2.1	7	46	10	20	3	36	34	58	7	178	9	143	2
Solyc08g006880.2.1	18	254	20	179	4	359	68	403	13	121	43	88	2
Solyc08g006890.2.1	7	2758	146	1681	148	1402	946	2845	91	9911	713	9974	157
Solyc08g006900.2.1	2	37	12	7	2	47	61	68	19	48	58	98	6
Solyc08g006910.2.1	0	0	0	0	0	0	0	0	0	0	0	0	0
Solyc08g006920.1.1	0	0	0	0	0	0	0	0	0	0	0	0	0
Solyc08g006930.2.1	4	5078	315	4606	3	36	288	240	5	190	131	376	2
Solyc08g006940.2.1	6	39	3	57	4	64	23	70	4	116	23	207	1
Solyc08g006950.2.1	3	12	0	8	1	30	1	26	0	27	18	28	1
Solyc08g006960.2.1	3	22	3	3	0	7	21	39	1	16	17	18	1
Solyc08g006970.2.1	4	8	4	3	2	6	42	22	18	19	3	11	0

Solyc08g006980.2.1	22	227	12	133	1	440	112	457	32	419	120	447	11
Solyc08g006990.1.1	0	0	0	0	0	0	0	0	0	0	0	0	0
Solyc08g007000.2.1	6	9	2	1	7	13	27	25	31	14	11	4	0
Solyc08g007010.2.1	8	125	8	70	4	160	71	222	5	35	11	51	0
Solyc08g007030.2.1	2	4	0	0	0	22	28	16	6	10	0	22	1
Solyc08g007040.2.1	2	320	32	143	2	0	90	5	0	8	6	28	0
Solyc08g007060.2.1	1	3	0	4	0	8	5	16	0	1	0	0	0
Solyc08g007070.2.1	0	0	0	0	0	0	0	0	0	0	0	0	0
Solyc08g007080.2.1	6	26	9	33	2	52	14	82	1	57	2	93	1
Solyc08g007090.1.1	0	0	0	0	0	0	0	0	0	0	0	0	0
Solyc08g007100.2.1	1	2	4	0	0	3	3	4	3	14	5	3	0
Solyc08g007110.2.1	17	103	13	101	10	434	235	509	32	304	54	266	6
Solyc08g007120.2.1	0	0	0	0	0	0	0	0	0	0	0	0	0
Solyc08g007130.2.1	6	41	3	19	2	17	45	17	3	11	2	4	2
Solyc08g007140.2.1	2	53	26	9	10	268	184	86	39	242	147	270	22
Solyc08g007150.1.1	0	0	0	0	0	0	0	0	0	0	0	0	0
Solyc08g007160.2.1	0	0	0	0	0	0	0	0	0	0	0	0	0
Solyc08g007170.1.1	7	56	4	27	3	104	35	128	18	113	16	72	3
Solyc08g007180.2.1	2	13	2	4	0	7	15	1	0	6	1	11	0
Solyc08g007190.2.1	7	17	2	11	0	12	48	37	17	21	7	26	0
Solyc08g007200.2.1	0	0	0	0	0	0	0	0	0	0	0	0	0
Solyc08g007210.2.1	10	33	3	1	44	80	126	17	173	20	8	6	2
Solyc08g007220.2.1	2	27	4	18	0	74	24	86	10	58	27	63	0
Solyc08g007240.2.1	5	78	4	46	0	14	19	81	0	588	0	427	0
Solyc08g007250.1.1	0	0	0	0	0	0	0	0	0	0	0	0	0
Solyc08g007260.1.1	0	0	0	0	0	0	0	0	0	0	0	0	0
Solyc08g007270.2.1	0	0	0	0	0	0	0	0	0	0	0	0	0
Solyc08g007280.2.1	0	0	0	0	0	0	0	0	0	0	0	0	0
Solyc08g007290.2.1	0	0	0	0	0	0	0	0	0	0	0	0	0
Solyc08g007300.1.1	0	0	0	0	0	0	0	0	0	0	0	0	0
Solyc08g007310.2.1	3	11	2	20	0	23	15	7	1	6	10	17	1
Solyc08g007320.2.1	3	23	2	1	0	30	37	28	4	30	9	33	0
Solyc08g007330.2.1	0	0	0	0	0	0	0	0	0	0	0	0	0
Solyc08g007340.2.1	17	127	20	87	8	176	178	176	66	92	58	156	4
Solyc08g007350.1.1	0	0	0	0	0	0	0	0	0	0	0	0	0
Solyc08g007360.2.1	22	91	12	84	14	239	124	305	14	142	37	122	2
Solyc08g007370.2.1	3	12	3	11	1	48	18	45	1	48	11	112	1
Solyc08g007380.2.1	0	0	0	0	0	0	0	0	0	0	0	0	0
Solyc08g007400.2.1	1	20	0	6	0	38	242	87	4	11	5	11	0
Solyc08g007410.2.1	0	0	0	0	0	0	0	0	0	0	0	0	0
Solyc08g007420.2.1	7	14	3	12	5	30	94	61	10	43	14	17	4

Solyc08g007430.1.1	0	0	0	0	0	0	0	0	0	0	0	0	0
Solyc08g007440.1.1	1	3	0	0	0	3	1	3	0	5	3	3	1
Solyc08g007460.2.1	5	101	9	14	4	238	90	103	47	9	16	1	0
Solyc08g007470.1.1	1	1	0	0	0	8	4	5	0	4	0	1	0
Solyc08g007480.1.1	0	0	0	0	0	0	0	0	0	0	0	0	0
Solyc08g007490.2.1	2	5	0	2	0	22	27	24	4	18	20	10	0
Solyc08g007500.2.1	1	3	0	15	0	0	35	7	1	1	5	3	0
Solyc08g007510.2.1	1	9	2	7	0	4	1	6	1	27	8	33	0
Solyc08g007520.2.1	5	4	3	7	0	9	34	12	4	26	10	14	0
Solyc08g007530.2.1	2	1	0	2	0	1	23	4	0	7	14	29	0
Solyc08g007540.2.1	1	3	0	3	0	1	5	0	0	4	0	5	0
Solyc08g007560.1.1	0	0	0	0	0	0	0	0	0	0	0	0	0
Solyc08g007570.2.1	0	0	0	0	0	0	0	0	0	0	0	0	0
Solyc08g007580.2.1	0	0	0	0	0	0	0	0	0	0	0	0	0
Solyc08g007590.2.1	6	22	11	17	12	62	81	37	16	105	23	41	0
Solyc08g007630.1.1	0	0	0	0	0	0	0	0	0	0	0	0	0
Solyc08g007640.1.1	1	0	0	0	0	3	3	3	0	3	0	2	0
Solyc08g007650.1.1	0	0	0	0	0	0	0	0	0	0	0	0	0
Solyc08g007660.2.1	0	0	0	0	0	0	0	0	0	0	0	0	0
Solyc08g007670.1.1	0	0	0	0	0	0	0	0	0	0	0	0	0
Solyc08g007680.1.1	0	0	0	0	0	0	0	0	0	0	0	0	0
Solyc08g007690.1.1	0	0	0	0	0	0	0	0	0	0	0	0	0
Solyc08g007710.2.1	0	0	0	0	0	0	0	0	0	0	0	0	0
Solyc08g007720.1.1	0	0	0	0	0	0	0	0	0	0	0	0	0
Solyc08g007730.2.1	1	1	0	2	0	12	3	3	0	6	0	0	0
Solyc08g007740.1.1	0	0	0	0	0	0	0	0	0	0	0	0	0
Solyc08g007750.2.1	2	11	2	1	0	11	6	16	1	22	6	21	0
Solyc08g007760.2.1	1	21	0	6	0	27	7	34	0	15	1	13	0
Solyc08g007770.2.1	5	50	6	72	6	24	114	66	12	101	19	130	2
Solyc08g007790.2.1	0	0	0	0	0	0	0	0	0	0	0	0	0
Solyc08g007800.2.1	2	10	0	5	2	18	0	25	1	25	10	5	0
Solyc08g007820.1.1	3	44	11	15	2	24	6	0	1	2	0	0	0
Solyc08g007830.1.1	0	0	0	0	0	0	0	0	0	0	0	0	0
Solyc08g007840.1.1	0	0	0	0	0	0	0	0	0	0	0	0	0
Solyc08g007860.2.1	8	55	9	15	7	83	156	85	22	88	48	49	1
Solyc08g007910.2.1	3	9	0	4	0	36	8	16	1	8	1	7	0
Solyc08g007920.1.1	0	0	0	0	0	0	0	0	0	0	0	0	0
Solyc08g007950.2.1	12	100	18	87	1	85	201	140	12	70	28	55	9
Solyc08g007970.2.1	4	29	3	31	1	17	9	24	1	37	8	10	4
Solyc08g007980.2.1	0	0	0	0	0	0	0	0	0	0	0	0	0
Solyc08g007990.2.1	8	84	7	49	17	142	86	172	21	520	61	345	2

Solyc08g008000.1.1	0	0	0	0	0	0	0	0	0	0	0	0	0
Solyc08g008010.2.1	1	1	0	1	0	5	8	8	0	2	0	3	0
Solyc08g008020.1.1	0	0	0	0	0	0	0	0	0	0	0	0	0
Solyc08g008030.2.1	9	36	11	11	1	188	34	105	7	44	10	39	3
Solyc08g008040.2.1	3	32	4	17	1	22	10	43	4	29	16	30	10
Solyc08g008050.2.1	0	0	0	0	0	0	0	0	0	0	0	0	0
Solyc08g008070.1.1	3	17	3	5	4	18	8	9	5	6	4	6	0
Solyc08g008080.1.1	0	0	0	0	0	0	0	0	0	0	0	0	0
Solyc08g008100.2.1	0	0	0	0	0	0	0	0	0	0	0	0	0
Solyc08g008110.2.1	0	0	0	0	0	0	0	0	0	0	0	0	0
Solyc08g008120.2.1	0	0	0	0	0	0	0	0	0	0	0	0	0
Solyc08g008130.2.1	6	43	6	23	1	52	36	45	18	92	14	47	0
Solyc08g008140.2.1	0	0	0	0	0	0	0	0	0	0	0	0	0
Solyc08g008150.1.1	0	0	0	0	0	0	0	0	0	0	0	0	0
Solyc08g008160.2.1	3	2	2	5	0	10	24	20	1	33	3	23	0
Solyc08g008170.2.1	4	29	4	19	5	32	31	47	5	78	18	39	2
Solyc08g008180.1.1	12	46	5	24	0	117	66	213	23	87	13	72	4
Solyc08g008200.1.1	0	0	0	0	0	0	0	0	0	0	0	0	0
Solyc08g008210.2.1	7	148	15	87	5	112	79	153	14	623	160	520	6
Solyc08g008220.2.1	4	272	12	96	4	351	81	450	12	826	51	548	17
Solyc08g008230.2.1	4	118	1	75	0	98	17	115	3	1323	13	885	3
Solyc08g008240.2.1	0	0	0	0	0	0	0	0	0	0	0	0	0
Solyc08g008250.1.1	3	4	4	7	0	18	8	7	1	16	2	22	0
Solyc08g008270.2.1	1	28	0	14	0	6	7	28	0	20	0	4	0
Solyc08g008280.2.1	4	125	32	93	5	7	1	9	1	6	0	42	3
Solyc08g008290.2.1	9	133	32	108	21	333	403	516	82	666	113	826	11
Solyc08g008310.2.1	0	0	0	0	0	0	0	0	0	0	0	0	0
Solyc08g008320.2.1	0	0	0	0	0	0	0	0	0	0	0	0	0
Solyc08g008330.2.1	1	14	1	11	9	12	2	18	1	7	13	10	0
Solyc08g008340.2.1	3	12	0	10	0	67	15	31	0	18	0	12	0
Solyc08g008350.2.1	3	79	5	14	0	72	71	96	5	100	4	74	3
Solyc08g008370.2.1	9	843	88	433	55	192	26	157	42	157	21	268	10
Solyc08g008380.2.1	2	8	0	5	0	3	4	6	1	7	2	15	0
Solyc08g008410.2.1	3	3	4	11	4	52	17	48	3	7	0	5	3
Solyc08g008420.2.1	1	9	0	9	1	7	7	34	1	0	0	0	0
Solyc08g008430.2.1	0	0	0	0	0	0	0	0	0	0	0	0	0
Solyc08g008440.1.1	0	0	0	0	0	0	0	0	0	0	0	0	0
Solyc08g008460.2.1	4	23	1	8	0	15	34	25	5	75	5	25	4
Solyc08g008470.1.1	1	12	0	6	0	10	10	12	1	54	5	51	0
Solyc08g008480.2.1	0	0	0	0	0	0	0	0	0	0	0	0	0
Solyc08g008490.2.1	9	50	3	29	2	115	27	109	16	41	17	48	0

Solyc08g008500.2.1	0	0	0	0	0	0	0	0	0	0	0	0	0
Solyc08g008510.2.1	7	36	6	43	6	88	60	93	29	45	20	28	6
Solyc08g008520.2.1	1	9	2	0	0	6	0	4	1	15	2	9	0
Solyc08g008530.1.1	7	42	3	17	1	24	35	50	5	68	26	40	0
Solyc08g008550.2.1	10	63	7	40	5	66	79	100	14	148	32	27	0
Solyc08g008580.2.1	0	0	0	0	0	0	0	0	0	0	0	0	0
Solyc08g008590.2.1	0	0	0	0	0	0	0	0	0	0	0	0	0
Solyc08g008610.2.1	1	10	0	2	3	3	9	2	9	0	4	0	1
Solyc08g008630.2.1	7	223	13	109	1	7	8	30	0	49	16	58	0
Solyc08g008660.2.1	0	0	0	0	0	0	0	0	0	0	0	0	0
Solyc08g008670.2.1	0	0	0	0	0	0	0	0	0	0	0	0	0
Solyc08g013670.2.1	5	2223	164	766	0	2	46	30	2	71	126	108	10
Solyc08g013690.1.1	0	0	0	0	0	0	0	0	0	0	0	0	0
Solyc08g013700.1.1	0	0	0	0	0	0	0	0	0	0	0	0	0
Solyc08g013710.2.1	3	59	8	36	3	258	85	72	11	148	19	41	1
Solyc08g013720.2.1	2	14	2	4	0	4	16	1	0	24	0	30	0
Solyc08g013730.2.1	2	63	33	63	0	149	122	92	4	52	16	24	0
Solyc08g013740.2.1	0	0	0	0	0	0	0	0	0	0	0	0	0
Solyc08g013750.2.1	0	0	0	0	0	0	0	0	0	0	0	0	0
Solyc08g013820.2.1	1	0	1	1	0	1	4	0	0	4	0	5	0
Solyc08g013840.2.1	1	5	4	0	0	11	3	5	1	2	0	0	0
Solyc08g013850.1.1	0	0	0	0	0	0	0	0	0	0	0	0	0
Solyc08g013860.2.1	11	80	10	34	3	266	126	327	35	276	35	165	5
Solyc08g013880.2.1	0	0	0	0	0	0	0	0	0	0	0	0	0
Solyc08g013890.2.1	0	0	0	0	0	0	0	0	0	0	0	0	0
Solyc08g013900.2.1	5	23	3	42	3	64	19	82	8	30	12	22	2
Solyc08g013940.2.1	0	0	0	0	0	0	0	0	0	0	0	0	0
Solyc08g013960.1.1	0	0	0	0	0	0	0	0	0	0	0	0	0
Solyc08g013970.1.1	0	0	0	0	0	0	0	0	0	0	0	0	0
Solyc08g013980.2.1	0	0	0	0	0	0	0	0	0	0	0	0	0
Solyc08g013990.2.1	5	87	6	48	3	93	119	117	17	366	39	202	3
Solyc08g014000.2.1	13	9	3	12	3	18	127	11	6	1361	559	2994	2
Solyc08g014010.2.1	0	0	0	0	0	0	0	0	0	0	0	0	0
Solyc08g014020.2.1	2	45	7	36	4	20	69	76	9	41	16	31	0
Solyc08g014030.1.1	0	0	0	0	0	0	0	0	0	0	0	0	0
Solyc08g014040.2.1	1	2	1	0	0	0	12	5	0	4	2	7	0
Solyc08g014050.1.1	0	0	0	0	0	0	0	0	0	0	0	0	0
Solyc08g014060.2.1	7	30	13	16	13	28	93	57	5	58	58	92	16
Solyc08g014080.2.1	4	14	6	0	6	24	75	18	1	34	16	75	1
Solyc08g014100.1.1	1	1	0	2	0	5	5	1	0	0	0	5	0
Solyc08g014120.2.1	1	36	7	27	1	15	14	31	6	55	10	17	0

Solyc08g014130.2.1	15	61	7	42	16	78	58	80	15	218	557	158	1
Solyc08g014150.2.1	2	5	0	13	2	8	20	11	4	3	0	2	0
Solyc08g014190.2.1	0	0	0	0	0	0	0	0	0	0	0	0	0
Solyc08g014230.2.1	0	0	0	0	0	0	0	0	0	0	0	0	0
Solyc08g014280.2.1	4	21	2	12	0	44	18	50	1	3	1	1	0
Solyc08g014290.1.1	4	29	6	16	0	56	9	27	9	45	11	20	3
Solyc08g014300.2.1	0	0	0	0	0	0	0	0	0	0	0	0	0
Solyc08g014330.2.1	0	0	0	0	0	0	0	0	0	0	0	0	0
Solyc08g014340.2.1	8	364	21	286	9	187	159	317	28	562	59	318	2
Solyc08g014350.1.1	0	0	0	0	0	0	0	0	0	0	0	0	0
Solyc08g014360.1.1	0	0	0	0	0	0	0	0	0	0	0	0	0
Solyc08g014370.1.1	1	5	0	0	0	3	1	20	0	27	3	29	0
Solyc08g014380.1.1	0	0	0	0	0	0	0	0	0	0	0	0	0
Solyc08g014420.2.1	6	66	6	43	1	50	38	94	8	116	21	79	2
Solyc08g014430.2.1	0	0	0	0	0	0	0	0	0	0	0	0	0
Solyc08g014440.2.1	4	7	0	0	0	7	14	9	0	2	4	11	0
Solyc08g014450.2.1	3	29	0	4	0	6	8	28	3	18	12	17	0
Solyc08g014470.2.1	10	200	2	256	7	190	81	288	44	419	22	533	5
Solyc08g014480.2.1	22	128	16	107	16	174	180	337	21	232	37	211	2
Solyc08g014490.1.1	0	0	0	0	0	0	0	0	0	0	0	0	0
Solyc08g014510.2.1	4	20	9	5	0	52	54	35	5	50	9	5	0
Solyc08g014550.2.1	1	16	0	0	0	40	3	18	1	34	3	40	0
Solyc08g014570.2.1	0	0	0	0	0	0	0	0	0	0	0	0	0
Solyc08g014580.2.1	0	0	0	0	0	0	0	0	0	0	0	0	0
Solyc08g014600.2.1	2	2	0	3	4	1	9	5	0	9	5	3	1
Solyc08g014610.2.1	0	0	0	0	0	0	0	0	0	0	0	0	0
Solyc08g015620.2.1	3	88	15	19	2	124	71	62	3	20	11	40	0
Solyc08g015630.2.1	0	0	0	0	0	0	0	0	0	0	0	0	0
Solyc08g015650.2.1	0	0	0	0	0	0	0	0	0	0	0	0	0
Solyc08g015660.2.1	5	35	3	18	2	13	29	27	4	34	10	53	2
Solyc08g015690.2.1	8	279	58	198	19	736	2071	738	234	1214	551	931	44
Solyc08g015730.1.1	0	0	0	0	0	0	0	0	0	0	0	0	0
Solyc08g015770.2.1	0	0	0	0	0	0	0	0	0	0	0	0	0
Solyc08g015780.2.1	6	147	13	94	6	241	100	229	15	227	36	214	1
Solyc08g015820.1.1	0	0	0	0	0	0	0	0	0	0	0	0	0
Solyc08g015860.2.1	7	189	8	124	6	479	171	562	40	387	65	379	7
Solyc08g015870.2.1	5	146	15	138	9	136	122	194	36	99	30	78	4
Solyc08g015940.2.1	0	0	0	0	0	0	0	0	0	0	0	0	0
Solyc08g015960.2.1	6	45	6	31	0	9	37	41	2	70	27	55	2
Solyc08g015990.2.1	8	78	18	34	1	175	84	162	25	168	34	123	9
Solyc08g016010.2.1	7	34	7	34	4	64	17	68	2	84	15	80	0

Solyc08g016020.2.1	2	8	0	3	0	1	5	9	2	19	2	4	0
Solyc08g016050.2.1	9	58	12	46	6	100	68	140	9	173	21	138	2
Solyc08g016060.1.1	1	7	0	2	0	3	6	8	0	6	0	1	0
Solyc08g016070.1.1	3	5	0	4	0	6	14	20	2	12	6	3	0
Solyc08g016080.2.1	5	107	22	45	1	20	38	42	4	46	7	52	0
Solyc08g016090.2.1	8	17	4	29	2	39	32	54	6	19	11	28	0
Solyc08g016150.1.1	4	223	26	261	0	3	0	14	0	4	0	66	0
Solyc08g016160.2.1	0	0	0	0	0	0	0	0	0	0	0	0	0
Solyc08g016170.2.1	5	72	8	49	4	244	68	374	10	74	41	127	0
Solyc08g016180.2.1	5	128	10	65	2	187	41	245	16	222	66	144	6
Solyc08g016200.2.1	0	0	0	0	0	0	0	0	0	0	0	0	0
Solyc08g016210.2.1	0	0	0	0	0	0	0	0	0	0	0	0	0
Solyc08g016220.1.1	0	0	0	0	0	0	0	0	0	0	0	0	0
Solyc08g016270.1.1	0	0	0	0	0	0	0	0	0	0	0	0	0
Solyc08g016310.2.1	0	0	0	0	0	0	0	0	0	0	0	0	0
Solyc08g016410.1.1	2	9	1	1	0	15	18	15	1	51	19	25	0
Solyc08g016420.2.1	2	5	0	6	0	13	10	15	0	20	2	17	0
Solyc08g016440.2.1	0	0	0	0	0	0	0	0	0	0	0	0	0
Solyc08g016500.2.1	0	0	0	0	0	0	0	0	0	0	0	0	0
Solyc08g016510.2.1	0	0	0	0	0	0	0	0	0	0	0	0	0
Solyc08g016580.1.1	0	0	0	0	0	0	0	0	0	0	0	0	0
Solyc08g016600.1.1	0	0	0	0	0	0	0	0	0	0	0	0	0
Solyc08g016620.2.1	0	0	0	0	0	0	0	0	0	0	0	0	0
Solyc08g016670.2.1	6	38	5	12	0	150	19	136	4	99	31	88	6
Solyc08g016720.1.1	5	2	4	2	0	56	24	31	1	21	22	29	2
Solyc08g016750.2.1	5	232	14	112	8	199	101	245	2	121	48	107	1
Solyc08g021820.2.1	1	0	1	8	3	8	20	75	4	0	6	0	0
Solyc08g021890.2.1	3	4	0	1	0	17	25	35	2	19	3	2	2
Solyc08g021900.1.1	0	0	0	0	0	0	0	0	0	0	0	0	0
Solyc08g021910.1.1	24	59	21	115	8	315	240	422	25	231	42	190	4
Solyc08g021920.2.1	1	15	11	17	4	56	81	34	3	48	6	39	4
Solyc08g021960.2.1	5	28	1	9	0	16	14	18	2	22	4	20	0
Solyc08g022030.2.1	11	117	17	53	3	48	55	77	12	66	28	57	2
Solyc08g022060.1.1	0	0	0	0	0	0	0	0	0	0	0	0	0
Solyc08g022070.2.1	6	17	6	30	0	82	23	107	6	32	0	16	0
Solyc08g022080.2.1	1	14	1	8	1	20	26	34	3	40	10	48	1
Solyc08g022100.1.1	0	0	0	0	0	0	0	0	0	0	0	0	0
Solyc08g022110.2.1	0	0	0	0	0	0	0	0	0	0	0	0	0
Solyc08g022120.2.1	0	0	0	0	0	0	0	0	0	0	0	0	0
Solyc08g022210.2.1	11	406	14	239	7	664	115	691	5	677	37	743	8
Solyc08g022240.1.1	0	0	0	0	0	0	0	0	0	0	0	0	0

Solyc08g023270.2.1	0	0	0	0	0	0	0	0	0	0	0	0	0
Solyc08g023280.2.1	1	3	0	0	0	3	11	1	0	23	4	17	0
Solyc08g023440.2.1	12	74	6	41	29	596	790	1100	349	172	141	156	12
Solyc08g023460.2.1	3	6	1	4	0	11	30	23	6	12	3	10	0
Solyc08g023470.2.1	4	27	4	7	0	21	15	25	4	43	11	19	0
Solyc08g023490.2.1	1	1	0	0	0	3	0	0	0	1	0	6	0
Solyc08g023500.2.1	0	0	0	0	0	0	0	0	0	0	0	0	0
Solyc08g023570.1.1	1	0	0	0	0	0	5	5	0	8	0	1	0
Solyc08g023580.2.1	1	2	1	0	0	14	3	11	0	19	2	4	1
Solyc08g023590.2.1	1	4	1	0	0	5	2	7	1	5	0	0	0
Solyc08g023640.1.1	0	0	0	0	0	0	0	0	0	0	0	0	0
Solyc08g023650.2.1	0	0	0	0	0	0	0	0	0	0	0	0	0
Solyc08g028690.2.1	1	12	3	33	0	11	12	47	0	14	0	30	0
Solyc08g028780.1.1	0	0	0	0	0	0	0	0	0	0	0	0	0
Solyc08g029000.2.1	0	0	0	0	0	0	0	0	0	0	0	0	0
Solyc08g029050.2.1	0	0	0	0	0	0	0	0	0	0	0	0	0
Solyc08g029090.2.1	4	4	0	16	0	0	25	8	1	16	5	17	0
Solyc08g029110.2.1	7	31	5	30	8	106	69	108	17	60	15	52	1
Solyc08g029120.1.1	3	1	2	8	0	5	8	7	0	14	7	12	0
Solyc08g029130.2.1	10	60	6	38	2	105	93	168	12	139	48	116	3
Solyc08g029150.1.1	2	5	3	6	1	8	5	7	0	2	0	3	0
Solyc08g029160.1.1	3	79	6	35	2	88	93	95	10	312	78	304	12
Solyc08g029170.2.1	7	21	5	16	0	119	26	82	3	60	9	79	1
Solyc08g029190.2.1	2	10	0	5	0	12	8	10	1	15	5	26	1
Solyc08g029200.2.1	0	0	0	0	0	0	0	0	0	0	0	0	0
Solyc08g029220.1.1	5	19	2	12	0	45	13	76	1	46	9	25	0
Solyc08g029230.2.1	3	8	0	1	0	16	16	16	5	8	4	7	0
Solyc08g029260.1.1	0	0	0	0	0	0	0	0	0	0	0	0	0
Solyc08g036430.1.1	0	0	0	0	0	0	0	0	0	0	0	0	0
Solyc08g036520.2.1	0	0	0	0	0	0	0	0	0	0	0	0	0
Solyc08g036560.2.1	1	1	0	0	0	7	7	4	0	0	0	1	0
Solyc08g036600.1.1	1	4	0	0	0	0	11	1	8	2	2	6	2
Solyc08g036640.2.1	0	0	0	0	0	0	0	0	0	0	0	0	0
Solyc08g036660.2.1	0	0	0	0	0	0	0	0	0	0	0	0	0
Solyc08g041700.1.1	0	0	0	0	0	0	0	0	0	0	0	0	0
Solyc08g041710.2.1	3	40	0	20	7	37	15	47	3	214	17	108	0
Solyc08g041770.2.1	1	11	2	11	0	11	13	18	6	22	11	25	4
Solyc08g041780.2.1	8	31	4	25	5	71	42	126	14	96	23	104	1
Solyc08g041800.2.1	0	0	0	0	0	0	0	0	0	0	0	0	0
Solyc08g041820.2.1	9	116	11	84	12	74	61	102	5	47	7	40	0
Solyc08g041860.1.1	4	240	46	194	10	190	49	105	10	146	20	129	0

Solyc08g041870.2.1	5	98	10	108	12	187	147	263	23	356	78	240	1
Solyc08g041890.2.1	5	95	4	49	4	70	38	168	12	274	21	225	1
Solyc08g041930.1.1	2	5	0	5	0	14	7	12	0	5	1	4	2
Solyc08g041980.2.1	2	47	2	33	1	195	33	249	12	81	21	46	2
Solyc08g042000.2.1	1	0	5	0	4	7	3	0	0	0	0	0	0
Solyc08g042010.2.1	0	0	0	0	0	0	0	0	0	0	0	0	0
Solyc08g042030.1.1	0	0	0	0	0	0	0	0	0	0	0	0	0
Solyc08g042040.2.1	1	2	0	0	0	10	3	3	0	3	6	7	0
Solyc08g042050.2.1	14	336	33	282	12	626	361	891	32	325	56	326	6
Solyc08g042080.2.1	5	23	4	25	2	51	26	60	2	20	14	22	0
Solyc08g042100.2.1	0	0	0	0	0	0	0	0	0	0	0	0	0
Solyc08g042120.1.1	0	0	0	0	0	0	0	0	0	0	0	0	0
Solyc08g042130.2.1	4	17	5	12	0	44	37	57	11	20	5	23	1
Solyc08g042140.2.1	0	0	0	0	0	0	0	0	0	0	0	0	0
Solyc08g043140.2.1	0	0	0	0	0	0	0	0	0	0	0	0	0
Solyc08g043170.2.1	12	62	9	44	8	187	149	310	22	139	32	130	4
Solyc08g043180.2.1	4	96	7	27	0	33	46	73	1	38	4	40	0
Solyc08g044260.2.1	9	23	7	15	34	749	970	241	326	58	50	9	9
Solyc08g044280.1.1	0	0	0	0	0	0	0	0	0	0	0	0	0
Solyc08g044400.1.1	9	37	2	46	9	57	66	116	13	85	26	45	4
Solyc08g044410.1.1	1	10	1	4	0	10	7	4	2	27	5	25	0
Solyc08g044540.1.1	0	0	0	0	0	0	0	0	0	0	0	0	0
Solyc08g045640.2.1	0	0	0	0	0	0	0	0	0	0	0	0	0
Solyc08g045710.1.1	0	0	0	0	0	0	0	0	0	0	0	0	0
Solyc08g045750.1.1	0	0	0	0	0	0	0	0	0	0	0	0	0
Solyc08g045780.2.1	3	21	1	3	0	6	7	13	0	31	2	20	0
Solyc08g045850.2.1	6	61	5	25	4	82	142	124	17	91	20	53	0
Solyc08g048240.2.1	0	0	0	0	0	0	0	0	0	0	0	0	0
Solyc08g048250.2.1	2	21	1	8	0	26	11	17	2	18	8	14	0
Solyc08g048280.1.1	0	0	0	0	0	0	0	0	0	0	0	0	0
Solyc08g048290.2.1	0	0	0	0	0	0	0	0	0	0	0	0	0
Solyc08g048370.2.1	4	16	2	12	0	18	10	23	1	14	0	6	0
Solyc08g048390.1.1	8	92	4	35	2	145	144	84	9	85	15	29	0
Solyc08g048420.2.1	0	0	0	0	0	0	0	0	0	0	0	0	0
Solyc08g048430.2.1	4	32	6	18	1	32	34	27	5	37	5	28	3
Solyc08g048450.2.1	6	17	6	9	1	270	544	251	14	111	94	141	1
Solyc08g048460.2.1	1	5	0	8	0	57	47	26	2	9	6	6	0
Solyc08g048500.2.1	17	106	15	97	19	320	206	430	53	215	98	187	8
Solyc08g048510.1.1	0	0	0	0	0	0	0	0	0	0	0	0	0
Solyc08g048540.1.1	0	0	0	0	0	0	0	0	0	0	0	0	0
Solyc08g048550.2.1	3	16	13	10	4	18	106	19	7	41	12	30	15

Solyc08g059660.1.1	13	430	61	489	61	625	466	756	113	776	267	849	12
Solyc08g059700.1.1	0	0	0	0	0	0	0	0	0	0	0	0	0
Solyc08g059710.2.1	0	0	0	0	0	0	0	0	0	0	0	0	0
Solyc08g059730.1.1	5	17	2	0	1	23	25	9	13	10	6	9	0
Solyc08g059760.2.1	4	39	8	9	0	37	75	17	0	22	4	44	0
Solyc08g059780.1.1	0	0	0	0	0	0	0	0	0	0	0	0	0
Solyc08g059800.2.1	7	43	3	12	4	9	18	31	2	50	20	26	0
Solyc08g060810.2.1	9	151	19	176	2	680	178	434	21	630	116	357	6
Solyc08g060830.2.1	1	6	0	9	0	16	7	16	1	18	0	6	0
Solyc08g060840.1.1	7	31	4	25	1	48	27	58	6	65	11	62	3
Solyc08g060860.2.1	3	9	3	6	1	8	28	7	0	23	12	6	1
Solyc08g060920.2.1	4	39	0	23	0	159	27	127	4	56	16	116	0
Solyc08g060940.1.1	2	49	0	56	0	91	17	60	1	182	7	161	0
Solyc08g060970.2.1	0	0	0	0	0	0	0	0	0	0	0	0	0
Solyc08g061000.2.1	5	40	8	27	2	76	62	185	13	44	11	37	3
Solyc08g061010.2.1	0	0	0	0	0	0	0	0	0	0	0	0	0
Solyc08g061060.1.1	0	0	0	0	0	0	0	0	0	0	0	0	0
Solyc08g061090.2.1	5	28	8	7	7	19	23	32	2	34	13	41	0
Solyc08g061100.2.1	19	1861	110	1522	230	1000	605	2086	151	4969	675	2609	59
Solyc08g061130.2.1	4	8	0	2	0	18	9	20	3	39	21	45	0
Solyc08g061140.2.1	11	39	11	26	5	104	132	156	14	79	15	43	0
Solyc08g061160.1.1	0	0	0	0	0	0	0	0	0	0	0	0	0
Solyc08g061220.2.1	5	25	2	13	1	79	38	39	5	27	9	24	1
Solyc08g061230.2.1	1	0	0	0	0	13	12	1	4	0	0	0	0
Solyc08g061240.1.1	1	3	1	4	0	13	48	14	4	0	1	0	0
Solyc08g061250.2.1	0	0	0	0	0	0	0	0	0	0	0	0	0
Solyc08g061260.2.1	3	140	10	108	10	524	266	671	81	88	34	112	0
Solyc08g061270.1.1	6	250	0	110	5	892	10	755	7	200	77	46	0
Solyc08g061320.2.1	11	175	14	167	14	297	185	349	27	220	50	353	3
Solyc08g061450.1.1	0	0	0	0	0	0	0	0	0	0	0	0	0
Solyc08g061480.2.1	7	9	0	3	2	135	48	52	1	5	5	5	0
Solyc08g061490.2.1	3	28	2	54	8	7	8	8	2	46	9	77	0
Solyc08g061500.1.1	7	21	3	27	8	48	76	65	5	32	12	22	3
Solyc08g061510.1.1	0	0	0	0	0	0	0	0	0	0	0	0	0
Solyc08g061520.2.1	2	6	0	4	0	12	22	49	2	13	0	7	0
Solyc08g061560.2.1	1	9	3	4	0	0	3	2	0	11	1	5	0
Solyc08g061570.1.1	0	0	0	0	0	0	0	0	0	0	0	0	0
Solyc08g061580.2.1	0	0	0	0	0	0	0	0	0	0	0	0	0
Solyc08g061590.2.1	6	68	5	48	0	47	43	47	11	314	23	194	2
Solyc08g061610.2.1	5	73	10	58	0	26	27	34	7	39	19	59	0
Solyc08g061630.2.1	4	94	3	32	0	5	90	52	0	43	4	13	1

Solyc08g061640.1.1	0	0	0	0	0	0	0	0	0	0	0	0	0
Solyc08g061650.2.1	0	0	0	0	0	0	0	0	0	0	0	0	0
Solyc08g061800.2.1	0	0	0	0	0	0	0	0	0	0	0	0	0
Solyc08g061810.2.1	2	28	2	23	0	20	17	13	1	28	0	13	0
Solyc08g061820.2.1	11	64	12	72	19	174	260	166	36	170	77	106	6
Solyc08g061850.2.1	3	82	3	52	1	124	37	227	7	284	38	302	2
Solyc08g061880.1.1	0	0	0	0	0	0	0	0	0	0	0	0	0
Solyc08g061890.2.1	0	0	0	0	0	0	0	0	0	0	0	0	0
Solyc08g061910.2.1	0	0	0	0	0	0	0	0	0	0	0	0	0
Solyc08g061930.2.1	4	36	4	14	2	0	59	16	0	10	1	5	0
Solyc08g061960.2.1	0	0	0	0	0	0	0	0	0	0	0	0	0
Solyc08g061970.2.1	2	21	8	7	0	6	13	22	0	3	12	2	0
Solyc08g061980.2.1	3	12	0	13	0	8	6	23	4	15	6	23	1
Solyc08g062100.1.1	1	21	3	5	10	51	91	34	52	54	81	11	3
Solyc08g062110.1.1	0	0	0	0	0	0	0	0	0	0	0	0	0
Solyc08g062180.1.1	3	21	1	4	0	14	12	10	0	0	0	1	0
Solyc08g062190.2.1	6	165	23	154	12	897	233	517	39	180	49	138	0
Solyc08g062210.2.1	12	174	14	136	17	250	92	578	66	272	78	171	2
Solyc08g062220.2.1	4	158	7	83	0	102	12	70	0	8	0	0	0
Solyc08g062330.2.1	0	0	0	0	0	0	0	0	0	0	0	0	0
Solyc08g062340.2.1	0	0	0	0	0	0	0	0	0	0	0	0	0
Solyc08g062360.2.1	3	37	8	84	1	80	8	27	0	3	0	1	0
Solyc08g062370.2.1	0	0	0	0	0	0	0	0	0	0	0	0	0
Solyc08g062380.1.1	0	0	0	0	0	0	0	0	0	0	0	0	0
Solyc08g062420.2.1	2	11	0	4	0	17	17	17	4	29	4	45	0
Solyc08g062450.1.1	2	6	0	2	0	2	0	0	0	33	26	307	3
Solyc08g062490.2.1	0	0	0	0	0	0	0	0	0	0	0	0	0
Solyc08g062500.2.1	15	42	2	34	1	99	164	130	19	83	27	45	0
Solyc08g062560.2.1	5	27	3	21	0	96	73	40	13	113	13	98	0
Solyc08g062610.2.1	8	48	33	20	1	42	264	70	17	146	63	143	1
Solyc08g062620.2.1	0	0	0	0	0	0	0	0	0	0	0	0	0
Solyc08g062630.2.1	19	300	22	325	10	779	298	1075	58	773	101	542	4
Solyc08g062640.2.1	2	52	7	40	7	35	15	80	7	38	6	28	0
Solyc08g062650.2.1	4	22	0	9	0	57	22	46	2	115	11	73	0
Solyc08g062660.2.1	5	152	16	73	3	205	76	324	21	680	72	479	5
Solyc08g062670.2.1	2	17	2	22	1	202	110	254	15	229	42	217	12
Solyc08g062680.1.1	7	279	24	150	12	205	45	128	21	16	6	70	0
Solyc08g062690.2.1	4	198	10	79	1	182	31	84	18	17	5	27	0
Solyc08g062700.2.1	2	8	1	6	5	23	2	28	4	51	16	61	0
Solyc08g062760.1.1	0	0	0	0	0	0	0	0	0	0	0	0	0
Solyc08g062770.2.1	4	20	4	13	0	89	26	76	12	45	9	32	0

Solyc08g062780.1.1	0	0	0	0	0	0	0	0	0	0	0	0	0
Solyc08g062800.2.1	6	155	9	128	11	417	174	396	24	427	80	336	3
Solyc08g062810.1.1	0	0	0	0	0	0	0	0	0	0	0	0	0
Solyc08g062820.2.1	2	8	4	14	0	191	58	159	7	49	4	36	0
Solyc08g062860.2.1	2	11	3	0	0	5	4	9	2	7	3	5	0
Solyc08g062910.2.1	0	0	0	0	0	0	0	0	0	0	0	0	0
Solyc08g062920.2.1	8	193	68	334	70	1222	770	824	125	673	439	910	15
Solyc08g062930.1.1	0	0	0	0	0	0	0	0	0	0	0	0	0
Solyc08g062940.2.1	0	0	0	0	0	0	0	0	0	0	0	0	0
Solyc08g062950.2.1	0	0	0	0	0	0	0	0	0	0	0	0	0
Solyc08g062960.2.1	6	19	1	13	3	51	9	92	1	284	37	159	1
Solyc08g062970.1.1	2	16	1	5	1	27	8	11	0	28	5	6	0
Solyc08g063000.2.1	4	26	1	12	0	21	4	23	0	94	30	80	2
Solyc08g063010.2.1	2	4	0	6	0	46	3	57	6	5	2	7	0
Solyc08g063030.2.1	0	0	0	0	0	0	0	0	0	0	0	0	0
Solyc08g063040.2.1	14	147	21	236	0	106	144	182	0	15	6	6	0
Solyc08g063050.2.1	9	104	8	75	27	190	169	235	43	140	95	168	6
Solyc08g063070.2.1	3	34	4	21	0	192	46	64	13	65	12	63	1
Solyc08g063080.2.1	8	106	19	89	1	191	198	278	20	177	52	103	4
Solyc08g063090.2.1	4	30	1	13	2	0	6	0	2	36	9	28	0
Solyc08g063130.2.1	0	0	0	0	0	0	0	0	0	0	0	0	0
Solyc08g065140.1.1	3	25	0	10	2	3	6	8	0	8	0	7	0
Solyc08g065150.1.1	7	131	16	60	15	54	46	91	58	95	21	47	0
Solyc08g065160.2.1	2	112	5	86	3	130	97	169	43	209	26	121	0
Solyc08g065190.2.1	4	35	3	20	0	232	8	230	6	71	40	53	2
Solyc08g065210.1.1	0	0	0	0	0	0	0	0	0	0	0	0	0
Solyc08g065220.2.1	16	2248	493	1966	15	194	635	624	18	471	179	443	19
Solyc08g065230.1.1	1	2	0	0	0	2	6	5	0	1	0	1	0
Solyc08g065250.2.1	7	19	1	3	0	23	15	39	12	24	3	25	1
Solyc08g065260.2.1	5	88	7	38	0	60	74	98	2	125	4	71	2
Solyc08g065310.2.1	0	0	0	0	0	0	0	0	0	0	0	0	0
Solyc08g065320.2.1	6	5	22	7	5	68	322	49	8	34	16	32	0
Solyc08g065330.2.1	6	34	8	15	1	97	233	127	19	15	9	24	0
Solyc08g065350.2.1	1	11	1	17	0	3	9	10	0	66	1	49	0
Solyc08g065360.2.1	5	22	14	9	7	8	14	8	4	38	1	20	0
Solyc08g065410.1.1	0	0	0	0	0	0	0	0	0	0	0	0	0
Solyc08g065420.2.1	0	0	0	0	0	0	0	0	0	0	0	0	0
Solyc08g065430.2.1	3	59	2	0	2	7	17	23	14	38	7	20	1
Solyc08g065440.2.1	0	0	0	0	0	0	0	0	0	0	0	0	0
Solyc08g065450.1.1	2	2	0	0	0	8	11	8	1	5	1	7	1
Solyc08g065460.1.1	1	6	0	4	0	8	4	11	0	14	1	6	1

Solyc08g065470.2.1	0	0	0	0	0	0	0	0	0	0	0	0	0
Solyc08g065480.2.1	10	19	8	39	3	173	49	88	33	34	25	98	2
Solyc08g065490.2.1	0	0	0	0	0	0	0	0	0	0	0	0	0
Solyc08g065540.2.1	0	0	0	0	0	0	0	0	0	0	0	0	0
Solyc08g065570.1.1	0	0	0	0	0	0	0	0	0	0	0	0	0
Solyc08g065600.1.1	0	0	0	0	0	0	0	0	0	0	0	0	0
Solyc08g065610.2.1	11	596	142	1260	36	2720	1890	3762	237	5242	699	634	16
Solyc08g065630.1.1	0	0	0	0	0	0	0	0	0	0	0	0	0
Solyc08g065640.2.1	3	26	3	22	3	39	29	97	1	89	12	71	0
Solyc08g065710.1.1	0	0	0	0	0	0	0	0	0	0	0	0	0
Solyc08g065720.1.1	0	0	0	0	0	0	0	0	0	0	0	0	0
Solyc08g065760.2.1	3	21	3	19	3	81	21	102	10	33	30	53	5
Solyc08g065780.1.1	0	0	0	0	0	0	0	0	0	0	0	0	0
Solyc08g065790.2.1	0	0	0	0	0	0	0	0	0	0	0	0	0
Solyc08g065800.1.1	0	0	0	0	0	0	0	0	0	0	0	0	0
Solyc08g065810.1.1	0	0	0	0	0	0	0	0	0	0	0	0	0
Solyc08g065820.1.1	0	0	0	0	0	0	0	0	0	0	0	0	0
Solyc08g065830.2.1	0	0	0	0	0	0	0	0	0	0	0	0	0
Solyc08g065840.2.1	2	13	0	2	0	11	15	22	0	12	6	12	0
Solyc08g065850.1.1	2	138	9	44	2	40	24	26	6	149	14	107	0
Solyc08g065860.2.1	0	0	0	0	0	0	0	0	0	0	0	0	0
Solyc08g065870.2.1	14	48	10	32	14	255	362	217	20	201	56	119	3
Solyc08g065880.2.1	4	58	4	34	5	163	29	154	28	195	19	240	5
Solyc08g065890.2.1	12	168	71	124	22	408	457	513	89	518	113	351	3
Solyc08g065900.2.1	5	119	12	50	4	145	128	174	24	209	69	106	9
Solyc08g065910.1.1	0	0	0	0	0	0	0	0	0	0	0	0	0
Solyc08g065940.2.1	0	0	0	0	0	0	0	0	0	0	0	0	0
Solyc08g065950.1.1	1	6	1	11	0	6	22	8	0	36	1	25	0
Solyc08g065970.2.1	4	32	3	12	3	2	20	16	0	17	10	20	1
Solyc08g065980.2.1	17	92	3	85	11	224	114	244	22	170	30	125	3
Solyc08g065990.2.1	6	33	2	20	0	138	59	98	3	111	19	99	0
Solyc08g066000.1.1	2	5	1	2	0	0	9	8	0	8	1	6	0
Solyc08g066010.2.1	7	5	1	12	0	17	51	41	12	67	17	60	5
Solyc08g066020.1.1	0	0	0	0	0	0	0	0	0	0	0	0	0
Solyc08g066030.2.1	3	2	0	7	0	1	14	4	1	33	4	30	1
Solyc08g066040.1.1	0	0	0	0	0	0	0	0	0	0	0	0	0
Solyc08g066050.2.1	0	0	0	0	0	0	0	0	0	0	0	0	0
Solyc08g066060.1.1	0	0	0	0	0	0	0	0	0	0	0	0	0
Solyc08g066070.1.1	15	184	32	116	20	221	256	233	32	241	19	283	4
Solyc08g066100.2.1	1	0	0	0	0	0	0	0	0	58	8	16	0
Solyc08g066110.2.1	7	297	32	152	42	448	537	340	95	787	213	626	28

Solyc08g066120.2.1	10	36	14	43	3	49	165	114	9	52	16	73	1
Solyc08g066130.1.1	1	2	1	8	0	8	22	16	1	5	14	9	0
Solyc08g066140.2.1	3	18	2	8	0	15	15	38	0	23	1	22	0
Solyc08g066160.2.1	1	12	4	0	0	2	5	14	0	34	9	22	0
Solyc08g066180.2.1	5	59	2	25	3	26	28	28	7	36	5	46	2
Solyc08g066200.2.1	6	27	4	23	2	70	66	91	14	86	22	47	0
Solyc08g066210.2.1	18	105	36	69	34	146	89	114	27	50	16	84	6
Solyc08g066240.2.1	0	0	0	0	0	0	0	0	0	0	0	0	0
Solyc08g066250.2.1	0	0	0	0	0	0	0	0	0	0	0	0	0
Solyc08g066310.1.1	0	0	0	0	0	0	0	0	0	0	0	0	0
Solyc08g066320.2.1	0	0	0	0	0	0	0	0	0	0	0	0	0
Solyc08g066330.1.1	3	50	8	32	4	46	25	71	3	173	14	189	0
Solyc08g066350.1.1	0	0	0	0	0	0	0	0	0	0	0	0	0
Solyc08g066360.2.1	2	3	1	0	20	2	10	3	5	7	0	0	0
Solyc08g066390.2.1	1	1	0	1	3	4	9	1	1	1	0	0	2
Solyc08g066420.1.1	0	0	0	0	0	0	0	0	0	0	0	0	0
Solyc08g066430.1.1	0	0	0	0	0	0	0	0	0	0	0	0	0
Solyc08g066440.2.1	5	79	6	42	1	79	26	46	11	42	23	36	0
Solyc08g066450.1.1	0	0	0	0	0	0	0	0	0	0	0	0	0
Solyc08g066490.2.1	7	13	2	12	0	94	19	39	0	31	18	32	0
Solyc08g066500.2.1	2	44	2	22	0	28	11	29	0	3	2	4	0
Solyc08g066510.2.1	0	0	0	0	0	0	0	0	0	0	0	0	0
Solyc08g066520.2.1	1	7	0	0	0	9	2	4	3	6	4	15	0
Solyc08g066530.2.1	1	9	2	3	1	5	5	2	0	0	0	0	0
Solyc08g066540.2.1	0	0	0	0	0	0	0	0	0	0	0	0	0
Solyc08g066550.2.1	0	0	0	0	0	0	0	0	0	0	0	0	0
Solyc08g066560.2.1	0	0	0	0	0	0	0	0	0	0	0	0	0
Solyc08g066570.1.1	11	42	7	36	0	155	97	158	8	255	25	201	2
Solyc08g066580.2.1	4	13	0	22	0	13	20	37	5	4	4	11	0
Solyc08g066600.2.1	14	138	11	57	2	53	57	96	17	201	43	165	8
Solyc08g066610.2.1	0	0	0	0	0	0	0	0	0	0	0	0	0
Solyc08g066620.2.1	0	0	0	0	0	0	0	0	0	0	0	0	0
Solyc08g066630.2.1	0	0	0	0	0	0	0	0	0	0	0	0	0
Solyc08g066640.2.1	3	11	0	8	5	25	9	28	0	36	1	7	0
Solyc08g066650.2.1	0	0	0	0	0	0	0	0	0	0	0	0	0
Solyc08g066660.1.1	0	0	0	0	0	0	0	0	0	0	0	0	0
Solyc08g066670.2.1	4	10	4	3	0	0	25	14	0	35	0	29	0
Solyc08g066680.2.1	4	9	0	7	1	18	15	26	9	27	24	17	2
Solyc08g066690.2.1	1	0	0	0	0	0	0	1	0	60	9	5	0
Solyc08g066700.2.1	4	12	7	14	2	8	52	29	6	98	33	54	6
Solyc08g066720.1.1	0	0	0	0	0	0	0	0	0	0	0	0	0

Solyc08g066730.2.1	4	10	1	15	0	21	16	68	4	31	14	27	1
Solyc08g066740.2.1	1	8	1	0	0	0	14	2	0	22	11	16	1
Solyc08g066750.2.1	2	28	1	24	1	17	10	21	1	75	12	69	0
Solyc08g066760.2.1	0	0	0	0	0	0	0	0	0	0	0	0	0
Solyc08g066770.2.1	3	14	3	3	2	33	1	19	1	0	1	0	0
Solyc08g066790.2.1	3	6	0	13	3	35	27	72	4	16	13	14	0
Solyc08g066800.2.1	14	210	25	112	20	219	91	370	25	438	32	292	7
Solyc08g066810.2.1	1	3	0	0	0	2	6	25	0	0	0	5	0
Solyc08g066820.2.1	2	29	4	9	0	72	11	38	0	25	0	25	0
Solyc08g066840.2.1	2	102	5	74	0	134	9	177	0	39	5	47	0
Solyc08g066850.2.1	7	86	23	40	2	28	112	60	2	21	6	37	0
Solyc08g066860.2.1	0	0	0	0	0	0	0	0	0	0	0	0	0
Solyc08g066870.2.1	6	39	3	39	24	113	91	139	39	90	16	27	1
Solyc08g066880.2.1	0	0	0	0	0	0	0	0	0	0	0	0	0
Solyc08g066920.1.1	0	0	0	0	0	0	0	0	0	0	0	0	0
Solyc08g066940.2.1	2	18	3	8	2	31	40	52	7	64	8	13	0
Solyc08g066950.2.1	9	45	3	28	5	51	46	60	9	57	26	65	4
Solyc08g066960.2.1	0	0	0	0	0	0	0	0	0	0	0	0	0
Solyc08g066970.2.1	1	5	6	2	1	9	4	9	2	5	4	4	0
Solyc08g066980.2.1	0	0	0	0	0	0	0	0	0	0	0	0	0
Solyc08g066990.2.1	0	0	0	0	0	0	0	0	0	0	0	0	0
Solyc08g067000.2.1	0	0	0	0	0	0	0	0	0	0	0	0	0
Solyc08g067020.2.1	2	51	3	27	2	112	13	152	5	114	3	75	0
Solyc08g067030.2.1	6	744	23	641	8	69	152	105	2	1645	44	1511	8
Solyc08g067040.2.1	7	24	8	15	3	66	60	97	11	115	18	84	3
Solyc08g067050.2.1	0	0	0	0	0	0	0	0	0	0	0	0	0
Solyc08g067060.1.1	2	1	1	3	0	9	7	20	0	8	3	7	0
Solyc08g067070.2.1	0	0	0	0	0	0	0	0	0	0	0	0	0
Solyc08g067090.2.1	9	103	7	48	2	262	74	354	30	195	29	153	3
Solyc08g067100.2.1	23	1002	56	700	10	1079	1163	3042	32	372	66	500	2
Solyc08g067150.2.1	4	6	0	5	3	15	15	18	1	26	2	10	0
Solyc08g067160.2.1	5	78	4	35	5	145	20	80	2	150	9	389	8
Solyc08g067170.1.1	0	0	0	0	0	0	0	0	0	0	0	0	0
Solyc08g067180.2.1	0	0	0	0	0	0	0	0	0	0	0	0	0
Solyc08g067190.2.1	0	0	0	0	0	0	0	0	0	0	0	0	0
Solyc08g067200.1.1	0	0	0	0	0	0	0	0	0	0	0	0	0
Solyc08g067210.2.1	3	4	0	6	0	25	21	13	0	5	4	5	0
Solyc08g067230.2.1	0	0	0	0	0	0	0	0	0	0	0	0	0
Solyc08g067240.2.1	0	0	0	0	0	0	0	0	0	0	0	0	0
Solyc08g067250.2.1	0	0	0	0	0	0	0	0	0	0	0	0	0
Solyc08g067260.2.1	15	183	9	5	84	246	137	24	266	546	73	81	26

Solyc08g067270.2.1	0	0	0	0	0	0	0	0	0	0	0	0	0
Solyc08g067280.1.1	0	0	0	0	0	0	0	0	0	0	0	0	0
Solyc08g067290.2.1	7	35	5	20	0	72	24	48	13	40	40	34	2
Solyc08g067300.1.1	0	0	0	0	0	0	0	0	0	0	0	0	0
Solyc08g067310.1.1	4	10	0	12	0	28	13	9	4	0	2	0	0
Solyc08g067320.1.1	3	198	79	73	0	1	21	1	0	1	4	1	0
Solyc08g067330.1.1	2	62	49	51	2	0	68	0	0	3	2	1	0
Solyc08g067340.2.1	0	0	0	0	0	0	0	0	0	0	0	0	0
Solyc08g067360.2.1	0	0	0	0	0	0	0	0	0	0	0	0	0
Solyc08g067370.1.1	13	304	13	189	0	594	161	975	1	161	5	231	0
Solyc08g067390.2.1	2	4	1	1	3	19	20	9	4	0	0	4	0
Solyc08g067410.1.1	10	85	0	24	4	405	128	312	115	81	3	44	0
Solyc08g067420.2.1	9	25	0	8	0	82	49	35	13	56	9	30	0
Solyc08g067430.2.1	3	65	13	28	1	48	42	59	10	52	12	45	3
Solyc08g067440.1.1	0	0	0	0	0	0	0	0	0	0	0	0	0
Solyc08g067450.1.1	0	0	0	0	0	0	0	0	0	0	0	0	0
Solyc08g067470.2.1	2	6	0	0	0	8	4	10	0	37	1	25	0
Solyc08g067480.2.1	4	73	5	20	4	92	55	86	13	75	18	79	1
Solyc08g067500.1.1	3	13	2	13	0	72	50	91	0	5	2	3	0
Solyc08g067510.1.1	0	0	0	0	0	0	0	0	0	0	0	0	0
Solyc08g067530.1.1	3	23	0	0	6	0	38	1	32	2	1	2	20
Solyc08g067540.1.1	0	0	0	0	0	0	0	0	0	0	0	0	0
Solyc08g067570.1.1	0	0	0	0	0	0	0	0	0	0	0	0	0
Solyc08g067610.2.1	0	0	0	0	0	0	0	0	0	0	0	0	0
Solyc08g067620.2.1	0	0	0	0	0	0	0	0	0	0	0	0	0
Solyc08g067630.2.1	4	9	1	1	0	256	274	301	20	2	0	0	0
Solyc08g067640.1.1	0	0	0	0	0	0	0	0	0	0	0	0	0
Solyc08g067670.2.1	0	0	0	0	0	0	0	0	0	0	0	0	0
Solyc08g067740.2.1	0	0	0	0	0	0	0	0	0	0	0	0	0
Solyc08g067760.2.1	0	0	0	0	0	0	0	0	0	0	0	0	0
Solyc08g067770.2.1	4	67	15	50	1	258	76	220	9	56	23	47	2
Solyc08g067780.2.1	0	0	0	0	0	0	0	0	0	0	0	0	0
Solyc08g067790.2.1	20	221	15	183	13	370	256	576	45	259	75	202	4
Solyc08g067800.1.1	0	0	0	0	0	0	0	0	0	0	0	0	0
Solyc08g067810.2.1	6	81	13	33	2	484	127	86	27	249	32	160	1
Solyc08g067820.2.1	3	2	0	7	2	30	10	14	1	16	3	20	0
Solyc08g067830.2.1	4	10	1	15	0	15	6	20	5	70	14	48	0
Solyc08g067840.2.1	2	10	0	4	0	6	18	13	0	8	0	8	0
Solyc08g067850.2.1	15	108	14	93	7	134	128	200	9	104	21	60	1
Solyc08g067860.1.1	1	5	2	5	0	5	6	3	0	2	0	0	0
Solyc08g067870.1.1	11	19	5	14	0	63	69	100	3	40	9	41	0

Solyc08g067880.2.1	4	41	5	39	4	114	89	103	18	92	36	67	0
Solyc08g067890.2.1	1	16	0	7	0	12	2	26	2	58	2	36	0
Solyc08g067900.2.1	2	10	2	1	0	9	7	28	7	10	0	6	0
Solyc08g067910.2.1	6	166	3	61	8	479	61	338	11	155	49	124	2
Solyc08g067920.2.1	21	90	9	104	2	212	152	255	21	107	36	143	2
Solyc08g067930.2.1	0	0	0	0	0	0	0	0	0	0	0	0	0
Solyc08g067950.2.1	1	26	0	4	0	12	4	24	5	89	7	43	0
Solyc08g067960.2.1	12	146	97	92	11	446	1270	325	45	447	92	353	10
Solyc08g067970.2.1	1	3	1	5	0	0	3	2	0	1	0	14	0
Solyc08g067990.1.1	0	0	0	0	0	0	0	0	0	0	0	0	0
Solyc08g068000.1.1	0	0	0	0	0	0	0	0	0	0	0	0	0
Solyc08g068010.1.1	6	12	5	23	1	158	37	87	8	28	10	32	1
Solyc08g068020.1.1	1	2	0	0	0	5	6	7	0	1	0	3	0
Solyc08g068030.1.1	0	0	0	0	0	0	0	0	0	0	0	0	0
Solyc08g068040.2.1	2	3	0	1	0	8	8	10	0	1	1	9	0
Solyc08g068050.2.1	0	0	0	0	0	0	0	0	0	0	0	0	0
Solyc08g068060.2.1	3	11	0	10	0	17	17	17	3	29	4	28	0
Solyc08g068070.2.1	3	20	2	12	0	3	5	6	0	45	30	52	6
Solyc08g068080.2.1	0	0	0	0	0	0	0	0	0	0	0	0	0
Solyc08g068090.2.1	1	16	2	7	0	15	5	5	0	5	0	4	0
Solyc08g068100.2.1	4	34	2	27	0	17	9	50	3	190	13	149	0
Solyc08g068110.1.1	0	0	0	0	0	0	0	0	0	0	0	0	0
Solyc08g068120.1.1	0	0	0	0	0	0	0	0	0	0	0	0	0
Solyc08g068130.1.1	0	0	0	0	0	0	0	0	0	0	0	0	0
Solyc08g068140.2.1	0	0	0	0	0	0	0	0	0	0	0	0	0
Solyc08g068150.2.1	15	1857	312	107	1180	13324	6506	971	12691	702	1665	58	390
Solyc08g068160.1.1	0	0	0	0	0	0	0	0	0	0	0	0	0
Solyc08g068170.1.1	0	0	0	0	0	0	0	0	0	0	0	0	0
Solyc08g068180.2.1	5	211	20	94	9	288	228	305	90	881	80	351	1
Solyc08g068200.2.1	7	45	4	21	0	14	27	43	1	23	6	19	0
Solyc08g068210.2.1	18	239	13	179	18	352	134	511	50	505	129	290	4
Solyc08g068220.2.1	0	0	0	0	0	0	0	0	0	0	0	0	0
Solyc08g068230.2.1	10	91	25	132	6	60	150	163	17	0	10	3	0
Solyc08g068240.1.1	0	0	0	0	0	0	0	0	0	0	0	0	0
Solyc08g068250.2.1	0	0	0	0	0	0	0	0	0	0	0	0	0
Solyc08g068260.1.1	0	0	0	0	0	0	0	0	0	0	0	0	0
Solyc08g068270.2.1	1	5	2	3	0	6	1	11	2	2	1	4	0
Solyc08g068290.2.1	5	130	8	20	7	45	55	109	3	71	29	42	0
Solyc08g068300.2.1	1	7	3	10	0	10	8	21	1	17	1	27	0
Solyc08g068310.2.1	6	63	3	24	2	72	60	126	16	74	16	59	9
Solyc08g068320.2.1	0	0	0	0	0	0	0	0	0	0	0	0	0

Solyc08g068330.2.1	4	8	0	4	1	67	35	25	12	83	6	89	1
Solyc08g068340.2.1	10	222	15	99	9	441	193	850	73	1150	140	710	28
Solyc08g068350.2.1	1	5	1	7	0	4	0	5	0	0	1	9	0
Solyc08g068370.2.1	11	105	17	57	0	172	135	210	33	104	28	83	1
Solyc08g068380.2.1	0	0	0	0	0	0	0	0	0	0	0	0	0
Solyc08g068390.2.1	2	43	1	27	0	8	8	27	0	70	0	40	0
Solyc08g068400.2.1	6	40	5	14	1	39	34	57	9	46	11	30	0
Solyc08g068420.2.1	0	0	0	0	0	0	0	0	0	0	0	0	0
Solyc08g068430.2.1	7	19	2	25	0	30	21	45	3	48	4	35	0
Solyc08g068460.1.1	0	0	0	0	0	0	0	0	0	0	0	0	0
Solyc08g068520.2.1	0	0	0	0	0	0	0	0	0	0	0	0	0
Solyc08g068530.2.1	0	0	0	0	0	0	0	0	0	0	0	0	0
Solyc08g068570.2.1	4	12	2	10	0	10	13	13	0	39	7	45	0
Solyc08g068580.2.1	4	15	1	7	1	31	23	26	2	29	6	20	0
Solyc08g068590.2.1	6	114	33	65	0	78	460	69	16	126	34	160	0
Solyc08g068600.2.1	1	3	4	0	0	0	0	0	0	0	0	8	0
Solyc08g068610.2.1	0	0	0	0	0	0	0	0	0	0	0	0	0
Solyc08g068640.2.1	0	0	0	0	0	0	0	0	0	0	0	0	0
Solyc08g068670.2.1	0	0	0	0	0	0	0	0	0	0	0	0	0
Solyc08g068680.2.1	0	0	0	0	0	0	0	0	0	0	0	0	0
Solyc08g068690.1.1	1	57	0	72	0	319	0	73	0	177	7	440	0
Solyc08g068700.1.1	0	0	0	0	0	0	0	0	0	0	0	0	0
Solyc08g068710.1.1	0	0	0	0	0	0	0	0	0	0	0	0	0
Solyc08g068730.1.1	1	7	0	0	0	111	4	67	1	0	1	0	0
Solyc08g068770.1.1	0	0	0	0	0	0	0	0	0	0	0	0	0
Solyc08g068780.1.1	0	0	0	0	0	0	0	0	0	0	0	0	0
Solyc08g068790.1.1	0	0	0	0	0	0	0	0	0	0	0	0	0
Solyc08g068800.2.1	0	0	0	0	0	0	0	0	0	0	0	0	0
Solyc08g068810.2.1	7	26	0	39	3	64	24	64	0	63	10	39	4
Solyc08g068820.2.1	0	0	0	0	0	0	0	0	0	0	0	0	0
Solyc08g068830.2.1	3	14	0	7	0	14	14	17	2	12	10	10	0
Solyc08g068840.2.1	1	2	0	1	2	5	2	0	0	0	5	1	0
Solyc08g068850.2.1	0	0	0	0	0	0	0	0	0	0	0	0	0
Solyc08g068860.2.1	0	0	0	0	0	0	0	0	0	0	0	0	0
Solyc08g068870.2.1	0	0	0	0	0	0	0	0	0	0	0	0	0
Solyc08g068880.1.1	8	106	6	71	12	115	88	150	18	136	52	65	1
Solyc08g068920.2.1	1	4	0	0	0	10	10	4	0	4	2	4	0
Solyc08g068930.2.1	0	0	0	0	0	0	0	0	0	0	0	0	0
Solyc08g068960.2.1	0	0	0	0	0	0	0	0	0	0	0	0	0
Solyc08g068970.2.1	0	0	0	0	0	0	0	0	0	0	0	0	0
Solyc08g069000.2.1	7	52	0	55	0	101	33	52	1	103	20	83	0

Solyc08g069010.2.1	3	33	6	6	0	1	9	5	0	9	2	22	0
Solyc08g069020.1.1	0	0	0	0	0	0	0	0	0	0	0	0	0
Solyc08g069030.2.1	9	134	33	97	5	262	461	301	26	134	67	80	5
Solyc08g069040.2.1	0	0	0	0	0	0	0	0	0	0	0	0	0
Solyc08g069050.2.1	8	42	10	37	0	80	23	51	6	34	17	41	0
Solyc08g069060.2.1	5	62	3	55	0	289	92	152	20	35	51	30	0
Solyc08g069080.2.1	2	32	3	9	0	12	23	14	6	56	10	24	0
Solyc08g069100.1.1	2	11	1	4	0	21	18	19	1	35	2	19	0
Solyc08g069110.2.1	0	0	0	0	0	0	0	0	0	0	0	0	0
Solyc08g069120.2.1	5	35	12	22	2	49	26	71	6	57	18	33	0
Solyc08g069130.1.1	0	0	0	0	0	0	0	0	0	0	0	0	0
Solyc08g069140.2.1	1	2	3	1	0	2	5	9	0	0	1	0	0
Solyc08g069160.2.1	2	12	0	10	0	29	4	18	2	9	7	8	0
Solyc08g069170.1.1	0	0	0	0	0	0	0	0	0	0	0	0	0
Solyc08g069180.2.1	0	0	0	0	0	0	0	0	0	0	0	0	0
Solyc08g069230.2.1	0	0	0	0	0	0	0	0	0	0	0	0	0
Solyc08g074240.2.1	2	6	1	6	0	20	5	45	3	60	6	37	4
Solyc08g074250.1.1	0	0	0	0	0	0	0	0	0	0	0	0	0
Solyc08g074260.2.1	0	0	0	0	0	0	0	0	0	0	0	0	0
Solyc08g074270.2.1	0	0	0	0	0	0	0	0	0	0	0	0	0
Solyc08g074280.2.1	11	67	3	49	4	87	46	113	13	84	15	34	1
Solyc08g074290.2.1	5	128	8	58	5	196	49	343	26	121	16	70	0
Solyc08g074300.2.1	0	0	0	0	0	0	0	0	0	0	0	0	0
Solyc08g074360.2.1	1	5	0	3	0	5	7	5	2	8	1	6	1
Solyc08g074370.2.1	22	71	17	65	8	282	144	350	28	201	60	121	2
Solyc08g074380.1.1	3	6	1	9	0	23	3	49	3	17	4	8	0
Solyc08g074390.2.1	2	2	1	4	1	1	4	6	1	19	0	6	0
Solyc08g074410.2.1	5	40	11	24	1	95	110	159	11	169	35	93	0
Solyc08g074420.2.1	8	45	14	30	4	84	105	102	24	59	18	72	3
Solyc08g074440.2.1	11	148	5	85	0	194	42	247	17	300	40	203	0
Solyc08g074450.2.1	4	14	3	3	0	6	17	7	0	13	1	16	0
Solyc08g074480.1.1	2	1396	33	741	0	8	47	18	0	2387	131	4194	1
Solyc08g074490.2.1	0	0	0	0	0	0	0	0	0	0	0	0	0
Solyc08g074500.2.1	6	29	6	16	1	41	34	34	4	64	13	28	4
Solyc08g074510.1.1	0	0	0	0	0	0	0	0	0	0	0	0	0
Solyc08g074520.1.1	1	2	0	0	0	8	4	6	3	6	5	4	0
Solyc08g074550.2.1	8	63	7	49	6	260	94	193	28	115	24	88	2
Solyc08g074560.2.1	5	144	19	113	0	116	136	132	3	46	11	43	0
Solyc08g074570.1.1	8	75	8	38	6	138	52	162	25	205	16	102	4
Solyc08g074580.2.1	3	12	1	0	0	20	38	39	7	15	21	16	4
Solyc08g074590.2.1	2	5	0	1	0	8	13	10	3	16	10	10	0

Solyc08g074600.2.1	4	3	0	1	0	0	7	3	1	7	3	6	0
Solyc08g074610.1.1	0	0	0	0	0	0	0	0	0	0	0	0	0
Solyc08g074620.1.1	4	42	6	0	5	0	19	0	0	0	8	0	11
Solyc08g074630.1.1	9	27	37	37	1	59	589	80	2	1	0	2	1
Solyc08g074660.1.1	1	16	0	2	2	5	7	5	9	6	9	0	0
Solyc08g074670.2.1	0	0	0	0	0	0	0	0	0	0	0	0	0
Solyc08g074680.2.1	1	0	0	2	0	14	6	10	0	0	5	0	0
Solyc08g074700.1.1	0	0	0	0	0	0	0	0	0	0	0	0	0
Solyc08g074720.2.1	0	0	0	0	0	0	0	0	0	0	0	0	0
Solyc08g074730.1.1	0	0	0	0	0	0	0	0	0	0	0	0	0
Solyc08g074740.2.1	0	0	0	0	0	0	0	0	0	0	0	0	0
Solyc08g074750.2.1	1	28	3	8	0	17	16	37	4	68	14	48	3
Solyc08g074760.2.1	10	204	23	123	23	197	91	259	26	498	132	322	3
Solyc08g074780.2.1	4	31	3	9	3	17	21	15	3	22	11	7	0
Solyc08g074790.2.1	7	233	8	83	1	270	80	287	25	475	70	368	6
Solyc08g074800.2.1	0	0	0	0	0	0	0	0	0	0	0	0	0
Solyc08g074830.2.1	0	0	0	0	0	0	0	0	0	0	0	0	0
Solyc08g074890.2.1	2	14	0	3	0	23	12	31	3	23	2	14	0
Solyc08g074910.2.1	2	2	0	5	2	19	5	26	1	10	6	6	0
Solyc08g074940.2.1	0	0	0	0	0	0	0	0	0	0	0	0	0
Solyc08g074960.2.1	0	0	0	0	0	0	0	0	0	0	0	0	0
Solyc08g074970.1.1	3	2	0	4	0	25	11	18	0	3	0	2	0
Solyc08g075010.2.1	2	3	1	2	0	12	13	29	0	0	8	6	0
Solyc08g075020.2.1	5	115	5	93	7	1798	220	1404	7	37	5	13	0
Solyc08g075070.2.1	1	2	0	0	0	6	11	3	1	4	2	1	0
Solyc08g075080.2.1	1	5	5	0	0	0	4	2	0	4	1	2	3
Solyc08g075090.2.1	0	0	0	0	0	0	0	0	0	0	0	0	0
Solyc08g075100.2.1	8	86	8	62	0	33	39	101	3	85	29	106	2
Solyc08g075120.2.1	6	65	4	29	0	16	14	27	0	90	11	78	0
Solyc08g075150.2.1	1	0	0	0	0	4	14	0	0	4	0	5	0
Solyc08g075160.2.1	9	50	4	67	0	55	58	85	6	134	39	137	1
Solyc08g075210.1.1	20	438	64	75	1	50	4	50	0	561	164	641	7
Solyc08g075230.1.1	0	0	0	0	0	0	0	0	0	0	0	0	0
Solyc08g075240.2.1	1	0	0	2	1	0	4	0	2	4	3	0	0
Solyc08g075260.1.1	0	0	0	0	0	0	0	0	0	0	0	0	0
Solyc08g075270.1.1	0	0	0	0	0	0	0	0	0	0	0	0	0
Solyc08g075280.2.1	5	22	26	26	5	198	150	179	10	101	21	94	2
Solyc08g075290.1.1	0	0	0	0	0	0	0	0	0	0	0	0	0
Solyc08g075300.2.1	0	0	0	0	0	0	0	0	0	0	0	0	0
Solyc08g075310.2.1	31	267	26	213	5	650	279	805	63	463	117	347	3
Solyc08g075320.2.1	1	0	0	0	0	135	1	9	1	0	0	0	0

Solyc08g075340.2.1	11	37	3	48	5	95	46	141	6	132	41	134	1
Solyc08g075350.1.1	0	0	0	0	0	0	0	0	0	0	0	0	0
Solyc08g075360.1.1	2	1	0	0	0	15	15	3	2	11	3	9	3
Solyc08g075370.2.1	4	589	20	284	14	1434	767	1858	132	199	96	135	2
Solyc08g075380.2.1	4	49	4	28	1	68	86	116	12	60	19	76	0
Solyc08g075390.2.1	12	74	5	57	0	145	60	254	13	206	34	195	0
Solyc08g075400.2.1	4	13	0	3	0	36	10	26	0	28	9	12	2
Solyc08g075410.2.1	2	15	0	9	0	21	3	24	1	22	6	13	0
Solyc08g075420.2.1	17	72	15	88	5	104	104	212	12	238	35	175	2
Solyc08g075430.2.1	4	7	2	7	0	29	13	95	0	7	2	10	0
Solyc08g075440.2.1	4	33	3	26	3	76	15	76	6	58	15	33	3
Solyc08g075450.2.1	0	0	0	0	0	0	0	0	0	0	0	0	0
Solyc08g075460.2.1	1	2	0	0	0	0	2	4	0	2	4	0	0
Solyc08g075470.2.1	0	0	0	0	0	0	0	0	0	0	0	0	0
Solyc08g075480.2.1	0	0	0	0	0	0	0	0	0	0	0	0	0
Solyc08g075490.2.1	30	2346	704	2229	48	99	60	226	1	561	171	269	27
Solyc08g075500.2.1	0	0	0	0	0	0	0	0	0	0	0	0	0
Solyc08g075510.2.1	1	0	0	1	2	7	2	2	1	0	3	1	0
Solyc08g075520.2.1	9	39	31	69	11	136	286	230	89	90	25	66	37
Solyc08g075530.2.1	17	44	8	62	0	413	260	516	8	278	28	240	3
Solyc08g075540.2.1	9	532	79	331	44	528	69	154	23	81	77	34	1
Solyc08g075550.2.1	1	0	1	0	0	29	8	5	3	0	0	0	0
Solyc08g075570.2.1	3	106	9	137	0	1	4	2	10	16	28	2	0
Solyc08g075580.1.1	0	0	0	0	0	0	0	0	0	0	0	0	0
Solyc08g075630.2.1	0	0	0	0	0	0	0	0	0	0	0	0	0
Solyc08g075640.2.1	0	0	0	0	0	0	0	0	0	0	0	0	0
Solyc08g075650.2.1	0	0	0	0	0	0	0	0	0	0	0	0	0
Solyc08g075670.1.1	3	11	1	7	0	20	31	30	3	19	1	16	0
Solyc08g075680.2.1	0	0	0	0	0	0	0	0	0	0	0	0	0
Solyc08g075690.2.1	2	974	26	570	39	543	130	574	46	7752	178	5449	24
Solyc08g075700.2.1	2	17	1	17	0	35	10	29	2	68	21	85	7
Solyc08g075710.2.1	2	12	0	10	0	27	20	26	2	9	1	6	0
Solyc08g075720.1.1	3	19	6	16	1	34	57	40	6	30	19	17	0
Solyc08g075730.2.1	5	47	2	33	5	71	49	134	9	73	17	88	4
Solyc08g075740.1.1	1	9	1	2	0	8	3	1	1	6	3	5	0
Solyc08g075750.2.1	7	94	20	51	2	178	394	305	29	77	80	54	9
Solyc08g075760.2.1	0	0	0	0	0	0	0	0	0	0	0	0	0
Solyc08g075770.2.1	3	7	0	19	0	23	16	40	3	25	3	13	0
Solyc08g075780.2.1	3	16	1	14	0	20	18	23	0	37	13	18	1
Solyc08g075790.2.1	0	0	0	0	0	0	0	0	0	0	0	0	0
Solyc08g075810.2.1	2	3	0	2	0	7	9	3	2	5	1	2	0

Solyc08g075820.2.1	0	0	0	0	0	0	0	0	0	0	0	0	0
Solyc08g075840.2.1	15	77	17	78	3	270	69	169	14	271	82	171	2
Solyc08g075850.2.1	1	2	0	3	0	20	1	8	0	15	3	6	0
Solyc08g075860.2.1	8	532	117	372	45	1040	1178	1517	177	655	351	577	3
Solyc08g075870.2.1	7	27	4	19	13	45	36	61	25	397	20	446	0
Solyc08g075880.2.1	0	0	0	0	0	0	0	0	0	0	0	0	0
Solyc08g075890.2.1	12	197	9	178	17	334	159	311	32	349	89	450	14
Solyc08g075910.1.1	3	12	4	6	0	4	10	34	0	24	11	25	0
Solyc08g075920.2.1	0	0	0	0	0	0	0	0	0	0	0	0	0
Solyc08g075930.2.1	0	0	0	0	0	0	0	0	0	0	0	0	0
Solyc08g075940.2.1	0	0	0	0	0	0	0	0	0	0	0	0	0
Solyc08g075950.1.1	0	0	0	0	0	0	0	0	0	0	0	0	0
Solyc08g075960.2.1	2	23	5	19	0	51	27	86	3	93	10	92	2
Solyc08g075970.2.1	7	71	8	11	1	394	40	180	11	68	6	27	0
Solyc08g075990.2.1	2	29	4	34	0	4	35	1	2	2	2	6	6
Solyc08g076000.2.1	1	5	0	8	0	7	0	2	0	0	0	0	0
Solyc08g076010.2.1	0	0	0	0	0	0	0	0	0	0	0	0	0
Solyc08g076020.2.1	7	10	4	6	3	88	70	107	6	39	9	21	0
Solyc08g076030.2.1	15	120	14	92	10	193	101	238	27	219	55	134	5
Solyc08g076050.2.1	1	7	0	14	0	165	2	51	2	12	4	0	0
Solyc08g076060.2.1	8	54	9	24	1	109	91	158	87	143	51	95	4
Solyc08g076080.2.1	0	0	0	0	0	0	0	0	0	0	0	0	0
Solyc08g076090.2.1	11	91	4	55	0	317	153	320	6	119	13	71	0
Solyc08g076100.2.1	5	11	1	15	0	41	18	53	5	23	15	14	0
Solyc08g076110.2.1	0	0	0	0	0	0	0	0	0	0	0	0	0
Solyc08g076120.2.1	0	0	0	0	0	0	0	0	0	0	0	0	0
Solyc08g076130.2.1	1	2	0	6	0	6	2	12	0	1	2	2	0
Solyc08g076140.2.1	15	84	6	43	3	347	94	320	14	199	48	111	3
Solyc08g076150.2.1	2	16	6	13	0	2	3	0	1	9	6	1	0
Solyc08g076160.2.1	9	79	6	100	2	146	108	184	20	146	8	102	0
Solyc08g076170.1.1	2	14	0	1	0	5	13	4	5	6	0	0	2
Solyc08g076180.2.1	4	76	2	23	0	26	35	69	7	99	33	55	2
Solyc08g076190.2.1	0	0	0	0	0	0	0	0	0	0	0	0	0
Solyc08g076200.2.1	9	113	9	81	5	341	182	338	13	354	28	216	0
Solyc08g076210.2.1	3	17	2	6	0	3	7	35	4	12	6	30	0
Solyc08g076220.2.1	15	4505	814	2706	17	281	649	181	3	487	374	1069	14
Solyc08g076230.1.1	0	0	0	0	0	0	0	0	0	0	0	0	0
Solyc08g076250.1.1	0	0	0	0	0	0	0	0	0	0	0	0	0
Solyc08g076290.2.1	8	564	75	352	0	8	14	34	0	7	3	23	0
Solyc08g076300.2.1	2	7	0	10	0	36	5	28	3	16	6	3	0
Solyc08g076310.2.1	6	22	3	22	0	41	25	60	8	64	7	31	0

Solyc08g076320.2.1	28	172	13	147	3	275	107	273	8	330	303	410	12
Solyc08g076340.2.1	0	0	0	0	0	0	0	0	0	0	0	0	0
Solyc08g076350.2.1	0	0	0	0	0	0	0	0	0	0	0	0	0
Solyc08g076360.2.1	12	95	54	48	19	197	841	252	41	77	137	63	12
Solyc08g076370.2.1	3	12	8	0	5	11	13	0	18	0	1	0	0
Solyc08g076390.2.1	3	27	4	26	1	44	10	17	1	23	0	20	3
Solyc08g076400.2.1	0	0	0	0	0	0	0	0	0	0	0	0	0
Solyc08g076410.2.1	2	17	5	5	0	1	28	10	1	2	6	6	0
Solyc08g076420.2.1	20	621	92	473	61	1036	677	1075	254	930	256	1146	50
Solyc08g076430.2.1	0	0	0	0	0	0	0	0	0	0	0	0	0
Solyc08g076440.2.1	3	31	0	46	4	100	5	94	4	216	12	206	0
Solyc08g076450.2.1	8	332	11	275	1	228	94	330	4	181	2	174	0
Solyc08g076460.2.1	1	6	1	1	0	23	1	8	0	8	12	15	1
Solyc08g076470.2.1	5	116	10	49	1	101	45	53	0	32	2	32	0
Solyc08g076480.2.1	12	350	72	187	4	536	266	379	9	514	29	286	7
Solyc08g076490.2.1	2	42	8	26	3	20	24	26	7	34	1	19	0
Solyc08g076510.2.1	3	26	1	24	0	29	22	39	4	40	5	21	3
Solyc08g076520.2.1	4	56	11	16	6	1	31	12	2	44	0	28	0
Solyc08g076530.2.1	1	8	1	4	0	2	3	4	0	16	2	1	0
Solyc08g076540.2.1	6	128	10	82	5	149	118	233	16	303	33	216	1
Solyc08g076570.2.1	0	0	0	0	0	0	0	0	0	0	0	0	0
Solyc08g076580.2.1	1	5	0	2	0	0	7	4	2	5	1	3	0
Solyc08g076590.2.1	2	3	1	1	1	26	28	22	4	1	7	1	0
Solyc08g076610.2.1	0	0	0	0	0	0	0	0	0	0	0	0	0
Solyc08g076620.1.1	0	0	0	0	0	0	0	0	0	0	0	0	0
Solyc08g076630.1.1	0	0	0	0	0	0	0	0	0	0	0	0	0
Solyc08g076650.2.1	15	211	23	236	14	430	186	678	36	702	186	755	10
Solyc08g076660.2.1	10	48	3	50	0	143	37	134	8	75	31	79	0
Solyc08g076670.2.1	6	68	10	23	3	184	128	189	11	69	55	58	1
Solyc08g076680.1.1	2	4	8	2	0	8	3	13	2	8	0	5	0
Solyc08g076690.1.1	3	23	0	4	4	39	33	40	2	37	17	18	0
Solyc08g076720.2.1	6	17	1	1	6	20	4	9	14	142	26	41	14
Solyc08g076730.2.1	6	49	2	42	2	1114	161	646	64	166	47	93	1
Solyc08g076740.2.1	3	40	0	39	2	118	16	36	5	32	7	33	1
Solyc08g076770.2.1	1	16	0	3	0	7	4	5	0	10	6	2	0
Solyc08g076790.2.1	0	0	0	0	0	0	0	0	0	0	0	0	0
Solyc08g076810.2.1	0	0	0	0	0	0	0	0	0	0	0	0	0
Solyc08g076820.2.1	0	0	0	0	0	0	0	0	0	0	0	0	0
Solyc08g076830.1.1	1	3	0	0	0	7	17	8	0	0	2	1	0
Solyc08g076840.2.1	2	29	2	25	6	41	52	55	2	20	0	13	1
Solyc08g076850.2.1	0	0	0	0	0	0	0	0	0	0	0	0	0

Solyc08g076860.2.1	8	400	111	326	43	1103	1144	1153	304	376	112	192	19
Solyc08g076870.1.1	2	21	7	20	1	81	17	30	17	1	0	3	0
Solyc08g076880.2.1	4	448	245	232	4	1053	533	691	4	23	140	10	0
Solyc08g076890.2.1	8	1756	45	1094	0	146	136	167	1	444	89	926	1
Solyc08g076900.2.1	10	593	154	286	4	19	102	14	0	203	174	429	5
Solyc08g076910.2.1	4	10	0	2	0	19	16	19	5	9	4	6	0
Solyc08g076920.2.1	7	74	11	56	5	308	92	270	13	192	20	157	1
Solyc08g076930.1.1	21	438	61	494	32	291	199	219	35	292	79	385	17
Solyc08g076960.1.1	1	32	1	0	0	0	26	17	5	23	0	10	0
Solyc08g076970.2.1	14	1987	31	713	3	1958	257	3573	0	937	0	483	0
Solyc08g076980.2.1	0	0	0	0	0	0	0	0	0	0	0	0	0
Solyc08g076990.2.1	8	69	2	57	3	107	146	223	25	173	34	113	0
Solyc08g077020.1.1	0	0	0	0	0	0	0	0	0	0	0	0	0
Solyc08g077030.2.1	2	18	1	12	2	28	5	46	5	24	15	24	0
Solyc08g077040.2.1	4	22	7	17	7	37	41	29	2	59	14	30	0
Solyc08g077050.2.1	6	54	13	25	1	18	79	20	2	53	15	49	4
Solyc08g077060.2.1	2	25	2	17	1	2	7	8	0	473	36	333	3
Solyc08g077080.1.1	0	0	0	0	0	0	0	0	0	0	0	0	0
Solyc08g077090.2.1	4	9	0	10	0	6	7	7	1	12	8	15	0
Solyc08g077100.2.1	0	0	0	0	0	0	0	0	0	0	0	0	0
Solyc08g077110.2.1	1	3	0	5	0	10	10	28	1	9	0	1	0
Solyc08g077120.2.1	6	36	2	18	0	53	90	73	7	3	7	32	2
Solyc08g077130.2.1	1	7	4	4	0	25	3	26	1	3	0	8	0
Solyc08g077140.1.1	7	56	14	39	0	68	155	109	5	16	18	24	0
Solyc08g077150.2.1	7	23	3	2	0	60	7	45	8	44	29	54	0
Solyc08g077170.2.1	1	11	0	2	0	5	2	3	0	0	2	0	0
Solyc08g077180.2.1	7	145	15	111	5	259	34	104	9	212	58	144	0
Solyc08g077190.1.1	0	0	0	0	0	0	0	0	0	0	0	0	0
Solyc08g077200.2.1	3	18	4	23	2	26	29	55	3	21	7	18	0
Solyc08g077210.2.1	16	921	57	637	3	238	36	188	5	17	17	40	0
Solyc08g077220.2.1	3	153	9	65	8	93	64	126	21	213	42	149	2
Solyc08g077230.2.1	16	46	10	19	10	150	144	127	46	26	150	4	9
Solyc08g077240.2.1	16	503	21	484	31	214	157	441	26	898	166	729	2
Solyc08g077250.2.1	3	24	2	11	0	54	12	53	1	37	1	22	0
Solyc08g077260.2.1	3	80	8	50	2	113	58	68	16	124	12	82	0
Solyc08g077270.2.1	4	13	0	0	0	6	11	10	0	32	10	9	1
Solyc08g077280.1.1	3	24	4	2	0	17	32	11	6	19	3	17	1
Solyc08g077290.1.1	3	26	0	10	1	39	12	28	2	15	11	6	0
Solyc08g077300.2.1	5	42	2	6	0	49	40	65	0	33	14	30	0
Solyc08g077310.2.1	17	125	12	96	5	272	120	210	19	234	62	196	11
Solyc08g077320.1.1	0	0	0	0	0	0	0	0	0	0	0	0	0

Solyc08g077330.2.1	2	12	0	19	0	587	56	232	0	5	11	26	0
Solyc08g077340.2.1	0	0	0	0	0	0	0	0	0	0	0	0	0
Solyc08g077350.2.1	3	46	2	14	8	32	55	53	6	45	4	44	0
Solyc08g077360.2.1	0	0	0	0	0	0	0	0	0	0	0	0	0
Solyc08g077370.2.1	0	0	0	0	0	0	0	0	0	0	0	0	0
Solyc08g077400.1.1	0	0	0	0	0	0	0	0	0	0	0	0	0
Solyc08g077410.2.1	0	0	0	0	0	0	0	0	0	0	0	0	0
Solyc08g077420.2.1	13	61	9	87	0	77	100	125	17	102	11	55	2
Solyc08g077430.2.1	16	140	14	88	3	241	169	368	6	289	56	386	3
Solyc08g077440.2.1	18	400	43	240	10	1425	854	872	109	345	72	337	3
Solyc08g077450.2.1	5	21	4	10	0	64	18	55	3	62	9	33	0
Solyc08g077460.2.1	7	80	11	66	1	1018	382	565	8	383	15	328	6
Solyc08g077470.2.1	2	10	0	1	0	12	11	13	0	24	7	9	0
Solyc08g077480.2.1	5	351	17	225	7	504	67	310	4	451	53	522	5
Solyc08g077490.1.1	2	1	1	7	0	23	20	17	0	1	1	3	0
Solyc08g077500.2.1	33	71	9	32	9	555	405	407	30	197	52	168	6
Solyc08g077510.2.1	10	251	18	61	25	249	433	572	128	187	51	119	1
Solyc08g077520.2.1	0	0	0	0	0	0	0	0	0	0	0	0	0
Solyc08g077530.2.1	14	383	5	193	0	487	443	722	3	625	13	460	12
Solyc08g077540.1.1	0	0	0	0	0	0	0	0	0	0	0	0	0
Solyc08g077550.2.1	1	2	2	5	0	2	4	1	2	5	1	1	0
Solyc08g077560.2.1	4	31	9	25	4	1	4	7	2	8	10	36	2
Solyc08g077630.2.1	0	0	0	0	0	0	0	0	0	0	0	0	0
Solyc08g077680.2.1	6	87	6	43	9	202	12	127	10	45	17	29	2
Solyc08g077690.2.1	4	7	2	0	0	6	43	24	5	46	5	15	0
Solyc08g077700.2.1	0	0	0	0	0	0	0	0	0	0	0	0	0
Solyc08g077710.2.1	1	4	0	9	0	24	8	9	3	52	6	28	0
Solyc08g077720.2.1	2	11	0	3	0	16	7	12	1	20	1	25	0
Solyc08g077730.2.1	7	27	2	36	1	127	100	309	3	113	9	40	2
Solyc08g077740.1.1	0	0	0	0	0	0	0	0	0	0	0	0	0
Solyc08g077750.2.1	8	68	7	45	6	131	69	211	30	125	24	139	0
Solyc08g077770.2.1	2	17	0	11	0	44	13	25	1	14	2	20	1
Solyc08g077780.2.1	5	60	6	45	1	144	123	118	24	78	23	22	3
Solyc08g077790.2.1	7	100	7	54	3	146	40	223	8	167	17	81	1
Solyc08g077800.2.1	3	3	1	1	1	9	9	12	11	6	9	6	0
Solyc08g077830.1.1	3	53	3	20	2	43	19	85	7	131	6	33	0
Solyc08g077840.2.1	7	11	13	24	1	37	156	46	10	46	32	31	3
Solyc08g077850.1.1	1	3	0	0	0	3	0	6	1	0	5	10	0
Solyc08g077860.2.1	0	0	0	0	0	0	0	0	0	0	0	0	0
Solyc08g077870.2.1	0	0	0	0	0	0	0	0	0	0	0	0	0
Solyc08g077880.2.1	7	776	62	581	8	507	133	391	8	296	65	354	10

Solyc08g077890.2.1	4	208	17	123	8	144	134	292	9	139	17	111	0
Solyc08g077900.2.1	8	18	1	8	2	1149	590	979	0	8	0	0	0
Solyc08g077910.2.1	0	0	0	0	0	0	0	0	0	0	0	0	0
Solyc08g077920.2.1	3	34	3	27	5	96	39	140	16	35	24	33	1
Solyc08g077930.2.1	3	50	6	33	1	108	68	163	25	70	8	89	6
Solyc08g077940.1.1	0	0	0	0	0	0	0	0	0	0	0	0	0
Solyc08g077950.1.1	1	2	0	0	0	2	4	1	0	9	2	18	0
Solyc08g077960.2.1	5	30	5	29	1	58	61	66	8	24	6	17	0
Solyc08g077970.2.1	0	0	0	0	0	0	0	0	0	0	0	0	0
Solyc08g077980.2.1	10	329	27	230	2	465	180	409	64	326	157	307	1
Solyc08g077990.2.1	0	0	0	0	0	0	0	0	0	0	0	0	0
Solyc08g078000.2.1	2	22	0	4	1	58	2	33	0	10	0	14	0
Solyc08g078010.2.1	8	366	21	181	2	87	131	140	2	103	23	108	3
Solyc08g078020.1.1	4	1929	73	1090	22	26	44	26	0	24	2	9	0
Solyc08g078030.2.1	0	0	0	0	0	0	0	0	0	0	0	0	0
Solyc08g078040.2.1	3	8	0	5	0	79	11	34	0	3	0	0	0
Solyc08g078050.1.1	2	4	2	4	0	157	114	44	5	4	3	1	0
Solyc08g078060.2.1	1	36	2	19	2	25	0	28	1	17	4	26	0
Solyc08g078070.2.1	2	238	78	177	51	808	618	407	117	113	124	86	7
Solyc08g078080.2.1	0	0	0	0	0	0	0	0	0	0	0	0	0
Solyc08g078090.1.1	6	28	6	35	0	275	16	56	0	25	15	59	3
Solyc08g078100.1.1	5	13	0	18	3	1	30	20	1	52	7	61	0
Solyc08g078130.2.1	9	32	3	11	0	45	32	31	8	69	30	63	0
Solyc08g078140.2.1	1	0	0	0	0	6	9	5	1	0	0	1	0
Solyc08g078150.1.1	3	19	0	17	4	19	16	27	0	38	7	74	0
Solyc08g078160.2.1	2	7	0	0	0	9	0	5	0	0	43	5	0
Solyc08g078180.1.1	0	0	0	0	0	0	0	0	0	0	0	0	0
Solyc08g078190.1.1	2	18	19	19	2	7	22	0	2	12	1	42	2
Solyc08g078210.2.1	7	110	6	45	6	58	71	86	11	63	13	45	3
Solyc08g078220.2.1	3	20	1	11	0	11	15	23	5	10	1	29	0
Solyc08g078230.2.1	18	109	11	73	3	203	225	218	30	381	28	241	4
Solyc08g078240.2.1	2	11	2	2	1	14	4	14	1	26	4	40	0
Solyc08g078250.2.1	7	125	10	70	4	159	115	222	18	139	44	93	6
Solyc08g078270.2.1	8	42	4	30	2	42	58	65	12	56	23	44	2
Solyc08g078280.1.1	0	0	0	0	0	0	0	0	0	0	0	0	0
Solyc08g078290.1.1	6	10	4	22	0	18	43	28	0	32	4	45	0
Solyc08g078300.2.1	2	21	1	18	2	12	3	7	0	26	3	31	0
Solyc08g078310.2.1	4	4	1	14	1	12	9	8	12	5	24	15	2
Solyc08g078320.2.1	16	37	12	55	7	96	180	108	18	116	95	109	13
Solyc08g078340.2.1	10	168	25	91	0	264	137	286	15	74	21	68	0
Solyc08g078370.2.1	5	8	0	7	7	38	17	40	3	39	11	44	0

Solyc08g078380.2.1	0	0	0	0	0	0	0	0	0	0	0	0	0
Solyc08g078390.2.1	12	126	12	75	13	809	222	915	61	202	91	143	4
Solyc08g078400.2.1	0	0	0	0	0	0	0	0	0	0	0	0	0
Solyc08g078430.2.1	9	130	8	132	11	281	125	276	20	219	66	269	2
Solyc08g078440.2.1	10	46	5	18	6	112	44	126	9	161	31	119	6
Solyc08g078460.2.1	0	0	0	0	0	0	0	0	0	0	0	0	0
Solyc08g078470.2.1	0	0	0	0	0	0	0	0	0	0	0	0	0
Solyc08g078480.2.1	2	49	3	9	0	33	19	15	1	34	7	46	0
Solyc08g078490.2.1	0	0	0	0	0	0	0	0	0	0	0	0	0
Solyc08g078500.2.1	0	0	0	0	0	0	0	0	0	0	0	0	0
Solyc08g078510.2.1	10	274	67	146	32	527	118	364	102	175	47	177	14
Solyc08g078520.2.1	1	19	0	6	0	2	0	0	0	5	0	1	0
Solyc08g078530.2.1	72	512	78	512	59	1074	555	1463	104	793	412	648	5
Solyc08g078540.2.1	1	1	0	0	0	2	0	12	0	7	4	7	0
Solyc08g078550.1.1	1	11	1	23	0	311	23	52	27	43	137	3	0
Solyc08g078560.2.1	0	0	0	0	0	0	0	0	0	0	0	0	0
Solyc08g078570.1.1	0	0	0	0	0	0	0	0	0	0	0	0	0
Solyc08g078580.1.1	0	0	0	0	0	0	0	0	0	0	0	0	0
Solyc08g078600.2.1	6	9	3	8	1	24	25	21	8	23	6	28	4
Solyc08g078610.2.1	5	19	0	36	4	56	22	81	4	66	4	43	0
Solyc08g078630.2.1	0	0	0	0	0	0	0	0	0	0	0	0	0
Solyc08g078650.2.1	0	0	0	0	0	0	0	0	0	0	0	0	0
Solyc08g078660.2.1	0	0	0	0	0	0	0	0	0	0	0	0	0
Solyc08g078670.2.1	8	295	6	176	0	134	35	62	3	350	21	230	0
Solyc08g078680.2.1	0	0	0	0	0	0	0	0	0	0	0	0	0
Solyc08g078690.2.1	0	0	0	0	0	0	0	0	0	0	0	0	0
Solyc08g078700.2.1	4	2	0	1	6	10	8	3	3	7	134	27	0
Solyc08g078710.1.1	0	0	0	0	0	0	0	0	0	0	0	0	0
Solyc08g078720.2.1	0	0	0	0	0	0	0	0	0	0	0	0	0
Solyc08g078730.2.1	0	0	0	0	0	0	0	0	0	0	0	0	0
Solyc08g078740.1.1	3	9	3	2	0	0	13	6	1	23	0	9	0
Solyc08g078750.2.1	6	19	1	14	2	60	33	48	4	29	9	40	2
Solyc08g078760.1.1	3	4	0	14	0	46	15	74	0	5	0	4	0
Solyc08g078770.1.1	0	0	0	0	0	0	0	0	0	0	0	0	0
Solyc08g078780.2.1	1	20	0	9	3	19	5	7	0	8	0	4	0
Solyc08g078790.1.1	0	0	0	0	0	0	0	0	0	0	0	0	0
Solyc08g078800.1.1	3	20	0	13	0	25	15	36	5	30	7	45	0
Solyc08g078810.1.1	1	3	0	0	0	2	4	7	0	1	1	10	0
Solyc08g078820.2.1	6	33	5	30	0	134	153	168	11	73	16	66	0
Solyc08g078840.2.1	0	0	0	0	0	0	0	0	0	0	0	0	0
Solyc08g078850.2.1	0	0	0	0	0	0	0	0	0	0	0	0	0

Solyc08g078860.2.1	2	44	0	21	1	110	26	80	4	246	12	120	0
Solyc08g078870.1.1	2	75	1	12	0	18	57	79	0	1	6	0	0
Solyc08g078880.2.1	0	0	0	0	0	0	0	0	0	0	0	0	0
Solyc08g078890.1.1	0	0	0	0	0	0	0	0	0	0	0	0	0
Solyc08g078900.1.1	0	0	0	0	0	0	0	0	0	0	0	0	0
Solyc08g078920.1.1	0	0	0	0	0	0	0	0	0	0	0	0	0
Solyc08g078950.2.1	11	201	8	158	0	160	54	152	9	88	1	69	0
Solyc08g078960.2.1	7	31	5	12	4	53	23	48	28	13	19	5	0
Solyc08g078970.1.1	0	0	0	0	0	0	0	0	0	0	0	0	0
Solyc08g078990.1.1	2	18	0	0	0	11	11	10	1	4	1	7	0
Solyc08g079000.2.1	0	0	0	0	0	0	0	0	0	0	0	0	0
Solyc08g079010.1.1	0	0	0	0	0	0	0	0	0	0	0	0	0
Solyc08g079020.2.1	2	2	0	0	0	8	4	6	0	28	15	23	0
Solyc08g079030.2.1	0	0	0	0	0	0	0	0	0	0	0	0	0
Solyc08g079040.1.1	3	22	4	5	2	37	19	23	6	64	9	66	0
Solyc08g079060.2.1	0	0	0	0	0	0	0	0	0	0	0	0	0
Solyc08g079070.2.1	12	133	12	87	19	245	327	404	20	379	50	340	12
Solyc08g079080.2.1	0	0	0	0	0	0	0	0	0	0	0	0	0
Solyc08g079090.2.1	6	167	18	40	6	37	1	17	0	136	49	117	4
Solyc08g079100.2.1	0	0	0	0	0	0	0	0	0	0	0	0	0
Solyc08g079110.2.1	6	59	15	23	0	22	60	14	1	42	31	112	0
Solyc08g079120.1.1	0	0	0	0	0	0	0	0	0	0	0	0	0
Solyc08g079140.1.1	0	0	0	0	0	0	0	0	0	0	0	0	0
Solyc08g079150.1.1	0	0	0	0	0	0	0	0	0	0	0	0	0
Solyc08g079160.2.1	0	0	0	0	0	0	0	0	0	0	0	0	0
Solyc08g079170.2.1	9	157	19	98	21	173	143	259	22	418	131	243	4
Solyc08g079180.2.1	9	277	28	248	1	140	72	173	6	135	23	205	0
Solyc08g079190.1.1	0	0	0	0	0	0	0	0	0	0	0	0	0
Solyc08g079200.1.1	0	0	0	0	0	0	0	0	0	0	0	0	0
Solyc08g079240.2.1	4	111	10	93	0	6	49	61	1	39	7	58	0
Solyc08g079250.2.1	6	32	2	17	0	158	41	108	12	298	28	250	10
Solyc08g079260.2.1	14	329	41	304	29	850	659	1169	72	1105	239	931	14
Solyc08g079270.2.1	0	0	0	0	0	0	0	0	0	0	0	0	0
Solyc08g079280.2.1	4	60	7	52	0	21	24	32	2	80	6	47	0
Solyc08g079310.2.1	0	0	0	0	0	0	0	0	0	0	0	0	0
Solyc08g079330.1.1	0	0	0	0	0	0	0	0	0	0	0	0	0
Solyc08g079350.1.1	0	0	0	0	0	0	0	0	0	0	0	0	0
Solyc08g079370.1.1	0	0	0	0	0	0	0	0	0	0	0	0	0
Solyc08g079420.2.1	11	72	14	74	0	1211	49	603	8	3	0	19	0
Solyc08g079430.2.1	15	222	713	423	422	4632	6355	7979	644	769	423	615	59
Solyc08g079440.1.1	7	59	7	31	3	104	43	82	21	38	18	24	0

Solyc08g079450.2.1	7	96	3	55	3	147	156	237	18	135	39	89	4
Solyc08g079460.2.1	10	43	11	17	15	45	52	28	44	25	17	27	0
Solyc08g079470.2.1	5	9	1	5	0	86	28	49	20	66	18	52	0
Solyc08g079480.2.1	0	0	0	0	0	0	0	0	0	0	0	0	0
Solyc08g079490.2.1	1	3	0	0	0	0	0	15	0	1	0	2	0
Solyc08g079500.2.1	13	45	9	51	0	189	51	181	14	175	31	80	2
Solyc08g079510.2.1	6	146	10	66	12	2278	81	338	41	50	111	9	5
Solyc08g079520.2.1	6	19	1	6	2	70	30	81	17	31	10	38	0
Solyc08g079530.2.1	0	0	0	0	0	0	0	0	0	0	0	0	0
Solyc08g079540.2.1	0	0	0	0	0	0	0	0	0	0	0	0	0
Solyc08g079550.1.1	6	33	4	26	4	0	9	16	10	61	22	47	0
Solyc08g079570.2.1	1	12	0	2	6	17	6	27	1	3	0	6	0
Solyc08g079590.1.1	0	0	0	0	0	0	0	0	0	0	0	0	0
Solyc08g079600.2.1	1	41	0	19	0	106	3	46	0	18	2	0	0
Solyc08g079610.2.1	1	12	3	3	0	18	5	14	1	39	3	45	0
Solyc08g079620.2.1	6	253	12	196	0	197	137	363	6	324	23	271	2
Solyc08g079630.2.1	2	16	1	3	0	6	4	10	1	13	4	3	0
Solyc08g079640.1.1	0	0	0	0	0	0	0	0	0	0	0	0	0
Solyc08g079650.2.1	11	121	13	91	8	155	88	88	56	99	106	92	1
Solyc08g079660.2.1	5	120	16	58	1	120	84	116	15	86	12	64	2
Solyc08g079670.2.1	4	44	6	13	2	33	37	56	5	16	7	23	0
Solyc08g079680.2.1	1	3	0	4	0	0	4	1	0	1	0	0	0
Solyc08g079690.2.1	1	4	1	11	5	2	3	10	0	15	10	34	1
Solyc08g079700.1.1	4	392	21	219	12	50	13	30	2	131	5	190	0
Solyc08g079710.2.1	2	8	2	6	0	0	15	2	3	4	0	0	0
Solyc08g079730.1.1	1	1	0	0	0	4	2	6	0	6	2	1	0
Solyc08g079740.2.1	0	0	0	0	0	0	0	0	0	0	0	0	0
Solyc08g079750.2.1	7	49	1	41	0	34	21	36	0	11	1	12	0
Solyc08g079760.2.1	0	0	0	0	0	0	0	0	0	0	0	0	0
Solyc08g079770.2.1	0	0	0	0	0	0	0	0	0	0	0	0	0
Solyc08g079790.1.1	2	19	1	4	5	0	3	2	0	15	2	8	0
Solyc08g079800.2.1	0	0	0	0	0	0	0	0	0	0	0	0	0
Solyc08g079810.2.1	8	19	8	19	0	27	40	73	10	31	1	15	2
Solyc08g079820.2.1	9	16	6	19	10	33	80	41	4	55	20	75	1
Solyc08g079830.2.1	19	135	22	21	7	180	80	118	20	168	53	198	1
Solyc08g079840.1.1	0	0	0	0	0	0	0	0	0	0	0	0	0
Solyc08g079850.1.1	0	0	0	0	0	0	0	0	0	0	0	0	0
Solyc08g079860.1.1	0	0	0	0	0	0	0	0	0	0	0	0	0
Solyc08g079870.1.1	1	66	3	96	0	41	0	94	0	20	0	0	0
Solyc08g079880.1.1	0	0	0	0	0	0	0	0	0	0	0	0	0
Solyc08g079890.1.1	12	109	1	59	0	66	198	110	0	9	1	13	0

Solyc08g079900.1.1	0	0	0	0	0	0	0	0	0	0	0	0	0
Solyc08g079910.1.1	0	0	0	0	0	0	0	0	0	0	0	0	0
Solyc08g079920.1.1	0	0	0	0	0	0	0	0	0	0	0	0	0
Solyc08g079930.1.1	0	0	0	0	0	0	0	0	0	0	0	0	0
Solyc08g080000.2.1	1	12	2	24	0	8	12	9	0	32	1	3	2
Solyc08g080010.1.1	0	0	0	0	0	0	0	0	0	0	0	0	0
Solyc08g080030.2.1	3	7	2	7	2	16	13	12	0	3	4	22	0
Solyc08g080040.2.1	2	56	0	19	1	6	9	7	0	3	1	2	0
Solyc08g080050.2.1	3	223	47	187	0	16	38	42	0	10	8	29	0
Solyc08g080080.2.1	2	17	1	12	1	13	4	12	5	9	7	11	0
Solyc08g080090.2.1	7	94	3	82	0	538	122	654	29	232	36	69	0
Solyc08g080100.2.1	0	0	0	0	0	0	0	0	0	0	0	0	0
Solyc08g080110.2.1	16	91	6	54	11	204	133	230	64	153	27	97	2
Solyc08g080120.2.1	6	61	4	37	4	84	35	82	19	79	15	77	0
Solyc08g080130.2.1	8	37	7	32	4	89	87	80	33	72	14	29	0
Solyc08g080140.2.1	11	578	60	386	24	1120	378	506	72	1202	225	985	8
Solyc08g080150.1.1	1	4	2	1	0	12	7	1	0	17	4	20	0
Solyc08g080170.2.1	9	1234	56	256	24	279	55	314	8	398	213	255	25
Solyc08g080180.1.1	0	0	0	0	0	0	0	0	0	0	0	0	0
Solyc08g080190.2.1	8	124	7	9	33	90	28	19	84	88	28	25	16
Solyc08g080200.2.1	5	86	2	48	2	79	26	95	10	146	2	81	0
Solyc08g080210.2.1	0	0	0	0	0	0	0	0	0	0	0	0	0
Solyc08g080220.1.1	0	0	0	0	0	0	0	0	0	0	0	0	0
Solyc08g080240.2.1	0	0	0	0	0	0	0	0	0	0	0	0	0
Solyc08g080250.2.1	9	48	6	33	0	24	35	69	4	41	9	51	1
Solyc08g080260.2.1	3	16	0	12	0	29	16	42	3	19	4	20	0
Solyc08g080270.2.1	0	0	0	0	0	0	0	0	0	0	0	0	0
Solyc08g080280.2.1	0	0	0	0	0	0	0	0	0	0	0	0	0
Solyc08g080290.2.1	0	0	0	0	0	0	0	0	0	0	0	0	0
Solyc08g080310.1.1	0	0	0	0	0	0	0	0	0	0	0	0	0
Solyc08g080320.2.1	6	62	8	46	0	13	45	34	0	3	0	0	0
Solyc08g080340.2.1	3	30	5	11	6	11	7	20	10	14	7	5	0
Solyc08g080360.2.1	7	123	8	72	5	43	31	56	1	133	17	60	0
Solyc08g080370.2.1	22	408	26	216	2	695	317	629	26	788	70	674	8
Solyc08g080380.2.1	12	54	33	87	5	79	219	118	10	93	20	70	11
Solyc08g080390.2.1	2	4	2	4	1	14	16	17	9	9	0	6	0
Solyc08g080400.1.1	1	1	0	0	0	3	8	1	5	0	8	0	0
Solyc08g080410.2.1	8	71	6	59	2	294	75	281	29	87	40	111	0
Solyc08g080420.2.1	10	44	5	40	2	35	96	94	14	87	31	112	5
Solyc08g080470.2.1	3	11	0	1	0	0	0	11	2	14	12	11	0
Solyc08g080480.2.1	0	0	0	0	0	0	0	0	0	0	0	0	0

Solyc08g080490.2.1	11	0	0	0	0	0	6	0	0	40	1174	57	10
Solyc08g080500.2.1	8	91	9	27	7	161	49	172	49	131	89	131	8
Solyc08g080510.2.1	3	5	0	0	1	8	10	15	0	13	16	12	0
Solyc08g080520.2.1	6	2	0	0	0	14	19	15	2	12	8	8	2
Solyc08g080540.2.1	2	28	1	21	2	24	16	9	2	2	3	18	0
Solyc08g080550.1.1	11	51	9	33	1	74	77	147	3	61	15	63	0
Solyc08g080560.1.1	0	0	0	0	0	0	0	0	0	0	0	0	0
Solyc08g080570.2.1	16	298	19	125	6	273	873	1009	42	1032	77	480	2
Solyc08g080580.2.1	0	0	0	0	0	0	0	0	0	0	0	0	0
Solyc08g080590.2.1	0	0	0	0	0	0	0	0	0	0	0	0	0
Solyc08g080620.1.1	2	38	0	16	8	1	14	3	2	0	0	0	0
Solyc08g080630.2.1	0	0	0	0	0	0	0	0	0	0	0	0	0
Solyc08g080640.1.1	4	44	1	11	11	692	150	829	28	12	7	5	0
Solyc08g080650.1.1	5	324	2	144	1	565	26	562	7	113	28	259	0
Solyc08g080660.1.1	0	0	0	0	0	0	0	0	0	0	0	0	0
Solyc08g080670.1.1	0	0	0	0	0	0	0	0	0	0	0	0	0
Solyc08g080680.2.1	0	0	0	0	0	0	0	0	0	0	0	0	0
Solyc08g080690.2.1	25	90	17	89	10	210	213	292	10	228	24	157	4
Solyc08g080720.2.1	3	22	0	5	1	5	2	7	0	62	13	23	0
Solyc08g080730.2.1	0	0	0	0	0	0	0	0	0	0	0	0	0
Solyc08g080750.2.1	0	0	0	0	0	0	0	0	0	0	0	0	0
Solyc08g080770.2.1	1	4	0	5	0	15	5	5	0	0	0	0	0
Solyc08g080780.2.1	4	49	1	9	5	13	2	5	1	107	15	112	0
Solyc08g080830.2.1	7	72	4	64	7	66	84	57	2	28	47	27	0
Solyc08g080850.2.1	7	20	2	5	5	51	29	48	5	38	19	16	0
Solyc08g080860.2.1	2	4	4	5	2	19	6	3	1	12	3	1	3
Solyc08g080870.2.1	0	0	0	0	0	0	0	0	0	0	0	0	0
Solyc08g080890.2.1	0	0	0	0	0	0	0	0	0	0	0	0	0
Solyc08g080900.2.1	5	81	6	47	0	206	113	236	9	211	16	131	1
Solyc08g080910.2.1	3	6	2	2	0	32	8	53	3	25	11	20	0
Solyc08g080920.2.1	3	43	2	10	2	41	35	52	8	54	31	31	4
Solyc08g080940.2.1	12	411	15	143	10	2469	391	1272	136	559	198	462	2
Solyc08g080960.2.1	0	0	0	0	0	0	0	0	0	0	0	0	0
Solyc08g080980.2.1	0	0	0	0	0	0	0	0	0	0	0	0	0
Solyc08g081000.2.1	9	38	5	35	1	110	107	44	11	81	25	65	1
Solyc08g081010.2.1	18	191	13	183	0	662	178	717	43	318	165	303	0
Solyc08g081030.1.1	12	322	60	162	13	508	344	497	39	135	50	107	3
Solyc08g081040.2.1	0	0	0	0	0	0	0	0	0	0	0	0	0
Solyc08g081050.2.1	8	32	6	18	0	51	36	89	9	37	4	15	0
Solyc08g081060.2.1	0	0	0	0	0	0	0	0	0	0	0	0	0
Solyc08g081070.2.1	2	1	0	3	0	1	10	5	0	9	1	6	6

Solyc08g081080.2.1	4	14	7	12	0	62	29	34	0	33	4	32	0
Solyc08g081100.2.1	2	9	5	11	2	16	23	20	4	22	8	10	1
Solyc08g081110.2.1	0	0	0	0	0	0	0	0	0	0	0	0	0
Solyc08g081120.2.1	1	5	0	0	0	2	3	6	0	2	0	7	0
Solyc08g081130.2.1	1	2	0	3	0	2	4	4	2	5	0	17	0
Solyc08g081140.2.1	3	26	1	8	0	57	17	36	1	49	0	17	1
Solyc08g081150.2.1	8	15	4	7	4	46	38	50	11	86	31	70	3
Solyc08g081160.2.1	0	0	0	0	0	0	0	0	0	0	0	0	0
Solyc08g081170.2.1	5	23	3	15	0	37	35	116	0	35	3	26	0
Solyc08g081180.2.1	12	196	26	174	1	658	229	686	62	268	25	210	2
Solyc08g081190.2.1	9	5268	122	2792	70	4284	304	4046	85	3425	706	3203	20
Solyc08g081200.2.1	8	57	9	82	2	254	147	274	10	138	12	65	0
Solyc08g081210.2.1	2	5	0	10	0	4	4	6	1	5	0	8	0
Solyc08g081220.1.1	17	138	12	2	43	27	63	25	171	1518	106	35	114
Solyc08g081230.1.1	15	257	124	308	14	83	36	38	9	40	15	75	1
Solyc08g081240.2.1	5	60	3	37	9	258	30	84	8	39	12	37	0
Solyc08g081250.2.1	22	922	161	625	24	1107	1284	1255	143	1063	286	985	15
Solyc08g081260.1.1	0	0	0	0	0	0	0	0	0	0	0	0	0
Solyc08g081270.2.1	1	3	0	10	0	11	0	1	0	8	2	1	0
Solyc08g081280.2.1	3	19	0	6	0	51	9	38	5	24	6	16	0
Solyc08g081290.2.1	4	26	0	9	5	34	9	47	5	38	12	35	1
Solyc08g081300.2.1	4	9	1	4	0	35	20	87	5	14	4	11	0
Solyc08g081310.2.1	7	129	10	60	2	33	28	36	1	204	23	113	1
Solyc08g081320.2.1	12	766	26	295	24	673	216	1075	74	1089	72	756	20
Solyc08g081330.2.1	7	91	8	57	1	198	75	298	18	424	24	232	6
Solyc08g081340.2.1	9	50	9	21	2	53	62	110	5	105	18	58	1
Solyc08g081350.2.1	0	0	0	0	0	0	0	0	0	0	0	0	0
Solyc08g081360.2.1	0	0	0	0	0	0	0	0	0	0	0	0	0
Solyc08g081370.1.1	1	9	3	3	0	60	6	40	0	15	0	0	0
Solyc08g081380.2.1	5	24	0	7	0	19	21	64	2	79	18	49	0
Solyc08g081390.2.1	1	10	1	0	0	0	3	4	1	0	2	0	0
Solyc08g081400.2.1	10	198	20	135	24	417	134	389	26	16	10	11	15
Solyc08g081410.2.1	15	112	16	105	9	257	247	244	23	354	41	203	9
Solyc08g081420.2.1	9	31	3	17	1	119	24	64	8	186	12	76	0
Solyc08g081430.2.1	0	0	0	0	0	0	0	0	0	0	0	0	0
Solyc08g081450.2.1	0	0	0	0	0	0	0	0	0	0	0	0	0
Solyc08g081470.2.1	1	31	0	2	0	20	2	4	0	0	0	0	0
Solyc08g081480.2.1	9	116	14	56	6	56	121	92	1	24	11	7	0
Solyc08g081490.2.1	4	9	2	1	0	26	12	25	0	9	2	2	0
Solyc08g081520.1.1	1	2	0	17	0	5	6	4	0	2	0	0	0
Solyc08g081530.2.1	9	44	11	25	4	131	103	115	17	127	26	100	0

Solyc08g081540.2.1	2	4	1	2	0	9	8	1	18	0	6	10	1
Solyc08g081550.2.1	0	0	0	0	0	0	0	0	0	0	0	0	0
Solyc08g081570.2.1	7	162	4	116	0	249	61	292	6	79	7	83	3
Solyc08g081580.2.1	13	113	27	63	6	167	175	320	28	273	42	243	3
Solyc08g081610.2.1	0	0	0	0	0	0	0	0	0	0	0	0	0
Solyc08g081620.2.1	6	73	7	20	0	48	44	96	0	7	314	5	2
Solyc08g081640.2.1	7	15	1	13	8	33	20	38	1	81	9	73	0
Solyc08g081650.1.1	0	0	0	0	0	0	0	0	0	0	0	0	0
Solyc08g081670.1.1	1	0	0	3	0	12	0	11	0	3	4	1	0
Solyc08g081680.1.1	0	0	0	0	0	0	0	0	0	0	0	0	0
Solyc08g081690.2.1	4	15	1	16	0	25	4	21	0	2	7	3	0
Solyc08g081700.1.1	1	6	5	6	0	9	0	2	2	0	0	0	0
Solyc08g081710.2.1	0	0	0	0	0	0	0	0	0	0	0	0	0
Solyc08g081730.2.1	15	598	98	390	52	985	538	1104	441	991	409	793	29
Solyc08g081740.2.1	0	0	0	0	0	0	0	0	0	0	0	0	0
Solyc08g081750.2.1	9	42	14	23	0	62	37	70	11	14	4	22	0
Solyc08g081760.1.1	30	368	41	304	18	1182	544	1481	137	1418	327	1526	23
Solyc08g081770.2.1	11	93	6	22	2	32	44	42	0	24	1	31	0
Solyc08g081780.1.1	0	0	0	0	0	0	0	0	0	0	0	0	0
Solyc08g081790.1.1	0	0	0	0	0	0	0	0	0	0	0	0	0
Solyc08g081810.2.1	0	0	0	0	0	0	0	0	0	0	0	0	0
Solyc08g081820.2.1	0	0	0	0	0	0	0	0	0	0	0	0	0
Solyc08g081830.2.1	4	6	0	1	5	31	18	38	1	2	7	4	0
Solyc08g081840.2.1	3	6	0	2	0	17	9	10	2	10	7	3	2
Solyc08g081850.1.1	0	0	0	0	0	0	0	0	0	0	0	0	0
Solyc08g081890.2.1	12	13	0	9	0	33	9	52	0	113	90	156	0
Solyc08g081900.2.1	15	155	16	84	14	350	176	333	11	213	75	138	5
Solyc08g081910.2.1	2	20	5	11	2	67	57	75	7	42	25	39	3
Solyc08g081920.2.1	6	23	8	25	0	107	61	80	6	120	16	106	0
Solyc08g081930.2.1	3	105	17	30	5	54	112	37	20	89	4	34	5
Solyc08g081940.2.1	9	84	5	28	1	11	55	61	0	131	7	90	2
Solyc08g081950.2.1	3	345	22	265	18	733	198	587	25	1671	174	1543	21
Solyc08g081960.1.1	0	0	0	0	0	0	0	0	0	0	0	0	0
Solyc08g081980.2.1	0	0	0	0	0	0	0	0	0	0	0	0	0
Solyc08g081990.2.1	7	24	5	10	0	37	39	81	13	32	14	38	5
Solyc08g082000.2.1	7	16	3	4	0	8	33	20	6	30	16	16	0
Solyc08g082010.2.1	25	190	19	120	7	91	119	163	14	102	82	107	5
Solyc08g082020.2.1	5	22	5	19	1	33	45	46	11	27	7	40	0
Solyc08g082040.2.1	5	28	3	1	1	48	45	43	28	35	3	31	1
Solyc08g082050.1.1	0	0	0	0	0	0	0	0	0	0	0	0	0
Solyc08g082060.2.1	7	60	4	21	1	62	28	73	8	63	9	63	1

Solyc08g082070.2.1	0	0	0	0	0	0	0	0	0	0	0	0	0
Solyc08g082080.2.1	4	7	2	6	0	91	18	141	4	67	14	14	7
Solyc08g082090.1.1	3	203	13	91	2	22	8	3	4	52	5	168	12
Solyc08g082100.2.1	2	12	0	2	0	13	13	19	2	19	2	18	0
Solyc08g082110.2.1	1	119	4	128	0	6	2	13	0	5	1	57	1
Solyc08g082120.2.1	9	2523	188	900	1	433	836	1130	0	222	22	194	0
Solyc08g082130.2.1	1	3	3	5	0	1	2	3	0	1	0	3	0
Solyc08g082160.2.1	0	0	0	0	0	0	0	0	0	0	0	0	0
Solyc08g082170.2.1	8	837	58	646	75	103	238	381	16	2338	98	2162	18
Solyc08g082180.2.1	2	18	1	28	1	22	18	67	0	97	8	86	2
Solyc08g082190.2.1	5	3536	373	2486	3	4099	2294	5670	1	731	169	538	0
Solyc08g082200.2.1	0	0	0	0	0	0	0	0	0	0	0	0	0
Solyc08g082210.2.1	4	49	4	5	2	67	57	36	75	3	2	10	0
Solyc08g082230.2.1	6	16	2	21	1	49	17	63	1	30	27	28	0
Solyc08g082240.1.1	2	0	0	3	1	12	3	5	0	3	1	3	0
Solyc08g082250.2.1	10	120	11	104	15	38	29	89	12	651	133	441	6
Solyc08g082270.2.1	3	5	2	2	0	15	5	5	0	5	7	6	1
Solyc08g082280.2.1	17	613	65	388	32	698	468	1077	162	201	127	148	9
Solyc08g082290.2.1	2	5	0	6	0	14	12	26	3	23	4	10	0
Solyc08g082300.2.1	0	0	0	0	0	0	0	0	0	0	0	0	0
Solyc08g082310.2.1	5	21	3	15	0	53	11	23	5	40	16	35	0
Solyc08g082320.2.1	6	13	3	25	0	20	22	31	4	39	17	20	3
Solyc08g082340.2.1	5	38	2	24	0	82	46	72	6	171	23	62	4
Solyc08g082350.2.1	0	0	0	0	0	0	0	0	0	0	0	0	0
Solyc08g082370.1.1	1	10	1	3	1	15	15	4	26	1	2	0	0
Solyc08g082380.1.1	0	0	0	0	0	0	0	0	0	0	0	0	0
Solyc08g082390.2.1	2	10	0	2	0	24	22	9	9	18	12	5	0
Solyc08g082400.1.1	5	337	32	202	3	74	51	89	2	225	17	149	0
Solyc08g082410.2.1	35	155	20	119	4	322	309	380	75	213	125	173	7
Solyc08g082420.2.1	0	0	0	0	0	0	0	0	0	0	0	0	0
Solyc08g082430.2.1	7	307	28	141	34	309	247	366	36	378	99	370	22
Solyc08g082440.2.1	6	44	5	7	1	170	156	214	88	64	33	31	0
Solyc08g082450.2.1	2	11	1	1	3	22	6	15	2	10	8	17	0
Solyc08g082460.2.1	0	0	0	0	0	0	0	0	0	0	0	0	0
Solyc08g082470.1.1	0	0	0	0	0	0	0	0	0	0	0	0	0
Solyc08g082480.2.1	3	3	0	0	0	2	14	6	2	13	0	9	0
Solyc08g082490.1.1	0	0	0	0	0	0	0	0	0	0	0	0	0
Solyc08g082530.2.1	12	90	13	49	5	213	175	257	7	99	16	90	2
Solyc08g082540.2.1	3	13	0	16	0	35	16	41	3	10	11	25	0
Solyc08g082560.2.1	3	11	0	16	0	29	18	23	5	32	25	54	0
Solyc08g082570.2.1	3	53	0	31	2	3	30	2	0	10	16	33	0

Solyc08g082580.2.1	1	10	0	9	1	23	3	10	1	22	9	19	0
Solyc08g082590.2.1	8	224	49	164	2	40	6	34	4	2	9	15	0
Solyc08g082610.2.1	9	611	20	479	13	914	139	949	126	222	62	377	8
Solyc08g082620.2.1	1	1	1	0	0	4	0	8	1	14	4	6	0
Solyc08g082630.2.1	0	0	0	0	0	0	0	0	0	0	0	0	0
Solyc08g082640.2.1	0	0	0	0	0	0	0	0	0	0	0	0	0
Solyc08g082650.2.1	0	0	0	0	0	0	0	0	0	0	0	0	0
Solyc08g082660.2.1	0	0	0	0	0	0	0	0	0	0	0	0	0
Solyc08g082670.2.1	0	0	0	0	0	0	0	0	0	0	0	0	0
Solyc08g082680.2.1	2	14	0	3	0	16	16	43	0	5	0	12	0
Solyc08g082690.2.1	3	157	5	90	0	43	27	114	0	55	1	53	2
Solyc08g082700.2.1	5	136	15	73	11	448	116	349	68	327	83	250	11
Solyc08g082710.2.1	1	3	0	0	0	2	13	2	0	8	1	7	0
Solyc08g082720.2.1	0	0	0	0	0	0	0	0	0	0	0	0	0
Solyc08g082730.2.1	4	38	1	28	0	42	26	68	2	62	7	47	0
Solyc08g082740.2.1	2	53	2	14	2	86	12	83	4	54	3	28	0
Solyc08g082750.2.1	5	54	8	34	1	63	29	84	3	43	21	18	0
Solyc08g082760.2.1	4	232	36	109	18	202	178	125	32	183	31	181	4
Solyc08g082770.2.1	0	0	0	0	0	0	0	0	0	0	0	0	0
Solyc08g082790.2.1	0	0	0	0	0	0	0	0	0	0	0	0	0
Solyc08g082800.2.1	0	0	0	0	0	0	0	0	0	0	0	0	0
Solyc08g082810.2.1	3	19	2	6	0	25	19	18	4	17	4	13	0
Solyc08g082820.2.1	13	269	15	103	10	668	248	941	81	376	346	294	4
Solyc08g082830.2.1	7	88	7	88	0	213	44	256	3	79	12	45	4
Solyc08g082840.1.1	0	0	0	0	0	0	0	0	0	0	0	0	0
Solyc08g082850.2.1	11	200	24	192	8	449	208	594	31	558	106	484	8
Solyc08g082860.2.1	1	0	1	3	0	4	12	12	1	12	1	3	0
Solyc08g082870.2.1	1	2	0	3	0	9	9	14	0	4	1	7	0
Solyc08g082880.2.1	6	13	2	19	0	38	26	54	8	32	16	63	2
Solyc08g082890.2.1	0	0	0	0	0	0	0	0	0	0	0	0	0
Solyc08g082900.2.1	1	0	0	2	0	5	6	4	5	1	0	3	0
Solyc08g082910.1.1	0	0	0	0	0	0	0	0	0	0	0	0	0
Solyc08g082920.2.1	0	0	0	0	0	0	0	0	0	0	0	0	0
Solyc08g082930.1.1	0	0	0	0	0	0	0	0	0	0	0	0	0
Solyc08g082940.2.1	0	0	0	0	0	0	0	0	0	0	0	0	0
Solyc08g082960.2.1	0	0	0	0	0	0	0	0	0	0	0	0	0
Solyc08g082970.2.1	11	102	16	72	4	161	151	279	31	267	47	132	1
Solyc08g082980.2.1	8	5	16	1	3	26	328	18	37	74	51	26	8
Solyc08g082990.2.1	11	232	8	95	5	85	6	100	3	180	60	10	1
Solyc08g083010.2.1	4	120	19	53	1	134	326	255	6	43	12	28	0
Solyc08g083020.2.1	0	0	0	0	0	0	0	0	0	0	0	0	0

Solyc08g083030.2.1	5	15	2	9	0	31	31	44	3	57	7	42	3
Solyc08g083040.2.1	4	18	3	17	0	44	8	53	3	28	7	28	0
Solyc08g083050.1.1	6	324	27	188	9	52	4	11	1	41	4	85	3
Solyc08g083060.2.1	14	202	25	138	0	33	102	156	0	201	25	267	4
Solyc08g083070.2.1	7	47	7	30	2	74	57	112	8	143	16	69	0
Solyc08g083080.2.1	0	0	0	0	0	0	0	0	0	0	0	0	0
Solyc08g083090.1.1	0	0	0	0	0	0	0	0	0	0	0	0	0
Solyc08g083100.1.1	0	0	0	0	0	0	0	0	0	0	0	0	0
Solyc08g083110.2.1	21	50	10	18	1	722	1620	949	15	356	96	245	1
Solyc08g083120.2.1	2	133	17	58	12	522	232	302	55	178	74	144	2
Solyc08g083130.2.1	6	9	4	3	0	476	263	491	38	22	11	3	0
Solyc08g083140.2.1	3	14	0	3	0	5	21	18	3	19	2	6	0
Solyc08g083150.1.1	0	0	0	0	0	0	0	0	0	0	0	0	0
Solyc08g083190.2.1	16	96	1	14	3	3	24	2	87	10	93	1	14
Solyc08g083200.2.1	1	1	0	0	0	4	8	1	5	10	0	5	0
Solyc08g083210.2.1	0	0	0	0	0	0	0	0	0	0	0	0	0
Solyc08g083220.1.1	0	0	0	0	0	0	0	0	0	0	0	0	0
Solyc08g083230.1.1	0	0	0	0	0	0	0	0	0	0	0	0	0
Solyc08g083240.2.1	0	0	0	0	0	0	0	0	0	0	0	0	0
Solyc08g083250.2.1	7	41	6	26	0	44	43	57	13	75	22	41	0
Solyc08g083260.2.1	2	18	2	7	0	11	8	12	0	9	1	4	1
Solyc08g083270.1.1	0	0	0	0	0	0	0	0	0	0	0	0	0
Solyc08g083280.2.1	1	9	0	3	3	0	6	2	0	2	0	3	0
Solyc08g083290.2.1	10	28	11	27	8	50	49	69	5	57	23	46	0
Solyc08g083300.2.1	13	49	6	62	4	139	130	171	9	107	26	121	4
Solyc08g083310.2.1	3	17	0	3	0	12	20	9	3	27	12	12	2
Solyc08g083320.2.1	8	1172	191	647	17	1119	320	516	7	680	175	681	16
Solyc08g083330.2.1	10	453	168	291	14	481	627	526	31	318	123	310	17
Solyc08g083350.2.1	3	103	5	30	0	30	83	52	0	58	5	84	0
Solyc08g083360.2.1	0	0	0	0	0	0	0	0	0	0	0	0	0
Solyc08g083370.2.1	0	0	0	0	0	0	0	0	0	0	0	0	0
Solyc08g083380.2.1	1	5	0	5	0	7	9	11	1	18	5	12	0
Solyc08g083400.2.1	0	0	0	0	0	0	0	0	0	0	0	0	0
Solyc08g083500.1.1	0	0	0	0	0	0	0	0	0	0	0	0	0
Solyc09g005000.1.1	3	58	3	26	1	180	19	60	5	4	1	2	0
Solyc09g005010.2.1	6	11	0	1	0	60	14	84	3	34	16	68	1
Solyc09g005020.1.1	0	0	0	0	0	0	0	0	0	0	0	0	0
Solyc09g005030.1.1	0	0	0	0	0	0	0	0	0	0	0	0	0
Solyc09g005040.1.1	0	0	0	0	0	0	0	0	0	0	0	0	0
Solyc09g005060.1.1	0	0	0	0	0	0	0	0	0	0	0	0	0
Solyc09g005080.1.1	1	5	1	3	0	0	1	4	0	0	2	6	0

Solyc09g005090.1.1	0	0	0	0	0	0	0	0	0	0	0	0	0
Solyc09g005100.2.1	2	22	1	6	1	32	112	55	2	46	8	15	1
Solyc09g005110.2.1	0	0	0	0	0	0	0	0	0	0	0	0	0
Solyc09g005120.2.1	5	14	2	14	0	29	21	37	6	18	35	45	0
Solyc09g005130.2.1	1	1	0	1	0	7	4	5	0	4	0	7	0
Solyc09g005140.1.1	0	0	0	0	0	0	0	0	0	0	0	0	0
Solyc09g005160.1.1	0	0	0	0	0	0	0	0	0	0	0	0	0
Solyc09g005180.2.1	0	0	0	0	0	0	0	0	0	0	0	0	0
Solyc09g005200.2.1	4	22	3	17	0	16	42	40	0	20	4	10	0
Solyc09g005210.2.1	0	0	0	0	0	0	0	0	0	0	0	0	0
Solyc09g005220.1.1	0	0	0	0	0	0	0	0	0	0	0	0	0
Solyc09g005230.2.1	1	0	0	1	0	3	6	3	0	11	0	5	0
Solyc09g005250.2.1	6	20	1	24	0	23	31	32	1	45	33	58	4
Solyc09g005260.2.1	6	228	22	126	0	1007	208	963	0	28	8	7	0
Solyc09g005270.2.1	1	5	1	0	0	5	11	2	0	4	0	6	0
Solyc09g005280.2.1	5	18	5	20	5	42	43	40	11	50	13	24	0
Solyc09g005290.1.1	0	0	0	0	0	0	0	0	0	0	0	0	0
Solyc09g005300.2.1	1	18	0	4	0	40	4	50	2	35	2	36	0
Solyc09g005310.2.1	3	12	1	1	0	11	7	15	3	17	8	10	0
Solyc09g005320.2.1	0	0	0	0	0	0	0	0	0	0	0	0	0
Solyc09g005330.2.1	7	28	2	9	0	124	42	92	7	67	16	34	0
Solyc09g005340.2.1	5	22	1	24	1	57	28	69	3	141	13	85	0
Solyc09g005350.2.1	4	31	1	13	0	35	27	85	7	64	11	34	1
Solyc09g005360.2.1	5	76	4	49	5	99	61	159	15	119	13	132	1
Solyc09g005390.2.1	5	25	6	15	0	31	67	36	10	40	18	23	1
Solyc09g005430.1.1	0	0	0	0	0	0	0	0	0	0	0	0	0
Solyc09g005450.1.1	0	0	0	0	0	0	0	0	0	0	0	0	0
Solyc09g005460.1.1	0	0	0	0	0	0	0	0	0	0	0	0	0
Solyc09g005470.2.1	0	0	0	0	0	0	0	0	0	0	0	0	0
Solyc09g005480.2.1	0	0	0	0	0	0	0	0	0	0	0	0	0
Solyc09g005500.2.1	0	0	0	0	0	0	0	0	0	0	0	0	0
Solyc09g005530.2.1	0	0	0	0	0	0	0	0	0	0	0	0	0
Solyc09g005550.2.1	1	12	3	18	1	17	19	19	1	14	26	18	0
Solyc09g005560.2.1	1	1	0	0	0	12	4	11	1	0	2	0	0
Solyc09g005570.2.1	0	0	0	0	0	0	0	0	0	0	0	0	0
Solyc09g005580.2.1	1	3	1	1	1	1	11	4	4	1	11	1	0
Solyc09g005590.2.1	1	10	7	6	7	1	69	9	21	24	18	17	6
Solyc09g005600.2.1	7	20	11	18	10	58	102	29	7	100	48	140	8
Solyc09g005610.2.1	0	0	0	0	0	0	0	0	0	0	0	0	0
Solyc09g005620.2.1	8	156	26	89	1	195	165	208	4	147	15	140	1
Solyc09g005630.2.1	0	0	0	0	0	0	0	0	0	0	0	0	0

Solyc09g005640.2.1	2	2	0	0	0	17	6	13	0	13	5	6	0
Solyc09g005650.1.1	0	0	0	0	0	0	0	0	0	0	0	0	0
Solyc09g005660.2.1	10	14	0	4	0	134	66	234	6	41	8	25	0
Solyc09g005680.1.1	0	0	0	0	0	0	0	0	0	0	0	0	0
Solyc09g005690.2.1	0	0	0	0	0	0	0	0	0	0	0	0	0
Solyc09g005700.2.1	5	76	4	55	0	68	69	101	0	212	21	187	9
Solyc09g005710.1.1	1	1	0	4	0	14	9	18	1	7	4	8	0
Solyc09g005720.2.1	2	133	8	60	8	353	72	562	43	314	49	213	0
Solyc09g005730.2.1	0	0	0	0	0	0	0	0	0	0	0	0	0
Solyc09g005740.1.1	4	21	2	10	0	56	15	31	2	66	14	19	0
Solyc09g005750.2.1	2	5	1	11	0	0	1	5	0	35	3	13	0
Solyc09g005760.2.1	12	57	4	27	8	93	65	136	19	108	25	76	6
Solyc09g005810.2.1	1	11	1	1	0	9	4	22	0	24	0	10	0
Solyc09g005830.1.1	0	0	0	0	0	0	0	0	0	0	0	0	0
Solyc09g005840.1.1	0	0	0	0	0	0	0	0	0	0	0	0	0
Solyc09g005850.2.1	3	18	3	7	0	9	14	30	1	40	8	45	4
Solyc09g005860.2.1	4	29	8	42	1	10	18	15	3	15	9	45	7
Solyc09g005870.1.1	1	1	0	0	0	15	3	4	1	3	0	4	0
Solyc09g005880.1.1	0	0	0	0	0	0	0	0	0	0	0	0	0
Solyc09g005890.2.1	0	0	0	0	0	0	0	0	0	0	0	0	0
Solyc09g005910.2.1	16	128	20	74	12	261	268	215	32	163	47	105	4
Solyc09g005920.2.1	11	95	12	57	13	154	140	170	50	139	61	146	0
Solyc09g005930.2.1	10	78	17	31	5	158	141	232	29	217	39	121	5
Solyc09g005960.2.1	5	52	3	30	0	121	39	68	13	62	25	45	0
Solyc09g005970.1.1	0	0	0	0	0	0	0	0	0	0	0	0	0
Solyc09g005980.2.1	8	82	5	40	5	129	120	195	11	153	16	112	1
Solyc09g005990.2.1	0	0	0	0	0	0	0	0	0	0	0	0	0
Solyc09g006000.2.1	1	12	0	6	0	10	5	12	0	6	3	9	0
Solyc09g006010.2.1	0	0	0	0	0	0	0	0	0	0	0	0	0
Solyc09g007010.1.1	2	11	6	1	0	10	3	49	0	1	0	0	0
Solyc09g007020.1.1	0	0	0	0	0	0	0	0	0	0	0	0	0
Solyc09g007030.2.1	1	0	0	0	0	4	1	7	0	1	3	2	0
Solyc09g007040.1.1	0	0	0	0	0	0	0	0	0	0	0	0	0
Solyc09g007050.1.1	0	0	0	0	0	0	0	0	0	0	0	0	0
Solyc09g007060.2.1	1	0	0	5	0	0	5	0	0	13	0	0	0
Solyc09g007070.1.1	0	0	0	0	0	0	0	0	0	0	0	0	0
Solyc09g007080.2.1	6	23	5	8	1	69	29	56	9	49	10	32	0
Solyc09g007090.2.1	2	6	1	8	10	34	13	39	1	48	2	93	6
Solyc09g007100.2.1	8	25	15	49	10	43	80	133	12	182	43	104	9
Solyc09g007110.2.1	0	0	0	0	0	0	0	0	0	0	0	0	0
Solyc09g007120.2.1	0	0	0	0	0	0	0	0	0	0	0	0	0

Solyc09g007130.2.1	0	0	0	0	0	0	0	0	0	0	0	0	0
Solyc09g007140.2.1	0	0	0	0	0	0	0	0	0	0	0	0	0
Solyc09g007150.2.1	14	457	17	96	5	24	15	25	1	126	139	32	0
Solyc09g007160.2.1	4	14	3	6	0	10	14	24	1	26	2	16	0
Solyc09g007170.2.1	4	28	0	10	2	26	16	32	6	29	8	24	0
Solyc09g007180.2.1	10	187	12	108	8	325	123	356	27	394	91	330	13
Solyc09g007190.2.1	13	116	67	44	4	19	105	41	3	97	31	104	1
Solyc09g007200.2.1	5	16	7	9	0	22	23	22	1	18	4	19	0
Solyc09g007210.2.1	4	14	2	4	0	12	20	32	6	22	7	25	0
Solyc09g007220.1.1	0	0	0	0	0	0	0	0	0	0	0	0	0
Solyc09g007230.2.1	6	108	7	52	1	284	99	244	27	180	45	102	0
Solyc09g007240.2.1	0	0	0	0	0	0	0	0	0	0	0	0	0
Solyc09g007250.2.1	9	455	73	351	55	1542	660	1995	154	1563	546	1318	42
Solyc09g007260.2.1	0	0	0	0	0	0	0	0	0	0	0	0	0
Solyc09g007270.2.1	5	23	8	28	0	73	77	52	4	449	321	607	4
Solyc09g007280.2.1	0	0	0	0	0	0	0	0	0	0	0	0	0
Solyc09g007290.2.1	2	17	1	24	1	18	5	38	5	60	3	41	0
Solyc09g007300.1.1	2	2	4	9	0	12	8	8	0	20	8	11	3
Solyc09g007310.2.1	25	28	12	35	5	99	259	182	27	133	37	136	7
Solyc09g007320.2.1	9	62	26	65	0	74	352	204	16	82	80	60	2
Solyc09g007330.2.1	0	0	0	0	0	0	0	0	0	0	0	0	0
Solyc09g007340.2.1	0	0	0	0	0	0	0	0	0	0	0	0	0
Solyc09g007350.2.1	1	34	4	10	0	20	26	56	9	55	10	35	1
Solyc09g007360.2.1	0	0	0	0	0	0	0	0	0	0	0	0	0
Solyc09g007370.2.1	0	0	0	0	0	0	0	0	0	0	0	0	0
Solyc09g007380.2.1	0	0	0	0	0	0	0	0	0	0	0	0	0
Solyc09g007390.2.1	1	1	0	2	0	1	4	3	0	4	0	8	0
Solyc09g007410.2.1	9	34	5	38	3	43	40	22	7	147	57	89	2
Solyc09g007420.2.1	1	0	0	1	0	9	8	3	0	0	0	0	0
Solyc09g007430.1.1	4	9	0	18	0	22	11	14	1	13	6	24	1
Solyc09g007440.2.1	3	3	0	1	0	3	11	18	0	23	3	23	0
Solyc09g007450.2.1	2	4	0	2	0	29	5	13	0	7	2	10	0
Solyc09g007460.2.1	1	0	0	2	0	6	5	2	1	0	0	0	0
Solyc09g007470.1.1	1	2	2	0	0	0	2	0	0	2	2	10	2
Solyc09g007480.2.1	0	0	0	0	0	0	0	0	0	0	0	0	0
Solyc09g007490.2.1	15	927	52	510	36	2134	907	1819	243	1694	182	1154	19
Solyc09g007500.2.1	1	16	1	10	0	48	5	21	2	59	1	45	0
Solyc09g007510.1.1	1	2	0	1	0	0	6	3	0	22	0	2	0
Solyc09g007520.2.1	0	0	0	0	0	0	0	0	0	0	0	0	0
Solyc09g007530.2.1	9	126	12	102	12	416	133	362	25	310	36	226	5
Solyc09g007540.2.1	14	126	16	135	5	218	107	469	11	604	60	427	3

Solyc09g007550.2.1	2	4	0	1	0	2	3	20	0	13	6	8	0
Solyc09g007560.2.1	8	404	75	142	4	236	147	190	9	329	63	182	7
Solyc09g007570.1.1	1	0	0	0	0	2	3	4	0	2	0	5	0
Solyc09g007590.1.1	0	0	0	0	0	0	0	0	0	0	0	0	0
Solyc09g007630.2.1	6	28	3	6	1	87	20	72	13	22	26	24	0
Solyc09g007640.2.1	2	15	0	15	0	14	8	8	0	6	2	3	0
Solyc09g007650.1.1	0	0	0	0	0	0	0	0	0	0	0	0	0
Solyc09g007660.1.1	2	22	1	23	0	15	16	12	0	1	0	0	0
Solyc09g007670.2.1	1	0	0	0	0	8	3	3	0	3	1	9	0
Solyc09g007680.2.1	7	17	2	15	0	25	41	52	6	40	11	39	0
Solyc09g007700.2.1	4	133	3	83	1	149	45	213	14	164	41	122	2
Solyc09g007710.2.1	0	0	0	0	0	0	0	0	0	0	0	0	0
Solyc09g007720.2.1	0	0	0	0	0	0	0	0	0	0	0	0	0
Solyc09g007730.2.1	0	0	0	0	0	0	0	0	0	0	0	0	0
Solyc09g007750.2.1	0	0	0	0	0	0	0	0	0	0	0	0	0
Solyc09g007760.2.1	0	0	0	0	0	0	0	0	0	0	0	0	0
Solyc09g007770.2.1	1	337	0	119	1	81	5	159	1	3	0	13	0
Solyc09g007780.1.1	0	0	0	0	0	0	0	0	0	0	0	0	0
Solyc09g007790.1.1	6	3	1	19	4	145	225	256	17	151	24	178	3
Solyc09g007800.2.1	0	0	0	0	0	0	0	0	0	0	0	0	0
Solyc09g007810.2.1	1	13	1	2	0	6	1	11	2	10	6	2	5
Solyc09g007830.2.1	1	9	3	9	0	0	1	2	0	0	0	0	0
Solyc09g007840.2.1	0	0	0	0	0	0	0	0	0	0	0	0	0
Solyc09g007850.2.1	5	801	145	1081	13	107	910	650	36	513	38	500	16
Solyc09g007860.2.1	7	9	0	3	1	91	35	43	7	54	8	34	0
Solyc09g007870.2.1	34	482	25	432	30	1298	527	1938	81	1063	153	808	14
Solyc09g007880.1.1	3	39	0	4	2	67	16	42	2	67	14	39	0
Solyc09g007890.1.1	0	0	0	0	0	0	0	0	0	0	0	0	0
Solyc09g007900.2.1	2	46	54	61	8	1	58	10	20	12	42	29	0
Solyc09g007910.2.1	3	42	15	26	3	9	24	6	7	21	33	19	2
Solyc09g007920.2.1	1	88	10	45	0	35	4	37	4	90	10	43	0
Solyc09g007940.2.1	2	23	3	13	1	3	9	8	6	36	6	23	6
Solyc09g007950.1.1	1	2	2	1	0	5	7	8	3	12	4	2	1
Solyc09g007980.1.1	5	39	6	22	0	30	32	33	1	4	6	4	0
Solyc09g007990.2.1	8	33	10	50	9	133	71	123	9	118	17	91	4
Solyc09g008000.2.1	0	0	0	0	0	0	0	0	0	0	0	0	0
Solyc09g008010.2.1	4	48	2	66	1	198	24	96	7	64	4	68	0
Solyc09g008020.2.1	3	3	1	0	0	27	29	28	4	8	2	8	0
Solyc09g008030.1.1	0	0	0	0	0	0	0	0	0	0	0	0	0
Solyc09g008040.2.1	8	43	6	31	1	20	65	67	5	21	12	31	0
Solyc09g008050.2.1	9	31	6	17	0	120	106	102	17	64	2	21	3

Solyc09g008060.2.1	0	0	0	0	0	0	0	0	0	0	0	0	0
Solyc09g008070.2.1	0	0	0	0	0	0	0	0	0	0	0	0	0
Solyc09g008080.2.1	1	8	0	13	0	0	3	3	0	1	0	0	0
Solyc09g008090.2.1	0	0	0	0	0	0	0	0	0	0	0	0	0
Solyc09g008100.2.1	13	48	11	25	8	90	38	88	10	88	27	41	5
Solyc09g008120.2.1	3	2	0	0	0	11	8	9	2	13	14	7	0
Solyc09g008130.2.1	3	5	1	0	7	20	12	20	0	6	7	4	0
Solyc09g008140.2.1	0	0	0	0	0	0	0	0	0	0	0	0	0
Solyc09g008150.1.1	1	7	0	0	0	17	5	13	0	12	3	15	0
Solyc09g008160.2.1	2	172	19	112	3	60	158	109	15	185	25	342	1
Solyc09g008190.2.1	0	0	0	0	0	0	0	0	0	0	0	0	0
Solyc09g008200.2.1	17	391	36	296	12	1079	243	1076	12	1839	205	1084	50
Solyc09g008230.2.1	1	32	0	11	0	102	10	66	0	38	3	13	0
Solyc09g008240.2.1	21	378	59	313	10	102	96	309	37	405	65	349	1
Solyc09g008250.2.1	0	0	0	0	0	0	0	0	0	0	0	0	0
Solyc09g008260.2.1	1	7	0	3	0	5	4	7	0	1	1	1	3
Solyc09g008270.2.1	2	38	1	14	0	3	17	3	1	12	0	8	0
Solyc09g008280.1.1	4	1567	842	1470	915	7850	4081	3861	2273	3648	1279	2162	26
Solyc09g008290.2.1	5	25	4	4	1	28	100	26	5	62	6	55	1
Solyc09g008300.2.1	1	0	0	0	0	4	4	3	0	2	2	1	0
Solyc09g008310.2.1	6	21	19	25	4	25	65	34	1	27	4	24	1
Solyc09g008320.2.1	9	167	3	127	2	26	64	300	3	426	7	251	3
Solyc09g008330.2.1	1	1	0	0	0	16	2	8	2	5	2	6	0
Solyc09g008340.2.1	4	115	5	42	3	142	63	168	15	76	15	43	1
Solyc09g008350.2.1	4	22	10	46	0	8	10	38	10	20	7	25	2
Solyc09g008370.1.1	1	10	1	7	0	19	0	6	0	8	1	3	0
Solyc09g008380.2.1	0	0	0	0	0	0	0	0	0	0	0	0	0
Solyc09g008400.2.1	6	18	2	21	1	85	16	47	7	44	5	44	0
Solyc09g008410.2.1	2	69	5	17	3	50	35	72	8	68	9	45	0
Solyc09g008430.2.1	4	1	0	0	2	36	76	15	1	3	5	13	0
Solyc09g008440.1.1	0	0	0	0	0	0	0	0	0	0	0	0	0
Solyc09g008450.2.1	2	2	2	0	0	28	40	3	5	15	15	12	0
Solyc09g008460.2.1	1	13	3	22	0	11	2	3	0	20	0	52	0
Solyc09g008470.2.1	6	98	9	34	10	191	120	267	15	134	71	124	0
Solyc09g008480.2.1	13	166	14	126	21	355	119	467	17	172	49	182	1
Solyc09g008490.2.1	10	36	5	42	2	129	112	89	7	83	12	72	1
Solyc09g008520.2.1	4	26	1	26	2	51	19	20	0	34	3	41	0
Solyc09g008530.1.1	0	0	0	0	0	0	0	0	0	0	0	0	0
Solyc09g008550.2.1	8	29	6	21	7	22	24	77	0	34	7	22	0
Solyc09g008560.2.1	0	0	0	0	0	0	0	0	0	0	0	0	0
Solyc09g008600.2.1	2	5	0	14	0	36	14	26	0	23	5	12	0

Solyc09g008610.2.1	25	262	10	113	26	522	262	710	68	430	82	274	6
Solyc09g008620.1.1	0	0	0	0	0	0	0	0	0	0	0	0	0
Solyc09g008630.2.1	0	0	0	0	0	0	0	0	0	0	0	0	0
Solyc09g008640.1.1	2	5	6	5	0	8	13	8	0	2	0	0	1
Solyc09g008650.2.1	3	72	10	22	0	2	32	1	1	4	9	12	0
Solyc09g008660.2.1	0	0	0	0	0	0	0	0	0	0	0	0	0
Solyc09g008670.2.1	32	472	59	325	1	5032	9516	2302	18	19	4	9	0
Solyc09g008680.2.1	0	0	0	0	0	0	0	0	0	0	0	0	0
Solyc09g008690.1.1	0	0	0	0	0	0	0	0	0	0	0	0	0
Solyc09g008700.1.1	8	28	4	15	6	32	66	84	6	44	18	41	2
Solyc09g008730.2.1	3	8	2	5	0	16	28	31	1	38	7	44	0
Solyc09g008770.2.1	3	1	0	0	0	47	38	21	2	1	24	1	0
Solyc09g008780.2.1	17	1059	85	1028	3	2314	1569	2606	71	1684	400	289	5
Solyc09g008790.2.1	10	26	2	32	4	76	68	85	4	48	7	56	0
Solyc09g008800.2.1	4	107	28	68	29	449	393	339	125	495	204	284	14
Solyc09g008810.2.1	0	0	0	0	0	0	0	0	0	0	0	0	0
Solyc09g008820.2.1	0	0	0	0	0	0	0	0	0	0	0	0	0
Solyc09g008830.2.1	3	4	1	4	0	15	20	13	0	183	13	48	0
Solyc09g008840.2.1	4	10	3	0	0	1	6	5	3	52	18	77	0
Solyc09g008850.2.1	7	29	1	8	0	40	27	54	5	35	9	25	0
Solyc09g008860.2.1	0	0	0	0	0	0	0	0	0	0	0	0	0
Solyc09g008920.2.1	9	272	42	146	11	469	279	717	17	323	54	271	3
Solyc09g008930.2.1	3	10	0	4	2	23	7	10	0	69	0	80	0
Solyc09g008940.2.1	6	39	0	19	0	23	30	34	2	57	13	35	3
Solyc09g008970.1.1	0	0	0	0	0	0	0	0	0	0	0	0	0
Solyc09g008990.2.1	0	0	0	0	0	0	0	0	0	0	0	0	0
Solyc09g009000.2.1	0	0	0	0	0	0	0	0	0	0	0	0	0
Solyc09g009010.2.1	0	0	0	0	0	0	0	0	0	0	0	0	0
Solyc09g009020.2.1	5	181	23	180	18	2174	222	1204	67	1119	211	1611	28
Solyc09g009030.2.1	11	172	18	88	16	389	205	459	38	451	109	374	12
Solyc09g009040.2.1	7	166	2	59	0	23	13	66	1	48	19	29	1
Solyc09g009070.1.1	0	0	0	0	0	0	0	0	0	0	0	0	0
Solyc09g009080.2.1	12	67	7	41	5	57	63	108	6	31	11	32	0
Solyc09g009090.2.1	11	42	5	35	0	125	100	144	11	93	14	64	8
Solyc09g009100.2.1	9	179	10	127	1	39	14	73	13	270	8	267	0
Solyc09g009120.2.1	22	107	14	152	18	365	206	357	32	136	46	181	9
Solyc09g009130.2.1	4	239	4	68	4	282	49	176	1	16	6	17	0
Solyc09g009140.2.1	0	0	0	0	0	0	0	0	0	0	0	0	0
Solyc09g009150.1.1	0	0	0	0	0	0	0	0	0	0	0	0	0
Solyc09g009160.1.1	0	0	0	0	0	0	0	0	0	0	0	0	0
Solyc09g009170.2.1	12	84	23	27	19	139	126	127	30	172	63	92	11

Solyc09g009180.2.1	7	28	10	21	4	122	48	121	19	56	18	48	6
Solyc09g009190.2.1	14	252	57	108	7	137	184	342	2	210	69	324	10
Solyc09g009200.1.1	0	0	0	0	0	0	0	0	0	0	0	0	0
Solyc09g009210.2.1	2	13	0	10	0	11	1	28	2	33	4	16	0
Solyc09g009220.2.1	13	44	0	14	4	191	156	165	3	6	13	0	0
Solyc09g009240.1.1	0	0	0	0	0	0	0	0	0	0	0	0	0
Solyc09g009250.2.1	0	0	0	0	0	0	0	0	0	0	0	0	0
Solyc09g009260.2.1	19	805	64	656	109	1888	493	2016	345	5194	840	3930	30
Solyc09g009270.1.1	1	0	0	0	0	2	0	3	0	9	4	6	0
Solyc09g009290.2.1	5	14	7	2	0	19	69	26	6	18	6	14	0
Solyc09g009300.2.1	0	0	0	0	0	0	0	0	0	0	0	0	0
Solyc09g009340.1.1	2	14	3	10	0	11	6	15	0	16	1	4	0
Solyc09g009350.2.1	1	1	0	0	0	2	4	0	0	5	0	13	0
Solyc09g009370.2.1	14	47	6	36	1	250	135	319	25	163	34	152	4
Solyc09g009380.2.1	0	0	0	0	0	0	0	0	0	0	0	0	0
Solyc09g009390.2.1	2	229	26	117	15	150	108	279	28	162	40	165	0
Solyc09g009400.2.1	1	2	0	2	0	5	7	11	0	0	0	1	0
Solyc09g009420.1.1	2	187	18	70	0	4	4	1	0	15	4	2	0
Solyc09g009430.2.1	5	152	16	98	5	124	125	251	15	264	13	177	2
Solyc09g009440.1.1	2	125	3	42	0	105	29	149	4	30	3	24	0
Solyc09g009450.1.1	0	0	0	0	0	0	0	0	0	0	0	0	0
Solyc09g009460.2.1	0	0	0	0	0	0	0	0	0	0	0	0	0
Solyc09g009470.1.1	3	6	2	10	0	4	6	8	3	10	4	17	2
Solyc09g009480.1.1	0	0	0	0	0	0	0	0	0	0	0	0	0
Solyc09g009490.2.1	0	0	0	0	0	0	0	0	0	0	0	0	0
Solyc09g009500.2.1	2	16	0	16	0	38	16	34	1	15	2	10	0
Solyc09g009510.2.1	9	53	1	26	2	76	27	68	7	96	19	115	1
Solyc09g009520.2.1	0	0	0	0	0	0	0	0	0	0	0	0	0
Solyc09g009530.2.1	9	246	24	131	6	211	169	382	61	1051	254	342	0
Solyc09g009540.2.1	0	0	0	0	0	0	0	0	0	0	0	0	0
Solyc09g009550.2.1	0	0	0	0	0	0	0	0	0	0	0	0	0
Solyc09g009570.1.1	0	0	0	0	0	0	0	0	0	0	0	0	0
Solyc09g009580.2.1	0	0	0	0	0	0	0	0	0	0	0	0	0
Solyc09g009590.1.1	4	15	0	13	0	201	6	192	2	44	17	37	0
Solyc09g009600.1.1	0	0	0	0	0	0	0	0	0	0	0	0	0
Solyc09g009630.2.1	1	6	0	1	3	0	1	8	0	1	3	5	0
Solyc09g009640.2.1	5	136	21	84	5	294	648	414	60	217	73	129	4
Solyc09g009650.2.1	0	0	0	0	0	0	0	0	0	0	0	0	0
Solyc09g009660.2.1	3	11	4	19	3	1	15	26	2	32	3	33	0
Solyc09g009670.2.1	2	1	0	0	0	0	10	1	1	14	1	13	0
Solyc09g009680.2.1	8	31	3	17	2	51	52	103	8	14	14	26	0

Solyc09g009690.2.1	6	67	4	41	1	160	39	188	3	496	24	307	2
Solyc09g009700.2.1	4	43	4	24	0	77	17	99	0	24	1	8	0
Solyc09g009710.2.1	4	2	0	0	0	2	18	3	3	16	5	9	0
Solyc09g009720.1.1	4	27	1	5	1	15	17	17	2	61	21	62	0
Solyc09g009760.1.1	0	0	0	0	0	0	0	0	0	0	0	0	0
Solyc09g009770.2.1	1	20	1	25	0	3	2	8	0	17	2	4	0
Solyc09g009780.2.1	2	11	0	5	0	56	22	19	0	16	4	19	0
Solyc09g009790.2.1	1	0	0	0	0	6	4	5	1	0	2	0	0
Solyc09g009800.2.1	8	51	23	39	3	206	107	105	14	185	19	105	6
Solyc09g009810.1.1	1	3	0	0	0	9	3	21	0	0	1	0	0
Solyc09g009820.2.1	22	714	102	646	4	867	498	1476	18	222	48	211	4
Solyc09g009830.2.1	3	84	19	47	0	3	2	8	0	6	8	10	0
Solyc09g009840.1.1	1	14	0	12	0	72	0	19	5	12	4	22	0
Solyc09g009850.2.1	3	12	0	14	1	24	10	22	1	47	10	30	0
Solyc09g009860.1.1	0	0	0	0	0	0	0	0	0	0	0	0	0
Solyc09g009870.2.1	5	43	19	32	0	120	146	123	14	94	25	73	1
Solyc09g009880.2.1	1	6	0	11	0	9	3	27	0	5	0	3	0
Solyc09g009890.2.1	9	91	6	58	4	128	147	245	23	93	26	104	2
Solyc09g009900.2.1	0	0	0	0	0	0	0	0	0	0	0	0	0
Solyc09g009910.2.1	1	1	0	0	0	0	6	6	0	5	0	0	0
Solyc09g009920.1.1	2	10	1	2	3	1	9	22	0	26	0	4	0
Solyc09g009940.2.1	10	216	25	119	1	176	247	231	15	240	36	128	1
Solyc09g009950.2.1	0	0	0	0	0	0	0	0	0	0	0	0	0
Solyc09g009960.2.1	2	11	2	6	0	82	11	49	1	26	6	31	0
Solyc09g009970.2.1	0	0	0	0	0	0	0	0	0	0	0	0	0
Solyc09g009980.1.1	0	0	0	0	0	0	0	0	0	0	0	0	0
Solyc09g009990.2.1	10	68	15	45	3	28	64	76	5	185	44	101	2
Solyc09g010000.2.1	2	2	2	0	0	35	31	31	3	4	4	2	0
Solyc09g010020.2.1	10	278	34	162	8	306	159	282	25	138	33	81	3
Solyc09g010030.1.1	0	0	0	0	0	0	0	0	0	0	0	0	0
Solyc09g010040.1.1	0	0	0	0	0	0	0	0	0	0	0	0	0
Solyc09g010050.1.1	0	0	0	0	0	0	0	0	0	0	0	0	0
Solyc09g010060.2.1	0	0	0	0	0	0	0	0	0	0	0	0	0
Solyc09g010070.1.1	0	0	0	0	0	0	0	0	0	0	0	0	0
Solyc09g010080.2.1	1	0	0	0	0	0	0	0	0	10	1	9	0
Solyc09g010090.2.1	1	0	0	0	0	0	0	1	1	4	5	2	0
Solyc09g010100.2.1	0	0	0	0	0	0	0	0	0	0	0	0	0
Solyc09g010110.2.1	13	92	9	85	15	195	226	148	16	87	20	87	11
Solyc09g010120.2.1	3	249	5	151	0	28	14	52	0	55	16	44	0
Solyc09g010130.2.1	5	78	1	47	0	54	31	86	12	76	7	53	0
Solyc09g010140.1.1	0	0	0	0	0	0	0	0	0	0	0	0	0

Solyc09g010150.2.1	3	13	6	15	0	18	11	12	1	12	3	17	1
Solyc09g010160.1.1	0	0	0	0	0	0	0	0	0	0	0	0	0
Solyc09g010170.2.1	1	8	0	0	0	8	5	0	0	8	1	4	0
Solyc09g010180.2.1	15	84	12	64	6	130	141	200	29	176	46	166	8
Solyc09g010190.2.1	1	2	0	0	0	5	2	3	0	6	4	3	0
Solyc09g010210.2.1	4	6	0	8	0	0	11	12	10	9	64	8	0
Solyc09g010220.2.1	0	0	0	0	0	0	0	0	0	0	0	0	0
Solyc09g010230.1.1	5	43	6	38	0	390	111	138	1	0	1	1	0
Solyc09g010260.2.1	0	0	0	0	0	0	0	0	0	0	0	0	0
Solyc09g010270.1.1	1	6	0	10	0	10	4	11	2	2	0	1	0
Solyc09g010280.2.1	16	232	19	172	4	327	259	457	29	310	53	413	4
Solyc09g010300.2.1	5	30	8	17	3	22	28	50	3	44	18	37	0
Solyc09g010320.2.1	1	1	1	0	0	17	6	9	1	4	2	0	0
Solyc09g010330.2.1	3	129	3	49	2	192	32	138	16	215	13	97	0
Solyc09g010350.2.1	5	23	13	5	0	11	27	22	2	67	17	89	1
Solyc09g010360.2.1	3	19	18	3	1	7	25	6	1	37	64	24	0
Solyc09g010370.2.1	5	222	10	81	3	512	82	203	19	609	18	372	8
Solyc09g010380.2.1	3	104	1	20	1	15	19	35	3	395	19	262	5
Solyc09g010390.2.1	2	4	0	1	0	5	14	20	1	5	2	5	0
Solyc09g010400.2.1	3	85	8	28	4	40	63	86	5	155	13	103	0
Solyc09g010410.2.1	9	198	16	70	10	387	100	297	33	264	62	130	4
Solyc09g010420.2.1	8	34	8	20	0	24	40	73	1	90	17	101	10
Solyc09g010430.1.1	1	21	0	0	0	0	0	1	0	130	4	90	0
Solyc09g010440.2.1	1	84	7	14	0	51	29	97	5	136	17	116	5
Solyc09g010450.1.1	4	7	3	11	0	8	38	30	3	35	5	6	0
Solyc09g010460.2.1	14	241	22	212	15	696	165	843	91	424	110	316	7
Solyc09g010470.2.1	3	39	1	55	3	47	25	45	2	21	6	13	0
Solyc09g010480.1.1	0	0	0	0	0	0	0	0	0	0	0	0	0
Solyc09g010500.2.1	2	35	1	18	1	39	30	41	6	53	18	54	2
Solyc09g010510.2.1	0	0	0	0	0	0	0	0	0	0	0	0	0
Solyc09g010520.2.1	8	230	8	85	13	236	107	340	40	951	111	436	7
Solyc09g010530.2.1	0	0	0	0	0	0	0	0	0	0	0	0	0
Solyc09g010540.2.1	0	0	0	0	0	0	0	0	0	0	0	0	0
Solyc09g010550.2.1	0	0	0	0	0	0	0	0	0	0	0	0	0
Solyc09g010560.1.1	14	45	8	56	0	153	98	218	14	157	29	136	1
Solyc09g010570.1.1	1	0	1	0	0	5	18	2	9	0	0	0	0
Solyc09g010600.1.1	0	0	0	0	0	0	0	0	0	0	0	0	0
Solyc09g010610.1.1	0	0	0	0	0	0	0	0	0	0	0	0	0
Solyc09g010630.2.1	20	4399	578	3160	395	6433	4056	5461	337	19050	2243	14406	151
Solyc09g010640.2.1	13	272	36	183	12	465	98	256	29	141	72	89	10
Solyc09g010650.1.1	1	10	0	12	0	19	7	19	0	2	0	9	0

Solyc09g010660.2.1	9	46	6	43	4	53	24	54	3	30	10	25	0
Solyc09g010670.2.1	0	0	0	0	0	0	0	0	0	0	0	0	0
Solyc09g010680.2.1	5	23	3	11	0	20	27	28	0	6	0	15	0
Solyc09g010690.2.1	4	39	1	66	0	57	17	281	0	348	13	313	6
Solyc09g010700.2.1	0	0	0	0	0	0	0	0	0	0	0	0	0
Solyc09g010710.2.1	0	0	0	0	0	0	0	0	0	0	0	0	0
Solyc09g010720.2.1	0	0	0	0	0	0	0	0	0	0	0	0	0
Solyc09g010740.2.1	0	0	0	0	0	0	0	0	0	0	0	0	0
Solyc09g010770.2.1	0	0	0	0	0	0	0	0	0	0	0	0	0
Solyc09g010780.2.1	0	0	0	0	0	0	0	0	0	0	0	0	0
Solyc09g010800.2.1	1	346	0	152	0	360	33	348	0	165	0	195	1
Solyc09g010810.2.1	0	0	0	0	0	0	0	0	0	0	0	0	0
Solyc09g010820.2.1	1	3	3	0	0	5	4	1	0	4	3	5	0
Solyc09g010830.2.1	30	89	19	85	13	206	373	338	28	349	81	252	4
Solyc09g010840.1.1	7	76	9	55	0	16	33	27	0	181	50	204	3
Solyc09g010850.2.1	2	62	6	63	0	28	8	52	8	15	3	2	0
Solyc09g010860.2.1	4	4	0	8	1	9	6	21	3	13	13	23	0
Solyc09g010870.2.1	13	99	12	85	2	100	111	112	4	162	32	68	0
Solyc09g010880.2.1	3	16	0	0	0	48	37	60	10	27	6	23	1
Solyc09g010920.1.1	6	38	1	33	0	55	22	58	3	32	0	26	0
Solyc09g010930.2.1	11	325	9	207	11	526	114	423	22	234	87	138	0
Solyc09g010940.2.1	11	735	14	735	2	2940	354	3601	54	557	73	784	1
Solyc09g010950.2.1	9	50	3	30	3	190	91	157	23	72	36	58	3
Solyc09g010970.2.1	6	128	15	73	3	96	84	106	31	52	31	28	2
Solyc09g010980.1.1	1	19	10	11	1	3	5	9	2	0	0	5	0
Solyc09g010990.2.1	0	0	0	0	0	0	0	0	0	0	0	0	0
Solyc09g011000.2.1	0	0	0	0	0	0	0	0	0	0	0	0	0
Solyc09g011010.2.1	1	48	1	20	0	4	2	3	1	26	3	31	0
Solyc09g011020.2.1	10	36	5	12	4	102	75	100	6	134	27	55	1
Solyc09g011030.2.1	18	244	12	189	7	468	86	600	13	523	88	414	2
Solyc09g011040.1.1	0	0	0	0	0	0	0	0	0	0	0	0	0
Solyc09g011050.2.1	0	0	0	0	0	0	0	0	0	0	0	0	0
Solyc09g011060.2.1	0	0	0	0	0	0	0	0	0	0	0	0	0
Solyc09g011070.1.1	0	0	0	0	0	0	0	0	0	0	0	0	0
Solyc09g011080.2.1	7	602	110	272	0	12	2	28	1	31	31	80	0
Solyc09g011140.2.1	3	184	2	74	0	0	19	2	0	48	0	50	0
Solyc09g011160.2.1	1	4	0	0	0	1	0	0	1	0	7	4	0
Solyc09g011170.2.1	17	118	24	68	3	246	141	460	10	128	16	127	1
Solyc09g011190.1.1	0	0	0	0	0	0	0	0	0	0	0	0	0
Solyc09g011210.2.1	1	8	0	12	0	18	14	8	0	16	2	31	0
Solyc09g011220.2.1	1	1	0	1	0	3	0	8	0	22	5	14	0

Solyc09g011230.2.1	0	0	0	0	0	0	0	0	0	0	0	0	0
Solyc09g011240.2.1	2	17	0	30	0	120	10	56	2	29	4	21	2
Solyc09g011250.2.1	0	0	0	0	0	0	0	0	0	0	0	0	0
Solyc09g011270.2.1	0	0	0	0	0	0	0	0	0	0	0	0	0
Solyc09g011300.2.1	4	51	7	34	2	33	44	36	10	75	8	53	0
Solyc09g011310.2.1	3	24	4	12	3	74	45	95	24	38	30	25	2
Solyc09g011320.2.1	9	23	6	10	2	55	113	76	24	30	25	28	1
Solyc09g011330.1.1	0	0	0	0	0	0	0	0	0	0	0	0	0
Solyc09g011340.2.1	4	39	3	10	0	22	19	46	1	67	5	59	0
Solyc09g011350.1.1	0	0	0	0	0	0	0	0	0	0	0	0	0
Solyc09g011360.2.1	1	0	0	14	0	12	1	3	3	7	6	10	0
Solyc09g011370.1.1	11	39	2	14	9	102	101	115	15	47	38	23	0
Solyc09g011380.2.1	5	72	7	46	1	7	34	43	4	105	12	66	0
Solyc09g011390.2.1	0	0	0	0	0	0	0	0	0	0	0	0	0
Solyc09g011400.1.1	0	0	0	0	0	0	0	0	0	0	0	0	0
Solyc09g011410.2.1	9	45	6	29	0	70	72	105	5	31	8	33	0
Solyc09g011420.2.1	2	77	12	37	0	176	52	369	31	113	22	71	1
Solyc09g011450.2.1	6	108	3	56	0	179	54	174	5	252	18	139	1
Solyc09g011470.2.1	3	2	0	16	2	148	45	154	2	33	7	25	0
Solyc09g011480.2.1	14	74	6	60	0	54	41	83	2	352	33	210	1
Solyc09g011490.2.1	1	12	4	4	3	3	4	9	2	0	1	0	1
Solyc09g011500.2.1	0	0	0	0	0	0	0	0	0	0	0	0	0
Solyc09g011520.2.1	0	0	0	0	0	0	0	0	0	0	0	0	0
Solyc09g011540.2.1	0	0	0	0	0	0	0	0	0	0	0	0	0
Solyc09g011550.2.1	0	0	0	0	0	0	0	0	0	0	0	0	0
Solyc09g011560.2.1	0	0	0	0	0	0	0	0	0	0	0	0	0
Solyc09g011570.2.1	0	0	0	0	0	0	0	0	0	0	0	0	0
Solyc09g011580.2.1	5	415	17	180	8	574	19	175	9	249	17	132	0
Solyc09g011590.2.1	0	0	0	0	0	0	0	0	0	0	0	0	0
Solyc09g011600.2.1	0	0	0	0	0	0	0	0	0	0	0	0	0
Solyc09g011610.2.1	3	2	1	4	1	6	2	3	1	0	2	0	0
Solyc09g011620.1.1	0	0	0	0	0	0	0	0	0	0	0	0	0
Solyc09g011630.2.1	1	11	4	1	0	2	1	4	0	0	0	0	1
Solyc09g011640.2.1	0	0	0	0	0	0	0	0	0	0	0	0	0
Solyc09g011650.2.1	3	22	8	3	4	36	8	19	0	24	5	25	1
Solyc09g011660.2.1	6	85	16	43	0	158	155	207	28	75	18	118	0
Solyc09g011670.2.1	4	207	10	113	0	550	124	327	0	421	26	421	4
Solyc09g011690.2.1	21	1052	150	393	77	144	102	202	24	281	44	249	17
Solyc09g011710.2.1	10	864	92	669	0	1627	481	1907	1	457	54	635	0
Solyc09g011720.2.1	5	44	17	43	2	1	1	5	0	247	10	214	0
Solyc09g011730.2.1	0	0	0	0	0	0	0	0	0	0	0	0	0

Solyc09g011740.2.1	1	3	0	0	0	2	1	7	1	0	0	0	0
Solyc09g011750.2.1	2	2	0	4	0	0	0	0	0	4	2	0	0
Solyc09g011760.2.1	1	178	2	140	2	1021	46	520	4	684	31	292	1
Solyc09g011770.2.1	3	9	0	3	2	3	0	1	0	18	15	13	0
Solyc09g011780.2.1	0	0	0	0	0	0	0	0	0	0	0	0	0
Solyc09g011800.2.1	0	0	0	0	0	0	0	0	0	0	0	0	0
Solyc09g011810.2.1	11	781	251	333	1	27	131	70	0	39	66	119	0
Solyc09g011820.2.1	0	0	0	0	0	0	0	0	0	0	0	0	0
Solyc09g011830.2.1	6	32	2	28	0	19	31	50	0	54	3	34	0
Solyc09g011840.1.1	8	101	12	77	20	15	17	52	9	124	82	92	10
Solyc09g011850.2.1	8	16	1	19	0	30	39	67	2	8	3	24	0
Solyc09g011860.2.1	3	72	5	106	2	474	19	126	6	6	3	21	2
Solyc09g011870.1.1	0	0	0	0	0	0	0	0	0	0	0	0	0
Solyc09g011880.2.1	7	188	21	127	2	342	128	295	67	234	26	197	1
Solyc09g011910.2.1	0	0	0	0	0	0	0	0	0	0	0	0	0
Solyc09g011920.2.1	1	12	4	3	0	13	16	43	4	20	5	14	0
Solyc09g011930.2.1	8	44	10	33	2	110	408	57	19	106	43	86	3
Solyc09g011960.1.1	3	7	0	0	2	19	20	15	0	1	0	0	0
Solyc09g011970.1.1	0	0	0	0	0	0	0	0	0	0	0	0	0
Solyc09g011980.1.1	0	0	0	0	0	0	0	0	0	0	0	0	0
Solyc09g011990.1.1	0	0	0	0	0	0	0	0	0	0	0	0	0
Solyc09g012000.1.1	0	0	0	0	0	0	0	0	0	0	0	0	0
Solyc09g012040.1.1	0	0	0	0	0	0	0	0	0	0	0	0	0
Solyc09g013070.2.1	0	0	0	0	0	0	0	0	0	0	0	0	0
Solyc09g013080.2.1	19	321	73	271	73	2440	1396	1498	198	528	477	384	32
Solyc09g013090.2.1	2	4	1	12	0	22	9	21	0	23	6	13	0
Solyc09g013100.2.1	5	18	2	22	0	21	23	40	0	26	12	37	0
Solyc09g013110.2.1	1	1	0	6	0	0	0	0	0	12	3	9	0
Solyc09g013120.2.1	13	90	2	69	2	222	62	191	13	111	46	168	0
Solyc09g013130.2.1	13	58	3	28	2	94	71	126	18	109	13	76	2
Solyc09g013140.1.1	1	9	1	1	0	3	8	3	0	9	3	4	0
Solyc09g013150.2.1	2	3	0	11	0	0	5	1	0	38	14	62	6
Solyc09g013160.2.1	0	0	0	0	0	0	0	0	0	0	0	0	0
Solyc09g014160.1.1	1	0	0	2	0	8	7	2	0	0	0	0	0
Solyc09g014170.2.1	8	23	3	24	1	51	51	65	15	61	8	55	0
Solyc09g014210.1.1	0	0	0	0	0	0	0	0	0	0	0	0	0
Solyc09g014240.2.1	6	87	4	40	0	138	66	123	1	0	4	1	0
Solyc09g014250.2.1	5	22	2	11	3	20	30	23	7	11	10	15	3
Solyc09g014280.1.1	19	342	20	274	5	615	199	255	2	100	196	72	2
Solyc09g014300.2.1	12	142	11	117	6	142	53	215	9	259	56	149	1
Solyc09g014310.2.1	0	0	0	0	0	0	0	0	0	0	0	0	0

Solyc09g014320.2.1	8	79	7	25	3	208	56	168	10	64	24	48	0
Solyc09g014350.2.1	9	190	20	5	47	78	135	5	117	1110	111	43	95
Solyc09g014370.2.1	0	0	0	0	0	0	0	0	0	0	0	0	0
Solyc09g014380.2.1	6	101	21	30	8	43	26	115	3	30	4	8	1
Solyc09g014400.2.1	11	80	11	33	8	37	42	93	5	75	16	59	2
Solyc09g014410.2.1	0	0	0	0	0	0	0	0	0	0	0	0	0
Solyc09g014430.1.1	1	3	0	1	0	6	4	8	0	0	0	8	0
Solyc09g014440.2.1	7	27	5	24	0	116	63	100	10	39	20	32	0
Solyc09g014450.2.1	5	9	0	14	0	20	20	59	2	51	24	41	0
Solyc09g014480.1.1	0	0	0	0	0	0	0	0	0	0	0	0	0
Solyc09g014490.2.1	0	0	0	0	0	0	0	0	0	0	0	0	0
Solyc09g014500.2.1	1	8	0	15	3	127	3	26	1	100	3	23	0
Solyc09g014510.2.1	1	0	0	0	0	3	4	1	0	1	0	8	0
Solyc09g014520.2.1	5	4200	333	2472	42	83	226	109	12	336	364	322	20
Solyc09g014530.2.1	0	0	0	0	0	0	0	0	0	0	0	0	0
Solyc09g014550.2.1	0	0	0	0	0	0	0	0	0	0	0	0	0
Solyc09g014580.2.1	0	0	0	0	0	0	0	0	0	0	0	0	0
Solyc09g014590.2.1	0	0	0	0	0	0	0	0	0	0	0	0	0
Solyc09g014610.2.1	0	0	0	0	0	0	0	0	0	0	0	0	0
Solyc09g014620.2.1	0	0	0	0	0	0	0	0	0	0	0	0	0
Solyc09g014720.1.1	0	0	0	0	0	0	0	0	0	0	0	0	0
Solyc09g014740.2.1	1	4	0	1	0	0	4	2	2	2	0	5	0
Solyc09g014750.1.1	0	0	0	0	0	0	0	0	0	0	0	0	0
Solyc09g014760.2.1	10	235	51	211	4	298	289	409	9	314	39	266	3
Solyc09g014770.2.1	20	198	33	224	10	379	341	1001	45	641	66	341	7
Solyc09g014780.2.1	14	126	5	74	17	180	123	323	33	230	59	171	7
Solyc09g014790.2.1	19	387	82	399	15	855	664	1366	114	297	118	327	9
Solyc09g014820.2.1	0	0	0	0	0	0	0	0	0	0	0	0	0
Solyc09g014860.2.1	2	66	1	39	8	57	23	105	33	0	1	3	0
Solyc09g014890.2.1	2	5	1	1	0	8	7	10	0	15	1	12	0
Solyc09g014900.2.1	19	48	5	35	1	129	230	122	15	42	124	48	0
Solyc09g014910.2.1	0	0	0	0	0	0	0	0	0	0	0	0	0
Solyc09g014930.1.1	0	0	0	0	0	0	0	0	0	0	0	0	0
Solyc09g014940.1.1	0	0	0	0	0	0	0	0	0	0	0	0	0
Solyc09g014980.2.1	25	136	19	72	8	212	118	254	27	144	41	155	5
Solyc09g014990.2.1	1	28	10	16	0	12	0	12	0	0	0	6	0
Solyc09g015000.2.1	5	16	0	19	0	15	0	20	0	40	39	69	0
Solyc09g015020.1.1	0	0	0	0	0	0	0	0	0	0	0	0	0
Solyc09g015030.1.1	0	0	0	0	0	0	0	0	0	0	0	0	0
Solyc09g015040.1.1	17	476	31	217	2	183	129	158	55	62	52	90	3
Solyc09g015070.2.1	0	0	0	0	0	0	0	0	0	0	0	0	0

Solyc09g015080.2.1	1	4	0	8	0	3	0	2	0	7	3	9	0
Solyc09g015120.2.1	4	7	0	1	0	21	24	27	4	15	11	30	0
Solyc09g015160.2.1	1	4	1	0	0	10	3	11	1	2	1	11	0
Solyc09g015170.2.1	0	0	0	0	0	0	0	0	0	0	0	0	0
Solyc09g015180.2.1	4	14	2	8	0	41	44	21	5	46	18	26	0
Solyc09g015350.2.1	0	0	0	0	0	0	0	0	0	0	0	0	0
Solyc09g015360.2.1	5	95	6	73	0	93	57	88	2	32	8	36	0
Solyc09g015370.1.1	0	0	0	0	0	0	0	0	0	0	0	0	0
Solyc09g015380.1.1	0	0	0	0	0	0	0	0	0	0	0	0	0
Solyc09g015440.1.1	0	0	0	0	0	0	0	0	0	0	0	0	0
Solyc09g015450.2.1	0	0	0	0	0	0	0	0	0	0	0	0	0
Solyc09g015470.2.1	0	0	0	0	0	0	0	0	0	0	0	0	0
Solyc09g015490.2.1	0	0	0	0	0	0	0	0	0	0	0	0	0
Solyc09g015520.2.1	0	0	0	0	0	0	0	0	0	0	0	0	0
Solyc09g015530.2.1	9	33	3	30	1	108	46	54	25	104	22	55	0
Solyc09g015560.1.1	0	0	0	0	0	0	0	0	0	0	0	0	0
Solyc09g015570.1.1	0	0	0	0	0	0	0	0	0	0	0	0	0
Solyc09g015650.2.1	1	10	2	0	0	23	32	22	7	24	16	14	3
Solyc09g015660.2.1	2	6	1	6	0	6	8	9	0	9	3	9	0
Solyc09g015670.2.1	17	239	24	157	10	844	600	794	51	335	79	229	4
Solyc09g015770.2.1	8	178	92	236	19	138	103	64	4	172	11	223	3
Solyc09g015820.2.1	2	35	2	33	0	63	11	101	6	48	7	25	0
Solyc09g015830.1.1	8	446	18	212	25	248	113	205	38	247	93	263	2
Solyc09g015840.2.1	2	16	2	6	2	5	10	11	6	23	4	29	0
Solyc09g015850.2.1	0	0	0	0	0	0	0	0	0	0	0	0	0
Solyc09g015860.1.1	0	0	0	0	0	0	0	0	0	0	0	0	0
Solyc09g015870.2.1	0	0	0	0	0	0	0	0	0	0	0	0	0
Solyc09g015880.2.1	1	0	0	14	13	7	2	5	7	3	2	4	14
Solyc09g015890.2.1	0	0	0	0	0	0	0	0	0	0	0	0	0
Solyc09g015930.2.1	9	44	11	43	11	99	147	104	15	79	47	87	4
Solyc09g016940.2.1	5	23	2	10	0	51	17	45	11	29	14	23	3
Solyc09g016950.2.1	0	0	0	0	0	0	0	0	0	0	0	0	0
Solyc09g018010.2.1	8	652	56	29	306	11	191	29	235	50	34	1	70
Solyc09g018020.2.1	0	0	0	0	0	0	0	0	0	0	0	0	0
Solyc09g018030.2.1	1	3	0	2	0	16	3	1	1	2	0	1	0
Solyc09g018040.1.1	0	0	0	0	0	0	0	0	0	0	0	0	0
Solyc09g018060.2.1	2	4	2	6	0	26	49	19	9	17	6	4	0
Solyc09g018070.1.1	0	0	0	0	0	0	0	0	0	0	0	0	0
Solyc09g018160.2.1	4	5	2	9	0	10	22	8	1	23	8	20	0
Solyc09g018170.2.1	2	6	2	2	0	5	23	18	3	0	0	0	0
Solyc09g018220.1.1	0	0	0	0	0	0	0	0	0	0	0	0	0

Solyc09g018230.1.1	3	26	4	24	5	0	6	7	1	8	2	4	0
Solyc09g018280.1.1	15	127	65	111	9	239	524	428	15	512	95	188	4
Solyc09g018310.2.1	9	44	3	46	3	186	111	279	19	88	27	106	0
Solyc09g018340.2.1	0	0	0	0	0	0	0	0	0	0	0	0	0
Solyc09g018370.2.1	12	33	4	21	2	102	58	56	6	81	26	94	2
Solyc09g018430.2.1	0	0	0	0	0	0	0	0	0	0	0	0	0
Solyc09g018450.2.1	26	371	56	318	47	697	640	997	120	646	263	515	27
Solyc09g018460.1.1	0	0	0	0	0	0	0	0	0	0	0	0	0
Solyc09g018490.2.1	0	0	0	0	0	0	0	0	0	0	0	0	0
Solyc09g018500.1.1	0	0	0	0	0	0	0	0	0	0	0	0	0
Solyc09g018510.2.1	6	87	7	40	5	27	72	66	11	76	7	81	6
Solyc09g018630.2.1	0	0	0	0	0	0	0	0	0	0	0	0	0
Solyc09g018640.1.1	0	0	0	0	0	0	0	0	0	0	0	0	0
Solyc09g018650.2.1	1	4	1	0	0	6	20	13	2	2	4	2	0
Solyc09g018670.2.1	0	0	0	0	0	0	0	0	0	0	0	0	0
Solyc09g018690.2.1	6	6	6	14	0	31	61	27	7	15	8	28	17
Solyc09g018700.1.1	0	0	0	0	0	0	0	0	0	0	0	0	0
Solyc09g018710.2.1	0	0	0	0	0	0	0	0	0	0	0	0	0
Solyc09g018720.2.1	0	0	0	0	0	0	0	0	0	0	0	0	0
Solyc09g018730.2.1	4	39	1	28	0	58	21	75	3	86	18	75	1
Solyc09g018750.2.1	7	238	46	191	9	391	713	753	50	407	45	269	6
Solyc09g018760.2.1	1	3	0	0	0	1	4	3	0	0	0	3	0
Solyc09g018780.2.1	1	4	0	3	0	22	6	26	1	5	0	9	0
Solyc09g018790.2.1	5	402	18	166	5	181	76	161	14	403	52	524	4
Solyc09g018830.2.1	4	15	6	5	1	6	24	10	10	16	12	24	0
Solyc09g018840.2.1	1	5	0	0	0	0	4	0	0	5	1	14	0
Solyc09g018850.2.1	0	0	0	0	0	0	0	0	0	0	0	0	0
Solyc09g018890.2.1	3	144	39	51	0	1	28	9	0	3	0	1	0
Solyc09g018950.2.1	3	4	0	3	0	12	11	15	1	28	2	29	0
Solyc09g018960.2.1	15	68	3	72	7	230	69	379	23	239	24	165	0
Solyc09g019970.2.1	11	81	14	92	6	117	75	141	11	77	17	69	0
Solyc09g019980.2.1	1	4	0	2	0	2	4	12	3	3	0	2	0
Solyc09g020000.2.1	2	18	0	5	1	27	14	37	5	12	10	3	1
Solyc09g020010.1.1	2	5	0	1	0	17	19	17	5	5	1	10	0
Solyc09g020110.2.1	3	31	0	6	1	40	11	55	1	30	3	33	0
Solyc09g020130.2.1	8	102	11	66	4	173	132	189	26	240	56	375	2
Solyc09g020140.2.1	1	56	6	21	1	172	71	140	27	51	54	26	2
Solyc09g020150.2.1	0	0	0	0	0	0	0	0	0	0	0	0	0
Solyc09g020190.2.1	1	23	0	9	0	47	1	34	1	100	3	17	0
Solyc09g025200.1.1	0	0	0	0	0	0	0	0	0	0	0	0	0
Solyc09g025210.2.1	6	0	0	0	0	0	0	0	0	44	2024	37	21

Solyc09g025220.1.1	0	0	0	0	0	0	0	0	0	0	0	0	0
Solyc09g025230.2.1	0	0	0	0	0	0	0	0	0	0	0	0	0
Solyc09g025240.2.1	13	153	8	61	14	283	136	386	41	286	41	185	3
Solyc09g025250.2.1	2	21	0	5	0	32	15	59	3	41	12	20	0
Solyc09g025260.2.1	9	18	3	8	0	67	61	97	13	23	8	24	2
Solyc09g025270.2.1	15	192	38	163	3	126	112	211	34	187	54	165	4
Solyc09g025280.1.1	0	0	0	0	0	0	0	0	0	0	0	0	0
Solyc09g025310.2.1	0	0	0	0	0	0	0	0	0	0	0	0	0
Solyc09g030370.2.1	0	0	0	0	0	0	0	0	0	0	0	0	0
Solyc09g030390.2.1	19	95	13	69	18	90	163	208	28	189	69	124	10
Solyc09g030450.2.1	5	15	0	12	12	1	7	9	0	24	36	1	2
Solyc09g031580.2.1	7	23	2	14	3	32	41	63	0	53	12	50	1
Solyc09g031590.2.1	0	0	0	0	0	0	0	0	0	0	0	0	0
Solyc09g031600.2.1	8	23	0	12	0	42	48	66	8	68	12	16	0
Solyc09g031610.2.1	9	51	6	39	0	66	73	68	7	61	17	92	1
Solyc09g031650.2.1	40	343	46	248	33	787	493	1273	102	610	210	591	11
Solyc09g031680.1.1	1	2	0	2	0	4	5	4	0	12	3	3	1
Solyc09g031690.2.1	0	0	0	0	0	0	0	0	0	0	0	0	0
Solyc09g031700.2.1	6	42	7	18	0	93	51	131	9	32	7	39	0
Solyc09g031750.2.1	11	137	16	90	7	225	214	225	16	65	20	62	0
Solyc09g031760.1.1	0	0	0	0	0	0	0	0	0	0	0	0	0
Solyc09g031770.2.1	0	0	0	0	0	0	0	0	0	0	0	0	0
Solyc09g031780.2.1	20	165	16	166	0	329	210	601	44	404	98	365	6
Solyc09g031790.2.1	2	2	0	1	0	15	7	19	0	1	1	3	0
Solyc09g031900.2.1	0	0	0	0	0	0	0	0	0	0	0	0	0
Solyc09g031920.1.1	3	39	2	11	4	181	8	35	9	64	0	46	0
Solyc09g031930.2.1	3	51	5	20	3	37	61	100	8	119	19	86	1
Solyc09g031940.1.1	5	68	3	49	0	23	18	70	2	128	5	77	2
Solyc09g031970.2.1	31	929	286	524	29	1221	2650	1516	75	863	209	868	10
Solyc09g042230.1.1	0	0	0	0	0	0	0	0	0	0	0	0	0
Solyc09g042250.1.1	1	3	0	8	0	5	7	3	0	3	0	3	0
Solyc09g042260.2.1	8	35	5	21	0	121	51	123	12	45	7	46	1
Solyc09g042290.1.1	0	0	0	0	0	0	0	0	0	0	0	0	0
Solyc09g042660.2.1	0	0	0	0	0	0	0	0	0	0	0	0	0
Solyc09g042690.2.1	0	0	0	0	0	0	0	0	0	0	0	0	0
Solyc09g042700.2.1	9	165	9	90	1	129	296	185	28	93	21	55	0
Solyc09g042710.2.1	0	0	0	0	0	0	0	0	0	0	0	0	0
Solyc09g042740.2.1	12	125	7	85	2	167	123	316	22	276	42	185	4
Solyc09g042750.2.1	1	5	0	4	0	7	1	16	3	5	0	17	0
Solyc09g042760.1.1	0	0	0	0	0	0	0	0	0	0	0	0	0
Solyc09g042770.2.1	6	50	9	28	0	76	40	132	1	74	7	59	0

Solyc09g042780.2.1	0	0	0	0	0	0	0	0	0	0	0	0	0
Solyc09g047840.2.1	0	0	0	0	0	0	0	0	0	0	0	0	0
Solyc09g047870.2.1	2	4	2	1	0	10	10	5	0	0	3	1	0
Solyc09g047900.1.1	0	0	0	0	0	0	0	0	0	0	0	0	0
Solyc09g047910.2.1	0	0	0	0	0	0	0	0	0	0	0	0	0
Solyc09g047930.1.1	1	2	4	0	0	4	0	1	0	9	0	11	0
Solyc09g048970.1.1	0	0	0	0	0	0	0	0	0	0	0	0	0
Solyc09g048980.2.1	0	0	0	0	0	0	0	0	0	0	0	0	0
Solyc09g050020.1.1	0	0	0	0	0	0	0	0	0	0	0	0	0
Solyc09g050040.1.1	0	0	0	0	0	0	0	0	0	0	0	0	0
Solyc09g050110.1.1	0	0	0	0	0	0	0	0	0	0	0	0	0
Solyc09g055170.1.1	0	0	0	0	0	0	0	0	0	0	0	0	0
Solyc09g055180.2.1	5	15	2	15	2	31	58	68	12	42	7	12	2
Solyc09g055230.2.1	23	78	16	117	4	134	167	198	19	220	30	135	2
Solyc09g055260.2.1	27	65	16	70	10	426	354	409	19	188	60	142	3
Solyc09g055310.2.1	14	87	10	65	5	198	132	265	9	181	35	84	0
Solyc09g055320.1.1	0	0	0	0	0	0	0	0	0	0	0	0	0
Solyc09g055340.2.1	6	41	2	46	10	159	248	169	15	137	63	170	7
Solyc09g055350.2.1	4	37	5	21	0	111	121	144	15	30	21	25	0
Solyc09g055420.1.1	0	0	0	0	0	0	0	0	0	0	0	0	0
Solyc09g055470.1.1	0	0	0	0	0	0	0	0	0	0	0	0	0
Solyc09g055570.2.1	10	39	9	30	6	60	90	76	11	79	22	78	1
Solyc09g055590.2.1	1	2	0	1	0	5	0	5	2	3	2	2	0
Solyc09g055660.1.1	0	0	0	0	0	0	0	0	0	0	0	0	0
Solyc09g055680.1.1	0	0	0	0	0	0	0	0	0	0	0	0	0
Solyc09g055700.2.1	2	13	5	11	2	21	21	45	10	11	7	3	0
Solyc09g055710.2.1	1	1	0	1	3	0	1	8	2	5	4	5	0
Solyc09g055760.2.1	7	38	7	11	8	120	90	101	10	143	27	111	3
Solyc09g055830.1.1	0	0	0	0	0	0	0	0	0	0	0	0	0
Solyc09g055850.2.1	0	0	0	0	0	0	0	0	0	0	0	0	0
Solyc09g055870.2.1	9	64	3	29	2	123	45	155	9	137	20	84	0
Solyc09g055890.2.1	1	1	0	1	0	4	3	2	1	6	0	7	0
Solyc09g055900.2.1	1	7	0	1	0	37	2	14	2	2	2	4	0
Solyc09g055910.2.1	10	34	2	18	0	14	27	63	3	113	11	52	3
Solyc09g055920.2.1	1	1	0	2	0	3	3	4	0	8	0	3	0
Solyc09g055930.2.1	3	13	1	26	0	23	18	27	0	41	5	49	0
Solyc09g055940.2.1	5	40	0	5	0	50	32	59	7	58	16	67	0
Solyc09g055990.1.1	0	0	0	0	0	0	0	0	0	0	0	0	0
Solyc09g056160.2.1	10	93	7	40	2	114	50	162	5	222	20	224	0
Solyc09g056170.2.1	0	0	0	0	0	0	0	0	0	0	0	0	0
Solyc09g056180.2.1	0	0	0	0	0	0	0	0	0	0	0	0	0

Solyc09g056240.1.1	0	0	0	0	0	0	0	0	0	0	0	0	0
Solyc09g056260.2.1	5	12	2	15	3	22	51	22	1	14	7	13	0
Solyc09g056270.1.1	0	0	0	0	0	0	0	0	0	0	0	0	0
Solyc09g056340.2.1	4	48	1	10	7	25	23	21	7	93	4	67	1
Solyc09g056350.2.1	8	42	4	46	3	115	45	153	3	82	20	89	5
Solyc09g056360.2.1	0	0	0	0	0	0	0	0	0	0	0	0	0
Solyc09g056370.1.1	0	0	0	0	0	0	0	0	0	0	0	0	0
Solyc09g056380.1.1	0	0	0	0	0	0	0	0	0	0	0	0	0
Solyc09g056390.1.1	1	52	0	15	3	9	3	18	2	0	0	4	0
Solyc09g056430.2.1	0	0	0	0	0	0	0	0	0	0	0	0	0
Solyc09g056450.2.1	17	739	52	374	24	240	80	202	15	389	58	553	4
Solyc09g057570.2.1	0	0	0	0	0	0	0	0	0	0	0	0	0
Solyc09g057580.2.1	2	9	2	13	0	4	24	23	0	6	1	7	0
Solyc09g057630.2.1	3	40	3	0	13	2	23	7	0	53	7	36	0
Solyc09g057640.2.1	2	8	4	0	0	15	8	10	0	19	2	8	0
Solyc09g057650.2.1	6	213	16	107	9	268	84	346	26	759	76	426	7
Solyc09g057660.2.1	10	30	10	25	2	81	52	164	4	84	34	62	8
Solyc09g057670.2.1	3	122	4	45	4	135	54	220	28	212	45	174	3
Solyc09g057680.2.1	0	0	0	0	0	0	0	0	0	0	0	0	0
Solyc09g057710.2.1	4	7	3	4	0	2	5	6	1	10	5	31	2
Solyc09g057760.2.1	1	2	0	0	0	2	4	5	1	5	0	1	0
Solyc09g057790.2.1	0	0	0	0	0	0	0	0	0	0	0	0	0
Solyc09g057870.2.1	0	0	0	0	0	0	0	0	0	0	0	0	0
Solyc09g057880.2.1	0	0	0	0	0	0	0	0	0	0	0	0	0
Solyc09g057900.1.1	0	0	0	0	0	0	0	0	0	0	0	0	0
Solyc09g057910.2.1	2	2	0	3	2	11	7	3	3	7	6	15	0
Solyc09g057920.2.1	2	3	0	0	0	6	8	9	0	4	2	4	1
Solyc09g057950.2.1	0	0	0	0	0	0	0	0	0	0	0	0	0
Solyc09g057960.1.1	0	0	0	0	0	0	0	0	0	0	0	0	0
Solyc09g058980.2.1	0	0	0	0	0	0	0	0	0	0	0	0	0
Solyc09g058990.2.1	2	5	0	10	2	31	10	20	1	5	3	5	0
Solyc09g059020.2.1	0	0	0	0	0	0	0	0	0	0	0	0	0
Solyc09g059030.2.1	11	104	20	116	22	340	1795	534	176	421	236	385	2
Solyc09g059040.2.1	7	169	9	149	6	387	124	426	42	373	31	247	3
Solyc09g059170.1.1	1	14	0	3	3	0	2	0	0	8	3	7	0
Solyc09g059220.1.1	1	7	3	2	0	3	15	5	2	16	18	16	0
Solyc09g059240.2.1	16	266	66	58	18	91	5	35	0	0	3	9	0
Solyc09g059260.2.1	0	0	0	0	0	0	0	0	0	0	0	0	0
Solyc09g059270.2.1	3	20	3	8	1	35	37	94	4	66	14	52	0
Solyc09g059280.1.1	1	1	0	0	0	0	4	5	1	15	0	5	0
Solyc09g059290.2.1	0	0	0	0	0	0	0	0	0	0	0	0	0

Solyc09g059300.1.1	0	0	0	0	0	0	0	0	0	0	0	0	0
Solyc09g059310.2.1	11	98	5	67	7	112	63	183	23	136	60	180	1
Solyc09g059400.2.1	0	0	0	0	0	0	0	0	0	0	0	0	0
Solyc09g059430.2.1	3	15	5	17	0	16	2	11	2	37	8	15	0
Solyc09g059440.1.1	0	0	0	0	0	0	0	0	0	0	0	0	0
Solyc09g059450.2.1	5	35	1	30	6	50	45	129	8	34	12	35	2
Solyc09g059460.2.1	0	0	0	0	0	0	0	0	0	0	0	0	0
Solyc09g059470.2.1	0	0	0	0	0	0	0	0	0	0	0	0	0
Solyc09g059500.2.1	9	53	4	30	0	53	45	91	2	116	35	86	0
Solyc09g059510.2.1	3	81	3	30	9	25	10	24	4	16	8	7	5
Solyc09g059520.2.1	7	20	2	20	0	15	20	38	4	95	15	34	5
Solyc09g059540.1.1	0	0	0	0	0	0	0	0	0	0	0	0	0
Solyc09g059550.1.1	0	0	0	0	0	0	0	0	0	0	0	0	0
Solyc09g059570.2.1	0	0	0	0	0	0	0	0	0	0	0	0	0
Solyc09g059580.1.1	0	0	0	0	0	0	0	0	0	0	0	0	0
Solyc09g059600.2.1	12	48	7	37	1	131	163	110	19	62	22	80	2
Solyc09g059610.2.1	0	0	0	0	0	0	0	0	0	0	0	0	0
Solyc09g059620.2.1	3	27	0	29	0	27	22	80	3	57	8	66	2
Solyc09g059630.1.1	0	0	0	0	0	0	0	0	0	0	0	0	0
Solyc09g059670.2.1	1	2	0	0	0	2	3	5	0	20	0	17	0
Solyc09g059930.1.1	0	0	0	0	0	0	0	0	0	0	0	0	0
Solyc09g059950.2.1	0	0	0	0	0	0	0	0	0	0	0	0	0
Solyc09g059960.2.1	6	29	3	6	4	218	137	133	23	82	58	74	1
Solyc09g059970.2.1	0	0	0	0	0	0	0	0	0	0	0	0	0
Solyc09g059990.1.1	1	5	1	5	0	1	0	0	0	5	14	3	2
Solyc09g060080.2.1	10	28	5	23	5	81	77	71	15	52	8	44	3
Solyc09g060090.2.1	4	15	2	6	2	52	5	33	1	7	9	8	0
Solyc09g060100.2.1	5	67	13	59	1	12	17	24	1	13	7	4	0
Solyc09g060110.2.1	1	10	0	0	0	1	6	5	0	2	0	0	0
Solyc09g060120.1.1	0	0	0	0	0	0	0	0	0	0	0	0	0
Solyc09g061230.2.1	0	0	0	0	0	0	0	0	0	0	0	0	0
Solyc09g061250.2.1	1	0	0	3	0	2	9	3	0	4	1	1	0
Solyc09g061270.2.1	3	4	0	0	0	13	13	9	3	11	10	7	0
Solyc09g061280.2.1	4	19	1	8	0	139	2	31	0	4	15	4	0
Solyc09g061290.2.1	15	354	22	292	6	430	248	431	42	408	82	231	8
Solyc09g061310.2.1	12	223	31	88	17	929	184	438	55	186	155	192	13
Solyc09g061320.2.1	2	10	0	1	0	6	5	4	0	0	1	10	0
Solyc09g061340.1.1	0	0	0	0	0	0	0	0	0	0	0	0	0
Solyc09g061380.2.1	4	21	3	5	0	34	14	51	0	14	3	14	0
Solyc09g061410.1.1	2	23	1	30	0	46	22	31	1	7	5	12	0
Solyc09g061420.2.1	4	32	6	10	6	11	25	27	2	19	7	18	2

Solyc09g061430.2.1	3	28	2	5	0	58	11	107	3	30	6	43	0
Solyc09g061440.2.1	5	249	32	170	0	697	228	652	13	333	75	182	5
Solyc09g061620.2.1	2	24	2	8	3	29	21	34	6	59	17	48	0
Solyc09g061680.2.1	3	19	1	0	0	4	6	27	5	60	18	39	0
Solyc09g061700.2.1	0	0	0	0	0	0	0	0	0	0	0	0	0
Solyc09g061710.2.1	7	27	2	33	0	32	26	79	0	111	19	52	4
Solyc09g061720.2.1	0	0	0	0	0	0	0	0	0	0	0	0	0
Solyc09g061730.1.1	0	0	0	0	0	0	0	0	0	0	0	0	0
Solyc09g061750.1.1	5	18	3	25	0	33	22	62	9	73	13	71	0
Solyc09g061760.1.1	0	0	0	0	0	0	0	0	0	0	0	0	0
Solyc09g061790.1.1	0	0	0	0	0	0	0	0	0	0	0	0	0
Solyc09g061830.1.1	0	0	0	0	0	0	0	0	0	0	0	0	0
Solyc09g061840.2.1	4	22	1	13	2	92	31	76	1	24	14	13	0
Solyc09g061860.2.1	8	215	14	76	4	63	34	53	0	76	29	19	0
Solyc09g061890.2.1	2	22	2	0	1	9	8	4	3	99	0	6	0
Solyc09g061930.2.1	1	0	1	0	0	0	1	0	1	2	7	0	7
Solyc09g061940.1.1	0	0	0	0	0	0	0	0	0	0	0	0	0
Solyc09g062970.1.1	0	0	0	0	0	0	0	0	0	0	0	0	0
Solyc09g062980.1.1	0	0	0	0	0	0	0	0	0	0	0	0	0
Solyc09g063010.2.1	6	38	12	33	2	12	38	23	1	46	38	28	0
Solyc09g063020.2.1	2	9	1	2	0	7	5	11	1	5	1	9	0
Solyc09g063030.2.1	19	243	28	159	7	253	119	249	18	708	66	480	5
Solyc09g063070.2.1	6	30	3	9	3	28	69	76	14	29	2	23	0
Solyc09g063080.1.1	9	78	3	68	7	376	90	291	4	250	37	138	0
Solyc09g063090.2.1	3	8	2	10	0	25	22	51	2	32	4	18	0
Solyc09g063130.2.1	2	1282	138	616	14	101	394	248	5	99	68	92	3
Solyc09g063140.2.1	6	96	12	43	0	56	64	97	2	78	6	68	5
Solyc09g063150.2.1	4	6	0	2	0	69	42	75	2	41	6	27	0
Solyc09g064200.2.1	14	149	11	136	12	320	149	371	46	239	87	141	4
Solyc09g064230.1.1	0	0	0	0	0	0	0	0	0	0	0	0	0
Solyc09g064240.2.1	3	13	4	5	0	22	6	18	0	31	4	22	0
Solyc09g064270.2.1	1	3	0	4	1	1	5	6	0	2	0	1	0
Solyc09g064280.2.1	0	0	0	0	0	0	0	0	0	0	0	0	0
Solyc09g064310.2.1	1	1	0	2	0	7	1	13	2	47	5	13	0
Solyc09g064320.2.1	1	2	0	0	1	1	1	10	0	4	4	3	0
Solyc09g064370.2.1	5	318	21	182	8	1107	329	964	26	792	93	537	2
Solyc09g064380.2.1	1	8	0	2	0	1	3	2	1	5	1	9	0
Solyc09g064390.2.1	6	3	3	23	2	9	7	15	1	48	37	139	12
Solyc09g064420.2.1	6	16	4	14	0	55	16	44	0	45	13	42	0
Solyc09g064430.2.1	12	81	15	34	0	78	78	88	10	83	25	52	1
Solyc09g064440.2.1	25	92	9	44	5	381	390	310	32	186	35	143	5

Solyc09g064450.2.1	4	102	7	96	6	466	160	433	32	445	87	208	3
Solyc09g064460.1.1	0	0	0	0	0	0	0	0	0	0	0	0	0
Solyc09g064470.2.1	5	90	11	47	13	65	48	57	17	174	9	134	7
Solyc09g064480.1.1	0	0	0	0	0	0	0	0	0	0	0	0	0
Solyc09g064500.2.1	3	125	16	45	1	2	42	5	0	59	30	57	2
Solyc09g064510.2.1	7	52	14	71	17	36	79	50	23	20	10	20	0
Solyc09g064520.2.1	4	8	0	4	1	18	5	28	1	15	16	8	0
Solyc09g064530.2.1	5	17	0	15	0	36	13	17	0	22	16	18	0
Solyc09g064540.2.1	5	32	7	40	4	48	49	75	5	34	6	21	0
Solyc09g064590.2.1	6	24	2	23	6	109	38	131	5	59	28	100	3
Solyc09g064600.2.1	0	0	0	0	0	0	0	0	0	0	0	0	0
Solyc09g064610.2.1	4	16	3	15	1	20	20	24	3	14	2	23	0
Solyc09g064630.2.1	7	514	46	379	0	467	25	385	8	65	16	56	0
Solyc09g064660.2.1	0	0	0	0	0	0	0	0	0	0	0	0	0
Solyc09g064720.2.1	12	275	6	165	13	317	83	150	15	595	48	577	7
Solyc09g064740.2.1	2	1	0	1	0	9	5	4	0	3	0	0	0
Solyc09g064750.2.1	0	0	0	0	0	0	0	0	0	0	0	0	0
Solyc09g064780.2.1	0	0	0	0	0	0	0	0	0	0	0	0	0
Solyc09g064790.2.1	0	0	0	0	0	0	0	0	0	0	0	0	0
Solyc09g064800.1.1	17	67	5	30	1	96	99	93	5	89	45	66	5
Solyc09g064810.2.1	0	0	0	0	0	0	0	0	0	0	0	0	0
Solyc09g064820.1.1	2	14	0	2	1	21	55	15	2	0	0	0	0
Solyc09g064840.2.1	10	26	5	34	1	83	58	105	15	87	7	58	1
Solyc09g064850.2.1	7	38	2	31	0	77	53	86	15	27	13	47	1
Solyc09g064860.2.1	13	151	19	99	16	311	233	406	34	355	33	334	8
Solyc09g064870.2.1	0	0	0	0	0	0	0	0	0	0	0	0	0
Solyc09g064890.2.1	0	0	0	0	0	0	0	0	0	0	0	0	0
Solyc09g064900.1.1	0	0	0	0	0	0	0	0	0	0	0	0	0
Solyc09g064910.1.1	0	0	0	0	0	0	0	0	0	0	0	0	0
Solyc09g064920.2.1	5	13	2	7	2	28	26	13	2	36	7	37	1
Solyc09g064930.2.1	5	140	3	68	0	188	39	215	7	155	22	134	4
Solyc09g064940.2.1	6	91	0	57	0	820	63	400	1	498	31	163	0
Solyc09g065000.2.1	10	15	3	12	0	90	112	69	8	45	19	43	0
Solyc09g065010.2.1	9	35	5	24	4	222	44	165	9	55	14	29	5
Solyc09g065020.2.1	1	1	0	0	0	11	10	8	0	3	3	0	0
Solyc09g065030.2.1	3	12	3	16	0	83	17	118	1	8	2	31	0
Solyc09g065040.1.1	0	0	0	0	0	0	0	0	0	0	0	0	0
Solyc09g065070.1.1	0	0	0	0	0	0	0	0	0	0	0	0	0
Solyc09g065090.1.1	0	0	0	0	0	0	0	0	0	0	0	0	0
Solyc09g065100.1.1	1	5	0	4	0	0	0	0	0	0	7	0	0
Solyc09g065110.2.1	1	2	0	4	0	7	4	12	3	18	0	11	0

Solyc09g065120.2.1	13	78	14	81	7	90	73	149	6	160	27	238	0
Solyc09g065130.2.1	12	104	7	59	2	152	97	286	20	198	35	162	12
Solyc09g065150.1.1	2	10	1	18	0	1	8	16	0	10	3	10	0
Solyc09g065160.2.1	6	33	14	21	0	5	40	23	2	29	13	24	1
Solyc09g065180.2.1	7	413	50	230	1	47	91	103	5	71	15	86	0
Solyc09g065190.1.1	10	16	4	7	1	40	65	48	7	40	17	47	3
Solyc09g065200.2.1	2	22	1	5	1	10	8	36	17	10	1	9	0
Solyc09g065210.2.1	12	99	7	70	10	165	55	180	14	232	30	225	10
Solyc09g065240.2.1	7	3	0	1	0	1	2	2	0	56	33	84	0
Solyc09g065260.1.1	0	0	0	0	0	0	0	0	0	0	0	0	0
Solyc09g065270.2.1	6	352	31	150	5	73	138	155	3	99	17	130	5
Solyc09g065280.2.1	23	263	31	207	16	676	358	717	101	753	163	546	15
Solyc09g065290.2.1	4	5	0	0	0	40	48	23	1	25	14	21	0
Solyc09g065300.2.1	2	9	2	6	0	10	22	19	0	5	0	1	0
Solyc09g065330.2.1	1	38	1	13	0	69	4	53	1	38	6	21	0
Solyc09g065340.2.1	20	49	5	44	2	58	74	92	18	72	32	63	3
Solyc09g065370.1.1	5	75	8	38	0	194	355	247	5	69	11	68	5
Solyc09g065380.1.1	6	107	24	68	5	334	270	424	88	412	91	247	4
Solyc09g065390.1.1	3	11	0	9	0	24	30	48	0	112	0	59	0
Solyc09g065400.1.1	5	17	0	3	3	78	18	40	2	21	3	15	3
Solyc09g065410.2.1	0	0	0	0	0	0	0	0	0	0	0	0	0
Solyc09g065420.2.1	0	0	0	0	0	0	0	0	0	0	0	0	0
Solyc09g065430.2.1	1	0	0	0	1	9	39	2	7	0	7	0	0
Solyc09g065440.2.1	0	0	0	0	0	0	0	0	0	0	0	0	0
Solyc09g065470.2.1	0	0	0	0	0	0	0	0	0	0	0	0	0
Solyc09g065480.1.1	0	0	0	0	0	0	0	0	0	0	0	0	0
Solyc09g065510.2.1	1	17	0	10	0	16	4	16	0	23	3	10	0
Solyc09g065520.2.1	5	8	7	11	0	59	57	61	40	38	13	41	1
Solyc09g065540.2.1	18	44	8	50	11	644	1141	646	164	278	71	187	49
Solyc09g065550.2.1	28	268	17	183	15	474	260	626	81	378	136	353	15
Solyc09g065560.2.1	19	73	55	66	0	1416	367	734	4	23	6	1	1
Solyc09g065570.2.1	0	0	0	0	0	0	0	0	0	0	0	0	0
Solyc09g065580.2.1	0	0	0	0	0	0	0	0	0	0	0	0	0
Solyc09g065590.2.1	3	8	0	8	0	3	5	7	2	11	21	6	0
Solyc09g065600.2.1	0	0	0	0	0	0	0	0	0	0	0	0	0
Solyc09g065610.1.1	3	19	1	11	4	15	15	30	2	27	3	38	0
Solyc09g065620.2.1	0	0	0	0	0	0	0	0	0	0	0	0	0
Solyc09g065630.2.1	3	14	1	2	1	11	26	22	7	54	5	17	0
Solyc09g065640.2.1	14	196	13	117	22	187	221	360	54	123	35	107	7
Solyc09g065650.2.1	8	17	3	13	3	29	101	46	8	39	21	21	6
Solyc09g065660.2.1	0	0	0	0	0	0	0	0	0	0	0	0	0

Solyc09g065670.2.1	3	2	0	0	0	0	0	1	0	47	10	22	0
Solyc09g065690.2.1	3	4	2	0	0	11	37	11	2	9	8	11	0
Solyc09g065700.2.1	16	34	11	47	5	109	102	173	18	123	31	104	6
Solyc09g065710.1.1	0	0	0	0	0	0	0	0	0	0	0	0	0
Solyc09g065720.2.1	12	33	4	20	0	36	48	91	8	94	18	68	2
Solyc09g065730.2.1	3	32	4	6	0	33	36	58	8	84	13	99	0
Solyc09g065740.1.1	0	0	0	0	0	0	0	0	0	0	0	0	0
Solyc09g065750.2.1	0	0	0	0	0	0	0	0	0	0	0	0	0
Solyc09g065760.2.1	15	21	5	8	5	148	90	138	11	55	45	116	0
Solyc09g065780.2.1	0	0	0	0	0	0	0	0	0	0	0	0	0
Solyc09g065800.2.1	0	0	0	0	0	0	0	0	0	0	0	0	0
Solyc09g065810.2.1	2	15	3	13	0	15	17	17	5	16	3	32	0
Solyc09g065820.2.1	1	12	4	12	0	28	25	26	1	11	9	13	2
Solyc09g065830.2.1	5	153	10	113	28	205	193	274	38	307	94	276	19
Solyc09g065840.2.1	6	29	2	14	0	29	41	26	1	70	1	72	2
Solyc09g065850.2.1	7	255	12	140	5	125	40	55	4	82	56	90	7
Solyc09g065860.2.1	2	19	2	14	5	9	20	17	0	69	1	31	0
Solyc09g065870.2.1	2	3	0	5	0	13	21	30	0	11	5	9	5
Solyc09g065880.2.1	1	1	0	0	0	0	1	0	0	3	4	9	0
Solyc09g065890.2.1	10	45	50	38	21	32	398	44	114	6	49	11	12
Solyc09g065900.2.1	9	272	29	219	7	360	159	409	10	129	57	164	1
Solyc09g065910.1.1	1	1008	62	557	8	80	385	92	10	382	64	149	5
Solyc09g065920.2.1	10	55	4	31	0	132	58	114	8	83	12	70	0
Solyc09g065930.2.1	0	0	0	0	0	0	0	0	0	0	0	0	0
Solyc09g065940.2.1	0	0	0	0	0	0	0	0	0	0	0	0	0
Solyc09g065950.2.1	4	27	2	20	0	63	33	52	3	60	0	50	0
Solyc09g065990.2.1	3	3	0	10	0	9	10	17	2	27	0	27	0
Solyc09g066000.1.1	2	1	0	0	0	3	4	2	5	4	6	5	0
Solyc09g066010.2.1	3	3	4	0	0	6	4	11	0	6	4	4	0
Solyc09g066050.2.1	0	0	0	0	0	0	0	0	0	0	0	0	0
Solyc09g066080.1.1	0	0	0	0	0	0	0	0	0	0	0	0	0
Solyc09g066090.2.1	0	0	0	0	0	0	0	0	0	0	0	0	0
Solyc09g066100.2.1	7	64	2	50	2	19	59	61	2	188	7	69	0
Solyc09g066120.2.1	0	0	0	0	0	0	0	0	0	0	0	0	0
Solyc09g066130.2.1	9	23	2	23	0	34	28	43	1	44	10	26	0
Solyc09g066140.2.1	5	9	6	1	0	6	34	16	2	18	9	16	0
Solyc09g066150.1.1	19	50	11	34	0	14	243	35	1	251	173	124	3
Solyc09g066210.2.1	1	2	0	0	0	3	5	2	0	0	0	2	0
Solyc09g066280.2.1	2	6	1	1	0	13	3	8	0	37	5	50	3
Solyc09g066290.2.1	3	0	0	2	0	8	8	14	0	14	7	12	0
Solyc09g066410.1.1	0	0	0	0	0	0	0	0	0	0	0	0	0

Solyc09g066420.2.1	1	0	0	0	0	14	4	2	0	3	0	4	0
Solyc09g066430.2.1	1	78	7	17	0	49	44	104	8	82	14	70	5
Solyc09g066440.2.1	4	34	3	15	2	102	29	93	3	32	8	41	0
Solyc09g066460.2.1	4	5	0	1	0	22	19	20	1	16	8	19	1
Solyc09g066470.2.1	3	16	0	15	0	17	26	10	0	30	8	12	0
Solyc09g066480.2.1	23	64	13	49	8	147	114	193	31	93	21	86	4
Solyc09g066490.2.1	3	28	1	5	2	23	8	29	4	61	23	76	6
Solyc09g066500.2.1	3	43	2	10	3	80	27	64	8	49	8	81	0
Solyc09g072560.2.1	3	1	1	0	0	0	0	0	0	65	1829	23	18
Solyc09g072570.2.1	3	27	6	46	16	108	105	83	10	49	32	64	1
Solyc09g072580.2.1	0	0	0	0	0	0	0	0	0	0	0	0	0
Solyc09g072590.2.1	0	0	0	0	0	0	0	0	0	0	0	0	0
Solyc09g072610.2.1	0	0	0	0	0	0	0	0	0	0	0	0	0
Solyc09g072620.2.1	5	27	2	3	0	12	35	34	2	35	2	41	0
Solyc09g072630.2.1	0	0	0	0	0	0	0	0	0	0	0	0	0
Solyc09g072640.2.1	0	0	0	0	0	0	0	0	0	0	0	0	0
Solyc09g072650.2.1	2	7	1	18	1	5	4	23	1	11	3	10	0
Solyc09g072660.2.1	4	8	0	5	0	21	26	28	0	0	0	0	0
Solyc09g072670.2.1	1	4	0	8	0	7	12	7	6	4	3	2	0
Solyc09g072680.1.1	5	12	1	7	4	30	68	58	4	37	6	39	1
Solyc09g072690.1.1	0	0	0	0	0	0	0	0	0	0	0	0	0
Solyc09g072700.2.1	0	0	0	0	0	0	0	0	0	0	0	0	0
Solyc09g072710.2.1	1	72	8	15	0	45	48	15	3	31	16	11	0
Solyc09g072720.1.1	0	0	0	0	0	0	0	0	0	0	0	0	0
Solyc09g072770.1.1	0	0	0	0	0	0	0	0	0	0	0	0	0
Solyc09g072790.2.1	0	0	0	0	0	0	0	0	0	0	0	0	0
Solyc09g072800.2.1	0	0	0	0	0	0	0	0	0	0	0	0	0
Solyc09g072810.2.1	0	0	0	0	0	0	0	0	0	0	0	0	0
Solyc09g072820.2.1	10	89	3	88	1	80	62	152	0	7	32	18	0
Solyc09g072830.2.1	8	82	5	31	0	261	79	274	21	37	8	38	1
Solyc09g072850.1.1	2	8	0	1	0	16	6	17	1	5	0	7	0
Solyc09g072860.1.1	1	1	0	2	0	4	4	4	0	4	0	3	0
Solyc09g072870.2.1	1	74	2	17	0	101	8	83	4	289	13	188	0
Solyc09g072880.2.1	4	31	7	20	4	94	51	41	7	38	22	8	1
Solyc09g072890.1.1	12	26	15	12	17	53	241	54	41	94	91	68	4
Solyc09g072900.2.1	6	84	9	50	0	166	98	111	5	106	42	108	2
Solyc09g072970.2.1	5	51	12	22	0	54	84	61	14	162	19	42	9
Solyc09g072980.2.1	2	41	1	5	4	73	17	34	0	19	7	18	0
Solyc09g072990.2.1	15	378	132	318	9	134	135	189	21	156	159	300	3
Solyc09g073000.2.1	5	35	1	9	6	103	50	186	8	93	8	73	1
Solyc09g073030.2.1	2	5	0	4	0	23	8	16	0	8	0	11	0

Solyc09g073040.2.1	0	0	0	0	0	0	0	0	0	0	0	0	0
Solyc09g074040.1.1	0	0	0	0	0	0	0	0	0	0	0	0	0
Solyc09g074050.2.1	6	61	2	29	7	141	17	112	18	10	26	10	0
Solyc09g074060.2.1	12	238	14	108	6	210	60	304	11	356	77	256	1
Solyc09g074080.1.1	0	0	0	0	0	0	0	0	0	0	0	0	0
Solyc09g074090.2.1	1	3	0	3	0	12	0	6	0	4	3	11	0
Solyc09g074100.2.1	7	18	5	30	0	33	108	60	4	51	6	45	0
Solyc09g074110.2.1	9	34	14	70	7	277	283	319	15	82	24	102	2
Solyc09g074180.2.1	0	0	0	0	0	0	0	0	0	0	0	0	0
Solyc09g074210.2.1	3	5	2	5	1	24	2	9	3	25	19	40	1
Solyc09g074230.2.1	4	40	0	20	3	97	8	37	0	33	23	40	0
Solyc09g074240.1.1	2	7	2	14	1	8	3	7	1	11	6	8	0
Solyc09g074250.2.1	0	0	0	0	0	0	0	0	0	0	0	0	0
Solyc09g074260.1.1	0	0	0	0	0	0	0	0	0	0	0	0	0
Solyc09g074270.2.1	10	62	34	22	16	299	172	140	20	17	79	36	1
Solyc09g074280.1.1	0	0	0	0	0	0	0	0	0	0	0	0	0
Solyc09g074300.1.1	0	0	0	0	0	0	0	0	0	0	0	0	0
Solyc09g074310.2.1	1	4	0	0	0	12	11	0	0	4	0	9	0
Solyc09g074320.2.1	13	110	16	84	0	556	805	675	50	261	168	223	5
Solyc09g074330.2.1	9	70	5	74	6	152	72	147	12	135	12	77	0
Solyc09g074340.2.1	0	0	0	0	0	0	0	0	0	0	0	0	0
Solyc09g074350.1.1	2	10	1	2	0	25	25	3	9	0	1	1	0
Solyc09g074360.2.1	3	28	1	4	0	10	18	18	1	33	2	13	0
Solyc09g074370.2.1	2	5	1	0	4	16	12	10	0	1	4	0	0
Solyc09g074380.2.1	15	67	14	40	7	100	142	140	24	80	43	42	2
Solyc09g074400.2.1	11	81	2	93	3	121	61	227	15	169	45	149	1
Solyc09g074410.2.1	4	13	0	38	4	7	6	3	5	22	7	11	0
Solyc09g074420.2.1	5	53	19	43	5	4	95	23	4	42	3	74	6
Solyc09g074430.2.1	0	0	0	0	0	0	0	0	0	0	0	0	0
Solyc09g074450.1.1	0	0	0	0	0	0	0	0	0	0	0	0	0
Solyc09g074460.1.1	7	235	20	113	6	232	102	214	33	755	100	427	3
Solyc09g074470.2.1	0	0	0	0	0	0	0	0	0	0	0	0	0
Solyc09g074510.2.1	9	142	17	90	19	208	101	257	28	206	62	120	1
Solyc09g074520.2.1	8	118	18	107	2	47	69	62	6	279	67	176	6
Solyc09g074530.2.1	0	0	0	0	0	0	0	0	0	0	0	0	0
Solyc09g074550.2.1	0	0	0	0	0	0	0	0	0	0	0	0	0
Solyc09g074560.2.1	14	96	1	66	8	129	83	91	24	224	63	237	13
Solyc09g074570.1.1	1	98	2	32	0	92	1	19	0	8	0	16	0
Solyc09g074610.2.1	4	141	15	56	1	21	125	27	0	15	2	3	0
Solyc09g074620.2.1	1	1	0	0	0	6	5	8	0	2	2	0	0
Solyc09g074630.2.1	3	5	0	14	0	0	9	5	0	64	17	115	0

Solyc09g074640.2.1	2	7	0	6	3	15	7	19	1	26	6	22	0
Solyc09g074650.2.1	6	37	5	10	0	23	30	46	1	34	6	17	0
Solyc09g074660.2.1	16	92	5	62	3	436	137	395	20	101	46	141	0
Solyc09g074670.2.1	8	24	2	22	1	94	42	82	7	40	13	33	0
Solyc09g074680.2.1	11	61	11	33	3	179	90	191	14	127	40	141	1
Solyc09g074690.2.1	2	6	0	6	0	5	6	8	1	7	1	6	1
Solyc09g074700.1.1	0	0	0	0	0	0	0	0	0	0	0	0	0
Solyc09g074710.2.1	3	9	0	5	0	17	8	10	2	29	7	11	0
Solyc09g074720.1.1	0	0	0	0	0	0	0	0	0	0	0	0	0
Solyc09g074730.1.1	1	4	0	0	0	17	6	10	0	3	1	1	0
Solyc09g074740.2.1	2	1	1	1	0	5	18	7	1	10	7	20	0
Solyc09g074750.2.1	1	1	0	0	1	5	10	5	0	12	3	16	0
Solyc09g074780.2.1	0	0	0	0	0	0	0	0	0	0	0	0	0
Solyc09g074820.2.1	0	0	0	0	0	0	0	0	0	0	0	0	0
Solyc09g074830.2.1	0	0	0	0	0	0	0	0	0	0	0	0	0
Solyc09g074840.2.1	0	0	0	0	0	0	0	0	0	0	0	0	0
Solyc09g074850.2.1	1	0	3	0	0	1	1	2	0	14	4	6	0
Solyc09g074860.2.1	0	0	0	0	0	0	0	0	0	0	0	0	0
Solyc09g074870.2.1	3	31	1	18	1	94	10	50	2	132	9	93	0
Solyc09g074880.2.1	4	63	11	37	0	16	17	33	0	72	5	73	0
Solyc09g074890.1.1	0	0	0	0	0	0	0	0	0	0	0	0	0
Solyc09g074900.2.1	8	24	2	8	0	47	18	76	1	31	15	17	0
Solyc09g074910.1.1	4	10	1	4	0	250	25	107	2	27	12	28	4
Solyc09g074920.2.1	0	0	0	0	0	0	0	0	0	0	0	0	0
Solyc09g074930.2.1	11	59	13	55	18	42	41	79	29	496	64	256	9
Solyc09g074940.1.1	15	191	24	186	6	302	264	334	18	566	105	514	13
Solyc09g074950.2.1	8	106	10	87	12	166	135	234	26	299	50	219	1
Solyc09g074970.2.1	0	0	0	0	0	0	0	0	0	0	0	0	0
Solyc09g074980.2.1	1	0	1	3	0	0	1	7	0	5	0	1	0
Solyc09g074990.2.1	0	0	0	0	0	0	0	0	0	0	0	0	0
Solyc09g075000.2.1	3	5	0	2	0	17	14	7	0	25	6	27	0
Solyc09g075010.2.1	3	161	22	95	18	538	316	488	80	364	126	209	0
Solyc09g075020.2.1	47	1887	141	1804	25	5721	1004	4086	86	180	256	184	11
Solyc09g075030.2.1	3	17	0	9	0	38	11	27	0	44	7	34	0
Solyc09g075040.2.1	0	0	0	0	0	0	0	0	0	0	0	0	0
Solyc09g075060.2.1	0	0	0	0	0	0	0	0	0	0	0	0	0
Solyc09g075070.2.1	8	20	1	20	0	94	59	113	4	19	5	10	0
Solyc09g075080.2.1	4	32	1	16	0	30	7	21	2	23	13	29	0
Solyc09g075090.1.1	4	75	5	50	4	163	35	181	3	248	24	133	6
Solyc09g075100.2.1	6	26	1	16	0	106	33	58	6	48	21	68	3
Solyc09g075110.1.1	2	10	2	3	0	9	10	10	0	24	6	28	0

Solyc09g075120.2.1	5	59	12	31	2	79	10	90	14	82	49	86	7
Solyc09g075130.2.1	2	61	0	16	0	34	5	80	3	102	2	64	0
Solyc09g075140.2.1	10	51	28	80	9	780	840	455	91	115	32	80	2
Solyc09g075150.2.1	6	123	4	63	7	178	122	232	32	263	75	286	3
Solyc09g075160.2.1	1	32	0	26	0	44	4	46	1	18	1	15	0
Solyc09g075170.1.1	0	0	0	0	0	0	0	0	0	0	0	0	0
Solyc09g075180.2.1	10	246	54	102	14	254	229	289	15	166	77	137	0
Solyc09g075190.2.1	1	1	3	3	0	0	1	9	1	10	0	6	0
Solyc09g075200.2.1	5	75	2	25	2	106	6	143	6	97	20	85	0
Solyc09g075210.2.1	2	255	14	93	6	57	74	139	4	4321	491	3051	30
Solyc09g075220.1.1	5	45	4	12	2	54	42	51	10	122	18	92	5
Solyc09g075230.1.1	3	62	5	27	0	33	20	60	13	39	17	26	1
Solyc09g075240.1.1	0	0	0	0	0	0	0	0	0	0	0	0	0
Solyc09g075260.2.1	0	0	0	0	0	0	0	0	0	0	0	0	0
Solyc09g075270.1.1	0	0	0	0	0	0	0	0	0	0	0	0	0
Solyc09g075280.1.1	16	145	23	104	15	190	184	194	29	124	72	141	10
Solyc09g075290.2.1	3	122	9	36	0	113	9	104	6	304	18	174	1
Solyc09g075300.2.1	0	0	0	0	0	0	0	0	0	0	0	0	0
Solyc09g075320.1.1	0	0	0	0	0	0	0	0	0	0	0	0	0
Solyc09g075330.2.1	0	0	0	0	0	0	0	0	0	0	0	0	0
Solyc09g075350.2.1	1	9	0	6	0	4	2	7	6	0	0	0	0
Solyc09g075360.2.1	0	0	0	0	0	0	0	0	0	0	0	0	0
Solyc09g075370.2.1	1	2	0	4	0	10	1	9	0	5	2	10	0
Solyc09g075380.1.1	0	0	0	0	0	0	0	0	0	0	0	0	0
Solyc09g075390.2.1	13	126	21	113	2	121	79	129	3	137	15	287	4
Solyc09g075400.2.1	0	0	0	0	0	0	0	0	0	0	0	0	0
Solyc09g075420.2.1	7	30	1	22	0	33	13	36	1	94	59	47	1
Solyc09g075430.2.1	10	756	62	310	66	846	409	1103	205	1187	294	721	162
Solyc09g075440.2.1	16	95	3	85	5	684	210	483	23	223	218	142	2
Solyc09g075450.2.1	6	66	4	35	1	120	43	110	18	143	31	57	7
Solyc09g075460.2.1	6	174	4	126	1	35	37	59	12	630	45	384	1
Solyc09g075470.2.1	0	0	0	0	0	0	0	0	0	0	0	0	0
Solyc09g075480.2.1	0	0	0	0	0	0	0	0	0	0	0	0	0
Solyc09g075490.2.1	6	91	3	58	6	146	81	172	9	53	20	71	0
Solyc09g075500.2.1	0	0	0	0	0	0	0	0	0	0	0	0	0
Solyc09g075520.1.1	1	3	0	2	0	1	0	2	0	0	0	4	0
Solyc09g075530.2.1	3	9	0	6	0	12	11	4	1	1	5	23	0
Solyc09g075540.1.1	0	0	0	0	0	0	0	0	0	0	0	0	0
Solyc09g075550.2.1	0	0	0	0	0	0	0	0	0	0	0	0	0
Solyc09g075560.1.1	1	3	1	3	0	0	7	0	0	5	0	3	0
Solyc09g075570.1.1	2	15	3	5	0	10	12	34	2	22	5	9	0

Solyc09g075580.1.1	2	4	0	8	0	7	11	5	0	0	1	3	0
Solyc09g075590.1.1	0	0	0	0	0	0	0	0	0	0	0	0	0
Solyc09g075600.1.1	0	0	0	0	0	0	0	0	0	0	0	0	0
Solyc09g075630.1.1	0	0	0	0	0	0	0	0	0	0	0	0	0
Solyc09g075640.2.1	18	68	9	64	0	85	112	117	7	61	25	79	0
Solyc09g075650.2.1	0	0	0	0	0	0	0	0	0	0	0	0	0
Solyc09g075660.2.1	0	0	0	0	0	0	0	0	0	0	0	0	0
Solyc09g075670.1.1	2	8	4	0	0	9	14	9	1	0	0	1	0
Solyc09g075680.1.1	0	0	0	0	0	0	0	0	0	0	0	0	0
Solyc09g075690.1.1	0	0	0	0	0	0	0	0	0	0	0	0	0
Solyc09g075700.1.1	0	0	0	0	0	0	0	0	0	0	0	0	0
Solyc09g075710.1.1	0	0	0	0	0	0	0	0	0	0	0	0	0
Solyc09g075720.2.1	3	13	0	15	0	9	15	31	4	5	2	16	1
Solyc09g075730.2.1	0	0	0	0	0	0	0	0	0	0	0	0	0
Solyc09g075750.1.1	4	46	3	36	7	86	27	59	20	0	0	0	0
Solyc09g075760.2.1	11	83	17	61	8	217	193	251	54	154	41	100	3
Solyc09g075770.1.1	2	2	1	0	3	98	36	9	39	2	2	1	0
Solyc09g075790.2.1	0	0	0	0	0	0	0	0	0	0	0	0	0
Solyc09g075800.2.1	13	64	2	69	7	237	82	216	38	93	37	74	6
Solyc09g075810.2.1	2	26	0	6	0	12	4	30	0	28	0	6	0
Solyc09g075820.2.1	2	11	0	5	1	120	5	68	8	16	2	1	0
Solyc09g075830.2.1	47	664	101	601	79	2381	1681	2656	373	1326	313	1104	24
Solyc09g075850.2.1	7	32	3	36	3	60	59	58	12	54	4	26	4
Solyc09g075860.2.1	1	22	1	2	1	9	3	13	0	13	0	7	0
Solyc09g075870.1.1	0	0	0	0	0	0	0	0	0	0	0	0	0
Solyc09g075880.2.1	0	0	0	0	0	0	0	0	0	0	0	0	0
Solyc09g075890.2.1	3	24	24	12	11	23	332	11	22	71	41	143	4
Solyc09g075900.2.1	0	0	0	0	0	0	0	0	0	0	0	0	0
Solyc09g075910.1.1	0	0	0	0	0	0	0	0	0	0	0	0	0
Solyc09g075920.1.1	0	0	0	0	0	0	0	0	0	0	0	0	0
Solyc09g075930.1.1	0	0	0	0	0	0	0	0	0	0	0	0	0
Solyc09g075940.2.1	0	0	0	0	0	0	0	0	0	0	0	0	0
Solyc09g075950.1.1	6	4	3	3	0	2	2	11	3	51	25	48	1
Solyc09g075960.1.1	0	0	0	0	0	0	0	0	0	0	0	0	0
Solyc09g075970.2.1	0	0	0	0	0	0	0	0	0	0	0	0	0
Solyc09g075980.2.1	0	0	0	0	0	0	0	0	0	0	0	0	0
Solyc09g075990.2.1	1	0	0	1	2	2	6	9	1	4	5	4	0
Solyc09g076000.2.1	6	78	6	22	1	86	46	50	13	31	4	29	0
Solyc09g076010.2.1	17	50	9	45	13	123	58	100	8	112	42	75	6
Solyc09g076020.2.1	3	42	2	17	0	43	41	57	1	37	8	46	0
Solyc09g076030.2.1	6	66	9	30	0	47	61	70	3	50	11	35	0

Solyc09g076040.2.1	5	46	2	35	1	114	44	130	4	112	8	73	0
Solyc09g076050.2.1	9	716	72	587	38	609	384	1216	82	750	89	412	17
Solyc09g082050.2.1	9	16	6	24	3	49	54	38	9	55	13	24	0
Solyc09g082060.2.1	12	604	56	308	142	527	368	561	364	546	52	378	253
Solyc09g082080.1.1	0	0	0	0	0	0	0	0	0	0	0	0	0
Solyc09g082100.2.1	0	0	0	0	0	0	0	0	0	0	0	0	0
Solyc09g082110.2.1	7	11	1	8	2	33	25	55	3	16	77	15	0
Solyc09g082120.2.1	7	189	15	87	9	469	151	489	12	312	63	144	3
Solyc09g082130.2.1	4	13	1	2	2	36	12	41	2	15	3	11	5
Solyc09g082140.2.1	7	35	4	20	5	32	25	102	7	60	12	38	4
Solyc09g082150.1.1	0	0	0	0	0	0	0	0	0	0	0	0	0
Solyc09g082160.2.1	2	33	0	8	0	19	4	34	0	28	1	27	0
Solyc09g082170.2.1	3	0	0	0	0	18	15	6	5	6	6	13	0
Solyc09g082180.2.1	0	0	0	0	0	0	0	0	0	0	0	0	0
Solyc09g082190.1.1	0	0	0	0	0	0	0	0	0	0	0	0	0
Solyc09g082200.2.1	3	23	1	19	0	22	17	31	0	9	1	16	0
Solyc09g082210.2.1	8	93	2	52	8	132	126	178	17	375	42	357	6
Solyc09g082240.2.1	0	0	0	0	0	0	0	0	0	0	0	0	0
Solyc09g082250.2.1	0	0	0	0	0	0	0	0	0	0	0	0	0
Solyc09g082270.2.1	1	3	1	7	0	4	18	9	3	0	1	0	0
Solyc09g082290.2.1	0	0	0	0	0	0	0	0	0	0	0	0	0
Solyc09g082300.2.1	1	0	0	0	0	20	19	5	25	0	3	0	0
Solyc09g082320.2.1	5	94	12	30	0	252	54	224	21	147	24	89	1
Solyc09g082340.2.1	18	2	1	2	0	113	972	1079	0	29	1651	49	26
Solyc09g082370.2.1	0	0	0	0	0	0	0	0	0	0	0	0	0
Solyc09g082380.2.1	0	0	0	0	0	0	0	0	0	0	0	0	0
Solyc09g082390.1.1	1	3	1	0	0	2	9	3	0	0	4	2	0
Solyc09g082400.1.1	0	0	0	0	0	0	0	0	0	0	0	0	0
Solyc09g082450.1.1	4	2	0	3	0	8	12	17	0	15	2	2	0
Solyc09g082460.2.1	0	0	0	0	0	0	0	0	0	0	0	0	0
Solyc09g082470.2.1	6	96	6	48	2	378	78	330	32	28	9	33	1
Solyc09g082490.2.1	0	0	0	0	0	0	0	0	0	0	0	0	0
Solyc09g082500.2.1	2	25	4	6	2	24	2	27	3	25	7	7	0
Solyc09g082510.2.1	43	361	42	212	53	360	423	1052	395	460	72	342	14
Solyc09g082520.2.1	10	191	93	106	24	356	750	498	106	865	351	646	40
Solyc09g082530.1.1	0	0	0	0	0	0	0	0	0	0	0	0	0
Solyc09g082540.2.1	1	5	0	3	0	8	1	3	1	11	1	4	0
Solyc09g082550.2.1	0	0	0	0	0	0	0	0	0	0	0	0	0
Solyc09g082560.2.1	1	19	0	4	0	1	4	1	2	0	0	0	0
Solyc09g082570.2.1	7	68	1	10	5	114	45	44	102	28	10	21	2
Solyc09g082580.2.1	8	164	12	49	23	32	78	60	1	113	8	37	8

Solyc09g082630.2.1	4	288	35	227	1	176	59	199	0	108	14	141	0
Solyc09g082650.2.1	5	1106	194	647	58	1955	1408	2361	185	6863	749	2836	71
Solyc09g082660.2.1	0	0	0	0	0	0	0	0	0	0	0	0	0
Solyc09g082670.2.1	0	0	0	0	0	0	0	0	0	0	0	0	0
Solyc09g082680.2.1	9	39	9	22	10	84	155	117	23	144	22	88	8
Solyc09g082690.2.1	6	79	4	59	9	22	13	25	7	150	248	303	3
Solyc09g082700.2.1	0	0	0	0	0	0	0	0	0	0	0	0	0
Solyc09g082710.2.1	4	76	0	14	3	72	26	78	10	207	25	157	1
Solyc09g082720.2.1	0	0	0	0	0	0	0	0	0	0	0	0	0
Solyc09g082730.2.1	10	354	44	335	4	492	267	851	25	398	71	387	3
Solyc09g082760.2.1	5	197	24	60	0	30	0	43	0	1	3	0	0
Solyc09g082770.2.1	4	28	5	11	6	34	53	77	15	57	17	42	0
Solyc09g082780.2.1	8	405	14	175	19	402	706	802	76	698	29	471	12
Solyc09g082790.2.1	0	0	0	0	0	0	0	0	0	0	0	0	0
Solyc09g082800.2.1	11	21	4	17	8	53	44	74	19	48	21	35	1
Solyc09g082810.2.1	8	2561	29	68	81	7982	3649	13127	2943	68	129	211	0
Solyc09g082830.2.1	10	29	11	42	5	21	146	41	8	44	15	42	1
Solyc09g082860.2.1	8	201	3	74	7	104	19	143	10	97	217	17	11
Solyc09g082870.1.1	4	74	6	54	0	14	12	9	0	1	0	0	0
Solyc09g082890.1.1	1	0	0	0	0	12	2	6	2	0	4	4	0
Solyc09g082900.2.1	0	0	0	0	0	0	0	0	0	0	0	0	0
Solyc09g082920.1.1	0	0	0	0	0	0	0	0	0	0	0	0	0
Solyc09g082940.2.1	1	4	0	1	0	0	3	2	0	0	5	9	0
Solyc09g082950.1.1	7	64	3	36	0	74	31	128	17	100	12	83	5
Solyc09g082970.2.1	11	87	7	49	7	152	102	278	35	413	71	271	25
Solyc09g082980.2.1	7	87	1	111	10	21	43	52	4	281	153	214	11
Solyc09g082990.2.1	10	1147	80	884	39	707	455	805	55	1017	292	833	24
Solyc09g083000.2.1	2	23	1	2	0	22	15	22	4	83	4	31	0
Solyc09g083010.2.1	0	0	0	0	0	0	0	0	0	0	0	0	0
Solyc09g083020.1.1	4	106	6	78	4	64	15	103	3	256	6	192	0
Solyc09g083030.1.1	5	55	5	26	0	39	25	57	0	131	17	104	0
Solyc09g083050.2.1	20	152	1	16	17	1218	557	229	926	3	9	0	1
Solyc09g083060.2.1	9	154	10	116	8	234	72	196	13	30	3	18	1
Solyc09g083070.2.1	2	9	4	4	0	9	17	23	3	11	8	7	0
Solyc09g083080.2.1	11	95	4	74	4	331	94	380	18	223	115	319	4
Solyc09g083090.2.1	0	0	0	0	0	0	0	0	0	0	0	0	0
Solyc09g083100.1.1	0	0	0	0	0	0	0	0	0	0	0	0	0
Solyc09g083110.1.1	1	0	0	0	0	3	2	12	0	6	1	1	1
Solyc09g083120.2.1	1	3	1	1	0	0	8	2	0	1	0	2	0
Solyc09g083130.2.1	18	116	30	92	0	33	99	87	0	94	18	20	0
Solyc09g083140.2.1	0	0	0	0	0	0	0	0	0	0	0	0	0

Solyc09g083150.2.1	8	157	21	110	0	2	47	25	0	23	5	47	0
Solyc09g083190.2.1	4	159	44	75	0	2	101	13	0	13	11	39	1
Solyc09g083200.2.1	0	0	0	0	0	0	0	0	0	0	0	0	0
Solyc09g083210.2.1	7	58	6	82	9	66	42	60	36	33	25	38	6
Solyc09g083220.2.1	7	37	4	53	0	77	82	123	5	66	21	59	1
Solyc09g083230.1.1	1	2	0	0	0	0	11	5	2	7	0	3	0
Solyc09g083240.2.1	2	9	0	2	2	13	12	9	1	12	1	3	0
Solyc09g083250.2.1	0	0	0	0	0	0	0	0	0	0	0	0	0
Solyc09g083260.2.1	0	0	0	0	0	0	0	0	0	0	0	0	0
Solyc09g083270.2.1	2	7	0	2	0	1	4	8	3	6	8	8	1
Solyc09g083280.2.1	2	33	10	15	8	32	28	12	29	1	1	3	1
Solyc09g083290.2.1	5	390	24	178	19	13	25	28	6	4	0	0	0
Solyc09g083320.1.1	1	6	0	0	0	1	6	3	1	2	2	5	0
Solyc09g083330.2.1	2	9	1	7	1	8	17	14	0	0	2	0	0
Solyc09g083340.1.1	1	3	0	0	0	1	7	7	0	1	0	5	0
Solyc09g083350.2.1	15	73	10	41	3	129	104	272	4	63	13	71	3
Solyc09g083360.2.1	0	0	0	0	0	0	0	0	0	0	0	0	0
Solyc09g083370.2.1	3	8	1	3	3	15	8	13	1	20	6	32	0
Solyc09g083380.2.1	4	50	2	19	1	59	15	23	0	68	35	53	2
Solyc09g083390.1.1	12	148	14	128	29	183	79	235	43	3	8	1	0
Solyc09g083400.2.1	5	61	4	39	4	9	32	37	0	17	9	21	5
Solyc09g083410.2.1	15	78	13	74	4	225	182	263	24	418	51	300	6
Solyc09g083420.2.1	8	20	6	35	3	51	34	95	5	57	20	39	0
Solyc09g083440.2.1	0	0	0	0	0	0	0	0	0	0	0	0	0
Solyc09g084440.2.1	2	2	1	15	0	3	15	12	0	14	2	74	0
Solyc09g084450.2.1	0	0	0	0	0	0	0	0	0	0	0	0	0
Solyc09g084460.2.1	0	0	0	0	0	0	0	0	0	0	0	0	0
Solyc09g084470.2.1	6	204	140	794	0	42	527	57	2	1	0	1	0
Solyc09g084480.2.1	0	0	0	0	0	0	0	0	0	0	0	0	0
Solyc09g084490.2.1	4	19	3	14	1	6	77	43	0	1	6	0	0
Solyc09g089490.2.1	0	0	0	0	0	0	0	0	0	0	0	0	0
Solyc09g089500.2.1	0	0	0	0	0	0	0	0	0	0	0	0	0
Solyc09g089510.2.1	1	0	1	0	0	5	7	7	0	2	0	0	0
Solyc09g089520.2.1	0	0	0	0	0	0	0	0	0	0	0	0	0
Solyc09g089540.2.1	0	0	0	0	0	0	0	0	0	0	0	0	0
Solyc09g089550.2.1	0	0	0	0	0	0	0	0	0	0	0	0	0
Solyc09g089560.2.1	19	48	7	45	6	103	173	98	16	95	40	107	5
Solyc09g089580.2.1	19	40	0	38	1	8337	64	6226	109	945	2250	224	0
Solyc09g089610.2.1	2	0	0	0	2	15	4	7	0	9	6	0	0
Solyc09g089620.1.1	0	0	0	0	0	0	0	0	0	0	0	0	0
Solyc09g089630.2.1	0	0	0	0	0	0	0	0	0	0	0	0	0

Solyc09g089640.2.1	2	6	0	1	0	10	3	13	0	1	3	0	0
Solyc09g089650.1.1	2	10	0	0	0	7	4	31	0	4	0	4	0
Solyc09g089660.2.1	3	2	7	4	0	7	54	15	7	20	19	11	0
Solyc09g089670.2.1	5	31	2	2	2	49	48	72	5	35	30	22	0
Solyc09g089680.2.1	0	0	0	0	0	0	0	0	0	0	0	0	0
Solyc09g089690.2.1	8	206	18	107	0	53	51	80	2	185	3	158	0
Solyc09g089700.2.1	0	0	0	0	0	0	0	0	0	0	0	0	0
Solyc09g089720.1.1	0	0	0	0	0	0	0	0	0	0	0	0	0
Solyc09g089730.2.1	3	91	14	145	0	255	5	190	0	0	3	10	0
Solyc09g089740.2.1	0	0	0	0	0	0	0	0	0	0	0	0	0
Solyc09g089750.1.1	0	0	0	0	0	0	0	0	0	0	0	0	0
Solyc09g089780.2.1	0	0	0	0	0	0	0	0	0	0	0	0	0
Solyc09g089790.2.1	0	0	0	0	0	0	0	0	0	0	0	0	0
Solyc09g089850.2.1	2	7	0	1	0	3	6	9	1	2	0	0	0
Solyc09g089860.2.1	0	0	0	0	0	0	0	0	0	0	0	0	0
Solyc09g089870.2.1	2	3	0	2	0	6	10	12	4	16	8	33	0
Solyc09g089880.2.1	6	27	3	31	0	63	91	137	18	112	18	50	0
Solyc09g089890.1.1	0	0	0	0	0	0	0	0	0	0	0	0	0
Solyc09g089910.1.1	0	0	0	0	0	0	0	0	0	0	0	0	0
Solyc09g089930.1.1	2	16	0	35	0	285	61	62	0	6	2	2	0
Solyc09g089940.1.1	13	51	5	32	0	354	71	174	46	169	38	137	5
Solyc09g089950.1.1	0	0	0	0	0	0	0	0	0	0	0	0	0
Solyc09g089980.1.1	0	0	0	0	0	0	0	0	0	0	0	0	0
Solyc09g089990.2.1	0	0	0	0	0	0	0	0	0	0	0	0	0
Solyc09g090020.2.1	8	78	2	8	0	45	29	52	5	24	0	14	0
Solyc09g090030.1.1	0	0	0	0	0	0	0	0	0	0	0	0	0
Solyc09g090040.2.1	8	150	16	55	28	150	147	400	75	255	60	229	8
Solyc09g090050.2.1	1	2	0	0	0	6	6	0	0	6	0	8	0
Solyc09g090060.2.1	1	6	3	3	0	5	21	34	1	8	2	12	0
Solyc09g090070.1.1	9	178	17	212	26	875	81	871	37	424	2	116	0
Solyc09g090080.1.1	1	1	0	2	0	8	6	33	2	0	0	0	0
Solyc09g090090.1.1	0	0	0	0	0	0	0	0	0	0	0	0	0
Solyc09g090100.2.1	13	137	17	107	4	217	147	489	9	242	27	243	0
Solyc09g090110.2.1	1	83	10	44	0	109	39	104	10	87	20	67	2
Solyc09g090120.2.1	3	6	0	9	0	8	3	19	0	2	5	1	0
Solyc09g090130.2.1	0	0	0	0	0	0	0	0	0	0	0	0	0
Solyc09g090140.2.1	2	230	11	140	11	298	45	338	10	340	63	357	2
Solyc09g090150.2.1	3	0	0	0	0	0	0	0	0	10	499	13	16
Solyc09g090160.2.1	11	192	12	115	20	417	169	413	48	355	56	298	15
Solyc09g090180.1.1	2	27	4	27	0	49	8	44	1	6	1	4	0
Solyc09g090190.2.1	8	184	7	77	15	298	111	224	105	107	38	77	1

Solyc09g090200.2.1	5	70	0	36	2	169	26	121	6	100	7	59	0
Solyc09g090220.2.1	0	0	0	0	0	0	0	0	0	0	0	0	0
Solyc09g090230.1.1	0	0	0	0	0	0	0	0	0	0	0	0	0
Solyc09g090240.1.1	0	0	0	0	0	0	0	0	0	0	0	0	0
Solyc09g090250.2.1	5	177	14	93	5	994	84	364	26	307	49	202	4
Solyc09g090260.1.1	0	0	0	0	0	0	0	0	0	0	0	0	0
Solyc09g090270.2.1	14	109	2	65	7	124	64	301	10	156	45	122	8
Solyc09g090290.2.1	0	0	0	0	0	0	0	0	0	0	0	0	0
Solyc09g090300.2.1	0	0	0	0	0	0	0	0	0	0	0	0	0
Solyc09g090320.1.1	0	0	0	0	0	0	0	0	0	0	0	0	0
Solyc09g090330.2.1	5	438	32	245	9	175	196	181	7	104	70	121	1
Solyc09g090340.1.1	3	9	1	4	0	14	6	11	6	17	8	21	0
Solyc09g090350.2.1	2	12	1	7	4	86	10	30	1	1	0	3	0
Solyc09g090360.2.1	1	4	1	5	0	2	1	0	0	4	4	3	0
Solyc09g090370.2.1	4	17	1	16	2	27	14	25	4	25	5	6	1
Solyc09g090380.1.1	1	8	0	3	0	28	8	26	3	11	6	7	0
Solyc09g090390.1.1	10	6	1	10	41	1	14	13	25	183	293	5	43
Solyc09g090400.2.1	0	0	0	0	0	0	0	0	0	0	0	0	0
Solyc09g090410.2.1	3	57	2	23	0	13	15	14	0	16	7	13	0
Solyc09g090420.2.1	16	59	5	39	6	188	83	155	18	79	36	65	2
Solyc09g090430.2.1	8	325	11	157	2	1012	616	730	25	179	76	89	3
Solyc09g090440.1.1	1	2	0	2	2	0	4	2	2	2	1	3	0
Solyc09g090460.2.1	6	19	4	6	0	27	45	21	0	44	5	32	3
Solyc09g090470.2.1	0	0	0	0	0	0	0	0	0	0	0	0	0
Solyc09g090480.2.1	0	0	0	0	0	0	0	0	0	0	0	0	0
Solyc09g090500.2.1	0	0	0	0	0	0	0	0	0	0	0	0	0
Solyc09g090510.2.1	1	5	1	3	0	1	2	2	0	19	4	7	0
Solyc09g090520.2.1	7	406	32	292	6	892	153	692	61	658	106	410	1
Solyc09g090530.2.1	0	0	0	0	0	0	0	0	0	0	0	0	0
Solyc09g090540.2.1	0	0	0	0	0	0	0	0	0	0	0	0	0
Solyc09g090550.2.1	0	0	0	0	0	0	0	0	0	0	0	0	0
Solyc09g090560.1.1	0	0	0	0	0	0	0	0	0	0	0	0	0
Solyc09g090570.2.1	3	207	8	68	0	79	2	12	1	15	23	42	1
Solyc09g090580.2.1	2	46	1	22	0	29	6	57	2	125	8	115	6
Solyc09g090600.2.1	0	0	0	0	0	0	0	0	0	0	0	0	0
Solyc09g090610.2.1	1	5	1	10	0	12	2	6	2	11	4	13	0
Solyc09g090620.1.1	0	0	0	0	0	0	0	0	0	0	0	0	0
Solyc09g090630.1.1	0	0	0	0	0	0	0	0	0	0	0	0	0
Solyc09g090640.2.1	16	164	42	151	22	97	128	67	13	123	249	95	15
Solyc09g090650.2.1	0	0	0	0	0	0	0	0	0	0	0	0	0
Solyc09g090660.2.1	16	111	10	74	6	168	111	301	24	213	58	244	2

Solyc09g090670.2.1	0	0	0	0	0	0	0	0	0	0	0	0	0
Solyc09g090680.2.1	0	0	0	0	0	0	0	0	0	0	0	0	0
Solyc09g090690.2.1	0	0	0	0	0	0	0	0	0	0	0	0	0
Solyc09g090700.1.1	13	178	33	91	8	293	199	414	25	385	94	275	6
Solyc09g090710.2.1	3	13	0	1	0	129	20	68	2	17	6	7	1
Solyc09g090720.2.1	6	53	6	42	0	213	18	169	8	97	12	73	0
Solyc09g090730.1.1	9	232	27	251	2	208	47	198	21	10	5	4	0
Solyc09g090740.2.1	5	4	0	12	0	55	18	41	2	14	7	20	0
Solyc09g090750.2.1	0	0	0	0	0	0	0	0	0	0	0	0	0
Solyc09g090760.2.1	0	0	0	0	0	0	0	0	0	0	0	0	0
Solyc09g090770.1.1	0	0	0	0	0	0	0	0	0	0	0	0	0
Solyc09g090780.1.1	1	0	1	0	0	2	5	0	0	11	1	18	0
Solyc09g090800.1.1	0	0	0	0	0	0	0	0	0	0	0	0	0
Solyc09g090810.1.1	2	15	1	4	1	19	12	14	0	16	3	5	0
Solyc09g090820.2.1	3	19	2	5	2	25	14	27	3	45	11	70	2
Solyc09g090830.2.1	0	0	0	0	0	0	0	0	0	0	0	0	0
Solyc09g090840.2.1	7	61	11	24	2	74	124	72	3	39	21	47	0
Solyc09g090860.2.1	0	0	0	0	0	0	0	0	0	0	0	0	0
Solyc09g090870.2.1	2	6	0	4	0	9	14	11	1	7	0	6	0
Solyc09g090890.1.1	0	0	0	0	0	0	0	0	0	0	0	0	0
Solyc09g090900.2.1	0	0	0	0	0	0	0	0	0	0	0	0	0
Solyc09g090910.1.1	1	3	1	7	0	6	5	12	0	14	6	2	0
Solyc09g090930.2.1	4	50	4	23	1	95	19	46	1	29	15	40	0
Solyc09g090940.2.1	8	156	51	71	23	190	295	286	57	155	90	123	1
Solyc09g090960.2.1	4	177	20	94	3	52	51	105	8	54	22	133	1
Solyc09g090970.2.1	3	4	0	1	0	5	10	10	8	6	19	1	0
Solyc09g090980.2.1	3	203	8	54	19	367	189	581	449	1073	146	430	64
Solyc09g090990.2.1	2	2	0	6	0	14	4	19	0	0	0	1	0
Solyc09g091000.2.1	1	3	0	0	0	8	3	11	7	3	0	0	0
Solyc09g091010.2.1	10	80	5	36	2	100	107	159	2	146	19	143	2
Solyc09g091020.2.1	18	106	8	78	11	245	227	345	29	167	48	123	2
Solyc09g091030.2.1	21	842	190	503	48	10092	4202	8725	150	263	144	216	5
Solyc09g091050.2.1	9	18	5	26	1	26	59	52	2	99	18	57	3
Solyc09g091060.2.1	0	0	0	0	0	0	0	0	0	0	0	0	0
Solyc09g091070.1.1	9	77	19	67	4	156	147	130	18	102	58	78	0
Solyc09g091100.2.1	4	70	5	73	0	25	35	42	1	35	4	56	0
Solyc09g091110.2.1	0	0	0	0	0	0	0	0	0	0	0	0	0
Solyc09g091120.2.1	11	30	4	13	2	65	57	73	11	42	9	52	0
Solyc09g091150.2.1	0	0	0	0	0	0	0	0	0	0	0	0	0
Solyc09g091160.1.1	0	0	0	0	0	0	0	0	0	0	0	0	0
Solyc09g091170.2.1	0	0	0	0	0	0	0	0	0	0	0	0	0

Solyc09g091180.2.1	4	29	1	12	3	88	31	95	12	143	94	168	2
Solyc09g091190.2.1	3	31	0	10	0	44	16	33	4	58	10	52	0
Solyc09g091230.2.1	2	19	1	9	0	24	11	27	5	14	1	24	0
Solyc09g091240.2.1	4	20	0	15	0	13	13	25	0	16	3	17	0
Solyc09g091250.2.1	6	227	18	172	14	247	74	261	16	179	89	131	4
Solyc09g091260.2.1	3	16	5	9	0	3	13	5	0	18	1	11	0
Solyc09g091270.1.1	1	0	0	0	0	0	0	3	0	4	0	0	0
Solyc09g091280.2.1	13	38	7	31	1	77	65	119	8	73	40	71	0
Solyc09g091290.1.1	0	0	0	0	0	0	0	0	0	0	0	0	0
Solyc09g091370.2.1	0	0	0	0	0	0	0	0	0	0	0	0	0
Solyc09g091380.1.1	0	0	0	0	0	0	0	0	0	0	0	0	0
Solyc09g091390.2.1	0	0	0	0	0	0	0	0	0	0	0	0	0
Solyc09g091400.2.1	0	0	0	0	0	0	0	0	0	0	0	0	0
Solyc09g091420.2.1	1	3	1	7	0	0	3	4	0	4	2	1	0
Solyc09g091430.2.1	4	74	7	19	7	1	7	19	2	8	326	1	0
Solyc09g091440.2.1	12	121	5	56	7	238	113	285	19	212	54	244	4
Solyc09g091450.2.1	1	7	0	1	4	6	6	9	4	13	4	6	0
Solyc09g091460.2.1	0	0	0	0	0	0	0	0	0	0	0	0	0
Solyc09g091470.2.1	10	249	33	170	35	1375	3140	1210	338	485	993	324	20
Solyc09g091480.2.1	7	14	0	24	9	29	18	41	1	44	12	28	2
Solyc09g091500.2.1	0	0	0	0	0	0	0	0	0	0	0	0	0
Solyc09g091510.2.1	16	410	27	99	165	140	248	35	117	147	76	59	1
Solyc09g091520.1.1	0	0	0	0	0	0	0	0	0	0	0	0	0
Solyc09g091530.1.1	0	0	0	0	0	0	0	0	0	0	0	0	0
Solyc09g091550.2.1	0	0	0	0	0	0	0	0	0	0	0	0	0
Solyc09g091560.2.1	13	55	2	35	5	175	74	119	20	77	7	51	0
Solyc09g091570.2.1	11	23	8	22	6	123	117	238	29	185	25	83	0
Solyc09g091580.2.1	10	385	20	255	9	179	35	251	0	125	30	172	1
Solyc09g091590.2.1	4	72	7	22	0	29	16	38	5	78	18	49	0
Solyc09g091600.2.1	4	45	8	28	1	81	19	43	0	12	24	1	0
Solyc09g091610.2.1	0	0	0	0	0	0	0	0	0	0	0	0	0
Solyc09g091650.2.1	0	0	0	0	0	0	0	0	0	0	0	0	0
Solyc09g091660.2.1	19	449	57	440	20	1005	250	706	318	33	18	38	0
Solyc09g091670.2.1	1	1	1	0	0	4	2	2	2	0	0	0	0
Solyc09g091690.1.1	1	6	0	12	0	1	0	4	0	17	0	12	0
Solyc09g091700.2.1	0	0	0	0	0	0	0	0	0	0	0	0	0
Solyc09g091710.1.1	5	27	4	16	1	58	16	55	5	44	8	23	6
Solyc09g091740.2.1	2	43	4	18	0	47	14	70	0	109	7	37	0
Solyc09g091750.2.1	10	102	7	104	8	148	65	339	19	176	36	149	1
Solyc09g091760.1.1	0	0	0	0	0	0	0	0	0	0	0	0	0
Solyc09g091780.2.1	0	0	0	0	0	0	0	0	0	0	0	0	0

Solyc09g091790.2.1	3	27	1	37	5	32	23	63	3	53	6	38	0
Solyc09g091800.2.1	1	0	0	0	0	0	3	2	0	52	2	30	0
Solyc09g091820.2.1	14	523	60	214	20	1207	580	1322	125	1198	348	1024	24
Solyc09g091830.2.1	7	65	3	20	0	38	28	54	4	14	6	18	0
Solyc09g091840.2.1	12	111	28	86	9	544	211	553	65	233	144	163	3
Solyc09g091850.2.1	13	33	4	28	1	67	71	126	10	73	7	73	0
Solyc09g091860.2.1	3	12	2	8	1	1	9	7	1	14	1	3	3
Solyc09g091880.2.1	0	0	0	0	0	0	0	0	0	0	0	0	0
Solyc09g091900.2.1	1	7	3	4	4	21	38	21	6	5	5	3	2
Solyc09g091910.1.1	0	0	0	0	0	0	0	0	0	0	0	0	0
Solyc09g091920.2.1	0	0	0	0	0	0	0	0	0	0	0	0	0
Solyc09g091930.2.1	6	14	1	24	1	21	27	30	3	35	8	15	1
Solyc09g091940.2.1	1	46	4	24	1	66	10	64	1	162	4	108	1
Solyc09g091960.2.1	2	27	1	10	0	2	0	2	0	0	3	6	1
Solyc09g091970.2.1	6	85	2	28	0	13	35	28	2	6	6	20	4
Solyc09g091980.2.1	2	1	2	2	0	2	6	11	3	59	10	39	1
Solyc09g091990.2.1	3	21	4	29	0	50	12	57	11	26	6	37	4
Solyc09g092000.2.1	0	0	0	0	0	0	0	0	0	0	0	0	0
Solyc09g092010.1.1	0	0	0	0	0	0	0	0	0	0	0	0	0
Solyc09g092020.2.1	0	0	0	0	0	0	0	0	0	0	0	0	0
Solyc09g092030.2.1	1	16	1	0	0	14	24	19	4	18	5	2	0
Solyc09g092040.2.1	1	38	0	18	0	52	5	44	0	138	2	39	0
Solyc09g092050.2.1	0	0	0	0	0	0	0	0	0	0	0	0	0
Solyc09g092070.2.1	5	13	2	8	0	35	40	42	4	52	32	43	2
Solyc09g092080.2.1	2	21	1	21	1	45	32	83	9	47	1	31	0
Solyc09g092090.2.1	0	0	0	0	0	0	0	0	0	0	0	0	0
Solyc09g092100.2.1	6	10	6	5	0	54	24	53	3	28	4	31	0
Solyc09g092110.2.1	6	172	42	306	4	8	880	25	3	35	12	12	1
Solyc09g092120.2.1	7	97	4	40	0	251	39	263	9	125	6	115	1
Solyc09g092130.2.1	11	34	3	25	1	45	123	73	2	37	8	135	0
Solyc09g092140.2.1	12	36	4	17	4	139	89	230	12	135	13	160	3
Solyc09g092150.1.1	0	0	0	0	0	0	0	0	0	0	0	0	0
Solyc09g092160.2.1	16	25	8	47	8	113	277	120	36	97	26	78	1
Solyc09g092180.2.1	1	0	0	1	0	4	1	5	0	5	1	0	0
Solyc09g092210.2.1	6	20	3	11	4	25	16	36	4	23	12	23	0
Solyc09g092220.2.1	0	0	0	0	0	0	0	0	0	0	0	0	0
Solyc09g092230.2.1	0	0	0	0	0	0	0	0	0	0	0	0	0
Solyc09g092240.2.1	8	4	1	2	1	24	33	59	13	32	14	29	2
Solyc09g092250.2.1	18	92	16	55	15	167	104	183	19	171	48	109	2
Solyc09g092260.2.1	9	36	16	22	11	166	702	316	33	531	26	288	18
Solyc09g092270.2.1	7	171	1	4	0	274	48	14	50	24	2	1	0

Solyc09g092290.1.1	0	0	0	0	0	0	0	0	0	0	0	0	0
Solyc09g092310.1.1	0	0	0	0	0	0	0	0	0	0	0	0	0
Solyc09g092320.2.1	4	15	0	4	1	19	6	17	5	53	11	63	0
Solyc09g092330.1.1	13	475	58	206	97	45	292	335	155	447	287	310	4
Solyc09g092340.2.1	3	18	2	5	1	6	20	12	3	64	15	30	0
Solyc09g092350.1.1	0	0	0	0	0	0	0	0	0	0	0	0	0
Solyc09g092360.2.1	4	20	5	22	4	60	77	41	12	54	18	39	0
Solyc09g092370.1.1	0	0	0	0	0	0	0	0	0	0	0	0	0
Solyc09g092380.2.1	6	781	92	633	125	777	645	1484	140	2846	1767	1982	18
Solyc09g092390.2.1	6	691	47	253	39	855	371	1073	285	857	231	617	14
Solyc09g092400.1.1	0	0	0	0	0	0	0	0	0	0	0	0	0
Solyc09g092420.1.1	0	0	0	0	0	0	0	0	0	0	0	0	0
Solyc09g092430.2.1	8	158	14	131	3	815	1734	1304	83	414	51	140	6
Solyc09g092440.1.1	0	0	0	0	0	0	0	0	0	0	0	0	0
Solyc09g092450.2.1	12	32	3	16	0	91	58	145	4	71	9	22	8
Solyc09g092460.2.1	1	2	0	3	0	7	3	4	0	2	2	0	0
Solyc09g092480.1.1	1	3	0	13	0	4	4	3	0	0	0	0	0
Solyc09g092490.2.1	4	88	3	13	0	93	7	36	0	1	11	0	0
Solyc09g092500.1.1	13	446	8	153	0	585	89	405	1	54	116	61	0
Solyc09g092510.2.1	0	0	0	0	0	0	0	0	0	0	0	0	0
Solyc09g092520.2.1	4	222	10	50	0	17	55	13	0	38	17	1	0
Solyc09g092530.2.1	5	30	7	21	0	54	49	118	5	71	21	76	1
Solyc09g092540.1.1	0	0	0	0	0	0	0	0	0	0	0	0	0
Solyc09g092550.2.1	24	191	20	91	24	106	60	151	10	299	84	206	9
Solyc09g092560.2.1	0	0	0	0	0	0	0	0	0	0	0	0	0
Solyc09g092580.2.1	0	0	0	0	0	0	0	0	0	0	0	0	0
Solyc09g092600.2.1	1	0	1	0	0	19	184	19	3	1	3	0	0
Solyc09g092620.2.1	0	0	0	0	0	0	0	0	0	0	0	0	0
Solyc09g092640.2.1	3	22	3	4	0	126	9	84	0	0	0	0	0
Solyc09g092670.2.1	5	73	0	27	0	9	5	30	0	102	11	76	0
Solyc09g092680.2.1	5	16	3	14	3	50	48	86	14	60	8	20	0
Solyc09g092690.2.1	0	0	0	0	0	0	0	0	0	0	0	0	0
Solyc09g092700.2.1	0	0	0	0	0	0	0	0	0	0	0	0	0
Solyc09g092710.2.1	4	14	30	1	79	72	2253	21	688	2	2	2	0
Solyc09g092720.2.1	0	0	0	0	0	0	0	0	0	0	0	0	0
Solyc09g092740.2.1	1	12	0	0	0	6	0	4	0	0	0	0	0
Solyc09g092750.2.1	0	0	0	0	0	0	0	0	0	0	0	0	0
Solyc09g092760.2.1	0	0	0	0	0	0	0	0	0	0	0	0	0
Solyc09g097760.2.1	19	44	5	13	17	602	1803	1437	1717	17	926	6	7
Solyc09g097770.2.1	2	3147	151	918	104	833	449	417	48	5	0	4	1
Solyc09g097780.2.1	9	939	116	23	323	514	1629	175	1259	235	31	1	2

Solyc09g097800.2.1	0	0	0	0	0	0	0	0	0	0	0	0	0
Solyc09g097810.2.1	0	0	0	0	0	0	0	0	0	0	0	0	0
Solyc09g097820.2.1	7	34	1	28	0	220	70	137	0	34	2	31	1
Solyc09g097830.2.1	12	78	17	65	1	296	256	393	47	238	47	152	9
Solyc09g097840.1.1	1	5	0	9	0	28	18	27	1	7	4	27	0
Solyc09g097850.1.1	8	58	3	28	8	344	143	155	20	208	110	177	29
Solyc09g097860.2.1	0	0	0	0	0	0	0	0	0	0	0	0	0
Solyc09g097870.2.1	3	42	3	40	0	11	8	21	4	49	12	41	0
Solyc09g097880.2.1	14	58	8	43	3	85	129	172	23	159	36	100	1
Solyc09g097890.2.1	2	42	15	11	0	11	0	4	0	0	0	0	0
Solyc09g097900.2.1	0	0	0	0	0	0	0	0	0	0	0	0	0
Solyc09g097910.2.1	2	24	8	17	0	3	9	11	1	7	3	9	0
Solyc09g097920.1.1	6	9	0	15	0	21	22	19	3	11	0	14	0
Solyc09g097930.2.1	0	0	0	0	0	0	0	0	0	0	0	0	0
Solyc09g097960.2.1	0	0	0	0	0	0	0	0	0	0	0	0	0
Solyc09g097970.2.1	3	8	8	1	0	2	19	5	9	43	4	14	5
Solyc09g097980.2.1	0	0	0	0	0	0	0	0	0	0	0	0	0
Solyc09g098030.2.1	0	0	0	0	0	0	0	0	0	0	0	0	0
Solyc09g098040.2.1	37	632	117	628	36	1628	2044	1871	95	1093	187	905	13
Solyc09g098050.2.1	0	0	0	0	0	0	0	0	0	0	0	0	0
Solyc09g098070.2.1	4	38	8	10	0	84	73	76	12	68	10	75	0
Solyc09g098080.2.1	0	0	0	0	0	0	0	0	0	0	0	0	0
Solyc09g098090.2.1	0	0	0	0	0	0	0	0	0	0	0	0	0
Solyc09g098100.2.1	8	12	1	27	1	42	41	57	1	23	1	16	0
Solyc09g098110.2.1	0	0	0	0	0	0	0	0	0	0	0	0	0
Solyc09g098120.2.1	0	0	0	0	0	0	0	0	0	0	0	0	0
Solyc09g098130.1.1	1	0	0	1	0	8	3	4	0	1	1	0	0
Solyc09g098140.2.1	2	24	2	6	0	66	16	58	3	34	0	35	2
Solyc09g098150.2.1	3	56	6	34	12	115	62	178	24	197	28	166	3
Solyc09g098160.2.1	6	6	4	6	0	91	12	47	4	1	18	1	0
Solyc09g098170.2.1	4	83	4	53	1	79	14	38	1	151	27	208	6
Solyc09g098180.2.1	0	0	0	0	0	0	0	0	0	0	0	0	0
Solyc09g098190.2.1	0	0	0	0	0	0	0	0	0	0	0	0	0
Solyc09g098200.2.1	0	0	0	0	0	0	0	0	0	0	0	0	0
Solyc09g098220.2.1	0	0	0	0	0	0	0	0	0	0	0	0	0
Solyc09g098230.2.1	9	42	13	42	5	120	86	124	9	112	21	61	1
Solyc09g098240.2.1	14	82	18	71	13	147	95	247	32	343	80	332	4
Solyc09g098250.2.1	4	13	0	6	0	5	15	16	3	10	9	17	0
Solyc09g098260.1.1	2	5	2	7	0	10	10	8	4	6	3	5	2
Solyc09g098270.2.1	1	0	0	0	0	0	5	0	25	0	0	0	0
Solyc09g098280.2.1	5	63	1	30	3	169	43	176	19	59	7	75	2

Solyc09g098290.2.1	7	50	7	46	0	8	15	5	0	355	23	427	4
Solyc09g098300.2.1	3	309	5	66	3	386	147	430	75	368	68	256	3
Solyc09g098310.2.1	1	97	7	66	4	405	78	278	51	71	17	70	1
Solyc09g098330.2.1	7	59	12	17	4	198	449	128	28	97	45	110	3
Solyc09g098340.2.1	1	4	0	0	0	7	2	5	0	7	2	4	0
Solyc09g098350.2.1	0	0	0	0	0	0	0	0	0	0	0	0	0
Solyc09g098360.2.1	3	59	25	53	9	73	122	69	10	611	50	333	19
Solyc09g098370.2.1	3	13	3	20	0	42	14	22	0	22	6	23	0
Solyc09g098380.1.1	0	0	0	0	0	0	0	0	0	0	0	0	0
Solyc09g098390.2.1	6	21	0	0	0	12	32	40	5	21	2	17	0
Solyc09g098440.2.1	0	0	0	0	0	0	0	0	0	0	0	0	0
Solyc09g098450.2.1	10	29	3	13	1	22	44	21	6	90	14	64	5
Solyc09g098490.2.1	0	0	0	0	0	0	0	0	0	0	0	0	0
Solyc09g098500.2.1	0	0	0	0	0	0	0	0	0	0	0	0	0
Solyc09g098510.2.1	2	110	0	56	0	21	3	48	1	32	10	33	0
Solyc09g098520.2.1	7	34	6	26	2	51	28	52	2	89	6	79	1
Solyc09g098530.2.1	11	94	15	76	6	236	130	263	11	48	12	35	0
Solyc09g098540.2.1	9	1159	28	931	154	187	214	275	42	5757	655	8729	68
Solyc09g098550.2.1	11	70	3	52	2	119	67	120	16	328	27	159	3
Solyc09g098560.2.1	0	0	0	0	0	0	0	0	0	0	0	0	0
Solyc09g098580.2.1	3	11	5	12	0	24	43	22	1	19	6	6	0
Solyc09g098590.2.1	9	59	1	30	7	1019	61	407	10	116	13	104	0
Solyc09g098610.1.1	0	0	0	0	0	0	0	0	0	0	0	0	0
Solyc09g098770.1.1	0	0	0	0	0	0	0	0	0	0	0	0	0
Solyc10g005000.2.1	0	0	0	0	0	0	0	0	0	0	0	0	0
Solyc10g005010.2.1	0	0	0	0	0	0	0	0	0	0	0	0	0
Solyc10g005020.2.1	0	0	0	0	0	0	0	0	0	0	0	0	0
Solyc10g005030.2.1	10	141	16	103	17	73	4	26	1	27	27	46	0
Solyc10g005040.2.1	7	24	3	20	0	1078	65	505	1	8	5	21	0
Solyc10g005050.2.1	2	206	25	99	0	5	37	15	1	47	13	23	0
Solyc10g005060.2.1	6	15	1	15	0	31	21	42	1	1	23	7	0
Solyc10g005080.2.1	15	417	38	234	40	94	14	54	10	31	80	35	0
Solyc10g005090.2.1	2	5	0	4	0	2	6	2	0	2	2	6	0
Solyc10g005100.2.1	7	956	18	696	17	845	857	1128	99	3302	244	1644	2
Solyc10g005110.2.1	5	218	15	136	1	188	146	336	5	77	28	64	1
Solyc10g005120.1.1	0	0	0	0	0	0	0	0	0	0	0	0	0
Solyc10g005130.2.1	7	23	6	19	4	71	30	50	5	42	10	34	0
Solyc10g005170.1.1	0	0	0	0	0	0	0	0	0	0	0	0	0
Solyc10g005180.2.1	6	69	22	49	4	60	140	51	5	112	44	114	4
Solyc10g005200.2.1	10	77	3	40	7	42	6	28	3	205	45	157	0
Solyc10g005210.2.1	3	10	0	9	0	2	13	2	0	19	3	29	0

Solyc10g005220.2.1	0	0	0	0	0	0	0	0	0	0	0	0	0
Solyc10g005230.2.1	1	9	0	2	0	16	5	14	3	73	12	45	1
Solyc10g005250.2.1	0	0	0	0	0	0	0	0	0	0	0	0	0
Solyc10g005260.2.1	7	90	11	66	10	50	48	104	3	242	58	223	15
Solyc10g005280.1.1	2	4	1	13	0	7	5	16	2	27	4	34	0
Solyc10g005290.2.1	2	5	0	1	0	11	6	4	0	6	2	7	0
Solyc10g005300.2.1	0	0	0	0	0	0	0	0	0	0	0	0	0
Solyc10g005310.2.1	5	10	5	1	4	15	30	15	2	18	172	39	4
Solyc10g005320.2.1	0	0	0	0	0	0	0	0	0	0	0	0	0
Solyc10g005330.2.1	14	79	5	0	8	70	101	23	128	29	5	3	6
Solyc10g005340.2.1	1	6	0	9	0	0	4	2	0	0	0	1	0
Solyc10g005360.2.1	0	0	0	0	0	0	0	0	0	0	0	0	0
Solyc10g005370.2.1	5	63	6	41	7	98	41	110	5	69	10	82	2
Solyc10g005380.2.1	0	0	0	0	0	0	0	0	0	0	0	0	0
Solyc10g005390.2.1	0	0	0	0	0	0	0	0	0	0	0	0	0
Solyc10g005400.2.1	0	0	0	0	0	0	0	0	0	0	0	0	0
Solyc10g005410.2.1	0	0	0	0	0	0	0	0	0	0	0	0	0
Solyc10g005440.1.1	0	0	0	0	0	0	0	0	0	0	0	0	0
Solyc10g005450.1.1	0	0	0	0	0	0	0	0	0	0	0	0	0
Solyc10g005460.2.1	0	0	0	0	0	0	0	0	0	0	0	0	0
Solyc10g005470.2.1	0	0	0	0	0	0	0	0	0	0	0	0	0
Solyc10g005480.2.1	5	64	32	34	4	26	16	6	2	10	2	22	0
Solyc10g005490.2.1	8	19	4	7	1	46	57	63	6	38	23	61	0
Solyc10g005500.2.1	2	6	0	4	0	6	13	14	0	1	0	1	0
Solyc10g005510.2.1	11	18	3	1	8	59	44	121	27	148	70	117	1
Solyc10g005520.2.1	5	4	2	0	0	12	21	27	1	19	7	21	1
Solyc10g005530.2.1	16	83	23	102	3	184	190	343	19	418	93	338	1
Solyc10g005540.2.1	1	3	3	0	0	0	0	3	0	1	1	2	0
Solyc10g005550.1.1	1	0	0	0	0	11	5	0	10	0	0	0	0
Solyc10g005560.2.1	3	196	20	149	7	512	342	520	68	202	96	179	8
Solyc10g005570.1.1	3	10	0	1	1	15	5	11	0	20	5	25	0
Solyc10g005580.2.1	2	5	3	8	2	4	6	17	1	23	3	31	3
Solyc10g005590.2.1	10	115	5	53	10	150	84	292	28	172	38	126	4
Solyc10g005600.2.1	6	8	1	10	0	21	17	33	3	15	3	41	0
Solyc10g005610.1.1	0	0	0	0	0	0	0	0	0	0	0	0	0
Solyc10g005620.2.1	5	45	10	18	15	8	18	15	7	28	13	19	0
Solyc10g005630.2.1	6	60	2	47	5	67	19	45	3	87	1	126	0
Solyc10g005640.2.1	2	20	6	8	0	5	11	12	0	29	10	15	0
Solyc10g005650.2.1	14	114	21	92	12	279	312	435	34	154	57	98	7
Solyc10g005660.2.1	3	11	0	7	5	21	21	29	2	22	7	25	0
Solyc10g005670.1.1	0	0	0	0	0	0	0	0	0	0	0	0	0

Solyc10g005690.2.1	0	0	0	0	0	0	0	0	0	0	0	0	0
Solyc10g005700.2.1	25	157	24	101	9	180	193	248	34	194	43	113	6
Solyc10g005720.1.1	3	35	9	28	0	38	22	66	3	123	6	121	0
Solyc10g005730.2.1	4	153	12	97	3	271	173	302	23	201	43	143	1
Solyc10g005740.1.1	0	0	0	0	0	0	0	0	0	0	0	0	0
Solyc10g005750.2.1	2	8	1	0	0	6	4	5	0	4	9	14	0
Solyc10g005770.2.1	15	53	9	47	8	74	187	130	5	409	65	335	11
Solyc10g005780.2.1	0	0	0	0	0	0	0	0	0	0	0	0	0
Solyc10g005790.2.1	8	15	5	19	1	0	23	12	2	28	26	34	4
Solyc10g005800.2.1	6	99	20	83	1	218	120	167	14	310	93	148	7
Solyc10g005810.2.1	2	0	0	0	0	0	22	4	3	8	0	7	0
Solyc10g005820.1.1	0	0	0	0	0	0	0	0	0	0	0	0	0
Solyc10g005830.2.1	3	35	0	20	2	64	22	82	4	41	6	32	0
Solyc10g005840.2.1	4	29	4	11	0	39	57	33	5	46	11	41	5
Solyc10g005850.2.1	7	40	4	18	0	121	37	117	15	72	24	85	1
Solyc10g005860.2.1	1	10	0	6	0	34	3	38	1	11	2	7	3
Solyc10g005870.2.1	3	7	1	8	0	2	19	7	0	14	0	12	0
Solyc10g005880.2.1	8	53	3	23	0	222	71	121	6	228	25	131	0
Solyc10g005890.2.1	9	117	16	109	2	283	155	355	30	258	22	215	2
Solyc10g005900.2.1	10	33	3	13	3	112	46	57	16	92	25	74	5
Solyc10g005910.2.1	1	2	0	0	0	0	0	3	1	2	2	4	3
Solyc10g005920.2.1	24	138	24	103	7	91	234	316	11	488	55	392	9
Solyc10g005930.1.1	5	28	0	19	0	22	25	48	5	58	2	40	0
Solyc10g005950.2.1	3	8	4	12	0	19	18	27	4	0	5	5	0
Solyc10g005960.1.1	10	765	41	218	23	48	58	43	5	382	20	232	3
Solyc10g005980.1.1	0	0	0	0	0	0	0	0	0	0	0	0	0
Solyc10g005990.2.1	7	34	4	23	1	140	31	90	11	67	17	68	8
Solyc10g006000.2.1	0	0	0	0	0	0	0	0	0	0	0	0	0
Solyc10g006010.2.1	4	47	1	24	0	30	23	74	5	53	15	45	0
Solyc10g006020.2.1	5	19	4	15	0	59	30	76	6	24	10	59	0
Solyc10g006030.2.1	2	155	8	57	3	17	144	63	3	57	21	51	2
Solyc10g006040.1.1	2	6	0	8	0	3	9	6	0	6	5	10	0
Solyc10g006050.2.1	6	31	3	12	1	78	39	59	4	126	14	86	0
Solyc10g006060.2.1	0	0	0	0	0	0	0	0	0	0	0	0	0
Solyc10g006070.2.1	5	129	8	97	6	392	50	298	18	282	87	152	8
Solyc10g006080.1.1	0	0	0	0	0	0	0	0	0	0	0	0	0
Solyc10g006090.2.1	24	153	18	169	15	261	234	317	33	518	84	418	10
Solyc10g006100.2.1	4	20	15	9	0	27	79	54	12	23	40	46	10
Solyc10g006120.1.1	0	0	0	0	0	0	0	0	0	0	0	0	0
Solyc10g006130.1.1	5	141	51	114	18	16	3	12	2	72	7	101	14
Solyc10g006140.2.1	0	0	0	0	0	0	0	0	0	0	0	0	0

Solyc10g006150.2.1	0	0	0	0	0	0	0	0	0	0	0	0	0
Solyc10g006170.2.1	1	5	0	2	0	0	3	3	0	3	1	5	0
Solyc10g006180.2.1	0	0	0	0	0	0	0	0	0	0	0	0	0
Solyc10g006190.2.1	1	9	1	5	0	5	27	9	1	14	0	6	0
Solyc10g006200.2.1	0	0	0	0	0	0	0	0	0	0	0	0	0
Solyc10g006210.1.1	0	0	0	0	0	0	0	0	0	0	0	0	0
Solyc10g006220.2.1	1	4	0	0	0	7	10	3	0	0	0	2	4
Solyc10g006230.2.1	7	4460	1492	2966	1308	85	451	51	42	681	621	682	408
Solyc10g006240.2.1	1	53	5	21	2	34	36	40	2	53	9	29	1
Solyc10g006250.2.1	3	6	2	5	0	16	21	43	5	27	7	20	1
Solyc10g006260.2.1	21	199	24	101	13	364	209	397	51	331	52	225	2
Solyc10g006270.2.1	1	5	2	7	0	19	10	21	0	9	0	19	0
Solyc10g006290.2.1	4	96	12	30	0	84	39	108	3	59	21	134	0
Solyc10g006300.2.1	8	79	5	74	7	92	102	219	11	43	16	12	0
Solyc10g006310.2.1	1	6	0	0	0	1	14	2	0	4	0	3	0
Solyc10g006320.2.1	3	21	0	3	0	55	17	23	2	35	3	38	1
Solyc10g006330.2.1	18	280	59	222	5	67	130	123	16	183	47	237	1
Solyc10g006340.2.1	0	0	0	0	0	0	0	0	0	0	0	0	0
Solyc10g006350.2.1	3	31	2	61	3	25	35	23	2	124	15	63	0
Solyc10g006360.1.1	0	0	0	0	0	0	0	0	0	0	0	0	0
Solyc10g006370.2.1	2	30	4	7	0	6	30	6	5	9	0	13	0
Solyc10g006390.2.1	4	6	0	11	0	12	11	27	0	24	2	20	0
Solyc10g006400.2.1	4	34	6	15	0	34	23	42	11	61	9	52	2
Solyc10g006410.2.1	5	14	1	5	0	28	42	35	5	27	7	27	0
Solyc10g006440.2.1	0	0	0	0	0	0	0	0	0	0	0	0	0
Solyc10g006450.2.1	0	0	0	0	0	0	0	0	0	0	0	0	0
Solyc10g006460.2.1	7	34	4	13	0	58	53	74	18	59	27	46	3
Solyc10g006470.2.1	1	157	5	32	6	141	79	166	10	137	19	80	1
Solyc10g006480.1.1	12	1124	78	885	64	4128	1343	5948	230	1224	283	724	13
Solyc10g006490.2.1	3	19	2	14	0	23	18	26	6	44	8	69	0
Solyc10g006500.2.1	1	9	0	6	0	13	5	17	0	0	0	0	0
Solyc10g006510.2.1	1	11	0	2	0	3	7	8	0	0	1	0	0
Solyc10g006520.2.1	1	3	0	1	0	0	6	1	1	6	2	9	0
Solyc10g006530.2.1	2	27	1	11	0	0	11	1	0	0	0	5	4
Solyc10g006540.2.1	10	61	13	41	4	78	41	60	5	43	7	42	0
Solyc10g006550.1.1	0	0	0	0	0	0	0	0	0	0	0	0	0
Solyc10g006560.2.1	3	30	11	11	8	39	26	53	26	26	10	33	16
Solyc10g006570.2.1	1	2	0	0	0	0	8	1	0	6	0	4	0
Solyc10g006580.2.1	11	473	24	187	15	820	339	1039	123	1280	316	1008	27
Solyc10g006600.2.1	1	2	1	0	1	2	11	2	0	6	1	13	0
Solyc10g006610.2.1	3	4	5	0	0	10	41	14	3	1	0	5	0

Solyc10g006640.2.1	0	0	0	0	0	0	0	0	0	0	0	0	0
Solyc10g006650.2.1	3	68	4	46	8	50	17	37	6	67	11	63	1
Solyc10g006660.2.1	1	28	6	22	0	0	0	0	0	11	0	22	1
Solyc10g006670.1.1	0	0	0	0	0	0	0	0	0	0	0	0	0
Solyc10g006700.1.1	1	100	5	94	5	9	1	6	0	24	2	30	0
Solyc10g006710.2.1	19	15	12	10	5	85	137	81	30	115	97	79	5
Solyc10g006720.2.1	0	0	0	0	0	0	0	0	0	0	0	0	0
Solyc10g006730.1.1	4	5	0	0	0	4	12	3	5	11	6	3	0
Solyc10g006740.2.1	0	0	0	0	0	0	0	0	0	0	0	0	0
Solyc10g006750.2.1	0	0	0	0	0	0	0	0	0	0	0	0	0
Solyc10g006760.2.1	5	44	3	3	0	86	9	75	3	60	17	37	1
Solyc10g006770.2.1	10	23	2	22	1	47	54	80	9	57	25	32	5
Solyc10g006780.2.1	10	24	4	25	0	46	34	48	7	17	12	12	0
Solyc10g006790.2.1	5	58	1	51	0	449	31	269	5	58	12	63	2
Solyc10g006800.2.1	12	106	24	120	0	672	156	1000	0	13	1	0	0
Solyc10g006810.2.1	0	0	0	0	0	0	0	0	0	0	0	0	0
Solyc10g006820.2.1	11	57	4	65	3	61	63	85	11	102	12	128	0
Solyc10g006830.2.1	0	0	0	0	0	0	0	0	0	0	0	0	0
Solyc10g006840.2.1	0	0	0	0	0	0	0	0	0	0	0	0	0
Solyc10g006850.2.1	0	0	0	0	0	0	0	0	0	0	0	0	0
Solyc10g006860.2.1	0	0	0	0	0	0	0	0	0	0	0	0	0
Solyc10g006870.1.1	1	4	0	9	6	0	2	1	0	0	2	0	0
Solyc10g006880.2.1	1	0	0	0	0	0	0	0	0	11	19	4	0
Solyc10g006890.2.1	10	66	14	59	17	53	62	95	13	94	31	93	6
Solyc10g006900.2.1	3	142	11	80	0	9	11	10	0	19	11	6	0
Solyc10g006920.2.1	0	0	0	0	0	0	0	0	0	0	0	0	0
Solyc10g006930.2.1	16	74	18	66	3	108	150	164	18	293	15	277	3
Solyc10g006950.1.1	0	0	0	0	0	0	0	0	0	0	0	0	0
Solyc10g006960.1.1	0	0	0	0	0	0	0	0	0	0	0	0	0
Solyc10g006970.2.1	5	1117	249	670	7	258	429	274	0	60	31	72	2
Solyc10g006980.2.1	3	58	3	70	7	36	56	99	11	130	30	151	3
Solyc10g006990.2.1	13	68	8	34	7	131	62	138	24	100	15	65	2
Solyc10g007000.2.1	7	8	1	19	7	103	53	61	13	39	13	28	0
Solyc10g007010.1.1	0	0	0	0	0	0	0	0	0	0	0	0	0
Solyc10g007020.1.1	0	0	0	0	0	0	0	0	0	0	0	0	0
Solyc10g007030.1.1	3	76	3	49	5	136	16	126	7	192	31	179	6
Solyc10g007040.2.1	0	0	0	0	0	0	0	0	0	0	0	0	0
Solyc10g007050.2.1	8	88	4	36	0	74	59	92	0	12	7	17	0
Solyc10g007060.2.1	2	0	1	0	1	2	3	2	5	7	8	5	6
Solyc10g007070.2.1	7	62	4	49	0	299	458	565	122	75	10	47	2
Solyc10g007100.2.1	0	0	0	0	0	0	0	0	0	0	0	0	0

Solyc10g007110.2.1	8	118	29	34	3	97	320	93	0	109	291	65	0
Solyc10g007120.2.1	1	28	2	9	0	26	27	32	1	18	1	25	0
Solyc10g007130.2.1	15	26	4	28	3	65	40	96	12	73	27	49	1
Solyc10g007140.2.1	13	68	10	50	4	97	86	95	18	101	26	73	2
Solyc10g007150.2.1	9	66	13	38	3	156	166	209	46	102	44	47	2
Solyc10g007180.2.1	10	291	18	200	10	429	180	626	45	724	92	966	11
Solyc10g007190.2.1	0	0	0	0	0	0	0	0	0	0	0	0	0
Solyc10g007200.2.1	2	14	0	0	0	15	3	12	1	40	8	27	2
Solyc10g007260.2.1	3	30	3	29	0	76	13	73	2	82	7	42	1
Solyc10g007270.2.1	0	0	0	0	0	0	0	0	0	0	0	0	0
Solyc10g007280.2.1	3	46	11	45	0	14	2	21	3	10	0	3	1
Solyc10g007290.2.1	11	37	11	26	14	6	22	35	5	128	14	109	1
Solyc10g007300.1.1	0	0	0	0	0	0	0	0	0	0	0	0	0
Solyc10g007310.1.1	5	23	0	23	0	59	1	50	0	397	139	476	4
Solyc10g007320.2.1	8	79	8	42	0	76	41	112	2	58	11	90	1
Solyc10g007330.1.1	2	2	2	0	0	5	13	14	1	3	2	3	1
Solyc10g007340.2.1	10	34	8	29	5	79	66	105	9	93	23	37	2
Solyc10g007350.2.1	0	0	0	0	0	0	0	0	0	0	0	0	0
Solyc10g007360.2.1	0	0	0	0	0	0	0	0	0	0	0	0	0
Solyc10g007390.2.1	5	119	3	59	9	186	78	240	32	211	31	156	4
Solyc10g007400.2.1	0	0	0	0	0	0	0	0	0	0	0	0	0
Solyc10g007410.2.1	4	47	2	24	0	7	19	29	0	105	5	62	0
Solyc10g007420.2.1	0	0	0	0	0	0	0	0	0	0	0	0	0
Solyc10g007440.2.1	2	8	0	2	0	9	4	25	1	3	0	11	0
Solyc10g007460.2.1	0	0	0	0	0	0	0	0	0	0	0	0	0
Solyc10g007470.2.1	6	18	2	27	1	35	27	61	8	64	8	49	0
Solyc10g007480.2.1	13	298	11	215	24	379	153	526	47	816	80	599	7
Solyc10g007500.2.1	13	150	15	63	8	100	64	122	16	232	22	144	3
Solyc10g007520.1.1	1	0	0	17	0	0	3	9	0	12	1	10	0
Solyc10g007550.2.1	4	12	0	13	0	22	42	28	2	25	11	77	2
Solyc10g007560.1.1	0	0	0	0	0	0	0	0	0	0	0	0	0
Solyc10g007570.2.1	1	0	0	0	0	17	10	5	0	5	3	15	0
Solyc10g007590.2.1	3	23	1	17	0	53	19	96	1	13	7	12	1
Solyc10g007600.2.1	1	375	3	136	0	34	10	163	0	824	4	276	0
Solyc10g007610.2.1	6	21	2	27	0	18	13	22	0	20	4	43	0
Solyc10g007640.2.1	0	0	0	0	0	0	0	0	0	0	0	0	0
Solyc10g007650.2.1	0	0	0	0	0	0	0	0	0	0	0	0	0
Solyc10g007660.2.1	0	0	0	0	0	0	0	0	0	0	0	0	0
Solyc10g007670.2.1	2	20	1	5	0	3	8	6	0	12	0	6	1
Solyc10g007680.2.1	1	1	0	0	1	9	6	6	1	5	0	3	0
Solyc10g007690.2.1	5	3815	352	2230	30	58	178	150	18	483	242	412	3

Solyc10g007700.2.1	4	35	3	18	1	23	19	64	4	70	4	31	0
Solyc10g007720.2.1	17	89	15	40	10	121	145	196	30	156	43	139	0
Solyc10g007730.2.1	1	1	0	10	0	10	6	3	0	0	1	3	0
Solyc10g007740.2.1	9	27	4	13	4	54	40	54	4	38	34	29	1
Solyc10g007750.1.1	0	0	0	0	0	0	0	0	0	0	0	0	0
Solyc10g007760.2.1	3	65	6	16	10	56	78	93	3	94	16	150	3
Solyc10g007770.2.1	22	222	17	179	21	494	412	646	43	317	53	124	1
Solyc10g007780.1.1	4	25	1	6	2	41	19	55	3	21	1	2	0
Solyc10g007800.2.1	0	0	0	0	0	0	0	0	0	0	0	0	0
Solyc10g007810.2.1	1	5	1	7	0	9	12	11	0	27	2	5	0
Solyc10g007820.2.1	0	0	0	0	0	0	0	0	0	0	0	0	0
Solyc10g007830.1.1	0	0	0	0	0	0	0	0	0	0	0	0	0
Solyc10g007840.2.1	2	1	0	14	0	2	10	13	0	5	2	9	0
Solyc10g007850.2.1	6	26	3	15	1	80	79	69	7	103	29	44	0
Solyc10g007870.2.1	0	0	0	0	0	0	0	0	0	0	0	0	0
Solyc10g007880.2.1	2	2	0	1	0	16	3	35	0	3	9	0	0
Solyc10g007890.2.1	1	2	0	4	0	17	4	8	0	0	0	5	0
Solyc10g007900.2.1	2	0	2	0	0	0	23	1	0	0	0	0	0
Solyc10g007930.2.1	1	16	3	9	0	23	61	42	0	9	0	11	2
Solyc10g007940.1.1	0	0	0	0	0	0	0	0	0	0	0	0	0
Solyc10g007950.2.1	3	8	4	10	1	6	9	31	1	21	13	14	2
Solyc10g007980.2.1	4	123	1	42	5	128	13	211	3	257	24	178	2
Solyc10g007990.2.1	5	41	0	2	0	49	17	87	8	50	6	28	8
Solyc10g008000.1.1	2	35	0	45	0	18	13	20	1	3	0	9	0
Solyc10g008010.2.1	13	261	15	265	9	645	204	823	50	265	58	170	4
Solyc10g008020.2.1	4	124	93	62	0	5	67	23	0	8	23	9	1
Solyc10g008040.2.1	0	0	0	0	0	0	0	0	0	0	0	0	0
Solyc10g008050.1.1	0	0	0	0	0	0	0	0	0	0	0	0	0
Solyc10g008060.2.1	3	4	1	1	0	1	8	6	0	15	0	15	0
Solyc10g008070.2.1	3	9	0	7	0	12	7	31	1	14	4	8	0
Solyc10g008090.1.1	0	0	0	0	0	0	0	0	0	0	0	0	0
Solyc10g008100.2.1	0	0	0	0	0	0	0	0	0	0	0	0	0
Solyc10g008110.2.1	17	191	20	150	28	404	324	469	54	332	51	244	2
Solyc10g008120.2.1	1	4	1	2	0	0	3	2	0	4	0	0	0
Solyc10g008130.2.1	0	0	0	0	0	0	0	0	0	0	0	0	0
Solyc10g008140.2.1	2	45	2	19	3	225	25	185	13	142	14	180	2
Solyc10g008160.2.1	1	13	4	4	0	0	0	0	0	2	1	10	0
Solyc10g008180.1.1	0	0	0	0	0	0	0	0	0	0	0	0	0
Solyc10g008190.2.1	4	43	2	17	1	30	17	42	5	75	15	29	0
Solyc10g008200.2.1	0	0	0	0	0	0	0	0	0	0	0	0	0
Solyc10g008210.2.1	0	0	0	0	0	0	0	0	0	0	0	0	0

Solyc10g008220.2.1	2	0	0	8	0	8	13	6	0	9	1	5	0
Solyc10g008230.1.1	3	8	3	11	0	7	29	19	1	9	3	8	0
Solyc10g008240.2.1	2	1	0	0	0	8	9	4	0	0	0	3	0
Solyc10g008250.1.1	0	0	0	0	0	0	0	0	0	0	0	0	0
Solyc10g008270.2.1	0	0	0	0	0	0	0	0	0	0	0	0	0
Solyc10g008280.2.1	12	43	5	79	9	168	75	133	22	144	58	155	3
Solyc10g008300.2.1	5	110	15	77	4	103	36	82	12	185	22	105	0
Solyc10g008310.1.1	0	0	0	0	0	0	0	0	0	0	0	0	0
Solyc10g008320.1.1	3	11	1	5	0	2	3	11	1	10	7	19	0
Solyc10g008340.2.1	9	41	6	22	2	87	52	95	14	39	14	30	4
Solyc10g008350.2.1	3	107	10	39	6	79	34	26	11	5	17	38	0
Solyc10g008390.2.1	0	0	0	0	0	0	0	0	0	0	0	0	0
Solyc10g008400.1.1	9	66	51	60	14	41	144	143	8	462	4	447	19
Solyc10g008410.1.1	4	854	6	246	0	31	0	35	0	57	14	49	0
Solyc10g008420.2.1	12	26	4	31	1	56	113	50	14	43	28	64	1
Solyc10g008430.1.1	1	2	2	5	0	1	27	19	3	2	1	1	0
Solyc10g008440.2.1	0	0	0	0	0	0	0	0	0	0	0	0	0
Solyc10g008450.1.1	0	0	0	0	0	0	0	0	0	0	0	0	0
Solyc10g008460.2.1	3	3	0	1	2	12	10	6	1	4	1	5	0
Solyc10g008470.2.1	1	2	0	0	0	2	3	1	0	2	4	6	1
Solyc10g008480.2.1	3	32	1	19	3	30	19	25	27	7	2	14	0
Solyc10g008490.2.1	2	16	0	3	0	1	7	20	11	0	0	0	0
Solyc10g008500.2.1	6	59	4	14	0	119	68	160	4	75	12	46	0
Solyc10g008510.1.1	0	0	0	0	0	0	0	0	0	0	0	0	0
Solyc10g008520.2.1	3	117	6	91	4	17	23	53	9	5	24	14	1
Solyc10g008560.2.1	6	38	4	28	1	87	17	115	9	135	30	89	3
Solyc10g008570.2.1	0	0	0	0	0	0	0	0	0	0	0	0	0
Solyc10g008580.1.1	5	4	1	0	0	12	4	11	0	14	11	5	2
Solyc10g008590.1.1	0	0	0	0	0	0	0	0	0	0	0	0	0
Solyc10g008600.2.1	6	21	3	9	1	47	37	59	2	23	4	21	0
Solyc10g008630.2.1	9	50	6	30	3	81	58	68	3	110	23	76	1
Solyc10g008640.2.1	5	86	6	47	4	65	37	107	3	246	20	95	0
Solyc10g008670.2.1	1	7	2	0	3	25	1	3	0	22	4	7	1
Solyc10g008700.1.1	0	0	0	0	0	0	0	0	0	0	0	0	0
Solyc10g008710.2.1	0	0	0	0	0	0	0	0	0	0	0	0	0
Solyc10g008720.2.1	0	0	0	0	0	0	0	0	0	0	0	0	0
Solyc10g008730.2.1	0	0	0	0	0	0	0	0	0	0	0	0	0
Solyc10g008740.2.1	5	267	31	175	0	34	96	107	1	188	63	157	0
Solyc10g008750.2.1	1	2	0	0	0	3	5	3	0	3	0	3	0
Solyc10g008760.2.1	1	0	1	0	0	2	2	3	1	6	2	1	0
Solyc10g008770.2.1	8	170	12	172	12	535	323	253	21	445	50	270	4

Solyc10g008790.2.1	7	36	3	17	2	46	58	50	3	71	9	52	1
Solyc10g008800.2.1	4	9	1	19	0	36	24	46	7	29	9	30	0
Solyc10g008840.2.1	1	0	1	0	0	0	16	3	0	19	0	22	0
Solyc10g008850.1.1	1	1	0	8	0	5	6	10	0	6	0	6	0
Solyc10g008870.2.1	1	23	1	4	0	10	0	15	0	18	6	22	1
Solyc10g008880.2.1	8	63	9	49	0	189	93	301	9	97	36	109	0
Solyc10g008890.2.1	0	0	0	0	0	0	0	0	0	0	0	0	0
Solyc10g008910.1.1	2	11	0	2	0	3	1	1	0	18	5	38	0
Solyc10g008930.1.1	0	0	0	0	0	0	0	0	0	0	0	0	0
Solyc10g008950.2.1	8	36	6	44	1	108	44	143	11	68	15	105	0
Solyc10g008960.1.1	0	0	0	0	0	0	0	0	0	0	0	0	0
Solyc10g008980.2.1	10	3692	321	2638	42	1517	1262	1686	33	2060	201	1797	19
Solyc10g008990.2.1	5	19	3	18	0	41	26	36	1	65	6	24	0
Solyc10g009000.2.1	3	10	2	0	0	59	9	29	3	8	2	3	1
Solyc10g009010.2.1	0	0	0	0	0	0	0	0	0	0	0	0	0
Solyc10g009020.2.1	3	6	0	0	0	39	11	38	6	5	6	3	0
Solyc10g009030.2.1	3	29	3	10	0	16	25	7	0	46	3	39	0
Solyc10g009040.2.1	0	0	0	0	0	0	0	0	0	0	0	0	0
Solyc10g009050.2.1	5	23	0	24	5	30	17	18	2	18	9	14	0
Solyc10g009060.1.1	1	3	0	5	0	15	1	6	0	0	4	5	0
Solyc10g009070.2.1	6	21	0	40	0	44	18	110	3	56	17	56	0
Solyc10g009080.2.1	2	23	12	43	8	12	10	2	0	14	5	25	0
Solyc10g009090.2.1	0	0	0	0	0	0	0	0	0	0	0	0	0
Solyc10g009110.1.1	11	183	30	294	8	225	38	96	9	179	16	404	1
Solyc10g009120.1.1	0	0	0	0	0	0	0	0	0	0	0	0	0
Solyc10g009130.2.1	0	0	0	0	0	0	0	0	0	0	0	0	0
Solyc10g009140.2.1	0	0	0	0	0	0	0	0	0	0	0	0	0
Solyc10g009150.2.1	0	0	0	0	0	0	0	0	0	0	0	0	0
Solyc10g009160.2.1	1	0	0	0	0	2	5	4	0	3	0	2	0
Solyc10g009170.1.1	0	0	0	0	0	0	0	0	0	0	0	0	0
Solyc10g009180.2.1	3	72	0	50	0	138	12	149	14	170	5	96	2
Solyc10g009190.1.1	6	226	24	49	44	304	344	167	658	28	224	28	54
Solyc10g009200.2.1	2	8	0	3	2	22	8	14	1	8	1	1	0
Solyc10g009210.2.1	8	99	9	77	3	79	48	120	18	38	30	49	0
Solyc10g009220.2.1	9	66	2	30	0	80	41	135	8	82	31	84	0
Solyc10g009230.1.1	8	49	7	43	2	78	22	87	4	28	2	24	0
Solyc10g009240.2.1	15	65	6	12	17	920	515	128	725	0	16	1	3
Solyc10g009270.2.1	0	0	0	0	0	0	0	0	0	0	0	0	0
Solyc10g009280.2.1	4	21	2	17	0	80	100	45	17	43	19	55	1
Solyc10g009290.1.1	2	2	4	1	3	26	25	0	2	3	1	9	0
Solyc10g009320.2.1	0	0	0	0	0	0	0	0	0	0	0	0	0

Solyc10g009330.2.1	4	5	2	8	2	24	17	34	5	10	6	16	0
Solyc10g009340.1.1	3	222	12	90	0	186	25	122	7	28	0	13	0
Solyc10g009350.2.1	0	0	0	0	0	0	0	0	0	0	0	0	0
Solyc10g009380.2.1	0	0	0	0	0	0	0	0	0	0	0	0	0
Solyc10g009400.2.1	1	0	0	0	0	1	2	6	3	3	1	4	0
Solyc10g009410.1.1	15	78	9	19	6	102	55	174	36	42	117	40	25
Solyc10g009420.2.1	0	0	0	0	0	0	0	0	0	0	0	0	0
Solyc10g009430.2.1	0	0	0	0	0	0	0	0	0	0	0	0	0
Solyc10g009480.2.1	0	0	0	0	0	0	0	0	0	0	0	0	0
Solyc10g009490.1.1	8	49	4	32	0	112	112	114	12	43	14	56	0
Solyc10g009520.2.1	0	0	0	0	0	0	0	0	0	0	0	0	0
Solyc10g009550.2.1	0	0	0	0	0	0	0	0	0	0	0	0	0
Solyc10g009570.2.1	16	420	39	183	0	19	37	21	2	117	34	190	5
Solyc10g009580.2.1	9	38	22	32	0	3	66	32	0	63	11	66	0
Solyc10g011660.2.1	2	16	3	23	0	53	5	101	2	15	12	2	0
Solyc10g011670.2.1	7	31	7	14	5	76	43	74	6	53	33	97	3
Solyc10g011680.2.1	1	4	0	9	0	2	0	0	0	3	5	7	0
Solyc10g011690.2.1	1	6	1	10	0	1	3	9	0	11	4	7	0
Solyc10g011700.2.1	1	4	1	2	0	4	3	9	0	30	0	9	0
Solyc10g011730.2.1	2	9	0	0	0	1	1	0	0	3	8	8	2
Solyc10g011740.2.1	6	302	18	123	7	174	15	121	5	64	19	99	1
Solyc10g011750.2.1	0	0	0	0	0	0	0	0	0	0	0	0	0
Solyc10g011760.2.1	0	0	0	0	0	0	0	0	0	0	0	0	0
Solyc10g011770.2.1	3	211	6	79	0	27	45	39	0	46	12	54	4
Solyc10g011790.2.1	6	34	4	26	4	82	32	71	14	59	6	31	0
Solyc10g011810.2.1	1	8	2	2	1	0	3	3	4	0	0	0	0
Solyc10g011820.2.1	2	51	2	22	1	13	9	5	1	9	5	9	4
Solyc10g011870.2.1	9	176	22	101	0	324	78	460	14	525	46	421	9
Solyc10g011880.1.1	0	0	0	0	0	0	0	0	0	0	0	0	0
Solyc10g011910.2.1	0	0	0	0	0	0	0	0	0	0	0	0	0
Solyc10g011930.1.1	0	0	0	0	0	0	0	0	0	0	0	0	0
Solyc10g011940.1.1	0	0	0	0	0	0	0	0	0	0	0	0	0
Solyc10g011960.1.1	1	2	0	0	0	5	3	4	0	4	3	0	0
Solyc10g011980.2.1	2	2	0	0	0	16	6	10	2	2	2	14	6
Solyc10g011990.1.1	0	0	0	0	0	0	0	0	0	0	0	0	0
Solyc10g012030.2.1	15	73	11	60	1	118	105	201	13	124	17	89	0
Solyc10g012050.2.1	9	47	7	30	0	27	83	20	1	0	0	0	0
Solyc10g012060.2.1	2	2	0	0	1	2	14	16	2	8	2	10	0
Solyc10g012070.2.1	11	220	25	195	14	322	402	455	52	376	100	254	9
Solyc10g012080.2.1	0	0	0	0	0	0	0	0	0	0	0	0	0
Solyc10g012160.2.1	2	4	2	1	0	33	7	19	0	29	6	8	0

Solyc10g012170.2.1	1	10	1	0	0	29	7	13	1	1	2	4	0
Solyc10g012210.1.1	0	0	0	0	0	0	0	0	0	0	0	0	0
Solyc10g012240.2.1	4	21	4	9	0	26	31	21	2	189	16	80	2
Solyc10g012370.2.1	2	8	0	1	0	17	9	27	0	14	0	14	0
Solyc10g012400.2.1	0	0	0	0	0	0	0	0	0	0	0	0	0
Solyc10g012410.2.1	1	34	1	23	0	1	3	13	0	19	7	4	0
Solyc10g012420.2.1	4	24	2	6	0	78	134	92	14	42	37	71	6
Solyc10g012430.2.1	8	12	2	0	1	61	146	129	26	3	1	7	0
Solyc10g017490.1.1	0	0	0	0	0	0	0	0	0	0	0	0	0
Solyc10g017510.2.1	0	0	0	0	0	0	0	0	0	0	0	0	0
Solyc10g017530.2.1	3	19	3	17	2	50	23	65	6	70	17	82	0
Solyc10g017570.2.1	0	0	0	0	0	0	0	0	0	0	0	0	0
Solyc10g017580.2.1	4	18	3	23	0	64	54	73	0	43	1	27	0
Solyc10g017600.2.1	5	26	4	8	0	28	34	29	7	49	18	36	0
Solyc10g017620.2.1	0	0	0	0	0	0	0	0	0	0	0	0	0
Solyc10g017810.1.1	1	1	0	0	0	6	7	3	1	2	2	0	0
Solyc10g017850.2.1	9	230	21	71	8	200	242	271	7	67	18	57	2
Solyc10g017880.1.1	10	71	4	38	1	125	61	141	17	171	30	171	2
Solyc10g017960.1.1	0	0	0	0	0	0	0	0	0	0	0	0	0
Solyc10g017970.1.1	0	0	0	0	0	0	0	0	0	0	0	0	0
Solyc10g017980.1.1	0	0	0	0	0	0	0	0	0	0	0	0	0
Solyc10g018120.1.1	5	7	1	0	0	85	55	158	1	12	0	13	0
Solyc10g018140.1.1	0	0	0	0	0	0	0	0	0	0	0	0	0
Solyc10g018150.1.1	0	0	0	0	0	0	0	0	0	0	0	0	0
Solyc10g018160.1.1	2	20	4	6	0	43	10	47	5	13	2	26	0
Solyc10g018250.1.1	0	0	0	0	0	0	0	0	0	0	0	0	0
Solyc10g018260.1.1	0	0	0	0	0	0	0	0	0	0	0	0	0
Solyc10g018300.1.1	14	1701	215	1226	12	471	578	951	17	323	133	407	9
Solyc10g018350.1.1	4	96	3	22	1	116	26	128	12	62	17	34	0
Solyc10g018510.1.1	0	0	0	0	0	0	0	0	0	0	0	0	0
Solyc10g018520.1.1	14	102	9	78	9	215	113	352	16	252	52	261	11
Solyc10g018530.1.1	0	0	0	0	0	0	0	0	0	0	0	0	0
Solyc10g018540.1.1	0	0	0	0	0	0	0	0	0	0	0	0	0
Solyc10g018590.1.1	5	121	9	55	0	30	32	49	3	33	18	28	0
Solyc10g018600.1.1	9	96	3	59	0	121	112	131	8	164	18	183	0
Solyc10g018610.1.1	0	0	0	0	0	0	0	0	0	0	0	0	0
Solyc10g018750.1.1	0	0	0	0	0	0	0	0	0	0	0	0	0
Solyc10g018760.1.1	1	1	0	0	0	5	6	0	9	1	1	0	0
Solyc10g018780.1.1	0	0	0	0	0	0	0	0	0	0	0	0	0
Solyc10g018810.1.1	0	0	0	0	0	0	0	0	0	0	0	0	0
Solyc10g018850.1.1	5	11	0	20	1	39	31	37	2	38	12	25	0

Solyc10g018870.1.1	3	42	0	33	2	100	77	142	10	149	24	75	0
Solyc10g018890.1.1	0	0	0	0	0	0	0	0	0	0	0	0	0
Solyc10g018900.1.1	0	0	0	0	0	0	0	0	0	0	0	0	0
Solyc10g019040.1.1	7	61	6	33	1	59	49	126	5	86	15	44	1
Solyc10g024290.1.1	0	0	0	0	0	0	0	0	0	0	0	0	0
Solyc10g024300.1.1	0	0	0	0	0	0	0	0	0	0	0	0	0
Solyc10g024310.1.1	1	2	0	1	0	12	5	10	1	11	0	9	0
Solyc10g024320.1.1	2	3	1	6	0	23	19	20	8	9	12	21	0
Solyc10g024360.1.1	1	1	1	12	0	3	8	11	0	0	0	0	0
Solyc10g024410.1.1	1	16	1	16	0	8	8	22	0	22	0	19	0
Solyc10g024420.1.1	7	0	0	2	0	53	8	31	0	29	50	4	0
Solyc10g024470.1.1	9	34	3	34	0	179	49	164	6	84	18	43	1
Solyc10g024490.1.1	0	0	0	0	0	0	0	0	0	0	0	0	0
Solyc10g037880.1.1	0	0	0	0	0	0	0	0	0	0	0	0	0
Solyc10g037890.1.1	2	11	0	10	0	14	11	19	0	11	1	4	0
Solyc10g037900.1.1	5	45	19	24	1	45	140	67	3	26	32	18	0
Solyc10g037950.1.1	2	15	0	5	0	36	4	28	2	33	9	15	1
Solyc10g037960.1.1	3	29	1	14	1	33	9	40	1	56	12	98	1
Solyc10g037980.1.1	4	30	3	15	0	147	23	91	6	136	20	77	1
Solyc10g038000.1.1	5	8	1	11	0	42	32	57	8	63	3	37	0
Solyc10g038060.1.1	0	0	0	0	0	0	0	0	0	0	0	0	0
Solyc10g038070.1.1	0	0	0	0	0	0	0	0	0	0	0	0	0
Solyc10g038080.1.1	0	0	0	0	0	0	0	0	0	0	0	0	0
Solyc10g038110.1.1	2	10	0	20	0	8	12	19	5	7	11	15	0
Solyc10g038120.1.1	6	27	3	23	1	138	59	139	11	91	26	101	0
Solyc10g038130.1.1	2	28	2	20	2	5	7	13	0	28	5	22	0
Solyc10g038190.1.1	0	0	0	0	0	0	0	0	0	0	0	0	0
Solyc10g039190.1.1	4	22	1	26	0	103	20	109	2	78	18	51	1
Solyc10g039270.1.1	3	3	1	1	0	10	49	18	3	26	1	13	1
Solyc10g039280.1.1	8	62	3	47	9	44	37	78	15	158	14	110	0
Solyc10g039290.1.1	4	88	12	48	0	21	10	74	0	12	7	11	0
Solyc10g039360.1.1	7	40	4	13	11	84	36	74	5	48	9	59	3
Solyc10g039370.1.1	0	0	0	0	0	0	0	0	0	0	0	0	0
Solyc10g039380.1.1	1	1	1	1	0	3	3	8	1	6	6	8	0
Solyc10g039430.1.1	1	10	1	7	0	35	22	65	5	36	8	11	0
Solyc10g044450.1.1	1	8	1	0	0	9	18	16	0	32	2	13	0
Solyc10g044460.1.1	0	0	0	0	0	0	0	0	0	0	0	0	0
Solyc10g044470.1.1	10	138	14	121	4	209	66	230	25	109	95	186	0
Solyc10g044480.1.1	6	31	4	30	2	53	39	90	11	116	10	85	0
Solyc10g044510.1.1	0	0	0	0	0	0	0	0	0	0	0	0	0
Solyc10g044520.1.1	0	0	0	0	0	0	0	0	0	0	0	0	0

Solyc10g044630.1.1	1	4	0	0	0	2	6	3	0	0	1	6	0
Solyc10g044640.1.1	0	0	0	0	0	0	0	0	0	0	0	0	0
Solyc10g044670.1.1	13	52	2	83	10	131	163	257	21	161	60	123	5
Solyc10g044680.1.1	0	0	0	0	0	0	0	0	0	0	0	0	0
Solyc10g044710.1.1	2	14	7	12	1	1	39	11	2	5	9	12	0
Solyc10g044720.1.1	0	0	0	0	0	0	0	0	0	0	0	0	0
Solyc10g044740.1.1	3	4	0	1	2	2	14	8	0	19	1	2	0
Solyc10g044750.1.1	0	0	0	0	0	0	0	0	0	0	0	0	0
Solyc10g044780.1.1	4	17	9	8	1	29	10	45	3	40	2	25	0
Solyc10g044830.1.1	0	0	0	0	0	0	0	0	0	0	0	0	0
Solyc10g044840.1.1	4	30	7	15	0	0	8	4	0	31	8	45	0
Solyc10g044880.1.1	0	0	0	0	0	0	0	0	0	0	0	0	0
Solyc10g044890.1.1	3	5	1	0	0	22	14	20	5	6	0	6	0
Solyc10g044900.1.1	1	13	0	2	1	15	8	22	3	21	5	6	0
Solyc10g044910.1.1	5	52	4	26	4	65	71	71	7	42	17	48	2
Solyc10g044960.1.1	2	10	0	6	0	38	6	17	2	8	4	24	0
Solyc10g045050.1.1	0	0	0	0	0	0	0	0	0	0	0	0	0
Solyc10g045170.1.1	0	0	0	0	0	0	0	0	0	0	0	0	0
Solyc10g045180.1.1	3	71	1	19	5	110	15	109	7	181	28	45	1
Solyc10g045190.1.1	1	7	0	1	0	27	2	14	0	54	2	33	0
Solyc10g045220.1.1	0	0	0	0	0	0	0	0	0	0	0	0	0
Solyc10g045240.1.1	1	14	0	0	0	1	1	4	1	2	4	0	0
Solyc10g045270.1.1	0	0	0	0	0	0	0	0	0	0	0	0	0
Solyc10g045280.1.1	0	0	0	0	0	0	0	0	0	0	0	0	0
Solyc10g045290.1.1	0	0	0	0	0	0	0	0	0	0	0	0	0
Solyc10g045310.1.1	4	33	1	11	0	24	19	92	2	40	6	62	1
Solyc10g045350.1.1	1	9	0	0	0	3	4	12	1	9	0	0	0
Solyc10g045380.1.1	0	0	0	0	0	0	0	0	0	0	0	0	0
Solyc10g045390.1.1	0	0	0	0	0	0	0	0	0	0	0	0	0
Solyc10g045400.1.1	5	41	3	28	0	65	32	93	4	60	12	54	0
Solyc10g045420.1.1	0	0	0	0	0	0	0	0	0	0	0	0	0
Solyc10g045430.1.1	0	0	0	0	0	0	0	0	0	0	0	0	0
Solyc10g045480.1.1	0	0	0	0	0	0	0	0	0	0	0	0	0
Solyc10g045490.1.1	1	2	0	0	0	7	3	4	2	6	3	4	0
Solyc10g045540.1.1	7	18	3	3	1	31	42	39	5	38	19	40	2
Solyc10g045550.1.1	2	42	1	7	0	68	12	24	1	29	6	35	0
Solyc10g045600.1.1	3	13	8	28	3	90	77	73	6	69	12	38	0
Solyc10g045620.1.1	3	65	16	38	0	205	79	241	9	152	10	81	2
Solyc10g045630.1.1	0	0	0	0	0	0	0	0	0	0	0	0	0
Solyc10g045640.1.1	0	0	0	0	0	0	0	0	0	0	0	0	0
Solyc10g046770.1.1	0	0	0	0	0	0	0	0	0	0	0	0	0

Solyc10g046930.1.1	12	84	9	54	4	189	148	225	36	324	57	217	2
Solyc10g046970.1.1	4	58	4	33	0	68	40	97	3	63	12	67	0
Solyc10g047000.1.1	1	13	0	11	0	49	9	31	4	43	6	8	0
Solyc10g047040.1.1	0	0	0	0	0	0	0	0	0	0	0	0	0
Solyc10g047050.1.1	2	42	1	29	2	16	23	20	1	71	21	76	0
Solyc10g047060.1.1	0	0	0	0	0	0	0	0	0	0	0	0	0
Solyc10g047120.1.1	3	13	1	6	0	27	10	47	3	12	5	19	1
Solyc10g047130.1.1	6	138	3	38	0	57	30	96	11	386	32	217	1
Solyc10g047140.1.1	2	14	2	6	4	26	22	42	1	30	0	38	0
Solyc10g047200.1.1	1	1	1	3	0	3	4	14	0	3	1	14	0
Solyc10g047210.1.1	1	2	3	3	4	4	1	4	0	18	5	6	0
Solyc10g047220.1.1	4	50	10	45	2	86	68	123	8	87	18	65	0
Solyc10g047240.1.1	2	3	0	2	0	5	6	1	0	41	3	3	0
Solyc10g047250.1.1	5	23	1	15	0	14	4	29	1	22	2	15	0
Solyc10g047260.1.1	1	3	3	1	0	2	1	4	0	5	3	0	0
Solyc10g047270.1.1	8	35	1	19	4	68	40	78	1	88	10	59	3
Solyc10g047290.1.1	7	42	4	26	1	63	75	91	16	89	25	59	0
Solyc10g047300.1.1	5	27	6	13	3	97	66	128	13	97	37	53	0
Solyc10g047320.1.1	5	37	6	33	9	26	48	52	5	92	11	44	4
Solyc10g047350.1.1	0	0	0	0	0	0	0	0	0	0	0	0	0
Solyc10g047360.1.1	3	52	0	33	1	55	32	74	4	65	34	47	0
Solyc10g047370.1.1	0	0	0	0	0	0	0	0	0	0	0	0	0
Solyc10g047410.1.1	0	0	0	0	0	0	0	0	0	0	0	0	0
Solyc10g047530.1.1	0	0	0	0	0	0	0	0	0	0	0	0	0
Solyc10g047540.1.1	0	0	0	0	0	0	0	0	0	0	0	0	0
Solyc10g047630.1.1	6	32	5	21	0	71	41	95	4	60	6	33	0
Solyc10g047640.1.1	1	5	1	5	0	6	28	15	1	19	7	1	1
Solyc10g047650.1.1	3	75	2	53	0	75	4	94	1	173	10	94	0
Solyc10g047670.1.1	7	26	1	37	3	94	42	106	80	68	17	49	1
Solyc10g047840.1.1	0	0	0	0	0	0	0	0	0	0	0	0	0
Solyc10g047890.1.1	0	0	0	0	0	0	0	0	0	0	0	0	0
Solyc10g047900.1.1	0	0	0	0	0	0	0	0	0	0	0	0	0
Solyc10g047930.1.1	6	97	4	24	1	72	54	98	5	37	9	38	0
Solyc10g047950.1.1	0	0	0	0	0	0	0	0	0	0	0	0	0
Solyc10g047960.1.1	5	38	4	18	2	15	25	50	3	96	26	115	2
Solyc10g048010.1.1	0	0	0	0	0	0	0	0	0	0	0	0	0
Solyc10g048030.1.1	6	213	33	19	34	46	84	11	41	2	3	0	0
Solyc10g048060.1.1	0	0	0	0	0	0	0	0	0	0	0	0	0
Solyc10g049210.1.1	4	58	1	34	0	19	16	21	1	55	6	21	0
Solyc10g049270.1.1	0	0	0	0	0	0	0	0	0	0	0	0	0
Solyc10g049360.1.1	3	24	0	24	0	22	20	41	1	216	3	192	0

Solyc10g049420.1.1	1	5	0	10	1	0	5	5	0	0	0	0	0
Solyc10g049450.1.1	0	0	0	0	0	0	0	0	0	0	0	0	0
Solyc10g049560.1.1	2	0	0	0	0	10	6	3	0	3	1	11	0
Solyc10g049570.1.1	1	0	0	0	0	0	1	1	1	6	4	4	0
Solyc10g049580.1.1	3	140	7	48	0	124	30	126	0	2	1	2	0
Solyc10g049590.1.1	0	0	0	0	0	0	0	0	0	0	0	0	0
Solyc10g049600.1.1	3	28	1	23	13	8	8	25	7	9	0	3	1
Solyc10g049620.1.1	12	281	17	110	7	370	193	310	24	677	171	349	1
Solyc10g049630.1.1	12	219	51	155	10	196	71	87	25	41	34	91	12
Solyc10g049640.1.1	0	0	0	0	0	0	0	0	0	0	0	0	0
Solyc10g049650.1.1	3	8	0	7	0	10	22	11	3	17	0	11	0
Solyc10g049710.1.1	2	120	6	170	4	181	36	323	12	79	33	44	0
Solyc10g049740.1.1	0	0	0	0	0	0	0	0	0	0	0	0	0
Solyc10g049800.1.1	0	0	0	0	0	0	0	0	0	0	0	0	0
Solyc10g049850.1.1	4	97	5	43	2	152	25	161	9	143	15	147	0
Solyc10g049860.1.1	0	0	0	0	0	0	0	0	0	0	0	0	0
Solyc10g049870.1.1	0	0	0	0	0	0	0	0	0	0	0	0	0
Solyc10g049890.1.1	2	23	0	5	2	112	8	90	1	56	13	34	1
Solyc10g049920.1.1	0	0	0	0	0	0	0	0	0	0	0	0	0
Solyc10g049970.1.1	5	73	19	58	15	98	479	185	52	138	38	98	4
Solyc10g050010.1.1	0	0	0	0	0	0	0	0	0	0	0	0	0
Solyc10g050020.1.1	0	0	0	0	0	0	0	0	0	0	0	0	0
Solyc10g050060.1.1	6	96	33	68	10	38	19	81	4	63	18	78	2
Solyc10g050110.1.1	6	74	41	157	1	26	69	102	3	993	21	497	8
Solyc10g050160.1.1	7	59	8	51	3	152	173	360	48	8	15	9	0
Solyc10g050170.1.1	11	56	4	41	0	97	88	162	14	120	58	104	1
Solyc10g050200.1.1	0	0	0	0	0	0	0	0	0	0	0	0	0
Solyc10g050210.1.1	0	0	0	0	0	0	0	0	0	0	0	0	0
Solyc10g050220.1.1	0	0	0	0	0	0	0	0	0	0	0	0	0
Solyc10g050230.1.1	1	0	0	4	0	8	0	2	0	7	7	11	0
Solyc10g050430.1.1	6	33	6	11	0	9	42	52	0	28	14	5	0
Solyc10g050440.1.1	2	287	37	188	0	23	163	72	0	106	11	104	1
Solyc10g050450.1.1	1	0	0	0	0	13	3	23	1	0	2	3	0
Solyc10g050470.1.1	0	0	0	0	0	0	0	0	0	0	0	0	0
Solyc10g050510.1.1	0	0	0	0	0	0	0	0	0	0	0	0	0
Solyc10g050540.1.1	6	12	2	14	0	36	17	33	5	41	18	26	0
Solyc10g050550.1.1	0	0	0	0	0	0	0	0	0	0	0	0	0
Solyc10g050680.1.1	0	0	0	0	0	0	0	0	0	0	0	0	0
Solyc10g050730.1.1	0	0	0	0	0	0	0	0	0	0	0	0	0
Solyc10g050770.1.1	0	0	0	0	0	0	0	0	0	0	0	0	0
Solyc10g050840.1.1	21	97	30	99	7	309	667	349	35	308	118	243	11

Solyc10g050850.1.1	4	23	1	20	0	16	15	43	3	12	4	4	0
Solyc10g050860.1.1	11	311	35	220	20	972	281	612	44	1400	239	663	17
Solyc10g050870.1.1	0	0	0	0	0	0	0	0	0	0	0	0	0
Solyc10g050880.1.1	0	0	0	0	0	0	0	0	0	0	0	0	0
Solyc10g050890.1.1	7	62	5	26	0	22	26	37	2	55	14	59	1
Solyc10g050910.1.1	0	0	0	0	0	0	0	0	0	0	0	0	0
Solyc10g050920.1.1	0	0	0	0	0	0	0	0	0	0	0	0	0
Solyc10g050960.1.1	0	0	0	0	0	0	0	0	0	0	0	0	0
Solyc10g050970.1.1	0	0	0	0	0	0	0	0	0	0	0	0	0
Solyc10g050990.1.1	0	0	0	0	0	0	0	0	0	0	0	0	0
Solyc10g051010.1.1	0	0	0	0	0	0	0	0	0	0	0	0	0
Solyc10g051020.1.1	18	114	52	119	26	1618	191	718	130	80	75	14	0
Solyc10g051030.1.1	15	26	26	42	12	168	203	146	33	66	59	48	6
Solyc10g051050.1.1	1	4	0	5	0	0	8	0	0	0	0	0	0
Solyc10g051070.1.1	0	0	0	0	0	0	0	0	0	0	0	0	0
Solyc10g051110.1.1	16	583	74	359	10	47	120	106	2	159	28	168	5
Solyc10g051120.1.1	0	0	0	0	0	0	0	0	0	0	0	0	0
Solyc10g051130.1.1	4	4	0	1	6	14	13	25	2	0	7	0	0
Solyc10g051140.1.1	1	0	0	0	0	7	10	0	4	0	1	0	0
Solyc10g051150.1.1	0	0	0	0	0	0	0	0	0	0	0	0	0
Solyc10g051200.1.1	12	42	21	34	6	83	89	87	4	63	7	47	5
Solyc10g051240.1.1	0	0	0	0	0	0	0	0	0	0	0	0	0
Solyc10g051270.1.1	4	26	2	21	1	25	15	14	0	14	6	17	0
Solyc10g051300.1.1	2	86	11	31	0	254	88	149	18	93	29	104	5
Solyc10g051310.1.1	4	54	2	28	1	77	49	77	2	42	6	53	0
Solyc10g051340.1.1	9	161	9	68	5	141	110	237	45	342	53	226	7
Solyc10g051350.1.1	5	45	2	37	0	108	40	143	10	119	52	65	2
Solyc10g051380.1.1	1	155	1	78	0	50	6	127	0	25	1	35	0
Solyc10g051390.1.1	1	91	0	15	5	53	6	81	2	90	12	63	2
Solyc10g052490.1.1	7	108	1	12	0	262	70	311	0	30	23	18	0
Solyc10g052500.1.1	0	0	0	0	0	0	0	0	0	0	0	0	0
Solyc10g052510.1.1	1	10	0	0	0	0	0	3	0	10	9	2	0
Solyc10g052600.1.1	0	0	0	0	0	0	0	0	0	0	0	0	0
Solyc10g052650.1.1	2	11	0	6	0	12	30	43	0	28	0	36	0
Solyc10g052660.1.1	0	0	0	0	0	0	0	0	0	0	0	0	0
Solyc10g052750.1.1	0	0	0	0	0	0	0	0	0	0	0	0	0
Solyc10g052770.1.1	0	0	0	0	0	0	0	0	0	0	0	0	0
Solyc10g052790.1.1	0	0	0	0	0	0	0	0	0	0	0	0	0
Solyc10g052880.1.1	3	74	6	51	0	21	6	39	1	33	2	17	1
Solyc10g053970.1.1	10	61	4	19	0	136	31	119	0	92	25	57	2
Solyc10g054010.1.1	1	1	0	10	0	0	3	1	0	6	5	4	0

Solyc10g054030.1.1	8	115	7	117	2	588	51	231	15	58	21	54	0
Solyc10g054040.1.1	0	0	0	0	0	0	0	0	0	0	0	0	0
Solyc10g054060.1.1	2	17	0	8	1	18	18	13	0	25	15	15	1
Solyc10g054080.1.1	13	81	14	41	8	54	48	60	1	126	31	135	0
Solyc10g054100.1.1	0	0	0	0	0	0	0	0	0	0	0	0	0
Solyc10g054110.1.1	3	11	2	42	0	1024	129	757	10	5	0	0	0
Solyc10g054130.1.1	0	0	0	0	0	0	0	0	0	0	0	0	0
Solyc10g054280.1.1	0	0	0	0	0	0	0	0	0	0	0	0	0
Solyc10g054320.1.1	4	6	0	9	0	18	12	15	3	4	2	0	0
Solyc10g054330.1.1	4	36	3	30	3	42	21	57	5	121	7	97	2
Solyc10g054380.1.1	0	0	0	0	0	0	0	0	0	0	0	0	0
Solyc10g054390.1.1	0	0	0	0	0	0	0	0	0	0	0	0	0
Solyc10g054400.1.1	0	0	0	0	0	0	0	0	0	0	0	0	0
Solyc10g054420.1.1	10	250	44	92	0	23	143	60	0	40	6	27	0
Solyc10g054440.1.1	24	843	410	638	143	3397	2094	2348	225	110	251	431	37
Solyc10g054560.1.1	0	0	0	0	0	0	0	0	0	0	0	0	0
Solyc10g054570.1.1	0	0	0	0	0	0	0	0	0	0	0	0	0
Solyc10g054590.1.1	0	0	0	0	0	0	0	0	0	0	0	0	0
Solyc10g054660.1.1	1	12	2	0	3	24	5	18	1	98	2	63	0
Solyc10g054670.1.1	0	0	0	0	0	0	0	0	0	0	0	0	0
Solyc10g054720.1.1	0	0	0	0	0	0	0	0	0	0	0	0	0
Solyc10g054730.1.1	0	0	0	0	0	0	0	0	0	0	0	0	0
Solyc10g054780.1.1	3	25	1	21	6	31	9	37	3	54	5	7	0
Solyc10g054790.1.1	0	0	0	0	0	0	0	0	0	0	0	0	0
Solyc10g054810.1.1	0	0	0	0	0	0	0	0	0	0	0	0	0
Solyc10g054820.1.1	0	0	0	0	0	0	0	0	0	0	0	0	0
Solyc10g054840.1.1	2	8	0	0	2	7	0	0	1	43	6	10	3
Solyc10g054850.1.1	0	0	0	0	0	0	0	0	0	0	0	0	0
Solyc10g054860.1.1	8	11	2	6	0	40	50	62	5	39	3	19	7
Solyc10g054870.1.1	2	80	12	53	0	0	1	2	0	0	4	2	0
Solyc10g054880.1.1	2	58	4	22	0	88	15	85	17	127	13	55	8
Solyc10g054900.1.1	5	3	0	0	2	28	3	36	10	27	49	2	2
Solyc10g054910.1.1	2	20	0	12	0	10	0	6	0	5	13	7	0
Solyc10g054930.1.1	3	9	0	13	2	23	19	11	1	17	7	17	0
Solyc10g055200.1.1	1	29	0	8	0	13	9	19	0	0	0	1	0
Solyc10g055230.1.1	0	0	0	0	0	0	0	0	0	0	0	0	0
Solyc10g055240.1.1	6	149	24	101	9	0	5	6	3	48	14	29	0
Solyc10g055250.1.1	0	0	0	0	0	0	0	0	0	0	0	0	0
Solyc10g055260.1.1	0	0	0	0	0	0	0	0	0	0	0	0	0
Solyc10g055390.1.1	6	117	9	106	0	60	108	96	1	27	5	29	0
Solyc10g055410.1.1	5	68	0	17	7	69	28	67	14	44	3	27	0

Solyc10g055420.1.1	0	0	0	0	0	0	0	0	0	0	0	0	0
Solyc10g055450.1.1	24	123	15	105	17	377	313	440	55	226	56	319	4
Solyc10g055470.1.1	0	0	0	0	0	0	0	0	0	0	0	0	0
Solyc10g055550.1.1	0	0	0	0	0	0	0	0	0	0	0	0	0
Solyc10g055560.1.1	0	0	0	0	0	0	0	0	0	0	0	0	0
Solyc10g055610.1.1	0	0	0	0	0	0	0	0	0	0	0	0	0
Solyc10g055620.1.1	1	0	0	0	0	6	5	1	0	0	0	4	0
Solyc10g055630.1.1	8	219	42	102	0	35	130	59	0	104	141	121	2
Solyc10g055640.1.1	1	3	0	5	0	4	6	20	0	7	4	9	0
Solyc10g055650.1.1	6	471	10	191	10	559	218	419	22	1017	33	284	15
Solyc10g055660.1.1	0	0	0	0	0	0	0	0	0	0	0	0	0
Solyc10g055670.1.1	7	85	11	43	18	76	116	95	15	180	37	207	4
Solyc10g055680.1.1	9	265	18	168	4	157	458	88	42	49	78	19	0
Solyc10g055700.1.1	4	14	5	8	5	26	18	22	4	9	2	2	0
Solyc10g055710.1.1	0	0	0	0	0	0	0	0	0	0	0	0	0
Solyc10g055720.1.1	8	67	24	22	1	31	34	55	4	48	11	70	0
Solyc10g055730.1.1	1	5	0	4	0	0	3	0	0	0	0	0	0
Solyc10g055740.1.1	0	0	0	0	0	0	0	0	0	0	0	0	0
Solyc10g055760.1.1	4	255	11	323	2	99	1	59	0	81	0	120	0
Solyc10g055770.1.1	1	1	0	5	0	0	1	0	0	8	3	3	0
Solyc10g055780.1.1	0	0	0	0	0	0	0	0	0	0	0	0	0
Solyc10g055800.1.1	8	685	3	178	6	2108	171	1652	8	29	42	23	0
Solyc10g055810.1.1	4	284	4	110	1	200	0	342	25	40	7	6	0
Solyc10g055820.1.1	0	0	0	0	0	0	0	0	0	0	0	0	0
Solyc10g061930.1.1	1	11	1	5	0	10	1	26	1	5	2	9	0
Solyc10g061940.1.1	0	0	0	0	0	0	0	0	0	0	0	0	0
Solyc10g061950.1.1	1	6	0	0	0	20	5	7	0	19	0	9	0
Solyc10g061960.1.1	1	12	1	4	0	25	10	36	1	14	0	2	0
Solyc10g061970.1.1	0	0	0	0	0	0	0	0	0	0	0	0	0
Solyc10g061990.1.1	0	0	0	0	0	0	0	0	0	0	0	0	0
Solyc10g062020.1.1	0	0	0	0	0	0	0	0	0	0	0	0	0
Solyc10g062160.1.1	1	3	0	0	0	3	8	3	1	0	8	0	1
Solyc10g062180.1.1	16	76	17	53	4	167	127	246	23	144	41	92	1
Solyc10g062200.1.1	0	0	0	0	0	0	0	0	0	0	0	0	0
Solyc10g062220.1.1	1	6	3	3	0	30	10	14	0	2	7	3	1
Solyc10g062340.1.1	7	71	7	65	1	165	112	207	9	153	13	103	5
Solyc10g074350.1.1	0	0	0	0	0	0	0	0	0	0	0	0	0
Solyc10g074440.1.1	0	0	0	0	0	0	0	0	0	0	0	0	0
Solyc10g074470.1.1	3	10	1	17	0	15	4	4	0	32	6	22	0
Solyc10g074500.1.1	9	181	7	79	0	355	68	372	24	151	38	111	3
Solyc10g074510.1.1	0	0	0	0	0	0	0	0	0	0	0	0	0

Solyc10g074520.1.1	0	0	0	0	0	0	0	0	0	0	0	0	0
Solyc10g074540.1.1	10	170	9	58	3	0	14	3	2	345	37	179	0
Solyc10g074550.1.1	0	0	0	0	0	0	0	0	0	0	0	0	0
Solyc10g074560.1.1	5	8	0	4	1	57	19	43	15	35	33	45	0
Solyc10g074570.1.1	3	19	0	3	0	29	9	37	2	14	3	14	0
Solyc10g074580.1.1	10	217	11	159	0	128	128	173	0	76	6	64	0
Solyc10g074590.1.1	5	40	2	33	0	41	126	138	10	84	21	46	2
Solyc10g074600.1.1	5	9	4	15	0	20	33	27	6	52	16	97	2
Solyc10g074610.1.1	5	16	0	6	0	18	22	29	0	28	1	47	0
Solyc10g074620.1.1	0	0	0	0	0	0	0	0	0	0	0	0	0
Solyc10g074630.1.1	2	46	4	13	0	56	17	49	5	54	3	19	0
Solyc10g074650.1.1	18	75	6	98	5	256	99	300	17	192	19	170	2
Solyc10g074680.1.1	0	0	0	0	0	0	0	0	0	0	0	0	0
Solyc10g074690.1.1	7	51	2	8	0	89	78	175	13	121	20	72	0
Solyc10g074700.1.1	5	12	0	2	0	41	19	56	7	13	1	19	0
Solyc10g074710.1.1	5	79	9	27	14	17	31	42	4	137	38	49	2
Solyc10g074720.1.1	0	0	0	0	0	0	0	0	0	0	0	0	0
Solyc10g074730.1.1	0	0	0	0	0	0	0	0	0	0	0	0	0
Solyc10g074740.1.1	4	326	37	196	31	96	27	61	26	193	46	202	5
Solyc10g074750.1.1	0	0	0	0	0	0	0	0	0	0	0	0	0
Solyc10g074790.1.1	11	91	10	77	5	298	360	295	7	868	345	863	13
Solyc10g074800.1.1	0	0	0	0	0	0	0	0	0	0	0	0	0
Solyc10g074810.1.1	7	35	3	18	3	84	61	81	6	48	12	65	4
Solyc10g074820.1.1	1	2	0	6	0	3	0	5	0	2	4	5	0
Solyc10g074860.1.1	0	0	0	0	0	0	0	0	0	0	0	0	0
Solyc10g074870.1.1	5	48	4	38	2	31	28	88	21	137	30	139	0
Solyc10g074880.1.1	8	44	1	63	8	978	102	746	47	206	83	202	2
Solyc10g074890.1.1	6	20	0	8	0	36	36	47	10	4	1	6	0
Solyc10g074900.1.1	1	1	0	0	0	3	2	8	0	10	2	5	0
Solyc10g074910.1.1	7	165	40	150	1	604	440	244	5	52	19	58	5
Solyc10g074920.1.1	10	42	8	20	0	67	95	176	0	10	10	3	0
Solyc10g074930.1.1	7	81	4	64	2	86	38	101	10	69	24	112	0
Solyc10g074940.1.1	13	112	11	91	12	175	102	201	20	300	33	186	4
Solyc10g074950.1.1	4	25	4	10	0	32	13	46	0	7	0	6	0
Solyc10g074960.1.1	1	3	0	4	0	0	5	4	0	5	0	16	0
Solyc10g074970.1.1	7	32	3	49	4	62	85	125	8	169	48	180	1
Solyc10g074980.1.1	8	301	35	198	7	949	565	1102	172	473	98	513	12
Solyc10g074990.1.1	2	2	2	0	0	11	9	7	1	7	3	13	0
Solyc10g075010.1.1	0	0	0	0	0	0	0	0	0	0	0	0	0
Solyc10g075020.1.1	1	24	4	7	0	13	24	9	4	6	3	7	2
Solyc10g075030.1.1	25	120	6	134	9	142	91	231	4	157	27	101	0

Solyc10g075040.1.1	7	68	13	39	11	83	41	57	7	83	16	40	12
Solyc10g075050.1.1	0	0	0	0	0	0	0	0	0	0	0	0	0
Solyc10g075070.1.1	13	4034	231	232	572	24765	13616	5776	6002	5847	4059	1615	1513
Solyc10g075090.1.1	6	254	12	10	34	3363	1236	77	756	371	412	7	177
Solyc10g075100.1.1	12	2505	90	34	281	6078	1364	221	3048	1787	233	299	207
Solyc10g075110.1.1	4	340	61	30	177	2476	1963	482	1841	15	232	8	17
Solyc10g075130.1.1	0	0	0	0	0	0	0	0	0	0	0	0	0
Solyc10g075150.1.1	2	173	2	109	0	662	21	762	6	44	0	5	0
Solyc10g075160.1.1	5	915	10	389	0	32	17	34	0	65	5	46	0
Solyc10g076180.1.1	0	0	0	0	0	0	0	0	0	0	0	0	0
Solyc10g076240.1.1	0	0	0	0	0	0	0	0	0	0	0	0	0
Solyc10g076250.1.1	14	24	1	15	1	351	1294	432	27	29	21	28	4
Solyc10g076260.1.1	1	2	0	1	3	7	7	6	1	20	4	23	0
Solyc10g076270.1.1	4	5	3	4	6	30	21	16	0	23	11	12	0
Solyc10g076280.1.1	0	0	0	0	0	0	0	0	0	0	0	0	0
Solyc10g076290.1.1	1	1	0	3	2	2	4	0	1	1	0	1	0
Solyc10g076300.1.1	0	0	0	0	0	0	0	0	0	0	0	0	0
Solyc10g076350.1.1	2	45	8	18	0	11	30	35	0	66	22	43	0
Solyc10g076360.1.1	3	36	3	7	7	21	11	10	5	77	3	31	0
Solyc10g076370.1.1	0	0	0	0	0	0	0	0	0	0	0	0	0
Solyc10g076410.1.1	0	0	0	0	0	0	0	0	0	0	0	0	0
Solyc10g076420.1.1	2	10	0	0	0	9	8	11	4	23	0	7	0
Solyc10g076450.1.1	0	0	0	0	0	0	0	0	0	0	0	0	0
Solyc10g076460.1.1	0	0	0	0	0	0	0	0	0	0	0	0	0
Solyc10g076480.1.1	2	4	3	7	2	60	6	32	3	5	3	5	4
Solyc10g076500.1.1	0	0	0	0	0	0	0	0	0	0	0	0	0
Solyc10g076510.1.1	15	24	3	20	4	265	30	84	8	1214	389	1128	4
Solyc10g076530.1.1	0	0	0	0	0	0	0	0	0	0	0	0	0
Solyc10g076540.1.1	0	0	0	0	0	0	0	0	0	0	0	0	0
Solyc10g076550.1.1	0	0	0	0	0	0	0	0	0	0	0	0	0
Solyc10g076560.1.1	0	0	0	0	0	0	0	0	0	0	0	0	0
Solyc10g076570.1.1	0	0	0	0	0	0	0	0	0	0	0	0	0
Solyc10g076580.1.1	1	2	0	0	0	8	6	9	1	5	0	3	0
Solyc10g076590.1.1	6	27	5	13	7	123	85	75	17	75	5	31	2
Solyc10g076600.1.1	8	22	5	20	0	205	141	267	26	96	4	73	1
Solyc10g076610.1.1	0	0	0	0	0	0	0	0	0	0	0	0	0
Solyc10g076660.1.1	1	2	0	0	2	5	11	5	27	0	0	0	0
Solyc10g076670.1.1	0	0	0	0	0	0	0	0	0	0	0	0	0
Solyc10g076680.1.1	0	0	0	0	0	0	0	0	0	0	0	0	0
Solyc10g076690.1.1	3	23	5	5	0	36	12	19	4	9	5	10	0
Solyc10g076700.1.1	0	0	0	0	0	0	0	0	0	0	0	0	0

Solyc10g076710.1.1	21	370	35	196	49	66	123	71	33	421	145	552	9
Solyc10g076720.1.1	4	20	0	3	0	2	37	4	4	21	7	15	1
Solyc10g076730.1.1	0	0	0	0	0	0	0	0	0	0	0	0	0
Solyc10g076740.1.1	4	10	0	11	0	24	25	36	3	21	0	19	0
Solyc10g076750.1.1	1	0	6	1	1	1	0	2	0	7	2	1	0
Solyc10g076760.1.1	5	21	2	16	1	76	38	19	0	29	0	28	1
Solyc10g076780.1.1	0	0	0	0	0	0	0	0	0	0	0	0	0
Solyc10g076790.1.1	0	0	0	0	0	0	0	0	0	0	0	0	0
Solyc10g076800.1.1	5	28	2	8	1	87	20	73	10	54	19	25	0
Solyc10g076810.1.1	1	4	0	2	3	0	3	2	0	10	0	4	0
Solyc10g076820.1.1	0	0	0	0	0	0	0	0	0	0	0	0	0
Solyc10g076830.1.1	0	0	0	0	0	0	0	0	0	0	0	0	0
Solyc10g076840.1.1	6	5	5	11	5	8	20	28	20	27	9	23	0
Solyc10g076860.1.1	4	37	2	5	0	17	50	32	1	28	21	28	0
Solyc10g076870.1.1	17	90	23	99	9	198	131	259	16	165	31	113	2
Solyc10g076880.1.1	0	0	0	0	0	0	0	0	0	0	0	0	0
Solyc10g076890.1.1	2	58	3	30	0	34	7	28	1	103	11	66	0
Solyc10g076900.1.1	0	0	0	0	0	0	0	0	0	0	0	0	0
Solyc10g076910.1.1	18	80	11	68	5	202	174	300	17	133	36	140	0
Solyc10g076920.1.1	0	0	0	0	0	0	0	0	0	0	0	0	0
Solyc10g076930.1.1	0	0	0	0	0	0	0	0	0	0	0	0	0
Solyc10g076990.1.1	22	120	17	123	6	207	108	280	19	330	50	257	9
Solyc10g077000.1.1	24	70	13	47	13	245	231	331	21	143	25	151	7
Solyc10g077010.1.1	1	22	3	34	0	50	13	57	0	127	3	146	1
Solyc10g077020.1.1	12	52	12	37	3	128	101	165	13	117	16	68	0
Solyc10g077030.1.1	1	23	0	6	0	17	12	47	2	36	3	13	0
Solyc10g077040.1.1	12	3064	623	2027	26	125	238	191	26	174	498	182	16
Solyc10g077050.1.1	8	35	7	21	0	29	117	79	10	109	24	106	1
Solyc10g077060.1.1	0	0	0	0	0	0	0	0	0	0	0	0	0
Solyc10g077070.1.1	18	329	16	383	4	849	424	1180	54	550	121	389	6
Solyc10g077080.1.1	0	0	0	0	0	0	0	0	0	0	0	0	0
Solyc10g077090.1.1	0	0	0	0	0	0	0	0	0	0	0	0	0
Solyc10g077100.1.1	0	0	0	0	0	0	0	0	0	0	0	0	0
Solyc10g077110.1.1	15	62	9	39	5	97	108	163	50	102	34	89	6
Solyc10g077120.1.1	11	8226	1109	4188	44	298	2867	946	79	1209	476	1342	37
Solyc10g077130.1.1	13	134	30	108	5	33	177	54	101	27	28	31	6
Solyc10g078150.1.1	6	162	18	77	8	268	184	379	30	267	51	219	6
Solyc10g078160.1.1	11	43	3	40	1	14	32	33	7	183	26	148	1
Solyc10g078170.1.1	0	0	0	0	0	0	0	0	0	0	0	0	0
Solyc10g078180.1.1	3	121	5	50	6	157	57	231	13	201	42	64	7
Solyc10g078190.1.1	3	1	0	1	0	0	3	3	0	13	9	36	0

Solyc10g078220.1.1	0	0	0	0	0	0	0	0	0	0	0	0	0
Solyc10g078230.1.1	0	0	0	0	0	0	0	0	0	0	0	0	0
Solyc10g078240.1.1	12	77	4	54	10	183	12	123	15	143	49	156	1
Solyc10g078250.1.1	0	0	0	0	0	0	0	0	0	0	0	0	0
Solyc10g078260.1.1	8	45	6	36	0	46	52	43	0	25	0	35	0
Solyc10g078270.1.1	0	0	0	0	0	0	0	0	0	0	0	0	0
Solyc10g078280.2.1	3	7	2	4	0	4	11	3	0	7	1	8	0
Solyc10g078290.1.1	13	174	28	120	11	332	199	540	55	249	98	160	2
Solyc10g078300.1.1	4	164	9	90	7	110	98	152	17	235	44	155	3
Solyc10g078310.1.1	7	94	9	37	7	47	52	48	3	70	15	25	1
Solyc10g078320.1.1	3	9	0	2	0	20	8	9	2	4	6	6	3
Solyc10g078330.1.1	0	0	0	0	0	0	0	0	0	0	0	0	0
Solyc10g078340.1.1	2	7	0	9	4	3	15	3	2	0	0	4	0
Solyc10g078350.1.1	0	0	0	0	0	0	0	0	0	0	0	0	0
Solyc10g078370.1.1	0	0	0	0	0	0	0	0	0	0	0	0	0
Solyc10g078380.1.1	0	0	0	0	0	0	0	0	0	0	0	0	0
Solyc10g078390.1.1	7	74	5	46	6	25	58	64	32	108	11	150	1
Solyc10g078400.1.1	2	4	2	4	0	7	10	10	0	4	2	1	0
Solyc10g078410.1.1	0	0	0	0	0	0	0	0	0	0	0	0	0
Solyc10g078420.1.1	0	0	0	0	0	0	0	0	0	0	0	0	0
Solyc10g078430.1.1	1	11	4	1	0	4	1	6	3	20	0	1	0
Solyc10g078440.1.1	1	47	12	48	4	73	2	30	1	50	1	38	0
Solyc10g078450.1.1	0	0	0	0	0	0	0	0	0	0	0	0	0
Solyc10g078470.1.1	0	0	0	0	0	0	0	0	0	0	0	0	0
Solyc10g078480.1.1	3	12	0	16	0	43	10	27	0	16	5	15	0
Solyc10g078490.1.1	3	34	4	27	3	44	31	118	11	40	37	31	4
Solyc10g078500.1.1	1	5	0	0	0	1	2	5	0	2	5	0	0
Solyc10g078510.1.1	2	23	1	7	2	9	12	7	5	3	0	5	0
Solyc10g078520.1.1	6	70	5	29	0	56	44	54	3	217	14	214	0
Solyc10g078530.1.1	13	332	33	161	38	60	43	43	34	179	31	135	3
Solyc10g078540.1.1	4	46	2	31	2	80	48	72	13	125	32	94	2
Solyc10g078550.1.1	12	300	7	183	12	211	295	318	16	55	6	15	0
Solyc10g078560.1.1	7	148	24	110	2	120	204	190	8	33	3	16	0
Solyc10g078570.1.1	1	16	0	7	0	26	3	17	0	8	0	2	0
Solyc10g078580.1.1	0	0	0	0	0	0	0	0	0	0	0	0	0
Solyc10g078590.1.1	5	281	17	132	11	648	159	600	29	287	10	203	0
Solyc10g078600.1.1	16	247	21	174	0	197	63	255	0	63	19	89	0
Solyc10g078610.1.1	0	0	0	0	0	0	0	0	0	0	0	0	0
Solyc10g078620.1.1	4	491	16	213	3	494	130	697	30	1614	141	1485	15
Solyc10g078630.1.1	2	629	1	167	2	135	44	364	11	528	108	521	25
Solyc10g078660.1.1	1	21	0	5	4	11	9	26	0	20	6	30	0

Solyc10g078670.1.1	0	0	0	0	0	0	0	0	0	0	0	0	0
Solyc10g078680.1.1	1	1	0	3	0	0	0	1	0	7	6	4	0
Solyc10g078690.1.1	7	254	9	226	9	550	128	279	31	835	153	1038	5
Solyc10g078700.1.1	0	0	0	0	0	0	0	0	0	0	0	0	0
Solyc10g078710.1.1	1	19	1	4	0	15	8	20	0	33	7	18	1
Solyc10g078720.1.1	0	0	0	0	0	0	0	0	0	0	0	0	0
Solyc10g078730.1.1	3	5	1	15	0	2	1	13	0	18	7	7	3
Solyc10g078740.1.1	5	19	4	4	20	10	13	33	10	130	36	94	4
Solyc10g078750.1.1	6	39	2	23	2	91	32	60	15	45	13	8	2
Solyc10g078770.1.1	1	10	0	2	0	2367	300	1482	5	7	21	1	0
Solyc10g078780.1.1	0	0	0	0	0	0	0	0	0	0	0	0	0
Solyc10g078790.1.1	7	82	5	20	0	73	53	100	16	76	32	49	3
Solyc10g078830.1.1	3	83	3	71	5	184	55	227	14	202	36	145	1
Solyc10g078860.1.1	4	5	0	3	0	27	12	18	0	22	4	33	1
Solyc10g078880.1.1	0	0	0	0	0	0	0	0	0	0	0	0	0
Solyc10g078900.1.1	5	40	1	28	0	63	20	46	7	25	12	22	0
Solyc10g078910.1.1	11	113	11	100	16	108	42	100	13	56	18	77	0
Solyc10g078920.1.1	4	25	0	18	0	27	35	35	0	78	5	41	1
Solyc10g078930.1.1	16	237	15	163	4	476	47	522	8	538	183	410	1
Solyc10g078940.1.1	0	0	0	0	0	0	0	0	0	0	0	0	0
Solyc10g078950.1.1	10	129	14	124	7	371	155	514	33	304	40	336	3
Solyc10g078960.1.1	2	71	2	43	1	136	7	110	4	276	40	285	2
Solyc10g079000.1.1	3	29	2	13	7	35	52	25	12	41	5	39	1
Solyc10g079010.1.1	20	123	8	115	4	333	145	510	20	225	29	240	2
Solyc10g079050.1.1	0	0	0	0	0	0	0	0	0	0	0	0	0
Solyc10g079070.1.1	6	35	6	30	9	24	19	18	4	100	25	87	0
Solyc10g079080.1.1	5	280	42	115	23	334	675	347	149	483	185	270	8
Solyc10g079090.1.1	3	18	0	6	0	21	18	17	2	13	15	12	0
Solyc10g079100.1.1	0	0	0	0	0	0	0	0	0	0	0	0	0
Solyc10g079110.1.1	6	12	0	14	0	14	17	16	1	13	1	16	1
Solyc10g079120.1.1	17	82	8	88	5	232	110	198	9	145	26	156	0
Solyc10g079130.1.1	4	106	8	57	3	76	20	88	11	71	7	104	0
Solyc10g079140.1.1	6	18	1	6	0	76	8	59	4	69	33	100	1
Solyc10g079150.1.1	0	0	0	0	0	0	0	0	0	0	0	0	0
Solyc10g079160.1.1	1	4	0	0	0	4	4	10	0	0	0	1	0
Solyc10g079170.1.1	0	0	0	0	0	0	0	0	0	0	0	0	0
Solyc10g079190.1.1	6	24	0	26	1	87	48	71	11	90	7	58	2
Solyc10g079200.1.1	7	822	34	278	6	77	27	96	2	48	16	39	0
Solyc10g079210.1.1	0	0	0	0	0	0	0	0	0	0	0	0	0
Solyc10g079240.1.1	0	0	0	0	0	0	0	0	0	0	0	0	0
Solyc10g079250.1.1	0	0	0	0	0	0	0	0	0	0	0	0	0

Solyc10g079260.1.1	3	26	7	14	1	81	29	73	1	25	13	45	0
Solyc10g079270.1.1	0	0	0	0	0	0	0	0	0	0	0	0	0
Solyc10g079280.1.1	3	5	1	4	0	24	5	15	1	41	4	15	0
Solyc10g079290.1.1	1	0	0	4	0	0	11	1	0	4	3	4	0
Solyc10g079300.1.1	0	0	0	0	0	0	0	0	0	0	0	0	0
Solyc10g079310.1.1	2	4	1	0	0	3	9	9	0	6	2	13	0
Solyc10g079320.1.1	0	0	0	0	0	0	0	0	0	0	0	0	0
Solyc10g079330.1.1	0	0	0	0	0	0	0	0	0	0	0	0	0
Solyc10g079350.1.1	0	0	0	0	0	0	0	0	0	0	0	0	0
Solyc10g079360.1.1	0	0	0	0	0	0	0	0	0	0	0	0	0
Solyc10g079370.1.1	2	23	1	11	0	16	28	26	1	133	6	51	0
Solyc10g079380.1.1	0	0	0	0	0	0	0	0	0	0	0	0	0
Solyc10g079410.1.1	0	0	0	0	0	0	0	0	0	0	0	0	0
Solyc10g079420.1.1	9	392	32	301	21	344	69	406	8	202	30	206	3
Solyc10g079430.1.1	1	5	0	8	0	15	3	8	0	12	1	4	0
Solyc10g079440.1.1	1	0	0	0	0	1	3	3	0	1	0	1	0
Solyc10g079450.1.1	3	51	0	34	1	52	26	74	7	55	6	43	1
Solyc10g079460.1.1	0	0	0	0	0	0	0	0	0	0	0	0	0
Solyc10g079470.2.1	8	71	39	69	9	174	149	169	41	90	47	74	7
Solyc10g079480.1.1	11	117	9	59	1	108	71	109	3	12	4	13	0
Solyc10g079490.1.1	3	20	2	8	0	8	7	5	1	27	4	28	0
Solyc10g079500.1.1	16	169	17	71	5	230	136	397	36	497	78	380	10
Solyc10g079510.1.1	0	0	0	0	0	0	0	0	0	0	0	0	0
Solyc10g079520.1.1	0	0	0	0	0	0	0	0	0	0	0	0	0
Solyc10g079530.1.1	0	0	0	0	0	0	0	0	0	0	0	0	0
Solyc10g079550.1.1	0	0	0	0	0	0	0	0	0	0	0	0	0
Solyc10g079560.1.1	8	10	2	25	0	38	40	56	14	26	18	21	1
Solyc10g079580.1.1	2	0	1	2	0	2	2	3	0	9	5	1	0
Solyc10g079600.1.1	0	0	0	0	0	0	0	0	0	0	0	0	0
Solyc10g079610.1.1	8	72	2	36	4	20	35	60	1	125	36	50	1
Solyc10g079620.1.1	7	227	57	150	15	111	14	48	0	44	19	30	13
Solyc10g079640.1.1	5	124	12	20	0	25	49	36	2	31	11	9	0
Solyc10g079690.1.1	2	4	0	7	0	27	6	7	0	11	70	3	0
Solyc10g079700.1.1	0	0	0	0	0	0	0	0	0	0	0	0	0
Solyc10g079710.1.1	3	9	3	22	0	24	23	33	6	56	3	29	4
Solyc10g079720.1.1	9	50	9	57	2	75	67	119	3	103	30	107	8
Solyc10g079730.1.1	0	0	0	0	0	0	0	0	0	0	0	0	0
Solyc10g079740.1.1	2	19	4	14	2	28	16	25	2	77	8	25	0
Solyc10g079750.1.1	0	0	0	0	0	0	0	0	0	0	0	0	0
Solyc10g079770.1.1	18	89	10	69	2	172	200	234	39	109	10	63	2
Solyc10g079780.1.1	0	0	0	0	0	0	0	0	0	0	0	0	0

Solyc10g079790.1.1	1	0	0	0	0	0	0	0	0	4	3	7	0
Solyc10g079810.1.1	1	31	10	13	2	13	6	17	2	2	2	8	0
Solyc10g079820.1.1	2	604	82	218	17	552	356	547	48	246	26	127	5
Solyc10g079830.1.1	1	5	0	1	0	2	4	2	0	14	0	3	0
Solyc10g079840.1.1	0	0	0	0	0	0	0	0	0	0	0	0	0
Solyc10g079850.1.1	20	54	4	57	1	232	106	172	16	137	53	79	4
Solyc10g079860.1.1	3	26	5	13	0	76	5	163	0	1	0	0	0
Solyc10g079870.1.1	0	0	0	0	0	0	0	0	0	0	0	0	0
Solyc10g079880.1.1	13	470	74	303	62	1531	540	1267	188	1610	490	931	47
Solyc10g079890.1.1	5	192	48	110	7	531	276	468	151	171	82	140	17
Solyc10g079910.1.1	0	0	0	0	0	0	0	0	0	0	0	0	0
Solyc10g079920.1.1	0	0	0	0	0	0	0	0	0	0	0	0	0
Solyc10g079930.1.1	15	161	40	79	5	434	158	327	13	56	0	45	1
Solyc10g079940.1.1	0	0	0	0	0	0	0	0	0	0	0	0	0
Solyc10g079950.1.1	1	9	0	1	0	16	12	12	1	14	5	6	0
Solyc10g079960.1.1	1	3	1	4	0	10	9	15	1	10	1	9	0
Solyc10g079970.1.1	7	33	9	26	0	46	83	69	8	326	59	117	1
Solyc10g079980.1.1	0	0	0	0	0	0	0	0	0	0	0	0	0
Solyc10g079990.1.1	1	4	0	2	0	4	2	11	2	12	2	10	0
Solyc10g080000.1.1	0	0	0	0	0	0	0	0	0	0	0	0	0
Solyc10g080010.1.1	0	0	0	0	0	0	0	0	0	0	0	0	0
Solyc10g080020.1.1	7	8	1	4	1	42	35	59	11	71	16	84	1
Solyc10g080030.1.1	0	0	0	0	0	0	0	0	0	0	0	0	0
Solyc10g080040.1.1	10	58	1	55	0	76	90	169	0	49	6	44	0
Solyc10g080070.1.1	0	0	0	0	0	0	0	0	0	0	0	0	0
Solyc10g080080.1.1	9	231	15	170	1	120	77	174	4	60	13	113	2
Solyc10g080090.1.1	4	17	0	6	2	46	40	52	4	37	16	3	1
Solyc10g080100.1.1	8	36	1	20	0	38	37	55	6	25	11	1	0
Solyc10g080110.1.1	5	8	2	6	1	8	27	26	1	74	5	91	0
Solyc10g080120.1.1	0	0	0	0	0	0	0	0	0	0	0	0	0
Solyc10g080130.1.1	0	0	0	0	0	0	0	0	0	0	0	0	0
Solyc10g080150.1.1	0	0	0	0	0	0	0	0	0	0	0	0	0
Solyc10g080160.1.1	6	37	1	25	8	65	26	66	3	116	23	76	0
Solyc10g080180.1.1	4	12	0	11	2	56	19	46	7	80	14	66	6
Solyc10g080190.1.1	2	43	4	15	4	21	22	46	0	77	20	34	4
Solyc10g080200.1.1	0	0	0	0	0	0	0	0	0	0	0	0	0
Solyc10g080210.1.1	0	0	0	0	0	0	0	0	0	0	0	0	0
Solyc10g080220.1.1	0	0	0	0	0	0	0	0	0	0	0	0	0
Solyc10g080230.1.1	3	7	3	13	3	27	18	17	7	9	3	10	1
Solyc10g080240.1.1	1	11	3	11	0	17	3	17	1	6	0	15	0
Solyc10g080260.1.1	8	28	3	43	7	30	46	16	4	63	31	36	2

Solyc10g080280.1.1	0	0	0	0	0	0	0	0	0	0	0	0	0
Solyc10g080290.1.1	0	0	0	0	0	0	0	0	0	0	0	0	0
Solyc10g080300.1.1	2	20	0	13	0	3	3	24	0	34	3	12	1
Solyc10g080320.1.1	6	132	3	89	5	372	49	371	6	433	66	254	1
Solyc10g080330.1.1	6	8	2	9	0	11	19	41	0	12	9	30	0
Solyc10g080340.1.1	0	0	0	0	0	0	0	0	0	0	0	0	0
Solyc10g080350.1.1	15	20	10	16	6	143	134	203	25	170	41	134	2
Solyc10g080360.1.1	2	182	1	39	0	12	3	6	0	81	0	29	0
Solyc10g080370.1.1	1	91	7	47	2	437	84	189	35	20	9	25	1
Solyc10g080380.1.1	1	25	0	34	0	3	5	9	0	0	0	0	0
Solyc10g080390.1.1	4	36	9	30	0	0	6	11	0	35	8	28	0
Solyc10g080400.1.1	0	0	0	0	0	0	0	0	0	0	0	0	0
Solyc10g080410.1.1	0	0	0	0	0	0	0	0	0	0	0	0	0
Solyc10g080420.1.1	11	164	6	102	13	133	115	221	22	502	65	504	13
Solyc10g080430.1.1	0	0	0	0	0	0	0	0	0	0	0	0	0
Solyc10g080440.1.1	8	50	2	31	1	94	82	151	16	72	20	77	0
Solyc10g080450.1.1	1	13	0	6	1	3	5	18	1	25	2	4	7
Solyc10g080460.1.1	0	0	0	0	0	0	0	0	0	0	0	0	0
Solyc10g080470.1.1	7	45	1	26	0	58	59	72	2	65	23	58	2
Solyc10g080480.1.1	3	15	1	1	1	43	18	20	8	10	5	12	0
Solyc10g080490.1.1	0	0	0	0	0	0	0	0	0	0	0	0	0
Solyc10g080500.1.1	6	400	31	180	12	181	40	211	12	500	77	265	2
Solyc10g080520.1.1	8	93	10	76	2	158	66	229	9	151	28	138	1
Solyc10g080530.1.1	1	4	0	0	0	3	4	4	0	6	1	1	0
Solyc10g080540.1.1	1	1	0	0	8	0	7	1	0	3	0	15	1
Solyc10g080550.1.1	0	0	0	0	0	0	0	0	0	0	0	0	0
Solyc10g080560.1.1	0	0	0	0	0	0	0	0	0	0	0	0	0
Solyc10g080570.1.1	0	0	0	0	0	0	0	0	0	0	0	0	0
Solyc10g080590.1.1	2	55	2	24	0	60	24	96	5	164	12	74	2
Solyc10g080600.1.1	0	0	0	0	0	0	0	0	0	0	0	0	0
Solyc10g080610.1.1	17	1510	67	1168	47	2333	2715	3653	89	4202	157	3322	42
Solyc10g080620.1.1	1	13	4	7	3	0	13	5	1	5	0	0	0
Solyc10g080630.1.1	17	51	0	62	3	223	94	202	26	139	23	154	0
Solyc10g080640.1.1	0	0	0	0	0	0	0	0	0	0	0	0	0
Solyc10g080660.1.1	4	67	1	29	0	64	55	127	10	106	14	70	1
Solyc10g080670.1.1	8	271	52	308	10	3816	1434	3110	129	203	77	113	10
Solyc10g080680.1.1	0	0	0	0	0	0	0	0	0	0	0	0	0
Solyc10g080690.1.1	0	0	0	0	0	0	0	0	0	0	0	0	0
Solyc10g080700.1.1	7	113	4	84	2	134	53	180	17	234	18	141	2
Solyc10g080710.1.1	11	236	10	221	7	919	140	946	18	642	63	615	5
Solyc10g080720.1.1	5	62	5	49	0	301	33	128	13	145	18	109	2

Solyc10g080730.1.1	0	0	0	0	0	0	0	0	0	0	0	0	0
Solyc10g080740.1.1	16	130	38	124	20	277	306	421	68	526	85	324	10
Solyc10g080760.1.1	0	0	0	0	0	0	0	0	0	0	0	0	0
Solyc10g080770.1.1	6	73	9	65	2	133	54	231	12	36	18	68	1
Solyc10g080810.1.1	6	37	13	22	1	33	62	58	11	39	11	44	2
Solyc10g080840.1.1	0	0	0	0	0	0	0	0	0	0	0	0	0
Solyc10g080870.2.1	0	0	0	0	0	0	0	0	0	0	0	0	0
Solyc10g080880.1.1	0	0	0	0	0	0	0	0	0	0	0	0	0
Solyc10g080890.1.1	1	3	0	0	4	1	5	4	2	6	1	2	0
Solyc10g080900.1.1	7	38	3	15	3	986	27	325	5	20	149	4	0
Solyc10g080910.1.1	0	0	0	0	0	0	0	0	0	0	0	0	0
Solyc10g080920.1.1	1	4	0	4	0	0	0	1	0	1	6	1	0
Solyc10g080930.1.1	2	7	3	3	0	16	9	16	2	7	4	12	0
Solyc10g080940.1.1	10	478	47	299	53	91	211	258	30	2010	401	1668	63
Solyc10g080950.1.1	0	0	0	0	0	0	0	0	0	0	0	0	0
Solyc10g080960.1.1	0	0	0	0	0	0	0	0	0	0	0	0	0
Solyc10g080970.1.1	9	196	10	111	6	219	99	393	19	223	33	195	1
Solyc10g080980.1.1	5	1	7	0	0	14	25	42	0	29	1	11	0
Solyc10g080990.1.1	0	0	0	0	0	0	0	0	0	0	0	0	0
Solyc10g081000.1.1	0	0	0	0	0	0	0	0	0	0	0	0	0
Solyc10g081010.1.1	6	27	2	17	0	4	17	23	3	56	5	20	0
Solyc10g081020.1.1	42	533	46	444	52	1014	515	1586	127	1124	207	758	20
Solyc10g081030.1.1	6	183	21	121	8	213	194	380	53	324	143	364	4
Solyc10g081040.1.1	4	771	35	554	28	170	13	77	5	360	25	681	27
Solyc10g081050.1.1	4	11	1	6	3	13	17	29	1	17	1	24	0
Solyc10g081060.1.1	5	54	5	15	0	80	196	92	12	90	10	27	1
Solyc10g081070.1.1	4	19	1	4	0	43	16	94	5	58	4	37	0
Solyc10g081080.1.1	0	0	0	0	0	0	0	0	0	0	0	0	0
Solyc10g081090.1.1	6	64	4	31	3	109	66	128	10	100	39	65	2
Solyc10g081100.1.1	6	424	67	148	10	73	419	124	7	171	42	132	6
Solyc10g081110.1.1	2	9	0	8	0	9	13	17	4	7	5	16	0
Solyc10g081120.1.1	15	137	33	96	10	200	519	573	42	239	19	150	3
Solyc10g081130.1.1	4	38	4	18	2	73	35	95	13	34	17	19	1
Solyc10g081140.1.1	1	2	0	4	0	2	4	4	2	2	0	1	0
Solyc10g081150.1.1	8	30	10	16	3	30	88	37	28	80	47	91	7
Solyc10g081160.1.1	5	62	13	30	2	3	25	18	19	126	13	71	0
Solyc10g081170.1.1	1	337	32	63	7	570	150	462	59	111	21	123	4
Solyc10g081180.1.1	5	226	13	76	9	503	133	441	56	313	128	167	9
Solyc10g081190.1.1	6	335	27	164	22	729	390	901	62	396	85	365	21
Solyc10g081200.1.1	19	48	9	48	7	88	140	210	15	182	33	140	0
Solyc10g081210.1.1	0	0	0	0	0	0	0	0	0	0	0	0	0

Solyc10g081220.1.1	1	3	2	4	0	10	3	10	1	4	0	3	0
Solyc10g081230.1.1	0	0	0	0	0	0	0	0	0	0	0	0	0
Solyc10g081240.1.1	10	44	8	38	1	45	90	153	4	68	26	77	0
Solyc10g081250.1.1	4	7	0	5	0	19	14	10	2	22	3	24	0
Solyc10g081260.1.1	19	105	13	68	8	153	21	135	7	618	230	341	2
Solyc10g081270.1.1	3	14	0	5	3	46	6	46	2	22	3	14	0
Solyc10g081280.1.1	0	0	0	0	0	0	0	0	0	0	0	0	0
Solyc10g081290.1.1	0	0	0	0	0	0	0	0	0	0	0	0	0
Solyc10g081300.1.1	5	29	2	4	0	19	47	45	0	2	0	1	0
Solyc10g081310.1.1	0	0	0	0	0	0	0	0	0	0	0	0	0
Solyc10g081320.1.1	0	0	0	0	0	0	0	0	0	0	0	0	0
Solyc10g081330.1.1	14	184	9	51	10	288	90	327	24	668	63	593	2
Solyc10g081340.1.1	0	0	0	0	0	0	0	0	0	0	0	0	0
Solyc10g081350.1.1	2	4	1	7	0	0	5	18	0	13	3	7	0
Solyc10g081360.1.1	2	8	0	13	2	12	7	38	5	35	12	26	0
Solyc10g081370.1.1	1	13	1	13	0	2	6	12	5	15	8	13	1
Solyc10g081380.1.1	4	33	4	15	1	41	21	30	0	25	4	14	0
Solyc10g081410.1.1	0	0	0	0	0	0	0	0	0	0	0	0	0
Solyc10g081420.1.1	0	0	0	0	0	0	0	0	0	0	0	0	0
Solyc10g081430.1.1	0	0	0	0	0	0	0	0	0	0	0	0	0
Solyc10g081440.1.1	6	168	13	45	4	83	62	163	19	111	28	66	1
Solyc10g081450.1.1	3	7	2	10	0	10	41	13	0	5	0	0	0
Solyc10g081460.1.1	16	383	30	283	11	304	311	450	22	1150	148	801	10
Solyc10g081470.1.1	9	111	11	50	0	33	88	58	1	70	11	52	1
Solyc10g081480.1.1	4	75	2	72	0	94	34	69	9	188	7	196	1
Solyc10g081490.1.1	0	0	0	0	0	0	0	0	0	0	0	0	0
Solyc10g081500.1.1	0	0	0	0	0	0	0	0	0	0	0	0	0
Solyc10g081510.1.1	17	1748	150	1034	274	2171	1324	2201	497	3292	2548	2676	71
Solyc10g081530.1.1	5	665	85	561	37	5830	1135	2425	185	1889	385	1127	21
Solyc10g081540.1.1	3	4	2	3	0	5	10	14	5	10	6	10	0
Solyc10g081560.1.1	0	0	0	0	0	0	0	0	0	0	0	0	0
Solyc10g081570.1.1	0	0	0	0	0	0	0	0	0	0	0	0	0
Solyc10g081580.1.1	5	121	11	30	9	106	54	117	22	181	42	112	2
Solyc10g081590.1.1	3	2	0	1	0	1	0	4	1	40	1	37	0
Solyc10g081600.1.1	7	67	5	26	12	144	61	112	9	129	16	45	3
Solyc10g081610.1.1	23	138	22	124	8	357	353	555	60	299	110	269	5
Solyc10g081620.1.1	7	22	2	20	5	50	52	60	7	92	24	53	3
Solyc10g081630.1.1	0	0	0	0	0	0	0	0	0	0	0	0	0
Solyc10g081650.1.1	15	147	24	114	6	276	213	393	11	179	48	126	1
Solyc10g081660.1.1	6	65	6	38	1	69	31	113	1	58	10	39	0
Solyc10g081670.1.1	0	0	0	0	0	0	0	0	0	0	0	0	0

Solyc10g081680.1.1	1	61	2	45	0	29	10	59	1	286	1	244	0
Solyc10g081700.1.1	0	0	0	0	0	0	0	0	0	0	0	0	0
Solyc10g081710.1.1	9	75	12	42	5	80	88	108	19	93	12	70	5
Solyc10g081720.1.1	12	753	49	316	71	65	128	214	93	1276	216	972	16
Solyc10g081730.1.1	10	226	11	93	18	17	68	39	14	712	34	423	12
Solyc10g081740.1.1	0	0	0	0	0	0	0	0	0	0	0	0	0
Solyc10g081750.1.1	2	46	12	14	5	49	41	50	5	57	22	56	0
Solyc10g081760.1.1	0	0	0	0	0	0	0	0	0	0	0	0	0
Solyc10g081770.1.1	0	0	0	0	0	0	0	0	0	0	0	0	0
Solyc10g081790.1.1	0	0	0	0	0	0	0	0	0	0	0	0	0
Solyc10g081810.1.1	0	0	0	0	0	0	0	0	0	0	0	0	0
Solyc10g081820.1.1	4	31	3	8	3	21	19	30	3	65	10	37	0
Solyc10g081830.1.1	1	8	1	0	0	4	3	1	2	8	1	2	0
Solyc10g081840.1.1	2	1	0	0	0	24	18	14	8	0	0	1	0
Solyc10g081850.1.1	6	59	20	38	9	63	28	38	14	56	15	67	4
Solyc10g081860.1.1	0	0	0	0	0	0	0	0	0	0	0	0	0
Solyc10g081870.1.1	1	3	0	3	0	4	5	0	1	12	4	7	0
Solyc10g081880.1.1	0	0	0	0	0	0	0	0	0	0	0	0	0
Solyc10g081900.1.1	12	108	12	69	13	279	55	255	12	98	21	89	1
Solyc10g081910.1.1	12	70	8	81	11	28	101	87	1	75	18	24	0
Solyc10g081920.1.1	16	208	28	178	36	315	144	409	47	609	78	496	8
Solyc10g081930.1.1	0	0	0	0	0	0	0	0	0	0	0	0	0
Solyc10g081940.1.1	0	0	0	0	0	0	0	0	0	0	0	0	0
Solyc10g081960.1.1	2	27	4	19	9	7	23	41	8	89	3	63	2
Solyc10g081970.1.1	0	0	0	0	0	0	0	0	0	0	0	0	0
Solyc10g081980.1.1	1	44	7	62	0	20	3	20	0	21	2	25	0
Solyc10g082000.1.1	0	0	0	0	0	0	0	0	0	0	0	0	0
Solyc10g082030.1.1	6	970	112	356	3	291	153	472	16	165	50	223	1
Solyc10g082050.1.1	0	0	0	0	0	0	0	0	0	0	0	0	0
Solyc10g083110.1.1	3	32	1	15	3	30	11	80	7	124	14	72	2
Solyc10g083120.1.1	1	21	4	4	0	15	34	28	10	53	32	13	1
Solyc10g083140.1.1	1	9	2	0	5	22	11	2	29	12	2	0	0
Solyc10g083150.1.1	10	44	9	62	4	162	97	183	11	64	12	54	2
Solyc10g083160.1.1	8	24	10	35	1	69	56	56	4	97	17	62	0
Solyc10g083180.1.1	2	4	0	10	4	1	12	2	0	12	5	8	0
Solyc10g083190.1.1	4	39	1	13	1	26	11	50	1	33	4	35	0
Solyc10g083200.1.1	10	35	4	10	0	37	63	85	19	71	29	47	3
Solyc10g083210.1.1	0	0	0	0	0	0	0	0	0	0	0	0	0
Solyc10g083220.1.1	0	0	0	0	0	0	0	0	0	0	0	0	0
Solyc10g083230.1.1	0	0	0	0	0	0	0	0	0	0	0	0	0
Solyc10g083240.1.1	2	8	2	7	0	16	2	25	1	56	4	34	0

Solyc10g083260.1.1	1	3	0	0	0	16	5	36	1	7	0	4	0
Solyc10g083270.1.1	9	13	1	23	1	48	44	40	7	84	14	64	2
Solyc10g083280.1.1	1	23	3	18	4	40	9	62	3	170	17	85	0
Solyc10g083290.1.1	2	17	2	10	0	76	5	63	5	0	2	0	0
Solyc10g083300.1.1	0	0	0	0	0	0	0	0	0	0	0	0	0
Solyc10g083310.1.1	0	0	0	0	0	0	0	0	0	0	0	0	0
Solyc10g083320.1.1	0	0	0	0	0	0	0	0	0	0	0	0	0
Solyc10g083330.1.1	5	51	14	67	1	18	33	37	4	57	28	68	1
Solyc10g083340.1.1	0	0	0	0	0	0	0	0	0	0	0	0	0
Solyc10g083350.1.1	9	57	28	43	2	30	240	61	5	26	6	31	1
Solyc10g083360.1.1	13	283	37	252	1	75	101	188	2	85	8	92	1
Solyc10g083370.1.1	0	0	0	0	0	0	0	0	0	0	0	0	0
Solyc10g083380.1.1	4	5	0	21	0	238	21	74	0	35	2	37	0
Solyc10g083400.1.1	2	0	0	0	0	0	0	0	0	14	11	3	5
Solyc10g083410.1.1	0	0	0	0	0	0	0	0	0	0	0	0	0
Solyc10g083440.1.1	6	82	0	29	6	128	5	80	14	144	17	73	0
Solyc10g083460.1.1	2	15	0	10	0	43	17	68	3	7	1	5	0
Solyc10g083470.1.1	0	0	0	0	0	0	0	0	0	0	0	0	0
Solyc10g083480.1.1	2	18	5	9	0	30	8	13	2	26	9	13	0
Solyc10g083490.1.1	2	11	0	3	0	18	13	14	1	4	3	21	0
Solyc10g083500.1.1	3	9	0	13	0	22	26	39	3	17	5	12	0
Solyc10g083510.1.1	0	0	0	0	0	0	0	0	0	0	0	0	0
Solyc10g083520.1.1	3	88	4	39	1	90	23	76	2	24	13	35	0
Solyc10g083530.1.1	0	0	0	0	0	0	0	0	0	0	0	0	0
Solyc10g083560.1.1	0	0	0	0	0	0	0	0	0	0	0	0	0
Solyc10g083570.1.1	7	152	16	69	30	132	198	200	51	839	1004	720	28
Solyc10g083580.1.1	5	0	0	0	0	3	5	5	1	147	256	287	0
Solyc10g083590.1.1	16	52	0	47	6	133	75	134	28	80	41	79	2
Solyc10g083600.1.1	4	40	3	29	1	167	40	172	10	156	30	86	0
Solyc10g083610.1.1	11	49	5	25	2	120	43	99	6	74	34	77	0
Solyc10g083630.1.1	20	102	14	62	6	87	96	151	19	207	127	134	9
Solyc10g083640.1.1	14	402	42	245	13	771	449	893	93	626	234	460	7
Solyc10g083650.1.1	5	75	12	50	0	99	38	128	5	134	41	121	0
Solyc10g083670.1.1	9	280	10	157	20	142	40	137	0	565	66	365	0
Solyc10g083690.2.1	0	0	0	0	0	0	0	0	0	0	0	0	0
Solyc10g083700.2.1	0	0	0	0	0	0	0	0	0	0	0	0	0
Solyc10g083710.1.1	0	0	0	0	0	0	0	0	0	0	0	0	0
Solyc10g083720.1.1	8	145	9	58	2	554	68	481	32	106	63	114	0
Solyc10g083730.1.1	4	47	1	63	1	146	29	193	1	301	8	161	0
Solyc10g083740.1.1	6	51	5	8	0	57	16	65	9	138	11	228	1
Solyc10g083760.1.1	13	212	16	109	32	70	64	109	16	701	90	334	0

Solyc10g083780.1.1	1	8	0	4	2	5	17	10	2	17	2	16	4
Solyc10g083790.1.1	7	77	8	50	0	44	56	63	1	60	8	47	2
Solyc10g083800.1.1	12	43	10	22	0	106	48	84	5	81	26	44	0
Solyc10g083830.1.1	5	7	0	17	0	33	37	65	0	38	2	36	0
Solyc10g083840.1.1	0	0	0	0	0	0	0	0	0	0	0	0	0
Solyc10g083860.1.1	2	134	10	317	6	10	3	19	0	135	0	222	0
Solyc10g083870.1.1	2	16	3	7	3	66	38	50	4	94	11	89	0
Solyc10g083880.1.1	0	0	0	0	0	0	0	0	0	0	0	0	0
Solyc10g083910.1.1	3	41	0	9	0	32	21	30	1	36	4	30	0
Solyc10g083920.1.1	11	60	24	61	15	180	220	201	27	124	15	45	5
Solyc10g083930.1.1	3	10	1	9	0	10	11	29	1	14	5	5	0
Solyc10g083940.1.1	0	0	0	0	0	0	0	0	0	0	0	0	0
Solyc10g083950.1.1	9	22	2	25	0	62	85	134	7	18	4	8	0
Solyc10g083960.1.1	5	15	4	29	0	13	45	38	0	15	2	28	0
Solyc10g083970.1.1	7	744	23	392	17	513	168	772	119	333	67	401	2
Solyc10g083990.1.1	2	117	6	67	0	19	10	35	3	20	1	9	0
Solyc10g084000.1.1	0	0	0	0	0	0	0	0	0	0	0	0	0
Solyc10g084040.1.1	4	36	2	8	0	53	28	56	0	29	2	38	0
Solyc10g084050.1.1	25	1362	39	729	43	3023	501	2358	273	1407	247	1019	12
Solyc10g084060.1.1	0	0	0	0	0	0	0	0	0	0	0	0	0
Solyc10g084070.1.1	0	0	0	0	0	0	0	0	0	0	0	0	0
Solyc10g084080.1.1	0	0	0	0	0	0	0	0	0	0	0	0	0
Solyc10g084090.1.1	0	0	0	0	0	0	0	0	0	0	0	0	0
Solyc10g084100.1.1	0	0	0	0	0	0	0	0	0	0	0	0	0
Solyc10g084120.1.1	0	0	0	0	0	0	0	0	0	0	0	0	0
Solyc10g084140.1.1	9	39	11	27	1	90	90	148	10	64	26	79	0
Solyc10g084150.1.1	0	0	0	0	0	0	0	0	0	0	0	0	0
Solyc10g084160.1.1	13	64	9	29	12	116	188	175	26	100	43	71	3
Solyc10g084180.1.1	16	183	15	99	0	197	170	262	0	121	19	131	1
Solyc10g084210.1.1	3	27	8	6	2	25	28	25	9	52	5	36	1
Solyc10g084220.1.1	5	9	6	9	4	25	11	61	7	54	15	25	0
Solyc10g084230.1.1	0	0	0	0	0	0	0	0	0	0	0	0	0
Solyc10g084240.1.1	0	0	0	0	0	0	0	0	0	0	0	0	0
Solyc10g084250.1.1	2	21	0	5	1	25	10	21	0	71	5	20	0
Solyc10g084260.1.1	4	52	2	16	1	80	59	45	8	126	15	64	0
Solyc10g084270.1.1	7	77	3	79	11	151	114	167	21	70	22	46	1
Solyc10g084280.1.1	13	215	10	67	11	112	150	171	12	269	24	140	8
Solyc10g084290.1.1	0	0	0	0	0	0	0	0	0	0	0	0	0
Solyc10g084300.1.1	0	0	0	0	0	0	0	0	0	0	0	0	0
Solyc10g084310.1.1	1	28	0	13	2	10	5	27	0	119	7	105	0
Solyc10g084320.1.1	0	0	0	0	0	0	0	0	0	0	0	0	0

Solyc10g084340.1.1	1	3	0	0	0	0	3	4	0	6	1	1	0
Solyc10g084350.1.1	5	129	17	75	13	1132	112	729	30	471	49	261	6
Solyc10g084360.1.1	1	2	0	0	0	3	2	5	0	3	1	3	0
Solyc10g084370.1.1	3	32	5	11	0	14	5	3	0	2	4	5	0
Solyc10g084380.1.1	0	0	0	0	0	0	0	0	0	0	0	0	0
Solyc10g084400.1.1	7	49	1	38	0	52	7	65	0	16	25	26	0
Solyc10g084420.1.1	0	0	0	0	0	0	0	0	0	0	0	0	0
Solyc10g084430.1.1	0	0	0	0	0	0	0	0	0	0	0	0	0
Solyc10g084440.1.1	1	1	0	2	0	0	3	5	0	3	0	5	0
Solyc10g084510.1.1	0	0	0	0	0	0	0	0	0	0	0	0	0
Solyc10g084520.1.1	1	2	0	8	0	8	6	12	0	18	9	11	3
Solyc10g084530.1.1	1	15	2	6	5	8	0	27	5	44	6	43	0
Solyc10g084540.1.1	0	0	0	0	0	0	0	0	0	0	0	0	0
Solyc10g084560.1.1	0	0	0	0	0	0	0	0	0	0	0	0	0
Solyc10g084590.2.1	0	0	0	0	0	0	0	0	0	0	0	0	0
Solyc10g084600.1.1	0	0	0	0	0	0	0	0	0	0	0	0	0
Solyc10g084610.1.1	0	0	0	0	0	0	0	0	0	0	0	0	0
Solyc10g084620.1.1	8	24	2	33	0	48	36	52	4	29	9	29	1
Solyc10g084630.1.1	4	8	0	7	0	13	10	37	0	9	4	23	0
Solyc10g084640.1.1	7	64	11	42	4	170	117	264	16	199	43	148	0
Solyc10g084650.1.1	11	51	8	36	7	79	62	118	20	34	20	45	0
Solyc10g084660.1.1	2	9	3	2	0	3	5	0	1	1	1	45	2
Solyc10g084670.1.1	0	0	0	0	0	0	0	0	0	0	0	0	0
Solyc10g084680.1.1	0	0	0	0	0	0	0	0	0	0	0	0	0
Solyc10g084690.1.1	0	0	0	0	0	0	0	0	0	0	0	0	0
Solyc10g084700.1.1	3	9	1	0	0	34	14	29	2	24	4	21	0
Solyc10g084750.1.1	0	0	0	0	0	0	0	0	0	0	0	0	0
Solyc10g084760.1.1	33	117	16	142	1	254	209	472	29	321	92	362	4
Solyc10g084770.1.1	1	15	1	0	0	0	5	19	2	4	2	7	1
Solyc10g084800.1.1	6	167	17	115	18	214	65	272	22	278	36	167	3
Solyc10g084810.1.1	0	0	0	0	0	0	0	0	0	0	0	0	0
Solyc10g084820.1.1	0	0	0	0	0	0	0	0	0	0	0	0	0
Solyc10g084830.1.1	0	0	0	0	0	0	0	0	0	0	0	0	0
Solyc10g084840.1.1	0	0	0	0	0	0	0	0	0	0	0	0	0
Solyc10g084850.1.1	2	16	1	9	0	0	19	16	0	6	6	19	0
Solyc10g084860.1.1	0	0	0	0	0	0	0	0	0	0	0	0	0
Solyc10g084880.2.1	9	42	33	95	29	24	76	15	37	6	4	67	4
Solyc10g084890.1.1	0	0	0	0	0	0	0	0	0	0	0	0	0
Solyc10g084920.1.1	2	6	1	10	0	8	4	9	0	19	3	9	0
Solyc10g084930.1.1	10	26	5	13	3	47	99	62	19	33	33	30	6
Solyc10g084940.1.1	12	69	6	53	0	87	68	58	0	1	1	3	0

Solyc10g084950.1.1	2	11	0	18	0	0	2	8	0	0	7	0	0
Solyc10g084960.1.1	1	1	1	0	0	22	3	11	4	2	0	0	0
Solyc10g084970.1.1	5	16	7	5	0	7	13	25	1	37	22	16	0
Solyc10g084980.1.1	0	0	0	0	0	0	0	0	0	0	0	0	0
Solyc10g084990.1.1	1	1	0	2	0	0	1	0	0	7	6	4	0
Solyc10g085000.1.1	3	55	0	23	1	36	8	29	0	48	7	66	0
Solyc10g085010.1.1	8	177	17	227	7	383	19	223	25	19	50	143	0
Solyc10g085020.1.1	10	357	2	181	8	190	23	267	6	731	116	594	3
Solyc10g085030.1.1	0	0	0	0	0	0	0	0	0	0	0	0	0
Solyc10g085040.1.1	7	74	7	47	6	35	31	52	4	114	81	85	6
Solyc10g085050.1.1	0	0	0	0	0	0	0	0	0	0	0	0	0
Solyc10g085060.1.1	6	10	2	11	0	17	56	57	1	55	23	67	1
Solyc10g085070.1.1	1	8	0	10	0	24	5	32	0	4	0	0	0
Solyc10g085080.1.1	2	5	0	14	0	0	15	3	0	4	0	0	0
Solyc10g085090.1.1	3	25	2	19	0	21	9	33	0	0	0	0	0
Solyc10g085100.1.1	8	79	23	68	3	41	139	38	3	50	22	30	3
Solyc10g085120.1.1	0	0	0	0	0	0	0	0	0	0	0	0	0
Solyc10g085130.1.1	0	0	0	0	0	0	0	0	0	0	0	0	0
Solyc10g085140.1.1	3	17	104	11	1	539	822	337	29	5	11	3	0
Solyc10g085150.1.1	3	2	4	8	0	48	4	25	0	5	3	3	0
Solyc10g085160.1.1	2	9	0	11	3	33	1	8	1	53	8	50	0
Solyc10g085180.1.1	6	23	7	11	0	53	15	41	15	22	25	34	5
Solyc10g085190.1.1	1	0	0	0	0	9	4	29	2	0	0	0	0
Solyc10g085200.1.1	5	49	3	40	5	107	159	241	16	42	1	63	0
Solyc10g085210.1.1	0	0	0	0	0	0	0	0	0	0	0	0	0
Solyc10g085220.1.1	10	177	6	94	7	77	29	106	3	101	26	174	0
Solyc10g085230.1.1	1	0	2	0	0	0	6	0	0	11	1	29	0
Solyc10g085240.1.1	0	0	0	0	0	0	0	0	0	0	0	0	0
Solyc10g085280.1.1	2	12	8	9	0	14	100	6	2	49	14	16	0
Solyc10g085290.1.1	7	53	2	13	0	57	12	57	9	59	14	51	0
Solyc10g085300.1.1	5	94	14	98	9	148	56	82	19	78	23	45	0
Solyc10g085310.1.1	0	0	0	0	0	0	0	0	0	0	0	0	0
Solyc10g085320.1.1	5	24	1	9	0	8	20	27	1	64	16	40	0
Solyc10g085330.1.1	0	0	0	0	0	0	0	0	0	0	0	0	0
Solyc10g085340.1.1	3	17	2	16	0	21	7	17	0	13	5	15	0
Solyc10g085350.1.1	0	0	0	0	0	0	0	0	0	0	0	0	0
Solyc10g085390.1.1	2	5	0	3	0	1	8	9	0	114	3	95	1
Solyc10g085400.1.1	0	0	0	0	0	0	0	0	0	0	0	0	0
Solyc10g085410.1.1	10	52	5	37	0	72	39	90	11	64	25	41	0
Solyc10g085420.1.1	4	104	50	82	13	61	7	38	5	12	0	41	0
Solyc10g085430.1.1	8	165	10	96	2	211	121	245	20	265	73	207	1

Solyc10g085440.1.1	0	0	0	0	0	0	0	0	0	0	0	0	0
Solyc10g085450.1.1	5	17	9	8	1	35	57	28	7	28	14	24	3
Solyc10g085460.1.1	2	25	3	15	3	3	1	8	0	20	4	9	0
Solyc10g085480.1.1	0	0	0	0	0	0	0	0	0	0	0	0	0
Solyc10g085490.1.1	1	1	0	0	0	2	2	2	0	1	2	1	0
Solyc10g085500.1.1	4	10	1	0	0	0	25	7	17	34	3	1	1
Solyc10g085540.1.1	0	0	0	0	0	0	0	0	0	0	0	0	0
Solyc10g085550.1.1	3	19	1	14	0	18	13	18	0	12	10	24	0
Solyc10g085560.1.1	4	17	0	11	1	49	24	59	9	43	24	48	2
Solyc10g085570.1.1	5	15	7	1	1	32	54	18	5	34	24	27	3
Solyc10g085580.1.1	5	17	5	14	0	61	14	106	8	75	12	59	5
Solyc10g085590.1.1	9	382	5	212	0	157	66	145	15	128	11	215	3
Solyc10g085600.1.1	2	7	0	8	0	36	20	40	2	13	2	12	2
Solyc10g085610.1.1	1	5	0	0	0	0	20	0	10	0	0	0	0
Solyc10g085620.1.1	0	0	0	0	0	0	0	0	0	0	0	0	0
Solyc10g085630.1.1	2	3	0	3	0	14	11	8	1	29	2	43	0
Solyc10g085660.1.1	1	1	0	0	0	1	10	19	0	14	4	18	0
Solyc10g085670.1.1	0	0	0	0	0	0	0	0	0	0	0	0	0
Solyc10g085680.1.1	7	41	3	36	1	11	9	23	2	30	12	24	1
Solyc10g085690.1.1	4	40	2	12	0	23	11	24	1	47	4	43	0
Solyc10g085710.1.1	14	81	11	65	2	149	84	223	13	114	51	101	5
Solyc10g085720.1.1	11	61	29	27	15	47	49	47	9	27	27	46	1
Solyc10g085740.1.1	0	0	0	0	0	0	0	0	0	0	0	0	0
Solyc10g085750.1.1	0	0	0	0	0	0	0	0	0	0	0	0	0
Solyc10g085790.1.1	6	12	2	10	3	33	54	49	17	40	36	65	0
Solyc10g085800.1.1	2	11	0	4	0	22	16	10	4	10	1	0	0
Solyc10g085810.1.1	3	113	9	68	0	68	64	86	7	97	12	150	0
Solyc10g085820.1.1	2	19	4	26	1	21	24	22	6	67	24	79	7
Solyc10g085830.1.1	14	423	26	159	17	155	42	64	21	70	123	28	4
Solyc10g085860.1.1	0	0	0	0	0	0	0	0	0	0	0	0	0
Solyc10g085870.1.1	0	0	0	0	0	0	0	0	0	0	0	0	0
Solyc10g085880.1.1	9	48	0	67	0	321	42	216	1	216	74	15	0
Solyc10g085890.1.1	0	0	0	0	0	0	0	0	0	0	0	0	0
Solyc10g085900.1.1	4	2	1	3	0	17	16	26	3	7	12	7	0
Solyc10g085910.1.1	2	56	2	33	0	48	25	50	3	32	8	41	1
Solyc10g085920.1.1	0	0	0	0	0	0	0	0	0	0	0	0	0
Solyc10g085930.1.1	2	7	0	2	0	8	4	12	0	7	2	11	0
Solyc10g085950.1.1	3	15	0	22	0	24	10	24	1	57	26	5	0
Solyc10g085960.1.1	0	0	0	0	0	0	0	0	0	0	0	0	0
Solyc10g085970.1.1	4	68	4	42	0	92	29	127	5	100	21	82	1
Solyc10g085980.1.1	3	43	3	14	0	11	19	15	3	10	3	15	0

Solyc10g085990.1.1	3	14	4	4	0	65	66	33	2	37	5	38	0
Solyc10g086000.1.1	19	60	17	25	3	429	157	544	26	130	30	61	5
Solyc10g086010.1.1	8	198	9	128	12	817	209	860	50	291	99	244	3
Solyc10g086020.1.1	3	50	0	16	6	84	27	61	8	237	21	127	5
Solyc10g086030.1.1	1	7	0	1	0	11	3	2	0	0	3	4	0
Solyc10g086040.1.1	1	8	0	6	0	25	4	9	2	3	2	6	0
Solyc10g086060.1.1	13	250	15	126	9	475	197	235	28	203	24	155	1
Solyc10g086070.1.1	5	12	0	23	0	21	31	56	10	22	9	6	0
Solyc10g086100.1.1	0	0	0	0	0	0	0	0	0	0	0	0	0
Solyc10g086120.1.1	3	3	4	16	0	5	6	8	0	27	13	24	0
Solyc10g086150.1.1	5	297	51	196	0	61	272	430	11	75	31	170	7
Solyc10g086170.1.1	16	170	27	75	6	65	155	142	21	51	69	46	1
Solyc10g086180.1.1	15	611	35	149	6	1317	377	1312	310	38	807	27	1
Solyc10g086190.1.1	6	655	57	217	16	768	349	1888	178	412	49	345	2
Solyc10g086210.1.1	1	1	3	2	0	5	9	10	2	7	6	18	0
Solyc10g086220.1.1	9	311	3	204	0	143	50	338	0	110	2	80	0
Solyc10g086240.1.1	0	0	0	0	0	0	0	0	0	0	0	0	0
Solyc10g086250.1.1	0	0	0	0	0	0	0	0	0	0	0	0	0
Solyc10g086280.1.1	5	313	49	265	11	21	7	17	2	29	0	178	0
Solyc10g086310.1.1	0	0	0	0	0	0	0	0	0	0	0	0	0
Solyc10g086320.1.1	0	0	0	0	0	0	0	0	0	0	0	0	0
Solyc10g086330.1.1	4	57	3	16	7	96	36	101	6	103	27	182	3
Solyc10g086340.1.1	0	0	0	0	0	0	0	0	0	0	0	0	0
Solyc10g086350.1.1	6	71	5	69	4	287	113	164	20	245	17	124	2
Solyc10g086360.1.1	2	6	0	6	0	6	5	10	1	14	3	28	0
Solyc10g086380.1.1	1	2	0	0	0	1	6	10	0	2	0	1	0
Solyc10g086390.1.1	0	0	0	0	0	0	0	0	0	0	0	0	0
Solyc10g086400.1.1	13	129	13	70	7	151	79	146	14	441	36	287	5
Solyc10g086410.2.1	13	398	29	212	25	1298	228	635	60	232	169	254	14
Solyc10g086420.1.1	10	83	11	63	2	279	208	238	13	75	9	71	0
Solyc10g086430.1.1	0	0	0	0	0	0	0	0	0	0	0	0	0
Solyc10g086440.1.1	0	0	0	0	0	0	0	0	0	0	0	0	0
Solyc10g086460.1.1	0	0	0	0	0	0	0	0	0	0	0	0	0
Solyc10g086470.1.1	3	38	4	4	2	22	29	20	2	21	1	9	0
Solyc10g086480.1.1	0	0	0	0	0	0	0	0	0	0	0	0	0
Solyc10g086490.1.1	0	0	0	0	0	0	0	0	0	0	0	0	0
Solyc10g086500.1.1	2	45	5	22	0	8	5	11	0	1	6	5	0
Solyc10g086510.1.1	1	7	0	5	0	20	1	15	2	8	5	13	0
Solyc10g086520.1.1	0	0	0	0	0	0	0	0	0	0	0	0	0
Solyc10g086530.1.1	0	0	0	0	0	0	0	0	0	0	0	0	0
Solyc10g086540.1.1	4	3	0	5	0	13	22	18	8	10	3	0	5

Solyc10g086550.1.1	17	217	19	184	3	637	217	571	54	403	74	300	3
Solyc10g086560.1.1	8	146	6	103	8	104	84	133	10	299	13	154	5
Solyc10g086570.2.1	7	245	21	166	3	8	438	56	8	163	8	124	1
Solyc10g086580.1.1	7	11868	1395	5524	7	76	352	428	9	1299	273	1501	7
Solyc10g086620.1.1	1	3	0	0	5	0	2	1	2	37	3	21	9
Solyc10g086630.1.1	0	0	0	0	0	0	0	0	0	0	0	0	0
Solyc10g086640.1.1	0	0	0	0	0	0	0	0	0	0	0	0	0
Solyc10g086650.1.1	4	7	4	10	4	2	7	4	0	39	17	36	0
Solyc10g086660.1.1	8	63	6	52	0	30	27	39	0	116	101	51	0
Solyc10g086680.1.1	1	2	0	0	0	14	4	10	0	0	1	0	0
Solyc10g086690.1.1	2	1	2	0	0	325	5	55	4	7	2	4	0
Solyc10g086700.1.1	0	0	0	0	0	0	0	0	0	0	0	0	0
Solyc10g086710.1.1	2	9	0	0	0	10	5	22	0	4	199	0	0
Solyc10g086720.1.1	0	0	0	0	0	0	0	0	0	0	0	0	0
Solyc10g086730.1.1	6	220	42	133	5	32	56	41	2	175	26	198	2
Solyc10g086760.1.1	2	12	3	2	2	0	7	3	0	12	15	21	0
Solyc10g086780.1.1	0	0	0	0	0	0	0	0	0	0	0	0	0
Solyc10g087010.1.1	0	0	0	0	0	0	0	0	0	0	0	0	0
Solyc10g087030.1.1	0	0	0	0	0	0	0	0	0	0	0	0	0
Solyc11g005000.1.1	1	20	3	8	0	52	19	65	3	39	3	23	0
Solyc11g005010.1.1	4	37	3	14	1	38	38	43	5	26	20	29	0
Solyc11g005020.1.1	2	3	1	3	0	5	7	8	2	5	5	3	0
Solyc11g005030.1.1	6	34	3	17	0	61	18	35	1	61	11	30	0
Solyc11g005040.1.1	1	1	0	5	0	5	5	1	0	2	1	4	0
Solyc11g005050.1.1	2	106	2	47	0	102	10	154	9	191	13	174	2
Solyc11g005060.1.1	2	3	0	6	0	25	2	20	0	23	3	17	0
Solyc11g005070.1.1	0	0	0	0	0	0	0	0	0	0	0	0	0
Solyc11g005080.1.1	8	93	28	103	7	45	349	156	21	137	67	179	8
Solyc11g005090.1.1	0	0	0	0	0	0	0	0	0	0	0	0	0
Solyc11g005100.1.1	0	0	0	0	0	0	0	0	0	0	0	0	0
Solyc11g005110.1.1	5	23	2	11	0	86	59	50	5	75	6	48	0
Solyc11g005130.1.1	4	17	2	9	1	35	25	35	3	13	18	21	0
Solyc11g005140.1.1	10	97	3	76	6	586	40	261	19	190	53	168	0
Solyc11g005150.1.1	10	142	2	67	1	20	121	24	0	445	45	320	1
Solyc11g005170.1.1	10	316	21	202	9	886	222	810	55	504	109	419	11
Solyc11g005180.1.1	0	0	0	0	0	0	0	0	0	0	0	0	0
Solyc11g005190.1.1	0	0	0	0	0	0	0	0	0	0	0	0	0
Solyc11g005200.1.1	4	18	2	8	0	40	21	31	5	28	32	16	0
Solyc11g005220.1.1	1	56	1	14	0	44	10	44	4	146	1	45	0
Solyc11g005230.1.1	0	0	0	0	0	0	0	0	0	0	0	0	0
Solyc11g005240.1.1	0	0	0	0	0	0	0	0	0	0	0	0	0

Solyc11g005250.1.1	4	28	5	19	0	44	57	63	4	14	6	31	0
Solyc11g005260.1.1	2	1	1	10	6	8	7	14	0	10	7	6	0
Solyc11g005270.1.1	0	0	0	0	0	0	0	0	0	0	0	0	0
Solyc11g005280.1.1	0	0	0	0	0	0	0	0	0	0	0	0	0
Solyc11g005290.1.1	0	0	0	0	0	0	0	0	0	0	0	0	0
Solyc11g005300.1.1	0	0	0	0	0	0	0	0	0	0	0	0	0
Solyc11g005310.1.1	0	0	0	0	0	0	0	0	0	0	0	0	0
Solyc11g005330.1.1	17	1952	239	1346	108	3127	2129	3450	543	7090	953	5636	91
Solyc11g005340.1.1	3	41	5	50	1	25	16	5	0	0	0	0	0
Solyc11g005350.1.1	3	23	0	59	1	21	20	56	0	7	0	5	0
Solyc11g005360.1.1	2	39	0	4	0	85	10	32	0	62	0	28	0
Solyc11g005370.1.1	1	74	3	21	3	89	25	54	3	102	8	67	0
Solyc11g005380.1.1	19	215	16	95	4	597	172	315	26	116	45	88	0
Solyc11g005390.1.1	0	0	0	0	0	0	0	0	0	0	0	0	0
Solyc11g005400.1.1	1	8	0	2	2	39	10	24	1	0	1	6	0
Solyc11g005420.1.1	0	0	0	0	0	0	0	0	0	0	0	0	0
Solyc11g005430.1.1	0	0	0	0	0	0	0	0	0	0	0	0	0
Solyc11g005440.1.1	0	0	0	0	0	0	0	0	0	0	0	0	0
Solyc11g005450.1.1	10	214	10	159	0	217	95	315	16	316	18	247	2
Solyc11g005460.1.1	3	23	8	17	1	39	17	28	7	16	4	21	0
Solyc11g005480.1.1	0	0	0	0	0	0	0	0	0	0	0	0	0
Solyc11g005490.1.1	0	0	0	0	0	0	0	0	0	0	0	0	0
Solyc11g005510.1.1	0	0	0	0	0	0	0	0	0	0	0	0	0
Solyc11g005550.1.1	2	3	0	0	0	16	6	13	0	11	0	6	0
Solyc11g005560.1.1	2	1	1	2	0	15	9	5	1	7	0	10	0
Solyc11g005570.1.1	1	1	0	5	0	6	9	5	0	5	4	10	0
Solyc11g005580.1.1	0	0	0	0	0	0	0	0	0	0	0	0	0
Solyc11g005590.1.1	3	13	2	2	8	23	27	10	3	13	4	22	0
Solyc11g005600.1.1	6	68	2	29	0	150	44	134	9	115	10	93	0
Solyc11g005610.1.1	0	0	0	0	0	0	0	0	0	0	0	0	0
Solyc11g005620.1.1	7	83	14	27	8	72	117	137	14	147	19	66	4
Solyc11g005630.1.1	8	154	17	94	18	127	8	100	1	28	4	13	6
Solyc11g005640.1.1	3	1	1	1	0	13	4	4	2	7	7	4	0
Solyc11g005650.1.1	0	0	0	0	0	0	0	0	0	0	0	0	0
Solyc11g005660.1.1	2	6	5	6	0	2	17	6	1	19	0	7	0
Solyc11g005670.1.1	10	203	98	180	52	553	383	431	73	634	112	848	22
Solyc11g005680.1.1	1	34	3	17	3	71	9	41	4	81	17	78	0
Solyc11g005690.1.1	0	0	0	0	0	0	0	0	0	0	0	0	0
Solyc11g005700.1.1	2	14	0	17	0	11	0	11	1	23	14	33	0
Solyc11g005710.1.1	0	0	0	0	0	0	0	0	0	0	0	0	0
Solyc11g005720.1.1	0	0	0	0	0	0	0	0	0	0	0	0	0

Solyc11g005730.1.1	0	0	0	0	0	0	0	0	0	0	0	0	0
Solyc11g005750.1.1	0	0	0	0	0	0	0	0	0	0	0	0	0
Solyc11g005760.1.1	4	18	0	12	0	23	5	42	1	0	31	0	0
Solyc11g005770.1.1	11	13	2	6	0	38	87	35	19	57	9	30	1
Solyc11g005790.1.1	0	0	0	0	0	0	0	0	0	0	0	0	0
Solyc11g005800.1.1	2	3	0	2	0	6	0	2	0	15	1	15	0
Solyc11g005810.1.1	6	43	3	13	3	63	36	74	2	35	15	54	2
Solyc11g005820.1.1	0	0	0	0	0	0	0	0	0	0	0	0	0
Solyc11g005870.1.1	0	0	0	0	0	0	0	0	0	0	0	0	0
Solyc11g005880.1.1	0	0	0	0	0	0	0	0	0	0	0	0	0
Solyc11g005900.1.1	9	24	4	12	3	30	34	56	6	64	8	49	0
Solyc11g005910.1.1	10	40	4	22	3	74	88	145	10	65	17	40	1
Solyc11g005920.1.1	15	116	16	156	7	255	137	295	45	295	44	212	3
Solyc11g005930.1.1	0	0	0	0	0	0	0	0	0	0	0	0	0
Solyc11g005940.1.1	4	4	1	10	0	23	36	25	8	22	5	18	0
Solyc11g005950.1.1	7	47	7	22	4	82	36	106	4	58	10	57	0
Solyc11g005960.1.1	0	0	0	0	0	0	0	0	0	0	0	0	0
Solyc11g005970.1.1	0	0	0	0	0	0	0	0	0	0	0	0	0
Solyc11g005980.1.1	0	0	0	0	0	0	0	0	0	0	0	0	0
Solyc11g006000.1.1	9	92	11	60	3	135	92	174	30	100	22	62	1
Solyc11g006010.1.1	0	0	0	0	0	0	0	0	0	0	0	0	0
Solyc11g006020.1.1	4	141	22	71	0	7	81	8	0	13	9	9	0
Solyc11g006030.1.1	0	0	0	0	0	0	0	0	0	0	0	0	0
Solyc11g006040.1.1	4	50	10	45	0	9	8	2	0	4	2	7	0
Solyc11g006060.1.1	12	796	65	445	1	468	637	880	21	302	88	189	1
Solyc11g006070.1.1	3	21	4	5	9	40	12	65	14	59	147	56	6
Solyc11g006080.1.1	9	35	10	26	2	35	62	56	10	89	34	70	0
Solyc11g006110.1.1	0	0	0	0	0	0	0	0	0	0	0	0	0
Solyc11g006130.1.1	0	0	0	0	0	0	0	0	0	0	0	0	0
Solyc11g006140.1.1	0	0	0	0	0	0	0	0	0	0	0	0	0
Solyc11g006150.1.1	0	0	0	0	0	0	0	0	0	0	0	0	0
Solyc11g006170.1.1	2	7	0	9	0	13	4	23	0	12	0	17	3
Solyc11g006180.1.1	4	21	4	12	3	79	29	45	2	22	5	10	0
Solyc11g006190.1.1	8	122	26	91	5	219	225	288	27	207	29	63	4
Solyc11g006200.1.1	4	5	2	15	0	17	14	47	7	23	10	15	3
Solyc11g006210.1.1	0	0	0	0	0	0	0	0	0	0	0	0	0
Solyc11g006220.1.1	0	0	0	0	0	0	0	0	0	0	0	0	0
Solyc11g006230.1.1	0	0	0	0	0	0	0	0	0	0	0	0	0
Solyc11g006250.1.1	8	91	2	7	40	5	4	26	1	2549	80	53	236
Solyc11g006270.1.1	2	17	2	9	0	53	50	46	3	129	1	75	4
Solyc11g006290.1.1	1	4	0	5	0	181	2	48	0	1	0	3	0

Solyc11g006300.1.1	1	14	0	12	0	8	5	1	0	0	5	5	0
Solyc11g006310.1.1	0	0	0	0	0	0	0	0	0	0	0	0	0
Solyc11g006320.1.1	10	24	5	31	7	138	62	85	24	130	20	103	6
Solyc11g006340.1.1	12	28	16	47	15	103	99	105	12	42	35	59	0
Solyc11g006350.1.1	4	39	1	20	1	68	43	98	4	56	29	39	2
Solyc11g006360.1.1	5	85	9	52	2	175	191	342	16	423	19	220	6
Solyc11g006370.1.1	4	16	0	3	0	21	43	64	7	74	4	42	0
Solyc11g006380.1.1	6	13	4	8	0	44	18	50	3	59	10	30	0
Solyc11g006390.1.1	21	85	16	93	8	186	144	215	30	236	47	278	2
Solyc11g006400.1.1	2	4	1	2	0	7	13	15	0	16	4	12	0
Solyc11g006410.1.1	15	45	6	27	1	159	43	182	18	37	52	33	0
Solyc11g006420.1.1	1	4	0	1	0	0	4	7	0	3	1	4	0
Solyc11g006430.1.1	0	0	0	0	0	0	0	0	0	0	0	0	0
Solyc11g006450.1.1	0	0	0	0	0	0	0	0	0	0	0	0	0
Solyc11g006460.1.1	5	3949	850	2813	431	5223	5572	2993	305	15519	2319	10290	321
Solyc11g006470.1.1	6	171	21	163	4	179	105	338	3	154	25	75	8
Solyc11g006480.1.1	3	27	5	8	1	33	15	40	3	48	0	38	0
Solyc11g006490.1.1	0	0	0	0	0	0	0	0	0	0	0	0	0
Solyc11g006500.1.1	1	6	0	5	2	8	0	3	0	9	3	1	0
Solyc11g006510.1.1	0	0	0	0	0	0	0	0	0	0	0	0	0
Solyc11g006520.1.1	0	0	0	0	0	0	0	0	0	0	0	0	0
Solyc11g006530.1.1	0	0	0	0	0	0	0	0	0	0	0	0	0
Solyc11g006540.1.1	3	18	1	6	0	5	3	10	2	6	6	11	0
Solyc11g006550.1.1	6	40	1	18	0	100	60	95	6	116	26	110	0
Solyc11g006560.1.1	3	7	0	10	0	38	8	21	0	30	4	14	0
Solyc11g006570.1.1	0	0	0	0	0	0	0	0	0	0	0	0	0
Solyc11g006580.1.1	1	10	0	14	0	5	4	6	0	19	4	20	0
Solyc11g006590.1.1	5	163	6	43	0	27	15	48	0	25	0	21	0
Solyc11g006620.1.1	8	62	6	30	5	124	86	241	22	58	29	95	0
Solyc11g006630.1.1	0	0	0	0	0	0	0	0	0	0	0	0	0
Solyc11g006640.1.1	0	0	0	0	0	0	0	0	0	0	0	0	0
Solyc11g006650.1.1	0	0	0	0	0	0	0	0	0	0	0	0	0
Solyc11g006660.1.1	6	21	2	28	2	80	18	87	3	76	12	96	0
Solyc11g006670.1.1	1	0	0	0	0	0	4	0	0	8	0	4	0
Solyc11g006680.1.1	15	74	4	48	0	39	80	104	1	142	18	59	1
Solyc11g006690.1.1	2	59	2	35	0	99	28	155	7	176	30	135	5
Solyc11g006700.1.1	0	0	0	0	0	0	0	0	0	0	0	0	0
Solyc11g006710.1.1	1	3	2	0	4	6	14	0	1	4	3	1	0
Solyc11g006720.1.1	2	23	0	12	0	58	18	20	2	18	5	12	0
Solyc11g006740.1.1	0	0	0	0	0	0	0	0	0	0	0	0	0
Solyc11g006750.1.1	3	0	2	3	2	7	19	18	7	72	33	81	0

Solyc11g006760.1.1	6	96	11	38	11	107	61	137	18	126	25	79	2
Solyc11g006770.1.1	8	174	8	95	1	495	109	434	24	161	39	95	13
Solyc11g006780.1.1	8	131	15	93	5	301	202	434	59	300	70	357	12
Solyc11g006790.1.1	0	0	0	0	0	0	0	0	0	0	0	0	0
Solyc11g006820.1.1	1	26	0	8	0	11	8	19	2	35	12	12	3
Solyc11g006830.1.1	1	2	0	0	1	5	4	4	0	1	0	0	0
Solyc11g006860.1.1	4	4	0	2	0	6	5	10	0	13	13	14	1
Solyc11g006870.1.1	0	0	0	0	0	0	0	0	0	0	0	0	0
Solyc11g006880.1.1	1	15	1	12	0	46	10	46	7	25	4	34	0
Solyc11g006890.1.1	1	2	0	0	0	0	4	2	0	5	0	3	2
Solyc11g006900.1.1	0	0	0	0	0	0	0	0	0	0	0	0	0
Solyc11g006910.1.1	0	0	0	0	0	0	0	0	0	0	0	0	0
Solyc11g006920.1.1	4	20	1	10	0	26	38	17	2	17	14	47	1
Solyc11g006930.1.1	0	0	0	0	0	0	0	0	0	0	0	0	0
Solyc11g006940.1.1	0	0	0	0	0	0	0	0	0	0	0	0	0
Solyc11g006950.1.1	0	0	0	0	0	0	0	0	0	0	0	0	0
Solyc11g006960.1.1	5	31	4	27	2	62	25	84	12	103	7	30	2
Solyc11g006970.1.1	3	20	1	13	0	68	105	124	4	470	13	435	8
Solyc11g006980.1.1	0	0	0	0	0	0	0	0	0	0	0	0	0
Solyc11g006990.1.1	9	80	9	57	0	45	92	69	5	492	31	111	4
Solyc11g007000.1.1	0	0	0	0	0	0	0	0	0	0	0	0	0
Solyc11g007010.1.1	2	15	5	13	0	26	15	38	6	13	2	10	0
Solyc11g007020.1.1	3	34	6	9	6	14	4	31	0	52	4	34	1
Solyc11g007030.1.1	1	6	1	0	0	28	8	19	7	0	0	0	0
Solyc11g007040.1.1	3	18	4	8	0	25	13	16	1	14	7	6	0
Solyc11g007060.1.1	7	29	3	26	3	54	50	78	1	79	8	95	0
Solyc11g007070.1.1	11	27	5	22	2	40	71	43	6	75	56	63	3
Solyc11g007080.1.1	2	5	3	12	3	18	1	11	0	16	4	20	0
Solyc11g007090.1.1	2	6	3	2	0	15	12	21	1	4	0	6	0
Solyc11g007110.1.1	6	43	3	27	5	83	37	91	15	47	14	61	1
Solyc11g007120.1.1	12	129	9	59	0	278	71	309	9	259	37	155	0
Solyc11g007130.1.1	4	27	3	37	3	26	72	81	15	109	5	76	5
Solyc11g007140.1.1	1	3	0	2	0	0	4	3	0	1	0	16	0
Solyc11g007160.1.1	2	4	0	2	0	0	2	0	0	16	3	14	0
Solyc11g007170.1.1	5	114	14	77	2	236	79	257	13	236	44	145	4
Solyc11g007190.1.1	0	0	0	0	0	0	0	0	0	0	0	0	0
Solyc11g007200.1.1	9	462	48	240	3	316	134	567	1	57	74	33	3
Solyc11g007230.1.1	0	0	0	0	0	0	0	0	0	0	0	0	0
Solyc11g007250.1.1	1	21	4	1	4	0	0	0	0	29	2	3	3
Solyc11g007260.1.1	0	0	0	0	0	0	0	0	0	0	0	0	0
Solyc11g007270.1.1	13	185	23	81	2	129	144	255	15	126	32	62	1

Solyc11g007290.1.1	0	0	0	0	0	0	0	0	0	0	0	0	0
Solyc11g007300.1.1	0	0	0	0	0	0	0	0	0	0	0	0	0
Solyc11g007310.1.1	2	2	1	0	0	6	5	4	0	11	0	13	0
Solyc11g007320.1.1	4	27	5	17	0	40	47	63	4	113	8	105	0
Solyc11g007330.1.1	0	0	0	0	0	0	0	0	0	0	0	0	0
Solyc11g007340.1.1	3	23	0	11	0	21	15	28	4	13	8	5	0
Solyc11g007350.1.1	0	0	0	0	0	0	0	0	0	0	0	0	0
Solyc11g007360.1.1	0	0	0	0	0	0	0	0	0	0	0	0	0
Solyc11g007390.1.1	0	0	0	0	0	0	0	0	0	0	0	0	0
Solyc11g007400.1.1	0	0	0	0	0	0	0	0	0	0	0	0	0
Solyc11g007410.1.1	0	0	0	0	0	0	0	0	0	0	0	0	0
Solyc11g007430.1.1	0	0	0	0	0	0	0	0	0	0	0	0	0
Solyc11g007460.1.1	0	0	0	0	0	0	0	0	0	0	0	0	0
Solyc11g007480.1.1	0	0	0	0	0	0	0	0	0	0	0	0	0
Solyc11g007490.1.1	0	0	0	0	0	0	0	0	0	0	0	0	0
Solyc11g007500.1.1	0	0	0	0	0	0	0	0	0	0	0	0	0
Solyc11g007510.1.1	4	55	7	31	4	134	68	36	7	93	1	43	8
Solyc11g007530.1.1	0	0	0	0	0	0	0	0	0	0	0	0	0
Solyc11g007540.1.1	3	62	3	3	10	3	7	0	16	111	8	8	6
Solyc11g007550.1.1	0	0	0	0	0	0	0	0	0	0	0	0	0
Solyc11g007570.1.1	0	0	0	0	0	0	0	0	0	0	0	0	0
Solyc11g007580.1.1	11	67	10	32	4	85	56	51	16	81	41	91	3
Solyc11g007590.1.1	3	39	3	30	0	33	28	34	10	19	21	38	1
Solyc11g007600.1.1	0	0	0	0	0	0	0	0	0	0	0	0	0
Solyc11g007610.1.1	2	1	1	2	4	12	15	20	2	1	6	3	6
Solyc11g007650.1.1	0	0	0	0	0	0	0	0	0	0	0	0	0
Solyc11g007660.1.1	9	199	13	152	3	160	45	128	11	65	17	68	1
Solyc11g007670.1.1	9	30	4	35	6	225	93	157	18	107	28	146	0
Solyc11g007680.1.1	1	44	2	25	0	56	22	27	1	27	2	24	0
Solyc11g007690.1.1	14	164	29	127	9	64	88	46	2	142	10	190	1
Solyc11g007700.1.1	1	2	0	2	0	6	2	3	0	3	4	3	0
Solyc11g007710.1.1	0	0	0	0	0	0	0	0	0	0	0	0	0
Solyc11g007720.1.1	7	56	1	17	2	116	40	98	5	105	17	49	1
Solyc11g007730.1.1	0	0	0	0	0	0	0	0	0	0	0	0	0
Solyc11g007740.1.1	1	1	1	1	0	5	2	10	1	20	3	0	0
Solyc11g007750.1.1	0	0	0	0	0	0	0	0	0	0	0	0	0
Solyc11g007760.1.1	6	71	11	57	0	392	108	205	9	48	8	37	1
Solyc11g007770.1.1	10	53	7	88	18	83	26	64	13	16	48	16	0
Solyc11g007780.1.1	3	19	2	15	4	60	23	84	6	33	2	19	0
Solyc11g007800.1.1	1	0	1	1	0	1	3	9	0	16	0	6	0
Solyc11g007810.1.1	3	32	2	15	0	26	40	30	4	98	14	81	1

Solyc11g007820.1.1	0	0	0	0	0	0	0	0	0	0	0	0	0
Solyc11g007830.1.1	11	238	1	104	2	256	471	388	8	415	16	305	6
Solyc11g007840.1.1	1	4	0	0	0	0	1	2	0	11	3	2	0
Solyc11g007850.1.1	15	33	0	44	0	143	117	271	5	111	23	113	3
Solyc11g007860.1.1	3	19	3	19	1	31	40	54	4	41	18	45	1
Solyc11g007870.1.1	0	0	0	0	0	0	0	0	0	0	0	0	0
Solyc11g007880.1.1	0	0	0	0	0	0	0	0	0	0	0	0	0
Solyc11g007890.1.1	0	0	0	0	0	0	0	0	0	0	0	0	0
Solyc11g007900.1.1	8	108	7	74	2	23	23	73	5	30	12	48	0
Solyc11g007910.1.1	13	72	3	38	1	108	59	110	13	109	28	104	1
Solyc11g007920.1.1	2	37	1	26	0	37	38	72	5	194	23	208	2
Solyc11g007930.1.1	0	0	0	0	0	0	0	0	0	0	0	0	0
Solyc11g007940.1.1	0	0	0	0	0	0	0	0	0	0	0	0	0
Solyc11g007950.1.1	6	180	6	258	0	461	151	456	2	5	1	16	0
Solyc11g007960.1.1	12	98	2	53	12	87	124	121	6	259	45	223	6
Solyc11g007990.1.1	11	725	79	567	26	728	369	879	66	474	225	703	43
Solyc11g008000.1.1	11	155	14	98	7	277	132	292	38	196	72	124	6
Solyc11g008010.1.1	10	97	5	34	0	258	98	497	31	171	28	173	2
Solyc11g008020.1.1	5	8	1	3	0	35	16	30	6	43	14	12	0
Solyc11g008040.1.1	0	0	0	0	0	0	0	0	0	0	0	0	0
Solyc11g008050.1.1	0	0	0	0	0	0	0	0	0	0	0	0	0
Solyc11g008060.1.1	0	0	0	0	0	0	0	0	0	0	0	0	0
Solyc11g008080.1.1	0	0	0	0	0	0	0	0	0	0	0	0	0
Solyc11g008090.1.1	2	2	0	4	0	6	12	3	0	28	0	18	1
Solyc11g008100.1.1	0	0	0	0	0	0	0	0	0	0	0	0	0
Solyc11g008110.1.1	0	0	0	0	0	0	0	0	0	0	0	0	0
Solyc11g008120.1.1	0	0	0	0	0	0	0	0	0	0	0	0	0
Solyc11g008130.1.1	1	9	0	5	0	29	14	38	0	32	4	26	0
Solyc11g008140.1.1	1	11	4	2	0	0	5	0	1	15	0	14	0
Solyc11g008150.1.1	42	343	51	275	25	217	352	597	75	665	179	495	15
Solyc11g008200.1.1	0	0	0	0	0	0	0	0	0	0	0	0	0
Solyc11g008210.1.1	5	103	14	45	8	174	81	237	21	135	28	103	1
Solyc11g008220.1.1	0	0	0	0	0	0	0	0	0	0	0	0	0
Solyc11g008250.1.1	11	156	25	156	1	115	50	216	15	15	32	23	0
Solyc11g008260.1.1	8	16	0	14	0	197	38	75	22	12	28	5	0
Solyc11g008270.1.1	4	342	26	218	11	260	146	264	29	311	73	185	36
Solyc11g008280.1.1	6	136	1	52	3	400	63	554	10	37	12	54	0
Solyc11g008290.1.1	2	8	0	6	0	19	6	20	2	10	1	14	0
Solyc11g008310.1.1	5	43	12	11	1	90	41	106	10	41	25	74	1
Solyc11g008320.1.1	10	36	5	17	2	130	93	127	9	163	24	58	6
Solyc11g008340.1.1	18	80	15	50	5	68	132	125	29	100	26	91	2

Solyc11g008350.1.1	1	4	0	5	0	6	4	2	0	9	0	30	0
Solyc11g008360.1.1	8	56	14	30	0	79	92	89	7	182	15	80	1
Solyc11g008370.1.1	2	1	4	0	0	16	24	13	0	2	3	6	0
Solyc11g008380.1.1	8	8	2	5	1	148	36	106	16	31	27	15	0
Solyc11g008390.1.1	21	79	15	125	4	230	252	276	44	143	44	109	5
Solyc11g008420.1.1	3	8	0	1	0	24	10	16	4	1	2	4	0
Solyc11g008430.1.1	4	172	1	66	2	314	45	234	6	307	6	187	0
Solyc11g008440.1.1	16	31	0	28	0	670	1726	1055	4	29	1	47	0
Solyc11g008450.1.1	0	0	0	0	0	0	0	0	0	0	0	0	0
Solyc11g008460.1.1	15	163	19	122	16	221	144	359	34	161	59	81	1
Solyc11g008470.1.1	6	159	13	120	4	210	69	313	7	164	27	141	3
Solyc11g008480.1.1	0	0	0	0	0	0	0	0	0	0	0	0	0
Solyc11g008490.1.1	0	0	0	0	0	0	0	0	0	0	0	0	0
Solyc11g008500.1.1	3	23	3	6	0	33	41	44	7	37	15	41	0
Solyc11g008510.1.1	3	126	9	54	26	131	72	207	19	217	58	334	7
Solyc11g008520.1.1	0	0	0	0	0	0	0	0	0	0	0	0	0
Solyc11g008530.1.1	1	1	0	7	0	3	0	0	0	2	4	3	0
Solyc11g008540.1.1	5	160	7	49	0	7	12	25	1	129	25	102	2
Solyc11g008550.1.1	12	52	1	25	0	46	49	116	13	73	28	44	7
Solyc11g008570.1.1	3	10	1	2	0	16	16	21	3	15	7	26	0
Solyc11g008580.1.1	12	180	26	94	12	222	228	280	25	246	40	134	7
Solyc11g008590.1.1	2	3	0	0	0	5	12	10	0	7	0	23	3
Solyc11g008600.1.1	0	0	0	0	0	0	0	0	0	0	0	0	0
Solyc11g008610.1.1	2	5	1	0	3	17	4	17	1	13	1	5	0
Solyc11g008620.1.1	11	572	60	332	2	120	101	161	4	70	19	161	2
Solyc11g008630.1.1	5	150	16	0	63	30	34	4	19	78	12	19	12
Solyc11g008670.1.1	1	2	1	0	0	2	2	0	0	1	1	4	0
Solyc11g008680.1.1	4	7	1	6	0	4	12	8	4	43	48	32	1
Solyc11g008690.1.1	0	0	0	0	0	0	0	0	0	0	0	0	0
Solyc11g008700.1.1	5	7	5	12	0	36	28	73	5	47	14	26	0
Solyc11g008710.1.1	3	9	0	6	0	27	22	22	15	32	10	11	0
Solyc11g008720.1.1	6	1	0	2	0	108	22	68	1	3	46	4	0
Solyc11g008730.1.1	4	3	1	3	0	4	8	3	2	4	1	3	0
Solyc11g008740.1.1	18	118	12	94	5	316	156	329	42	172	34	163	1
Solyc11g008760.1.1	0	0	0	0	0	0	0	0	0	0	0	0	0
Solyc11g008770.1.1	14	110	10	51	5	325	85	343	36	115	50	100	2
Solyc11g008780.1.1	9	73	18	73	17	31	47	52	8	45	117	25	0
Solyc11g008790.1.1	0	0	0	0	0	0	0	0	0	0	0	0	0
Solyc11g008800.1.1	27	73	12	80	3	502	1067	403	81	241	54	178	1
Solyc11g008810.1.1	6	13	2	9	1	1	11	9	1	28	7	29	0
Solyc11g008820.1.1	3	1	0	0	0	0	0	3	0	24	4	8	0

Solyc11g008830.1.1	0	0	0	0	0	0	0	0	0	0	0	0	0
Solyc11g008850.1.1	0	0	0	0	0	0	0	0	0	0	0	0	0
Solyc11g008860.1.1	0	0	0	0	0	0	0	0	0	0	0	0	0
Solyc11g008870.1.1	8	205	35	83	51	98	140	192	122	412	117	436	16
Solyc11g008880.1.1	0	0	0	0	0	0	0	0	0	0	0	0	0
Solyc11g008890.1.1	4	103	12	63	3	98	67	105	25	186	21	137	3
Solyc11g008900.1.1	7	182	4	41	3	76	71	96	11	189	16	111	2
Solyc11g008910.1.1	0	0	0	0	0	0	0	0	0	0	0	0	0
Solyc11g008940.1.1	3	9	1	17	0	38	15	73	3	44	8	4	0
Solyc11g008960.1.1	2	27	2	10	0	6	12	27	3	2	8	13	0
Solyc11g008970.1.1	0	0	0	0	0	0	0	0	0	0	0	0	0
Solyc11g008980.1.1	5	126	16	55	1	52	77	117	7	72	8	28	0
Solyc11g008990.1.1	3	57	1	38	5	60	31	87	3	31	7	55	2
Solyc11g009000.1.1	2	6	1	11	0	12	17	34	11	25	8	3	0
Solyc11g009010.1.1	3	12	4	18	0	44	16	32	7	43	13	12	0
Solyc11g009020.1.1	6	103	5	52	1	3	23	7	0	0	5	6	0
Solyc11g009030.1.1	1	6	1	2	0	0	2	5	0	5	0	4	0
Solyc11g009040.1.1	0	0	0	0	0	0	0	0	0	0	0	0	0
Solyc11g009050.1.1	4	46	7	22	11	28	35	38	11	191	70	165	16
Solyc11g009060.1.1	0	0	0	0	0	0	0	0	0	0	0	0	0
Solyc11g009070.1.1	5	35	10	11	0	19	20	33	2	35	11	9	0
Solyc11g009080.1.1	12	819	136	355	14	408	324	639	73	807	187	468	20
Solyc11g009090.1.1	4	113	2	50	2	183	36	287	4	224	13	286	2
Solyc11g009100.1.1	0	0	0	0	0	0	0	0	0	0	0	0	0
Solyc11g010100.1.1	1	25	1	15	0	6	10	15	2	35	8	24	1
Solyc11g010110.1.1	9	36	6	31	2	94	64	163	4	61	24	55	1
Solyc11g010120.1.1	1	13	0	5	0	1	5	15	1	9	0	11	0
Solyc11g010130.1.1	0	0	0	0	0	0	0	0	0	0	0	0	0
Solyc11g010140.1.1	0	0	0	0	0	0	0	0	0	0	0	0	0
Solyc11g010150.1.1	0	0	0	0	0	0	0	0	0	0	0	0	0
Solyc11g010160.1.1	0	0	0	0	0	0	0	0	0	0	0	0	0
Solyc11g010170.1.1	10	118	2	84	7	487	108	440	48	199	48	115	4
Solyc11g010180.1.1	2	14	0	4	1	2	0	20	2	92	3	55	1
Solyc11g010190.1.1	5	33	7	26	4	112	78	194	20	145	57	155	6
Solyc11g010200.1.1	5	177	9	76	14	63	66	103	17	389	128	385	30
Solyc11g010210.1.1	0	0	0	0	0	0	0	0	0	0	0	0	0
Solyc11g010220.1.1	0	0	0	0	0	0	0	0	0	0	0	0	0
Solyc11g010230.1.1	4	253	94	282	34	508	566	297	125	557	156	480	23
Solyc11g010240.1.1	3	26	2	5	0	20	6	40	0	16	4	41	0
Solyc11g010250.1.1	5	356	108	233	57	167	23	22	8	6	1	33	5
Solyc11g010260.1.1	0	0	0	0	0	0	0	0	0	0	0	0	0

Solyc11g010270.1.1	3	276	31	178	21	135	73	233	4	257	58	271	9
Solyc11g010280.1.1	0	0	0	0	0	0	0	0	0	0	0	0	0
Solyc11g010290.1.1	0	0	0	0	0	0	0	0	0	0	0	0	0
Solyc11g010300.1.1	4	17	2	1	0	5	26	20	1	24	5	12	0
Solyc11g010310.1.1	12	28	20	16	9	70	212	106	13	77	111	57	2
Solyc11g010320.1.1	0	0	0	0	0	0	0	0	0	0	0	0	0
Solyc11g010330.1.1	12	212	42	264	35	487	241	266	22	485	117	352	15
Solyc11g010350.1.1	0	0	0	0	0	0	0	0	0	0	0	0	0
Solyc11g010370.1.1	2	1	0	0	0	0	3	7	0	8	1	4	0
Solyc11g010380.1.1	11	223	9	117	1	187	202	186	5	66	36	31	4
Solyc11g010390.1.1	2	49	1	16	0	2	3	14	2	21	1	2	0
Solyc11g010420.1.1	0	0	0	0	0	0	0	0	0	0	0	0	0
Solyc11g010430.1.1	0	0	0	0	0	0	0	0	0	0	0	0	0
Solyc11g010440.1.1	5	8	0	1	0	0	4	1	0	93	15	103	0
Solyc11g010450.1.1	4	27	0	24	0	110	13	81	2	364	19	232	17
Solyc11g010460.1.1	0	0	0	0	0	0	0	0	0	0	0	0	0
Solyc11g010470.1.1	5	349	19	237	15	444	104	427	6	331	77	391	4
Solyc11g010480.1.1	23	687	42	467	0	367	219	876	17	231	56	324	1
Solyc11g010490.1.1	9	25	1	16	0	50	41	55	19	66	60	114	1
Solyc11g010500.1.1	6	412	39	258	3	256	34	190	0	126	6	324	16
Solyc11g010510.1.1	2	7	0	5	0	62	31	27	3	16	1	19	0
Solyc11g010520.1.1	1	0	0	0	0	5	8	3	0	0	9	1	0
Solyc11g010530.1.1	0	0	0	0	0	0	0	0	0	0	0	0	0
Solyc11g010540.1.1	0	0	0	0	0	0	0	0	0	0	0	0	0
Solyc11g010550.1.1	1	0	0	0	0	14	0	6	2	12	5	4	0
Solyc11g010560.1.1	15	293	30	294	7	152	54	163	8	458	57	441	6
Solyc11g010570.1.1	0	0	0	0	0	0	0	0	0	0	0	0	0
Solyc11g010580.2.1	4	33	0	13	2	5	14	29	5	26	10	18	0
Solyc11g010590.1.1	0	0	0	0	0	0	0	0	0	0	0	0	0
Solyc11g010600.1.1	14	1454	133	706	136	2046	1631	1855	273	1540	486	1105	33
Solyc11g010610.1.1	7	27	2	28	1	69	39	72	5	60	18	45	0
Solyc11g010620.1.1	0	0	0	0	0	0	0	0	0	0	0	0	0
Solyc11g010630.2.1	3	38	4	16	1	38	161	67	5	36	3	29	0
Solyc11g010650.2.1	7	117	3	59	10	68	27	149	9	146	11	114	2
Solyc11g010660.1.1	18	93	11	77	2	145	85	204	28	105	111	79	4
Solyc11g010670.1.1	1	20	1	15	5	1	3	12	0	1	1	11	0
Solyc11g010680.2.1	0	0	0	0	0	0	0	0	0	0	0	0	0
Solyc11g010690.1.1	2	3	1	0	0	5	6	13	0	11	4	5	0
Solyc11g010700.1.1	9	29	6	24	0	50	44	80	2	35	5	7	0
Solyc11g010710.1.1	2	0	0	4	0	45	0	12	0	3	6	2	3
Solyc11g010720.1.1	3	22	1	12	1	57	18	86	4	81	1	24	1

Solyc11g010730.1.1	0	0	0	0	0	0	0	0	0	0	0	0	0
Solyc11g010740.1.1	0	0	0	0	0	0	0	0	0	0	0	0	0
Solyc11g010760.1.1	0	0	0	0	0	0	0	0	0	0	0	0	0
Solyc11g010780.1.1	0	0	0	0	0	0	0	0	0	0	0	0	0
Solyc11g010790.1.1	0	0	0	0	0	0	0	0	0	0	0	0	0
Solyc11g010810.1.1	0	0	0	0	0	0	0	0	0	0	0	0	0
Solyc11g010830.1.1	0	0	0	0	0	0	0	0	0	0	0	0	0
Solyc11g010840.1.1	7	17	0	19	1	39	23	49	5	32	20	61	3
Solyc11g010850.1.1	12	915	17	186	10	136	31	93	5	8	47	8	1
Solyc11g010890.1.1	7	19	4	11	1	25	27	47	5	45	12	49	1
Solyc11g010900.1.1	3	4	0	6	0	2	17	7	0	6	1	0	0
Solyc11g010910.1.1	1	5	0	4	0	33	2	24	0	6	0	7	0
Solyc11g010920.1.1	0	0	0	0	0	0	0	0	0	0	0	0	0
Solyc11g010930.1.1	3	7	1	3	3	7	9	7	13	2	12	3	0
Solyc11g010940.1.1	0	0	0	0	0	0	0	0	0	0	0	0	0
Solyc11g010950.1.1	0	0	0	0	0	0	0	0	0	0	0	0	0
Solyc11g010960.1.1	11	181	8	4	56	22	73	15	79	1827	21	353	32
Solyc11g010980.1.1	0	0	0	0	0	0	0	0	0	0	0	0	0
Solyc11g010990.1.1	0	0	0	0	0	0	0	0	0	0	0	0	0
Solyc11g011010.1.1	0	0	0	0	0	0	0	0	0	0	0	0	0
Solyc11g011020.1.1	0	0	0	0	0	0	0	0	0	0	0	0	0
Solyc11g011030.1.1	4	88	0	31	1	32	3	2	0	6	0	4	0
Solyc11g011040.1.1	3	13	4	16	0	35	16	49	5	40	5	41	0
Solyc11g011050.1.1	0	0	0	0	0	0	0	0	0	0	0	0	0
Solyc11g011060.1.1	0	0	0	0	0	0	0	0	0	0	0	0	0
Solyc11g011070.1.1	0	0	0	0	0	0	0	0	0	0	0	0	0
Solyc11g011080.1.1	7	16	4	9	2	34	24	57	1	29	10	23	0
Solyc11g011090.1.1	3	5	0	2	1	27	25	36	5	6	4	10	0
Solyc11g011110.1.1	0	0	0	0	0	0	0	0	0	0	0	0	0
Solyc11g011120.1.1	13	39	0	21	0	70	97	67	5	80	86	47	2
Solyc11g011130.1.1	7	253	13	100	5	442	183	345	35	167	36	135	7
Solyc11g011140.1.1	1	30	0	18	0	23	1	44	1	24	4	18	0
Solyc11g011150.1.1	3	0	4	3	0	10	6	6	2	6	1	8	0
Solyc11g011160.1.1	1	11	0	6	2	2	0	10	0	5	3	5	0
Solyc11g011170.1.1	6	142	26	188	3	83	35	75	3	81	41	66	0
Solyc11g011180.1.1	0	0	0	0	0	0	0	0	0	0	0	0	0
Solyc11g011190.1.1	3	57	1	29	0	27	7	29	0	81	6	16	0
Solyc11g011200.1.1	0	0	0	0	0	0	0	0	0	0	0	0	0
Solyc11g011210.1.1	0	0	0	0	0	0	0	0	0	0	0	0	0
Solyc11g011220.1.1	6	25	2	18	0	55	18	67	6	57	42	84	2
Solyc11g011230.1.1	2	2	3	0	0	6	0	4	3	0	1	5	0

Solyc11g011240.1.1	0	0	0	0	0	0	0	0	0	0	0	0	0
Solyc11g011250.1.1	10	197	20	134	2	158	169	372	11	101	27	142	5
Solyc11g011260.1.1	13	289	15	164	4	102	46	176	10	252	42	284	4
Solyc11g011290.1.1	0	0	0	0	0	0	0	0	0	0	0	0	0
Solyc11g011300.1.1	1	0	0	0	0	1	0	12	0	12	8	0	0
Solyc11g011310.1.1	0	0	0	0	0	0	0	0	0	0	0	0	0
Solyc11g011330.1.1	0	0	0	0	0	0	0	0	0	0	0	0	0
Solyc11g011340.1.1	5	9	0	1	0	161	60	25	14	0	4	0	0
Solyc11g011350.1.1	0	0	0	0	0	0	0	0	0	0	0	0	0
Solyc11g011360.1.1	0	0	0	0	0	0	0	0	0	0	0	0	0
Solyc11g011370.1.1	0	0	0	0	0	0	0	0	0	0	0	0	0
Solyc11g011380.1.1	8	328	28	174	7	1301	176	1021	52	920	156	606	6
Solyc11g011390.1.1	3	10	0	0	0	5	5	14	3	20	3	14	3
Solyc11g011400.1.1	0	0	0	0	0	0	0	0	0	0	0	0	0
Solyc11g011410.1.1	2	7	0	7	2	18	9	16	0	25	1	25	0
Solyc11g011420.1.1	4	28	0	13	0	77	16	65	1	46	5	40	0
Solyc11g011440.1.1	13	146	20	124	58	982	905	518	237	70	18	62	0
Solyc11g011450.1.1	0	0	0	0	0	0	0	0	0	0	0	0	0
Solyc11g011460.1.1	0	0	0	0	0	0	0	0	0	0	0	0	0
Solyc11g011470.1.1	10	174	27	128	31	576	351	694	43	908	164	573	13
Solyc11g011480.1.1	8	23	9	3	11	58	39	59	5	37	25	25	0
Solyc11g011490.1.1	0	0	0	0	0	0	0	0	0	0	0	0	0
Solyc11g011500.1.1	0	0	0	0	0	0	0	0	0	0	0	0	0
Solyc11g011530.1.1	0	0	0	0	0	0	0	0	0	0	0	0	0
Solyc11g011540.1.1	1	3	2	1	0	5	6	4	0	1	0	5	0
Solyc11g011550.1.1	0	0	0	0	0	0	0	0	0	0	0	0	0
Solyc11g011570.1.1	0	0	0	0	0	0	0	0	0	0	0	0	0
Solyc11g011580.1.1	0	0	0	0	0	0	0	0	0	0	0	0	0
Solyc11g011590.1.1	0	0	0	0	0	0	0	0	0	0	0	0	0
Solyc11g011600.1.1	0	0	0	0	0	0	0	0	0	0	0	0	0
Solyc11g011610.1.1	0	0	0	0	0	0	0	0	0	0	0	0	0
Solyc11g011620.1.1	0	0	0	0	0	0	0	0	0	0	0	0	0
Solyc11g011760.1.1	5	69	14	23	0	198	523	243	67	112	78	228	8
Solyc11g011770.1.1	0	0	0	0	0	0	0	0	0	0	0	0	0
Solyc11g011780.1.1	10	117	9	96	12	226	89	265	33	138	53	74	1
Solyc11g011790.1.1	0	0	0	0	0	0	0	0	0	0	0	0	0
Solyc11g011800.1.1	0	0	0	0	0	0	0	0	0	0	0	0	0
Solyc11g011810.1.1	0	0	0	0	0	0	0	0	0	0	0	0	0
Solyc11g011840.1.1	1	21	9	6	0	17	88	29	0	8	12	6	0
Solyc11g011850.1.1	3	36	2	36	3	39	37	62	8	43	8	25	1
Solyc11g011860.1.1	1	0	0	0	0	4	16	5	4	4	5	0	0

Solyc11g011870.1.1	0	0	0	0	0	0	0	0	0	0	0	0	0
Solyc11g011880.1.1	0	0	0	0	0	0	0	0	0	0	0	0	0
Solyc11g011890.1.1	0	0	0	0	0	0	0	0	0	0	0	0	0
Solyc11g011910.1.1	9	43	1	37	4	168	63	173	19	183	31	225	4
Solyc11g011920.1.1	10	1474	208	898	81	1640	661	1489	236	840	198	1079	55
Solyc11g011930.1.1	12	78	4	48	8	370	385	278	40	313	89	187	1
Solyc11g011940.1.1	0	0	0	0	0	0	0	0	0	0	0	0	0
Solyc11g011960.1.1	11	394	11	104	9	360	110	557	24	1095	98	878	14
Solyc11g011970.1.1	4	37	6	25	0	65	11	25	4	65	8	42	4
Solyc11g011980.1.1	0	0	0	0	0	0	0	0	0	0	0	0	0
Solyc11g011990.1.1	16	157	8	57	3	498	422	601	22	287	46	212	3
Solyc11g012000.1.1	5	36	3	15	9	145	30	154	10	34	4	26	0
Solyc11g012020.1.1	0	0	0	0	0	0	0	0	0	0	0	0	0
Solyc11g012040.1.1	0	0	0	0	0	0	0	0	0	0	0	0	0
Solyc11g012050.1.1	0	0	0	0	0	0	0	0	0	0	0	0	0
Solyc11g012060.1.1	1	4	0	2	0	4	7	6	0	8	3	8	2
Solyc11g012070.1.1	3	12	0	1	0	39	18	28	4	9	1	15	0
Solyc11g012080.1.1	2	34	1	15	1	8	19	32	3	41	6	22	3
Solyc11g012090.1.1	0	0	0	0	0	0	0	0	0	0	0	0	0
Solyc11g012100.1.1	2	7	0	2	0	7	11	22	5	8	13	9	0
Solyc11g012110.1.1	5	330	35	156	13	660	212	752	68	432	102	416	3
Solyc11g012120.1.1	0	0	0	0	0	0	0	0	0	0	0	0	0
Solyc11g012130.1.1	5	324	6	177	9	145	123	233	7	21	274	16	3
Solyc11g012140.1.1	14	102	7	74	4	275	210	326	21	359	46	303	11
Solyc11g012150.1.1	5	29	3	27	1	58	36	92	15	48	5	55	0
Solyc11g012160.1.1	8	42	5	31	3	108	39	94	10	197	20	156	2
Solyc11g012170.1.1	0	0	0	0	0	0	0	0	0	0	0	0	0
Solyc11g012180.1.1	1	1	1	0	3	8	4	1	1	1	0	6	0
Solyc11g012190.1.1	0	0	0	0	0	0	0	0	0	0	0	0	0
Solyc11g012200.1.1	0	0	0	0	0	0	0	0	0	0	0	0	0
Solyc11g012210.1.1	0	0	0	0	0	0	0	0	0	0	0	0	0
Solyc11g012220.1.1	0	0	0	0	0	0	0	0	0	0	0	0	0
Solyc11g012230.1.1	0	0	0	0	0	0	0	0	0	0	0	0	0
Solyc11g012240.1.1	0	0	0	0	0	0	0	0	0	0	0	0	0
Solyc11g012250.1.1	0	0	0	0	0	0	0	0	0	0	0	0	0
Solyc11g012260.1.1	5	53	2	32	0	20	15	43	1	53	38	13	0
Solyc11g012270.1.1	1	9	0	2	0	5	11	7	0	10	0	10	0
Solyc11g012280.1.1	1	8	0	4	0	12	2	16	1	21	3	25	0
Solyc11g012290.1.1	4	6	0	0	0	35	21	43	2	15	1	22	1
Solyc11g012300.1.1	0	0	0	0	0	0	0	0	0	0	0	0	0
Solyc11g012310.1.1	0	0	0	0	0	0	0	0	0	0	0	0	0

Solyc11g012320.1.1	13	7175	97	2459	88	20286	7852	17418	475	56119	3915	43998	175
Solyc11g012360.1.1	21	487	157	313	5	1121	263	1234	7	31	248	29	0
Solyc11g012390.1.1	1	11	1	19	5	7	8	15	3	7	0	11	2
Solyc11g012400.1.1	6	80	12	36	0	164	92	203	9	152	28	109	6
Solyc11g012410.1.1	8	126	16	108	4	205	145	209	14	131	57	173	1
Solyc11g012420.1.1	0	0	0	0	0	0	0	0	0	0	0	0	0
Solyc11g012440.1.1	14	260	12	175	3	388	88	376	10	986	70	467	6
Solyc11g012450.1.1	15	79	6	39	6	107	182	110	17	163	30	149	2
Solyc11g012460.1.1	3	17	0	22	0	23	12	16	2	43	12	25	6
Solyc11g012470.1.1	3	75	10	54	1	52	57	81	11	84	15	60	0
Solyc11g012480.1.1	9	27	2	5	0	70	48	94	4	55	10	38	3
Solyc11g012510.1.1	16	294	50	166	9	225	93	251	19	255	47	239	16
Solyc11g012520.1.1	0	0	0	0	0	0	0	0	0	0	0	0	0
Solyc11g012540.1.1	2	0	0	0	0	4	0	7	0	3	4	5	0
Solyc11g012550.1.1	2	5	0	0	0	17	11	10	0	18	0	5	0
Solyc11g012560.1.1	2	7	0	1	1	3	11	1	2	6	0	7	0
Solyc11g012570.1.1	0	0	0	0	0	0	0	0	0	0	0	0	0
Solyc11g012580.1.1	0	0	0	0	0	0	0	0	0	0	0	0	0
Solyc11g012590.1.1	0	0	0	0	0	0	0	0	0	0	0	0	0
Solyc11g012610.1.1	0	0	0	0	0	0	0	0	0	0	0	0	0
Solyc11g012620.1.1	1	3	0	1	0	3	6	4	0	20	0	6	0
Solyc11g012640.1.1	2	45	2	30	0	6	7	18	0	1	0	3	0
Solyc11g012660.1.1	1	0	0	1	0	9	5	16	0	2	0	0	0
Solyc11g012670.1.1	4	17	0	5	0	40	32	64	4	36	7	22	0
Solyc11g012680.1.1	9	183	10	176	4	147	95	161	21	47	4	13	0
Solyc11g012690.1.1	0	0	0	0	0	0	0	0	0	0	0	0	0
Solyc11g012700.1.1	20	736	26	539	0	926	163	977	8	589	72	450	1
Solyc11g012710.1.1	3	12	0	3	0	7	10	12	6	22	10	23	0
Solyc11g012720.1.1	1	2	0	0	0	2	4	4	0	4	2	4	0
Solyc11g012730.1.1	2	64	1	17	6	79	63	44	10	90	14	63	1
Solyc11g012740.1.1	3	50	3	42	1	71	43	109	3	34	15	25	1
Solyc11g012750.1.1	0	0	0	0	0	0	0	0	0	0	0	0	0
Solyc11g012760.1.1	2	11	1	7	0	57	7	24	1	57	3	80	0
Solyc11g012770.1.1	33	193	30	187	20	377	355	551	62	329	95	248	5
Solyc11g012780.1.1	1	2	0	0	0	13	5	4	0	2	3	4	0
Solyc11g012790.1.1	6	61	13	48	14	89	46	64	13	115	18	38	1
Solyc11g012800.1.1	0	0	0	0	0	0	0	0	0	0	0	0	0
Solyc11g012810.1.1	2	4	0	8	0	18	8	15	2	38	5	6	0
Solyc11g012820.1.1	8	27	8	18	7	10	62	43	5	33	28	63	10
Solyc11g012830.1.1	7	43	17	29	14	58	78	125	12	89	15	84	1
Solyc11g012840.1.1	2	27	5	3	0	16	32	35	4	52	4	26	0

Solyc11g012850.1.1	9	194	29	149	5	96	27	123	6	43	38	45	2
Solyc11g012860.1.1	4	43	7	22	0	26	40	28	0	184	11	195	1
Solyc11g012870.1.1	3	33	1	14	0	22	7	61	1	59	32	58	3
Solyc11g012880.1.1	0	0	0	0	0	0	0	0	0	0	0	0	0
Solyc11g012890.1.1	2	37	4	32	1	152	22	119	4	47	7	28	0
Solyc11g012900.1.1	12	112	16	59	1	426	270	345	36	217	90	134	2
Solyc11g012910.1.1	4	27	3	14	0	25	33	36	7	137	19	114	0
Solyc11g012920.1.1	4	17	0	5	1	9	17	13	6	19	6	13	0
Solyc11g012930.1.1	6	38	5	39	0	291	366	232	0	98	30	24	0
Solyc11g012940.1.1	3	25	6	13	0	21	36	29	1	21	9	27	3
Solyc11g012950.1.1	0	0	0	0	0	0	0	0	0	0	0	0	0
Solyc11g012960.1.1	0	0	0	0	0	0	0	0	0	0	0	0	0
Solyc11g012970.1.1	7	46	5	51	1	134	32	181	8	177	23	70	2
Solyc11g012980.1.1	1	19	2	17	0	0	1	0	0	5	0	3	0
Solyc11g013000.1.1	7	40	6	17	0	134	60	91	16	41	40	58	0
Solyc11g013010.1.1	6	17	4	13	3	53	59	45	47	20	7	26	0
Solyc11g013020.1.1	2	39	0	23	2	5	41	31	1	33	8	57	2
Solyc11g013030.1.1	7	51	2	17	16	122	75	110	19	75	14	33	0
Solyc11g013060.1.1	0	0	0	0	0	0	0	0	0	0	0	0	0
Solyc11g013070.1.1	0	0	0	0	0	0	0	0	0	0	0	0	0
Solyc11g013080.1.1	0	0	0	0	0	0	0	0	0	0	0	0	0
Solyc11g013090.1.1	0	0	0	0	0	0	0	0	0	0	0	0	0
Solyc11g013100.1.1	1	35	0	30	0	5	30	29	7	31	42	14	0
Solyc11g013110.1.1	16	354	63	131	307	364	279	258	147	2583	133	1963	20
Solyc11g013120.1.1	11	176	25	143	14	247	200	259	17	298	70	310	4
Solyc11g013130.1.1	2	40	0	21	0	8	3	43	3	129	5	84	0
Solyc11g013150.1.1	0	0	0	0	0	0	0	0	0	0	0	0	0
Solyc11g013160.1.1	0	0	0	0	0	0	0	0	0	0	0	0	0
Solyc11g013170.1.1	16	138	13	101	5	585	225	795	51	300	53	199	2
Solyc11g013180.1.1	10	102	18	72	14	182	100	139	58	102	18	71	3
Solyc11g013190.1.1	18	143	14	73	5	300	200	261	47	142	98	147	3
Solyc11g013200.1.1	5	11	1	1	0	23	15	30	7	17	5	7	1
Solyc11g013240.1.1	0	0	0	0	0	0	0	0	0	0	0	0	0
Solyc11g013250.1.1	0	0	0	0	0	0	0	0	0	0	0	0	0
Solyc11g013260.1.1	2	11	0	30	0	36	14	69	3	29	2	87	0
Solyc11g013270.1.1	1	6	0	0	0	0	0	0	0	11	6	10	0
Solyc11g013280.1.1	13	60	5	49	6	178	95	236	7	255	27	237	5
Solyc11g013290.1.1	5	5	1	8	4	17	30	32	4	43	3	23	0
Solyc11g013300.1.1	0	0	0	0	0	0	0	0	0	0	0	0	0
Solyc11g013310.1.1	1	4	4	3	0	1	3	17	1	15	0	11	0
Solyc11g013330.1.1	0	0	0	0	0	0	0	0	0	0	0	0	0

Solyc11g013340.1.1	0	0	0	0	0	0	0	0	0	0	0	0	0
Solyc11g013350.1.1	1	2	0	0	0	5	4	7	1	2	0	0	0
Solyc11g013370.1.1	20	87	16	70	11	241	172	282	51	206	59	151	8
Solyc11g013390.1.1	0	0	0	0	0	0	0	0	0	0	0	0	0
Solyc11g013400.1.1	1	15	0	1	0	5	19	9	0	1	13	9	0
Solyc11g013440.1.1	4	26	4	23	0	34	28	120	16	52	10	44	0
Solyc11g013450.1.1	0	0	0	0	0	0	0	0	0	0	0	0	0
Solyc11g013460.1.1	5	21	1	20	0	26	24	34	8	38	7	38	9
Solyc11g013470.1.1	1	1	0	8	0	22	5	5	0	0	1	1	0
Solyc11g013480.1.1	2	1	0	3	0	136	20	48	2	9	4	11	2
Solyc11g013490.1.1	4	28	1	14	2	66	25	36	4	26	2	22	0
Solyc11g013500.1.1	0	0	0	0	0	0	0	0	0	0	0	0	0
Solyc11g013520.1.1	6	49	6	66	0	120	57	105	12	92	18	60	1
Solyc11g013530.1.1	25	61	11	79	17	215	126	203	26	87	34	182	0
Solyc11g013550.1.1	1	9	3	2	0	5	11	7	0	17	2	6	0
Solyc11g013750.1.1	6	23	3	27	2	26	41	56	0	59	10	44	0
Solyc11g013760.1.1	24	172	39	135	16	424	183	315	34	236	81	173	0
Solyc11g013770.1.1	0	0	0	0	0	0	0	0	0	0	0	0	0
Solyc11g013810.1.1	19	1046	144	532	3	344	15	146	0	69	46	364	0
Solyc11g013820.1.1	2	6	0	0	1	1	5	12	0	4	9	79	0
Solyc11g013830.1.1	17	57	12	43	7	59	83	66	13	123	57	144	8
Solyc11g013840.1.1	3	19	3	12	3	48	20	32	2	26	7	40	0
Solyc11g013850.1.1	1	2	1	3	0	3	0	8	0	1	2	0	0
Solyc11g013880.1.1	10	86	10	38	7	76	36	75	6	68	11	60	0
Solyc11g013890.1.1	0	0	0	0	0	0	0	0	0	0	0	0	0
Solyc11g015900.1.1	0	0	0	0	0	0	0	0	0	0	0	0	0
Solyc11g015910.1.1	0	0	0	0	0	0	0	0	0	0	0	0	0
Solyc11g016930.1.1	0	0	0	0	0	0	0	0	0	0	0	0	0
Solyc11g016940.1.1	2	9	3	4	1	55	18	30	11	38	7	11	0
Solyc11g016970.1.1	0	0	0	0	0	0	0	0	0	0	0	0	0
Solyc11g016990.1.1	2	2	1	0	0	6	47	7	0	7	13	8	6
Solyc11g017000.1.1	3	6	0	0	0	22	7	35	0	6	2	4	0
Solyc11g017010.1.1	13	861	38	547	10	495	457	877	262	239	45	186	0
Solyc11g017040.1.1	17	47	8	33	4	125	130	153	20	96	20	58	1
Solyc11g017050.1.1	0	0	0	0	0	0	0	0	0	0	0	0	0
Solyc11g017060.1.1	4	25	1	10	0	43	21	83	3	67	13	20	0
Solyc11g017070.1.1	4	88	1	18	0	125	52	200	13	93	44	136	0
Solyc11g017130.1.1	0	0	0	0	0	0	0	0	0	0	0	0	0
Solyc11g017140.1.1	7	35	4	23	2	28	39	26	3	36	26	36	1
Solyc11g017170.1.1	9	19	1	5	0	36	49	67	17	35	20	16	1
Solyc11g017180.1.1	0	0	0	0	0	0	0	0	0	0	0	0	0

Solyc11g017190.1.1	3	2	0	2	0	14	6	15	8	19	3	11	0
Solyc11g017200.1.1	6	33	2	18	0	12	48	66	5	44	12	11	0
Solyc11g017220.1.1	0	0	0	0	0	0	0	0	0	0	0	0	0
Solyc11g017230.1.1	2	3	0	0	0	3	13	7	0	3	2	4	0
Solyc11g017240.1.1	9	86	5	54	0	183	105	214	15	52	5	50	0
Solyc11g017250.1.1	10	49	4	18	6	49	77	107	12	32	24	63	2
Solyc11g017270.1.1	5	90	189	65	84	80	361	42	40	42	190	57	12
Solyc11g017280.1.1	0	0	0	0	0	0	0	0	0	0	0	0	0
Solyc11g017290.1.1	0	0	0	0	0	0	0	0	0	0	0	0	0
Solyc11g017300.1.1	5	54	3	44	2	124	57	147	10	159	19	107	6
Solyc11g017360.1.1	0	0	0	0	0	0	0	0	0	0	0	0	0
Solyc11g017370.1.1	0	0	0	0	0	0	0	0	0	0	0	0	0
Solyc11g017380.1.1	0	0	0	0	0	0	0	0	0	0	0	0	0
Solyc11g017390.1.1	4	0	0	0	0	30	39	16	15	0	0	0	0
Solyc11g017400.1.1	0	0	0	0	0	0	0	0	0	0	0	0	0
Solyc11g017410.1.1	1	5	0	0	0	3	16	7	0	1	4	1	0
Solyc11g017420.1.1	4	15	4	6	0	58	12	32	10	35	14	24	3
Solyc11g017430.1.1	6	53	13	12	0	42	50	49	1	12	4	7	0
Solyc11g017440.1.1	0	0	0	0	0	0	0	0	0	0	0	0	0
Solyc11g017460.1.1	3	14	1	12	0	10	9	16	2	30	14	15	3
Solyc11g017470.1.1	9	171	10	56	6	364	37	157	31	195	66	83	11
Solyc11g018490.1.1	0	0	0	0	0	0	0	0	0	0	0	0	0
Solyc11g018500.1.1	0	0	0	0	0	0	0	0	0	0	0	0	0
Solyc11g018510.1.1	0	0	0	0	0	0	0	0	0	0	0	0	0
Solyc11g018520.1.1	0	0	0	0	0	0	0	0	0	0	0	0	0
Solyc11g018550.2.1	4	113	8	49	0	52	37	59	1	96	2	102	1
Solyc11g018560.1.1	0	0	0	0	0	0	0	0	0	0	0	0	0
Solyc11g018580.1.1	14	244	17	124	13	534	154	524	31	287	193	231	17
Solyc11g018590.1.1	0	0	0	0	0	0	0	0	0	0	0	0	0
Solyc11g018610.1.1	4	22	2	20	0	99	21	50	2	16	0	7	0
Solyc11g018620.1.1	1	1	3	4	0	4	6	7	1	13	0	0	0
Solyc11g018660.1.1	0	0	0	0	0	0	0	0	0	0	0	0	0
Solyc11g018670.1.1	13	88	7	39	2	97	148	134	16	149	54	110	7
Solyc11g018680.1.1	3	22	2	42	0	392	17	150	0	48	5	51	0
Solyc11g018690.1.1	3	2	2	1	0	14	17	23	0	11	0	1	0
Solyc11g018710.1.1	0	0	0	0	0	0	0	0	0	0	0	0	0
Solyc11g018720.1.1	1	1	0	0	0	3	2	13	0	2	1	9	0
Solyc11g018740.1.1	1	1	0	0	0	4	6	7	0	3	3	1	0
Solyc11g018770.1.1	0	0	0	0	0	0	0	0	0	0	0	0	0
Solyc11g018810.1.1	0	0	0	0	0	0	0	0	0	0	0	0	0
Solyc11g018820.1.1	0	0	0	0	0	0	0	0	0	0	0	0	0

Solyc11g019910.1.1	3	33	1	23	0	0	67	0	0	0	0	3	4
Solyc11g019920.1.1	4	25	4	15	4	172	104	99	7	26	18	27	0
Solyc11g019930.1.1	4	16	0	5	0	13	13	26	1	26	10	11	0
Solyc11g019950.1.1	8	46	0	8	3	118	88	147	10	114	22	102	1
Solyc11g019960.1.1	1	0	0	0	0	4	8	7	1	8	5	1	0
Solyc11g019970.1.1	2	3	0	0	0	1	4	16	1	1	0	8	0
Solyc11g020020.1.1	0	0	0	0	0	0	0	0	0	0	0	0	0
Solyc11g020040.1.1	29	25	29	59	6	305	277	404	37	335	220	354	2
Solyc11g020050.1.1	0	0	0	0	0	0	0	0	0	0	0	0	0
Solyc11g020060.1.1	1	1	0	0	0	0	5	0	2	4	4	2	0
Solyc11g020100.1.1	0	0	0	0	0	0	0	0	0	0	0	0	0
Solyc11g020180.1.1	0	0	0	0	0	0	0	0	0	0	0	0	0
Solyc11g020190.1.1	0	0	0	0	0	0	0	0	0	0	0	0	0
Solyc11g020210.1.1	0	0	0	0	0	0	0	0	0	0	0	0	0
Solyc11g020230.1.1	1	14	4	7	0	0	0	0	2	0	0	1	0
Solyc11g020280.1.1	22	344	59	314	14	900	385	803	34	666	190	343	7
Solyc11g020290.1.1	0	0	0	0	0	0	0	0	0	0	0	0	0
Solyc11g020300.1.1	11	134	25	137	6	468	226	621	12	287	65	306	6
Solyc11g020330.1.1	0	0	0	0	0	0	0	0	0	0	0	0	0
Solyc11g020590.1.1	7	53	6	47	0	80	91	69	21	49	27	37	8
Solyc11g020610.1.1	2	10	1	19	0	11	7	15	0	29	1	18	0
Solyc11g020670.1.1	15	158	43	66	33	113	572	307	95	1171	19	268	12
Solyc11g020710.1.1	0	0	0	0	0	0	0	0	0	0	0	0	0
Solyc11g020720.1.1	3	3	3	4	0	1	11	7	0	35	2	20	0
Solyc11g020730.1.1	1	8	2	7	0	14	3	10	2	30	6	13	0
Solyc11g020750.1.1	0	0	0	0	0	0	0	0	0	0	0	0	0
Solyc11g020760.1.1	1	1	0	0	1	0	2	1	0	3	4	7	0
Solyc11g020770.1.1	0	0	0	0	0	0	0	0	0	0	0	0	0
Solyc11g020780.1.1	4	29	3	15	2	41	29	84	7	30	8	14	0
Solyc11g020810.1.1	2	31	1	15	0	47	15	60	4	39	3	24	0
Solyc11g020820.1.1	0	0	0	0	0	0	0	0	0	0	0	0	0
Solyc11g020870.1.1	9	36	1	9	4	70	86	195	22	160	45	85	1
Solyc11g020890.1.1	1	6	0	5	1	7	4	14	4	11	3	25	0
Solyc11g020910.1.1	2	5	0	0	0	4	5	5	0	0	4	3	0
Solyc11g020920.1.1	0	0	0	0	0	0	0	0	0	0	0	0	0
Solyc11g020960.1.1	0	0	0	0	0	0	0	0	0	0	0	0	0
Solyc11g020980.1.1	0	0	0	0	0	0	0	0	0	0	0	0	0
Solyc11g020990.1.1	0	0	0	0	0	0	0	0	0	0	0	0	0
Solyc11g021020.1.1	0	0	0	0	0	0	0	0	0	0	0	0	0
Solyc11g021060.1.1	0	0	0	0	0	0	0	0	0	0	0	0	0
Solyc11g021150.1.1	0	0	0	0	0	0	0	0	0	0	0	0	0

Solyc11g021170.1.1	0	0	0	0	0	0	0	0	0	0	0	0	0
Solyc11g022380.1.1	7	18	5	22	1	63	63	82	15	50	29	53	0
Solyc11g022390.1.1	8	27	4	7	0	104	95	83	13	78	27	66	3
Solyc11g022400.1.1	6	14	0	19	5	31	37	29	0	43	5	52	1
Solyc11g022450.1.1	0	0	0	0	0	0	0	0	0	0	0	0	0
Solyc11g022460.1.1	4	37	6	26	2	35	76	31	3	343	31	157	2
Solyc11g022470.1.1	5	70	8	18	12	66	48	84	10	192	26	135	0
Solyc11g022530.1.1	0	0	0	0	0	0	0	0	0	0	0	0	0
Solyc11g022540.1.1	2	16	4	11	3	73	43	34	4	76	25	45	6
Solyc11g022590.1.1	0	0	0	0	0	0	0	0	0	0	0	0	0
Solyc11g022600.1.1	0	0	0	0	0	0	0	0	0	0	0	0	0
Solyc11g027630.1.1	0	0	0	0	0	0	0	0	0	0	0	0	0
Solyc11g027660.1.1	1	0	2	4	9	3	9	0	2	8	65	0	3
Solyc11g027670.1.1	0	0	0	0	0	0	0	0	0	0	0	0	0
Solyc11g027690.1.1	0	0	0	0	0	0	0	0	0	0	0	0	0
Solyc11g027710.1.1	0	0	0	0	0	0	0	0	0	0	0	0	0
Solyc11g027730.1.1	0	0	0	0	0	0	0	0	0	0	0	0	0
Solyc11g027750.1.1	0	0	0	0	0	0	0	0	0	0	0	0	0
Solyc11g027760.1.1	0	0	0	0	0	0	0	0	0	0	0	0	0
Solyc11g027770.1.1	0	0	0	0	0	0	0	0	0	0	0	0	0
Solyc11g027790.1.1	0	0	0	0	0	0	0	0	0	0	0	0	0
Solyc11g027810.1.1	0	0	0	0	0	0	0	0	0	0	0	0	0
Solyc11g027820.1.1	0	0	0	0	0	0	0	0	0	0	0	0	0
Solyc11g027830.1.1	1	7	1	3	0	6	2	6	0	10	0	10	0
Solyc11g027840.1.1	1	4	1	2	0	1	15	10	0	0	6	0	2
Solyc11g027870.1.1	0	0	0	0	0	0	0	0	0	0	0	0	0
Solyc11g027880.1.1	11	115	15	78	5	76	96	89	23	206	46	158	1
Solyc11g028000.1.1	4	16	1	0	0	8	5	14	0	5	3	20	0
Solyc11g028010.1.1	3	29	2	21	1	74	34	92	5	43	7	74	1
Solyc11g028020.1.1	0	0	0	0	0	0	0	0	0	0	0	0	0
Solyc11g028080.1.1	2	11	0	16	0	35	40	44	6	14	4	11	0
Solyc11g028090.1.1	0	0	0	0	0	0	0	0	0	0	0	0	0
Solyc11g028100.1.1	6	145	27	47	6	142	57	193	12	586	18	241	2
Solyc11g028170.1.1	0	0	0	0	0	0	0	0	0	0	0	0	0
Solyc11g030380.1.1	0	0	0	0	0	0	0	0	0	0	0	0	0
Solyc11g030390.1.1	1	9	0	13	0	13	6	16	1	16	7	22	0
Solyc11g030550.1.1	1	9	0	9	2	9	1	5	0	5	1	10	0
Solyc11g030570.1.1	0	0	0	0	0	0	0	0	0	0	0	0	0
Solyc11g030600.2.1	3	53	4	19	0	118	45	118	2	106	9	59	0
Solyc11g030700.1.1	3	33	5	15	6	40	29	41	26	40	15	46	8
Solyc11g030710.1.1	6	58	11	24	2	55	77	70	6	73	14	45	0

Solyc11g030720.1.1	3	9	1	15	4	38	13	31	5	15	7	19	0
Solyc11g030730.1.1	0	0	0	0	0	0	0	0	0	0	0	0	0
Solyc11g030910.1.1	0	0	0	0	0	0	0	0	0	0	0	0	0
Solyc11g031950.1.1	1	2	0	8	0	2	1	6	0	0	3	0	0
Solyc11g031960.1.1	0	0	0	0	0	0	0	0	0	0	0	0	0
Solyc11g031970.1.1	6	56	4	34	2	9	22	17	2	7	0	5	0
Solyc11g032040.1.1	0	0	0	0	0	0	0	0	0	0	0	0	0
Solyc11g032050.1.1	0	0	0	0	0	0	0	0	0	0	0	0	0
Solyc11g032090.1.1	0	0	0	0	0	0	0	0	0	0	0	0	0
Solyc11g032100.1.1	1	3	0	0	0	0	6	3	0	3	0	3	0
Solyc11g032130.1.1	2	2	1	5	0	28	26	9	1	28	4	12	0
Solyc11g032190.1.1	0	0	0	0	0	0	0	0	0	0	0	0	0
Solyc11g032200.1.1	5	49	7	48	1	49	111	83	12	46	7	45	0
Solyc11g032220.1.1	0	0	0	0	0	0	0	0	0	0	0	0	0
Solyc11g033260.1.1	2	5	0	4	0	30	11	78	1	44	2	30	0
Solyc11g033270.1.1	13	80	13	56	4	126	112	191	20	287	54	180	10
Solyc11g033280.1.1	7	93	8	62	9	268	96	394	28	266	56	180	2
Solyc11g039650.1.1	13	144	31	111	24	402	317	745	120	335	102	257	4
Solyc11g039740.1.1	3	38	3	41	0	15	21	18	4	37	11	37	0
Solyc11g039830.1.1	8	56	3	35	1	165	55	252	8	100	18	82	3
Solyc11g039840.1.1	4	485	17	286	19	731	204	989	69	1607	115	1606	9
Solyc11g039870.1.1	13	29	4	23	2	128	93	162	14	88	17	61	1
Solyc11g039880.1.1	1	2	0	0	0	32	6	28	0	8	1	3	0
Solyc11g039890.1.1	1	0	0	0	0	2	6	8	0	2	0	0	0
Solyc11g039910.1.1	5	91	5	74	11	212	122	169	21	370	75	414	12
Solyc11g039950.1.1	2	36	1	2	0	16	4	52	3	19	4	10	3
Solyc11g039960.1.1	0	0	0	0	0	0	0	0	0	0	0	0	0
Solyc11g039980.1.1	0	0	0	0	0	0	0	0	0	0	0	0	0
Solyc11g039990.1.1	0	0	0	0	0	0	0	0	0	0	0	0	0
Solyc11g040040.1.1	0	0	0	0	0	0	0	0	0	0	0	0	0
Solyc11g040050.1.1	3	20	0	7	1	38	23	34	0	26	16	14	0
Solyc11g040060.1.1	3	0	1	0	0	0	7	0	3	0	0	0	0
Solyc11g040110.1.1	2	10	0	8	0	44	10	34	1	33	4	33	0
Solyc11g040120.1.1	5	31	3	3	5	18	2	22	2	62	15	69	0
Solyc11g040130.1.1	3	34	0	24	1	145	18	90	13	82	11	33	1
Solyc11g040140.1.1	0	0	0	0	0	0	0	0	0	0	0	0	0
Solyc11g040150.1.1	9	84	3	42	0	40	80	96	3	74	10	40	8
Solyc11g040170.1.1	0	0	0	0	0	0	0	0	0	0	0	0	0
Solyc11g040180.1.1	4	22	2	0	2	43	40	29	7	46	9	37	1
Solyc11g040220.1.1	0	0	0	0	0	0	0	0	0	0	0	0	0
Solyc11g040240.1.1	2	5	0	3	0	18	14	20	1	6	0	1	4

Solyc11g040250.1.1	0	0	0	0	0	0	0	0	0	0	0	0	0
Solyc11g040260.1.1	2	7	0	0	0	6	11	18	0	14	1	26	0
Solyc11g040330.1.1	2	1	2	8	1	30	6	86	0	2	1	1	0
Solyc11g040340.1.1	0	0	0	0	0	0	0	0	0	0	0	0	0
Solyc11g040370.1.1	20	218	24	160	11	808	297	712	49	447	158	341	4
Solyc11g040380.1.1	0	0	0	0	0	0	0	0	0	0	0	0	0
Solyc11g040390.1.1	8	22	4	27	1	71	73	96	6	53	21	65	4
Solyc11g042430.1.1	8	48	6	42	1	77	28	77	0	73	7	84	1
Solyc11g042440.1.1	4	21	2	7	1	6	16	8	2	35	6	33	0
Solyc11g042460.1.1	3	5	4	3	0	9	11	11	11	4	4	10	2
Solyc11g042470.1.1	2	4	3	1	3	6	8	8	2	19	1	4	0
Solyc11g042480.1.1	0	0	0	0	0	0	0	0	0	0	0	0	0
Solyc11g042490.1.1	0	0	0	0	0	0	0	0	0	0	0	0	0
Solyc11g042500.1.1	1	5	3	0	0	9	17	10	1	10	0	3	0
Solyc11g042510.1.1	0	0	0	0	0	0	0	0	0	0	0	0	0
Solyc11g042560.1.1	0	0	0	0	0	0	0	0	0	0	0	0	0
Solyc11g042610.1.1	3	59	1	24	0	123	28	124	1	124	27	54	0
Solyc11g042620.1.1	4	83	12	31	2	30	93	32	4	49	50	33	4
Solyc11g042630.1.1	0	0	0	0	0	0	0	0	0	0	0	0	0
Solyc11g042640.1.1	2	59	6	25	1	13	28	13	1	24	14	22	0
Solyc11g042800.1.1	0	0	0	0	0	0	0	0	0	0	0	0	0
Solyc11g042820.1.1	0	0	0	0	0	0	0	0	0	0	0	0	0
Solyc11g042930.1.1	0	0	0	0	0	0	0	0	0	0	0	0	0
Solyc11g042940.1.1	5	44	12	18	0	11	32	25	0	13	0	23	0
Solyc11g042950.1.1	1	14	2	20	0	19	16	7	0	6	0	13	0
Solyc11g042990.1.1	14	81	12	60	7	159	129	177	35	203	67	141	0
Solyc11g043120.1.1	8	29	7	30	8	60	151	95	24	137	37	101	4
Solyc11g043130.1.1	15	48	3	27	11	73	116	189	19	233	19	88	0
Solyc11g043150.1.1	1	0	0	0	0	0	5	12	0	6	0	3	0
Solyc11g043170.1.1	1	5	0	2	0	22	2	20	0	10	1	3	0
Solyc11g043190.1.1	0	0	0	0	0	0	0	0	0	0	0	0	0
Solyc11g043200.1.1	3	14	0	7	1	31	9	45	5	8	15	13	0
Solyc11g043210.1.1	10	170	27	187	7	685	320	529	55	445	112	427	12
Solyc11g044230.1.1	0	0	0	0	0	0	0	0	0	0	0	0	0
Solyc11g044240.1.1	2	40	3	10	0	3	2	3	0	4	2	11	0
Solyc11g044250.1.1	2	61	2	22	1	4	7	8	0	20	3	11	0
Solyc11g044260.1.1	3	32	6	17	0	19	5	16	3	5	4	7	0
Solyc11g044270.1.1	4	10	4	9	3	19	49	39	8	39	12	49	0
Solyc11g044280.1.1	3	25	6	7	2	33	80	76	4	37	13	28	1
Solyc11g044310.1.1	5	16	2	40	0	182	42	99	8	94	8	51	0
Solyc11g044320.1.1	2	8	0	4	0	26	7	36	0	7	8	18	0

Solyc11g044330.1.1	0	0	0	0	0	0	0	0	0	0	0	0	0
Solyc11g044340.1.1	9	62	8	72	2	316	45	140	5	82	16	60	0
Solyc11g044350.1.1	1	0	0	5	0	2	5	0	0	6	2	0	0
Solyc11g044360.1.1	3	46	8	26	0	11	61	40	2	32	3	34	0
Solyc11g044450.1.1	0	0	0	0	0	0	0	0	0	0	0	0	0
Solyc11g044460.1.1	0	0	0	0	0	0	0	0	0	0	0	0	0
Solyc11g044470.1.1	2	25	6	6	1	22	23	36	11	16	7	8	0
Solyc11g044480.1.1	12	67	9	44	0	167	113	192	8	122	18	136	2
Solyc11g044510.1.1	4	15	0	2	0	10	19	48	1	26	0	33	0
Solyc11g044530.1.1	4	42	5	25	0	9	27	25	2	3	2	13	1
Solyc11g044540.1.1	12	162	50	102	12	366	547	355	49	310	87	200	8
Solyc11g044560.1.1	4	26	3	25	4	59	24	61	3	65	14	105	1
Solyc11g044610.1.1	0	0	0	0	0	0	0	0	0	0	0	0	0
Solyc11g044620.1.1	0	0	0	0	0	0	0	0	0	0	0	0	0
Solyc11g044630.1.1	0	0	0	0	0	0	0	0	0	0	0	0	0
Solyc11g044640.1.1	0	0	0	0	0	0	0	0	0	0	0	0	0
Solyc11g044740.1.1	0	0	0	0	0	0	0	0	0	0	0	0	0
Solyc11g044750.1.1	3	6	0	10	1	22	15	27	3	4	4	6	0
Solyc11g044800.1.1	5	25	0	11	0	110	19	40	5	64	15	47	0
Solyc11g044840.1.1	0	0	0	0	0	0	0	0	0	0	0	0	0
Solyc11g044860.1.1	0	0	0	0	0	0	0	0	0	0	0	0	0
Solyc11g044870.1.1	7	31	4	10	4	41	54	98	3	44	22	59	0
Solyc11g044880.1.1	6	46	1	18	4	18	34	47	7	50	10	44	0
Solyc11g044900.1.1	18	107	9	38	14	129	86	169	22	68	18	91	0
Solyc11g044910.1.1	24	49	1	28	10	194	2535	676	32	10	64	6	3
Solyc11g044940.1.1	1	3	0	3	0	0	5	1	1	2	0	0	0
Solyc11g044950.1.1	0	0	0	0	0	0	0	0	0	0	0	0	0
Solyc11g044960.1.1	1	3	1	1	0	3	32	1	0	9	8	8	0
Solyc11g044970.1.1	1	0	1	1	0	1	5	5	0	4	0	7	0
Solyc11g044980.1.1	0	0	0	0	0	0	0	0	0	0	0	0	0
Solyc11g044990.1.1	0	0	0	0	0	0	0	0	0	0	0	0	0
Solyc11g045000.1.1	1	4	0	0	0	3	4	5	0	2	0	5	1
Solyc11g045010.1.1	0	0	0	0	0	0	0	0	0	0	0	0	0
Solyc11g045020.1.1	0	0	0	0	0	0	0	0	0	0	0	0	0
Solyc11g045030.1.1	0	0	0	0	0	0	0	0	0	0	0	0	0
Solyc11g045040.1.1	0	0	0	0	0	0	0	0	0	0	0	0	0
Solyc11g045050.1.1	1	2	0	0	0	5	8	6	0	1	2	0	0
Solyc11g045100.1.1	7	24	8	11	1	36	55	23	5	67	36	88	5
Solyc11g045110.1.1	6	22	3	32	0	25	14	45	0	8	6	9	0
Solyc11g045120.1.1	13	48	13	28	9	63	80	83	17	108	32	93	0
Solyc11g045130.1.1	8	219	53	169	42	382	309	308	30	186	60	269	11

Solyc11g045140.1.1	0	0	0	0	0	0	0	0	0	0	0	0	0
Solyc11g045150.1.1	0	0	0	0	0	0	0	0	0	0	0	0	0
Solyc11g045160.1.1	9	9	4	1	0	21	50	63	7	19	13	10	2
Solyc11g045170.1.1	6	32	4	19	0	68	54	71	4	44	15	41	0
Solyc11g045180.1.1	4	24	9	19	3	69	75	76	0	10	14	19	0
Solyc11g045190.1.1	9	20	6	15	3	58	93	68	4	37	1	39	0
Solyc11g045210.1.1	4	4	0	8	0	5	13	15	1	23	2	2	0
Solyc11g045220.1.1	1	1	0	2	0	0	6	5	0	6	0	9	1
Solyc11g045230.1.1	0	0	0	0	0	0	0	0	0	0	0	0	0
Solyc11g045240.1.1	1	7	1	3	0	4	1	19	0	30	8	29	0
Solyc11g045250.1.1	1	4	1	3	0	0	2	2	0	7	2	13	0
Solyc11g045260.1.1	0	0	0	0	0	0	0	0	0	0	0	0	0
Solyc11g045270.1.1	3	12	0	7	0	28	14	15	3	19	6	9	0
Solyc11g045280.1.1	0	0	0	0	0	0	0	0	0	0	0	0	0
Solyc11g045290.1.1	0	0	0	0	0	0	0	0	0	0	0	0	0
Solyc11g045310.1.1	2	9	0	2	0	1	14	6	0	2	3	2	0
Solyc11g045330.1.1	6	7	2	17	0	25	41	44	1	56	11	22	0
Solyc11g045350.1.1	18	134	10	125	6	269	88	369	25	449	47	254	1
Solyc11g045370.1.1	0	0	0	0	0	0	0	0	0	0	0	0	0
Solyc11g045380.1.1	1	7	0	3	0	7	0	9	0	2	2	2	0
Solyc11g045390.1.1	0	0	0	0	0	0	0	0	0	0	0	0	0
Solyc11g045410.1.1	0	0	0	0	0	0	0	0	0	0	0	0	0
Solyc11g045430.1.1	0	0	0	0	0	0	0	0	0	0	0	0	0
Solyc11g045440.1.1	0	0	0	0	0	0	0	0	0	0	0	0	0
Solyc11g045450.1.1	0	0	0	0	0	0	0	0	0	0	0	0	0
Solyc11g045460.1.1	0	0	0	0	0	0	0	0	0	0	0	0	0
Solyc11g045470.1.1	0	0	0	0	0	0	0	0	0	0	0	0	0
Solyc11g045480.1.1	0	0	0	0	0	0	0	0	0	0	0	0	0
Solyc11g045510.1.1	0	0	0	0	0	0	0	0	0	0	0	0	0
Solyc11g045520.1.1	0	0	0	0	0	0	0	0	0	0	0	0	0
Solyc11g045530.1.1	0	0	0	0	0	0	0	0	0	0	0	0	0
Solyc11g045610.1.1	1	0	0	4	0	0	2	11	0	11	0	9	0
Solyc11g045670.1.1	2	12	7	0	2	16	42	21	0	40	3	8	0
Solyc11g051060.1.1	0	0	0	0	0	0	0	0	0	0	0	0	0
Solyc11g051160.1.1	22	1304	61	840	81	481	330	680	129	2720	194	1954	9
Solyc11g051190.1.1	0	0	0	0	0	0	0	0	0	0	0	0	0
Solyc11g051200.1.1	0	0	0	0	0	0	0	0	0	0	0	0	0
Solyc11g051230.1.1	0	0	0	0	0	0	0	0	0	0	0	0	0
Solyc11g056250.1.1	0	0	0	0	0	0	0	0	0	0	0	0	0
Solyc11g056270.1.1	0	0	0	0	0	0	0	0	0	0	0	0	0
Solyc11g056310.1.1	0	0	0	0	0	0	0	0	0	0	0	0	0

Solyc11g056320.1.1	0	0	0	0	0	0	0	0	0	0	0	0	0
Solyc11g056330.1.1	0	0	0	0	0	0	0	0	0	0	0	0	0
Solyc11g056340.1.1	0	0	0	0	0	0	0	0	0	0	0	0	0
Solyc11g056350.1.1	0	0	0	0	0	0	0	0	0	0	0	0	0
Solyc11g056360.1.1	0	0	0	0	0	0	0	0	0	0	0	0	0
Solyc11g056400.1.1	0	0	0	0	0	0	0	0	0	0	0	0	0
Solyc11g056410.1.1	0	0	0	0	0	0	0	0	0	0	0	0	0
Solyc11g056480.1.1	0	0	0	0	0	0	0	0	0	0	0	0	0
Solyc11g056500.1.1	0	0	0	0	0	0	0	0	0	0	0	0	0
Solyc11g056620.1.1	3	2	0	0	0	10	12	13	6	2	0	0	0
Solyc11g056640.1.1	0	0	0	0	0	0	0	0	0	0	0	0	0
Solyc11g056650.1.1	17	24	4	13	0	485	311	448	98	3	2	2	0
Solyc11g056670.1.1	0	0	0	0	0	0	0	0	0	0	0	0	0
Solyc11g056680.1.1	17	362	67	300	31	207	851	324	8	5574	676	5705	73
Solyc11g061720.1.1	5	34	3	15	6	124	157	19	10	42	25	15	4
Solyc11g061760.1.1	0	0	0	0	0	0	0	0	0	0	0	0	0
Solyc11g061770.1.1	5	48	8	47	3	26	23	109	4	99	18	68	0
Solyc11g061960.1.1	0	0	0	0	0	0	0	0	0	0	0	0	0
Solyc11g061980.1.1	0	0	0	0	0	0	0	0	0	0	0	0	0
Solyc11g062010.1.1	34	120	15	195	22	476	375	769	70	277	89	217	7
Solyc11g062020.1.1	4	55	3	31	1	49	93	36	6	25	8	34	0
Solyc11g062060.1.1	0	0	0	0	0	0	0	0	0	0	0	0	0
Solyc11g062070.1.1	3	19	5	7	0	25	11	56	0	43	5	68	0
Solyc11g062080.1.1	0	0	0	0	0	0	0	0	0	0	0	0	0
Solyc11g062100.1.1	11	35	17	54	0	51	86	83	0	77	11	54	0
Solyc11g062120.1.1	4	18	2	22	0	40	21	66	0	23	14	15	0
Solyc11g062130.1.1	1	47	1	48	9	23	20	51	7	177	30	169	2
Solyc11g062190.1.1	2	133	47	62	40	228	236	372	69	335	267	339	20
Solyc11g062220.1.1	17	73	3	76	1	195	80	390	29	110	43	114	0
Solyc11g062240.1.1	20	127	9	143	6	283	130	411	36	228	51	245	0
Solyc11g062250.1.1	0	0	0	0	0	0	0	0	0	0	0	0	0
Solyc11g062260.1.1	1	3	0	3	0	16	1	7	0	4	3	6	0
Solyc11g062270.1.1	15	147	7	82	13	239	171	219	38	253	74	183	0
Solyc11g062280.1.1	0	0	0	0	0	0	0	0	0	0	0	0	0
Solyc11g062300.1.1	3	19	3	10	2	24	31	38	1	36	8	53	0
Solyc11g062310.1.1	6	21	2	8	4	51	34	33	8	34	13	42	0
Solyc11g062320.1.1	2	5	0	5	3	16	12	28	0	19	3	13	0
Solyc11g062330.1.1	0	0	0	0	0	0	0	0	0	0	0	0	0
Solyc11g062350.1.1	3	30	6	17	1	4	6	7	2	1	1	8	0
Solyc11g062360.1.1	12	22	13	17	18	29	100	65	16	42	23	59	3
Solyc11g062370.1.1	4	25	3	18	8	13	13	31	7	20	6	22	0

Solyc11g062390.1.1	1	11	0	5	0	4	5	5	0	1	0	0	1
Solyc11g062400.1.1	0	0	0	0	0	0	0	0	0	0	0	0	0
Solyc11g062410.1.1	2	22	2	34	0	15	22	72	0	34	4	32	2
Solyc11g062430.1.1	2	7	1	7	1	4	12	6	0	12	0	17	0
Solyc11g062440.1.1	4	58	3	31	0	31	15	9	0	39	3	61	3
Solyc11g063500.1.1	0	0	0	0	0	0	0	0	0	0	0	0	0
Solyc11g063510.1.1	0	0	0	0	0	0	0	0	0	0	0	0	0
Solyc11g063520.1.1	0	0	0	0	0	0	0	0	0	0	0	0	0
Solyc11g063600.1.1	1	1	0	2	0	0	4	1	0	0	0	3	0
Solyc11g063610.1.1	0	0	0	0	0	0	0	0	0	0	0	0	0
Solyc11g064750.1.1	0	0	0	0	0	0	0	0	0	0	0	0	0
Solyc11g064770.1.1	0	0	0	0	0	0	0	0	0	0	0	0	0
Solyc11g064780.1.1	0	0	0	0	0	0	0	0	0	0	0	0	0
Solyc11g064790.1.1	8	105	7	30	0	167	52	198	21	163	50	127	0
Solyc11g064800.1.1	1	3	0	4	0	10	9	12	0	0	0	0	0
Solyc11g064830.1.1	5	36	4	54	1	41	37	61	11	83	7	173	1
Solyc11g064840.1.1	0	0	0	0	0	0	0	0	0	0	0	0	0
Solyc11g064850.1.1	7	19	0	4	5	37	24	30	4	87	39	51	2
Solyc11g064860.1.1	0	0	0	0	0	0	0	0	0	0	0	0	0
Solyc11g064870.1.1	3	4	0	8	0	35	10	24	2	3	2	4	1
Solyc11g064880.1.1	3	12	0	7	0	7	7	14	0	17	2	3	0
Solyc11g064890.1.1	9	98	20	76	14	116	130	174	17	168	36	169	5
Solyc11g064900.1.1	0	0	0	0	0	0	0	0	0	0	0	0	0
Solyc11g064920.1.1	9	59	9	51	6	252	323	331	35	155	59	157	7
Solyc11g064950.1.1	4	52	6	26	1	70	22	55	6	60	11	28	0
Solyc11g064980.1.1	2	0	2	4	6	25	4	1	0	0	2	0	0
Solyc11g065000.1.1	8	137	7	97	4	113	50	143	2	108	13	121	4
Solyc11g065020.1.1	1	4	1	1	0	3	4	3	0	2	0	1	0
Solyc11g065030.1.1	1	9	1	3	0	1	4	1	0	1	0	0	0
Solyc11g065040.1.1	1	6	0	2	0	2	5	2	0	19	1	10	0
Solyc11g065070.1.1	7	56	14	16	21	177	170	138	100	56	10	20	8
Solyc11g065080.1.1	3	31	1	0	5	17	8	17	1	18	11	8	0
Solyc11g065090.1.1	7	80	4	14	0	33	73	49	3	85	20	108	3
Solyc11g065100.1.1	2	6	1	11	0	33	5	16	2	19	7	40	0
Solyc11g065110.1.1	12	49	11	56	1	135	68	142	4	87	30	60	6
Solyc11g065120.1.1	5	7	6	6	2	16	38	36	2	39	5	55	0
Solyc11g065140.1.1	7	12	3	14	9	40	38	77	9	24	11	24	2
Solyc11g065150.1.1	6	6	1	6	1	23	20	35	3	34	4	25	0
Solyc11g065160.1.1	7	30	3	33	6	113	43	76	6	60	9	36	2
Solyc11g065180.1.1	9	30	1	15	3	55	34	34	11	45	49	29	4
Solyc11g065190.1.1	0	0	0	0	0	0	0	0	0	0	0	0	0

Solyc11g065210.1.1	4	9	6	10	1	18	8	36	3	27	8	10	0
Solyc11g065220.1.1	0	0	0	0	0	0	0	0	0	0	0	0	0
Solyc11g065240.1.1	6	62	10	43	1	108	109	133	1	69	13	55	0
Solyc11g065250.1.1	0	0	0	0	0	0	0	0	0	0	0	0	0
Solyc11g065260.1.1	9	40	2	22	0	54	38	63	11	70	3	34	0
Solyc11g065270.1.1	4	7	4	15	3	43	52	42	1	27	10	35	7
Solyc11g065320.1.1	12	74	3	54	0	43	50	135	2	144	14	56	4
Solyc11g065340.1.1	2	18	3	9	0	16	15	11	5	4	0	0	0
Solyc11g065350.1.1	13	134	22	3	27	323	224	68	415	168	39	43	31
Solyc11g065360.1.1	0	0	0	0	0	0	0	0	0	0	0	0	0
Solyc11g065370.1.1	6	15	4	3	1	15	51	40	4	18	8	6	0
Solyc11g065380.1.1	2	7	0	1	0	12	3	5	7	6	8	6	0
Solyc11g065420.1.1	0	0	0	0	0	0	0	0	0	0	0	0	0
Solyc11g065490.1.1	9	88	4	40	9	83	34	72	10	298	79	286	9
Solyc11g065520.1.1	0	0	0	0	0	0	0	0	0	0	0	0	0
Solyc11g065530.1.1	9	13	0	0	4	335	27	11	19	107	57	195	10
Solyc11g065600.1.1	0	0	0	0	0	0	0	0	0	0	0	0	0
Solyc11g065620.1.1	14	300	54	211	12	350	220	481	17	271	149	245	7
Solyc11g065630.1.1	0	0	0	0	0	0	0	0	0	0	0	0	0
Solyc11g065640.1.1	1	1	0	0	0	2	4	1	2	5	0	5	0
Solyc11g065660.1.1	1	5	0	7	0	23	5	17	3	6	0	3	0
Solyc11g065670.1.1	6	172	3	73	5	255	23	218	18	384	44	412	4
Solyc11g065680.1.1	2	28	1	28	0	1	29	16	1	17	4	19	0
Solyc11g065690.1.1	4	13	1	11	1	39	8	53	4	18	13	29	1
Solyc11g065700.1.1	8	59	0	33	1	91	16	118	7	100	25	79	0
Solyc11g065720.1.1	0	0	0	0	0	0	0	0	0	0	0	0	0
Solyc11g065730.1.1	6	39	6	11	3	47	49	82	12	56	17	41	0
Solyc11g065740.1.1	7	329	18	91	0	788	63	252	8	31	20	21	0
Solyc11g065750.1.1	8	77	2	54	2	127	43	186	8	350	15	225	1
Solyc11g065760.1.1	0	0	0	0	0	0	0	0	0	0	0	0	0
Solyc11g065770.1.1	0	0	0	0	0	0	0	0	0	0	0	0	0
Solyc11g065780.1.1	0	0	0	0	0	0	0	0	0	0	0	0	0
Solyc11g065790.1.1	0	0	0	0	0	0	0	0	0	0	0	0	0
Solyc11g065800.1.1	1	0	1	1	0	1	2	3	1	3	0	9	0
Solyc11g065810.1.1	1	2	0	4	2	5	6	3	0	12	1	4	0
Solyc11g065820.1.1	5	16	4	21	0	64	30	43	0	6	0	17	0
Solyc11g065830.1.1	8	345	37	226	9	400	50	162	8	118	46	57	3
Solyc11g065840.1.1	0	0	0	0	0	0	0	0	0	0	0	0	0
Solyc11g065870.1.1	2	38	6	15	1	8	67	44	5	28	1	8	0
Solyc11g065880.1.1	4	9	0	9	0	35	33	51	3	40	16	39	0
Solyc11g065890.1.1	0	0	0	0	0	0	0	0	0	0	0	0	0

Solyc11g065900.1.1	0	0	0	0	0	0	0	0	0	0	0	0	0
Solyc11g065920.1.1	31	142	14	136	2	1002	369	1197	53	350	73	323	1
Solyc11g065930.1.1	5	41	0	39	0	32	24	63	3	28	4	22	0
Solyc11g065940.1.1	11	81	5	89	4	247	88	366	41	150	11	139	3
Solyc11g065950.1.1	7	18	4	9	0	77	21	64	8	44	13	73	1
Solyc11g065960.1.1	0	0	0	0	0	0	0	0	0	0	0	0	0
Solyc11g065980.1.1	0	0	0	0	0	0	0	0	0	0	0	0	0
Solyc11g065990.1.1	0	0	0	0	0	0	0	0	0	0	0	0	0
Solyc11g066000.1.1	0	0	0	0	0	0	0	0	0	0	0	0	0
Solyc11g066010.1.1	0	0	0	0	0	0	0	0	0	0	0	0	0
Solyc11g066020.1.1	11	238	23	126	17	417	217	302	29	841	58	435	21
Solyc11g066040.1.1	9	36	0	5	1	65	57	91	6	24	21	16	0
Solyc11g066050.1.1	0	0	0	0	0	0	0	0	0	0	0	0	0
Solyc11g066060.1.1	12	3722	301	3223	273	5231	1433	2376	358	5032	2422	4216	78
Solyc11g066070.1.1	0	0	0	0	0	0	0	0	0	0	0	0	0
Solyc11g066080.1.1	7	66	12	72	5	11	36	13	2	52	16	68	3
Solyc11g066090.1.1	1	2	0	0	0	2	9	2	1	4	1	2	0
Solyc11g066100.1.1	26	49	5	58	7	222	30	157	7	231	707	233	61
Solyc11g066110.1.1	7	254	59	199	3	243	142	203	12	138	16	141	0
Solyc11g066120.1.1	0	0	0	0	0	0	0	0	0	0	0	0	0
Solyc11g066130.1.1	16	68	5	45	0	128	368	289	0	415	100	208	1
Solyc11g066140.1.1	0	0	0	0	0	0	0	0	0	0	0	0	0
Solyc11g066150.1.1	5	18	1	4	0	8	19	21	4	16	10	39	1
Solyc11g066160.1.1	0	0	0	0	0	0	0	0	0	0	0	0	0
Solyc11g066240.1.1	0	0	0	0	0	0	0	0	0	0	0	0	0
Solyc11g066250.1.1	17	439	2	204	1	132	8	107	4	16	633	9	1
Solyc11g066260.1.1	2	15	1	0	0	5	16	33	0	29	0	11	0
Solyc11g066270.1.1	0	0	0	0	0	0	0	0	0	0	0	0	0
Solyc11g066280.1.1	5	39	12	33	0	40	42	67	3	73	16	44	0
Solyc11g066290.1.1	11	73	15	79	10	412	132	534	17	580	80	407	17
Solyc11g066300.1.1	8	26	3	7	0	49	80	30	4	11	14	10	2
Solyc11g066310.1.1	2	1	0	0	0	0	12	6	1	4	0	4	0
Solyc11g066320.1.1	4	18	6	4	5	8	19	44	13	97	15	37	0
Solyc11g066330.1.1	10	61	21	46	13	94	309	246	18	65	19	54	2
Solyc11g066340.1.1	0	0	0	0	0	0	0	0	0	0	0	0	0
Solyc11g066360.1.1	4	43	0	27	0	14	1	25	0	49	24	34	0
Solyc11g066370.1.1	7	18	2	27	2	60	31	32	12	63	20	68	0
Solyc11g066380.1.1	4	48	9	10	11	21	26	35	11	30	20	24	0
Solyc11g066390.1.1	6	238	1	17	10	124	36	57	5	195	9	147	0
Solyc11g066400.1.1	0	0	0	0	0	0	0	0	0	0	0	0	0
Solyc11g066410.1.1	2	89	6	61	4	27	9	47	0	29	1	31	0

Solyc11g066430.1.1	3	51	4	11	4	82	58	73	1	32	22	40	0
Solyc11g066440.1.1	12	198	20	164	0	661	841	767	18	184	94	211	2
Solyc11g066450.1.1	1	4	0	0	0	9	7	5	0	6	0	3	2
Solyc11g066460.1.1	2	13	2	7	0	32	13	27	2	38	9	16	0
Solyc11g066470.1.1	1	106	1	45	2	51	10	72	0	42	3	79	5
Solyc11g066480.1.1	12	47	5	43	8	153	95	196	19	85	22	90	1
Solyc11g066490.1.1	1	3	1	0	0	0	4	4	0	1	1	0	0
Solyc11g066500.1.1	9	42	6	19	0	148	34	100	3	53	21	65	0
Solyc11g066510.1.1	2	9	0	14	0	3	15	7	0	4	2	3	0
Solyc11g066520.1.1	2	14	1	15	0	18	13	4	0	0	1	0	0
Solyc11g066540.1.1	3	9	2	4	0	8	7	16	3	18	5	12	0
Solyc11g066550.1.1	4	9	0	11	0	15	19	39	4	22	4	12	0
Solyc11g066560.1.1	9	50	6	72	5	213	194	125	23	164	74	192	3
Solyc11g066580.1.1	0	0	0	0	0	0	0	0	0	0	0	0	0
Solyc11g066590.1.1	1	7	3	3	0	0	2	2	0	0	0	0	0
Solyc11g066610.1.1	0	0	0	0	0	0	0	0	0	0	0	0	0
Solyc11g066620.1.1	11	155	5	103	5	485	135	537	28	156	94	123	3
Solyc11g066630.1.1	2	6	0	0	0	6	6	10	1	12	2	11	0
Solyc11g066650.1.1	9	27	26	49	17	80	224	92	43	89	193	37	12
Solyc11g066660.1.1	8	89	14	58	3	10	14	32	3	38	11	59	0
Solyc11g066670.1.1	16	7905	1005	7162	163	3810	506	1023	76	1140	3004	505	121
Solyc11g066680.1.1	1	12	3	2	0	0	0	2	0	1	3	1	0
Solyc11g066690.1.1	0	0	0	0	0	0	0	0	0	0	0	0	0
Solyc11g066720.1.1	7	659	56	157	180	169	320	133	397	436	139	328	47
Solyc11g066730.1.1	3	26	1	4	0	38	9	21	9	18	15	14	0
Solyc11g066740.1.1	6	40	1	23	0	147	112	165	4	47	11	27	2
Solyc11g066750.1.1	0	0	0	0	0	0	0	0	0	0	0	0	0
Solyc11g066760.1.1	0	0	0	0	0	0	0	0	0	0	0	0	0
Solyc11g066770.1.1	5	31	0	8	1	54	19	43	6	49	9	33	0
Solyc11g066780.1.1	10	32	3	33	0	94	21	89	2	27	7	21	0
Solyc11g066790.1.1	0	0	0	0	0	0	0	0	0	0	0	0	0
Solyc11g066800.1.1	3	0	0	0	0	55	27	49	3	0	19	0	0
Solyc11g066820.1.1	8	463	40	365	67	75	86	368	16	1717	234	1064	25
Solyc11g066830.1.1	5	68	6	51	4	176	61	168	7	75	45	125	2
Solyc11g066840.1.1	10	108	14	84	24	269	91	324	40	1341	135	1644	18
Solyc11g066850.1.1	1	0	0	8	0	3	6	2	0	4	0	3	0
Solyc11g066860.1.1	1	12	1	6	0	79	14	24	0	53	0	35	0
Solyc11g066870.1.1	13	119	23	91	4	113	219	158	5	117	32	194	4
Solyc11g066890.1.1	5	109	6	33	4	92	39	79	20	59	13	69	5
Solyc11g066900.1.1	5	46	4	16	5	19	41	49	1	53	0	44	0
Solyc11g066910.1.1	3	16	3	9	0	29	21	44	8	56	4	31	0

Solyc11g066920.1.1	0	0	0	0	0	0	0	0	0	0	0	0	0
Solyc11g066930.1.1	0	0	0	0	0	0	0	0	0	0	0	0	0
Solyc11g066940.1.1	0	0	0	0	0	0	0	0	0	0	0	0	0
Solyc11g066950.1.1	0	0	0	0	0	0	0	0	0	0	0	0	0
Solyc11g066960.1.1	2	1	0	0	0	1	14	4	1	6	0	6	0
Solyc11g066970.1.1	0	0	0	0	0	0	0	0	0	0	0	0	0
Solyc11g066980.1.1	1	9	1	5	0	16	13	26	4	11	4	20	0
Solyc11g066990.1.1	3	3	0	16	2	19	17	24	4	12	2	14	0
Solyc11g067000.1.1	0	0	0	0	0	0	0	0	0	0	0	0	0
Solyc11g067010.1.1	5	60	0	15	0	57	20	53	1	192	18	74	1
Solyc11g067020.1.1	3	16	0	11	3	63	27	50	1	19	13	23	0
Solyc11g067030.1.1	0	0	0	0	0	0	0	0	0	0	0	0	0
Solyc11g067040.1.1	0	0	0	0	0	0	0	0	0	0	0	0	0
Solyc11g067050.1.1	13	110	4	80	8	98	119	155	9	232	25	176	4
Solyc11g067060.1.1	1	10	1	7	0	4	7	6	0	3	0	6	0
Solyc11g067070.1.1	4	36	3	18	0	34	32	29	10	60	8	37	0
Solyc11g067080.1.1	7	67	0	49	5	89	8	128	4	100	17	47	0
Solyc11g067090.1.1	6	17	3	4	0	19	46	41	13	36	18	30	0
Solyc11g067100.1.1	1	16	1	7	4	7	22	18	5	73	13	53	3
Solyc11g067110.1.1	0	0	0	0	0	0	0	0	0	0	0	0	0
Solyc11g067120.1.1	0	0	0	0	0	0	0	0	0	0	0	0	0
Solyc11g067130.1.1	0	0	0	0	0	0	0	0	0	0	0	0	0
Solyc11g067140.1.1	0	0	0	0	0	0	0	0	0	0	0	0	0
Solyc11g067150.1.1	0	0	0	0	0	0	0	0	0	0	0	0	0
Solyc11g067160.1.1	9	177	40	87	5	50	261	105	5	78	67	69	2
Solyc11g067170.1.1	0	0	0	0	0	0	0	0	0	0	0	0	0
Solyc11g067180.1.1	0	0	0	0	0	0	0	0	0	0	0	0	0
Solyc11g067190.1.1	9	4	0	1	5	138	128	54	244	0	3	1	0
Solyc11g067200.1.1	18	27	5	25	3	76	108	143	19	100	55	75	5
Solyc11g067210.1.1	0	0	0	0	0	0	0	0	0	0	0	0	0
Solyc11g067230.1.1	8	85	8	51	4	186	76	150	26	123	35	91	0
Solyc11g067240.1.1	1	12	4	9	0	18	5	17	2	13	5	19	0
Solyc11g067250.1.1	0	0	0	0	0	0	0	0	0	0	0	0	0
Solyc11g067260.1.1	9	22	2	18	0	103	87	126	15	81	25	78	4
Solyc11g067270.1.1	0	0	0	0	0	0	0	0	0	0	0	0	0
Solyc11g067280.1.1	13	209	11	105	11	372	171	287	24	304	57	255	11
Solyc11g067300.1.1	0	0	0	0	0	0	0	0	0	0	0	0	0
Solyc11g068360.1.1	0	0	0	0	0	0	0	0	0	0	0	0	0
Solyc11g068370.1.1	0	0	0	0	0	0	0	0	0	0	0	0	0
Solyc11g068380.1.1	0	0	0	0	0	0	0	0	0	0	0	0	0
Solyc11g068400.1.1	1	8	0	3	0	7	3	10	0	3	2	9	0

Solyc11g068420.1.1	1	69	1	17	4	142	25	143	12	192	14	63	1
Solyc11g068430.1.1	4	65	1	6	5	284	21	272	1	707	49	549	7
Solyc11g068440.1.1	11	50	10	38	2	81	23	35	6	106	40	141	9
Solyc11g068450.1.1	0	0	0	0	0	0	0	0	0	0	0	0	0
Solyc11g068460.1.1	3	14	0	11	2	6	11	18	3	31	13	16	9
Solyc11g068470.1.1	0	0	0	0	0	0	0	0	0	0	0	0	0
Solyc11g068480.1.1	1	7	1	2	0	26	7	13	3	25	28	39	0
Solyc11g068490.1.1	1	61	1	24	3	30	2	41	0	80	2	50	0
Solyc11g068500.1.1	10	51	8	32	5	76	77	97	11	111	38	81	5
Solyc11g068510.1.1	1	38	10	22	1	43	31	100	15	70	25	35	0
Solyc11g068520.1.1	7	75	7	43	0	49	38	73	0	27	12	22	0
Solyc11g068530.1.1	1	63	14	17	3	5	16	2	2	1	4	0	0
Solyc11g068540.1.1	5	57	8	52	0	162	78	178	5	173	30	162	4
Solyc11g068550.1.1	0	0	0	0	0	0	0	0	0	0	0	0	0
Solyc11g068610.1.1	5	68	0	52	7	121	22	103	11	84	6	56	2
Solyc11g068620.1.1	0	0	0	0	0	0	0	0	0	0	0	0	0
Solyc11g068640.1.1	14	120	18	99	12	281	126	230	24	348	49	235	19
Solyc11g068650.1.1	7	59	4	42	0	257	77	165	19	164	34	128	3
Solyc11g068660.1.1	13	105	17	83	5	156	237	184	61	132	92	124	8
Solyc11g068670.1.1	8	70	29	37	4	135	117	165	29	76	28	78	0
Solyc11g068680.1.1	0	0	0	0	0	0	0	0	0	0	0	0	0
Solyc11g068690.1.1	3	5	0	1	0	15	8	6	0	24	5	15	0
Solyc11g068700.1.1	0	0	0	0	0	0	0	0	0	0	0	0	0
Solyc11g068710.1.1	1	48	6	35	0	18	4	8	2	9	0	23	0
Solyc11g068720.1.1	0	0	0	0	0	0	0	0	0	0	0	0	0
Solyc11g068730.1.1	6	118	2	44	0	24	24	56	2	81	40	55	0
Solyc11g068740.1.1	2	49	0	31	0	38	21	47	1	47	3	43	0
Solyc11g068750.1.1	1	1	0	0	0	2	8	5	1	10	0	0	0
Solyc11g068760.1.1	1	6	0	0	0	6	7	4	0	5	1	9	0
Solyc11g068770.1.1	2	28	4	15	0	25	14	8	2	12	6	2	0
Solyc11g068780.1.1	0	0	0	0	0	0	0	0	0	0	0	0	0
Solyc11g068790.1.1	7	79	8	40	0	42	53	20	3	35	1	31	1
Solyc11g068800.1.1	1	3	0	3	0	6	4	3	0	0	0	4	0
Solyc11g068810.1.1	0	0	0	0	0	0	0	0	0	0	0	0	0
Solyc11g068820.1.1	4	380	21	144	0	59	100	156	8	104	29	108	0
Solyc11g068830.1.1	7	166	6	86	5	534	69	403	12	775	37	433	5
Solyc11g068870.1.1	3	6	0	2	0	11	35	11	6	29	5	7	0
Solyc11g068880.1.1	0	0	0	0	0	0	0	0	0	0	0	0	0
Solyc11g068900.1.1	1	0	0	0	0	0	4	3	2	6	1	6	0
Solyc11g068910.1.1	0	0	0	0	0	0	0	0	0	0	0	0	0
Solyc11g068930.1.1	0	0	0	0	0	0	0	0	0	0	0	0	0

Solyc11g068940.1.1	0	0	0	0	0	0	0	0	0	0	0	0	0
Solyc11g068950.1.1	27	679	87	789	43	1471	1333	3130	219	2757	431	2612	15
Solyc11g068960.1.1	22	140	10	172	8	260	158	556	11	363	16	165	1
Solyc11g068970.1.1	0	0	0	0	0	0	0	0	0	0	0	0	0
Solyc11g068980.1.1	2	6	0	7	1	21	10	24	1	11	2	11	1
Solyc11g068990.1.1	2	3	0	3	0	2	3	16	1	14	2	11	0
Solyc11g069000.1.1	5	129	29	116	12	432	183	405	33	521	95	425	8
Solyc11g069010.1.1	8	24	10	27	0	5	33	43	5	65	17	20	1
Solyc11g069020.1.1	0	0	0	0	0	0	0	0	0	0	0	0	0
Solyc11g069040.2.1	5	116	20	37	4	997	486	389	60	151	66	193	15
Solyc11g069050.1.1	12	143	12	94	9	673	209	666	66	184	19	193	5
Solyc11g069070.1.1	0	0	0	0	0	0	0	0	0	0	0	0	0
Solyc11g069080.1.1	0	0	0	0	0	0	0	0	0	0	0	0	0
Solyc11g069090.1.1	14	2726	364	1940	159	3659	2466	3375	220	9901	1666	8430	120
Solyc11g069120.1.1	0	0	0	0	0	0	0	0	0	0	0	0	0
Solyc11g069150.1.1	9	146	14	81	11	285	185	319	56	233	54	189	4
Solyc11g069160.1.1	13	120	18	88	10	176	151	262	65	209	50	199	7
Solyc11g069170.1.1	14	16	3	25	3	24	60	79	18	93	36	62	0
Solyc11g069180.1.1	12	90	2	71	5	664	423	493	26	119	8	57	0
Solyc11g069190.1.1	3	60	2	50	0	17	7	30	0	364	13	204	2
Solyc11g069220.1.1	0	0	0	0	0	0	0	0	0	0	0	0	0
Solyc11g069230.1.1	0	0	0	0	0	0	0	0	0	0	0	0	0
Solyc11g069240.1.1	0	0	0	0	0	0	0	0	0	0	0	0	0
Solyc11g069250.1.1	2	42	0	16	0	49	8	47	0	0	0	0	0
Solyc11g069260.1.1	0	0	0	0	0	0	0	0	0	0	0	0	0
Solyc11g069270.1.1	18	443	32	188	16	636	812	987	123	329	78	212	9
Solyc11g069280.1.1	0	0	0	0	0	0	0	0	0	0	0	0	0
Solyc11g069290.1.1	1	11	0	2	0	2	1	8	0	56	4	48	0
Solyc11g069300.1.1	1	0	0	5	0	8	5	10	0	19	2	2	0
Solyc11g069310.1.1	2	11	0	16	0	20	17	22	4	14	7	23	0
Solyc11g069320.1.1	3	35	0	20	0	61	17	69	0	91	6	99	0
Solyc11g069330.1.1	25	285	155	224	42	1307	1905	854	19	966	294	857	34
Solyc11g069340.1.1	20	66	6	68	13	286	108	321	22	212	48	161	2
Solyc11g069350.1.1	3	11	4	18	1	19	31	28	2	34	74	30	8
Solyc11g069360.1.1	3	19	1	18	0	14	17	33	4	42	13	27	0
Solyc11g069370.1.1	2	16	0	12	0	7	3	7	2	14	7	28	0
Solyc11g069380.1.1	19	642	67	635	10	723	461	1147	25	388	87	539	9
Solyc11g069390.1.1	0	0	0	0	0	0	0	0	0	0	0	0	0
Solyc11g069400.1.1	12	141	15	106	8	183	94	278	17	310	60	245	0
Solyc11g069410.1.1	0	0	0	0	0	0	0	0	0	0	0	0	0
Solyc11g069420.1.1	0	0	0	0	0	0	0	0	0	0	0	0	0

Solyc11g069430.1.1	8	1387	102	1059	51	580	289	1085	32	10076	378	5695	83
Solyc11g069440.1.1	2	64	4	15	4	21	38	51	12	109	26	74	2
Solyc11g069450.1.1	3	35	0	26	0	48	21	30	0	7	1	11	0
Solyc11g069460.1.1	3	12	7	3	15	2	23	13	15	21	3	16	1
Solyc11g069470.1.1	16	142	24	96	1	133	205	155	7	131	49	75	21
Solyc11g069480.1.1	3	15	1	7	1	34	19	23	3	23	6	12	0
Solyc11g069490.1.1	11	108	11	57	7	184	124	217	24	214	33	90	6
Solyc11g069500.1.1	7	25	4	15	10	143	81	118	5	186	24	208	1
Solyc11g069510.1.1	7	70	5	46	0	33	25	48	2	56	6	37	1
Solyc11g069520.1.1	1	1	3	0	0	4	2	7	4	1	0	20	0
Solyc11g069530.1.1	8	12	3	13	0	37	36	57	8	39	12	22	0
Solyc11g069540.1.1	1	7	1	4	0	12	2	22	0	2	2	0	0
Solyc11g069550.1.1	1	15	0	10	5	47	23	18	0	53	14	36	1
Solyc11g069560.1.1	0	0	0	0	0	0	0	0	0	0	0	0	0
Solyc11g069570.1.1	7	310	8	188	0	228	83	153	0	15	11	27	0
Solyc11g069580.1.1	1	17	0	10	0	13	1	8	0	1	1	2	0
Solyc11g069590.1.1	3	1	0	0	2	3	19	8	0	37	13	29	11
Solyc11g069600.1.1	4	41	6	45	8	35	22	36	1	43	7	27	0
Solyc11g069610.1.1	11	21	2	19	3	61	38	93	4	45	21	23	6
Solyc11g069620.1.1	0	0	0	0	0	0	0	0	0	0	0	0	0
Solyc11g069640.1.1	1	4	0	3	0	1	5	4	0	0	0	2	0
Solyc11g069650.1.1	0	0	0	0	0	0	0	0	0	0	0	0	0
Solyc11g069660.1.1	0	0	0	0	0	0	0	0	0	0	0	0	0
Solyc11g069690.1.1	6	32	1	15	5	26	19	42	8	28	2	14	0
Solyc11g069700.1.1	9	515	42	282	50	775	370	915	322	1063	243	786	50
Solyc11g069710.1.1	4	5	0	5	0	2	14	9	0	21	4	29	2
Solyc11g069720.1.1	8	379	38	261	15	1124	422	1014	97	520	106	302	3
Solyc11g069730.1.1	0	0	0	0	0	0	0	0	0	0	0	0	0
Solyc11g069770.1.1	0	0	0	0	0	0	0	0	0	0	0	0	0
Solyc11g069780.1.1	3	15	0	6	0	31	0	33	0	36	9	15	0
Solyc11g069790.1.1	11	457	64	271	9	321	368	614	15	496	315	693	11
Solyc11g069800.1.1	1	14	5	6	0	10	0	19	0	10	1	9	0
Solyc11g069810.1.1	2	4	0	2	0	2	14	7	0	3	0	7	0
Solyc11g069820.1.1	0	0	0	0	0	0	0	0	0	0	0	0	0
Solyc11g069830.1.1	0	0	0	0	0	0	0	0	0	0	0	0	0
Solyc11g069840.1.1	0	0	0	0	0	0	0	0	0	0	0	0	0
Solyc11g069850.1.1	0	0	0	0	0	0	0	0	0	0	0	0	0
Solyc11g069860.1.1	2	12	0	10	0	19	7	18	0	37	0	37	0
Solyc11g069890.1.1	1	0	4	0	0	0	3	0	0	6	0	15	0
Solyc11g069900.1.1	0	0	0	0	0	0	0	0	0	0	0	0	0
Solyc11g069910.1.1	5	88	3	62	3	130	80	133	18	144	20	69	2

Solyc11g069940.1.1	1	37	5	49	0	20	1	16	0	137	0	81	0
Solyc11g069950.1.1	10	147	13	101	14	337	157	442	29	186	43	118	8
Solyc11g069960.1.1	0	0	0	0	0	0	0	0	0	0	0	0	0
Solyc11g069970.1.1	0	0	0	0	0	0	0	0	0	0	0	0	0
Solyc11g069980.1.1	0	0	0	0	0	0	0	0	0	0	0	0	0
Solyc11g069990.1.1	1	4	0	1	0	9	10	18	1	7	0	8	0
Solyc11g070000.1.1	1	1	0	1	0	7	0	2	0	3	0	7	0
Solyc11g070020.1.1	6	13	0	3	0	40	35	44	2	34	5	23	0
Solyc11g070030.1.1	9	279	37	106	9	634	420	514	58	603	138	575	11
Solyc11g070040.1.1	0	0	0	0	0	0	0	0	0	0	0	0	0
Solyc11g070050.1.1	1	13	0	10	0	2	5	16	0	15	2	3	0
Solyc11g070060.1.1	0	0	0	0	0	0	0	0	0	0	0	0	0
Solyc11g070070.1.1	1	1	0	3	0	10	2	6	1	7	0	7	0
Solyc11g070100.1.1	13	128	12	127	5	136	98	164	14	476	62	307	3
Solyc11g070110.1.1	1	1	0	0	0	7	8	7	0	6	0	6	0
Solyc11g070120.1.1	2	7	1	10	0	2	15	9	2	22	2	31	0
Solyc11g070130.1.1	0	0	0	0	0	0	0	0	0	0	0	0	0
Solyc11g070140.1.1	0	0	0	0	0	0	0	0	0	0	0	0	0
Solyc11g070150.1.1	0	0	0	0	0	0	0	0	0	0	0	0	0
Solyc11g070160.1.1	1	6	0	0	0	7	1	4	0	7	2	3	0
Solyc11g070170.1.1	4	29	1	17	0	59	18	72	6	27	10	8	0
Solyc11g070190.1.1	0	0	0	0	0	0	0	0	0	0	0	0	0
Solyc11g071200.1.1	0	0	0	0	0	0	0	0	0	0	0	0	0
Solyc11g071210.1.1	0	0	0	0	0	0	0	0	0	0	0	0	0
Solyc11g071220.1.1	2	9	0	7	0	9	13	21	0	25	10	20	0
Solyc11g071230.1.1	1	4	0	0	0	0	4	4	0	1	0	0	0
Solyc11g071240.1.1	2	8	1	14	0	2	2	2	0	18	4	11	0
Solyc11g071250.1.1	5	28	6	4	1	47	13	49	5	36	13	34	0
Solyc11g071260.1.1	10	298	1	121	2	474	71	442	18	308	40	195	10
Solyc11g071270.1.1	12	33	5	18	1	180	64	91	16	159	40	132	3
Solyc11g071280.1.1	7	5	8	5	0	20	5	30	0	35	42	45	0
Solyc11g071300.1.1	0	0	0	0	0	0	0	0	0	0	0	0	0
Solyc11g071320.1.1	4	9	1	7	1	21	15	22	1	63	21	92	7
Solyc11g071330.1.1	1	7	1	0	5	12	8	5	3	3	16	8	0
Solyc11g071340.1.1	4	11	3	11	0	25	25	42	5	18	1	15	0
Solyc11g071350.1.1	0	0	0	0	0	0	0	0	0	0	0	0	0
Solyc11g071370.1.1	0	0	0	0	0	0	0	0	0	0	0	0	0
Solyc11g071420.1.1	0	0	0	0	0	0	0	0	0	0	0	0	0
Solyc11g071430.1.1	1	2	0	0	0	2	4	10	0	0	0	3	0
Solyc11g071460.1.1	1	3	2	0	0	0	0	4	1	5	2	8	0
Solyc11g071470.1.1	0	0	0	0	0	0	0	0	0	0	0	0	0

Solyc11g071480.1.1	0	0	0	0	0	0	0	0	0	0	0	0	0
Solyc11g071490.1.1	1	9	1	21	0	13	8	35	2	36	11	57	2
Solyc11g071500.1.1	2	3	0	0	0	7	3	4	1	20	13	7	0
Solyc11g071510.1.1	10	52	3	31	0	59	36	65	3	225	15	105	1
Solyc11g071520.1.1	0	0	0	0	0	0	0	0	0	0	0	0	0
Solyc11g071530.1.1	0	0	0	0	0	0	0	0	0	0	0	0	0
Solyc11g071540.1.1	8	25	5	22	0	46	48	64	10	56	19	33	1
Solyc11g071550.1.1	0	0	0	0	0	0	0	0	0	0	0	0	0
Solyc11g071560.1.1	0	0	0	0	0	0	0	0	0	0	0	0	0
Solyc11g071570.1.1	0	0	0	0	0	0	0	0	0	0	0	0	0
Solyc11g071580.1.1	0	0	0	0	0	0	0	0	0	0	0	0	0
Solyc11g071600.1.1	0	0	0	0	0	0	0	0	0	0	0	0	0
Solyc11g071610.1.1	0	0	0	0	0	0	0	0	0	0	0	0	0
Solyc11g071620.1.1	16	186	39	73	39	74	376	93	53	679	48	568	15
Solyc11g071640.1.1	13	150	5	89	4	10	89	39	2	14	24	60	0
Solyc11g071660.1.1	15	113	18	107	4	281	151	389	34	275	85	216	2
Solyc11g071670.1.1	0	0	0	0	0	0	0	0	0	0	0	0	0
Solyc11g071680.1.1	3	8	3	6	0	13	8	12	2	15	9	4	1
Solyc11g071690.1.1	10	23	6	16	0	28	99	60	11	51	17	51	3
Solyc11g071700.1.1	17	54	10	45	0	158	143	243	34	151	55	122	5
Solyc11g071710.1.1	3	30	2	12	0	12	12	28	0	1	0	8	0
Solyc11g071720.1.1	0	0	0	0	0	0	0	0	0	0	0	0	0
Solyc11g071730.1.1	0	0	0	0	0	0	0	0	0	0	0	0	0
Solyc11g071740.1.1	4	183	23	93	5	71	0	7	2	5	0	3	0
Solyc11g071760.1.1	0	0	0	0	0	0	0	0	0	0	0	0	0
Solyc11g071770.1.1	16	25	4	28	0	138	57	173	13	121	26	68	1
Solyc11g071780.1.1	1	2	1	1	0	1	2	13	0	2	0	1	0
Solyc11g071790.1.1	5	68	9	14	1	125	55	102	12	124	18	109	2
Solyc11g071800.1.1	6	96	0	48	0	19	44	85	25	176	27	167	0
Solyc11g071810.1.1	2	8	0	0	0	3	2	5	0	20	2	14	0
Solyc11g071820.1.1	1	4	3	1	0	0	0	1	1	25	3	7	0
Solyc11g071830.1.1	11	18	0	10	0	62	11	43	1	79	131	76	0
Solyc11g071840.1.1	1	0	3	0	0	0	2	1	0	6	0	5	0
Solyc11g071850.1.1	7	27	7	7	0	37	170	68	25	87	7	97	1
Solyc11g071860.1.1	0	0	0	0	0	0	0	0	0	0	0	0	0
Solyc11g071870.1.1	1	3	0	6	0	9	11	16	0	8	0	6	0
Solyc11g071890.1.1	0	0	0	0	0	0	0	0	0	0	0	0	0
Solyc11g071910.1.1	20	123	16	158	12	307	148	398	29	337	71	220	13
Solyc11g071920.1.1	15	41	2	68	4	90	73	138	14	102	29	92	10
Solyc11g071930.1.1	0	0	0	0	0	0	0	0	0	0	0	0	0
Solyc11g071940.1.1	0	0	0	0	0	0	0	0	0	0	0	0	0

Solyc11g071950.1.1	6	14	0	5	4	15	24	32	6	31	19	30	3
Solyc11g071960.1.1	3	3	0	9	0	17	39	16	0	22	6	27	5
Solyc11g071970.1.1	2	6	0	2	0	2	18	9	0	30	2	16	0
Solyc11g071980.1.1	0	0	0	0	0	0	0	0	0	0	0	0	0
Solyc11g071990.1.1	0	0	0	0	0	0	0	0	0	0	0	0	0
Solyc11g072010.1.1	2	3	0	0	0	2	14	6	1	21	6	19	0
Solyc11g072030.1.1	7	119	4	2	2	252	27	40	39	67	4	10	11
Solyc11g072040.1.1	0	0	0	0	0	0	0	0	0	0	0	0	0
Solyc11g072050.1.1	5	40	8	19	3	27	128	34	14	14	31	18	0
Solyc11g072070.1.1	1	1	0	5	0	1	2	5	0	22	4	0	0
Solyc11g072080.1.1	0	0	0	0	0	0	0	0	0	0	0	0	0
Solyc11g072090.1.1	6	12	5	14	0	31	55	49	14	47	18	35	1
Solyc11g072100.1.1	0	0	0	0	0	0	0	0	0	0	0	0	0
Solyc11g072110.1.1	5	333	9	120	0	142	22	75	2	504	42	311	0
Solyc11g072120.1.1	0	0	0	0	0	0	0	0	0	0	0	0	0
Solyc11g072130.1.1	0	0	0	0	0	0	0	0	0	0	0	0	0
Solyc11g072140.1.1	0	0	0	0	0	0	0	0	0	0	0	0	0
Solyc11g072150.1.1	4	6	0	5	0	9	18	14	2	9	7	4	0
Solyc11g072160.1.1	15	76	4	62	3	275	139	304	31	42	56	60	1
Solyc11g072170.1.1	2	16	0	8	0	6	10	4	0	2	5	7	0
Solyc11g072180.1.1	1	5	1	0	0	0	2	6	0	19	1	5	0
Solyc11g072190.1.1	11	873	56	457	40	1217	332	1274	94	3803	575	3414	48
Solyc11g072200.1.1	0	0	0	0	0	0	0	0	0	0	0	0	0
Solyc11g072220.1.1	2	6	0	8	0	11	2	8	1	1	2	5	0
Solyc11g072230.1.1	0	0	0	0	0	0	0	0	0	0	0	0	0
Solyc11g072240.1.1	1	36	2	32	4	44	30	55	11	56	11	20	1
Solyc11g072250.1.1	0	0	0	0	0	0	0	0	0	0	0	0	0
Solyc11g072260.1.1	1	13	0	9	0	4	6	15	0	37	1	17	0
Solyc11g072270.1.1	2	17	2	0	0	30	12	13	2	8	9	6	0
Solyc11g072280.1.1	0	0	0	0	0	0	0	0	0	0	0	0	0
Solyc11g072290.1.1	1	2	0	0	0	4	2	7	0	7	4	5	0
Solyc11g072300.1.1	1	1	0	0	1	0	8	1	0	7	0	4	1
Solyc11g072310.1.1	2	0	0	0	0	7	0	9	0	2	17	5	0
Solyc11g072320.1.1	1	2	0	13	1	15	1	15	0	36	5	53	0
Solyc11g072330.1.1	4	4	1	0	0	8	11	8	1	19	3	23	0
Solyc11g072340.1.1	8	319	15	151	7	301	100	368	18	402	58	311	4
Solyc11g072380.1.1	20	4	0	10	0	453	72	200	1	20	346	28	6
Solyc11g072390.1.1	1	3	0	1	1	4	1	3	0	7	3	0	0
Solyc11g072400.1.1	0	0	0	0	0	0	0	0	0	0	0	0	0
Solyc11g072410.1.1	0	0	0	0	0	0	0	0	0	0	0	0	0
Solyc11g072420.1.1	6	12	5	1	2	14	49	36	4	39	7	37	0

Solyc11g072430.1.1	1	0	0	0	0	10	4	5	0	6	0	2	0
Solyc11g072440.1.1	10	58	8	18	0	84	60	118	20	107	45	87	2
Solyc11g072450.1.1	3	126	0	75	0	217	45	359	5	109	19	96	0
Solyc11g072460.1.1	3	21	1	19	0	13	29	41	1	173	14	99	0
Solyc11g072470.1.1	3	16	0	5	0	185	1	69	0	61	28	20	0
Solyc11g072480.1.1	9	169	17	81	3	47	98	98	13	59	5	42	0
Solyc11g072490.1.1	6	64	6	54	14	4	17	16	7	132	31	117	4
Solyc11g072500.1.1	1	1	1	0	0	6	2	9	0	0	1	0	0
Solyc11g072510.1.1	2	9	0	4	0	8	11	25	0	17	3	4	0
Solyc11g072520.1.1	1	18	2	10	0	25	4	31	0	14	2	27	0
Solyc11g072530.1.1	6	87	15	32	11	134	85	120	14	63	21	75	9
Solyc11g072540.1.1	11	83	6	32	1	88	129	139	10	161	32	117	2
Solyc11g072550.1.1	5	132	13	44	16	314	242	282	37	122	49	130	3
Solyc11g072580.1.1	0	0	0	0	0	0	0	0	0	0	0	0	0
Solyc11g072590.1.1	21	144	41	131	12	184	302	413	42	296	66	285	9
Solyc11g072600.1.1	5	12	1	7	0	25	22	19	2	23	4	15	0
Solyc11g072610.1.1	1	1	0	6	0	5	10	0	0	10	4	5	1
Solyc11g072620.1.1	0	0	0	0	0	0	0	0	0	0	0	0	0
Solyc11g072630.1.1	0	0	0	0	0	0	0	0	0	0	0	0	0
Solyc11g072640.1.1	4	11	1	4	5	36	19	26	4	43	8	18	2
Solyc11g072660.1.1	4	9	2	10	2	59	20	54	2	22	5	16	0
Solyc11g072670.1.1	0	0	0	0	0	0	0	0	0	0	0	0	0
Solyc11g072680.1.1	0	0	0	0	0	0	0	0	0	0	0	0	0
Solyc11g072690.1.1	1	1	0	3	0	5	9	7	0	30	0	10	0
Solyc11g072710.1.1	26	665	196	529	56	137	462	314	8	1157	115	947	38
Solyc11g072720.1.1	0	0	0	0	0	0	0	0	0	0	0	0	0
Solyc11g072730.1.1	1	0	0	0	0	4	5	9	0	0	0	0	0
Solyc11g072740.1.1	1	17	0	3	5	14	2	11	5	6	2	6	0
Solyc11g072750.1.1	0	0	0	0	0	0	0	0	0	0	0	0	0
Solyc11g072760.1.1	0	0	0	0	0	0	0	0	0	0	0	0	0
Solyc11g072800.1.1	0	0	0	0	0	0	0	0	0	0	0	0	0
Solyc11g072810.1.1	23	101	9	83	13	244	186	322	47	123	80	117	2
Solyc11g072820.1.1	7	52	1	41	3	17	12	40	2	161	18	67	2
Solyc11g072830.1.1	0	0	0	0	0	0	0	0	0	0	0	0	0
Solyc11g072840.1.1	0	0	0	0	0	0	0	0	0	0	0	0	0
Solyc11g072850.1.1	0	0	0	0	0	0	0	0	0	0	0	0	0
Solyc11g072860.1.1	1	33	1	0	0	5	5	5	0	31	35	41	0
Solyc11g072880.1.1	12	175	12	146	13	255	118	469	35	226	117	218	3
Solyc11g072890.1.1	0	0	0	0	0	0	0	0	0	0	0	0	0
Solyc11g072910.1.1	3	9	2	16	1	33	35	20	3	39	16	31	0
Solyc11g072930.1.1	0	0	0	0	0	0	0	0	0	0	0	0	0

Solyc11g072940.1.1	8	50	12	31	6	147	99	167	25	123	28	99	2
Solyc11g072950.1.1	14	52	18	36	0	122	169	189	24	103	33	93	0
Solyc11g072960.1.1	0	0	0	0	0	0	0	0	0	0	0	0	0
Solyc11g072970.1.1	9	26	6	22	2	59	78	81	7	99	13	53	0
Solyc11g072980.1.1	0	0	0	0	0	0	0	0	0	0	0	0	0
Solyc11g072990.1.1	5	53	0	0	10	170	89	13	139	33	1	7	7
Solyc11g073010.1.1	0	0	0	0	0	0	0	0	0	0	0	0	0
Solyc11g073020.1.1	9	64	11	17	0	116	149	144	23	0	0	0	0
Solyc11g073030.1.1	0	0	0	0	0	0	0	0	0	0	0	0	0
Solyc11g073050.1.1	4	72	2	29	0	86	34	66	7	92	23	34	0
Solyc11g073060.1.1	0	0	0	0	0	0	0	0	0	0	0	0	0
Solyc11g073080.1.1	0	0	0	0	0	0	0	0	0	0	0	0	0
Solyc11g073090.1.1	0	0	0	0	0	0	0	0	0	0	0	0	0
Solyc11g073110.1.1	0	0	0	0	0	0	0	0	0	0	0	0	0
Solyc11g073120.1.1	2	11	1	5	0	12	3	12	0	2	4	15	0
Solyc11g073130.1.1	1	71	0	10	6	6	1	12	0	17	2	9	0
Solyc11g073150.1.1	0	0	0	0	0	0	0	0	0	0	0	0	0
Solyc11g073160.1.1	1	5	1	6	0	0	5	12	0	0	2	0	0
Solyc11g073180.1.1	0	0	0	0	0	0	0	0	0	0	0	0	0
Solyc11g073190.1.1	0	0	0	0	0	0	0	0	0	0	0	0	0
Solyc11g073200.1.1	6	11	0	3	1	56	38	36	11	73	7	67	0
Solyc11g073210.1.1	0	0	0	0	0	0	0	0	0	0	0	0	0
Solyc11g073230.1.1	0	0	0	0	0	0	0	0	0	0	0	0	0
Solyc11g073240.1.1	9	133	13	44	4	267	206	290	22	136	50	59	0
Solyc11g073250.1.1	3	58	5	24	3	180	72	240	2	101	22	107	0
Solyc11g073260.1.1	1	15	3	1	2	12	9	4	0	5	3	6	0
Solyc11g073270.1.1	0	0	0	0	0	0	0	0	0	0	0	0	0
Solyc11g073280.1.1	1	41	1	23	1	42	21	54	3	43	4	15	0
Solyc11g073290.1.1	0	0	0	0	0	0	0	0	0	0	0	0	0
Solyc11g073300.1.1	2	21	0	5	0	15	5	32	1	25	4	35	0
Solyc12g005000.1.1	0	0	0	0	0	0	0	0	0	0	0	0	0
Solyc12g005010.1.1	12	24	5	19	3	51	38	65	4	71	24	45	0
Solyc12g005020.1.1	0	0	0	0	0	0	0	0	0	0	0	0	0
Solyc12g005030.1.1	0	0	0	0	0	0	0	0	0	0	0	0	0
Solyc12g005040.1.1	2	5	2	5	0	3	11	3	0	3	0	2	0
Solyc12g005050.1.1	0	0	0	0	0	0	0	0	0	0	0	0	0
Solyc12g005060.1.1	4	12	0	4	1	13	17	30	1	33	3	16	0
Solyc12g005070.1.1	0	0	0	0	0	0	0	0	0	0	0	0	0
Solyc12g005080.1.1	3	48	7	28	7	155	26	82	4	137	13	111	1
Solyc12g005090.1.1	0	0	0	0	0	0	0	0	0	0	0	0	0
Solyc12g005100.1.1	1	2	1	3	2	12	4	10	2	2	1	3	0

Solyc12g005110.1.1	0	0	0	0	0	0	0	0	0	0	0	0	0
Solyc12g005120.1.1	0	0	0	0	0	0	0	0	0	0	0	0	0
Solyc12g005130.1.1	1	5	0	2	0	3	4	6	0	1	1	10	0
Solyc12g005140.1.1	0	0	0	0	0	0	0	0	0	0	0	0	0
Solyc12g005150.1.1	2	23	5	8	3	22	45	19	0	75	20	102	6
Solyc12g005180.1.1	12	159	18	51	3	225	189	211	0	280	28	235	6
Solyc12g005190.1.1	0	0	0	0	0	0	0	0	0	0	0	0	0
Solyc12g005200.1.1	4	2	3	0	0	23	25	38	5	10	9	12	0
Solyc12g005220.1.1	6	32	0	16	0	73	40	73	8	115	7	104	1
Solyc12g005230.1.1	2	1	0	5	2	4	4	3	0	7	0	4	1
Solyc12g005240.1.1	8	41	12	37	0	17	52	65	3	16	8	31	0
Solyc12g005250.1.1	0	0	0	0	0	0	0	0	0	0	0	0	0
Solyc12g005270.1.1	3	71	5	47	2	62	88	100	19	92	45	146	12
Solyc12g005280.1.1	3	4	0	10	0	5	12	14	0	8	0	10	0
Solyc12g005290.1.1	2	3	0	2	1	5	9	5	0	9	0	14	0
Solyc12g005300.1.1	6	266	38	70	3	24	120	42	0	0	0	2	0
Solyc12g005310.1.1	0	0	0	0	0	0	0	0	0	0	0	0	0
Solyc12g005320.1.1	0	0	0	0	0	0	0	0	0	0	0	0	0
Solyc12g005330.1.1	1	22	0	14	0	37	14	33	2	111	9	119	1
Solyc12g005340.1.1	4	246	32	182	9	49	0	30	0	38	1	81	0
Solyc12g005350.1.1	0	0	0	0	0	0	0	0	0	0	0	0	0
Solyc12g005380.1.1	0	0	0	0	0	0	0	0	0	0	0	0	0
Solyc12g005390.1.1	5	40	3	32	0	52	56	45	10	40	9	33	1
Solyc12g005400.1.1	16	204	72	150	0	442	499	1028	1	6	4	8	0
Solyc12g005410.1.1	3	4	2	0	1	1	23	12	6	10	3	7	1
Solyc12g005420.1.1	0	0	0	0	0	0	0	0	0	0	0	0	0
Solyc12g005430.1.1	0	0	0	0	0	0	0	0	0	0	0	0	0
Solyc12g005450.1.1	0	0	0	0	0	0	0	0	0	0	0	0	0
Solyc12g005470.1.1	8	27	4	2	4	38	65	24	6	20	10	15	1
Solyc12g005480.1.1	0	0	0	0	0	0	0	0	0	0	0	0	0
Solyc12g005550.1.1	0	0	0	0	0	0	0	0	0	0	0	0	0
Solyc12g005560.1.1	0	0	0	0	0	0	0	0	0	0	0	0	0
Solyc12g005570.1.1	0	0	0	0	0	0	0	0	0	0	0	0	0
Solyc12g005580.1.1	0	0	0	0	0	0	0	0	0	0	0	0	0
Solyc12g005590.1.1	2	5	0	4	0	10	5	12	1	11	12	10	0
Solyc12g005610.1.1	0	0	0	0	0	0	0	0	0	0	0	0	0
Solyc12g005620.1.1	0	0	0	0	0	0	0	0	0	0	0	0	0
Solyc12g005630.1.1	2	794	61	472	8	95	249	191	2	64	59	124	1
Solyc12g005640.1.1	0	0	0	0	0	0	0	0	0	0	0	0	0
Solyc12g005660.1.1	1	8	3	5	0	0	1	0	0	0	0	0	0
Solyc12g005690.1.1	0	0	0	0	0	0	0	0	0	0	0	0	0

Solyc12g005700.1.1	0	0	0	0	0	0	0	0	0	0	0	0	0
Solyc12g005720.1.1	1	9	0	3	0	27	5	30	1	0	0	0	0
Solyc12g005730.1.1	3	3	0	8	0	76	10	13	5	8	3	6	0
Solyc12g005750.1.1	2	1	1	1	0	24	2	0	0	20	11	46	0
Solyc12g005760.1.1	2	13	0	13	0	24	6	21	1	26	4	40	0
Solyc12g005770.1.1	5	43	4	50	5	142	54	128	26	86	14	108	0
Solyc12g005780.1.1	5	51	6	25	0	138	51	144	7	65	13	47	0
Solyc12g005820.1.1	0	0	0	0	0	0	0	0	0	0	0	0	0
Solyc12g005840.1.1	5	19	10	2	5	14	26	11	24	120	3	111	0
Solyc12g005850.1.1	7	22	4	11	2	36	36	53	10	30	16	33	0
Solyc12g005860.1.1	28	556	72	334	97	3624	3200	5123	293	3362	713	1944	20
Solyc12g005870.1.1	12	29	10	22	0	89	133	89	6	120	50	107	1
Solyc12g005880.1.1	10	83	18	46	3	228	125	169	33	117	60	77	0
Solyc12g005900.1.1	0	0	0	0	0	0	0	0	0	0	0	0	0
Solyc12g005910.1.1	6	181	9	77	10	988	258	1201	211	112	32	82	4
Solyc12g005920.1.1	0	0	0	0	0	0	0	0	0	0	0	0	0
Solyc12g005930.1.1	3	9	1	6	0	9	1	11	0	13	4	9	0
Solyc12g005940.1.1	3	2	0	5	0	12	5	27	5	4	10	0	2
Solyc12g005950.1.1	20	120	13	89	17	157	116	184	18	160	56	106	2
Solyc12g005960.1.1	2	5	0	3	0	9	8	11	0	2	0	4	0
Solyc12g005970.1.1	1	1	0	0	0	11	5	5	0	2	0	2	0
Solyc12g005980.1.1	24	357	27	246	16	958	251	514	49	377	178	279	15
Solyc12g005990.1.1	6	33	1	25	8	57	87	60	7	31	12	37	1
Solyc12g006000.1.1	0	0	0	0	0	0	0	0	0	0	0	0	0
Solyc12g006010.1.1	1	14	1	9	2	14	6	17	2	7	5	9	0
Solyc12g006020.1.1	5	40	17	46	0	8	7	11	0	0	1	13	0
Solyc12g006030.1.1	2	15	0	20	2	42	19	23	1	29	9	38	1
Solyc12g006040.1.1	0	0	0	0	0	0	0	0	0	0	0	0	0
Solyc12g006050.1.1	10	61	2	34	0	456	366	543	10	9	1	0	0
Solyc12g006060.1.1	2	2	0	1	0	13	4	19	0	3	6	5	0
Solyc12g006120.1.1	2	12	0	4	0	29	22	15	1	15	6	8	2
Solyc12g006140.1.1	8	2252	365	765	9	3	26	6	0	20	251	7	2
Solyc12g006160.1.1	1	10	3	7	0	9	16	16	2	30	21	46	5
Solyc12g006170.1.1	8	63	6	14	7	86	93	94	15	96	29	73	5
Solyc12g006180.1.1	5	78	5	30	1	102	35	141	5	81	12	82	1
Solyc12g006200.1.1	0	0	0	0	0	0	0	0	0	0	0	0	0
Solyc12g006210.1.1	0	0	0	0	0	0	0	0	0	0	0	0	0
Solyc12g006220.1.1	0	0	0	0	0	0	0	0	0	0	0	0	0
Solyc12g006230.1.1	4	3	1	3	0	96	66	149	6	76	9	36	0
Solyc12g006240.1.1	1	0	0	0	0	3	27	6	6	2	0	0	0
Solyc12g006250.1.1	1	0	0	3	1	3	15	8	1	9	2	7	0

Solyc12g006260.1.1	0	0	0	0	0	0	0	0	0	0	0	0	0
Solyc12g006270.1.1	0	0	0	0	0	0	0	0	0	0	0	0	0
Solyc12g006280.1.1	0	0	0	0	0	0	0	0	0	0	0	0	0
Solyc12g006290.1.1	8	157	1	90	1	168	38	157	4	271	8	187	0
Solyc12g006300.1.1	0	0	0	0	0	0	0	0	0	0	0	0	0
Solyc12g006310.1.1	1	1	0	3	0	3	8	1	4	4	1	2	4
Solyc12g006320.1.1	19	590	81	462	27	863	1651	1268	44	1583	150	1650	9
Solyc12g006330.1.1	0	0	0	0	0	0	0	0	0	0	0	0	0
Solyc12g006340.1.1	0	0	0	0	0	0	0	0	0	0	0	0	0
Solyc12g006350.1.1	3	5	0	12	0	11	15	25	12	27	7	6	0
Solyc12g006370.1.1	9	148	18	94	9	92	25	87	5	52	16	41	3
Solyc12g006380.1.1	0	0	0	0	0	0	0	0	0	0	0	0	0
Solyc12g006420.1.1	15	108	21	72	15	206	177	136	26	33	48	102	6
Solyc12g006430.1.1	0	0	0	0	0	0	0	0	0	0	0	0	0
Solyc12g006440.1.1	0	0	0	0	0	0	0	0	0	0	0	0	0
Solyc12g006450.1.1	6	52	5	49	0	70	159	101	4	284	25	193	0
Solyc12g006460.1.1	13	746	70	265	0	138	41	205	0	103	143	50	0
Solyc12g006470.1.1	21	5623	744	2307	29	280	323	545	2	1107	854	657	20
Solyc12g006480.1.1	18	32	7	39	13	90	74	229	10	164	34	140	2
Solyc12g006490.1.1	0	0	0	0	0	0	0	0	0	0	0	0	0
Solyc12g006500.1.1	0	0	0	0	0	0	0	0	0	0	0	0	0
Solyc12g006510.1.1	0	0	0	0	0	0	0	0	0	0	0	0	0
Solyc12g006520.1.1	0	0	0	0	0	0	0	0	0	0	0	0	0
Solyc12g006530.1.1	0	0	0	0	0	0	0	0	0	0	0	0	0
Solyc12g006540.1.1	5	188	8	77	5	479	315	505	81	328	66	226	3
Solyc12g006550.1.1	5	30	4	50	4	91	25	114	10	156	29	126	3
Solyc12g006570.1.1	0	0	0	0	0	0	0	0	0	0	0	0	0
Solyc12g006590.1.1	4	6	7	7	0	26	25	34	0	2	1	2	0
Solyc12g006600.1.1	0	0	0	0	0	0	0	0	0	0	0	0	0
Solyc12g006620.1.1	0	0	0	0	0	0	0	0	0	0	0	0	0
Solyc12g006630.1.1	2	10	5	2	0	0	0	3	0	2	0	0	0
Solyc12g006650.1.1	5	16	1	9	2	73	30	84	2	24	9	15	0
Solyc12g006660.1.1	0	0	0	0	0	0	0	0	0	0	0	0	0
Solyc12g006670.1.1	1	7	0	0	0	9	6	4	2	7	1	10	0
Solyc12g006680.1.1	0	0	0	0	0	0	0	0	0	0	0	0	0
Solyc12g006690.1.1	0	0	0	0	0	0	0	0	0	0	0	0	0
Solyc12g006700.1.1	14	143	28	92	13	275	254	235	41	344	51	246	14
Solyc12g006710.1.1	2	7	2	1	0	34	11	3	4	14	1	12	0
Solyc12g006760.1.1	0	0	0	0	0	0	0	0	0	0	0	0	0
Solyc12g006770.1.1	0	0	0	0	0	0	0	0	0	0	0	0	0
Solyc12g006790.1.1	0	0	0	0	0	0	0	0	0	0	0	0	0

Solyc12g006800.1.1	1	2	0	4	0	2	1	13	0	16	6	3	0
Solyc12g006810.1.1	4	48	24	21	0	10	81	26	3	7	41	3	1
Solyc12g006830.1.1	2	22	2	8	0	7	12	17	1	24	3	20	0
Solyc12g006840.1.1	0	0	0	0	0	0	0	0	0	0	0	0	0
Solyc12g006850.1.1	5	20	1	8	1	42	36	13	18	3	3	4	1
Solyc12g006860.1.1	0	0	0	0	0	0	0	0	0	0	0	0	0
Solyc12g006870.1.1	4	27	6	13	0	31	60	53	7	74	20	35	1
Solyc12g006880.1.1	1	3	0	5	0	6	1	7	0	4	0	12	0
Solyc12g006900.1.1	12	30	6	16	8	162	67	131	17	108	30	66	0
Solyc12g006910.1.1	0	0	0	0	0	0	0	0	0	0	0	0	0
Solyc12g006920.1.1	7	89	6	56	8	195	85	184	14	97	5	29	2
Solyc12g006930.1.1	6	322	29	144	30	260	169	219	121	165	17	66	19
Solyc12g006940.1.1	4	24	4	18	6	14	18	41	7	58	5	68	0
Solyc12g006970.1.1	0	0	0	0	0	0	0	0	0	0	0	0	0
Solyc12g006980.1.1	24	1580	88	772	93	180	121	332	29	1399	414	1740	21
Solyc12g006990.1.1	16	201	23	175	16	343	198	472	43	207	52	235	6
Solyc12g007000.1.1	5	56	0	29	1	79	62	137	11	62	10	144	1
Solyc12g007010.1.1	11	544	35	348	3	79	141	127	3	266	58	386	2
Solyc12g007020.1.1	0	0	0	0	0	0	0	0	0	0	0	0	0
Solyc12g007030.1.1	5	76	2	36	0	137	27	93	1	16	27	3	1
Solyc12g007050.1.1	2	23	12	17	0	8	1	15	0	2	0	0	0
Solyc12g007060.1.1	0	0	0	0	0	0	0	0	0	0	0	0	0
Solyc12g007070.1.1	3	31	1	22	0	6	28	18	20	103	17	56	0
Solyc12g007090.1.1	9	80	12	67	1	69	83	126	1	84	4	71	0
Solyc12g007100.1.1	6	60	3	18	0	41	15	22	2	58	24	55	5
Solyc12g007110.1.1	10	272	14	134	10	378	70	123	11	240	50	117	1
Solyc12g007120.1.1	5	29	3	14	2	30	59	55	4	14	12	12	0
Solyc12g007130.1.1	0	0	0	0	0	0	0	0	0	0	0	0	0
Solyc12g007140.1.1	0	0	0	0	0	0	0	0	0	0	0	0	0
Solyc12g007150.1.1	0	0	0	0	0	0	0	0	0	0	0	0	0
Solyc12g007170.1.1	16	144	15	133	12	822	463	1205	54	233	33	276	12
Solyc12g007180.1.1	0	0	0	0	0	0	0	0	0	0	0	0	0
Solyc12g007190.1.1	0	0	0	0	0	0	0	0	0	0	0	0	0
Solyc12g007200.1.1	5	32	0	20	1	54	18	43	2	118	20	79	0
Solyc12g007220.1.1	0	0	0	0	0	0	0	0	0	0	0	0	0
Solyc12g007230.1.1	3	47	2	34	2	18	8	37	1	48	2	71	0
Solyc12g007240.1.1	0	0	0	0	0	0	0	0	0	0	0	0	0
Solyc12g007280.1.1	6	30	13	15	6	59	70	77	8	81	22	76	0
Solyc12g007290.1.1	0	0	0	0	0	0	0	0	0	0	0	0	0
Solyc12g007300.1.1	0	0	0	0	0	0	0	0	0	0	0	0	0
Solyc12g007310.1.1	12	333	27	349	7	536	315	638	8	320	75	359	8

Solyc12g007320.1.1	0	0	0	0	0	0	0	0	0	0	0	0	0
Solyc12g008320.1.1	0	0	0	0	0	0	0	0	0	0	0	0	0
Solyc12g008340.1.1	5	10	0	5	6	11	31	33	3	22	21	24	1
Solyc12g008350.1.1	3	285	3	222	0	11	3	50	2	303	9	267	0
Solyc12g008360.1.1	12	452	42	272	33	1395	457	1290	92	327	198	376	10
Solyc12g008370.1.1	8	61	10	103	4	155	94	153	10	198	43	137	1
Solyc12g008380.1.1	1	0	1	0	0	6	7	0	1	0	0	3	0
Solyc12g008390.1.1	13	121	11	115	19	397	178	318	45	213	31	220	2
Solyc12g008400.1.1	0	0	0	0	0	0	0	0	0	0	0	0	0
Solyc12g008410.1.1	2	3	0	0	2	0	11	10	1	14	0	13	0
Solyc12g008420.1.1	4	20	0	16	1	29	41	26	7	99	7	76	1
Solyc12g008430.1.1	0	0	0	0	0	0	0	0	0	0	0	0	0
Solyc12g008450.1.1	0	0	0	0	0	0	0	0	0	0	0	0	0
Solyc12g008460.1.1	2	16	2	1	0	7	14	14	0	26	1	25	0
Solyc12g008470.1.1	1	16	2	0	0	0	4	3	2	13	11	5	0
Solyc12g008490.1.1	3	18	0	1	3	2	16	7	0	17	30	20	0
Solyc12g008500.1.1	16	37	39	53	15	483	213	244	31	36	18	16	1
Solyc12g008510.1.1	1	7	0	10	0	3	0	18	0	0	5	2	0
Solyc12g008520.1.1	0	0	0	0	0	0	0	0	0	0	0	0	0
Solyc12g008530.1.1	0	0	0	0	0	0	0	0	0	0	0	0	0
Solyc12g008540.1.1	0	0	0	0	0	0	0	0	0	0	0	0	0
Solyc12g008550.1.1	3	8	1	4	0	22	15	14	1	27	1	9	0
Solyc12g008560.1.1	1	70	8	23	6	22	344	37	2	346	6	46	1
Solyc12g008570.1.1	10	99	8	58	1	252	102	282	18	143	20	180	0
Solyc12g008580.1.1	2	10	1	6	0	2	1	6	0	23	8	9	2
Solyc12g008590.1.1	5	275	7	56	5	86	72	117	5	343	97	215	1
Solyc12g008600.1.1	0	0	0	0	0	0	0	0	0	0	0	0	0
Solyc12g008610.1.1	0	0	0	0	0	0	0	0	0	0	0	0	0
Solyc12g008620.1.1	3	7	1	11	9	34	25	36	2	24	1	30	0
Solyc12g008630.1.1	12	223	47	199	33	722	334	946	106	724	151	843	23
Solyc12g008640.1.1	13	171	24	139	9	178	170	451	27	692	157	403	8
Solyc12g008650.1.1	10	17	0	16	6	0	117	8	4	363	0	64	1
Solyc12g008660.1.1	5	10	0	29	1	135	23	111	5	60	1	25	0
Solyc12g008670.1.1	0	0	0	0	0	0	0	0	0	0	0	0	0
Solyc12g008680.1.1	0	0	0	0	0	0	0	0	0	0	0	0	0
Solyc12g008690.1.1	6	112	6	50	2	254	38	190	5	201	18	146	0
Solyc12g008700.1.1	2	32	2	13	0	51	28	84	6	73	19	38	5
Solyc12g008710.1.1	12	96	14	61	7	405	101	330	22	203	44	158	6
Solyc12g008720.1.1	5	136	8	74	14	346	134	220	34	163	200	339	9
Solyc12g008730.1.1	1	32	4	17	4	30	14	30	2	30	5	10	0
Solyc12g008740.1.1	0	0	0	0	0	0	0	0	0	0	0	0	0

Solyc12g008750.1.1	9	69	4	70	8	202	154	280	24	164	48	148	5
Solyc12g008760.1.1	3	32	1	6	0	53	18	63	8	70	4	29	1
Solyc12g008770.1.1	9	39	1	19	0	23	37	44	12	50	3	71	0
Solyc12g008780.1.1	5	31	4	9	0	39	31	46	4	63	25	30	1
Solyc12g008790.1.1	1	7	2	6	0	0	1	5	2	1	2	4	4
Solyc12g008800.1.1	0	0	0	0	0	0	0	0	0	0	0	0	0
Solyc12g008810.1.1	2	7	4	3	0	36	36	28	10	20	17	22	2
Solyc12g008820.1.1	1	1	2	1	0	17	12	10	5	13	3	3	0
Solyc12g008830.1.1	1	3	0	0	0	3	7	11	0	0	0	0	0
Solyc12g008840.1.1	12	28	8	26	11	387	981	416	130	21	7	6	0
Solyc12g008860.1.1	1	53	1	58	0	222	30	159	3	301	20	314	5
Solyc12g008870.1.1	4	9	0	10	0	2	9	21	0	25	8	24	0
Solyc12g008890.1.1	0	0	0	0	0	0	0	0	0	0	0	0	0
Solyc12g008900.1.1	0	0	0	0	0	0	0	0	0	0	0	0	0
Solyc12g008910.1.1	2	6	0	3	0	13	4	5	2	13	6	34	1
Solyc12g008940.1.1	5	102	7	54	2	143	131	169	17	128	43	129	11
Solyc12g008950.1.1	0	0	0	0	0	0	0	0	0	0	0	0	0
Solyc12g008960.1.1	2	2	2	0	0	3	6	5	0	2	0	1	1
Solyc12g008970.1.1	2	18	0	22	0	76	5	112	1	8	12	20	0
Solyc12g008980.1.1	11	153	25	51	0	9	176	49	2	15	64	26	1
Solyc12g008990.1.1	0	0	0	0	0	0	0	0	0	0	0	0	0
Solyc12g009000.1.1	6	110	38	76	11	15	2	0	2	12	3	130	3
Solyc12g009010.1.1	0	0	0	0	0	0	0	0	0	0	0	0	0
Solyc12g009020.1.1	1	24	1	14	0	89	7	32	1	27	0	18	0
Solyc12g009030.1.1	4	43	1	11	0	39	22	43	1	64	22	29	3
Solyc12g009040.1.1	0	0	0	0	0	0	0	0	0	0	0	0	0
Solyc12g009050.1.1	0	0	0	0	0	0	0	0	0	0	0	0	0
Solyc12g009060.1.1	0	0	0	0	0	0	0	0	0	0	0	0	0
Solyc12g009070.1.1	4	265	44	132	0	1	32	3	0	13	13	10	0
Solyc12g009080.1.1	4	8	3	8	0	34	53	10	2	55	13	38	1
Solyc12g009090.1.1	6	24	0	13	0	40	26	46	4	29	11	48	1
Solyc12g009100.1.1	1	6	0	4	0	20	15	6	0	1	2	8	0
Solyc12g009110.1.1	0	0	0	0	0	0	0	0	0	0	0	0	0
Solyc12g009120.1.1	3	42	1	21	0	36	28	34	7	50	10	39	0
Solyc12g009130.1.1	0	0	0	0	0	0	0	0	0	0	0	0	0
Solyc12g009140.1.1	10	316	22	210	21	1293	325	1173	94	531	83	492	12
Solyc12g009150.1.1	7	52	2	35	0	138	73	133	14	128	26	97	0
Solyc12g009160.1.1	0	0	0	0	0	0	0	0	0	0	0	0	0
Solyc12g009170.1.1	0	0	0	0	0	0	0	0	0	0	0	0	0
Solyc12g009180.1.1	5	98	5	32	4	54	148	96	33	255	45	252	15
Solyc12g009190.1.1	0	0	0	0	0	0	0	0	0	0	0	0	0

Solyc12g009200.1.1	5	393	105	131	0	1	150	27	0	6	7	9	0
Solyc12g009210.1.1	6	15	4	3	0	23	67	28	5	35	13	29	0
Solyc12g009220.1.1	3	75	11	72	15	66	8	25	1	0	1	10	5
Solyc12g009230.1.1	4	14	4	9	0	7	9	24	2	36	8	24	1
Solyc12g009240.1.1	1	66	9	56	0	0	0	0	0	24	2	15	4
Solyc12g009250.1.1	8	234	48	140	1	151	273	247	22	331	111	350	11
Solyc12g009260.1.1	3	0	3	1	13	0	9	2	4	19	0	5	2
Solyc12g009270.1.1	2	30	0	22	0	0	0	6	1	276	21	302	6
Solyc12g009300.1.1	17	13	2	30	8	211	100	147	27	7157	292	4901	63
Solyc12g009310.1.1	0	0	0	0	0	0	0	0	0	0	0	0	0
Solyc12g009340.1.1	1	4	0	5	0	11	21	31	4	5	0	18	0
Solyc12g009380.1.1	1	3	2	0	0	5	4	9	1	0	2	0	0
Solyc12g009390.1.1	4	4	0	4	0	7	11	9	2	10	4	0	0
Solyc12g009400.1.1	6	62	6	23	2	30	18	82	8	104	32	71	1
Solyc12g009410.1.1	7	94	7	68	21	170	90	161	36	193	65	154	7
Solyc12g009420.1.1	1	0	0	0	0	0	0	0	0	8	4	5	0
Solyc12g009430.1.1	2	9	1	6	0	19	11	18	2	42	5	21	0
Solyc12g009440.1.1	6	541	8	270	2	151	151	260	2	267	2	161	4
Solyc12g009450.1.1	0	0	0	0	0	0	0	0	0	0	0	0	0
Solyc12g009460.1.1	0	0	0	0	0	0	0	0	0	0	0	0	0
Solyc12g009470.1.1	12	420	49	280	6	625	831	796	30	216	50	208	3
Solyc12g009480.1.1	0	0	0	0	0	0	0	0	0	0	0	0	0
Solyc12g009500.1.1	5	31	5	10	3	54	80	56	6	46	16	57	0
Solyc12g009510.1.1	0	0	0	0	0	0	0	0	0	0	0	0	0
Solyc12g009520.1.1	0	0	0	0	0	0	0	0	0	0	0	0	0
Solyc12g009530.1.1	0	0	0	0	0	0	0	0	0	0	0	0	0
Solyc12g009550.1.1	0	0	0	0	0	0	0	0	0	0	0	0	0
Solyc12g009560.1.1	12	100	18	109	10	809	410	309	62	364	125	158	1
Solyc12g009570.1.1	5	40	6	12	10	37	40	14	11	6	6	10	0
Solyc12g009580.1.1	0	0	0	0	0	0	0	0	0	0	0	0	0
Solyc12g009590.1.1	4	10	0	3	0	15	14	24	9	14	11	14	0
Solyc12g009600.1.1	3	67	10	38	2	0	15	3	1	11	3	4	0
Solyc12g009610.1.1	11	62	8	33	3	80	95	125	41	243	40	289	7
Solyc12g009620.1.1	1	10	0	9	0	10	7	9	0	6	2	10	0
Solyc12g009630.1.1	1	15	0	10	0	5	5	12	1	4	0	1	0
Solyc12g009640.1.1	7	44	4	46	7	39	46	86	7	43	14	49	0
Solyc12g009650.1.1	3	931	17	204	0	7	7	13	2	5	6	2	0
Solyc12g009680.1.1	5	13	0	0	2	49	46	28	67	11	1	0	0
Solyc12g009700.1.1	0	0	0	0	0	0	0	0	0	0	0	0	0
Solyc12g009770.1.1	0	0	0	0	0	0	0	0	0	0	0	0	0
Solyc12g009780.1.1	0	0	0	0	0	0	0	0	0	0	0	0	0

Solyc12g009790.1.1	6	57	1	18	1	47	28	84	5	59	1	38	0
Solyc12g009800.1.1	3	161	9	140	1	53	164	164	0	477	0	59	0
Solyc12g009810.1.1	1	9	0	2	1	11	3	2	0	24	4	25	4
Solyc12g009820.1.1	1	2	0	0	0	8	2	0	0	13	0	10	0
Solyc12g009830.1.1	2	6	1	13	0	29	10	77	5	45	6	53	5
Solyc12g009840.1.1	0	0	0	0	0	0	0	0	0	0	0	0	0
Solyc12g009860.1.1	6	140	3	58	3	171	41	192	8	127	27	55	0
Solyc12g009880.1.1	2	6	1	18	0	31	36	34	0	24	0	36	0
Solyc12g009890.1.1	12	58	7	29	3	76	107	88	6	104	57	93	0
Solyc12g009920.1.1	1	2	0	3	0	1	7	11	0	2	0	3	0
Solyc12g009930.1.1	16	1473	44	367	6	363	22	657	1	467	89	137	0
Solyc12g009940.1.1	1	2	0	3	0	2	0	5	1	11	2	3	0
Solyc12g009960.1.1	27	426	37	348	16	697	362	928	63	1425	228	1267	5
Solyc12g009970.1.1	4	14	1	4	0	10	11	22	4	12	4	16	0
Solyc12g009980.1.1	1	38	4	18	0	15	20	41	0	24	3	16	0
Solyc12g009990.1.1	14	205	49	99	11	388	262	315	71	298	134	184	9
Solyc12g010010.1.1	12	151	17	140	14	187	193	286	53	318	40	256	9
Solyc12g010020.1.1	1	2	0	2	0	242	16	203	0	0	0	0	0
Solyc12g010030.1.1	0	0	0	0	0	0	0	0	0	0	0	0	0
Solyc12g010040.1.1	6	194	10	174	1	695	237	799	18	488	91	352	8
Solyc12g010050.1.1	1	1	2	0	2	2	4	0	1	12	1	5	5
Solyc12g010060.1.1	1	438	62	470	12	325	1094	817	333	481	136	508	4
Solyc12g010110.1.1	15	15	5	5	0	77	50	76	26	42	22	28	1
Solyc12g010120.1.1	0	0	0	0	0	0	0	0	0	0	0	0	0
Solyc12g010130.1.1	9	302	53	263	1	2324	1716	767	33	256	351	96	2
Solyc12g010150.1.1	0	0	0	0	0	0	0	0	0	0	0	0	0
Solyc12g010160.1.1	0	0	0	0	0	0	0	0	0	0	0	0	0
Solyc12g010170.1.1	0	0	0	0	0	0	0	0	0	0	0	0	0
Solyc12g010180.1.1	4	52	4	20	0	46	17	78	6	107	12	116	1
Solyc12g010200.1.1	3	0	0	0	1	11	4	4	3	0	4	0	4
Solyc12g010210.1.1	0	0	0	0	0	0	0	0	0	0	0	0	0
Solyc12g010220.1.1	0	0	0	0	0	0	0	0	0	0	0	0	0
Solyc12g010230.1.1	2	27	1	18	0	3	11	3	0	3	1	14	0
Solyc12g010250.1.1	0	0	0	0	0	0	0	0	0	0	0	0	0
Solyc12g010260.1.1	0	0	0	0	0	0	0	0	0	0	0	0	0
Solyc12g010270.1.1	0	0	0	0	0	0	0	0	0	0	0	0	0
Solyc12g010280.1.1	0	0	0	0	0	0	0	0	0	0	0	0	0
Solyc12g010290.1.1	0	0	0	0	0	0	0	0	0	0	0	0	0
Solyc12g010300.1.1	0	0	0	0	0	0	0	0	0	0	0	0	0
Solyc12g010310.1.1	0	0	0	0	0	0	0	0	0	0	0	0	0
Solyc12g010320.1.1	2	44	1	15	1	16	28	48	4	160	141	120	3

Solyc12g010330.1.1	9	54	2	20	0	43	67	106	10	69	10	49	4
Solyc12g010340.1.1	1	0	0	0	0	5	3	2	1	6	3	1	0
Solyc12g010350.1.1	2	21	2	4	3	15	22	24	8	48	21	22	0
Solyc12g010360.1.1	0	0	0	0	0	0	0	0	0	0	0	0	0
Solyc12g010380.1.1	10	179	65	121	0	23	52	43	5	140	12	159	0
Solyc12g010390.1.1	16	411	25	113	32	271	142	311	36	404	137	373	10
Solyc12g010400.1.1	0	0	0	0	0	0	0	0	0	0	0	0	0
Solyc12g010410.1.1	3	0	0	14	0	34	7	37	2	6	2	0	0
Solyc12g010420.1.1	0	0	0	0	0	0	0	0	0	0	0	0	0
Solyc12g010430.1.1	6	44	3	43	0	87	78	72	14	64	14	44	0
Solyc12g010440.1.1	4	171	16	157	0	64	35	78	14	9	9	29	0
Solyc12g010450.1.1	1	3	0	0	0	5	1	6	0	0	0	0	0
Solyc12g010480.1.1	0	0	0	0	0	0	0	0	0	0	0	0	0
Solyc12g010500.1.1	0	0	0	0	0	0	0	0	0	0	0	0	0
Solyc12g010520.1.1	7	32	5	21	4	112	33	64	16	86	41	108	0
Solyc12g010540.1.1	9	357	31	136	8	32	5	12	1	199	4	214	7
Solyc12g010570.1.1	0	0	0	0	0	0	0	0	0	0	0	0	0
Solyc12g010590.1.1	2	13	0	0	10	5	29	0	47	1	0	0	7
Solyc12g010640.1.1	8	75	4	46	6	154	34	126	15	128	16	111	1
Solyc12g010650.1.1	0	0	0	0	0	0	0	0	0	0	0	0	0
Solyc12g010670.1.1	2	0	0	1	0	12	14	10	1	0	0	1	0
Solyc12g010680.1.1	9	20	5	13	2	39	31	51	3	24	16	22	6
Solyc12g010690.1.1	3	8	9	7	0	17	18	13	4	7	6	6	0
Solyc12g010710.1.1	0	0	0	0	0	0	0	0	0	0	0	0	0
Solyc12g010720.1.1	0	0	0	0	0	0	0	0	0	0	0	0	0
Solyc12g010730.1.1	2	29	0	11	0	22	6	26	1	40	5	30	0
Solyc12g010740.1.1	9	12	2	14	0	66	55	69	3	630	30	308	0
Solyc12g010750.1.1	0	0	0	0	0	0	0	0	0	0	0	0	0
Solyc12g010790.1.1	2	25	2	19	3	14	12	17	1	43	3	29	3
Solyc12g010800.1.1	0	0	0	0	0	0	0	0	0	0	0	0	0
Solyc12g010820.1.1	2	0	0	7	0	127	11	8	1	2	9	0	0
Solyc12g010830.1.1	11	45	7	15	2	137	120	148	26	53	10	45	1
Solyc12g010840.1.1	18	292	32	192	1	215	201	373	10	271	64	556	7
Solyc12g010850.1.1	0	0	0	0	0	0	0	0	0	0	0	0	0
Solyc12g010860.1.1	8	171	12	107	4	245	95	324	16	337	35	300	5
Solyc12g010870.1.1	2	46	2	37	0	53	9	50	0	16	5	12	0
Solyc12g010890.1.1	3	14	2	8	3	55	30	48	12	12	9	13	0
Solyc12g010900.1.1	9	19	5	29	4	43	87	109	4	34	9	31	1
Solyc12g010910.1.1	5	43	12	55	0	8	10	22	0	30	13	51	0
Solyc12g010920.1.1	6	2	0	0	0	116	248	46	3	1	207	2	0
Solyc12g010930.1.1	2	46	4	17	2	156	30	189	13	160	17	144	0

Solyc12g010940.1.1	0	0	0	0	0	0	0	0	0	0	0	0	0
Solyc12g010950.1.1	0	0	0	0	0	0	0	0	0	0	0	0	0
Solyc12g010960.1.1	0	0	0	0	0	0	0	0	0	0	0	0	0
Solyc12g010970.1.1	0	0	0	0	0	0	0	0	0	0	0	0	0
Solyc12g010980.1.1	0	0	0	0	0	0	0	0	0	0	0	0	0
Solyc12g011000.1.1	6	64	5	57	1	567	153	526	39	162	22	111	3
Solyc12g011010.1.1	6	129	8	1	47	4	14	0	11	7	8	1	0
Solyc12g011020.1.1	2	4	2	6	0	0	4	2	3	5	3	4	0
Solyc12g011030.1.1	6	101	3	21	7	3	1	3	0	5	35	9	2
Solyc12g011040.1.1	0	0	0	0	0	0	0	0	0	0	0	0	0
Solyc12g011050.1.1	12	78	8	70	3	215	115	285	27	160	38	126	6
Solyc12g011070.1.1	0	0	0	0	0	0	0	0	0	0	0	0	0
Solyc12g011100.1.1	0	0	0	0	0	0	0	0	0	0	0	0	0
Solyc12g011130.1.1	2	8	0	1	0	23	14	16	1	18	1	7	1
Solyc12g011140.1.1	0	0	0	0	0	0	0	0	0	0	0	0	0
Solyc12g011150.1.1	0	0	0	0	0	0	0	0	0	0	0	0	0
Solyc12g011160.1.1	12	59	8	63	10	220	93	344	18	103	56	81	4
Solyc12g011170.1.1	0	0	0	0	0	0	0	0	0	0	0	0	0
Solyc12g011180.1.1	4	20	0	3	0	32	18	28	1	31	1	30	4
Solyc12g011190.1.1	1	3	0	5	0	0	6	1	0	3	0	0	0
Solyc12g011200.1.1	0	0	0	0	0	0	0	0	0	0	0	0	0
Solyc12g011230.1.1	0	0	0	0	0	0	0	0	0	0	0	0	0
Solyc12g011270.1.1	2	10	0	1	3	23	13	41	3	27	7	22	4
Solyc12g011280.1.1	4	458	98	254	4	3	11	2	0	17	69	1	2
Solyc12g011290.1.1	0	0	0	0	0	0	0	0	0	0	0	0	0
Solyc12g011310.1.1	0	0	0	0	0	0	0	0	0	0	0	0	0
Solyc12g011320.1.1	2	0	1	8	0	7	2	4	0	32	8	16	2
Solyc12g011330.2.1	4	12	3	10	3	25	28	41	6	31	4	40	1
Solyc12g011340.1.1	3	48	2	32	3	230	30	91	3	87	22	95	0
Solyc12g011370.1.1	4	34	3	14	3	47	25	47	10	32	3	3	0
Solyc12g011380.1.1	0	0	0	0	0	0	0	0	0	0	0	0	0
Solyc12g011400.1.1	1	0	1	0	0	3	8	7	2	4	0	0	0
Solyc12g011410.1.1	0	0	0	0	0	0	0	0	0	0	0	0	0
Solyc12g011420.1.1	10	5	1	2	6	6	55	27	13	150	26	127	0
Solyc12g011430.1.1	0	0	0	0	0	0	0	0	0	0	0	0	0
Solyc12g011440.1.1	5	89	9	34	4	29	86	103	9	94	7	71	1
Solyc12g011450.1.1	6	9711	656	6697	10	92	40	80	2	703	496	513	1
Solyc12g013500.1.1	1	13	0	3	0	13	2	9	0	46	4	64	0
Solyc12g013510.1.1	0	0	0	0	0	0	0	0	0	0	0	0	0
Solyc12g013520.1.1	0	0	0	0	0	0	0	0	0	0	0	0	0
Solyc12g013530.1.1	5	47	0	34	0	43	33	47	1	130	17	150	1

Solyc12g013540.1.1	5	63	5	45	4	68	33	57	1	167	10	75	0
Solyc12g013550.1.1	2	17	1	14	0	21	5	29	1	67	15	52	2
Solyc12g013560.1.1	0	0	0	0	0	0	0	0	0	0	0	0	0
Solyc12g013570.1.1	0	0	0	0	0	0	0	0	0	0	0	0	0
Solyc12g013580.1.1	5	23	3	16	0	18	30	34	0	42	0	27	0
Solyc12g013590.1.1	0	0	0	0	0	0	0	0	0	0	0	0	0
Solyc12g013600.1.1	1	4	0	0	0	0	4	5	0	2	0	0	0
Solyc12g013610.1.1	0	0	0	0	0	0	0	0	0	0	0	0	0
Solyc12g013620.1.1	14	41	3	22	0	3321	896	1282	178	43	32	17	0
Solyc12g013650.1.1	0	0	0	0	0	0	0	0	0	0	0	0	0
Solyc12g013680.1.1	0	0	0	0	0	0	0	0	0	0	0	0	0
Solyc12g013690.1.1	7	12	19	7	0	26	101	63	2	125	21	97	0
Solyc12g013700.1.1	2	0	1	0	8	0	3	3	0	35	7	16	0
Solyc12g013710.1.1	13	653	170	492	2	1	37	7	2	96	108	22	14
Solyc12g013740.1.1	0	0	0	0	0	0	0	0	0	0	0	0	0
Solyc12g013750.1.1	0	0	0	0	0	0	0	0	0	0	0	0	0
Solyc12g013790.1.1	3	8	2	0	1	65	14	31	8	29	10	18	2
Solyc12g013800.1.1	5	46	3	24	17	66	41	67	8	49	13	31	1
Solyc12g013810.1.1	3	487	67	341	0	244	446	281	4	341	19	225	4
Solyc12g013820.1.1	0	0	0	0	0	0	0	0	0	0	0	0	0
Solyc12g013830.1.1	0	0	0	0	0	0	0	0	0	0	0	0	0
Solyc12g013840.1.1	9	84	15	61	4	29	36	87	2	52	21	39	0
Solyc12g013850.1.1	0	0	0	0	0	0	0	0	0	0	0	0	0
Solyc12g013860.1.1	0	0	0	0	0	0	0	0	0	0	0	0	0
Solyc12g013870.1.1	0	0	0	0	0	0	0	0	0	0	0	0	0
Solyc12g013880.1.1	2	6	3	12	0	3	8	3	1	7	0	9	0
Solyc12g013890.1.1	6	66	21	69	2	66	201	156	9	131	11	117	1
Solyc12g013900.1.1	16	784	45	477	85	2660	1185	2927	911	114	21	79	2
Solyc12g013910.1.1	5	14	0	9	1	92	130	66	26	26	19	23	1
Solyc12g013920.1.1	3	24	2	11	0	19	13	37	2	40	3	41	2
Solyc12g013950.1.1	9	37	1	18	4	110	100	124	9	57	31	70	1
Solyc12g013960.1.1	4	5	0	3	0	33	24	24	16	5	0	3	1
Solyc12g013970.1.1	0	0	0	0	0	0	0	0	0	0	0	0	0
Solyc12g013980.1.1	1	0	2	0	2	0	0	0	0	5	2	0	2
Solyc12g013990.1.1	3	14	2	9	0	10	4	32	1	26	6	18	0
Solyc12g014000.1.1	0	0	0	0	0	0	0	0	0	0	0	0	0
Solyc12g014010.1.1	2	5	0	7	0	17	9	26	0	0	0	0	0
Solyc12g014020.1.1	0	0	0	0	0	0	0	0	0	0	0	0	0
Solyc12g014030.1.1	0	0	0	0	0	0	0	0	0	0	0	0	0
Solyc12g014040.1.1	0	0	0	0	0	0	0	0	0	0	0	0	0
Solyc12g014050.1.1	5	20	5	27	4	15	126	52	19	60	35	112	4

Solyc12g014060.1.1	4	24	3	1	0	22	5	33	7	43	6	21	0
Solyc12g014070.1.1	17	84	8	64	9	173	127	310	12	135	8	148	6
Solyc12g014080.1.1	1	7	1	1	1	18	6	19	5	17	1	3	0
Solyc12g014090.1.1	0	0	0	0	0	0	0	0	0	0	0	0	0
Solyc12g014100.1.1	11	52	11	59	1	458	534	710	32	186	18	98	0
Solyc12g014110.1.1	0	0	0	0	0	0	0	0	0	0	0	0	0
Solyc12g014120.1.1	1	4	0	0	0	0	1	1	0	1	4	7	0
Solyc12g014130.1.1	2	14	4	0	0	7	12	10	0	3	1	0	0
Solyc12g014140.1.1	10	319	14	162	5	12	48	113	3	4	0	0	2
Solyc12g014150.1.1	2	9	8	12	0	6	40	19	0	29	10	9	0
Solyc12g014160.1.1	0	0	0	0	0	0	0	0	0	0	0	0	0
Solyc12g014170.1.1	13	57	3	57	2	109	73	196	24	65	34	91	0
Solyc12g014180.1.1	5	38	0	4	1	89	19	124	4	55	7	44	0
Solyc12g014190.1.1	4	16	2	12	0	24	27	29	0	26	1	32	0
Solyc12g014200.1.1	0	0	0	0	0	0	0	0	0	0	0	0	0
Solyc12g014210.1.1	5	113	4	70	1	265	37	249	7	150	10	108	0
Solyc12g014220.1.1	5	31	6	11	1	26	26	36	5	27	4	50	0
Solyc12g014230.1.1	11	54	2	22	7	202	53	209	38	110	24	64	11
Solyc12g014240.1.1	0	0	0	0	0	0	0	0	0	0	0	0	0
Solyc12g014250.1.1	16	391	62	283	13	97	85	255	11	476	130	431	14
Solyc12g014260.1.1	0	0	0	0	0	0	0	0	0	0	0	0	0
Solyc12g014270.1.1	2	6	2	8	0	18	4	3	0	8	6	7	5
Solyc12g014280.1.1	4	15	0	9	0	27	17	28	0	9	0	24	3
Solyc12g014290.1.1	3	37	3	18	0	62	51	76	7	190	26	126	4
Solyc12g014310.1.1	8	51	11	20	11	34	235	93	54	1	2	5	0
Solyc12g014320.1.1	0	0	0	0	0	0	0	0	0	0	0	0	0
Solyc12g014330.1.1	0	0	0	0	0	0	0	0	0	0	0	0	0
Solyc12g014350.1.1	5	13	3	30	0	47	123	70	20	50	5	39	0
Solyc12g014360.1.1	0	0	0	0	0	0	0	0	0	0	0	0	0
Solyc12g014370.1.1	0	0	0	0	0	0	0	0	0	0	0	0	0
Solyc12g014380.1.1	4	30	6	11	1	29	40	26	5	67	6	68	0
Solyc12g014390.1.1	3	61	3	11	0	16	14	30	0	85	0	46	4
Solyc12g014400.1.1	4	144	9	69	5	301	264	320	18	414	34	210	2
Solyc12g014410.1.1	5	39	6	37	0	72	25	65	3	51	10	69	4
Solyc12g014420.1.1	20	80	12	36	30	693	482	820	957	28	10	7	3
Solyc12g014430.1.1	4	2	1	10	0	25	25	40	16	5	2	9	5
Solyc12g014440.1.1	6	26	1	14	6	58	22	30	1	140	13	94	0
Solyc12g014450.1.1	3	47	1	10	2	52	60	3	34	0	0	0	0
Solyc12g014460.1.1	6	22	6	17	0	53	58	37	4	62	16	45	0
Solyc12g014470.1.1	2	6	2	0	0	2	5	6	2	10	5	13	0
Solyc12g014480.1.1	2	2	1	8	0	9	5	12	0	22	4	18	0

Solyc12g014490.1.1	11	28	13	24	10	21	105	32	17	107	45	111	3
Solyc12g014500.1.1	0	0	0	0	0	0	0	0	0	0	0	0	0
Solyc12g014530.1.1	1	1	0	0	0	1	3	0	1	7	2	15	0
Solyc12g014540.1.1	3	48	0	19	2	38	21	38	4	100	5	45	0
Solyc12g014560.1.1	0	0	0	0	0	0	0	0	0	0	0	0	0
Solyc12g014570.1.1	0	0	0	0	0	0	0	0	0	0	0	0	0
Solyc12g014580.1.1	0	0	0	0	0	0	0	0	0	0	0	0	0
Solyc12g014590.1.1	0	0	0	0	0	0	0	0	0	0	0	0	0
Solyc12g014600.1.1	5	47	5	19	5	55	9	74	6	78	19	18	0
Solyc12g014610.1.1	9	33	1	29	2	14	42	48	4	35	28	41	0
Solyc12g014630.1.1	1	4	0	1	0	0	0	2	0	293	4	38	0
Solyc12g015630.1.1	2	46	5	7	1	2	13	2	0	9	0	12	0
Solyc12g015640.1.1	3	8	0	2	0	18	11	30	3	14	2	16	0
Solyc12g015650.1.1	1	2	1	2	0	5	2	2	0	0	3	5	0
Solyc12g015660.1.1	1	1	0	0	0	9	5	5	0	1	0	1	0
Solyc12g015680.1.1	4	130	8	56	3	94	56	60	6	42	5	27	2
Solyc12g015690.1.1	3	21	3	16	13	0	14	2	0	122	13	119	0
Solyc12g015710.1.1	10	35	0	21	0	33	58	86	0	53	4	58	2
Solyc12g015730.1.1	0	0	0	0	0	0	0	0	0	0	0	0	0
Solyc12g015740.1.1	5	66	0	44	2	61	21	72	4	161	5	45	1
Solyc12g015770.1.1	12	63	35	27	52	120	132	202	12	76	17	56	2
Solyc12g015780.1.1	9	47	5	32	1	176	66	247	21	119	27	98	1
Solyc12g015800.1.1	2	15	1	7	0	6	4	7	0	0	2	0	0
Solyc12g015820.1.1	0	0	0	0	0	0	0	0	0	0	0	0	0
Solyc12g015830.1.1	0	0	0	0	0	0	0	0	0	0	0	0	0
Solyc12g015840.1.1	0	0	0	0	0	0	0	0	0	0	0	0	0
Solyc12g015850.1.1	0	0	0	0	0	0	0	0	0	0	0	0	0
Solyc12g015860.1.1	7	427	27	150	7	58	22	148	2	225	93	224	1
Solyc12g015870.1.1	1	22	0	1	0	5	3	9	6	12	1	9	0
Solyc12g015880.1.1	19	1156	133	628	69	1469	502	1627	166	986	818	1340	28
Solyc12g015910.1.1	5	83	4	34	1	96	46	74	5	70	13	61	0
Solyc12g015970.1.1	5	184	6	104	8	48	109	123	1	181	5	193	0
Solyc12g016190.1.1	5	23	1	2	5	29	45	23	4	30	16	18	5
Solyc12g016210.1.1	1	12	0	0	0	8	3	15	0	11	0	35	0
Solyc12g016220.1.1	0	0	0	0	0	0	0	0	0	0	0	0	0
Solyc12g017230.1.1	11	8	10	11	9	71	43	52	24	65	30	49	0
Solyc12g017240.1.1	9	229	67	104	30	4	1	4	1	36	31	219	3
Solyc12g017250.1.1	2	0	1	0	0	26	472	0	7	95	302	3	1
Solyc12g017260.1.1	17	81	3	155	4	316	134	263	34	249	50	171	0
Solyc12g017280.1.1	7	7	5	13	1	24	55	44	9	72	23	32	0
Solyc12g017360.1.1	0	0	0	0	0	0	0	0	0	0	0	0	0

Solyc12g017370.1.1	3	16	0	27	0	37	24	53	0	1	0	10	0
Solyc12g017380.1.1	0	0	0	0	0	0	0	0	0	0	0	0	0
Solyc12g017410.1.1	4	9	2	10	4	4	23	14	1	35	2	20	0
Solyc12g017420.1.1	8	48	3	32	0	25	59	77	2	73	12	81	0
Solyc12g017430.1.1	0	0	0	0	0	0	0	0	0	0	0	0	0
Solyc12g017440.1.1	1	1	0	2	0	2	6	2	0	4	0	6	0
Solyc12g017450.1.1	1	3	0	3	2	3	6	0	0	2	4	0	0
Solyc12g017460.1.1	5	77	15	20	0	68	12	144	0	180	15	33	0
Solyc12g017470.1.1	0	0	0	0	0	0	0	0	0	0	0	0	0
Solyc12g017480.1.1	0	0	0	0	0	0	0	0	0	0	0	0	0
Solyc12g017510.1.1	13	68	8	46	0	130	166	220	3	22	12	44	1
Solyc12g017530.1.1	1	0	0	0	0	27	11	4	0	4	1	0	0
Solyc12g017540.1.1	4	21	0	26	0	32	13	51	3	45	15	34	0
Solyc12g017570.1.1	1	9	1	6	0	8	4	11	0	11	1	22	2
Solyc12g017610.1.1	0	0	0	0	0	0	0	0	0	0	0	0	0
Solyc12g017630.1.1	3	12	5	7	4	17	18	20	2	24	5	33	0
Solyc12g017690.1.1	2	8	0	4	0	2	6	11	0	6	2	10	0
Solyc12g017830.1.1	10	67	4	53	8	193	89	323	36	210	17	169	0
Solyc12g017840.1.1	0	0	0	0	0	0	0	0	0	0	0	0	0
Solyc12g017850.1.1	3	5	0	1	0	21	26	27	0	27	6	30	0
Solyc12g017860.1.1	4	10	4	1	0	18	18	11	3	22	12	23	0
Solyc12g017880.1.1	0	0	0	0	0	0	0	0	0	0	0	0	0
Solyc12g017910.1.1	12	252	18	158	5	417	164	456	17	302	44	303	0
Solyc12g017920.1.1	0	0	0	0	0	0	0	0	0	0	0	0	0
Solyc12g017960.1.1	0	0	0	0	0	0	0	0	0	0	0	0	0
Solyc12g017970.1.1	0	0	0	0	0	0	0	0	0	0	0	0	0
Solyc12g018990.1.1	3	7	2	14	0	60	60	76	13	66	42	69	0
Solyc12g019000.1.1	6	34	3	19	0	67	53	157	9	42	15	28	0
Solyc12g019010.1.1	1	20	1	3	2	25	8	11	0	60	1	37	0
Solyc12g019060.1.1	8	160	10	158	2	158	87	234	7	224	16	223	0
Solyc12g019080.1.1	0	0	0	0	0	0	0	0	0	0	0	0	0
Solyc12g019100.1.1	2	2	0	8	1	2	10	9	3	12	1	1	0
Solyc12g019110.1.1	1	5	2	1	0	4	5	4	0	0	2	5	0
Solyc12g019210.1.1	0	0	0	0	0	0	0	0	0	0	0	0	0
Solyc12g019230.1.1	0	0	0	0	0	0	0	0	0	0	0	0	0
Solyc12g019290.1.1	8	36	1	15	1	178	16	191	7	88	21	94	1
Solyc12g019300.1.1	0	0	0	0	0	0	0	0	0	0	0	0	0
Solyc12g019310.1.1	1	3	0	0	0	5	5	17	0	5	2	3	0
Solyc12g019320.1.1	6	55	5	16	0	7	34	50	2	0	1	0	0
Solyc12g019410.1.1	3	15	2	6	15	8	22	42	16	5	4	0	0
Solyc12g019460.1.1	7	74	6	56	0	269	152	227	31	125	18	124	6

Solyc12g019470.1.1	5	57	3	30	3	205	38	85	3	69	10	51	0
Solyc12g019480.1.1	0	0	0	0	0	0	0	0	0	0	0	0	0
Solyc12g019490.1.1	0	0	0	0	0	0	0	0	0	0	0	0	0
Solyc12g019540.1.1	0	0	0	0	0	0	0	0	0	0	0	0	0
Solyc12g019550.1.1	0	0	0	0	0	0	0	0	0	0	0	0	0
Solyc12g019560.1.1	1	7	2	1	0	2	0	19	0	8	2	10	3
Solyc12g019570.1.1	4	9	0	11	0	13	11	17	1	7	2	3	0
Solyc12g019680.1.1	0	0	0	0	0	0	0	0	0	0	0	0	0
Solyc12g019690.1.1	2	7	0	6	0	17	11	13	1	16	5	6	10
Solyc12g019710.1.1	0	0	0	0	0	0	0	0	0	0	0	0	0
Solyc12g019740.1.1	10	134	16	108	4	729	380	866	29	340	78	325	3
Solyc12g019750.1.1	7	75	8	22	5	157	164	254	7	161	27	141	5
Solyc12g019860.1.1	5	66	12	61	12	215	104	210	18	156	47	140	1
Solyc12g019890.1.1	0	0	0	0	0	0	0	0	0	0	0	0	0
Solyc12g019960.1.1	6	30	2	18	1	54	32	147	11	79	18	132	7
Solyc12g019970.1.1	0	0	0	0	0	0	0	0	0	0	0	0	0
Solyc12g019980.1.1	3	31	2	29	0	66	33	63	1	58	21	43	0
Solyc12g019990.1.1	1	4	0	1	0	7	8	11	0	3	0	5	0
Solyc12g020000.1.1	10	110	8	94	7	174	152	250	27	124	70	113	4
Solyc12g020050.1.1	12	82	6	50	2	233	158	364	45	221	82	195	5
Solyc12g020110.1.1	1	1	0	0	0	5	0	3	0	3	7	7	0
Solyc12g020120.1.1	0	0	0	0	0	0	0	0	0	0	0	0	0
Solyc12g021130.1.1	8	35	3	11	4	33	45	93	4	148	25	171	2
Solyc12g021160.1.1	0	0	0	0	0	0	0	0	0	0	0	0	0
Solyc12g021170.1.1	6	12	5	9	5	46	136	76	9	35	21	35	4
Solyc12g021180.1.1	0	0	0	0	0	0	0	0	0	0	0	0	0
Solyc12g021190.1.1	0	0	0	0	0	0	0	0	0	0	0	0	0
Solyc12g021230.1.1	8	34	6	21	1	119	117	138	5	86	4	45	0
Solyc12g021280.1.1	4	115	46	89	9	32	87	59	4	61	20	32	0
Solyc12g021290.1.1	2	2	1	5	1	9	11	17	2	8	2	0	0
Solyc12g021310.1.1	1	5	0	0	0	2	5	5	1	0	1	4	2
Solyc12g021320.1.1	0	0	0	0	0	0	0	0	0	0	0	0	0
Solyc12g021330.1.1	0	0	0	0	0	0	0	0	0	0	0	0	0
Solyc12g021340.1.1	4	80	6	29	0	44	32	55	1	66	5	32	5
Solyc12g021360.1.1	4	83	16	42	2	5	58	28	2	57	23	68	1
Solyc12g026400.1.1	3	16	3	17	5	55	102	83	17	54	15	42	0
Solyc12g026420.1.1	0	0	0	0	0	0	0	0	0	0	0	0	0
Solyc12g026430.1.1	3	11	0	7	3	15	8	11	0	22	3	35	0
Solyc12g026470.1.1	0	0	0	0	0	0	0	0	0	0	0	0	0
Solyc12g027560.1.1	0	0	0	0	0	0	0	0	0	0	0	0	0
Solyc12g027580.1.1	2	6	5	5	2	5	12	16	0	4	2	9	3

Solyc12g027590.1.1	1	0	0	5	0	12	8	4	0	14	1	2	0
Solyc12g027600.1.1	1	0	0	1	0	6	13	5	3	2	0	0	0
Solyc12g027740.1.1	2	16	1	6	0	16	8	26	1	40	7	44	0
Solyc12g027750.1.1	1	2	0	0	0	3	4	3	0	13	2	2	0
Solyc12g027770.1.1	0	0	0	0	0	0	0	0	0	0	0	0	0
Solyc12g027780.1.1	0	0	0	0	0	0	0	0	0	0	0	0	0
Solyc12g027800.1.1	1	6	0	3	0	0	8	0	0	0	0	1	0
Solyc12g027850.1.1	1	1	2	7	0	6	3	4	2	9	1	0	0
Solyc12g027860.1.1	0	0	0	0	0	0	0	0	0	0	0	0	0
Solyc12g027870.1.1	2	6	2	6	2	48	14	33	2	28	3	13	0
Solyc12g027880.1.1	0	0	0	0	0	0	0	0	0	0	0	0	0
Solyc12g027890.1.1	0	0	0	0	0	0	0	0	0	0	0	0	0
Solyc12g032930.1.1	0	0	0	0	0	0	0	0	0	0	0	0	0
Solyc12g032950.1.1	9	132	41	87	0	499	484	752	49	238	71	205	6
Solyc12g035130.1.1	8	884	37	677	23	1203	355	951	42	3529	256	2664	4
Solyc12g035170.1.1	0	0	0	0	0	0	0	0	0	0	0	0	0
Solyc12g035190.1.1	0	0	0	0	0	0	0	0	0	0	0	0	0
Solyc12g035240.1.1	11	66	4	45	7	115	133	134	26	180	43	125	6
Solyc12g035250.1.1	4	10	2	13	0	18	22	36	7	18	4	12	1
Solyc12g035360.1.1	1	1	1	2	1	6	1	7	0	14	4	0	0
Solyc12g035400.1.1	0	0	0	0	0	0	0	0	0	0	0	0	0
Solyc12g035420.1.1	0	0	0	0	0	0	0	0	0	0	0	0	0
Solyc12g035470.1.1	0	0	0	0	0	0	0	0	0	0	0	0	0
Solyc12g035490.1.1	0	0	0	0	0	0	0	0	0	0	0	0	0
Solyc12g035500.1.1	2	2	1	0	0	1	9	5	1	15	4	9	0
Solyc12g035520.1.1	8	59	5	51	6	87	53	96	6	148	22	152	3
Solyc12g035650.1.1	1	2	0	3	0	2	4	14	2	13	0	14	0
Solyc12g035670.1.1	13	29	10	34	4	50	133	89	35	71	42	33	13
Solyc12g035710.1.1	0	0	0	0	0	0	0	0	0	0	0	0	0
Solyc12g035840.1.1	0	0	0	0	0	0	0	0	0	0	0	0	0
Solyc12g036120.1.1	1	2	0	3	0	0	5	1	0	4	0	0	0
Solyc12g036130.1.1	0	0	0	0	0	0	0	0	0	0	0	0	0
Solyc12g036140.1.1	0	0	0	0	0	0	0	0	0	0	0	0	0
Solyc12g036150.1.1	0	0	0	0	0	0	0	0	0	0	0	0	0
Solyc12g036160.1.1	2	23	5	17	0	2	6	15	0	16	4	23	0
Solyc12g036170.1.1	3	511	64	252	1	3	6	15	0	17	0	10	0
Solyc12g036200.1.1	2	11	0	5	0	9	10	24	0	41	2	79	0
Solyc12g036220.1.1	7	11	0	12	0	19	45	33	5	20	10	20	0
Solyc12g036250.1.1	2	7	0	3	0	13	7	12	0	15	0	6	3
Solyc12g036300.1.1	2	1	0	0	0	5	2	6	0	2	1	6	0
Solyc12g036320.1.1	0	0	0	0	0	0	0	0	0	0	0	0	0

Solyc12g036330.1.1	0	0	0	0	0	0	0	0	0	0	0	0	0
Solyc12g036340.1.1	0	0	0	0	0	0	0	0	0	0	0	0	0
Solyc12g036390.1.1	0	0	0	0	0	0	0	0	0	0	0	0	0
Solyc12g036400.1.1	0	0	0	0	0	0	0	0	0	0	0	0	0
Solyc12g036410.1.1	4	11	1	3	0	6	3	21	1	7	5	19	0
Solyc12g036420.1.1	0	0	0	0	0	0	0	0	0	0	0	0	0
Solyc12g036470.1.1	0	0	0	0	0	0	0	0	0	0	0	0	0
Solyc12g036480.1.1	0	0	0	0	0	0	0	0	0	0	0	0	0
Solyc12g036500.1.1	5	20	4	17	1	11	25	22	0	14	3	22	0
Solyc12g036650.1.1	4	43	10	23	8	4	21	7	6	8	20	13	3
Solyc12g036670.1.1	0	0	0	0	0	0	0	0	0	0	0	0	0
Solyc12g036710.1.1	0	0	0	0	0	0	0	0	0	0	0	0	0
Solyc12g036790.1.1	2	314	5	90	6	73	49	293	8	307	27	136	6
Solyc12g036800.1.1	0	0	0	0	0	0	0	0	0	0	0	0	0
Solyc12g036810.1.1	0	0	0	0	0	0	0	0	0	0	0	0	0
Solyc12g036850.1.1	0	0	0	0	0	0	0	0	0	0	0	0	0
Solyc12g037970.1.1	2	8	4	6	0	4	15	23	2	5	8	4	4
Solyc12g037980.1.1	3	20	6	13	5	95	108	85	23	66	44	71	1
Solyc12g038140.1.1	0	0	0	0	0	0	0	0	0	0	0	0	0
Solyc12g038160.1.1	10	34	4	13	3	74	44	58	6	244	45	141	6
Solyc12g038200.1.1	0	0	0	0	0	0	0	0	0	0	0	0	0
Solyc12g038340.1.1	5	25	1	23	0	138	28	75	5	58	12	60	0
Solyc12g038350.1.1	0	0	0	0	0	0	0	0	0	0	0	0	0
Solyc12g038360.1.1	0	0	0	0	0	0	0	0	0	0	0	0	0
Solyc12g038370.1.1	0	0	0	0	0	0	0	0	0	0	0	0	0
Solyc12g038400.1.1	8	117	9	30	8	269	315	281	21	172	40	97	8
Solyc12g038480.1.1	0	0	0	0	0	0	0	0	0	0	0	0	0
Solyc12g038490.1.1	16	46	9	42	5	113	115	209	11	181	34	83	0
Solyc12g038510.1.1	0	0	0	0	0	0	0	0	0	0	0	0	0
Solyc12g038520.1.1	0	0	0	0	0	0	0	0	0	0	0	0	0
Solyc12g038530.1.1	2	5	0	0	1	10	7	15	0	15	4	2	0
Solyc12g038540.1.1	0	0	0	0	0	0	0	0	0	0	0	0	0
Solyc12g038550.1.1	0	0	0	0	0	0	0	0	0	0	0	0	0
Solyc12g038560.1.1	0	0	0	0	0	0	0	0	0	0	0	0	0
Solyc12g038570.1.1	1	0	0	1	0	8	8	4	0	3	3	10	0
Solyc12g038580.1.1	0	0	0	0	0	0	0	0	0	0	0	0	0
Solyc12g038590.1.1	1	12	0	9	0	28	4	16	1	11	4	18	0
Solyc12g038600.1.1	0	0	0	0	0	0	0	0	0	0	0	0	0
Solyc12g038630.1.1	3	85	0	42	3	64	49	72	3	389	17	321	1
Solyc12g038670.1.1	10	48	0	37	7	25	41	50	6	90	26	75	6
Solyc12g038700.1.1	0	0	0	0	0	0	0	0	0	0	0	0	0

Solyc12g038800.1.1	0	0	0	0	0	0	0	0	0	0	0	0	0
Solyc12g038820.1.1	7	19	0	12	2	16	35	19	3	114	11	37	2
Solyc12g038920.1.1	1	4	0	1	0	10	4	14	0	14	1	6	0
Solyc12g038950.1.1	0	0	0	0	0	0	0	0	0	0	0	0	0
Solyc12g038970.1.1	11	129	16	66	12	98	95	261	22	703	62	347	2
Solyc12g038980.1.1	3	50	1	14	0	87	26	86	11	133	26	99	0
Solyc12g039010.1.1	0	0	0	0	0	0	0	0	0	0	0	0	0
Solyc12g039080.1.1	0	0	0	0	0	0	0	0	0	0	0	0	0
Solyc12g039120.1.1	1	182	11	86	0	138	21	141	10	376	40	312	5
Solyc12g039170.1.1	0	0	0	0	0	0	0	0	0	0	0	0	0
Solyc12g039180.1.1	4	30	13	24	8	92	162	91	75	135	22	92	2
Solyc12g040320.1.1	1	0	0	1	0	0	3	0	0	8	0	4	0
Solyc12g040390.1.1	0	0	0	0	0	0	0	0	0	0	0	0	0
Solyc12g040460.1.1	1	5	0	1	0	0	3	4	0	9	1	7	0
Solyc12g040470.1.1	0	0	0	0	0	0	0	0	0	0	0	0	0
Solyc12g040480.1.1	0	0	0	0	0	0	0	0	0	0	0	0	0
Solyc12g040500.1.1	1	3	0	1	0	3	5	5	0	8	1	3	0
Solyc12g040510.1.1	2	37	1	8	0	67	7	66	0	49	4	21	0
Solyc12g040640.1.1	0	0	0	0	0	0	0	0	0	0	0	0	0
Solyc12g040680.1.1	8	77	22	55	2	215	121	176	26	128	29	119	6
Solyc12g040690.1.1	0	0	0	0	0	0	0	0	0	0	0	0	0
Solyc12g040700.1.1	0	0	0	0	0	0	0	0	0	0	0	0	0
Solyc12g040740.1.1	0	0	0	0	0	0	0	0	0	0	0	0	0
Solyc12g040790.1.1	4	282	19	70	1	2	2	12	6	10	0	1	0
Solyc12g040800.1.1	2	5	2	6	0	0	15	1	0	34	8	15	0
Solyc12g040810.1.1	1	4	0	0	0	13	2	5	0	16	1	3	0
Solyc12g040860.1.1	0	0	0	0	0	0	0	0	0	0	0	0	0
Solyc12g040870.1.1	0	0	0	0	0	0	0	0	0	0	0	0	0
Solyc12g041880.1.1	6	90	3	48	0	48	32	68	3	83	8	108	0
Solyc12g041940.1.1	0	0	0	0	0	0	0	0	0	0	0	0	0
Solyc12g041950.1.1	0	0	0	0	0	0	0	0	0	0	0	0	0
Solyc12g041960.1.1	0	0	0	0	0	0	0	0	0	0	0	0	0
Solyc12g041980.1.1	0	0	0	0	0	0	0	0	0	0	0	0	0
Solyc12g041990.1.1	0	0	0	0	0	0	0	0	0	0	0	0	0
Solyc12g042000.1.1	0	0	0	0	0	0	0	0	0	0	0	0	0
Solyc12g042060.1.1	15	1605	181	2023	41	5233	2454	3863	96	1695	287	1399	5
Solyc12g042070.1.1	18	100	6	85	7	133	211	320	19	272	40	259	3
Solyc12g042080.1.1	2	64	3	5	0	79	7	120	6	286	17	152	1
Solyc12g042120.1.1	0	0	0	0	0	0	0	0	0	0	0	0	0
Solyc12g042170.1.1	3	7	2	5	0	5	14	0	0	18	6	13	0
Solyc12g042180.1.1	0	0	0	0	0	0	0	0	0	0	0	0	0

Solyc12g042200.1.1	6	7	2	5	0	25	48	42	6	41	9	32	5
Solyc12g042210.1.1	0	0	0	0	0	0	0	0	0	0	0	0	0
Solyc12g042230.1.1	0	0	0	0	0	0	0	0	0	0	0	0	0
Solyc12g042240.1.1	0	0	0	0	0	0	0	0	0	0	0	0	0
Solyc12g042260.1.1	0	0	0	0	0	0	0	0	0	0	0	0	0
Solyc12g042300.1.1	0	0	0	0	0	0	0	0	0	0	0	0	0
Solyc12g042360.1.1	2	34	9	12	9	16	18	35	11	49	9	38	0
Solyc12g042380.1.1	18	400	55	272	15	830	652	1054	163	707	225	787	26
Solyc12g042400.1.1	0	0	0	0	0	0	0	0	0	0	0	0	0
Solyc12g042430.1.1	4	16	0	19	1	28	19	12	2	41	2	13	0
Solyc12g042440.1.1	10	29	4	16	0	40	48	71	4	101	15	79	0
Solyc12g042460.1.1	4	6	9	5	0	46	60	46	6	2	2	0	0
Solyc12g042470.1.1	12	199	55	127	26	176	264	188	61	9	11	32	0
Solyc12g042480.1.1	0	0	0	0	0	0	0	0	0	0	0	0	0
Solyc12g042500.1.1	2	9	0	8	0	10	4	5	0	10	84	16	0
Solyc12g042520.1.1	0	0	0	0	0	0	0	0	0	0	0	0	0
Solyc12g042530.1.1	0	0	0	0	0	0	0	0	0	0	0	0	0
Solyc12g042540.1.1	2	15	3	3	0	26	18	22	3	16	11	18	0
Solyc12g042570.1.1	3	10	1	7	0	23	25	35	5	40	4	30	0
Solyc12g042580.1.1	0	0	0	0	0	0	0	0	0	0	0	0	0
Solyc12g042590.1.1	0	0	0	0	0	0	0	0	0	0	0	0	0
Solyc12g042600.1.1	10	80	9	73	4	33	117	47	1	1	1	0	0
Solyc12g042650.1.1	5	628	17	136	0	1748	120	1428	30	1939	108	991	8
Solyc12g042690.1.1	0	0	0	0	0	0	0	0	0	0	0	0	0
Solyc12g042700.1.1	0	0	0	0	0	0	0	0	0	0	0	0	0
Solyc12g042720.1.1	0	0	0	0	0	0	0	0	0	0	0	0	0
Solyc12g042730.1.1	0	0	0	0	0	0	0	0	0	0	0	0	0
Solyc12g042740.1.1	5	16	2	13	2	55	32	70	5	63	2	68	0
Solyc12g042750.1.1	0	0	0	0	0	0	0	0	0	0	0	0	0
Solyc12g042760.1.1	0	0	0	0	0	0	0	0	0	0	0	0	0
Solyc12g042770.1.1	11	1357	44	781	13	516	590	929	10	363	59	514	7
Solyc12g042780.1.1	0	0	0	0	0	0	0	0	0	0	0	0	0
Solyc12g042880.1.1	1	1	2	0	0	0	2	3	0	4	0	4	0
Solyc12g042890.1.1	1	0	0	0	0	14	6	5	0	0	0	1	0
Solyc12g042900.1.1	3	47	1	13	0	53	57	147	6	81	13	43	0
Solyc12g042910.1.1	4	5	2	6	4	23	6	8	5	15	16	21	0
Solyc12g042920.1.1	4	319	29	121	6	98	80	142	18	36	27	33	0
Solyc12g042930.1.1	0	0	0	0	0	0	0	0	0	0	0	0	0
Solyc12g042950.1.1	12	240	23	123	8	579	495	538	27	692	82	501	2
Solyc12g042960.1.1	0	0	0	0	0	0	0	0	0	0	0	0	0
Solyc12g042990.1.1	15	78	8	36	2	109	93	156	12	51	15	81	0

Solyc12g043000.1.1	5	35	4	49	4	146	45	150	5	147	15	128	3
Solyc12g043020.1.1	4	5	3	9	2	6	17	18	5	72	9	54	28
Solyc12g043030.1.1	0	0	0	0	0	0	0	0	0	0	0	0	0
Solyc12g043040.1.1	1	2	0	0	0	7	5	9	0	2	1	2	0
Solyc12g043050.1.1	0	0	0	0	0	0	0	0	0	0	0	0	0
Solyc12g043060.1.1	1	3	1	0	0	0	4	1	2	1	0	2	0
Solyc12g043070.1.1	0	0	0	0	0	0	0	0	0	0	0	0	0
Solyc12g043090.1.1	8	72	2	46	3	105	37	129	5	77	25	65	1
Solyc12g043100.1.1	0	0	0	0	0	0	0	0	0	0	0	0	0
Solyc12g043110.1.1	18	238	56	222	22	448	344	635	75	932	278	830	11
Solyc12g043120.1.1	9	109	2	78	5	416	35	355	6	167	36	180	2
Solyc12g043150.1.1	0	0	0	0	0	0	0	0	0	0	0	0	0
Solyc12g043160.1.1	0	0	0	0	0	0	0	0	0	0	0	0	0
Solyc12g044190.1.1	0	0	0	0	0	0	0	0	0	0	0	0	0
Solyc12g044200.1.1	0	0	0	0	0	0	0	0	0	0	0	0	0
Solyc12g044220.1.1	8	20	2	24	0	67	74	54	20	80	30	32	7
Solyc12g044230.1.1	4	9	1	6	1	58	39	94	23	7	1	26	0
Solyc12g044240.1.1	1	13	2	11	0	23	50	23	0	618	12	326	0
Solyc12g044250.1.1	0	0	0	0	0	0	0	0	0	0	0	0	0
Solyc12g044260.1.1	2	16	0	6	0	5	17	15	0	3	0	1	0
Solyc12g044270.1.1	0	0	0	0	0	0	0	0	0	0	0	0	0
Solyc12g044280.1.1	1	58	8	54	0	0	4	1	0	1	0	1	3
Solyc12g044290.1.1	5	97	3	33	28	81	36	129	32	198	25	85	14
Solyc12g044300.1.1	8	126	15	45	22	68	66	137	55	358	45	80	17
Solyc12g044310.1.1	1	10	0	6	0	0	4	2	0	6	0	0	0
Solyc12g044320.1.1	0	0	0	0	0	0	0	0	0	0	0	0	0
Solyc12g044330.1.1	3	136	26	47	0	4	69	6	0	17	2	4	0
Solyc12g044340.1.1	0	0	0	0	0	0	0	0	0	0	0	0	0
Solyc12g044350.1.1	0	0	0	0	0	0	0	0	0	0	0	0	0
Solyc12g044360.1.1	0	0	0	0	0	0	0	0	0	0	0	0	0
Solyc12g044370.1.1	1	1	0	1	1	9	4	5	0	5	0	8	0
Solyc12g044380.1.1	0	0	0	0	0	0	0	0	0	0	0	0	0
Solyc12g044390.1.1	0	0	0	0	0	0	0	0	0	0	0	0	0
Solyc12g044400.1.1	9	58	14	23	2	111	89	131	4	51	22	68	1
Solyc12g044410.1.1	6	48	10	62	6	32	56	50	0	23	12	27	0
Solyc12g044420.1.1	7	12	5	11	0	18	171	31	0	38	15	45	0
Solyc12g044440.1.1	0	0	0	0	0	0	0	0	0	0	0	0	0
Solyc12g044450.1.1	0	0	0	0	0	0	0	0	0	0	0	0	0
Solyc12g044490.1.1	0	0	0	0	0	0	0	0	0	0	0	0	0
Solyc12g044600.2.1	10	1370	91	1088	52	4023	508	2356	103	4016	475	2890	32
Solyc12g044610.1.1	2	8	0	1	0	2	7	6	0	2	0	2	0

Solyc12g044620.1.1	0	0	0	0	0	0	0	0	0	0	0	0	0
Solyc12g044630.1.1	0	0	0	0	0	0	0	0	0	0	0	0	0
Solyc12g044640.1.1	8	87	11	42	5	158	102	138	14	179	28	159	5
Solyc12g044700.1.1	8	13	10	3	8	16	61	35	24	20	33	21	1
Solyc12g044720.1.1	1	19	0	10	0	30	3	18	0	58	15	35	2
Solyc12g044730.1.1	0	0	0	0	0	0	0	0	0	0	0	0	0
Solyc12g044740.1.1	4	42	5	26	0	124	100	181	22	75	31	78	1
Solyc12g044800.1.1	6	36	2	31	0	80	75	81	10	103	11	106	4
Solyc12g044810.1.1	0	0	0	0	0	0	0	0	0	0	0	0	0
Solyc12g044820.1.1	3	18	0	0	0	3	10	6	0	18	2	23	0
Solyc12g044840.1.1	0	0	0	0	0	0	0	0	0	0	0	0	0
Solyc12g044850.1.1	3	309	14	584	10	658	139	401	15	431	81	519	4
Solyc12g044860.1.1	4	38	0	16	3	29	17	45	2	53	6	41	0
Solyc12g044880.1.1	1	9	0	5	0	6	2	3	0	28	3	3	0
Solyc12g044890.1.1	2	2	0	4	3	4	16	0	8	12	1	4	0
Solyc12g044900.1.1	0	0	0	0	0	0	0	0	0	0	0	0	0
Solyc12g044910.1.1	0	0	0	0	0	0	0	0	0	0	0	0	0
Solyc12g044920.1.1	2	0	2	0	0	3	15	0	10	1	1	3	2
Solyc12g044930.1.1	0	0	0	0	0	0	0	0	0	0	0	0	0
Solyc12g044940.1.1	5	106	1	65	2	37	13	41	5	139	11	97	1
Solyc12g044950.1.1	6	262	24	129	12	25	5	16	6	2	3	11	0
Solyc12g044980.1.1	0	0	0	0	0	0	0	0	0	0	0	0	0
Solyc12g045020.1.1	0	0	0	0	0	0	0	0	0	0	0	0	0
Solyc12g045030.1.1	6	89	48	80	10	50	14	58	2	15	5	27	3
Solyc12g049030.1.1	0	0	0	0	0	0	0	0	0	0	0	0	0
Solyc12g049200.1.1	0	0	0	0	0	0	0	0	0	0	0	0	0
Solyc12g049210.1.1	0	0	0	0	0	0	0	0	0	0	0	0	0
Solyc12g049220.1.1	1	4	1	1	0	3	5	7	2	3	1	10	1
Solyc12g049230.1.1	11	45	7	36	1	208	83	226	18	140	17	103	7
Solyc12g049270.1.1	7	27	0	11	0	244	35	183	1	143	12	135	0
Solyc12g049280.1.1	3	13	3	12	0	4	12	19	0	0	3	3	0
Solyc12g049290.1.1	1	19	0	1	0	93	12	50	5	7	0	18	0
Solyc12g049310.1.1	0	0	0	0	0	0	0	0	0	0	0	0	0
Solyc12g049320.1.1	0	0	0	0	0	0	0	0	0	0	0	0	0
Solyc12g049330.1.1	3	13	3	7	0	11	7	21	4	18	7	8	0
Solyc12g049360.1.1	0	0	0	0	0	0	0	0	0	0	0	0	0
Solyc12g049370.1.1	0	0	0	0	0	0	0	0	0	0	0	0	0
Solyc12g049380.1.1	0	0	0	0	0	0	0	0	0	0	0	0	0
Solyc12g049390.1.1	9	90	13	43	1	30	55	100	5	120	19	126	4
Solyc12g049400.1.1	0	0	0	0	0	0	0	0	0	0	0	0	0
Solyc12g049410.1.1	8	19	6	28	2	61	77	78	10	58	22	61	3

Solyc12g049420.1.1	2	14	0	4	1	28	15	24	3	25	2	14	0
Solyc12g049430.1.1	0	0	0	0	0	0	0	0	0	0	0	0	0
Solyc12g049500.1.1	0	0	0	0	0	0	0	0	0	0	0	0	0
Solyc12g049510.1.1	1	1	1	5	0	0	0	3	0	13	1	1	0
Solyc12g049520.1.1	0	0	0	0	0	0	0	0	0	0	0	0	0
Solyc12g049530.1.1	0	0	0	0	0	0	0	0	0	0	0	0	0
Solyc12g049540.1.1	5	16	5	5	6	40	26	31	5	36	10	24	0
Solyc12g049550.1.1	0	0	0	0	0	0	0	0	0	0	0	0	0
Solyc12g049560.1.1	3	17	1	16	0	18	10	16	0	38	9	26	2
Solyc12g049570.1.1	9	57	10	48	17	107	158	181	47	99	35	150	37
Solyc12g049580.1.1	0	0	0	0	0	0	0	0	0	0	0	0	0
Solyc12g049590.1.1	4	2	3	4	0	16	5	8	0	20	2	9	0
Solyc12g055710.1.1	1	9	6	24	5	0	13	1	12	1	0	8	0
Solyc12g055720.1.1	17	253	39	255	6	307	346	502	103	480	182	365	5
Solyc12g055730.1.1	0	0	0	0	0	0	0	0	0	0	0	0	0
Solyc12g055740.1.1	11	67	21	36	12	183	215	265	30	293	41	153	4
Solyc12g055750.1.1	2	11	2	5	0	15	2	5	1	4	7	14	0
Solyc12g055760.1.1	4	60	4	17	4	91	31	72	6	239	35	171	0
Solyc12g055770.1.1	0	0	0	0	0	0	0	0	0	0	0	0	0
Solyc12g055780.1.1	9	36	3	30	2	43	44	86	8	90	20	70	6
Solyc12g055790.1.1	4	29	0	14	0	74	9	100	2	61	13	19	0
Solyc12g055800.1.1	9	292	15	204	9	625	401	776	75	1224	107	965	9
Solyc12g055810.1.1	0	0	0	0	0	0	0	0	0	0	0	0	0
Solyc12g055820.1.1	8	31	2	19	0	37	53	50	3	72	18	32	0
Solyc12g055830.1.1	6	383	42	163	25	189	231	443	31	1082	70	440	5
Solyc12g055840.1.1	12	226	11	152	2	28	77	81	0	585	56	544	18
Solyc12g055850.1.1	4	22	2	2	0	13	21	29	0	50	8	58	0
Solyc12g055860.1.1	0	0	0	0	0	0	0	0	0	0	0	0	0
Solyc12g055900.1.1	0	0	0	0	0	0	0	0	0	0	0	0	0
Solyc12g055910.1.1	11	18	4	10	1	24	57	72	8	27	15	35	0
Solyc12g055920.1.1	1	7	0	3	0	2	8	2	0	0	0	0	0
Solyc12g055930.1.1	1	20	0	0	0	25	5	11	3	6	0	3	0
Solyc12g055940.1.1	9	64	8	47	0	25	64	108	3	64	12	68	0
Solyc12g055950.1.1	4	14	0	3	0	39	11	48	0	28	7	14	0
Solyc12g055970.1.1	4	18	2	27	0	15	20	67	0	19	10	10	0
Solyc12g055980.1.1	0	0	0	0	0	0	0	0	0	0	0	0	0
Solyc12g055990.1.1	7	64	12	32	1	35	21	40	2	114	15	113	0
Solyc12g056000.1.1	0	0	0	0	0	0	0	0	0	0	0	0	0
Solyc12g056030.1.1	2	6	0	1	2	12	6	20	1	11	6	4	0
Solyc12g056080.1.1	7	75	1	63	6	149	52	197	6	140	27	79	0
Solyc12g056090.1.1	1	4	1	1	0	8	2	6	0	11	2	5	0

Solyc12g056100.1.1	1	126	9	80	11	165	103	304	14	724	42	419	0
Solyc12g056110.1.1	6	32	5	28	3	75	12	90	0	167	10	210	0
Solyc12g056120.1.1	12	251	15	82	14	199	59	381	23	392	87	265	3
Solyc12g056130.1.1	5	8	0	24	0	31	10	23	2	23	2	19	0
Solyc12g056140.1.1	0	0	0	0	0	0	0	0	0	0	0	0	0
Solyc12g056150.1.1	3	11	3	12	0	11	45	24	0	10	0	3	0
Solyc12g056160.1.1	0	0	0	0	0	0	0	0	0	0	0	0	0
Solyc12g056170.1.1	5	45	2	26	0	133	10	112	12	105	16	38	0
Solyc12g056190.1.1	0	0	0	0	0	0	0	0	0	0	0	0	0
Solyc12g056200.1.1	3	14	0	10	0	0	5	3	3	60	14	42	1
Solyc12g056210.1.1	2	28	1	23	0	42	9	38	1	27	6	30	0
Solyc12g056220.1.1	5	802	50	565	25	166	292	846	18	1272	134	1053	14
Solyc12g056230.1.1	4	84	2	28	2	92	91	61	21	116	28	59	3
Solyc12g056240.1.1	2	13	0	4	0	16	21	17	4	18	1	10	0
Solyc12g056250.1.1	3	95	4	13	10	211	94	194	26	67	15	49	3
Solyc12g056270.1.1	0	0	0	0	0	0	0	0	0	0	0	0	0
Solyc12g056300.1.1	4	22	0	14	0	7	21	27	1	19	0	10	0
Solyc12g056340.1.1	3	3	0	3	0	18	16	13	3	22	7	12	3
Solyc12g056350.1.1	0	0	0	0	0	0	0	0	0	0	0	0	0
Solyc12g056360.1.1	2	29	0	23	7	9	10	9	3	4	0	9	0
Solyc12g056390.1.1	2	9	1	0	0	15	16	8	10	0	0	0	0
Solyc12g056400.1.1	4	21	1	18	0	29	59	14	2	43	11	40	4
Solyc12g056410.1.1	11	234	17	217	4	320	50	132	12	223	53	255	1
Solyc12g056420.1.1	0	0	0	0	0	0	0	0	0	0	0	0	0
Solyc12g056440.1.1	14	43	5	40	4	112	119	110	14	71	12	63	3
Solyc12g056450.1.1	2	6	0	6	0	6	10	18	4	12	5	16	0
Solyc12g056460.1.1	0	0	0	0	0	0	0	0	0	0	0	0	0
Solyc12g056490.1.1	0	0	0	0	0	0	0	0	0	0	0	0	0
Solyc12g056510.1.1	16	99	10	51	14	33	108	54	25	161	30	118	4
Solyc12g056520.1.1	1	4	0	0	0	2	4	7	1	10	0	8	0
Solyc12g056530.1.1	9	116	20	57	6	83	198	128	45	274	78	213	9
Solyc12g056540.1.1	0	0	0	0	0	0	0	0	0	0	0	0	0
Solyc12g056550.1.1	4	30	10	10	0	9	12	23	1	36	8	29	0
Solyc12g056560.1.1	0	0	0	0	0	0	0	0	0	0	0	0	0
Solyc12g056580.1.1	12	443	29	226	46	493	97	211	51	464	77	352	11
Solyc12g056590.1.1	0	0	0	0	0	0	0	0	0	0	0	0	0
Solyc12g056600.2.1	0	0	0	0	0	0	0	0	0	0	0	0	0
Solyc12g056610.1.1	1	7	1	0	5	0	3	0	0	0	0	0	0
Solyc12g056620.1.1	3	23	0	8	0	4	19	10	0	8	1	4	0
Solyc12g056630.1.1	2	4	0	4	0	2	4	6	1	7	7	9	0
Solyc12g056640.1.1	10	163	10	74	9	168	70	266	23	199	41	170	2

Solyc12g056650.1.1	23	217	39	159	24	1469	868	1330	151	1060	186	1164	18
Solyc12g056660.1.1	0	0	0	0	0	0	0	0	0	0	0	0	0
Solyc12g056700.1.1	0	0	0	0	0	0	0	0	0	0	0	0	0
Solyc12g056710.1.1	0	0	0	0	0	0	0	0	0	0	0	0	0
Solyc12g056730.1.1	0	0	0	0	0	0	0	0	0	0	0	0	0
Solyc12g056740.1.1	17	109	16	71	9	101	231	241	14	173	45	124	4
Solyc12g056760.1.1	0	0	0	0	0	0	0	0	0	0	0	0	0
Solyc12g056770.1.1	3	36	4	43	5	85	16	42	4	101	43	123	3
Solyc12g056780.1.1	4	45	3	10	1	90	33	67	18	215	32	126	1
Solyc12g056790.1.1	15	121	27	73	19	228	215	385	37	202	54	154	8
Solyc12g056800.1.1	11	101	18	17	14	35	12	45	0	39	41	25	1
Solyc12g056830.1.1	3	1890	550	1335	24	49	1554	401	34	254	539	285	8
Solyc12g056840.1.1	9	37	3	17	2	29	25	46	11	163	27	92	1
Solyc12g056850.1.1	7	84	1	34	1	156	43	154	4	4	2	4	0
Solyc12g056860.1.1	6	20	3	6	0	106	100	128	0	6	10	22	0
Solyc12g056880.1.1	0	0	0	0	0	0	0	0	0	0	0	0	0
Solyc12g056890.1.1	0	0	0	0	0	0	0	0	0	0	0	0	0
Solyc12g056900.1.1	1	13	0	5	4	5	18	11	0	23	1	19	0
Solyc12g056910.1.1	1	13	0	0	1	13	8	13	0	11	3	19	0
Solyc12g056920.1.1	0	0	0	0	0	0	0	0	0	0	0	0	0
Solyc12g056930.1.1	4	14	0	2	0	105	10	67	12	8	8	26	0
Solyc12g056940.1.1	34	303	20	153	30	2082	1111	2715	515	319	59	154	10
Solyc12g056950.1.1	7	43	1	52	3	15	14	36	2	93	9	113	1
Solyc12g056960.1.1	0	0	0	0	0	0	0	0	0	0	0	0	0
Solyc12g056980.1.1	3	35	0	17	4	3	29	47	18	12	1	8	0
Solyc12g056990.1.1	1	1	0	10	0	7	4	1	0	1	1	0	0
Solyc12g057000.1.1	0	0	0	0	0	0	0	0	0	0	0	0	0
Solyc12g057010.1.1	0	0	0	0	0	0	0	0	0	0	0	0	0
Solyc12g057030.1.1	0	0	0	0	0	0	0	0	0	0	0	0	0
Solyc12g057040.1.1	12	257	42	264	15	72	201	262	10	465	54	460	5
Solyc12g057060.1.1	0	0	0	0	0	0	0	0	0	0	0	0	0
Solyc12g057080.1.1	2	50	0	10	0	1	17	0	0	0	0	0	0
Solyc12g057090.1.1	0	0	0	0	0	0	0	0	0	0	0	0	0
Solyc12g057110.2.1	4	163	7	55	4	68	45	95	7	306	67	267	5
Solyc12g057120.1.1	3	35	4	14	1	19	24	45	6	36	15	38	0
Solyc12g057140.1.1	1	15	0	10	0	14	8	12	4	5	3	0	0
Solyc12g057150.1.1	1	38	1	9	2	10	5	4	3	6	0	9	0
Solyc12g057160.1.1	0	0	0	0	0	0	0	0	0	0	0	0	0
Solyc12g062200.1.1	0	0	0	0	0	0	0	0	0	0	0	0	0
Solyc12g062250.1.1	7	69	5	43	2	27	54	41	0	57	3	72	0
Solyc12g062260.1.1	0	0	0	0	0	0	0	0	0	0	0	0	0

Solyc12g062280.1.1	3	20	3	15	2	49	15	78	16	30	19	24	0
Solyc12g062290.1.1	2	3	0	0	3	17	8	27	1	10	3	15	5
Solyc12g062300.1.1	0	0	0	0	0	0	0	0	0	0	0	0	0
Solyc12g062480.1.1	0	0	0	0	0	0	0	0	0	0	0	0	0
Solyc12g062550.1.1	0	0	0	0	0	0	0	0	0	0	0	0	0
Solyc12g062670.1.1	0	0	0	0	0	0	0	0	0	0	0	0	0
Solyc12g062690.1.1	1	29	1	28	0	21	14	19	0	5	0	3	0
Solyc12g062860.1.1	4	18	0	14	0	27	25	33	0	63	15	83	1
Solyc12g062870.1.1	7	273	32	215	21	772	524	809	61	409	180	219	12
Solyc12g062920.1.1	0	0	0	0	0	0	0	0	0	0	0	0	0
Solyc12g062940.1.1	0	0	0	0	0	0	0	0	0	0	0	0	0
Solyc12g062960.1.1	0	0	0	0	0	0	0	0	0	0	0	0	0
Solyc12g062970.1.1	0	0	0	0	0	0	0	0	0	0	0	0	0
Solyc12g062980.1.1	0	0	0	0	0	0	0	0	0	0	0	0	0
Solyc12g062990.1.1	0	0	0	0	0	0	0	0	0	0	0	0	0
Solyc12g063000.1.1	0	0	0	0	0	0	0	0	0	0	0	0	0
Solyc12g063050.1.1	19	236	20	170	21	328	132	426	33	501	74	411	4
Solyc12g068070.1.1	10	83	10	77	1	72	59	186	8	151	17	125	0
Solyc12g070080.1.1	1	0	0	0	0	16	6	17	3	0	1	0	0
Solyc12g070090.1.1	0	0	0	0	0	0	0	0	0	0	0	0	0
Solyc12g070100.1.1	12	75	6	71	0	215	161	286	22	110	37	85	0
Solyc12g070120.1.1	0	0	0	0	0	0	0	0	0	0	0	0	0
Solyc12g070200.1.1	0	0	0	0	0	0	0	0	0	0	0	0	0
Solyc12g070270.1.1	2	14	2	25	0	2	2	14	0	5	8	9	0
Solyc12g070280.1.1	2	93	5	58	0	20	14	36	1	25	9	28	0
Solyc12g076320.1.1	0	0	0	0	0	0	0	0	0	0	0	0	0
Solyc12g076330.1.1	0	0	0	0	0	0	0	0	0	0	0	0	0
Solyc12g076340.1.1	0	0	0	0	0	0	0	0	0	0	0	0	0
Solyc12g076350.1.1	0	0	0	0	0	0	0	0	0	0	0	0	0
Solyc12g077380.1.1	0	0	0	0	0	0	0	0	0	0	0	0	0
Solyc12g077420.1.1	0	0	0	0	0	0	0	0	0	0	0	0	0
Solyc12g077520.1.1	0	0	0	0	0	0	0	0	0	0	0	0	0
Solyc12g077540.1.1	5	39	4	23	0	34	42	57	11	33	12	35	1
Solyc12g077590.1.1	1	17	0	3	0	28	17	10	0	25	1	27	0
Solyc12g077620.1.1	0	0	0	0	0	0	0	0	0	0	0	0	0
Solyc12g077660.1.1	0	0	0	0	0	0	0	0	0	0	0	0	0
Solyc12g087790.1.1	0	0	0	0	0	0	0	0	0	0	0	0	0
Solyc12g087810.1.1	1	5	1	0	0	1	2	6	0	5	0	4	0
Solyc12g087820.1.1	0	0	0	0	0	0	0	0	0	0	0	0	0
Solyc12g087830.1.1	6	54	1	41	0	91	13	83	4	108	10	46	0
Solyc12g087840.1.1	0	0	0	0	0	0	0	0	0	0	0	0	0

Solyc12g087860.1.1	0	0	0	0	0	0	0	0	0	0	0	0	0
Solyc12g087870.1.1	0	0	0	0	0	0	0	0	0	0	0	0	0
Solyc12g087880.1.1	0	0	0	0	0	0	0	0	0	0	0	0	0
Solyc12g087890.1.1	8	62	8	34	3	57	35	44	5	82	17	99	0
Solyc12g087920.1.1	0	0	0	0	0	0	0	0	0	0	0	0	0
Solyc12g087930.1.1	0	0	0	0	0	0	0	0	0	0	0	0	0
Solyc12g087940.1.1	2	5	0	5	0	0	1	4	0	1	0	0	0
Solyc12g087950.1.1	11	114	6	61	1	316	152	266	51	76	5	38	0
Solyc12g087960.1.1	3	14	0	2	0	15	44	46	4	38	14	10	3
Solyc12g087970.1.1	7	36	0	17	3	48	40	71	11	56	10	45	8
Solyc12g087980.1.1	2	0	0	0	1	0	6	1	5	31	10	4	5
Solyc12g087990.1.1	0	0	0	0	0	0	0	0	0	0	0	0	0
Solyc12g088010.1.1	0	0	0	0	0	0	0	0	0	0	0	0	0
Solyc12g088020.1.1	6	17	0	13	8	29	61	27	7	21	6	7	0
Solyc12g088030.1.1	0	0	0	0	0	0	0	0	0	0	0	0	0
Solyc12g088040.1.1	1	2	0	2	0	14	6	12	1	0	0	0	0
Solyc12g088050.1.1	15	40	1	34	0	81	115	91	1	33	4	46	1
Solyc12g088070.1.1	0	0	0	0	0	0	0	0	0	0	0	0	0
Solyc12g088110.1.1	0	0	0	0	0	0	0	0	0	0	0	0	0
Solyc12g088140.1.1	0	0	0	0	0	0	0	0	0	0	0	0	0
Solyc12g088160.1.1	0	0	0	0	0	0	0	0	0	0	0	0	0
Solyc12g088170.1.1	0	0	0	0	0	0	0	0	0	0	0	0	0
Solyc12g088180.1.1	0	0	0	0	0	0	0	0	0	0	0	0	0
Solyc12g088190.1.1	10	26	2	42	0	1836	495	1704	59	30	5	6	0
Solyc12g088200.1.1	9	40	11	25	3	99	30	94	4	51	11	59	0
Solyc12g088210.1.1	7	35	3	22	0	34	48	103	7	41	14	47	1
Solyc12g088220.1.1	0	0	0	0	0	0	0	0	0	0	0	0	0
Solyc12g088230.1.1	23	94	9	48	6	553	172	683	56	139	108	155	1
Solyc12g088240.1.1	0	0	0	0	0	0	0	0	0	0	0	0	0
Solyc12g088250.1.1	0	0	0	0	0	0	0	0	0	0	0	0	0
Solyc12g088290.1.1	16	98	15	76	0	183	101	269	26	225	48	162	7
Solyc12g088300.1.1	0	0	0	0	0	0	0	0	0	0	0	0	0
Solyc12g088310.1.1	4	18	2	11	0	23	14	22	3	25	5	20	0
Solyc12g088320.1.1	0	0	0	0	0	0	0	0	0	0	0	0	0
Solyc12g088340.1.1	8	54	11	39	4	137	92	134	12	131	17	57	0
Solyc12g088360.1.1	2	22	1	20	0	0	0	4	1	2	2	16	0
Solyc12g088370.1.1	5	17	0	2	0	76	10	48	0	15	13	13	2
Solyc12g088390.1.1	0	0	0	0	0	0	0	0	0	0	0	0	0
Solyc12g088400.1.1	0	0	0	0	0	0	0	0	0	0	0	0	0
Solyc12g088410.1.1	0	0	0	0	0	0	0	0	0	0	0	0	0
Solyc12g088420.1.1	1	5	1	0	0	13	5	5	1	7	0	5	0

Solyc12g088430.1.1	2	8	1	1	0	0	1	0	2	5	17	9	0
Solyc12g088440.1.1	7	76	11	41	1	68	24	51	5	44	5	66	0
Solyc12g088450.1.1	1	0	0	4	0	5	7	0	2	2	2	1	0
Solyc12g088460.1.1	2	7	0	2	0	11	6	10	2	2	4	5	0
Solyc12g088480.1.1	0	0	0	0	0	0	0	0	0	0	0	0	0
Solyc12g088510.1.1	0	0	0	0	0	0	0	0	0	0	0	0	0
Solyc12g088520.1.1	0	0	0	0	0	0	0	0	0	0	0	0	0
Solyc12g088530.1.1	0	0	0	0	0	0	0	0	0	0	0	0	0
Solyc12g088640.1.1	0	0	0	0	0	0	0	0	0	0	0	0	0
Solyc12g088650.1.1	2	16	15	5	6	20	91	8	5	7	68	6	8
Solyc12g088660.1.1	1	3	0	2	0	1	5	6	2	8	3	4	0
Solyc12g088670.1.1	17	3948	292	4188	144	7089	4031	16809	672	1916	188	300	53
Solyc12g088680.1.1	0	0	0	0	0	0	0	0	0	0	0	0	0
Solyc12g088690.1.1	0	0	0	0	0	0	0	0	0	0	0	0	0
Solyc12g088700.1.1	1	8	0	0	0	0	4	29	0	21	4	26	0
Solyc12g088720.1.1	12	940	80	671	82	3558	1315	3270	230	2211	548	1572	48
Solyc12g088730.1.1	3	5	0	2	1	31	11	43	2	18	11	21	0
Solyc12g088740.1.1	16	75	20	64	6	114	113	146	17	87	21	97	4
Solyc12g088750.1.1	3	41	3	15	0	0	5	8	0	16	19	6	0
Solyc12g088760.1.1	7	91	13	42	0	14	75	41	0	0	1	1	0
Solyc12g088800.1.1	0	0	0	0	0	0	0	0	0	0	0	0	0
Solyc12g088810.1.1	6	10	1	6	0	9	34	14	1	32	9	21	4
Solyc12g088820.1.1	0	0	0	0	0	0	0	0	0	0	0	0	0
Solyc12g088830.1.1	3	10	3	5	0	3	8	11	0	81	15	112	0
Solyc12g088840.1.1	9	66	12	74	0	10	19	80	0	43	7	30	0
Solyc12g088870.1.1	1	9	2	6	0	3	6	18	0	0	0	0	0
Solyc12g088880.1.1	5	43	1	24	0	72	93	109	14	32	6	30	1
Solyc12g088890.1.1	2	0	0	2	1	12	6	5	0	0	4	3	0
Solyc12g088900.1.1	1	0	0	3	0	14	6	12	0	20	4	14	0
Solyc12g088910.1.1	2	11	2	9	0	0	4	5	0	22	2	17	0
Solyc12g088920.1.1	9	27	1	23	2	97	75	93	10	61	13	49	0
Solyc12g088930.1.1	0	0	0	0	0	0	0	0	0	0	0	0	0
Solyc12g088940.1.1	5	7	1	21	0	62	15	30	0	0	2	3	0
Solyc12g088950.1.1	0	0	0	0	0	0	0	0	0	0	0	0	0
Solyc12g088960.1.1	1	4	0	0	0	5	3	8	0	17	1	2	0
Solyc12g088980.1.1	0	0	0	0	0	0	0	0	0	0	0	0	0
Solyc12g088990.1.1	15	52	6	80	4	140	61	139	8	177	35	123	2
Solyc12g089000.1.1	6	12	4	9	0	42	60	63	23	64	22	53	1
Solyc12g089010.1.1	0	0	0	0	0	0	0	0	0	0	0	0	0
Solyc12g089020.1.1	4	4	0	0	1	13	17	6	0	16	3	32	1
Solyc12g089030.1.1	2	6	7	6	0	29	43	31	12	109	10	28	2

Solyc12g089040.1.1	9	120	5	102	16	126	55	121	10	127	17	104	1
Solyc12g089050.1.1	0	0	0	0	0	0	0	0	0	0	0	0	0
Solyc12g089060.1.1	1	4	0	0	0	2	10	2	0	14	2	22	4
Solyc12g089090.1.1	8	42	2	24	1	94	84	76	13	45	19	32	3
Solyc12g089100.1.1	1	5	0	5	0	21	1	4	0	0	4	2	0
Solyc12g089110.1.1	0	0	0	0	0	0	0	0	0	0	0	0	0
Solyc12g089130.1.1	0	0	0	0	0	0	0	0	0	0	0	0	0
Solyc12g089140.1.1	2	12	1	9	4	40	40	45	21	57	13	25	2
Solyc12g089150.1.1	5	103	3	62	2	188	70	178	11	103	14	105	0
Solyc12g089160.1.1	29	398	57	292	23	855	423	972	17	234	106	210	3
Solyc12g089180.1.1	22	201	8	48	4	488	254	647	11	181	21	111	1
Solyc12g089200.1.1	0	0	0	0	0	0	0	0	0	0	0	0	0
Solyc12g089210.1.1	8	56	9	30	0	42	37	93	1	69	11	42	0
Solyc12g089220.1.1	19	467	21	269	2	1454	854	1740	26	163	42	123	0
Solyc12g089230.1.1	0	0	0	0	0	0	0	0	0	0	0	0	0
Solyc12g089240.1.1	0	0	0	0	0	0	0	0	0	0	0	0	0
Solyc12g089250.1.1	0	0	0	0	0	0	0	0	0	0	0	0	0
Solyc12g089260.1.1	4	15	4	14	0	22	15	32	1	10	0	28	0
Solyc12g089280.1.1	9	40	5	37	1	83	49	134	8	102	15	96	1
Solyc12g089290.1.1	0	0	0	0	0	0	0	0	0	0	0	0	0
Solyc12g089300.1.1	0	0	0	0	0	0	0	0	0	0	0	0	0
Solyc12g089310.1.1	0	0	0	0	0	0	0	0	0	0	0	0	0
Solyc12g089320.1.1	12	58	3	28	9	85	110	102	11	56	58	50	0
Solyc12g089330.1.1	0	0	0	0	0	0	0	0	0	0	0	0	0
Solyc12g089340.1.1	7	32	5	17	0	66	108	87	6	81	16	56	1
Solyc12g089350.1.1	0	0	0	0	0	0	0	0	0	0	0	0	0
Solyc12g094380.1.1	11	71	42	28	1	200	1077	321	10	70	50	32	2
Solyc12g094390.1.1	0	0	0	0	0	0	0	0	0	0	0	0	0
Solyc12g094400.1.1	0	0	0	0	0	0	0	0	0	0	0	0	0
Solyc12g094410.1.1	0	0	0	0	0	0	0	0	0	0	0	0	0
Solyc12g094420.1.1	1	0	0	1	0	0	2	2	0	8	0	2	0
Solyc12g094430.1.1	7	57	29	39	16	41	333	52	20	133	44	96	25
Solyc12g094440.1.1	0	0	0	0	0	0	0	0	0	0	0	0	0
Solyc12g094450.1.1	7	51	5	43	4	112	84	103	10	75	9	51	2
Solyc12g094460.1.1	2	2	1	4	0	36	9	27	0	0	0	0	0
Solyc12g094480.1.1	0	0	0	0	0	0	0	0	0	0	0	0	0
Solyc12g094490.1.1	11	49	13	45	1	87	86	200	17	103	22	54	1
Solyc12g094500.1.1	3	64	5	14	0	6	11	8	6	77	1	60	1
Solyc12g094510.1.1	0	0	0	0	0	0	0	0	0	0	0	0	0
Solyc12g094520.1.1	19	180	52	98	13	341	329	222	37	87	67	119	7
Solyc12g094540.1.1	2	8	0	3	1	10	9	6	0	21	6	41	0

Solyc12g094550.1.1	6	84	24	44	13	246	244	154	30	92	62	42	0
Solyc12g094570.1.1	3	5	0	0	0	6	16	4	2	0	1	14	0
Solyc12g094580.1.1	0	0	0	0	0	0	0	0	0	0	0	0	0
Solyc12g094590.1.1	5	9	0	26	4	46	32	32	3	35	5	23	0
Solyc12g094600.1.1	0	0	0	0	0	0	0	0	0	0	0	0	0
Solyc12g094610.1.1	0	0	0	0	0	0	0	0	0	0	0	0	0
Solyc12g094620.1.1	7	9233	463	9866	234	5527	2751	19787	63	10516	717	7532	10
Solyc12g094630.1.1	6	440	39	392	1	699	229	795	3	341	31	268	2
Solyc12g094640.1.1	9	1083	398	1295	5	13	23	9	4	11	19	42	0
Solyc12g094650.1.1	7	42	11	37	1	84	41	87	10	10	0	16	1
Solyc12g094660.1.1	14	27	7	31	16	91	77	86	18	37	14	25	1
Solyc12g094690.1.1	0	0	0	0	0	0	0	0	0	0	0	0	0
Solyc12g094700.1.1	3	20	0	5	0	17	19	31	0	0	0	0	0
Solyc12g094710.1.1	4	15	1	9	1	49	37	33	4	31	7	27	0
Solyc12g094720.1.1	0	0	0	0	0	0	0	0	0	0	0	0	0
Solyc12g095750.1.1	0	0	0	0	0	0	0	0	0	0	0	0	0
Solyc12g095760.1.1	8	98	10	54	1	74	91	216	20	490	59	342	2
Solyc12g095770.1.1	3	192	34	54	2	95	66	97	12	127	8	75	7
Solyc12g095780.1.1	4	33	5	36	1	21	20	53	1	65	31	53	4
Solyc12g095790.1.1	2	25	0	8	0	30	8	22	3	26	4	32	0
Solyc12g095800.1.1	9	46	8	30	4	90	127	113	10	157	20	117	6
Solyc12g095810.1.1	4	2	0	0	0	19	27	69	1	16	1	3	0
Solyc12g095820.1.1	0	0	0	0	0	0	0	0	0	0	0	0	0
Solyc12g095830.1.1	16	183	32	151	13	325	353	348	43	211	73	190	6
Solyc12g095840.1.1	3	8	1	18	3	8	10	11	3	26	8	16	3
Solyc12g095850.1.1	6	21	4	9	2	19	43	27	3	22	12	23	0
Solyc12g095860.1.1	4	68	1	37	1	71	26	58	0	109	10	75	0
Solyc12g095870.1.1	0	0	0	0	0	0	0	0	0	0	0	0	0
Solyc12g095880.1.1	8	39	16	33	4	116	141	149	26	40	43	42	0
Solyc12g095890.1.1	12	90	14	81	10	154	158	117	22	271	76	205	11
Solyc12g095900.1.1	17	89	15	103	0	181	193	279	22	255	23	177	1
Solyc12g095910.1.1	18	824	25	661	31	1964	826	2676	103	1960	142	1415	12
Solyc12g095920.1.1	5	22	3	8	1	21	36	57	5	51	18	35	0
Solyc12g095930.1.1	14	58	26	37	6	77	280	167	44	76	62	82	1
Solyc12g095940.1.1	1	2	0	1	0	11	5	10	1	6	2	6	0
Solyc12g095950.1.1	1	32	1	24	3	30	14	55	2	79	5	48	1
Solyc12g095960.1.1	9	221	26	143	10	253	167	506	29	600	86	407	10
Solyc12g095970.1.1	0	0	0	0	0	0	0	0	0	0	0	0	0
Solyc12g095980.1.1	0	0	0	0	0	0	0	0	0	0	0	0	0
Solyc12g095990.1.1	4	86	18	61	5	186	120	271	17	183	84	210	4
Solyc12g096000.1.1	4	132	19	90	2	251	73	254	21	517	65	330	5

Solyc12g096010.1.1	2	10	0	1	0	10	7	10	9	66	1	23	0
Solyc12g096020.1.1	7	166	37	131	8	118	20	129	2	87	13	85	1
Solyc12g096030.1.1	5	8	0	8	0	8	10	32	2	29	19	27	1
Solyc12g096040.1.1	4	15	2	25	0	34	24	34	5	54	10	24	0
Solyc12g096050.1.1	3	8	3	12	2	18	33	42	5	40	5	37	1
Solyc12g096060.1.1	5	57	2	8	1	61	37	79	3	67	19	52	0
Solyc12g096070.1.1	1	3	0	1	0	4	4	18	1	2	7	2	0
Solyc12g096090.1.1	0	0	0	0	0	0	0	0	0	0	0	0	0
Solyc12g096100.1.1	5	23	5	10	0	2	58	18	0	19	1	5	0
Solyc12g096120.1.1	1	3	1	0	0	9	9	6	1	18	0	16	0
Solyc12g096130.1.1	0	0	0	0	0	0	0	0	0	0	0	0	0
Solyc12g096140.1.1	7	20	1	18	2	85	36	58	3	47	15	40	1
Solyc12g096150.1.1	12	181	5	70	12	398	100	270	35	356	110	380	7
Solyc12g096160.1.1	6	17	5	9	4	11	38	42	3	28	5	13	0
Solyc12g096170.1.1	0	0	0	0	0	0	0	0	0	0	0	0	0
Solyc12g096180.1.1	0	0	0	0	0	0	0	0	0	0	0	0	0
Solyc12g096190.1.1	17	128	6	158	20	494	55	1040	20	2208	208	2830	24
Solyc12g096220.1.1	7	648	24	242	18	869	227	980	51	2025	157	1597	12
Solyc12g096230.1.1	0	0	0	0	0	0	0	0	0	0	0	0	0
Solyc12g096250.1.1	0	0	0	0	0	0	0	0	0	0	0	0	0
Solyc12g096270.1.1	3	12	1	8	0	24	8	18	3	18	5	9	0
Solyc12g096280.1.1	0	0	0	0	0	0	0	0	0	0	0	0	0
Solyc12g096290.1.1	2	11	1	5	0	7	8	21	0	11	6	15	0
Solyc12g096300.1.1	5	218	19	65	16	415	238	563	74	476	202	380	10
Solyc12g096310.1.1	0	0	0	0	0	0	0	0	0	0	0	0	0
Solyc12g096320.1.1	1	2	0	1	0	0	6	1	1	4	0	2	0
Solyc12g096330.1.1	1	4	0	0	0	9	5	4	0	0	0	4	0
Solyc12g096340.1.1	5	87	9	59	6	177	42	106	15	140	36	139	3
Solyc12g096350.1.1	1	6	1	10	2	6	0	2	1	4	4	12	0
Solyc12g096370.1.1	3	12	0	7	0	11	13	5	0	7	6	17	0
Solyc12g096390.1.1	0	0	0	0	0	0	0	0	0	0	0	0	0
Solyc12g096400.1.1	0	0	0	0	0	0	0	0	0	0	0	0	0
Solyc12g096410.1.1	0	0	0	0	0	0	0	0	0	0	0	0	0
Solyc12g096420.1.1	18	161	27	151	1	480	318	645	26	137	53	112	0
Solyc12g096430.1.1	0	0	0	0	0	0	0	0	0	0	0	0	0
Solyc12g096440.1.1	0	0	0	0	0	0	0	0	0	0	0	0	0
Solyc12g096450.1.1	6	32	4	9	0	99	20	66	11	81	21	79	5
Solyc12g096460.1.1	3	9	0	1	0	16	35	14	7	16	7	8	1
Solyc12g096470.1.1	2	31	2	17	0	137	65	99	7	73	2	38	0
Solyc12g096480.1.1	0	0	0	0	0	0	0	0	0	0	0	0	0
Solyc12g096490.1.1	0	0	0	0	0	0	0	0	0	0	0	0	0

Solyc12g096500.1.1	13	927	159	777	56	401	433	591	12	231	28	231	0
Solyc12g096510.1.1	0	0	0	0	0	0	0	0	0	0	0	0	0
Solyc12g096520.1.1	5	47	2	21	1	239	114	233	9	84	31	86	2
Solyc12g096530.1.1	0	0	0	0	0	0	0	0	0	0	0	0	0
Solyc12g096540.1.1	3	258	8	81	1	399	47	286	6	1331	35	544	14
Solyc12g096550.1.1	12	413	42	272	4	310	152	314	1	248	36	295	0
Solyc12g096560.1.1	7	71	8	52	3	159	92	89	6	406	61	199	15
Solyc12g096570.1.1	1	7	0	1	0	45	35	8	2	0	3	0	0
Solyc12g096580.1.1	7	31	4	13	0	42	34	15	1	41	7	65	0
Solyc12g096590.1.1	0	0	0	0	0	0	0	0	0	0	0	0	0
Solyc12g096600.1.1	0	0	0	0	0	0	0	0	0	0	0	0	0
Solyc12g096610.1.1	3	15	2	19	0	34	8	15	0	9	1	1	0
Solyc12g096630.1.1	0	0	0	0	0	0	0	0	0	0	0	0	0
Solyc12g096650.1.1	1	20	0	7	0	25	10	31	1	39	5	19	0
Solyc12g096660.1.1	0	0	0	0	0	0	0	0	0	0	0	0	0
Solyc12g096670.1.1	15	126	6	84	2	490	113	379	6	229	56	247	1
Solyc12g096680.1.1	0	0	0	0	0	0	0	0	0	0	0	0	0
Solyc12g096690.1.1	6	239	7	76	11	710	122	578	46	249	40	257	0
Solyc12g096700.1.1	2	95	3	46	0	166	19	238	10	260	27	265	3
Solyc12g096710.1.1	1	3	3	1	0	6	1	8	0	1	0	0	0
Solyc12g096720.1.1	0	0	0	0	0	0	0	0	0	0	0	0	0
Solyc12g096730.1.1	0	0	0	0	0	0	0	0	0	0	0	0	0
Solyc12g096760.1.1	11	287	21	132	11	178	230	277	24	602	89	563	12
Solyc12g096770.1.1	3	149	12	51	0	24	18	11	0	7	4	10	0
Solyc12g096780.1.1	0	0	0	0	0	0	0	0	0	0	0	0	0
Solyc12g096790.1.1	0	0	0	0	0	0	0	0	0	0	0	0	0
Solyc12g096800.1.1	0	0	0	0	0	0	0	0	0	0	0	0	0
Solyc12g096810.1.1	0	0	0	0	0	0	0	0	0	0	0	0	0
Solyc12g096820.1.1	0	0	0	0	0	0	0	0	0	0	0	0	0
Solyc12g096830.1.1	13	48	5	52	12	106	116	125	4	131	10	129	0
Solyc12g096840.1.1	0	0	0	0	0	0	0	0	0	0	0	0	0
Solyc12g096860.1.1	0	0	0	0	0	0	0	0	0	0	0	0	0
Solyc12g096870.1.1	7	74	6	30	12	15	26	27	4	90	36	70	3
Solyc12g096880.1.1	0	0	0	0	0	0	0	0	0	0	0	0	0
Solyc12g096900.1.1	1	30	3	2	5	0	1	3	6	7	1	0	0
Solyc12g096920.1.1	0	0	0	0	0	0	0	0	0	0	0	0	0
Solyc12g096940.1.1	0	0	0	0	0	0	0	0	0	0	0	0	0
Solyc12g096950.1.1	0	0	0	0	0	0	0	0	0	0	0	0	0
Solyc12g096960.1.1	0	0	0	0	0	0	0	0	0	0	0	0	0
Solyc12g096970.1.1	0	0	0	0	0	0	0	0	0	0	0	0	0
Solyc12g096990.1.1	1	3	0	9	0	0	0	6	0	6	4	8	0

Solyc12g097000.1.1	0	0	0	0	0	0	0	0	0	0	0	0	0
Solyc12g097010.1.1	0	0	0	0	0	0	0	0	0	0	0	0	0
Solyc12g097030.1.1	5	98	4	55	0	5	13	32	0	34	1	45	1
Solyc12g097040.1.1	1	8	3	19	0	13	5	9	1	26	12	32	0
Solyc12g097060.1.1	0	0	0	0	0	0	0	0	0	0	0	0	0
Solyc12g097070.1.1	1	3	0	5	0	1	3	6	0	0	1	1	0
Solyc12g098100.1.1	0	0	0	0	0	0	0	0	0	0	0	0	0
Solyc12g098120.1.1	0	0	0	0	0	0	0	0	0	0	0	0	0
Solyc12g098130.1.1	0	0	0	0	0	0	0	0	0	0	0	0	0
Solyc12g098150.1.1	4	133	7	68	0	444	81	388	20	127	34	67	2
Solyc12g098160.1.1	1	3	0	2	0	1	4	5	0	0	1	3	0
Solyc12g098190.1.1	0	0	0	0	0	0	0	0	0	0	0	0	0
Solyc12g098220.1.1	0	0	0	0	0	0	0	0	0	0	0	0	0
Solyc12g098230.1.1	0	0	0	0	0	0	0	0	0	0	0	0	0
Solyc12g098310.1.1	6	58	9	50	4	122	50	105	8	70	14	49	5
Solyc12g098330.1.1	1	24	1	8	1	30	21	106	3	24	19	52	0
Solyc12g098340.1.1	1	0	0	0	0	0	0	2	0	262	7	427	0
Solyc12g098350.1.1	5	120	15	61	5	43	120	97	4	81	35	67	1
Solyc12g098360.1.1	1	3	1	4	0	4	4	13	0	1	0	10	0
Solyc12g098370.1.1	4	21	4	12	0	3	10	26	0	25	0	12	0
Solyc12g098380.1.1	15	67	24	31	16	77	217	190	28	53	52	72	3
Solyc12g098390.1.1	0	0	0	0	0	0	0	0	0	0	0	0	0
Solyc12g098400.1.1	0	0	0	0	0	0	0	0	0	0	0	0	0
Solyc12g098410.1.1	0	0	0	0	0	0	0	0	0	0	0	0	0
Solyc12g098420.1.1	11	146	14	106	19	315	196	334	44	157	56	131	1
Solyc12g098430.1.1	0	0	0	0	0	0	0	0	0	0	0	0	0
Solyc12g098440.1.1	1	0	2	0	3	0	7	0	2	1	4	0	6
Solyc12g098450.1.1	0	0	0	0	0	0	0	0	0	0	0	0	0
Solyc12g098470.1.1	5	31	2	9	0	63	37	61	7	76	11	37	0
Solyc12g098490.1.1	6	77	8	66	37	223	34	111	24	30	6	77	0
Solyc12g098500.1.1	5	28	2	15	23	41	49	112	27	151	7	63	0
Solyc12g098510.1.1	1	7	1	15	0	0	7	3	0	19	9	18	1
Solyc12g098520.1.1	6	6	0	17	0	32	25	49	4	95	6	37	2
Solyc12g098530.1.1	0	0	0	0	0	0	0	0	0	0	0	0	0
Solyc12g098540.1.1	0	0	0	0	0	0	0	0	0	0	0	0	0
Solyc12g098570.1.1	11	69	6	51	2	30	16	48	2	73	54	72	0
Solyc12g098580.1.1	0	0	0	0	0	0	0	0	0	0	0	0	0
Solyc12g098590.1.1	0	0	0	0	0	0	0	0	0	0	0	0	0
Solyc12g098600.1.1	20	160	12	98	3	20	230	81	3	144	43	148	9
Solyc12g098610.1.1	0	0	0	0	0	0	0	0	0	0	0	0	0
Solyc12g098630.1.1	0	0	0	0	0	0	0	0	0	0	0	0	0

Solyc12g098640.1.1	10	428	15	91	13	113	159	208	8	455	151	245	0
Solyc12g098660.1.1	1	27	1	5	0	19	15	7	0	3	0	4	0
Solyc12g098670.1.1	7	67	8	35	1	104	150	141	24	211	51	196	12
Solyc12g098680.1.1	7	114	10	87	7	201	86	164	14	688	39	587	6
Solyc12g098690.1.1	12	54	4	34	4	129	69	190	24	134	45	75	0
Solyc12g098700.1.1	12	53	4	51	2	141	131	208	20	67	43	26	3
Solyc12g098710.1.1	10	96	12	61	0	137	81	205	4	142	8	96	1
Solyc12g098720.1.1	0	0	0	0	0	0	0	0	0	0	0	0	0
Solyc12g098740.1.1	22	569	48	279	24	765	450	878	86	1808	152	887	14
Solyc12g098760.1.1	1	2	0	1	1	1	1	3	4	0	5	0	4
Solyc12g098780.1.1	0	0	0	0	0	0	0	0	0	0	0	0	0
Solyc12g098810.1.1	4	20	0	13	1	41	16	30	4	172	6	212	0
Solyc12g098820.1.1	5	78	5	37	0	236	163	194	59	100	26	76	0
Solyc12g098830.1.1	8	42	8	39	0	105	98	126	8	169	48	239	2
Solyc12g098840.1.1	0	0	0	0	0	0	0	0	0	0	0	0	0
Solyc12g098850.1.1	19	65	32	165	37	160	120	271	50	983	253	34	2
Solyc12g098860.1.1	2	11	0	10	0	11	10	13	2	26	4	8	0
Solyc12g098870.1.1	0	0	0	0	0	0	0	0	0	0	0	0	0
Solyc12g098890.1.1	4	318	33	137	5	27	88	76	8	83	13	65	9
Solyc12g098900.1.1	15	253	2	41	2	1662	611	696	32	4	148	0	0
Solyc12g098910.1.1	2	21	1	6	4	0	11	1	5	2	3	0	0
Solyc12g098920.1.1	18	218	33	143	19	124	134	244	8	188	38	152	2
Solyc12g098930.1.1	0	0	0	0	0	0	0	0	0	0	0	0	0
Solyc12g098940.1.1	0	0	0	0	0	0	0	0	0	0	0	0	0
Solyc12g098950.1.1	9	250	13	113	4	149	76	227	6	432	16	297	0
Solyc12g098960.1.1	3	13	2	10	0	37	14	30	3	6	1	5	0
Solyc12g098970.1.1	0	0	0	0	0	0	0	0	0	0	0	0	0
Solyc12g098980.1.1	5	63	2	40	0	43	53	72	10	107	14	67	2
Solyc12g098990.1.1	1	3	0	0	0	15	8	19	1	13	2	3	0
Solyc12g099000.1.1	13	896	56	373	13	347	246	362	41	1270	269	972	7
Solyc12g099010.1.1	2	3	0	5	0	4	16	10	1	6	0	7	0
Solyc12g099020.1.1	0	0	0	0	0	0	0	0	0	0	0	0	0
Solyc12g099030.1.1	2	227	9	101	5	325	38	487	19	429	36	283	0
Solyc12g099080.1.1	7	71	40	23	14	87	193	107	11	397	350	269	34
Solyc12g099090.1.1	13	64	12	86	14	153	128	245	47	140	84	112	0
Solyc12g099100.1.1	2	9	0	1	0	41	16	49	9	39	12	44	0
Solyc12g099110.1.1	2	2	0	0	0	12	6	16	1	23	6	21	1
Solyc12g099120.1.1	0	0	0	0	0	0	0	0	0	0	0	0	0
Solyc12g099140.1.1	0	0	0	0	0	0	0	0	0	0	0	0	0
Solyc12g099160.1.1	8	9	1	4	0	26	53	40	2	111	34	108	0
Solyc12g099170.1.1	1	2	1	5	0	7	4	10	0	9	3	6	0

Solyc12g099180.1.1	0	0	0	0	0	0	0	0	0	0	0	0	0
Solyc12g099190.1.1	13	51	12	60	1	115	47	84	0	48	522	34	0
Solyc12g099200.1.1	4	115	3	58	0	200	54	415	0	184	14	179	0
Solyc12g099220.1.1	0	0	0	0	0	0	0	0	0	0	0	0	0
Solyc12g099240.1.1	0	0	0	0	0	0	0	0	0	0	0	0	0
Solyc12g099250.1.1	6	46	6	59	2	121	64	132	8	71	6	57	2
Solyc12g099260.1.1	3	35	3	18	0	9	8	20	0	36	9	27	1
Solyc12g099270.1.1	10	96	13	48	2	121	144	182	20	173	40	202	5
Solyc12g099280.1.1	1	1	0	0	0	8	16	3	0	5	0	5	0
Solyc12g099290.1.1	6	93	6	18	3	50	13	26	2	34	25	21	0
Solyc12g099310.1.1	3	128	9	114	1	293	65	212	12	138	58	81	1
Solyc12g099320.1.1	8	43	4	17	1	73	23	88	5	52	7	42	0
Solyc12g099330.1.1	0	0	0	0	0	0	0	0	0	0	0	0	0
Solyc12g099340.1.1	8	10	0	4	1	127	32	82	7	109	43	94	0
Solyc12g099350.1.1	0	0	0	0	0	0	0	0	0	0	0	0	0
Solyc12g099360.1.1	18	6	0	5	0	6	16	16	0	576	138	738	2
Solyc12g099370.1.1	6	13	5	0	1	14	51	17	13	33	16	41	0
Solyc12g099390.1.1	4	54	8	30	0	42	47	72	7	161	45	113	8
Solyc12g099400.1.1	6	40	4	27	3	67	28	43	3	17	20	42	5
Solyc12g099420.1.1	0	0	0	0	0	0	0	0	0	0	0	0	0
Solyc12g099430.1.1	7	22	10	28	5	242	163	95	29	142	12	338	0
Solyc12g099440.1.1	22	593	66	373	61	4545	2058	4035	300	1434	584	1042	26
Solyc12g099470.1.1	3	23	1	17	1	60	18	76	3	52	6	39	2
Solyc12g099490.1.1	1	14	0	0	0	15	2	15	0	10	8	19	0
Solyc12g099500.1.1	3	209	14	101	5	1	5	6	2	125	19	243	22
Solyc12g099510.1.1	5	67	6	80	1	134	48	188	10	71	22	141	4
Solyc12g099530.1.1	10	11	2	19	1	54	41	57	3	22	8	37	0
Solyc12g099540.1.1	0	0	0	0	0	0	0	0	0	0	0	0	0
Solyc12g099550.1.1	0	0	0	0	0	0	0	0	0	0	0	0	0
Solyc12g099560.1.1	4	18	1	13	0	41	20	32	0	10	1	10	1
Solyc12g099570.1.1	0	0	0	0	0	0	0	0	0	0	0	0	0
Solyc12g099580.1.1	0	0	0	0	0	0	0	0	0	0	0	0	0
Solyc12g099590.1.1	0	0	0	0	0	0	0	0	0	0	0	0	0
Solyc12g099600.1.1	1	1	0	3	0	2	6	2	0	5	0	3	0
Solyc12g099620.1.1	0	0	0	0	0	0	0	0	0	0	0	0	0
Solyc12g099630.1.1	1	1	1	0	0	2	0	6	0	2	3	7	0
Solyc12g099640.1.1	0	0	0	0	0	0	0	0	0	0	0	0	0
Solyc12g099650.1.1	4	3770	312	1368	5	32	399	57	8	200	94	156	13
Solyc12g099660.1.1	14	76	8	69	3	141	64	221	9	218	63	243	1
Solyc12g099700.1.1	11	43	3	24	5	54	45	111	6	86	43	63	0
Solyc12g099710.1.1	1	4	0	3	0	8	7	5	2	12	7	13	0

Solyc12g099740.1.1	6	56	7	19	4	27	51	57	9	50	4	51	2
Solyc12g099750.1.1	0	0	0	0	0	0	0	0	0	0	0	0	0
Solyc12g099760.1.1	9	44	5	31	5	152	64	111	23	96	64	51	5
Solyc12g099770.1.1	4	29	2	20	0	44	42	85	38	89	15	51	0
Solyc12g099780.1.1	0	0	0	0	0	0	0	0	0	0	0	0	0
Solyc12g099790.1.1	0	0	0	0	0	0	0	0	0	0	0	0	0
Solyc12g099800.1.1	2	25	2	13	0	12	9	12	0	34	1	29	0
Solyc12g099810.1.1	1	1	0	0	0	1	1	2	0	3	0	3	0
Solyc12g099820.1.1	1	3	0	0	0	1	6	7	1	14	2	6	0
Solyc12g099830.1.1	1	5	0	1	0	1	15	7	0	3	0	1	0
Solyc12g099840.1.1	6	5	1	2	0	25	17	27	0	7	4	13	0
Solyc12g099850.1.1	0	0	0	0	0	0	0	0	0	0	0	0	0
Solyc12g099860.1.1	0	0	0	0	0	0	0	0	0	0	0	0	0
Solyc12g099870.1.1	0	0	0	0	0	0	0	0	0	0	0	0	0
Solyc12g099890.1.1	7	32	6	24	4	127	61	103	18	63	18	25	0
Solyc12g099900.1.1	4	34	0	10	0	116	62	142	9	57	14	15	3
Solyc12g099910.1.1	20	111	12	80	1	205	104	284	19	179	59	197	5
Solyc12g099920.1.1	5	19	1	14	1	105	52	82	10	49	21	43	1
Solyc12g099930.1.1	6	2881	304	3145	15	1247	705	746	8	165	117	532	4
Solyc12g099940.1.1	3	4	1	0	1	10	27	24	1	21	3	4	0
Solyc12g099950.1.1	0	0	0	0	0	0	0	0	0	0	0	0	0
Solyc12g099960.1.1	1	2	0	0	3	0	5	4	0	8	1	18	0
Solyc12g099970.1.1	8	94	16	41	5	60	181	144	6	149	22	118	2
Solyc12g099980.1.1	0	0	0	0	0	0	0	0	0	0	0	0	0
Solyc12g099990.1.1	2	269	16	111	16	272	84	172	26	623	87	609	18
Solyc12g100000.1.1	8	32	7	28	1	153	30	107	3	64	13	61	0
Solyc12g100010.1.1	0	0	0	0	0	0	0	0	0	0	0	0	0
Solyc12g100030.1.1	0	0	0	0	0	0	0	0	0	0	0	0	0
Solyc12g100040.1.1	8	35	1	33	3	50	47	56	5	33	22	29	1
Solyc12g100050.1.1	3	27	5	5	0	40	15	56	7	150	26	47	4
Solyc12g100060.1.1	5	366	63	250	40	276	330	286	46	2244	216	1158	38
Solyc12g100070.1.1	0	0	0	0	0	0	0	0	0	0	0	0	0
Solyc12g100120.1.1	18	297	19	180	18	156	138	386	18	270	95	278	6
Solyc12g100130.1.1	0	0	0	0	0	0	0	0	0	0	0	0	0
Solyc12g100140.1.1	7	89	8	44	8	173	36	99	16	119	16	110	0
Solyc12g100160.1.1	5	363	30	124	0	30	139	143	3	93	13	64	1
Solyc12g100170.1.1	0	0	0	0	0	0	0	0	0	0	0	0	0
Solyc12g100180.1.1	0	0	0	0	0	0	0	0	0	0	0	0	0
Solyc12g100190.1.1	0	0	0	0	0	0	0	0	0	0	0	0	0
Solyc12g100200.1.1	21	215	26	196	13	572	367	640	29	223	77	148	6
Solyc12g100220.1.1	0	0	0	0	0	0	0	0	0	0	0	0	0

Solyc12g100230.1.1	0	0	0	0	0	0	0	0	0	0	0	0	0
Solyc12g100250.1.1	1	4	4	0	1	0	0	5	5	5	1	0	0
Solyc12g100260.1.1	0	0	0	0	0	0	0	0	0	0	0	0	0
Solyc12g100270.1.1	0	0	0	0	0	0	0	0	0	0	0	0	0
Solyc12g100290.1.1	0	0	0	0	0	0	0	0	0	0	0	0	0
Solyc12g100310.1.1	3	17	0	6	0	13	22	23	0	33	9	22	0
Solyc12g100320.1.1	2	2	0	2	0	3	9	14	7	22	4	6	11
Solyc12g100330.1.1	4	28	3	9	2	20	11	28	3	122	19	60	2
Solyc12g100350.1.1	0	0	0	0	0	0	0	0	0	0	0	0	0
Solyc12g100360.1.1	8	37	9	33	6	43	77	75	17	87	15	76	2
