# Supplementary material for: Using a periclinal chimera to unravel layer-specific gene expression in plants
Source: Plant J. 2013 Jul 19;75(6):1039–49. doi: 10.1111/tpj.12250 (PMC4223383; doi:10.1111/tpj.12250)
Supplement: Supplementary file 12 [file tpj0075-1039-sd12.pdf]

Supplemental Table S5. List of genes classified as L1, either specific or related.

Within each class, genes are ordered with decreasing fold change with respect to the interaction effect: i.e. the ratio of the difference of penn allele-specific expression between wild- ype and chimera over the difference for lyc expresssion. Among all tissue samples, the highest fold change is reported. For each gene, we report which tissues support the classification as L1: L(eaf), D(ehydrated leaf), F(ruit). Fold change, p-values, tissue support and classification as related or specific originate from the differential expression analysis conducted on allele-specific expression values based on polymorphims detected by Varid. After correcting for multiple testing using the Benjamini and Hochberg approach (P adjusted value), a cut-off value of 0.05 was applied for the false discovery rate.

Gene ID	log fold change	P value	P adjusted value	Prediction	Tissues	Human readable description
Solyc07g054310.1.1	-9.788099205	2.36E-69	1.62E-65	L1-specific	F	Gty37 protein (AHRD V1 ***- Q65A67_GERHY)
Solyc11g006250.1.1	-9.394289788	2.70E-73	3.70E-69	L1-specific	L,F	GDSL esterase/lipase At5g33370 (AHRD V1 ***- GDL79_ARATH)%3B contains Interpro domain(s) IPR001087 Lipase%2C GDSL
Solyc11g008630.1.1	-9.230585959	2.14E-25	1.95E-22	L1-specific	L,D,F	Hydroxycinnamoyl CoA shikimate/quinate hydroxycinnamoyltransferase-like protein (Fragment) (AHRD V1 **-- B9GF60_POPTR)%3B contains Interpro domain(s) IPR003480 Transferase
Solyc02g077330.2.1	-9.067922983	1.97E-16	4.83E-14	L1-specific	L,D,F	GDSL esterase/lipase At5g45950 (AHRD V1 ***- GDL85_ARATH)%3B contains Interpro domain(s) IPR001087 Lipase%2C GDSL
Solyc06g074390.2.1	-8.802207524	5.88E-26	1.86E-23	L1-specific	D	Fatty acyl coA reductase (AHRD V1 **** Q8L4M0_WHEAT)%3B contains Interpro domain(s) IPR013120 Male sterility%2C NAD-binding
Solyc10g075100.1.1	-8.542791888	1.81E-72	2.51E-68	L1-specific	L,D,F	Non-specific lipid-transfer protein (AHRD V1 **** Q4A1N1_SOLLC)%3B contains Interpro domain(s) IPR000528 Plant lipid transfer protein/Par allergen
Solyc01g105450.2.1	-8.444204607	2.11E-13	1.60E-11	L1-specific	D	ABC transporter G family member 11 (AHRD V1 ***- AB11G_ARATH)%3B contains Interpro domain(s) IPR013525 ABC-2 type transporter
Solyc07g005900.2.1	-8.426960466	4.27E-07	3.27E-05	L1-specific	F	Calcineurin subunit B (AHRD V1 ***- B6T814_MAIZE)%3B contains Interpro domain(s) IPR011992 EF-Hand type
Solyc07g052950.2.1	-8.112285678	7.88E-12	4.58E-10	L1-specific	D	Unknown Protein (AHRD V1)
Solyc08g067260.2.1	-8.105706358	1.58E-29	2.40E-26	L1-specific	L,D,F	Fatty acid elongase 3-ketoacyl-CoA synthase (AHRD V1 **** Q6DUV6_BRANA)%3B contains Interpro domain(s) IPR012392 Very-long-chain 3-ketoacyl-CoA synthase
Solyc07g049440.2.1	-8.088072683	4.33E-21	2.20E-18	L1-specific	L,D,F	GDSL esterase/lipase At2g04570 (AHRD V1 ***- GDL34_ARATH)%3B contains Interpro domain(s) IPR001087 Lipase%2C GDSL
Solyc09g018010.2.1	-7.976891957	4.73E-23	4.64E-20	L1-specific	L,F	Non-specific lipid-transfer protein (AHRD V1 **** Q8H6L4_SOLTU)%3B contains Interpro domain(s) IPR013770 Plant lipid transfer protein and hydrophobic protein%2C helical
Solyc11g010960.1.1	-7.928475619	1.35E-25	1.32E-22	L1-specific	L,F	Alcohol dehydrogenase (AHRD V1 ***- ADH2_BACST)%3B contains Interpro domain(s) IPR002085 Alcohol dehydrogenase superfamily%2C zinc-containing
Solyc08g081220.1.1	-7.78700673	3.71E-50	1.70E-46	L1-specific	L,F	Cytochrome P450
Solyc06g075330.1.1	-7.721495725	6.40E-12	3.77E-10	L1-specific	D	LOB domain protein 1 (AHRD V1 *-*- D7KHW8_ARALY)%3B contains Interpro domain(s) IPR004883 Lateral organ boundaries%2C LOB
Solyc08g007210.2.1	-7.658959999	4.89E-14	8.51E-12	L1-specific	L,D	Hydroxycinnamoyl CoA shikimate/quinate hydroxycinnamoyltransferase-like protein (AHRD V1 *--- B9IAS9_POPTR)%3B contains Interpro domain(s) IPR003480 Transferase
Solyc03g121180.2.1	-7.614363807	3.72E-31	7.29E-28	L1-specific	L,D,F	GDSL esterase/lipase At5g22810 (AHRD V1 ***- GDL78_ARATH)%3B contains Interpro domain(s) IPR001087 Lipase%2C GDSL
Solyc05g055330.2.1	-7.587120552	7.28E-18	1.05E-15	L1-specific	D	ATP-binding cassette transporter (AHRD V1 ***- D8RL77_SELML)%3B contains Interpro domain(s) IPR013525 ABC-2 type transporter
Solyc06g035580.2.1	-7.549084747	9.18E-46	3.15E-42	L1-specific	L,F	Choline dehydrogenase (AHRD V1 ***- C0G363_9RHIZ)%3B contains Interpro domain(s) IPR012132 Glucose-methanol-choline oxidoreductase
Solyc06g082570.1.1	-7.528719784	1.99E-06	4.36E-05	L1-specific	L,D	Unknown Protein (AHRD V1)%3B contains Interpro domain(s) IPR011684 KIP1-like
Solyc01g100760.2.1	-7.389152115	1.62E-43	3.75E-40	L1-specific	D	Susceptibility homeodomain transcription factor (Fragment) (AHRD V1 *-*- Q8SAA7_ORYSA)%3B contains Interpro domain(s) IPR007493 Protein of unknown function DUF538
Solyc08g068150.2.1	-7.349044628	1.81E-58	8.38E-55	L1-specific	L,D,F	BURP domain-containing protein (AHRD V1 **-- B2ZPK7_SOLLC)%3B contains Interpro domain(s) IPR004873 BURP
Solyc10g075090.1.1	-7.330233728	1.21E-53	4.19E-50	L1-specific	L,D,F	Non-specific lipid-transfer protein (AHRD V1 **** Q4A1N0_SOLLC)%3B contains Interpro domain(s) IPR013770 Plant lipid transfer protein and hydrophobic protein%2C helical
Solyc09g057630.2.1	-7.244550988	6.17E-09	4.98E-07	L1-specific	L	Glucan endo-1 3-beta-glucosidase A6 (AHRD V1 ***- B6TIF7_MAIZE)%3B contains Interpro domain(s) IPR000490 Glycoside hydrolase%2C family 17
Solyc01g006300.2.1	-7.124116852	8.14E-41	1.51E-37	L1-specific	L,D	Peroxidase (AHRD V1 **** Q43774_SOLLC)%3B contains Interpro domain(s) IPR002016 Haem peroxidase%2C plant/fungal/bacterial
Solyc03g111550.2.1	-7.000171763	8.69E-41	1.51E-37	L1-specific	D,F	GDSL esterase/lipase At3g48460 (AHRD V1 ***- GDL57_ARATH)%3B contains Interpro domain(s) IPR001087 Lipase%2C GDSL
Solyc10g005330.2.1	-6.986534657	4.52E-08	2.94E-06	L1-specific	L,D,F	Homeobox-leucine zipper protein ATHB-14 (AHRD V1 ***- ATB14_ARATH)%3B contains Interpro domain(s) IPR002913 Lipid-binding START
Solyc11g065530.1.1	-6.930082347	1.97E-21	4.42E-19	L1-specific	D	Lipase (Fragment) (AHRD V1 ***- Q9ZTW1_DIACA)%3B contains Interpro domain(s) IPR002921 Lipase%2C class 3
Solyc04g080750.2.1	-6.915987517	1.29E-12	8.51E-11	L1-specific	D	Transmembrane protein 136 (AHRD V1 ***- B9EI42_MOUSE)%3B contains Interpro domain(s) IPR006634 TRAM%2C LAG1 and CLN8 homology
Solyc10g083140.1.1	-6.868681802	7.82E-14	6.59E-12	L1-specific	D	Unknown Protein (AHRD V1)
Solyc09g092270.2.1	-6.862266962	5.16E-30	3.12E-27	L1-specific	D	Hydroxycinnamoyl-CoA shikimate/quinate hydroxycinnamoyl transferase (AHRD V1 *--* D1GJ93_COFAR)%3B contains Interpro domain(s) IPR003480 Transferase
Solyc11g072990.1.1	-6.856081765	1.47E-33	1.28E-30	L1-specific	D,F	Fatty acid elongase 3-ketoacyl-CoA synthase (AHRD V1 **** Q6DUV5_BRANA)%3B contains Interpro domain(s) IPR012392 Very-long-chain 3-ketoacyl-CoA synthase
Solyc09g097780.2.1	-6.84068379	1.81E-39	6.76E-36	L1-specific	L,D,F	Glycine-rich protein (AHRD V1 *-*- D2K2U2_TOBAC)%3B contains Interpro domain(s) IPR010800 Glycine rich
Solyc07g006300.2.1	-6.829513488	2.86E-18	9.12E-16	L1-specific	L,D,F	CER1 protein (Fragment) (AHRD V1 *--- Q0WLZ2_ARATH)%3B contains Interpro domain(s) IPR006694 Fatty acid hydroxylase
Solyc04g050490.2.1	-6.828323029	3.00E-16	1.21E-13	L1-specific	L,F	Carboxyl-terminal proteinase (AHRD V1 ***- B6SL47_MAIZE)%3B contains Interpro domain(s) IPR004314 Protein of unknown function DUF239%2C plant
Solyc01g094700.2.1	-6.816609655	1.52E-20	6.96E-18	L1-specific	L,D,F	Glycerol-3-phosphate acyltransferase 4 (AHRD V1 **** D7KQ54_ARALY)%3B contains Interpro domain(s) IPR002123 Phospholipid/glycerol acyltransferase
Solyc09g014350.2.1	-6.719821574	8.24E-39	1.88E-35	L1-specific	L,D,F	Glycerol-3-phosphate acyltransferase 6 (AHRD V1 **** D7LKT2_ARALY)%3B contains Interpro domain(s) IPR002123 Phospholipid/glycerol acyltransferase
Solyc01g098490.2.1	-6.713189722	2.72E-13	2.04E-11	L1-specific	D	Solute carrier family 2 facilitated glucose transporter member 8 (AHRD V1 ***- B6SSR3_MAIZE)%3B contains Interpro domain(s) IPR003663 Sugar/inositol transporter
Solyc11g065080.1.1	-6.664606562	1.79E-05	0.000621783	L1-specific	L	S2 self-incompatibility locus-linked pollen 3.2 protein (AHRD V1 ***- Q7XAE8_PETIN)
Solyc08g076370.2.1	-6.633689344	4.30E-08	1.37E-06	L1-specific	D	Homeobox-leucine zipper protein ATHB-9 (AHRD V1 *-*- ATBH9_ARATH)%3B contains Interpro domain(s) IPR002913 Lipid-binding START
Solyc12g009000.1.1	-6.608154252	0.00024484	0.002764226	L1-specific	D	Os07g0175100 protein (Fragment) (AHRD V1 ***- Q0D898_ORYSJ)
Solyc06g074260.2.1	-6.59837108	2.31E-44	6.34E-41	L1-specific	L,F	Genomic DNA chromosome 5 P1 clone MWD9 (AHRD V1 *-*- Q9FMQ8_ARATH)%3B contains Interpro domain(s) IPR007608 Protein of unknown function DUF584
Solyc06g053260.1.1	-6.571628376	6.82E-27	2.56E-24	L1-specific	L,D	Auxin-responsive family protein (AHRD V1 ***- D7LF78_ARALY)%3B contains Interpro domain(s) IPR003676 Auxin responsive SAUR protein
Solyc01g095940.2.1	-6.550175848	4.15E-23	1.09E-20	L1-specific	D	O-acyltransferase WSD1 (AHRD V1 **-- WSD1_ARATH)%3B contains Interpro domain(s) IPR009721 Protein of unknown function DUF1298
Solyc06g009770.1.1	-6.528609668	1.84E-24	1.94E-21	L1-specific	D,F	Binding protein (AHRD V1 ***- D7LT81_ARALY)
Solyc05g056020.2.1	-6.512821203	8.46E-08	8.06E-06	L1-specific	F	V-type proton ATPase subunit G 2 (AHRD V1 **** VATG2_TOBAC)%3B contains Interpro domain(s) IPR005124 Vacuolar (H+)-ATPase G subunit
Solyc03g113950.2.1	-6.507391275	2.00E-13	1.54E-11	L1-specific	L,D	Calmodulin-binding protein (AHRD V1 **** Q9FKL6_ARATH)%3B contains Interpro domain(s) IPR012416 Calmodulin binding protein-like
Solyc09g083050.2.1	-6.419494064	1.17E-11	1.48E-09	L1-specific	L,D	Fatty acid elongase 3-ketoacyl-CoA synthase (AHRD V1 **** Q6DUV5_BRANA)%3B contains Interpro domain(s) IPR012392 Very-long-chain 3-ketoacyl-CoA synthase
Solyc03g025680.2.1	-6.370654938	5.52E-11	2.87E-09	L1-specific	D	PAR-1c protein (AHRD V1 ***- Q43589_TOBAC)%3B contains Interpro domain(s) IPR009489 PAR1
Solyc07g053140.2.1	-6.363041754	0.00069325	0.006563172	L1-specific	D	CONSTANS-like zinc finger protein (AHRD V1 *-*- D0EP07_SOYBN)%3B contains Interpro domain(s) IPR000315 Zinc finger%2C B-box
Solyc08g080190.2.1	-6.345430738	4.12E-26	1.36E-23	L1-specific	L,D,F	Choline dehydrogenase (AHRD V1 ***- A8NUQ9_COPC7)%3B contains Interpro domain(s) IPR012132 Glucose-methanol-choline oxidoreductase
Solyc11g066390.1.1	-6.270017486	1.26E-09	1.16E-07	L1-specific	L	Superoxide dismutase (AHRD V1 ***- Q7XAV2_SOLLC)%3B contains Interpro domain(s) IPR018152 Superoxide dismutase%2C copper/zinc%2C binding site
Solyc02g088090.1.1	-6.26350608	3.74E-09	1.47E-07	L1-specific	D	Calmodulin-like protein (AHRD V1 *-*- B6TXW9_MAIZE)%3B contains Interpro domain(s) IPR011992 EF-Hand type
Solyc05g055400.2.1	-6.175568481	4.59E-10	4.50E-08	L1-specific	L,D,F	Cytochrome P450
Solyc11g072030.1.1	-6.175068141	5.13E-11	9.26E-09	L1-specific	L,D,F	Non-specific lipid-transfer protein-like protein (AHRD V1 ***- C0KHK0_9CARY)%3B contains Interpro domain(s) IPR003612 Plant lipid transfer protein/seed storage/trypsin-alpha amylase inhibitor
Solyc03g025320.2.1	-6.11965974	8.45E-28	9.67E-25	L1-specific	L,D,F	Hydroxycinnamoyl transferase (AHRD V1 **-* D2XJ64_9MAGN)%3B contains Interpro domain(s) IPR003480 Transferase
Solyc10g080450.1.1	-6.077551635	2.47E-07	1.96E-05	L1-specific	F	Unknown Protein (AHRD V1)
Solyc07g008140.2.1	-6.061610091	0.00198967	0.034077684	L1-specific	D,F	Blue copper protein (Fragment) (AHRD V1 *--- O82576_MAIZE)%3B contains Interpro domain(s) IPR003245 Plastocyanin-like
Solyc09g075770.1.1	-6.028057506	1.10E-19	2.03E-17	L1-specific	D	Long-chain fatty acid CoA ligase (AHRD V1 **** Q9GLP3_CALJA)%3B contains Interpro domain(s) IPR000873 AMP-dependent synthetase and ligase
Solyc04g014220.1.1	-6.023191751	0.00015625	0.001872096	L1-specific	D	RING finger protein (AHRD V1 *-*- C6EUD3_SOYBN)%3B contains Interpro domain(s) IPR018957 Zinc finger%2C C3HC4 RING-type
Solyc00g006820.2.1	-5.954113179	2.87E-09	2.45E-07	L1-specific	L,D,F	Peptide transporter-like protein (AHRD V1 **-* Q9LYR6_ARATH)%3B contains Interpro domain(s) IPR000109 TGF-beta receptor%2C type I/II extracellular region
Solyc10g009240.2.1	-5.936582825	5.22E-38	7.25E-35	L1-specific	L,D	Fatty acid elongase 3-ketoacyl-CoA synthase (AHRD V1 **** Q6DUV6_BRANA)%3B contains Interpro domain(s) IPR012392 Very-long-chain 3-ketoacyl-CoA synthase
Solyc01g095750.2.1	-5.930082347	0.00020157	0.002339909	L1-specific	D	Long-chain-fatty-acid-CoA ligase (AHRD V1 **** B2WS80_ARAHA)%3B contains Interpro domain(s) IPR000873 AMP-dependent synthetase and ligase
Solyc04g007820.2.1	-5.923041733	2.47E-12	3.61E-10	L1-specific	L	Major latex-like protein (AHRD V1 **-- Q9AXU0_PRUPE)%3B contains Interpro domain(s) IPR000916 Bet v I allergen
Solyc08g080000.2.1	-5.913359133	0.00028521	0.007763585	L1-specific	F	Unknown Protein (AHRD V1)%3B contains Interpro domain(s) IPR016146 Calponin-homology
Solyc05g012920.1.1	-5.868965013	2.52E-06	0.000153522	L1-specific	F	Pentatricopeptide repeat-containing protein (AHRD V1 ***- D7LEU3_ARALY)%3B contains Interpro domain(s) IPR002885 Pentatricopeptide repeat
Solyc03g097170.2.1	-5.828926412	1.63E-27	6.28E-25	L1-specific	L,D	Cinnamoyl-CoA reductase-like protein (AHRD V1 ***- Q9M0B3_ARATH)%3B contains Interpro domain(s) IPR016040 NAD(P)-binding domain
Solyc01g088400.2.1	-5.780083779	2.30E-13	3.71E-11	L1-specific	L,D	CER1 (AHRD V1 **-- B6TFH3_MAIZE)%3B contains Interpro domain(s) IPR006694 Fatty acid hydroxylase
Solyc04g010200.1.1	-5.739781209	2.80E-10	1.31E-08	L1-specific	D	ABC transporter G family member 6 (AHRD V1 ***- AB6G_ARATH)%3B contains Interpro domain(s) IPR013525 ABC-2 type transporter
Solyc11g065350.1.1	-5.726138325	7.32E-33	5.65E-30	L1-specific	L,D,F	ABC transporter G family member 15 (AHRD V1 ***- AB15G_ARATH)%3B contains Interpro domain(s) IPR003439 ABC transporter-like
Solyc06g068800.2.1	-5.723500251	0.00016226	0.004073023	L1-specific	L	CRAL/TRIO domain containing protein expressed (AHRD V1 **-- Q75GU9_ORYSJ)%3B contains Interpro domain(s) IPR001251 Cellular retinaldehyde-binding/triple function%2C C-terminal
Solyc03g019760.2.1	-5.709367199	1.06E-34	1.05E-31	L1-specific	L,D,F	ABC transporter G family member 11 (AHRD V1 ***- AB11G_ARATH)%3B contains Interpro domain(s) IPR013525 ABC-2 type transporter
Solyc07g007800.2.1	-5.692720193	7.37E-24	2.01E-21	L1-specific	D	Adiponectin receptor (AHRD V1 ***- Q16FL3_AEDAE)%3B contains Interpro domain(s) IPR004254 Hly-III related
Solyc04g081770.2.1	-5.631499946	8.77E-31	5.54E-28	L1-specific	L,D	GDSL esterase/lipase At5g42170 (AHRD V1 ***- GDL90_ARATH)%3B contains Interpro domain(s) IPR001087 Lipase%2C GDSL
Solyc03g083240.2.1	-5.611025521	0.00015368	0.003914973	L1-specific	L	Transcription factor jumonji domain-containing protein (AHRD V1 **-* D7M504_ARALY)%3B contains Interpro domain(s) IPR013129 Transcription factor jumonji
Solyc11g007250.1.1	-5.605930608	8.73E-05	0.003024839	L1-specific	F	Protein kinase like protein (AHRD V1 ***- Q0WN21_ARATH)%3B contains Interpro domain(s) IPR002290 Serine/threonine protein kinase
Solyc11g007540.1.1	-5.558862354	1.01E-07	9.11E-06	L1-specific	L,F	Cytochrome P450
Solyc03g117800.2.1	-5.548518769	3.30E-34	3.05E-31	L1-specific	L,D,F	CER1 protein (Fragment) (AHRD V1 *--- Q0WLZ2_ARATH)%3B contains Interpro domain(s) IPR006694 Fatty acid hydroxylase
Solyc12g014450.1.1	-5.534620386	5.92E-13	4.20E-11	L1-specific	D	Extracellular ligand-gated ion channel (AHRD V1 ***- D7LAY2_ARALY)
Solyc02g063140.2.1	-5.512603468	5.62E-05	0.001674068	L1-specific	L	Fatty acid elongase 3-ketoacyl-CoA synthase (AHRD V1 **** Q6DUV5_BRANA)%3B contains Interpro domain(s) IPR012392 Very-long-chain 3-ketoacyl-CoA synthase
Solyc05g018510.2.1	-5.512150026	6.66E-16	2.54E-13	L1-specific	L,F	ABC transporter G family member 32 (AHRD V1 ***- AB32G_ARATH)%3B contains Interpro domain(s) IPR013525 ABC-2 type transporter

Solyc10g075070.1.1	-5.498221208	1.25E-33	2.87E-30	L1-specific	L,D,F	Non-specific lipid-transfer protein (AHRD V1 ***- A7UGG9_SOLTU)%3B contains Interpro domain(s) IPR013770 Plant lipid transfer protein and hydrophobic protein%2C helical
Solyc01g099910.2.1	-5.467976594	1.80E-09	7.31E-08	L1-specific	D	Epoxide hydrolase (AHRD V1 ***- B3VMR4_NICBE)%3B contains Interpro domain(s) IPR000639 Epoxide hydrolase-like
Solyc02g071220.2.1	-5.43822925	5.37E-07	1.34E-05	L1-specific	D	Response regulator 5 (AHRD V1 ***- Q9FRZ0_MAIZE)%3B contains Interpro domain(s) IPR001789 Signal transduction response regulator%2C receiver region
Solyc05g015490.2.1	-5.438109742	8.78E-15	1.72E-12	L1-specific	L,D	Non-specific lipid-transfer protein (AHRD V1 ***- Q700A6_CICAR)%3B contains Interpro domain(s) IPR003612 Plant lipid transfer protein/seed storage/trypsin-alpha amylase inhibitor
Solyc08g078550.1.1	-5.388028437	4.07E-18	6.08E-16	L1-specific	D	Matrix metalloproteinase (AHRD V1 **-- D7LBU9_ARALY)%3B contains Interpro domain(s) IPR001818 Peptidase M10A and M12B%2C matrixin and adamalysin
Solyc04g049090.2.1	-5.337944794	2.20E-19	3.83E-17	L1-specific	D	MLO-like protein 17 (AHRD V1 ***- C6EWF0_VITVI)%3B contains Interpro domain(s) IPR004326 Mlo-related protein
Solyc02g084980.2.1	-5.314193874	1.52E-26	5.41E-24	L1-specific	D	Galactinol synthase (AHRD V1 ***- C7G304_SOLLC)%3B contains Interpro domain(s) IPR002495 Glycosyl transferase%2C family 8
Solyc08g074620.1.1	-5.283516394	0.00018314	0.004522738	L1-specific	L	Polyphenol oxidase (AHRD V1 ***- Q41428_SOLTU)%3B contains Interpro domain(s) IPR016213 Polyphenol oxidase%2C plant
Solyc03g007790.2.1	-5.254146186	1.68E-18	2.59E-16	L1-specific	D	Receptor-like protein kinase (AHRD V1 **** Q9FLV4_ARATH)%3B contains Interpro domain(s) IPR002290 Serine/threonine protein kinase
Solyc02g084840.2.1	-5.184667916	1.11E-28	5.12E-26	L1-specific	D	Dehydrin DHN1 (AHRD V1 *-*- DHN1_PEA)%3B contains Interpro domain(s) IPR000167 Dehydrin
Solyc03g097500.2.1	-5.152474768	1.21E-08	4.33E-07	L1-specific	D	Hydroxycinnamoyl CoA shikimate/quinate hydroxycinnamoyltransferase-like protein (AHRD V1 **-* B9IFG0_POPTR)%3B contains Interpro domain(s) IPR003480 Transferase
Solyc02g079640.2.1	-5.15004803	2.38E-08	8.01E-07	L1-specific	D	Serine/threonine-protein kinase receptor (AHRD V1 **** B6U2B7_MAIZE)%3B contains Interpro domain(s) IPR000858 S-locus glycoprotein
Solyc02g071610.2.1	-5.137858675	6.89E-14	1.17E-11	L1-specific	L	GDSL esterase/lipase At5g45670 (AHRD V1 ***- GDL82_ARATH)%3B contains Interpro domain(s) IPR001087 Lipase%2C GDSL
Solyc09g082570.2.1	-5.124729777	3.39E-21	7.27E-19	L1-specific	L,D	Neurogenic locus notch protein-like (AHRD V1 ***- B6SSE6_MAIZE)
Solyc03g065250.2.1	-5.110523374	3.15E-21	1.66E-18	L1-specific	L,D	CER1 (AHRD V1 **-- B6TFH3_MAIZE)%3B contains Interpro domain(s) IPR006694 Fatty acid hydroxylase
Solyc06g084190.2.1	-5.076784807	1.99E-13	1.54E-11	L1-specific	D	Non-specific lipid-transfer protein (AHRD V1 ***- B9NBN9_POPTR)%3B contains Interpro domain(s) IPR013770 Plant lipid transfer protein and hydrophobic protein%2C helical
Solyc01g005990.2.1	-5.067936977	1.58E-16	3.94E-14	L1-specific	L,F	Non-specific lipid-transfer protein (AHRD V1 *-*- B9REN2_RICCO)%3B contains Interpro domain(s) IPR003612 Plant lipid transfer protein/seed storage/trypsin-alpha amylase inhibitor
Solyc10g075110.1.1	-5.0606128	6.82E-22	4.07E-19	L1-specific	L,D	Non-specific lipid-transfer protein (AHRD V1 ***- A7UGG9_SOLTU)%3B contains Interpro domain(s) IPR000528 Plant lipid transfer protein/Par allergen
Solyc02g083860.2.1	-5.006998759	2.65E-09	2.29E-07	L1-specific	L,D	Flavanone 3-hydroxylase (AHRD V1 ***- A9ZMI9_TOBAC)%3B contains Interpro domain(s) IPR005123 Oxoglutarate and iron-dependent oxygenase
Solyc01g111160.2.1	-4.995933355	6.62E-21	3.25E-18	L1-specific	L,D,F	Unknown Protein (AHRD V1)
Solyc06g073360.2.1	-4.945892672	0.00050695	0.010709044	L1-specific	L	Unknown Protein (AHRD V1)
Solyc07g006770.2.1	-4.936855575	1.10E-12	7.44E-11	L1-specific	D	Receptor like kinase%2C RLK
Solyc06g059840.2.1	-4.873386213	3.24E-12	1.99E-10	L1-specific	D,F	3-methyl-2-oxobutanoate dehydrogenase (2-methylpropanoyl-transferring) (AHRD V1 **** A1U0F1_MARAV)%3B contains Interpro domain(s) IPR001017 Dehydrogenase%2C E1 component
Solyc11g067190.1.1	-4.871146294	1.65E-22	4.17E-20	L1-specific	D	Fatty acyl coA reductase (AHRD V1 ***- Q8L4M0_WHEAT)%3B contains Interpro domain(s) IPR013120 Male sterility%2C NAD-binding
Solyc07g053890.2.1	-4.864429926	1.35E-22	3.47E-20	L1-specific	D	O-acyltransferase WSD1 (AHRD V1 **-- WSD1_ARATH)%3B contains Interpro domain(s) IPR009721 Protein of unknown function DUF1298
Solyc01g109180.2.1	-4.849802756	2.61E-17	1.12E-14	L1-specific	L,F	Long-chain-fatty-acid-CoA ligase (AHRD V1 ***- B2WS80_ARAHA)%3B contains Interpro domain(s) IPR000873 AMP-dependent synthetase and ligase
Solyc03g116160.1.1	-4.834531563	0.00144913	0.024687274	L1-specific	L	Phytochrome kinase substrate 1 (AHRD V1 ***- D7LQB9_ARALY)
Solyc02g079280.2.1	-4.827442304	1.41E-18	2.25E-16	L1-specific	D	MYB transcription factor (AHRD V1 **-* Q9LDR8_ARATH)%3B contains Interpro domain(s) IPR015495 Myb transcription factor
Solyc04g007530.2.1	-4.826861626	1.61E-12	1.04E-10	L1-specific	D	Multidrug resistance protein mdtK (AHRD V1 ***- MDTK_YERE8)%3B contains Interpro domain(s) IPR015521 MATE family transporter related protein
Solyc01g094990.2.1	-4.804551464	0.0005831	0.005702989	L1-specific	D	Glycosyltransferase-like protein (AHRD V1 **-- D8RX86_SELML)%3B contains Interpro domain(s) IPR006740 Protein of unknown function DUF604
Solyc04g074950.2.1	-4.804551464	8.90E-07	2.12E-05	L1-specific	D	Ovarian cancer-associated gene 2 protein homolog (AHRD V1 **-- B5X7T4_SALSA)%3B contains Interpro domain(s) IPR005645 Protein of unknown function DUF341
Solyc09g082300.2.1	-4.794020797	1.22E-08	4.35E-07	L1-specific	D	Non-specific lipid-transfer protein (AHRD V1 *-*- B9GSL1_POPTR)%3B contains Interpro domain(s) IPR003612 Plant lipid transfer protein/seed storage/trypsin-alpha amylase inhibitor
Solyc03g120260.2.1	-4.745786795	5.60E-16	6.18E-14	L1-specific	D	Coatomer beta%26apos subunit (AHRD V1 ***- C4YSS2_CANAW)%3B contains Interpro domain(s) IPR020472 G-protein beta WD-40 repeat%2C region
Solyc06g053730.1.1	-4.707689925	0.00619788	0.037386332	L1-specific	D	Serine/threonine protein kinase (AHRD V1 **** Q16PN5_AEDAE)%3B contains Interpro domain(s) IPR002290 Serine/threonine protein kinase
Solyc01g094010.2.1	-4.702896561	2.63E-12	3.81E-10	L1-specific	L,D,F	CXE carboxylesterase (AHRD V1 **-- Q0ZPW6_9ROSA)%3B contains Interpro domain(s) IPR013094 Alpha/beta hydrolase fold-3
Solyc07g054490.2.1	-4.686974375	0.00018125	0.004484297	L1-specific	L	Secretory carrier membrane protein (AHRD V1 ***- B3TLU6_ELAGV)%3B contains Interpro domain(s) IPR007273 SCAMP
Solyc09g091510.2.1	-4.686466964	2.08E-19	7.73E-17	L1-specific	L,D	Chalcone synthase (AHRD V1 ***- Q56I79_9SOLN)%3B contains Interpro domain(s) IPR011141 Polyketide synthase%2C type III
Solyc02g088630.2.1	-4.646088322	2.36E-14	2.12E-12	L1-specific	D	Glycosyltransferase (AHRD V1 ***- B9IK47_POPTR)%3B contains Interpro domain(s) IPR002495 Glycosyl transferase%2C family 8
Solyc08g021890.2.1	-4.605930608	0.00277585	0.043324167	L1-specific	F	DnaJ homolog subfamily C member 13 (AHRD V1 **-- DJC13_HUMAN)
Solyc07g008920.2.1	-4.579007906	3.50E-09	1.37E-07	L1-specific	D	Oxysterol-binding protein (AHRD V1 **** Q2HUM2_MEDTR)%3B contains Interpro domain(s) IPR000648 Oxysterol-binding protein
Solyc05g054090.2.1	-4.503243574	5.63E-13	8.69E-11	L1-specific	L,F	Unknown Protein (AHRD V1)
Solyc02g080890.2.1	-4.492677034	4.08E-12	2.49E-10	L1-specific	D	Transcription factor WRKY (AHRD V1 ***- C7E5X8_CAPAN)%3B contains Interpro domain(s) IPR003657 DNA-binding WRKY
Solyc02g083600.2.1	-4.48262337	0.00065612	0.006280115	L1-specific	D	Replication factor C large subunit (AHRD V1 *--- D4GSN1_HALVD)%3B contains Interpro domain(s) IPR004582 Checkpoint protein Rad24
Solyc09g090390.1.1	-4.482361242	2.54E-11	4.90E-09	L1-specific	F	Unknown Protein (AHRD V1)
Solyc12g006710.1.1	-4.474402863	0.0001847	0.002168026	L1-specific	D	Tudor domain containing 3 (Fragment) (AHRD V1 *-*- B1AMN9_HUMAN)%3B contains Interpro domain(s) IPR013894 Region of unknown function DUF1767
Solyc03g083080.1.1	-4.427582006	0.00058518	0.005719281	L1-specific	D	Unknown Protein (AHRD V1)
Solyc02g071710.2.1	-4.419494064	2.65E-05	0.000868319	L1-specific	L	GDSL esterase/lipase At1g29670 (AHRD V1 ***- GDL15_ARATH)%3B contains Interpro domain(s) IPR001087 Lipase%2C GDSL
Solyc10g009190.1.1	-4.402184365	3.08E-22	7.51E-20	L1-specific	L,D	Cold shock protein-1 (AHRD V1 *-*- B6SP63_MAIZE)
Solyc07g040730.1.1	-4.37732461	0.00043591	0.009495197	L1-specific	L	Unknown Protein (AHRD V1)
Solyc04g071900.2.1	-4.371824812	1.03E-08	7.74E-07	L1-specific	L	Peroxidase (AHRD V1 **** Q94IQ1_TOBAC)%3B contains Interpro domain(s) IPR002016 Haem peroxidase%2C plant/fungal/bacterial
Solyc05g010320.2.1	-4.34784155	7.23E-14	1.21E-11	L1-specific	L	Chalcone--flavonone isomerase (AHRD V1 ***- D7LV11_ARALY)%3B contains Interpro domain(s) IPR003466 Chalcone isomerase%2C subgroup
Solyc05g008000.2.1	-4.345119846	0.0008214	0.007560129	L1-specific	D	RNA pyrophosphohydrolase (AHRD V1 ***- C9CZW5_9RHOB)%3B contains Interpro domain(s) IPR000086 NUDIX hydrolase domain
Solyc03g031760.2.1	-4.31995839	8.76E-05	0.002455215	L1-specific	L	Homeobox-leucine zipper protein ATHB-14 (AHRD V1 *-*- ATB14_ARATH)%3B contains Interpro domain(s) IPR002913 Lipid-binding START
Solyc06g009070.2.1	-4.30329967	0.00473245	0.030156606	L1-specific	D	ATP-dependent RNA helicase (AHRD V1 *-*- Q16ZW5_AEDAE)%3B contains Interpro domain(s) IPR014001 DEAD-like helicase%2C N-terminal
Solyc08g082090.1.1	-4.283719301	0.00044439	0.004541286	L1-specific	D	Avr9/Cf-9 rapidly elicited protein 194 (AHRD V1 ***- Q9FQZ4_TOBAC)
Solyc07g042390.1.1	-4.276041274	0.00297756	0.04249984	L1-specific	L	Pectinesterase (AHRD V1 **** B7SIX2_9CONI)%3B contains Interpro domain(s) IPR006501 Pectinesterase inhibitor
Solyc01g079240.2.1	-4.249569062	1.12E-10	1.29E-08	L1-specific	L,D	Long-chain-fatty-acid--CoA ligase family protein (AHRD V1 **** D7LGL1_ARALY)%3B contains Interpro domain(s) IPR000873 AMP-dependent synthetase and ligase
Solyc10g009290.1.1	-4.247564536	0.00871608	0.048864921	L1-specific	D	BHLH transcription factor (AHRD V1 *-** Q401N4_9LILI)%3B contains Interpro domain(s) IPR011598 Helix-loop-helix DNA-binding
Solyc02g078570.2.1	-4.224148414	2.81E-18	1.43E-15	L1-specific	F	Epoxide hydrolase 3 (AHRD V1 **** D8L7V9_PRUPE)%3B contains Interpro domain(s) IPR000639 Epoxide hydrolase-like
Solyc06g073990.1.1	-4.194322438	8.83E-16	9.42E-14	L1-specific	D	Unknown Protein (AHRD V1)
Solyc01g111440.2.1	-4.184599669	1.20E-19	2.16E-17	L1-specific	D	Senescence-associated protein 12 (AHRD V1 *-*- O81657_HEMSP)%3B contains Interpro domain(s) IPR009686 Senescence-associated
Solyc02g086530.2.1	-4.172521184	7.87E-18	1.10E-15	L1-specific	D	Alpha-galactosidase (AHRD V1 **-- C0NUM4_AJECG)%3B contains Interpro domain(s) IPR008811 Raffinose synthase
Solyc03g116100.2.1	-4.17129039	2.18E-17	2.91E-15	L1-specific	D	MYB transcription factor (AHRD V1 **-* Q6R036_ARATH)%3B contains Interpro domain(s) IPR015495 Myb transcription factor
Solyc03g120070.2.1	-4.171090446	0.00452811	0.029067734	L1-specific	D	Pentatricopeptide repeat-containing protein (AHRD V1 ***- D7MRA3_ARALY)%3B contains Interpro domain(s) IPR002885 Pentatricopeptide repeat
Solyc06g074500.1.1	-4.142363452	2.96E-07	7.94E-06	L1-specific	D	AT5G28150-like protein (Fragment) (AHRD V1 *-*- D6PS16_9BRAS)%3B contains Interpro domain(s) IPR008586 Protein of unknown function DUF868%2C plant
Solyc06g061280.2.1	-4.136956096	7.81E-20	1.49E-17	L1-specific	D	Cinnamoyl-CoA reductase-like protein (AHRD V1 **** Q9M0B3_ARATH)%3B contains Interpro domain(s) IPR016040 NAD(P)-binding domain
Solyc02g070500.1.1	-4.105303564	3.55E-18	5.36E-16	L1-specific	D	Susceptibility homeodomain transcription factor (Fragment) (AHRD V1 ***- Q8SAA7_ORYSA)%3B contains Interpro domain(s) IPR007493 Protein of unknown function DUF538
Solyc06g082440.1.1	-4.092460539	1.54E-14	1.43E-12	L1-specific	D	CBL-interacting protein kinase 16 (AHRD V1 ***- A0MNK3_POPTR)%3B contains Interpro domain(s) IPR002290 Serine/threonine protein kinase
Solyc12g087980.1.1	-4.0876053	0.00023255	0.006578067	L1-specific	F	Hydroxycinnamoyl CoA quinate transferase 2 (AHRD V1 **-- D8L7F0_CYNSC)%3B contains Interpro domain(s) IPR003480 Transferase
Solyc02g077710.1.1	-4.049430001	4.71E-19	2.58E-16	L1-specific	F	E6-2 protein kinase (AHRD V1 *-*- B6E139_GOSHI)
Solyc02g079590.2.1	-4.047225443	4.88E-16	5.56E-14	L1-specific	D	Serine/threonine kinase receptor (AHRD V1 **** Q7DMS5_BRANA)%3B contains Interpro domain(s) IPR002290 Serine/threonine protein kinase
Solyc02g084640.2.1	-4.042780862	3.78E-14	3.28E-12	L1-specific	L,D,F	Aldehyde dehydrogenase (AHRD V1 **** Q71LY7_ORYSI)%3B contains Interpro domain(s) IPR012394 Aldehyde dehydrogenase NAD(P)-dependent IPR015590 Aldehyde dehydrogenase
Solyc03g006620.2.1	-4.03699755	0.00038733	0.004044451	L1-specific	D	Cotton fiber expressed protein 1 (AHRD V1 ***- O81373_GOSHI)%3B contains Interpro domain(s) IPR008480 Protein of unknown function DUF761%2C plant
Solyc01g095930.2.1	-4.036715195	1.58E-15	1.62E-13	L1-specific	D	O-acyltransferase WSD1 (AHRD V1 **-- WSD1_ARATH)%3B contains Interpro domain(s) IPR004255 Uncharacterised protein family UPF0089
Solyc12g096900.1.1	-4.033840371	0.00148699	0.025207261	L1-specific	L	Tir%2C resistance protein fragment
Solyc07g042230.1.1	-4.022789516	7.44E-16	8.02E-14	L1-specific	D	Ethylene-responsive transcription factor 7 (AHRD V1 *-** ERF83_ARATH)%3B contains Interpro domain(s) IPR001471 Pathogenesis-related transcriptional factor and ERF%2C DNA-binding
Solyc05g051660.1.1	-4.011595643	7.29E-20	1.41E-17	L1-specific	D	Gibberellin receptor GID1L2 (AHRD V1 **-- B6T2M3_MAIZE)%3B contains Interpro domain(s) IPR013094 Alpha/beta hydrolase fold-3
Solyc08g076870.1.1	-4.011509946	1.61E-08	5.61E-07	L1-specific	D	Unknown Protein (AHRD V1)
Solyc09g083280.2.1	-4.001018253	2.19E-08	7.45E-07	L1-specific	D	Auxin responsive protein (AHRD V1 ***- D9IQE6_CATRO)%3B contains Interpro domain(s) IPR003311 AUX/IAA protein
Solyc02g087330.2.1	-3.992818102	3.87E-08	1.25E-06	L1-specific	D	B12D-like protein (AHRD V1 ***- D2XQY6_WOLAR)%3B contains Interpro domain(s) IPR010530 B12D
Solyc07g063910.2.1	-3.96228711	0.0006946	0.013624984	L1-specific	L,D	Unknown Protein (AHRD V1)
Solyc06g009190.2.1	-3.950590013	5.59E-13	8.69E-11	L1-specific	L	Pectinesterase (AHRD V1 ***- B9SAU4_RICCO)%3B contains Interpro domain(s) IPR000070 Pectinesterase%2C catalytic
Solyc09g074350.1.1	-3.930082347	6.27E-05	0.000865048	L1-specific	D	Unknown Protein (AHRD V1)
Solyc02g080610.2.1	-3.914031658	2.62E-05	0.000407487	L1-specific	D	Ankyrin repeat domain-containing protein 13C-A (AHRD V1 **** C0H9Z7_SALSA)%3B contains Interpro domain(s) IPR002110 Ankyrin
Solyc04g071620.2.1	-3.889627176	4.47E-18	1.40E-15	L1-specific	L	ASR4 (Fragment) (AHRD V1 *-*- Q2QJU1_SOLLC)%3B contains Interpro domain(s) IPR003496 ABA/WDS induced protein
Solyc04g072890.2.1	-3.873090732	7.48E-18	1.07E-15	L1-specific	D	WD-40 repeat family protein (AHRD V1 **-- D7KN06_ARALY)%3B contains Interpro domain(s) IPR017986 WD40 repeat%2C region
Solyc01g108250.2.1	-3.857792679	5.46E-17	7.02E-15	L1-specific	D	Vacuolar import and degradation protein VID27 (AHRD V1 *--- B3LP56_YEAS1)%3B contains Interpro domain(s) IPR013863 Vacuolar import and degradation%2C Vid27-related
Solyc07g066570.2.1	-3.850647879	7.34E-09	2.72E-07	L1-specific	D	%26gtF3O9.30 (AHRD V1 ***- Q9SA48_ARATH)
Solyc04g009900.2.1	-3.832196725	5.72E-14	4.84E-12	L1-specific	D	Calcium-dependent protein kinase 2 (AHRD V1 ***- B4FZS4_MAIZE)%3B contains Interpro domain(s) IPR002290 Serine/threonine protein kinase
Solyc03g114830.2.1	-3.821860945	3.69E-06	7.48E-05	L1-specific	D	MADS box transcription factor (AHRD V1 ***- Q9SBQ1_PETHY)%3B contains Interpro domain(s) IPR002487 Transcription factor%2C K-box
Solyc11g066720.1.1	-3.8091414	3.10E-15	6.56E-13	L1-specific	L	Bifunctional polymyxin resistance arnA protein (AHRD V1 ***- B6TQB1_MAIZE)%3B contains Interpro domain(s) IPR001509 NAD-dependent epimerase/dehydratase
Solyc12g042910.1.1	-3.80079933	0.00052269	0.005207401	L1-specific	D	Cryptochrome-like protein 1 (AHRD V1 ***- Q5IFN1_OSTTA)%3B contains Interpro domain(s) IPR005101 DNA photolyase%2C FAD-binding/Cryptochrome%2C C-terminal

Solyc09g092450.2.1	-3.778362465	1.94E-05	0.000881303	L1-specific	F	Long-chain-fatty-acid CoA ligase (AHRD V1 ***- Q9LK39_ARATH)%3B contains Interpro domain(s) IPR000873 AMP-dependent synthetase and ligase
Solyc07g066360.1.1	-3.778079253	0.00041189	0.0042752	L1-specific	D	Unknown Protein (AHRD V1)
Solyc06g084170.2.1	-3.766583614	3.06E-06	6.33E-05	L1-specific	D	Unknown Protein (AHRD V1)
Solyc02g069580.2.1	-3.728883324	5.12E-14	4.37E-12	L1-specific	D	UDP-glucose 4-epimerase (AHRD V1 **** B1XPB1_SYNP2)%3B contains Interpro domain(s) IPR005886 UDP-glucose 4-epimerase
Solyc04g016330.2.1	-3.699912575	2.23E-07	6.14E-06	L1-specific	D	Glycerol-3-phosphate dehydrogenase (AHRD V1 **** O22216_ARATH)%3B contains Interpro domain(s) IPR006168 NAD-dependent glycerol-3-phosphate dehydrogenase
Solyc02g090290.2.1	-3.669554796	2.16E-05	0.000347319	L1-specific	D	Cytochrome P450
Solyc07g052220.1.1	-3.659740807	1.13E-05	0.000419929	L1-specific	L	Unknown Protein (AHRD V1)
Solyc05g012230.2.1	-3.582159043	1.49E-05	0.00024821	L1-specific	D	Unknown Protein (AHRD V1)
Solyc02g081030.2.1	-3.578190938	1.73E-16	2.12E-14	L1-specific	D	Uncharacterized membrane protein C2G11.09 (AHRD V1 **-- YAB9_SCHPO)%3B contains Interpro domain(s) IPR003864 Protein of unknown function DUF221
Solyc10g048030.1.1	-3.560707089	5.48E-08	1.73E-06	L1-specific	L,D	Major latex-like protein (AHRD V1 **-- B5THI3_PANGI)%3B contains Interpro domain(s) IPR000916 Bet v I allergen
Solyc04g077980.1.1	-3.554347808	1.16E-13	9.30E-12	L1-specific	D	Zinc-finger protein (AHRD V1 ***- Q40899_PETHY)%3B contains Interpro domain(s) IPR007087 Zinc finger%2C C2H2-type
Solyc02g080380.2.1	-3.53907568	0.00174977	0.028567647	L1-specific	L	Derlin-2 (AHRD V1 ***- B6TKJ7_MAIZE)%3B contains Interpro domain(s) IPR007599 Der1-like
Solyc02g071130.2.1	-3.518602667	2.45E-05	0.000383564	L1-specific	D	WRKY transcription factor 29 (AHRD V1 ***- C9DI18_9ROSI)%3B contains Interpro domain(s) IPR003657 DNA-binding WRKY
Solyc04g072910.2.1	-3.517049373	0.00033456	0.007506199	L1-specific	L	Endoplasmic reticulum-Golgi intermediate compartment protein 3 (AHRD V1 ***- C0H9D0_SALSA)%3B contains Interpro domain(s) IPR012936 Protein of unknown function DUF1692
Solyc06g062600.2.1	-3.49618582	3.14E-10	1.44E-08	L1-specific	D	Choline dehydrogenase (AHRD V1 ***- A0QXW0_MYCS2)%3B contains Interpro domain(s) IPR012132 Glucose-methanol-choline oxidoreductase
Solyc01g103590.2.1	-3.328926854	7.75E-05	0.00222535	L1-specific	L	Glyoxalase/bleomycin resistance protein/dioxygenase (AHRD V1 **-- Q2HVV5_MEDTR)%3B contains Interpro domain(s) IPR004360 Glyoxalase/bleomycin resistance protein/dioxygenase
Solyc00g127660.2.1	-3.324361286	0.00113147	0.009815927	L1-specific	D	Unknown Protein (AHRD V1)
Solyc02g093150.2.1	-3.302345822	5.41E-09	2.05E-07	L1-specific	D	AP2-like ethylene-responsive transcription factor At1g16060 (AHRD V1 *-*- AP2L1_ARATH)%3B contains Interpro domain(s) IPR001471 Pathogenesis-related transcriptional factor and ERF%2C DNA-binding
Solyc12g006850.1.1	-3.265538232	7.06E-06	0.000131448	L1-specific	D	Potassium channel (AHRD V1 ***- Q9LEG6_SOLLC)%3B contains Interpro domain(s) IPR002110 Ankyrin
Solyc04g078450.2.1	-3.255044502	3.04E-06	6.30E-05	L1-specific	D	Unknown Protein (AHRD V1)
Solyc11g008260.1.1	-3.227743095	9.92E-08	2.95E-06	L1-specific	D	Cysteine proteinase cathepsin F (AHRD V1 **-- D3TN89_GLOMM)%3B contains Interpro domain(s) IPR013128 Peptidase C1A%2C papain
Solyc11g011340.1.1	-3.223717794	2.52E-06	5.38E-05	L1-specific	D	Alcohol dehydrogenase (AHRD V1 ***- Q8KRC3_MYXXA)%3B contains Interpro domain(s) IPR002085 Alcohol dehydrogenase superfamily%2C zinc-containing
Solyc09g072970.2.1	-3.206465912	4.12E-05	0.001636785	L1-specific	F	U6 snRNA-associated Sm-like protein LSm2 (AHRD V1 **-- B9EPQ0_SALSA)%3B contains Interpro domain(s) IPR016654 U6 snRNA-associated Sm-like protein LSm2
Solyc08g076720.2.1	-3.20355952	3.81E-06	0.000216135	L1-specific	F	Uncharacterized ABC transporter ATP-binding protein TM_0288 (AHRD V1 *--* Y288_THEMA)%3B contains Interpro domain(s) IPR003439 ABC transporter-like
Solyc01g091270.2.1	-3.201486486	1.22E-09	5.06E-08	L1-specific	D	AT2G45380 protein (Fragment) (AHRD V1 ***- B9DGW8_ARATH)
Solyc03g114160.1.1	-3.194300574	6.73E-11	3.42E-09	L1-specific	D	U-box domain-containing protein (AHRD V1 ***- D7KBV1_ARALY)%3B contains Interpro domain(s) IPR011989 Armadillo-like helical
Solyc04g072880.2.1	-3.186946985	1.38E-05	0.000231289	L1-specific	D	RNA polymerase II holoenzyme cyclin-like subunit (AHRD V1 **** SSN8_NEUCR)%3B contains Interpro domain(s) IPR015432 Cyclin H
Solyc12g088670.1.1	-3.147662535	7.31E-11	1.25E-08	L1-specific	F	Cathepsin B-like cysteine proteinase (AHRD V1 **-- CYSP_SCHMA)%3B contains Interpro domain(s) IPR013128 Peptidase C1A%2C papain IPR000169 Peptidase%2C cysteine peptidase active site IPR000668 Peptidase C1A%2C papain C-terminal
Solyc01g080010.2.1	-3.123658486	3.23E-13	2.42E-11	L1-specific	D	Xylanase inhibitor (Fragment) (AHRD V1 **-- Q53IQ4_WHEAT)%3B contains Interpro domain(s) IPR001461 Peptidase A1
Solyc09g090980.2.1	-3.122712783	1.41E-05	0.000507319	L1-specific	L,F	Major allergen Mal d 1 (AHRD V1 ***- Q84LA7_MALDO)%3B contains Interpro domain(s) IPR000916 Bet v I allergen
Solyc06g065670.2.1	-3.083183503	1.24E-05	0.000601099	L1-specific	F	ATP-binding cassette transporter (AHRD V1 **** D8RL93_SELML)%3B contains Interpro domain(s) IPR013525 ABC-2 type transporter
Solyc12g044300.1.1	-3.078355589	8.61E-07	6.09E-05	L1-specific	F	Acyl-CoA synthetase (AMP-forming)/AMP-acid ligase II (AHRD V1 **** C7MWS2_SACVD)%3B contains Interpro domain(s) IPR000873 AMP-dependent synthetase and ligase
Solyc04g073950.1.1	-3.057756111	7.24E-07	1.76E-05	L1-specific	D	VQ motif family protein expressed (AHRD V1 *-*- Q10KE8_ORYSJ)%3B contains Interpro domain(s) IPR008889 VQ
Solyc09g089940.1.1	-3.013282204	4.66E-09	1.81E-07	L1-specific	D	Genomic DNA chromosome 3 TAC clone K7P8 (AHRD V1 *--- Q9LRY2_ARATH)%3B contains Interpro domain(s) IPR011205 Uncharacterized conserved protein UCP015417%2C vWA
Solyc01g090740.2.1	-3.007603765	0.00016419	0.001950487	L1-specific	D	Zinc ion binding protein (AHRD V1 ***- D7LRR6_ARALY)
Solyc07g042630.2.1	-3.000160782	1.36E-05	0.000229976	L1-specific	D	Beta-Amyrin Synthase (AHRD V1 **** O82146_PANGI)%3B contains Interpro domain(s) IPR018333 Squalene cyclase
Solyc05g012070.2.1	-2.981176695	5.13E-07	1.29E-05	L1-specific	D	Alpha-1 4-glucan-protein synthase (AHRD V1 **-* B9RLK7_RICCO)%3B contains Interpro domain(s) IPR004901 Alpha-1%2C4-glucan-protein synthase%2C UDP-forming
Solyc05g014790.2.1	-2.980108387	6.07E-06	0.00024305	L1-specific	L	Lipoxygenase (AHRD V1 **** B9RMJ4_RICCO)%3B contains Interpro domain(s) IPR001246 Lipoxygenase%2C plant
Solyc12g056580.1.1	-2.914330903	5.16E-09	1.98E-07	L1-specific	D	Cellulose synthase (AHRD V1 ***- B9GTH4_POPTR)%3B contains Interpro domain(s) IPR005150 Cellulose synthase
Solyc10g085830.1.1	-2.904355521	1.97E-06	4.33E-05	L1-specific	D	O-methyltransferase 1 (AHRD V1 ***- D7MTQ7_ARALY)%3B contains Interpro domain(s) IPR016461 O-methyltransferase%2C COMT%2C eukaryota
Solyc08g067810.2.1	-2.894406615	1.38E-07	3.93E-06	L1-specific	D	CID5/IPD1 (AHRD V1 ***- D7M3Q7_ARALY)%3B contains Interpro domain(s) IPR003892 Ubiquitin system component Cue
Solyc08g007460.2.1	-2.885749202	2.46E-08	8.25E-07	L1-specific	D	Non-specific lipid-transfer protein (AHRD V1 -*** B9RGU5_RICCO)%3B contains Interpro domain(s) IPR003612 Plant lipid transfer protein/seed storage/trypsin-alpha amylase inhibitor
Solyc05g009000.2.1	-2.883788694	0.00069511	0.006574683	L1-specific	D	Kinase family protein (AHRD V1 ***- D7KAJ2_ARALY)%3B contains Interpro domain(s) IPR011009 Protein kinase-like
Solyc06g072610.2.1	-2.880526522	1.32E-06	3.01E-05	L1-specific	D	Palmitoyltransferase-like protein (AHRD V1 *-** C6YXK4_ORYSJ)%3B contains Interpro domain(s) IPR001594 Zinc finger%2C DHHC-type
Solyc04g081530.1.1	-2.823995088	3.16E-06	6.48E-05	L1-specific	D	Chaperone protein dnaJ 11 (AHRD V1 *-*- B6SUM8_MAIZE)%3B contains Interpro domain(s) IPR001623 Heat shock protein DnaJ%2C N-terminal
Solyc03g115370.2.1	-2.76369689	1.85E-05	0.000637366	L1-specific	L,D	Diacylglycerol kinase 1 (AHRD V1 **** A8QM12_MAIZE)%3B contains Interpro domain(s) IPR000756 Diacylglycerol kinase accessory region
Solyc01g112080.2.1	-2.752712533	3.14E-07	8.39E-06	L1-specific	D	LysM-domain GPI-anchored protein (AHRD V1 **-- Q4VVD8_ORYSJ)%3B contains Interpro domain(s) IPR002482 Peptidoglycan-binding Lysin subgroup
Solyc09g015530.2.1	-2.740829729	4.75E-06	9.29E-05	L1-specific	D	Os05g0478200 protein (Fragment) (AHRD V1 *-*- Q0DHB3_ORYSJ)
Solyc08g044260.2.1	-2.66985714	1.00E-09	4.26E-08	L1-specific	D,F	CER1 (AHRD V1 **-- B6TFH3_MAIZE)%3B contains Interpro domain(s) IPR006694 Fatty acid hydroxylase
Solyc06g009640.1.1	-2.644974525	8.79E-06	0.000159364	L1-specific	D	BZIP transcription factor (AHRD V1 **-* Q1HW69_CAPAN)%3B contains Interpro domain(s) IPR011700 Basic leucine zipper
Solyc10g008350.2.1	-2.630874328	0.00035185	0.003738831	L1-specific	D	Unknown Protein (AHRD V1)
Solyc09g092710.2.1	-2.628635564	7.64E-07	1.85E-05	L1-specific	D	Glycine rich protein (AHRD V1 ***- Q9FXS8_TOBAC)%3B contains Interpro domain(s) IPR010800 Glycine rich
Solyc03g117270.1.1	-2.597507007	0.00157004	0.012782922	L1-specific	D	F-box family protein (AHRD V1 ***- B9HV09_POPTR)%3B contains Interpro domain(s) IPR001810 Cyclin-like F-box
Solyc10g080480.1.1	-2.597265936	0.00196974	0.015345003	L1-specific	D	Zinc finger CCCH domain-containing protein 14 (AHRD V1 ***- C3H14_ORYSJ)%3B contains Interpro domain(s) IPR018111 K Homology%2C type 1%2C subgroup
Solyc02g091920.2.1	-2.574354236	5.49E-09	4.46E-07	L1-specific	L	Xyloglucan endotransglucosylase/hydrolase 2 (AHRD V1 **** Q2MK81_9ROSA)%3B contains Interpro domain(s) IPR016455 Xyloglucan endotransglucosylase/hydrolase
Solyc06g073410.2.1	-2.525692091	0.00350716	0.02399293	L1-specific	D	Gamma-secretase subunit APH-1B (AHRD V1 ***- B6TH01_MAIZE)%3B contains Interpro domain(s) IPR009294 Aph-1
Solyc08g068330.2.1	-2.525692091	0.00049577	0.004971268	L1-specific	D	Aspartate aminotransferase (AHRD V1 **** B9H920_POPTR)%3B contains Interpro domain(s) IPR000796 Aspartate/other aminotransferase
Solyc06g069040.2.1	-2.470023333	2.01E-05	0.000324634	L1-specific	D	Glutathione S-transferase (AHRD V1 **** Q84T17_PHAAT)%3B contains Interpro domain(s) IPR004046 Glutathione S-transferase%2C C-terminal
Solyc06g053360.2.1	-2.455859144	0.00025237	0.002831678	L1-specific	D	Os10g0479800 protein (Fragment) (AHRD V1 *-*- Q0IWY0_ORYSJ)%3B contains Interpro domain(s) IPR009769 Protein of unknown function DUF1336
Solyc04g005340.2.1	-2.411543499	8.93E-09	3.25E-07	L1-specific	D	Alpha-1 4-glucan protein synthase (AHRD V1 **-* Q8H8T0_ORYSJ)%3B contains Interpro domain(s) IPR004901 Alpha-1%2C4-glucan-protein synthase%2C UDP-forming
Solyc01g106050.2.1	-2.370484644	1.69E-05	0.000276505	L1-specific	D	Dynamin-2 (AHRD V1 ***- D0MQ53_PHYIN)%3B contains Interpro domain(s) IPR000375 Dynamin central region
Solyc12g010390.1.1	-2.277208975	7.83E-05	0.002240816	L1-specific	L	Transmembrane 9 superfamily protein member 2 (AHRD V1 **** B6ST61_MAIZE)%3B contains Interpro domain(s) IPR004240 Nonaspanin (TM9SF)
Solyc02g083460.2.1	-2.257909894	2.11E-06	4.59E-05	L1-specific	D	Aspartyl protease family protein (AHRD V1 **-- D7LAA8_ARALY)%3B contains Interpro domain(s) IPR001461 Peptidase A1
Solyc04g080850.2.1	-2.248659751	9.47E-08	2.84E-06	L1-specific	D	Thioredoxin (AHRD V1 ***- Q201Z4_9SOLN)%3B contains Interpro domain(s) IPR015467 Thioredoxin%2C core
Solyc12g056800.1.1	-2.241107484	0.0025955	0.038611912	L1-specific	L	Oxidoreductase family protein (AHRD V1 ***- D7LXC3_ARALY)%3B contains Interpro domain(s) IPR016040 NAD(P)-binding domain
Solyc01g096230.2.1	-2.178986424	0.0002176	0.002493191	L1-specific	D	ATPase AAA family protein expressed (AHRD V1 **-- Q8GZX0_ORYSJ)%3B contains Interpro domain(s) IPR003959 ATPase%2C AAA-type%2C core
Solyc04g080290.2.1	-2.164388151	0.00051441	0.010849923	L1-specific	L	CRAL/TRIO domain containing protein expressed (AHRD V1 **-- Q75GU9_ORYSJ)%3B contains Interpro domain(s) IPR001251 Cellular retinaldehyde-binding/triple function%2C C-terminal
Solyc03g121620.1.1	-2.055966787	0.00021204	0.002447357	L1-specific	D	Harpin-induced protein-like (Fragment) (AHRD V1 **-- D2CFH8_COFAR)%3B contains Interpro domain(s) IPR010847 Harpin-induced 1
Solyc03g114860.2.1	-2.05050948	2.85E-05	0.000438426	L1-specific	D	Alpha-1 4-glucan-protein synthase (AHRD V1 **-* B9RLK7_RICCO)%3B contains Interpro domain(s) IPR004901 Alpha-1%2C4-glucan-protein synthase%2C UDP-forming
Solyc03g095700.1.1	-2.036703284	0.00132049	0.011115794	L1-specific	D	Genomic DNA chromosome 5 TAC clone K18I23 (AHRD V1 ***- Q9FLD2_ARATH)
Solyc05g052240.2.1	-2.027968891	0.00054948	0.005404597	L1-specific	D	Chalcone--flavonone isomerase (AHRD V1 ***- B4G1R6_MAIZE)%3B contains Interpro domain(s) IPR003466 Chalcone isomerase%2C subgroup
Solyc08g007140.2.1	-2.027219753	8.80E-05	0.001162065	L1-specific	D	60S ribosomal protein L37a (AHRD V1 ***- Q5GMM4_CAPCH)%3B contains Interpro domain(s) IPR002674 Ribosomal protein L37ae
Solyc06g060910.1.1	-1.930768075	0.0001049	0.001352387	L1-specific	D	Glycerol-3-phosphate transporter (AHRD V1 **** B0SG38_LEPBA)%3B contains Interpro domain(s) IPR016196 Major facilitator superfamily%2C general substrate transporter
Solyc02g062370.2.1	-1.876064279	6.38E-05	0.000877878	L1-specific	D	Zinc finger CCCH domain-containing protein 19 (AHRD V1 *-*- C3H19_ARATH)%3B contains Interpro domain(s) IPR003169 GYF
Solyc01g103160.2.1	-1.64717614	0.00244149	0.018184259	L1-specific	D	Pentatricopeptide repeat-containing protein (AHRD V1 *-*- D7LST4_ARALY)%3B contains Interpro domain(s) IPR002885 Pentatricopeptide repeat
Solyc11g011440.1.1	-1.601061653	0.0002017	0.002339909	L1-specific	D	Aspartic proteinase nepenthesin-1 (AHRD V1 **-- B6TS94_MAIZE)%3B contains Interpro domain(s) IPR001461 Peptidase A1
Solyc01g102350.2.1	-1.589427219	0.00042447	0.004376595	L1-specific	D	Pectinacetylesterase like protein (Fragment) (AHRD V1 *--- Q56WP8_ARATH)%3B contains Interpro domain(s) IPR004963 Pectinacetylesterase
Solyc01g096040.2.1	-1.495000589	0.00149518	0.012281315	L1-specific	D	Aspartic proteinase nepenthesin I (AHRD V1 **-- A9ZMF9_NEPAL)%3B contains Interpro domain(s) IPR001461 Peptidase A1
Solyc05g053070.2.1	-1.460817524	0.0005263	0.005239645	L1-specific	D	Unknown Protein (AHRD V1)
Solyc11g010230.1.1	-1.210582576	0.00664852	0.039657153	L1-specific	D	Histone H3 (AHRD V1 ***- A8MRL0_ARATH)%3B contains Interpro domain(s) IPR000164 Histone H3
Solyc08g078070.2.1	-1.204572252	0.00657629	0.039259975	L1-specific	D	Ras-related protein Rab-1A (AHRD V1 ***- B6RB23_HALDI)%3B contains Interpro domain(s) IPR003579 Ras small GTPase%2C Rab type
Solyc02g087210.2.1	-6.965706256	1.11E-10	5.50E-09	L1-related	D	Zinc finger AN1 domain-containing stress-associated protein 12 (AHRD V1 ***- SAP12_ARATH)%3B contains Interpro domain(s) IPR000058 Zinc finger%2C AN1-type
Solyc01g006290.2.1	-6.67854358	3.11E-05	0.000474788	L1-related	D	Peroxidase (AHRD V1 **** Q42964_TOBAC)%3B contains Interpro domain(s) IPR002016 Haem peroxidase%2C plant/fungal/bacterial
Solyc08g075320.2.1	-6.373688998	0.00068162	0.006470716	L1-related	D	Cytochrome P450
Solyc04g079350.1.1	-5.456151158	0.00538678	0.033377378	L1-related	D	Pheromone receptor-like protein (Fragment) (AHRD V1 *--- Q7XHJ1_QUERO)%3B contains Interpro domain(s) IPR012442 Protein of unknown function DUF1645
Solyc07g007350.1.1	-5.122721606	0.00077638	0.016853062	L1-related	F	Jp18 (AHRD V1 *-*- Q8H6R4_PONTR)%3B contains Interpro domain(s) IPR010993 Sterile alpha motif homology
Solyc09g031920.1.1	-5.098026984	4.46E-09	1.73E-07	L1-related	D	Harpin-induced 1 (AHRD V1 ***- Q1STP6_MEDTR)%3B contains Interpro domain(s) IPR010847 Harpin-induced 1
Solyc10g086690.1.1	-4.886493078	1.31E-05	0.000221592	L1-related	D	Phosphatidylcholine:ceramide cholinephosphotransferase 2 (AHRD V1 *-*- SMS2_MACFA)
Solyc03g006970.1.1	-4.852233565	0.00060678	0.012361537	L1-related	L	Subtilisin-like protease (AHRD V1 ***- B6UDX1_MAIZE)%3B contains Interpro domain(s) IPR015500 Peptidase S8%2C subtilisin-related IPR000183 Orn/DAP/Arg decarboxylase 2 IPR000209 Peptidase S8 and S53%2C subtilisin%2C kexin%2C sedolisin
Solyc04g009440.2.1	-4.679538885	3.48E-05	0.000521504	L1-related	D	NAC domain protein (AHRD V1 ***- Q6RH27_SOLLC)%3B contains Interpro domain(s) IPR003441 No apical meristem (NAM) protein
Solyc02g079440.1.1	-4.61813834	6.00E-05	0.000833557	L1-related	D	FAD-binding domain-containing protein (AHRD V1 **-- D7MFH6_ARALY)%3B contains Interpro domain(s) IPR006094 FAD linked oxidase%2C N-terminal
Solyc03g026220.2.1	-4.564005134	8.19E-14	6.86E-12	L1-related	D	BCL-2 binding anthanogene-1 (AHRD V1 **** B4FV61_MAIZE)%3B contains Interpro domain(s) IPR003103 Apoptosis regulator Bcl-2 protein%2C BAG

Solyc08g079510.2.1	-4.391963416	1.61E-17	2.18E-15	L1-related	D	Unknown Protein (AHRD V1)
Solyc04g072110.2.1	-4.391662432	6.21E-05	0.000858693	L1-related	D	Microsomal signal peptidase subunit(SPC25)-like protein (AHRD V1 ***- Q6Z7E3_ORYSJ)%3B contains Interpro domain(s) IPR009582 Microsomal signal peptidase 25 kDa subunit
Solyc03g118710.2.1	-4.388863028	8.88E-16	9.42E-14	L1-related	D	C2 domain-containing protein (AHRD V1 **-- D7L7Y3_ARALY)%3B contains Interpro domain(s) IPR018029 C2 membrane targeting protein
Solyc08g076050.2.1	-4.282753965	0.00223073	0.016932135	L1-related	D	ARK3 product/receptor-like serine/threonine protein kinase ARK3 (AHRD V1 **** Q9S971_ARATH)%3B contains Interpro domain(s) IPR002290 Serine/threonine protein kinase
Solyc12g005730.1.1	-4.193116752	4.33E-05	0.000626212	L1-related	D	Glycosyltransferase-like protein (AHRD V1 **-- D8QTD2_SELML)%3B contains Interpro domain(s) IPR006740 Protein of unknown function DUF604
Solyc01g104410.2.1	-4.100007348	0.00625365	0.037657351	L1-related	D	Sterol 3-beta-glucosyltransferase (AHRD V1 *-*- A4X1A3_SALTO)
Solyc11g010250.1.1	-4.061872219	1.29E-06	2.95E-05	L1-related	D	Avr9/Cf-9 rapidly elicited protein 75 (AHRD V1 ***- Q9FQY9_TOBAC)
Solyc02g070530.2.1	-4.044253366	0.00430329	0.027882117	L1-related	D	Potassium channel (AHRD V1 ***- O24382_SOLTU)%3B contains Interpro domain(s) IPR000595 Cyclic nucleotide-binding
Solyc06g060370.2.1	-4.031961961	4.07E-06	8.12E-05	L1-related	D	Organic anion transporter (AHRD V1 **-* B4G1R1_MAIZE)%3B contains Interpro domain(s) IPR004853 Protein of unknown function DUF250
Solyc01g095150.2.1	-3.895685491	1.45E-18	2.26E-16	L1-related	D	Late embryogenesis abundant protein (Fragment) (AHRD V1 **-- B7TGE2_PINSY)%3B contains Interpro domain(s) IPR013990 Water Stress and Hypersensitive response
Solyc02g072210.1.1	-3.871188657	0.00497668	0.031381982	L1-related	D	Unknown Protein (AHRD V1)
Solyc05g005280.2.1	-3.794427247	0.00054761	0.005393838	L1-related	D	Poly polymerase catalytic domain containing protein expressed (AHRD V1 **-- Q84T80_ORYSJ)%3B contains Interpro domain(s) IPR012317 Poly(ADP-ribose) polymerase%2C catalytic region
Solyc11g020670.1.1	-3.785808628	9.52E-08	8.70E-06	L1-related	F	TCP family transcription factor (AHRD V1 *-** A6MCZ2_9ORYZ)%3B contains Interpro domain(s) IPR005333 Transcription factor%2C TCP
Solyc08g075550.2.1	-3.760157345	0.00241867	0.018064752	L1-related	D	Alternative oxidase (AHRD V1 **** Q84V46_SOLLC)%3B contains Interpro domain(s) IPR002680 Alternative oxidase
Solyc03g111170.2.1	-3.752714455	5.33E-12	3.19E-10	L1-related	D	4-coumarate-coa ligase (AHRD V1 **** B9I3N1_POPTR)%3B contains Interpro domain(s) IPR000873 AMP-dependent synthetase and ligase
Solyc01g091910.2.1	-3.728962723	6.10E-06	0.000115871	L1-related	D	Phospholipase D (AHRD V1 **** Q9XGT0_GOSHI)%3B contains Interpro domain(s) IPR011402 Phospholipase D%2C plant
Solyc06g074940.2.1	-3.649453881	0.00065203	0.006245273	L1-related	D	ATP-binding cassette protein (AHRD V1 ***- Q5A2T2_CANAL)%3B contains Interpro domain(s) IPR003439 ABC transporter-like
Solyc03g063600.2.1	-3.634613266	5.67E-16	6.21E-14	L1-related	D	Guanylate kinase (AHRD V1 **** Q9FVC6_TOBAC)%3B contains Interpro domain(s) IPR017665 Guanylate kinase%2C sub-group
Solyc03g098140.2.1	-3.633070988	2.81E-05	0.000913409	L1-related	L	Arabinose-5-phosphate isomerase (AHRD V1 ***- B0TYU5_FRAP2)%3B contains Interpro domain(s) IPR004800 KpsF/GutQ
Solyc01g005410.2.1	-3.608154252	0.00216339	0.016483955	L1-related	D	Calcium binding protein Caleosin (AHRD V1 **-* A8N0P5_COPC7)%3B contains Interpro domain(s) IPR007736 Caleosin related
Solyc05g053290.2.1	-3.594033143	0.00071965	0.00678482	L1-related	D	Protein phosphatase-2C (AHRD V1 ***- O82469_MESCR)%3B contains Interpro domain(s) IPR015655 Protein phosphatase 2C
Solyc07g045160.2.1	-3.525843898	9.33E-06	0.00016671	L1-related	D	Phosphofructokinase family protein (AHRD V1 ***- D7MLU4_ARALY)%3B contains Interpro domain(s) IPR012004 Pyrophosphate-dependent phosphofructokinase TP0108
Solyc06g083550.1.1	-3.517006364	0.00305814	0.021552753	L1-related	D	F-box/Kelch repeat-containing F-box family protein (AHRD V1 ***- C4PVV9_ARATH)%3B contains Interpro domain(s) IPR015915 Kelch-type beta propeller
Solyc06g035960.2.1	-3.505659203	0.00010115	0.001310191	L1-related	D	4-coumarate-CoA ligase-like protein (AHRD V1 **** Q9SMT7_ARATH)%3B contains Interpro domain(s) IPR000873 AMP-dependent synthetase and ligase
Solyc07g062690.1.1	-3.376828706	0.0017165	0.013710327	L1-related	D	Unknown Protein (AHRD V1)
Solyc05g026310.2.1	-3.33986412	3.76E-05	0.000556243	L1-related	D	Derlin-3 (AHRD V1 ***- B6U3C7_MAIZE)%3B contains Interpro domain(s) IPR007599 Der1-like
Solyc07g065340.1.1	-3.254922037	0.00050408	0.005043734	L1-related	D	Serine acetyltransferase (AHRD V1 **** Q39533_CITLA)%3B contains Interpro domain(s) IPR005881 Serine O-acetyltransferase
Solyc10g051020.1.1	-3.227654062	9.03E-13	6.18E-11	L1-related	D	Cytochrome P450
Solyc07g045140.2.1	-3.227386654	0.00012406	0.001546359	L1-related	D	Uncharacterized membrane protein At3g27390 (AHRD V1 ***- Y3739_ARATH)
Solyc01g097240.2.1	-3.162442297	5.10E-12	3.07E-10	L1-related	D	Pathogenesis-related protein 4B (Fragment) (AHRD V1 **-- Q6LBM4_TOBAC)%3B contains Interpro domain(s) IPR018226 Barwin%2C conserved site IPR001153 Barwin
Solyc01g009100.2.1	-3.159352771	0.00011706	0.00147762	L1-related	D	Ribosomal protein L30 (AHRD V1 ***- B3TLP4_ELAGV)%3B contains Interpro domain(s) IPR000231 Ribosomal protein L30e
Solyc02g093270.2.1	-3.141766152	7.68E-13	5.31E-11	L1-related	D	Caffeoyl-CoA O-methyltransferase (AHRD V1 **** A8IFQ7_SOLLC)%3B contains Interpro domain(s) IPR002935 O-methyltransferase%2C family 3
Solyc02g087780.2.1	-3.122727424	0.00176828	0.014067294	L1-related	D	Aldose-1-epimerase-like protein (AHRD V1 **** O48971_TOBAC)%3B contains Interpro domain(s) IPR015443 Aldose-1-epimerase
Solyc12g098490.1.1	-3.122727424	1.39E-07	3.93E-06	L1-related	D	Serine hydroxymethyltransferase (AHRD V1 **** D2D306_GOSHI)%3B contains Interpro domain(s) IPR001085 Serine hydroxymethyltransferase
Solyc01g109120.2.1	-3.065572701	2.38E-07	6.52E-06	L1-related	D	WD-40 repeat family protein (AHRD V1 **-- Q1EPE0_MUSAC)%3B contains Interpro domain(s) IPR020472 G-protein beta WD-40 repeat%2C region
Solyc01g096220.2.1	-3.043503469	0.00299528	0.042689129	L1-related	L	Ras-related protein Rab-25 (AHRD V1 ***- RAB25_BOVIN)%3B contains Interpro domain(s) IPR015595 Rab11-related
Solyc08g077680.2.1	-3.032482069	7.28E-05	0.000986527	L1-related	D	Kinase interacting protein 1-like (AHRD V1 *-*- Q9AS78_ORYSJ)%3B contains Interpro domain(s) IPR011684 KIP1-like
Solyc02g061840.2.1	-3.014629737	0.00061812	0.005982288	L1-related	D	Nuclear SR-like RNA binding protein (AHRD V1 *--- A8JAT7_CHLRE)%3B contains Interpro domain(s) IPR012677 Nucleotide-binding%2C alpha-beta plait
Solyc06g005250.2.1	-3.008333792	0.00284022	0.020452545	L1-related	D	mRNA clone RAFL24-05-D16 (AHRD V1 ***- Q67YW5_ARATH)
Solyc09g011860.2.1	-2.996680095	0.00042711	0.004397025	L1-related	D	Os03g0169000 protein (Fragment) (AHRD V1 ***- Q0DUS7_ORYSJ)%3B contains Interpro domain(s) IPR004348 Protein of unknown function DUF246%2C plant
Solyc04g081510.2.1	-2.989833154	2.79E-07	7.52E-06	L1-related	D	Os01g0873900 protein (Fragment) (AHRD V1 *-*- Q0JHA9_ORYSJ)%3B contains Interpro domain(s) IPR007700 Protein of unknown function DUF668
Solyc01g104950.2.1	-2.975880217	4.70E-05	0.001832438	L1-related	F	Alpha-L-arabinofuranosidase/beta-D-xylosidase (AHRD V1 **** D9D7L0_MALDO)%3B contains Interpro domain(s) IPR001764 Glycoside hydrolase%2C family 3%2C N-terminal
Solyc08g062690.2.1	-2.962390667	2.81E-06	5.89E-05	L1-related	D	Unknown Protein (AHRD V1)
Solyc03g115220.2.1	-2.905245929	5.55E-05	0.002113929	L1-related	F	Cytochrome P450
Solyc11g040130.1.1	-2.849611579	4.81E-05	0.000687066	L1-related	D	SNARE associated Golgi protein (AHRD V1 *--- B9XJU7_9BACT)%3B contains Interpro domain(s) IPR015414 SNARE associated Golgi protein
Solyc08g075540.2.1	-2.834833242	5.62E-07	1.40E-05	L1-related	D	Alternative oxidase (AHRD V1 **** Q84V47_SOLLC)%3B contains Interpro domain(s) IPR002680 Alternative oxidase
Solyc08g076740.2.1	-2.791018309	0.00241739	0.018064752	L1-related	D	cDNA clone 002-116-F08 full insert sequence (AHRD V1 *-*- B7E9W2_ORYSJ)
Solyc08g082310.2.1	-2.778079253	0.0038019	0.025539047	L1-related	D	RING finger protein (AHRD V1 *-*- C6EUD3_SOYBN)%3B contains Interpro domain(s) IPR018957 Zinc finger%2C C3HC4 RING-type
Solyc06g054330.2.1	-2.754311087	0.00274465	0.019929536	L1-related	D	3-ketodihydrosphingosine reductase (AHRD V1 ***- B6TX51_MAIZE)%3B contains Interpro domain(s) IPR002198 Short-chain dehydrogenase/reductase SDR
Solyc01g067930.2.1	-2.701537698	0.00013868	0.001681574	L1-related	D	Alpha-1 6-xylosyltransferase (Fragment) (AHRD V1 **-- Q5TIN3_GOSRA)%3B contains Interpro domain(s) IPR008630 Galactosyl transferase
Solyc05g050560.1.1	-2.690498122	2.22E-05	0.000354088	L1-related	D	Transcription factor (AHRD V1 *--* Q9M4A8_MAIZE)%3B contains Interpro domain(s) IPR011598 Helix-loop-helix DNA-binding
Solyc11g011970.1.1	-2.689074247	0.00767326	0.04413059	L1-related	D	Tyrosine phosphatase family protein (AHRD V1 ***- B8N9Z8_ASPFN)%3B contains Interpro domain(s) IPR020428 Protein-tyrosine phosphatase%2C dual specificity phosphatase%2C eukaryotic
Solyc01g065530.2.1	-2.675268448	0.00118978	0.010194562	L1-related	D	COBRA-like protein (AHRD V1 ***- Q655F2_ORYSJ)%3B contains Interpro domain(s) IPR006918 Glycosyl-phosphatidyl inositol-anchored%2C plant
Solyc07g042170.2.1	-2.650053864	3.54E-08	1.15E-06	L1-related	D	Jasmonate ZIM-domain protein 3 (AHRD V1 **-- B3Y563_TOBAC)%3B contains Interpro domain(s) IPR010399 Tify
Solyc07g006360.1.1	-2.644680128	0.00053745	0.005312542	L1-related	D	RING finger protein (AHRD V1 *-*- C6EUD3_SOYBN)%3B contains Interpro domain(s) IPR018957 Zinc finger%2C C3HC4 RING-type
Solyc01g094040.2.1	-2.640717066	3.41E-06	6.94E-05	L1-related	D	Plastocyanin-like domain containing protein expressed (AHRD V1 **-- Q2QRP3_ORYSJ)%3B contains Interpro domain(s) IPR003245 Plastocyanin-like
Solyc01g079260.2.1	-2.616566491	1.62E-06	3.61E-05	L1-related	D	WRKY transcription factor 4 (AHRD V1 ***- A7UGD1_SOLTU)%3B contains Interpro domain(s) IPR003657 DNA-binding WRKY
Solyc07g005430.2.1	-2.579492866	0.00047678	0.004826146	L1-related	D	Unknown Protein (AHRD V1)
Solyc10g080370.1.1	-2.573619768	8.83E-07	2.11E-05	L1-related	D	Unknown Protein (AHRD V1)
Solyc04g081730.2.1	-2.528055713	4.99E-06	9.68E-05	L1-related	D	Unknown Protein (AHRD V1)
Solyc05g007190.2.1	-2.504702903	0.00504982	0.031742372	L1-related	D	Sucrose transporter-like protein (AHRD V1 **** Q9FVL6_SOLLC)%3B contains Interpro domain(s) IPR005989 Sucrose/H+ symporter%2C plant
Solyc05g026540.2.1	-2.493442593	7.19E-07	1.76E-05	L1-related	D	Transmembrane protein 214 (AHRD V1 *-*- TM214_BOVIN)
Solyc01g107170.2.1	-2.455597523	7.14E-08	2.20E-06	L1-related	D	Zinc finger protein (AHRD V1 ***- A0T3Q5_SOLTU)%3B contains Interpro domain(s) IPR007087 Zinc finger%2C C2H2-type
Solyc09g005000.1.1	-2.440297207	0.0060085	0.036497416	L1-related	D	Receptor like protein kinase (AHRD V1 **** Q39139_ARATH)%3B contains Interpro domain(s) IPR001220 Legume lectin%2C beta chain
Solyc08g063070.2.1	-2.431009546	0.0002428	0.002743416	L1-related	D	Esterase/lipase/thioesterase (Fragment) (AHRD V1 **-- B3VW20_POPTN)%3B contains Interpro domain(s) IPR000073 Alpha/beta hydrolase fold-1
Solyc02g068010.2.1	-2.419887614	0.00019963	0.00232172	L1-related	D	DNA-binding protein (Fragment) (AHRD V1 *-*- Q5IRD2_9ROSI)%3B contains Interpro domain(s) IPR006634 TRAM%2C LAG1 and CLN8 homology
Solyc04g071890.2.1	-2.396795016	0.00047793	0.010237846	L1-related	L	Peroxidase 4 (AHRD V1 **** B7UCP4_LITCN)%3B contains Interpro domain(s) IPR002016 Haem peroxidase%2C plant/fungal/bacterial
Solyc05g013780.2.1	-2.396650146	0.00014526	0.001755454	L1-related	D	Unknown Protein (AHRD V1)
Solyc05g056100.2.1	-2.396353242	0.00080806	0.007462108	L1-related	D	Double-stranded RNA binding protein (AHRD V1 *-** A9T6E4_PHYPA)%3B contains Interpro domain(s) IPR001159 Double-stranded RNA binding
Solyc01g081310.2.1	-2.342640116	4.07E-07	1.06E-05	L1-related	D	Glutathione-S-transferase (AHRD V1 ***- B2BY02_9BRAS)%3B contains Interpro domain(s) IPR004045 Glutathione S-transferase%2C N-terminal IPR017933 Glutathione S-transferase/chloride channel%2C C-terminal
Solyc12g096540.1.1	-2.310234054	0.00026144	0.007232947	L1-related	F	40S ribosomal protein S10-like (AHRD V1 ***- Q2XPV4_SOLTU)%3B contains Interpro domain(s) IPR005326 Plectin/S10%2C N-terminal
Solyc03g006500.2.1	-2.275857183	0.00247068	0.01837183	L1-related	D	Receptor-like protein kinase At3g21340 (AHRD V1 **** RLK6_ARATH)%3B contains Interpro domain(s) IPR002290 Serine/threonine protein kinase
Solyc10g054030.1.1	-2.251857362	0.00025534	0.00285499	L1-related	D	AT2G21500 protein (Fragment) (AHRD V1 **-- B9DHX0_ARATH)%3B contains Interpro domain(s) IPR018957 Zinc finger%2C C3HC4 RING-type
Solyc07g061950.2.1	-2.243498942	0.00081789	0.007542789	L1-related	D	Integral membrane single C2 domain protein (AHRD V1 **-- D7L359_ARALY)%3B contains Interpro domain(s) IPR018029 C2 membrane targeting protein
Solyc03g019950.2.1	-2.212378758	8.02E-07	1.93E-05	L1-related	D	Genomic DNA chromosome 5 P1 clone MAC12 (AHRD V1 **-- Q9FFX3_ARATH)%3B contains Interpro domain(s) IPR004328 BRO1
Solyc05g054140.2.1	-2.185883607	0.00621152	0.037452344	L1-related	D	Nucleic acid-binding OB-fold (AHRD V1 ***- A2Q5X5_MEDTR)%3B contains Interpro domain(s) IPR005576 RNA polymerase Rpb7%2C N-terminal
Solyc03g082660.2.1	-2.175891287	5.44E-05	0.00076168	L1-related	D	Major facilitator superfamily domain containing protein 5 (AHRD V1 ***- B2W8V3_PYRTR)%3B contains Interpro domain(s) IPR008509 Protein of unknown function DUF791
Solyc04g018110.1.1	-2.169846973	2.36E-05	0.000374149	L1-related	D	Calmodulin-like protein 1 (AHRD V1 *-*- B4FRR5_MAIZE)%3B contains Interpro domain(s) IPR011992 EF-Hand type
Solyc05g016060.2.1	-2.150208161	0.00302708	0.021483914	L1-related	D	Unknown Protein (AHRD V1)
Solyc02g083750.1.1	-2.134223063	3.28E-05	0.000496471	L1-related	D	Transcription regulator (AHRD V1 *--* D7L304_ARALY)%3B contains Interpro domain(s) IPR007592 Protein of unknown function DUF573
Solyc01g008820.2.1	-2.130905568	0.00034768	0.003702775	L1-related	D	Signal peptide peptidase family protein (AHRD V1 ***- D7LQE4_ARALY)%3B contains Interpro domain(s) IPR007369 Peptidase A22B%2C signal peptide peptidase
Solyc06g035690.2.1	-2.108227855	0.00046095	0.004685885	L1-related	D	Post-GPI attachment to proteins factor 3 (AHRD V1 **-- PGAP3_XENTR)%3B contains Interpro domain(s) IPR007217 Per1-like
Solyc04g012160.2.1	-2.099541984	3.31E-06	6.77E-05	L1-related	D	Serine-threonine protein kinase (AHRD V1 ***- Q09VQ1_WHEAT)%3B contains Interpro domain(s) IPR002290 Serine/threonine protein kinase
Solyc05g051240.1.1	-2.092164088	0.00043635	0.009495197	L1-related	L	Aspartic proteinase nepenthesin I (AHRD V1 ***- A9ZMF9_NEPAL)%3B contains Interpro domain(s) IPR001461 Peptidase A1
Solyc08g076730.2.1	-2.075878155	9.91E-06	0.000175453	L1-related	D	TPR domain protein (AHRD V1 *-*- B6U810_MAIZE)%3B contains Interpro domain(s) IPR011990 Tetratricopeptide-like helical
Solyc02g021400.1.1	-2.065627017	0.0016144	0.013067515	L1-related	D	40S ribosomal protein S28 (AHRD V1 ***- B6T1V2_MAIZE)%3B contains Interpro domain(s) IPR000289 Ribosomal protein S28e
Solyc01g098910.2.1	-2.024661023	0.00046642	0.004731284	L1-related	D	Mitochondrial carrier protein (AHRD V1 ***- C5P0Z3_COCP7)%3B contains Interpro domain(s) IPR001993 Mitochondrial substrate carrier
Solyc03g098020.2.1	-2.017857147	0.00200985	0.015563492	L1-related	D	Hydrolase alpha/beta fold family protein (AHRD V1 **-- D7KRI7_ARALY)%3B contains Interpro domain(s) IPR000073 Alpha/beta hydrolase fold-1
Solyc06g059860.2.1	-1.999223382	7.75E-05	0.001042933	L1-related	D	Unknown Protein (AHRD V1)
Solyc06g066370.2.1	-1.98967673	2.67E-05	0.000413484	L1-related	D	WRKY transcription factor 1 (AHRD V1 **** C9DHZ0_9ROSI)%3B contains Interpro domain(s) IPR003657 DNA-binding WRKY
Solyc06g074710.1.1	-1.975088453	0.00110618	0.009638653	L1-related	D	Hydroxycinnamoyl CoA shikimate/quinate hydroxycinnamoyltransferase-like protein (Fragment) (AHRD V1 **-* B9GF60_POPTR)%3B contains Interpro domain(s) IPR003480 Transferase
Solyc09g061310.2.1	-1.96713749	3.57E-05	0.00053077	L1-related	D	PPPDE peptidase domain containing 2a (AHRD V1 *--- Q6PC81_DANRE)%3B contains Interpro domain(s) IPR008580 Protein of unknown function DUF862%2C eukaryotic

Solyc08g075970.2.1	-1.961427704	0.00332661	0.023120309	L1-related	D	Transmembrane protein 45B (AHRD V1 **-- C3KGS0_ANOFI)%3B contains Interpro domain(s) IPR006904 Protein of unknown function DUF716
Solyc02g022920.1.1	-1.956584683	0.00261881	0.01925724	L1-related	D	Ethylene-responsive nuclear protein (AHRD V1 ***- Q38MV1_SOLLC)
Solyc10g085300.1.1	-1.941321421	0.00124023	0.010548751	L1-related	D	Os03g0210500 protein (Fragment) (AHRD V1 *-*- Q0DU33_ORYSJ)%3B contains Interpro domain(s) IPR007493 Protein of unknown function DUF538
Solyc12g096340.1.1	-1.916155942	0.00238418	0.017881993	L1-related	D	UDP-sugar transporter-like protein (AHRD V1 ***- A8PFD6_BRUMA)%3B contains Interpro domain(s) IPR004853 Protein of unknown function DUF250
Solyc06g068720.2.1	-1.903489149	0.00107728	0.009463994	L1-related	D	Calcium-binding mitochondrial carrier protein SCaMC-1 (AHRD V1 **** B5X2X8_SALSA)%3B contains Interpro domain(s) IPR002067 Mitochondrial carrier protein
Solyc09g010370.2.1	-1.885432589	0.00099041	0.008817889	L1-related	D	Ras-related protein Rab-8A (AHRD V1 ***- C1C123_9MAXI)%3B contains Interpro domain(s) IPR003579 Ras small GTPase%2C Rab type
Solyc02g036450.2.1	-1.866582749	0.0020476	0.015800942	L1-related	D	Ras-related protein Rab-5C (AHRD V1 ***- C1BU54_9MAXI)%3B contains Interpro domain(s) IPR015599 Rab5-related
Solyc01g087160.2.1	-1.815345364	0.00182385	0.014418562	L1-related	D	Unknown Protein (AHRD V1)
Solyc03g120470.2.1	-1.815313478	0.00042031	0.009209619	L1-related	L	Aquaporin (AHRD V1 *-*- O81186_VERFO)%3B contains Interpro domain(s) IPR012269 Aquaporin
Solyc02g080420.2.1	-1.800369191	9.88E-05	0.001288159	L1-related	D	RNA Binding Protein 45 (AHRD V1 **** Q9LEB4_NICPL)%3B contains Interpro domain(s) IPR000504 RNA recognition motif%2C RNP-1
Solyc01g058260.2.1	-1.796119524	0.00084108	0.007725092	L1-related	D	Poly(A) polymerase (AHRD V1 ***- Q56XM9_ARATH)%3B contains Interpro domain(s) IPR007012 Poly(A) polymerase%2C central region
Solyc07g007130.1.1	-1.792857802	0.00426774	0.027703449	L1-related	D	NHL repeat-containing protein (AHRD V1 **-- D7M6N9_ARALY)
Solyc03g096290.2.1	-1.792449396	0.00078161	0.014926653	L1-related	L	Aquaporin-like protein (AHRD V1 ***- Q8W1A9_PETHY)%3B contains Interpro domain(s) IPR012269 Aquaporin
Solyc09g010640.2.1	-1.744249823	0.00093695	0.008428279	L1-related	D	SLT1 protein (AHRD V1 **-- Q9FQY3_TOBAC)
Solyc03g083620.1.1	-1.695053746	0.00011315	0.001440086	L1-related	D	Unknown Protein (AHRD V1)
Solyc06g050990.2.1	-1.683536063	0.00211378	0.016230583	L1-related	D	Multiple inositol polyphosphate phosphatase (AHRD V1 ***- Q54ND5_DICDI)%3B contains Interpro domain(s) IPR016274 Histidine acid phosphatase%2C eukaryotic
Solyc12g013620.1.1	-1.655536757	0.00011789	0.001484063	L1-related	D	NAC domain protein IPR003441 (AHRD V1 ***- B9N1K9_POPTR)%3B contains Interpro domain(s) IPR003441 No apical meristem (NAM) protein
Solyc02g021560.2.1	-1.654633821	0.00032526	0.003493356	L1-related	D	Heterogeneous nuclear ribonucleoprotein A3-like protein 2 (AHRD V1 **-- B6TV58_MAIZE)%3B contains Interpro domain(s) IPR000504 RNA recognition motif%2C RNP-1
Solyc11g071270.1.1	-1.649533211	0.00690758	0.040807523	L1-related	D	Class E vacuolar protein-sorting machinery protein HSE1 (AHRD V1 *--- HSE1_USTMA)%3B contains Interpro domain(s) IPR018205 VHS subgroup
Solyc12g099990.1.1	-1.611779731	0.00306619	0.021598508	L1-related	D	Calmodulin 2 (AHRD V1 ***- Q710C9_BRAOL)%3B contains Interpro domain(s) IPR011992 EF-Hand type
Solyc06g084440.2.1	-1.515605008	0.00414554	0.027087296	L1-related	D	Nuclear protein localization 4 (AHRD V1 ***- D3B583_POLPA)%3B contains Interpro domain(s) IPR007717 NPL4
Solyc04g071770.2.1	-1.397049902	0.00651918	0.03893579	L1-related	D	Ethylene responsive transcription factor 2a (AHRD V1 *-*- C0J9I7_9ROSA)%3B contains Interpro domain(s) IPR001471 Pathogenesis-related transcriptional factor and ERF%2C DNA-binding
Solyc12g009560.1.1	-1.288093284	0.00560557	0.034471774	L1-related	D	F-box/LRR-repeat protein (AHRD V1 *-*- C0S347_PARBP)%3B contains Interpro domain(s) IPR001810 Cyclin-like F-box
Solyc10g081530.1.1	-1.262729716	0.00308306	0.021684374	L1-related	D	V-type proton ATPase subunit d 1 (AHRD V1 ***- D3PHZ2_9MAXI)%3B contains Interpro domain(s) IPR016727 ATPase%2C V0 complex%2C subunit D
Solyc02g078540.2.1	-1.195665524	0.0057634	0.035301747	L1-related	D	Unknown Protein (AHRD V1)%3B contains Interpro domain(s) IPR000996 Clathrin light chain
