# Supplementary material for: Using a periclinal chimera to unravel layer-specific gene expression in plants
Source: Plant J. 2013 Jul 19;75(6):1039–49. doi: 10.1111/tpj.12250 (PMC4223383; doi:10.1111/tpj.12250)
Supplement: Supplementary file 13 [file tpj0075-1039-sd13.pdf]

Supporting Information Table S6. List of genes classified as L2/L3, either specific or related.
Within each class, genes are ordered with decreasing fold change with respect to the interaction effect: i.e. the ratio of the difference of penn allele-specific expression between wild type and chimera over the difference for lyc expresssion. Among all tissue samples, the highest fold change is reported. For each gene, we report which tissues support the classification as L2/L3: L(eaf), D(ehydrated leaf), F(ruit). Fold change, p-values, tissue support and classification as related or specific originate from the differential expression analysis conducted on allele-specific expression values based on polymorphims detected by Varid. After correcting for multiple testing using the Benjamini and Hochberg approach (P adjusted value), a cut-off value of 0.05 was applied for the false discovery rate.

Gene ID	log fold change	P value	P adjusted value	Prediction	Tissues	Human readable description
Solyc09g082340.2.1	10.56206733	2.66E-46	7.40E-43	L2/L3-specific	D,F	Vicilin-like protein (Fragment) (AHRD V1 **-- Q9SEW4_9ROSI)%3B contains Interpro domain(s) IPR014710 RmlC-like jelly roll fold
Solyc07g007250.2.1	9.321266248	2.04E-33	1.67E-30	L2/L3-specific	L,D	Metallocarboxypeptidase inhibitor (AHRD V1 ***- MCPI_SOLLC)%3B contains Interpro domain(s) IPR004231 Proteinase inhibitor I37%2C carboxypeptidase A
Solyc01g099630.2.1	9.021833004	6.92E-33	1.19E-29	L2/L3-specific	D,F	Xyloglucan endotransglucosylase/hydrolase 5 (AHRD V1 **** C0IRG4_ACTDE)%3B contains Interpro domain(s) IPR016455 Xyloglucan endotransglucosylase/hydrolase IPR008263 Glycoside hydrolase%2C family 16%2C active site
Solyc09g084470.2.1	9.015238242	6.97E-31	1.20E-27	L2/L3-specific	L,D	Proteinase inhibitor I (AHRD V1 ***- Q43648_SOLTU)%3B contains Interpro domain(s) IPR000864 Proteinase inhibitor I13%2C potato inhibitor I
Solyc08g082120.2.1	8.482761542	5.77E-28	2.29E-25	L2/L3-specific	L,D	Methanol inducible protein (AHRD V1 ***- D1MAX5_NICBE)
Solyc03g098790.1.1	8.107895729	2.77E-28	3.46E-25	L2/L3-specific	L,D	Kunitz-type protease inhibitor (AHRD V1 **** Q3S488_SOLTU)%3B contains Interpro domain(s) IPR002160 Proteinase inhibitor I3%2C Kunitz legume
Solyc08g082190.2.1	8.024103433	5.96E-32	4.36E-29	L2/L3-specific	L,D,F	Unknown Protein (AHRD V1)
Solyc02g071000.1.1	7.980474819	7.12E-24	6.11E-21	L2/L3-specific	L	Chlorophyll a/b binding protein (AHRD V1 **** Q41425_SOLTU)%3B contains Interpro domain(s) IPR001344 Chlorophyll A-B binding protein
Solyc01g105660.2.1	7.711239891	3.96E-29	2.04E-26	L2/L3-specific	D	1-aminocyclopropane-1-carboxylate oxidase (AHRD V1 **-- ACCO_TOBAC)%3B contains Interpro domain(s) IPR005123 Oxoglutarate and iron-dependent oxygenase
Solyc02g092670.1.1	7.526883857	6.16E-24	1.71E-21	L2/L3-specific	L,D,F	Subtilisin-like protease (AHRD V1 ***- A9XG40_TOBAC)%3B contains Interpro domain(s) IPR015500 Peptidase S8%2C subtilisin-related
Solyc03g112760.2.1	7.448483463	1.28E-12	8.51E-11	L2/L3-specific	L,D	Unknown Protein (AHRD V1)
Solyc02g091560.2.1	7.442515516	2.42E-25	7.48E-23	L2/L3-specific	L,D,F	Serine hydroxymethyltransferase (AHRD V1 **** C6ZJZ0_SOYBN)%3B contains Interpro domain(s) IPR001085 Serine hydroxymethyltransferase
Solyc02g020940.2.1	7.352014591	1.67E-19	6.37E-17	L2/L3-specific	L,D	Glyceraldehyde-3-phosphate dehydrogenase (AHRD V1 **** D7UNZ5_BRARC)%3B contains Interpro domain(s) IPR000173 Glyceraldehyde 3-phosphate dehydrogenase
Solyc07g007760.2.1	7.322202128	9.31E-30	4.98E-27	L2/L3-specific	D	Defensin protein (AHRD V1 ***- B1N681_SOLPI)%3B contains Interpro domain(s) IPR008177 Gamma Purothionin
Solyc07g006030.2.1	7.287333775	1.44E-18	4.72E-16	L2/L3-specific	L,D,F	Protein TIF31 homolog (AHRD V1 *--- TIF31_COCIM)%3B contains Interpro domain(s) IPR011990 Tetratricopeptide-like helical
Solyc02g063150.2.1	7.285979618	7.73E-19	1.29E-16	L2/L3-specific	L,D,F	Ribulose bisphosphate carboxylase small chain (AHRD V1 ***- Q84QE5_TOBAC)%3B contains Interpro domain(s) IPR000894 Ribulose bisphosphate carboxylase%2C small chain
Solyc05g013450.2.1	7.169101064	8.93E-14	7.30E-12	L2/L3-specific	D	Mate efflux family protein (AHRD V1 **-- D7LR70_ARALY)%3B contains Interpro domain(s) IPR002528 Multi antimicrobial extrusion protein MatE
Solyc02g080540.1.1	7.14051226	5.16E-18	1.51E-15	L2/L3-specific	L,D,F	ATP synthase gamma chain (AHRD V1 ***- B7FIR4_MEDTR)%3B contains Interpro domain(s) IPR000131 ATPase%2C F1 complex%2C gamma subunit
Solyc07g006570.2.1	7.126555369	5.20E-16	5.82E-14	L2/L3-specific	D	S8-RNase (Fragment) (AHRD V1 **-- Q5XPJ2_MALDO)%3B contains Interpro domain(s) IPR001568 Ribonuclease T2
Solyc11g066130.1.1	7.088004589	5.02E-17	6.51E-15	L2/L3-specific	D	Thaumatin-like protein (AHRD V1 **-- D2KU75_CITJA)%3B contains Interpro domain(s) IPR017949 Thaumatin%2C conserved site IPR001938 Thaumatin%2C pathogenesis-related
Solyc01g079470.2.1	7.079124218	5.85E-26	1.86E-23	L2/L3-specific	D	CP12 (AHRD V1 ***- O24136_TOBAC)%3B contains Interpro domain(s) IPR003823 Protein of unknown function CP12
Solyc04g077500.2.1	7.028494169	8.19E-17	2.08E-14	L2/L3-specific	L,D	Os03g0729100 protein (Fragment) (AHRD V1 ***- Q0DNX2_ORYSJ)
Solyc06g060340.2.1	6.902893008	1.15E-15	4.15E-13	L2/L3-specific	L,F	Chloroplast photosystem II-associated protein (AHRD V1 ***- A8HPM2_CHLRE)%3B contains Interpro domain(s) IPR001344 Chlorophyll A-B binding protein
Solyc05g013630.1.1	6.847661886	5.11E-22	1.18E-19	L2/L3-specific	L,D,F	Unknown protein (Fragment) (AHRD V1 ***- Q43520_SOLLC)
Solyc06g073260.2.1	6.823188487	3.12E-16	7.25E-14	L2/L3-specific	L,D	NAD-dependent epimerase/dehydratase (AHRD V1 **-- C7QWY4_CYAP0)%3B contains Interpro domain(s) IPR016040 NAD(P)-binding domain
Solyc01g110110.2.1	6.79516217	6.15E-12	3.65E-10	L2/L3-specific	D	Cysteine proteinase cathepsin F (AHRD V1 **-* D3TN89_GLOMM)%3B contains Interpro domain(s) IPR013128 Peptidase C1A%2C papain
Solyc03g119080.2.1	6.716409341	6.88E-15	6.64E-13	L2/L3-specific	L,D	Beta-glucosidase (AHRD V1 **** D7L7Z3_ARALY)%3B contains Interpro domain(s) IPR001360 Glycoside hydrolase%2C family 1
Solyc10g083580.1.1	6.682093672	1.04E-14	3.30E-12	L2/L3-specific	F	Phytosulfokines 5 (AHRD V1 *-*- PSK5_ORYSJ)%3B contains Interpro domain(s) IPR009438 Phytosulfokine
Solyc12g005400.1.1	6.573494411	9.90E-20	1.86E-17	L2/L3-specific	L,D	Cyclic nucleotide gated channel (AHRD V1 ***- A9CRE4_MALDO)%3B contains Interpro domain(s) IPR000595 Cyclic nucleotide-binding
Solyc02g086910.2.1	6.483942163	5.67E-11	2.94E-09	L2/L3-specific	D	Peptidyl-prolyl cis-trans isomerase cyclophilin-type (AHRD V1 ***- A8IRU6_CHLRE)%3B contains Interpro domain(s) IPR002130 Peptidyl-prolyl cis-trans isomerase%2C cyclophilin-type
Solyc11g008440.1.1	6.478195184	1.26E-26	4.62E-24	L2/L3-specific	D	Amino acid transporter (AHRD V1 **** B9HLH9_POPTR)%3B contains Interpro domain(s) IPR013057 Amino acid transporter%2C transmembrane
Solyc05g051750.2.1	6.400295919	3.89E-13	2.84E-11	L2/L3-specific	L,D	Prosystemin (AHRD V1 ***- Q5MBK9_BETVU)%3B contains Interpro domain(s) IPR009966 Prosystemin
Solyc12g009800.1.1	6.369602675	2.81E-12	1.76E-10	L2/L3-specific	D	Purple acid phosphatase 3 (AHRD V1 ***- Q6J5M8_SOLTU)%3B contains Interpro domain(s) IPR015914 Purple acid phosphatase%2C N-terminal
Solyc08g077900.2.1	6.368079862	9.79E-14	7.86E-12	L2/L3-specific	D	Expansin-like protein (AHRD V1 ***- Q0WRS3_ARATH)%3B contains Interpro domain(s) IPR007112 Expansin 45%2C endoglucanase-like
Solyc10g050440.1.1	6.354259811	4.49E-11	2.38E-09	L2/L3-specific	L,D	Unknown Protein (AHRD V1)
Solyc01g095900.2.1	6.338510621	3.40E-21	7.27E-19	L2/L3-specific	L,D	Protein TIF31 homolog (AHRD V1 *--- TIF31_ASPCL)%3B contains Interpro domain(s) IPR011990 Tetratricopeptide-like helical
Solyc01g105050.2.1	6.320976666	3.46E-13	5.52E-11	L2/L3-specific	L	Chlorophyll a-b binding protein%2C chloroplastic (AHRD V1 **** CB12_PETHY)%3B contains Interpro domain(s) IPR001344 Chlorophyll A-B binding protein
Solyc08g083060.2.1	6.285504183	2.53E-11	1.39E-09	L2/L3-specific	L,D	Oligopeptide transporter (Fragment) (AHRD V1 **** B0DAK3_LACBS)%3B contains Interpro domain(s) IPR004813 Oligopeptide transporter OPT superfamily
Solyc01g098120.2.1	6.277542122	7.49E-12	4.37E-10	L2/L3-specific	D	Unknown Protein (AHRD V1)
Solyc08g076970.2.1	6.270495241	3.74E-13	2.77E-11	L2/L3-specific	D	Acetylornithine deacetylase or succinyl-diaminopimelate desuccinylase (AHRD V1 **-- D4MFM7_9ENTE)%3B contains Interpro domain(s) IPR011650 Peptidase M20%2C dimerisation
Solyc10g086580.1.1	6.264287146	7.92E-29	1.09E-25	L2/L3-specific	L,D,F	Ribulose-1 5-bisphosphate carboxylase/oxygenase activase 1 (AHRD V1 ***- Q9AXG1_GOSHI)%3B contains Interpro domain(s) IPR003959 ATPase%2C AAA-type%2C core
Solyc12g094640.1.1	6.233339336	8.88E-26	9.38E-23	L2/L3-specific	L	Glyceraldehyde-3-phosphate dehydrogenase B (AHRD V1 ***- B4F8L7_MAIZE)%3B contains Interpro domain(s) IPR000173 Glyceraldehyde 3-phosphate dehydrogenase
Solyc02g070800.2.1	6.230833995	2.15E-36	2.49E-33	L2/L3-specific	L,D	Ycf23 protein (AHRD V1 ***- Q7NE74_GLOVI)%3B contains Interpro domain(s) IPR007570 Protein of unknown function DUF561
Solyc10g006970.2.1	6.226518119	1.25E-12	8.32E-11	L2/L3-specific	L,D	Thioredoxin m (AHRD V1 ***- B9HZV8_POPTR)%3B contains Interpro domain(s) IPR005746 Thioredoxin
Solyc01g094890.2.1	6.2263855	1.05E-12	7.11E-11	L2/L3-specific	L,D	Ferric reductase oxidase (AHRD V1 **-* D6RVS5_HORVU)%3B contains Interpro domain(s) IPR013121 Ferric reductase%2C NAD binding
Solyc03g044200.2.1	6.20402611	1.72E-12	4.07E-10	L2/L3-specific	F	Alcohol dehydrogenase (AHRD V1 **** Q43169_SOLTU)%3B contains Interpro domain(s) IPR002085 Alcohol dehydrogenase superfamily%2C zinc-containing
Solyc05g013640.1.1	6.182220336	1.31E-12	8.56E-11	L2/L3-specific	D	Unknown Protein (AHRD V1)
Solyc06g062370.2.1	6.160502701	3.13E-08	1.04E-06	L2/L3-specific	L,D,F	Acid phosphatase (AHRD V1 **-- O49855_SOYBN)%3B contains Interpro domain(s) IPR010028 Acid phosphatase%2C plant
Solyc03g082920.2.1	6.150298985	2.05E-12	4.69E-10	L2/L3-specific	F	Heat shock protein (AHRD V1 ***- Q84KP8_CYAME)%3B contains Interpro domain(s) IPR013126 Heat shock protein 70
Solyc09g011710.2.1	6.090975713	9.17E-12	1.18E-09	L2/L3-specific	L,D,F	class I heat shock protein 3 (AHRD V1 ***- B4F976_MAIZE)%3B contains Interpro domain(s) IPR008978 HSP20-like chaperone
Solyc08g006930.2.1	6.083419811	1.21E-21	6.64E-19	L2/L3-specific	L,D,F	Photosystem I reaction center subunit X psaK (AHRD V1 ***- Q84QE6_TOBAC)%3B contains Interpro domain(s) IPR017493 Photosystem I reaction center%2C PsaK%2C plant
Solyc07g006520.2.1	5.956496679	3.41E-09	1.34E-07	L2/L3-specific	D	Preprotein translocase secY subunit (AHRD V1 ***- A8IS14_CHLRE)%3B contains Interpro domain(s) IPR002208 SecY protein
Solyc10g054420.1.1	5.907545587	2.80E-09	1.11E-07	L2/L3-specific	L,D	Photosystem II oxygen evolving complex protein PsbP (AHRD V1 ***- A0YXJ6_LYNSP)%3B contains Interpro domain(s) IPR002683 Photosystem II oxygen evolving complex protein PsbP
Solyc01g095530.2.1	5.864690936	7.09E-11	1.23E-08	L2/L3-specific	F	Unknown Protein (AHRD V1)
Solyc04g009030.2.1	5.803566631	8.85E-24	7.15E-21	L2/L3-specific	L,D,F	Glyceraldehyde-3-phosphate dehydrogenase (AHRD V1 **** D7UNZ5_BRARC)%3B contains Interpro domain(s) IPR000173 Glyceraldehyde 3-phosphate dehydrogenase
Solyc09g011810.2.1	5.778632065	5.13E-09	1.97E-07	L2/L3-specific	L,D,F	Fructose-1 6-bisphosphatase class 1 (AHRD V1 **** D0MHZ8_RHOM4)%3B contains Interpro domain(s) IPR000146 Fructose-1%2C6-bisphosphatase
Solyc02g014150.2.1	5.775550041	1.96E-13	1.52E-11	L2/L3-specific	L,D	Photosystem II stability/assembly factor Ycf48-like protein (AHRD V1 **-- B0C6T3_ACAM1)%3B contains Interpro domain(s) IPR016705 Photosystem II stability/assembly factor%2C HCF136
Solyc02g079780.1.1	5.766461169	2.32E-09	9.26E-08	L2/L3-specific	D	Pro-resilin (AHRD V1 *--- B6T4Q5_MAIZE)
Solyc08g079890.1.1	5.756797045	9.69E-10	4.16E-08	L2/L3-specific	D	Subtilisin-like protease (AHRD V1 **-- Q9LWA3_SOLLC)%3B contains Interpro domain(s) IPR015500 Peptidase S8%2C subtilisin-related
Solyc03g071590.2.1	5.749397753	4.89E-09	1.88E-07	L2/L3-specific	L,D	Malate dehydrogenase (AHRD V1 **** Q5NE17_SOLLC)%3B contains Interpro domain(s) IPR011273 Malate dehydrogenase%2C NADP-dependent%2C plants
Solyc03g080180.2.1	5.73964835	3.77E-10	3.78E-08	L2/L3-specific	L,F	O-methyltransferase (AHRD V1 **** Q9LWB8_9ROSI)%3B contains Interpro domain(s) IPR016461 O-methyltransferase%2C COMT%2C eukaryota
Solyc02g036350.2.1	5.730338176	2.39E-26	8.30E-24	L2/L3-specific	D,F	1-aminocyclopropane-1-carboxylate oxidase (AHRD V1 ***- A4ZYQ6_SOLLC)%3B contains Interpro domain(s) IPR005123 Oxoglutarate and iron-dependent oxygenase
Solyc02g065400.2.1	5.712426043	6.82E-19	2.34E-16	L2/L3-specific	L,D,F	Oxygen-evolving enhancer protein 1 of photosystem II (AHRD V1 ***- A8J0E4_CHLRE)%3B contains Interpro domain(s) IPR002628 Photosystem II manganese-stabilizing protein PsbO
Solyc03g005780.1.1	5.696246531	1.03E-21	5.89E-19	L2/L3-specific	L	Chlorophyll a-b binding protein 3C-like (AHRD V1 **** Q2XTE0_SOLTU)%3B contains Interpro domain(s) IPR001344 Chlorophyll A-B binding protein
Solyc01g110360.2.1	5.691472307	5.49E-15	5.37E-13	L2/L3-specific	L,D,F	Fructose-bisphosphate aldolase (AHRD V1 ***- B0FPD8_SOLTU)%3B contains Interpro domain(s) IPR000741 Fructose-bisphosphate aldolase%2C class-I
Solyc07g054860.1.1	5.677791673	1.03E-09	1.43E-07	L2/L3-specific	F	Aromatic amino acid decarboxylase (AHRD V1 **** D4NUX2_CIMRA)%3B contains Interpro domain(s) IPR010977 Aromatic-L-amino-acid decarboxylase
Solyc10g007110.2.1	5.658154243	1.63E-09	6.65E-08	L2/L3-specific	D,F	Tyrosine aminotransferase (AHRD V1 **** Q8GUE9_SOLSC)%3B contains Interpro domain(s) IPR005958 Tyrosine/nicotianamine aminotransferase
Solyc03g111730.2.1	5.654880154	1.27E-09	5.25E-08	L2/L3-specific	D	Cathepsin B-like cysteine proteinase (AHRD V1 **-* CYSP_SCHMA)%3B contains Interpro domain(s) IPR013128 Peptidase C1A%2C papain
Solyc04g077010.2.1	5.652025482	4.15E-18	6.14E-16	L2/L3-specific	L,D	Receptor like kinase%2C RLK
Solyc01g095320.2.1	5.610418375	1.04E-24	1.19E-21	L2/L3-specific	L,D,F	BCL-2-associated athanogene 6 (AHRD V1 *-*- D7LDX9_ARALY)%3B contains Interpro domain(s) IPR003103 Apoptosis regulator Bcl-2 protein%2C BAG
Solyc02g070970.1.1	5.610403939	9.61E-24	7.33E-21	L2/L3-specific	L,F	Chlorophyll a/b binding protein (AHRD V1 **** Q41422_SOLTU)%3B contains Interpro domain(s) IPR001344 Chlorophyll A-B binding protein
Solyc11g012930.1.1	5.585843587	9.99E-10	4.25E-08	L2/L3-specific	D	Nodulin family protein (AHRD V1 ***- A7L2Z5_GOSHI)%3B contains Interpro domain(s) IPR000620 Protein of unknown function DUF6%2C transmembrane
Solyc09g065560.2.1	5.584346686	1.56E-08	1.13E-06	L2/L3-specific	L	Sulfate transporter (AHRD V1 **** D7LTZ8_ARALY)%3B contains Interpro domain(s) IPR001902 Sulphate anion transporter
Solyc05g006440.2.1	5.57844009	8.53E-09	3.12E-07	L2/L3-specific	D	Homology to unknown gene (AHRD V1 *--- Q00X05_OSTTA)
Solyc03g115230.2.1	5.574965211	2.75E-13	7.02E-11	L2/L3-specific	F	ClpB chaperone (AHRD V1 ***- D6QXI5_TRYCR)%3B contains Interpro domain(s) IPR013093 ATPase associated with various cellular activities%2C AAA-2
Solyc09g011080.2.1	5.571514754	1.65E-09	1.49E-07	L2/L3-specific	L	Ribulose-1 5-bisphosphate carboxylase/oxygenase activase 1 (AHRD V1 **** Q9AXG1_GOSHI)%3B contains Interpro domain(s) IPR003959 ATPase%2C AAA-type%2C core
Solyc08g081940.2.1	5.56843448	2.62E-07	7.12E-06	L2/L3-specific	D	Receptor like kinase%2C RLK
Solyc02g093180.2.1	5.566857576	2.43E-16	2.89E-14	L2/L3-specific	D	N-hydroxycinnamoyl/benzoyltransferase 3 (AHRD V1 **-- Q00M87_SOYBN)%3B contains Interpro domain(s) IPR003480 Transferase
Solyc07g009130.2.1	5.56542259	1.11E-09	4.63E-08	L2/L3-specific	D	Potassium-transporting ATPase B chain (AHRD V1 *-*- ATKB_BACHK)%3B contains Interpro domain(s) IPR006404 ATPase%2C P-type%2C heavy metal-(Cd/Co/Hg/Pb/Zn)-translocating
Solyc08g005560.2.1	5.554391219	9.72E-09	3.50E-07	L2/L3-specific	D	Binding protein (AHRD V1 ***- D7MGU4_ARALY)
Solyc02g085760.2.1	5.516067681	3.24E-09	2.73E-07	L2/L3-specific	L,D	Rhomboid family protein (AHRD V1 ***- D7MJX8_ARALY)%3B contains Interpro domain(s) IPR002610 Peptidase S54%2C rhomboid
Solyc06g007180.2.1	5.516065427	3.70E-26	1.25E-23	L2/L3-specific	D	Asparagine synthase (Glutamine-hydrolyzing) (AHRD V1 **** A9EDI4_9FLAO)%3B contains Interpro domain(s) IPR006426 Asparagine synthase%2C glutamine-hydrolyzing
Solyc06g074000.1.1	5.501430401	3.16E-09	4.05E-07	L2/L3-specific	F	Aspartic proteinase nepenthesin-2 (AHRD V1 **-- B6SJT4_MAIZE)%3B contains Interpro domain(s) IPR001461 Peptidase A1

Solyc12g013810.1.1	5.494933958	5.19E-09	4.27E-07	L2/L3-specific	L,D	Thioredoxin (AHRD V1 ***- A7LNX7_9CARY)%3B contains Interpro domain(s) IPR005746 Thioredoxin
Solyc06g053140.2.1	5.492432217	3.98E-09	3.31E-07	L2/L3-specific	L,D,F	Senescence-associated protein DIN1 (AHRD V1 *--- B6U016_MAIZE)%3B contains Interpro domain(s) IPR001763 Rhodanese-like
Solyc08g076290.2.1	5.489700768	4.63E-09	3.83E-07	L2/L3-specific	L	Unknown Protein (AHRD V1)%3B contains Interpro domain(s) IPR007300 LrgB-like protein
Solyc10g008020.2.1	5.48644043	1.59E-08	1.14E-06	L2/L3-specific	L,D	Methyltransferase (AHRD V1 *-*- B8H501_CAUCN)%3B contains Interpro domain(s) IPR013216 Methyltransferase type 11
Solyc01g100490.2.1	5.464648283	1.55E-12	1.01E-10	L2/L3-specific	L,D	Nicotianamine synthase (AHRD V1 ***- A3DUI9_MALXI)%3B contains Interpro domain(s) IPR004298 Nicotianamine synthase
Solyc11g044910.1.1	5.451472369	2.07E-28	8.99E-26	L2/L3-specific	D	Beta-xylosidase 1 (AHRD V1 ***- D7MNR6_ARALY)%3B contains Interpro domain(s) IPR001764 Glycoside hydrolase%2C family 3%2C N-terminal
Solyc02g086730.1.1	5.446338113	8.64E-09	3.15E-07	L2/L3-specific	L,D	50S ribosomal protein L12-C (AHRD V1 ***- Q8LBJ7_ARATH)%3B contains Interpro domain(s) IPR015608 Ribosomal protein L12%2C chloroplast
Solyc09g083130.2.1	5.407670716	6.31E-08	1.97E-06	L2/L3-specific	L,D	Peptidase S9 prolyl oligopeptidase active site domain protein (AHRD V1 *-*- D1C565_SPHTD)%3B contains Interpro domain(s) IPR001375 Peptidase S9%2C prolyl oligopeptidase active site region
Solyc12g010380.1.1	5.4036343	2.12E-08	1.46E-06	L2/L3-specific	L	Adenylate kinase-like protein (AHRD V1 ***- Q8VYL1_ARATH)%3B contains Interpro domain(s) IPR018962 Domain of unknown function DUF1995
Solyc05g009570.2.1	5.403445745	8.43E-08	2.55E-06	L2/L3-specific	D	Ribosomal protein PSRP-3/Ycf65 (AHRD V1 *-*- B5W261_SPIMA)%3B contains Interpro domain(s) IPR006924 Ribosomal protein%2C PSRP-3/Ycf65
Solyc02g083280.2.1	5.400078644	2.05E-31	1.42E-28	L2/L3-specific	L,D,F	Thiosulfate sulfurtransferase/rhodanese-like domain-containing protein 1 (AHRD V1 *--* TSTD1_HUMAN)%3B contains Interpro domain(s) IPR001763 Rhodanese-like
Solyc03g025410.2.1	5.390619669	1.53E-08	1.12E-06	L2/L3-specific	L,D	Dehydrogenase/reductase SDR family member 13 (AHRD V1 ***- DHR13_BOVIN)%3B contains Interpro domain(s) IPR002347 Glucose/ribitol dehydrogenase
Solyc10g050110.1.1	5.387635419	1.36E-10	1.50E-08	L2/L3-specific	L	LRR receptor-like serine/threonine-protein kinase%2C RLP
Solyc07g041920.2.1	5.37193548	1.01E-08	7.66E-07	L2/L3-specific	L	Cathepsin L-like cysteine proteinase (AHRD V1 **-* A7LJ78_DERVA)%3B contains Interpro domain(s) IPR013128 Peptidase C1A%2C papain
Solyc06g069070.1.1	5.369918144	1.39E-08	4.94E-07	L2/L3-specific	D	Lipid transfer protein (AHRD V1 ***- A5JUZ7_SESIN)%3B contains Interpro domain(s) IPR013770 Plant lipid transfer protein and hydrophobic protein%2C helical
Solyc10g074920.1.1	5.356950958	3.18E-08	1.05E-06	L2/L3-specific	D	Mannan endo-1 4-beta-mannosidase (AHRD V1 ***- B4ADW3_BACPU)%3B contains Interpro domain(s) IPR001547 Glycoside hydrolase%2C family 5
Solyc06g061200.1.1	5.354696577	1.36E-08	1.00E-06	L2/L3-specific	L,F	Glycine-rich protein TomR2 (AHRD V1 *-*- Q7XJI7_SOLLC)
Solyc04g058150.2.1	5.347938692	1.26E-08	9.37E-07	L2/L3-specific	L,D,F	Type 2 metallothionein (AHRD V1 ***- B3VKV5_SOLNI)%3B contains Interpro domain(s) IPR000347 Plant metallothionein%2C family 15
Solyc08g063040.2.1	5.34598769	1.95E-08	6.72E-07	L2/L3-specific	L,D	Zinc finger protein (AHRD V1 *-*- Q9LVQ7_ARATH)%3B contains Interpro domain(s) IPR007087 Zinc finger%2C C2H2-type
Solyc12g013710.1.1	5.345542964	2.53E-14	4.64E-12	L2/L3-specific	L	Protochlorophyllide reductase (AHRD V1 ***- Q8LAV9_ARATH)%3B contains Interpro domain(s) IPR005979 Light-dependent protochlorophyllide reductase
Solyc05g005880.2.1	5.34450459	9.62E-14	7.78E-12	L2/L3-specific	D	30S ribosomal protein S13 (AHRD V1 ***- B6TG65_MAIZE)%3B contains Interpro domain(s) IPR019980 Ribosomal protein S13%2C bacterial-type
Solyc08g078870.1.1	5.323827325	4.17E-07	1.08E-05	L2/L3-specific	D	Proline-rich protein (AHRD V1 **-- Q41125_PHAVU)%3B contains Interpro domain(s) IPR013770 Plant lipid transfer protein and hydrophobic protein%2C helical
Solyc01g080280.2.1	5.316345168	2.99E-14	5.41E-12	L2/L3-specific	L,D	Glutamine synthetase (AHRD V1 ***- B5LAU9_CAPAN)%3B contains Interpro domain(s) IPR008146 Glutamine synthetase%2C catalytic region
Solyc03g058190.2.1	5.300413445	4.81E-08	3.08E-06	L2/L3-specific	L,D	Elongation factor family GTP-binding protein (AHRD V1 **** Q67QM4_SYMTH)%3B contains Interpro domain(s) IPR006298 GTP-binding protein TypA
Solyc03g005000.2.1	5.2961578	9.07E-13	6.18E-11	L2/L3-specific	D	Peptidase M48 Ste24p (AHRD V1 ***- B6TA12_MAIZE)%3B contains Interpro domain(s) IPR001915 Peptidase M48%2C Ste24p
Solyc12g011450.1.1	5.284635835	2.17E-22	1.35E-19	L2/L3-specific	L,F	Chlorophyll a-b binding protein 13%2C chloroplastic (AHRD V1 ***- CB23_SOLLC)%3B contains Interpro domain(s) IPR001344 Chlorophyll A-B binding protein
Solyc10g006800.2.1	5.259230061	1.65E-08	5.73E-07	L2/L3-specific	L,D	Cyclic nucleotide gated channel (AHRD V1 **** A9CRE4_MALDO)%3B contains Interpro domain(s) IPR000595 Cyclic nucleotide-binding
Solyc01g108630.2.1	5.257531808	4.12E-08	2.71E-06	L2/L3-specific	L	Nitrite reductase (AHRD V1 ***- Q76KB0_TOBAC)%3B contains Interpro domain(s) IPR006067 Nitrite and sulphite reductase 4Fe-4S region
Solyc08g080050.2.1	5.252552369	7.91E-08	4.93E-06	L2/L3-specific	L,D	PGR5-like protein 1A%2C chloroplastic (AHRD V1 ***- PGL1A_ARATH)
Solyc07g052480.2.1	5.244411025	1.94E-08	6.68E-07	L2/L3-specific	D,F	Isocitrate lyase (AHRD V1 **** Q9FQD2_IPOBA)%3B contains Interpro domain(s) IPR006254 Isocitrate lyase
Solyc01g099190.2.1	5.242907854	7.82E-23	7.15E-20	L2/L3-specific	F	Lipoxygenase (AHRD V1 **** Q42873_SOLLC)%3B contains Interpro domain(s) IPR001246 Lipoxygenase%2C plant
Solyc04g011440.2.1	5.238928186	5.96E-18	2.82E-15	L2/L3-specific	F	heat shock protein (AHRD V1 ***- B2D2G5_CAPSN)%3B contains Interpro domain(s) IPR013126 Heat shock protein 70
Solyc10g084180.1.1	5.219260632	3.46E-08	1.13E-06	L2/L3-specific	D	C2H2L domain class transcription factor (AHRD V1 **-* D9ZIU4_MALDO)%3B contains Interpro domain(s) IPR007087 Zinc finger%2C C2H2-type
Solyc12g055840.1.1	5.176818977	4.70E-07	1.20E-05	L2/L3-specific	D	Glucan endo-1 3-beta-glucosidase 7 (AHRD V1 ***- B6T478_MAIZE)%3B contains Interpro domain(s) IPR000490 Glycoside hydrolase%2C family 17
Solyc05g009500.2.1	5.176376557	1.15E-07	6.85E-06	L2/L3-specific	L	Peptide transporter (AHRD V1 **-* A6YJX4_9MAGN)%3B contains Interpro domain(s) IPR000109 TGF-beta receptor%2C type I/II extracellular region
Solyc10g055630.1.1	5.162234344	3.91E-07	1.02E-05	L2/L3-specific	L,D,F	Aquaporin (AHRD V1 ***- O8W506_TOBAC)%3B contains Interpro domain(s) IPR012269 Aquaporin
Solyc07g054210.2.1	5.136914677	4.69E-11	8.58E-09	L2/L3-specific	L,F	Protochlorophyllide reductase like protein (AHRD V1 ***- Q0WVW0_ARATH)%3B contains Interpro domain(s) IPR005979 Light-dependent protochlorophyllide reductase
Solyc03g044330.1.1	5.121393255	4.88E-13	1.20E-10	L2/L3-specific	F	Acetolactate synthase (AHRD V1 **** D7RPJ7_SOLTU)%3B contains Interpro domain(s) IPR012846 Acetolactate synthase%2C large subunit%2C biosynthetic
Solyc06g068500.2.1	5.116717962	1.31E-14	1.23E-12	L2/L3-specific	D	Chaperone protein DnaJ 1 (AHRD V1 *-*- D8USE3_9MICC)%3B contains Interpro domain(s) IPR001623 Heat shock protein DnaJ%2C N-terminal
Solyc12g006470.1.1	5.105064071	4.46E-13	3.22E-11	L2/L3-specific	L,D,F	Aminotransferase-like protein (AHRD V1 ***- Q9LIE2_ARATH)%3B contains Interpro domain(s) IPR005814 Aminotransferase class-III
Solyc01g097860.2.1	5.082998265	2.02E-07	1.18E-05	L2/L3-specific	L	Membrane protein (AHRD V1 *-*- A8JG04_CHLRE)%3B contains Interpro domain(s) IPR010903 Protein of unknown function DUF1517
Solyc05g056480.2.1	5.07426879	1.30E-07	3.74E-06	L2/L3-specific	D	Pyruvate dehydrogenase E1 component alpha subunit (AHRD V1 **** D6YUM5_WADCW)%3B contains Interpro domain(s) IPR001017 Dehydrogenase%2C E1 component
Solyc04g071340.2.1	5.068786798	2.41E-07	1.40E-05	L2/L3-specific	L,D,F	Fructose-1 6-bisphosphatase class 1 (AHRD V1 **** D3PE46_DEFDS)%3B contains Interpro domain(s) IPR000146 Fructose-1%2C6-bisphosphatase
Solyc10g044470.1.1	5.066494734	2.10E-07	1.72E-05	L2/L3-specific	F	Voltage-gated chloride channel (AHRD V1 **** Q96325_ARATH)%3B contains Interpro domain(s) IPR002251 Chloride channel plant CLC
Solyc02g080210.2.1	5.051430011	8.93E-07	4.46E-05	L2/L3-specific	L	Pectinesterase (AHRD V1 ***- B9RXQ4_RICCO)%3B contains Interpro domain(s) IPR000070 Pectinesterase%2C catalytic
Solyc10g080040.1.1	5.042244784	3.23E-07	8.59E-06	L2/L3-specific	D	Ribonuclease 3 (AHRD V1 *-*- C3BNS5_9BACI)%3B contains Interpro domain(s) IPR000999 Ribonuclease III
Solyc09g064630.2.1	5.036371777	2.99E-07	1.70E-05	L2/L3-specific	L	TPR domain protein (AHRD V1 *--- B6U810_MAIZE)%3B contains Interpro domain(s) IPR011990 Tetratricopeptide-like helical
Solyc06g068520.2.1	5.033803313	3.27E-07	2.55E-05	L2/L3-specific	F	Hydroxyproline-rich systemin (AHRD V1 ***- HSY1_SOLLC)
Solyc07g056420.2.1	5.033090705	1.15E-07	1.03E-05	L2/L3-specific	F	Glutathione S-transferase-like protein (AHRD V1 **** Q8GVD1_SOLLC)%3B contains Interpro domain(s) IPR004046 Glutathione S-transferase%2C C-terminal
Solyc10g086150.1.1	5.023678956	3.54E-07	1.98E-05	L2/L3-specific	L,D	Single-stranded DNA binding protein (AHRD V1 **-* Q8W214_SOLTU)%3B contains Interpro domain(s) IPR000504 RNA recognition motif%2C RNP-1
Solyc08g081010.2.1	5.021551818	1.44E-07	1.25E-05	L2/L3-specific	F	Glutamate-cysteine ligase (AHRD V1 **** Q7Y0R4_BRAJU)%3B contains Interpro domain(s) IPR011556 Glutamate--cysteine ligase%2C plant
Solyc07g064900.2.1	5.015964555	2.00E-07	1.64E-05	L2/L3-specific	F	Extracellular ligand-gated ion channel (AHRD V1 ***- D7LAY2_ARALY)
Solyc08g013730.2.1	5.00781878	1.76E-06	8.32E-05	L2/L3-specific	L	Aquaporin (AHRD V1 ***- B3GK86_ARATH)%3B contains Interpro domain(s) IPR000425 Major intrinsic protein
Solyc06g005710.2.1	5.005695608	4.13E-10	4.08E-08	L2/L3-specific	L	cDNA clone 002-143-C11 full insert sequence (AHRD V1 *--- B7F1B2_ORYSJ)%3B contains Interpro domain(s) IPR016040 NAD(P)-binding domain
Solyc03g119060.2.1	5.002397065	1.24E-18	2.00E-16	L2/L3-specific	D	Alpha-dioxygenase 2 (AHRD V1 **-- O5WM33_SOLLC)%3B contains Interpro domain(s) IPR002007 Haem peroxidase%2C animal
Solyc05g056070.2.1	4.973497175	3.02E-12	4.27E-10	L2/L3-specific	L	Chlorophyll a-b binding protein 6A%2C chloroplastic (AHRD V1 ***- CB11_SOLLC)%3B contains Interpro domain(s) IPR001344 Chlorophyll A-B binding protein
Solyc03g119540.2.1	4.960371618	8.18E-07	4.16E-05	L2/L3-specific	L	CONSTANS-like zinc finger protein (AHRD V1 **-- D0EP06_SOYBN)%3B contains Interpro domain(s) IPR010402 CCT domain
Solyc05g008350.2.1	4.956188045	1.07E-06	5.23E-05	L2/L3-specific	L,D	ABC transporter G family member 7 (AHRD V1 **** AB7G_ARATH)%3B contains Interpro domain(s) IPR013525 ABC-2 type transporter
Solyc06g060110.2.1	4.92754504	2.08E-10	9.98E-09	L2/L3-specific	L,D	Amino acid permease (AHRD V1 **** B9GU10_POPTR)%3B contains Interpro domain(s) IPR013057 Amino acid transporter%2C transmembrane
Solyc01g096240.2.1	4.914705378	9.07E-07	2.16E-05	L2/L3-specific	D	Homology to unknown gene (AHRD V1 ***- Q01FV7_OSTTA)
Solyc06g076630.2.1	4.913869486	1.25E-06	2.87E-05	L2/L3-specific	D	Peroxidase (AHRD V1 **** C0KKH9_9CARY)%3B contains Interpro domain(s) IPR002016 Haem peroxidase%2C plant/fungal/bacterial
Solyc06g051930.2.1	4.909331188	6.01E-07	4.38E-05	L2/L3-specific	L,F	Pyruvate kinase family protein (AHRD V1 ***- D7LSF1_ARALY)%3B contains Interpro domain(s) IPR001697 Pyruvate kinase
Solyc04g014400.2.1	4.904208516	1.03E-06	5.04E-05	L2/L3-specific	L,D	LRR receptor-like serine/threonine-protein kinase%2C RLP
Solyc08g016150.1.1	4.901311521	2.03E-06	9.40E-05	L2/L3-specific	L	Avr9/Cf-9 rapidly elicited protein 180 (AHRD V1 ***- Q9FQZ1_TOBAC)
Solyc03g096850.2.1	4.877192066	5.45E-09	2.06E-07	L2/L3-specific	L,D	Genomic DNA chromosome 5 P1 clone MKP11 (AHRD V1 **-- Q9FFJ2_ARATH)%3B contains Interpro domain(s) IPR001478 PDZ/DHR/GLGF
Solyc01g106820.2.1	4.871626012	4.89E-07	1.23E-05	L2/L3-specific	D	Peptidase M50 family (AHRD V1 ***- B6UET0_MAIZE)
Solyc12g005180.1.1	4.869442849	5.04E-07	1.27E-05	L2/L3-specific	D	Chloroplast lipocalin (AHRD V1 ***- Q38JB4_SOLTU)%3B contains Interpro domain(s) IPR013208 Lipocalin-like
Solyc06g065270.2.1	4.868534743	1.81E-06	8.50E-05	L2/L3-specific	L,D	Adenylate kinase (AHRD V1 **** B6STL7_MAIZE)%3B contains Interpro domain(s) IPR006259 Adenylate kinase%2C subfamily
Solyc01g097910.2.1	4.844515992	8.90E-11	4.47E-09	L2/L3-specific	L,D	Rubredoxin family protein (AHRD V1 *-*- D7KP11_ARALY)%3B contains Interpro domain(s) IPR004039 Rubredoxin-type Fe(Cys)4 protein
Solyc07g056540.2.1	4.836985852	2.31E-20	9.93E-18	L2/L3-specific	L,D,F	L-lactate dehydrogenase (AHRD V1 ***- C0S8Q7_PARBP)%3B contains Interpro domain(s) IPR012133 Alpha-hydroxy acid dehydrogenase%2C FMN-dependent
Solyc10g074580.1.1	4.834789244	9.12E-07	2.16E-05	L2/L3-specific	D	Aminoacylase ACY1 and related metalloexopeptidases (ISS) (AHRD V1 *-*- Q00SR9_OSTTA)
Solyc01g095330.2.1	4.820276299	5.55E-07	4.07E-05	L2/L3-specific	F	Unknown Protein (AHRD V1)
Solyc01g006020.2.1	4.817140925	1.92E-16	2.32E-14	L2/L3-specific	D	At3g32930-like protein (Fragment) (AHRD V1 *-*- B3SKU7_ARAHA)
Solyc08g067320.1.1	4.815112779	1.69E-06	8.07E-05	L2/L3-specific	L	Chlorophyll a/b binding protein (AHRD V1 ***- Q41422_SOLTU)%3B contains Interpro domain(s) IPR001344 Chlorophyll A-B binding protein
Solyc03g045070.1.1	4.809007416	2.13E-06	9.73E-05	L2/L3-specific	L	Ammonium transporter (AHRD V1 **** B9HP47_POPTR)%3B contains Interpro domain(s) IPR018047 Ammonium transporter%2C conserved site IPR001905 Ammonium transporter
Solyc00g136260.1.1	4.791453207	3.04E-10	1.41E-08	L2/L3-specific	D	Ring H2 finger protein (AHRD V1 *-*- D9ZHD8_HYPPE)%3B contains Interpro domain(s) IPR018957 Zinc finger%2C C3HC4 RING-type
Solyc11g010480.1.1	4.790720659	1.72E-06	8.17E-05	L2/L3-specific	L,D	Threonine endopeptidase (AHRD V1 *--- D7M9J7_ARALY)
Solyc06g073090.2.1	4.788640783	4.01E-16	4.65E-14	L2/L3-specific	D	Ribosomal subunit interface protein (AHRD V1 **-- Q2JIS4_SYNJB)%3B contains Interpro domain(s) IPR003489 Ribosomal protein S30Ae/sigma 54 modulation protein
Solyc07g064170.2.1	4.787820695	4.90E-21	3.73E-18	L2/L3-specific	F	Pectinesterase (AHRD V1 ***- B9RXQ4_RICCO)%3B contains Interpro domain(s) IPR018040 Pectinesterase%2C active site IPR000070 Pectinesterase%2C catalytic
Solyc12g005630.1.1	4.772667532	8.63E-11	4.35E-09	L2/L3-specific	L,D	Cytochrome b6-f complex iron-sulfur subunit (AHRD V1 ***- A9PEX3_POPTR)%3B contains Interpro domain(s) IPR005805 Rieske iron-sulphur protein%2C C-terminal
Solyc04g056580.2.1	4.76888005	2.11E-06	9.68E-05	L2/L3-specific	L,D	Homology to unknown gene (AHRD V1 ***- Q00YK5_OSTTA)
Solyc08g076890.2.1	4.761734574	1.76E-06	8.32E-05	L2/L3-specific	L,D,F	Unknown Protein (AHRD V1)
Solyc08g077530.2.1	4.753376114	8.14E-13	5.60E-11	L2/L3-specific	D	Beta-amylase (AHRD V1 **** Q5F305_SOYBN)%3B contains Interpro domain(s) IPR001371 Glycoside hydrolase%2C family 14B%2C plant
Solyc07g049450.2.1	4.752061176	2.16E-09	2.80E-07	L2/L3-specific	F	Thioredoxin/protein disulfide isomerase (AHRD V1 **-* D3TLX2_GLOMM)%3B contains Interpro domain(s) IPR005788 Disulphide isomerase
Solyc02g086820.2.1	4.749834191	4.87E-17	1.26E-14	L2/L3-specific	L	Carbonic anhydrase (AHRD V1 ***- Q5NE20_SOLLC)%3B contains Interpro domain(s) IPR015892 Carbonic anhydrase%2C prokaryotic-like%2C conserved site
Solyc09g007320.2.1	4.742357637	1.09E-05	0.000405949	L2/L3-specific	L,D	Pentatricopeptide repeat protein (AHRD V1 *-*- A7BJL0_RAPSA)%3B contains Interpro domain(s) IPR002885 Pentatricopeptide repeat
Solyc12g100160.1.1	4.736857456	1.17E-10	5.80E-09	L2/L3-specific	L,D	50S ribosomal protein L6 (AHRD V1 ***- B6SL57_MAIZE)%3B contains Interpro domain(s) IPR019906 Ribosomal protein L6%2C subgroup
Solyc06g066640.2.1	4.735364434	2.74E-09	2.35E-07	L2/L3-specific	L,D	Photosystem I reaction center subunit VI-1%2C chloroplastic (AHRD V1 ***- PSAH1_ARATH)%3B contains Interpro domain(s) IPR004928 Photosystem I reaction centre subunit VI
Solyc03g082960.2.1	4.733188301	4.31E-06	0.000182163	L2/L3-specific	L	Serine/threonine phosphatase family protein (AHRD V1 **** C1E0J1_9CHLO)%3B contains Interpro domain(s) IPR015655 Protein phosphatase 2C
Solyc07g043420.2.1	4.725882768	1.07E-14	1.01E-12	L2/L3-specific	L,D	2-oxoglutarate-dependent dioxygenase (AHRD V1 **-* Q9ZSH4_SOLCH)%3B contains Interpro domain(s) IPR005123 Oxoglutarate and iron-dependent oxygenase
Solyc04g072740.2.1	4.723994545	3.84E-06	0.000165463	L2/L3-specific	L	High affinity sulfate transporter 1 (AHRD V1 **** SUT1_STYHA)%3B contains Interpro domain(s) IPR001902 Sulphate anion transporter
Solyc09g063130.2.1	4.720168681	8.80E-14	7.24E-12	L2/L3-specific	L,D	Photosystem I reaction center subunit IV A (AHRD V1 *-*- B6TH55_MAIZE)%3B contains Interpro domain(s) IPR003375 Photosystem I reaction centre subunit IV/PsaE
Solyc12g032950.1.1	4.716822545	5.15E-06	0.000212244	L2/L3-specific	L	CBS domain-containing protein (AHRD V1 ***- D7LUC7_ARALY)%3B contains Interpro domain(s) IPR000644 Cystathionine beta-synthase%2C core

Solyc02g088350.2.1	4.706779597	3.05E-06	6.30E-05	L2/L3-specific	D	Genomic DNA chromosome 3 P1 clone MXO21 (AHRD V1 ***- Q9LS78_ARATH)
Solyc03g112200.1.1	4.701582148	6.83E-10	2.97E-08	L2/L3-specific	D	Unknown Protein (AHRD V1)
Solyc12g094380.1.1	4.686420779	5.76E-17	7.27E-15	L2/L3-specific	L,D	DSBA oxidoreductase (AHRD V1 ***- A4T4K6_MYCGI)%3B contains Interpro domain(s) IPR012336 Thioredoxin-like fold
Solyc08g076220.2.1	4.685934761	4.52E-20	1.83E-17	L2/L3-specific	L,F	Phosphoribulokinase/uridine kinase (AHRD V1 ***- B7KI62_CYAP7)%3B contains Interpro domain(s) IPR006083 Phosphoribulokinase/uridine kinase
Solyc08g081890.2.1	4.685522862	3.10E-06	0.000182384	L2/L3-specific	F	Multidrug resistance protein ABC transporter family (AHRD V1 **-- B9GX56_POPTR)%3B contains Interpro domain(s) IPR003439 ABC transporter-like
Solyc09g082690.2.1	4.685522194	6.37E-12	1.39E-09	L2/L3-specific	F	Early light-induced protein 7 (AHRD V1 ***- B2BJD6_RHOCT)
Solyc08g065220.2.1	4.675436467	2.66E-19	9.61E-17	L2/L3-specific	L,D	Glycine dehydrogenase P protein (AHRD V1 **** Q6V9T1_ORYSJ)%3B contains Interpro domain(s) IPR003437 Glycine cleavage system P-protein
Solyc07g047800.2.1	4.670092623	1.91E-13	5.13E-11	L2/L3-specific	F	Short-chain dehydrogenase/reductase family protein (AHRD V1 **-- D7L9J1_ARALY)%3B contains Interpro domain(s) IPR002347 Glucose/ribitol dehydrogenase
Solyc10g081910.1.1	4.665721714	9.98E-08	2.97E-06	L2/L3-specific	D	Receptor like kinase%2C RLK
Solyc01g106210.2.1	4.658436155	6.87E-11	1.21E-08	L2/L3-specific	F	Chaperone DnaK (AHRD V1 ***- A2Q199_MEDTR)%3B contains Interpro domain(s) IPR012725 Chaperone DnaK
Solyc12g094620.1.1	4.657906787	2.20E-24	6.24E-22	L2/L3-specific	D,F	Catalase (AHRD V1 ***- Q2PYW5_SOLTU)%3B contains Interpro domain(s) IPR018028 Catalase related subgroup
Solyc09g009190.2.1	4.651805018	2.00E-10	9.62E-09	L2/L3-specific	D	1 4-alpha-glucan branching enzyme II (AHRD V1 **** P93691_WHEAT)%3B contains Interpro domain(s) IPR006589 Glycosyl hydrolase%2C family 13%2C subfamily%2C catalytic region
Solyc07g053830.2.1	4.647229065	3.15E-08	2.11E-06	L2/L3-specific	L	Mitochondrial ADP/ATP carrier proteins (AHRD V1 **** Q2UU95_ASPOR)%3B contains Interpro domain(s) IPR002113 Adenine nucleotide translocator 1
Solyc05g007980.2.1	4.631015499	1.16E-05	0.000428824	L2/L3-specific	L,D	High affinity sulfate transporter 2 (AHRD V1 **** SUT2_STYHA)%3B contains Interpro domain(s) IPR001902 Sulphate anion transporter
Solyc04g005330.2.1	4.630209688	1.16E-05	0.000428331	L2/L3-specific	L	Periodic tryptophan protein 1 homolog (AHRD V1 **-- C1BVG9_9MAXI)%3B contains Interpro domain(s) IPR020472 G-protein beta WD-40 repeat%2C region
Solyc12g015710.1.1	4.629969452	1.28E-05	0.00021835	L2/L3-specific	D	Ribosomal RNA small subunit methyltransferase B (AHRD V1 ***- D5H7I5_SALRM)%3B contains Interpro domain(s) IPR001678 Bacterial Fmu (Sun)/eukaryotic nucleolar NOL1/Nop2p
Solyc04g082710.2.1	4.619425229	4.54E-06	8.90E-05	L2/L3-specific	D	Cathepsin B-like cysteine proteinase 3 (AHRD V1 **-- CPR3_CAEEL)%3B contains Interpro domain(s) IPR013128 Peptidase C1A%2C papain
Solyc10g008740.2.1	4.617363076	9.84E-08	2.94E-06	L2/L3-specific	L,D,F	Magnesium chelatase ATPase subunit I (AHRD V1 **** Q53RM0_ORYSJ)%3B contains Interpro domain(s) IPR011775 Magnesium chelatase%2C ATPase subunit I
Solyc09g007850.2.1	4.605325858	1.38E-20	2.81E-18	L2/L3-specific	L,D	RNA-binding protein (AHRD V1 **-* C6F119_SOYBN)%3B contains Interpro domain(s) IPR000504 RNA recognition motif%2C RNP-1
Solyc03g121660.2.1	4.600841051	2.17E-08	1.48E-06	L2/L3-specific	L,D,F	Zinc finger protein (AHRD V1 *-*- Q9LVQ7_ARATH)%3B contains Interpro domain(s) IPR007087 Zinc finger%2C C2H2-type
Solyc11g056680.1.1	4.600486557	1.75E-15	1.78E-13	L2/L3-specific	D	LRR receptor-like serine/threonine-protein kinase%2C RLP
Solyc01g009430.2.1	4.598835074	9.81E-09	3.52E-07	L2/L3-specific	D	Os02g0448600 protein (Fragment) (AHRD V1 *--- Q0E1G8_ORYSJ)
Solyc08g076880.2.1	4.594556859	1.21E-12	1.81E-10	L2/L3-specific	L,F	Unknown Protein (AHRD V1)
Solyc05g052600.2.1	4.594333944	6.73E-06	0.000267122	L2/L3-specific	L,D	Fructose-1 6-bisphosphatase class 1 (AHRD V1 **** B9M1N3_GEOSF)%3B contains Interpro domain(s) IPR000146 Fructose-1%2C6-bisphosphatase IPR000719 Protein kinase%2C core
Solyc07g066310.2.1	4.592494172	5.11E-22	1.18E-19	L2/L3-specific	L,D,F	photosystem II polypeptide (AHRD V1 ***- Q6V7X5_TRIPR)%3B contains Interpro domain(s) IPR006814 Photosystem II protein PsbR
Solyc05g014310.2.1	4.592290652	1.24E-05	0.000452098	L2/L3-specific	L	Metalloendopeptidase family-saccharolysin %26amp thimet oligopeptidase (ISS) (AHRD V1 *-*- Q01G48_OSTTA)
Solyc12g006460.1.1	4.580421061	4.64E-06	0.000194061	L2/L3-specific	L,D,F	Cytochrome P450
Solyc02g069460.2.1	4.574581102	6.45E-12	8.43E-10	L2/L3-specific	L,D	Photosystem I reaction center subunit III (AHRD V1 ***- Q9XQB4_PHAAU)%3B contains Interpro domain(s) IPR003666 Photosystem I reaction centre protein PsaF%2C subunit III
Solyc08g074480.1.1	4.573259581	9.89E-09	1.14E-06	L2/L3-specific	L,D,F	Cortical cell-delineating protein (AHRD V1 **-- B6UGA2_MAIZE)%3B contains Interpro domain(s) IPR013770 Plant lipid transfer protein and hydrophobic protein%2C helical
Solyc01g102330.2.1	4.572354089	7.91E-06	0.000145816	L2/L3-specific	D	Acetyl xylan esterase A (AHRD V1 **-- D0TLQ4_9BACE)%3B contains Interpro domain(s) IPR005181 Protein of unknown function DUF303%2C acetylesterase putative
Solyc02g083810.2.1	4.565530484	1.07E-18	1.77E-16	L2/L3-specific	L,D,F	Ferredoxin--NADP reductase (AHRD V1 ***- D7MN04_ARALY)%3B contains Interpro domain(s) IPR012146 Ferredoxin--NADP reductase
Solyc07g065900.2.1	4.562768712	4.03E-11	4.77E-09	L2/L3-specific	L	Fructose-bisphosphate aldolase (AHRD V1 ***- A9PEE1_POPTR)%3B contains Interpro domain(s) IPR000741 Fructose-bisphosphate aldolase%2C class-I
Solyc01g094910.2.1	4.562651615	3.32E-05	0.001049492	L2/L3-specific	L,D	Ferric reductase oxidase (AHRD V1 **-* D6RVS5_HORVU)%3B contains Interpro domain(s) IPR013121 Ferric reductase%2C NAD binding
Solyc05g052710.2.1	4.529376236	1.04E-05	0.000182185	L2/L3-specific	D	30S ribosomal protein S31%2C chloroplastic (AHRD V1 *-*- RR31_ARATH)
Solyc11g062100.1.1	4.526666949	1.45E-05	0.000241909	L2/L3-specific	L,D	Copper-transporting ATPase (AHRD V1 ***- C1H876_PARBA)%3B contains Interpro domain(s) IPR001757 ATPase%2C P-type%2C K/Mg/Cd/Cu/Zn/Na/Ca/Na/H-transporter
Solyc04g005910.2.1	4.512861149	1.03E-05	0.000180601	L2/L3-specific	D	Receptor like kinase%2C RLK
Solyc12g015970.1.1	4.512691928	1.25E-07	3.61E-06	L2/L3-specific	D	GPI-anchored protein (AHRD V1 ***- A2PZD8_IPONI)
Solyc01g098640.2.1	4.510216531	3.21E-12	4.50E-10	L2/L3-specific	L,D	UPF0603 protein At1g54780%2C chloroplastic (AHRD V1 **-- U603_ARATH)%3B contains Interpro domain(s) IPR007621 Protein of unknown function DUF477
Solyc01g007740.2.1	4.509272408	1.12E-05	0.000195041	L2/L3-specific	D	Peroxiredoxin (AHRD V1 **-- Q9FE12_PHAVU)%3B contains Interpro domain(s) IPR000866 Alkyl hydroperoxide reductase/ Thiol specific antioxidant/ Mal allergen
Solyc04g057980.2.1	4.501496729	1.42E-05	0.000509144	L2/L3-specific	L,D	NAD(P)H-quinone oxidoreductase subunit M (AHRD V1 *-*- D8G7H3_9CYAN)%3B contains Interpro domain(s) IPR018922 NAD(P)H-quinone oxidoreductase subunit M
Solyc04g014600.2.1	4.487758808	9.76E-06	0.000486887	L2/L3-specific	F	Universal stress protein family protein (AHRD V1 ***- D7L0F0_ARALY)%3B contains Interpro domain(s) IPR006016 UspA
Solyc12g010130.1.1	4.476463577	9.03E-08	5.59E-06	L2/L3-specific	L	CBL-interacting protein kinase 7 (AHRD V1 ***- A0MNJ4_POPTR)%3B contains Interpro domain(s) IPR002290 Serine/threonine protein kinase
Solyc04g054740.2.1	4.458992817	1.54E-05	0.000545281	L2/L3-specific	L	Inositol-3-phosphate synthase (AHRD V1 ***- C4PW06_ARATH)%3B contains Interpro domain(s) IPR002587 Myo-inositol-1-phosphate synthase
Solyc06g063370.2.1	4.455004669	1.50E-17	4.04E-15	L2/L3-specific	L,F	Chlorophyll a-b binding protein 1A%2C chloroplastic (AHRD V1 ***- CB2A_PYRPY)%3B contains Interpro domain(s) IPR001344 Chlorophyll A-B binding protein
Solyc04g081590.2.1	4.453400419	5.57E-09	2.10E-07	L2/L3-specific	D	Receptor like kinase%2C RLK
Solyc04g005610.2.1	4.452936993	1.17E-05	0.000202018	L2/L3-specific	D	NAC domain transcription factor (AHRD V1 **** Q5DM36_WHEAT)%3B contains Interpro domain(s) IPR003441 No apical meristem (NAM) protein
Solyc08g083110.2.1	4.44844599	1.36E-17	1.85E-15	L2/L3-specific	D,F	Cystathionine gamma-lyase (AHRD V1 ***- D2LF58_RHOVA)%3B contains Interpro domain(s) IPR000277 Cys/Met metabolism%2C pyridoxal phosphate-dependent enzyme
Solyc07g005830.2.1	4.44795085	1.46E-11	8.40E-10	L2/L3-specific	D	Translation initiation factor IF-3 (AHRD V1 ***- B9T4Q8_RICCO)%3B contains Interpro domain(s) IPR001288 Translation initiation factor 3
Solyc02g068100.2.1	4.445167329	1.20E-05	0.000585749	L2/L3-specific	F	SWI/SNF related matrix associated actin dependent regulator of chromatin subfamily d member 1 (AHRD V1 ***- Q802C8_DANRE)%3B contains Interpro domain(s) IPR019835 SWIB domain
Solyc03g113430.2.1	4.437300016	3.21E-05	0.001021602	L2/L3-specific	L,F	Peptide transporter (AHRD V1 **-* A6YJX4_9MAGN)%3B contains Interpro domain(s) IPR000109 TGF-beta receptor%2C type I/II extracellular region
Solyc07g007930.2.1	4.43055651	1.54E-19	2.74E-17	L2/L3-specific	D	Alkaline alpha galactosidase 2 (AHRD V1 **-- Q575Z7_MAIZE)%3B contains Interpro domain(s) IPR008811 Raffinose synthase
Solyc07g064180.2.1	4.42468224	8.13E-18	3.72E-15	L2/L3-specific	F	Pectinesterase (AHRD V1 ***- B9RXQ4_RICCO)%3B contains Interpro domain(s) IPR018040 Pectinesterase%2C active site IPR000070 Pectinesterase%2C catalytic
Solyc12g009070.1.1	4.412209035	2.19E-05	0.00073797	L2/L3-specific	L	Unknown Protein (AHRD V1)
Solyc04g008210.1.1	4.403696721	3.76E-08	1.21E-06	L2/L3-specific	L,D	Xyloglucan endotransglucosylase/hydrolase 13 (AHRD V1 **** C0IRH2_ACTDE)%3B contains Interpro domain(s) IPR016455 Xyloglucan endotransglucosylase/hydrolase
Solyc02g069290.2.1	4.402224947	5.95E-05	0.001755744	L2/L3-specific	L	Transducin-like (AHRD V1 **-- Q6H6D6_ORYSJ)%3B contains Interpro domain(s) IPR011989 Armadillo-like helical
Solyc01g106480.2.1	4.382646267	9.29E-08	5.70E-06	L2/L3-specific	L,D	Malate dehydrogenase (AHRD V1 ***- A5BEJ8_VITVI)%3B contains Interpro domain(s) IPR010097 Malate dehydrogenase%2C NAD-dependent%2C eukaryotes and gamma proteobacteria
Solyc06g071790.2.1	4.378913203	7.51E-14	1.24E-11	L2/L3-specific	L,D,F	Elongation factor Tu (AHRD V1 **** D7MFK2_ARALY)%3B contains Interpro domain(s) IPR004541 Translation elongation factor EFTu/EF1A%2C bacterial and organelle
Solyc05g010420.1.1	4.377925238	9.30E-08	5.70E-06	L2/L3-specific	L,D,F	S-adenosylmethionine decarboxylase proenzyme (AHRD V1 **** Q7XZQ9_VITVI)%3B contains Interpro domain(s) IPR018167 S-adenosylmethionine decarboxylase subgroup IPR001985 S-adenosylmethionine decarboxylase
Solyc05g025600.1.1	4.375006678	1.50E-13	1.19E-11	L2/L3-specific	L,D	Chloroplast photosystem II subunit X (Fragment) (AHRD V1 ***- B1PPX5_9MYRT)%3B contains Interpro domain(s) IPR009518 Photosystem II protein PsbX
Solyc01g108910.2.1	4.367905718	5.87E-17	7.35E-15	L2/L3-specific	D	COSII_At2g15890 (Fragment) (AHRD V1 *-*- C0KFD9_9SOLN)
Solyc01g087730.2.1	4.352761367	3.91E-14	3.36E-12	L2/L3-specific	L,D	50S ribosomal protein L1 (AHRD V1 ***- A8YGI8_MICAE)%3B contains Interpro domain(s) IPR005878 Ribosomal protein L1%2C bacterial-type
Solyc05g013440.2.1	4.348412385	3.62E-05	0.001135671	L2/L3-specific	L	Primary amine oxidase (AHRD V1 **** B4WL51_9SYNE)%3B contains Interpro domain(s) IPR000269 Copper amine oxidase
Solyc09g083190.2.1	4.338208454	4.18E-05	0.001289124	L2/L3-specific	L,D	Genomic DNA chromosome 5 P1 clone MQD19 (AHRD V1 ***- Q9FG89_ARATH)
Solyc03g077920.1.1	4.33413025	4.67E-05	0.000669698	L2/L3-specific	D	Phosphoribosylanthranilate transferase (Fragment) (AHRD V1 *--- O43085_PEA)%3B contains Interpro domain(s) IPR013583 Phosphoribosyltransferase C-terminal%2C plant
Solyc06g006080.2.1	4.325661914	4.50E-13	3.24E-11	L2/L3-specific	D	Phosphomethylpyrimidine synthase (AHRD V1 ***- C7MPZ7_SACVD)%3B contains Interpro domain(s) IPR002817 Thiamine biosynthesis protein ThiC
Solyc12g056860.1.1	4.3198175	2.88E-05	0.000442392	L2/L3-specific	D	BZIP transcription factor (AHRD V1 **** A7Y1S1_BRAJU)%3B contains Interpro domain(s) IPR011616 bZIP transcription factor%2C bZIP-1
Solyc11g007200.1.1	4.310069435	1.33E-07	3.79E-06	L2/L3-specific	L,D	Copper chaperone (AHRD V1 *-*- D3GC04_9ROSI)%3B contains Interpro domain(s) IPR006121 Heavy metal transport/detoxification protein
Solyc09g008780.2.1	4.303799411	4.70E-10	4.58E-08	L2/L3-specific	L,D	Os01g0786800 protein (Fragment) (AHRD V1 *-*- Q0JIP0_ORYSJ)%3B contains Interpro domain(s) IPR002781 Protein of unknown function DUF81
Solyc10g086220.1.1	4.299487552	3.56E-05	0.000529341	L2/L3-specific	D	Flavin oxidoreductase/NADH oxidase (AHRD V1 **-- A0YMI2_LYNSP)%3B contains Interpro domain(s) IPR001155 NADH:flavin oxidoreductase/NADH oxidase%2C N-terminal
Solyc12g099930.1.1	4.299469707	2.10E-16	5.06E-14	L2/L3-specific	L,F	Serine-glyoxylate aminotransferase (AHRD V1 ***- Q3S2I1_SPIPO)%3B contains Interpro domain(s) IPR000192 Aminotransferase%2C class V/Cysteine desulfurase
Solyc03g005770.1.1	4.297418514	3.15E-19	1.11E-16	L2/L3-specific	L,F	Chlorophyll a-b binding protein 3C-like (AHRD V1 **** Q2XTE0_SOLTU)%3B contains Interpro domain(s) IPR001344 Chlorophyll A-B binding protein
Solyc09g072820.2.1	4.286663512	4.38E-05	0.000632092	L2/L3-specific	D	Cellulose synthase (AHRD V1 **** B8XPP6_9ROSI)%3B contains Interpro domain(s) IPR005150 Cellulose synthase
Solyc03g062790.2.1	4.280324307	3.47E-05	0.000520088	L2/L3-specific	D	cDNA clone 002-130-C06 full insert sequence (AHRD V1 **-- B7F0E5_ORYSJ)
Solyc11g072710.1.1	4.267472692	3.76E-13	2.77E-11	L2/L3-specific	D	Protein kinase (AHRD V1 *-*- Q7DMT0_PEA)%3B contains Interpro domain(s) IPR002290 Serine/threonine protein kinase
Solyc01g087260.2.1	4.249749484	9.81E-09	7.57E-07	L2/L3-specific	L,D	Carotenoid cleavage dioxygenase 1B
Solyc03g097110.2.1	4.236647848	3.53E-05	0.001441985	L2/L3-specific	F	RNA recognition motif-containing protein (AHRD V1 **-- D7M9G3_ARALY)%3B contains Interpro domain(s) IPR001395 Aldo/keto reductase
Solyc04g054190.2.1	4.234700493	2.08E-10	2.21E-08	L2/L3-specific	L	ABC-1 domain protein (AHRD V1 **-- D7E596_NOSA0)%3B contains Interpro domain(s) IPR004147 ABC-1
Solyc06g008300.2.1	4.234464529	3.90E-08	2.59E-06	L2/L3-specific	L	LRR receptor-like serine/threonine-protein kinase%2C RLP
Solyc01g005240.2.1	4.233845792	4.91E-05	0.001899323	L2/L3-specific	F	Aspartokinase (AHRD V1 ***- B9RGY9_RICCO)%3B contains Interpro domain(s) IPR001341 Aspartate kinase region
Solyc07g007660.1.1	4.233238894	0.0001159	0.003065034	L2/L3-specific	L	Unknown Protein (AHRD V1)
Solyc04g071140.2.1	4.231449567	0.0001109	0.00295313	L2/L3-specific	L,D	Decarboxylase family protein (AHRD V1 ***- B1ILJ6_CLOBK)%3B contains Interpro domain(s) IPR002129 Pyridoxal phosphate-dependent decarboxylase
Solyc08g006540.2.1	4.222918709	0.0001027	0.002804238	L2/L3-specific	L	Peptidyl-prolyl cis-trans isomerase (AHRD V1 *-** B1XHQ5_SYNP2)%3B contains Interpro domain(s) IPR001179 Peptidyl-prolyl cis-trans isomerase%2C FKBP-type
Solyc03g112220.1.1	4.22244205	3.71E-05	0.000550035	L2/L3-specific	D	Unknown Protein (AHRD V1)
Solyc07g063600.2.1	4.216952609	9.43E-09	7.32E-07	L2/L3-specific	L	Chlorophyll a-b binding protein 13%2C chloroplastic (AHRD V1 ***- CB23_SOLLC)%3B contains Interpro domain(s) IPR001344 Chlorophyll A-B binding protein
Solyc06g073550.2.1	4.209681415	4.59E-05	0.001797432	L2/L3-specific	F	La related protein-like (AHRD V1 **-- Q5NBG8_ORYSJ)%3B contains Interpro domain(s) IPR006630 RNA-binding protein Lupus La
Solyc03g095620.2.1	4.208195143	6.47E-08	4.10E-06	L2/L3-specific	L,D	ABC-1 domain protein (AHRD V1 **-- A1TWJ2_MARAV)%3B contains Interpro domain(s) IPR004147 ABC-1
Solyc08g068590.2.1	4.206722849	0.000111	0.00295313	L2/L3-specific	L,D	PAP fibrillin family protein (AHRD V1 ***- B5W0K3_SPIMA)%3B contains Interpro domain(s) IPR006843 PAP fibrillin
Solyc03g093800.1.1	4.204384632	8.39E-09	3.08E-07	L2/L3-specific	D	Unknown Protein (AHRD V1)
Solyc03g019820.2.1	4.186092075	3.88E-05	0.00056893	L2/L3-specific	D	Aquaporin (AHRD V1 ***- D6BRE1_9ROSI)%3B contains Interpro domain(s) IPR012269 Aquaporin
Solyc03g120430.2.1	4.185499319	7.22E-07	3.74E-05	L2/L3-specific	L,D	Glycerate kinase (AHRD V1 **** C1DYY5_9CHLO)
Solyc01g109110.2.1	4.177795307	1.01E-07	6.08E-06	L2/L3-specific	L	Guanine nucleotide-binding protein alpha-1 subunit (AHRD V1 *-*- B6TWS6_MAIZE)%3B contains Interpro domain(s) IPR001019 Guanine nucleotide binding protein (G-protein)%2C alpha subunit
Solyc03g115900.2.1	4.174057046	4.76E-18	1.42E-15	L2/L3-specific	L,F	Chlorophyll a-b binding protein P4%2C chloroplastic (AHRD V1 ***- CB24_PEA)%3B contains Interpro domain(s) IPR001344 Chlorophyll A-B binding protein
Solyc04g080700.2.1	4.170038718	3.02E-12	1.87E-10	L2/L3-specific	D	Wound responsive protein (Fragment) (AHRD V1 **-- A6N0L4_ORYSI)%3B contains Interpro domain(s) IPR003729 Protein of unknown function DUF151

Solyc06g072350.2.1	4.159337843	6.37E-05	0.000877471	L2/L3-specific	D	UPF0497 membrane protein 17 (AHRD V1 ***- U497H_RICCO)%3B contains Interpro domain(s) IPR006702 Uncharacterised protein family UPF0497%2C trans-membrane plant
Solyc09g007270.2.1	4.159132593	1.28E-10	2.00E-08	L2/L3-specific	F	Ascorbate peroxidase (AHRD V1 **** B9VRH6_CITMA)%3B contains Interpro domain(s) IPR002207 Plant ascorbate peroxidase
Solyc07g005440.1.1	4.146424019	0.000122	0.003191492	L2/L3-specific	L,F	CBL-interacting protein kinase 9 (AHRD V1 **** A0MNJ6_POPTR)%3B contains Interpro domain(s) IPR002290 Serine/threonine protein kinase
Solyc06g036330.1.1	4.141882137	0.0002166	0.005216852	L2/L3-specific	L	Ornithine cyclodeaminase protein (AHRD V1 ***- A3I2L0_9BACT)%3B contains Interpro domain(s) IPR003462 Ornithine cyclodeaminase/mu-crystallin
Solyc10g074910.1.1	4.141273252	2.09E-06	9.60E-05	L2/L3-specific	L	Acetyltransferase-like protein (AHRD V1 *-*- Q31BX2_PROM9)%3B contains Interpro domain(s) IPR000182 GCN5-related N-acetyltransferase
Solyc09g009820.2.1	4.140750842	3.41E-10	3.47E-08	L2/L3-specific	L,D	Glutathione S-transferase domain protein (AHRD V1 *--- C6RLV9_ACIRA)%3B contains Interpro domain(s) IPR004045 Glutathione S-transferase%2C N-terminal
Solyc06g068220.2.1	4.136118989	6.69E-06	0.000125736	L2/L3-specific	L,D	Hydrolase alpha/beta fold family protein (AHRD V1 ***- D7KJH6_ARALY)%3B contains Interpro domain(s) IPR000639 Epoxide hydrolase-like
Solyc11g006060.1.1	4.127214044	8.56E-07	4.31E-05	L2/L3-specific	L,D	Homology to unknown gene (AHRD V1 ***- Q00S02_OSTTA)
Solyc11g013810.1.1	4.126211025	1.86E-09	1.65E-07	L2/L3-specific	L	Nitrate reductase (AHRD V1 ***- Q8LT13_SOLTU)%3B contains Interpro domain(s) IPR012137 Nitrate reductase NADH dependant
Solyc11g006680.1.1	4.124013174	3.17E-06	6.48E-05	L2/L3-specific	D	Pentatricopeptide repeat-containing protein (AHRD V1 ***- D7LY59_ARALY)%3B contains Interpro domain(s) IPR000504 RNA recognition motif%2C RNP-1
Solyc03g071690.2.1	4.116211306	7.95E-05	0.001062953	L2/L3-specific	D	Non-symbiotic hemoglobin 2 (AHRD V1 **-- D7L8Z8_ARALY)%3B contains Interpro domain(s) IPR001032 Leghaemoglobin
Solyc02g084440.2.1	4.114870409	4.58E-18	1.40E-15	L2/L3-specific	L,D,F	Fructose-bisphosphate aldolase (AHRD V1 ***- Q9SXX4_NICPA)%3B contains Interpro domain(s) IPR000741 Fructose-bisphosphate aldolase%2C class-I
Solyc01g107910.2.1	4.11421245	0.0002396	0.005682154	L2/L3-specific	L	Caffeoyl CoA 3-O-methyltransferase (AHRD V1 **** Q5I2D1_9ROSI)%3B contains Interpro domain(s) IPR002935 O-methyltransferase%2C family 3
Solyc03g111320.1.1	4.112180422	3.12E-06	0.000136495	L2/L3-specific	L	Exocyst complex protein EXO70 (AHRD V1 *--- EXO70_KLULA)%3B contains Interpro domain(s) IPR004140 Exo70 exocyst complex subunit
Solyc01g103030.2.1	4.108793449	6.25E-05	0.000862839	L2/L3-specific	D	Nitrate transporter (AHRD V1 **** Q7XAK5_PRUPE)%3B contains Interpro domain(s) IPR000109 TGF-beta receptor%2C type I/II extracellular region
Solyc07g056020.2.1	4.100147231	1.79E-11	2.18E-09	L2/L3-specific	L,D	Translation initiation factor IF-2 (AHRD V1 **** A5B2X2_VITVI)%3B contains Interpro domain(s) IPR000178 Initiation factor 2
Solyc03g116730.2.1	4.095442646	0.000146	0.003746438	L2/L3-specific	L	Stearoyl-CoA 9-desaturase (AHRD V1 **** D7E3I2_NOSA0)%3B contains Interpro domain(s) IPR015876 Fatty acid desaturase%2C type 1%2C core
Solyc02g081810.2.1	4.09543261	8.01E-05	0.002824386	L2/L3-specific	F	tRNA pseudouridine synthase B (AHRD V1 *-*- TRUB_PELTS)%3B contains Interpro domain(s) IPR004802 Pseudouridine synthase%2C putative
Solyc07g052290.1.1	4.093279537	1.99E-11	1.11E-09	L2/L3-specific	L,D	Membrane-associated zinc metalloprotease family protein expressed (AHRD V1 ***- Q84NY6_ORYSJ)%3B contains Interpro domain(s) IPR004387 Peptidase M50%2C putative membrane-associated zinc metallopeptidase
Solyc03g083570.2.1	4.09315221	2.21E-06	4.78E-05	L2/L3-specific	D	Unknown Protein (AHRD V1)
Solyc01g108340.2.1	4.090181819	7.49E-05	0.002670546	L2/L3-specific	F	Peptidyl-prolyl cis-trans isomerase D (AHRD V1 **** PPID_RAT)%3B contains Interpro domain(s) IPR002130 Peptidyl-prolyl cis-trans isomerase%2C cyclophilin-type
Solyc01g112060.2.1	4.079091006	0.0001986	0.004843099	L2/L3-specific	L	NmrA family protein (AHRD V1 **-- B2J8Q2_NOSP7)%3B contains Interpro domain(s) IPR016040 NAD(P)-binding domain
Solyc12g094630.1.1	4.076034691	2.34E-06	0.000105549	L2/L3-specific	L,D	Unknown Protein (AHRD V1)
Solyc01g079880.2.1	4.074840332	8.35E-07	2.00E-05	L2/L3-specific	L,D	Asparagine synthetase (AHRD V1 *--- Q9ZST6_ARATH)
Solyc02g005200.2.1	4.067605503	0.0003056	0.006981958	L2/L3-specific	L,F	PsaB translation factor (Fragment) (AHRD V1 ***- A4S2E4_OSTLU)%3B contains Interpro domain(s) IPR009472 Protein of unknown function DUF1092
Solyc03g117950.2.1	4.067403235	2.03E-06	9.40E-05	L2/L3-specific	L	Chaperone protein ClpB 1 (AHRD V1 **-- D0CK17_9SYNE)%3B contains Interpro domain(s) IPR013093 ATPase associated with various cellular activities%2C AAA-2
Solyc07g047850.2.1	4.060089523	2.28E-17	6.02E-15	L2/L3-specific	L,F	Chlorophyll a-b binding protein 4%2C chloroplastic (AHRD V1 **** CB24_SOLLC)%3B contains Interpro domain(s) IPR001344 Chlorophyll A-B binding protein
Solyc02g031740.2.1	4.052202919	0.0001081	0.001384808	L2/L3-specific	D	phloem lectin (AHRD V1 ***- Q8L5A9_CUCSA)
Solyc02g078400.2.1	4.051770628	0.0001706	0.004251402	L2/L3-specific	L	Allantoinase (AHRD V1 **** Q6S4R9_ROBPS)%3B contains Interpro domain(s) IPR017593 Allantoinase
Solyc02g090030.2.1	4.051022751	5.92E-15	5.75E-13	L2/L3-specific	L,D	Oxygen-evolving enhancer protein 1 of photosystem II (AHRD V1 ***- A8J0E4_CHLRE)%3B contains Interpro domain(s) IPR002628 Photosystem II manganese-stabilizing protein PsbO
Solyc10g009570.2.1	4.048169567	0.0001462	0.003746461	L2/L3-specific	L	Rhodanese-related sulfurtransferase (AHRD V1 *--- A4U0Q7_9PROT)%3B contains Interpro domain(s) IPR001763 Rhodanese-like
Solyc06g048410.2.1	4.045412038	0.0001072	0.003569741	L2/L3-specific	L,D,F	Superoxide dismutase (AHRD V1 **** Q7YK44_SOLLC)%3B contains Interpro domain(s) IPR019833 Manganese/iron superoxide dismutase%2C binding site IPR001189 Manganese/iron superoxide dismutase
Solyc11g066440.1.1	4.042370956	0.0002813	0.006492067	L2/L3-specific	L,D,F	Pheophorbide a oxygenase (Fragment) (AHRD V1 *--- D1MWF5_BRAOL)%3B contains Interpro domain(s) IPR013626 Pheophorbide a oxygenase
Solyc06g069090.2.1	4.038735858	0.0001087	0.003603592	L2/L3-specific	F	40S ribosomal protein S7-like protein (AHRD V1 ***- Q3HRX6_SOLTU)%3B contains Interpro domain(s) IPR000554 Ribosomal protein S7e
Solyc08g081480.2.1	4.028851883	3.89E-06	7.83E-05	L2/L3-specific	D	Polygalacturonase-like protein (AHRD V1 **-- Q84LI7_FRAAN)%3B contains Interpro domain(s) IPR012334 Pectin lyase fold
Solyc08g067100.2.1	4.026789993	4.60E-17	6.04E-15	L2/L3-specific	L,D	Aspartic proteinase nepenthesin-1 (AHRD V1 **-- C0JA51_9ORYZ)%3B contains Interpro domain(s) IPR001461 Peptidase A1
Solyc02g087340.2.1	4.02009711	1.63E-09	1.48E-07	L2/L3-specific	L	Seed maturation protein PM23 (Fragment) (AHRD V1 ***- Q9SEL1_SOYBN)
Solyc11g012360.1.1	4.003057598	2.87E-10	3.00E-08	L2/L3-specific	L,D,F	Sodium-dependent dicarboxylate transporter (AHRD V1 ***- O35055_RAT)%3B contains Interpro domain(s) IPR001898 Sodium/sulphate symporter
Solyc10g083360.1.1	4.001529908	4.65E-06	0.000194061	L2/L3-specific	L,D	Calmodulin binding protein (AHRD V1 ***- B6SL12_MAIZE)
Solyc01g111750.2.1	4.000491219	4.80E-11	2.54E-09	L2/L3-specific	D,F	Heat shock protein DnaJ domain protein (AHRD V1 *--- B7K9K5_CYAP7)
Solyc11g020040.1.1	3.998295338	6.80E-08	6.67E-06	L2/L3-specific	F	Chaperone DnaK (AHRD V1 ***- Q1SKX2_MEDTR)%3B contains Interpro domain(s) IPR012725 Chaperone DnaK
Solyc08g075860.2.1	3.992802784	4.83E-09	5.92E-07	L2/L3-specific	F	Os06g0115800 protein (Fragment) (AHRD V1 *-*- Q0DF43_ORYSJ)
Solyc01g109300.2.1	3.992753239	6.75E-09	5.39E-07	L2/L3-specific	L	4-hydroxy-3-methylbut-2-enyl diphosphate reductase (AHRD V1 ***- A2TGW4_9LAMI)%3B contains Interpro domain(s) IPR003451 LytB protein
Solyc12g042770.1.1	3.986494886	7.07E-13	4.91E-11	L2/L3-specific	D	Chloroplast post-illumination chlorophyll fluorescence increase protein (AHRD V1 **-- A1Y9I8_TOBAC)
Solyc09g072990.2.1	3.97692451	1.46E-08	1.59E-06	L2/L3-specific	L,F	Unknown Protein (AHRD V1)
Solyc07g008540.2.1	3.969363437	4.01E-06	8.04E-05	L2/L3-specific	D	CONSTANS-like zinc finger protein (AHRD V1 *-*- D0EP06_SOYBN)%3B contains Interpro domain(s) IPR010402 CCT domain
Solyc01g010640.2.1	3.965525909	0.0003183	0.007224776	L2/L3-specific	L	Uncharacterized membrane protein (AHRD V1 **-- Q46JN3_PROMT)%3B contains Interpro domain(s) IPR001395 Aldo/keto reductase
Solyc02g086710.2.1	3.955121357	0.0005414	0.011281581	L2/L3-specific	L	Monodehydroascorbate reductase (NADH)-like protein (AHRD V1 **** Q0WUJ1_ARATH)%3B contains Interpro domain(s) IPR013027 FAD-dependent pyridine nucleotide-disulphide oxidoreductase
Solyc05g055430.2.1	3.951075121	7.46E-06	0.000292932	L2/L3-specific	L	(RAP Annotation release2) Galactose-binding like domain containing protein (AHRD V1 **-- Q10RT4_ORYSJ)%3B contains Interpro domain(s) IPR018971 Protein of unknown function DUF1997
Solyc01g107660.2.1	3.950259884	0.0003007	0.006882191	L2/L3-specific	L,D	Stress enhanced protein 1 (Fragment) (AHRD V1 *-*- C6JT32_9MYRT)
Solyc01g095470.2.1	3.949600782	5.87E-09	2.21E-07	L2/L3-specific	D,F	Aldose 1-epimerase family protein (AHRD V1 ***- D7LSC1_ARALY)%3B contains Interpro domain(s) IPR008183 Aldose 1-epimerase
Solyc02g062290.2.1	3.94594726	0.0002217	0.00632993	L2/L3-specific	F	Polyadenylate-binding protein (AHRD V1 *--- C1GL98_PARBD)%3B contains Interpro domain(s) IPR000504 RNA recognition motif%2C RNP-1
Solyc06g065990.1.1	3.942723752	1.73E-08	1.22E-06	L2/L3-specific	L	ATP synthase subunit b%26apos (AHRD V1 ***- D8G4T7_9CYAN)%3B contains Interpro domain(s) IPR002146 ATPase%2C F0 complex%2C subunit B/B'%2C bacterial and chloroplast
Solyc02g068090.2.1	3.937279885	3.07E-10	1.42E-08	L2/L3-specific	D	30S ribosomal protein S21%2C chloroplastic (Fragment) (AHRD V1 *--- RR21_SPIOL)%3B contains Interpro domain(s) IPR001911 Ribosomal protein S21
Solyc08g077980.2.1	3.933417951	2.46E-06	0.000150876	L2/L3-specific	L,F	Bax inhibitor (AHRD V1 ***- Q6U1L7_SOLLC)%3B contains Interpro domain(s) IPR006214 Uncharacterised protein family UPF0005
Solyc04g015450.2.1	3.932376725	2.81E-06	5.90E-05	L2/L3-specific	L,D	GTP-binding protein lepA (AHRD V1 **** A8YK29_MICAE)%3B contains Interpro domain(s) IPR006297 GTP-binding protein LepA
Solyc01g005560.2.1	3.925777928	2.38E-06	0.000146111	L2/L3-specific	F	Isocitrate dehydrogenase (AHRD V1 ***- D2D324_GOSHI)%3B contains Interpro domain(s) IPR004790 Isocitrate dehydrogenase NADP-dependent%2C eukaryotic
Solyc12g036170.1.1	3.925717327	4.12E-06	0.000175163	L2/L3-specific	L	Unknown Protein (AHRD V1)
Solyc01g057830.2.1	3.92413495	2.30E-08	7.80E-07	L2/L3-specific	L,D	30S ribosomal protein S1 (AHRD V1 **-- B4FUZ5_MAIZE)%3B contains Interpro domain(s) IPR003029 Ribosomal protein S1%2C RNA binding domain
Solyc06g034290.2.1	3.924053288	2.66E-08	8.89E-07	L2/L3-specific	D	Glycerol-3-phosphate transporter (AHRD V1 **** A0PYC4_CLONN)%3B contains Interpro domain(s) IPR016196 Major facilitator superfamily%2C general substrate transporter
Solyc07g061940.2.1	3.912509945	0.0001878	0.005642039	L2/L3-specific	F	Acetolactate synthase (AHRD V1 **** D7RPJ7_SOLTU)%3B contains Interpro domain(s) IPR012001 Thiamine pyrophosphate enzyme%2C N-terminal TPP binding region
Solyc01g109040.2.1	3.91232321	4.58E-06	0.000192785	L2/L3-specific	L	Plastoquinol-plastocyanin reductase (AHRD V1 **-* B6SRD7_MAIZE)%3B contains Interpro domain(s) IPR012595 PetM of cytochrome b6/f complex subunit 7
Solyc02g068080.2.1	3.910719059	0.0001893	0.002218072	L2/L3-specific	D	Voltage-gated chloride channel (AHRD V1 **** Q96325_ARATH)%3B contains Interpro domain(s) IPR002251 Chloride channel plant CLC
Solyc03g078000.2.1	3.910498946	8.42E-11	9.80E-09	L2/L3-specific	L,D	High-affinity fructose transporter ght6 (AHRD V1 **** GHT6_SCHPO)%3B contains Interpro domain(s) IPR003663 Sugar/inositol transporter
Solyc09g075010.2.1	3.907175038	0.000187	0.005642039	L2/L3-specific	F	Prostaglandin E synthase 3 (AHRD V1 *--- B4FLE3_MAIZE)%3B contains Interpro domain(s) IPR017447 CS
Solyc03g083480.2.1	3.905993279	9.91E-07	4.93E-05	L2/L3-specific	L	Receptor-like kinase (AHRD V1 *-*- Q9LL53_ORYSA)
Solyc06g083040.2.1	3.90476046	6.14E-06	0.000116497	L2/L3-specific	D	Serine carboxypeptidase 1 (AHRD V1 ***- B6TDA5_MAIZE)%3B contains Interpro domain(s) IPR001563 Peptidase S10%2C serine carboxypeptidase
Solyc07g045310.2.1	3.900756421	7.01E-08	2.16E-06	L2/L3-specific	L,D	SET domain-containing protein (AHRD V1 ***- D7L6N3_ARALY)%3B contains Interpro domain(s) IPR011192 Rubisco methyltransferase
Solyc03g094080.2.1	3.899525791	0.0002001	0.00590435	L2/L3-specific	F	Polyadenylate-binding protein family protein (AHRD V1 **** D7MT99_ARALY)%3B contains Interpro domain(s) IPR000504 RNA recognition motif%2C RNP-1
Solyc08g074560.2.1	3.895264958	0.0005945	0.012182915	L2/L3-specific	L,D	Uncharacterized aarF domain-containing protein kinase 1 (AHRD V1 *--- ADCK1_CHICK)%3B contains Interpro domain(s) IPR004147 ABC-1
Solyc09g059030.2.1	3.891136084	1.59E-07	1.36E-05	L2/L3-specific	D,F	Alcohol dehydrogenase zinc-containing (AHRD V1 **-* Q637W0_BACCZ)%3B contains Interpro domain(s) IPR002085 Alcohol dehydrogenase superfamily%2C zinc-containing
Solyc03g062720.2.1	3.887788077	0.0004824	0.010289094	L2/L3-specific	L	Galactose mutarotase-like (AHRD V1 **-- A2Q5N9_MEDTR)%3B contains Interpro domain(s) IPR011013 Glycoside hydrolase-type carbohydrate-binding
Solyc10g051110.1.1	3.882250971	1.02E-06	2.40E-05	L2/L3-specific	L,D	NAD dependent epimerase/dehydratase family protein (AHRD V1 *--- B0CAN3_ACAM1)%3B contains Interpro domain(s) IPR016040 NAD(P)-binding domain
Solyc12g056830.1.1	3.881178902	4.37E-15	8.82E-13	L2/L3-specific	L,D,F	ATP synthase delta subunit (AHRD V1 ***- Q7XYM8_BIGNA)%3B contains Interpro domain(s) IPR000711 ATPase%2C F1 complex%2C OSCP/delta subunit
Solyc03g119150.2.1	3.870948772	2.60E-09	2.26E-07	L2/L3-specific	L,D	Peptidyl-prolyl cis-trans isomerase (AHRD V1 *-*- B4VX45_9CYAN)%3B contains Interpro domain(s) IPR001179 Peptidyl-prolyl cis-trans isomerase%2C FKBP-type
Solyc08g081620.2.1	3.868901843	0.0004924	0.004944424	L2/L3-specific	D,F	Endoglucanase 1 (AHRD V1 ***- B6U0P7_MAIZE)%3B contains Interpro domain(s) IPR008928 Six-hairpin glycosidase-like IPR012341 Six-hairpin glycosidase IPR018221 Glycoside hydrolase%2C family 9%2C active site IPR001701 Glycoside hydrolase%2C family 9
Solyc08g083010.2.1	3.860822055	5.60E-10	2.47E-08	L2/L3-specific	D	Homology to unknown gene (Fragment) (AHRD V1 *-*- Q00TF3_OSTTA)
Solyc10g077040.1.1	3.855041234	3.35E-15	6.96E-13	L2/L3-specific	L,F	Magnesium-protoporphyrin IX monomethyl ester (AHRD V1 **** B9SCF8_RICCO)%3B contains Interpro domain(s) IPR008434 Magnesium-protoporphyrin IX monomethyl ester aerobic oxidative cyclase
Solyc09g092110.2.1	3.852159217	4.68E-08	3.03E-06	L2/L3-specific	L,D	Light regulated protein-like protein (AHRD V1 ***- A8IXK8_BRACM)%3B contains Interpro domain(s) IPR009856 Light regulated Lir1
Solyc10g078930.1.1	3.848412564	3.89E-06	0.000217955	L2/L3-specific	F	Activator of heat shock protein ATPase homolog 1 (AHRD V1 **-* D3PJW3_9MAXI)%3B contains Interpro domain(s) IPR015310 Activator of Hsp90 ATPase%2C N-terminal
Solyc07g019670.2.1	3.848321639	1.51E-13	4.24E-11	L2/L3-specific	F	Fatty acid oxidation complex subunit alpha (AHRD V1 ***- FADJ_ECOHS)%3B contains Interpro domain(s) IPR006176 3-hydroxyacyl-CoA dehydrogenase%2C NAD binding
Solyc01g010520.2.1	3.848141299	0.0003008	0.003273731	L2/L3-specific	D	Uncharacterized plant-specific domain TIGR01589 family protein (AHRD V1 ***- Q60CZ4_SOLDE)%3B contains Interpro domain(s) IPR006476 Conserved hypothetical protein CHP01589%2C plant
Solyc03g063560.2.1	3.847183691	9.49E-14	7.71E-12	L2/L3-specific	L,D	Glutamate synthase (Ferredoxin) (AHRD V1 ***- B8HTZ7_CYAP4)%3B contains Interpro domain(s) IPR002932 Glutamate synthase%2C central-C
Solyc03g031700.2.1	3.844671168	1.24E-05	0.000452098	L2/L3-specific	L,D	ABC transporter FeS assembly protein SufB (AHRD V1 ***- C7JBP7_ACEP3)%3B contains Interpro domain(s) IPR010231 SUF system FeS cluster assembly%2C SufB
Solyc01g096880.2.1	3.842594499	0.0006707	0.01332438	L2/L3-specific	L	Nitrate transporter (AHRD V1 **** Q9SZY4_ARATH)%3B contains Interpro domain(s) IPR000109 TGF-beta receptor%2C type I/II extracellular region
Solyc12g009440.1.1	3.834789244	4.36E-07	1.12E-05	L2/L3-specific	D	Bundle-sheath defective protein 2 family (AHRD V1 ***- D7LRE2_ARALY)%3B contains Interpro domain(s) IPR001305 Heat shock protein DnaJ%2C cysteine-rich region
Solyc02g014860.2.1	3.834454201	3.88E-15	3.85E-13	L2/L3-specific	D	Chaperone protein DnaJ (AHRD V1 *-*- D2QAE1_BIFDB)%3B contains Interpro domain(s) IPR003095 Heat shock protein DnaJ
Solyc10g055390.1.1	3.829205578	1.37E-05	0.000230673	L2/L3-specific	D	Nodulin family protein (AHRD V1 ***- D7MDH3_ARALY)%3B contains Interpro domain(s) IPR010658 Nodulin-like
Solyc08g076900.2.1	3.825140055	7.93E-09	6.19E-07	L2/L3-specific	L,D,F	Unknown Protein (AHRD V1)
Solyc02g077080.2.1	3.824447195	8.70E-14	7.20E-12	L2/L3-specific	D	Os06g0207500 protein (Fragment) (AHRD V1 **-- Q0DDQ9_ORYSJ)%3B contains Interpro domain(s) IPR004253 Protein of unknown function DUF231%2C plant
Solyc02g086890.2.1	3.824431519	0.0007652	0.01467551	L2/L3-specific	L	Calcium homeostasis regulator chor1 (Fragment) (AHRD V1 *-*- A6N1L7_ORYSI)
Solyc07g064330.2.1	3.81264813	6.05E-08	1.89E-06	L2/L3-specific	D	DNA polymerase (AHRD V1 ***- Q3LHT0_TOBAC)%3B contains Interpro domain(s) IPR001098 DNA-directed DNA polymerase%2C family A
Solyc01g098870.2.1	3.799651037	6.71E-06	0.000353921	L2/L3-specific	F	Nucleolar GTPase (AHRD V1 *-*- A6YTC8_CUCME)

Solyc12g009250.1.1	3.798100189	1.51E-05	0.000534567	L2/L3-specific	L,D	chaperonin (AHRD V1 *-*- D1U6H2_9DELT)%3B contains Interpro domain(s) IPR001476 Chaperonin Cpn10
Solyc06g073290.1.1	3.796712166	7.02E-08	4.42E-06	L2/L3-specific	L,F	Gun4-like protein (AHRD V1 *-*- D8RVB0_SELML)%3B contains Interpro domain(s) IPR008629 GUN4-like
Solyc01g105460.2.1	3.792521917	9.87E-06	0.000175276	L2/L3-specific	D	1 4-dihydroxy-2-naphthoate octaprenyltransferase (AHRD V1 **** B6UA12_MAIZE)%3B contains Interpro domain(s) IPR011937 1%2C4-dihydroxy-2-naphthoate phytyltransferase
Solyc10g052490.1.1	3.788076097	0.000381	0.003990299	L2/L3-specific	D	Isoflavone reductase-like protein 5 (AHRD V1 ***- Q3KN68_VITVI)%3B contains Interpro domain(s) IPR008030 NmrA-like
Solyc11g045180.1.1	3.777276786	0.0005818	0.00569412	L2/L3-specific	D	ATP-dependent RNA helicase (AHRD V1 *-*- D3BPQ9_POLPA)%3B contains Interpro domain(s) IPR001650 DNA/RNA helicase%2C C-terminal
Solyc06g082950.2.1	3.773944258	3.99E-13	6.30E-11	L2/L3-specific	L,D	Photosystem I reaction center subunit XI (AHRD V1 ***- B6SHL1_MAIZE)%3B contains Interpro domain(s) IPR003757 Photosystem I reaction centre%2C subunit XI PsaL
Solyc02g080810.2.1	3.770539887	1.80E-10	8.77E-09	L2/L3-specific	L,D,F	Aminomethyltransferase (AHRD V1 ***- C6TF02_SOYBN)%3B contains Interpro domain(s) IPR006223 Glycine cleavage system T protein
Solyc09g065180.2.1	3.751043365	1.63E-05	0.000571318	L2/L3-specific	L	NAD-dependent epimerase/dehydratase (AHRD V1 **-- B7K7X4_CYAP7)%3B contains Interpro domain(s) IPR016040 NAD(P)-binding domain
Solyc01g088660.2.1	3.746722792	1.60E-06	3.58E-05	L2/L3-specific	D	Os04g0585900 protein (Fragment) (AHRD V1 *-*- Q0JAP2_ORYSJ)%3B contains Interpro domain(s) IPR007650 Protein of unknown function DUF581
Solyc09g005920.2.1	3.740583126	0.0005665	0.013238726	L2/L3-specific	F	G protein-coupled seven transmembrane receptor (AHRD V1 **-- A8J5P4_CHLRE)%3B contains Interpro domain(s) IPR009637 Transmembrane receptor%2C eukaryota
Solyc03g111700.2.1	3.738269433	2.79E-06	0.000123183	L2/L3-specific	L,D	Inactivated Zn-dependent hydrolase of the beta-lactamase fold (AHRD V1 ***- B5ILK8_9CHRO)
Solyc09g092380.2.1	3.734214718	1.87E-13	5.13E-11	L2/L3-specific	F	Adenosylhomocysteinase (AHRD V1 **** Q2XTD0_SOLTU)%3B contains Interpro domain(s) IPR000043 S-adenosyl-L-homocysteine hydrolase
Solyc04g009630.2.1	3.723365391	3.27E-07	1.85E-05	L2/L3-specific	L	Alpha-glucosidase 1 (AHRD V1 **** O22444_ARATH)%3B contains Interpro domain(s) IPR000322 Glycoside hydrolase%2C family 31
Solyc01g111020.2.1	3.721296022	0.0009138	0.017025238	L2/L3-specific	L,D	Pentatricopeptide repeat-containing protein (AHRD V1 *-*- D7L4E9_ARALY)%3B contains Interpro domain(s) IPR002885 Pentatricopeptide repeat
Solyc01g109250.2.1	3.721016209	4.50E-05	0.001372775	L2/L3-specific	L,D	TMV response-related protein (AHRD V1 **-- B6SLK0_MAIZE)
Solyc11g007830.1.1	3.702565592	2.47E-10	1.17E-08	L2/L3-specific	D	Dual-specificity protein-like phosphatase 3 (AHRD V1 ***- Q3S4H5_MAIZE)%3B contains Interpro domain(s) IPR000387 Dual-specific/protein-tyrosine phosphatase%2C conserved region IPR020422 Dual specificity phosphatase%2C subgroup%2C catalytic domain
Solyc04g049330.2.1	3.69724128	1.74E-08	1.86E-06	L2/L3-specific	F	V-type proton ATPase subunit G 1 (AHRD V1 ***- VATG1_TOBAC)%3B contains Interpro domain(s) IPR005124 Vacuolar (H+)-ATPase G subunit
Solyc08g075490.2.1	3.69335141	1.76E-15	3.84E-13	L2/L3-specific	L,D	Carotenoid cleavage dioxygenase 4B
Solyc07g054290.1.1	3.692945443	0.0009993	0.018301888	L2/L3-specific	L	Photosystem II family protein (AHRD V1 *-*- D7KCG4_ARALY)
Solyc06g076640.2.1	3.69155273	1.16E-05	0.000572333	L2/L3-specific	F	Tubulin beta chain (AHRD V1 ***- B9GKJ5_POPTR)%3B contains Interpro domain(s) IPR002453 Beta tubulin
Solyc03g121330.2.1	3.691066075	0.0006203	0.014230647	L2/L3-specific	F	60S ribosomal protein L28 (AHRD V1 ***- B3TLS3_ELAGV)%3B contains Interpro domain(s) IPR002672 Ribosomal protein L28e
Solyc02g062000.2.1	3.68701842	0.0006202	0.014230647	L2/L3-specific	F	RUN and FYVE domain-containing protein 1 (AHRD V1 *--- B0WS48_CULQU)%3B contains Interpro domain(s) IPR000306 Zinc finger%2C FYVE-type
Solyc05g012100.2.1	3.672011319	3.42E-08	1.12E-06	L2/L3-specific	D	Fructokinase-like protein 2 (AHRD V1 **-- D9IWP0_NICBE)%3B contains Interpro domain(s) IPR011611 Carbohydrate/purine kinase
Solyc12g096550.1.1	3.667670839	1.54E-05	0.000255378	L2/L3-specific	L,D	Pheophorbide a oxygenase family (AHRD V1 **-- B4VSS3_9CYAN)%3B contains Interpro domain(s) IPR013626 Pheophorbide a oxygenase
Solyc03g034070.1.1	3.665712803	0.0010961	0.019597126	L2/L3-specific	L,D	P-166-4_1 (Fragment) (AHRD V1 *-*- Q4VDD8_PINRE)
Solyc07g017510.2.1	3.663308776	5.82E-16	6.32E-14	L2/L3-specific	D,F	Phosphatidylinositol-4-phosphate 5-kinase family protein (AHRD V1 **-* D7L328_ARALY)%3B contains Interpro domain(s) IPR016034 Phosphatidylinositol-4-phosphate 5-kinase%2C core%2C subgroup
Solyc02g062340.2.1	3.663061163	3.02E-11	3.61E-09	L2/L3-specific	L,D,F	Fructose-bisphosphate aldolase (AHRD V1 ***- Q9SXX4_NICPA)%3B contains Interpro domain(s) IPR000741 Fructose-bisphosphate aldolase%2C class-I
Solyc08g067030.2.1	3.662908528	3.36E-06	6.86E-05	L2/L3-specific	D	Os01g0611000 protein (Fragment) (AHRD V1 ***- Q0JLB5_ORYSJ)%3B contains Interpro domain(s) IPR006946 Protein of unknown function DUF642
Solyc06g062670.2.1	3.658632289	0.0007713	0.007184314	L2/L3-specific	L,D	Zinc finger family protein (AHRD V1 *-*- D7M7Z8_ARALY)%3B contains Interpro domain(s) IPR007087 Zinc finger%2C C2H2-type
Solyc07g045540.2.1	3.657948656	7.94E-05	0.002266536	L2/L3-specific	L,D	Glucose-6-phosphate 1-dehydrogenase (AHRD V1 **** O65856_TOBAC)%3B contains Interpro domain(s) IPR001282 Glucose-6-phosphate dehydrogenase
Solyc02g079950.2.1	3.65353219	1.46E-06	7.03E-05	L2/L3-specific	L,D	Oxygen-evolving enhancer protein 3 (AHRD V1 ***- Q7Y1T5_PEA)%3B contains Interpro domain(s) IPR008797 Photosystem II oxygen evolving complex protein PsbQ
Solyc10g011870.2.1	3.648737687	0.0013874	0.023843074	L2/L3-specific	L	Reticulon family protein (AHRD V1 ***- B2WS91_9BRAS)%3B contains Interpro domain(s) IPR003388 Reticulon
Solyc03g005760.1.1	3.644318524	5.43E-06	0.000221424	L2/L3-specific	L	Chlorophyll a-b binding protein 3C-like (AHRD V1 **** Q2XTE0_SOLTU)%3B contains Interpro domain(s) IPR001344 Chlorophyll A-B binding protein
Solyc12g007090.1.1	3.643564841	3.95E-05	0.0005776	L2/L3-specific	D	Os08g0387500 protein (Fragment) (AHRD V1 ***- Q0J5Y7_ORYSJ)
Solyc06g054260.1.1	3.641137233	8.04E-16	1.81E-13	L2/L3-specific	L,D,F	Photosystem I reaction center subunit II (AHRD V1 ***- B4FAW3_MAIZE)%3B contains Interpro domain(s) IPR003685 Photosystem I protein PsaD
Solyc12g096420.1.1	3.635788372	7.21E-05	0.002084077	L2/L3-specific	L,D	RING-finger protein like (AHRD V1 *-*- B6SVN2_MAIZE)%3B contains Interpro domain(s) IPR018957 Zinc finger%2C C3HC4 RING-type
Solyc07g063190.2.1	3.630705381	1.20E-12	8.06E-11	L2/L3-specific	D	Thioredoxin (AHRD V1 ***- A7LNX7_9CARY)%3B contains Interpro domain(s) IPR005746 Thioredoxin
Solyc09g065370.1.1	3.629482675	1.51E-08	5.28E-07	L2/L3-specific	D	Unknown Protein (AHRD V1)
Solyc11g013760.1.1	3.627749739	0.0007984	0.017195099	L2/L3-specific	F	Unknown Protein (AHRD V1)
Solyc09g074320.2.1	3.62279385	0.0020597	0.032065128	L2/L3-specific	L,D,F	Serine/threonine-protein phosphatase (AHRD V1 **** B9I9Z0_POPTR)%3B contains Interpro domain(s) IPR012391 Serine/threonine protein phosphatase%2C BSU1
Solyc11g008250.1.1	3.620795657	8.56E-05	0.002408271	L2/L3-specific	L	Single-stranded nucleic acid binding R3H domain protein (AHRD V1 **-- B4B323_9CHRO)%3B contains Interpro domain(s) IPR003959 ATPase%2C AAA-type%2C core
Solyc08g080570.2.1	3.619177958	2.78E-14	2.45E-12	L2/L3-specific	D	UDP-glucose 4-epimerase (AHRD V1 **** B0M3E8_PEA)%3B contains Interpro domain(s) IPR005886 UDP-glucose 4-epimerase
Solyc12g099360.1.1	3.606979105	1.74E-06	0.000111704	L2/L3-specific	F	Acyl-CoA synthetase/AMP-acid ligase II (AHRD V1 **** Q2W3Z0_MAGSA)%3B contains Interpro domain(s) IPR000873 AMP-dependent synthetase and ligase
Solyc06g084050.2.1	3.601533755	0.0014154	0.024214011	L2/L3-specific	L	Photosystem II reaction center W protein (AHRD V1 ***- B6TMB3_MAIZE)%3B contains Interpro domain(s) IPR009806 Photosystem II protein PsbW%2C class 2
Solyc03g114250.2.1	3.599606035	0.0018728	0.029867166	L2/L3-specific	L	Phosphoglycerate mutase family protein (AHRD V1 **-- B6TTB7_MAIZE)%3B contains Interpro domain(s) IPR013078 Phosphoglycerate mutase
Solyc10g083830.1.1	3.59670454	0.0018864	0.014803642	L2/L3-specific	D	Glutamine synthetase I (AHRD V1 ***- Q9SC91_MEDTR)%3B contains Interpro domain(s) IPR008146 Glutamine synthetase%2C catalytic region
Solyc09g065270.2.1	3.586735674	9.45E-07	2.23E-05	L2/L3-specific	D	Ribosome recycling factor (AHRD V1 **** B6TQ52_MAIZE)%3B contains Interpro domain(s) IPR015998 Ribosome recycling factor%2C bacterial-like
Solyc10g005050.2.1	3.571164889	0.0017028	0.027994445	L2/L3-specific	L	Thylakoid membrane phosphoprotein 14 kDa%2C chloroplastic (AHRD V1 **-- TMP14_ARATH)
Solyc10g077120.1.1	3.571086245	1.49E-14	2.85E-12	L2/L3-specific	L,D,F	Photosystem II core complex proteins psbY (AHRD V1 *-*- B6SR26_MAIZE)
Solyc03g095310.2.1	3.570199372	3.56E-07	9.34E-06	L2/L3-specific	D	Cytochrome P450
Solyc06g069440.2.1	3.566990551	0.0001076	0.002884737	L2/L3-specific	L	Zinc finger CCCH domain-containing protein 55 (AHRD V1 *-*- C3H55_ORYSJ)
Solyc07g061790.2.1	3.565715662	6.98E-09	8.39E-07	L2/L3-specific	L,D,F	Heme-binding protein 2 (AHRD V1 **** B6T0C0_MAIZE)%3B contains Interpro domain(s) IPR006917 SOUL haem-binding protein
Solyc03g059260.2.1	3.563266207	3.32E-05	0.001373282	L2/L3-specific	F	Carboxyl-terminal-processing protease (AHRD V1 ***- B6T6L2_MAIZE)%3B contains Interpro domain(s) IPR004447 Peptidase S41A%2C C-terminal protease
Solyc03g120850.2.1	3.559871217	1.12E-07	3.28E-06	L2/L3-specific	D	chaperonin (AHRD V1 ***- B2IXD2_NOSP7)%3B contains Interpro domain(s) IPR001844 Chaperonin Cpn60
Solyc07g005600.2.1	3.559534746	0.0010609	0.021083173	L2/L3-specific	F	Transmembrane 9 superfamily protein member 4 (AHRD V1 **** B6SXZ2_MAIZE)%3B contains Interpro domain(s) IPR004240 Nonaspanin (TM9SF)
Solyc06g082220.2.1	3.553940729	0.0010567	0.021071085	L2/L3-specific	F	HIV-1 Tat specific factor 1 (Fragment) (AHRD V1 *--- Q5H918_HUMAN)%3B contains Interpro domain(s) IPR012677 Nucleotide-binding%2C alpha-beta plait
Solyc03g113920.2.1	3.541397473	0.0011362	0.009839278	L2/L3-specific	L,D	Calmodulin-binding protein (AHRD V1 **** D7MMC2_ARALY)%3B contains Interpro domain(s) IPR012416 Calmodulin binding protein-like
Solyc02g087000.2.1	3.53590676	7.66E-07	3.91E-05	L2/L3-specific	L,D	Potassium transporter (AHRD V1 **** D2JYH2_GOSHI)%3B contains Interpro domain(s) IPR018519 Potassium uptake protein%2C kup IPR003855 K+ potassium transporter
Solyc03g121530.2.1	3.534355924	7.06E-05	0.000963494	L2/L3-specific	D	Ribonuclease III (AHRD V1 ***- A2Q464_MEDTR)%3B contains Interpro domain(s) IPR000999 Ribonuclease III
Solyc08g082590.2.1	3.531790651	9.04E-06	0.000345809	L2/L3-specific	L	Glutaredoxin family protein (AHRD V1 **-- D7KS91_ARALY)%3B contains Interpro domain(s) IPR012335 Thioredoxin fold
Solyc08g078010.2.1	3.531397101	6.90E-06	0.00012914	L2/L3-specific	D	50S ribosomal protein L19 (AHRD V1 *-*- B6T8M1_MAIZE)%3B contains Interpro domain(s) IPR001857 Ribosomal protein L19
Solyc08g078530.2.1	3.530112429	2.06E-08	2.19E-06	L2/L3-specific	F	Agenet domain containing protein expressed (AHRD V1 *-*- Q108Y7_ORYSJ)%3B contains Interpro domain(s) IPR008395 Agenet
Solyc12g017510.1.1	3.530029599	8.56E-07	2.05E-05	L2/L3-specific	D	Os03g0425000 protein (Fragment) (AHRD V1 ***- Q0DR24_ORYSJ)%3B contains Interpro domain(s) IPR013584 RAP domain
Solyc04g049930.2.1	3.519458493	0.0013126	0.024872638	L2/L3-specific	F	Unknown Protein (AHRD V1)
Solyc03g111160.2.1	3.518980299	5.32E-05	0.000748002	L2/L3-specific	D	Dual-specificity protein-like phosphatase 3 (AHRD V1 **-- Q3S4H5_MAIZE)%3B contains Interpro domain(s) IPR020422 Dual specificity phosphatase%2C subgroup%2C catalytic domain
Solyc03g118040.2.1	3.516714195	9.71E-13	2.34E-10	L2/L3-specific	F	Calcium-binding protein Calnexin (AHRD V1 **-* Q9BLH3_HALRO)%3B contains Interpro domain(s) IPR018124 Calreticulin/calnexin%2C conserved site IPR001580 Calreticulin/calnexin
Solyc05g013160.2.1	3.506920544	0.0001274	0.00329993	L2/L3-specific	L,D	Set domain protein (AHRD V1 ***- C1EFC8_9CHLO)%3B contains Interpro domain(s) IPR011192 Rubisco methyltransferase
Solyc08g081180.2.1	3.504402734	0.000142	0.003650184	L2/L3-specific	L	Signal peptide peptidase (AHRD V1 **** Q9MA44_ARATH)%3B contains Interpro domain(s) IPR007369 Peptidase A22B%2C signal peptide peptidase
Solyc10g018300.1.1	3.502707935	1.49E-10	1.64E-08	L2/L3-specific	L,D	Transketolase 1 (AHRD V1 **** O78327_CAPAN)%3B contains Interpro domain(s) IPR005478 Bacterial transketolase
Solyc04g015750.2.1	3.494961465	1.36E-14	2.64E-12	L2/L3-specific	L,F	Magnesium chelatase H subunit (AHRD V1 ***- D5KXY0_VITVI)%3B contains Interpro domain(s) IPR011771 Magnesium-chelatase%2C subunit H
Solyc06g053600.2.1	3.484576013	0.0021293	0.032999167	L2/L3-specific	L,D	Oxidoreductase aldo/keto reductase family protein expressed (AHRD V1 ***- Q10GW4_ORYSJ)%3B contains Interpro domain(s) IPR020471 Aldo/keto reductase subgroup
Solyc11g008620.1.1	3.483117814	8.00E-06	0.000313128	L2/L3-specific	L,D	Phosphoglycolate phosphatase (AHRD V1 **-- C1FHY2_9CHLO)%3B contains Interpro domain(s) IPR006349 2-phosphoglycolate phosphatase%2C eukaryotic
Solyc11g066110.1.1	3.476749288	2.18E-06	9.89E-05	L2/L3-specific	L	Inner membrane protein oxaA (AHRD V1 *-*- B9L0L4_THERP)%3B contains Interpro domain(s) IPR001708 Membrane insertion protein%2C OxaA/YidC
Solyc11g065240.1.1	3.471110328	8.15E-05	0.001083831	L2/L3-specific	D	Saccharopine dehydrogenase family protein expressed (AHRD V1 ***- Q2QY08_ORYSJ)%3B contains Interpro domain(s) IPR005097 Saccharopine dehydrogenase
Solyc02g072240.2.1	3.46984826	0.0029255	0.020925598	L2/L3-specific	D	Cellulose synthase (AHRD V1 **** Q6J8W9_9ROSI)%3B contains Interpro domain(s) IPR005150 Cellulose synthase
Solyc10g005920.2.1	3.468177268	1.89E-09	7.66E-08	L2/L3-specific	D	Enhancer of mRNA-decapping protein 4 (AHRD V1 *--- EDC4_DANRE)%3B contains Interpro domain(s) IPR017986 WD40 repeat%2C region
Solyc04g011390.1.1	3.454583177	0.0016599	0.02972921	L2/L3-specific	F	Histone H4 (AHRD V1 ***- B6T0P4_MAIZE)%3B contains Interpro domain(s) IPR001951 Histone H4
Solyc03g059180.2.1	3.453690919	3.95E-08	2.60E-06	L2/L3-specific	L,D	Extracellular calcium sensing receptor (AHRD V1 *-*- C7BCU7_LIQFO)%3B contains Interpro domain(s) IPR001763 Rhodanese-like
Solyc07g055470.2.1	3.441476516	2.22E-06	4.79E-05	L2/L3-specific	D	Cytochrome P450
Solyc12g057040.1.1	3.439720043	6.70E-09	2.51E-07	L2/L3-specific	D	Cryptochrome 1b
Solyc07g064940.2.1	3.433446653	2.49E-06	5.33E-05	L2/L3-specific	D	Thioredoxin family protein (AHRD V1 *-*- Q1EPD8_MUSAC)%3B contains Interpro domain(s) IPR013766 Thioredoxin domain
Solyc02g086150.2.1	3.433192992	0.0001235	0.001540833	L2/L3-specific	D	Peptidyl-tRNA hydrolase family protein (AHRD V1 **** D7M817_ARALY)%3B contains Interpro domain(s) IPR001328 Peptidyl-tRNA hydrolase
Solyc03g080060.1.1	3.433172261	1.39E-06	3.15E-05	L2/L3-specific	D	Receptor-like kinase (AHRD V1 **** A7VM32_MARPO)%3B contains Interpro domain(s) IPR001220 Legume lectin%2C beta chain
Solyc03g019650.2.1	3.430243005	6.45E-05	0.002405001	L2/L3-specific	L,F	Nodulin-like protein (AHRD V1 *-*- B6U8U8_MAIZE)%3B contains Interpro domain(s) IPR010658 Nodulin-like
Solyc07g043600.2.1	3.428579522	2.40E-06	5.15E-05	L2/L3-specific	D	Pentatricopeptide repeat-containing protein (AHRD V1 ***- D7LWA0_ARALY)%3B contains Interpro domain(s) IPR002885 Pentatricopeptide repeat
Solyc04g008710.2.1	3.421049523	0.0001951	0.004791804	L2/L3-specific	L,D	Unknown Protein (AHRD V1)
Solyc04g074850.2.1	3.41945021	0.0017789	0.0141116	L2/L3-specific	D,F	Multidrug resistance protein mdtK (AHRD V1 **-- MDTK_YERP3)%3B contains Interpro domain(s) IPR002528 Multi antimicrobial extrusion protein MatE
Solyc01g111630.2.1	3.417331405	2.34E-08	7.92E-07	L2/L3-specific	L,D	Glyoxylate/hydroxypyruvate reductase B (AHRD V1 **** GHRB_YERPS)%3B contains Interpro domain(s) IPR006140 D-isomer specific 2-hydroxyacid dehydrogenase%2C NAD-binding
Solyc12g088050.1.1	3.415836695	0.0001323	0.001623193	L2/L3-specific	D	Rhamnogalacturonate lyase (AHRD V1 ***- A3XLZ2_LEEBM)%3B contains Interpro domain(s) IPR010325 Rhamnogalacturonate lyase
Solyc01g090680.2.1	3.414322358	5.89E-06	0.000112569	L2/L3-specific	D	Genomic DNA chromosome 5 TAC clone K11J9 (AHRD V1 *-*- Q9FKH0_ARATH)
Solyc10g007690.2.1	3.411944725	1.19E-06	8.16E-05	L2/L3-specific	L,F	Chlorophyll a-b binding protein 8%2C chloroplastic (AHRD V1 ***- CB13_SOLLC)%3B contains Interpro domain(s) IPR001344 Chlorophyll A-B binding protein
Solyc03g114450.2.1	3.411259994	1.00E-05	0.00037815	L2/L3-specific	L,D	Extracellular calcium sensing receptor (AHRD V1 ***- C7BCU7_LIQFO)%3B contains Interpro domain(s) IPR001763 Rhodanese-like
Solyc06g048540.2.1	3.404484988	0.0001309	0.001610064	L2/L3-specific	D	FeS assembly ATPase SufC (AHRD V1 **** B5W7S4_SPIMA)%3B contains Interpro domain(s) IPR010230 ATPase SufC%2C SUF system FeS cluster assembly

Solyc02g064950.2.1	3.399481318	0.0025882	0.019072349	L2/L3-specific	D	CBS domain-containing protein-like (AHRD V1 ***- B8AP73_ORYSI)%3B contains Interpro domain(s) IPR000644 Cystathionine beta-synthase%2C core
Solyc08g067500.1.1	3.398008487	0.0027847	0.020135838	L2/L3-specific	D	Non-specific lipid-transfer protein (AHRD V1 ***- A9YUH6_PLAOI)%3B contains Interpro domain(s) IPR013770 Plant lipid transfer protein and hydrophobic protein%2C helical
Solyc01g108540.2.1	3.388387574	1.51E-06	3.39E-05	L2/L3-specific	D	Acetyl esterase (AHRD V1 *--- AES_SHIFL)%3B contains Interpro domain(s) IPR013094 Alpha/beta hydrolase fold-3
Solyc02g089900.1.1	3.384636957	8.84E-06	0.00033924	L2/L3-specific	L	Receptor-like kinase (AHRD V1 *-*- A7VM35_MARPO)%3B contains Interpro domain(s) IPR002290 Serine/threonine protein kinase
Solyc03g118020.2.1	3.382850893	1.40E-06	9.36E-05	L2/L3-specific	F	Tudor / nuclease domain-containing protein (AHRD V1 ***- B6KG97_TOXGO)%3B contains Interpro domain(s) IPR016685 RNA-induced silencing complex%2C nuclease component Tudor-SN
Solyc12g011280.1.1	3.379787558	7.03E-07	3.67E-05	L2/L3-specific	L	Chlorophyll a-b binding protein 8%2C chloroplastic (AHRD V1 ***- CB13_SOLLC)%3B contains Interpro domain(s) IPR001344 Chlorophyll A-B binding protein
Solyc02g092700.2.1	3.374183284	1.36E-05	0.000491973	L2/L3-specific	L	Ycf36 protein (AHRD V1 *-*- D4ZSA0_SPIPL)%3B contains Interpro domain(s) IPR009631 Protein of unknown function DUF1230
Solyc05g010670.2.1	3.372973872	8.13E-06	0.000316365	L2/L3-specific	L,D	Chaperone protein htpG (AHRD V1 ***- HTPG_MYXXD)%3B contains Interpro domain(s) IPR001404 Heat shock protein Hsp90
Solyc06g069170.2.1	3.368416076	3.13E-12	1.93E-10	L2/L3-specific	L,D,F	Unknown Protein (AHRD V1)
Solyc12g010840.1.1	3.362450241	0.0002258	0.005392759	L2/L3-specific	L,D	Ketol-acid reductoisomerase (AHRD V1 ***- D7LW57_ARALY)%3B contains Interpro domain(s) IPR016206 Ketol-acid reductoisomerase%2C plant
Solyc07g063430.2.1	3.354217842	1.54E-08	1.12E-06	L2/L3-specific	L,D	Mpv17 protein (AHRD V1 *--- B0WWR6_CULQU)%3B contains Interpro domain(s) IPR007248 Mpv17/PMP22
Solyc04g006970.2.1	3.345228882	1.53E-08	1.12E-06	L2/L3-specific	L,F	Phosphoenolpyruvate carboxylase (AHRD V1 **** A6YM33_RICCO)%3B contains Interpro domain(s) IPR015813 Pyruvate/Phosphoenolpyruvate kinase%2C catalytic core
Solyc01g087680.2.1	3.338857294	0.0002237	0.00536038	L2/L3-specific	L,D	Uncharacterized membrane protein (AHRD V1 ***- A5GLU3_SYNPW)%3B contains Interpro domain(s) IPR016804 Uncharacterised conserved protein UCP022348
Solyc06g076850.2.1	3.333628481	0.0027328	0.042846841	L2/L3-specific	D,F	Binding protein (AHRD V1 ***- D7LLC0_ARALY)%3B contains Interpro domain(s) IPR011990 Tetratricopeptide-like helical
Solyc04g057870.2.1	3.330445204	0.0003241	0.00348322	L2/L3-specific	D	Os11g0110100 protein (Fragment) (AHRD V1 ***- Q0IV61_ORYSJ)
Solyc08g077730.2.1	3.326181636	4.91E-06	9.57E-05	L2/L3-specific	D	1-phosphatidylinositol-4-phosphate 5-kinase-like protein (AHRD V1 *--- Q67UY9_ORYSJ)%3B contains Interpro domain(s) IPR003409 MORN motif
Solyc00g024150.2.1	3.313742112	4.17E-06	8.28E-05	L2/L3-specific	D	Pterin-4-alpha-carbinolamine dehydratase (AHRD V1 *-** B6T9C1_MAIZE)%3B contains Interpro domain(s) IPR001533 Transcriptional coactivator/pterin dehydratase
Solyc10g082030.1.1	3.29698768	3.88E-06	0.000166306	L2/L3-specific	L	Peroxiredoxin (AHRD V1 **-- Q9FE12_PHAVU)%3B contains Interpro domain(s) IPR000866 Alkyl hydroperoxide reductase/ Thiol specific antioxidant/ Mal allergen
Solyc06g005940.2.1	3.296810589	2.36E-11	4.62E-09	L2/L3-specific	F	Protein disulfide isomerase (AHRD V1 **** Q6IV17_IPOBA)%3B contains Interpro domain(s) IPR005792 Protein disulphide isomerase
Solyc07g044860.2.1	3.2885997	2.56E-08	1.73E-06	L2/L3-specific	L,D,F	Oxygen-evolving enhancer protein 2%2C chloroplastic (AHRD V1 ***- PSBP_SOLLC)%3B contains Interpro domain(s) IPR002683 Photosystem II oxygen evolving complex protein PsbP
Solyc02g020980.2.1	3.283572242	0.0001066	0.002865966	L2/L3-specific	L	4-alpha-glucanotransferase (AHRD V1 **** Q6R608_SOLTU)%3B contains Interpro domain(s) IPR003385 Glycoside hydrolase%2C family 77
Solyc02g088000.2.1	3.282926018	0.0002481	0.005793937	L2/L3-specific	L,D	Glycogen synthase (AHRD V1 **** D5MJP6_9BACT)%3B contains Interpro domain(s) IPR011835 Glycogen/starch synthases%2C ADP-glucose type
Solyc03g111840.2.1	3.280004493	0.0002734	0.006331457	L2/L3-specific	L,D	Polyadenylate-binding protein 1-like (AHRD V1 **-- PAP1L_HUMAN)%3B contains Interpro domain(s) IPR000504 RNA recognition motif%2C RNP-1
Solyc05g056580.2.1	3.27320657	1.98E-05	0.000894119	L2/L3-specific	F	Dehydration-responsive family protein (AHRD V1 **-- D7LF23_ARALY)%3B contains Interpro domain(s) IPR004159 Protein of unknown function DUF248%2C methyltransferase putative
Solyc03g118530.2.1	3.271514875	7.77E-06	0.000143492	L2/L3-specific	D	Protein serine/threonine kinase (AHRD V1 *-*- D3BAM4_POLPA)%3B contains Interpro domain(s) IPR002290 Serine/threonine protein kinase
Solyc05g056260.2.1	3.27088277	7.78E-05	0.001045074	L2/L3-specific	D	Phenylalanyl-tRNA synthetase alpha chain (AHRD V1 *-** Q5N0F1_SYNP6)%3B contains Interpro domain(s) IPR004530 Phenylalanyl-tRNA synthetase%2C class IIc%2C mitochondrial
Solyc09g018280.1.1	3.265962603	1.07E-09	4.51E-08	L2/L3-specific	L,D	Calcium/calmodulin-dependent protein kinase type 1 (AHRD V1 *-** KCC1A_MOUSE)%3B contains Interpro domain(s) IPR002290 Serine/threonine protein kinase
Solyc02g078210.2.1	3.264251405	2.08E-09	1.83E-07	L2/L3-specific	L,D	Ubiquitin-conjugating enzyme 22 (AHRD V1 ***- D7L888_ARALY)%3B contains Interpro domain(s) IPR000608 Ubiquitin-conjugating enzyme%2C E2
Solyc12g015860.1.1	3.264188568	0.0002128	0.006133039	L2/L3-specific	F	Farnesyl pyrophosphate synthase (AHRD V1 ***- O65004_SOLLC)%3B contains Interpro domain(s) IPR000092 Polyprenyl synthetase
Solyc03g095990.1.1	3.255784199	0.0042989	0.027866747	L2/L3-specific	D	Unknown Protein (AHRD V1)
Solyc04g076300.2.1	3.254256676	8.97E-05	0.002493422	L2/L3-specific	L,D	Transporter small conductance mechanosensitive ion channel (MscS) family (AHRD V1 *-** A7ZLG1_ECO24)%3B contains Interpro domain(s) IPR006685 Mechanosensitive ion channel MscS
Solyc08g077210.2.1	3.247126607	8.65E-06	0.000332547	L2/L3-specific	L	Inositol 1 4 5-trisphosphate 5-phosphatase (AHRD V1 *-** Q712G2_ARATH)%3B contains Interpro domain(s) IPR000300 Inositol polyphosphate related phosphatase
Solyc11g068520.1.1	3.242845143	0.0056247	0.034573839	L2/L3-specific	D	Rho GTPase activating protein 2 (AHRD V1 *-*- Q6UQ72_ORYSJ)%3B contains Interpro domain(s) IPR000198 RhoGAP
Solyc08g081230.1.1	3.239485401	5.32E-09	4.35E-07	L2/L3-specific	L	Potassium channel tetramerization domain-containing protein (AHRD V1 ***- D7MIW8_ARALY)%3B contains Interpro domain(s) IPR003131 Potassium channel%2C voltage dependent%2C Kv%2C tetramerisation
Solyc07g066150.1.1	3.237121685	1.14E-09	1.07E-07	L2/L3-specific	L,D	Photosystem I reaction center subunit V (AHRD V1 ***- B6U534_MAIZE)%3B contains Interpro domain(s) IPR017494 Photosystem I reaction center%2C PsaG%2C plant
Solyc01g010770.2.1	3.235542517	6.42E-10	2.80E-08	L2/L3-specific	D	Spfh domain / band 7 family protein (AHRD V1 **-- B4AS86_FRANO)%3B contains Interpro domain(s) IPR001107 Band 7 protein
Solyc03g113270.2.1	3.235308579	2.60E-05	0.001130432	L2/L3-specific	F	Homeobox-leucine zipper-like protein (AHRD V1 *-*- Q3HRT1_PICGL)%3B contains Interpro domain(s) IPR001356 Homeobox IPR017970 Homeobox%2C conserved site
Solyc07g061900.2.1	3.229281386	1.31E-05	0.000221592	L2/L3-specific	D	50S ribosomal protein L4 (AHRD V1 ***- B6TG27_MAIZE)%3B contains Interpro domain(s) IPR015498 Ribosomal protein L4
Solyc09g091010.2.1	3.225487362	4.99E-05	0.00070869	L2/L3-specific	D	Unknown Protein (AHRD V1)
Solyc06g071000.2.1	3.224837621	2.42E-13	1.83E-11	L2/L3-specific	D	N-succinylglutamate 5-semialdehyde dehydrogenase (AHRD V1 *-*- ASTD_VIBCH)%3B contains Interpro domain(s) IPR015590 Aldehyde dehydrogenase
Solyc12g007010.1.1	3.219664773	1.42E-05	0.000238575	L2/L3-specific	L,D	Cytosolic Fe-S cluster assembly factor NUBP2 homolog (AHRD V1 *--- NUBP2_NEMVE)%3B contains Interpro domain(s) IPR019591 ATPase-like%2C ParA/MinD
Solyc08g077240.2.1	3.214314627	2.68E-05	0.001161487	L2/L3-specific	F	Dehydration-responsive family protein (AHRD V1 ***- D7MCX6_ARALY)%3B contains Interpro domain(s) IPR004159 Protein of unknown function DUF248%2C methyltransferase putative
Solyc01g096660.2.1	3.208170847	4.48E-05	0.001368981	L2/L3-specific	L,D	Genomic DNA chromosome 5 P1 clone MOJ9 (AHRD V1 ***- Q9FL44_ARATH)
Solyc01g101120.2.1	3.204760196	0.0050363	0.031685793	L2/L3-specific	D	Glucan endo-1 3-beta-glucosidase 1 (AHRD V1 *-*- B4FGE4_MAIZE)%3B contains Interpro domain(s) IPR012946 X8
Solyc04g009950.2.1	3.194712689	0.00028	0.007650949	L2/L3-specific	F	Pre-mRNA splicing factor (AHRD V1 **-- C5K279_AJEDS)%3B contains Interpro domain(s) IPR015495 Myb transcription factor
Solyc07g062190.2.1	3.194038965	9.91E-06	0.000175453	L2/L3-specific	D	50S ribosomal protein L13 (AHRD V1 ***- B6SVH3_MAIZE)%3B contains Interpro domain(s) IPR005823 Ribosomal protein L13%2C bacterial-type
Solyc02g062350.2.1	3.190980164	2.61E-06	5.54E-05	L2/L3-specific	D	Heat shock protein DnaJ domain protein (AHRD V1 **-- D7DZS3_NOSA0)
Solyc01g067890.2.1	3.189510979	2.99E-06	6.22E-05	L2/L3-specific	L,D	1-deoxy-D-xylulose 5-phosphate synthase 1 (AHRD V1 ***- C7U110_SOLLC)%3B contains Interpro domain(s) IPR005474 Transketolase%2C N-terminal IPR005475 Transketolase%2C central region IPR005477 Deoxyxylulose-5-phosphate synthase
Solyc10g049620.1.1	3.188331891	0.0002442	0.006837491	L2/L3-specific	F	Progesterone 5-beta-reductase (AHRD V1 ***- D6NIX2_ATRBE)%3B contains Interpro domain(s) IPR016040 NAD(P)-binding domain
Solyc03g117980.2.1	3.186892286	2.62E-11	3.15E-09	L2/L3-specific	L	Respiratory burst oxidase-like protein (AHRD V1 **** C1IHQ9_9ROSI)%3B contains Interpro domain(s) IPR013121 Ferric reductase%2C NAD binding
Solyc05g005020.2.1	3.180191427	3.74E-08	1.21E-06	L2/L3-specific	D,F	R1 protein alpha-glucan water dikinase (AHRD V1 **** A8I6T5_CHLRE)%3B contains Interpro domain(s) IPR002192 Pyruvate phosphate dikinase%2C PEP/pyruvate-binding
Solyc04g054310.2.1	3.173322039	1.35E-06	3.06E-05	L2/L3-specific	D	Alanine-glyoxylate aminotransferase (AHRD V1 **** D2KZ10_WHEAT)%3B contains Interpro domain(s) IPR005814 Aminotransferase class-III
Solyc01g100650.2.1	3.171220253	6.31E-05	0.000869958	L2/L3-specific	D	NHL repeat containing 2 (AHRD V1 *--- B2RR26_MOUSE)%3B contains Interpro domain(s) IPR011042 Six-bladed beta-propeller%2C TolB-like
Solyc02g085100.2.1	3.156610509	0.0006266	0.012615552	L2/L3-specific	L,D	Aldose 1-epimerase family protein (AHRD V1 **** D7MMG0_ARALY)%3B contains Interpro domain(s) IPR014718 Glycoside hydrolase-type carbohydrate-binding%2C subgroup
Solyc04g051800.2.1	3.145968025	2.15E-09	1.88E-07	L2/L3-specific	L	ABC transporter ATP-binding protein (AHRD V1 ***- D4ZPJ5_SPIPL)%3B contains Interpro domain(s) IPR003439 ABC transporter-like
Solyc12g006320.1.1	3.144579475	1.54E-11	8.75E-10	L2/L3-specific	D	ATP-dependent RNA helicase (AHRD V1 *-*- Q6L724_HORVU)%3B contains Interpro domain(s) IPR011545 DNA/RNA helicase%2C DEAD/DEAH box type%2C N-terminal
Solyc09g014760.2.1	3.144144967	9.43E-06	0.00035765	L2/L3-specific	L,D	Chlorophyll synthase (AHRD V1 **** C3W4Q2_TOBAC)%3B contains Interpro domain(s) IPR011799 Chlorophyll synthase%2C ChlG
Solyc01g006940.2.1	3.142067439	0.0001153	0.001460851	L2/L3-specific	D	Poly(U)-binding-splicing factor PUF60 (AHRD V1 *--* PUF60_BOVIN)%3B contains Interpro domain(s) IPR003954 RNA recognition%2C region 1
Solyc06g061230.2.1	3.13653297	2.55E-10	1.20E-08	L2/L3-specific	D	Unknown Protein (AHRD V1)%3B contains Interpro domain(s) IPR009632 Putative metallocarboxypeptidase inhibitor
Solyc04g072780.2.1	3.135826893	5.37E-05	0.000754528	L2/L3-specific	D	Genomic DNA chromosome 5 TAC clone K14A3 (AHRD V1 *--- Q9FGQ9_ARATH)%3B contains Interpro domain(s) IPR007650 Protein of unknown function DUF581
Solyc03g113620.2.1	3.128698061	1.61E-06	3.60E-05	L2/L3-specific	L,D	MYB transcription factor (AHRD V1 **** B2CZJ3_CAPAN)%3B contains Interpro domain(s) IPR006447 Myb-like DNA-binding region%2C SHAQKYF class
Solyc03g005230.2.1	3.128168086	5.28E-09	2.02E-07	L2/L3-specific	D	Menaquinone biosynthesis methyltransferase ubiE (AHRD V1 *-** A1BDQ8_CHLPD)%3B contains Interpro domain(s) IPR013216 Methyltransferase type 11
Solyc10g081260.1.1	3.126840944	4.85E-05	0.001884876	L2/L3-specific	F	Multidrug resistance protein mdtK (AHRD V1 ***- MDTK_ERWAM)%3B contains Interpro domain(s) IPR002528 Multi antimicrobial extrusion protein MatE
Solyc02g077990.2.1	3.092672542	1.06E-07	3.10E-06	L2/L3-specific	L,D	30S ribosomal protein S5 (AHRD V1 ***- B6TT32_MAIZE)%3B contains Interpro domain(s) IPR005712 Ribosomal protein S5%2C bacterial-type
Solyc04g074180.2.1	3.088524439	6.45E-06	0.000121812	L2/L3-specific	D	Cryptochrome 1a
Solyc10g018120.1.1	3.085818871	0.0007732	0.007197235	L2/L3-specific	D	Unknown Protein (AHRD V1)
Solyc06g009630.1.1	3.083702189	6.86E-08	2.13E-06	L2/L3-specific	D	CP12 (AHRD V1 ***- O24136_TOBAC)%3B contains Interpro domain(s) IPR003823 Protein of unknown function CP12
Solyc08g082170.2.1	3.082238825	1.79E-08	6.20E-07	L2/L3-specific	D	Glycoside hydrolase family 28 protein/polygalacturonase family protein (AHRD V1 **-- Q1PF10_ARATH)%3B contains Interpro domain(s) IPR012334 Pectin lyase fold
Solyc11g066870.1.1	3.068235902	4.02E-06	8.04E-05	L2/L3-specific	D	Genomic DNA chromosome 5 P1 clone MDJ22 (AHRD V1 **-- Q9FNJ7_ARATH)%3B contains Interpro domain(s) IPR003409 MORN motif
Solyc10g005100.2.1	3.06198354	6.28E-05	0.002347435	L2/L3-specific	F	Salt stress root protein RS1 (AHRD V1 **-- SRS1_ORYSJ)%3B contains Interpro domain(s) IPR008469 DREPP plasma membrane polypeptide
Solyc06g051080.2.1	3.058462806	0.0011189	0.019951966	L2/L3-specific	L	UDP-sugar pyrophosphorylase (AHRD V1 *--- USP_ORYSI)
Solyc06g066620.2.1	3.054471742	0.0001583	0.001892243	L2/L3-specific	L,D	Threonine endopeptidase (AHRD V1 *--- B6SNJ7_MAIZE)
Solyc02g080570.2.1	3.053492532	5.27E-07	2.85E-05	L2/L3-specific	L,D	Glycogen synthase (AHRD V1 *-** D4ZXV2_SPIPL)%3B contains Interpro domain(s) IPR011835 Glycogen/starch synthases%2C ADP-glucose type
Solyc06g082980.2.1	3.050454723	1.84E-07	1.52E-05	L2/L3-specific	L,F	3-beta-hydroxysteroid-Delta8 Delta7-isomerase (AHRD V1 **-* C3KH60_ANOFI)%3B contains Interpro domain(s) IPR007905 Emopamil-binding
Solyc09g092330.1.1	3.037410242	6.73E-06	0.000353921	L2/L3-specific	F	NAD dependent epimerase/dehydratase family protein expressed (AHRD V1 ***- Q2MJA7_ORYSJ)%3B contains Interpro domain(s) IPR016040 NAD(P)-binding domain
Solyc08g006160.2.1	3.036273472	4.43E-05	0.00135777	L2/L3-specific	L,F	Plastid fibrillin 3 (Fragment) (AHRD V1 **-- A6XBK9_COFCA)%3B contains Interpro domain(s) IPR006843 PAP fibrillin
Solyc11g006470.1.1	3.03305457	3.33E-05	0.000502103	L2/L3-specific	D	AT5G22070 protein (Fragment) (AHRD V1 ***- B9DHZ2_ARATH)%3B contains Interpro domain(s) IPR004949 Protein of unknown function DUF266%2C plant
Solyc12g042060.1.1	3.024250645	2.17E-06	0.0001367	L2/L3-specific	L,D,F	ATP-dependent clp protease ATP-binding subunit (AHRD V1 **-- Q85G08_CYAME)%3B contains Interpro domain(s) IPR013093 ATPase associated with various cellular activities%2C AAA-2
Solyc12g096500.1.1	3.011358937	4.52E-08	1.44E-06	L2/L3-specific	D	CONSTANS-like protein (AHRD V1 ***- Q0MQL9_SOLTU)%3B contains Interpro domain(s) IPR010402 CCT domain
Solyc09g082730.2.1	3.010923149	2.41E-05	0.000803366	L2/L3-specific	L	Aldo/keto reductase family protein (AHRD V1 **** D7KWQ6_ARALY)%3B contains Interpro domain(s) IPR001395 Aldo/keto reductase
Solyc11g069470.1.1	3.004253634	0.0017257	0.02820912	L2/L3-specific	L,D	Class III homeodomain-leucine zipper (AHRD V1 ***- Q1WD30_GINBI)%3B contains Interpro domain(s) IPR013978 MEKHLA
Solyc07g066610.2.1	3.002999948	6.27E-08	3.99E-06	L2/L3-specific	L	Phosphoglycerate kinase (AHRD V1 ***- O81394_SOLTU)%3B contains Interpro domain(s) IPR001576 Phosphoglycerate kinase
Solyc04g007860.2.1	3.00155491	0.0007262	0.016043591	L2/L3-specific	L,F	Phosphatidylinositol binding clathrin assembly protein like (AHRD V1 *-** Q6AXI5_DANRE)%3B contains Interpro domain(s) IPR011417 ANTH
Solyc12g035130.1.1	2.994161291	7.24E-06	0.000377778	L2/L3-specific	F	ATP dependent RNA helicase (AHRD V1 ***- B3LNW9_YEAS1)%3B contains Interpro domain(s) IPR011545 DNA/RNA helicase%2C DEAD/DEAH box type%2C N-terminal
Solyc12g015880.1.1	2.977923381	9.40E-10	1.33E-07	L2/L3-specific	F	Heat shock protein 90 (AHRD V1 ***- Q14TB1_TOBAC)%3B contains Interpro domain(s) IPR019805 Heat shock protein Hsp90%2C conserved site IPR018249 EF-HAND 2 IPR001404 Heat shock protein Hsp90
Solyc06g082940.2.1	2.973222166	3.18E-10	3.29E-08	L2/L3-specific	L,D	Photosystem I reaction center subunit XI (AHRD V1 ***- B6SHL1_MAIZE)%3B contains Interpro domain(s) IPR003757 Photosystem I reaction centre%2C subunit XI PsaL
Solyc09g025270.2.1	2.970853125	0.0001105	0.002950858	L2/L3-specific	L	U-box domain-containing protein 3 (AHRD V1 **-- PUB3_ARATH)%3B contains Interpro domain(s) IPR011989 Armadillo-like helical
Solyc10g005530.2.1	2.964921368	0.000889	0.018677166	L2/L3-specific	F	Nuclear cap-binding protein subunit 1 (AHRD V1 *-** B0X1G0_CULQU)%3B contains Interpro domain(s) IPR016024 Armadillo-type fold
Solyc01g094340.2.1	2.964750058	0.0001693	0.004226108	L2/L3-specific	L,D	Acetyl-coenzyme A carboxylase carboxyl transferase subunit alpha (AHRD V1 *-*- A0YRC5_LYNSP)%3B contains Interpro domain(s) IPR001095 Acetyl-CoA carboxylase%2C alpha subunit
Solyc01g108490.2.1	2.962240869	3.08E-06	6.34E-05	L2/L3-specific	D	Serine carboxypeptidase 1 (AHRD V1 **** B6SW38_MAIZE)%3B contains Interpro domain(s) IPR001563 Peptidase S10%2C serine carboxypeptidase
Solyc08g006780.2.1	2.957992627	1.22E-07	3.57E-06	L2/L3-specific	D,F	Complex interacting protein 9 (AHRD V1 ***- Q8LDX3_ARATH)%3B contains Interpro domain(s) IPR004401 Uncharacterised protein family UPF0133
Solyc10g080610.1.1	2.953932489	2.45E-11	1.35E-09	L2/L3-specific	D	Kelch-like protein 14 (AHRD V1 ***- B6T7I5_MAIZE)%3B contains Interpro domain(s) IPR015915 Kelch-type beta propeller
Solyc03g123610.2.1	2.951693185	0.0001738	0.00533296	L2/L3-specific	F	Alanine aminotransferase (AHRD V1 ***- Q6VEJ5_CAPAN)%3B contains Interpro domain(s) IPR004839 Aminotransferase%2C class I and II

Solyc08g065610.2.1	2.949539349	1.66E-09	1.49E-07	L2/L3-specific	L	Vacuolar processing enzyme-1b (AHRD V1 **-- Q852T2_TOBAC)%3B contains Interpro domain(s) IPR001096 Peptidase C13%2C legumain
Solyc08g006770.2.1	2.942280566	0.0010066	0.00895043	L2/L3-specific	D	Anthocyanidin synthase (Fragment) (AHRD V1 **-* P93120_DIACA)%3B contains Interpro domain(s) IPR005123 Oxoglutarate and iron-dependent oxygenase
Solyc07g017780.2.1	2.933693354	4.15E-05	0.001640841	L2/L3-specific	L,F	H-ATPase (AHRD V1 **** Q8L6I3_ORYSJ)%3B contains Interpro domain(s) IPR006534 ATPase%2C P-type%2C plasma-membrane proton-efflux
Solyc08g062920.2.1	2.932059664	3.15E-08	3.25E-06	L2/L3-specific	F	Elongation factor EF-2 (AHRD V1 **** Q9SGT4_ARATH)%3B contains Interpro domain(s) IPR000795 Protein synthesis factor%2C GTP-binding
Solyc02g081330.2.1	2.923982961	1.08E-06	5.25E-05	L2/L3-specific	L	Phytoene synthase 2
Solyc11g069790.1.1	2.916505546	1.83E-07	1.52E-05	L2/L3-specific	L,D,F	chaperonin (AHRD V1 ***- B2IXD2_NOSP7)%3B contains Interpro domain(s) IPR001844 Chaperonin Cpn60
Solyc10g006330.2.1	2.908757614	1.87E-05	0.000640443	L2/L3-specific	L	Unknown Protein (AHRD V1)
Solyc03g093690.2.1	2.904620243	6.36E-06	0.000339437	L2/L3-specific	L,D,F	Peptide methionine sulfoxide reductase msrA (AHRD V1 **** D2B4D8_STRRD)%3B contains Interpro domain(s) IPR002569 Methionine sulphoxide reductase A
Solyc06g071430.2.1	2.89436073	3.28E-05	0.001039047	L2/L3-specific	L,D	T17H3.1 protein (Fragment) (AHRD V1 ***- Q9SXD0_ARATH)
Solyc08g076480.2.1	2.887514034	4.01E-05	0.001244086	L2/L3-specific	L,D	Plastid lipid-associated protein 3%2C chloroplastic (AHRD V1 **-- PAP3_BRACM)%3B contains Interpro domain(s) IPR006843 PAP fibrillin
Solyc07g043330.1.1	2.881746052	0.0016991	0.013610089	L2/L3-specific	D	GRAS family transcription factor (AHRD V1 **-- B9IHC5_POPTR)%3B contains Interpro domain(s) IPR005202 GRAS transcription factor
Solyc03g118130.2.1	2.880992106	2.82E-07	1.61E-05	L2/L3-specific	L,F	Rubredoxin-like protein (AHRD V1 *--- A8JGB1_CHLRE)%3B contains Interpro domain(s) IPR004039 Rubredoxin-type Fe(Cys)4 protein
Solyc05g015390.2.1	2.880221875	7.95E-08	7.63E-06	L2/L3-specific	L,F	REF-like stress related protein 1 (AHRD V1 *--- Q6XNP4_HEVBR)%3B contains Interpro domain(s) IPR008802 Rubber elongation factor
Solyc02g064720.2.1	2.879711378	1.28E-07	3.68E-06	L2/L3-specific	D	Phototropic-responsive NPH3 family protein (AHRD V1 **-- D7MMM6_ARALY)%3B contains Interpro domain(s) IPR004249 NPH3
Solyc10g074790.1.1	2.873152467	2.53E-06	5.39E-05	L2/L3-specific	D,F	Auxin efflux carrier family protein (AHRD V1 ***- D7M6D2_ARALY)%3B contains Interpro domain(s) IPR004776 Auxin efflux carrier
Solyc09g005620.2.1	2.872475789	0.0027817	0.040462806	L2/L3-specific	L,D	Glutaredoxin (AHRD V1 *-*- D7G070_ECTSI)%3B contains Interpro domain(s) IPR004480 Glutaredoxin-related protein
Solyc06g054020.2.1	2.865813004	3.39E-05	0.000508462	L2/L3-specific	D	Helicase (AHRD V1 *--- B3LLX3_YEAS1)
Solyc03g112150.1.1	2.864860826	0.0006037	0.01231766	L2/L3-specific	L	Elongation factor Tu (AHRD V1 **** D7MFK2_ARALY)%3B contains Interpro domain(s) IPR004541 Translation elongation factor EFTu/EF1A%2C bacterial and organelle
Solyc03g118430.2.1	2.86415914	7.07E-07	3.68E-05	L2/L3-specific	L,D	Peptidase M16 family (AHRD V1 *--- D3IC43_9BACT)%3B contains Interpro domain(s) IPR011237 Peptidase M16%2C core
Solyc07g042380.2.1	2.861563283	0.0003141	0.008334962	L2/L3-specific	F	Ribosomal RNA processing protein (AHRD V1 *--- A6ZWN4_YEAS7)%3B contains Interpro domain(s) IPR012978 Region of unknown function%2C NUC173
Solyc09g083350.2.1	2.854552499	4.68E-05	0.000670981	L2/L3-specific	D	Group II intron splicing factor CRS1-like (AHRD V1 *--- Q5JK62_ORYSJ)%3B contains Interpro domain(s) IPR001890 RNA-binding%2C CRM domain
Solyc07g007220.2.1	2.853718746	0.0007616	0.014666525	L2/L3-specific	L	Serine/threonine phosphatase family protein (AHRD V1 **** C1E0J1_9CHLO)%3B contains Interpro domain(s) IPR015655 Protein phosphatase 2C
Solyc07g062440.1.1	2.848293509	0.0024167	0.018064752	L2/L3-specific	D	Genomic DNA chromosome 3 TAC clone K15M2 (AHRD V1 ***- Q9LKB4_ARATH)
Solyc05g008460.2.1	2.846358886	2.30E-06	0.000142659	L2/L3-specific	F	ATP synthase subunit beta (AHRD V1 ***- O82722_NICSY)%3B contains Interpro domain(s) IPR005722 ATPase%2C F1 complex%2C beta subunit
Solyc03g119700.2.1	2.843279716	0.0006776	0.013367535	L2/L3-specific	L,D	ATP-dependent Clp protease adaptor protein ClpS containing protein (AHRD V1 ***- B4FRY9_MAIZE)%3B contains Interpro domain(s) IPR003769 Adaptor protein ClpS%2C core
Solyc04g082250.2.1	2.841948257	2.90E-08	3.02E-06	L2/L3-specific	L,D,F	ATP-dependent zinc metalloprotease FTSH%2C chloroplastic (Fragment) (AHRD V1 ***- FTSH_CAPAN)%3B contains Interpro domain(s) IPR005936 Peptidase M41%2C FtsH
Solyc10g005110.2.1	2.840917949	1.76E-05	0.000287408	L2/L3-specific	D	Coproporphyrinogen III oxidase aerobic (AHRD V1 ***- A9DTF4_9FLAO)%3B contains Interpro domain(s) IPR001260 Coproporphyrinogen III oxidase
Solyc06g071500.2.1	2.840601373	1.16E-06	2.68E-05	L2/L3-specific	L,D	Boron transporter (AHRD V1 **-* A5JUZ5_9ROSI)%3B contains Interpro domain(s) IPR003020 Bicarbonate transporter%2C eukaryotic
Solyc09g031970.2.1	2.82821599	2.66E-10	1.26E-08	L2/L3-specific	L,D,F	Phosphorylase (AHRD V1 **** B9HP81_POPTR)%3B contains Interpro domain(s) IPR011833 Glycogen/starch/alpha-glucan phosphorylase
Solyc01g100320.2.1	2.821531902	6.39E-06	0.000339654	L2/L3-specific	F	Thioredoxin/protein disulfide isomerase (AHRD V1 **** Q2U681_ASPOR)%3B contains Interpro domain(s) IPR005788 Disulphide isomerase
Solyc07g063570.2.1	2.817609654	0.0021233	0.016268074	L2/L3-specific	D	Cytochrome c biogenesis protein family (AHRD V1 ***- D7MU78_ARALY)%3B contains Interpro domain(s) IPR003834 Cytochrome c assembly protein%2C transmembrane region
Solyc03g115980.1.1	2.81298543	1.63E-08	1.16E-06	L2/L3-specific	L,F	Geranylgeranyl reductase (AHRD V1 ***- Q1ZYL0_OLEEU)%3B contains Interpro domain(s) IPR011774 Geranylgeranyl reductase%2C plants and cyanobacteria
Solyc03g113730.2.1	2.805592292	5.61E-06	0.000107448	L2/L3-specific	D	B12D protein (AHRD V1 ***- Q9XHD5_IPOBA)%3B contains Interpro domain(s) IPR010530 B12D
Solyc12g009960.1.1	2.800819538	1.42E-05	0.000678944	L2/L3-specific	F	Eukaryotic translation initiation factor 4 (AHRD V1 *-*- D8TN78_VOLCA)%3B contains Interpro domain(s) IPR016021 MIF4-like%2C type 1/2/3
Solyc07g065380.2.1	2.800688497	0.0019704	0.033917287	L2/L3-specific	F	Zinc transporter 2 (AHRD V1 **** B6UH65_MAIZE)%3B contains Interpro domain(s) IPR003689 Zinc/iron permease
Solyc05g007070.2.1	2.799933737	0.001751	0.030996262	L2/L3-specific	D,F	Alpha amylase 2 (IC) (AHRD V1 *-** Q010P9_OSTTA)%3B contains Interpro domain(s) IPR006589 Glycosyl hydrolase%2C family 13%2C subfamily%2C catalytic region
Solyc10g007310.1.1	2.784573245	4.97E-05	0.00191646	L2/L3-specific	F	Light-dependent short hypocotyls 1 (AHRD V1 *-*- D7M6V0_ARALY)%3B contains Interpro domain(s) IPR006936 Protein of unknown function DUF640
Solyc01g108030.2.1	2.78256247	1.87E-08	6.46E-07	L2/L3-specific	D	Methylcrotonoyl-CoA carboxylase beta subunit (AHRD V1 **** D4XQJ0_ACIHA)%3B contains Interpro domain(s) IPR000022 Carboxyl transferase
Solyc03g120640.2.1	2.77859203	2.51E-05	0.000831819	L2/L3-specific	L,D	Photosystem I reaction center subunit VI-1%2C chloroplastic (AHRD V1 ***- PSAH1_ARATH)%3B contains Interpro domain(s) IPR004928 Photosystem I reaction centre subunit VI
Solyc05g009930.2.1	2.778104448	5.32E-08	3.40E-06	L2/L3-specific	L,D,F	Chloroplast unusual positioning 1A (AHRD V1 *--- B3IYT7_ADICA)
Solyc08g016750.2.1	2.777966905	0.0005283	0.005252432	L2/L3-specific	D	Pyruvate dehydrogenase E1 component subunit beta (AHRD V1 ***- B6TQ36_MAIZE)%3B contains Interpro domain(s) IPR005475 Transketolase%2C central region
Solyc08g083330.2.1	2.777620851	4.43E-07	2.43E-05	L2/L3-specific	L,D	Membrane related protein (AHRD V1 **-- Q6ZXK5_ORYSJ)%3B contains Interpro domain(s) IPR002913 Lipid-binding START
Solyc03g096380.2.1	2.776783641	0.0024896	0.018472924	L2/L3-specific	D	Amino acid permease-like protein proline transporter-like protein (AHRD V1 **** Q9FJ34_ARATH)%3B contains Interpro domain(s) IPR013057 Amino acid transporter%2C transmembrane
Solyc12g007310.1.1	2.776595338	2.70E-06	5.72E-05	L2/L3-specific	D	Lactoylglutathione lyase (AHRD V1 **** B6SSK1_MAIZE)%3B contains Interpro domain(s) IPR004361 Glyoxalase I
Solyc02g076710.2.1	2.769546497	4.20E-06	0.000233265	L2/L3-specific	F	Cathepsin B-like cysteine proteinase (AHRD V1 *--* CYSP_SCHMA)%3B contains Interpro domain(s) IPR013128 Peptidase C1A%2C papain
Solyc02g090890.2.1	2.762437376	7.37E-09	5.82E-07	L2/L3-specific	L,D,F	Zeaxanthin epoxidase%2C chloroplastic
Solyc10g008980.2.1	2.759310014	7.05E-09	2.62E-07	L2/L3-specific	L,D	Triose phosphate/phosphate translocator (AHRD V1 **** Q9MSB6_MESCR)%3B contains Interpro domain(s) IPR004696 Tpt phosphate/phosphoenolpyruvate translocator
Solyc06g066000.1.1	2.756398645	1.19E-06	2.74E-05	L2/L3-specific	L,D	ATP synthase subunit b%26apos (AHRD V1 ***- D8G4T7_9CYAN)%3B contains Interpro domain(s) IPR002146 ATPase%2C F0 complex%2C subunit B/B'%2C bacterial and chloroplast
Solyc06g071350.2.1	2.755492468	2.99E-05	0.001271646	L2/L3-specific	F	Os02g0742100 protein (Fragment) (AHRD V1 *-*- Q0DXN7_ORYSJ)%3B contains Interpro domain(s) IPR017923 TFIIS N-terminal IPR001025 Bromo adjacent region
Solyc10g017850.2.1	2.753069402	8.58E-06	0.000156399	L2/L3-specific	D	Peroxisomal membrane protein 11-1 (AHRD V1 ***- PX111_ORYSJ)%3B contains Interpro domain(s) IPR008733 Peroxisomal biogenesis factor 11
Solyc01g067740.2.1	2.749676581	0.0001611	0.005023571	L2/L3-specific	F	Superoxide dismutase (AHRD V1 **** B1Q475_CAPCH)%3B contains Interpro domain(s) IPR001424 Superoxide dismutase%2C copper/zinc binding
Solyc03g083090.2.1	2.736707983	0.0005429	0.012752555	L2/L3-specific	F	Glycogen synthase (AHRD V1 ***- D5MWN9_BACSU)%3B contains Interpro domain(s) IPR011835 Glycogen/starch synthases%2C ADP-glucose type
Solyc05g050120.2.1	2.730434219	1.01E-06	4.98E-05	L2/L3-specific	L,D,F	Malic enzyme (AHRD V1 ***- O04936_SOLLC)%3B contains Interpro domain(s) IPR012302 Malic enzyme%2C NAD-binding
Solyc06g063070.2.1	2.728206082	1.61E-09	1.48E-07	L2/L3-specific	L,D,F	Ethylene responsive transcription factor 2a (AHRD V1 ***- C0J9I7_9ROSA)%3B contains Interpro domain(s) IPR001471 Pathogenesis-related transcriptional factor and ERF%2C DNA-binding
Solyc01g111510.2.1	2.726666779	3.05E-05	0.001280337	L2/L3-specific	F	Ascorbate peroxidase (AHRD V1 **** Q8W4V7_CAPAN)%3B contains Interpro domain(s) IPR002207 Plant ascorbate peroxidase
Solyc04g071350.2.1	2.724700363	0.0006438	0.014597971	L2/L3-specific	F	Exocyst complex component 2 (AHRD V1 ***- Q6PGY8_DANRE)
Solyc07g042180.2.1	2.718045316	0.0001879	0.005642039	L2/L3-specific	F	Polyadenylate-binding protein (AHRD V1 **** A8N215_COPC7)%3B contains Interpro domain(s) IPR000504 RNA recognition motif%2C RNP-1
Solyc12g006050.1.1	2.703462869	2.13E-06	4.61E-05	L2/L3-specific	D	Nitrate transporter (AHRD V1 ***- Q8LG02_ARATH)%3B contains Interpro domain(s) IPR000109 TGF-beta receptor%2C type I/II extracellular region
Solyc02g082900.2.1	2.701701011	0.000733	0.006883558	L2/L3-specific	D	Strictosidine synthase family protein (AHRD V1 **-- D7LTU8_ARALY)%3B contains Interpro domain(s) IPR004141 Strictosidine synthase
Solyc03g043750.2.1	2.692768434	0.0007905	0.017079131	L2/L3-specific	F	FAD-dependent pyridine nucleotide-disulphide oxidoreductase (AHRD V1 ***- A0YND6_LYNSP)%3B contains Interpro domain(s) IPR013027 FAD-dependent pyridine nucleotide-disulphide oxidoreductase
Solyc08g081190.2.1	2.692595466	9.49E-08	8.70E-06	L2/L3-specific	F	Aquaporin 1 (AHRD V1 ***- O24662_TOBAC)%3B contains Interpro domain(s) IPR012269 Aquaporin
Solyc07g022910.2.1	2.683503956	0.0007525	0.01455258	L2/L3-specific	L	Myosin heavy chain-like (AHRD V1 ***- Q8LHQ6_ORYSJ)%3B contains Interpro domain(s) IPR008545 Protein of unknown function DUF827%2C plant
Solyc01g005210.2.1	2.68142418	1.47E-08	5.19E-07	L2/L3-specific	D	Alpha alpha-trehalose-phosphate synthase (UDP-forming) (AHRD V1 **** A3CXN9_METMJ)%3B contains Interpro domain(s) IPR001830 Glycosyl transferase%2C family 20
Solyc04g081330.2.1	2.680415246	0.0001559	0.001870011	L2/L3-specific	D	Chromosome 11 contig 1 DNA sequence. (Fragment) (AHRD V1 *-*- Q00Z75_OSTTA)
Solyc07g055320.2.1	2.680264167	9.98E-09	3.58E-07	L2/L3-specific	L,D	ATP-dependent Zn protease cell division protein FtsH homolog (AHRD V1 **** B2XTF7_HETA2)%3B contains Interpro domain(s) IPR005936 Peptidase M41%2C FtsH
Solyc05g005740.2.1	2.680144868	1.66E-05	0.000272038	L2/L3-specific	D	Potassium transporter (AHRD V1 **** D2JYH2_GOSHI)%3B contains Interpro domain(s) IPR018519 Potassium uptake protein%2C kup IPR003855 K+ potassium transporter
Solyc01g005520.2.1	2.670198402	0.0001602	0.004036385	L2/L3-specific	L,D	Tetratricopeptide TPR_2 repeat protein (AHRD V1 *--- A8ZVN6_DESOH)%3B contains Interpro domain(s) IPR001478 PDZ/DHR/GLGF
Solyc06g062950.1.1	2.668866415	4.47E-09	1.73E-07	L2/L3-specific	D	Subtilisin-like protease (AHRD V1 **-- A9XG40_TOBAC)%3B contains Interpro domain(s) IPR015500 Peptidase S8%2C subtilisin-related
Solyc03g093830.2.1	2.666375542	2.97E-06	0.000130096	L2/L3-specific	L,D,F	Chaperone protein dnaJ-like (AHRD V1 **-- Q6H3Y3_ORYSJ)
Solyc04g079420.2.1	2.658344359	1.87E-05	0.000640443	L2/L3-specific	L	Nbs-lrr%2C resistance protein
Solyc01g104910.2.1	2.655429857	4.29E-07	1.11E-05	L2/L3-specific	L,D	Os11g0223400 protein (Fragment) (AHRD V1 *--- Q0ITS2_ORYSJ)
Solyc01g098610.2.1	2.652373299	0.004715	0.030059013	L2/L3-specific	D	Glutathione synthetase (AHRD V1 **** Q6F4I7_ZINEL)%3B contains Interpro domain(s) IPR005615 Glutathione synthase%2C eukaryotic
Solyc01g111350.2.1	2.651545266	9.75E-05	0.003311253	L2/L3-specific	F	Nodulin family protein (AHRD V1 ***- D7MDH3_ARALY)%3B contains Interpro domain(s) IPR010658 Nodulin-like
Solyc02g088560.2.1	2.642497899	9.92E-08	2.95E-06	L2/L3-specific	D	Cyclic nucleotide gated channel (AHRD V1 **** A9CRE4_MALDO)%3B contains Interpro domain(s) IPR000595 Cyclic nucleotide-binding
Solyc12g089220.1.1	2.635635906	7.35E-08	2.24E-06	L2/L3-specific	D	Wound responsive protein (Fragment) (AHRD V1 *--- A6N0L4_ORYSI)%3B contains Interpro domain(s) IPR003729 Protein of unknown function DUF151
Solyc07g043390.2.1	2.627483981	1.02E-05	0.000384996	L2/L3-specific	L	Cellulose synthase family protein expressed (AHRD V1 **** Q339N6_ORYSJ)%3B contains Interpro domain(s) IPR005150 Cellulose synthase
Solyc01g111570.2.1	2.618450629	0.000342	0.008870167	L2/L3-specific	F	Receptor-like kinase (AHRD V1 *-*- A7VM42_MARPO)%3B contains Interpro domain(s) IPR002290 Serine/threonine protein kinase
Solyc11g007850.1.1	2.607064137	0.0001094	0.001400083	L2/L3-specific	D	Plastid DNA-binding protein (Fragment) (AHRD V1 ***- Q5DW96_PRUYE)
Solyc01g079790.2.1	2.606684945	0.0002447	0.005753535	L2/L3-specific	L	Glucose-1-phosphate adenylyltransferase (AHRD V1 **** P93230_SOLLC)%3B contains Interpro domain(s) IPR011831 Glucose-1-phosphate adenylyltransferase
Solyc08g077430.2.1	2.602495531	4.67E-05	0.000669698	L2/L3-specific	D	Glycine-rich protein (AHRD V1 ***- D7MQL9_ARALY)
Solyc03g117430.2.1	2.598763004	0.0004241	0.009272007	L2/L3-specific	L,D	Cobalamin synthesis protein P (AHRD V1 ***- D3M8Z8_9ACTO)%3B contains Interpro domain(s) IPR003495 Cobalamin (vitamin B12) biosynthesis CobW-like
Solyc02g036470.2.1	2.596112033	0.0001231	0.001538899	L2/L3-specific	D	Drug/metabolite transporter (AHRD V1 ***- B4TLZ1_SALSV)%3B contains Interpro domain(s) IPR000620 Protein of unknown function DUF6%2C transmembrane
Solyc01g108300.2.1	2.584795273	0.0002122	0.002447357	L2/L3-specific	D	Myb family transcription factor (AHRD V1 **-- D7LQI3_ARALY)%3B contains Interpro domain(s) IPR006447 Myb-like DNA-binding region%2C SHAQKYF class
Solyc01g068460.2.1	2.581866485	0.0004209	0.010480359	L2/L3-specific	F	Pathogen-induced calmodulin-binding protein (Fragment) (AHRD V1 *-*- Q8H6W8_PHAVU)%3B contains Interpro domain(s) IPR012417 Calmodulin-binding%2C plant
Solyc01g107380.2.1	2.575878969	9.41E-06	0.000167705	L2/L3-specific	D	Seed maturation protein PM23 (Fragment) (AHRD V1 ***- Q9SEL1_SOYBN)
Solyc08g079620.2.1	2.571453303	6.55E-05	0.000897089	L2/L3-specific	D	ATP-dependent Clp protease proteolytic subunit (AHRD V1 **** A5ADV7_VITVI)%3B contains Interpro domain(s) IPR001907 Peptidase S14%2C ClpP
Solyc01g097340.2.1	2.570775111	2.59E-05	0.001130432	L2/L3-specific	D,F	NAD-dependent epimerase/dehydratase family protein-like protein (AHRD V1 ***- Q2XPW6_SOLTU)%3B contains Interpro domain(s) IPR016040 NAD(P)-binding domain
Solyc12g010060.1.1	2.570733985	0.0001995	0.005898016	L2/L3-specific	L,F	Eukaryotic translation initiation factor 5A (AHRD V1 ***- Q207T7_GYMCO)%3B contains Interpro domain(s) IPR019769 Translation elongation factor%2C IF5A%2C hypusine site
Solyc08g075530.2.1	2.570360256	1.70E-05	0.000277414	L2/L3-specific	D	Alpha glucosidase-like protein (AHRD V1 **** Q93Y12_ARATH)%3B contains Interpro domain(s) IPR000322 Glycoside hydrolase%2C family 31
Solyc11g010500.1.1	2.56909435	0.0008472	0.016001894	L2/L3-specific	L	Mitochondrial carrier family (AHRD V1 ***- C1MMX1_MICPS)%3B contains Interpro domain(s) IPR001993 Mitochondrial substrate carrier
Solyc02g071280.2.1	2.566643425	0.0001177	0.001482514	L2/L3-specific	D	Ribosome maturation factor rimM (AHRD V1 *-*- A4CTC3_SYNPV)%3B contains Interpro domain(s) IPR011961 16S rRNA processing protein RimM
Solyc10g081510.1.1	2.563002771	9.51E-09	1.11E-06	L2/L3-specific	F	5-methyltetrahydropteroyltriglutamate--homocysteine methyltransferase (AHRD V1 ***- B6UF55_MAIZE)%3B contains Interpro domain(s) IPR006276 5-methyltetrahydropteroyltriglutamate--homocysteine S-methyltransferase
Solyc01g010480.2.1	2.557190293	2.74E-06	5.78E-05	L2/L3-specific	L,D	Potassium voltage-gated channel subfamily H member 8 (AHRD V1 *--- KCNH8_HUMAN)%3B contains Interpro domain(s) IPR013767 PAS fold

Solyc02g030170.2.1	2.552760374	2.71E-07	7.33E-06	L2/L3-specific	D	FAD linked oxidase domain protein (AHRD V1 **-- D1VPW7_9ACTO)%3B contains Interpro domain(s) IPR006094 FAD linked oxidase%2C N-terminal
Solyc05g017990.2.1	2.551717085	1.37E-05	0.000230673	L2/L3-specific	D	tRNA/rRNA methyltransferase (AHRD V1 *-** D9V4V2_9ACTO)%3B contains Interpro domain(s) IPR001537 tRNA/rRNA methyltransferase%2C SpoU
Solyc03g119450.2.1	2.54505595	1.22E-05	0.000209791	L2/L3-specific	D	Chloroplastic group IIA intron splicing facilitator CRS1%2C chloroplastic (AHRD V1 **-- CRS1_ARATH)%3B contains Interpro domain(s) IPR001890 RNA-binding%2C CRM domain
Solyc11g069380.1.1	2.53259519	2.08E-05	0.000707024	L2/L3-specific	L,D	4-hydroxy-3-methylbut-2-en-1-yl diphosphate synthase (AHRD V1 ***- A9ZN14_HEVBR)%3B contains Interpro domain(s) IPR017178 4-hydroxy-3-methylbut-2-en-1-yl diphosphate synthase%2C atypical
Solyc03g065340.2.1	2.531080616	0.0006484	0.006223448	L2/L3-specific	D	Phosphorylase (AHRD V1 **** B9SJB6_RICCO)%3B contains Interpro domain(s) IPR011833 Glycogen/starch/alpha-glucan phosphorylase
Solyc03g007600.2.1	2.527999616	5.01E-05	0.000710298	L2/L3-specific	D	Pentatricopeptide repeat-containing protein At4g21190 (AHRD V1 ***- PP332_ARATH)%3B contains Interpro domain(s) IPR002885 Pentatricopeptide repeat
Solyc06g073320.2.1	2.524205942	1.01E-08	7.66E-07	L2/L3-specific	L,F	GDP-L-galactose phosphorylase 1
Solyc07g054080.1.1	2.523629646	4.08E-07	1.06E-05	L2/L3-specific	D	RING finger protein 5 (AHRD V1 *--- B6TI40_MAIZE)%3B contains Interpro domain(s) IPR018957 Zinc finger%2C C3HC4 RING-type
Solyc02g065680.2.1	2.508883454	8.04E-06	0.000408641	L2/L3-specific	L,D,F	Amino acid transporter (AHRD V1 ***- B9N1V7_POPTR)%3B contains Interpro domain(s) IPR013057 Amino acid transporter%2C transmembrane
Solyc06g050980.2.1	2.507541192	0.0001201	0.003150158	L2/L3-specific	L	Ferritin (AHRD V1 ***- Q308A9_SOLTU)%3B contains Interpro domain(s) IPR001519 Ferritin%2C N-terminal
Solyc03g114030.2.1	2.505094489	2.93E-06	0.000174591	L2/L3-specific	F	Nucleobase ascorbate transporter (AHRD V1 **-* B9I369_POPTR)%3B contains Interpro domain(s) IPR006043 Xanthine/uracil/vitamin C permease
Solyc02g066950.2.1	2.50358119	2.28E-05	0.000362437	L2/L3-specific	D	Alpha amylase 2 (IC) (AHRD V1 *-*- Q010P9_OSTTA)%3B contains Interpro domain(s) IPR006589 Glycosyl hydrolase%2C family 13%2C subfamily%2C catalytic region
Solyc04g082630.2.1	2.502903495	3.68E-07	2.86E-05	L2/L3-specific	L,F	Glyceraldehyde-3-phosphate dehydrogenase B (AHRD V1 **** B4F8L7_MAIZE)%3B contains Interpro domain(s) IPR000173 Glyceraldehyde 3-phosphate dehydrogenase
Solyc06g035970.2.1	2.502037968	0.0006311	0.014359158	L2/L3-specific	F	Tubulin beta chain (AHRD V1 ***- B9GKJ5_POPTR)%3B contains Interpro domain(s) IPR002453 Beta tubulin
Solyc01g108600.2.1	2.49334113	1.67E-06	8.01E-05	L2/L3-specific	L	Presequence protease%2C mitochondrial (AHRD V1 **-- PREP_DANRE)%3B contains Interpro domain(s) IPR013578 Peptidase M16C associated
Solyc03g031720.2.1	2.490065937	0.0001423	0.00451747	L2/L3-specific	F	RNA Binding Protein 45 (AHRD V1 **** Q9LEB4_NICPL)%3B contains Interpro domain(s) IPR000504 RNA recognition motif%2C RNP-1
Solyc06g036260.2.1	2.487611639	0.000324	0.00348322	L2/L3-specific	D	Beta-carotene hydroxylase 1
Solyc12g009470.1.1	2.486506223	3.06E-07	8.19E-06	L2/L3-specific	L,D	Heme oxygenase 1 (AHRD V1 **** Q94FW7_SOLLC)%3B contains Interpro domain(s) IPR016951 Haem oxygenase (decyclizing)%2C plant
Solyc08g083320.2.1	2.479256143	2.74E-06	0.000121554	L2/L3-specific	L	Granule-bound starch synthase (AHRD V1 **** B0ZTE3_SOLTU)%3B contains Interpro domain(s) IPR011835 Glycogen/starch synthases%2C ADP-glucose type
Solyc09g009220.2.1	2.476543428	0.0009223	0.008317782	L2/L3-specific	D	Unknown Protein (AHRD V1)
Solyc07g065980.2.1	2.47551872	1.65E-07	4.65E-06	L2/L3-specific	D	Alkaline alpha galactosidase I (AHRD V1 **-- Q3Y5A3_CUCSA)%3B contains Interpro domain(s) IPR008811 Raffinose synthase
Solyc04g015970.2.1	2.471650503	0.0001129	0.001438563	L2/L3-specific	L,D	ATP-binding cassette 1 (AHRD V1 *-*- Q80ZB2_RAT)%3B contains Interpro domain(s) IPR003439 ABC transporter-like
Solyc11g010380.1.1	2.464524773	0.0002371	0.002694498	L2/L3-specific	D	Mate efflux family protein (AHRD V1 **-- D7MN36_ARALY)%3B contains Interpro domain(s) IPR002528 Multi antimicrobial extrusion protein MatE
Solyc04g051850.2.1	2.462147446	8.01E-06	0.000147373	L2/L3-specific	D	Unknown Protein (AHRD V1)
Solyc06g065020.2.1	2.45103228	1.93E-07	5.35E-06	L2/L3-specific	D	Peptide transporter (AHRD V1 **-* A6YJX5_9MAGN)%3B contains Interpro domain(s) IPR000109 TGF-beta receptor%2C type I/II extracellular region
Solyc12g019750.1.1	2.448740651	0.0001013	0.001310263	L2/L3-specific	D	Polypyrimidine tract binding protein 1 (AHRD V1 **-- Q9BUQ0_HUMAN)%3B contains Interpro domain(s) IPR012677 Nucleotide-binding%2C alpha-beta plait
Solyc09g090100.2.1	2.447824633	4.09E-05	0.000596367	L2/L3-specific	D	Cryptochrome 2
Solyc05g007020.2.1	2.444355858	0.0001522	0.003883763	L2/L3-specific	L	Potassium voltage-gated channel subfamily H member 1 (AHRD V1 *--- KCNH1_RAT)%3B contains Interpro domain(s) IPR013655 PAS fold-3
Solyc05g007120.2.1	2.440186682	7.63E-06	0.000394965	L2/L3-specific	D,F	Receptor like kinase%2C RLK
Solyc01g107870.2.1	2.439301634	1.20E-07	1.06E-05	L2/L3-specific	D,F	Poly(A) RNA binding protein (AHRD V1 **** B2CJ74_9HYPO)%3B contains Interpro domain(s) IPR006515 Polyadenylate binding protein%2C human types 1%2C 2%2C 3%2C 4
Solyc07g041150.2.1	2.437739591	0.0009617	0.019723043	L2/L3-specific	F	Myosin XI (AHRD V1 ***- Q5R216_TOBAC)%3B contains Interpro domain(s) IPR001609 Myosin head%2C motor region
Solyc09g092430.2.1	2.437657023	4.53E-08	1.44E-06	L2/L3-specific	D	Selenium binding protein (AHRD V1 **** Q93WS1_MEDSA)%3B contains Interpro domain(s) IPR008826 Selenium-binding protein
Solyc05g056540.2.1	2.436807523	0.0039349	0.026178776	L2/L3-specific	D	Alcohol dehydrogenase-like protein (AHRD V1 **** Q8LDF7_ARATH)%3B contains Interpro domain(s) IPR002085 Alcohol dehydrogenase superfamily%2C zinc-containing
Solyc03g083910.2.1	2.435376043	9.25E-07	2.19E-05	L2/L3-specific	D	Acid beta-fructofuranosidase (AHRD V1 **-- Q575T1_WHEAT)%3B contains Interpro domain(s) IPR001362 Glycoside hydrolase%2C family 32
Solyc11g073250.1.1	2.409751256	0.003473	0.023856536	L2/L3-specific	D	Histone H2A (AHRD V1 ***- C6TMV8_SOYBN)%3B contains Interpro domain(s) IPR002119 Histone H2A
Solyc12g099000.1.1	2.407360764	7.95E-05	0.002812154	L2/L3-specific	F	S-adenosylmethionine synthase (AHRD V1 ***- B8LFH4_IPOBA)%3B contains Interpro domain(s) IPR002133 S-adenosylmethionine synthetase
Solyc07g041970.2.1	2.400115455	2.96E-05	0.001259502	L2/L3-specific	F	Subtilisin-like protease (AHRD V1 **-- B6SZ82_MAIZE)%3B contains Interpro domain(s) IPR015500 Peptidase S8%2C subtilisin-related
Solyc11g068950.1.1	2.392969657	6.13E-06	0.000328305	L2/L3-specific	F	BEL1-like homeodomain protein 1 (AHRD V1 **-- BLH1_ARATH)%3B contains Interpro domain(s) IPR006563 POX
Solyc05g018650.2.1	2.389018122	2.11E-06	4.59E-05	L2/L3-specific	D	Ferrochelatase (AHRD V1 **** O64391_SOLTU)%3B contains Interpro domain(s) IPR001015 Ferrochelatase
Solyc03g058970.2.1	2.385852411	2.42E-06	5.18E-05	L2/L3-specific	D,F	Alkaline alpha galactosidase (AHRD V1 **-- B5UAS6_TETTT)%3B contains Interpro domain(s) IPR008811 Raffinose synthase
Solyc03g097370.2.1	2.374665867	0.000106	0.002865966	L2/L3-specific	L,F	DNA helicase-like (AHRD V1 **** O9FGC2_ARATH)%3B contains Interpro domain(s) IPR003593 ATPase%2C AAA+ type%2C core
Solyc04g077020.2.1	2.372814488	1.66E-07	4.67E-06	L2/L3-specific	D	Tubulin alpha-3 chain (AHRD V1 ***- B6SPX4_MAIZE)%3B contains Interpro domain(s) IPR002452 Alpha tubulin
Solyc07g007060.1.1	2.37179818	3.92E-05	0.000574491	L2/L3-specific	D	Zinc transporter (AHRD V1 **** Q947R8_EUCGR)%3B contains Interpro domain(s) IPR002524 Cation efflux protein
Solyc01g099690.2.1	2.354729657	3.00E-06	6.23E-05	L2/L3-specific	D	ATP-dependent Clp protease proteolytic subunit (AHRD V1 ***- B9RFA9_RICCO)%3B contains Interpro domain(s) IPR001907 Peptidase S14%2C ClpP
Solyc09g007560.2.1	2.344448597	0.001086	0.019467277	L2/L3-specific	L	50S ribosomal protein L5 (AHRD V1 ***- B5TV71_CAMSI)%3B contains Interpro domain(s) IPR002132 Ribosomal protein L5
Solyc09g014790.2.1	2.339338718	2.85E-05	0.000922057	L2/L3-specific	L	Class E vacuolar protein-sorting machinery protein hse1 (AHRD V1 *--- HSE1_EMENI)%3B contains Interpro domain(s) IPR018205 VHS subgroup
Solyc07g007330.1.1	2.334498449	2.44E-05	0.000383373	L2/L3-specific	L,D	Serine-rich protein (AHRD V1 ***- A1YMW6_BRACM)
Solyc05g010450.1.1	2.33384378	0.0020709	0.015936415	L2/L3-specific	D	Unknown Protein (AHRD V1)
Solyc01g095430.2.1	2.329102537	0.0009574	0.008584887	L2/L3-specific	L,D	Threonine endopeptidase (AHRD V1 **-- B6TCN7_MAIZE)
Solyc05g010810.1.1	2.327635046	9.32E-05	0.003197645	L2/L3-specific	F	Phosphatidylinositol kinase (AHRD V1 *-** D0MQD8_PHYIN)%3B contains Interpro domain(s) IPR000403 Phosphatidylinositol 3- and 4-kinase%2C catalytic
Solyc11g040370.1.1	2.317449941	0.000901	0.01878542	L2/L3-specific	F	Far upstream element-binding protein 3 (AHRD V1 *-*- B5X224_SALSA)%3B contains Interpro domain(s) IPR018111 K Homology%2C type 1%2C subgroup
Solyc04g025040.1.1	2.310900839	0.000383	0.008509336	L2/L3-specific	L,D	Unknown Protein (AHRD V1)
Solyc08g077880.2.1	2.310815416	0.0002069	0.005011409	L2/L3-specific	L	Light harvesting-like protein 3 (AHRD V1 **-- B9N2V9_POPTR)
Solyc02g082760.2.1	2.310623205	5.75E-06	0.000231699	L2/L3-specific	L,D	Catalase (AHRD V1 **** O24511_TOBAC)%3B contains Interpro domain(s) IPR018028 Catalase related subgroup
Solyc09g075020.2.1	2.304803413	5.03E-06	0.000208115	L2/L3-specific	L,F	Multidrug resistance protein ABC transporter family (AHRD V1 **-- B9GS96_POPTR)%3B contains Interpro domain(s) IPR001140 ABC transporter%2C transmembrane region
Solyc01g088370.2.1	2.298974809	0.000196	0.005831605	L2/L3-specific	F	Eukaryotic translation initiation factor 3 subunit B (AHRD V1 **** B2VZ72_PYRTR)%3B contains Interpro domain(s) IPR011400 Translation initiation factor eIF-3b
Solyc10g075030.1.1	2.291589268	0.0014069	0.011688504	L2/L3-specific	D	AP2 domain-containing transcription factor (AHRD V1 *--* B9HWL7_POPTR)%3B contains Interpro domain(s) IPR003340 Transcriptional factor B3
Solyc02g081190.2.1	2.28731147	1.55E-05	0.000255474	L2/L3-specific	D	1-aminocyclopropane-1-carboxylate oxidase (AHRD V1 ***- Q9ZWP2_SOLLC)%3B contains Interpro domain(s) IPR005123 Oxoglutarate and iron-dependent oxygenase
Solyc08g075210.1.1	2.274322869	0.0002434	0.006828003	L2/L3-specific	F	Acyltransferase-like protein (AHRD V1 **-- Q589X7_TOBAC)%3B contains Interpro domain(s) IPR003480 Transferase
Solyc12g006980.1.1	2.268022771	7.48E-06	0.00038859	L2/L3-specific	F	Leucine-rich repeat receptor-like protein kinase PEPR2 (AHRD V1 *--* PEPR2_ARATH)%3B contains Interpro domain(s) IPR001611 Leucine-rich repeat
Solyc02g081170.2.1	2.264052947	9.26E-06	0.000352111	L2/L3-specific	L	Plastid-lipid-associated protein%2C chloroplastic (AHRD V1 **-- PAP_CITUN)%3B contains Interpro domain(s) IPR006843 PAP fibrillin
Solyc01g007220.2.1	2.257662077	0.0003843	0.009637718	L2/L3-specific	F	NHL1 (Fragment) (AHRD V1 **-- B0LZM3_ARATH)%3B contains Interpro domain(s) IPR010847 Harpin-induced 1
Solyc03g059310.2.1	2.247563692	0.0032801	0.022862126	L2/L3-specific	D	WD-40 repeat family protein (AHRD V1 **-- Q5D6Y8_GOSHI)%3B contains Interpro domain(s) IPR020472 G-protein beta WD-40 repeat%2C region
Solyc02g078140.2.1	2.240183735	0.0007134	0.013932562	L2/L3-specific	L	Protein kinase (AHRD V1 ***- Q39886_SOYBN)%3B contains Interpro domain(s) IPR002290 Serine/threonine protein kinase
Solyc07g062970.2.1	2.238876878	3.11E-05	0.000474788	L2/L3-specific	D	Serine/threonine phosphatase family protein (AHRD V1 **** C1E0J1_9CHLO)%3B contains Interpro domain(s) IPR015655 Protein phosphatase 2C
Solyc05g053210.2.1	2.237874712	0.0011248	0.020032058	L2/L3-specific	L	CBL-interacting protein kinase 1 (AHRD V1 **** A0MNI9_POPTR)%3B contains Interpro domain(s) IPR002290 Serine/threonine protein kinase
Solyc05g008200.2.1	2.236766229	0.0008066	0.017290784	L2/L3-specific	F	Phosphatidylinositol-4-phosphate 5-kinase family protein (AHRD V1 *--- D7L328_ARALY)%3B contains Interpro domain(s) IPR016034 Phosphatidylinositol-4-phosphate 5-kinase%2C core%2C subgroup
Solyc02g085350.2.1	2.232738023	3.28E-05	0.001357773	L2/L3-specific	F	Succinate dehydrogenase flavoprotein subunit (AHRD V1 **** B8CCT7_THAPS)%3B contains Interpro domain(s) IPR011281 Succinate dehydrogenase%2C flavoprotein subunit
Solyc03g019880.2.1	2.21300647	0.0018218	0.014410199	L2/L3-specific	L,D	UPF0426 protein At1g28150%2C chloroplastic (AHRD V1 ***- Y1815_ARATH)
Solyc02g072130.2.1	2.212407364	2.70E-05	0.001163721	L2/L3-specific	F	Protein transport protein SEC61 alpha subunit (AHRD V1 ***- D3B1K5_POLPA)%3B contains Interpro domain(s) IPR002208 SecY protein
Solyc08g059660.1.1	2.211195202	7.08E-05	0.002583333	L2/L3-specific	F	Phosphatidylinositol binding clathrin assembly protein like (AHRD V1 *--* Q6AXI5_DANRE)%3B contains Interpro domain(s) IPR011417 ANTH
Solyc01g099770.2.1	2.208123801	5.45E-07	4.02E-05	L2/L3-specific	F	Translationally-controlled tumor protein homolog (AHRD V1 ***- B5XDL0_SALSA)%3B contains Interpro domain(s) IPR001983 Translationally controlled tumour-associated TCTP
Solyc12g089180.1.1	2.207396032	0.0001003	0.001302574	L2/L3-specific	D	Glucose transporter 8 (AHRD V1 ***- Q2KKJ3_SOLIN)%3B contains Interpro domain(s) IPR003663 Sugar/inositol transporter
Solyc01g091730.2.1	2.200334486	0.0028012	0.040671479	L2/L3-specific	L	Peroxisomal membrane protein 11-5 (AHRD V1 ***- PX115_ORYSJ)%3B contains Interpro domain(s) IPR008733 Peroxisomal biogenesis factor 11
Solyc07g006860.2.1	2.196100777	0.0020184	0.015610414	L2/L3-specific	D	Xyloglucan endotransglucosylase/hydrolase 3 (AHRD V1 **** Q06BI4_CUCME)%3B contains Interpro domain(s) IPR016455 Xyloglucan endotransglucosylase/hydrolase
Solyc07g066600.2.1	2.180107705	0.0011076	0.021787065	L2/L3-specific	F	Phosphoglycerate kinase (AHRD V1 ***- Q2VCK2_SOLTU)%3B contains Interpro domain(s) IPR001576 Phosphoglycerate kinase
Solyc02g070550.2.1	2.176926196	1.94E-05	0.000314429	L2/L3-specific	D	La-related protein 6 (AHRD V1 *--- LARP6_HUMAN)%3B contains Interpro domain(s) IPR006630 RNA-binding protein Lupus La
Solyc01g095620.2.1	2.167763281	5.63E-06	0.000227908	L2/L3-specific	L	UDP-glucosyltransferase (AHRD V1 **-* B8QI32_9MAGN)%3B contains Interpro domain(s) IPR002213 UDP-glucuronosyl/UDP-glucosyltransferase
Solyc01g089870.2.1	2.166857369	0.000635	0.006107033	L2/L3-specific	D	Pentatricopeptide repeat-containing protein (AHRD V1 ***- D7KP29_ARALY)%3B contains Interpro domain(s) IPR002885 Pentatricopeptide repeat
Solyc01g100380.2.1	2.163556994	5.11E-06	0.000279412	L2/L3-specific	F	Calreticulin 2 calcium-binding protein (AHRD V1 **** A8HMC0_CHLRE)%3B contains Interpro domain(s) IPR009169 Calreticulin
Solyc04g054810.2.1	2.148998066	2.58E-05	0.00112937	L2/L3-specific	F	Pollen allergen Phl p 11 (AHRD V1 ***- B6T2Z8_MAIZE)%3B contains Interpro domain(s) IPR006041 Pollen Ole e 1 allergen and extensin
Solyc10g083640.1.1	2.148815742	0.0005207	0.01236835	L2/L3-specific	F	Splicing factor U2af subunit (AHRD V1 *-*- B6T3Q5_MAIZE)%3B contains Interpro domain(s) IPR009145 U2 auxiliary factor small subunit
Solyc08g080690.2.1	2.147538782	0.00026	0.00289098	L2/L3-specific	D	Methyl binding domain protein (AHRD V1 *-*- B9I4C0_POPTR)%3B contains Interpro domain(s) IPR001739 Methyl-CpG DNA binding
Solyc05g005490.2.1	2.136280672	5.15E-06	9.95E-05	L2/L3-specific	D	Carbonic anhydrase (AHRD V1 ***- Q5NE21_SOLLC)%3B contains Interpro domain(s) IPR015892 Carbonic anhydrase%2C prokaryotic-like%2C conserved site
Solyc03g006140.2.1	2.135358786	3.30E-05	0.00049916	L2/L3-specific	D	Neutral ceramidase (AHRD V1 **** A9YFM2_WHEAT)%3B contains Interpro domain(s) IPR006823 Neutral/alkaline nonlysosomal ceramidase
Solyc12g089160.1.1	2.13467021	4.60E-05	0.000662439	L2/L3-specific	D,F	Receptor like kinase%2C RLK
Solyc04g077050.2.1	2.131385286	0.000858	0.007835039	L2/L3-specific	D	Amino acid permease 6 (AHRD V1 **** Q7Y076_BRANA)%3B contains Interpro domain(s) IPR013057 Amino acid transporter%2C transmembrane
Solyc07g063390.2.1	2.128369891	0.0002844	0.003126883	L2/L3-specific	D	Beta-glucosidase (AHRD V1 **** B6SYQ7_MAIZE)%3B contains Interpro domain(s) IPR001360 Glycoside hydrolase%2C family 1
Solyc03g097790.2.1	2.128276031	0.0001426	0.004518507	L2/L3-specific	F	V-type proton ATPase subunit C (AHRD V1 **** VATC_ARATH)%3B contains Interpro domain(s) IPR004907 ATPase%2C V1 complex%2C subunit C
Solyc08g082530.2.1	2.121037636	0.0007806	0.007257106	L2/L3-specific	D	Cell division protease ftsH homolog (AHRD V1 *-*- FTSH_PORPU)%3B contains Interpro domain(s) IPR003959 ATPase%2C AAA-type%2C core
Solyc09g014520.2.1	2.113324931	6.71E-06	0.000266892	L2/L3-specific	L,D,F	Chlorophyll a-b binding protein 6A%2C chloroplastic (AHRD V1 **** CB11_SOLLC)%3B contains Interpro domain(s) IPR001344 Chlorophyll A-B binding protein
Solyc10g078550.1.1	2.103328432	0.0001053	0.001356494	L2/L3-specific	D	Glutamate dehydrogenase (AHRD V1 **** Q8L6A0_TOBAC)%3B contains Interpro domain(s) IPR014362 Glutamate dehydrogenase
Solyc09g008240.2.1	2.096381627	0.0005674	0.011681277	L2/L3-specific	L	Uncharacterized ABC transporter ATP-binding protein/permease C9B6.09c (AHRD V1 *--* YNT9_SCHPO)%3B contains Interpro domain(s) IPR003439 ABC transporter-like

Solyc05g055910.2.1	2.096135589	0.0002084	0.002415298	L2/L3-specific	D	Genomic DNA chromosome 5 P1 clone MRH10 (AHRD V1 **-- Q9FND1_ARATH)%3B contains Interpro domain(s) IPR009291 Protein of unknown function DUF946%2C plant
Solyc02g069570.2.1	2.092159351	0.0071271	0.041621139	L2/L3-specific	D	Pentatricopeptide repeat-containing protein (AHRD V1 *-*- D7LDK6_ARALY)%3B contains Interpro domain(s) IPR002885 Pentatricopeptide repeat
Solyc12g043110.1.1	2.088127694	0.0002215	0.00632993	L2/L3-specific	F	Heat shock protein 4 (AHRD V1 ***- B6U237_MAIZE)%3B contains Interpro domain(s) IPR013126 Heat shock protein 70
Solyc02g093620.1.1	2.078330562	0.0036189	0.048887639	L2/L3-specific	L,D	Pentatricopeptide repeat-containing protein (AHRD V1 ***- D7M6I3_ARALY)%3B contains Interpro domain(s) IPR002885 Pentatricopeptide repeat
Solyc05g008530.2.1	2.077487517	0.0007257	0.0140742	L2/L3-specific	L	Sec-independent protein translocase TatC (AHRD V1 ***- Q3MG73_ANAVT)%3B contains Interpro domain(s) IPR019822 Twin arginine-targeting protein translocase%2C TatC
Solyc08g079070.2.1	2.076343923	7.08E-05	0.000965041	L2/L3-specific	D	Transaldolase (AHRD V1 **** D6N3G8_GOSHI)%3B contains Interpro domain(s) IPR001585 Transaldolase
Solyc03g114340.2.1	2.063478183	0.0005084	0.005079375	L2/L3-specific	D	1-deoxy-D-xylulose-5-phosphate reductoisomerase (AHRD V1 **** Q947C3_SOLLC)%3B contains Interpro domain(s) IPR003821 1-deoxy-D-xylulose 5-phosphate reductoisomerase
Solyc09g090330.2.1	2.062260589	0.0011848	0.01015836	L2/L3-specific	D,F	Harpin binding protein 1 (AHRD V1 **-- Q5QJB4_SOLLC)%3B contains Interpro domain(s) IPR006843 PAP fibrillin
Solyc07g062530.2.1	2.061138799	9.31E-06	0.000467859	L2/L3-specific	D,F	Phosphoenolpyruvate carboxylase 2 (AHRD V1 ***- Q9SCB2_SOLLC)%3B contains Interpro domain(s) IPR015813 Pyruvate/Phosphoenolpyruvate kinase%2C catalytic core IPR001449 Phosphoenolpyruvate carboxylase
Solyc06g068450.1.1	2.060179229	0.0003222	0.003468041	L2/L3-specific	D	CBL-interacting protein kinase (AHRD V1 **** A5YAI9_GOSHI)%3B contains Interpro domain(s) IPR002290 Serine/threonine protein kinase
Solyc10g081650.1.1	2.056659887	0.0003519	0.003738831	L2/L3-specific	D	Carotenoid isomerase%2C chloroplastic
Solyc08g081250.2.1	2.056181761	5.44E-05	0.001627713	L2/L3-specific	L,F	Aminopeptidase N (AHRD V1 ***- D5C0C8_NITHN)%3B contains Interpro domain(s) IPR012779 Peptidase M1%2C alanyl aminopeptidase
Solyc01g103010.2.1	2.055965439	0.0014745	0.027447637	L2/L3-specific	F	Cullin-associated NEDD8-dissociated protein 2 (AHRD V1 ***- B0WPV6_CULQU)%3B contains Interpro domain(s) IPR011989 Armadillo-like helical
Solyc01g089970.2.1	2.055855709	0.0003603	0.009221722	L2/L3-specific	F	Nucleoside diphosphate kinase (AHRD V1 ***- Q3Y5A4_SOLCH)%3B contains Interpro domain(s) IPR001564 Nucleoside diphosphate kinase%2C core
Solyc03g116480.1.1	2.047891347	0.0022215	0.01687145	L2/L3-specific	D	F-box family protein (AHRD V1 **-- B9GZP5_POPTR)%3B contains Interpro domain(s) IPR015915 Kelch-type beta propeller
Solyc12g014100.1.1	2.042202116	2.62E-05	0.000407487	L2/L3-specific	D	Homogentisate 1 2-dioxygenase (AHRD V1 ***- D7MTP0_ARALY)%3B contains Interpro domain(s) IPR005708 Homogentisate 1%2C2-dioxygenase
Solyc05g013380.2.1	2.03464633	0.0028537	0.04120378	L2/L3-specific	L	Alanine aminotransferase 2 (AHRD V1 **** A8IKE5_SOYBN)%3B contains Interpro domain(s) IPR004839 Aminotransferase%2C class I and II
Solyc11g012130.1.1	2.026582219	0.0015948	0.012938906	L2/L3-specific	D,F	Blue copper protein (AHRD V1 *--- B6U7P6_MAIZE)%3B contains Interpro domain(s) IPR008972 Cupredoxin
Solyc01g080460.2.1	2.019300124	3.03E-05	0.000974342	L2/L3-specific	L,D,F	Pyruvate phosphate dikinase (AHRD V1 ***- Q84N32_SORBI)%3B contains Interpro domain(s) IPR010121 Pyruvate%2C phosphate dikinase
Solyc12g005860.1.1	2.013481705	7.25E-05	0.002624699	L2/L3-specific	D,F	3-isopropylmalate dehydratase large subunit (AHRD V1 *--- LEUC_BACWK)%3B contains Interpro domain(s) IPR006249 Aconitase/iron regulatory protein 2
Solyc09g098040.2.1	2.005362207	5.03E-06	9.74E-05	L2/L3-specific	D	Phosphoglucan water dikinase (AHRD V1 *-** A8J6C3_CHLRE)%3B contains Interpro domain(s) IPR013783 Immunoglobulin-like fold
Solyc04g074300.2.1	1.996167716	0.0001917	0.005728547	L2/L3-specific	F	30S ribosomal protein S5 (AHRD V1 ***- C5K1T7_AJEDS)%3B contains Interpro domain(s) IPR005711 Ribosomal protein S5%2C eukaryotic/archaeal
Solyc01g008070.2.1	1.988052615	1.63E-05	0.000266994	L2/L3-specific	D	Alpha/beta superfamily hydrolase (AHRD V1 *--- D0CIF5_9SYNE)%3B contains Interpro domain(s) IPR010765 Protein of unknown function DUF1350
Solyc09g091030.2.1	1.98068207	3.78E-06	7.64E-05	L2/L3-specific	D,F	Beta-amylase (AHRD V1 **** E0AE02_SOLLC)%3B contains Interpro domain(s) IPR013781 Glycoside hydrolase%2C subgroup%2C catalytic core
Solyc03g117590.2.1	1.967984399	0.0009601	0.008597524	L2/L3-specific	D	Chaperone protein dnaJ (AHRD V1 *-*- C5UZI2_CLOBO)%3B contains Interpro domain(s) IPR001623 Heat shock protein DnaJ%2C N-terminal
Solyc09g008920.2.1	1.962526851	0.0002136	0.002454605	L2/L3-specific	D	Geranylgeranyl pyrophosphate synthase (AHRD V1 ***- Q0WUL9_ARATH)%3B contains Interpro domain(s) IPR000092 Polyprenyl synthetase
Solyc03g111310.2.1	1.96105598	9.47E-06	0.000168459	L2/L3-specific	D	AKIN gamma (AHRD V1 **-- Q7XYY0_MEDTR)%3B contains Interpro domain(s) IPR000644 Cystathionine beta-synthase%2C core
Solyc01g088170.2.1	1.959330034	0.0066721	0.039775756	L2/L3-specific	D	Aldehyde oxidase (AHRD V1 ***- Q9FV24_SOLLC)%3B contains Interpro domain(s) IPR002346 Molybdopterin dehydrogenase%2C FAD-binding
Solyc02g087760.2.1	1.956585806	0.001955	0.015260534	L2/L3-specific	D	Calmodulin binding protein (AHRD V1 **-* B6U6R2_MAIZE)%3B contains Interpro domain(s) IPR000048 IQ calmodulin-binding region
Solyc02g078120.1.1	1.938370769	0.0010781	0.021311725	L2/L3-specific	F	Eukaryotic translation initiation factor 3 subunit 7 (AHRD V1 ***- Q9FKV6_ARATH)%3B contains Interpro domain(s) IPR007783 Eukaryotic translation initiation factor 3%2C subunit 7
Solyc06g076750.2.1	1.935473327	0.0028009	0.020232088	L2/L3-specific	D	Peptide transporter (AHRD V1 **-* O48542_HORVU)%3B contains Interpro domain(s) IPR000109 TGF-beta receptor%2C type I/II extracellular region
Solyc01g103450.2.1	1.933831942	1.13E-05	0.000195267	L2/L3-specific	L,D,F	Chaperone DnaK (AHRD V1 ***- Q1SKX2_MEDTR)%3B contains Interpro domain(s) IPR012725 Chaperone DnaK
Solyc01g109090.2.1	1.931055504	0.0001208	0.001515126	L2/L3-specific	D	Unknown Protein (AHRD V1)
Solyc05g013720.2.1	1.927297418	0.0002102	0.002432267	L2/L3-specific	D	Alpha-galactosidase (AHRD V1 *-*- D4LCA1_9FIRM)%3B contains Interpro domain(s) IPR013785 Aldolase-type TIM barrel
Solyc01g103990.2.1	1.915381149	0.0003568	0.009150635	L2/L3-specific	F	T-complex protein 11 (AHRD V1 ***- D7KML1_ARALY)%3B contains Interpro domain(s) IPR008862 T-complex 11
Solyc04g054480.2.1	1.912834133	8.12E-05	0.001080914	L2/L3-specific	D,F	C2 domain-containing protein-like (AHRD V1 *-*- Q67UI5_ORYSJ)%3B contains Interpro domain(s) IPR011989 Armadillo-like helical
Solyc11g009080.1.1	1.907750758	0.0005932	0.012175802	L2/L3-specific	L	Phospho-2-dehydro-3-deoxyheptonate aldolase 1 (AHRD V1 ***- B6TUB8_MAIZE)%3B contains Interpro domain(s) IPR002480 DAHP synthetase%2C class II
Solyc05g015610.2.1	1.905145393	5.25E-05	0.000739046	L2/L3-specific	D	Histidine kinase cytokinin receptor (AHRD V1 **** B9GZP2_POPTR)%3B contains Interpro domain(s) IPR006189 CHASE
Solyc06g070980.2.1	1.893178257	0.0003033	0.008167041	L2/L3-specific	F	Ubiquitin-conjugating enzyme E2 2 (AHRD V1 **** UBC2_MEDSA)%3B contains Interpro domain(s) IPR019775 WD40 repeat%2C conserved site IPR000608 Ubiquitin-conjugating enzyme%2C E2
Solyc03g111640.2.1	1.891251978	0.0001211	0.001518215	L2/L3-specific	D	Eukaryotic translation initiation factor 4 gamma 2 (Fragment) (AHRD V1 *--- A3KQB2_DANRE)%3B contains Interpro domain(s) IPR003891 Initiation factor eIF-4 gamma%2C MA3
Solyc01g065980.2.1	1.888640836	6.55E-05	0.002433907	L2/L3-specific	D,F	Ethylene responsive transcription factor 2b (AHRD V1 *-** C0J9I6_9ROSA)%3B contains Interpro domain(s) IPR001471 Pathogenesis-related transcriptional factor and ERF%2C DNA-binding
Solyc01g007070.2.1	1.879481706	0.0002373	0.006685757	L2/L3-specific	F	BEL1-like homeodomain protein 1 (AHRD V1 **-- BLH1_ARATH)%3B contains Interpro domain(s) IPR006563 POX
Solyc03g111630.2.1	1.877848249	0.0001497	0.001799388	L2/L3-specific	D	Programmed cell death 4a (AHRD V1 *--- D3TLD2_GLOMM)%3B contains Interpro domain(s) IPR003891 Initiation factor eIF-4 gamma%2C MA3
Solyc09g065910.1.1	1.873121714	0.0028669	0.041349643	L2/L3-specific	L,D	Photosystem II reaction center W protein (AHRD V1 ***- B6TMB3_MAIZE)%3B contains Interpro domain(s) IPR009806 Photosystem II protein PsbW%2C class 2
Solyc01g008110.2.1	1.871261837	0.0003267	0.007366619	L2/L3-specific	L,F	Cytochrome P450
Solyc05g005080.2.1	1.87052475	0.0001019	0.003425418	L2/L3-specific	F	Endo-1 4-beta-glucanase (AHRD V1 ***- O04890_SOLLC)%3B contains Interpro domain(s) IPR001701 Glycoside hydrolase%2C family 9
Solyc08g076090.2.1	1.864823899	0.0043219	0.027976509	L2/L3-specific	D	At1g32160/F3C3_6 (AHRD V1 ***- Q9FVR1_ARATH)%3B contains Interpro domain(s) IPR008479 Protein of unknown function DUF760
Solyc11g011990.1.1	1.863521317	0.0002546	0.002851631	L2/L3-specific	D	Plastid terminal oxydase
Solyc06g060250.2.1	1.853192026	0.0013905	0.011600129	L2/L3-specific	D	Aldehyde dehydrogenase family protein expressed (AHRD V1 **** Q53NG8_ORYSJ)%3B contains Interpro domain(s) IPR012394 Aldehyde dehydrogenase NAD(P)-dependent IPR015590 Aldehyde dehydrogenase
Solyc06g073540.2.1	1.847408003	0.0009794	0.019958819	L2/L3-specific	F	Argonaute 4-like protein (AHRD V1 ***- D5FQ91_PELHO)%3B contains Interpro domain(s) IPR003165 Stem cell self-renewal protein Piwi
Solyc03g046340.2.1	1.837578389	0.0013642	0.011428502	L2/L3-specific	D	Cell division protease ftsH homolog 4 (AHRD V1 *-*- FTSH4_SYNY3)%3B contains Interpro domain(s) IPR003959 ATPase%2C AAA-type%2C core
Solyc05g050280.2.1	1.827512116	0.0007201	0.00678482	L2/L3-specific	D	GH3 family protein (AHRD V1 ***- B9GQG9_POPTR)%3B contains Interpro domain(s) IPR004993 GH3 auxin-responsive promoter
Solyc07g017610.2.1	1.81937284	3.88E-05	0.00056893	L2/L3-specific	D	Saccharopine dehydrogenase (NAD(+) L-glutamate-forming) (AHRD V1 *-** C7PUW7_CHIPD)%3B contains Interpro domain(s) IPR005097 Saccharopine dehydrogenase
Solyc07g053280.2.1	1.811433835	0.0002053	0.006018881	L2/L3-specific	F	Ketol-acid reductoisomerase (AHRD V1 ***- D6QSY0_CATRO)%3B contains Interpro domain(s) IPR016206 Ketol-acid reductoisomerase%2C plant
Solyc02g082920.2.1	1.799309487	7.79E-05	0.002232388	L2/L3-specific	L	Endochitinase (Chitinase) (AHRD V1 **** Q43184_SOLTU)%3B contains Interpro domain(s) IPR000726 Glycoside hydrolase%2C family 19%2C catalytic
Solyc01g009420.2.1	1.793261548	0.0003983	0.004142919	L2/L3-specific	D	Bifunctional polymyxin resistance arnA protein (AHRD V1 ***- B6TQB1_MAIZE)%3B contains Interpro domain(s) IPR001509 NAD-dependent epimerase/dehydratase
Solyc08g006890.2.1	1.775925061	5.66E-05	0.000788484	L2/L3-specific	D	Tubulin alpha-3 chain (AHRD V1 ***- B6SPX4_MAIZE)%3B contains Interpro domain(s) IPR002452 Alpha tubulin
Solyc09g074110.2.1	1.74457989	0.0014591	0.012049432	L2/L3-specific	D	AGAP009276-PA (Fragment) (AHRD V1 *--- Q7PVH5_ANOGA)
Solyc03g117890.2.1	1.736439747	0.000983	0.018117833	L2/L3-specific	L	Unknown protein DS12 from 2D-PAGE of leaf%2C chloroplastic (AHRD V1 **-- UP12_ORYSJ)
Solyc10g081460.1.1	1.718004202	0.0008624	0.007853927	L2/L3-specific	D	Amino acid transporter (AHRD V1 ***- C3GEP6_BACTU)%3B contains Interpro domain(s) IPR015606 Cationic amino acid transporter
Solyc01g104170.2.1	1.700554764	0.0009929	0.02014944	L2/L3-specific	F	Ankyrin repeat domain-containing protein 2 (AHRD V1 ***- B6TNC0_MAIZE)%3B contains Interpro domain(s) IPR002110 Ankyrin
Solyc01g103710.2.1	1.689896741	0.0019815	0.015385053	L2/L3-specific	L,D	Fe/S biogenesis protein nfuA (AHRD V1 *--- NFUA_BUCCC)%3B contains Interpro domain(s) IPR001075 NIF system FeS cluster assembly%2C NifU%2C C-terminal
Solyc04g051510.1.1	1.649406017	0.0016917	0.013566673	L2/L3-specific	D	Receptor like kinase%2C RLK
Solyc07g044840.2.1	1.64294919	0.0029424	0.045153199	L2/L3-specific	F	2 3-bisphosphoglycerate-independent phosphoglycerate mutase (AHRD V1 **** B9S1V6_RICCO)%3B contains Interpro domain(s) IPR005995 Phosphoglycerate mutase%2C 2%2C3-bisphosphoglycerate-independent
Solyc01g097770.2.1	1.628007651	0.0027206	0.039967779	L2/L3-specific	L	Serine/threonine protein kinase (AHRD V1 *-** D2V878_NAEGR)%3B contains Interpro domain(s) IPR002290 Serine/threonine protein kinase
Solyc08g081760.1.1	1.594412464	0.0015627	0.028547245	L2/L3-specific	F	Os11g0223400 protein (Fragment) (AHRD V1 *--- Q0ITS2_ORYSJ)
Solyc07g008880.2.1	1.592705671	0.0019006	0.032922173	L2/L3-specific	F	Pre-mRNA-processing-splicing factor 8 (AHRD V1 ***- D0NFV2_PHYIN)%3B contains Interpro domain(s) IPR012592 PROCN
Solyc01g103540.2.1	1.566480208	0.0002137	0.002454605	L2/L3-specific	D	YTH domain family 2 (Predicted) (AHRD V1 *-*- B2GUU1_RAT)%3B contains Interpro domain(s) IPR007275 YT521-B-like protein
Solyc01g111760.2.1	1.540692687	0.0009478	0.008514864	L2/L3-specific	D	V-type ATP synthase beta chain (AHRD V1 ***- VATB_PYRHO)%3B contains Interpro domain(s) IPR005723 ATPase%2C V1 complex%2C subunit B
Solyc02g086880.2.1	1.523739496	0.0004416	0.004522746	L2/L3-specific	D	Formate dehydrogenase (AHRD V1 **** Q5NE18_SOLLC)%3B contains Interpro domain(s) IPR006140 D-isomer specific 2-hydroxyacid dehydrogenase%2C NAD-binding
Solyc03g078400.2.1	1.498495665	0.0011854	0.02290446	L2/L3-specific	F	Actin (AHRD V1 ***- Q7XZJ2_GOSHI)%3B contains Interpro domain(s) IPR004000 Actin/actin-like
Solyc04g007980.2.1	1.487746346	0.0022848	0.017220094	L2/L3-specific	D	1-aminocyclopropane-1-carboxylate oxidase (AHRD V1 **-* B6SUN2_MAIZE)%3B contains Interpro domain(s) IPR005123 Oxoglutarate and iron-dependent oxygenase
Solyc12g042950.1.1	1.432919509	0.0039235	0.026115188	L2/L3-specific	D	ADP/ATP carrier protein (AHRD V1 ***- Q5L657_CHLAB)%3B contains Interpro domain(s) IPR004667 ADP/ATP carrier protein
Solyc07g065840.2.1	1.429662843	0.0025743	0.041166047	L2/L3-specific	F	Heat shock protein 90 (AHRD V1 ***- Q14TB1_TOBAC)%3B contains Interpro domain(s) IPR003594 ATP-binding region%2C ATPase-like IPR001404 Heat shock protein Hsp90
Solyc04g017690.2.1	1.42601536	0.0008054	0.007445345	L2/L3-specific	D	Early response to dehydration 15-like protein (Fragment) (AHRD V1 *--- Q5MEE1_PSEMZ)%3B contains Interpro domain(s) IPR009818 Ataxin-2%2C C-terminal
Solyc05g054760.2.1	1.291548413	0.0084502	0.047662907	L2/L3-specific	D,F	Dehydroascorbate reductase (Fragment) (AHRD V1 ***- Q1G0W3_SOLLC)%3B contains Interpro domain(s) IPR017933 Glutathione S-transferase/chloride channel%2C C-terminal
Solyc01g067660.2.1	1.205656542	0.0056052	0.034471774	L2/L3-specific	D	Beta-amylase (AHRD V1 **** Q5F304_SOYBN)%3B contains Interpro domain(s) IPR013781 Glycoside hydrolase%2C subgroup%2C catalytic core
Solyc07g007750.2.1	8.674603246	2.06E-15	2.07E-13	L2/L3-related	L,D	Defensin protein (AHRD V1 ***- B1N678_SOLLC)%3B contains Interpro domain(s) IPR008177 Gamma Purothionin
Solyc05g009310.2.1	8.075279041	2.41E-16	2.88E-14	L2/L3-related	D	Zinc finger protein CONSTANS-LIKE 16 (AHRD V1 **-- B6TEI1_MAIZE)%3B contains Interpro domain(s) IPR010402 CCT domain
Solyc08g005960.1.1	7.457766704	5.47E-13	3.90E-11	L2/L3-related	D	Cortical cell-delineating protein (AHRD V1 *--- B6U436_MAIZE)%3B contains Interpro domain(s) IPR013770 Plant lipid transfer protein and hydrophobic protein%2C helical
Solyc06g036290.2.1	7.448164913	2.76E-13	7.02E-11	L2/L3-related	F	Heat shock protein 90 (Fragment) (AHRD V1 **-- Q00NU9_WHEAT)%3B contains Interpro domain(s) IPR003594 ATP-binding region%2C ATPase-like IPR001404 Heat shock protein Hsp90
Solyc01g095080.2.1	7.427951194	1.64E-09	2.19E-07	L2/L3-related	F	1-aminocyclopropane-1-carboxylate synthase (AHRD V1 **** O65209_NICGU)%3B contains Interpro domain(s) IPR004839 Aminotransferase%2C class I and II IPR004838 Aminotransferases%2C class-I%2C pyridoxal-phosphate-binding site
Solyc02g076690.2.1	7.077178574	6.64E-11	3.39E-09	L2/L3-related	D	Cathepsin B-like cysteine proteinase (AHRD V1 **-* CYSP_SCHMA)%3B contains Interpro domain(s) IPR013128 Peptidase C1A%2C papain
Solyc08g061630.2.1	7.042598342	6.13E-11	3.17E-09	L2/L3-related	D	Conserved hypothetical membrane protein (AHRD V1 *--- Q7V1G3_PROMP)%3B contains Interpro domain(s) IPR003425 Protein of unknown function YGGT
Solyc06g071750.2.1	6.660709548	3.93E-10	5.68E-08	L2/L3-related	F	Octicosapeptide/Phox/Bem1p domain-containing protein (AHRD V1 **-- D7LRK8_ARALY)%3B contains Interpro domain(s) IPR000270 Octicosapeptide/Phox/Bem1p
Solyc08g078700.2.1	6.600168006	1.42E-09	1.90E-07	L2/L3-related	F	Heat shock protein 22 (AHRD V1 ***- Q27JQ0_METAN)%3B contains Interpro domain(s) IPR002068 Heat shock protein Hsp20
Solyc10g009580.2.1	6.502329058	4.28E-08	1.37E-06	L2/L3-related	L,D	UDP-glucosyltransferase (AHRD V1 **-* B8QI32_9MAGN)%3B contains Interpro domain(s) IPR002213 UDP-glucuronosyl/UDP-glucosyltransferase
Solyc03g121540.2.1	6.490490549	7.78E-29	3.73E-26	L2/L3-related	D	Beta-galactosidase (AHRD V1 ***- Q9ZP11_SOLLC)%3B contains Interpro domain(s) IPR019801 Glycoside hydrolase%2C family 35%2C conserved site IPR001944 Glycoside hydrolase%2C family 35 IPR000922 D-galactoside/L-rhamnose binding SUEL lectin
Solyc04g082010.1.1	6.414018472	2.69E-19	4.62E-17	L2/L3-related	D	Plastocyanin (AHRD V1 ***- Q0W9E5_SOLCO)%3B contains Interpro domain(s) IPR000923 Blue (type 1) copper domain
Solyc02g068310.2.1	6.393253943	2.16E-06	4.67E-05	L2/L3-related	D	2-oxoglutarate-dependent dioxygenase (AHRD V1 **-* B9GJL0_POPTR)%3B contains Interpro domain(s) IPR005123 Oxoglutarate and iron-dependent oxygenase
Solyc03g005020.2.1	6.381026285	8.96E-20	5.59E-17	L2/L3-related	F	Lipase (AHRD V1 **** Q1XBG1_RICCO)%3B contains Interpro domain(s) IPR006693 AB-hydrolase associated lipase region
Solyc09g084490.2.1	6.329324664	9.23E-09	3.34E-07	L2/L3-related	D	Proteinase inhibitor I (AHRD V1 ***- Q53X28_SOLTU)%3B contains Interpro domain(s) IPR000864 Proteinase inhibitor I13%2C potato inhibitor I
Solyc01g005000.2.1	6.292450081	1.48E-10	7.27E-09	L2/L3-related	D	Glutamate decarboxylase (AHRD V1 ***- Q1I1D8_CITSI)%3B contains Interpro domain(s) IPR010107 Glutamate decarboxylase

Solyc07g049530.2.1	6.276856031	2.02E-12	4.69E-10	L2/L3-related	F	1-aminocyclopropane-1-carboxylate oxidase (AHRD V1 ***- Q94F66_SOLTU)%3B contains Interpro domain(s) IPR005123 Oxoglutarate and iron-dependent oxygenase
Solyc12g099190.1.1	6.261237796	8.12E-11	1.34E-08	L2/L3-related	F	Invertase inhibitor (AHRD V1 ***- C1KBP2_SOLTU)%3B contains Interpro domain(s) IPR006501 Pectinesterase inhibitor
Solyc02g078150.2.1	6.167949736	2.42E-14	2.14E-12	L2/L3-related	D	Plant-specific domain TIGR01615 family protein (AHRD V1 *-*- B6UDN7_MAIZE)%3B contains Interpro domain(s) IPR006502 Protein of unknown function DUF506%2C plant
Solyc01g102860.2.1	6.092285466	1.75E-06	3.89E-05	L2/L3-related	D	Unknown Protein (AHRD V1)
Solyc12g010920.1.1	6.001399706	0.000913	0.019006723	L2/L3-related	F	Oleosin (AHRD V1 ***- Q8LGL8_OLEEU)%3B contains Interpro domain(s) IPR000136 Oleosin
Solyc12g096100.1.1	5.93745381	5.26E-06	0.000101089	L2/L3-related	D	ABC-type Co2+ transport system permease component (AHRD V1 *--- B6TSG4_MAIZE)
Solyc02g090680.2.1	5.92663496	1.49E-08	5.23E-07	L2/L3-related	D	Cyclin-dependent kinase inhibitor 7 (AHRD V1 *-** KRP7_ARATH)%3B contains Interpro domain(s) IPR016701 Cyclin-dependent kinase inhibitor%2C plant
Solyc10g086180.1.1	5.896161673	8.03E-12	1.72E-09	L2/L3-related	F	Phenylalanine ammonia-lyase (AHRD V1 ***- D2CFQ1_9SOLA)%3B contains Interpro domain(s) IPR005922 Phenylalanine ammonia-lyase
Solyc07g055720.2.1	5.808009913	1.30E-06	2.98E-05	L2/L3-related	D	Heat shock protein Hsp20 (AHRD V1 *-*- D4H6X0_DENA2)%3B contains Interpro domain(s) IPR008978 HSP20-like chaperone
Solyc10g086570.2.1	5.662962889	5.14E-15	5.06E-13	L2/L3-related	D	Palmitoyltransferase PFA4 (AHRD V1 **-- B6TA04_MAIZE)%3B contains Interpro domain(s) IPR001594 Zinc finger%2C DHHC-type
Solyc12g008980.1.1	5.596416893	6.63E-11	3.39E-09	L2/L3-related	D	Lycopene epsilon cyclase
Solyc09g066150.1.1	5.585617492	7.21E-10	3.12E-08	L2/L3-related	D	Cytochrome P450
Solyc12g044420.1.1	5.57018299	1.25E-07	3.62E-06	L2/L3-related	D	Avr9/Cf-9 rapidly elicited protein 146 (AHRD V1 **-- Q9FQZ6_TOBAC)
Solyc08g074630.1.1	5.444413799	1.91E-13	1.49E-11	L2/L3-related	L,D	Polyphenol oxidase (AHRD V1 ***- Q41428_SOLTU)%3B contains Interpro domain(s) IPR016213 Polyphenol oxidase%2C plant
Solyc05g006970.2.1	5.426611167	4.89E-08	1.55E-06	L2/L3-related	D	Small multidrug export protein (AHRD V1 *-*- D0GL52_9FUSO)%3B contains Interpro domain(s) IPR009577 Putative small multi-drug export
Solyc08g014130.2.1	5.378214695	4.10E-12	9.07E-10	L2/L3-related	F	2-isopropylmalate synthase 1 (AHRD V1 **** Q30DX9_9BRAS)%3B contains Interpro domain(s) IPR005671 Bacterial 2-isopropylmalate synthase
Solyc06g007990.2.1	5.357630033	3.07E-05	0.000469939	L2/L3-related	D	Unknown Protein (AHRD V1)
Solyc02g082070.2.1	5.280335337	3.85E-07	1.01E-05	L2/L3-related	D	Cytochrome P450
Solyc11g066250.1.1	5.258801771	6.98E-07	5.02E-05	L2/L3-related	F	Serine carboxypeptidase (AHRD V1 **** Q9XH61_9ASTR)%3B contains Interpro domain(s) IPR001563 Peptidase S10%2C serine carboxypeptidase
Solyc10g050430.1.1	5.224102863	3.87E-06	7.81E-05	L2/L3-related	D	Plant viral-response family protein (AHRD V1 **-- B6SQA7_MAIZE)%3B contains Interpro domain(s) IPR006904 Protein of unknown function DUF716
Solyc12g098600.1.1	5.208829372	4.06E-12	2.49E-10	L2/L3-related	D	UDP-glucosyltransferase family 1 protein (AHRD V1 ***- C6KI44_CITSI)%3B contains Interpro domain(s) IPR002213 UDP-glucuronosyl/UDP-glucosyltransferase
Solyc02g055440.2.1	5.19920067	1.15E-06	2.65E-05	L2/L3-related	D	Genomic DNA chromosome 3 P1 clone MPN9 (AHRD V1 ***- Q9LT28_ARATH)
Solyc04g076740.2.1	5.158558686	3.42E-08	1.12E-06	L2/L3-related	D	Tic20-like protein (AHRD V1 ***- Q2V9A0_SOLTU)%3B contains Interpro domain(s) IPR005691 Chloroplast protein import component Tic20
Solyc07g049660.2.1	5.149361634	1.64E-10	2.53E-08	L2/L3-related	F	Acetyl coenzyme A cis-3-hexen-1-ol acetyl transferase (AHRD V1 **-* Q9SRQ2_ARATH)%3B contains Interpro domain(s) IPR003480 Transferase
Solyc02g091140.2.1	5.138795431	5.40E-09	2.05E-07	L2/L3-related	D	S-adenosyl-L-methionine carboxyl methyltransferase family protein (AHRD V1 **** D7KVS6_ARALY)%3B contains Interpro domain(s) IPR005299 SAM dependent carboxyl methyltransferase
Solyc12g088760.1.1	5.125200089	2.74E-06	5.78E-05	L2/L3-related	D	Subtilisin-like protease (AHRD V1 **-- Q38708_ALNGL)%3B contains Interpro domain(s) IPR015500 Peptidase S8%2C subtilisin-related
Solyc10g085140.1.1	5.049638956	1.01E-06	4.98E-05	L2/L3-related	L	Undecaprenyl pyrophosphate synthase (AHRD V1 ***- Q5QXS4_IDILO)%3B contains Interpro domain(s) IPR001441 Di-trans-poly-cis-decaprenylcistransferase-like
Solyc05g007780.2.1	5.005082703	1.86E-05	0.000302457	L2/L3-related	L,D	Oxygen evolving enhancer protein 3 (AHRD V1 ***- B6SZC8_MAIZE)%3B contains Interpro domain(s) IPR008797 Photosystem II oxygen evolving complex protein PsbQ
Solyc12g008560.1.1	4.961701357	1.49E-09	6.13E-08	L2/L3-related	D	Unknown Protein (AHRD V1)
Solyc08g080490.2.1	4.955439901	1.47E-15	5.04E-13	L2/L3-related	F	2S albumin seed storage protein (Fragment) (AHRD V1 *-*- Q7Y1C2_JUGNI)%3B contains Interpro domain(s) IPR003612 Plant lipid transfer protein/seed storage/trypsin-alpha amylase inhibitor
Solyc04g009960.2.1	4.920568632	7.86E-05	0.002787105	L2/L3-related	F	L-allo-threonine aldolase (AHRD V1 **** D7RJ69_CARPA)%3B contains Interpro domain(s) IPR001597 Aromatic amino acid beta-eliminating lyase/threonine aldolase
Solyc01g109630.2.1	4.914287165	1.17E-05	0.000202778	L2/L3-related	D	mTERF domain-containing protein 3%2C mitochondrial (AHRD V1 *--- MTER3_HUMAN)%3B contains Interpro domain(s) IPR003690 Mitochodrial transcription termination factor-related
Solyc02g093290.2.1	4.89396718	1.51E-05	0.000249932	L2/L3-related	D	Nicotinate phosphoribosyltransferase-like protein (AHRD V1 **** Q6XQM5_MEDTR)%3B contains Interpro domain(s) IPR006405 Nicotinate phosphoribosyltransferase putative
Solyc03g044010.2.1	4.889674624	1.11E-06	7.59E-05	L2/L3-related	F	Mitochondrial porin (Voltage-dependent anion channel) outer membrane protein (AHRD V1 **** C4R1Z2_PICPG)%3B contains Interpro domain(s) IPR001925 Porin%2C eukaryotic type
Solyc06g007160.2.1	4.889412496	6.15E-05	0.000850726	L2/L3-related	D	NADH dehydrogenase (AHRD V1 **** Q38A31_9TRYP)%3B contains Interpro domain(s) IPR013027 FAD-dependent pyridine nucleotide-disulphide oxidoreductase
Solyc09g014900.2.1	4.870226861	2.63E-06	0.00015985	L2/L3-related	F	Cytochrome P450
Solyc07g043490.1.1	4.784366125	7.71E-11	1.31E-08	L2/L3-related	F	UDP-glucosyltransferase family 1 protein (AHRD V1 **-* C6KI43_CITSI)%3B contains Interpro domain(s) IPR002213 UDP-glucuronosyl/UDP-glucosyltransferase
Solyc09g092500.1.1	4.759198804	3.75E-06	0.000214972	L2/L3-related	F	UDP-glucosyltransferase family 1 protein (AHRD V1 **** C6KI44_CITSI)%3B contains Interpro domain(s) IPR002213 UDP-glucuronosyl/UDP-glucosyltransferase
Solyc03g081260.2.1	4.753789522	2.50E-05	0.000391089	L2/L3-related	D	Subtilisin-like protease-like protein (AHRD V1 **-- Q9T0B5_ARATH)%3B contains Interpro domain(s) IPR015500 Peptidase S8%2C subtilisin-related
Solyc12g042500.1.1	4.753419646	7.17E-05	0.002601685	L2/L3-related	F	Gibberellin-regulated family protein (AHRD V1 **-- D7MT35_ARALY)%3B contains Interpro domain(s) IPR003854 Gibberellin regulated protein
Solyc09g074610.2.1	4.717048975	1.95E-05	0.000316396	L2/L3-related	D	Chaperone protein dnaJ (AHRD V1 *-*- B7GKC9_ANOFW)%3B contains Interpro domain(s) IPR015609 Molecular chaperone%2C heat shock protein%2C Hsp40%2C DnaJ
Solyc02g090490.2.1	4.699696485	1.63E-05	0.000571048	L2/L3-related	L	Patatin-like protein 3 (AHRD V1 ***- Q9FZ08_TOBAC)%3B contains Interpro domain(s) IPR002641 Patatin
Solyc04g078200.2.1	4.691969473	2.42E-11	1.34E-09	L2/L3-related	D	Gibberellin-regulated family protein (AHRD V1 **-- D7MT35_ARALY)%3B contains Interpro domain(s) IPR003854 Gibberellin regulated protein
Solyc03g093360.2.1	4.684627498	0.0003374	0.003604144	L2/L3-related	D	Wound/stress protein (AHRD V1 **-- Q672Q3_SOLLC)%3B contains Interpro domain(s) IPR001024 Lipoxygenase%2C LH2
Solyc04g054340.1.1	4.675705802	0.0001684	0.001994874	L2/L3-related	D	Unknown Protein (AHRD V1)
Solyc11g011840.1.1	4.604544773	4.23E-05	0.000613474	L2/L3-related	D	Oxidoreductase family protein (AHRD V1 ***- D7LXC3_ARALY)%3B contains Interpro domain(s) IPR016040 NAD(P)-binding domain
Solyc04g082030.1.1	4.575707768	2.14E-05	0.000722684	L2/L3-related	L,F	Ornithine decarboxylase (AHRD V1 **** Q9SXF3_TOBAC)%3B contains Interpro domain(s) IPR002433 Ornithine decarboxylase
Solyc11g005150.1.1	4.574121853	4.71E-05	0.000674356	L2/L3-related	D	Leucine-rich repeat family protein (AHRD V1 *--- D7M406_ARALY)%3B contains Interpro domain(s) IPR006706 Extensin-like region
Solyc01g110000.2.1	4.559774277	5.14E-21	1.08E-18	L2/L3-related	D	Beta-galactosidase (AHRD V1 ***- B9H0V1_POPTR)%3B contains Interpro domain(s) IPR001944 Glycoside hydrolase%2C family 35
Solyc08g083350.2.1	4.557887315	2.20E-05	0.000350936	L2/L3-related	D	50S ribosomal protein L11 (AHRD V1 ***- B6U1J2_MAIZE)%3B contains Interpro domain(s) IPR006519 Ribosomal protein L11%2C bacterial-type
Solyc01g108800.2.1	4.546663857	4.14E-05	0.000602568	L2/L3-related	D	3-hydroxyisobutyryl-CoA hydrolase (AHRD V1 **** D3NUG5_AZOS1)%3B contains Interpro domain(s) IPR001753 Crotonase%2C core
Solyc01g101100.2.1	4.534317537	0.0002781	0.003064931	L2/L3-related	D	Receptor-like protein kinase (AHRD V1 ***- Q9LYS5_ARATH)%3B contains Interpro domain(s) IPR002290 Serine/threonine protein kinase
Solyc05g053100.2.1	4.510490245	4.69E-10	2.09E-08	L2/L3-related	D	Dihydrolipoyl dehydrogenase (AHRD V1 ***- B9RZN2_RICCO)%3B contains Interpro domain(s) IPR006258 Dihydrolipoamide dehydrogenase
Solyc03g098440.2.1	4.482193103	5.03E-11	2.64E-09	L2/L3-related	L,D	Small glutamine-rich tetratricopeptide repeat-containing protein A (AHRD V1 *--- C1BM93_OSMMO)%3B contains Interpro domain(s) IPR011990 Tetratricopeptide-like helical
Solyc03g122350.2.1	4.443917895	9.12E-05	0.002529011	L2/L3-related	L	Cytochrome P450
Solyc01g104690.2.1	4.437453747	8.99E-10	3.87E-08	L2/L3-related	D	Unknown Protein (AHRD V1)
Solyc06g008920.2.1	4.421302961	3.96E-05	0.001584287	L2/L3-related	F	Acyl-CoA synthetase/AMP-acid ligase II (AHRD V1 **** D0C359_9GAMM)%3B contains Interpro domain(s) IPR000873 AMP-dependent synthetase and ligase
Solyc01g103510.2.1	4.411178743	4.40E-05	0.001734082	L2/L3-related	F	Ribosomal protein L3-like (AHRD V1 ***- Q2VCJ2_SOLTU)%3B contains Interpro domain(s) IPR000597 Ribosomal protein L3
Solyc10g006030.2.1	4.401829837	2.30E-08	7.80E-07	L2/L3-related	D	30S ribosomal protein S10-like (AHRD V1 ***- A1YMX4_BRACM)%3B contains Interpro domain(s) IPR005731 Ribosomal protein S10%2C bacterial
Solyc10g083960.1.1	4.393455001	0.000151	0.001813452	L2/L3-related	D	Phosphoadenosine phosphosulfate reductase domain containing protein (AHRD V1 *--- C5PEQ4_COCP7)%3B contains Interpro domain(s) IPR002500 Phosphoadenosine phosphosulphate reductase
Solyc03g006360.2.1	4.389916515	3.44E-14	3.01E-12	L2/L3-related	D	Auxin-repressed protein (AHRD V1 ***- Q6PXE1_9SOLN)%3B contains Interpro domain(s) IPR008406 Dormancyauxin associated
Solyc06g019200.2.1	4.38368249	1.09E-06	2.55E-05	L2/L3-related	D	Peptidase M50 family protein (AHRD V1 ***- D7LZ04_ARALY)%3B contains Interpro domain(s) IPR008915 Peptidase M50
Solyc05g006110.2.1	4.372674739	7.11E-05	0.000967915	L2/L3-related	D	Unknown Protein (AHRD V1)
Solyc07g043160.1.1	4.342839627	6.29E-13	4.41E-11	L2/L3-related	D	UDP-glucosyltransferase (AHRD V1 **** A7M6U9_IPONI)%3B contains Interpro domain(s) IPR002213 UDP-glucuronosyl/UDP-glucosyltransferase
Solyc06g069570.2.1	4.339640409	0.0001251	0.00325401	L2/L3-related	L	SNAP25 homologous protein SNAP33 (AHRD V1 **-- SNP33_ARATH)%3B contains Interpro domain(s) IPR000727 Target SNARE coiled-coil region
Solyc01g080680.2.1	4.337874738	0.0008897	0.008076985	L2/L3-related	D	Glucose transporter 8 (AHRD V1 ***- Q2KKJ3_SOLIN)%3B contains Interpro domain(s) IPR003663 Sugar/inositol transporter
Solyc07g006000.2.1	4.321400064	0.0001292	0.001592371	L2/L3-related	D	50S ribosomal protein L35 (AHRD V1 *-*- D7LGR4_ARALY)%3B contains Interpro domain(s) IPR001706 Ribosomal protein L35
Solyc01g074030.2.1	4.288742437	1.80E-14	1.63E-12	L2/L3-related	D	Beta-glucosidase 01 (AHRD V1 ***- B5M9E4_SOLLC)%3B contains Interpro domain(s) IPR013781 Glycoside hydrolase%2C subgroup%2C catalytic core
Solyc12g006020.1.1	4.287317788	0.0002173	0.005226369	L2/L3-related	L	LRR receptor-like serine/threonine-protein kinase%2C RLP
Solyc07g055050.2.1	4.287312838	3.61E-10	1.63E-08	L2/L3-related	D	ATP synthase I-like protein (AHRD V1 **-- A8HNA8_CHLRE)%3B contains Interpro domain(s) IPR008413 ATPase%2C F0 complex%2C subunit I%2C bacillus-type
Solyc12g014150.1.1	4.263970926	0.0008192	0.007549519	L2/L3-related	D	Single-stranded nucleic acid binding R3H domain protein (AHRD V1 **-- C7QND6_CYAP0)%3B contains Interpro domain(s) IPR003959 ATPase%2C AAA-type%2C core
Solyc03g025230.2.1	4.262461916	0.0001183	0.00311111	L2/L3-related	L,D	Multidrug resistance protein mdtK (AHRD V1 *--- MDTK_YERE8)%3B contains Interpro domain(s) IPR002528 Multi antimicrobial extrusion protein MatE
Solyc07g062490.1.1	4.259727808	1.71E-14	1.57E-12	L2/L3-related	D	S-locus-specific glycoprotein (AHRD V1 **-- SLSG0_BRAOA)%3B contains Interpro domain(s) IPR001480 Curculin-like (mannose-binding) lectin
Solyc07g053360.2.1	4.240765206	3.02E-05	0.001277494	L2/L3-related	F	Seed biotin-containing protein SBP65 (AHRD V1 *--- SBP65_PEA)%3B contains Interpro domain(s) IPR004238 Late embryogenesis abundant protein
Solyc02g078380.2.1	4.238079026	3.14E-10	1.44E-08	L2/L3-related	L,D	Aluminum-induced protein-like protein (AHRD V1 **-- Q8S2R9_THEHA)
Solyc08g080320.2.1	4.237335799	0.0003069	0.003329452	L2/L3-related	D	BCL-2 binding anthanogene-1 (AHRD V1 **** B4FV61_MAIZE)%3B contains Interpro domain(s) IPR003103 Apoptosis regulator Bcl-2 protein%2C BAG
Solyc06g073180.2.1	4.224478546	4.58E-07	3.47E-05	L2/L3-related	F	Zinc finger protein CONSTANS-LIKE 1 (AHRD V1 *--- COL1_ARATH)%3B contains Interpro domain(s) IPR000315 Zinc finger%2C B-box
Solyc02g087840.2.1	4.20562694	0.0003104	0.003357205	L2/L3-related	D	Homeobox-leucine zipper-like protein (AHRD V1 *-*- Q3HRT1_PICGL)%3B contains Interpro domain(s) IPR001356 Homeobox
Solyc07g043500.1.1	4.203472143	8.13E-05	0.002850986	L2/L3-related	F	UDP-glucosyltransferase (AHRD V1 ***- Q8LKG3_STERE)%3B contains Interpro domain(s) IPR002213 UDP-glucuronosyl/UDP-glucosyltransferase
Solyc03g111050.2.1	4.202060325	0.0001329	0.001626907	L2/L3-related	D	Plastid RNA-binding protein (AHRD V1 **-- D6QX33_NICBE)%3B contains Interpro domain(s) IPR003029 Ribosomal protein S1%2C RNA binding domain
Solyc01g094790.2.1	4.199009014	3.50E-22	8.39E-20	L2/L3-related	D,F	Cysteine synthase (AHRD V1 ***- Q9FS29_SOLTU)%3B contains Interpro domain(s) IPR005859 Cysteine synthase A
Solyc10g007930.2.1	4.187344313	0.0001999	0.002323452	L2/L3-related	D	Cytochrome P450
Solyc08g007400.2.1	4.168759557	3.85E-09	1.50E-07	L2/L3-related	D	Autophagy-related protein 8 (AHRD V1 **-- C1EJ88_9CHLO)%3B contains Interpro domain(s) IPR004241 Light chain 3 (LC3)
Solyc11g071640.1.1	4.16123282	4.08E-06	8.15E-05	L2/L3-related	D	Beta-D-glucosidase (AHRD V1 ***- O82074_TROMA)%3B contains Interpro domain(s) IPR001764 Glycoside hydrolase%2C family 3%2C N-terminal
Solyc01g108190.2.1	4.146733251	0.0001334	0.001631776	L2/L3-related	L,D	Calmodulin-like protein (AHRD V1 ***- Q67TZ4_ORYSJ)%3B contains Interpro domain(s) IPR011992 EF-Hand type
Solyc06g019170.2.1	4.124906888	0.0001789	0.002114487	L2/L3-related	D	Gamma-glutamyl phosphate reductase (AHRD V1 ***- D3TQ43_GLOMM)%3B contains Interpro domain(s) IPR005766 Delta l-pyrroline-5-carboxylate synthetase
Solyc09g074270.2.1	4.077453946	2.04E-05	0.00091632	L2/L3-related	F	Acetyl esterase (AHRD V1 *--- AES_ECO81)%3B contains Interpro domain(s) IPR013094 Alpha/beta hydrolase fold-3
Solyc01g056780.2.1	4.071325848	4.94E-06	9.61E-05	L2/L3-related	D	50S ribosomal protein L34 (AHRD V1 *-*- D7KDQ0_ARALY)%3B contains Interpro domain(s) IPR000271 Ribosomal protein L34
Solyc07g005550.2.1	4.041972415	0.000154	0.004823033	L2/L3-related	F	Unknown Protein (AHRD V1)
Solyc03g122120.2.1	4.036121085	0.0001282	0.004118767	L2/L3-related	F	3-oxoacyl-(Acyl-carrier-protein) synthase 2 (AHRD V1 **** D0ZZB8_CHLPP)%3B contains Interpro domain(s) IPR000794 Beta-ketoacyl synthase
Solyc01g107330.2.1	4.02473082	7.61E-11	3.85E-09	L2/L3-related	L,D	SWIB/MDM2 domain protein (AHRD V1 ***- D6VG39_9BURK)%3B contains Interpro domain(s) IPR019835 SWIB domain
Solyc09g025210.2.1	3.996346479	1.90E-11	3.77E-09	L2/L3-related	F	Legumin 11S-globulin (AHRD V1 **-- Q39770_GINBI)%3B contains Interpro domain(s) IPR014710 RmlC-like jelly roll fold
Solyc03g082560.2.1	3.984098285	3.03E-10	1.41E-08	L2/L3-related	L,D	Aldo/keto reductase-like protein (AHRD V1 ***- Q56Y42_ARATH)%3B contains Interpro domain(s) IPR020471 Aldo/keto reductase subgroup

Solyc12g056150.1.1	3.974301393	0.0011291	0.009801511	L2/L3-related	D	Peptidase C45 acyl-coenzyme A/6-aminopenicillanic acid acyl-transferase (AHRD V1 **-- B6THR2_MAIZE)%3B contains Interpro domain(s) IPR005079 Peptidase C45%2C acyl-coenzyme A:6-aminopenicillanic acid acyl-transferase
Solyc06g072430.1.1	3.966295905	0.0004531	0.011099788	L2/L3-related	F	Bcl-2-associated athanogene-like protein (AHRD V1 *-*- D1MIX4_VITVI)%3B contains Interpro domain(s) IPR003103 Apoptosis regulator Bcl-2 protein%2C BAG
Solyc05g054570.2.1	3.953255258	0.0001912	0.00572675	L2/L3-related	F	CRAL/TRIO domain containing protein expressed (AHRD V1 **-- Q84TA5_ORYSJ)%3B contains Interpro domain(s) IPR001251 Cellular retinaldehyde-binding/triple function%2C C-terminal
Solyc01g006540.2.1	3.930620174	7.12E-06	0.000372693	L2/L3-related	F	Lipoxygenase (AHRD V1 **** Q96573_SOLLC)%3B contains Interpro domain(s) IPR001246 Lipoxygenase%2C plant
Solyc03g111610.2.1	3.924716013	6.07E-06	0.000115529	L2/L3-related	D	HAD-superfamily hydrolase subfamily IA variant 3 containing protein expressed (AHRD V1 **** Q10I42_ORYSJ)%3B contains Interpro domain(s) IPR005834 Haloacid dehalogenase-like hydrolase
Solyc09g074420.2.1	3.91791456	3.25E-05	0.000492298	L2/L3-related	D	Serine protease (AHRD V1 **** D8UJN6_VOLCA)%3B contains Interpro domain(s) IPR015724 Serine endopeptidase DegP2
Solyc03g006980.2.1	3.917646177	2.05E-05	0.000330774	L2/L3-related	D	Alpha-L-fucosidase 1 (AHRD V1 **** B6U1Y8_MAIZE)%3B contains Interpro domain(s) IPR000933 Glycoside hydrolase%2C family 29
Solyc09g082110.2.1	3.913936865	0.0013478	0.025433143	L2/L3-related	F	Seed maturation protein (AHRD V1 **-- Q2Q4X9_MEDTR)%3B contains Interpro domain(s) IPR007011 Seed maturation protein
Solyc01g099750.2.1	3.904915331	3.75E-11	2.01E-09	L2/L3-related	L,D,F	Heparan-alpha-glucosaminide N-acetyltransferase (AHRD V1 **-- D2QUR2_SPILD)%3B contains Interpro domain(s) IPR019259 Protein of unknown function DUF2261%2C transmembrane
Solyc10g005310.2.1	3.902698202	5.06E-07	3.80E-05	L2/L3-related	F	Adenosine deaminase-like protein (Predicted) (AHRD V1 **** B2KIP2_RHIFE)%3B contains Interpro domain(s) IPR001365 Adenosine/AMP deaminase
Solyc06g068880.2.1	3.896569733	0.0003465	0.008968672	L2/L3-related	F	Serine carboxypeptidase 1 (AHRD V1 **** B6U0V5_MAIZE)%3B contains Interpro domain(s) IPR001563 Peptidase S10%2C serine carboxypeptidase
Solyc11g067160.1.1	3.895801362	7.58E-09	2.79E-07	L2/L3-related	D	Aldo/keto reductase family protein (AHRD V1 ***- D7LJ14_ARALY)%3B contains Interpro domain(s) IPR001395 Aldo/keto reductase
Solyc02g077160.2.1	3.883698845	8.81E-06	0.00015947	L2/L3-related	D	Lipase-like protein (AHRD V1 ***- Q8LF19_ARATH)%3B contains Interpro domain(s) IPR002921 Lipase%2C class 3
Solyc03g005580.2.1	3.869387121	1.66E-06	0.000107478	L2/L3-related	F	Legumin 11S-globulin (AHRD V1 **-- Q39770_GINBI)%3B contains Interpro domain(s) IPR014710 RmlC-like jelly roll fold
Solyc02g067180.2.1	3.86775667	5.87E-15	1.96E-12	L2/L3-related	F	Cystathionine gamma synthase (AHRD V1 ***- Q6R8F6_SOLLC)%3B contains Interpro domain(s) IPR000277 Cys/Met metabolism%2C pyridoxal phosphate-dependent enzyme
Solyc10g081100.1.1	3.862422185	6.41E-10	2.80E-08	L2/L3-related	D	Unknown Protein (AHRD V1)
Solyc04g072160.2.1	3.856992618	9.37E-09	1.11E-06	L2/L3-related	F	Prostaglandin E synthase 3 (AHRD V1 **-- B4FLE3_MAIZE)%3B contains Interpro domain(s) IPR017447 CS
Solyc12g044410.1.1	3.852766985	0.0006652	0.006344565	L2/L3-related	D	Class III homeodomain-leucine zipper (AHRD V1 ***- Q1WD30_GINBI)%3B contains Interpro domain(s) IPR013978 MEKHLA
Solyc01g110270.2.1	3.833209074	2.79E-08	9.29E-07	L2/L3-related	D	Crs1/yhby domain containing protein (Fragment) (AHRD V1 *-*- A6N061_ORYSI)%3B contains Interpro domain(s) IPR001890 RNA-binding%2C CRM domain
Solyc06g064550.2.1	3.830031661	3.76E-08	2.51E-06	L2/L3-related	L	Aspartokinase-homoserine dehydrogenase (AHRD V1 **** O63067_SOYBN)%3B contains Interpro domain(s) IPR001341 Aspartate kinase region
Solyc10g083720.1.1	3.820020552	0.0004259	0.010546305	L2/L3-related	F	Pyruvate kinase (AHRD V1 ***- Q94KE3_ARATH)%3B contains Interpro domain(s) IPR001697 Pyruvate kinase
Solyc01g081490.2.1	3.815421099	0.000794	0.015079084	L2/L3-related	L,D	RNA polymerase sigma factor (AHRD V1 *-*- Q59965_SYNP2)%3B contains Interpro domain(s) IPR016262 RNA polymerase sigma factor%2C SigB/SigC/SigD%2C plastid
Solyc11g072380.1.1	3.81309799	2.90E-07	2.29E-05	L2/L3-related	F	Vicilin-like protein (Fragment) (AHRD V1 **-- Q9SEW4_9ROSI)%3B contains Interpro domain(s) IPR011051 Cupin%2C RmlC-type
Solyc12g099090.1.1	3.806016034	0.0004017	0.010037694	L2/L3-related	F	YTH domain family 2 (Predicted) (AHRD V1 *--- B2GUU1_RAT)%3B contains Interpro domain(s) IPR007275 YT521-B-like protein
Solyc02g085520.2.1	3.786872458	0.0013759	0.01150542	L2/L3-related	D	Adenylosuccinate synthetase%2C chloroplastic (AHRD V1 **** PURA_SOLBU)%3B contains Interpro domain(s) IPR001114 Adenylosuccinate synthetase
Solyc11g008780.1.1	3.775518299	0.0009572	0.019718334	L2/L3-related	F	Acetolactate synthase small subunit (AHRD V1 ***- Q9SMC2_NICPL)%3B contains Interpro domain(s) IPR004789 Acetolactate synthase%2C small subunit
Solyc12g042600.1.1	3.771988457	4.09E-05	0.000596367	L2/L3-related	D	UDP-glucosyltransferase family 1 protein (AHRD V1 ***- C6KI43_CITSI)%3B contains Interpro domain(s) IPR002213 UDP-glucuronosyl/UDP-glucosyltransferase
Solyc09g018890.2.1	3.762814662	0.0007632	0.01467551	L2/L3-related	L	Genome sequencing data contig C320 (AHRD V1 ***- A8YJ83_MICAE)
Solyc12g062250.1.1	3.758167963	0.0011225	0.009762113	L2/L3-related	D	5%26apos-AMP-activated protein kinase beta-1 subunit (AHRD V1 *--- C6LYW2_GIALA)%3B contains Interpro domain(s) IPR001478 PDZ/DHR/GLGF
Solyc01g111040.2.1	3.732045995	0.0006792	0.015325425	L2/L3-related	F	EF-Hand containing protein-like (AHRD V1 ***- Q9T0I9_ARATH)%3B contains Interpro domain(s) IPR018248 EF hand
Solyc09g075180.2.1	3.726219871	0.0005575	0.013052919	L2/L3-related	F	Cryptochrome DASH family (AHRD V1 **-- B5W072_SPIMA)%3B contains Interpro domain(s) IPR006050 DNA photolyase%2C N-terminal
Solyc12g013690.1.1	3.724421087	8.42E-06	0.000154401	L2/L3-related	D	Monooxygenase FAD-binding protein (AHRD V1 **-- Q1B616_MYCSS)%3B contains Interpro domain(s) IPR003042 Aromatic-ring hydroxylase-like
Solyc10g083350.1.1	3.719772584	1.57E-07	4.42E-06	L2/L3-related	L,D	Soul heme-binding family protein (AHRD V1 ***- D7L8V8_ARALY)%3B contains Interpro domain(s) IPR006917 SOUL haem-binding protein
Solyc03g093240.2.1	3.714640587	1.97E-06	4.33E-05	L2/L3-related	D	GTP-binding protein YqeH (AHRD V1 **-- C6VPQ3_LACPJ)%3B contains Interpro domain(s) IPR002917 GTP-binding protein%2C HSR1-related
Solyc08g067330.1.1	3.702409423	8.52E-06	0.000328736	L2/L3-related	L	Chlorophyll a-b binding protein 3C-like (AHRD V1 ***- Q2XTE0_SOLTU)%3B contains Interpro domain(s) IPR001344 Chlorophyll A-B binding protein
Solyc08g066850.2.1	3.699818552	8.99E-06	0.000161803	L2/L3-related	D	Lactoylglutathione lyase (AHRD V1 ***- B6SSK1_MAIZE)%3B contains Interpro domain(s) IPR004361 Glyoxalase I
Solyc07g007180.2.1	3.697059002	8.43E-08	2.55E-06	L2/L3-related	D	RING-H2 finger protein (AHRD V1 *-*- B6TJA7_MAIZE)%3B contains Interpro domain(s) IPR018957 Zinc finger%2C C3HC4 RING-type
Solyc05g012510.2.1	3.691983917	1.09E-09	4.56E-08	L2/L3-related	D	Phosphorylase (AHRD V1 **** B9HXL0_POPTR)%3B contains Interpro domain(s) IPR011833 Glycogen/starch/alpha-glucan phosphorylase
Solyc02g083970.1.1	3.672059744	9.72E-05	0.001270152	L2/L3-related	D	Chromosome 18 contig 1 DNA sequence (AHRD V1 *-*- Q00SS6_OSTTA)
Solyc10g081470.1.1	3.662759387	7.12E-05	0.000968551	L2/L3-related	D	Peptidase M50 family protein (AHRD V1 ***- C1FJH6_9CHLO)%3B contains Interpro domain(s) IPR008915 Peptidase M50
Solyc11g072950.1.1	3.649816364	0.002303	0.035135622	L2/L3-related	L	E3 UFM1-protein ligase 1 homolog (AHRD V1 ***- UFL1_ARATH)%3B contains Interpro domain(s) IPR018611 Protein of unknown function DUF2042
Solyc06g053810.2.1	3.648185502	0.000108	0.001384209	L2/L3-related	D	At1g04280-like protein (Fragment) (AHRD V1 *-*- B0FVI9_ARALY)
Solyc02g063030.2.1	3.646874318	0.0060924	0.036894095	L2/L3-related	D	LuxR family transcriptional regulator (Fragment) (AHRD V1 *-*- D9ZBL3_9CARY)
Solyc12g088230.1.1	3.640301014	2.72E-05	0.00116762	L2/L3-related	F	Early-responsive to dehydration protein-like (AHRD V1 **-- Q5JKX1_ORYSJ)%3B contains Interpro domain(s) IPR003864 Protein of unknown function DUF221
Solyc04g072400.2.1	3.631911144	3.45E-07	9.10E-06	L2/L3-related	D	Endoribonuclease E-like protein (AHRD V1 **-- Q656E2_ORYSJ)
Solyc08g048450.2.1	3.628245293	3.35E-05	0.001381504	L2/L3-related	D,F	Ornithine-oxo-acid transaminase (AHRD V1 **** A8CF49_BRACM)%3B contains Interpro domain(s) IPR010164 Ornithine aminotransferase
Solyc04g015490.2.1	3.611283203	4.82E-05	0.000687616	L2/L3-related	D	Magnesium chelatase subunit D (AHRD V1 *-*- A8I531_CHLRE)%3B contains Interpro domain(s) IPR011776 Magnesium chelatase%2C ATPase subunit D
Solyc02g079170.2.1	3.604272405	3.27E-05	0.001357773	L2/L3-related	F	NADH dehydrogenase like protein (AHRD V1 **** O65414_ARATH)%3B contains Interpro domain(s) IPR013027 FAD-dependent pyridine nucleotide-disulphide oxidoreductase
Solyc01g087520.2.1	3.602468393	1.46E-06	3.31E-05	L2/L3-related	D	Ferredoxin-thioredoxin reductase variable chain (AHRD V1 *-*- B6UCW8_MAIZE)%3B contains Interpro domain(s) IPR004207 Ferredoxin thioredoxin reductase%2C alpha chain
Solyc06g071070.1.1	3.588242961	0.002706	0.019721152	L2/L3-related	D	Short-chain dehydrogenase/reductase family protein (AHRD V1 ***- D7L204_ARALY)%3B contains Interpro domain(s) IPR002347 Glucose/ribitol dehydrogenase
Solyc03g097000.2.1	3.56177075	0.0006281	0.006049057	L2/L3-related	D	Pentatricopeptide repeat-containing protein (AHRD V1 ***- D7M672_ARALY)%3B contains Interpro domain(s) IPR002885 Pentatricopeptide repeat
Solyc01g104720.2.1	3.561451957	1.41E-13	1.12E-11	L2/L3-related	L,D	Unknown Protein (AHRD V1)
Solyc03g097470.2.1	3.545049678	2.17E-07	1.75E-05	L2/L3-related	F	3-oxoacyl- (AHRD V1 ***- D5A3B9_SPIPL)%3B contains Interpro domain(s) IPR004655 Beta-ketoacyl-acyl carrier protein synthase III (FabH)
Solyc02g078950.2.1	3.531397101	0.0001319	0.001619892	L2/L3-related	D	Beta-galactosidase (AHRD V1 **** B9HYZ2_POPTR)%3B contains Interpro domain(s) IPR001944 Glycoside hydrolase%2C family 35
Solyc02g082690.2.1	3.526841396	8.73E-08	8.26E-06	L2/L3-related	F	Syntaxin binding protein 5 (Tomosyn) (AHRD V1 *--- Q14DD4_HUMAN)%3B contains Interpro domain(s) IPR011046 WD40 repeat-like
Solyc02g087220.2.1	3.508954051	0.0003789	0.003973976	L2/L3-related	D	Protein bicaudal C homolog 1-B (AHRD V1 *--- BIC1B_XENLA)%3B contains Interpro domain(s) IPR001660 Sterile alpha motif SAM
Solyc07g021630.2.1	3.492559613	0.0001186	0.00149133	L2/L3-related	D	Branched-chain-amino-acid aminotransferase (AHRD V1 **** Q94CF8_CAPAN)%3B contains Interpro domain(s) IPR005786 Branched-chain amino acid aminotransferase II
Solyc03g116900.2.1	3.489721632	0.0021621	0.036314618	L2/L3-related	F	Manganese transporter MntH (AHRD V1 **-* D3KPZ0_LISMO)%3B contains Interpro domain(s) IPR001046 Natural resistance-associated macrophage protein
Solyc08g082820.2.1	3.479011676	1.65E-07	1.40E-05	L2/L3-related	F	Heat shock protein (AHRD V1 ***- Q84KP8_CYAME)%3B contains Interpro domain(s) IPR013126 Heat shock protein 70
Solyc12g098570.1.1	3.477728394	0.0021092	0.035679887	L2/L3-related	F	Receptor like kinase%2C RLK
Solyc08g078290.1.1	3.461330849	0.00448	0.028798559	L2/L3-related	D	Glycoprotein homolog (AHRD V1 *-*- Q8LCM1_ARATH)
Solyc12g089320.1.1	3.438175865	0.0027272	0.042807703	L2/L3-related	F	Bromo-adjacent homology (BAH) domain-containing protein-like (AHRD V1 *-*- Q6Z809_ORYSJ)
Solyc10g011770.2.1	3.429980877	0.003827	0.025669896	L2/L3-related	D	Threonine endopeptidase (AHRD V1 **-- B6TCN7_MAIZE)
Solyc02g093460.2.1	3.403503081	0.0003701	0.003893528	L2/L3-related	D	tRNA pseudouridine synthase B (AHRD V1 *-*- D0MI93_RHOM4)%3B contains Interpro domain(s) IPR020103 Pseudouridine synthase%2C catalytic domain
Solyc09g005100.2.1	3.390023013	5.42E-05	0.000759764	L2/L3-related	D	Aprataxin (AHRD V1 *--- B1AXX8_MOUSE)%3B contains Interpro domain(s) IPR011151 Histidine triad motif
Solyc06g074200.2.1	3.382567935	2.41E-05	0.000381228	L2/L3-related	D	Sex-linked protein 9 (Fragment) (AHRD V1 ***- D7RW11_SILCU)%3B contains Interpro domain(s) IPR017498 Photosystem I PsaO
Solyc03g113590.2.1	3.37007952	6.77E-06	0.000127063	L2/L3-related	D	5-formyltetrahydrofolate cyclo-ligase (AHRD V1 **** B9I331_POPTR)%3B contains Interpro domain(s) IPR002698 5-formyltetrahydrofolate cyclo-ligase
Solyc04g049130.2.1	3.362929406	2.69E-05	0.000415743	L2/L3-related	D	At5g03900 (Fragment) (AHRD V1 *-*- B0LQ56_ARATH)%3B contains Interpro domain(s) IPR000361 FeS cluster biogenesis
Solyc12g099970.1.1	3.341457219	3.49E-07	9.16E-06	L2/L3-related	D	5-amp-activated protein kinase beta subunit (AHRD V1 **** Q17IU7_AEDAE)%3B contains Interpro domain(s) IPR006828 5-AMP-activated protein kinase%2C beta subunit%2C complex-interacting region
Solyc03g120230.2.1	3.330445204	7.71E-05	0.001038562	L2/L3-related	D	MAR-binding filament-like protein 1 (AHRD V1 **** MFP1_SOLLC)%3B contains Interpro domain(s) IPR009053 Prefoldin
Solyc02g070490.2.1	3.325459437	1.32E-05	0.000224166	L2/L3-related	D	Alpha/beta hydrolase fold (AHRD V1 ***- B6SU76_MAIZE)%3B contains Interpro domain(s) IPR000073 Alpha/beta hydrolase fold-1
Solyc01g080020.2.1	3.310164888	2.18E-11	1.21E-09	L2/L3-related	D	Xylanase inhibitor (Fragment) (AHRD V1 **-- Q53IQ4_WHEAT)%3B contains Interpro domain(s) IPR001461 Peptidase A1
Solyc02g087870.2.1	3.309071553	8.08E-05	0.00107809	L2/L3-related	D	Uncharacterized ABC transporter ATP-binding protein/permease C9B6.09c (AHRD V1 *--* YNT9_SCHPO)%3B contains Interpro domain(s) IPR001140 ABC transporter%2C transmembrane region
Solyc04g051580.2.1	3.300238369	9.24E-05	0.001212761	L2/L3-related	D	Mitochondrial carrier C12B10.09 (AHRD V1 ***- B6TYC7_MAIZE)%3B contains Interpro domain(s) IPR002067 Mitochondrial carrier protein
Solyc09g014280.1.1	3.284699882	3.96E-05	0.001584287	L2/L3-related	F	Hydroxycinnamoyl transferase (AHRD V1 **-* D2XJ64_9MAGN)%3B contains Interpro domain(s) IPR003480 Transferase
Solyc02g082260.2.1	3.281088785	1.16E-09	1.59E-07	L2/L3-related	F	Hydroxy-methylglutaryl-coenzyme A reductase (AHRD V1 **** O48624_TOBAC)%3B contains Interpro domain(s) IPR004554 Hydroxymethylglutaryl-CoA reductase%2C class I%2C catalytic
Solyc02g081850.2.1	3.276834326	1.55E-05	0.000255767	L2/L3-related	D	Amino acid transporter (AHRD V1 **** C2SWS0_BACCE)%3B contains Interpro domain(s) IPR015606 Cationic amino acid transporter
Solyc07g063960.2.1	3.261313409	1.55E-05	0.000256297	L2/L3-related	D	50S ribosomal protein L24 (AHRD V1 ***- B4FTJ3_MAIZE)%3B contains Interpro domain(s) IPR003256 Ribosomal protein L24
Solyc09g075440.2.1	3.258338461	2.79E-05	0.001195961	L2/L3-related	F	Ethylene receptor (AHRD V1 **** Q41341_SOLLC)%3B contains Interpro domain(s) IPR005467 Signal transduction histidine kinase%2C core
Solyc00g009100.2.1	3.242437764	0.0001586	0.001893124	L2/L3-related	D	30S ribosomal protein S1-like (AHRD V1 ***- Q5ZC75_ORYSJ)%3B contains Interpro domain(s) IPR003029 Ribosomal protein S1%2C RNA binding domain
Solyc10g076250.1.1	3.222018805	7.33E-11	3.72E-09	L2/L3-related	D	Aminotransferase like protein (AHRD V1 ***- Q0WWF1_ARATH)%3B contains Interpro domain(s) IPR005814 Aminotransferase class-III
Solyc01g080810.2.1	3.198330056	4.74E-07	1.21E-05	L2/L3-related	D	Isoleucyl-tRNA synthetase (AHRD V1 ***- Q9FI67_ARATH)%3B contains Interpro domain(s) IPR002301 Isoleucyl-tRNA synthetase%2C class Ia IPR018353 Isoleucyl-tRNA synthetase
Solyc09g007190.2.1	3.190155531	0.0001144	0.001452039	L2/L3-related	L,D	C1orf93 homolog (AHRD V1 **-- B5X9L9_SALSA)%3B contains Interpro domain(s) IPR012335 Thioredoxin fold
Solyc10g008400.1.1	3.179437416	7.46E-07	1.82E-05	L2/L3-related	D	RING finger protein 5 (AHRD V1 *--- B6TI40_MAIZE)%3B contains Interpro domain(s) IPR018957 Zinc finger%2C C3HC4 RING-type
Solyc03g112640.2.1	3.178659892	0.006933	0.040897808	L2/L3-related	D	CRAL/TRIO domain containing protein (AHRD V1 **-- B6UD95_MAIZE)%3B contains Interpro domain(s) IPR001251 Cellular retinaldehyde-binding/triple function%2C C-terminal
Solyc03g117480.2.1	3.176701885	2.67E-06	0.000161547	L2/L3-related	F	Pyrophosphate-energized proton pump (Pyrophosphate-energized inorganic pyrophosphatase) (H(+)-PPase) (AHRD V1 **** C7HW54_9FIRM)%3B contains Interpro domain(s) IPR004131 Inorganic H+ pyrophosphatase
Solyc10g005770.2.1	3.16607641	2.77E-06	5.83E-05	L2/L3-related	D	Ribosomal protein S6 kinase alpha-3 (AHRD V1 *-** KS6A3_HUMAN)%3B contains Interpro domain(s) IPR002290 Serine/threonine protein kinase
Solyc11g005080.1.1	3.154684034	1.33E-08	4.74E-07	L2/L3-related	D	Protein tolB (AHRD V1 *-*- D5MJZ0_9BACT)%3B contains Interpro domain(s) IPR011042 Six-bladed beta-propeller%2C TolB-like
Solyc03g121090.2.1	3.150157145	1.37E-09	5.67E-08	L2/L3-related	D	Cold induced protein-like (AHRD V1 **-- Q94JH8_ORYSJ)
Solyc12g098350.1.1	3.144059116	1.58E-05	0.000260215	L2/L3-related	D	Translation initiation factor 1 (AHRD V1 *-*- D1M7Q2_PARAR)%3B contains Interpro domain(s) IPR006196 S1%2C IF1 type
Solyc07g045070.2.1	3.138875026	1.92E-07	5.33E-06	L2/L3-related	D	Ribosomal RNA small subunit methyltransferase B (AHRD V1 *-*- B4AE47_BACPU)%3B contains Interpro domain(s) IPR001678 Bacterial Fmu (Sun)/eukaryotic nucleolar NOL1/Nop2p
Solyc07g005570.2.1	3.130813695	3.84E-05	0.000565339	L2/L3-related	D	APO protein 2%2C chloroplastic (AHRD V1 ***- APO2_ARATH)%3B contains Interpro domain(s) IPR008512 Protein of unknown function DUF794%2C plant
Solyc06g074290.1.1	3.119986938	3.87E-06	0.000217955	L2/L3-related	F	Unknown Protein (AHRD V1)

Solyc10g037900.1.1	3.095297986	6.09E-05	0.000843656	L2/L3-related	D	Unknown Protein (AHRD V1)
Solyc04g080830.2.1	3.087839561	0.0016893	0.013559833	L2/L3-related	D	Pentatricopeptide repeat-containing protein (AHRD V1 ***- D7KGE6_ARALY)%3B contains Interpro domain(s) IPR002885 Pentatricopeptide repeat
Solyc06g008220.2.1	3.056535442	9.55E-06	0.000169717	L2/L3-related	D	DAG protein (AHRD V1 **-- B6TEQ2_MAIZE)
Solyc02g092310.2.1	3.054116991	2.42E-05	0.000381752	L2/L3-related	D	Pentatricopeptide repeat-containing protein (AHRD V1 *-*- D7L2Y3_ARALY)%3B contains Interpro domain(s) IPR002885 Pentatricopeptide repeat
Solyc01g097160.2.1	3.051155591	3.13E-05	0.000476727	L2/L3-related	D	FAD dependent oxidoreductase (AHRD V1 ***- B7G7N7_PHATR)%3B contains Interpro domain(s) IPR006076 FAD dependent oxidoreductase
Solyc03g096840.2.1	3.048342119	2.15E-09	8.66E-08	L2/L3-related	D	Seed specific protein Bn15D1B (AHRD V1 ***- B6TIB8_MAIZE)
Solyc04g007580.1.1	3.045287987	0.0005217	0.005201357	L2/L3-related	D	cDNA clone J100026I16 full insert sequence (AHRD V1 **-- B7FA06_ORYSJ)
Solyc12g006810.1.1	3.044853636	0.0007241	0.00681794	L2/L3-related	D	Soul heme-binding family protein (AHRD V1 **** D7LZY3_ARALY)%3B contains Interpro domain(s) IPR006917 SOUL haem-binding protein
Solyc04g082960.1.1	3.0366999	0.0001739	0.004324839	L2/L3-related	L	TMV response-related protein (AHRD V1 *--- B6UGH4_MAIZE)
Solyc07g053020.1.1	3.02779674	0.0002422	0.002738701	L2/L3-related	D	Cc-nbs-lrr%2C resistance protein
Solyc01g099840.2.1	3.018686582	3.51E-10	1.60E-08	L2/L3-related	D	Auxin-repressed protein (AHRD V1 ***- B4FA62_MAIZE)%3B contains Interpro domain(s) IPR008406 Dormancyauxin associated
Solyc04g054470.2.1	3.017712361	1.48E-06	9.79E-05	L2/L3-related	F	Myosin class II heavy chain (ISS) (AHRD V1 *-*- Q014V4_OSTTA)
Solyc02g087110.2.1	2.972129589	5.17E-05	0.001552487	L2/L3-related	L	Alpha-dioxygenase (AHRD V1 **-- Q5GQ66_PEA)%3B contains Interpro domain(s) IPR002007 Haem peroxidase%2C animal
Solyc05g050500.1.1	2.967271029	1.68E-06	0.000107987	L2/L3-related	L,D,F	ATP synthase F1 delta subunit (AHRD V1 ***- B4B4T7_9CHRO)%3B contains Interpro domain(s) IPR000711 ATPase%2C F1 complex%2C OSCP/delta subunit
Solyc12g013890.1.1	2.956660695	1.49E-06	3.36E-05	L2/L3-related	D	Genomic DNA chromosome 5 TAC clone K19P17 (AHRD V1 ***- Q9FN40_ARATH)
Solyc11g010630.2.1	2.948793873	3.55E-05	0.000529157	L2/L3-related	D	Unknown Protein (AHRD V1)
Solyc07g008310.2.1	2.944386771	0.0025739	0.019017377	L2/L3-related	D	Rieske (2Fe-2S) domain protein (AHRD V1 **-- Q024N8_SOLUE)%3B contains Interpro domain(s) IPR001663 Aromatic-ring-hydroxylating dioxygenase%2C alpha subunit
Solyc07g005960.2.1	2.942336032	0.00018	0.002125893	L2/L3-related	D	Serine carboxypeptidase K10B2.2 (AHRD V1 **** B6TM29_MAIZE)%3B contains Interpro domain(s) IPR001563 Peptidase S10%2C serine carboxypeptidase
Solyc09g090150.2.1	2.942242234	0.000187	0.005642039	L2/L3-related	F	Legumin 11S-globulin (AHRD V1 **-- Q39770_GINBI)%3B contains Interpro domain(s) IPR014710 RmlC-like jelly roll fold
Solyc09g091180.2.1	2.93184503	0.0002615	0.007232947	L2/L3-related	F	chaperonin (AHRD V1 ***- Q6N0B9_9PROT)%3B contains Interpro domain(s) IPR001844 Chaperonin Cpn60
Solyc09g007900.2.1	2.93139811	1.61E-05	0.000568109	L2/L3-related	L	Phenylalanine ammonia-lyase (AHRD V1 **** B5LAW0_CAPAN)%3B contains Interpro domain(s) IPR005922 Phenylalanine ammonia-lyase
Solyc09g092600.2.1	2.923227209	0.0007957	0.00737729	L2/L3-related	D	Cytochrome P450
Solyc05g012600.2.1	2.915407704	0.0001018	0.001315506	L2/L3-related	D	Unknown Protein (AHRD V1)
Solyc12g006450.1.1	2.914523971	4.64E-05	0.00066719	L2/L3-related	D	Aminotransferase-like protein (AHRD V1 ***- Q9LIE2_ARATH)%3B contains Interpro domain(s) IPR005814 Aminotransferase class-III
Solyc04g077440.2.1	2.911560531	0.0002123	0.00613067	L2/L3-related	F	Squalene monooxygenase (AHRD V1 ***- Q506K3_DATIN)%3B contains Interpro domain(s) IPR013698 Squalene epoxidase
Solyc02g086830.2.1	2.90166367	9.00E-08	2.71E-06	L2/L3-related	D	Protease Do-like (S2 serine-type protease) (AHRD V1 **** Q7UQ70_RHOBA)%3B contains Interpro domain(s) IPR001254 Peptidase S1 and S6%2C chymotrypsin/Hap
Solyc09g090940.2.1	2.892586219	0.0015102	0.027846488	L2/L3-related	F	Ras GTPase-activating protein-binding protein 2 (AHRD V1 *--- B5X4A8_SALSA)%3B contains Interpro domain(s) IPR018222 Nuclear transport factor 2%2C Eukaryote
Solyc07g054450.2.1	2.869802823	0.0018158	0.031855693	L2/L3-related	F	Transcription factor (Fragment) (AHRD V1 ***- D6MKF6_9ASPA)%3B contains Interpro domain(s) IPR000770 SAND
Solyc05g015860.2.1	2.846553484	3.43E-08	1.12E-06	L2/L3-related	D	Sphingosine-1-phosphate lyase (AHRD V1 **-* Q549V9_ARATH)%3B contains Interpro domain(s) IPR002129 Pyridoxal phosphate-dependent decarboxylase
Solyc09g072560.2.1	2.846266288	6.01E-06	0.000324607	L2/L3-related	F	Legumin 11S-globulin (AHRD V1 **-- Q39770_GINBI)%3B contains Interpro domain(s) IPR014710 RmlC-like jelly roll fold
Solyc03g005190.2.1	2.845563716	0.0005963	0.005812095	L2/L3-related	D	Ferredoxin (AHRD V1 *-*- Q8LD29_ARATH)%3B contains Interpro domain(s) IPR010241 Ferredoxin [2Fe-2S]%2C plant
Solyc11g066330.1.1	2.798554263	2.55E-07	6.95E-06	L2/L3-related	D	Nodulin family protein (AHRD V1 ***- D7LIF1_ARALY)%3B contains Interpro domain(s) IPR010658 Nodulin-like
Solyc05g056120.2.1	2.779085325	0.0005757	0.01343231	L2/L3-related	F	GATA transcription factor 1 (AHRD V1 *-*- Q0WTQ5_ARATH)%3B contains Interpro domain(s) IPR016679 Transcription factor%2C GATA%2C plant
Solyc08g077140.1.1	2.765120723	6.06E-05	0.000841905	L2/L3-related	D	Pentatricopeptide repeat-containing protein (AHRD V1 ***- D7MRC5_ARALY)%3B contains Interpro domain(s) IPR002885 Pentatricopeptide repeat
Solyc02g076720.2.1	2.760484602	8.44E-05	0.002388681	L2/L3-related	L	Phosphoribosylformylglycinamidine synthase (AHRD V1 **** A9Y5J1_ARATH)%3B contains Interpro domain(s) IPR010073 Phosphoribosylformylglycinamidine synthase%2C eukaryotes and proteobacteria
Solyc07g049290.2.1	2.758831118	5.99E-08	1.88E-06	L2/L3-related	D	Nitrate transporter (AHRD V1 **** Q8LG02_ARATH)%3B contains Interpro domain(s) IPR000109 TGF-beta receptor%2C type I/II extracellular region
Solyc07g062500.2.1	2.741532579	0.0003399	0.008847715	L2/L3-related	F	Cytochrome P450
Solyc01g104310.2.1	2.736018115	3.54E-05	0.000528363	L2/L3-related	D	Patatin-like phospholipase domain-containing protein (AHRD V1 *-*- B6KR35_TOXGO)%3B contains Interpro domain(s) IPR002641 Patatin
Solyc02g085700.1.1	2.729227496	2.57E-05	0.000400951	L2/L3-related	D	Geranylgeranyl pyrophosphate synthase (AHRD V1 ***- D0FZ25_9ASTE)%3B contains Interpro domain(s) IPR000092 Polyprenyl synthetase
Solyc04g050540.2.1	2.717986095	0.0012324	0.010488674	L2/L3-related	D	Pentatricopeptide repeat protein (AHRD V1 *-*- Q1G1I8_MAIZE)%3B contains Interpro domain(s) IPR003034 DNA-binding SAP
Solyc05g007510.2.1	2.716776331	0.0005813	0.005693258	L2/L3-related	D	RNA-dependent RNA polymerase (AHRD V1 **** C1I213_NICGU)%3B contains Interpro domain(s) IPR007855 RNA-dependent RNA polymerase%2C eukaryotic-type
Solyc07g054730.1.1	2.710779785	4.83E-10	2.14E-08	L2/L3-related	D	Wound-responsive protein-related (AHRD V1 ***- A8IXM2_BRACM)
Solyc09g091470.2.1	2.70026551	1.44E-07	1.25E-05	L2/L3-related	F	3-ketoacyl CoA thiolase 2 (AHRD V1 **** C8YNG7_PETHY)%3B contains Interpro domain(s) IPR002155 Thiolase
Solyc09g092260.2.1	2.686398524	5.58E-08	1.76E-06	L2/L3-related	D	Chaperone protein dnaJ 20 (AHRD V1 ***- B6U349_MAIZE)%3B contains Interpro domain(s) IPR001623 Heat shock protein DnaJ%2C N-terminal
Solyc09g074100.2.1	2.681376475	0.0004206	0.004349659	L2/L3-related	D	tRNA-specific 2-thiouridylase mnmA (AHRD V1 **** A6DKA8_9BACT)%3B contains Interpro domain(s) IPR018318 tRNA methyl transferase-like
Solyc02g083310.2.1	2.678810171	0.00021	0.002432267	L2/L3-related	D	Wound responsive protein (Fragment) (AHRD V1 *--- A6N0L4_ORYSI)%3B contains Interpro domain(s) IPR003729 Protein of unknown function DUF151
Solyc11g006970.1.1	2.655097936	0.0002483	0.00279823	L2/L3-related	D	Unknown protein DS12 from 2D-PAGE of leaf%2C chloroplastic (AHRD V1 **-- UP12_ORYSJ)
Solyc01g009230.2.1	2.652318989	5.44E-05	0.000761764	L2/L3-related	D	Xanthine dehydrogenase/oxidase (AHRD V1 ***- A9YL93_RABIT)%3B contains Interpro domain(s) IPR016208 Aldehyde oxidase/xanthine dehydrogenase
Solyc01g087250.2.1	2.650591012	0.0005043	0.012073623	L2/L3-related	F	Carotenoid cleavage dioxygenase 1A
Solyc03g005730.2.1	2.634435609	0.0001363	0.004367479	L2/L3-related	F	3-isopropylmalate dehydratase large subunit 2 (AHRD V1 ***- B6U6T6_MAIZE)%3B contains Interpro domain(s) IPR011826 Homoaconitase/3-isopropylmalate dehydratase%2C large subunit%2C prokaryotic
Solyc10g083570.1.1	2.61060621	8.89E-08	8.35E-06	L2/L3-related	F	Fructose-bisphosphate aldolase (AHRD V1 ***- Q308A5_SOLTU)%3B contains Interpro domain(s) IPR000741 Fructose-bisphosphate aldolase%2C class-I
Solyc06g076770.2.1	2.602206856	2.62E-05	0.000407487	L2/L3-related	D	Myb family transcription factor (Fragment) (AHRD V1 *--* D7M1F0_ARALY)%3B contains Interpro domain(s) IPR006447 Myb-like DNA-binding region%2C SHAQKYF class
Solyc12g056740.1.1	2.589374043	4.34E-06	8.58E-05	L2/L3-related	D	ATP-dependent RNA helicase (AHRD V1 ***- D3BPQ9_POLPA)%3B contains Interpro domain(s) IPR011545 DNA/RNA helicase%2C DEAD/DEAH box type%2C N-terminal
Solyc02g088610.2.1	2.588339856	1.58E-05	0.000738704	L2/L3-related	D,F	ATP-dependent chaperone ClpB (AHRD V1 **** B5W0X8_SPIMA)%3B contains Interpro domain(s) IPR017730 Chaperonin ClpB
Solyc04g025990.2.1	2.588077029	9.72E-08	2.91E-06	L2/L3-related	D	Potassium transporter family protein (AHRD V1 **** D7KXB7_ARALY)%3B contains Interpro domain(s) IPR003855 K+ potassium transporter
Solyc08g076320.2.1	2.57579246	3.98E-06	0.000222148	L2/L3-related	F	Cellulose synthase-like protein (AHRD V1 **** B9IPJ4_POPTR)%3B contains Interpro domain(s) IPR005150 Cellulose synthase
Solyc05g041530.2.1	2.571754838	0.0075465	0.043573628	L2/L3-related	D	Dienelactone hydrolase domain protein (AHRD V1 *--- C3HWX7_BACTU)
Solyc10g078560.1.1	2.559966437	3.34E-05	0.000502331	L2/L3-related	D	Chaperone protein dnaJ (AHRD V1 *-*- A2SS06_METLZ)%3B contains Interpro domain(s) IPR001623 Heat shock protein DnaJ%2C N-terminal
Solyc11g072880.1.1	2.546879109	0.0006296	0.01434775	L2/L3-related	F	Calcium-transporting ATPase (AHRD V1 ***- A8NHF4_COPC7)%3B contains Interpro domain(s) IPR005782 ATPase%2C P-type%2C calcium-transporting
Solyc09g015770.2.1	2.542514127	3.39E-06	0.000147286	L2/L3-related	L	WRKY transcription factor 6 (AHRD V1 **** A7UGD3_SOLTU)%3B contains Interpro domain(s) IPR003657 DNA-binding WRKY
Solyc01g068030.2.1	2.530280464	0.0004426	0.00452974	L2/L3-related	D	Cyclase/dehydrase (AHRD V1 ***- B6T2Z5_MAIZE)%3B contains Interpro domain(s) IPR005031 Streptomyces cyclase/dehydrase
Solyc07g062030.2.1	2.522199014	0.0002891	0.00316866	L2/L3-related	D	Chalcone--flavonone isomerase (AHRD V1 ***- B6SK22_MAIZE)%3B contains Interpro domain(s) IPR003466 Chalcone isomerase%2C subgroup
Solyc03g119040.2.1	2.521215935	0.0003145	0.008334962	L2/L3-related	F	Guanine nucleotide-binding protein beta subunit-like protein (AHRD V1 **-* B4FKM1_MAIZE)%3B contains Interpro domain(s) IPR020472 G-protein beta WD-40 repeat%2C region
Solyc10g081120.1.1	2.501804042	1.88E-07	5.25E-06	L2/L3-related	D	Alpha-L-arabinofuranosidase (AHRD V1 **** Q76LU4_SOLLC)%3B contains Interpro domain(s) IPR017853 Glycoside hydrolase%2C catalytic core
Solyc07g005210.2.1	2.49473863	0.0049415	0.031230873	L2/L3-related	D	Outer membrane lipoprotein blc (AHRD V1 **-- D0RZG9_ACICA)%3B contains Interpro domain(s) IPR002345 Lipocalin
Solyc01g087890.2.1	2.492438729	4.94E-05	0.000702498	L2/L3-related	D	Choline transporter-like protein 5-A (Fragment) (AHRD V1 *--- CTL5A_DANRE)%3B contains Interpro domain(s) IPR007603 Protein of unknown function DUF580
Solyc12g010320.1.1	2.453690966	0.001154	0.022457	L2/L3-related	F	Outer membrane lipoprotein blc (AHRD V1 **-- D0RZG9_ACICA)%3B contains Interpro domain(s) IPR002345 Lipocalin
Solyc01g081570.2.1	2.436239868	0.0009141	0.008265556	L2/L3-related	D	Carboxyl-terminal peptidase (AHRD V1 ***- B6TV70_MAIZE)%3B contains Interpro domain(s) IPR004314 Protein of unknown function DUF239%2C plant
Solyc02g062430.2.1	2.429980877	0.0019795	0.015383569	L2/L3-related	D	D-lactate dehydrogenase 2 mitochondrial (AHRD V1 **-* A5DXM0_LODEL)%3B contains Interpro domain(s) IPR004113 FAD-linked oxidase%2C C-terminal
Solyc10g007760.2.1	2.417325334	0.0021358	0.016354777	L2/L3-related	D	Ribose-phosphate pyrophosphokinase 4 (AHRD V1 ***- D7LJE3_ARALY)%3B contains Interpro domain(s) IPR005946 Phosphoribosyl pyrophosphokinase
Solyc01g080910.2.1	2.407465372	0.0003417	0.003644978	L2/L3-related	D	Unknown Protein (AHRD V1)
Solyc04g076880.2.1	2.387826978	8.60E-07	2.05E-05	L2/L3-related	D	Phosphoenolpyruvate carboxykinase (AHRD V1 **** Q8W505_SOLLC)%3B contains Interpro domain(s) IPR001272 Phosphoenolpyruvate carboxykinase%2C ATP-utilising
Solyc01g080780.2.1	2.373698402	4.25E-05	0.000615476	L2/L3-related	D	Translocase of chloroplast 34 (AHRD V1 *-*- B6TBT7_MAIZE)%3B contains Interpro domain(s) IPR005690 Chloroplast protein import component Toc86/159
Solyc03g122310.2.1	2.372080917	0.0001201	0.003885085	L2/L3-related	F	Aldehyde dehydrogenase 7b (AHRD V1 **** D9IFB7_WHEAT)%3B contains Interpro domain(s) IPR015590 Aldehyde dehydrogenase
Solyc07g018360.2.1	2.369702865	4.88E-07	1.23E-05	L2/L3-related	D	Elongation factor Ts (AHRD V1 ***- Q9SZD6_ARATH)%3B contains Interpro domain(s) IPR014039 Translation elongation factor EFTs/EF1B%2C dimerisation
Solyc03g120420.2.1	2.368924733	0.0005103	0.012174175	L2/L3-related	D,F	Unknown Protein (AHRD V1)
Solyc08g014060.2.1	2.361472099	0.0015958	0.012939217	L2/L3-related	D	Eukaryotic translation initiation factor 3 subunit 9-like protein (AHRD V1 **** D2VSS4_NAEGR)%3B contains Interpro domain(s) IPR013979 Eukaryotic translation initiation factor 2A%2C central region
Solyc02g031840.2.1	2.357508749	0.0002878	0.007804106	L2/L3-related	D,F	Potassium transporter (AHRD V1 **** A9SMZ3_PHYPA)%3B contains Interpro domain(s) IPR003855 K+ potassium transporter
Solyc09g082830.2.1	2.354478896	0.0009588	0.008591523	L2/L3-related	D	ARGONAUTE 1 (AHRD V1 ***- D6RUV9_TOBAC)%3B contains Interpro domain(s) IPR003165 Stem cell self-renewal protein Piwi
Solyc06g061020.2.1	2.347685064	3.40E-05	0.001396011	L2/L3-related	F	Baculoviral IAP repeat-containing protein 3 (AHRD V1 *--- BIRC3_HUMAN)%3B contains Interpro domain(s) IPR017066 S-ribonuclease binding protein%2C SBP1%2C pollen
Solyc02g050260.2.1	2.324089709	6.47E-05	0.000889693	L2/L3-related	D	Protease II (Oligopeptidase B) (AHRD V1 **-- A6GXZ7_FLAPJ)%3B contains Interpro domain(s) IPR002470 Peptidase S9A%2C prolyl oligopeptidase
Solyc09g090840.2.1	2.318635901	0.0030032	0.021371731	L2/L3-related	D	Aspartyl/glutamyl-tRNA(Asn/Gln) amidotransferase subunit B (AHRD V1 ***- D4ZRW8_SPIPL)%3B contains Interpro domain(s) IPR004413 Glutamyl-tRNA(Gln) amidotransferase%2C B subunit
Solyc05g006980.2.1	2.310994154	6.31E-06	0.000119486	L2/L3-related	D	Homeobox-leucine zipper-like protein (AHRD V1 *-*- Q3HRT1_PICGL)%3B contains Interpro domain(s) IPR001356 Homeobox
Solyc05g005480.2.1	2.302252568	7.31E-05	0.002634694	L2/L3-related	F	Oxidoreductase zinc-binding dehydrogenase (AHRD V1 **** C2RQQ3_BACCE)%3B contains Interpro domain(s) IPR002085 Alcohol dehydrogenase superfamily%2C zinc-containing
Solyc03g113630.2.1	2.300776168	0.0002679	0.00296911	L2/L3-related	D	Pentatricopeptide repeat-containing protein (AHRD V1 ***- D7LDK6_ARALY)%3B contains Interpro domain(s) IPR002885 Pentatricopeptide repeat
Solyc06g075610.1.1	2.298973452	3.97E-06	0.000169419	L2/L3-related	L	Exocyst complex component 7 (AHRD V1 *--- EXOC7_DROME)%3B contains Interpro domain(s) IPR004140 Exo70 exocyst complex subunit
Solyc01g104320.2.1	2.290699025	0.0016389	0.013227662	L2/L3-related	D	PAP fibrillin (AHRD V1 ***- A2Q498_MEDTR)%3B contains Interpro domain(s) IPR006843 PAP fibrillin
Solyc08g067960.2.1	2.289589749	0.0001588	0.004009216	L2/L3-related	L,D	CHY zinc finger family protein expressed (AHRD V1 ***- Q337P2_ORYSJ)%3B contains Interpro domain(s) IPR008913 Zinc finger%2C CHY-type
Solyc01g010810.2.1	2.286663512	0.0031537	0.022091832	L2/L3-related	D	RAN guanine nucleotide release factor (AHRD V1 **-* B6TPU9_MAIZE)%3B contains Interpro domain(s) IPR016123 Mog1/PsbP%2C alpha/beta/alpha sandwich
Solyc08g080380.2.1	2.286663512	0.0001771	0.002097081	L2/L3-related	D	Cytochrome P450
Solyc06g005970.2.1	2.283308291	3.02E-05	0.000462908	L2/L3-related	D	Beta-D-glucosidase (AHRD V1 ***- Q7XAS3_GOSHI)%3B contains Interpro domain(s) IPR001764 Glycoside hydrolase%2C family 3%2C N-terminal
Solyc07g019460.2.1	2.256959473	0.0001118	0.003668073	L2/L3-related	F	Cytochrome P450 NADPH-reductase (AHRD V1 **** B3RFK3_PETHY)%3B contains Interpro domain(s) IPR015702 NADPH Cytochrome P450 Reductase
Solyc01g087090.2.1	2.232115293	0.0001593	0.001896952	L2/L3-related	D	cDNA clone J023009A03 full insert sequence (AHRD V1 ***- B7EG04_ORYSJ)

Solyc09g018750.2.1	2.14291154	4.28E-06	8.47E-05	L2/L3-related	D	Inosine-5%26apos-monophosphate dehydrogenase (AHRD V1 ***- Q0KFK7_RALEH)%3B contains Interpro domain(s) IPR000644 Cystathionine beta-synthase%2C core
Solyc02g084340.1.1	2.129455259	0.0046464	0.029703667	L2/L3-related	D	Pre-mRNA-splicing factor CWC25-like protein (AHRD V1 *-*- D0MR21_PHYIN)%3B contains Interpro domain(s) IPR019339 CBF1-interacting co-repressor CIR%2C N-terminal
Solyc02g072160.2.1	2.109355467	0.0001565	0.003964657	L2/L3-related	L	NADH ubiquinone oxidoreductase complex I intermediate-associated protein 30 (AHRD V1 **-- B2IZH1_NOSP7)%3B contains Interpro domain(s) IPR013857 NADH:ubiquinone oxidoreductase intermediate-associated protein 30
Solyc12g008360.1.1	2.094059198	0.0003413	0.008868465	L2/L3-related	F	Oligosaccharyl transferase STT3 subunit family protein (AHRD V1 ***- Q23H40_TETTH)%3B contains Interpro domain(s) IPR003674 Oligosaccharyl transferase%2C STT3 subunit
Solyc08g065320.2.1	2.092642835	0.0014804	0.012188389	L2/L3-related	D	Transmembrane protein 222 (Fragment) (AHRD V1 *--- Q5SSD9_HUMAN)%3B contains Interpro domain(s) IPR008496 Protein of unknown function DUF778
Solyc02g070390.2.1	2.092285466	0.0014873	0.012224196	L2/L3-related	D	PTAC12 (AHRD V1 ***- D7LH29_ARALY)
Solyc08g076360.2.1	2.070841703	1.91E-05	0.000310914	L2/L3-related	D	Gamma-tocopherol methyltransferase (AHRD V1 **** Q1PBH9_SOLLC)%3B contains Interpro domain(s) IPR013216 Methyltransferase type 11
Solyc08g007950.2.1	2.062904111	0.0004836	0.004870108	L2/L3-related	D	30S ribosomal protein S1 (AHRD V1 *-*- B9CNP3_9ACTN)%3B contains Interpro domain(s) IPR003029 Ribosomal protein S1%2C RNA binding domain
Solyc04g071480.1.1	2.057859463	0.0002455	0.005762044	L2/L3-related	L	Exocyst subunit Exo70-interacting protein Roh1 (Fragment) (AHRD V1 *-*- C1IJU1_TOBAC)%3B contains Interpro domain(s) IPR008511 Protein of unknown function DUF793
Solyc09g010870.2.1	2.039240055	0.0054291	0.033594891	L2/L3-related	D	Exoribonuclease R/ribonuclease II (AHRD V1 **-* A5GLA4_SYNPW)%3B contains Interpro domain(s) IPR001900 Ribonuclease II and R
Solyc03g044150.2.1	2.032456279	0.000106	0.00136434	L2/L3-related	D	Subtilisin-like protease (AHRD V1 ***- A9XG40_TOBAC)%3B contains Interpro domain(s) IPR015500 Peptidase S8%2C subtilisin-related
Solyc03g122340.2.1	2.025912886	1.47E-05	0.000522599	L2/L3-related	L	Lipoxygenase (AHRD V1 **** Q96574_SOLLC)%3B contains Interpro domain(s) IPR001246 Lipoxygenase%2C plant
Solyc06g068860.2.1	2.000630113	2.70E-05	0.000417858	L2/L3-related	D	Alpha-mannosidase (AHRD V1 **** D3TI68_SOLLC)%3B contains Interpro domain(s) IPR011682 Glycosyl hydrolases 38%2C C-terminal
Solyc02g094640.2.1	1.998204223	0.0003136	0.00338625	L2/L3-related	D	Acetyl-coenzyme A synthetase (AHRD V1 ***- Q2RNC6_RHORT)%3B contains Interpro domain(s) IPR011904 Acetate--CoA ligase
Solyc06g053790.2.1	1.973414166	0.0011054	0.021787065	L2/L3-related	F	Membrane related protein-like (AHRD V1 **-- Q9LS84_ARATH)%3B contains Interpro domain(s) IPR002913 Lipid-binding START
Solyc05g015050.2.1	1.960320126	0.0011562	0.009993144	L2/L3-related	D	Os12g0236050 protein (Fragment) (AHRD V1 *-*- C7J9Q6_ORYSJ)
Solyc01g106010.2.1	1.9464346	0.0038027	0.025539047	L2/L3-related	D	Fructose-1 6-bisphosphatase class 1 (AHRD V1 ***- D3PE46_DEFDS)%3B contains Interpro domain(s) IPR000146 Fructose-1%2C6-bisphosphatase
Solyc02g069100.2.1	1.939140616	1.54E-05	0.000255129	L2/L3-related	D	Cathepsin B (AHRD V1 ***- Q1HER6_NICBE)%3B contains Interpro domain(s) IPR015643 Peptidase C1A%2C cathepsin B
Solyc01g080840.2.1	1.933026557	0.0001788	0.002114487	L2/L3-related	D	Protein translocase subunit secA (AHRD V1 ***- B9I9A8_POPTR)%3B contains Interpro domain(s) IPR000185 SecA protein
Solyc02g086160.2.1	1.921737591	0.0003304	0.003543028	L2/L3-related	D	D-xylose transporter (AHRD V1 **** D0DTW3_LACFE)%3B contains Interpro domain(s) IPR003663 Sugar/inositol transporter
Solyc07g063530.2.1	1.917569046	0.0031512	0.022087725	L2/L3-related	D	Unknown Protein (AHRD V1)
Solyc06g068670.2.1	1.91564888	5.84E-05	0.000812329	L2/L3-related	D	Unknown Protein (AHRD V1)
Solyc04g049380.2.1	1.903656019	0.0009401	0.008451123	L2/L3-related	D	Uncharacterized aarF domain-containing protein kinase 1 (AHRD V1 *--- ADCK1_XENLA)%3B contains Interpro domain(s) IPR004147 ABC-1
Solyc01g094330.2.1	1.896056051	0.004014	0.026507259	L2/L3-related	D	Expressed protein with function in citronellol catabolism (AHRD V1 **-- C0VQA1_9GAMM)%3B contains Interpro domain(s) IPR010839 Protein of unknown function DUF1446
Solyc03g121480.2.1	1.892530412	0.0020534	0.015819474	L2/L3-related	D	Thioredoxin family protein (AHRD V1 ***- D7MCJ1_ARALY)%3B contains Interpro domain(s) IPR015467 Thioredoxin%2C core
Solyc02g078780.2.1	1.88807933	0.0022715	0.017185447	L2/L3-related	D	Receptor like kinase%2C RLK
Solyc08g075750.2.1	1.884236061	0.0001836	0.002156601	L2/L3-related	D	ATP-dependent Clp protease proteolytic subunit (AHRD V1 ***- A9PA38_POPTR)%3B contains Interpro domain(s) IPR001907 Peptidase S14%2C ClpP
Solyc12g021170.1.1	1.880147425	0.003988	0.026415341	L2/L3-related	D	Electron-transfer flavoprotein ubiquinone oxidoreductase (AHRD V1 ***- D7LK13_ARALY)%3B contains Interpro domain(s) IPR007859 Electron transfer flavoprotein-ubiquinone oxidoreductase
Solyc02g091240.1.1	1.866107113	0.0001234	0.00321439	L2/L3-related	L,D	Auxin efflux carrier protein-like (AHRD V1 **** Q6YZX7_ORYSJ)%3B contains Interpro domain(s) IPR004776 Auxin efflux carrier
Solyc05g055940.2.1	1.812070233	0.0002524	0.002831678	L2/L3-related	D	Myb family transcription factor (AHRD V1 **-* D7M706_ARALY)%3B contains Interpro domain(s) IPR006447 Myb-like DNA-binding region%2C SHAQKYF class
Solyc09g010830.2.1	1.792413719	0.0003756	0.003944145	L2/L3-related	D	Ubiquitin ligase E3 (Fragment) (AHRD V1 *-*- Q16LM2_AEDAE)%3B contains Interpro domain(s) IPR013993 Zinc finger%2C N-recognin%2C metazoa
Solyc03g119520.2.1	1.772728276	0.0053703	0.033304944	L2/L3-related	D	Unknown Protein (AHRD V1)
Solyc03g123750.2.1	1.75738384	0.0016899	0.013559833	L2/L3-related	D	Lipase (AHRD V1 ***- Q5S8F1_RICCO)%3B contains Interpro domain(s) IPR002921 Lipase%2C class 3
Solyc01g097810.2.1	1.750431886	0.000318	0.003428625	L2/L3-related	D	Zeta-carotene desaturase
Solyc03g098000.2.1	1.744618754	0.000238	0.00270005	L2/L3-related	D	At1g32160/F3C3_6 (AHRD V1 ***- Q9FVR1_ARATH)%3B contains Interpro domain(s) IPR008479 Protein of unknown function DUF760
Solyc07g043050.1.1	1.723715325	0.0022339	0.034319077	L2/L3-related	L,D	UDP-glucosyltransferase (AHRD V1 **-* A7M6U9_IPONI)%3B contains Interpro domain(s) IPR002213 UDP-glucuronosyl/UDP-glucosyltransferase
Solyc12g094430.1.1	1.71883565	0.0037169	0.025081128	L2/L3-related	D	Glutathione S-transferase (AHRD V1 ***- C7F8A3_ARAHY)%3B contains Interpro domain(s) IPR004046 Glutathione S-transferase%2C C-terminal
Solyc12g099080.1.1	1.702251973	0.0050401	0.031695788	L2/L3-related	D	ADP ribosylation factor (AHRD V1 *-*- Q08IJ1_TOBAC)%3B contains Interpro domain(s) IPR006688 ADP-ribosylation factor
Solyc08g006430.2.1	1.701480567	0.0014431	0.011937782	L2/L3-related	D	N-methyl-L-tryptophan oxidase (AHRD V1 *-*- D2ZAX1_9ENTR)%3B contains Interpro domain(s) IPR006281 Sarcosine oxidase%2C monomeric
Solyc07g054500.2.1	1.693401586	0.0034604	0.023831857	L2/L3-related	D	Receptor like kinase%2C RLK
Solyc12g099440.1.1	1.693091221	0.0006035	0.013975164	L2/L3-related	F	Fatty acid oxidation complex subunit alpha (AHRD V1 ***- FADJ_ECOHS)%3B contains Interpro domain(s) IPR006176 3-hydroxyacyl-CoA dehydrogenase%2C NAD binding
Solyc08g069030.2.1	1.688193038	0.0008519	0.007790623	L2/L3-related	D	Delta-aminolevulinic acid dehydratase (AHRD V1 ***- B9RJ27_RICCO)%3B contains Interpro domain(s) IPR001731 Tetrapyrrole biosynthesis%2C porphobilinogen synthase
Solyc04g081570.2.1	1.659844757	0.0012845	0.02450918	L2/L3-related	F	Chaperone protein htpG (AHRD V1 **-- HTPG_MYCA1)%3B contains Interpro domain(s) IPR015566 Molecular chaperone%2C heat shock protein%2C endoplasmin
Solyc07g014670.2.1	1.637974583	0.0041424	0.027086191	L2/L3-related	D	Cytochrome P450
Solyc03g007810.2.1	1.635643829	0.0007863	0.007304513	L2/L3-related	D	Pyruvate kinase (AHRD V1 ***- A5BTB0_VITVI)%3B contains Interpro domain(s) IPR001697 Pyruvate kinase
Solyc04g063350.2.1	1.590147316	0.0003719	0.003909308	L2/L3-related	D	3-methyl-2-oxobutanoate dehydrogenase (2-methylpropanoyl-transferring) (AHRD V1 **** A1U0F1_MARAV)%3B contains Interpro domain(s) IPR001017 Dehydrogenase%2C E1 component
Solyc07g040960.1.1	1.573733084	0.0004469	0.009668326	L2/L3-related	L	Os07g0175100 protein (Fragment) (AHRD V1 *--- Q0D898_ORYSJ)
Solyc03g118170.2.1	1.522949018	0.0022806	0.017197737	L2/L3-related	D	Glycyl-tRNA synthetase (AHRD V1 **** D7KEM8_ARALY)%3B contains Interpro domain(s) IPR002315 Glycyl-tRNA synthetase%2C alpha2 dimer
Solyc06g009140.2.1	1.48125845	0.0006221	0.006012893	L2/L3-related	D	Late embryogenesis abundant 3 family protein (AHRD V1 ***- D7KBC5_ARALY)%3B contains Interpro domain(s) IPR004926 Late embryogenesis abundant protein 3
Solyc08g079430.2.1	1.477035871	0.0003757	0.003944145	L2/L3-related	D	Primary amine oxidase (AHRD V1 ***- B9RBR2_RICCO)%3B contains Interpro domain(s) IPR000269 Copper amine oxidase
Solyc02g062970.2.1	1.475432858	0.0009237	0.008325613	L2/L3-related	D	Xaa-Pro aminopeptidase 2 (AHRD V1 ***- Q93X45_SOLLC)%3B contains Interpro domain(s) IPR001131 Peptidase M24B%2C X-Pro dipeptidase/aminopeptidase P%2C conserved site IPR000994 Peptidase M24%2C structural domain
Solyc07g064130.1.1	1.367938761	0.0010142	0.020462462	L2/L3-related	F	Ubiquitin (AHRD V1 ***- Q39257_PEA)%3B contains Interpro domain(s) IPR019956 Ubiquitin subgroup
Solyc01g101240.2.1	1.366427988	0.0015658	0.012756126	L2/L3-related	D	Aspartic proteinase (AHRD V1 **** ASPRX_ORYSJ)%3B contains Interpro domain(s) IPR001461 Peptidase A1
