# Supplementary material for: Using a periclinal chimera to unravel layer-specific gene expression in plants
Source: Plant J. 2013 Jul 19;75(6):1039–49. doi: 10.1111/tpj.12250 (PMC4223383; doi:10.1111/tpj.12250)
Supplement: Supplementary file 14 — Methods S1. Supporting methods. [file tpj0075-1039-sd14.docx]

**Supporting methods**

**Water Stress**

Water stress was induced by excising 8^th^ leaves from *S. lycopersicum*, *S. pennellii* and Periclinal1 (Peri1) of 8 weeks old plants, placing them on a dry filter paper, and allowing them to develop 24 hours. The rate of dehydration was estimated as the percentage of initial fresh weight (FW) that remained after treatment.

**ABA measurements**

ABA extraction and measurement were carried out using the same samples used for the poly(A)+ RNA extraction, i.e. 8^th^ leaf from the apex (C) and 8^th^ leaf with 30% loss of fresh weight (D) of 8 weeks old plants. Method used is as described by Forcat et al., 2008.

**Poly A RNA purification**

Total RNA was isolated from 1) 8^th^ leaf from the apex of 8 week old plants; 2) 8^th^ leaf with 30% loss of fresh weight; 3) mature green fruits of *S. lycopersicum*, *S. pennellii* and peri1. Frozen ground tissue was homogenized in RNA extraction buffer (8M guanidinium hydrochloride; 20mM MES; pH7) and phenol:chloroform:isoamyl alcohol (v/v 50:48:2). After centrifugation the aqueous phase was re-extracted with phenol:chloroform: isoamyl alcohol and then subjected to three successive precipitations with ethanol/sodium acetate; 4M LiCl; and ethanol/sodium acetate. The final pellet was washed in 70% ethanol and re-suspended in diethylpyrocarbonate treated water, as described in Experimental procedures. Highly purified poly(A)+ RNA was prepared using Magnetight Oligo(dT) Particles, Straight A’s TM mRNA Isolation System (Novagen, Inc.) following the manufacturer’s instructions.

**Example calculation of L1 gene expression based on Sanger sequencing of RT-PCR products**

Gene-specific primers were used to amplify cDNA from Peri1 mRNA and PCR products were sequenced using Sanger-based capillary sequencing by GATC Biotech (London).

The proportion of *S*. *pennelli* (L1 expression) of each polymorphic base in the periclinal line (Peri1) was calculated using the chromatograms and the trace peak heights for each base. The trace peak height that corresponds to the relative concentration of each base at a given position in the sequence was used to calculate the percentage level of the *S. pennellii* or *S. lycopersicum* allele at the base. These percentages were used to obtain an arithmetic mean of all the polymorphic sites assessed for a gene and this value provides the proportion of L1 expression for an allele in Peri1. We performed a similar analysis using genomic DNA to estimate the proportion of tissue that corresponds to the L1 layer. Approximately 20% of the tissue is L1 (*S. pennellii*) and therefore the expression levels were adjusted to account for this level.

mPph/(mLph+ mPph) X 100 = % Penn peak height

mPph = mean Penn allele-peak height at SNP

mLph = mean Lyc allele-peak height at SNP

Adjustment for amount of L1 tissue (20%)

L1 % = (%P/20)/((%P/20)+(100-%P)/80))x100

%P = %Penn peak height

e.g. if a gene has 70% penn allele peak height from only 20% of the tissue it means that this is equivalent to 90.3% of the gene expression is coming from L1 tissue.

i.e. L1 % = (70/20)/((70/20)+(100-70)/80))X100 = 90.3%

(Sanger trace files are available on request)

**Read mapping and global expression quantification**

Mapping was done using Applied Biosystems’ SOLiD BioScope whole transcriptome analysis single-read alignment pipeline version 1.2.1 (Life Technologies, 2010). Non-desirable contamination sequences were removed by initial mapping to filter reference. Screening was performed against NCBI’s Univec database (Kitts, 2010), the *Escherichia coli* genome (NC_000913.2), Tomato mosaic virus (NC_002692.1), and the *Solanum lycopersicum* chloroplast genome (NC_007898.1). Reads were mapped to the *Solanum lycopersicum* reference genome SL2.40 (TGC, 2012) and to a database of flanking sequences of junctions, containing known and putative junction sequences. The junction database was inferred from the reference genome based on *Solanum lycopersicum* genome annotation ITAG2.3 (TGC, 2012). In our study, only coding sequence regions were considered to avoid any bias due to the uncertainty associated with UTR annotations. Two mismatches were allowed in 25bp seed sequence starting at the beginning of the read, followed by extension of the seed alignment. In the case of unaligned reads, a second round of mapping was repeated with the seed start position at the 20^th^ base. Genome and splice junction mappings were merged producing the final alignments of unfiltered reads.

BioScope pipeline calculates expression for each annotation feature based on stringency criteria without exporting the reads considered. A custom perl script was written using Bio-SamTools (Li et al., 2009) to quantify gene expression on coding sequence (CDS) regions and to extract the final valid set of reads based on BioScope’s stringency filters. Only the best alignments for each read with the highest score, with minimum mapping quality of 10 and overlapping CDS coordinates were initially considered. Non-spliced alignments must not include more than 3 intron bases, while spliced ones must match exactly the exon-intron boundaries and must cover at least 8 exon bases on both sides of a junction. Summary of read mapping is shown in Table S2. The final valid set of read alignments to CDS was utilized for variant detection.

**Variant detection**

Variant detection was restricted to the 21,938 genes expressed in both parental lines and the chimera independent of tissue sample. Mapped reads across tissue samples were pooled for both parental lines to identify polymorphisms between lyc and penn alleles in the coding sequences. Variant detection was performed for both lyc and penn samples against the reference genome and positions with non-overlapping alleles were selected to uniquely characterize the parental origin in the chimera.

SNP detection was performed using Varid version 1.1.0e (Dalca et al., 2010), a Hidden Markov Model variant detection platform for both colour- and letter-space, and FreeBayes version 0.6.5 (Garrison and Marth, 2012), a Bayesian variant detector in letter-space. Bases with less than 4 read coverage in either lyc or penn samples were discarded. Varid was run in color-space mode and, in cases of consecutive SNPs, prediction was verified by including the letter-space signal.

Depending on the relative abundance of alleles in putative heterozygous sites, it is possible to either discard a position that significantly discriminates between the parental lines or to include a false polymorphism. Moreover, the read mapping bias for penn samples favours the reference allele at the expense of detecting homozygous alternate sites in the pennellii genome. Instead of considering various ambiguous maximum thresholds for the relative abundance of the dominating allele in order to differentiate between heterozygous and homozygous sites, a different approach was used. Varid was run with rank-sum heterozygous distribution penalties that weighted the probability of being heterozygous in the case of multiple distributions in the error qualities. FreeBayes was run twice with both lyc and penn genomes treated once as diploid and once as haploid. The latter increases the probability of detecting homozygous (alternate) sites and thus more polymorphisms between the lyc and penn alleles.

Results of the comparison of the three variant detection methods are presented in Figure S2. As expected, FreeBayes with monoploid considered genomes (FreeBayes-haploid) predicted more polymorphisms and consequently, more genes with at least one SNP, followed by Varid and then by FreeBayes. For the same variant positions, all 3 methods almost always detect the same alleles. However, only 25% of the commonly identified 13,277 genes with at least 1 SNP have the same variant positions predicted by all methods. The latter observation shaped our decision to conduct downstream analysis for all 3 sets of SNPs detected, instead of defining a universally identified set of SNPs. Summary of results of SNP detection are shown in Table S3.

**Parental-origin allele-specific expression (ASE) quantification**

SNP predictions were utilized to quantify parental-origin allele-specific expression in all three plant types. A custom perl script was written using Bio-SamTools in order to quantify the four expression values. Lyc allele-specific expression in wild-type (Lw) and in chimera (Lc), as well as penn allele-specific expression in wild-type (Pw) and in chimera (Pc), were calculated separately for each tissue, for each variant detection method and for all genes with at least one SNP detected. Ambiguous reads having both lyc- and penn-specific alleles were discarded.

**Differential expression analysis and gene classification**

The four parental-origin ASE values correspond to a 2x2 factorial experiment. One factor specifies the parental (lyc or pen) allele-specific expression while the second factor specifies the genome examined, chimeric or parental wild type. We utilized a treatment-contrast parametrization (Smyth, 2005) to identify genes that respond differently to "chimerization" with respect to their parental-origin ASE. The contrasts tested for differential expression are: a) difference of lyc allele-specific expression between wild type (Lw) and chimera (Lc); b) difference of penn allele-specific expression between wild type (Pw) and chimera (Pc); c) interaction effect, i.e. difference of the differences, and d) difference of penn and lyc allele-specific expressions in chimera (Pc vs Lc).

Differential expression analysis was conducted using the edgeR Bioconductor library (Robinson et al., 2010) and its implemented negative binomial generalized linear models (GLMs). Analysis was conducted separately for each tissue sample. L1 and L2/L3 layers in plants are expected to express diverse transcriptome repertoire. Thus, a trimmed mean of M values (TMM) normalization procedure (Robinson and Oshlack, 2010) as implemented in edgeR was applied. A common dispersion value of 0.02 was set for all four pseudo-samples (four parental-origin ASE). The square root of the common dispersion represents the coefficient of variation of biological samples. The selected value is large enough to ensure that the negative binomial model is not reduced to a poisson one and small enough to allow for detection of differentially expressed genes. Other approaches to estimate the common dispersion by treating samples across tissues or samples within the same tissue as biological replicates of each other led to no differentially expressed genes due to large observed dispersion. A value of 1 was added to all ASE values before normalization. 20% of the original gene classifications as L1 or L2/L3 were discarded due to this addition and only 5% of the new gene predictions were introduced in the final set. Manual inspection of the discarded cases revealed spurious differentially expressed genes: genes with low expression values in general and at least one value equal to zero giving infinite log fold changes that become small and non-significant upon the addition of 1.

Following the fitting of the negative binomial GLMs, genes were identified as differentially expressed with respect to each contrast, after correcting for multiple testing using the Benjamini and Hochberg approach (Benjamini and Hochberg, 1995; Benjamini and Yekutieli, 2001) and applying a cut-off value of 0.05 for the false discovery rate (FDR). Specific combinations of differential expression in the four contrasts examined classify genes as L1 or L2/L3. Genes that are down-regulated in the chimera compared to the wild-type genome with respect to their lyc origin and not with respect to their penn origin, and for which a statistical significant interaction effect is observed, are classified as L1 genes. Additionally, if in the chimera these genes are up-regulated with respect to their penn allele-specific expression and in comparison to their lyc one, they are denoted as L1 specific, else as L1 related. In a similar way, genes with a (highly) biased lyc allele-specific expression in the chimera are classified as L2/L3.

For each variant detection method, L1 and L2/L3 genes were identified for each tissue. Genes with contradicting classifications among tissues and for the same SNP detection method were discarded. Comparison of the classifications resulting from all three variant detectors is shown in Supporting Information Figure S3. Very few genes were classified as L1 by one method and as L2/L3 by another. As expected, many genes are not classified universally as layer-specific. We adopted a conservative approach to minimize any false predictions at the expense of coverage of layer-specific transcriptome. Thus, only genes classified as L1 or L2/L3 by all 3 methods were finally considered as layer specific genes. Comparison of the tissue support for classification of genes as L1 or L2/L3 (Supporting Information Figure S4) demonstrates that the majority of classifications are (solely) derived from dehydrated leaf samples.

Final discrimination between layer-specific and layer-related genes is performed based on the differential expression analysis done on polymorphisms detected by Varid. In all tables and figures, fold changes, p-values, tissue support and specific versus related classification are all calculated based on Varid polymorphisms.

**Gene Ontology (GO) term analysis**

GO-term enrichment analysis was performed using Ontologizer 2.0 (Bauer et al., 2008) and its implemented model-based gene set analysis (MGSA) (Bauer et al., 2010). MGSA is a Bayesian approach that takes into account the high overlap between GO categories by analyzing them at once and estimating the probability of categories to be active. As a population set, we defined all genes with GO assignment and with at least one SNP detected by Varid. GO terms were extracted from the *Solanum lycopersicum* genome annotation ITAG2.3 (The International Tomato Genome Sequencing Consortium) and all domains (Cellular Component, Biological Process, Molecular Function) were taken into account in subsequent analysis. 10,007 out of 13,950 genes with at least one SNP detected have at least one GO-term assigned to them. This population set contains 275 L1 and 916 L2/L3 genes. MGSA was run 20 times to calculate the average posterior probability and confidence intervals.

The top 10 over-represented GO-terms in L1 and L2/L3 genes are ranked based on the marginal posterior probability in Figure 5. Six terms in L1 and six in L2/L3 have a posterior > 0.5 and thus, considered to be active. L1 genes are involved in lipid and wax synthesis and cellulose synthesis in cell wall production, while L2/L3 genes are associated with photosynthesis and the chloroplast. The GO-terms that become active when considering only the L1-specific and the L2/L3-specific genes are also denoted in Figure 5. Supporting Information Figures S6 and S7 display the GO-graphs for L1 and L2/L3 genes. These graphs are induced by the enriched GO-terms, which are displayed in colour according to the GO domain (sub-ontology).

**Motif (L1-box) promoter analysis**

In order to assess whether L1-box (TAAATG(C/T)A) is over-represented in the sets of L1 and L2/L3 classified genes, promoter sequences were extracted for each gene in the population set. For such a set, we considered all genes examined in the differential expression analysis based on Varid polymorphisms: 13,950 genes with at least one SNP detected by Varid. The population set contains 3 classes of genes: the L1 genes, the L2/L3 genes and the genes classified neither as L1 nor as L2/L3 (L0). Promoter sequence was defined as a repeat-masked 1000 base pairs (bp) region upstream of the translation start site due to the uncertainty associated with UTR annotations. Overlapping promoter regions across classes of genes were discarded, while overlapping promoters within each class were collapsed. The former affected only five pairs of L0 and L2/L3 genes, and the latter occurred only for L0 genes.

We used mosdi (Marschall and Rahmann, 2009) to quantify the occurrences of the motif for each class of genes. L1-box (or its reverse complement) was found in 18% of L1 genes, 12% of L2/L3 genes and 13% of L0 genes. We used Fisher's exact test to assess the statistical significance of L1-box occurrence in L1 and L2/L3 genes with respect to the population set. As expected, only L1 genes exhibit significant enrichment of L1-box motif in their 1000 bp upstream region (p-value=0.005). The statistical significance is increased (p-value=6.3e-4) when considering only L1-specific genes in the L1 class.

**Statistical analysis**

All statistical analysis was performed in R unless otherwise noted. Venn diagrams were plotted using the R VennDiagram package (Chen and Boutros, 2011).
