# Supplementary material for: Using a periclinal chimera to unravel layer-specific gene expression in plants
Source: Plant J. 2013 Jul 19;75(6):1039–49. doi: 10.1111/tpj.12250 (PMC4223383; doi:10.1111/tpj.12250)
Supplement: Supplementary file 15 [file tpj0075-1039-sd15.docx]

**Supporting information** References

**Bauer, S., Gagneur, J., and Robinson, P.N.** (2010). GOing Bayesian: model-based gene set analysis of genome-scale data. Nucleic Acids Res **38,** 3523-3532.

**Bauer, S., Grossmann, S., Vingron, M., and Robinson, P.N.** (2008). Ontologizer 2.0 - a multifunctional tool for GO term enrichment analysis and data exploration. Bioinformatics **24,** 1650-1651.

**Benjamini, Y., and Hochberg, Y.** (1995). Controlling the False Discovery Rate - a Practical and Powerful Approach to Multiple Testing. Journal of the Royal Statistical Society Series B-Methodological **57,** 289-300.

**Benjamini, Y., and Yekutieli, D.** (2001). The control of the false discovery rate in multiple testing under dependency. Annals of Statistics **29,** 1165-1188.

**Chen, H., and Boutros, P.C.** (2011). VennDiagram: a package for the generation of highly-customizable Venn and Euler diagrams in R. Bmc Bioinformatics **12,** 35.

**Dalca, A.V., Rumble, S.M., Levy, S., and Brudno, M.** (2010). VARiD: A variation detection framework for color-space and letter-space platforms. Bioinformatics **26,** i343-i349.

**Forcat, S., Bennett, M.H., Mansfield, J.W., and Grant, M.R.** (2008). A rapid and robust method for simultaneously measuring changes in the phytohormones ABA, JA and SA in plants following biotic and abiotic stress. Plant Methods **4,** 16.

**Garrison, E. and Marth G**. (2012) Haplotype-based variant detection from short-read sequencing. <http://arxiv.org/abs/1207.3907>

**Kitts, P., Madden, T., Sicotte, H., Black, L., & Ostell, J. .** (2010). Univec database; build #6.0

**Li, H., Handsaker, B., Wysoker, A., Fennell, T., Ruan, J., Homer, N., Marth, G., Abecasis, G., and Durbin, R.** (2009). The Sequence Alignment/Map format and SAMtools. Bioinformatics **25,** 2078-2079.

**Life Technologies.** (2010). Applied Biosystems SOLiDTM System: BioScopeTM Software for Scientists Guide: Data Analysis Methods and Interpretation (version 1.2.1).

**Marschall, T., and Rahmann, S.** (2009). Efficient exact motif discovery. Bioinformatics **25,** i356-364.

**Robinson, M.D., and Oshlack, A.** (2010). A scaling normalization method for differential expression analysis of RNA-seq data. Genome Biol **11,** R25.

**Robinson, M.D., McCarthy, D.J., and Smyth, G.K.** (2010). edgeR: a Bioconductor package for differential expression analysis of digital gene expression data. Bioinformatics **26,** 139-140.

**Smyth, G.K.** (2005). Bioinformatics and Computational Biology Solutions Using R and Bioconductor.

**TGC- Tomato Genome Consortium.** (2012). The tomato genome sequence provides insights into fleshy fruit evolution. Nature **485,** 635-641.
